# Deoxygenative functionalization of alcohols, ethers, and carbonyl compounds by chromium catalysis

**DOI:** 10.1126/sciadv.aeg4594

**Published:** 2026-09-25

**Authors:** Wendi Chang, Tenggang Jiao, Changpeng Chen, Feijiang Zhou, Xiaogai Li, Shuya Wang, Jingxia Wang, Jinwei Zhang, Jing Liu, Ying Cao, Xiaoming Zeng, Xuefeng Cong

**Affiliations:** ^1^ Institute of Molecular Plus, Haihe Laboratory of Sustainable Chemical Transformations, Tianjin University, Tianjin 300072, P. R. China.; ^2^ State Key Laboratory of Advanced Optical Polymer and Manufacturing Technology, Qingdao Key Laboratory of Biomacromolecular Drug Discovery and Development, College of Chemical Engineering, Qingdao University of Science and Technology, Qingdao 266042, P. R. China.; ^3^ Key Laboratory of Green Chemistry & Technology, Ministry of Education, College of Chemistry, Sichuan University, Chengdu 610064, P. R. China.; ^4^ State Key Laboratory of Elemento-Organic Chemistry, Nankai University, Tianjin 300071, P. R. China.

## Abstract

The development of an efficient and unified methodology for converting ubiquitous alcohols, ethers, carbonyls, and esters into value-added products via the activation of C−O single and double bonds is of great interest and importance but remains a challenging goal. Herein, we report an efficient and general chromium-catalyzed strategy for the deoxygenative functionalization of alcohols, ethers, and carbonyl compounds with a number of electrophiles. This general strategy couples these oxygenated functional groups with a number of electrophiles, providing an efficient route for the synthesis of a broad range of synthetically appealing organosilicon, organogermanium, organostannane, and organoboron compounds. Moreover, this chromium-catalyzed strategy is demonstrated to be readily scalable and applicable to the late-stage functionalization of various biorelevant molecules. Mechanistic investigations suggest that this deoxygenative functionalization was initiated by two-electron oxidative addition of the C−O bond to a highly active, low-valent chromium species, generating an organochromium intermediate that reacts with a number of electrophiles to construct C−Si, C−Ge, C−Sn, and C−B bonds.

## INTRODUCTION

**Fig. 1.**
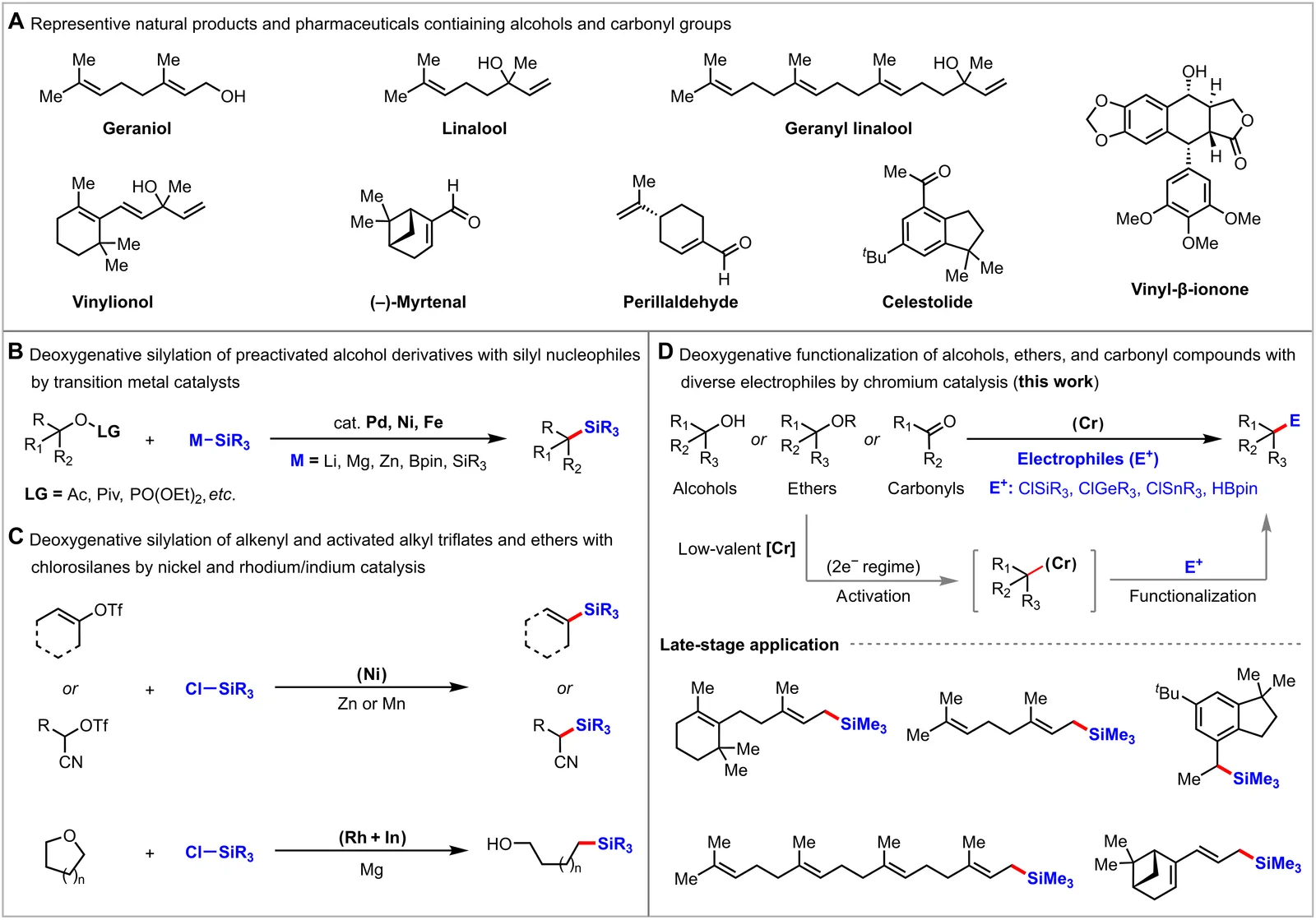
Background and reaction design. (**A**) Represent natural products and pharmaceuticals containing alcohols and carbonyl groups. (**B**) Deoxygenative silylation of preactivated alcohol derivatives with silyl nucleophiles by transition metal catalysts. (**C**) Deoxygenative silylation of alkenyl and activated alkyl triflates and ethers with chlorosilanes by nickel and rhodium/indium catalysis. (**D**) Deoxygenative functionalization of alcohols, ethers, and carbonyl compounds with diverse electrophiles by chromium catalysis (this work).

Alcohols, ethers, carbonyls, and esters are fundamental structural motifs in a large number of natural products, bioactive molecules, and pharmaceuticals (Fig. 1A) (*1*, *2*). They are also among the most accessible and versatile feedstock chemicals in synthetic organic chemistry. Therefore, the development of efficient and selective methods for converting these oxygen-containing groups into value-added functionalities via the direct activation and functionalization of C−O single and double bonds is of great interest and much importance. Among various deoxygenative functionalization (*3*–*8*), silylation of these oxygenated motifs is of particular interest as it provides an efficient and straightforward route for the synthesis of structurally diverse organosilanes, which represent a valuable class of synthetic linchpins with broad applications in organic synthesis, functional materials, and bioactive molecules (*9*–*18*). Remarkable advances have been recently made in transition metal–catalyzed deoxygenative silylation of alcohol derivatives with diverse silicon nucleophiles (Fig. 1B) (*19*–*27*). Very recently, Xu (*28*) and Lundberg (*29*) independently developed an efficient electrochemical method for the deoxygenative silylation of alcohols or ketones with hydrosilanes, substantially expanding access to functionalized organosilanes. However, the analogous deoxygenative silylation using silicon electrophiles, especially with readily accessible chlorosilanes that are raw materials for producing silicon nucleophiles and hydrosilanes, has remained underdeveloped. In particular, achieving this transformation directly from free alcohols and carbonyls presents a substantial and persistent challenge. Recent breakthroughs were achieved by Shu (*30*–*37*) and Oestreich (*38*, *39*), realizing the deoxygenative silylation of alkenyl triflates and activated alkyl triflates with chlorosilanes (Fig. 1C, top). Subsequently, the reductive coupling with chlorosilanes has attracted growing interest (*40*–*49*). One important example in this regard was reported by Nakao and coworkers, who demonstrated rhodium/indium heterobimetallic catalysis for the reductive C(sp^3^)–O silylation of ethers (Fig. 1C, bottom) (*42*). Very recently, Lin and coworkers developed an elegant two-stage, one-pot electrochemical protocol that enables the direct deoxygenative silylation of alcohols and carbonyl compounds with chlorosilanes via electrochemical reduction of in situ–generated silyl ethers (*50*, *51*), further highlighting the growing interest in this research field. Nevertheless, this electrochemical strategy has seen limited application in the deoxygenative silylation of propargyl alcohols and requires excess hydrosilane (e.g., methyldimethoxysilane) to generate silyl ethers. Consequently, the development of a unified and efficient catalytic strategy for the deoxygenative silylation of alcohols, ethers, and carbonyl compounds using readily available chlorosilanes remains highly desirable.

Chromium catalysis has recently emerged as a powerful tool for the activation and transformation of strong chemical bonds (*52*–*68*). In particular, low-valent chromium catalysts have demonstrated unique activity and selectivity in the activation and functionalization of unactivated C–O bonds of phenol and alcohol derivatives (*54*, *55*). This capability has enabled various challenging transformations (*69*–*75*), including the chemoselective cross-coupling of two distinct C(aryl)–O bonds (*70*), the reductive coupling between C(aryl)–N and C(aryl)–O bonds (*71*), and the multicomponent coupling of geminal C–O and C–H bonds in benzyl ethers with arylmagnesium reagents and electrophiles (*72*). Inspired by these achievements, we envisioned chromium catalysis as an efficient platform for the deoxygenative functionalization of alcohols, ethers, and carbonyls with diverse electrophiles. Herein, we report an efficient and general chromium-catalyzed strategy for the deoxygenative functionalization of alcohols, ethers, and carbonyl compounds with a number of electrophiles (Fig. 1D). This strategy couples these oxygenated functional groups with electrophiles via a two-electron oxidative addition process for the C–O bond activation, providing efficient access to a broad range of synthetically appealing organosilicon, organogermanium, organostannane, and organoboron building blocks. Moreover, this chromium-catalyzed protocol is demonstrated to be readily scalable and applicable to the direct functionalization and late-stage modification of various biorelevant molecules.

## RESULTS

### Screening of reaction conditions

**Fig. 2.**
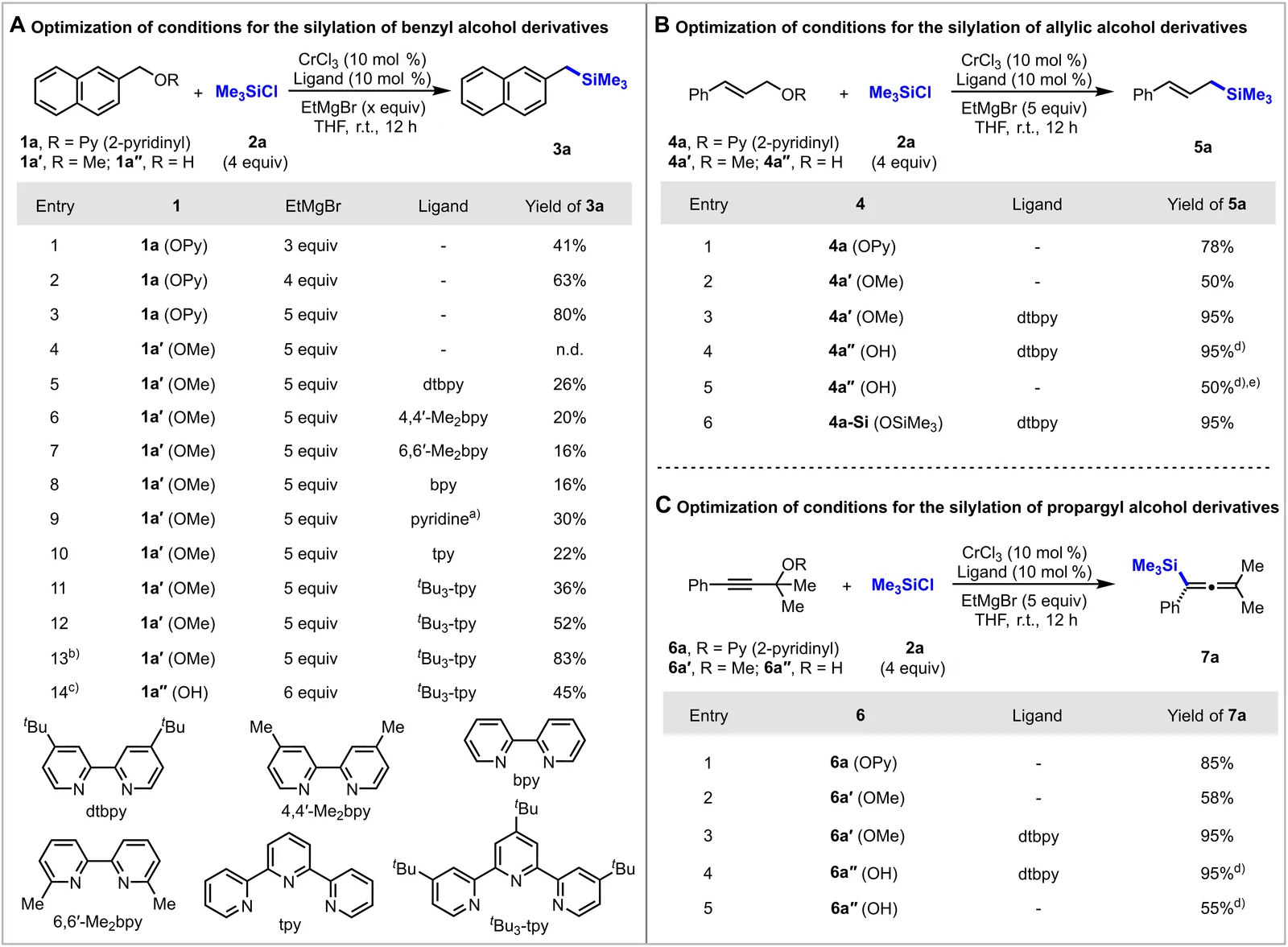
Optimization of reaction conditions. (**A**) Optimization of conditions for the silylation of benzyl alcohol derivatives. (**B**) Optimization of conditions for the silylation of allylic alcohol derivatives. (**C**) Optimization of conditions for the silylation of propargyl alcohol derivatives. Reaction conditions: **1**, **4,** or **6** (0.2 mmol); **2a** (0.8 mmol); CrCl_3_ (10 mol %); ligand (10 mol %); EtMgBr (0.6 to 1.0 mmol); and THF (2.0 ml); room temperature, 12 hours (h), isolated yield. *^a^*Pyridine (0.16 mmol). *^b^*CrCl_3_ (20 mol %), *^t^*Bu_3_-tpy (20 mol %), 70°C, 24 hours. *^c^*CrCl_3_ (25 mol %), *^t^*Bu_3_-tpy (25 mol %), **2a** (1.0 mmol), and EtMgBr (1.2 mmol), 70°C, 48 hours; trimethylsilyl 2-naphthylmethyl ether was formed in 46% yield. *^d^***2a** (1.0 mmol) and EtMgBr (1.2 mmol). *^e^*Trimethylsilyl cinnamyl ether (**4a-Si**) was formed in 49% yield. n.d., not detected.

We commenced our study by examining the reaction of 2-naphthylmethyl 2-pyridyl ether **1a** with chlorotrimethylsilane **2a** (Fig. 2A) as the pyridyl group can promote the C−O bond cleavage and stabilize the metalloalkane intermediates (*72*, *76*–*78*). Evaluation of transition metal catalysts revealed that the low-cost CrCl_3_, combined with ethylmagnesium bromide (EtMgBr) as the reductant, showed good activity for the deoxygenative silylation of **1a** with **2a**, affording the corresponding product **3a** in 80% yield at room temperature (Fig. 2A, entries 1 to 3, and see the Supplementary Materials). In contrast, the reaction of 2-naphthylmethyl methyl ether **1a′** with **2a** did not occur under the same conditions (Fig. 2A, entry 4), highlighting the essential role of the pyridyl group. This result prompted us to explore the use of external pyridyl ligands to facilitate the reaction of **1a′** with **2a**. As anticipated, 4,4′-di-*tert*-butyl-2,2′-dipyridyl (dtbpy) allowed the deoxygenative silylation of **1a′** with **2a** to occur at room temperature, albeit giving the product **3a** in 26% yield (Fig. 2A, entry 5). Other dipyridyl ligands such as 4,4′-dimethyl-2,2′-bipyridyl, 6,6′-di-methyl-2,2′-dipyridyl, and 2,2′-bipyridine showed less efficiency in promoting the silylation of **1a′** (Fig. 2A, entries 6 to 8). Pyridine (0.8 equiv) also facilitated the silylation of **1a′**, affording **3a** in 30% yield (Fig. 2A, entry 9). The use of terpyridine (tpy) afforded **3a** in 26% yield (Fig. 2A, entry 10). Replacing tpy with the *tert*-butyl–substituted analog *^t^*Bu_3_-tpy increased the yield of **3a** to 36% (Fig. 2A, entry 11). Gratifyingly, conducting the reaction of **1a′** with **2a** at 70°C in the presence of *^t^*Bu_3_-tpy increased the yield of **3a** to 52% (Fig. 2A, entry 12). Increasing the loading of CrCl_3_ and *^t^*Bu_3_-tpy to 20 mol % further enhanced the yield of **3a** to 83% (Fig. 2A, entry 13). Notably, the reaction of 2-naphthylmethanol **1a″** with **2a** at 70°C in the presence of *^t^*Bu_3_-tpy also occurred, affording **3a** in 45% yield, along with the benzyl trimethylsilyl ether in 46% yield (Fig. 2A, entry 14).

We next examined the analogous reactions of cinnamyl alcohol and its ether derivatives (Fig. 2B). The deoxygenative silylation of the cinnamyl 2-pyridyl ether **4a** with **2a** proceeded efficiently at room temperature using CrCl_3_ as the catalyst and EtMgBr as the reductant, affording the corresponding cinnamyltrimethylsilane **5a** in 78% yield (Fig. 2B, entry 1). The silylation of cinnamyl methyl ether **4a′** with **2a** also took place but gave a lower yield (Fig. 2B, entry 2). Gratifyingly, the use of dtbpy ligand substantially accelerated the reaction of **4a′** with **2a**, affording the desired product **5a** in 95% yield (Fig. 2B, entry 3). Notably, the deoxygenative silylation of cinnamyl alcohol **4a″** with **2a** also underwent smoothly under the same conditions, yielding **5a** in 95% yield (Fig. 2B, entry 4). In the absence of dtbpy, the reaction of **4a″** with **2a** gave **5a** in 50% yield along with the cinnamyl trimethylsilyl ether **4a-Si** in 49% yield (Fig. 2B, entry 5). The reaction of **4a-Si** with **2a** in the presence of dtbpy worked well to afford **5a** in 95% yield (Fig. 2B, entry 6). These results indicated that the cinnamyl trimethylsilyl ether **4a-Si** should be a key intermediate in the silylation of alcohol **4a″** with **2a**. Subsequently, the analogous reactions of propargyl alcohol and its ether derivatives were examined (Fig. 2C). Similar to **4a**, the reaction of the propargyl 2-pyridyl ether **6a** with **2a** also worked well at room temperature, affording the tetrasubstituted silylallene **7a** in 85% yield (Fig. 2C, entry 1). The analogous methyl ether **6a′** afforded **7a** in a relatively lower yield (Fig. 2C, entry 2). As observed for **4a′**, the dtbpy ligand also dramatically increased the yield of **7a** to 95% in the reaction of **6a′** (Fig. 2C, entry 3). The reaction of propargyl alcohol **6a″** showed a similar performance with that of **6a′**, giving **7a** in 95% yield in the presence of dtbpy but a relatively lower yield of **7a** in the absence of dtbpy (Fig. 2C, entries 4 and 5).

### Substrate scope

**Fig. 3.**
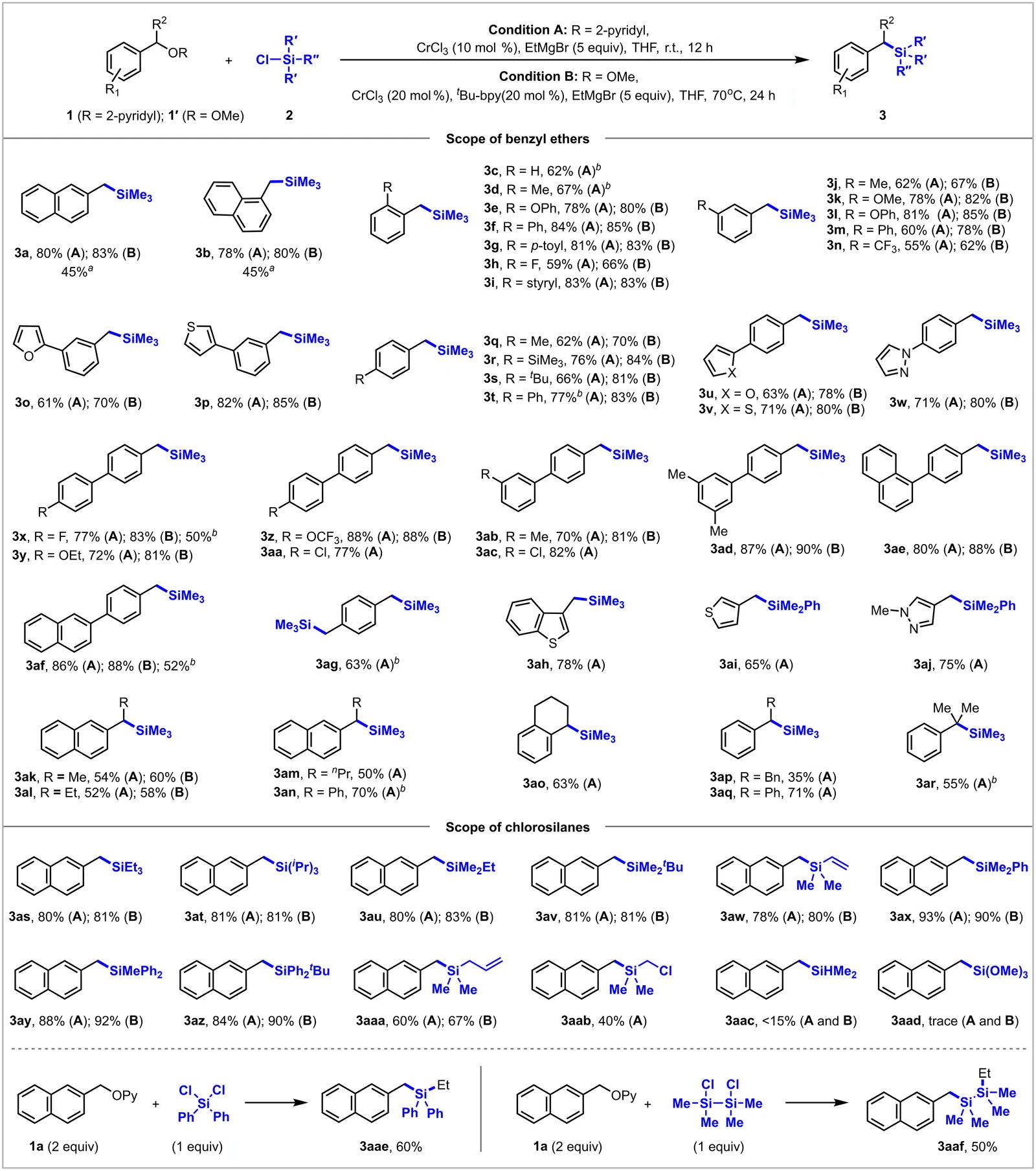
Deoxygenative silylation of (hetero)arylmethyl ethers and alcohols. Reaction condition **A**: **1** (0.2 mmol), **2** (0.8 mmol), CrCl_3_ (10 mol %), EtMgBr (1.0 mmol), and THF (2.0 ml), room temperature, 12 hours, isolated yields. Reaction condition **B**: **1′** (0.2 mmol), **2** (0.8 mmol), CrCl_3_ (20 mol %), *^t^*Bu_3_-bpy (20 mol %), EtMgBr (1.0 mmol), and THF (2.0 ml), room temperature, 12 hours, isolated yields. *^a^*70°C, 24 hours. *^b^*Alcohols **1″** (0.2 mmol), **2a** (1.0 mmol), CrCl_3_ (25 mol %), dtbpy (25 mol %), EtMgBr (1.2 mmol), and THF (2.0 ml), 70^o^C, 48 hours.

Having established the deoxygenative silylation of 2-naphthylmethyl alcohol and its ether derivatives **1a**–**1a″** with **2a**, we then investigated the silylation reactions of various (hetero)arylmethyl alcohol and their ether derivatives 1–1**″** with chlorosilanes **2**. Some representative results are summarized in Fig. 3. In addition to **1a**, 1-naphthylmethyl 2-pyridyl ether also reacted well with **2a**, affording the desired product **3b** in 78% yield. A wide range of substituted benzyl 2-pyridyl ethers also underwent the deoxygenative silylation with **2a** smoothly, affording the corresponding products **3c** to **3af** in 50 to 88% yields. Function groups such as OMe (**3k**), OPh (**3e** and **3l**), F (**3h** and **3x**), Cl (**3aa** and **3ac**), styryl (**3i**), CF_3_ (**3n**), OCF_3_ (**3z**), SiMe_3_ (**3r**), and aromatic heterocycles (**3o**, **3p**, **3u**, **3v**, and **3w**) were all well tolerated. Double C–O silylation of 1,4-bis[(pyridin-2-yloxy)methyl]benzene with **2a** furnished the disilylated product **3ag** in 63% yield. Heteroarylmethyl 2-pyridyl ethers such as benzo[b]thiophen-3-ylmethyl, thiophen-3-ylmethyl, and (1-methyl-1H-pyrazol-4-yl)methyl analogs were also suitable substrates, affording the corresponding products **3ah** to **3aj** in 63-to 78% yields. Notably, secondary 2-naphthylmethyl and benzyl 2-pyridyl ethers were also compatible, affording the desired products **3ak** to **3aq** in 35 to 70% yields. The sterically demanding tertiary 2-phenylpropan-2-yl 2-pyridyl ether also proved to be suitable substrate for the reaction with **2a**, affording the desired product **3a**r in 55% yield. Besides 2-pyridyl ethers, arylmethyl methyl ethers 1′ also reacted well with **2a** by CrCl_3_ in the presence of *^t^*Bu_3_-tpy at 70°C, affording the corresponding arylmethyl-substituted silanes in good yields. This catalytic system was also compatible with diverse functional groups such as alkyl, aryl, SiMe_3_, OMe, CF_3_, F, OCF_3_, and aromatic heterocycles. Several examples of arylmethyl alcohols 1″ were also tested, affording the desired products in moderate yields, along with the formation of the corresponding arylmethyl trimethylsilyl ethers.

The substrate scope of chlorosilanes 2 was then investigated. A series of alkyl-, phenyl-, and vinyl-substituted chlorosilanes proved to be effective for the reaction with **1a** and **1a′**, affording the corresponding silylation products **3as** to **3aaa** in good yields. Chloro(chloromethyl)dimethylsilane was also a suitable substrate for the reaction with **1a**, affording the desired product **3aab** in 40% yield. However, the reactions of chlorodimethylsilane and chloro(trimethoxy)silane with either **1a** or **1a′** were unsuccessful probably because of the electronic effect. Notably, in the reactions of dichlorosilanes with **1a**, one Si–Cl bond coupled selectively with the C–O bond, while the other Si–Cl bond reacted with EtMgBr, affording the 2-naphthylmethyl–substituted ethylsilanes **3aae** and **3aaf** in 75 and 50% yields, respectively.

**Fig. 4.**
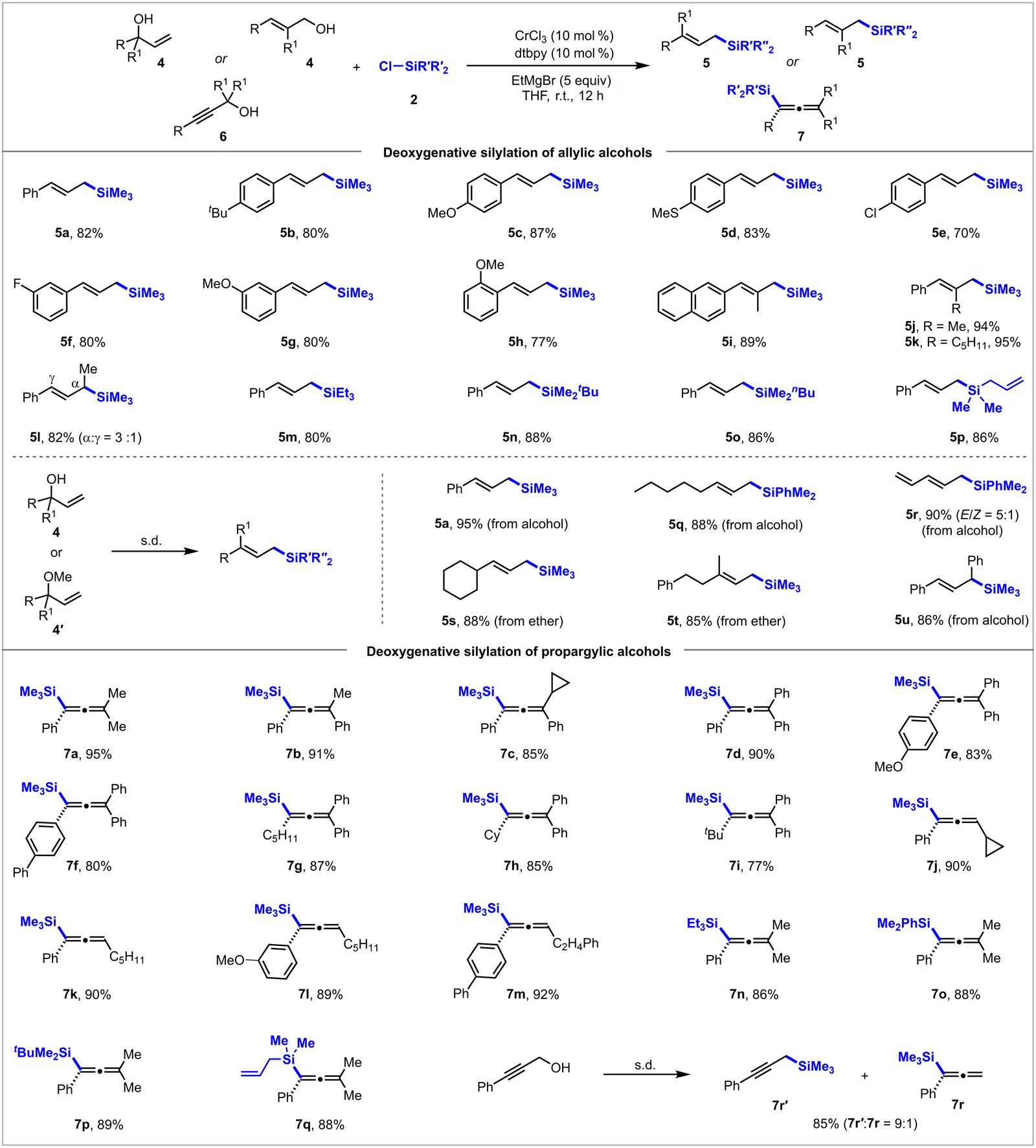
Deoxygenative silylation of allylic and propargylic alcohols. Reaction conditions: alcohols **4″** or **5″** (0.2 mmol), **2** (1.0 mmol), CrCl_3_ (10 mol %), dtbpy (10 mol %), EtMgBr (1.2 mmol), and THF (2.0 ml), room temperature, 12 hours, isolated yields.

Figure 4 summarizes the analogous deoxygenative silylation of allyl alcohols 4″ and propargyl alcohols 6″ with chlorosilanes **2** by CrCl_3_/dtbpy. Cinnamyl alcohols bearing diverse substituents at benzene ring all worked well with **2a**, affording the corresponding allylsilanes **5a**–**5h** in 70 to 87% yields with high stereoselectivity. Function groups such as OMe (**5c**, **5g**, and **5h**), SMe (**5d**), F (**5f**), and Cl (**5e**) were compatible. The α-methyl (5i and 5j) and -*n*-pentyl (5k) substituents did not hamper the reaction with **2a**, affording **5i** to **5k** in 89 to 95% yields. The secondary allylic alcohol (*E*)-4-phenylbut-3-en-2-ol also reacted efficiently with **2a**, affording the corresponding product **5l** in 82% yield with regioselectivity (3:1) favoring the less hindered site. In addition to **2a**, chlorotriethyl-, *tert*-butylchlorodimethyl-, *n*-butylchlorodimethyl, and allylchlorodimethylsilanes were also compatible for the reactions with **4a**, affording the corresponding products **5m** to **5p** in 80 to 88% yields. The reaction of 1-vinyl–substituted alcohols or their methyl ethers occurred selectively at the less hindered site, yielding the corresponding terminal allylsilanes **5a** and **5q** to **5t** in 85 to 95% yields. (*E*)-1,3-Diphenylprop-2-en-1-ol also reacted well with **2a** to give **5u** in 86% yields. The analogous reactions of propargyl alcohols **6″** with **2** were then examined. A series of propargyl alcohols with diverse substitution at the alkynyl or propargyl positions reacted well with **2a** to furnish tetra- and trisubstituted silylallenes **7a** to **7m** in 72 to 91% yields. Chlorotriethyl-, chlorodimethylphenyl, *tert*-butylchlorodimethyl- and allylchlorodimethylsilanes were also suitable for the reaction with **6a″**, affording **7n** to **7q** in 86 to 89% yields. The reaction of primary propargyl alcohol 3-phenylprop-2-yn-1-ol with **2a** gave the corresponding propargyl silane **7r′** as the major product, probably because of steric effect.

**Fig. 5.**
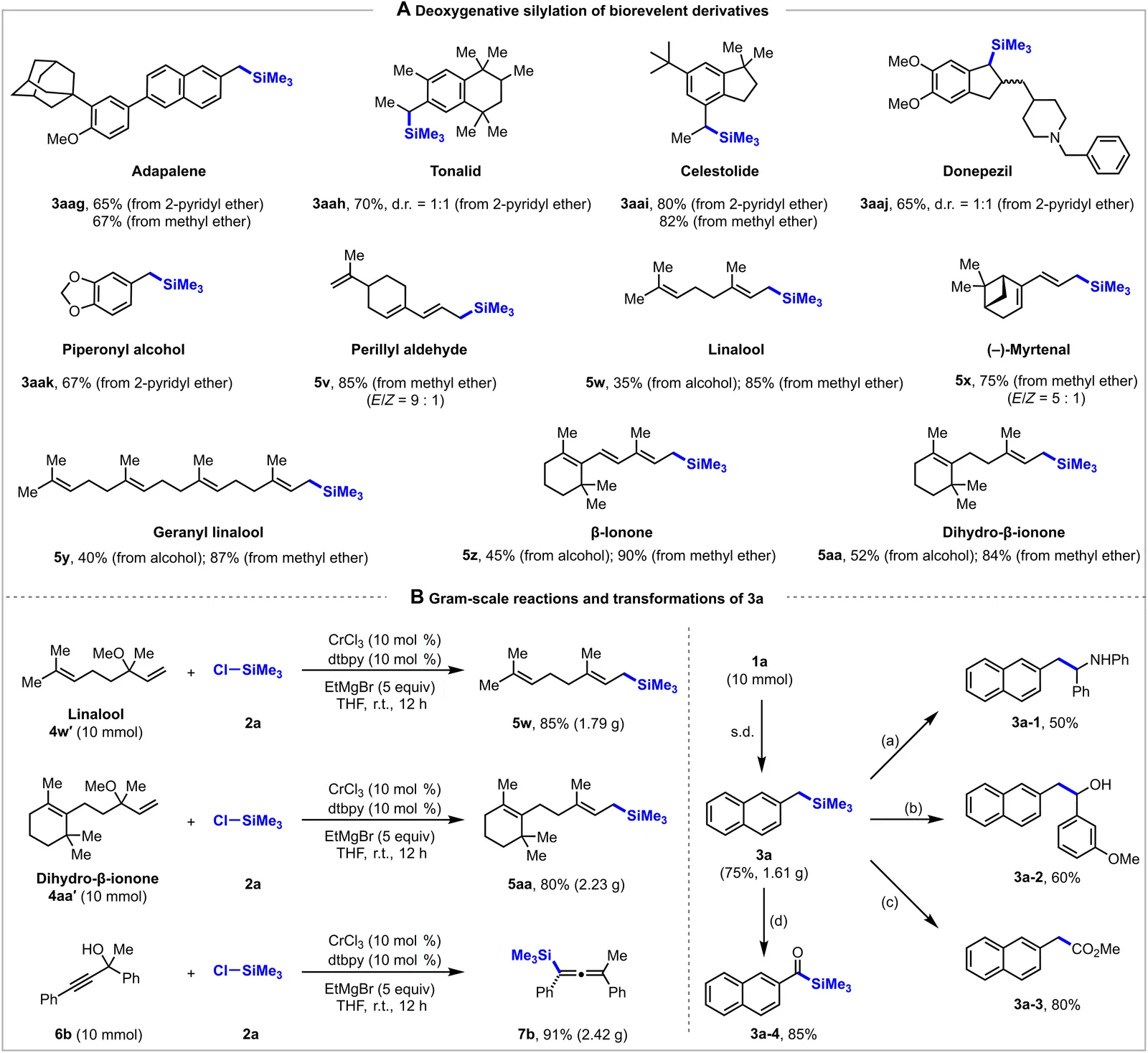
Deoxygenative silylation of biorelevant derivatives, gram-scale reactions, and transformations of 3a. (**A**) Deoxygenative silylation of biorevelent derivatives. (**B**) Gram-scale reactions and transformations of **3a**. For detailed conditions regarding the silylation of biorelevant derivatives and gram-scale reactions, see the Supplementary Materials. Reaction conditions for transformations of **3a**: (a) **3a** (0.2 mmol), (*E*)-*N*,1-diphenylmethanimine (0.15 mmol), TBAF (5 mol %), 4-Å MS (100 mg), and THF (1 ml), 80°C, 12 hours; (b) 3-Methoxybenzaldehyde (0.4 mmol), TBAT (5 mol %), and THF (1 ml), 80°C, 6 hours; (c) CO_2_ (1 atm), CsF (0.6 mmol), DMF (1 ml), 100°C, 4 hours, and then MeI (2 equiv), Cs_2_CO_3_ (2 equiv), r.t., 30 min; (d) NBS (0.4 mmol), AIBN (5 mol %), benzene (1 ml), 80°C, 12 hours, bad then AgOAc (0.4 mmol), H_2_O:Acetone:EtOH = 1:2:3 (2 ml), r.t., 24 hours.

This chromium-catalyzed silylation methodology could be applicable to the direct functionalization and late-stage modification of natural products, pharmaceuticals, and their derivatives, as demonstrated with adapalene, tonalid, celestlide, donepezil, piperonyl alcohol, perillyl aldehyde, linalool, myrtenal, geranyl linalool, β-ionone, and dihydro-β-ionone (Fig. 5A). To further highlight the practical value of the present protocol, some gram-scale reactions were carried out (Fig. 5B). The reactions of 10 mmol of allylic methyl ethers derived from linalool and dihydro-β-ionone with **2a** afforded the desired products **5w** and **5aa** in 85 and 80% yields, respectively. Similarly, the reactions of 10 mmol of propargyl alcohol **6b** with **2a** under the standard conditions yielded the tetrasubstituted silylallene **7b** in 91% yield. The reaction of 10 mmol of **1a** with **2a** under standard conditions proceeded efficiently to afford the desired product **3a** in 75% yield. Compound **3a** was readily converted into secondary amine (**3a-1**), secondary alcohol (**3a-2**), methyl 2-(naphthalen-2-yl)acetate (**3a-3**), and 2-naphthyl(trimethylsilyl)methanone (**3a-4**).

**Fig. 6.**
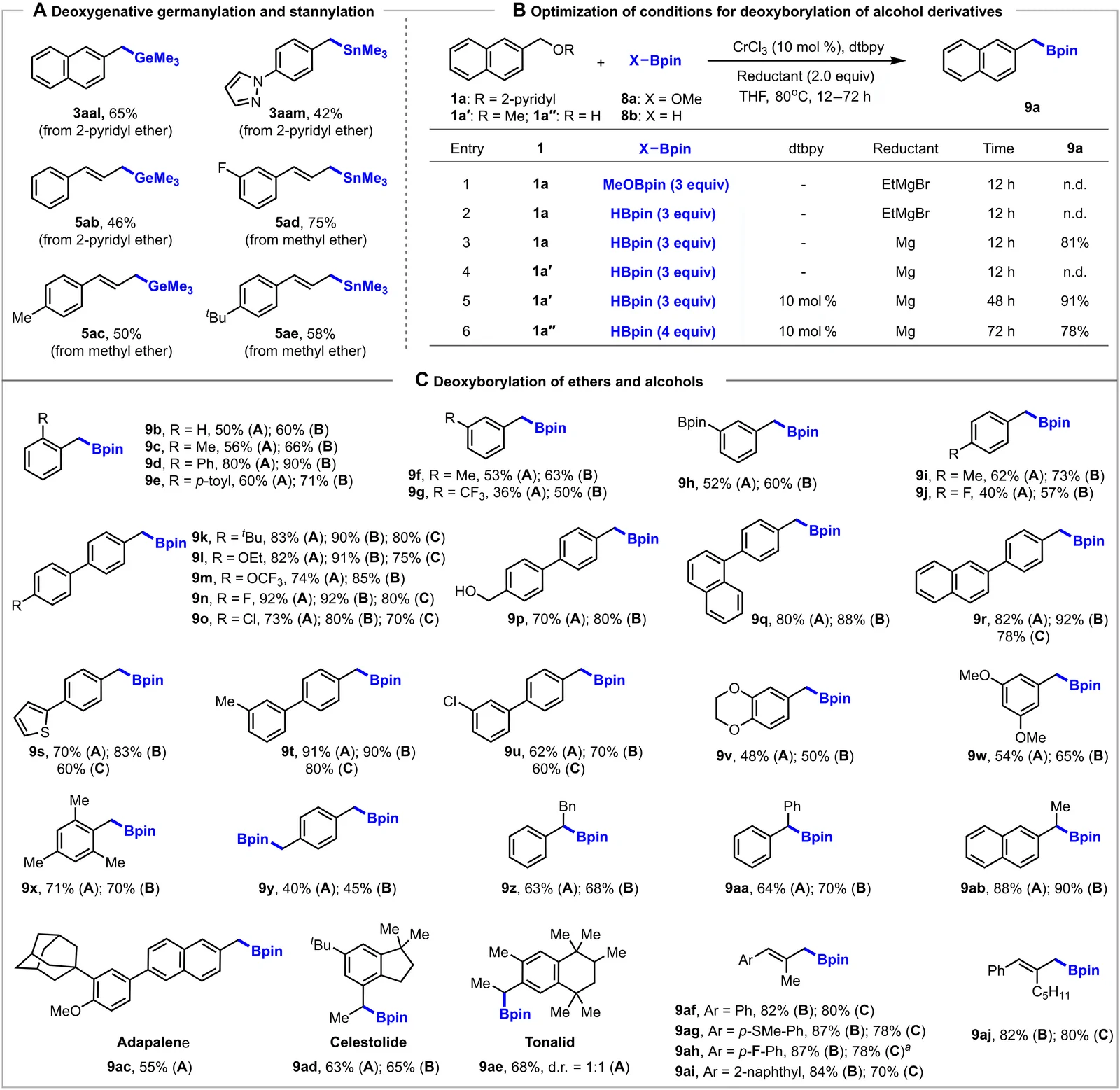
Deoxygenative germanylation, stannylation, and borylation of ethers and alcohols. (**A**) Deoxygenative germanylation and stannylation. (**B**) Optimization of conditions for deoxyborylation of alcohol derivatives. (**C**) Deoxyborylation of ethers and alcohols. Reaction conditions for the germanylation and stannylation of ethers: ethers (0.2 mmol), chlorotrimethylgermane or chlorotrimethylstannane (0.8 mmol), CrCl_3_ (10 mol %), dtbpy (10 mol %), EtMgBr (1.0 mmol), and THF (2.0 ml), 50°C, 12 hours, isolated yields. Reaction conditions for the deoxyborylation of ethers and alcohols, condition **A**: 2-pyridyl ethers 1 (0.2 mmol), HBpin (0.6 mmol), CrCl_3_ (10 mol %), Mg (0.4 mmol), and THF (2 ml), 80°C, 12 hours, isolated yields; condition **B**: methyl ethers 1′ and 4′ (0.2 mmol), HBpin (0.6 mmol), CrCl_3_ (10 mol %), dtbpy (10 mol %), Mg (0.4 mmol), and THF (2 ml), 80°C, 48 hours, isolated yields; condition **C**: alcohols (0.2 mmol), HBpin (0.8 mmol), CrCl_3_ (10 mol %), dtbpy (10 mol %), Mg (0.6 mmol), and THF (2 ml), 80°C, 72 hours, isolated yields. *^a^E*/*Z* = 2:1.

The analogous deoxygenative functionalization of alcohols and its ether derivatives with other electrophiles was then investigated (Fig. 6). As shown in Fig. 6A, chlorotrimethylgermane and chlorotrimethylstannane were also suitable electrophiles for the present deoxygenative functionalization, affording the corresponding organogermanium and organostannane compounds in 42 to 75% yields. However, boron electrophiles such as methyl pinacolyl borate (MeOBpin) and pinacolboane (HBpin) were incompatible for the deoxyborylation with **1a** under CrCl_3_/EtMgBr conditions (Fig. 6B, entries 1 and 2). To our delight, the deoxyborylation of **1a** with HBpin was successfully achieved by switching the reductant from EtMgBr to Mg, affording the corresponding product **9a** in 81% yield (Fig. 6B, entry 3). The reaction of the analogous methyl ether **1a′** with HBpin also worked well in the presence of dtbpy ligand and Mg reductant (*79*, *80*), giving the desired product **9a** in 91% yield; however, no product was formed in its absence (Fig. 6B, entries 4 and 5). The direct deoxyborylation of the free alcohol **1a″** with HBpin was also achieved, delivering **9a** in 78% yield (Fig. 6B, entry 6), albeit over a longer reaction time. Notably, this chromium-catalyzed deoxyborylation protocol is scalable and can be applied to the gram-scale synthesis of **9a** (see the Supplementary Materials).

As shown in Fig. 6C, the deoxyborylation of various alcohols and ethers with HBpin was also examined. A wide range of arylmethyl 2-pyridyl ethers reacted well with HBpin, affording the corresponding deoxyborylation products **9b** to **9y** in 40 to 92% yields. Function groups such as OMe (**9w**), OEt (**9l**), OH (**9p**), F (**9j** and **9n**), Cl (**9o** and **9u**), CF_3_ (**9g**), OCF_3_ (**9m**), Bpin (**9h**), and heterocycles (**9s** and **9v**) were compatible. Notably, the reactions of secondary benzyl and 2-naphthylmethyl 2-pyridyl ethers also underwent smoothly to afford the desired borylation products **9z** to **9ab** in 63 to 88% yields. Moreover, arylmethyl methyl ethers and alcohols are also compatible, affording the corresponding borylation products in good yields. This borylation methodology was also applicable to the late-stage functionalization of biorelevant molecules such as adapalene (**9ac**), celestolide (**9ad**), and tonalid (**9ae**). The reactions of allyl methyl ethers and alcohols also worked well, yielding the corresponding allyl boronic esters **9af** to **9aj** in good yields with excellent stereoselectivity (*81*–*86*).

**Fig. 7.**
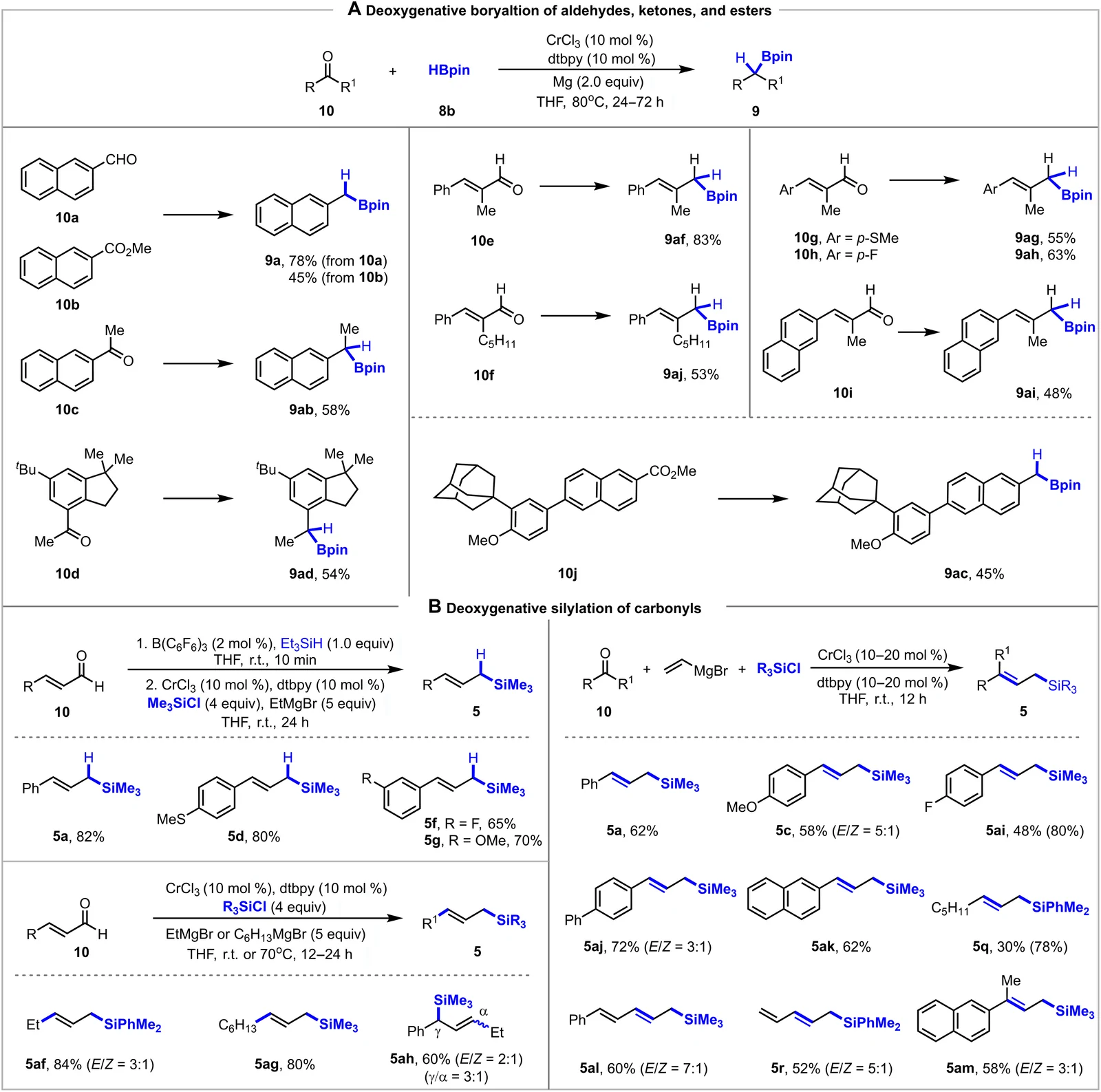
Deoxygenative silylation and borylation of carbonyl compounds. (**A**) Deoxygenative borylation of aldehydes, ketones, and esters. (**B**) Deoxygenative silylation of carbonyls. For detailed conditions, see the Supplementary Materials.

Building on the insight that in situ–generated borate ester intermediate ROBpin is a key intermediate for the deoxyborylation of alcohols with HBpin (*87*, *88*), we extended this chromium-catalyzed strategy to the deoxyborylation of aldehydes, ketones, and esters using HBpin as both an in situ–activating reagent and electrophile (Fig. 7A) (*89*–*94*). As anticipated, 2-naphthaldehyde and methyl 2-naphthoate proved to be suitable substrates, affording the corresponding deoxyborylation product **9a** in 78 and 45% yields, respectively. The deoxyborylation of 2-acetylnaphthalene **10c** and celestolide **10d** also worked well, giving the desired borylated product **9ab** and **9ad** in 58 and 54% yields, respectively. The methyl ester derived from adapalene was also converted to the corresponding borylated product **9ac** in 50% yield. Furthermore, substituted cinnamaldehydes were also compatible with this chromium-catalyzed deoxyborylation protocol, affording the corresponding allyl boronic esters **9af** to **9aj** in synthetic useful yields.

We subsequently explored the deoxygenative silylation of diverse aldehydes by leveraging their in situ conversion into reactive silyl ether intermediates (Fig. 7B). Using a B(C_6_F_5_)_3_-catalyzed hydrosilylation to generate silyl ethers in situ, the deoxygenative silylation of cinnamaldehydes was successfully achieved, affording the corresponding allylsilanes **5a** to **5g** in good yields. The reaction of acrylaldehyde with chlorosilanes in the presence of EtMgBr or *^n^*HexylMgBr proceeded efficiently to afford the deoxygenative silylation products **5af** and **5ag** in 84 and 85% yields, respectively. This reaction proceeds through nucleophilic addition of EtMgBr or *^n^*HexylMgBr to the carbonyl group, generating a silyl ether intermediate in situ. Similarly, cinnamaldehyde also reacted well with **2a**, affording the corresponding product **5ah** in 60% yield with moderate regio- and stereoselectivity. Notably, the deoxygenative silylation of aromatic and aliphatic aldehydes with **2a** using vinyl magnesium bromide instead of EtMgBr also underwent smoothly via in situ generation of 1-vinyl–substituted silyl ethers, affording the corresponding allylsianes **5a** to **5am** in good yields. Enhanced activity was achieved by using 1.2 equiv of vinylMgBr as an initiator, followed by the addition of EtMgBr (**5ai**, 48 versus 80% and **5q**, 30 versus 78%). Similarly, the reaction of 2-acetylnaphthalene **10c** with **2a** also worked well by using 1.2 equiv of vinylMgBr as an initiator, followed by the addition of EtMgBr as the reductant, affording the desired product **5am** in 58% yield.

### Mechanistic investigations

**Fig. 8.**
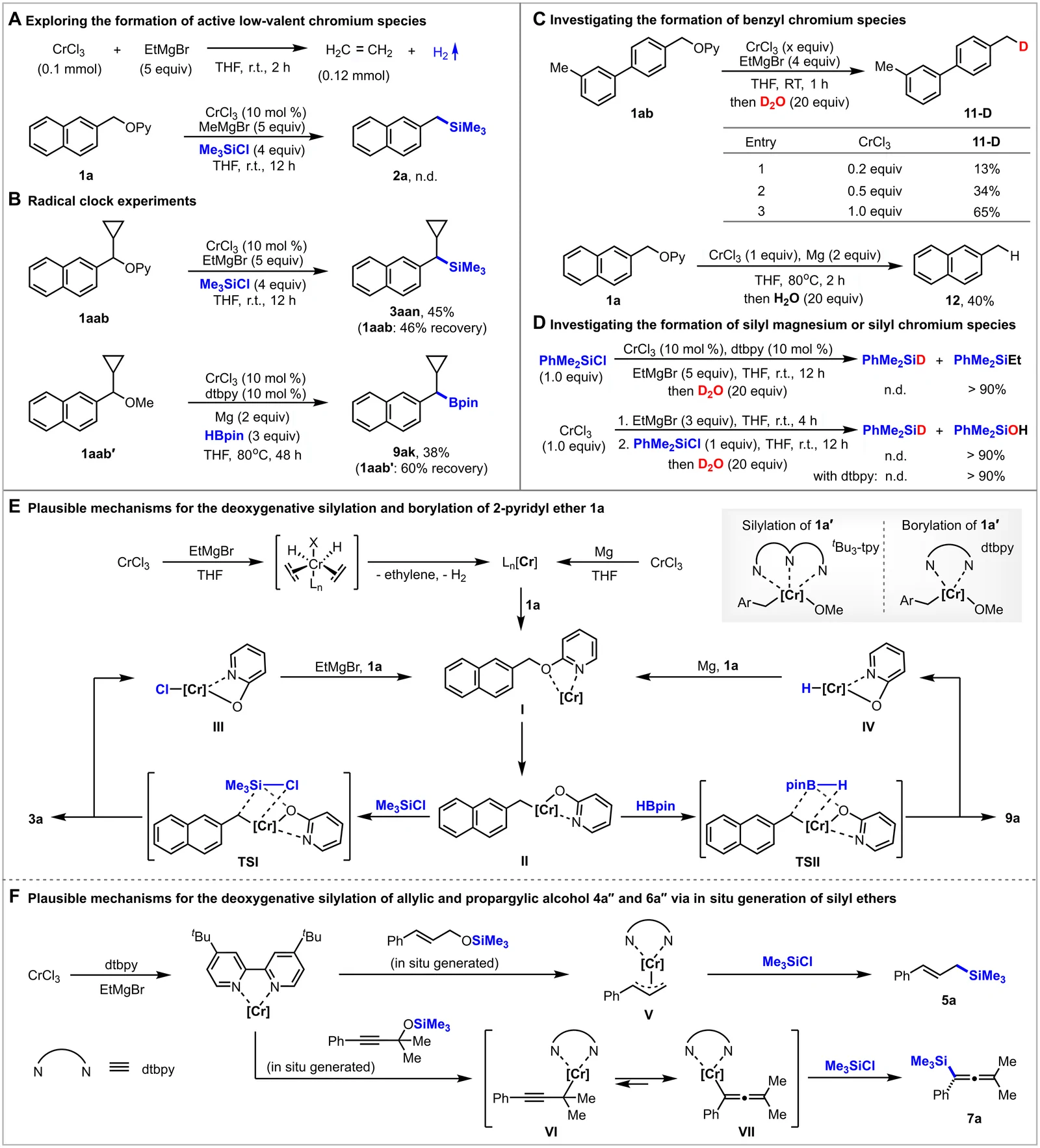
Mechanistic studies. (**A**) Exploring the formation of active low-valent chromium species. (**B**) Radical clock experiments. (**C**) Investigating the formation of benzyl chromium species. (**D**) Investigating the formation of silyl magnesium or silyl chromium species. (**E**) Plausible mechanisms for the deoxygenative silylation and borylation of 2-pyridyl ether **1a**. (**F**) Plausible mechanisms for the deoxygenative silylation of allylic and propargylic alcohol **4a″** and **6a″** via in situ generation of silyl ethers.

To gain information on the reaction mechanism, several mechanistic experiments were conducted. The reaction of CrCl_3_ with EtMgBr at room temperature for 2 hours released ethylene and hydrogen gas, as detected by gas chromatography–mass spectrometry (Fig. 8A, top). No substantial silylation was observed between **1a** and **2a** when using MeMgBr as the reductant (Fig. 8A, bottom). These observations indicate that a low-valent chromium species might be formed via transmetalation of CrCl_3_ followed by β-hydride elimination and hydride reduction (*95*). The radical clock experiments were then conducted to determine whether a radical pathway was involved in this deoxygenative functionalization reaction (Fig. 8B). The reactions of α-cyclopropyl–substituted 2-naphthylmethyl ethers afforded the corresponding silylation and borylation products **3aan** and **9ak** in 45 and 38% yields, respectively, with no observed ring-opening products. These results indicate that a single-electron transfer pathway might be not involved in this chromium-catalyzed deoxygenative functionalization. Treatment of **1a** with 0.2, 0.5, or 1.0 equiv of CrCl_3_ and 4.0 equiv of EtMgBr, followed by quenching with D_2_O, gave the corresponding deuterated product **11-D** in 13, 34, and 65% yields, respectively (Fig. 8C, top). The stoichiometric reaction of **1a** with 1.0 equiv of CrCl_3_ and 2.0 equiv of Mg at 80°C gave 2-methylnaphthalene **12** in 40% yield (Fig. 8C, bottom). These results suggest that an organochromium species might be generated in this chromium-catalyzed deoxygenative functionalization. In the reaction of chlorodimethyl(phenyl)silane with EtMgBr catalyzed by CrCl_3_/dtbpy, no deuterated dimethyl(phenyl)silane was observed (Fig. 8D), suggesting that a silyl magnesium reagent might be not generated in this reaction. The treatment of CrCl_3_ with EtMgBr, followed by addition of chlorodimethyl(phenyl)silane and subsequent quenching with D_2_O, did not yield deuterated dimethyl(phenyl)silane. This result indicates that a silyl chromium species might not be involved in this deoxygenative silylation reaction.

On the basis of the above experimental results and previous reports (*58*, *65*, *72*, *73*), possible mechanisms for the deoxygenative silylation and borylation of 2-pyridyl ether **1a**, and silylation of allylic and propargylic alcohol **4a″** and **5a″** are proposed as shown in Fig. 8. The coordination of the pyridyl species in **1a** to the low-valent chromium species, which was generated from the reduction of CrCl_3_ with EtMgBr or Mg, followed by two-electron oxidative addition of the C–O bond of **1a** by low-valent chromium would form an alkyl chromium intermediate **II**. The substitution reaction between **II** and **2a** via a transition state **TSI** would generate the product **3a** and a chromium species **III** (*48*, *72*). The reduction of **III** with EtMgBr followed by the coordination to **1a** would yield the intermediate **I** to restart the catalytic cycle (Fig. 8E). In the case of borylation with HBpin, the σ-bond metathesis between **II** and HBpin via a transition state **TSII** would occur to yield the product **9a** and a chromium-hydride species **IV** (Fig. 8E). The chromium hydride species **IV** was further validated by the formation of deoxygenative protonation byproducts from certain incompatible substrates (see the Supplementary Materials). For substrate **1a′**, *^t^*Bu_3_-tpy and dtbpy promoted the deoxygenative silylation and borylation by stabilizing the alkyl chromium intermediate (see the Supplementary Materials for the possible reaction mechanisms of the silylation and borylation of methyl ether **1a′**). In the reaction of cinnamyl alcohol **4a″**, the C−O bond cleavage of in situ–generated silyl ether by a dtbpy-ligated low-valent chromium species would give a π-allyl chromium intermediate **IV** (*96*, *97*). The substitution reaction between **IV** and **2a** favored at less steric site, affording the product **5a** (Fig. 8F). In the case of propargyl alcohol **5a″**, the C−O bond cleavage of in situ–generated silyl ether by a dtbpy-ligated low-valent chromium species would yield a propargyl chromium species **VI**, which would undergo a reversible 1,3-metal migration to give an allenyl chromium species **VII** (*96*). The substitution reaction between the allenyl chromium species **VII** and **2a** would selectively take place because of the steric reason, giving the desired allenylsilane product **7a** (Fig. 8F).

## DISCUSSION

In summary, we have developed an efficient, concise, and general chromium-catalyzed strategy for the deoxygenative functionalization of alcohols, ethers, carbonyls, and esters with a number of electrophiles. This strategy couples these oxygenated functionalities with a number of electrophiles, providing an efficient and straightforward route for the synthesis of a broad range of synthetically valuable organosilicon, organogermanium, organostannane, and organoboron building blocks. The utility of this efficient chromium-catalyzed protocol has also been demonstrated by reaction scale-up and the direct functionalization and late-stage modification of various biorelevant molecules. Mechanistic studies indicate that this deoxygenative functionalization reaction proceeds through two-electron oxidative addition of the C–O bond to a low-valent chromium species, generating an organochromium intermediate that subsequently undergoes reaction with a number of electrophiles to forge C–Si, C–Ge, C–Sn, and C–B bonds. This work not only establishes an efficient and unified platform for converting ubiquitous oxygenated compounds into diverse organosilicon, organogermanium, organostannane, and organoboron building blocks but also opens up a promising frontier for the study of chromium catalysis. Further studies on low-valent chromium catalysis are in progress.

## MATERIALS AND METHODS

### General procedure for the deoxygenative silylation of arylmethyl 2-pyridyl ethers with chlorosilanes by chromium

A dried Schlenk tube was charged with 2-pyridyl ethers (0.2 mmol), CrCl_3_ (10 mol %), and chlorosilanes (0.8 mmol), followed by adding a freshly distilled tetrahydrofuran (THF, 1 ml) by syringe under atmosphere of nitrogen. After stirring at room temperature for 5 min, EtMgBr (1.0 M in THF, 1 mmol) was added dropwise, and the reaction mixture was stirred at room temperature for 12 hours. After being quenched by aqueous NH_4_Cl (2 ml), the crude product was extracted with ethyl acetate (3 × 10 ml). The combined organic phases were dried over anhydrous Na_2_SO_4_ and concentrated under vacuum. The residue was purified by flash chromatography on silica gel to give the desired product.

### General procedure for the deoxygenative silylation of arylmethyl methyl ethers with chlorosilanes by chromium in the presence of ligand

A dried Schlenk tube was charged with methyl ethers (0.2 mmol), CrCl_3_ (20 mol %), ligand (20 mol %), and chlorosilanes (0.8 mmol), followed by adding a freshly distilled THF (1 ml) by syringe under atmosphere of nitrogen. After stirring at room temperature for 5 min, EtMgBr (1.0 M in THF, 1 mmol) was added dropwise, and the reaction mixture was stirred at 70°C for 24 hours. After being cooled to room temperature and quenched by aqueous NH_4_Cl (2 ml), the crude product was extracted with ethyl acetate (3 × 10 ml). The combined organic phases were dried over anhydrous Na_2_SO_4_ and concentrated under vacuum. The residue was purified by flash chromatography on silica gel to give the desired product.

### General procedure for the deoxygenative silylation of arylmethyl alcohols with by chromium in the presence of *^t^*Bu_3_-tpy

A dried Schlenk tube was charged with arylmethyl alcohols (0.2 mmol), CrCl_3_ (25 mol %), *^t^*Bu_3_-tpy (25 mol %), and chlorosilanes (1.0 mmol), followed by adding a freshly distilled THF (0.8 ml) by syringe under atmosphere of nitrogen. After stirring at room temperature for 5 min, EtMgBr (1.0 M in THF, 1.2 mmol) was added dropwise, and the reaction mixture was stirred at 70°C for 24 hours. After being cooled to room temperature and quenched by aqueous NH_4_Cl (2 ml), the crude product was extracted with ethyl acetate (3 × 10 ml). The combined organic phases were dried over anhydrous Na_2_SO_4_ and concentrated under vacuum. The residue was purified by flash chromatography on silica gel to give the desired product.

### General procedure for the deoxygenative silylation of allyl and propargyl alcohols with by chromium in the presence of dtbpy

A dried Schlenk tube was charged with allyl and propargyl alcohols (0.2 mmol), CrCl_3_ (10 mol %), dtbpy (10 mol %), and chlorosilanes (1.0 mmol), followed by adding a freshly distilled THF (0.8 ml) by syringe under atmosphere of nitrogen. After stirring at room temperature for 5 min, EtMgBr (1.0 M in THF, 1.2 mmol) was added dropwise, and the reaction mixture was stirred at room temperature for 12 hours. After being quenched by aqueous NH_4_Cl (2 ml), the crude product was extracted with ethyl acetate (3 × 10 ml). The combined organic phases were dried over anhydrous Na_2_SO_4_ and concentrated under vacuum. The residue was purified by flash chromatography on silica gel to give the desired product.

### General procedure for the deoxyborylation of 2-pyridyl ethers with HBpin by chromium

A dried Schlenk tube was charged with 2-pyridyl ethers (0.2 mmol), CrCl_3_ (10 mol %), Mg (0.4 mmol), and HBpin (0.6 mmol), followed by adding a freshly distilled THF (2 ml) by syringe under atmosphere of nitrogen. The reaction mixture was stirred at 80°C for 12 hours. After being cooled to room temperature, ethyl acetate (2 ml) was added into the reaction mixture and concentrated under vacuum. The residue was purified by flash chromatography on silica gel to give the desired product.

### General procedure for the deoxyborylation of methyl ethers or alcohols with HBpin by chromium in the presence of dtbpy

A dried Schlenk tube was charged with methyl ethers or alcohols (0.2 mmol), CrCl_3_ (10 mol %), dtbpy (10 mol %), Mg (0.4 mmol), and HBpin (0.6 mmol), followed by adding a freshly distilled THF (2 ml) by syringe under atmosphere of nitrogen. The reaction mixture was stirred at 80°C for 48 hours. After cooled room temperature, ethyl acetate (2 ml) was added into the reaction mixture and concentrated under vacuum. The residue was purified by flash chromatography on silica gel to give the desired product.

## Supplementary Materials

### This PDF file includes:

Supplementary Text Tables S1 to S8 Figs. S1 and S2 NMR Spectra References
