# Supplementary material for: Deoxygenative functionalization of alcohols, ethers, and carbonyl compounds by chromium catalysis
Source: Sci Adv. 2026 Sep 25;12(39):eaeg4594. doi: 10.1126/sciadv.aeg4594 (PMC13614368; doi:10.1126/sciadv.aeg4594)
Supplement: Supplementary file 1 [file sciadv.aeg4594_sm.pdf]

Science Advances
AAAS

Supplementary Materials for

Deoxygenative functionalization of alcohols, ethers, and carbonyl compounds by chromium catalysis

Wendi Chang *et al.*

Corresponding author: Changpeng Chen, cpchen@qust.edu.cn; Xiaoming Zeng, zengxiaoming@scu.edu.cn; Xuefeng Cong, xuefengcong@tju.edu.cn

Sci. Adv. **12**, eaeg4594 (2026)
DOI: 10.1126/sciadv.aeg4594

This PDF file includes:

Supplementary Text
Tables S1 to S8
Figs. S1 and S2
NMR Spectra
References

1. General and Materials

General. All manipulations of air- and moisture-sensitive compounds were performed under dry nitrogen atmosphere in a glovebox. Analytical thin-layer chromatography was performed on glass plates coated with 0.25 mm 230–400 mesh silica gel containing a fluorescent indicator (Merck). Flash silica gel column chromatography was performed on silica gel 60N (spherical and neutral, 140–325 mesh) as described by Still. All ^{1}H and ^{13}C NMR spectra of organic products were recorded on Bruker AVANCE III HD 400 NMR, JEOL JNM-ECS400 or JEOL JNM-ECS600 instrument. ^{1}H NMR spectra were recorded at 400 or 600 MHz in $CDCl_3$ were referenced internally to tetramethylsilane as a standard, and ^{13}C NMR spectra were recorded at 100 or 126 MHz and referenced to the solvent resonance. The data are reported as follows: chemical shift (δ ppm), multiplicity (s = singlet, d = doublet, t = triplet, q = quartet, m = multiplet, dd = doublet of doublets, dt = doublet of triplets, brs = broad singlet, coupling constant(s) in Hz, integration). Analytical gas chromatography (GC) was carried out on a Thermo Trace 1300 gas chromatograph or TECHCOMP GC-7900 (for analysis of hydrogen evolution), equipped with a flame ionization detector. High Resolution Mass Spectra were obtained on a Bruker microTOF-Q III (Ion source: EI, APCI, or FD).

Materials. Unless otherwise noted, materials were purchased from Tokyo Chemical Industry Co., Alfa Aesar, Adamas, and other commercial suppliers and used as received. Solvents were dried over sodium or CaH_2 by refluxing for overnight and freshly distilled prior to use. Mg (99.99 %) was purchased from Aldrich Inc. Benzyl, allyl and propargyl ethers (72,76,98-100) are synthesized according the known procedures.

2. Optimizing Reaction Parameters

Table S1. Investigation of the effect of *leaving group* for deoxygenative silylation of 2-naphthylmethyl ethers 1 with chlorotrimethylsilane 2a by chromium[a]

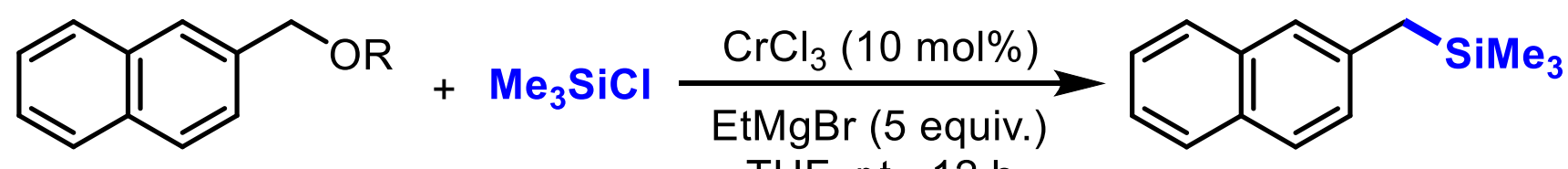

1 2a 3a

Entry	R	Yield (**3a**)
1	Me	0
2	Ph	0
3	2-Py	80%

4	3-Py	9%
5	4-Py	0

[a]Conditions: **1** (0.2 mmol), Me_3SiCl (0.8 mmol), EtMgBr (1 mmol), $CrCl_3$ (0.02 mmol), THF (1 mL), room temperature, 12 h. Isolated yields were given.

Table S2. Investigation of the effect of *amount of Grignard reagent* for deoxygenative silylation of 1a with 2a by chromium[a]

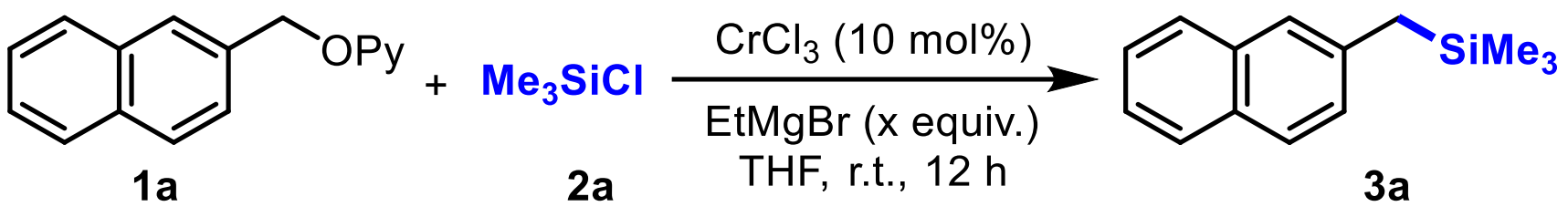

Entry	EtMgBr (equiv.)	Yield (**3a**)
1	0	0
2	2	11%
3	3	41%
4	4	63%
5	5	80%
6	6	78%

[a]Conditions: **1a** (0.2 mmol), Me_3SiCl (0.8 mmol), EtMgBr (x mmol), $CrCl_3$ (0.02 mmol), THF (1 mL), room temperature, 12 h. Isolated yields were given.

Table S3. Investigation of the effect of *amount of Me_3SiCl* for deoxygenative silylation of 1a with 2a by chromium[a]

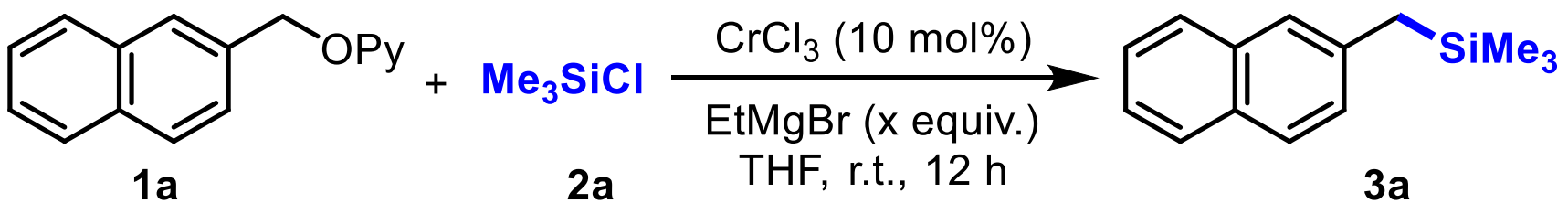

Entry	Me_3SiCl (equiv.)	Yield (**3a**)
1	-	0
2	2	44%
3	3	62%
4	4	80%
5	5	80%

[a]Conditions: **1a** (0.2 mmol), Me_3SiCl (0-1 mmol), EtMgBr (1 mmol), $CrCl_3$ (0.02 mmol), THF (1 mL), room temperature, 12 h. Isolated yields were given.

Table S4. Investigation of the effect of *metal catalyst* for deoxygenative silylation of 1a with 2a[a]

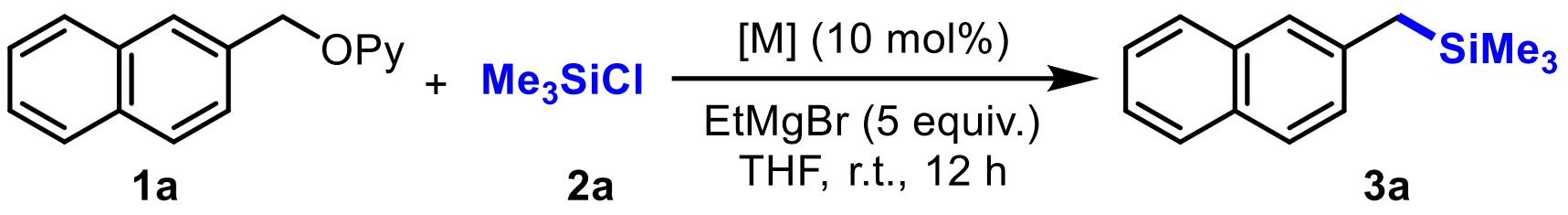

Entry	[M]	Yield (**3a**)
1	$CrCl_3$	80%
2	$CrCl_2$	78%
3	$Cr(acac)_3$	61%
4	$FeCl_3$	36%
5	$CoCl_3$	38%
6	$NiCl_2$	39%
7	-	0

[a]Conditions: **1a** (0.2 mmol), Me_3SiCl (0.8 mmol), EtMgBr (1 mmol), [M] (0.02 mmol), THF (1 mL), room temperature, 12 h. Isolated yields were given.

Table S5. Investigation of the effect of *metal catalyst* for deoxyborylation of 1a with 8b[a]

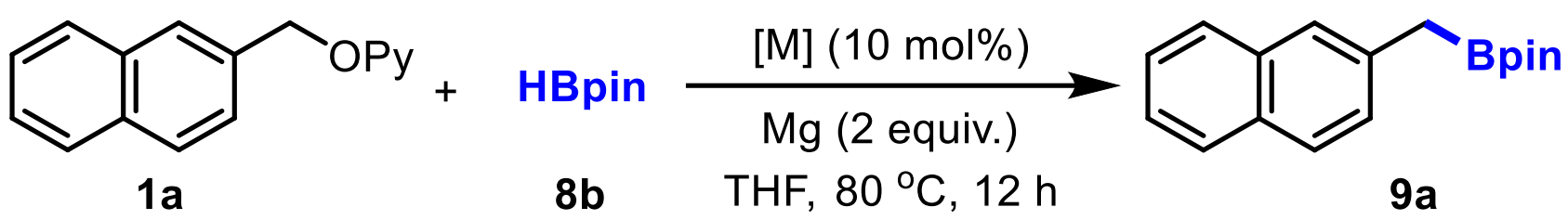

Entry	[M]	Yield (**9a**)
1	-	0
2	$CrCl_2$	79%
3	$CrCl_3$	81%
4	$Cr(acac)_3$	0
5	$FeCl_2$	20%
6	$PdCl_2$	0
7	$NiCl_2$	30%
8	$CoCl_2$	17%

[a]Conditions: **1a** (0.2 mmol), HBpin (0.6 mmol), [M] (0.02 mmol), Mg (0.4 mmol), THF (1 mL), 80 °C, 12 h. Isolated yields were given.

Table S6. Investigation of the effect of *amount of Mg* for deoxyborylation of 1a with 8b[a]

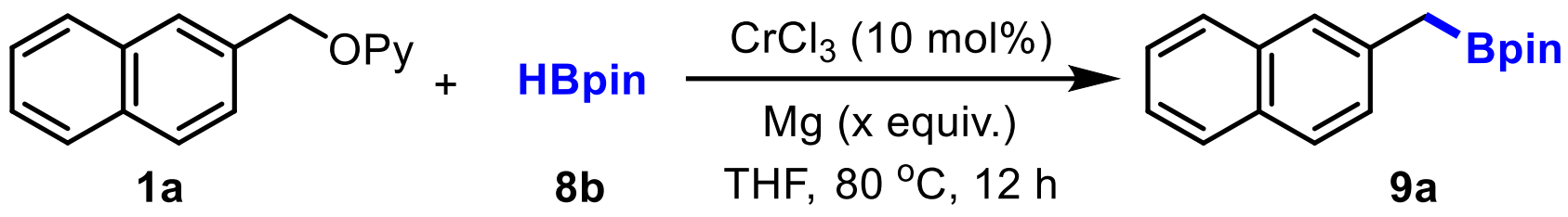

Entry	Mg (equiv.)	Yield (**9a**)
1	0	0
2	1	49%
3	1.5	77%
4	2	81%
5	2.5	80%

[a]Conditions: **1a** (0.2 mmol), HBpin (0.6 mmol), $CrCl_3$ (0.02 mmol), Mg (x mmol), THF (1 mL), 80 °C, 12 h. Isolated yields were given.

Table S7. Investigation of the effect of *amount of 8b* for deoxyborylation of 1a with 8b[a]

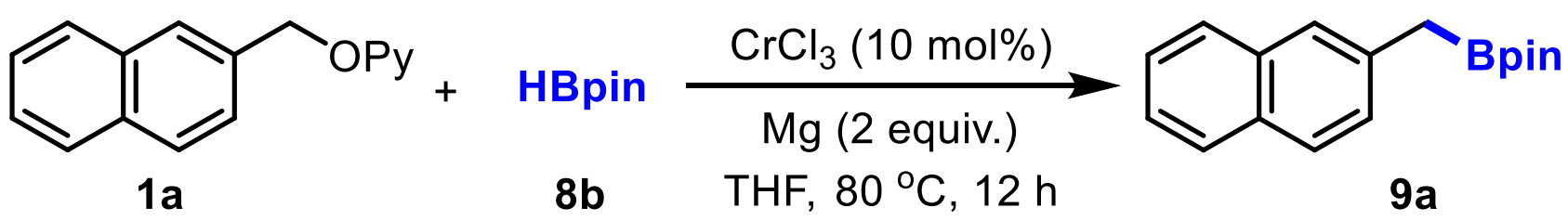

Entry	HBpin (equiv.)	Yield (**9a**)
1	1.5	51%
2	2	61%
3	2.5	74%
4	3	81%
5	3.5	79%

[a]Conditions: **1a** (0.2 mmol), HBpin (0.3-0.7 mmol), $CrCl_3$ (0.02 mmol), Mg (0.4 mmol), THF (1 mL), 80 °C, 12 h. Isolated yields were given.

Table S8. Investigation of the effect of *reaction temperature* for deoxyborylation of 1a with 8b[a]

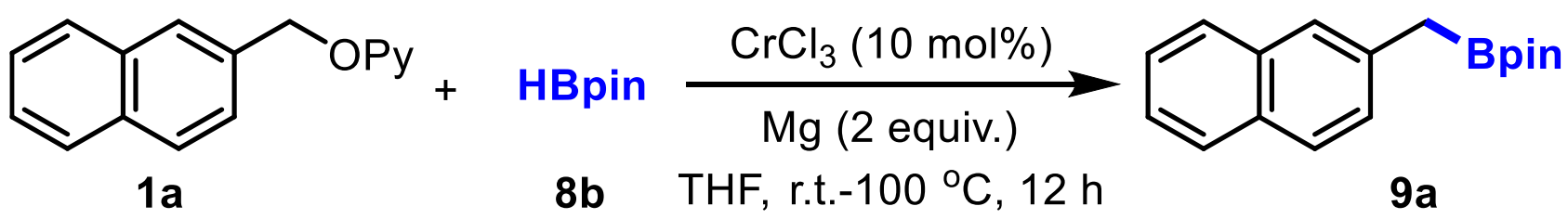

Entry	T/°C	Yield (**9a**)

1	r.t.	38%
2	40	71%
3	60	77%
4	80	81%
5	100	82%

[a]Conditions: **1a** (0.2 mmol), HBpin (0.6 mmol), $CrCl_3$ (0.02 mmol), Mg (0.4 mmol), THF (1 mL), r.t.-100 ^{o}C, 12 h. Isolated yields were given.

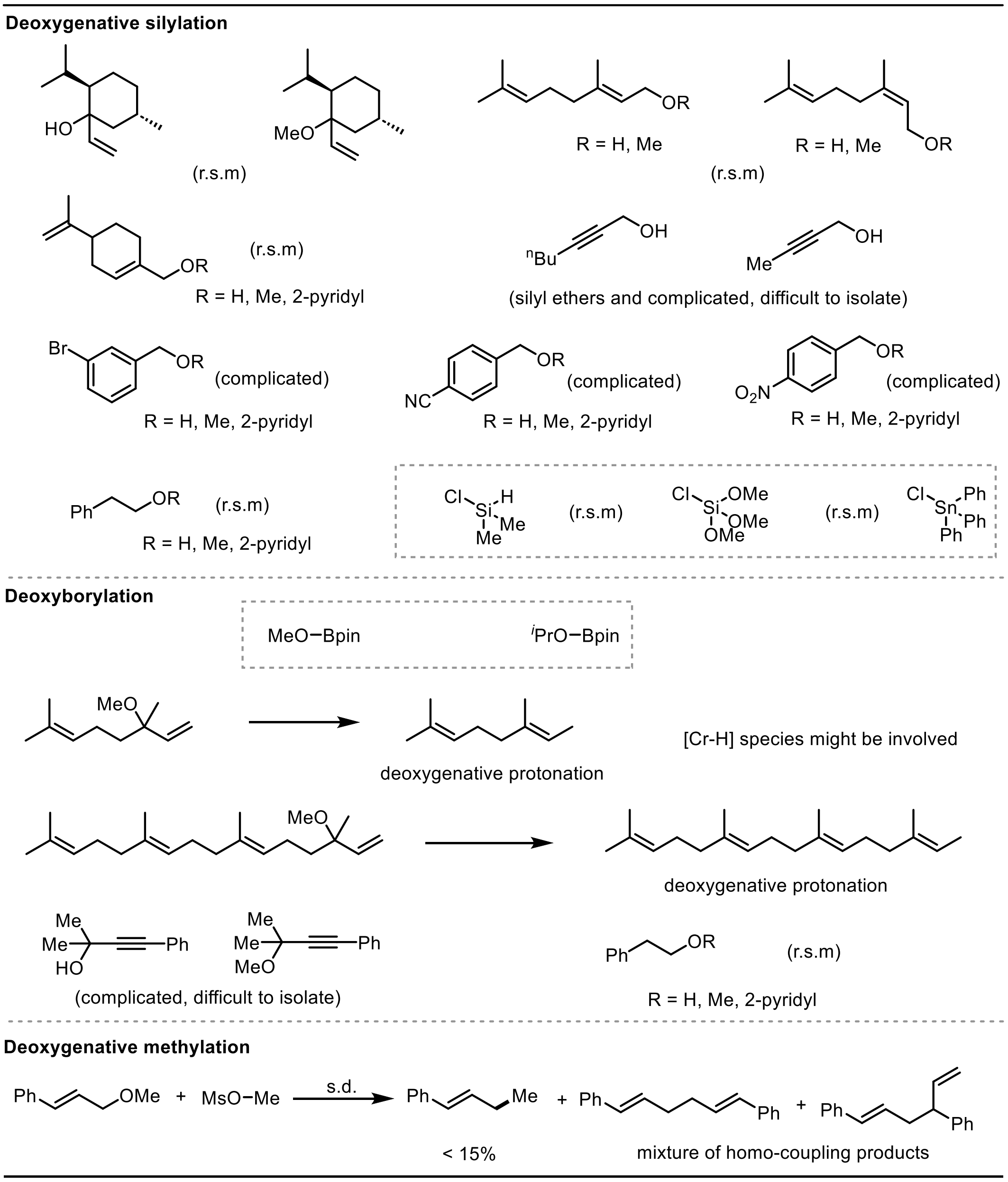

Figure S1. Unsuccessful substrates

3. The Preparation of Substrates

Synthesis of new 2-pyridyl ethers

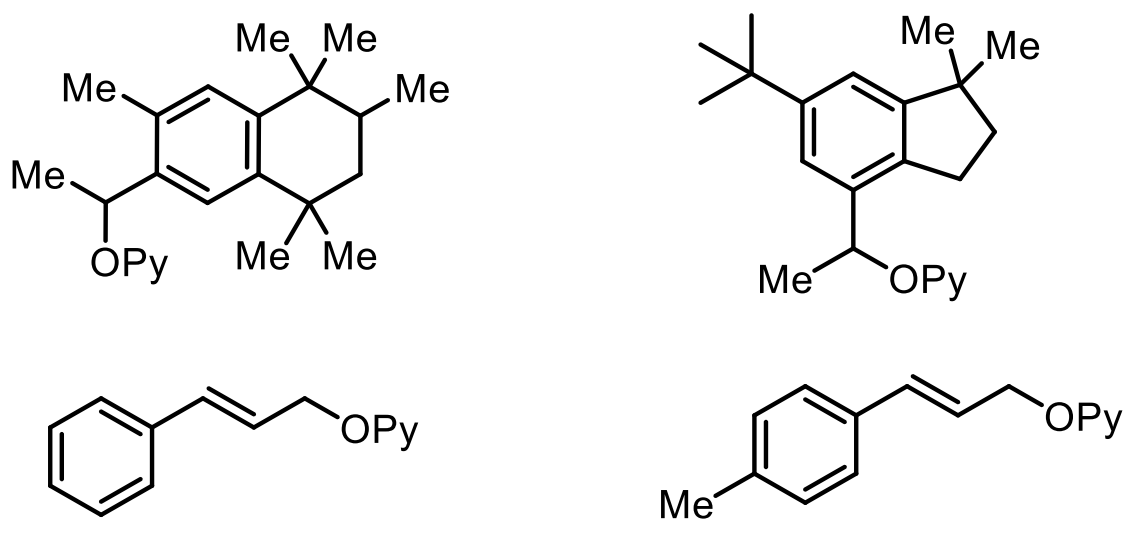

The above new 2-pyridyl ethers were prepared according to the known procedure (72,76,98).

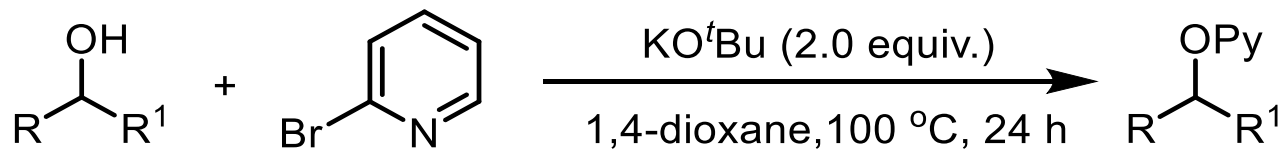

An oven-dried flask was charged with alcohol (10 mmol, 1 equiv.), 2-bromopyridine (12 mmol, 1.2 equiv.) and ^{t}BuOK (20 mmol, 2 equiv.). Then 1,4-dioxane (20 mL) was added by syringe, and the reaction mixture was stirred at 100 °C for 24 h. EtOAc (20 mL) and H_2O (20 mL) were added and the mixture was filtered through a Celite pad. The organic layer was separated and the aqueous layer was extracted with EtOAc (2 x 20 mL). The combined organic layers were washed with H_2O (2 x 20 mL), dried over anhydrous Na_2SO_4, and filtered through a pad of silica gel. The filtrate was concentrated and the residue was purified by column chromatography using PE/EA as eluent.

Synthesis of the corresponding new allyl methyl ethers

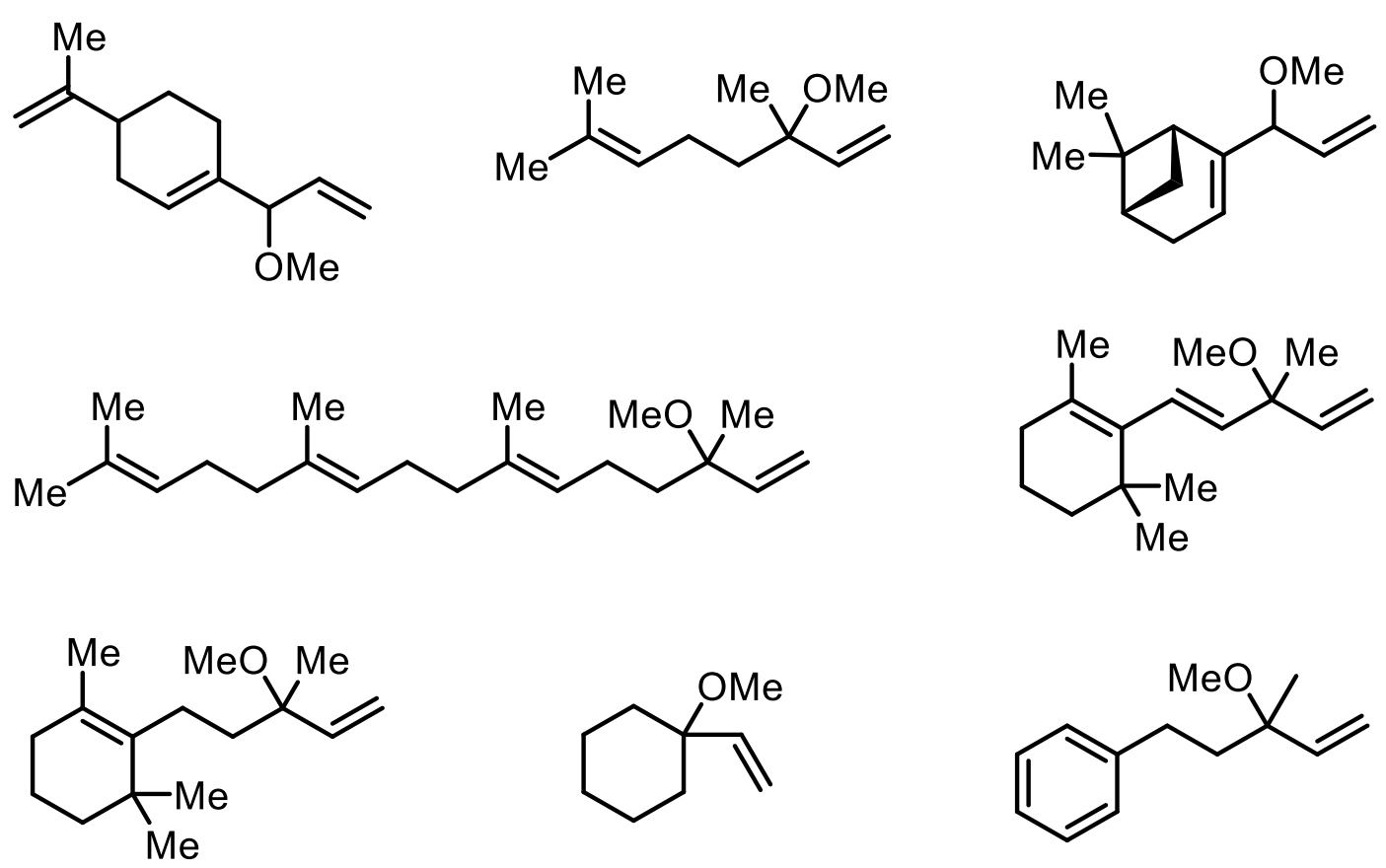

The above allyl methyl ethers were prepared according to the known procedure (99,100).

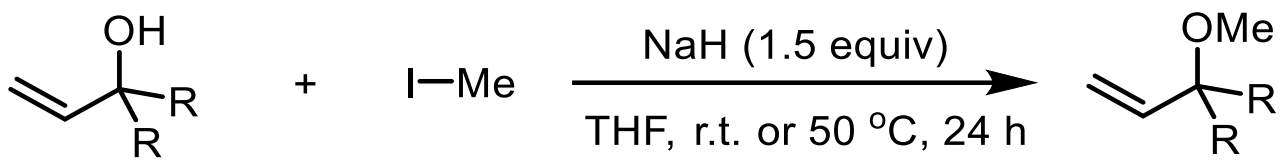

To a dried 100 mL flask were added NaH (1.08 g, 15.0 mmol) and THF (30 mL). The reaction mixture was cooled to 0 °C, followed by the addition of allyl alcohols (10 mmol). The resulting mixture was stirred at 0 °C for 30 min. Methyl iodide (15 mmol) was then added in one portion. The resulting mixture was then stirred at ambient temperature or 50 °C for 24 h. The reaction was quenched with water, and extracted three times with EtOAc. The combined organic layer was washed with brine, and dried over Na_2SO_4. After filtration and evaporation, the residue was purified by silica gel column chromatography (hexane/EtOAc = 49/1→19/1) to give the desired allyl methyl ethers.

New synthesized ethers were shown as following:

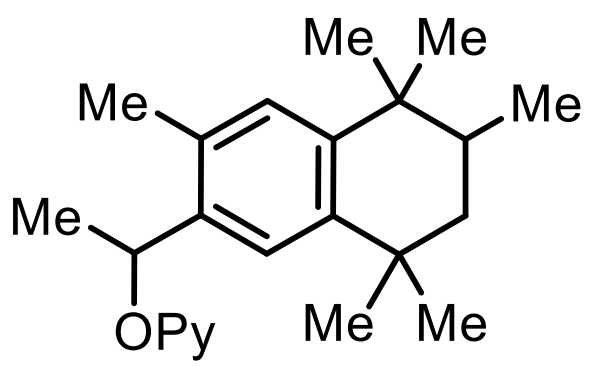

2-(1-(3,5,5,6,8,8-Hexamethyl-5,6,7,8-tetrahydronaphthalen-2-yl)ethoxy)pyridine

The title compound was prepared according to the general procedure as a yellow solid (total: 2.5 g, 75% yield; d.r. = 1:1). ^{1}H NMR (400 MHz, $CDCl_3$): δ = 8.14-8.12 (m, 1H), 7.54-7.48 (m, 1H), 7.44 (s, 1H), 7.10 (s, 1H), 6.81-6.78 (m, 1H), 6.73-6.70 (m, 1H), 6.37 (q, J = 6.4 Hz, 2H), 6.86 (d, J = 8.5 Hz, 2H), 4.46 (s, 2H), 3.78 (s, 3H), 3.51 (t, J = 7.0 Hz, 2H), 2.35 (s, 3H), 1.84-1.80 (m, 1H), 1.65-1.61 (m, 3H), 1.36-1.20 (m, 12H), 1.05 (d, J = 12.0 Hz, 3H), 0.97 (d, J = 6.8 Hz, 3H); ^{13}C NMR (101 MHz, $CDCl_3$): δ = 163.4, 147.0, 145.1, 145.0, 142.4, 138.6, 137.8, 132.5, 132.4, 128.8, 124.0, 123.8, 116.5, 111.7, 70.0, 43.8, 37.5, 34.7, 34.2, 32.2, 32.1, 28.7, 25.1, 25.0, 21.0, 19.1, 16.9. HRMS (ESI$^+$): calcd for $C_{23}H_{31}NONa$ $[M+Na]^+$ 360.2289, found 360.2290.

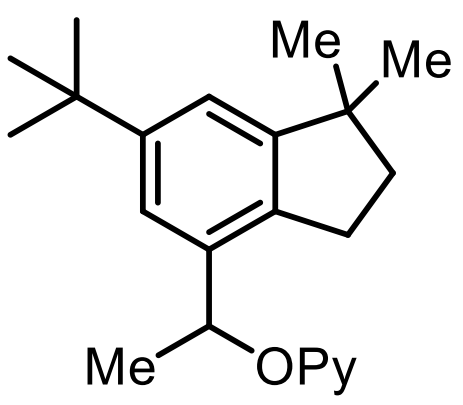

2-(1-(6-(*Tert*-butyl)-1,1-dimethyl-2,3-dihydro-1*H*-inden-4-yl)ethoxy)pyridine

The title compound was prepared according to the general procedure as a colorless oil (2.2 g, 80% yield). ^{1}H NMR (400 MHz, $CDCl_3$): δ = 8.16-8.13 (m, 1H), 7.55-7.51 (m,

1H), 7.41-7.39 (m, 1H), 7.12-7.10 (m, 1H), 6.82-6.73 (m, 2H), 6.32 (q, *J* = 6.4 Hz, 1H), 3.04-2.88 (m, 2H), 1.94 (t, *J* = 7.2 Hz, 2H), 1.66 (d, *J* = 6.4 Hz, 3H), 1.34 (s, 9H), 1.26 (s, 6H); ^{13}C NMR (101 MHz, $CDCl_3$): δ = 163.4, 152.7, 150.0, 147.0, 138.6, 137.7, 137.6, 120.9, 120.8, 118.1, 116.5, 111.7, 71.3, 44.0, 41.6, 34.9, 31.8, 28.9, 28.2, 21.2. HRMS (ESI$^+$): calcd for $C_{22}H_{29}NONa$ [M+Na]$^+$ 346.2142, found 346.2144.

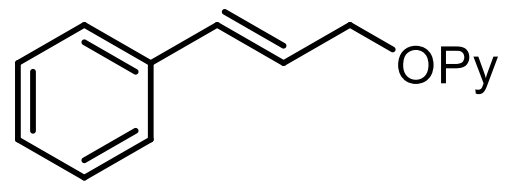

(*E*)-2-(Cinnamyloxy)pyridine

The title compound was prepared according to the general procedure as a yellow oil (1.7 g, 79% yield). ^{1}H NMR (400 MHz, $CDCl_3$): δ = 8.18-8.16 (m, 1H), 7.60-7.56 (m, 1H), 7.41 (d, *J* = 7.6 Hz, 2H), 7.33-7.21 (m, 3H), 6.89-6.86 (m, 1H), 6.80-6.77 (m, 1H), 6.75 (d, *J* = 16.0 Hz, 1H), 6.47 (dt, *J* = 16.0, 6.0 Hz, 1H), 5.00 (dd, *J* = 6.0, 1.6 Hz, 2H); ^{13}C NMR (101 MHz, $CDCl_3$): δ = 163.5, 146.9, 138.7, 136.7, 133.2, 128.6, 127.9, 126.7, 124.8, 116.9, 111.3, 66.4. HRMS (ESI$^+$): calcd for $C_{14}H_{13}NONa$ [M+Na]$^+$ 234.0890, found 234.0891.

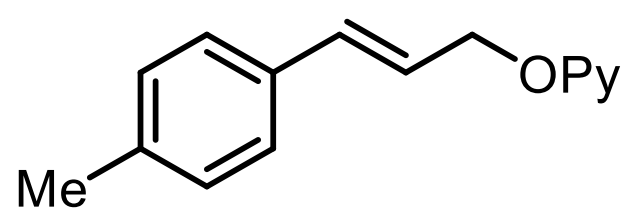

(*E*)-2-((3-(*p*-Tolyl)allyl)oxy)pyridine

The title compound was prepared according to the general procedure as a colorless oil (1.6 g, 70% yield). ^{1}H NMR (400 MHz, $CDCl_3$): δ = 8.19-8.16 (m, 1H), 7.60-7.55 (m, 1H), 7.32 (d, *J* = 8.0 Hz, 2H), 7.13 (d, *J* = 8.0 Hz, 2H), 6.86-6.85 (m, 1H), 6.79 (d, *J* = 8.4 Hz, 1H), 6.72 (d, *J* = 16.0 Hz, 1H), 6.46-6.39 (m, 1H), 4.99 (dd, *J* = 6.4, 1.2 Hz, 2H), 2.33 (s, 3H); ^{13}C NMR (101 MHz, $CDCl_3$): δ = 163.6, 146.9, 138.7, 137.7, 133.9, 133.3, 129.3, 126.6, 123.7, 116.9, 111.3, 66.5, 21.3. HRMS (ESI$^+$): calcd for $C_{15}H_{15}NONa$ [M+Na]$^+$ 248.1046, found 248.1040.

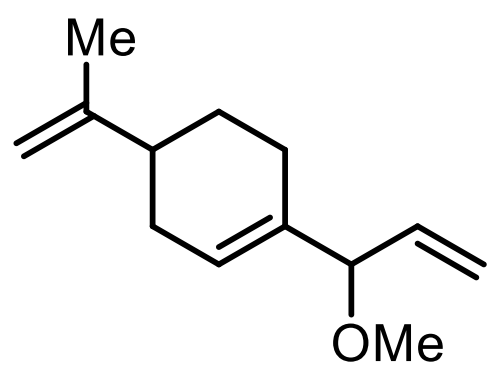

1-(1-Methoxyallyl)-4-(prop-1-en-2-yl)cyclohex-1-ene

The title compound was prepared according to the general procedure as a colorless oil (total: 1.6 g, 85% yield; d.r. = 1:1). ^{1}H NMR (400 MHz, $CDCl_3$): δ = 5.82-5.72 (m, 2H), 5.25-5.15 (m, 2H), 4.77-4.70 (m, 2H), 3.93 (d, *J* = 6.8 Hz, 1H), 3.27 (d, *J* = 14.0

Hz, 3H), 2.20-1.92 (m, 5H), 1.84-1.80 (m, 1H), 1.72 (s, 3H), 1.48-1.38 (m, 1H); ^{13}C NMR (101 MHz, $CDCl_3$): δ = 149.93, 149.88, 137.7, 137.6, 136.8, 136.4, 124.7, 124.4, 116.1, 108.7, 86.4, 56.0, 55.9, 41.4, 41.3, 30.7, 27.5, 24.2, 24.0, 20.9, 20.8. HRMS (ESI$^+$): calcd for $C_{13}H_{20}ONa$ $[M+Na]^+$ 215.1407, found 215.1407.

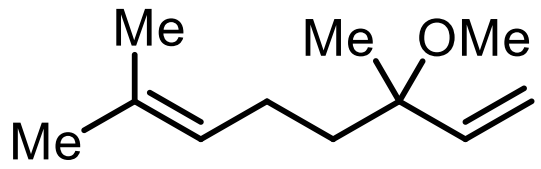

3-Methoxy-3,7-dimethylocta-1,6-diene

The title compound was prepared according to the general procedure as a colorless oil (1.4 g, 86% yield). ^{1}H NMR (400 MHz, $CDCl_3$): δ = 5.74 (dd, *J* = 17.6, 10.8 Hz, 1H), 5.18-5.06 (m, 3H), 3.14 (s, 3H), 1.96 (q, *J* = 8.4 Hz, 2H), 1.66 (s, 3H), 1.58 (s, 3H), 1.55-1.50 (m, 2H), 1.22 (s, 3H); ^{13}C NMR (101 MHz, $CDCl_3$): δ = 142.9, 131.5, 124.6, 114.7, 77.3, 50.1, 39.4, 25.8, 22.4, 21.6, 17.7. HRMS (ESI$^+$): calcd for $C_{11}H_{20}ONa$ $[M+Na]^+$ 191.1407, found 191.1400.

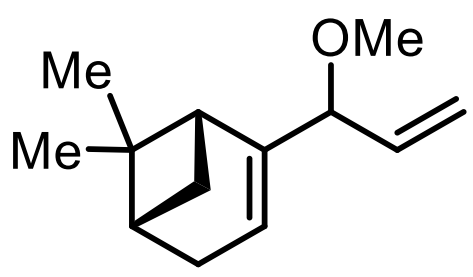

2-(1-Methoxyallyl)-6,6-dimethylbicyclo[3.1.1]hept-2-ene

The title compound was prepared according to the general procedure as a yellow oil (total: 1.7 g, 86% yield; d.r. = 1:1). ^{1}H NMR (400 MHz, $CDCl_3$): δ = 5.71-5.60 (m, 1H), 5.49 (s, 1H), 5.25-5.11 (m, 2H), 3.96 (dd, *J* = 29.6, 6.4 Hz, 1H), 3.49 (d, *J* = 10.0 Hz, 3H), 2.46-2.15 (m, 4H), 2.09-2.06 (m, 1H), 1.26-1.25 (m, 3H), 1.14 (dd, *J* = 14.0, 8.8 Hz, 1H), 0.83 (s, 3H); ^{13}C NMR (101 MHz, $CDCl_3$): δ = 147.0, 146.9, 137.1, 136.8, 120.8, 119.5, 116.4, 116.0, 85.2, 85.1, 56.2, 55.9, 42.4, 41.3, 41.1, 40.1, 37.90, 37.85, 32.0, 31.7, 31.5, 31.4, 26.2, 21.5, 21.3. HRMS (ESI$^+$): calcd for $C_{13}H_{20}ONa$ $[M+Na]^+$ 215.1407, found 215.1409.

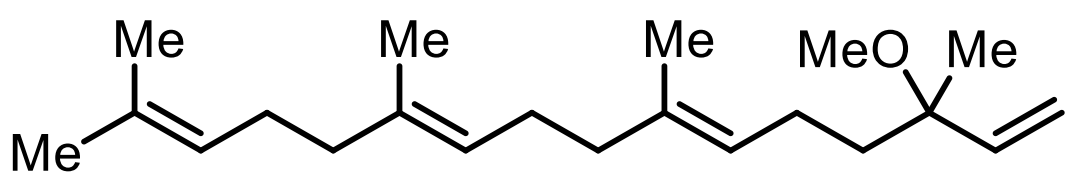

(6*E*,10*E*)-3-Methoxy-3,7,11,15-tetramethylhexadeca-1,6,10,14-tetraene

The title compound was prepared according to the general procedure as a colorless oil (2.3 g, 75% yield). ^{1}H NMR (400 MHz, $CDCl_3$): δ = 5.75 (dd, *J* = 17.6, 10.8 Hz, 1H), 5.18-5.14 (m, 2H), 5.10-5.06 (m, 3H), 3.14 (s, 3H), 2.08-1.95 (m, 12H), 1.67 (s, 3H), 1.59 (s, 9H), 1.22 (s, 3H); ^{13}C NMR (101 MHz, $CDCl_3$): δ = 143.0, 135.1, 135.0,

131.3, 124.5, 124.4, 124.2, 114.7, 77.3, 50.1, 39.8, 39.4, 26.9, 26.7, 25.8, 22.4, 21.7, 17.7, 16.1. HRMS (ESI$^+$): calcd for $C_{21}H_{36}ONa$ $[M+Na]^+$ 357.2659, found 357.2660.

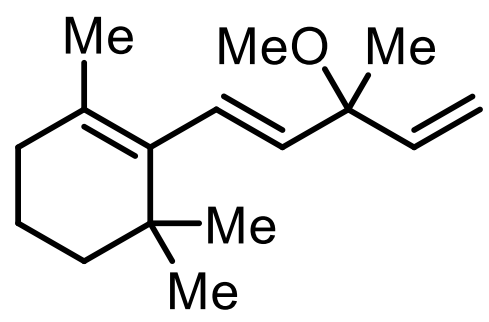

(*E*)-2-(3-Methoxy-3-methylpenta-1,4-dien-1-yl)-1,3,3-trimethylcyclohex-1-ene

The title compound was prepared according to the general procedure**)** as a yellow oil (2.1 g, 89% yield). ^{1}H NMR (400 MHz, $CDCl_3$): δ = 6.02 (d, *J* = 15.6 Hz, 1H), 5.89 (dd, *J* = 17.6, 11.0 Hz, 1H), 5.40 (d, *J* = 16.4 Hz, 1H), 5.24-5.14 (m, 2H), 3.22 (s, 3H), 1.96 (t, *J* = 6.4 Hz, 2H), 1.66 (s, 3H), 1.62-1.58 (m, 2H), 1.45-1.41 (m, 2H), 1.38 (s, 3H), 0.98 (s, 6H); ^{13}C NMR (101 MHz, $CDCl_3$): δ = 142.2, 137.2, 136.6, 128.4, 114.3, 78.2, 51.0, 39.4, 34.1, 32.7, 28.9, 24.0, 21.6, 19.4. HRMS (ESI$^+$): calcd for $C_{16}H_{26}ONa$ $[M+Na]^+$ 257.1876, found 257.1879.

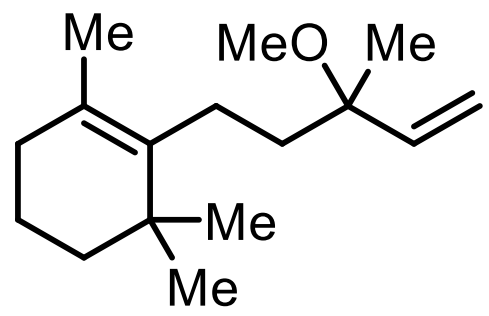

2-(3-Methoxy-3-methylpent-4-en-1-yl)-1,3,3-trimethylcyclohex-1-ene

The title compound was prepared according to the general procedure as a colorless oil (1.9 g, 81% yield). ^{1}H NMR (400 MHz, $CDCl_3$): δ = 5.77 (dd, *J* = 17.6, 11.0 Hz, 1H), 5.21-5.11 (m, 2H), 3.17 (s, 3H), 1.96 (t, *J* = 8.4 Hz, 2H), 1.87 (t, *J* = 6.4 Hz, 2H), 1.60-1.53 (m, 7H), 1.40-1.37 (m, 2H), 1.25 (s, 3H), 0.96 (s, 6H); ^{13}C NMR (101 MHz, $CDCl_3$): δ = 142.9, 136.9, 127.1, 114.9, 77.8, 50.2, 40.0, 39.8, 35.2, 32.9, 28.8, 22.7, 21.0, 19.9, 19.6. HRMS (ESI$^+$): calcd for $C_{16}H_{28}ONa$ $[M+Na]^+$ 259.2033, found 229.2034.

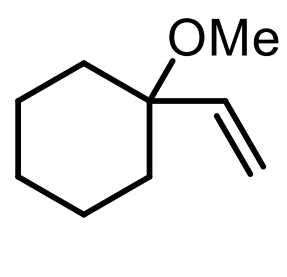

1-methoxy-1-vinylcyclohexane

The title compound was prepared according to the general procedure as a colorless oil (1.1 g, 80% yield). ^{1}H NMR (400 MHz, $CDCl_3$): δ = 5.66-5.58 (m, 1H), 5.22 (dd, *J* = 10.4, 2.0 Hz, 1H), 5.13 (dd, *J* = 17.2, 1.6 Hz, 1H), 3.24 (s, 3H), 1.87-1.84 (m, 1H), 1.72-1.61 (m, 4H), 1.45-1.38 (m, 1H), 1.21-1.18 (m, 2H), 1.00-0.92 (m, 2H); ^{13}C NMR (101 MHz, $CDCl_3$): δ = 137.4, 117.9, 88.0, 56.5, 42.4, 28.8, 26.7, 26.2, 22.8. HRMS (ESI$^+$): calcd for $C_9H_{16}ONa$ $[M+Na]^+$ 163.1094, found 163.1080.

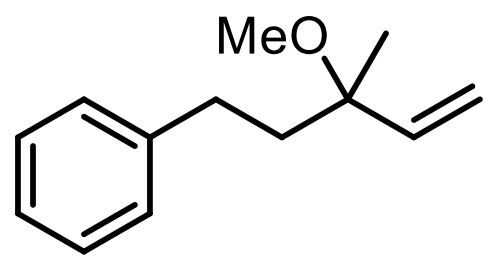

(3-Methoxy-3-methylpent-4-en-1-yl)benzene

The title compound was prepared according to the general procedure as a colorless oil (1.7 g, 90% yield). ^{1}H NMR (400 MHz, $CDCl_3$): δ = 7.28-7.25 (m, 2H), 7.19-7.17 (m, 3H), 5.81 (dd, J = 17.6, 11.2 Hz, 1H), 5.23-5.15 (m, 2H), 3.20 (s, 3H), 2.62 (t, J = 8.4 Hz, 2H), 1.86-1.81 (m, 2H), 1.30 (s, 3H); ^{13}C NMR (101 MHz, $CDCl_3$): δ = 142.8, 142.7, 128.4, 125.8, 115.1, 77.2, 50.2, 41.4, 30.1, 21.9. HRMS (ESI$^+$): calcd for $C_{13}H_{18}ONa$ [M+Na]$^+$ 213.1250, found 213.1250.

4. General Procedure for the Deoxygenative Silylation of Alcohols and Ethers with Chlorosilanes 2 by Chromium

General procedure for the deoxygenative silylation of arylmethyl 2-pyridyl ethers 1 with chlorosilanes 2 by chromium

A dried Schlenk tube was charged with 2-pyridyl ethers **1** (0.2 mmol), $CrCl_3$ (10 mol%), and chlorosilanes **2** (0.8 mmol), followed by adding a freshly distilled THF (1 mL) by syringe under atmosphere of nitrogen. After stirring at room temperature for 5 min, EtMgBr (1.0 M in THF, 1 mmol) was added dropwise, and the reaction mixture was stirred at room temperature for 12 h. After quenched by aqueous NH_4Cl (2 mL), the crude product was extracted with ethyl acetate (3 x 10 mL). The combined organic phases were dried over anhydrous Na_2SO_4 and concentrated under vacuum. The residue was purified by flash chromatography on silica gel to give the desired product **3**.

General procedure for the deoxygenative silylation of arylmethyl methyl ethers 1' with chlorosilanes 2 by chromium in the presence of *t*Bu-tpy

A dried Schlenk tube was charged with methyl ethers **1'** (0.2 mmol), $CrCl_3$ (20 mol%), ^{t}Bu-tpy (20 mol%) and chlorosilanes **2** (0.8 mmol), followed by adding a freshly distilled THF (1 mL) by syringe under atmosphere of nitrogen. After stirring at room temperature for 5 min, EtMgBr (1.0 M in THF, 1 mmol) was added dropwise, and the reaction mixture was stirred at 70 °C for 24 h. After cooled to room temperature and quenched by aqueous NH_4Cl (2 mL), the crude product was extracted with ethyl acetate (3 x 10 mL). The combined organic phases were dried over anhydrous Na_2SO_4 and concentrated under vacuum. The residue was purified by flash chromatography on silica gel to give the desired product **3**.

General procedure for the deoxygenative silylation of arylmethyl alcohols 1'' with 2a by chromium in the presence of ^{t}Bu-tpy

A dried Schlenk tube was charged with alcohol **1''** (0.2 mmol), $CrCl_3$ (25 mol%), ^{t}Bu-tpy (25 mol%) and **2a** (1.0 mmol), followed by adding a freshly distilled THF (0.8 mL) by syringe under atmosphere of nitrogen. After stirring at room temperature for 5 min, EtMgBr (1.0 M in THF, 1.2 mmol) was added dropwise, and the reaction mixture was stirred at 70 °C for 24 h. After cooled to room temperature and quenched by aqueous NH_4Cl (2 mL), the crude product was extracted with ethyl acetate (3 x 10 mL). The combined organic phases were dried over anhydrous Na_2SO_4 and concentrated under vacuum. The residue was purified by flash chromatography on silica gel to give the desired product **3**.

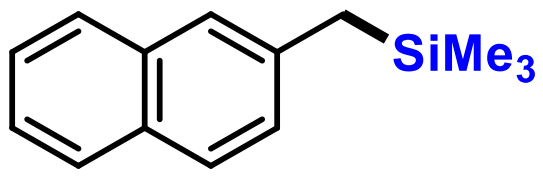

Trimethyl(naphthalen-2-ylmethyl)silane (3a)

The title compound was prepared according to the general procedure as a colorless solid (**A:** 34.3 mg, 80% yield; **B:** 35.6 mg, 83% yield; **Alcohol**: 19.3 mg, 45% yield). ^{1}H NMR (400 MHz, $CDCl_3$) δ = 7.81 (d, *J* = 8.0 Hz, 1H), 7.75 (t, *J* = 8.2 Hz, 2H), 7.49-7.43 (m, 2H), 7.40 (dd, *J* = 10.9, 4.1 Hz, 1H), 7.20 (dd, *J* = 8.4, 1.6 Hz, 1H), 2.29 (s, 2H), 0.06 (s, 9H); ^{13}C NMR (101 MHz, $CDCl_3$) δ = 138.4, 134.0, 131.1, 128.0, 127.7, 127.6, 127.1, 125.9, 125.3, 124.5, 27.5, -1.7. HRMS (ESI$^+$): Calcd for $C_{14}H_{19}Si[M+H]^+$ 215.1251, Found 215.1253.

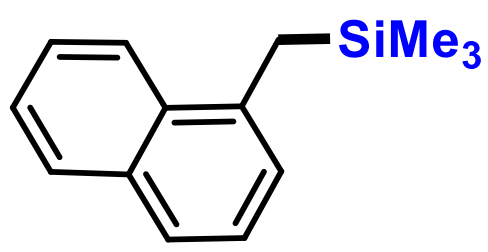

Trimethyl(naphthalen-1-ylmethyl)silane (3b)

The title compound was prepared according to the general procedure as a colorless solid (**A:** 33.4 mg, 78% yield; **B:** 34.4 mg, 80% yield; **Alcohol**: 19.3 mg, 45% yield). ^{1}H NMR (400 MHz, $CDCl_3$) δ = 8.12-8.07 (m, 1H), 7.99-7.94 (m, 1H), 7.76 (d, *J* = 8.2 Hz, 1H), 7.63-7.56 (m, 2H), 7.53-7.47 (m, 1H), 7.31 (t, *J* = 4.6 Hz, 1H), 2.72 (s, 2H), 0.15 (s, 9H); ^{13}C NMR (101 MHz, $CDCl_3$) δ = 137.4, 134.1, 131.8, 128.7, 125.6, 125.5, 125.4, 125.1, 124.9, 124.8, 23.6, -1.0. HRMS (ESI$^+$): Calcd for $C_{14}H_{19}Si[M+H]^+$ 215.1251, Found 215.1257.

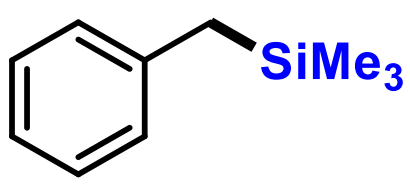

benzyltrimethylsilane (3c)

The title compound was prepared according to the general procedure as a colorless oil (**A:** 20.4 mg, 62% yield). The spectroscopic data are consistent with those previously reported in the literature (50). ^{1}H NMR (400 MHz, $CDCl_3$) δ = 7.21 (t, J = 7.7 Hz, 2H), 7.06 (t, J = 7.4 Hz, 1H), 6.99 (d, J = 6.7 Hz, 2H), 2.08 (s, 2H), -0.01 (s, 9H); ^{13}C NMR (101 MHz, $CDCl_3$) δ 140.65, 128.25, 128.18, 123.98, 27.20, -1.78. HRMS (ESI$^+$): Calcd for $C_{15}H_{19}Si[M+H]^+$ 165.1095, Found 165.1099.

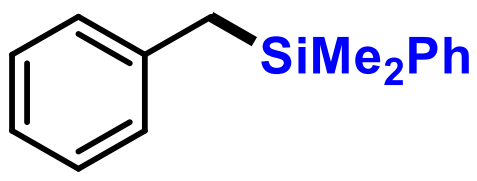

Benzyldimethyl(phenyl)silane (3c-DMP)

The title compound was prepared according to the general procedure as a colorless oil (**A:** 32.6 mg, 72% yield). ^{1}H NMR (400 MHz, $CDCl_3$) δ = 7.48-7.46 (m, 2H), 7.38-7.34 (m, 3H), 7.19 (t, *J* = 7.6 Hz, 2H), 7.08 (t, *J* = 7.2 Hz, 1H), 6.96 (d, *J* = 8.0 Hz, 2H), 2.32 (s, 2H), 0.26 (s, 9H); ^{13}C NMR (101 MHz, $CDCl_3$) δ = 139.8, 138.6, 133.8, 129.2, 128.4, 128.2, 127.8, 124.2, 26.3, 3.4. HRMS (ESI$^+$): Calcd for $C_{15}H_{19}Si[M+H]^+$ 227.1251, Found 227.1259.

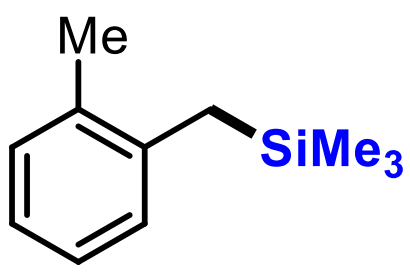

Trimethyl(2-methylbenzyl)silane (3d)

The title compound was prepared according to the general procedure as a colorless solid (**A:** 24.5 mg, 67% yield). ^{1}H NMR (400 MHz, $CDCl_3$) δ = 7.14 (d, *J* = 7.4 Hz, 1H), 7.10 (t, *J* = 7.4 Hz, 1H), 7.01 (dd, *J* = 14.8, 7.4 Hz, 2H), 2.26 (s, 3H), 2.13 (s, 2H), 0.04 (s, 9H); ^{13}C NMR (101 MHz, CDCl3) δ = 139.1, 134.7, 130.2, 128.8, 125.7, 124.2, 23.8, 20.5, -1.2. HRMS (ESI$^+$): Calcd for $C_{11}H_{18}SiNa[M+Na]^+$ 201.1070, Found 201.1071.

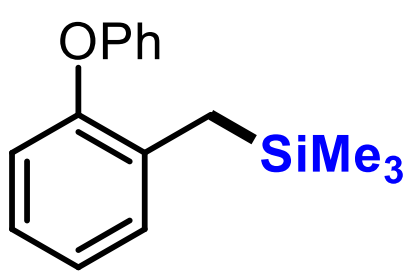

Trimethyl(2-phenoxybenzyl)silane (3e)

The title compound was prepared according to the general procedure as a colorless oil (**A:** 40.0 mg, 78% yield; **B:** 41.0 mg, 80% yield). ^{1}H NMR (400 MHz, $CDCl_3$) δ = 7.38-7.32 (m, 2H), 7.14 (dd, *J* = 6.9, 2.4 Hz, 1H), 7.12-7.05 (m, 3H), 7.01-6.96 (m,

2H), 6.89 (dd, *J* = 7.5, 1.8 Hz, 1H), 2.14 (s, 2H), 0.08 (s, 9H); ^{13}C NMR (101 MHz, $CDCl_3$) δ = 157.7, 153.5, 132.5, 130.5, 129.8, 125.4, 123.6, 122.6, 119.1, 118.0, 20.9, -1.3. HRMS (ESI^+): Calcd for $C_{16}H_{21}OSi[M+H]^+$ 257.1357, Found 257.1358.

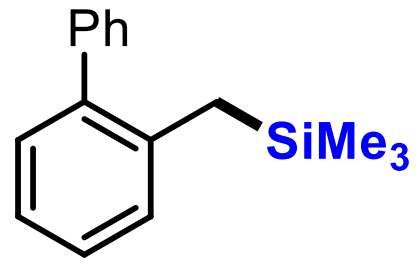

([1,1'-biphenyl]-2-ylmethyl)trimethylsilane (3f)

The title compound was prepared according to the general procedure as a colorless solid (**A:** 40.5 mg, 84% yield; **B:** 40.9 mg, 85% yield). ^{1}H NMR (400 MHz, $CDCl_3$) δ = 7.74-7.69 (m, 2H), 7.65 (dd, *J* = 6.9, 4.2 Hz, 3H), 7.54-7.48 (m, 2H), 7.45 (dd, *J* = 11.5, 4.3 Hz, 2H), 2.55 (s, 2H), 0.15 (s, 9H); ^{13}C NMR (101 MHz, $CDCl_3$) δ = 142.7, 140.8, 138.3, 130.5, 129.9, 129.4, 128.1, 127.1, 126.7, 124.2, 23.6, -1.2. HRMS (ESI^+): Calcd for $C_{16}H_{20}SiNa[M+Na]^+$ 263.1227, Found 263.1231.

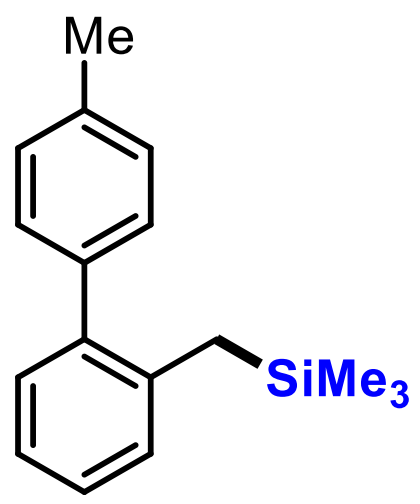

Trimethyl((4'-methyl-[1,1'-biphenyl]-2-yl)methyl)silane (3g)

The title compound was prepared according to the general procedure as a colorless solid (**A:** 41.2 mg, 81% yield; **B:** 42.2 mg, 83% yield). ^{1}H NMR (400 MHz, $CDCl_3$) δ = 7.54-7.50 (m, 5H), 7.47 (dd, *J* = 7.9, 1.7 Hz, 1H), 7.43 (dd, *J* = 10.5, 4.3 Hz, 2H), 2.71 (s, 3H), 2.54 (s, 2H), 0.15 (s, 9H); ^{13}C NMR (101 MHz, $CDCl_3$) δ = 140.8, 139.7, 138.3, 136.2, 130.5, 129.7, 129.3, 128.8, 127.0, 124.2, 23.6, 21.3, -1.1. HRMS (ESI^+): Calcd for $C_{17}H_{22}SiNa[M+Na]^+$ 277.1383, Found 277.1387.

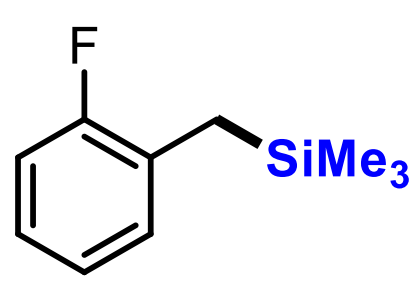

(2-Fluorobenzyl)trimethylsilane (3h)

The title compound was prepared according to the general procedure as a colorless oil (**A:** 21,5 mg, 59% yield; **B:** 24.1 mg, 66% yield). ^{1}H NMR (400 MHz, $CDCl_3$) δ = 7.06-6.94 (m, 4H), 2.08 (d, *J* = 1.9 Hz, 2H), 0.01 (d, *J* = 0.4 Hz, 9H); ^{13}C NMR (101 MHz, $CDCl_3$) δ = 160.4 (d, *J* = 243.4 Hz), 130.5 (d, *J*= 5.1 Hz), 127.8 (d, *J* = 16.2

Hz), 125.5 (d, J = 8.1 Hz), 123.8 (d, J = 3.0 Hz), 115.1 (d, J = 23.2 Hz), 19.6 (d, J = 2.0 Hz), -1.7; ^{19}F NMR (376 MHz, $CDCl_3$) δ = -117.42. HRMS (ESI$^+$): Calcd for $C_{10}H_{15}SiFNa[M+Na]^+$ 205.0819, Found 205.0822.

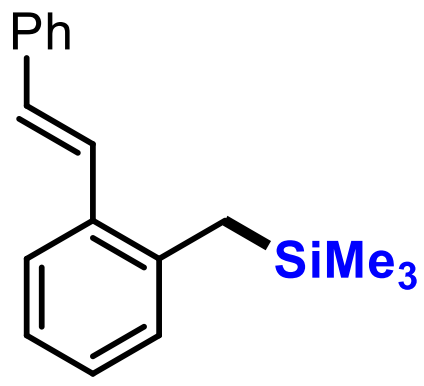

(*E*)-Trimethyl(2-styrylbenzyl)silane (3i)

The title compound was prepared according to the general procedure as a colorless oil (**A:** 44.2 mg, 83% yield; **B:** 44.2 mg, 83% yield). ^{1}H NMR (400 MHz, $CDCl_3$) δ = 7.62 (d, J = 7.5 Hz, 1H), 7.56-7.51 (m, 2H), 7.40 (t, J = 7.6 Hz, 2H), 7.36-7.27 (m, 2H), 7.21-7.12 (m, 2H), 7.04 (d, J = 7.4 Hz, 1H), 6.99 (d, J = 16.1 Hz, 1H), 2.30 (s, 2H), 0.05 (d, J = 1.2 Hz, 9H); ^{13}C NMR (101 MHz, $CDCl_3$) δ = 139.0, 138.0, 134.9, 129.7, 129.5, 128.8, 127.5, 127.5, 126.6, 125.8, 124.7, 24.3, -1.2. Calcd for $C_{18}H_{22}SiNa[M+Na]^+$ 289.1388, Found 289.1387.

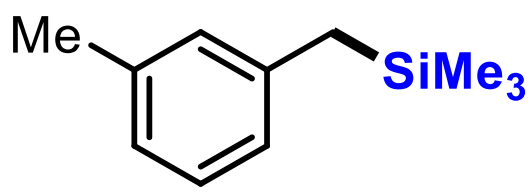

Trimethyl(3-methylbenzyl)silane (3j)

The title compound was prepared according to the general procedure as a colorless solid (**A:** 22.0 mg, 62% yield; **B:** 24.0 mg, 67% yield). ^{1}H NMR (400 MHz, $CDCl_3$) δ = 7.13 (t, J = 7.5 Hz, 1H), 6.91 (d, J = 7.5 Hz, 1H), 6.85-6.81 (m, 2H), 2.33 (s, 3H), 2.07 (s, 2H), 0.02 (s, 9H); ^{13}C NMR (101 MHz, $CDCl_3$) δ = 140.5, 137.7, 129.0, 128.1, 125.3, 124.8, 27.0, 21.6, -1.8. HRMS (ESI$^+$): Calcd for $C_{11}H_{18}SiNa[M+Na]^+$ 201.1070, Found 201.1074.

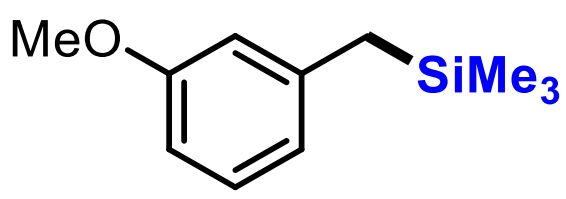

(3-Methoxybenzyl)trimethylsilane (3k)

The title compound was prepared according to the general procedure as a colorless oil (**A:** 30.3 mg, 78% yield; **B:** 32.0 mg, 82% yield). ^{1}H NMR (400 MHz, $CDCl_3$) δ = 7.13 (t, J = 7.9 Hz, 1H), 6.65-6.58 (m, 2H), 6.56 (d, J = 1.8 Hz, 1H), 3.79 (s, 3H), 2.07 (s, 2H), 0.01 (s, 9H); ^{13}C NMR (101 MHz, $CDCl_3$) δ = 159.6, 142.3, 129.1, 120.8, 113.9, 109.3, 55.2, 27.3, -1.7. HRMS (ESI$^+$): Calcd for $C_{11}H_{18}SiONa[M+Na]^+$ 217.1019, Found 217.1025.

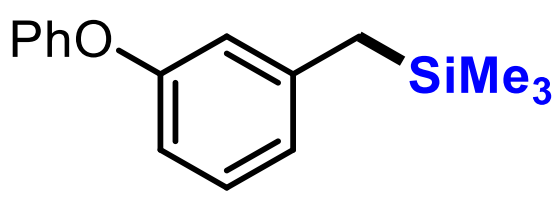

Trimethyl(3-phenoxybenzyl)silane (3l)

The title compound was prepared according to the general procedure as a colorless oil (**A:** 41.5 mg, 81% yield; **B:** 43.6 mg, 85% yield). ^{1}H NMR (400 MHz, $CDCl_3$) δ = 7.39-7.33 (m, 2H), 7.21 (t, *J* = 7.8 Hz, 1H), 7.12 (dd, *J* = 10.6, 4.2 Hz, 1H), 7.07-7.02 (m, 2H), 6.81-6.76 (m, 2H), 6.71 (d, *J* = 1.6 Hz, 1H), 2.10 (s, 2H), 0.04 (s, 9H); ^{13}C NMR (101 MHz, $CDCl_3$) δ = 157.7, 157.1, 142.8, 129.7, 129.4, 123.3, 123.0, 118.8, 114.8, 27.2, -1.8. HRMS (ESI^+): Calcd for $C_{16}H_{21}SiO[M+H]^+$ 257.1356, Found 257.1359.

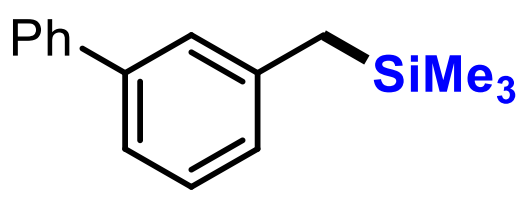

([1,1'-biphenyl]-3-ylmethyl)trimethylsilane (3m)

The title compound was prepared according to the general procedure as a colorless solid (**A:** 28.9 mg, 60% yield; **B:** 37.5 mg, 78% yield). ^{1}H NMR (400 MHz, $CDCl_3$) δ = 7.57 (dd, *J* = 6.5, 1.4 Hz, 2H), 7.43 (d, *J* = 7.3 Hz, 2H), 7.34–7.27 (m, 3H), 7.22 (d, *J* = 1.4 Hz, 1H), 7.00–6.97 (m, 1H), 2.15 (s, 2H), 0.03 (s, 9H); ^{13}C NMR (101 MHz, $CDCl_3$) δ = 141.7, 141.2, 141.1, 128.8, 128.6, 127.3, 127.2, 127.2, 127.0, 123.0, 27.3, -1.7. HRMS (ESI^+): Calcd for $C_{16}H_{21}Si[M+H]^+$ 241.1407, Found 241.1412.

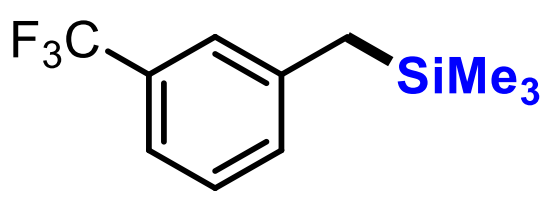

Trimethyl(3-(trifluoromethyl)benzyl)silane (3n)

The title compound was prepared according to the general procedure as a colorless oil (**A:** 25.6 mg, 55% yield; **B:** 28.8 mg, 62% yield). ^{1}H NMR (400 MHz, $CDCl_3$) δ = 7.47 (d, *J* = 6.5 Hz, 2H), 7.40 (s, 1H), 7.32 (d, *J* = 6.7 Hz, 1H), 2.30 (s, 2H), 0.15 (s, 9H); ^{13}C NMR (101 MHz, $CDCl_3$) δ = 141.8, 131.4, 131.61 (q, $J_{C\text{-}F}$ = 31 Hz), 128.6, 124.60 (d, $J_{C\text{-}F}$ =3 Hz), 124.54 (q, $J_{C\text{-}F}$ = 271 Hz), 120.90 (q, $J_{C\text{-}F}$ = 4 Hz), 27.3, -1.9; ^{19}F NMR (376 MHz, $CDCl_3$) δ = -62.65. HRMS (ESI^+): Calcd for $C_{11}H_{16}F_3Si[M+H]^+$ 233.0968, Found 233.0971.

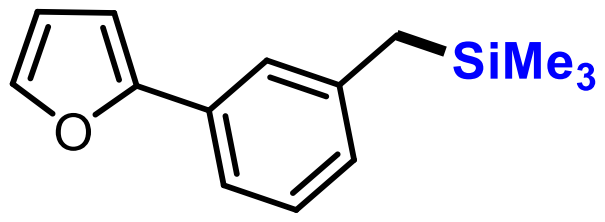

(3-(furan-2-yl)benzyl)trimethylsilane (3o)

The title compound was prepared according to the general procedure as a colorless oil (**A:** 28.1 mg, 61% yield; **B:** 32.3 mg, 70% yield). ^{1}H NMR (400 MHz, $CDCl_3$) δ = 7.58 (dd, *J* = 1.7, 0.6 Hz, 1H), 7.53 (d, *J* = 7.8 Hz, 1H), 7.47 (d, *J* = 1.3 Hz, 1H), 7.36 (t, *J* = 7.7 Hz, 1H), 7.04 (d, *J* = 7.6 Hz, 1H), 6.75 (dd, *J* = 3.4, 0.6 Hz, 1H), 6.59 (dd, *J* = 3.3, 1.8 Hz, 1H), 2.25 (s, 2H), 0.15 (s, 9H); ^{13}C NMR (101 MHz, $CDCl_3$) δ = 154.5, 142.0, 141.1, 130.8, 128.6, 127.3, 123.4, 119.8, 111.7, 104.8, 27.2, -1.8. HRMS (ESI$^+$): Calcd for $C_{14}H_{19}OSi[M+H]^+$ 231.1200, Found 231.1204.

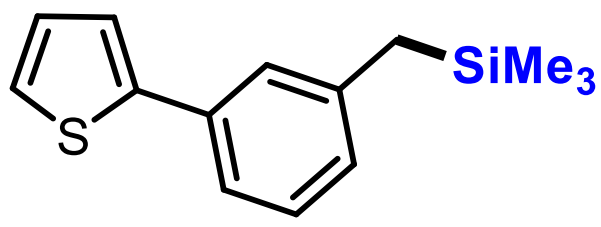

Trimethyl(3-(thiophen-2-yl)benzyl)silane (3p)

The title compound was prepared according to the general procedure as a pale yellow solid (**A:** 40.4 mg, 82% yield; **B:** 41.9 mg, 85% yield). ^{1}H NMR (400 MHz, $CDCl_3$) δ = 7.41 (dd, *J* = 2.4, 1.9 Hz, 1H), 7.37 (dd, *J* = 2.1, 1.0 Hz, 2H), 7.33-7.29 (m, 1H), 7.23 (dd, *J* = 4.5, 2.8 Hz, 2H), 6.95-6.91 (m, 1H), 2.13 (s, 2H), 0.02 (s, 9H); ^{13}C NMR (101 MHz, $CDCl_3$) δ = 142.9, 141.2, 135.8, 128.7, 127.1, 126.6, 126.3, 126.1, 122.4, 120.1, 27.3, -1.7. HRMS (ESI$^+$): Calcd for $C_{14}H_{19}SSi[M+H]^+$ 247.0977, Found 247.0977.

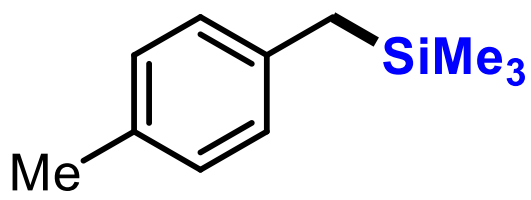

Trimethyl(4-methylbenzyl)silane (3q)

The title compound was prepared according to the general procedure as a colorless solid (**A:** 22.1 mg, 62% yield; **B:** 25.0 mg, 70% yield). ^{1}H NMR (400 MHz, $CDCl_3$) δ = 7.19 (d, *J* = 7.8 Hz, 2H), 7.06 (d, *J* = 7.9 Hz, 2H), 2.46 (s, 3H), 2.20 (s, 2H), 0.15 (s, 9H); ^{13}C NMR (101 MHz, $CDCl_3$) δ = 137.3, 133.2, 129.0, 128.1, 26.6, 21.0, -1.8. HRMS (ESI$^+$): Calcd for $C_{11}H_{18}SiNa[M+Na]^+$ 201.1070, Found 201.1084.

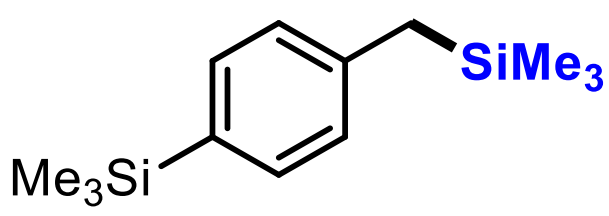

Trimethyl(4-(trimethylsilyl)benzyl)silane (3r)

The title compound was prepared according to the general procedure as a colorless oil (**A:** 35.9 mg, 76% yield; **B:** 39.7 mg, 84% yield). ^{1}H NMR (400 MHz, $CDCl_3$) δ = 7.38 (d, *J* = 8.0 Hz, 2H), 7.01 (d, *J* = 8.0 Hz, 2H), 2.09 (s, 2H), 0.26 (s, 9H), 0.01 (s, 9H); ^{13}C NMR (101 MHz, $CDCl_3$) δ = 141.4, 135.0, 133.4, 127.7, 27.3, -0.8, -1.7. HRMS (ESI$^+$): Calcd for $C_{13}H_{25}Si_2[M+H]^+$ 237.1489, Found 237.1500.

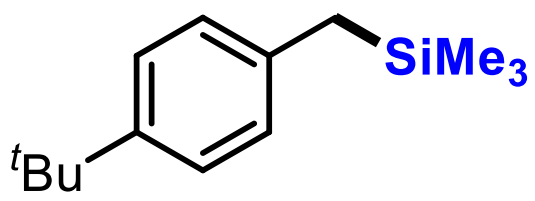

(4-(*Tert*-butyl)benzyl)trimethylsilane (3s)

The title compound was prepared according to the general procedure as a colorless oil (**A:** 29.1 mg, 66% yield; **B:** 35.7 mg, 81% yield). ^{1}H NMR (400 MHz, $CDCl_3$) δ = 7.38 (d, *J* = 8.3 Hz, 2H), 7.09 (d, *J* = 8.3 Hz, 2H), 2.19 (s, 2H), 1.46 (s, 9H), 0.15 (s, 9H); ^{13}C NMR (101 MHz, $CDCl_3$) δ = 146.6, 137.3, 127.9, 125.1, 34.3, 31.6, 26.5, -1.7. HRMS (ESI$^+$): Calcd for $C_{14}H_{25}Si$ [M+H]$^+$ 221.1720; found 221.1717.

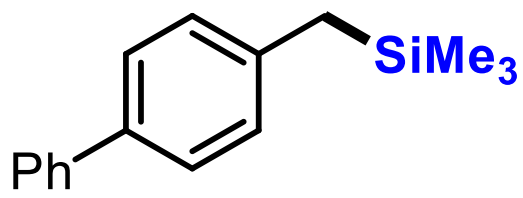

([1,1'-Biphenyl]-4-ylmethyl)trimethylsilane (3t)

The title compound was prepared according to the general procedure as a colorless solid (**A:** 37.0 mg, 77% yield; **B:** 39.9 mg, 83% yield). ^{1}H NMR (400 MHz, $CDCl_3$) δ = 7.68 (dd, *J* = 5.1, 3.3 Hz, 2H), 7.56 (dd, *J* = 6.9, 5.1 Hz, 2H), 7.53-7.47 (m, 2H), 7.42-7.36 (m, 1H), 7.16 (d, *J* = 8.1 Hz, 2H), 2.22 (s, 2H), 0.12 (s, 9H); ^{13}C NMR (101 MHz, $CDCl_3$) δ = 141.3, 139.9, 136.9, 128.8, 128.6, 127.0, 126.9, 126.9, 26.9, -1.7. HRMS (ESI$^+$): Calcd for $C_{16}H_{20}SiNa$[M+Na]$^+$ 263.1226, Found 263.1234.

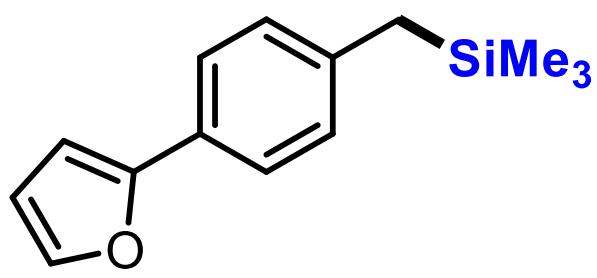

(4-(furan-2-yl)benzyl)trimethylsilane (3u)

The title compound was prepared according to the general procedure as a colorless oil (**A:** 29.0 mg, 63% yield; **B:** 35.9 mg, 78% yield). ^{1}H NMR (400 MHz, $CDCl_3$) δ = 7.60-7.53 (m, 2H), 7.45 (dd, *J* = 1.7, 0.6 Hz, 1H), 7.04 (d, *J* = 8.2 Hz, 2H), 6.59 (dd, *J* = 3.3, 0.5 Hz, 1H), 6.47 (dd, *J* = 3.3, 1.8 Hz, 1H), 2.12 (s, 2H), 0.03 (s, 9H); ^{13}C NMR (101 MHz, CDCl3) δ = 154.6, 141.6, 140.2, 128.4, 127.0, 123.9, 111.6, 103.9, 27.2, -1.8. HRMS (ESI$^+$): Calcd for $C_{14}H_{19}SiO$[M+H]$^+$ 231.1200, Found 231.1204.

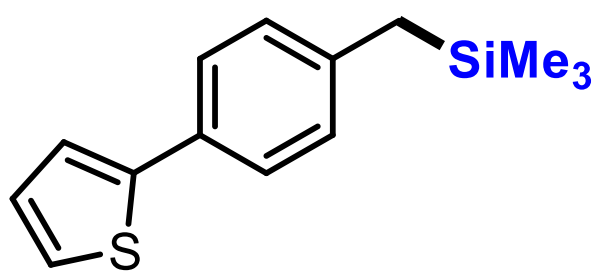

Trimethyl(4-(thiophen-2-yl)benzyl)silane (3v)

The title compound was prepared according to the general procedure as a colorless oil (**A:** 35.0 mg, 71% yield; **B:** 39.4 mg, 80% yield). ^{1}H NMR (400 MHz, $CDCl_3$) δ = 7.61 (d, *J* = 8.1 Hz, 2H), 7.40–7.36 (m, 1H), 7.33 (d, *J* = 5.1 Hz, 1H), 7.17 (dd, *J* = 5.0, 3.7 Hz, 1H), 7.14 (d, *J* = 8.1 Hz, 2H), 2.23 (s, 2H), 0.15 (s, 9H); ^{13}C NMR (101 MHz, $CDCl_3$) δ = 144.9, 140.2, 130.3, 128.6, 128.0, 125.9, 124.0, 122.3, 27.0, -1.8. HRMS (ESI$^+$): Calcd for $C_{14}H_{19}SiS$[M+H]$^+$ 247.0971, Found 247.0979.

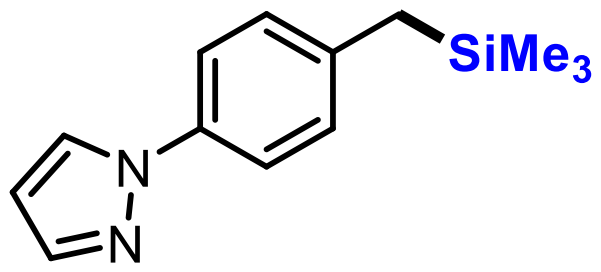

1-(4-((Trimethylsilyl)methyl)phenyl)-1*H*-pyrazole (3w)

The title compound was prepared according to the general procedure as a colorless oil (**A:** 32.7 mg, 71% yield; **B:** 36.9 mg, 80% yield). ^{1}H NMR (400 MHz, $CDCl_3$): *δ* = 7.85 (d, *J* = 2.4 Hz, 1H), 7.69 (d, *J* = 1.6 Hz, 1H), 7.53 (d, *J* = 8.4 Hz, 2H), 7.07 (d, *J* = 8.4 Hz, 2H), 6.42 (t, *J* = 2.0 Hz, 1H), 2.10 (s, 2H), 0.01 (s, 9H); ^{13}C NMR (101 MHz, $CDCl_3$): *δ* = 140.7, 139.2, 137.0, 128.8, 126.7, 119.4, 107.3, 26.7, -1.9. HRMS (ESI$^+$): Calcd for $C_{13}H_{19}N_2Si$ [M+H]$^+$ 231.1313, found 231.1314.

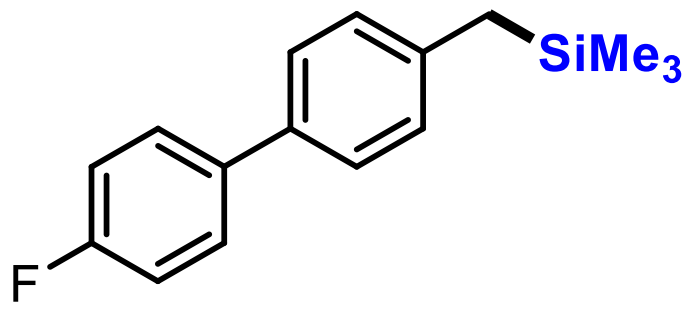

((4'-Fluoro-[1,1'-biphenyl]-4-yl)methyl)trimethylsilane (3x)

The title compound was prepared according to the general procedure as a colorless oil (**A:** 39.8 mg, 77% yield; **B:** 42.9 mg, 83% yield; **Alcohol**: 25.8 mg, 50% yield). ^{1}H NMR (400 MHz, $CDCl_3$) δ = 7.60-7.55 (m, 2H), 7.46 (d, *J* = 8.2 Hz, 2H), 7.14 (dd, *J* = 16.0, 8.4 Hz, 4H), 2.18 (s, 2H), 0.09 (s, 9H); ^{13}C NMR (101 MHz, $CDCl_3$) δ = 162.3 (d, J_{C-F} = 244 Hz), 139.9, 137.5 (d, J_{C-F} = 3 Hz), 135.9, 128.6, 128.4 (d, J_{C-F} = 8 Hz), 126.8, 115.6 (d, J_{C-F} = 21 Hz), 26.9, -1.8; ^{19}F NMR (376 MHz, $CDCl_3$) δ = -116.47. HRMS (ESI$^+$): Calcd for $C_{16}H_{20}SiF$[M+H]$^+$ 259.1313, Found 259.1318.

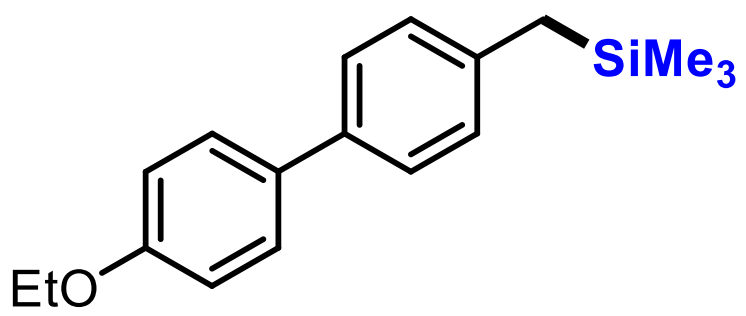

((4'-Ethoxy-[1,1'-biphenyl]-4-yl)methyl)trimethylsilane (3y)

The title compound was prepared according to the general procedure as a colorless oil (**A:** 41.0 mg, 72% yield; **B:** 46.1 mg, 81% yield). ^{1}H NMR (400 MHz, $CDCl_3$) δ =

7.54 (d, J = 8.8 Hz, 2H), 7.45 (d, J = 8.2 Hz, 2H), 7.08 (d, J = 8.1 Hz, 2H), 6.98 (d, J = 8.8 Hz, 2H), 4.09 (q, J = 7.0 Hz, 2H), 2.14 (s, 2H), 1.46 (t, J = 7.0 Hz, 3H), 0.06 (s, 9H); ^{13}C NMR (101 MHz, $CDCl_3$) δ = 158.2, 139.1, 136.5, 133.8, 128.5, 127.9, 126.5, 114.8, 63.6, 26.8, 15.0, -1.7. HRMS (ESI$^+$): Calcd for $C_{18}H_{25}SiO[M+H]^+$ 285.1669, Found 285.1678.

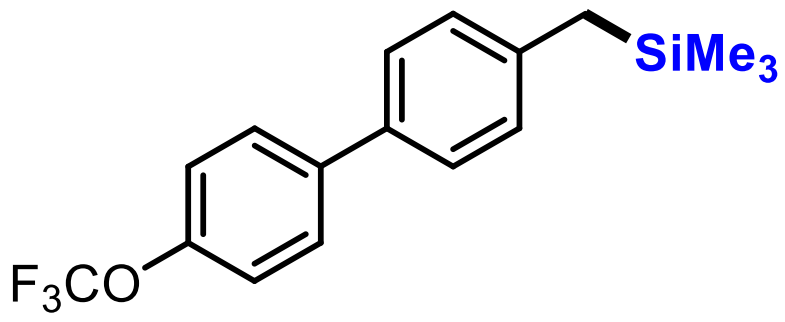

Trimethyl((4'-(trifluoromethoxy)-[1,1'-biphenyl]-4-yl)methyl)silane (3z)

The title compound was prepared according to the general procedure as a colorless oil (**A:** 57.1 mg, 88% yield; **B:** 57.1 mg, 88% yield). ^{1}H NMR (400 MHz, $CDCl_3$) δ = 7.62 (d, J = 8.8 Hz, 2H), 7.47 (d, J = 8.2 Hz, 2H), 7.31 (d, J = 8.1 Hz, 2H), 7.13 (d, J = 8.2 Hz, 2H), 2.18 (s, 2H), 0.09 (s, 9H); ^{13}C NMR (101 MHz, $CDCl_3$) δ = 148.4, 140.5, 140.2, 135.5, 128.7, 128.2, 126.9, 121.3, 120.8 (q, J_{C-F} = 255 Hz), 27.0, -1.8; ^{19}F NMR (376 MHz, $CDCl_3$) δ = -57.77. HRMS (ESI$^+$): Calcd for $C_{17}H_{20}SiF_3O[M+H]^+$ 325.1230, Found 325.1235.

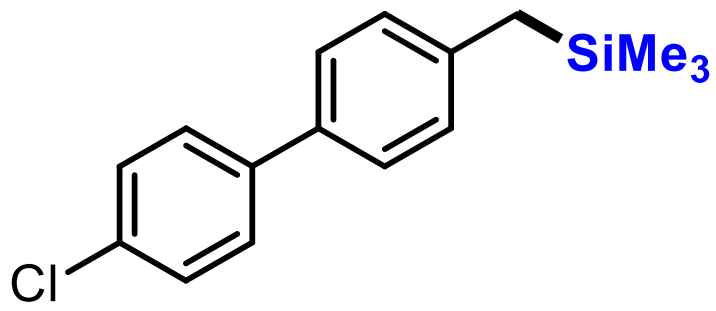

((4'-Chloro-[1,1'-biphenyl]-4-yl)methyl)trimethylsilane (3aa)

The title compound was prepared according to the general procedure as a colorless oil (**A:** 42.3 mg, 77% yield). ^{1}H NMR (400 MHz, $CDCl_3$) δ = 7.53 (d, J = 8.6 Hz, 2H), 7.42 (dd, J = 16.4, 8.4 Hz, 4H), 7.09 (d, J = 8.3 Hz, 2H), 2.15 (s, 2H), 0.06 (s, 9H); ^{13}C NMR (101 MHz, $CDCl_3$) δ = 140.4, 139.8, 135.6, 132.9, 128.9, 128.7, 128.1, 126.8, 27.0, -1.7. HRMS (ESI$^+$): Calcd for $C_{16}H_{20}SiCl[M+H]^+$ 275.1023, Found 275.1023.

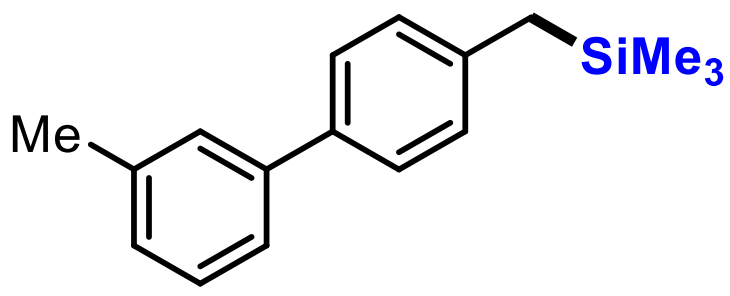

Trimethyl((3'-methyl-[1,1'-biphenyl]-4-yl)methyl)silane (3ab)

The title compound was prepared according to the general procedure as a colorless solid (**A:** 35.6 mg, 70% yield; **B:** 41.2 mg, 81% yield). ^{1}H NMR (400 MHz, $CDCl_3$) δ = 7.58-7.52 (m, 2H), 7.48 (d, J = 10.9 Hz, 2H), 7.39 (t, J = 7.5 Hz, 1H), 7.21 (d, J =

7.4 Hz, 1H), 7.15 (d, *J* = 8.1 Hz, 2H), 2.49 (s, 3H), 2.21 (s, 2H), 0.12 (s, 9H); ^{13}C NMR (101 MHz, $CDCl_3$) δ = 141.3, 139.7, 138.3, 137.0, 128.7, 128.5, 127.8, 127.6, 127.0, 124.0, 26.9, 21.7, -1.7. HRMS (ESI^+): Calcd for $C_{17}H_{22}SiNa$ $[M+Na]^+$ 277.1383, Found 277.1387.

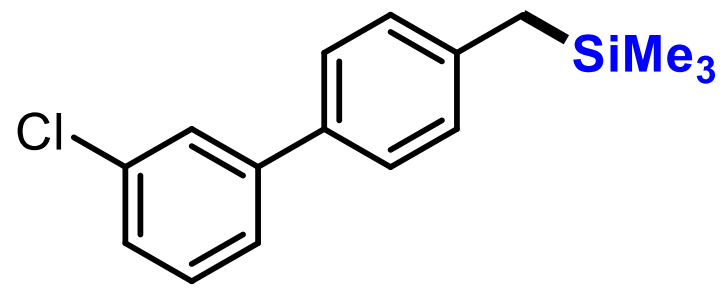

((3'-Chloro-[1,1'-biphenyl]-4-yl)methyl)trimethylsilane (3ac)

The title compound was prepared according to the general procedure as a colorless oil (**A:** 45.1 mg, 82% yield). ^{1}H NMR (400 MHz, $CDCl_3$) δ = 7.59 (t, *J* = 1.8 Hz, 1H), 7.50-7.44 (m, 3H), 7.35 (t, *J* = 7.8 Hz, 1H), 7.32-7.28 (m, 1H), 7.10 (d, *J* = 8.1 Hz, 2H), 2.15 (s, 2H), 0.05 (s, 9H); ^{13}C NMR (101 MHz, $CDCl_3$) δ = 143.2, 140.7, 135.5, 134.7, 130.0, 128.7, 127.1, 126.9, 126.8, 125.0, 27.0, -1.8. HRMS (ESI^+): Calcd for $C_{16}H_{20}SiCl[M+H]^+$ 275.1023, Found 275.1022.

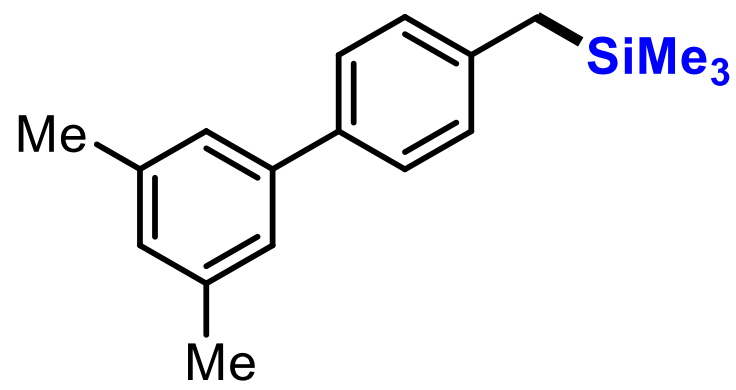

((3',5'-Dimethyl-[1,1'-biphenyl]-4-yl)methyl)trimethylsilane (3ad)

The title compound was prepared according to the general procedure as a colorless solid (**A:** 46.7 mg, 87% yield; **B:** 48.3 mg, 90% yield). ^{1}H NMR (400 MHz, $CDCl_3$) δ = 7.59-7.55 (m, 2H), 7.33 (s, 2H), 7.20-7.15 (m, 2H), 7.08 (s, 1H), 2.49 (s, 6H), 2.24 (s, 2H), 0.15 (s, 9H); ^{13}C NMR (101 MHz, $CDCl_3$) δ = 141.4, 139.6, 138.2, 137.1, 128.5, 128.5, 127.0, 124.9, 26.9, 21.6, -1.7. HRMS (ESI^+): Calcd for $C_{18}H_{24}SiNa[M+Na]^+$ 291.1539, Found 291.1545.

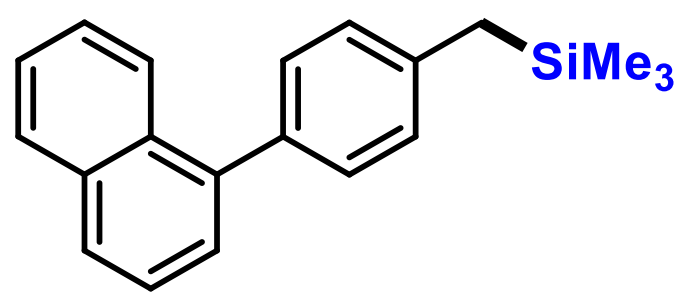

Trimethyl(4-(naphthalen-1-yl)benzyl)silane (3ae)

The title compound was prepared according to the general procedure as a colorless solid (**A:** 46.5 mg, 80% yield; **B:** 51.1 mg, 88% yield). ^{1}H NMR (400 MHz, $CDCl_3$) δ = 8.05 (d, *J* = 8.3 Hz, 1H), 7.95 (d, *J* = 8.0 Hz, 1H), 7.89 (d, *J* = 8.1 Hz, 1H), 7.57-7.46 (m, 4H), 7.45-7.41 (m, 2H), 7.19 (d, *J* = 8.1 Hz, 2H), 2.25 (s, 2H), 0.15 (s,

9H); ^{13}C NMR (101 MHz, $CDCl_3$) δ = 140.6, 139.7, 136.4, 134.0, 131.9, 130.0, 128.4, 128.0, 127.4, 127.0, 126.3, 126.0, 125.8, 125.5, 27.0, -1.7. HRMS (ESI$^+$): Calcd for $C_{20}H_{22}SiNa[M+Na]^+$ 313.1383, Found 313.1393.

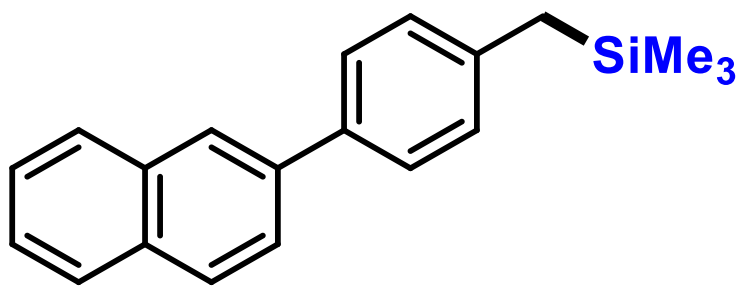

Trimethyl(4-(naphthalen-2-yl)benzyl)silane (3af)

The title compound was prepared according to the general procedure as a colorless solid (**A:** 50.0 mg, 86% yield; **B:** 51.1 mg, 88% yield; **Alcohol**: 30.2 mg, 52% yield). ^{1}H NMR (400 MHz, $CDCl_3$) δ = 8.12 (s, 1H), 7.94 (dd, *J* = 14.4, 7.8 Hz, 3H), 7.83 (dd, *J* = 8.5, 1.7 Hz, 1H), 7.69 (d, *J* = 8.1 Hz, 2H), 7.55-7.51(m, 2H), 7.20 (d, *J* = 8.1 Hz, 2H), 2.24 (s, 2H), 0.15 (s, 9H); ^{13}C NMR (101 MHz, $CDCl_3$) δ = 140.0, 138.7, 136.7, 133.9, 132.5, 128.7, 128.4, 128.2, 127.7, 127.2, 126.3, 125.7, 125.6, 125.3, 26.9, -1.7. HRMS (ESI$^+$): Calcd for $C_{20}H_{23}Si[M+H]^+$ 291.1564, Found 291.1562.

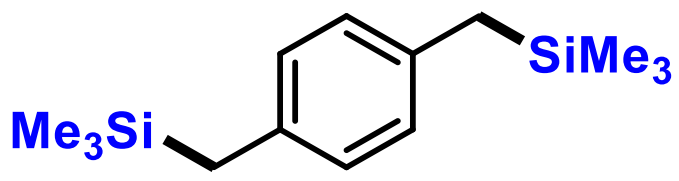

1,4-Bis((trimethylsilyl)methyl)benzene (3ag)

The title compound was prepared according to the general procedure as a colorless oil (**A:** 31.6 mg, 63% yield). ^{1}H NMR (400 MHz, $CDCl_3$) δ = 6.88 (s, 4H), 2.04 (s, 4H), 0.00 (s, 18H); ^{13}C NMR (101 MHz, $CDCl_3$) δ = 135.7, 128.0, 26.4, -1.7. HRMS (ESI$^+$): Calcd for $C_{14}H_{27}Si_2[M+H]^+$ 251.1646, Found: 251.1651.

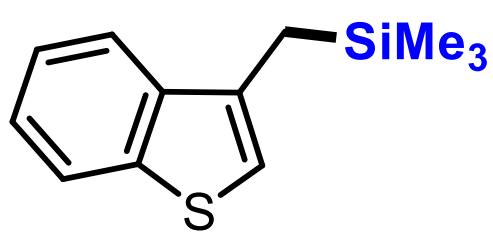

(Benzo[*b*]thiophen-3-ylmethyl)trimethylsilane (3ah)

The title compound was prepared according to the general procedure as a colorless oil (**A:** 34.3 mg, 78% yield). ^{1}H NMR (400 MHz, $CDCl_3$) δ = 7.85-7.83 (m, 1H), 7.67 (d, *J* = 7.6 Hz, 1H), 7.38-7.30 (m, 2H), 6.85 (s, 1H), 2.32 (s, 2H), 0.02 (s, 9H); ^{13}C NMR (101 MHz, $CDCl_3$) δ = 140.3, 139.4, 134.4, 123.9, 123.5, 122.8, 122.3, 118.4, 18.8, -1.2. HRMS (ESI$^+$): Calcd for $C_{12}H_{17}SSi[M+H]^+$ 221.0815, Found 221.0817.

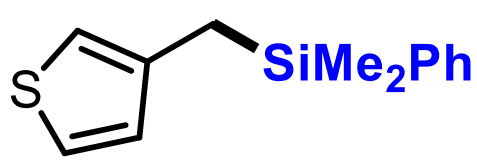

Dimethyl(phenyl)(thiophen-3-ylmethyl)silane (3ai)

The title compound was prepared according to the general procedure as a colorless oil (**A:** 30.2 mg, 65% yield). ^{1}H NMR (400 MHz, $CDCl_3$) δ = 7.47-7.45 (m, 2H), 7.37-7.34 (m, 3H), 7.17-7.15 (m, 1H), 6.70 (d, *J* = 4.8 Hz, 1H), 6.63 (d, *J* = 2.8 Hz, 1H), 2.33 (s, 2H), 0.26 (s, 6H); ^{13}C NMR (101 MHz, $CDCl_3$) δ = 138.8, 138.6, 133.8, 129.2, 129.1, 127.8, 124.8, 118.3, 20.3, -3.2. HRMS (ESI$^+$): Calcd for $C_{13}H_{17}SSi[M+H]^+$ 233.0815, Found 233.0820.

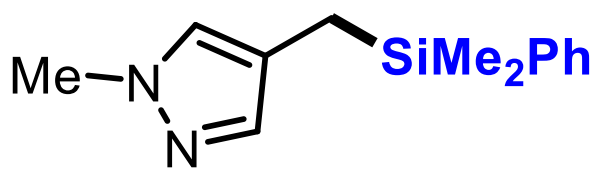

4-((Dimethyl(phenyl)silyl)methyl)-1-methyl-1*H*-pyrazole (3aj)

The title compound was prepared according to the general procedure as a colorless oil (**A:** 34.6 mg, 45% yield). ^{1}H NMR (400 MHz, $CDCl_3$) δ = 7.48-7.45 (m, 2H), 7.35-7.33 (m, 3H), 7.10 (s, 1H), 6.86 (s, 1H), 3.78 (s, 3H), 2.02 (s, 2H), 0.25 (s, 6H); ^{13}C NMR (101 MHz, $CDCl_3$) δ = 139.1, 138.7, 133.8, 132.0, 129.2, 127.8, 117.5, 38.8, 12.5, -3.3. HRMS (ESI$^+$): Calcd for $C_{13}H_{19}N_2Si[M+H]^+$ 231.1313, Found 231.1317.

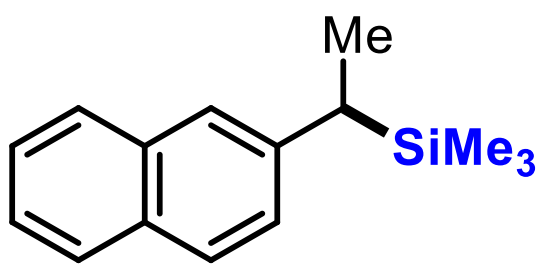

Trimethyl(1-(naphthalen-2-yl)ethyl)silane (3ak)

The title compound was prepared according to the general procedure as a colorless solid (**A:** 24.7mg, 54% yield; **B:** 27.4 mg, 60% yield). ^{1}H NMR (400 MHz, $CDCl_3$) δ = 7.83 (t, *J* = 8.4 Hz, 2H), 7.78 (d, *J* = 8.5 Hz, 1H), 7.53 (s, 1H), 7.49 (t, *J* = 7.4 Hz, 1H), 7.46-7.41 (m, 1H), 7.28 (d, *J* = 8.5 Hz, 1H), 2.41 (q, *J* = 7.4 Hz, 1H), 1.53 (d, *J* = 7.5 Hz, 3H), 0.04 (s, 9H); ^{13}C NMR (101 MHz, $CDCl_3$) δ = 143.9, 133.9, 131.4, 127.7, 127.4, 127.3, 127.2, 125.8, 124.6, 124.2, 30.2, 15.0, -3.1. HRMS (ESI$^+$): Calcd for $C_{15}H_{20}SiNa$ $[M+Na]^+$ 251.1226, Found 251.1232.

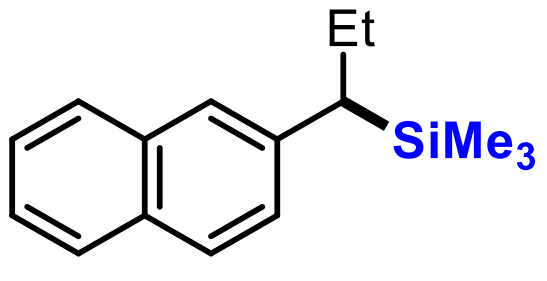

Trimethyl(1-(naphthalen-2-yl)propyl)silane (3al)

The title compound was prepared according to the general procedure as a colorless solid (**A:** 25.2mg, 52% yield; **B:** 28.1 mg, 58% yield). ^{1}H NMR (400 MHz, $CDCl_3$) δ = 7.84-7.74 (m, 3H), 7.48 (s, 1H), 7.47-7.38 (m, 2H), 7.23 (d, *J* = 8.4 Hz, 1H), 2.13 (dd, *J* = 10.5, 5.0 Hz, 1H), 1.97-1.91 (m, 2H), 0.93 (td, *J* = 7.2, 1.7 Hz, 3H), -0.00 (d,

J = 1.8 Hz, 9H); ^{13}C NMR (101 MHz, $CDCl_3$) δ = 141.6, 133.9, 131.5, 127.7, 127.5, 127.3, 125.8, 125.3, 124.5, 39.6, 22.6, 14.6, -2.7.HRMS (ESI$^+$): Calcd for $C_{16}H_{22}SiNa$ $[M+Na]^+$ 265.1383, Found:265.1388.

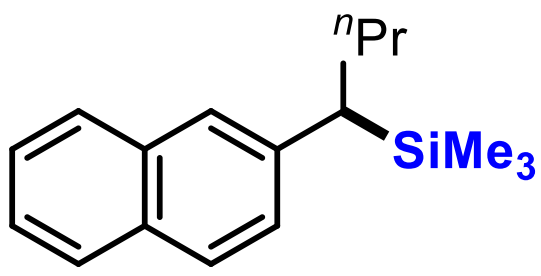

Trimethyl(1-(naphthalen-2-yl)butyl)silane (3am)

The title compound was prepared according to the general procedure as a colorless oil (**A:** 25.6 mg, 50% yield). ^{1}H NMR (400 MHz, $CDCl_3$) δ = 7.83-7.71 (m, 3H), 7.47-7.37 (m, 3H), 7.23 (dd, J = 8.4, 1.6 Hz, 1H), 2.25 (dd, J = 12.0, 3.6 Hz, 1H), 2.02-1.91 (m, 1H), 1.82-1.71 (m, 1H), 1.44-1.35 (m, 1H), 1.27-1.17 (m, 1H), 0.90 (t, J = 7.3 Hz, 3H), 0.00 (s, 9H); ^{13}C NMR (101 MHz, $CDCl_3$) δ = 141.9, 133.9, 131.5, 127.7, 127.5, 127.5, 127.3, 125.8, 125.3, 124.5, 37.1, 31.7, 22.5, 14.1, -2.7. HRMS (ESI$^+$): Calcd for $C_{17}H_{24}SiNa$ $[M+Na]^+$ 279.1539, Found 279.1539.

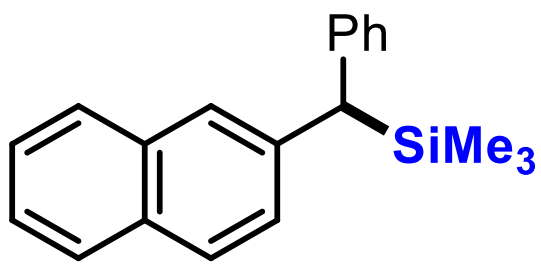

Trimethyl(naphthalen-2-yl(phenyl)methyl)silane (3an)

The title compound was prepared according to the general procedure as a colorless solid (**A:** 40.7 mg, 70% yield). ^{1}H NMR (400 MHz, $CDCl_3$) δ = 7.65 (dd, J = 24.1, 11.4 Hz, 4H), 7.37-7.26 (m, 3H), 7.25-7.16 (m, 4H), 7.10–7.03 (m, 1H), 3.61 (s, 1H), 0.00 (s, 9H); ^{13}C NMR (101 MHz, $CDCl_3$) δ = 143.1, 140.7, 133.8, 131.7, 129.0, 128.5, 128.3, 127.9, 127.6, 126.6, 126.0, 125.3, 125.1, 46.3, -1.4. HRMS (ESI$^+$): Calcd for $C_{20}H_{23}Si[M+H]^+$ 291.1564, Found 291.1566.

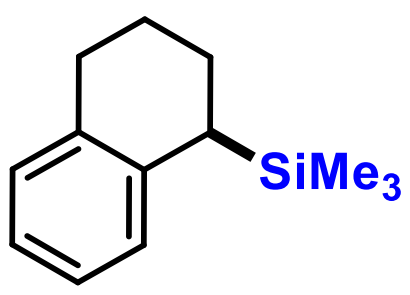

Trimethyl(1,2,3,4-tetrahydronaphthalen-1-yl)silane (3ao)

The title compound was prepared according to the general procedure as a colorless oli (**A:** 25.8 mg, 63% yield). ^{1}H NMR (400 MHz, $CDCl_3$): δ = 7.06-6.95 (m, 4H), 2.76-2.66 (m, 2H), 2.35 (t, J = 5.6 Hz, 1H), 1.96-1.69 (m, 4H), -0.01 (s, 9H); ^{13}C NMR (101 MHz, $CDCl_3$): δ = 139.7, 136.3, 129.3, 128.2, 125.2, 124.0, 29.7, 29.4,

24.9, 22.1, -1.5. HRMS (ESI$^+$): Calcd for $C_{13}H_{20}SiNa$ [M+Na]$^+$ 227.1227, found 227.1230.

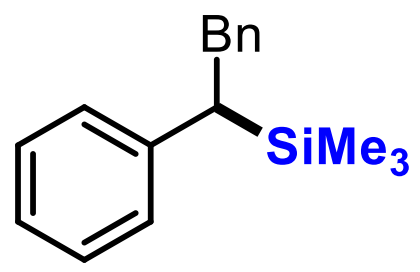

(1,2-diphenylethyl)trimethylsilane (3ap)

The title compound was prepared according to the general procedure as a colorless oil (**A:** 17.8 mg, 35% yield).^{1}H NMR (400 MHz, $CDCl_3$) δ = 7.38-7.31 (m, 4H), 7.26-7.23 (m, 3H), 7.22-7.17 (m, 3H), 3.27 (d, *J* = 7.9 Hz, 2H), 2.58 (t, *J* = 7.8 Hz, 1H), 0.15 (s, 9H); ^{13}C NMR (101 MHz, $CDCl_3$) δ = 143.3, 142.5, 128.6, 128.2, 128.1, 128.0, 125.7, 124.5, 38.8, 35.9, -2.7. HRMS (ESI$^+$): Calcd for $C_{17}H_{22}SiNa$ [M+Na]$^+$ 277.1383, Found 277.1386.

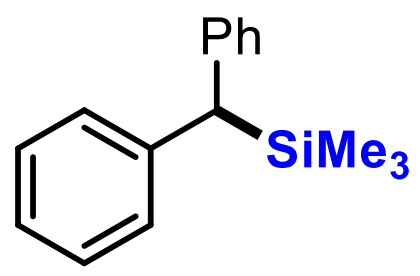

Benzhydryltrimethylsilane (3aq)

The title compound was prepared according to the general procedure as a colorless oil (**A:** 34.1 mg, 71% yield). ^{1}H NMR (400 MHz, $CDCl_3$) δ = 7.32-7.29 (m, 8H), 7.21-7.15 (m, 2H), 3.57 (s, 1H), 0.09 (s, 9H); ^{13}C NMR (101 MHz, $CDCl_3$) δ = 143.0, 128.9, 128.4, 125.2, 46.2, -1.6. HRMS (ESI$^+$): Calcd for $C_{16}H_{20}SiNa$ [M+Na]$^+$ 263.1226, Found 263.1230.

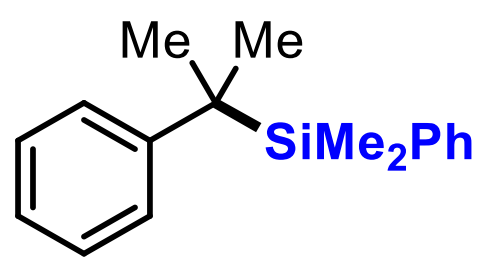

Dimethyl(phenyl)(2-phenylpropan-2-yl)silane (3ar)

The title compound was prepared according to the general procedure as a colorless oil (**A:** 28.0 mg, 55% yield). ^{1}H NMR (400 MHz, $CDCl_3$) δ = 7.36-7.32 (m, 1H), 7.27-7.26 (m, 4H), 7.22-7.18 (m, 2H), 7.10-7.07 (m, 1H), 7.04-7.02 (m, 2H), 1.34 (s, 6H), 0.20 (s, 6H); ^{13}C NMR (101 MHz, $CDCl_3$) δ = 148.3, 136.9, 134.8, 129.0, 127.6, 127.3, 126.5, 124.3, 27.5, 24.3, -5.7. HRMS (ESI$^+$): Calcd for $C_{17}H_{22}NaSi$ [M+Na]$^+$ 277.1383, Found 277.1368.

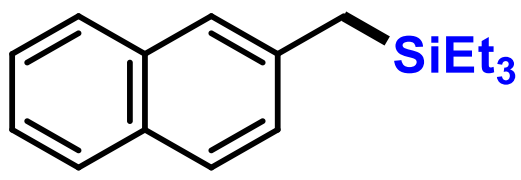

Triethyl(naphthalen-2-ylmethyl)silane (3as)

The title compound was prepared according to the general procedure as a colorless solid (**A:** 41.0 mg, 80% yield; **B:** 41.5 mg, 81% yield). ^{1}H NMR (400 MHz, $CDCl_3$) δ = 7.84 (d, *J* = 7.9 Hz, 1H), 7.78 (t, *J* = 8.5 Hz, 2H), 7.52 (s, 1H), 7.51-7.46 (m, 1H), 7.45-7.41 (m, 1H), 7.28-7.24 (m, 1H), 2.35 (s, 2H), 1.02 (t, *J* = 7.9 Hz, 9H), 0.62 (q, *J* = 7.9 Hz, 6H); ^{13}C NMR (101 MHz, $CDCl_3$) δ = 138.6, 134.0, 131.1, 128.1, 127.7, 127.7, 127.1, 125.9, 125.4, 124.4, 22.1, 7.5, 3.2. HRMS (ESI$^+$): Calcd for $C_{17}H_{24}SiNa[M+Na]^+$ 279.1539, Found 279.1544.

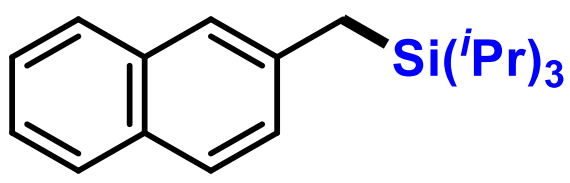

Triisopropyl(naphthalen-2-ylmethyl)silane (3at)

The title compound was prepared according to the general procedure as a colorless solid (**A:** 48.4 mg, 81% yield; **B:** 48.4 mg, 81% yield). ^{1}H NMR (400 MHz, $CDCl_3$) δ = 7.80 (d, *J* = 7.9 Hz, 1H), 7.74 (t, *J* = 8.5 Hz, 2H), 7.57 (s, 1H), 7.48-7.42 (m, 1H), 7.40 (dd, *J* = 10.9, 4.0 Hz, 1H), 7.31 (dd, *J* = 8.4, 1.5 Hz, 1H), 2.41 (s, 2H), 1.19-1.14 (m, 3H), 1.08 (d, *J* = 6.6 Hz, 18H); ^{13}C NMR (101 MHz, $CDCl_3$) δ = 139.0, 133.9, 131.1, 128.4, 127.7, 127.6, 127.1, 126.0, 125.9, 124.5, 19.5, 18.8, 11.2. HRMS (ESI$^+$): Calcd for $C_{20}H_{31}Si$ $[M+H]^+$ 299.2190, Found 299.2196.

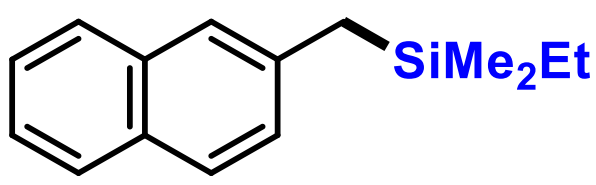

Ethyldimethyl(naphthalen-2-ylmethyl)silane (3au)

The title compound was prepared according to the general procedure as a colorless oil (**A:** 36.5 mg, 80% yield; **B:** 37.9 mg, 83% yield). ^{1}H NMR (400 MHz, $CDCl_3$) δ = 7.82 (d, *J* = 8.2 Hz, 1H), 7.76 (dd, *J* = 9.0, 7.1 Hz, 2H), 7.50-7.44 (m, 2H), 7.43-7.39 (m, 1H), 7.24-7.18 (m, 1H), 2.30 (d, *J* = 2.3 Hz, 2H), 1.00 (td, *J* = 7.9, 2.8 Hz, 3H), 0.64-0.52 (m, 2H), 0.04 (d, *J* = 2.8 Hz, 6H); ^{13}C NMR (101 MHz, $CDCl_3$) δ = 138.4, 134.0, 131.1, 128.1, 127.7, 127.6, 127.1, 125.9, 125.3, 124.5, 25.7, 7.4, 6.7, -3.9. HRMS (ESI$^+$): Calcd for $C_{15}H_{21}Si$ $[M+H]^+$ 229.1408, Found 229.1403.

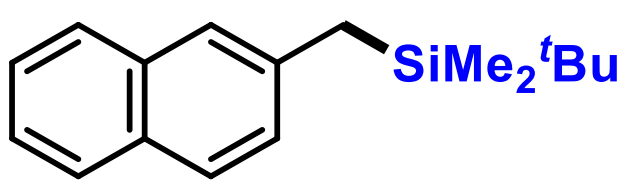

tert-Butyldimethyl(naphthalen-2-ylmethyl)silane (3av)

The title compound was prepared according to the general procedure as a colorless oil (**A:** 41.5 mg, 81% yield; **B:** 41.5 mg, 81% yield). ^{1}H NMR (500 MHz, $CDCl_3$): δ = 7.77 (d, J = 8.0 Hz, 1H), 7.70 (t, J = 7.2 Hz, 2H), 7.43-7.34 (m, 3H), 7.17 (dd, J = 8.4, 1.6 Hz, 1H), 2.26 (s, 2H), 0.94 (s, 9H), -0.09 (s, 6H); ^{13}C NMR (101 MHz, $CDCl_3$): δ = 138.5, 133.9, 131.0, 128.2, 127.7, 127.6, 127.0, 125.8, 125.5, 124.4, 26.7, 22.9, 16.9, -6.4. HRMS (ESI^{+}): Calcd for $C_{17}H_{25}Si$ $[M+H]^{+}$ 257.1721, Found 257.1722.

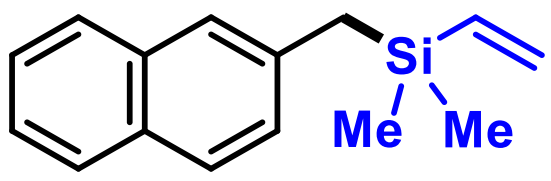

Dimethyl(naphthalen-2-ylmethyl)(vinyl)silane (3aw)

The title compound was prepared according to the general procedure as a colorless oil (**A:** 35.3 mg, 78% yield; **B:** 36.2 mg, 80% yield). ^{1}H NMR (400 MHz, $CDCl_3$) δ = 7.68 (d, J = 8.0 Hz, 1H), 7.62 (t, J = 8.7 Hz, 2H), 7.36-7.31 (m, 2H), 7.30-7.26 (m, 1H), 7.08 (dd, J = 8.3, 1.6 Hz, 1H), 6.07 (dd, J = 20.2, 14.7 Hz, 1H), 5.89 (dd, J = 14.7, 3.8 Hz, 1H), 5.60 (dd, J = 20.2, 3.8 Hz, 1H), 2.23 (s, 2H), -0.00 (s, 6H); ^{13}C NMR (101 MHz, $CDCl_3$) δ = 138.3, 137.8, 133.9, 132.5, 131.2, 128.1, 127.7, 127.6, 127.2, 125.9, 125.6, 124.5, 26.3, -3.5. HRMS (ESI^{+}): Calcd for $C_{15}H_{18}SiNa[M+Na]^{+}$ 249.1070, Found 249.1070.

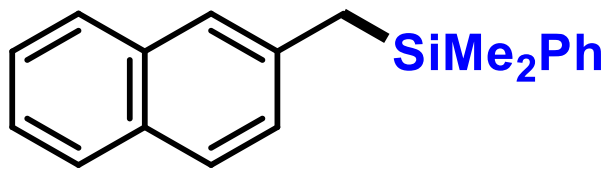

Dimethyl(naphthalen-2-ylmethyl)(phenyl)silane (3ax)

The title compound was prepared according to the general procedure as a colorless oil (**A:** 51.4 mg, 93% yield; **B:** 49.8 mg, 90% yield). ^{1}H NMR (400 MHz, $CDCl_3$) δ = 7.79 (d, J = 8.0 Hz, 1H), 7.72-7.66 (m, 2H), 7.53-7.48 (m, 2H), 7.45-7.34 (m, 6H), 7.10 (dd, J = 8.4, 1.7 Hz, 1H), 2.49 (s, 2H), 0.30 (s, 6H); ^{13}C NMR (101 MHz, $CDCl_3$) δ = 138.6, 137.6, 133.9, 131.2, 129.2, 128.1, 127.9, 127.7, 127.6, 127.2, 125.9, 125.7, 124.6, 26.7, -3.3. HRMS (ESI^{+}): Calcd for $C_{19}H_{20}SiNa[M+Na]^{+}$ 299.1226, Found 299.1222.

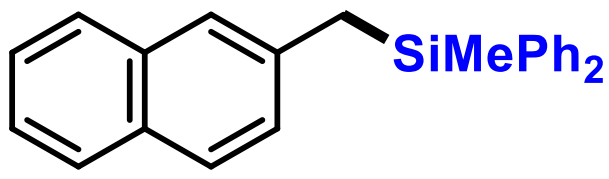

Methyl(naphthalen-2-ylmethyl)diphenylsilane (3ay)

The title compound was prepared according to the general procedure as a colorless oil (**A:** 59.6 mg, 88% yield; **B:** 62.3 mg, 92% yield). ^{1}H NMR (400 MHz, $CDCl_3$) δ = 7.72-7.67 (m, 1H), 7.57 (dd, *J* = 8.1, 3.7 Hz, 2H), 7.48-7.42 (m, 4H), 7.37-7.27 (m, 9H), 6.98 (dd, *J* = 8.4, 1.7 Hz, 1H), 2.77 (s, 2H), 0.47 (s, 3H); ^{13}C NMR (101 MHz, $CDCl_3$) δ = 136.7, 136.4, 134.8, 133.8, 131.3, 129.5, 128.3, 128.1, 127.7, 127.6, 127.2, 126.3, 125.8, 124.6, 24.9, -4.6. HRMS (ESI$^+$): Calcd for $C_{24}H_{22}SiNa[M+Na]^+$ 361.1383, Found 361.1382.

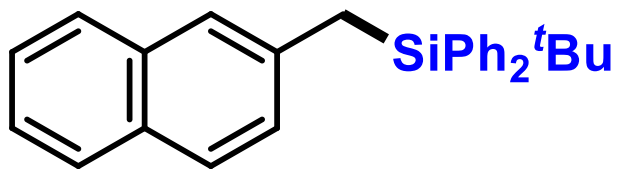

***tert*-butyl(naphthalen-2-ylmethyl)diphenylsilane (3az)**

The title compound was prepared according to the general procedure as a colorless oil (**A:** 63.9 mg, 84% yield; **B:** 68.5 mg, 90% yield). ^{1}H NMR (400 MHz, $CDCl_3$) δ = 7.60-7.55 (m, 1H), 7.47 (dd, *J* = 8.0, 1.4 Hz, 4H), 7.40 (dd, *J* = 7.7, 4.6 Hz, 2H), 7.32-7.27 (m, 2H), 7.21 (dd, *J* = 11.3, 4.4 Hz, 6H), 7.15 (s, 1H), 6.85 (dd, *J* = 8.5, 1.7 Hz, 1H), 2.81 (s, 2H), 0.99 (s, 9H); ^{13}C NMR (101 MHz, $CDCl_3$) δ = 137.0, 136.5, 134.5, 133.6, 131.2, 129.3, 128.7, 127.6, 127.5, 127.2, 127.2, 127.0, 125.6, 124.5, 28.1, 21.2, 18.8. HRMS (ESI$^+$): Calcd for $C_{27}H_{28}SiNa[M+Na]^+$ 403.1852, Found 403.1858.

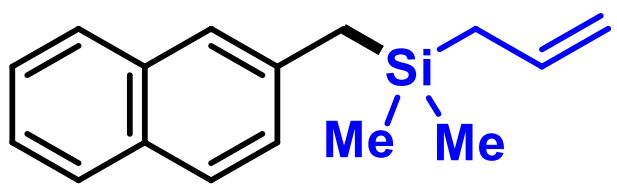

Allyldimethyl(naphthalen-2-ylmethyl)silane (3aaa)

The title compound was prepared according to the general procedure as a colorless oil (**A:** 28.9 mg, 60% yield; **B:** 32.2 mg, 67% yield). ^{1}H NMR (600 MHz, $CDCl_3$) δ = 7.78 (d, *J* = 7.8 Hz, 1H), 7.71 (t, *J* = 6.6 Hz, 2H), 7.43-7.41 (m, 2H), 7.38-7.35 (m, 1H), 7.16 (dd, *J* = 8.4, 1.8 Hz, 1H), 5.82-5.75 (m, 1H), 4.89-4.87 (m, 2H), 2.27 (s, 2H), 1.56 (d, *J* = 8.4 Hz, 2H), 0.01 (s, 6H); ^{13}C NMR (101 MHz, $CDCl_3$) δ = 137.9, 134.8, 133.9, 131.1, 128.0, 127.70, 127.67, 127.1, 125.9, 125.4, 124.5, 113.4, 25.5, 22.9, -3.8. HRMS (ESI$^+$): Calcd for $C_{16}H_{20}SiNa[M+Na]^+$ 263.1227, Found 263 .1230.

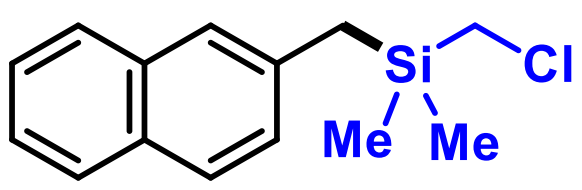

(Chloromethyl)dimethyl(naphthalen-2-ylmethyl)silane (3aab)

The title compound was prepared according to the general procedure as a colorless oil (**A:** 19.9 mg, 40% yield). ^{1}H NMR (600 MHz, $CDCl_3$) δ = 7.46 (s, 1H), 7.43 (t, *J* = 7.2 Hz, 1H), 7.37 (t, *J* = 7.2 Hz, 1H), 7.18 (dd, *J* = 8.4, 1.8 Hz, 1H), 2.75 (s, 2H), 2.39 (s, 2H), 0.12 (s, 6H); ^{13}C NMR (101 MHz, $CDCl_3$) δ = 136.7, 133.9, 131.2, 128.9, 128.0, 127.7, 127.1, 126.0, 125.5, 124.7, 29.6, 23.9, -4.7. HRMS (ESI^+): Calcd for $C_{14}H_{17}ClSiNa[M+Na]^+$ 271.0681, Found 271.0683.

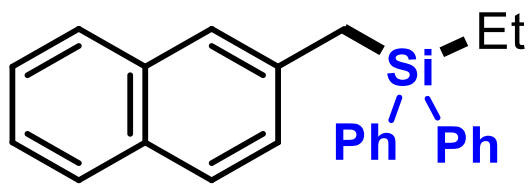

Ethyl(naphthalen-2-ylmethyl)diphenylsilane (3aae)

The title compound was prepared according to the general procedure as a colorless oil (42.3 mg, 60% yield). ^{1}H NMR (400 MHz, $CDCl_3$) δ = 7.74 (d, *J* = 6.4 Hz, 1H), 7.59-7.48 (m, 2H), 7.47-7.46 (m, 4H), 7.41-7.31 (m, 9H), 6.98 (d, *J* = 8.4 Hz, 1H) 2.82 (s, 2H), 1.06-1.99 (m, 5H); ^{13}C NMR (101 MHz, $CDCl_3$) δ = 136.7, 135.31, 135.28, 133.7, 131.2, 129.5, 128.3, 127.9, 127.6, 127.5, 127.2, 126.3, 125.8, 124.6, 23.1, 7.5, 3.6. HRMS (ESI^+): Calcd for $C_{25}H_{24}SiNa[M+Na]^+$ 375.1540, Found 375.1548.

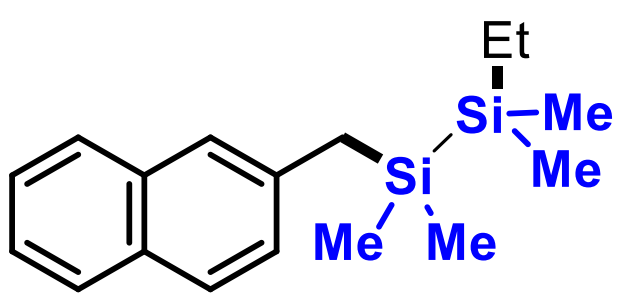

1-Ethyl-1,1,2,2-tetramethyl-2-(naphthalen-2-ylmethyl)disilane (3aaf)

The title compound was prepared according to the general procedure as a colorless oil (28.7 mg, 50% yield). ^{1}H NMR (600 MHz, $CDCl_3$) δ = 7.76 (d, *J* = 7.8 Hz, 1H), 7.69 (d, *J* = 8.4 Hz, 2H), 7.40 (t, *J* = 7.2 Hz, 2H), 7.35 (t, *J* = 7.8 Hz, 1H), 7.15 (d, *J* = 8.4 Hz, 1H), 2.30 (s, 2H), 0.92 (t, *J* = 7.8 Hz, 3H), 0.56 (q, *J* = 7.8 Hz, 2H), 0.02 (s, 6H), -0.01 (s, 6H); ^{13}C NMR (101 MHz, $CDCl_3$) δ = 138.4, 133.9, 131.1, 128.1, 127.7, 127.6, 127.0, 125.8, 125.3, 124.4, 25.3, 8.2, 6.9, -3.8, -4.4. HRMS (ESI^+): Calcd for $C_{17}H_{26}SiNa[M+Na]^+$ 309.1466, Found 309.1468.

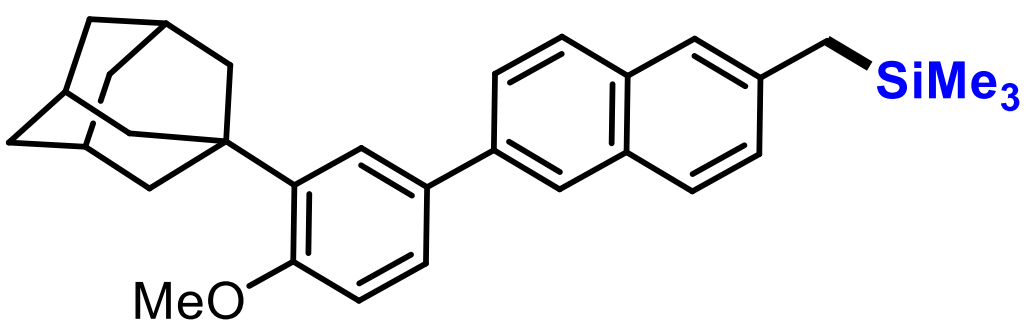

((6-(3-((3*r*,5*r*,7*r*)-Adamantan-1-yl)-4-methoxyphenyl)naphthalen-2-yl)methyl)trimethylsilane (3aag)

The title compound was prepared according to the general procedure as a colorless oil (2-pyridyl ether: 59.1 mg, 65% yield; methyl ether: 60.9 mg, 67% yield). ^{1}H NMR (400 MHz, $CDCl_3$) δ = 8.04 (s, 1H), 7.87 (dd, *J* = 8.3, 6.3 Hz, 2H), 7.79 (dd, *J* = 8.5, 1.7 Hz, 1H), 7.70 (d, *J* = 2.3 Hz, 1H), 7.62 (dd, *J* = 8.4, 2.3 Hz, 1H), 7.55 (s, 1H), 7.31-7.26 (m, 1H), 7.07 (d, *J* = 8.4 Hz, 1H), 3.98 (s, 3H), 2.37 (s, 2H), 2.31 (d, *J* = 2.3 Hz, 6H), 2.22 (s, 3H), 1.92 (s, 6H), 0.15 (s, 9H); ^{13}C NMR (101 MHz, $CDCl_3$) δ = 158.5, 138.9, 138.1, 137.7, 133.6, 132.8, 131.4, 128.4, 127.8, 127.5, 126.0, 125.8, 125.6, 125.0, 125.0, 112.2, 55.3, 40.8, 37.3, 29.3, 27.5, -1.7. HRMS (ESI$^+$): Calcd for $C_{31}H_{39}SiO$ $[M+H]^+$ 455.2765, Found 455.2775.

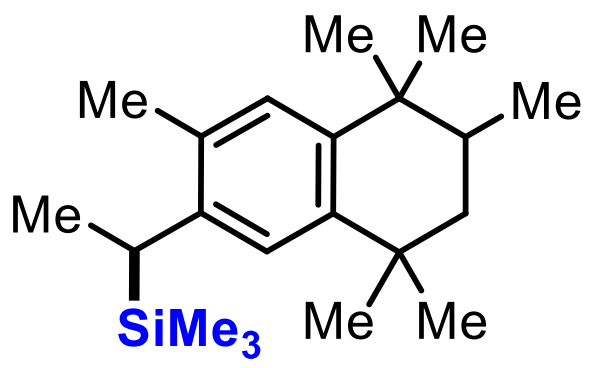

1-(3,5,5,6,8,8-Hexamethyl-5,6,7,8-tetrahydronaphthalen-2-yl)ethyl)trimethylsilane (3aah)

The title compound was prepared according to the general procedure as a colorless oil (2-pyridyl ether: total: 28.7 mg, 70% yield, d.r. = 1:1). ^{1}H NMR (400 MHz, $CDCl_3$) δ = 7.03 (d, *J* = 2.8 Hz, 1H), 6.92 (s, 1H), 2.33-2.28 (m, 1H), 2.19 (s, 3H), 1.87-1.80 (m, 1H), 1.65-1.57 (m, 1H), 1.35-1.21 (m, 13H), 1.02 (d, *J* = 8.4 Hz, 3H), 0.97 (d, *J* = 6.8 Hz, 3H), -0.06 (d, *J* = 4.8 Hz, 9H); ^{13}C NMR (101 MHz, $CDCl_3$) δ = 141.9, 141.7, 141.4, 141.3, 141.14, 141.11, 131.8, 131.7, 128.2, 128.1, 124.2, 124.0, 44.0, 37.2, 34.8, 34.11, 34.06, 32.5, 32.4, 32.2, 28.8, 28.6, 25.2, 25.1, 23.8, 23.7, 20.3, 17.0, 15.8, 15.7, -2.8. HRMS (ESI$^+$): Calcd for $C_{21}H_{36}SiNa$$[M+Na]^+$ 339.2479, Found 339.2480.

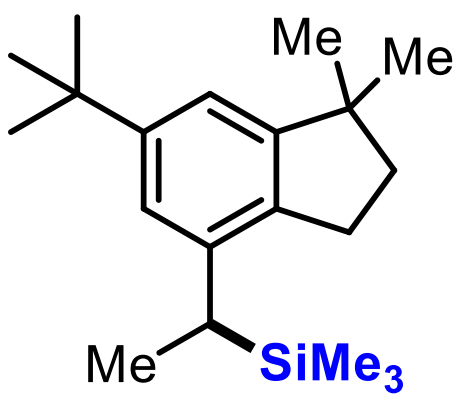

(1-(6-(*tert*-butyl)-1,1-dimethyl-2,3-dihydro-1*H*-inden-4-yl)ethyl)trimethylsilane (3aai)

The title compound was prepared according to the general procedure as a colorless oil (2-pyridyl ether: 48.4 mg, 80% yield; methyl ether: 49.6 mg, 82% yield). ^{1}H NMR (400 MHz, $CDCl_3$) δ = 6.92-6.89 (m, 2H), 2.77-2.67 (m, 2H), 2.27-2.22 (m, 1H), 1.87 (t, *J* = 6.8 Hz, 2H), 1.38 (d, *J* = 6.8 Hz, 3H), 1.32 (s, 9H), 1.28 (s, 3H), 1.20 (s, 3H), -0.06 (s, 9H); ^{13}C NMR (101 MHz, $CDCl_3$) δ = 151.7, 149.4, 141.1, 137.1, 121.1,

114.6, 44.3, 41.4, 34.8, 31.8, 29.1, 28.7, 28.5, 26.0, 15.2, -3.0. HRMS (ESI$^+$): Calcd for $C_{20}H_{34}SiNa[M+Na]^+$ 325.2322, Found 325.2320.

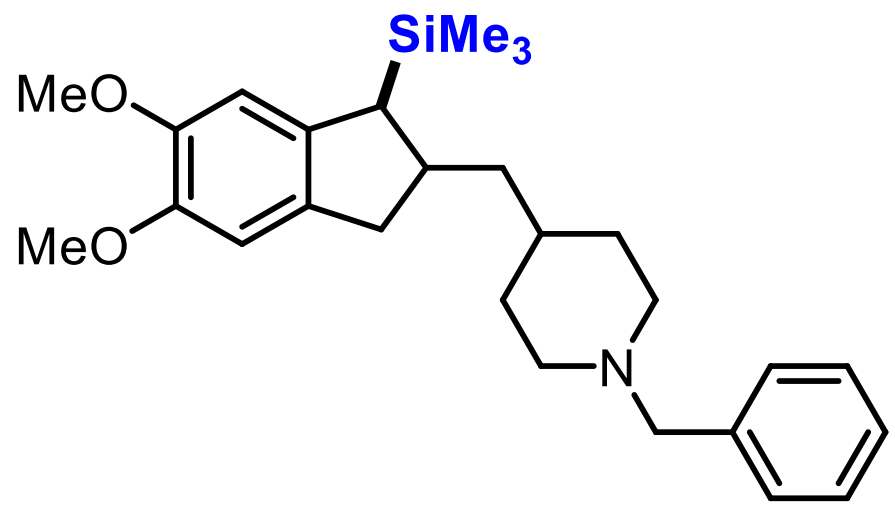

1-Benzyl-4-((-5,6-dimethoxy-1-(trimethylsilyl)-2,3-dihydro-1*H*-inden-2-yl)methyl)piperidine (3aaj)

The title compound was prepared according to the general procedure as a colorless solid (2-pyridyl ether: total: 56.9 mg, 65% yield, d.r. = 1:1). ^{1}H NMR (400 MHz, $CDCl_3$) δ = 7.64-7.59 (m, 1H), 7.50-7.48 (m, 1H), 7.31-7.30 (m, 3H), 6.69 (s, 1H), 6.57 (s, 1H), 3.82 (s, 6H), 3.50 (s, 2H), 3.01-2.95 (m, 1H), 2.97-2.87 (m, 2H), 2.50-2.40 (m, 2H), 1.98-1.88 (m, 2H), 1.71-1.64 (m, 2H), 1.29-1.24 (m, 6H), -0.03 (s, 9H); ^{13}C NMR (101 MHz, $CDCl_3$) δ = 147.6, 146.9, 139.6, 138.1, 136.8, 133.0, 132.2, 132.1, 129.6, 129.5, 129.4, 128.3, 127.1, 108.2, 107.6, 63.6, 56.0, 54.01, 53.96, 45.1, 41.8, 39.5, 37.6, 33.6, 32.6, 32.5, -2.5. HRMS (ESI$^+$): Calcd for $C_{27}H_{40}NO_2Si$ $[M+H]^+$ 438.2823, Found 438.2835.

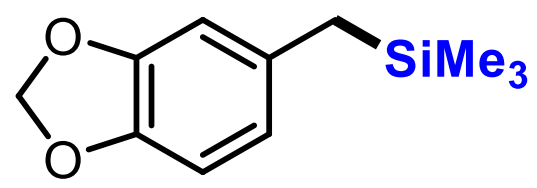

(Benzo[*d*][1,3]dioxol-5-ylmethyl)trimethylsilane (3aak)

The title compound was prepared according to the general procedure as a colorless oil (2-pyridyl ether: 27.9 mg, 67% yield). ^{1}H NMR (400 MHz, $CDCl_3$) δ = 6.68 (d, J = 7.9 Hz, 1H), 6.50 (s, 1H), 6.44 (d, J = 7.9 Hz, 1H), 5.89 (s, 2H), 2.00 (s, 2H), -0.01 (s, 9H); ^{13}C NMR (101 MHz, $CDCl_3$) δ = 147.5, 144.4, 134.3, 120.5, 108.8, 108.2, 100.7, 26.7, -1.8. HRMS (ESI$^+$): Calcd for $C_{11}H_{16}SiO_2Na[M+Na]^+$ 231.0812, Found 231.0814.

General procedure for the deoxygenative silylation of allyl alcohols 4'' with chlorosilanes 2 by chromium in the presence of dtbpy

A dried Schlenk tube was charged with allyl alcohol **4''** (0.2 mmol), $CrCl_3$ (10 mol%), dtbpy (10 mol%) and chlorosilanes **2** (1.0 mmol), followed by adding a freshly distilled THF (0.8 mL) by syringe under atmosphere of nitrogen. After stirring at room temperature for 5 min, EtMgBr (1.0 M in THF, 1.2 mmol) was added dropwise, and the reaction mixture was stirred at room temperature for 12 h. After

quenched by aqueous NH_4Cl (2 mL), the crude product was extracted with ethyl acetate (3 x 10 mL). The combined organic phases were dried over anhydrous Na_2SO_4 and concentrated under vacuum. The residue was purified by flash chromatography on silica gel (petroleum ether) to give the desired product **5**.

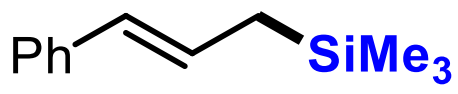

Cinnamyltrimethylsilane (5a)

The title compound was prepared according to the general procedure as a colorless oil (31.2 mg, 82% yield). ^{1}H NMR (400 MHz, $CDCl_3$) δ = 7.30 (dd, *J* = 13.3, 6.1 Hz, 4H), 7.18 (d, *J* = 6.9 Hz, 1H), 6.27 (t, *J* = 5.0 Hz, 2H), 1.71-1.67 (m, 2H), 0.08 (s, 9H); ^{13}C NMR (101 MHz, $CDCl_3$) δ = 138.6, 128.6, 128.4, 127.9, 126.3, 125.7, 24.1, -1.7. HRMS (ESI$^+$): Calcd for $C_{12}H_{18}SiNa[M+Na]^+$ 213.1070, Found 213.1081.

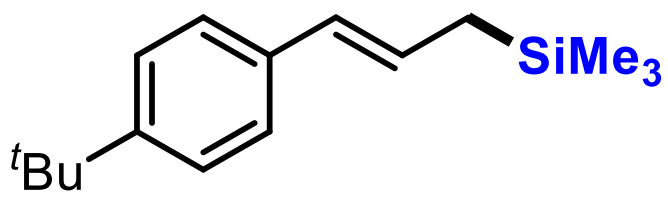

(*E*)-(3-(4-(*tert*-butyl)phenyl)allyl)trimethylsilane (5b)

The title compound was prepared according to the general procedure as a colorless oil (39.5 mg, 80% yield). ^{1}H NMR (400 MHz, $CDCl_3$) δ = 7.32 (d, *J* = 8.4 Hz, 2H), 7.26 (d, *J* = 8.4 Hz, 2H), 6.25-6.22 (m, 2H), 1.66 (d, *J* = 7.2 Hz, 2H), 1.33 (s, 9H), 0.06 (s, 9H); ^{13}C NMR (101 MHz, $CDCl_3$) δ = 149.2, 135.9, 128.1, 127.1, 125.5, 125.3, 34.6, 31.5, 24.0, -1.7. HRMS (ESI$^+$): Calcd for $C_{16}H_{26}SiNa[M+Na]^+$ 269.1696, Found 269.1697.

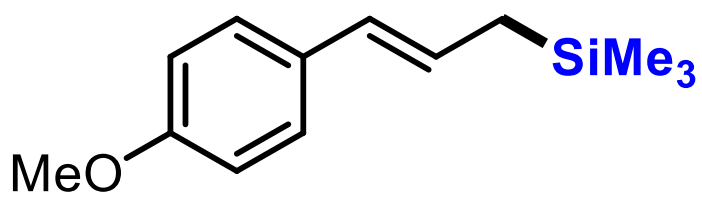

(*E*)-(3-(4-methoxyphenyl)allyl)trimethylsilane (5c)

The title compound was prepared according to the general procedure as a colorless oil (35.3 mg, 87% yield). ^{1}H NMR (400 MHz, $CDCl_3$) δ = 7.23 (d, *J* = 8.8 Hz, 2H), 6,82 (d, *J* = 8.8 Hz, 2H), 6.17 (d, *J* = 15.6 Hz, 1H), 6.10 (dt, *J* = 15.6, 7.8Hz, 1H), 3.80 (s, 3H), 1.63 (d, *J* = 8.4 Hz, 2H), 0.05 (s, 9H); ^{13}C NMR (101 MHz, $CDCl_3$) δ = 158.3, 131.5, 127.7, 126.6, 125.7, 114.0, 55.4, 23.8, 1.7. HRMS (ESI$^+$): Calcd for $C_{13}H_{20}OSiNa[M+Na]^+$ 243.1176, Found 243.1170.

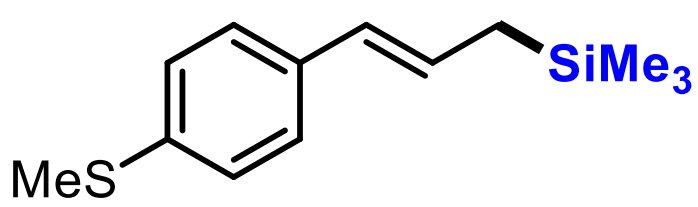

(*E*)-trimethyl(3-(4-(methylthio)phenyl)allyl)silane (5d)

The title compound was prepared according to the general procedure as a colorless oil (39.3 mg, 83% yield). ^{1}H NMR (600 MHz, $CDCl_3$) δ = 7.23-7.21 (m, 2H), 7.19 (d, *J* = 8.4 Hz, 2H), 6.24-6.16 (m, 2H), 2.46 (s, 3H), 1.66 (d, *J* = 7.2 Hz, 2H), 0.04 (s, 9H); ^{13}C NMR (101 MHz, $CDCl_3$) δ = 135.9, 135.8, 127.7, 127.6, 127.3, 126.0, 24.1, 16.4, -1.8. HRMS (ESI$^+$): Calcd for $C_{13}H_{20}SSiNa[M+Na]^+$ 259.0948, Found 259.0955.

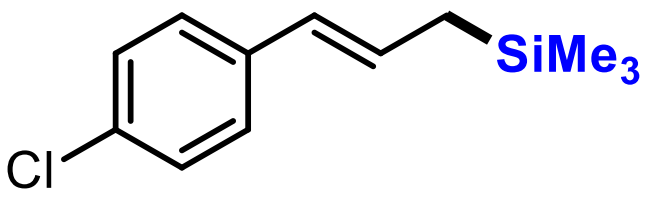

(*E*)-(3-(4-chlorophenyl)allyl)trimethylsilane (5e)

The title compound was prepared according to the general procedure as a colorless oil (31.5 mg, 70% yield). ^{1}H NMR (400 MHz, $CDCl_3$) δ = 7.26-7.18 (m, 4H), 6.26-6.14 (m, 2H), 1.67 (d, *J* = 7.2 Hz, 2H), 0.04 (s, 9H); ^{13}C NMR (101 MHz, $CDCl_3$) δ = 137.1, 131.7, 128.8, 128.6, 127.1, 126.7, 24.1, -1.8 HRMS (ESI$^+$): Calcd for $C_{12}H_{17}ClSiNa[M+Na]^+$ 247.0681, Found 247.0690.

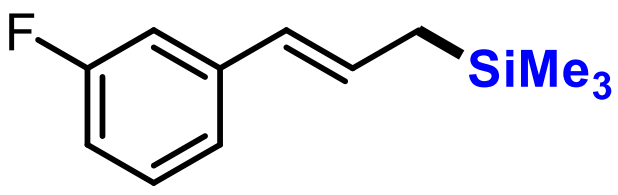

(*E*)-(3-(3-fluorophenyl)allyl)trimethylsilane (5f)

The title compound was prepared according to the general procedure as a colorless oil (33.3 mg, 80% yield). ^{1}H NMR (400 MHz, $CDCl_3$) δ = 7.24-7.18 (m, 1H), 7.05-6.97 (m, 2H), 6.83 (d, *J* = 10.8 Hz, 1H), 6.29-6.16 (m, 2H), 1.66 (d, *J* = 7.6 Hz, 2H), 0.04 (s, 9H); ^{13}C NMR (101 MHz, $CDCl_3$) δ = 163.3 (d, *J* = 248.5 Hz), 141.0 (d, *J* = 8.1 Hz), 129.9 (d, *J* = 9.1 Hz), 129.6, 127.3 (d, *J* = 3.0 Hz), 121.4, 113.0 (d, *J* = 21.2 Hz) , 111.9 (d, *J* = 22.2 Hz), 24.1, -1.8. HRMS (ESI$^+$): Calcd for $C_{12}H_{17}FSiNa[M+Na]^+$ 231.0976, Found 231.0980.

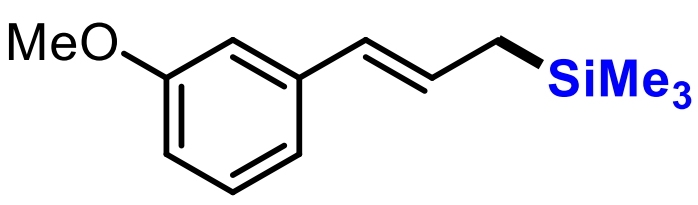

(*E*)-(3-(3-methoxyphenyl)allyl)trimethylsilane (5g)

The title compound was prepared according to the general procedure as a colorless oil (35.3 mg, 80% yield). ^{1}H NMR (400 MHz, $CDCl_3$) δ = 7.21 (d, *J* = 8.8 Hz, 2H), 6.83 (d, *J* = 8.4 Hz, 2H), 6.19-6.04 (m, 2H), 1.61 (d, *J* = 8.0 Hz, 2H), 0.03 (s, 9H); ^{13}C NMR (101 MHz, $CDCl_3$) δ = 158.3, 131.5, 127.7, 126.6, 125.7, 114.0, 55.4, 23.8, -1.8. HRMS (ESI$^+$): Calcd for $C_{13}H_{20}OSiNa[M+Na]^+$ 243.1176, Found 243.1172.

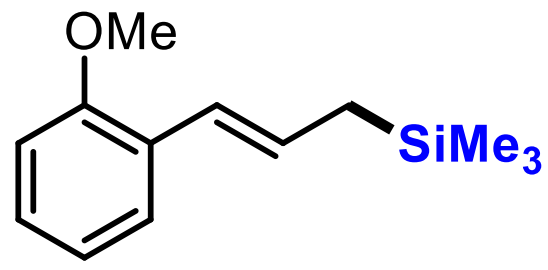

(*E*)-(3-(2-methoxyphenyl)allyl)trimethylsilane (5h)

The title compound was prepared according to the general procedure as a colorless oil (34.0 mg, 77% yield). ^{1}H NMR (400 MHz, $CDCl_3$) δ = 7.35 (d, *J* = 7.5 Hz, 1H), 7.15 -7.09 (m, 1H), 6.87 (t, *J* = 7.5 Hz, 1H), 6.81 (d, *J* = 8.2 Hz, 1H), 6.53 (d, *J* = 15.8 Hz, 1H), 6.21 (dt, *J* = 15.8, 8.2 Hz, 1H), 3.80 (s, 3H), 1.68 (d, *J* = 8.4 Hz, 2H), 0.04 (s, 9H); ^{13}C NMR (101 MHz, $CDCl_3$) δ = 156.1, 128.6, 127.8, 127.3, 126.2, 123.0, 120.8, 111.0, 55.6, 24.5, -1.7. HRMS (ESI$^+$): Calcd for $C_{13}H_{21}OSi[M+H]^+$ 221.1356, Found 221.1361.

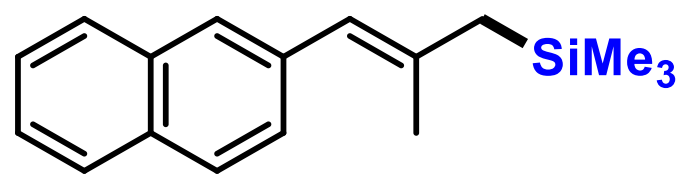

(*E*)-trimethyl(2-methyl-3-(naphthalen-2-yl)allyl)silane (5i)

The title compound was prepared according to the general procedure as a colorless oil (total: 45.3 mg, 89% yield; *E/Z* = 9:1). ^{1}H NMR (400 MHz, $CDCl_3$): *δ* = 7.81-7.76 (m, 3H), 7.67 (s, 1H), 7.47-7.36 (m, 3H), 6.31 (s, 1H), 1.77 (s, 3H), 1.94 (s, 2H), 0.07 (s, 9H); ^{13}C NMR (101 MHz, $CDCl_3$): *δ* = 138.6, 136.9, 133.6, 131.8, 127.8, 127.6, 127.5, 126.6, 125.9, 125.2, 122.7, 27.7, 24.2, -0.3. HRMS (ESI$^+$): Calcd for $C_{17}H_{23}Si[M+H]^+$ 255.1564, Found 255.1566.

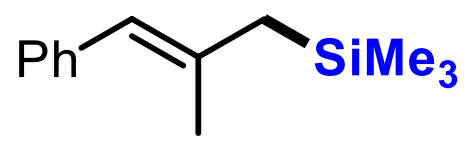

(*E*)-Trimethyl(2-methyl-3-phenylallyl)silane (5j)

The title compound was prepared according to the general procedure as a colorless oil (38.5 mg, 94% yield). ^{1}H NMR (400 MHz, $CDCl_3$) δ = 7.26 (t, *J* = 7.5 Hz, 2H), 7.22-7.16 (m, 2H), 7.12 (t, *J* = 7.2 Hz, 1H), 6.11 (d, *J* = 23.1 Hz, 1H), 1.89-1.81 (m, 5H), 0.00 (s, 9H); ^{13}C NMR (101 MHz, $CDCl_3$) δ = 139.4, 137.8, 128.6, 128.2, 125.6, 122.8, 27.6, 23.9, -0.4. HRMS (ESI$^+$): Calcd for $C_{13}H_{21}Si[M+H]^+$ 205.1407, Found 205.1401.

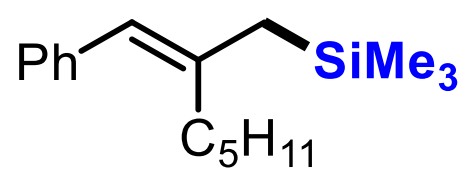

(*E*)-(2-Benzylideneheptyl)trimethylsilane (5k)

The title compound was prepared according to the general procedure as a colorless oil (49.5 mg, 95% yield). ^{1}H NMR (400 MHz, $CDCl_3$) δ = 7.28 (t, *J* = 7.8 Hz, 2H), 7.21 (d, *J* = 7.8 Hz, 2H), 7.18-7.12 (m, 1H), 6.14 (s, 1H), 2.09 (t, *J* = 7.2 Hz, 2H), 1.87 (s, 2H), 1.53-1.50 (m, 2H), 1.37-1.30 (m, 2H), 0.91 (t, *J* = 7.2 Hz, 3H), -0.01 (s, 9H); ^{13}C NMR (101 MHz, CDCl3) δ = 142.0, 139.5, 128.7, 128.1, 125.4, 121.9, 40.3, 31.8, 28.1, 22.7, 21.8, 14.2, -0.5. HRMS (ESI$^+$): Calcd for $C_{17}H_{28}SiNa[M+Na]^+$ 283.1853, Found 283.1858.

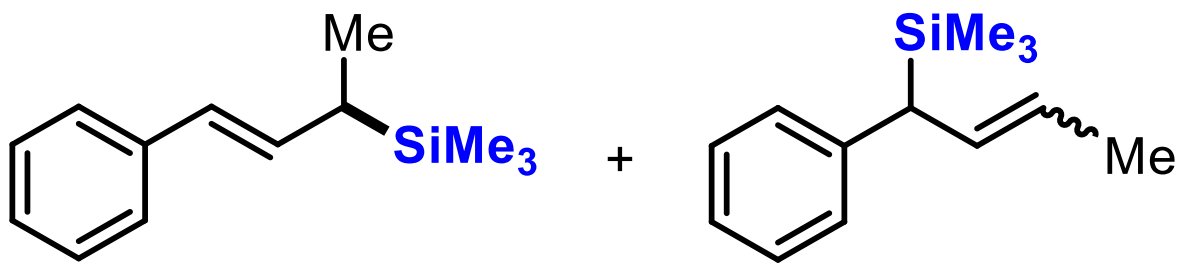

5l **5l'**

(*E*)-Trimethyl(4-phenylbut-3-en-2-yl)silane (5l), (*E*)-Trimethyl(1-phenylbut-2-en-1-yl)silane (*E*-5l') and (*Z*)-Trimethyl(1-phenylbut-2-en-1-yl)silane (*Z*-5l')

The mixture of title compounds was prepared according to the general procedure as a colorless oil (total: 33.5 mg, 82% yield; r.r = 3:1; *E*/*Z* = 1.7:1). **(5l):** ^{1}H NMR (400 MHz, $CDCl_3$) δ = 7.33-7.05 (m, 5H), 6.31 (dd, *J* = 15.8, 7.8 Hz, 1H), 6.20 (d, *J* = 15.8 Hz, 1H), 1.82-1.74 (m, 1H), 1.16 (d, *J* = 7.2 Hz, 3H), 0.02 (s, 9H); **(*E*-5l'):** ^{1}H NMR (400 MHz, $CDCl_3$) δ = 7.33-7.05 (m, 1H), 5.88-5.76 (m, 0.4H), 3.27 (d, *J* = 11.6 Hz, 0.2H), 1.64 (dd, *J* = 6.8, 1.8 Hz, 0.6H), -0.04 (s, 1.8H); **(Z-5l'):** ^{1}H NMR (400 MHz, $CDCl_3$) δ = 7.33-7.05 (m, 0.6H), 5.52-5.47 (m, 0.12H), 5.43-5.38 (m, 0.12H), 2.86 (d, *J* = 9.6 Hz, 0.12H), 1.70 (dd, *J* = 6.2, 1.6 Hz, 0.36H), -0.05 (s, 1.1H); ^{13}C NMR (101 MHz, CDCl3) δ = 138.7, 134.6, 130.3, 129.7, 128.6, 128.3, 127.20, 127.15, 126.3, 125.74, 125.67, 124.5, 123.6, 121.9, 42.9, 37.5, 27.4, 18.2, 13.6, 13.3, -2.89, -2.91, -3.3. HRMS (ESI$^+$): Calcd for $C_{13}H_{20}SiNa[M+Na]^+$ 227.1227, Found 227.1229.

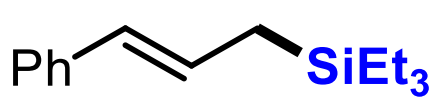

(*E*)-Cinnamyltriethylsilane (5m)

The title compound was prepared according to the general procedure as a colorless oil (37.2 mg, 80% yield). ^{1}H NMR (400 MHz, $CDCl_3$) δ = 7.32-7.25 (m, 4H), 7.17-7.09 (m, 1H), 6.29-6.21 (m, 2H), 1.71 (dd, *J* = 4.8, 2.0 Hz, 4H), 0.97 (t, *J* = 8.0 Hz, 9H), 0.58 (q, *J* = 8.0 Hz, 6H); ^{13}C NMR (101 MHz, $CDCl_3$) δ = 138.6, 128.5, 128.2, 128.1, 126.2, 125.5, 18.9, 7.5, 3.3. HRMS (ESI$^+$): Calcd for $C_{15}H_{24}SiNa[M+Na]^+$ 255.1540, Found 255.1550.

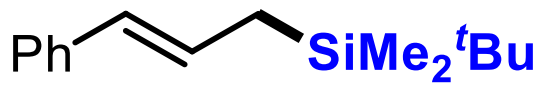

(*E*)-*tert*-butyl(cinnamyl)dimethylsilane (5n)

The title compound was prepared according to the general procedure as a colorless oil (41.1 mg, 88% yield). ^{1}H NMR (400 MHz, $CDCl_3$) δ = 7.30-7.26 (m, 4H), 7.17-7.14 (m, 1H), 6.31-6.21 (m, 2H), 1.71 (d, J = 6.8 Hz, 2H), 0.92 (s, 9H), -0.004 (s, 6H); ^{13}C NMR (101 MHz, $CDCl_3$) δ = 138.6, 128.6, 128.4, 128.3, 126.3, 125.6, 26.7, 20.1, 16.9, -6.3. HRMS (ESI$^+$): Calcd for $C_{15}H_{24}SiNa[M+Na]^+$ 255.1540, Found 255.1541.

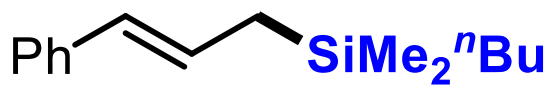

(*E*)-Butyl(cinnamyl)dimethylsilane (5o)

The title compound was prepared according to the general procedure as a colorless oil (40.2 mg, 86% yield). ^{1}H NMR (400 MHz, $CDCl_3$) δ = 7.30-7.25 (m, 4H), 7.17-7.13 (m, 1H), 6.25-6.22 (m, 2H), 1.68 (d, J = 8.0 Hz, 2H), 1.34-1.28 (m, 4H), 0.89 (t, J = 6.8 Hz, 3H), 0.58-0.53 (m, 2H), 0.02 (s, 6H); ^{13}C NMR (101 MHz, $CDCl_3$) δ = 138.6, 128.5, 128.2, 128.1, 126.3, 125.6, 26.7, 26.1, 22.7, 14.8, 14.0, -3.4. HRMS (ESI$^+$): Calcd for $C_{15}H_{24}SiNa[M+Na]^+$ 255.1540, Found 255.1550.

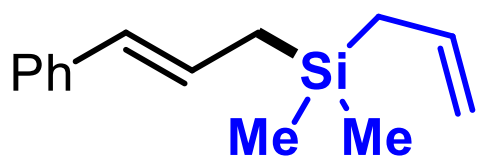

(*E*)-Allyl(cinnamyl)dimethylsilane (5p)

The title compound was prepared according to the general procedure as a colorless oil (38.1 mg, 86% yield). ^{1}H NMR (400 MHz, $CDCl_3$) δ = 7.30-7.27 (m, 4H), 7.17-7.14 (m, 1H), 6.24-6.21 (m, 2H), 5.85-5.74 (m, 1H), 4.89-4.85 (m, 2H), 1.70-1.68 (m, 2H), 1.56 (d, J = 8.0 Hz, 2H), 0.04 (s, 6H); ^{13}C NMR (101 MHz, $CDCl_3$) δ = 138.5, 134.8, 128.7, 128.5, 127.4, 126.4, 125.6, 113.4, 23.0, 22.1, -3.8. HRMS (ESI$^+$): Calcd for $C_{14}H_{20}SiNa[M+Na]^+$ 239.1227, Found 239.1230.

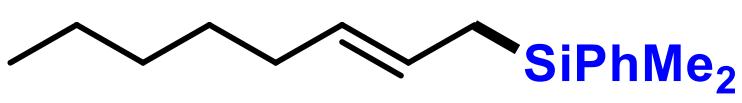

(*E*)-Dimethyl(oct-2-en-1-yl)(phenyl)silane (5q)

The title compound was prepared according to the general procedure as a colorless oil (43.4 mg, 88% yield). ^{1}H NMR (400 MHz, $CDCl_3$) δ = 7.51-7.49 (m, 2H), 7.35-7.34 (m, 3H), 5.38-5.33 (m, 1H), 5.28-5.23 (m, 1H), 1.94 (q, J = 7.2 Hz, 2H), 1.64 (d, J = 8.4 Hz, 2H), 1.31-1.21 (m, 6H), 0.87 (t, J = 7.2 Hz, 3H), 0.25 (s, 6H); ^{13}C NMR (101 MHz, $CDCl_3$) δ = 139.2, 133.7, 130.0, 129.0, 127.8, 125.3, 32.8, 31.4, 29.7, 22.6, 21.7, 14.2, -3.3. HRMS (ESI$^+$): Calcd for $C_{16}H_{26}SiNa[M+Na]^+$ 269.1696, Found 269.1695.

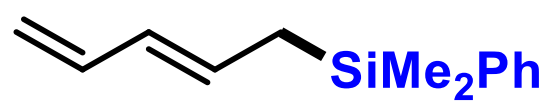

(*E*)-Dimethyl(penta-2,4-dien-1-yl)(phenyl)silane (5r)

The title compound was prepared according to the general procedure as a colorless oil (total: 36.4 mg, 90% yield; *E/Z* = 5:1). **(*E*):** ^{1}H NMR (400 MHz, $CDCl_3$) δ = 7.51-7.49 (m, 2H), 7.36-7.34 (m, 3H), 6.29-6.22 (m, 1H), 5.96-5.90 (m, 1H), 5.70-5.67 (m, 1H), 5.00 (dd, *J* = 16.8, 1.4 Hz, 1H), 4.85 (dd, *J* = 10.0, 1.4 Hz, 1H), 1.76 (d, *J* = 8.4 Hz, 2H), 0.28 (s, 6H); **(*Z*):** ^{1}H NMR (400 MHz, $CDCl_3$) δ = 7.51-7.49 (m, 0.4H), 7.36-7.34 (m, 0.6H), 6.59-6.49 (m, 0.2H), 5.96-5.90 (m, 0.2H), 5.51-5.44 (m, 0.2H), 5.12 (d, *J* = 16.8 Hz, 0.2H), 5.01-4.96 (m, 0.2H), 1.89 (dd, *J* = 8.8, 1.2Hz, 0.4H), 0.28 (s, 1.2H); ^{13}C NMR (101 MHz, $CDCl_3$) δ = 138.6, 137.6, 133.7, 132.4, 131.6, 130.5, 129.2, 127.9, 123.1, 22.7, 10.8, -3.3. HRMS (ESI$^+$): Calcd for $C_{13}H_{18}SiNa[M+Na]^+$ 225.1070, Found 225.1071.

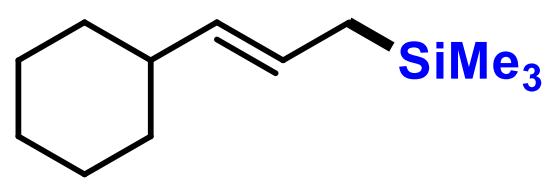

(*E*)-(3-Cyclohexylallyl)trimethylsilane (5s)

The title compound was prepared according to the general procedure as a colorless oil (34.6 mg, 88% yield). ^{1}H NMR (400 MHz, $CDCl_3$) δ = 5.32 (dt, *J* = 15.3, 7.7 Hz, 1H), 5.18 (dd, *J* = 15.2, 6.8 Hz, 1H), 1.92-1.85 (m, 1H), 1.08-1.01 (m, 2H), -0.04 (s, 9H); ^{13}C NMR (101 MHz, $CDCl_3$) δ = 135.3, 123.4, 41.1, 33.7, 26.3, 26.2, 22.6, -2.0. HRMS (ESI$^+$): Calcd for $C_{12}H_{24}SiNa[M+Na]^+$ 219.1540, Found 219.1545.

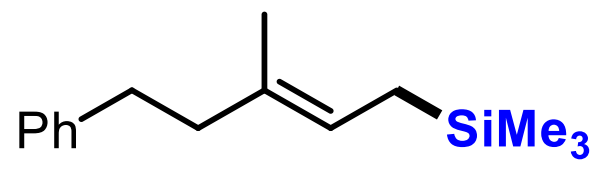

(*E*)-Trimethyl(3-methyl-5-phenylpent-2-en-1-yl)silane (5t)

The title compound was prepared according to the general procedure as a colorless oil (39.5 mg, 85% yield). ^{1}H NMR (400 MHz, $CDCl_3$) δ = 7.29-7.24 (m, 2H), 7.22-7.16 (m, 3H), 5.17 (t, *J* = 8.4 Hz, 1H), 2.70 (t, *J* = 8.4 Hz, 2H), 2.30 (t, *J* = 8.4 Hz, 2H), 1.60 (s, 3H), 1.39 (d, *J* = 8.4 Hz, 2H), -0.07 (s, 9H); ^{13}C NMR (101 MHz, $CDCl_3$) δ = 142.6, 131.8, 128.5, 128.3, 125.7, 121.0, 41.8, 35.1, 18.7, 16.0, -1.7. HRMS (ESI$^+$): Calcd for $C_{15}H_{24}SiNa[M+Na]^+$ 255.1540, Found 255.1542.

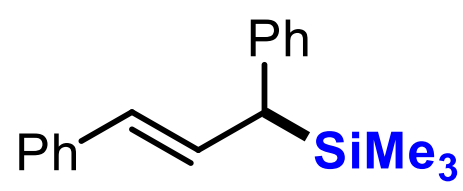

(*E*)-(1,3-Diphenylallyl)trimethylsilane (5u)

The title compound was prepared according to the general procedure as a colorless oil (45.8 mg, 86% yield). ^{1}H NMR (400 MHz, $CDCl_3$) δ = 7.38-7.36 (m, 2H), 7.33-7.28 (m, 4H), 7.21-7.12 (m, 4H), 6.62 (dd, *J* = 15.6, 10.0 Hz, 1H), 6.36 (d, *J* = 15.6 Hz, 1H), 3.16 (d, *J* = 10.0 Hz, 1H), 0.03 (s, 9H); ^{13}C NMR (101 MHz, $CDCl_3$) δ = 142.4, 138.3, 130.6, 128.6, 128.5, 128.2, 127.3, 126.7, 126.0, 124.9, 44.0, -2.7. HRMS (ESI$^+$): Calcd for $C_{18}H_{22}SiNa[M+Na]^+$ 289.1383, Found 289.1383.

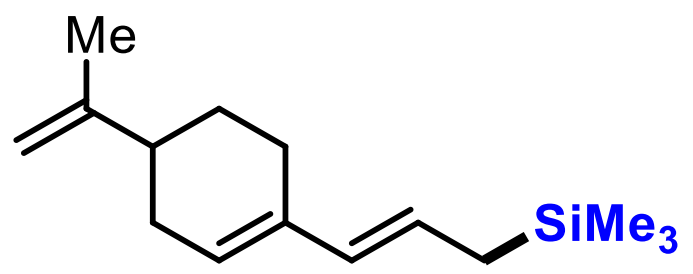

(*E*)-Trimethyl(3-(4-(prop-1-en-2-yl)cyclohex-1-en-1-yl)allyl)silane (5v)

The title compound was prepared according to the general procedure as a colorless oil (methyl ether: total: 39.9 mg, 85% yield, *E/Z* = 9:1). ^{1}H NMR (400 MHz, $CDCl_3$) δ = 5.92 (d, *J* = 15.2 Hz, 1H), 5.58-5.50 (m, 2H), 4.71 (s, 2H), 2.30-2.23 (m, 2H), 2.19-2.13 (m, 3H), 2.05-1.99 (m, 1H), 1.89-1.84 (m, 1H), 1.73 (s, 3H), 1.52 (d, *J* = 7.6 Hz, 2H), -0.01 (s, 9H); ^{13}C NMR (101 MHz, $CDCl_3$) δ = 150.2, 135.7, 131.7, 124.7, 123.6, 108.6, 41.5, 27.7, 25.3, 23.4, 20.9, -1.8. HRMS (ESI$^+$): Calcd for $C_{15}H_{26}SiNa[M+Na]^+$ 257.1696, Found 257.1694.

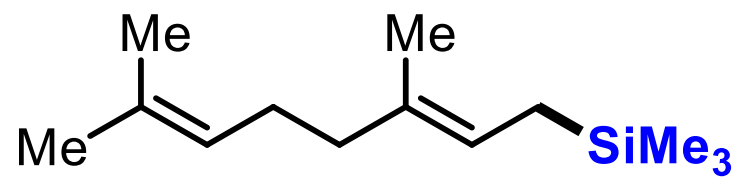

(*E*)-(3,7-Dimethylocta-2,6-dien-1-yl)trimethylsilane (5w)

The title compound was prepared according to the general procedure as a colorless oil (alcohol: 14.7 mg, 35% yield; methyl ether: 35.8 mg, 85% yield). ^{1}H NMR (400 MHz, $CDCl_3$) δ = 5.18-5.06 (m, 2H), 2.09-1.98 (m, 4H), 1.66 (s, 3H), 1.59 (s, 3H), 1.54 (s, 3H), 1.37 (d, *J* = 8.4 Hz, 2H), -0.03 (s, 9H); ^{13}C NMR (101 MHz, $CDCl_3$) δ = 132.3, 131.2, 124.7, 40.1, 26.9, 25.9, 18.7, 17.8, 15.8, -1.7. HRMS (ESI$^+$): Calcd for $C_{13}H_{26}SiNa[M+Na]^+$ 233.1696, Found 233.1698.

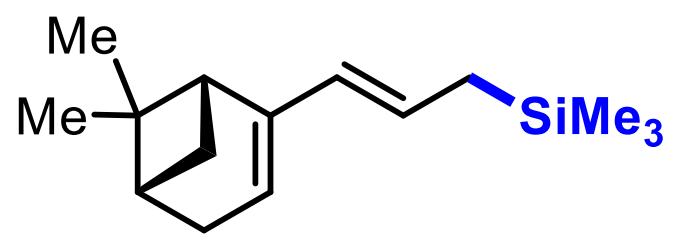

(*E*)-(3-(-6,6-Dimethylbicyclo[3.1.1]hept-2-en-2-yl)allyl)trimethylsilane (5x)

The title compound was prepared according to the general procedure as a colorless oil (methyl ether: total: 35.2 mg, 75% yield, *E/Z* = 5:1). **(*E*):** ^{1}H NMR (400 MHz, $CDCl_3$) δ = 5.88 (d, *J* = 15.6 Hz, 1H), 5.51-5.47 (m, 1H), 5.34-5.30 (m, 1H), 2.49-2.46 (m, 1H), 2.41-2.37 (m, 1H), 2.32-2.28 (m, 2H), 2.12-2.07 (m, 1H), 1.52 (d, *J* = 8.0 Hz,

2H), 1.30 (s, 3H), 1.13 (d, *J* = 8.4 Hz, 1H), 0.78 (s, 3H), -0.02 (s, 9H); **(*Z*):** ^{1}H NMR (400 MHz, $CDCl_3$) δ = 5.64-5.61 (m, 0.2H), 5.45-5.43 (m, 0.2H), 5.34-5.30 (m, 0.2H), 2.41-2.37 (m, 0.6H),, 2.32-2.28 (m, 0.2H), 2.23 (m, 0.2H), 1.75-1.68 (m, 0.4H), 1.28 (s, 0.6H), 1.23 (d, *J* = 4.8 Hz, 0.2H), 0.88 (s, 0.6H), 0.02 (s, 1.8H); ^{13}C NMR (101 MHz, $CDCl_3$) δ = 146.7, 145.4, 130.3, 127.8, 126.0, 123.1, 121.0, 119.6, 46.6, 41.3, 41.2, 40.6, 37.8, 31.9, 31.8, 31.6, 31.4, 26.5, 23.5, 20.8, 20.5, -1.5, -1.8. HRMS (ESI$^+$): Calcd for $C_{15}H_{26}SiNa[M+Na]^+$ 257.1696, Found 257.1700.

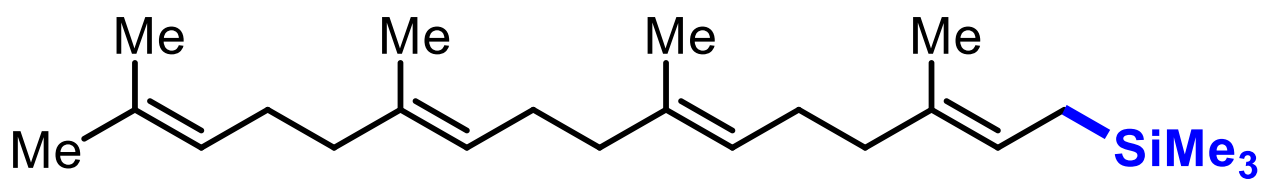

(*E*)-Trimethyl(3,7,11,15-tetramethylhexadeca-2,6,10,14-tetraen-1-yl)silane (5y)

The title compound was prepared according to the general procedure as a colorless oil (alcohol: 27.7 mg, 40% yield; methyl ether: 60.3 mg, 87% yield). ^{1}H NMR (400 MHz, $CDCl_3$) δ = 5.19-5.07 (m, 4H), 2.08-2.05 (m, 6H), 2.02-1.95 (m, 6H), 1.67 (s, 3H), 1.59 (s, 12H), 1.37 (d, *J* = 8.4 Hz, 2H), -0.03 (s, 9H); ^{13}C NMR (101 MHz, $CDCl_3$) δ = 144.9, 135.0, 134.9, 132.4, 131.4, 124.5, 120.3, 40.1, 39.9, 27.0, 26.9, 26.74, 26.69, 25.8, 18.7, 17.8, 16.1, 15.9, -1.6. HRMS (ESI$^+$): Calcd for $C_{23}H_{42}SiNa[M+Na]^+$ 369.2948, Found 369.2948.

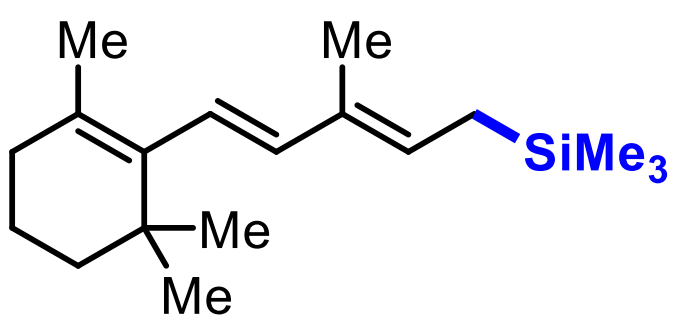

(*E*)-Trimethyl(3-methyl-5-(2,6,6-trimethylcyclohex-1-en-1-yl)penta-2,4-dien-1-yl) silane (5z)

The title compound was prepared according to the general procedure as a colorless oil (alcohol: 24.9 mg, 45% yield; methyl ether: 49.8 mg, 90% yield). ^{1}H NMR (400 MHz, $CDCl_3$) δ = 6.02 (d, *J* = 16.0 Hz, 1H), 5.88 (d, *J* = 16.0 Hz, 1H), 5.48 (t, *J* = 8.4 Hz, 1H), 1.99 (t, *J* = 6.4 Hz, 2H), 1.70 (d, *J* = 13.2 Hz, 6H), 1.63-1.57 (m, 4H), 1.47-1.44 (m, 2H), 1.01 (s, 6H), 0.01 (s, 9H); ^{13}C NMR (101 MHz, $CDCl_3$) δ = 138.4, 138.1, 132.0, 128.0, 127.9,. 122.3, 39.7, 34.3, 33.1, 29.0, 21.8, 20.0, 19.5, 12.3, -1.5. HRMS (ESI$^+$): Calcd for $C_{18}H_{32}SiNa[M+Na]^+$ 299.2166, Found 299.2167.

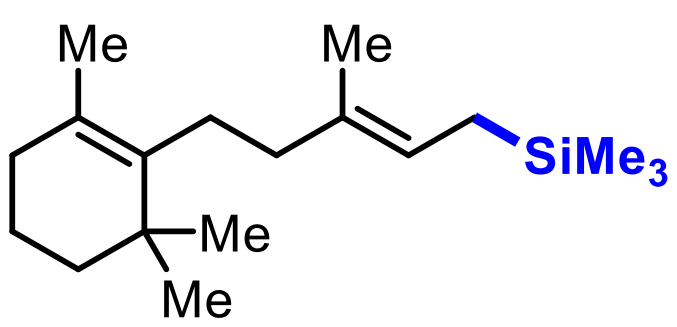

(*E*)-Trimethyl(3-methyl-5-(2,6,6-trimethylcyclohex-1-en-1-yl)pent-2-en-1-yl)silane (5aa)

The title compound was prepared according to the general procedure as a colorless oil (alcohol: 29.0 mg, 52% yield; methyl ether: 46.8 mg, 84% yield). ^{1}H NMR (400 MHz, $CDCl_3$) δ = 5.19 (t, *J* = 8.4 Hz, 1H), 2.06-2.00 (m, 4H), 1.91-1.88 (m, 2H), 1.60-1.59 (m, 6H), 1.57-1.54 (m, 2H), 1.42-1.38 (m, 4H), 0.99 (s, 6H), -0.02 (s, 9H); ^{13}C NMR (101 MHz, $CDCl_3$) δ = 137.4, 133.6, 126.9, 119.6, 40.8, 39.9, 35.1, 32.9, 28.7, 28.5, 19.9, 19.7, 18.5, 15.9, -1.6. HRMS (ESI$^+$): Calcd for $C_{18}H_{34}SiNa[M+Na]^+$ 301.2322, Found 301.2324.

General procedure for the deoxygenative silylation of propargyl alcohols 6'' with chlorosilanes 2 by chromium in the presence of dtbpy

A dried Schlenk tube was charged with propargyl alcohol **6''** (0.2 mmol), $CrCl_3$ (10 mol%), dtbpy (10 mol%) and chlorosilanes **2** (1.0 mmol), followed by adding a freshly distilled THF (0.8 mL) by syringe under atmosphere of nitrogen. After stirring at room temperature for 5 min, EtMgBr (1.0 M in THF, 1.2 mmol) was added dropwise, and the reaction mixture was stirred at room temperature for 12 h. After quenched by aqueous NH_4Cl (2 mL), the crude product was extracted with ethyl acetate (3 x 10 mL). The combined organic phases were dried over anhydrous Na_2SO_4 and concentrated under vacuum. The residue was purified by flash chromatography on silica gel to give the desired product **7**.

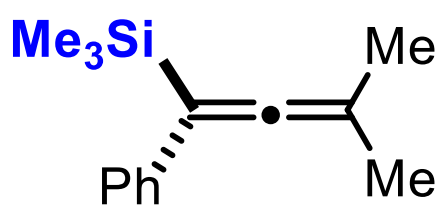

Trimethyl(3-methyl-1-phenylbuta-1,2-dien-1-yl)silane (7a)

The title compound was prepared according to the general procedure as a colorless oil (38.8 mg, 95% yield). ^{1}H NMR (600 MHz, $CDCl_3$) δ = 7.27-7.26 (m, 4H), 7.16-7.13 (m, 1H), 1.80 (s, 6H), 0.2 (s, 9H); ^{13}C NMR (101 MHz, $CDCl_3$) δ = 207.8, 139.0, 128.4, 127.8, 125.8, 98.6, 19.8, -0.1. HRMS (ESI$^+$): Calcd for $C_{14}H_{20}SiNa[M+Na]^+$ 239.1227, Found 239.1230.

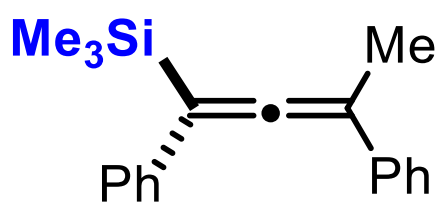

(1,3-Diphenylbuta-1,2-dien-1-yl)trimethylsilane (7b)

The title compound was prepared according to the general procedure as a colorless oil (50.7 mg, 91% yield). ^{1}H NMR (600 MHz, $CDCl_3$) δ = 7.41 (d, *J* = 8.2 Hz, 2H),

7.36-7.28 (m, 6H), 7.22-7.18 (m, 2H), 2.18 (s, 3H), 0.29 (s, 9H); ^{13}C NMR (101 MHz, $CDCl_3$) δ = 209.8, 137.4, 137.3, 128.6, 128.5, 128.0, 126.4, 126.2, 125.2, 103.0, 97.3, 16.3, 0.03. HRMS (ESI$^+$): Calcd for $C_{19}H_{22}SiNa[M+Na]^+$ 301.1383, Found 301.1383.

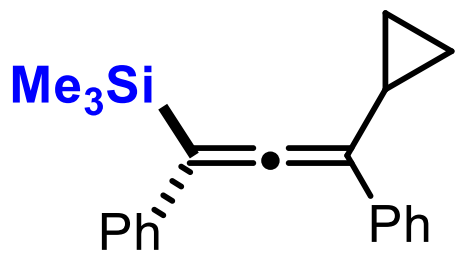

(3-Cyclopropyl-1,3-diphenylpropa-1,2-dien-1-yl)trimethylsilane (7c)

The title compound was prepared according to the general procedure as a colorless oil (51.8 mg, 85% yield). ^{1}H NMR (400 MHz, $CDCl_3$) δ = 7.59 (dd, J = 8.2, 1.2 Hz, 2H), 7.36-7.28 (m, 6H), 7.22-7.19 (m, 2H), 1.68-1.63 (m, 1H), 0.92-0.86 (m, 2H), 0.59-0.53 (m, 2H), 0.27 (s, 9H); ^{13}C NMR (101 MHz, $CDCl_3$) δ = 208.8, 137.4, 137.2, 128.6, 128.5, 127.8, 126.5, 126.4, 125.7, 105.7, 105.4, 10.6, 6.8, 0.1. HRMS (ESI$^+$): Calcd for $C_{21}H_{24}SiNa[M+Na]^+$ 327.1540, Found 327.1546.

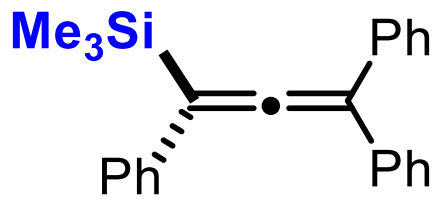

Trimethyl(1,3,3-triphenylpropa-1,2-dien-1-yl)silane (7d)

The title compound was prepared according to the general procedure as a colorless oil (61.3 mg, 90% yield). ^{1}H NMR (600 MHz, $CDCl_3$) δ = 7.42-7.31 (m, 12H), 7.27-7.20 (m, 3H), 0.32 (s, 9H); ^{13}C NMR (101 MHz, $CDCl_3$) δ = 209.6, 136.9, 136.7, 128.7, 128.6, 128.1, 128.0, 126.9, 126.7, 106.7, 103.7, 0.03. HRMS (ESI$^+$): Calcd for $C_{24}H_{24}SiNa[M+Na]^+$ 363.1540, Found 363.1544.

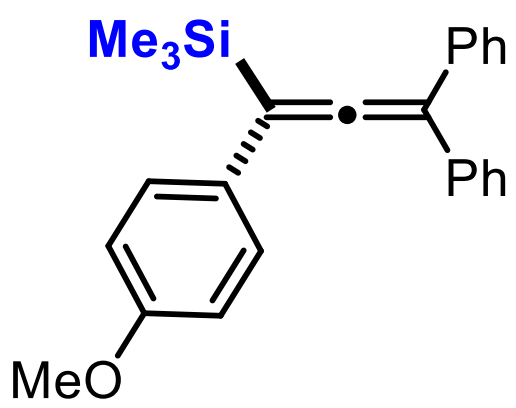

(1-(4-Methoxyphenyl)-3,3-diphenylpropa-1,2-dien-1-yl)trimethylsilane (7e)

The title compound was prepared according to the general procedure as a colorless oil (61.5 mg, 83% yield). ^{1}H NMR (400 MHz, $CDCl_3$) δ = 7.41-7.31 (m, 10H), 7.28-7.23 (m, 2H), 6.88 (d, J = 8.8 Hz, 2H), 3.80 (s, 3H), 0.31 (s, 9H); ^{13}C NMR (101 MHz, $CDCl_3$) δ = 209.2, 158.6, 137.1, 129.0, 128.6, 128.5, 128.1, 126.8, 114.2, 106.7, 103.0, 55.4, 0.1. HRMS (ESI$^+$): Calcd for $C_{25}H_{26}OSiNa[M+Na]^+$ 393.1646, Found 393.1650.

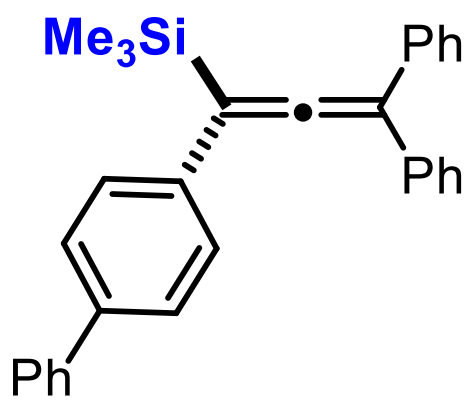

(1-([1,1'-Biphenyl]-4-yl)-3,3-diphenylpropa-1,2-dien-1-yl)trimethylsilane (7f)

The title compound was prepared according to the general procedure as a colorless oil (66.7 mg, 80% yield). ^{1}H NMR (400 MHz, $CDCl_3$) δ = 7.60-7.55 (m, 4H), 7.50 (d, *J* = 8.4 Hz, 2H), 7.45-7.33 (m, 11H), 7.28 (d, *J* = 7.2 Hz, 2H), 0.35 (s, 9H); ^{13}C NMR (101 MHz, $CDCl_3$) δ = 209.9, 140.9, 139.6, 136.8, 135.6, 128.9, 128.3, 128.1, 127.5, 127.3, 127.0, 126.9, 106.9, 103.4, 0.1. HRMS (ESI$^+$): Calcd for $C_{30}H_{28}SiNa[M+Na]^+$ 439.1853, Found:439.1858.

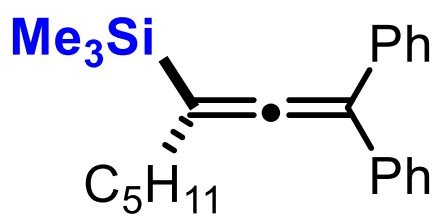

(1,1-Diphenylocta-1,2-dien-3-yl)trimethylsilane (7g)

The title compound was prepared according to the general procedure as a colorless oil (58.2 mg, 87% yield). ^{1}H NMR (400 MHz, $CDCl_3$) δ = 7.30 (d, *J* = 4.4 Hz, 8H), 7.23-7.18 (m, 2H), 2.14 (t, *J* = 7.2 Hz, 2H), 1.58-1.55 (m, 2H), 0.81 (t, *J* = 7.2 Hz, 3H), 0.15 (s, 9H); ^{13}C NMR (101 MHz, $CDCl_3$) δ = 205.3, 138.0, 128.3, 128.0, 126.3, 105.2, 100.4, 31.8, 29.8, 29.2, 22.6, 14.1, -1.1. HRMS (ESI$^+$): Calcd for $C_{23}H_{30}SiNa[M+Na]^+$ 357.2009, Found 357.2010.

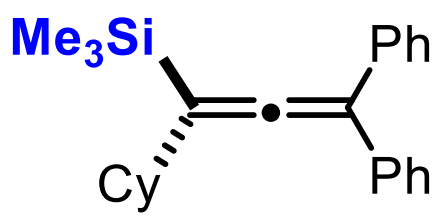

(1-Cyclohexyl-3,3-diphenylpropa-1,2-dien-1-yl)trimethylsilane (7h)

The title compound was prepared according to the general procedure as a colorless oil (58.9 mg, 85% yield). ^{1}H NMR (400 MHz, $CDCl_3$) δ = 7.33-7.29 (m, 8H), 7.22-7.20 (m, 2H), 2.04-2.00 (m, 1H), 1.91-1.89 (m, 2H), 1.75-1.73 (m, 2H), 1.65-1.63 (m, 1H), 1.32-1.25 (m, 4H), 1.20-1.16 (m, 1H), 0.16 (s, 9H); ^{13}C NMR (101 MHz, $CDCl_3$) δ = 205.3, 138.0, 128.4, 127.9, 126.2, 106.3, 105.8, 40.0, 34.5, 26.8, 26.2, -0.5. HRMS (ESI$^+$): Calcd for $C_{24}H_{30}SiNa[M+H]^+$ 369.2009, Found 369.2011.

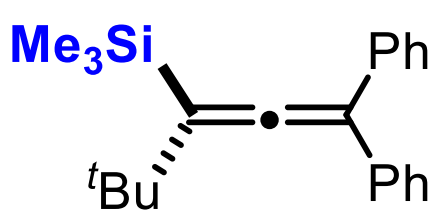

(4,4-Dimethyl-1,1-diphenylpenta-1,2-dien-3-yl)trimethylsilane (7i)

The title compound was prepared according to the general procedure as a colorless oil (49.4 mg, 77% yield). ^{1}H NMR (400 MHz, $CDCl_3$) δ = 7.36-7.27 (m, 8H), 7.25-7.19 (m, 2H), 1.22 (s, 9H), 0.22 (s, 9H); ^{13}C NMR (101 MHz, $CDCl_3$) δ = 205.2, 137.9, 128.4, 127.8, 126.3, 111.0, 105.5, 36.7, 31.6, 1.5. HRMS (ESI$^+$): Calcd for $C_{22}H_{28}SiNa[M+Na]^+$ 343.1853, Found 343.1858.

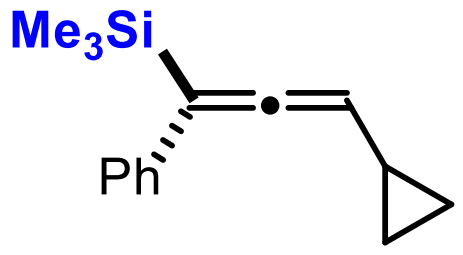

(3-Cyclopropyl-1-phenylpropa-1,2-dien-1-yl)trimethylsilane (7j)

The title compound was prepared according to the general procedure as a colorless oil (41.1 mg, 90% yield). ^{1}H NMR (400 MHz, $CDCl_3$) δ = 7.29-7.28 (m, 4H), 7.19-7.14 (m, 1H), 5.14 (d, *J* = 6.8 Hz, 1H), 1.29-1.25 (m, 1H), 0.70-0.67 (m, 2H), 0.38-0.35 (m, 2H), 0.22 (s, 9H); ^{13}C NMR (101 MHz, $CDCl_3$) δ = 208.4, 138.0, 128.5, 127.7, 126.1, 102.3, 91.4, 8.8, 6.9, 6.6, -0.2. HRMS (ESI$^+$): Calcd for $C_{15}H_{20}SiNa[M+H]^+$ 251.1227, Found 251.1228.

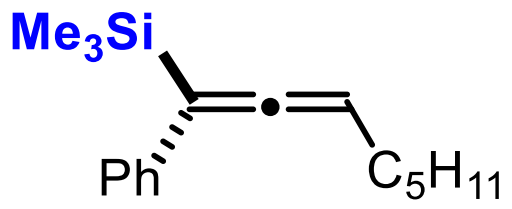

Trimethyl(1-phenylocta-1,2-dien-1-yl)silane (7k)

The title compound was prepared according to the general procedure as a colorless oil (39.8 mg, 90% yield). ^{1}H NMR (400 MHz, $CDCl_3$) δ = 7.34-7.28 (m, 4H), 7.18-7.14 (m, 1H), 5.15 (t, *J* = 6.8 Hz, 1H), 2.07 (q, *J* = 6.8 Hz, 2H), 1.48-1.42 (m, 2H), 1.35-1.26 (m, 4H), 0.88 (t, *J* = 6.8 Hz, 3H) 0.23 (s, 9H); ^{13}C NMR (101 MHz, $CDCl_3$) δ = 208.5, 138.3, 128.4, 127.7, 126.0, 100.2, 87.4, 31.6, 29.4, 28.3, 22.6, 14.2, -0.2. HRMS (ESI$^+$): Calcd for $C_{17}H_{26}SiNa[M+Na]^+$ 281.1696, Found 281.1700.

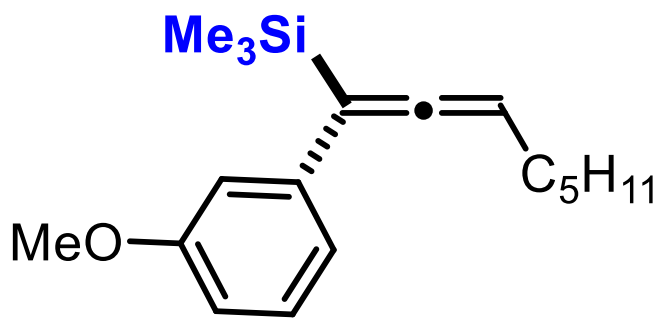

(1-(3-Methoxyphenyl)octa-1,2-dien-1-yl)trimethylsilane (7l)

The title compound was prepared according to the general procedure as a colorless oil (51.4 mg, 89% yield). ^{1}H NMR (400 MHz, $CDCl_3$) δ = 7.20 (t, *J* = 8.0 Hz, 1H), 6.90-6.86 (m, 2H), 6.72 (dd, *J* = 8.4, 2.4 Hz, 1H), 5.15 (t, *J* = 6.8 Hz, 1H), 3.79 (s, 3H), 2.06 (q, *J* = 7.2 Hz, 2H), 1.46-1.43 (m, 2H), 1.36-1.30 (m, 4H), 0.87 (t, *J* = 6.8 Hz, 3H), 0.21 (s, 9H); ^{13}C NMR (101 MHz, $CDCl_3$) δ = 208.5, 159.7, 139.8, 129.3,

120.3, 113.3, 111.5, 110.1, 87.5, 55.2, 31.6, 29.4, 28.3, 22.6, 14.2, -0.1. HRMS (ESI$^+$): Calcd for $C_{18}H_{28}OSiNa[M+H]^+$ 311.1802, Found 311.1800.

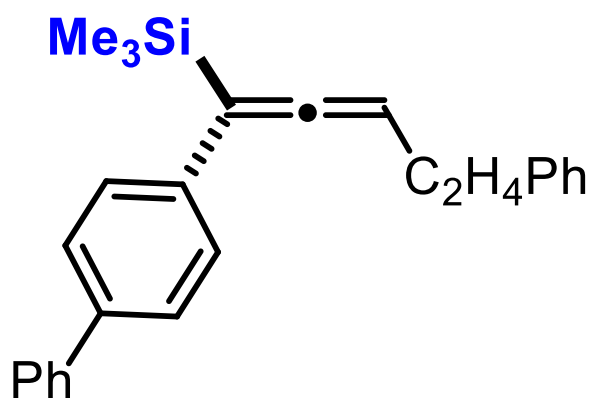

(1-([1,1'-biphenyl]-4-yl)-5-phenylpenta-1,2-dien-1-yl)trimethylsilane (7m)

The title compound was prepared according to the general procedure as a colorless oil (67.8 mg, 92% yield). ^{1}H NMR (400 MHz, $CDCl_3$) δ = 7.59 (d, J = 8.0 Hz, 2H), 7.51 (d, J = 8.2 Hz, 2H), 7.43 (t, J = 7.8 Hz, 2H), 7.33 (t, J = 7.2 Hz, 1H), 7.30–7.27 (m, 4H), 7.23–7.19 (m, 3H), 5.23 (t, J = 6.6 Hz, 1H), 2.84-2.75 (m, 2H), 2.47-2.41 (m, 2H), 0.26 (s, 9H); ^{13}C NMR (101 MHz, $CDCl_3$) δ = 208.5, 141.9, 141.1, 138.9, 137.0, 132.0, 128.8, 128.6, 128.5, 128.1, 127.2, 127.0, 126.0, 100.3, 86.9, 36.1. HRMS (ESI$^+$): Calcd for $C_{26}H_{28}SiNa[M+Na]^+$ 391.1853, Found 391.1860.

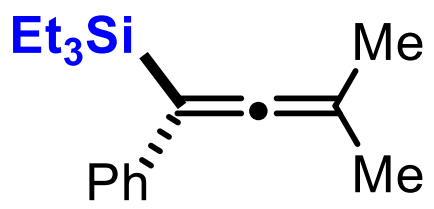

Triethyl(3-methyl-1-phenylbuta-1,2-dien-1-yl)silane (7n)

The title compound was prepared according to the general procedure as a colorless oil (44.5 mg, 86% yield). ^{1}H NMR (400 MHz, $CDCl_3$) δ = 7.28-7.21 (m, 4H), 7.16-7.12 (m, 1H), 1.76 (s, 6H), 0.94 (t, J = 7.6 Hz, 9H), 0.69 (q, J = 7.6 Hz, 6H); ^{13}C NMR (101 MHz, $CDCl_3$) δ = 208.8, 139.7, 128.3, 127.7, 125.7, 95.2, 90.2, 20.0, 7.5, 4.1. HRMS (ESI$^+$): Calcd for $C_{17}H_{26}SiNa[M+Na]^+$ 218.1696, Found 218.1690.

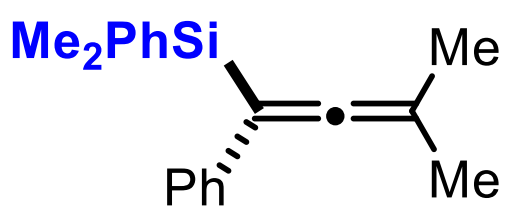

Dimethyl(3-methyl-1-phenylbuta-1,2-dien-1-yl)(phenyl)silane (7o)

The title compound was prepared according to the general procedure as a colorless oil (49.0 mg, 88% yield). ^{1}H NMR (400 MHz, $CDCl_3$) δ = 7.62-7.59 (m, 2H), 7.37-7.35 (m, 3H), 7.22-7.20 (m, 4H), 7.14-7.10 (m, 1H), 1.78 (s, 6H), 0.44 (s, 6H); ^{13}C NMR (101 MHz, $CDCl_3$) δ = 209.3, 139.2, 138.5, 134.0, 129.1, 128.3, 128.0, 127.9, 125.9, 96.9, 92.0, 19.8, -1.6. HRMS (ESI$^+$): Calcd for $C_{19}H_{22}SiNa[M+Na]^+$ 301.1383, Found 301.1388.

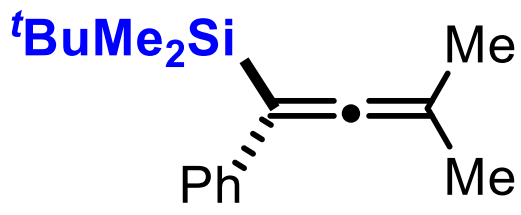

***Tert*-butyldimethyl(3-methyl-1-phenylbuta-1,2-dien-1-yl)silane (7p)**

The title compound was prepared according to the general procedure as a colorless oil (46.0 mg, 89% yield). ^{1}H NMR (400 MHz, $CDCl_3$) δ = 7.29-7.21 (m, 4H), 7.16-7.13 (m, 1H), 1.75 (s, 6H), 0.90 (s, 9H), 0.17 (s, 6H); ^{13}C NMR (101 MHz, $CDCl_3$) δ = 209.2, 140.1, 128.3, 125.7, 96.8, 90.2, 27.0, 19.9, 17.8, -4.2. HRMS (ESI$^+$): Calcd for $C_{17}H_{26}SiNa[M+H]^+$ 281.1696, Found 281.1695.

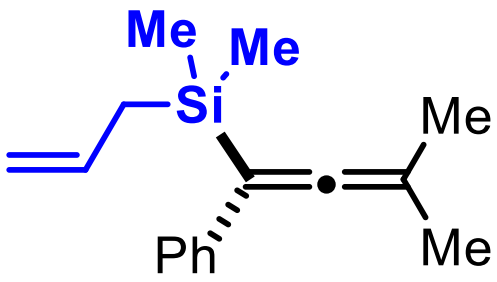

Allyldimethyl(3-methyl-1-phenylbuta-1,2-dien-1-yl)silane (7q)

The title compound was prepared according to the general procedure as a colorless oil (42.7 mg, 88% yield). ^{1}H NMR (400 MHz, $CDCl_3$) δ = 7.30-7.22 (m, 4H), 7.17-7.13 (m, 1H), 5.83-5.72 (m, 1H), 4.87-4.85 (m, 1H), 4.83 (s, 1H), 1.76 (m, 8H), 0.19 (s, 6H); ^{13}C NMR (101 MHz, $CDCl_3$) δ = 208.2, 138.9, 134.9, 128.4, 127.8, 125.9, 113.3, 97.3, 91.4, 24.1, 19.9, -2.3. HRMS (ESI$^+$): Calcd for $C_{16}H_{22}SiNa[M+Na]^+$ 265.1383, Found 265.1385.

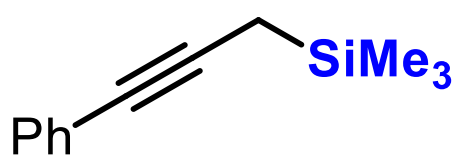

Trimethyl(3-phenylprop-2-yn-1-yl)silane (7r)

The title compound was prepared according to the general procedure as a colorless oil (29.0 mg, 77% yield). ^{1}H NMR (400 MHz, $CDCl_3$) δ = 7.37-7.35 (m, 2H), 7.28-7.22 (m, 3H), 1.69 (s, 2H), 0.16 (s, 9H); ^{13}C NMR (101 MHz, $CDCl_3$) δ = 131.5, 128.2, 127.1, 124.9, 88.6, 8.0, -1.9. HRMS (ESI$^+$): Calcd for $C_{12}H_{16}SiNa[M+H]^+$ 211.0914, Found 211.0915.

General procedure for deoxygenative germanylation and stannylation of ethers

(i) For 2-pyridyl ethers:

A dried Schlenk tube was charged with 2-pyridyl ethers (0.2 mmol), $CrCl_3$ (10 mol%) and chlorotrimethylgermane or chlorotrimethylstannane (1.0 mmol), followed by adding a freshly distilled THF (1.0 mL) by syringe under atmosphere of nitrogen. After stirring at room temperature for 5 min, EtMgBr (1.0 M in THF, 1.0 mmol) was added dropwise, and the reaction mixture was stirred at 70 ^{o}C for 24 h. After cooled to

room temperature, ethyl acetate (20 mL) was added directly and concentrated under vacuum. The residue was purified by flash chromatography on silica gel to give the desired product **3** or **5**.

(ii) For allylic methyl ethers:

A dried Schlenk tube was charged with $CrCl_3$ (10 mol%) and dtbpy (10 mol%), followed by adding a freshly distilled THF (1.0 mL) by syringe under atmosphere of nitrogen. After stirring at room temperature for 5 min, EtMgBr (1.0 M in THF, 1.0 mmol) was added dropwise, and the reaction mixture was stirred at room temperature for 20 min. Then allylic methyl ethers (0.2 mmol) and chlorotrimethylgermane or chlorotrimethylstannane (1.0 mmol) was added into the reaction mixture. The reaction mixture was stirred at 50 ^{o}C for 24 h. After cooled to room temperature, ethyl acetate (20 mL) was added directly and concentrated under vacuum. The residue was purified by flash chromatography on silica gel (petroleum ether) to give the desired product **5**.

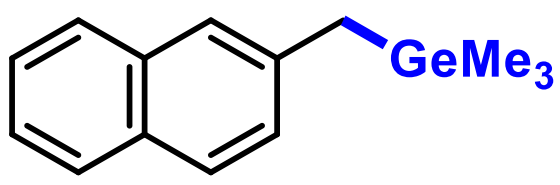

Trimethyl(naphthalen-2-ylmethyl)germane (3aal)

The title compound was prepared according to the general procedure (**i**) as a colorless oil (33.6 mg, 65% yield). ^{1}H NMR (400 MHz, $CDCl_3$) δ = 7.77 (t, *J* = 8.0 Hz, 1H), 7.70 (t, *J* = 8.0 Hz, 2H), 7.41-7.32 (m, 3H), 7.16 (d, *J* = 8.4 Hz, 1H), 2.36 (s, 2H), 0.12 (s, 9H); ^{13}C NMR (151 MHz, $CDCl_3$) δ = 139.3, 133.9, 131.0, 127.6, 127.1, 125.8, 124.6, 124.3, 26.7, -2.3. HRMS (ESI^{+}): Calcd for $C_{14}H_{18}GeNa[M+Na]^{+}$ 283.0513, Found 283.0516.

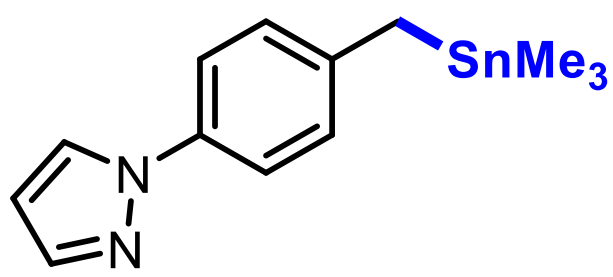

1-(4-((trimethylstannyl)methyl)phenyl)-1*H*-pyrazole (3aam)

The title compound was prepared according to the general procedure (**i**) as a colorless oil (27.0 mg, 42% yield). ^{1}H NMR (400 MHz, $CDCl_3$) δ = 7.84 (d, *J* = 2.0 Hz, 1H), 7.68 (s, 1H), 7.47 (d, *J* = 8.4 Hz, 2H), 7.02 (d, *J* = 8.4 Hz, 2H), 6.42 (s, 1H), 2.34 (s, 2H), 0.05 (s, 9H); ^{13}C NMR (101 MHz, $CDCl_3$) δ = 142.1, 140.6, 127.5, 126.7, 119.7, 107.2, 19.8, -9.9. HRMS (ESI^{+}): Calcd for $C_{13}H_{19}N_2Sn$ $[M+H]^{+}$ 323.0565, Found 323.0575.

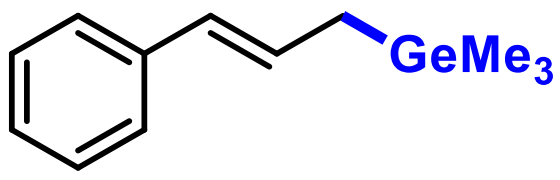

(*E*)-cinnamyltrimethylgermane (5ab)

The title compound was prepared according to the general procedure (**i**) as a colorless oil (19.7 mg, 42% yield). ^{1}H NMR (400 MHz, $CDCl_3$) δ = 7.30-7.28 (m, 4H), 7.15-7.12 (m, 1H), 6.33-6.20 (m, 2H), 1.79 (d, *J* = 7.6 Hz, 2H), 0.17 (s, 9H); ^{13}C NMR (101 MHz, $CDCl_3$) δ = 138.6, 128.9, 128.5, 127.5, 126.2, 125.6, 23.5, -2.3; HRMS (ESI$^+$): Calcd for $C_{12}H_{18}GeNa[M+H]^+$ 259.0513, Found 259.0515.

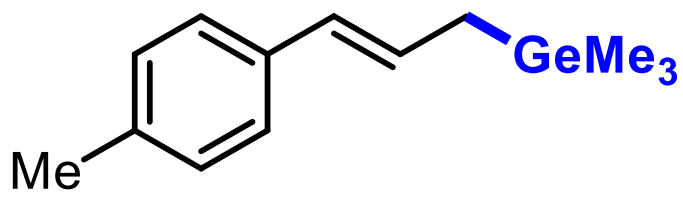

(*E*)-trimethyl(3-(p-tolyl)allyl)germane (5ac)

The title compound was prepared according to the general procedure (**ii**) as a colorless oil (25.0 mg, 50% yield). ^{1}H NMR (400 MHz, $CDCl_3$) δ = 7.21-7.18 (m, 2H), 7.08 (d, *J* = 8.0 Hz, 2H), 6.27-6.16 (m, 2H), 2.30 (s, 3H), 1.77 (d, *J* = 7.2 Hz, 2H), 0.16 (s, 9H); ^{13}C NMR (101 MHz, $CDCl_3$) δ = 135.9, 129.2, 127.8, 127.4, 125.8, 125.5, 23.4, 21.2, -2.4. HRMS (ESI$^+$): Calcd for $C_{13}H_{20}GeNa[M+Na]^+$ 273.0669, Found 273.0668.

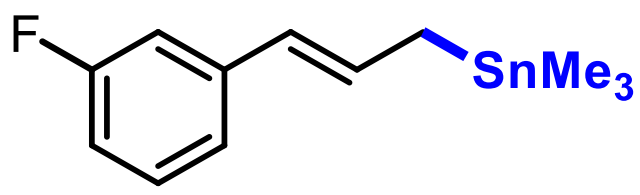

(*E*)-(3-(3-fluorophenyl)allyl)trimethylstannane (5ad)

The title compound was prepared according to the general procedure (**ii**) as a colorless oil (44.8 mg, 75% yield). ^{1}H NMR (400 MHz, $CDCl_3$) δ = 7.22-7.17 (m, 1H), 7.00 (d, *J* = 7.6 Hz, 1H), 6.97 (d, *J* = 10.8 Hz, 1H), 6.80 (t, *J* = 8.0 Hz, 1H), 6.46-6.37 (m, 1H), 6.17 (d, *J* = 15.6 Hz, 1H), 1.93 (d, *J* = 8.8 Hz, 2H), 0.14 (s, 9H); ^{13}C NMR (151 MHz, $CDCl_3$) δ = 164.1 (d, *J* = 244.6 Hz), 141.1, 132.3, 129.9 (d, *J* = 7.7 Hz), 124.3, 121.2, 112.6 (d, *J* = 21.4 Hz), 111.7 (d, *J* = 18.4 Hz), 17.8, -9.8. HRMS (ESI$^+$): Calcd for $C_{12}H_{17}FSnNa[M+Na]^+$ 323.0229, Found 323.0227.

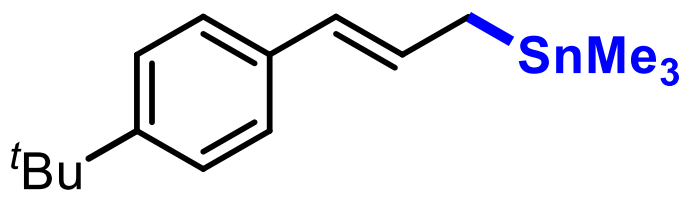

(*E*)-(3-(4-(*tert*-butyl)phenyl)allyl)trimethylstannane (5ae)

The title compound was prepared according to the general procedure as a colorless oil (39.0 mg, 58% yield). ^{1}H NMR (400 MHz, $CDCl_3$) δ = 7.30 (d, *J* = 8.8 Hz, 2H), 7.20

(d, *J* = 8.4 Hz, 1H), 6.39-6.31 (m, 1H), 6.20 (d, *J* = 15.6 Hz, 1H), 1.91 (d, *J* = 8.4 Hz, 2H), 1.30 (s, 9H), 0.12 (s, 9H); ^{13}C NMR (151 MHz, $CDCl_3$) δ = 148.8, 136.0, 129.7, 125.4, 125.11, 125.05, 34.5, 31.4, 17.8, -9.9; HRMS (ESI$^+$): Calcd for $C_{16}H_{26}SnNa[M+Na]^+$ 361.0949, Found 361.0950.

5. General Procedure for the Deoxyborylation of Alcohols and Ethers with HBpin 8b by Chromium

General procedure for the deoxyborylation of 2-pyridyl ethers 1 with HBpin 8b by chromium

A dried Schlenk tube was charged with **1** (0.2 mmol), $CrCl_3$ (10 mol%), Mg (0.4 mmol) and HBpin (0.6 mmol), followed by adding a freshly distilled THF (2 mL) by syringe under atmosphere of nitrogen. The reaction mixture was stirred at 80 °C for 12 h. After cooled room temperature, ethyl acetate (20 mL) was added into the reaction mixture and concentrated under vacuum. The residue was purified by flash chromatography on silica gel to give the desired product **9**.

General procedure for the deoxyborylation of methyl ethers 1' or 4' with HBpin 8b by chromium in the presence of dtbpy

A dried Schlenk tube was charged with methyl ethers **1'** or **4'** (0.2 mmol), $CrCl_3$ (10 mol%), dtbpy (10 mol%), Mg (0.4 mmol) and HBpin (0.6 mmol), followed by adding a freshly distilled THF (2 mL) by syringe under atmosphere of nitrogen. The reaction mixture was stirred at 80 °C for 48 h. After cooled room temperature, ethyl acetate (20 mL) was added into the reaction mixture and concentrated under vacuum. The residue was purified by flash chromatography on silica gel to give the desired product **9**.

General procedure for the deoxyborylation of alcohols 1'' or 4'' with HBpin 8b by chromium in the presence of dtbpy

A dried Schlenk tube was charged with alcohol **1''** or **4''** (0.2 mmol), $CrCl_3$ (10 mol%), dtbpy (10 mol%), Mg (0.6 mmol) and HBpin (0.8 mmol), followed by adding a freshly distilled THF (2 mL) by syringe under atmosphere of nitrogen. The reaction mixture was stirred at 80 °C for 72 h. After cooled room temperature, ethyl acetate (20 mL) was added into the reaction mixture and concentrated under vacuum. The residue was purified by flash chromatography on silica gel to give the desired product **9**.

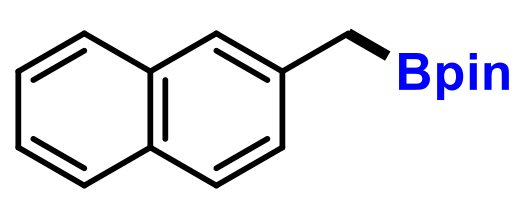

4,4,5,5-Tetramethyl-2-(naphthalen-2-ylmethyl)-1,3,2-dioxaborolane (9a)

The title compound was prepared according to the general procedure as a colorless solid (**A:** 43.4 mg, 81% yield; **B:** 48.8 mg, 91% yield; **Alcohol:** 41.8 mg, 78% yield). The spectroscopic data are consistent with those previously reported in the literature.[5] ^{1}H NMR (400 MHz, $CDCl_3$) δ = 7.81–7.74 (m, 3H), 7.65 (s, 1H), 7.47–7.34 (m, 3H), 2.49 (s, 2H), 1.26 (s, 12H); ^{13}C NMR (101 MHz, $CDCl_3$) δ = 136.5, 134.0, 131.6, 128.4, 127.8, 127.7, 127.4, 126.7, 125.8, 124.8, 24.9. HRMS (ESI$^+$): Calcd for $C_{17}H_{22}BO_2$ [M+H]$^+$ 269.1707, Found 269.1705.

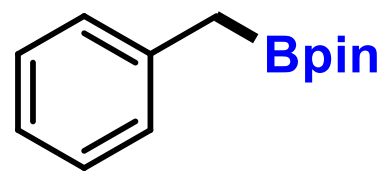

2-Benzyl-4,4,5,5-tetramethyl-1,3,2-dioxaborolane (9b)

The title compound was prepared according to the general procedure as a colorless oil (**A:** 21.8 mg, 50% yield; **B:** 26.2 mg, 60% yield). ^{1}H NMR (400 MHz, $CDCl_3$) δ = 7.23 (d, J = 7.2 Hz, 2H), 7.18 (d, J = 6.7 Hz, 2H), 7.12 (t, J = 7.1 Hz, 1H), 2.30 (s, 2H), 1.23 (s, 12H); ^{13}C NMR (101 MHz, $CDCl_3$) δ = 138.8, 129.1, 128.4, 125.0, 83.6, 24.9. HRMS (ESI$^+$): Calcd for $C_{13}H_{20}BO_2$ [M+H]$^+$ 219.1551, Found 219.1548.

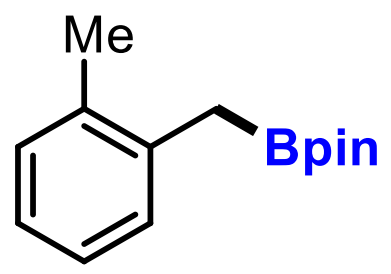

4,4,5,5-Tetramethyl-2-(2-methylbenzyl)-1,3,2-dioxaborolane (9c)

The title compound was prepared according to the general procedure as a colorless oil (**A:** 26.0 mg, 56% yield; **B:** 30.6 mg, 66% yield). ^{1}H NMR (400 MHz, $CDCl_3$) δ = 7.16 (dd, J = 9.2, 2.2 Hz, 1H), 7.13 (d, J = 1.6 Hz, 1H), 7.11-7.05 (m, 2H), 2.30 (s, 3H), 2.29 (s, 2H), 1.25 (s, 12H); ^{13}C NMR (101 MHz, $CDCl_3$) δ = 137.6, 136.0, 129.9, 129.6, 126.0, 125.3, 83.5, 24.8, 20.2. HRMS (ESI$^+$): Calcd for $C_{14}H_{22}BO_2$ [M+H]$^+$ 233.1707, Found 233.1705.

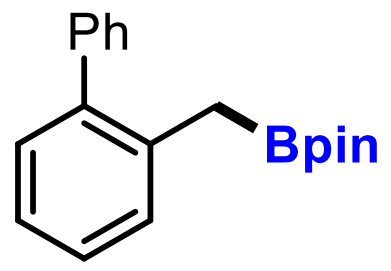

2-([1,1'-Biphenyl]-2-ylmethyl)-4,4,5,5-tetramethyl-1,3,2-dioxaborolane (9d)

The title compound was prepared according to the general procedure as a colorless solid (**A:** 47.1 mg, 80% yield; **B:** 53.0 mg, 90% yield). ^{1}H NMR (400 MHz, $CDCl_3$) δ = 7.42-7.36 (m, 5H), 7.28 (d, J = 3.6 Hz, 2H), 7.23-7.20 (m, 2H), 2.30 (s, 2H), 1.17 (s, 12H); ^{13}C NMR (101 MHz, $CDCl_3$) δ = 142.4, 141.8, 136.7, 130.3, 130.1, 129.6,

128.1, 127.4, 126.8, 125.3, 83.4, 24.8. HRMS (ESI$^+$): Calcd for $C_{19}H_{23}BO_2$ $[M+H]^+$ 295.1864, Found 295.1864.

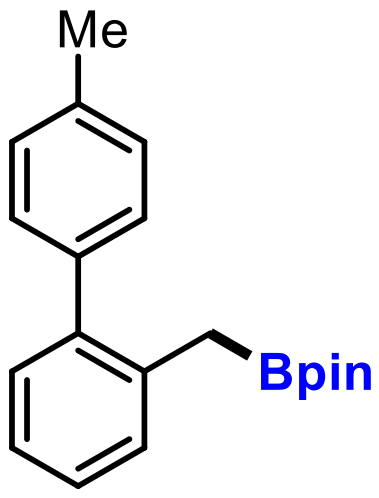

4,4,5,5-Tetramethyl-2-((4'-methyl-[1,1'-biphenyl]-2-yl)methyl)-1,3,2-dioxaborolane (9e)

The title compound was prepared according to the general procedure as a colorless solid (**A:** 37.0 mg, 60% yield; **B:** 43.8 mg, 71% yield). ^{1}H NMR (400 MHz, $CDCl_3$) δ = 7.16-7.13 (m, 4H), 7.11-7.07 (m, 4H), 2.28 (s, 3H), 2.18 (s, 2H), 1.05 (s, 12H); ^{13}C NMR (101 MHz, $CDCl_3$) δ = 141.7, 139.4, 136.7, 136.4, 130.2, 130.1, 129.5, 128.8, 127.2, 125.2, 83.4, 24.8, 21.3. HRMS (ESI$^+$): Calcd for $C_{20}H_{25}NaBO_2$ $[M+Na]^+$ 331.1840, Found 331.1837.

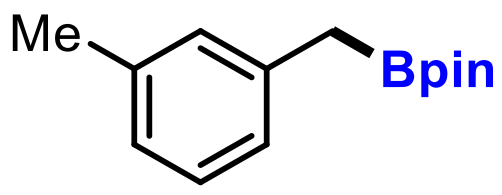

4,4,5,5-Tetramethyl-2-(3-methylbenzyl)-1,3,2-dioxaborolane (9f)

The title compound was prepared according to the general procedure as a colorless oil (**A:** 24.6 mg, 53% yield; **B:** 29.2 mg, 63% yield). ^{1}H NMR (400 MHz, $CDCl_3$) δ = 7.13 (t, J = 7.5 Hz, 1H), 6.99 (d, J = 8.0 Hz, 2H), 6.94 (d, J = 7.5 Hz, 1H), 2.31 (s, 3H), 2.26 (s, 2H), 1.24 (s, 12H); ^{13}C NMR (101 MHz, $CDCl_3$) δ = 138.6, 137.8, 130.0, 128.3, 126.1, 125.7, 83.5, 24.8, 21.5. HRMS (ESI$^+$): Calcd for $C_{14}H_{22}BO_2$ $[M+H]^+$ 233.1707, Found 233.1706.

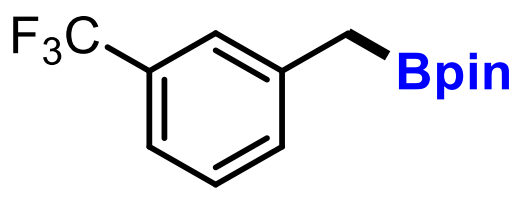

4,4,5,5-Tetramethyl-2-(3-(trifluoromethyl)benzyl)-1,3,2-dioxaborolane (9g)

The title compound was prepared according to the general procedure as a colorless oil (**A:** 20.6 mg, 36% yield; **B:** 28.6 mg, 50% yield). ^{1}H NMR (400 MHz, $CDCl_3$) δ = 7.12 (dd, J = 8.6, 5.5 Hz, 2H), 6.92 (t, J = 8.8 Hz, 2H), 2.25 (s, 2H), 1.23 (s, 12H); ^{13}C NMR (101 MHz, $CDCl_3$) δ = 162.1, 159.7, 134.2, 130.3, 115.1, 83.6, 24.9; ^{19}F NMR

(376 MHz, $CDCl_3$) δ = -119.36. HRMS (ESI$^+$): Calcd for $C_{14}H_{19}F_3BO_2$ $[M+H]^+$ 287.1425, Found 287.1429.

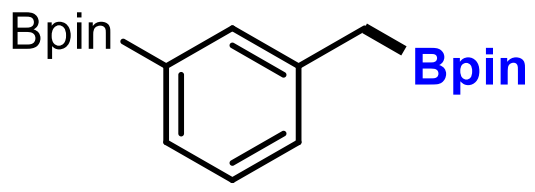

4,4,5,5-Tetramethyl-2-(3-(4,4,5,5-tetramethyl-1,3,2-dioxaborolan-2-yl)benzyl)-1,3,2-dioxaborolane (9h)

The title compound was prepared according to the general procedure as a colorless solid (**A:** 35.8 mg, 52% yield; **B:** 41.3 mg, 60% yield). ^{1}H NMR (400 MHz, $CDCl_3$) δ = 7.61 (s, 1H), 7.58 (d, J = 7.1 Hz, 1H), 7.30 (dd, J = 5.5, 3.8 Hz, 1H), 7.25 (t, J = 7.4 Hz, 1H), 2.30 (s, 2H), 1.34 (s, 12H), 1.23 (s, 12H); ^{13}C NMR (101 MHz, $CDCl_3$) δ = 138.0, 135.5, 132.0, 131.2, 127.8, 83.7, 83.5, 25.0, 24.8. HRMS (ESI$^+$): Calcd for $C_{19}H_{31}B_2O_4$ $[M+H]^+$ 345.2403, Found: 345.2415.

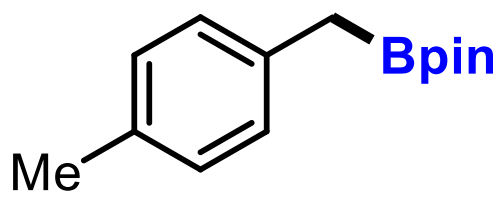

4,4,5,5-Tetramethyl-2-(4-methylbenzyl)-1,3,2-dioxaborolane (9i)

The title compound was prepared according to the general procedure as a colorless solid (**A:** 28.8 mg, 62% yield; **B:** 33.9 mg, 73% yield). ^{1}H NMR (400 MHz, $CDCl_3$) δ = 7.07 (m, 4H), 2.30 (s, 3H), 2.26 (s, 2H), 1.24 (s, 12H); ^{13}C NMR (101 MHz, $CDCl_3$) δ = 135.5, 134.2, 129.1, 129.0, 83.5, 24.9, 21.1. HRMS (ESI$^+$): Calcd for $C_{14}H_{22}BO_2$ $[M+H]^+$ 233.1707, Found 233.1703.

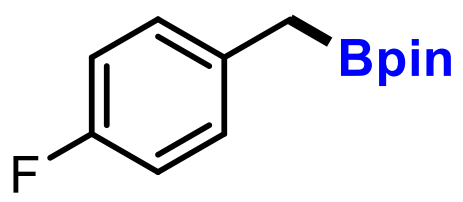

2-(4-Fluorobenzyl)-4,4,5,5-tetramethyl-1,3,2-dioxaborolane (9j)

The title compound was prepared according to the general procedure as a colorless solid (**A:** 18.9 mg, 40% yield; **B:** 26.9 mg, 57% yield). ^{1}H NMR (400 MHz, $CDCl_3$) δ = 7.44 (s, 1H), 7.40-7.33 (m, 3H), 2.35 (s, 2H), 1.23 (s, 12H); ^{13}C NMR (101 MHz, $CDCl_3$) δ = 139.8, 132.5, 128.7, 125.81(d, J_{C-F} = 4 Hz),121.91 (d, J_{C-F} = 4 Hz), 83.8, 24.8. ^{19}F NMR (376 MHz, $CDCl_3$) δ = -62.59. HRMS (ESI$^+$): Calcd for $C_{13}H_{18}BNaO_2$ $[M+Na]^+$ 259.1276, Found: 259.1275.

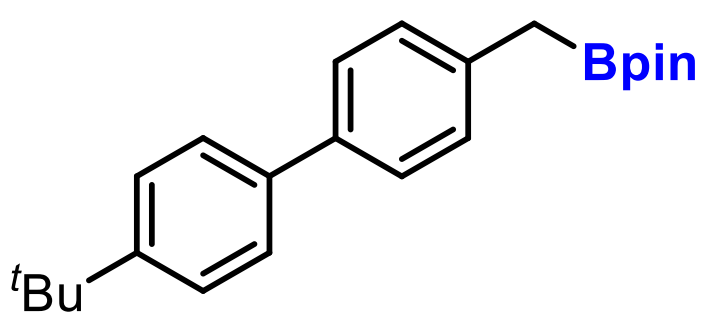

2-((4'-(*Tert*-butyl)-[1,1'-biphenyl]-4-yl)methyl)-4,4,5,5-tetramethyl-1,3,2-dioxaborolane (9k)

The title compound was prepared according to the general procedure as a colorless solid (**A:** 58.2 mg, 83% yield; **B:** 63.1 mg, 90% yield; **C:** 50.0 mg, 80% yield). ^{1}H NMR (400 MHz, $CDCl_3$) δ = 7.42 (d, *J* = 8.4 Hz, 2H), 7.37 (d, *J* = 8.1 Hz, 2H), 7.33 (d, *J* = 8.4 Hz, 2H), 7.14 (d, *J* = 8.1 Hz, 2H), 2.23 (s, 2H), 1.25 (s, 9H), 1.14 (s, 12H); ^{13}C NMR (101 MHz, $CDCl_3$) δ = 149.8, 138.4, 137.7, 137.6, 129.5, 127.0, 126.6, 125.7, 83.6, 34.6, 31.5, 24.8. HRMS (ESI$^+$): Calcd for $C_{23}H_{32}BO_2$ $[M+H]^+$ 351.2490, Found 351.2493.

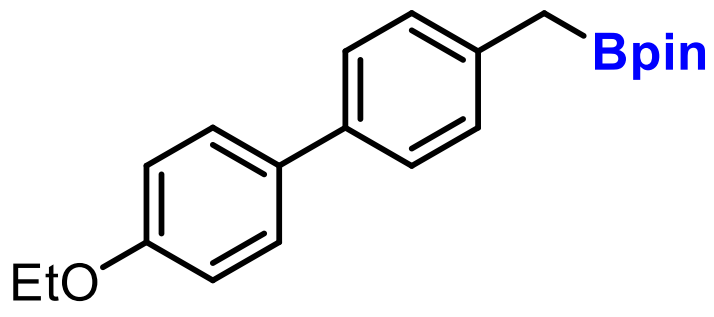

2-((4'-Ethoxy-[1,1'-biphenyl]-4-yl)methyl)-4,4,5,5-tetramethyl-1,3,2-dioxaborolane (9l)

The title compound was prepared according to the general procedure as a colorless solid (**A:** 55.5 mg, 82% yield; **B:** 61.6 mg, 91% yield; **C:** 50.7 mg, 75% yield). ^{1}H NMR (400 MHz, $CDCl_3$) δ = 7.48 (d, *J* = 8.8 Hz, 2H), 7.42 (d, *J* = 8.2 Hz, 2H), 7.22 (d, *J* = 8.2 Hz, 2H), 6.92 (d, *J* = 8.8 Hz, 2H), 4.01 (t, *J* = 7.0 Hz, 2H), 2.32 (s, 2H), 1.39 (d, *J* = 7.0 Hz, 3H), 1.23 (s, 12H); ^{13}C NMR (101 MHz, $CDCl_3$) δ = 158.2, 137.5, 137.1, 133.7, 129.4, 127.9, 126.6, 114.7, 83.5, 63.5, 24.8, 14.9. HRMS (ESI$^+$): Calcd for $C_{21}H_{28}BO_3$ $[M+H]^+$ 339.2126, Found 339.2121.

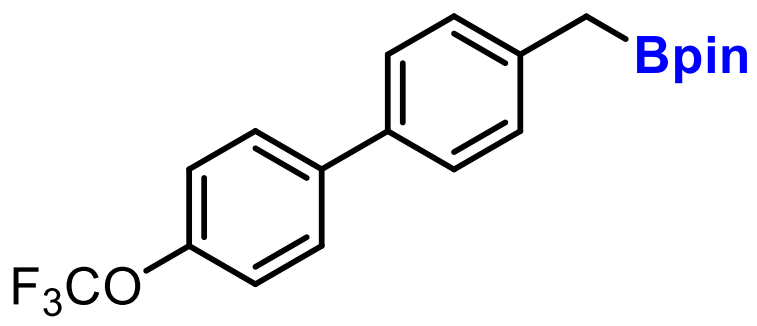

4,4,5,5-Tetramethyl-2-((4'-(trifluoromethoxy)-[1,1'-biphenyl]-4-yl)methyl)-1,3,2-dioxaborolane (9m)

The title compound was prepared according to the general procedure as a colorless solid (**A:** 56.0 mg, 74% yield; **B:** 64.3 mg, 85% yield). ^{1}H NMR (400 MHz, $CDCl_3$) δ = 7.57 (d, *J* = 8.8 Hz, 2H), 7.44 (d, *J* = 8.2 Hz, 2H), 7.27 (d, *J* = 1.7 Hz, 2H), 7.25 (d, *J* = 2.3 Hz, 2H), 2.34 (s, 2H), 1.25 (s, 12H); ^{13}C NMR (101 MHz, $CDCl_3$) δ = 140.2, 138.5, 136.5, 129.7, 128.3, 127.1, 121.3, 83.7, 29.9, 24.9; ^{19}F NMR (376 MHz, $CDCl_3$) δ = -57.81. HRMS (ESI$^+$): Calcd for $C_{20}H_{23}BF_3O_3$ $[M+H]^+$ 379.1687, Found 379.1686.

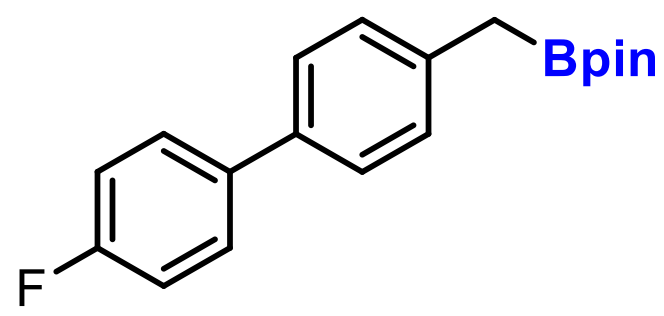

2-((4'-Fluoro-[1,1'-biphenyl]-4-yl)methyl)-4,4,5,5-tetramethyl-1,3,2-dioxaborolane (9n)

The title compound was prepared according to the general procedure as a colorless solid (**A:** 57.4 mg, 92% yield; **B:** 57.4 mg, 92% yield; **C:** 50.0 mg, 80% yield). ^{1}H NMR (400 MHz, $CDCl_3$) δ = 7.40 (dd, *J* = 8.7, 5.4 Hz, 2H), 7.31 (d, *J* = 8.1 Hz, 2H), 7.15 (d, *J* = 8.1 Hz, 2H), 6.98 (t, *J* = 8.7 Hz, 2H), 2.24 (s, 2H), 1.15 (s, 12H); ^{13}C NMR (101 MHz, $CDCl_3$) δ = 162.3 (d, J_{C-F} = 244 Hz), 137.43 (d, J_{C-F} = 111 Hz), 137.45 (d, J_{C-F} = 3 Hz), 129.6, 128.5, 128.4, 127.0 115.58 (d, J_{C-F} = 21 Hz), 83.6, 24.8; ^{19}F NMR (376 MHz, $CDCl_3$) δ = -116.53. HRMS (ESI$^+$): Calcd for $C_{19}H_{23}BFO_2$ $[M+H]^+$ 313.1770, Found 313.1770.

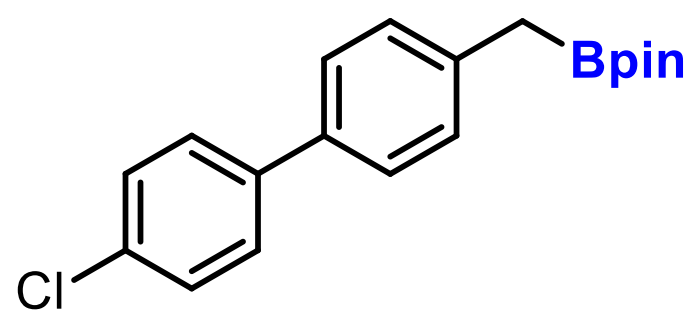

2-((4'-Chloro-[1,1'-biphenyl]-4-yl)methyl)-4,4,5,5-tetramethyl-1,3,2-dioxaborolane (9o)

The title compound was prepared according to the general procedure as a colorless solid (**A:** 60.5 mg, 73% yield; **B:** 52.6 mg, 80% yield; **C:** 46.0 mg, 70% yield). ^{1}H NMR (400 MHz, $CDCl_3$) δ = 7.39 (d, *J* = 8.5 Hz, 2H), 7.33 (d, *J* = 8.1 Hz, 2H), 7.26 (d, *J* = 8.4 Hz, 2H), 7.16 (d, *J* = 8.0 Hz, 2H), 2.24 (s, 2H), 1.15 (s, 12H); ^{13}C NMR (101 MHz, $CDCl_3$) δ = 139.8, 138.4, 136.6, 132.9, 129.6, 128.9, 128.2, 126.9, 83.6, 24.8. HRMS (ESI$^+$): Calcd for $C_{19}H_{23}BClO_2$ $[M+H]^+$ 329.1474, Found 329.1471.

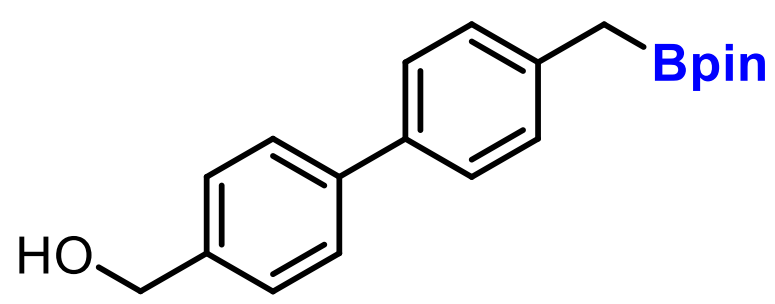

(4'-((4,4,5,5-Tetramethyl-1,3,2-dioxaborolan-2-yl)methyl)-[1,1'-biphenyl]-4-yl)methanol (9p)

The title compound was prepared according to the general procedure as a colorless solid (**A:** 45.4 mg, 70% yield; **B:** 51.9 mg, 80% yield). ^{1}H NMR (400 MHz, $CDCl_3$) δ = 7.55 (d, *J* = 8.2 Hz, 2H), 7.46 (d, *J* = 8.2 Hz, 2H), 7.38 (d, *J* = 8.2 Hz, 2H), 7.24 (d, *J* = 8.1 Hz, 2H), 4.67 (s, 2H), 2.33 (s, 2H), 1.24 (s, 12H); ^{13}C NMR (101 MHz, $CDCl_3$)

δ = 140.7, 139.6, 138.0, 137.4, 129.5, 127.5, 127.1, 127.0, 83.6, 65.1, 24.8. HRMS (ESI$^+$): Calcd for $C_{20}H_{25}BNaO_3$ [M+Na]$^+$ 347.1789, Found 347.1783.

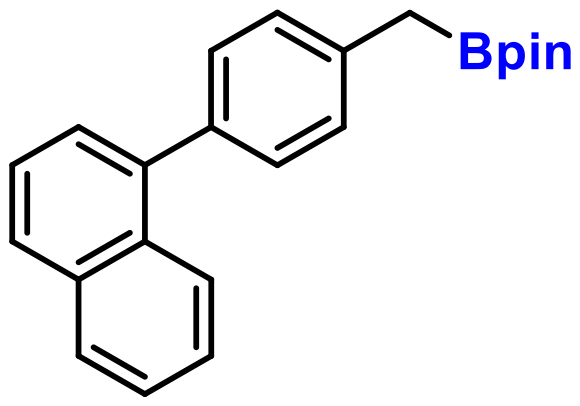

4,4,5,5-Tetramethyl-2-(4-(naphthalen-1-yl)benzyl)-1,3,2-dioxaborolane (9q)

The title compound was prepared according to the general procedure as a colorless solid (**A:** 55.1 mg, 80% yield; **B:** 60.6 mg, 88% yield). ^{1}H NMR (400 MHz, $CDCl_3$) δ = 7.95 (d, *J* = 8.4 Hz, 1H), 7.87 (d, *J* = 7.6 Hz, 1H), 7.81 (d, *J* = 8.2 Hz, 1H), 7.48 (dd, *J* = 10.3, 4.9 Hz, 2H), 7.39 (dd, *J* = 13.8, 7.4 Hz, 4H), 7.31 (d, *J* = 7.9 Hz, 2H), 2.40 (s, 2H), 1.27 (s, 12H); ^{13}C NMR (101 MHz, $CDCl_3$) δ = 140.4, 137.7, 137.3, 133.9, 131.8, 130.1, 129,0, 128.3, 127.4, 127.0, 126.3, 125.9, 125.7, 125.5, 83.6, 24.8. HRMS (ESI$^+$): Calcd for $C_{23}H_{25}BNaO_2$ [M+Na]$^+$ 367.1840, Found 367.1845.

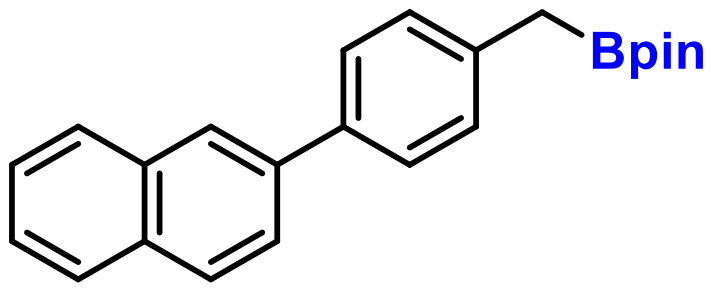

4,4,5,5-Tetramethyl-2-(4-(naphthalen-2-yl)benzyl)-1,3,2-dioxaborolane (9r)

The title compound was prepared according to the general procedure as a colorless solid (**A:** 56.5 mg, 82% yield; **B:** 63.3 mg, 92% yield; **C:** 53.7 mg, 78% yield). ^{1}H NMR (400 MHz, CDCl3) δ = 7.88 (s, 1H), 7.72 (dd, *J* = 10.1, 5.2 Hz, 3H), 7.60 (dd, *J* = 8.5, 1.7 Hz, 1H), 7.48 (d, *J* = 8.1 Hz, 2H), 7.35-7.29 (m, 2H), 7.19 (d, *J* = 8.1 Hz, 2H), 2.26 (s, 2H), 1.12 (s, 12H); ^{13}C NMR (101 MHz, $CDCl_3$) δ = 138.6, 138.1, 137.7, 133.8, 132.5, 129.6, 128.4, 128.2, 127.7, 127.4, 126.2, 125.7, 125.6, 125.4, 83.6, 24.8. HRMS (ESI$^+$): Calcd for $C_{23}H_{26}BO_2$ [M+H]$^+$ 345.2020, Found 345.2017.

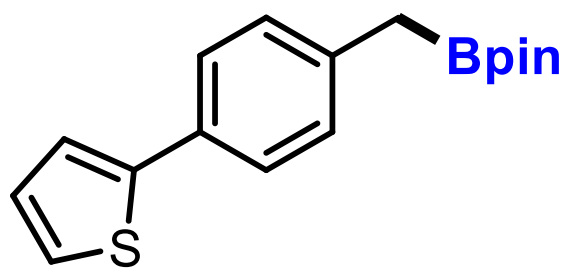

4,4,5,5-Tetramethyl-2-(4-(thiophen-2-yl)benzyl)-1,3,2-dioxaborolane (9s)

The title compound was prepared according to the general procedure as a pale yellow solid (**A:** 42.0 mg, 70% yield; **B:** 49.8 mg, 83% yield; **C:** 36.0 mg, 60% yield). ^{1}H NMR (400 MHz, $CDCl_3$) δ = 7.50 (d, *J* = 8.2 Hz, 2H), 7.40 (dd, *J* = 2.7, 1.6 Hz, 1H), 7.37 (dd, *J* = 3.7, 2.1 Hz, 2H), 7.23 (d, *J* = 8.2 Hz, 2H), 2.33 (s, 2H), 1.26 (s, 12H);

^{13}C NMR (101 MHz, $CDCl_3$) δ = 142.6, 137.8, 132.7, 129.5, 126.5, 126.4, 126.1, 119.5, 83.6, 24.8. HRMS (ESI^+): Calcd for $C_{17}H_{21}BNaSO_2$ $[M+Na]^+$ 323.1248, Found 323.1256.

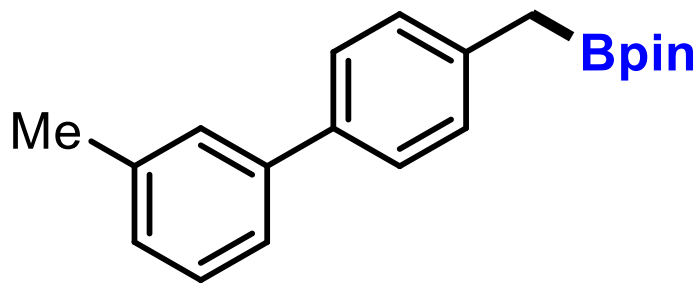

4,4,5,5-Tetramethyl-2-((3'-methyl-[1,1'-biphenyl]-4-yl)methyl)-1,3,2-dioxaborolane (9t)

The title compound was prepared according to the general procedure as a colorless solid (**A:** 56.1 mg, 91% yield; **B:** 55.5 mg, 90% yield; **C:** 49.3 mg, 80% yield). ^1H NMR (400 MHz, $CDCl_3$) δ = 7.47 (d, J = 8.2 Hz, 2H), 7.38 (d, J = 9.0 Hz, 2H), 7.30 (t, J = 7.5 Hz, 1H), 7.24 (d, J = 8.7 Hz, 2H), 7.12 (d, J = 7.5 Hz, 1H), 2.40 (s, 3H), 2.33 (s, 2H), 1.25 (s, 12H); ^{13}C NMR (101 MHz, $CDCl_3$) δ = 141.4, 138.3, 138.0, 137.8, 129.5, 128.7, 127.9, 127.7, 127.2, 124.2, 83.6, 24.9, 21.7. HRMS (ESI^+): Calcd for $C_{20}H_{26}BO_2$ $[M+H]^+$ 309.2020, Found 309.2022.

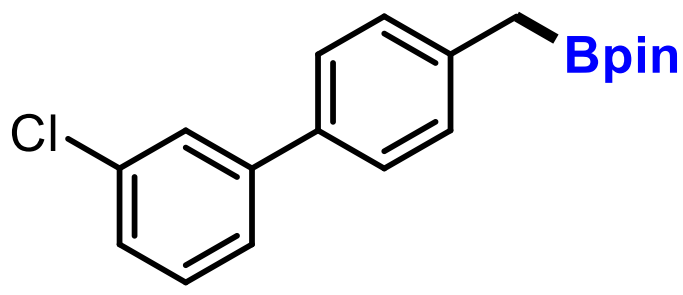

2-((3'-Chloro-[1,1'-biphenyl]-4-yl)methyl)-4,4,5,5-tetramethyl-1,3,2-dioxaborolane (9u)

The title compound was prepared according to the general procedure as a colorless solid (**A:** 40.8 mg, 62% yield; **B:** 46.0 mg, 70% yield; **C:** 39.4 mg, 60% yield). ^1H NMR (400 MHz, $CDCl_3$) δ = 7.56 (s, 1H), 7.45 (d, J = 8.0 Hz, 3H), 7.33 (t, J = 7.8 Hz, 1H), 7.29-7.24 (m, 3H), 2.34 (s, 2H), 1.25 (s, 12H); ^{13}C NMR (101 MHz, $CDCl_3$) δ = 143.2, 138.8, 136.5, 134.7, 130.0, 129.7, 127.2, 127.1, 126.9, 125.2, 83.7, 29.9, 24.9. HRMS (ESI^+): Calcd for $C_{19}H_{23}BClO_2$ $[M+H]^+$ 329.1474, Found 329.1477.

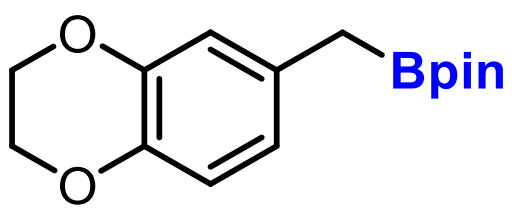

2-((2,3-Dihydrobenzo[*b*][1,4]dioxin-6-yl)methyl)-4,4,5,5-tetramethyl-1,3,2-dioxaborolane (9v)

The title compound was prepared according to the general procedure as a colorless oil (**A:** 26.5 mg, 48% yield; **B:** 27.6 mg, 50% yield). ^1H NMR (400 MHz, $CDCl_3$) δ = 6.71 (dd, J = 13.2, 5.1 Hz, 2H), 6.63 (dd, J = 8.2, 2.1 Hz, 1H), 4.23– 4.19 (m, 4H),

2.18 (s, 2H), 1.23 (s, 12H); ^{13}C NMR (101 MHz, $CDCl_3$) δ = 143.3, 141.1, 131.8, 122.1, 117.7, 117.0, 83.5, 64.5, 64.4, 24.8. HRMS (ESI^+): Calcd for $C_{15}H_{22}BO_4$ $[M+H]^+$ 277.1606, Found 277.1603.

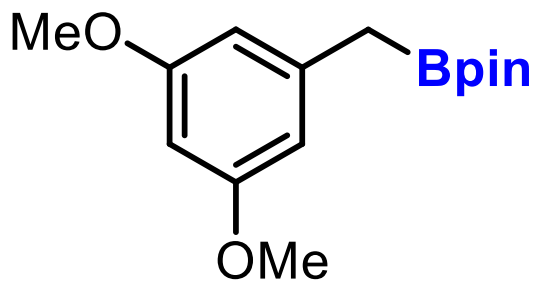

2-(3,5-Dimethoxybenzyl)-4,4,5,5-tetramethyl-1,3,2-dioxaborolane (9w)

The title compound was prepared according to the general procedure as a colorless oil (**A:** 30.0 mg, 54% yield; **B:** 36.2 mg, 65% yield). ^{1}H NMR (400 MHz, $CDCl_3$) δ = 6.35 (d, *J* = 2.3 Hz, 2H), 6.25 (t, *J* = 2.3 Hz, 1H), 3.75 (s, 6H), 2.24 (s, 2H), 1.23 (s, 12H); ^{13}C NMR (101 MHz, $CDCl_3$) δ = 160.7, 141.0, 107.2, 97.4, 83.6, 55.3, 24.8. HRMS (ESI^+): Calcd for $C_{15}H_{24}BO_4$ $[M+H]^+$ 279.1762, Found 279.1762.

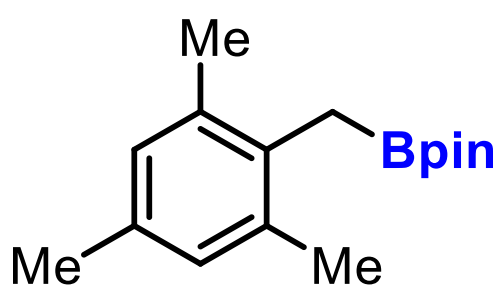

4,4,5,5-Tetramethyl-2-(2,4,6-trimethylbenzyl)-1,3,2-dioxaborolane (9x)

The title compound was prepared according to the general procedure as a colorless oil (**A:** 36.9 mg, 71% yield; **B:** 36.4 mg, 70% yield). ^{1}H NMR (400 MHz, $CDCl_3$) δ = 6.87 (s, 2H), 2.29 (s, 9H), 2.26 (s, 2H), 1.26 (s, 12H); ^{13}C NMR (101 MHz, $CDCl_3$) δ = 135.6, 133.7, 133.3, 128.5, 83.3, 24.8, 20.9, 20.8. HRMS (ESI^+): Calcd for $C_{16}H_{26}BO_2$ $[M+H]^+$ 261.2020, Found 261.2009.

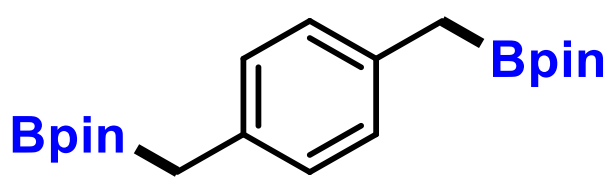

1,4-Bis((4,4,5,5-tetramethyl-1,3,2-dioxaborolan-2-yl)methyl)benzene (9y)

The title compound was prepared according to the general procedure as a colorless solid (**A:** 28.6 mg, 40% yield; **B:** 32.2 mg, 45% yield). ^{1}H NMR (400 MHz, $CDCl_3$) δ = 7.05 (s, 4H), 2.23 (s, 4H), 1.22 (s, 24H); ^{13}C NMR (101 MHz, $CDCl_3$) δ = 134.9, 129.1, 83.5, 24.8. HRMS (ESI^+): Calcd for $C_{20}H_{33}B_2O_4$ $[M+H]^+$ 359.2559, Found 359.2555.

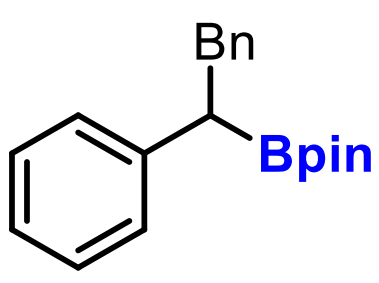

2-(1,2-Diphenylethyl)-4,4,5,5-tetramethyl-1,3,2-dioxaborolane (9z)

The title compound was prepared according to the general procedure as a colorless solid (**A:** 38.8 mg, 63% yield; **B:** 41.9 mg, 68% yield). ^{1}H NMR (400 MHz, $CDCl_3$) δ = 7.16 (d, *J* = 4.3 Hz, 4H), 7.14-7.08 (m, 4H), 7.07-7.03 (m, 2H), 3.07 (dd, *J* = 13.5, 9.8 Hz, 1H), 2.88 (dd, *J* = 13.5, 6.9 Hz, 1H), 2.60 (dd, *J* = 9.8, 6.9 Hz, 1H), 1.02 (d, *J* = 3.6 Hz, 12H); ^{13}C NMR (101 MHz, $CDCl_3$) δ = 142.7, 141.8, 129.0, 128.5, 128.4, 128.1, 125.9, 125.5, 83.5, 39.0, 24.7, 24.6. HRMS (ESI$^+$): Calcd for $C_{20}H_{26}BO_2$ $[M+H]^+$ 309.2020, Found 309.2019.

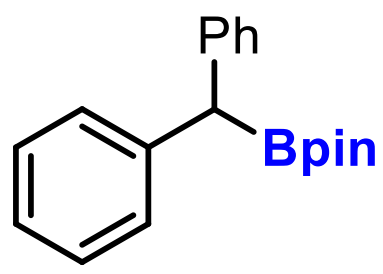

2-Benzhydryl-4,4,5,5-tetramethyl-1,3,2-dioxaborolane (9aa)

The title compound was prepared according to the general procedure as a colorless solid (**A:** 37.7 mg, 64% yield; **B:** 41.1 mg, 70% yield). ^{1}H NMR (400 MHz, $CDCl_3$) δ = 7.18-7.15 (m, 8H), 7.06 (td, *J* = 5.6, 2.8 Hz, 2H), 3.78 (s, 1H), 1.13 (s, 12H); ^{13}C NMR (101 MHz, $CDCl_3$) δ = 142.2, 129.2, 128.5, 125.7, 83.8, 24.7. HRMS (ESI$^+$): Calcd for $C_{19}H_{23}BO_2$ $[M+H]^+$ 295.1864, Found 295.1862.

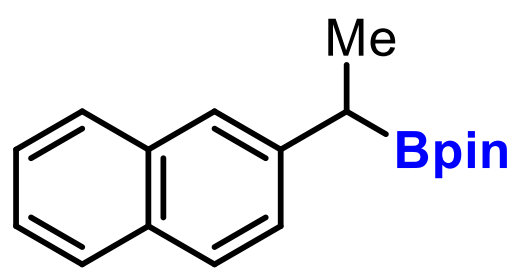

4,4,5,5-Tetramethyl-2-(1-(naphthalen-2-yl)ethyl)-1,3,2-dioxaborolane (9ab)

The title compound was prepared according to the general procedure as a colorless solid (**A:** 49.7 mg, 88% yield; **B:** 50.8 mg, 90% yield). ^{1}H NMR (400 MHz, $CDCl_3$) δ = 7.79-7.74 (m, 3H), 7.65 (s, 1H), 7.44-7.36 (m, 3H), 2.62 (q, *J* = 7.6 Hz, 1H), 1.44 (d, *J* = 7.6 Hz, 3H), 1.22 (d, *J* = 6.0 Hz, 12H); ^{13}C NMR (101 MHz, $CDCl_3$) δ = 142.7, 133.9, 131.8, 127.8, 127.61, 127.56, 127.3, 125.7, 125.4, 124.9, 83.5, 24.7, 16.9; ^{11}B NMR (193 MHz, $CDCl_3$) δ = 33.1. HRMS (ESI$^+$): Calcd for $C_{18}H_{24}BO_2$ $[M+H]^+$ 283.1864, Found 283.1860.

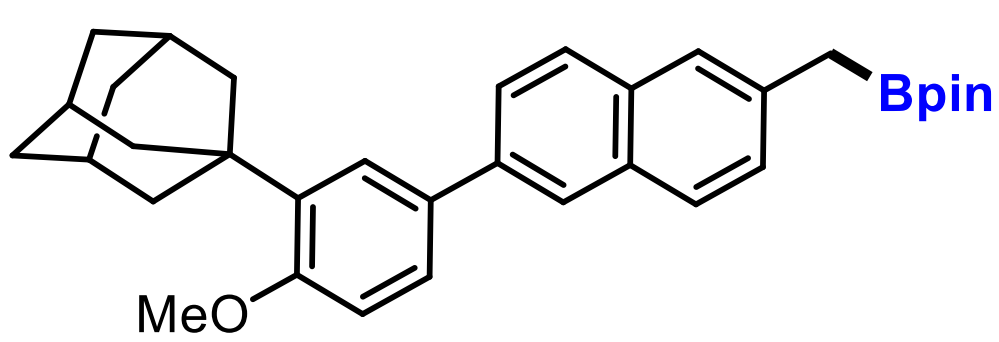

2-((6-(3-(Adamantan-1-yl)-4-methoxyphenyl)naphthalen-2-yl)methyl)-4,4,5,5-tetramethyl-1,3,2-dioxaborolane (9ac)

The title compound was prepared according to the general procedure as a colorless solid (**A:** 55.9 mg, 55% yield). ^{1}H NMR (400 MHz, $CDCl_3$) δ = 7.93 (s, 1H), 7.78 (dd, *J* = 8.4, 4.3 Hz, 2H), 7.68 (dd, *J* = 8.5, 1.8 Hz, 1H), 7.63 (s, 1H), 7.58 (d, *J* = 2.3 Hz, 1H), 7.52 (dd, *J* = 8.4, 2.3 Hz, 1H), 7.35 (dd, *J* = 8.4, 1.6 Hz, 1H), 6.98 (d, *J* = 8.4 Hz, 1H), 3.90 (s, 3H), 2.47 (s, 2H), 2.19 (d, *J* = 2.5 Hz, 6H), 2.11 (s, 3H), 1.81 (s, 6H), 1.25 (s, 12H); ^{13}C NMR (101 MHz, $CDCl_3$) δ = 158.5, 138.9, 138.0, 136.2, 133.6, 132.8, 131.9, 128.7, 128.0, 127.7, 126.5, 126.0, 125.7, 125.6, 124.9, 112.2, 83.6, 55.3, 40.8, 37.3, 29.3, 24.9. HRMS (ESI$^+$): Calcd for $C_{34}H_{42}BO_3$ [M+H]$^+$ 509.3222, Found: 509.3219.

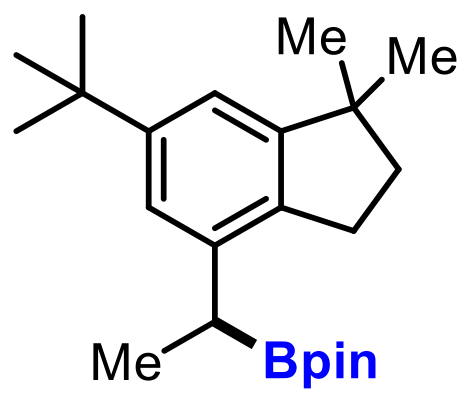

2-(1-(6-(*Tert*-butyl)-1,1-dimethyl-2,3-dihydro-1H-inden-4-yl)ethyl)-4,4,5,5-tetramethyl-1,3,2-dioxaborolane (9ad)

The title compound was prepared according to the general procedure as a colorless oil (**A:** 44.9 mg, 63% yield; **B:** 46.3 mg, 65% yield). ^{1}H NMR (400 MHz, $CDCl_3$) δ = 7.07 (s, 1H), 6.95 (s, 1H), 2.87-2.76 (m, 2H), 2.46 (q, *J* = 7.2 Hz, 1H), 1.89 (t, *J* = 7.2 Hz, 3H), 1.31 (s, 12H), 1.27-1.22 (m, 18H); ^{13}C NMR (101 MHz, $CDCl_3$) δ = 152.0, 149.6, 140.0, 138.3, 122.5, 115.8, 83.2, 44.3, 41.4, 34.8, 31.8, 29.0, 28.9, 28.1, 24.8, 16.9; ^{11}B NMR (193 MHz, $CDCl_3$) δ = 32.8. HRMS (ESI$^+$): Calcd for $C_{23}H_{38}BO_2$ [M+H]$^+$ 357.2960, Found 357.2961.

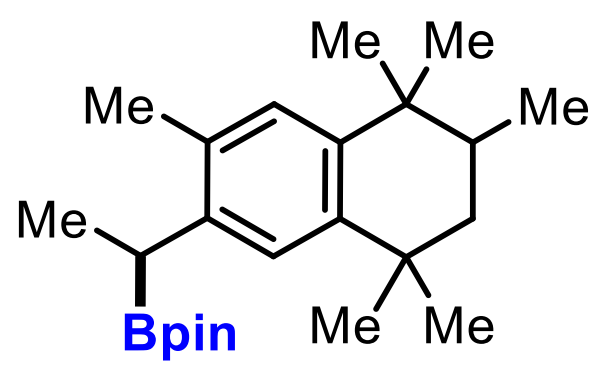

2-(-1-(3,5,5,6,8,8-Hexamethyl-5,6,7,8-tetrahydronaphthalen-2-yl)ethyl)-4,4,5,5-tetramethyl-1,3,2-dioxaborolane (9ae)

The title compound was prepared according to the general procedure as a colorless oil (**A:** total: 50.4 mg, 68% yield, d.r. = 1:1). ^{1}H NMR (400 MHz, $CDCl_3$) δ = 7.11 (d, *J* = 3.2 Hz, 1H), 7.06 (d, *J* = 1.6 Hz, 1H), 2.51-2.49 (m, 1H), 2.27 (s, 3H), 1.88-1.81 (m, 1H), 1.65-1.57 (m, 1H), 1.30-1.19 (m, 25H), 1.05 (d, *J* = 8.8 Hz, 3H), 0.97 (d, *J* = 6.8 Hz, 3H); ^{13}C NMR (101 MHz, $CDCl_3$) δ = 142.0, 140.30, 140.27, 132.83, 132.80, 128.49, 128.46, 125.8, 125.6, 83.2, 44.0, 37.3, 34.8, 34.18, 34.15, 32.54, 32.46, 32.3, 32.2, 28.8, 28.6, 25.2, 25.1, 24.9, 24.8, 19.71, 19.65, 17.5, 17.4, 17.0; ^{11}B NMR (193

MHz, $CDCl_3$) δ = 32.8. HRMS (ESI^+): Calcd for $C_{24}H_{40}BO_2$ $[M+H]^+$ 371.3116, Found 371.3118.

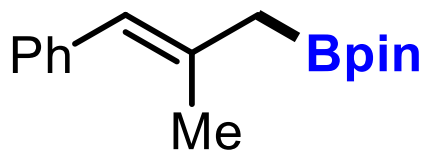

(*E*)-4,4,5,5-Tetramethyl-2-(2-methyl-3-phenylallyl)-1,3,2-dioxaborolane (9af)

The title compound was prepared according to the general procedure as a colorless oil (**B:** total: 42.3 mg, 82% yield; **C:** 31.3 mg, 80% yield). ^{1}H NMR (400 MHz, $CDCl_3$) δ = 7.29-7.28 (m, 2H), 7.27-7.25 (m, 2H), 7.16-7.12 (m, 1H), 6.26 (s, 1H), 1.93 (d, *J* = 1.2 Hz, 3H), 1.90 (s, 2H), 1.25 (s, 12H); ^{13}C NMR (101 MHz, $CDCl_3$) δ = 138.9, 136.5, 128.7, 128.1, 125.7, 124.3, 83.5, 29.8, 27.0, 24.8; ^{11}B NMR (193 MHz, $CDCl_3$) δ = 31.9,. HRMS (ESI^+): Calcd for $C_{16}H_{24}BO_2$ $[M+H]^+$ 259.1854, Found 259.1865.

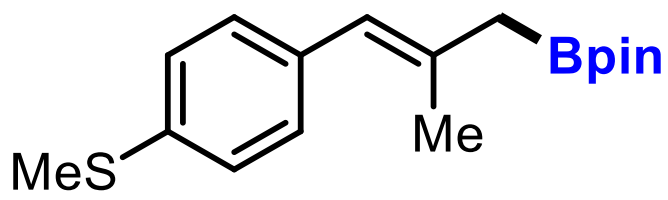

(*E*)-4,4,5,5-Tetramethyl-2-(2-methyl-3-(4-(methylthio)phenyl)allyl)-1,3,2-dioxaborolane (9ag)

The title compound was prepared according to the general procedure as a colorless oil (**B:** 52.9 mg, 87% yield; **C:** 47.4 mg, 78% yield). ^{1}H NMR (600 MHz, $CDCl_3$) δ = 7.21-7.15 (m, 4H), 6.20 (s, 1H), 2.46 (s, 3H), 1.92 (d, *J* = 1.8 Hz, 3H), 1.80 (s, 2H), 1.24 (s, 12H); ^{13}C NMR (101 MHz, $CDCl_3$) δ = 136.5, 136.0, 135.2, 129.2, 126.7, 123.7, 83.5, 27.0, 24.8, 16.2; ^{11}B NMR (193 MHz, $CDCl_3$) δ = 32.4. HRMS (ESI^+): Calcd for $C_{17}H_{26}BO_2S$ $[M+H]^+$ 305.1742, Found 305.1743.

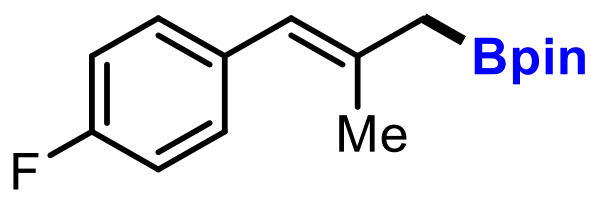

(*E*)-2-(3-(4-Fluorophenyl)-2-methylallyl)-4,4,5,5-tetramethyl-1,3,2-dioxaborolane (9ah)

The title compound was prepared according to the general procedure as a colorless oil (**B:** 48.1 mg, 87% yield; **C:** 43.1 mg, 78% yield, *E*/*Z* = 2:1). (***E***): ^{1}H NMR (400 MHz, $CDCl_3$) δ = 7.22-7.14 (m, 2H), 6.99-6.94 (m, 2H), 6.21 (s, 1H), 1.91 (d, *J* = 1.6 Hz, 3H), 1.85 (s, 2H), 1.25 (s, 12H); (***Z***): ^{1}H NMR (400 MHz, $CDCl_3$) δ = 7.22-7.14 (m, 1H), 6.99-6.94 (m, 1H), 6.21 (s, 0.5H), 1.86 (d, *J* = 1.2 Hz, 1.5H), 1.85 (s, 1H), 1.25 (s, 6H); ^{13}C NMR (101 MHz, $CDCl_3$) δ = 162.3 (d, *J* = 245.4 Hz), 162.2 (d, *J* = 245.4 Hz), 136.4, 136.2, 134.9, 130.4 (d, *J* = 8.1 Hz), 130.2 (d, *J* = 8.1 Hz), 123.7, 123.2, 115.0 (d, *J* = 21.2 Hz), 114.9 (d, *J* = 21.2 Hz), 83.5, 83.4, 26.9, 24.8, 19.7; ^{11}B NMR

(193 MHz, $CDCl_3$) δ = 32.2. HRMS (ESI^+): Calcd for $C_{16}H_{23}BFO_2$ $[M+H]^+$ 277.1770, Found 277.1770.

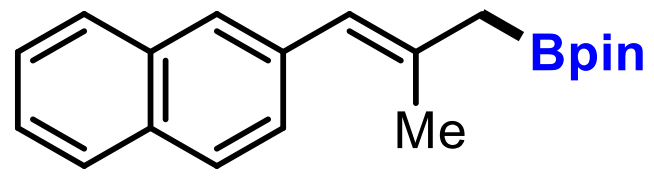

(*E*)-4,4,5,5-Tetramethyl-2-(2-methyl-3-(naphthalen-2-yl)allyl)-1,3,2-dioxaborolane (9ai)

The title compound was prepared according to the general procedure as a colorless solid (**B:** 51.8 mg, 84% yield; **C:** 43.2 mg, 70% yield). ^1H NMR (400 MHz, $CDCl_3$) δ = 7.79-7.74 (m, 4H), 7.43-7.37 (m, 3H), 6.42 (s, 1H), 1.99-1.98 (m, 5H), 1.28 (s, 12H); ^{13}C NMR (101 MHz, $CDCl_3$) δ = 137.2, 136.4, 133.6, 131.9, 127.9, 127.7, 127.6, 127.0, 125.9, 125.3, 124.3, 83.6, 27.1, 24.9; ^{11}B NMR (193 MHz, $CDCl_3$) δ = 32.1. HRMS (ESI^+): Calcd for $C_{20}H_{26}BO_2$ $[M+H]^+$ 309.2021, Found 309.2025.

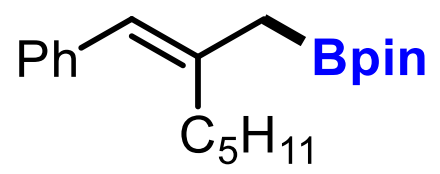

(*E*)-2-(2-Benzylideneheptyl)-4,4,5,5-tetramethyl-1,3,2-dioxaborolane (9aj)

The title compound was prepared according to the general procedure as a colorless solid (**B:** 51.5 mg, 82% yield; **C:** 50.3 mg, 80% yield). ^1H NMR (400 MHz, $CDCl_3$) δ = 7.28-7.27 (m, 3H), 7.17-7.13 (m, 2H), 6.26 (s, 1H), 1.93 (d, *J* = 1.2 Hz, 3H), 1.90 (s, 2H), 1.25 (s, 12H); ^{13}C NMR (101 MHz, $CDCl_3$) δ = 138.9, 136.5, 128.7, 128.1, 125.7, 124.3, 83.5, 29.8, 27.0, 24.8; ^{11}B NMR (193 MHz, $CDCl_3$) δ = 32.0. HRMS (ESI^+): Calcd for $C_{20}H_{32}BO_2$ $[M+H]^+$ 315.2490, Found 315.2500.

6. General Procedure for the Deoxyborylation of Aldehydes, Ketones and Esters with HBpin 8b by Chromium

A dried Schlenk tube was charged with aldehydes, ketones or esters **10** (0.2 mmol), $CrCl_3$ (10 mol%), Mg (0.6 mmol) and HBpin (0.8 mmol), followed by adding a freshly distilled THF (2 mL) by syringe under atmosphere of nitrogen. The reaction mixture was stirred at 80 °C for 24 h (for aldehydes) or 72 h (for ketones and esters). After cooled room temperature, ethyl acetate (20 mL) was added into the reaction mixture and concentrated under vacuum. The residue was purified by flash chromatography on silica gel to give the desired product **9**.

7. General Procedure for the Deoxygenative Silylation of Aldehydes with Chlorosilanes 2 by Chromium

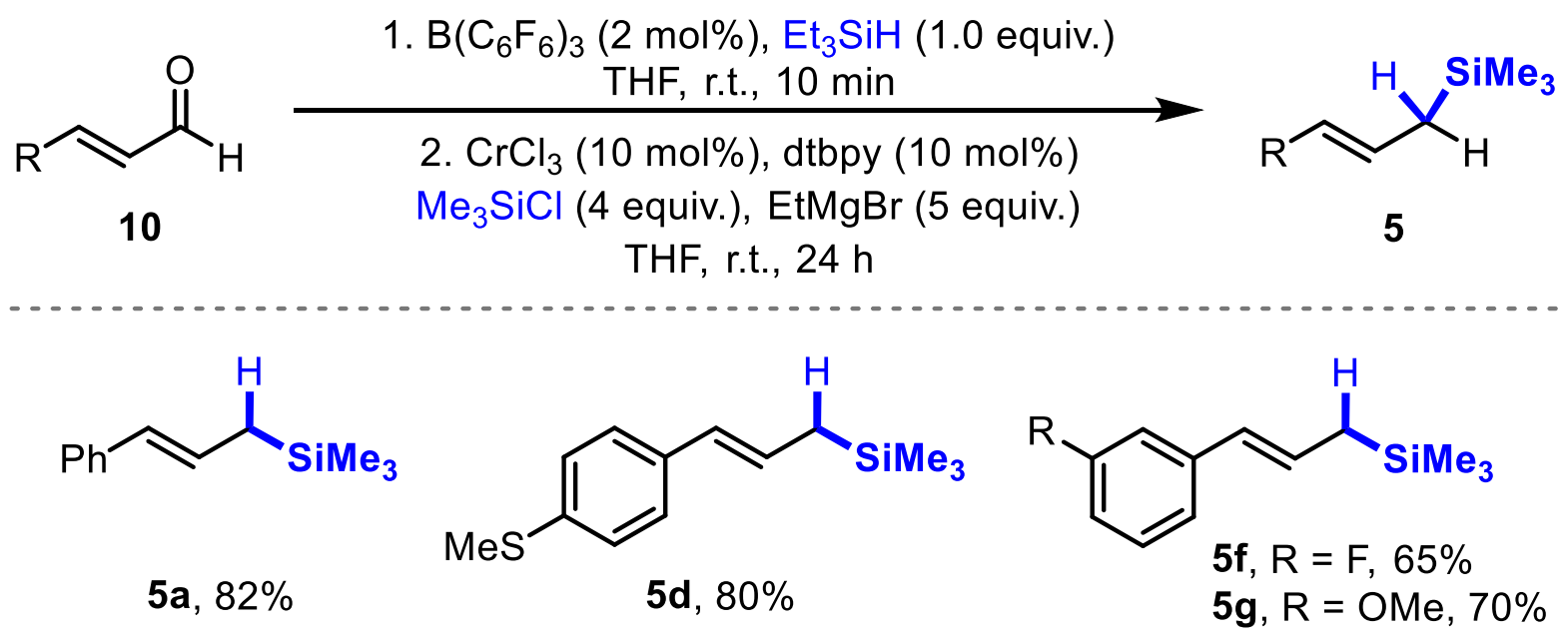

A dried Schlenk tube was charged with aldehydes **10** (0.2 mmol), $B(C_6F_5)_3$ (2 mol%) and Et_3SiH (0.24 mmol), followed by adding a freshly distilled THF (1 mL) by syringe under atmosphere of nitrogen. The reaction mixture was stirred at room temperature for 20 min. Then, $CrCl_3$ (10 mol%), dtbpy (10 mol%) and **2a** (0.8 mmol) were added into the reaction mixture. After stirring at room temperature for 5 min, EtMgBr (1.0 M in THF, 1 mmol) was added dropwise, and the reaction mixture was stirred at room temperature for 24 h. After quenched by aqueous NH_4Cl (2 mL), the crude product was extracted with ethyl acetate (2 x 10 mL). The combined organic phases were dried over anhydrous Na_2SO_4 and concentrated under vacuum. The residue was purified by flash chromatography on silica gel to give the product **5**.

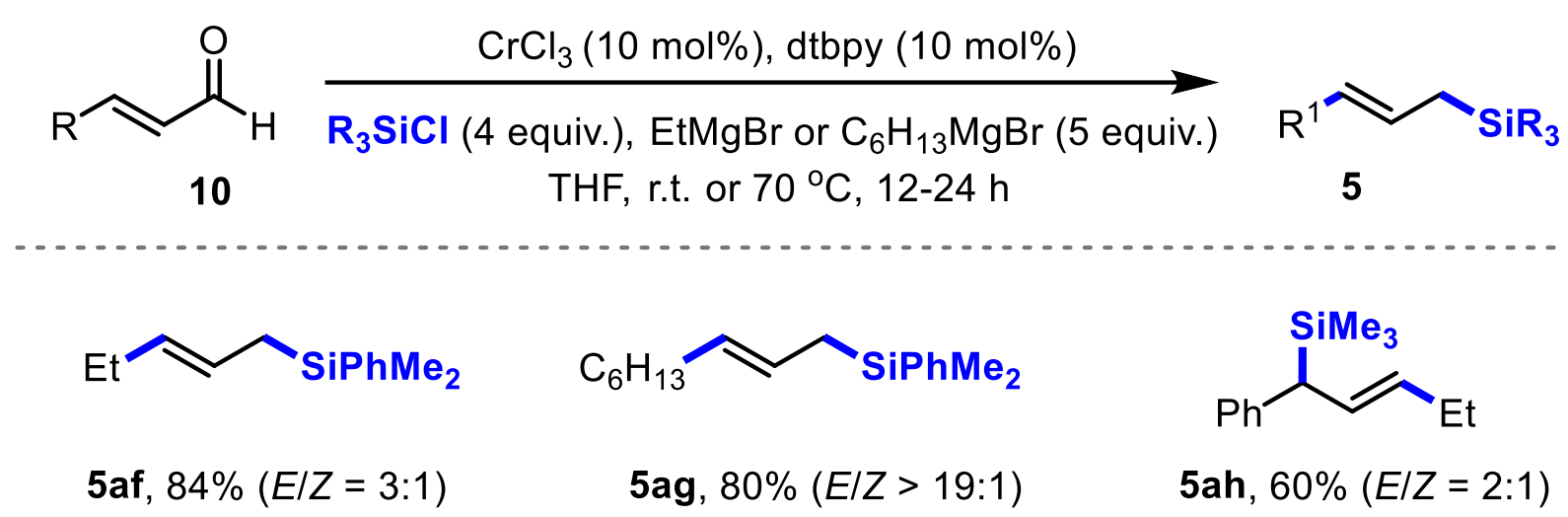

A dried Schlenk tube was charged with aldehydes **10** (0.2 mmol), $CrCl_3$ (10 mol%), dtbpy (10 mol%) and **2** (0.5 mmol), followed by adding a freshly distilled THF (0.8 mL) by syringe under atmosphere of nitrogen. After stirring at room temperature for 5 min, EtMgBr (1.0 M in THF, 1.2 mmol) was added dropwise, and the reaction mixture was stirred at room temperature (for acrylaldehyde) or 70 °C (for cinnamaldehyde) for 24 h. After quenched by aqueous NH_4Cl (2 mL), the crude product was extracted with ethyl acetate (2 x 10 mL). The combined organic phases were dried over anhydrous Na_2SO_4 and concentrated under vacuum. The residue was purified by flash chromatography on silica gel to give the desired product **5**.

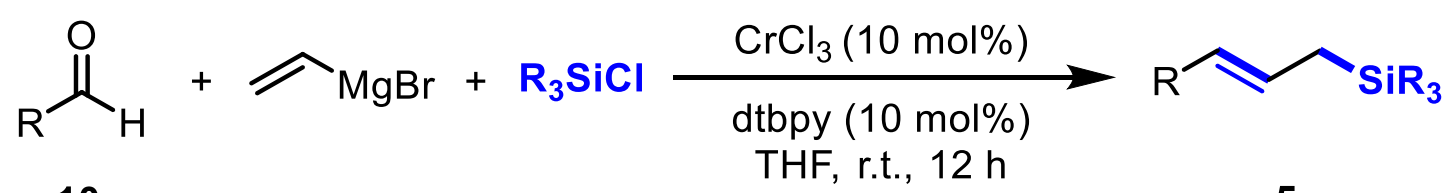

A dried Schlenk tube was charged with aldehydes **10** (0.2 mmol), $CrCl_3$ (10 mol%), dtbpy (10 mol%) and **2** (0.5 mmol), followed by adding a freshly distilled THF (0.8 mL) by syringe under atmosphere of nitrogen. After stirring at room temperature for 5 min, vinylmagnesium bromide (1.0 M in THF, 1.2 mmol) was added dropwise, and the reaction mixture was stirred at room temperature for 24 h. After quenched by aqueous NH_4Cl (2 mL), the crude product was extracted with ethyl acetate (2 x 10 mL). The combined organic phases were dried over anhydrous Na_2SO_4 and concentrated under vacuum. The residue was purified by flash chromatography on silica gel to give the desired product **5**.

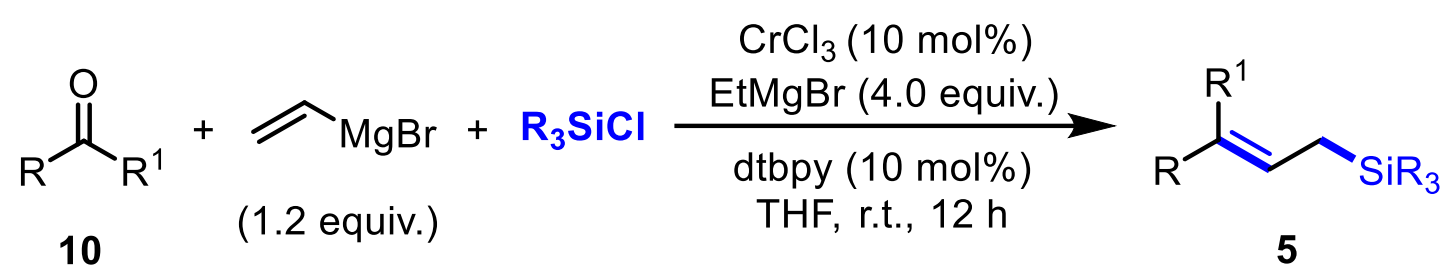

A dried Schlenk tube was charged with aldehydes **10** (0.2 mmol), $CrCl_3$ (10 mol%), dtbpy (10 mol%) and **2** (0.5 mmol), followed by adding a freshly distilled THF (0.8 mL) and vinylmagnesium bromide (1.0 M in THF, 0.24 mmol) by syringe under atmosphere of nitrogen. After stirring at room temperature for 2 h, EtMgBr (1.0 M in THF, 1.0 mmol) was added dropwise, and the reaction mixture was stirred at room temperature for 24 h. After quenched by aqueous NH_4Cl (2 mL), the crude product was extracted with ethyl acetate (2 x 10 mL). The combined organic phases were dried over anhydrous Na_2SO_4 and concentrated under vacuum. The residue was purified by flash chromatography on silica gel (petroleum ether) to give the desired product **5**. This method constitutes a superior alternative to the procedure above.

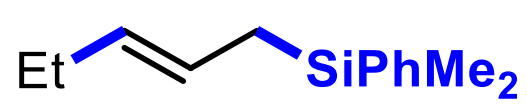

(*E*)-Dimethyl(pent-2-en-1-yl)(phenyl)silane (5af)

The title compound was prepared according to the general procedure as a colorless oil (total: 34.3 mg, 84% yield, *E*/*Z* = 3:1). **(*E*)**: ^{1}H NMR (400 MHz, $CDCl_3$): δ = 7.52-7.48 (m, 2H), 7.35-7.33 (m, 3H), 5.38-5.27 (m, 2H), 2.00-1.93 (m, 2H), 1.63 (d, J = 7.2 Hz, 2H), 0.96-0.86 (m, 3H), 0.25 (s, 6H); **(*Z*):** ^{1}H NMR (400 MHz, $CDCl_3$): δ = 7.52-7.48 (m, 0.6H), 7.35-7.33 (m, 0.9H), 5.38-5.27 (m, 0.6H), 2.00-1.93 (m, 0.6H), 1.68 (d, J = 9.2 Hz, 0.6H), 0.96-0.86 (m, 0.9H), 0.27 (s, 1.8H); ^{13}C NMR (101 MHz, $CDCl_3$): δ = 139.2, 133.7, 131.5, 130.2, 129.0, 127.8, 124.4, 27.9, 25.9, 22.8, 21.6,

17.5, 14.5, -3.0, -3.3. HRMS (ESI+): Calcd for $C_{13}H_{20}SiNa$ [M+Na]+ 227.1227, found 227.1225.

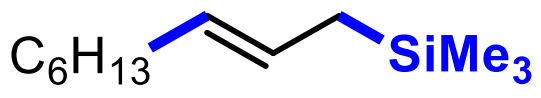

(*E*)-Trimethyl(non-2-en-1-yl)silane (5ag)

The title compound was prepared according to the general procedure as a colorless oil (35.0 mg, 80% yield). ^1H NMR (400 MHz, $CDCl_3$): δ = 5.39-5.19 (m, 2H), 1.98-1.93 (m, 2H), 1.37 (d, J = 7.6 Hz, 2H), 1.31-1.25 (m, 8H), 0.87 (t, J = 6.8 Hz, 3H), -0.03 (s, 9H); ^{13}C NMR (101 MHz, $CDCl_3$): δ = 129.1, 126.0, 32.9, 31.9, 30.1, 28.9, 22.8, 22.7, 14.2, -1.9. HRMS (ESI+): Calcd for $C_{12}H_{26}SiNa$ [M+Na]+ 221.1696, found 221.1690.

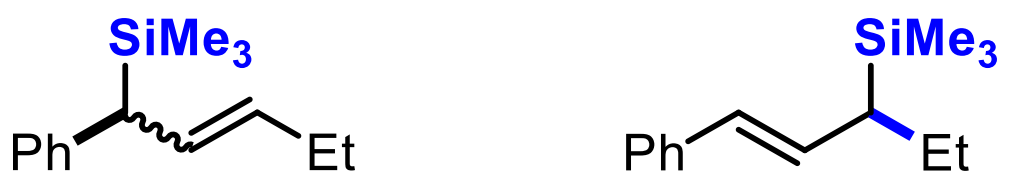

5ah (*E/Z* = 2:1) **5ah'**

(*E*)-Trimethyl(1-phenylpent-2-en-1-yl)silane (*E*-5ah), (*Z*)-trimethyl(1-phenylpent-2-en-1-yl)silane (*Z*-5ah) and (*E*)-trimethyl(1-phenylpent-1-en-3-yl)silane(5ah')

The mixture of title compound was prepared according to the general procedure as a colorless oil (total: 26.2 mg, 60% yield, r.r. = 3:1; *E*/*Z* = 2:1). **(*E*-5ah):** ^1H NMR (400 MHz, $CDCl_3$): δ = 7.33-7.05 (m, 5H), 5.81-5.73 (m, 1H), 5.46-5.36 (m, 1H), 2.86 (d, J = 10.0 Hz, 1H), 2.07-2.01 (m, 2H), 1.00-0.93 (m, 3H), -0.06 (s, 9H); **(*Z*-5ah):** ^1H NMR (400 MHz, $CDCl_3$): δ = 7.33-7.05 (m, 3.0H), 5.81-5.73 (m, 0.6H), 5.46-5.36 (m, 0.6H), 3.23 (d, J = 11.6 Hz, 1H), 2.07-2.01 (m, 1.2H), 1.00-0.93 (m, 1.8H), -0.05 (s, 5.4H); **(5ah'):** ^1H NMR (400 MHz, $CDCl_3$): δ = 7.33-7.05 (m, 2.5H), 6.22 (d, J = 15.6 Hz, 0.5H), 6.06 (dd, J = 15.6, 9.2 Hz, 0.5H), 2.15-2.11 (m, 0.5H), 1.57-1.54 (m, 2H), 1.00-0.93 (m, 1.5H), 0.01 (s, 4.5H); ^{13}C NMR (101 MHz, $CDCl_3$): δ = 143.3, 138.6, 133.2, 130.9, 129.9, 128.5, 128.3, 128.1, 128.0, 127.7, 127.2, 127.1, 126.3, 125.6, 124.5, 42.8, 37.7, 36.7, 26.0, 22.2, 20.8, 14.7, 14.4, -2.89, -2.92. HRMS (ESI+): Calcd for $C_{14}H_{22}SiNa$ [M+Na]+ 241.1383, found 241.1380.

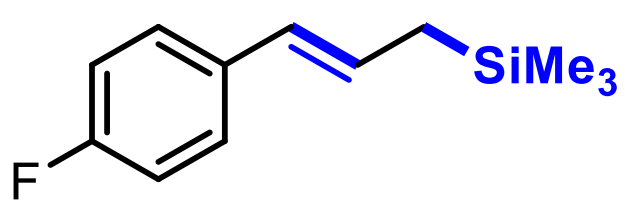

(*E*)-(3-(4-fluorophenyl)allyl)trimethylsilane (5ai)

The title compound was prepared according to the general procedure as a colorless oil (20.0 mg, 48% yield). ^1H NMR (400 MHz, $CDCl_3$): δ = 7.26-7.22 (m, 2H), 6.97-6.93 (m, 2H), 6.21-6.13 (m, 2H), 1.63 (d, J = 6.8 Hz, 2H), 0.04 (s, 9H); ^{13}C NMR (101

MHz, $CDCl_3$): δ = 162.8 (d, J = 245.4 Hz), 134.7 (d, J = 3.0 Hz), 127.7, 127.1, 126.9 (d, J = 7.0 Hz), 115.4 (d, J = 21.2 Hz), 23.9, -1.8. HRMS (ESI^+): Calcd for $C_{12}H_{17}FSiNa$ $[M+Na]^+$ 231.0976, found 231.0976.

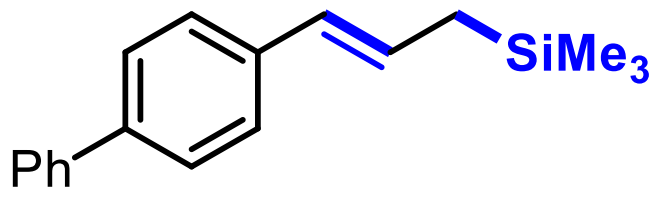

(*E*)-(3-([1,1'-biphenyl]-4-yl)allyl)trimethylsilane (5aj)

The title compound was prepared according to the general procedure as a colorless oil (total: 38.4 mg, 72% yield, *E*/*Z* = 3:1). **(*E*):** ^{1}H NMR (400 MHz, $CDCl_3$): δ = 7.60-7.58 (m, 2H), 7.53-7.51 (m, 2H), 7.45-7.37 (m, 4H), 7.34-7.32 (m, 1H), 6.53-6.24 (m, 2H), 1.69 (d, J = 6.8 Hz, 2H), 0.06 (s, 9H); **(*Z*):** ^{1}H NMR (400 MHz, $CDCl_3$): δ = 7.60-7.58 (m, 0.8H), 7.53-7.51 (m, 0.8H), 7.45-7.37 (m, 1.6H), 7.34-7.32 (m, 0.4H), 6.53-6.24 (m, 0.8H), 1.92 (d, J = 8.0 Hz, 0.8H), 0.06 (s, 3.6H); ^{13}C NMR (101 MHz, $CDCl_3$): δ = 141.1, 139.6, 139.1, 137.7, 137.1, 130.7, 129.1, 128.8, 128.3, 127.8, 127.3, 127.1, 127.0, 126.3, 126.0, 24.2, 18.7, -1.7. HRMS (ESI^+): Calcd for $C_{18}H_{22}SiNa$ $[M+Na]^+$ 289.1383, found 289.1390.

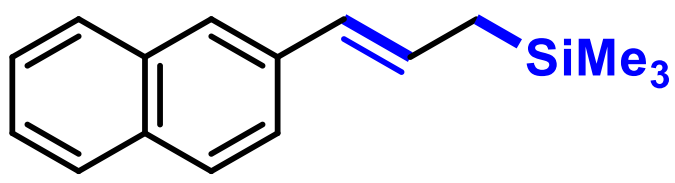

(*E*)-trimethyl(3-(naphthalen-2-yl)allyl)silane (5ak)

The title compound was prepared according to the general procedure as a colorless oil (29.8 mg, 62% yield). ^{1}H NMR (400 MHz, $CDCl_3$): δ = 7.77-7.73 (m, 3H), 7.62 (s, 1H), 7.56-7.54 (m, 1H), 7.44-7.36 (m, 2H), 6.42-6.37 (m, 2H), 1.71 (d, J = 6.8 Hz, 2H), 0.07 (s, 9H); ^{13}C NMR (101 MHz, $CDCl_3$): δ = 136.0, 133.9, 132.4, 128.58, 128.45, 128.1, 127.8, 127.7, 126.2, 125.2, 124.6, 123.5, 24.3, -1.7. HRMS (ESI^+): Calcd for $C_{16}H_{20}SiNa$ $[M+Na]^+$ 263.1227, found 263.1228.

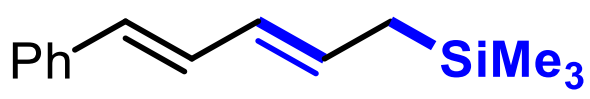

Trimethyl((2*E*,4*E*)-5-phenylpenta-2,4-dien-1-yl)silane (5al)

The title compound was prepared according to the general procedure as a colorless oil (total: 26.0 mg, 60% yield, *E*/*Z* = 7:1). ^{1}H NMR (400 MHz, $CDCl_3$): δ = 7.37 (d, J = 8.4 Hz, 2H), 7.28 (t, J = 8.4 Hz, 2H), 7.16 (d, J = 8.4 Hz, 1H), 6.75 (dd, J = 15.6, 10.4 Hz, 1H), 6.36 (d, J = 15.6 Hz, 1H), 6.07 (dd, J = 14.9, 10.4 Hz, 1H), 5.88-5.80 (m, 1H), 1.62 (d, J = 9.2 Hz, 2H), 0.03 (s, 9H); ^{13}C NMR (101 MHz, $CDCl_3$): δ = 138.0, 133.0, 130.0, 129.4, 128.6, 128.1, 126.8, 126.0, 24.2, -1.8. HRMS (ESI^+): Calcd for $C_{16}H_{20}SiNa$ $[M+Na]^+$ 239.1227, found 239.1228.

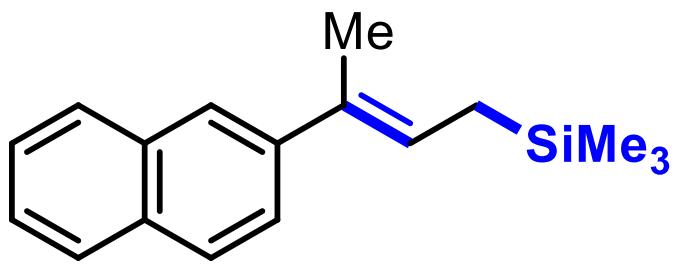

(*E*)-trimethyl(3-(naphthalen-2-yl)but-2-en-1-yl)silane (5am)

The title compound was prepared according to the general procedure as a colorless oil (total: 29.5 mg, 58% yield, *E*/*Z* = 3:1). **(*E*):** ^{1}H NMR (400 MHz, $CDCl_3$): δ = 7.81-7.72 (m, 4H), 7.57 (dd, *J* = 8.4, 1.6 Hz, 1H), 7.45-7.39 (m, 2H), 6.05 (t, *J* = 8.8 Hz, 1H), 2.09 (s, 3H), 1.74 (d, *J* = 8.4, 2H), 0.07 (s, 9H); **(*Z*):** ^{1}H NMR (400 MHz, $CDCl_3$): δ = 7.81-7.72 (m, 1.2H), 7.57 (dd, *J* = 8.4, 1.6 Hz, 0.3H), 7.45-7.39 (m, 0.6H), 6.05 (t, *J* = 8.8 Hz, 0.3H), 2.14 (s, 0.9H), 1.87 (d, *J* = 6.8, 0.6H), 0.07 (s, 1.8H); ^{13}C NMR (101 MHz, $CDCl_3$): δ = 141.6, 133.7, 128.1, 128.0, 127.6, 127.5, 126.0, 125.8, 125.4, 125.3, 124.4, 123.9, 123.5, 20.7, 15.7, 14.6, 0.1, -1.4. HRMS (ESI$^+$): Calcd for $C_{17}H_{22}SiNa$ [M+Na]$^+$ 277.1383, found 277.1387.

8. Gram-Scale Reactions

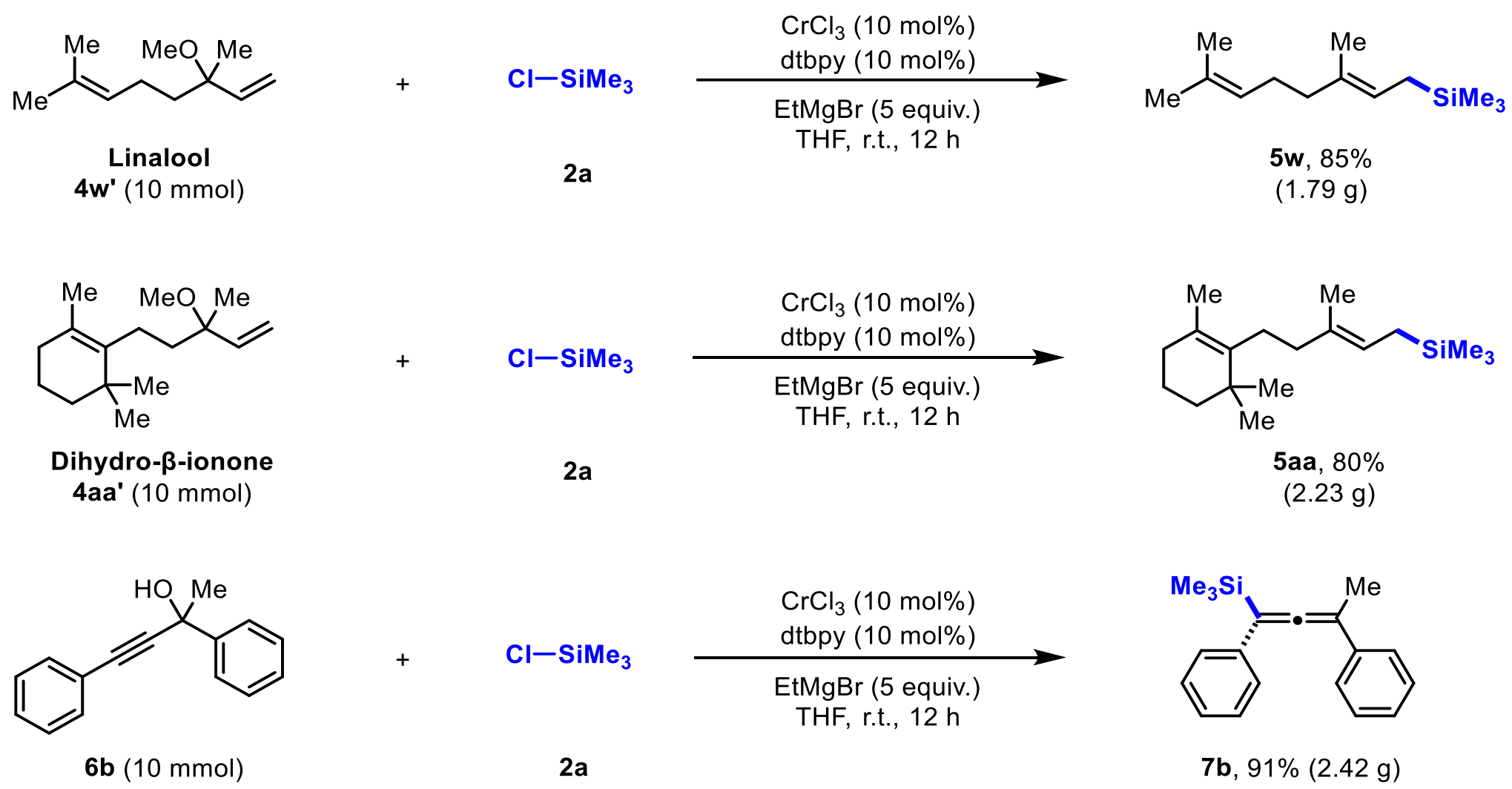

A dried Schlenk tube was charged with methyl ethers **4w'** or **4aa'** (10 mmol), $CrCl_3$ (10 mol%), dtbpy (10 mol%) and **2a** (40 mmol), followed by adding a freshly distilled THF (10 mL) by syringe under atmosphere of nitrogen. After stirring at room temperature for 5 min, EtMgBr (1.0 M in THF, 50 mmol) was added dropwise, and the reaction mixture was stirred at room temperature for 12 h. After quenched by aqueous NH_4Cl (50 mL), the crude product was extracted with ethyl acetate (2 x 50 mL). The combined organic phases were dried over anhydrous Na_2SO_4 and concentrated under vacuum. The residue was purified by flash chromatography on silica gel (petroleum ether) to give the desired product **5w** or **5aa**.

A dried Schlenk tube was charged with methyl ethers **6b** (10 mmol), $CrCl_3$ (10 mol%), dtbpy (10 mol%) and **2a** (50 mmol), followed by adding a freshly distilled THF (10 mL) by syringe under atmosphere of nitrogen. After stirring at room temperature for 5 min, EtMgBr (1.0 M in THF, 50 mmol) was added dropwise, and the reaction mixture was stirred at room temperature for 12 h. After quenched by aqueous NH_4Cl (50 mL), the crude product was extracted with ethyl acetate (2 x 50 mL). The combined organic phases were dried over anhydrous Na_2SO_4 and concentrated under vacuum. The residue was purified by flash chromatography on silica gel (petroleum ether) to give the desired product **7b**.

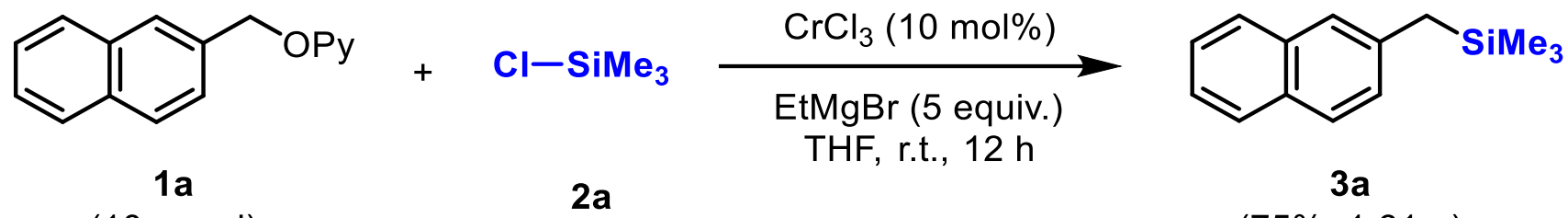

1a (10 mmol) **2a** **3a** (75%, 1.61 g)

A dried Schlenk tube was charged with methyl ethers **1a** (10 mmol), $CrCl_3$ (10 mol%) and **2a** (40 mmol), followed by adding a freshly distilled THF (10 mL) by syringe under atmosphere of nitrogen. After stirring at room temperature for 5 min, EtMgBr (1.0 M in THF, 40 mmol) was added dropwise, and the reaction mixture was stirred at room temperature for 12 h. After quenched by aqueous NH_4Cl (40 mL), the crude product was extracted with ethyl acetate (2 x 40 mL). The combined organic phases were dried over anhydrous Na_2SO_4 and concentrated under vacuum. The residue was purified by flash chromatography on silica gel (petroleum ether) to give the desired product **7b**.

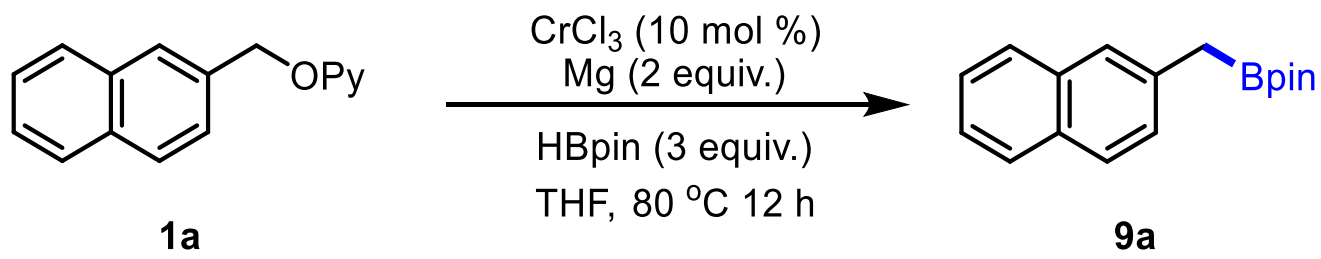

1a (7 mmol, 1.65 g) **9a** (67%, 1.26 g)

A dried Schlenk tube was charged with **1aa** (7 mmol), $CrCl_3$ (10 mol%), Mg (14 mmol) and HBpin (21 mmol), followed by adding a freshly distilled THF (20 mL) by syringe under atmosphere of nitrogen. The reaction mixture was stirred at 80 °C for 12 h. After quenched by aqueous NH_4Cl (20 mL), the crude product was extracted with ethyl acetate (3 x 30 mL). The combined organic phases were dried over anhydrous Na_2SO_4 and concentrated under vacuum. The residue was purified by flash chromatography on silica gel (petroleum ether) to give the desired product **9a** (67%, 1.26 g).

9. Transformations of 3a

Synthesis of *N*-(2-(naphthalen-2-yl)-1-phenylethyl)aniline

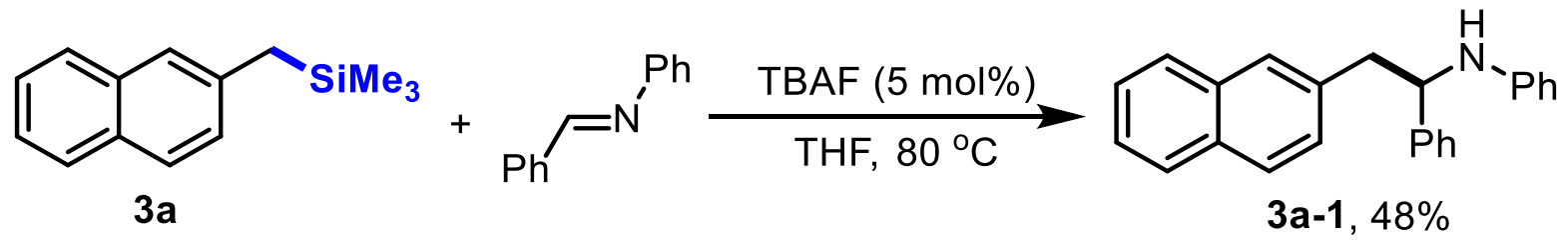

An oven-dried 8 mL screw-cap vial equipped with a magnetic stir bar was added **3a** (42.8 mg, 0.2 mmol, 1.0 equiv), *N*-Benzylideneaniline (54 mg, 0.3 mmol, 1.5 equiv), 4Å MS (100 mg), TBAF (0.01 mL, 0.01 mmol, 1M solution in anhydrous THF), anhydrous THF (1.0 mL), removed from the glove box and stirred at 80 °C for 12 h. After quenched with a saturated $NaHCO_3$ solution, the mixture was extracted with EtOAc. The organic layer was concentrated to give the crude product. The crude product was purified by column chromatography on silica gel (EtOAc/PE = 1/20) to afford the title compound as a yellow solid (32 mg, 48% yield).

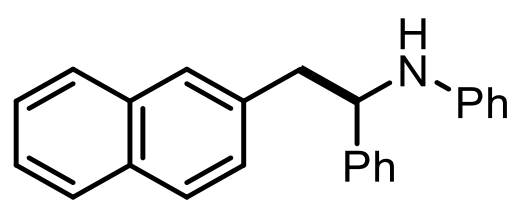

***N*-(2-(naphthalen-2-yl)-1-phenylethyl)aniline (3a-1)**

^{1}H NMR (400 MHz, $CDCl_3$) δ = 7.74–7.64 (m, 3H), 7.49 (s, 1H), 7.38–7.35 (m, 2H), 7.29–7.18 (m, 4H), 7.15 (t, *J* = 7.7 Hz, 2H), 6.95 (dd, *J* = 8.3, 7.5 Hz, 2H), 6.53 (t, *J* = 7.3 Hz, 1H), 6.37 (d, *J* = 7.8 Hz, 2H), 4.61 (dd, *J* = 8.0, 5.8 Hz, 1H), 4.07 (s, 1H), 3.21 (dd, *J* = 14.0, 5.6 Hz, 1H), 3.08 (dd, *J* = 14.0, 8.2 Hz, 1H). **^{13}C NMR** (101 MHz, $CDCl_3$) δ = 147.3, 143.5, 135.3, 133.6, 132.5, 129.1, 128.7, 128.3, 128.0, 127.8, 127.7, 127.5, 127.2, 126.6, 126.2, 125.7, 117.6, 113.8, 59.2, 45.5.**HRMS** (ESI$^+$): Calcd for $C_{24}H_{21}NNa[M+Na]^+$ 346.1566, Found:346.1565.

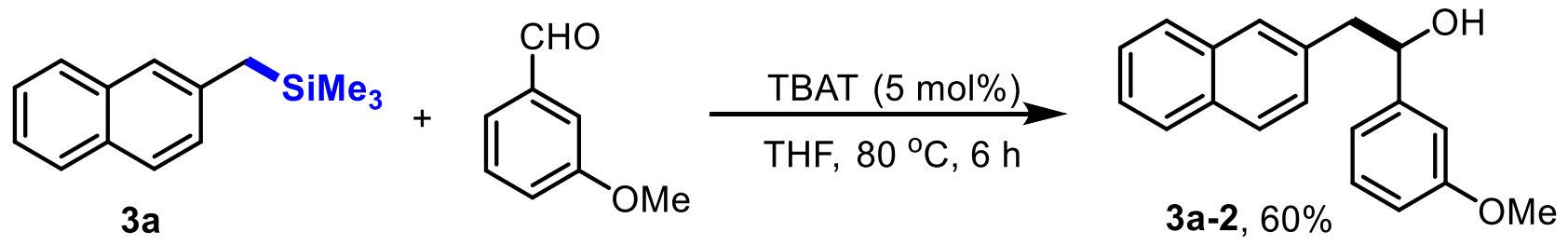

An oven-dried 8 mL screw-cap vial equipped with a magnetic stir bar was added 3a (42.8 mg, 0.2 mmol, 1.0 equiv), 3-methoxybenzaldehyde (54.0 mg, 0.4 mmol, 2.0 equiv), tetrabutylammonium triphenyldifluorosilicate (TBAT) (5.4 mg, 0.01 mmol, 5 mol %), anhydrous THF (1.0 mL), removed from the glove box and stirred at 80 °C for 6 h. After quenched with 1 M HCl (5 mL), the mixture was extracted with EtOAc. The organic layer was concentrated to give the crude product. The crude product was purified by column chromatography on silica gel (EtOAc/PE = 1/5) to afford the title compound as a colorless liquid (33 mg, 60% yield).

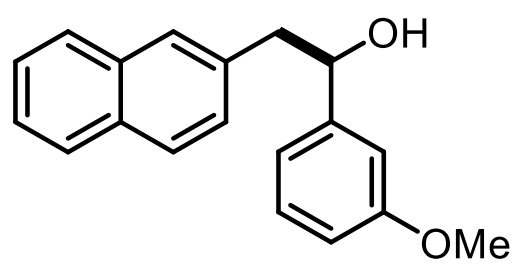

1-(3-methoxyphenyl)-2-(naphthalen-2-yl)ethanol (3a-2)

^{1}H NMR (400 MHz, $CDCl_3$) δ = 7.83–7.72 (m, 3H), 7.64 (s, 1H), 7.48–7.40 (m, 2H), 7.30 (dd, *J* = 8.4, 1.6 Hz, 1H), 7.24 (t, *J* = 8.1 Hz, 1H), 6.96–6.90 (m, 2H), 6.84–6.78 (m, 1H), 4.95–4.91 (m, 1H), 3.76 (s, 3H), 3.20–3.08 (m, 2H). **^{13}C NMR** (101 MHz, $CDCl_3$) δ = 159.8, 145.6, 135.6, 133.6, 132.4, 129.6, 128.2, 128.2, 127.9, 127.7, 127.7, 126.2, 125.6, 118.3, 113.3, 111.4, 75.2, 55.3, 46.3. **HRMS** (ESI$^+$): Calcd for $C_{19}H_{18}O_2Na[M+Na]^+$ 301.1199, Found:301.1197.

Synthesis of methyl 2-(naphthalen-2-yl)acetate

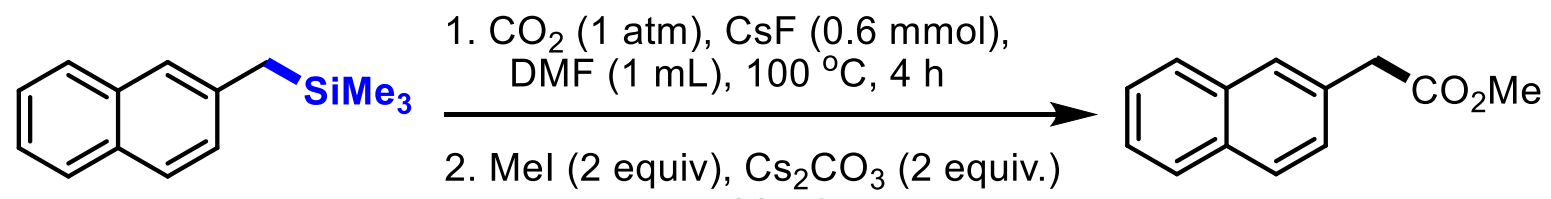

3a **3a-3**, 80%

An oven-dried 8 mL screw-cap vial equipped with a magnetic stir bar was added **3a** (42.8 mg, 0.2 mmol, 1.0 equiv), CsF (91.2 mg, 0.6 mmol, 3.0 equiv), CO_2 (1atm), DMF (1.0 mL), stirred at 100 °C for up to 4 h (the mixture was stirred at 800 rpm). After the reaction was complete, add to MeI (25.0 μL, 0.4 mmol, 2.0 equiv), Cs_2CO_3 (130.0 mg, 0.4 mmol, 2.0 equiv), then stirred at room temperature for 30 min. The reaction mixture was quenched with H_2O, and the mixture was extracted with EtOAc. The organic layer was concentrated to give the crude product. The crude product was purified by column chromatography on silica gel (EtOAc/PE = 1/20) to afford the title compound as a colorless liquid (32.0 mg, 80% yield).

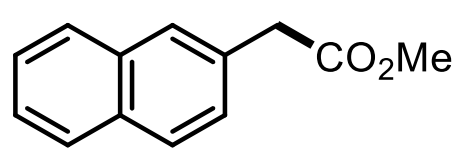

Methyl 2-(naphthalen-2-yl)acetate (3a-3)

^{1}H NMR (400 MHz, $CDCl_3$) δ = 7.86–7.80 (m, 3H), 7.74 (s, 1H), 7.50–7.46 (m, 2H), 7.45–7.41 (m, 1H), 3.81 (s, 2H), 3.72 (s, 3H). **^{13}C NMR** (101 MHz, $CDCl_3$) δ = 172.2, 133.5, 132.6, 131.6, 128.4, 128.1, 127.8, 127.8, 127.5, 126.3, 126.0, 52.3, 41.5. **HRMS** (ESI$^+$): Calcd for $C_{13}H_{12}O_2Na[M+Na]^+$ 223.0730, Found: 223.0732.

Synthesis of 2-naphthyl(trimethylsilyl)methanone

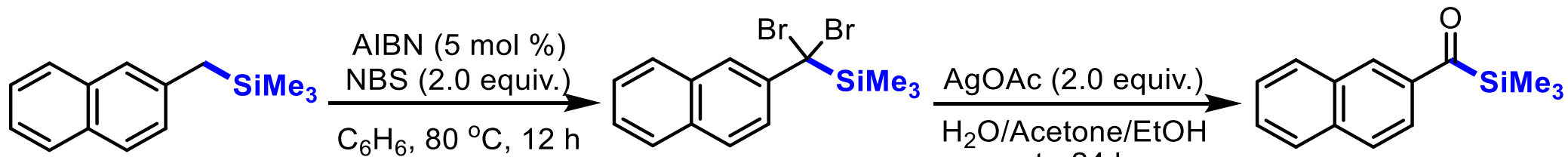

3a **3a-4**, 85%

A dried Schlenk tube was charged with **3a** (0.2 mmol), NBS (0.4 mmol), AIBN (5 mol%) followed by adding a freshly distilled benzene (1 mL). The reaction mixture was stirred at 80 °C for 12 h. Then quenched by aqueous NH_4Cl (2 mL), the crude product was extracted with ethyl acetate (2 x 10 mL). The combined organic phases were dried over anhydrous Na_2SO_4 and concentrated under vacuum. The residue was purified by flash chromatography on silica gel to give the bromination product. Then

the solution of bromination product, AgOAc (0.4 mmol) in H_2O:Acetone:EtOH=1:2:3 (2 mL) was stirred at room temperature for 24 h. The reaction mixure was extracted with ethyl acetate (2 x 10 mL). The combined organic phases were dried over anhydrous Na_2SO_4 and concentrated under vacuum. The residue was purified by flash chromatography on silica gel to give the desired product **3a-4** (85%).

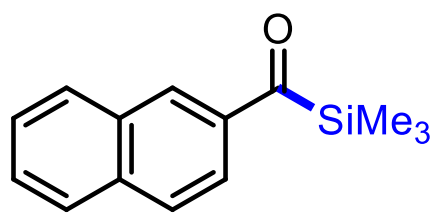

2-Naphthyl(trimethylsilyl)methanone (3a-4)

^{1}H NMR (400 MHz, $CDCl_3$) δ = 8.38 (s, 1H), 7.99 (d, J = 7.8 Hz, 1H), 7.91 (s, 2H), 7.87 (d, J = 8.1 Hz, 1H), 7.62–7.53 (m, 2H), 0.46 (s, 9H); **^{13}C NMR** (101 MHz, $CDCl_3$) δ = 235.5, 138.9, 135.6, 132.8, 130.9, 129.7, 128.8, 128.5, 128.0, 126.8, 122.5, -1.0; HRMS (ESI$^+$): Calcd for $C_{14}H_{17}OSi$ $[M+H]^+$ 229.1049, Found:229.1041.

10. Mechanistic Studies

10.1 Stoichiometric reaction of $CrCl_3$ with ethylmagnesium bromide

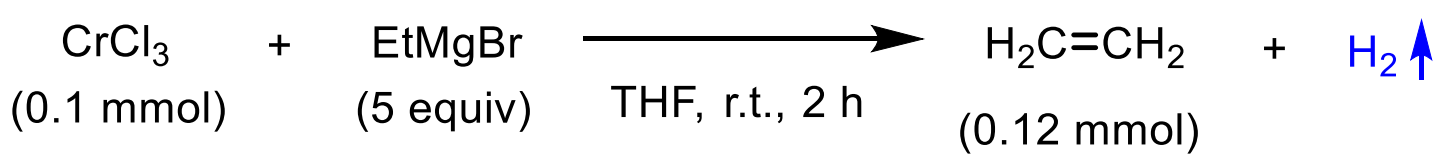

A dried Schlenk tube (25 mL) containing $CrCl_3$ (16 mg, 0.1 mmol) was added freshly distilled THF (2 mL) under nitrogen atmosphere. Ethylmagnesium bromide (5 mmol) was added by syringe at room temperature and the reaction mixture was stirred at room temperature for 2 h. The gas of hydrogen and ethylene were determined by GC-MS.

10.2 Radical clock experiments

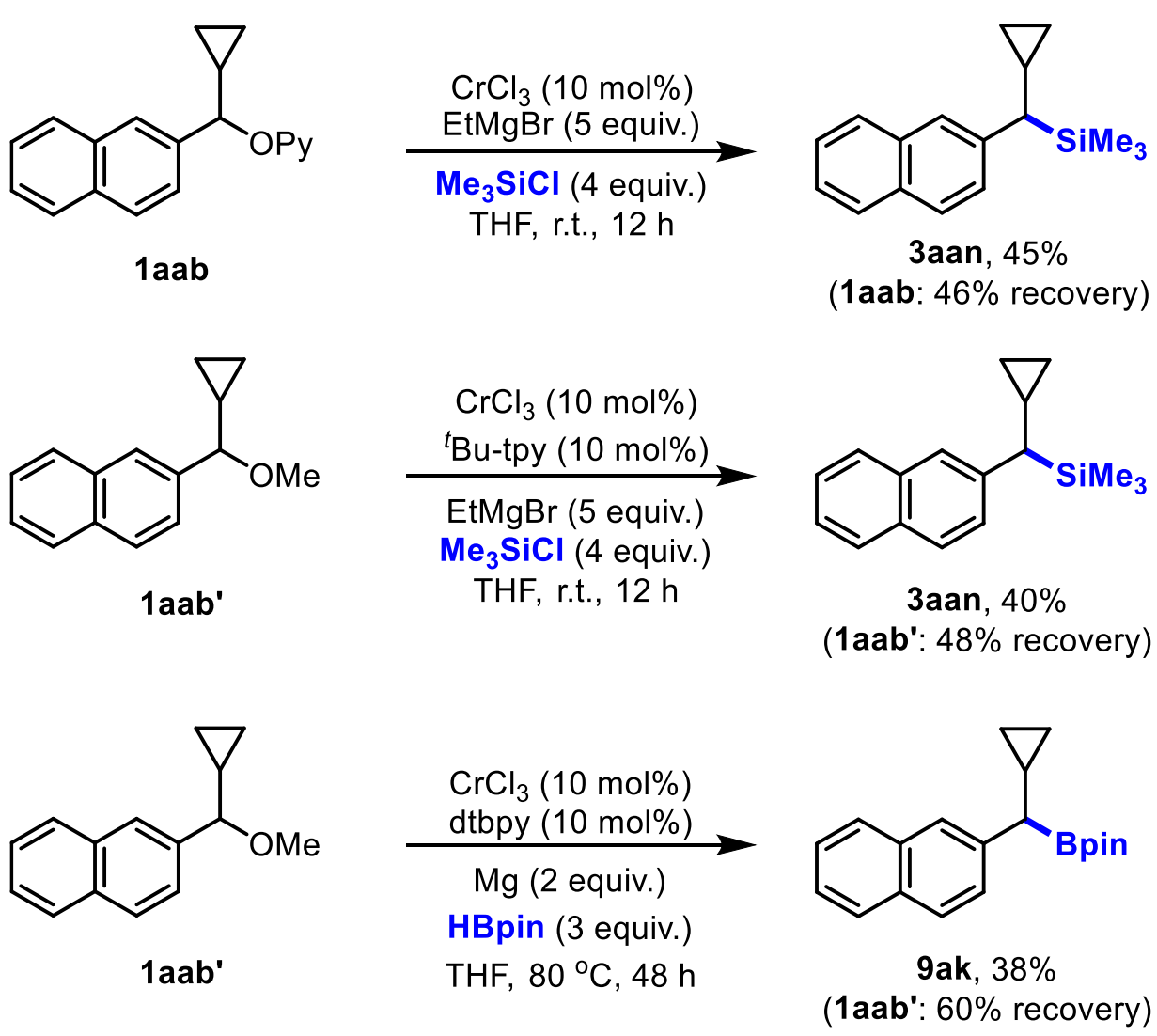

A dried Schlenk tube was charged with 2-pyridyl ether **1aab** (0.2 mmol), $CrCl_3$ (10 mol%), and **2a** (0.8 mmol), followed by adding a freshly distilled THF (1 mL) by syringe under atmosphere of nitrogen. After stirring at room temperature for 5 min, EtMgBr (1.0 M in THF, 1 mmol) was added dropwise, and the reaction mixture was stirred at room temperature for 12 h. After quenched by aqueous NH_4Cl (2 mL), the crude product was extracted with ethyl acetate (3 x 10 mL). The combined organic phases were dried over anhydrous Na_2SO_4 and concentrated under vacuum. The residue was purified by flash chromatography on silica gel (petroleum ether) to give the desired product **3aan**. The ring-opening product was not observed.

A dried Schlenk tube was charged with methyl ether **1aab'** (0.2 mmol), $CrCl_3$ (10 mol%), ^{t}Bu-tpy (10 mol%) and **2a** (0.8 mmol), followed by adding a freshly distilled THF (1 mL) by syringe under atmosphere of nitrogen. After stirring at room temperature for 5 min, EtMgBr (1.0 M in THF, 1 mmol) was added dropwise, and the reaction mixture was stirred at 70 °C for 24 h. After cooled to room temperature and quenched by aqueous NH_4Cl (2 mL), the crude product was extracted with ethyl acetate (3 x 10 mL). The combined organic phases were dried over anhydrous Na_2SO_4 and concentrated under vacuum. The residue was purified by flash chromatography on silica gel (petroleum ether) to give the desired product **3aan**. The ring-opening product was not observed.

A dried Schlenk tube was charged with methyl ether **1aab'** (0.2 mmol), $CrCl_3$ (10 mol%), dtbpy (10 mol%), Mg (0.4 mmol) and HBpin (0.6 mmol), followed by adding a freshly distilled THF (2 mL) by syringe under atmosphere of nitrogen. The reaction mixture was stirred at 80 °C for 48 h. After cooled to room temperature, ethyl acetate (20 mL) was added into the reaction mixture and concentrated under vacuum. The residue was purified by flash chromatography on silica gel to give the desired product **9ak**. The ring-opening product was not observed.

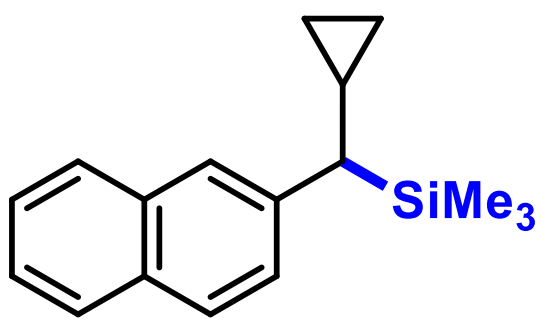

(Cyclopropyl(naphthalen-2-yl)methyl)trimethylsilane (3aan)

^{1}H NMR (400 MHz, $CDCl_3$) δ = 7.78-7.70 (m, 4H), 7.44-7.36 (m, 3H), 1.45 (d, J = 10.8 Hz, 1H), 1.33-1.26 (m, 1H), 0.76-0.71 (m, 1H), 0.53-0.49 (m, 1H), 0.30-0.25 (m, 1H), 0.02 (s, 9H), -0.04--0.07 (m, 1H). ^{13}C NMR (101 MHz, $CDCl_3$) δ = 142.9, 133.7, 131.4, 127.6, 127.4, 127.3, 127.2, 125.7, 124.8, 124.5, 42.8, 11.9, 7.9, 5.2, -2.4. HRMS (ESI$^+$): Calcd for $C_{17}H_{23}Si[M+H]^+$ 255.1564, Found 255.1565.

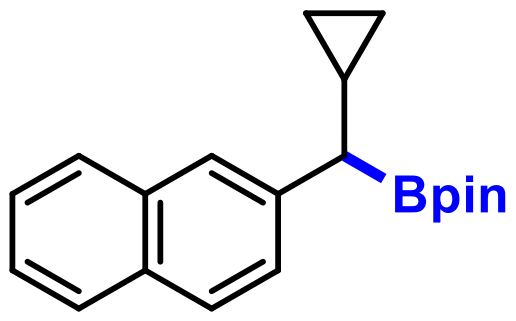

2-(Cyclopropyl(naphthalen-2-yl)methyl)-4,4,5,5-tetramethyl-1,3,2-dioxaborolane (9ak)

^{1}H NMR (400 MHz, $CDCl_3$) δ = 7.79-7.72 (m, 4H), 7.44-7.36 (m, 3H), 1.89 (d, *J* = 9.6 Hz, 1H), 1.31-1.22 (m, 13H), 0.64-0.58 (m, 1H), 0.53-0.47 (m, 1H), 0.34-0.29 (m, 1H), 0.14-0.10 (m, 1H). ^{13}C NMR (101 MHz, $CDCl_3$) δ = 140.9, 133.9, 131.9, 127.64, 127.56, 126.0, 125.74, 125.70, 124.9, 83.5, 24.7, 13.0, 5.1, 5.0; ^{11}B NMR (193 MHz, $CDCl_3$) δ = 32.5. HRMS (ESI$^+$): Calcd for $C_{20}H_{26}BO_2[M+H]^+$ 309.2021, Found 309.2025.

10.3 Investigating the formation of benzyl chromium species

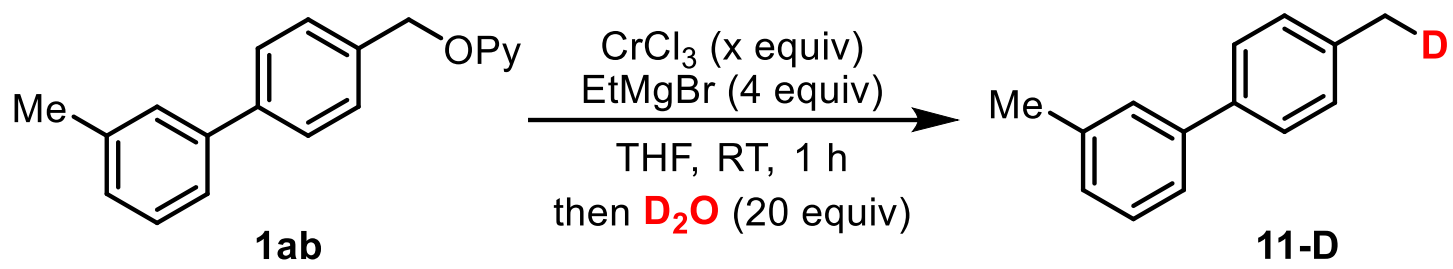

entry	$CrCl_3$	**11-D**
1	0.2 equiv.	13%
2	0.5 equiv.	34%
3	1.0 equiv.	65%

A dried Schlenk tube was charged with **1ab** (0.2 mmol), $CrCl_3$ (0.04, 0.1, 0.2 mmol), followed by adding a freshly distilled THF (1.2 mL) by syringe under atmosphere of nitrogen. After stirring at room temperature for 5 min, EtMgBr (1.0 M in THF, 0.8 mmol) was added dropwise, and the reaction mixture was stirred at room temperature for 1 h. After quenched by aqueous D_2O (4 mmol, 20 equiv.), the crude product was extracted with ethyl acetate (3 x 10 mL). The combined organic phases were dried over anhydrous Na_2SO_4 and concentrated under vacuum. The residue was purified by flash chromatography on silica gel (petroleum ether) to give the desired product **11-D** (13%, 34%, 65%).

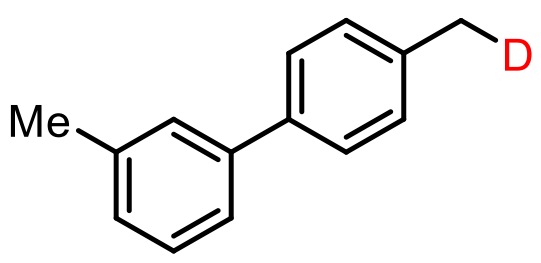

3-Methyl-4'-(methyl-*d*)-1,1'-biphenyl (11-D)

^{1}H NMR (400 MHz, $CDCl_3$) δ = 7.48 (d, *J* = 8.2 Hz, 2H), 7.40–7.35 (m, 2H), 7.31 (t, *J* = 7.5 Hz, 1H), 7.23 (d, *J* = 8.3 Hz, 2H), 7.14 (d, *J* = 7.4 Hz, 1H), 2.41 (s, 3H), 2.38–2.35 (m, 2H); ^{13}C NMR (101 MHz, $CDCl_3$) δ = 141.3, 138.6, 138.4, 137.0,

129.6, 128.8, 127.9, 127.9, 127.1, 124.2, 21.7, 20.96 (t, J_{C-D} =20 Hz). HRMS (ESI^+): Calcd for $C_{14}H_{14}D$ $[M+H]^+$ 184.1237, Found:184.1236.

^{1}H NMR spectrum of 11-D (400 MHz, $CDCl_3$)

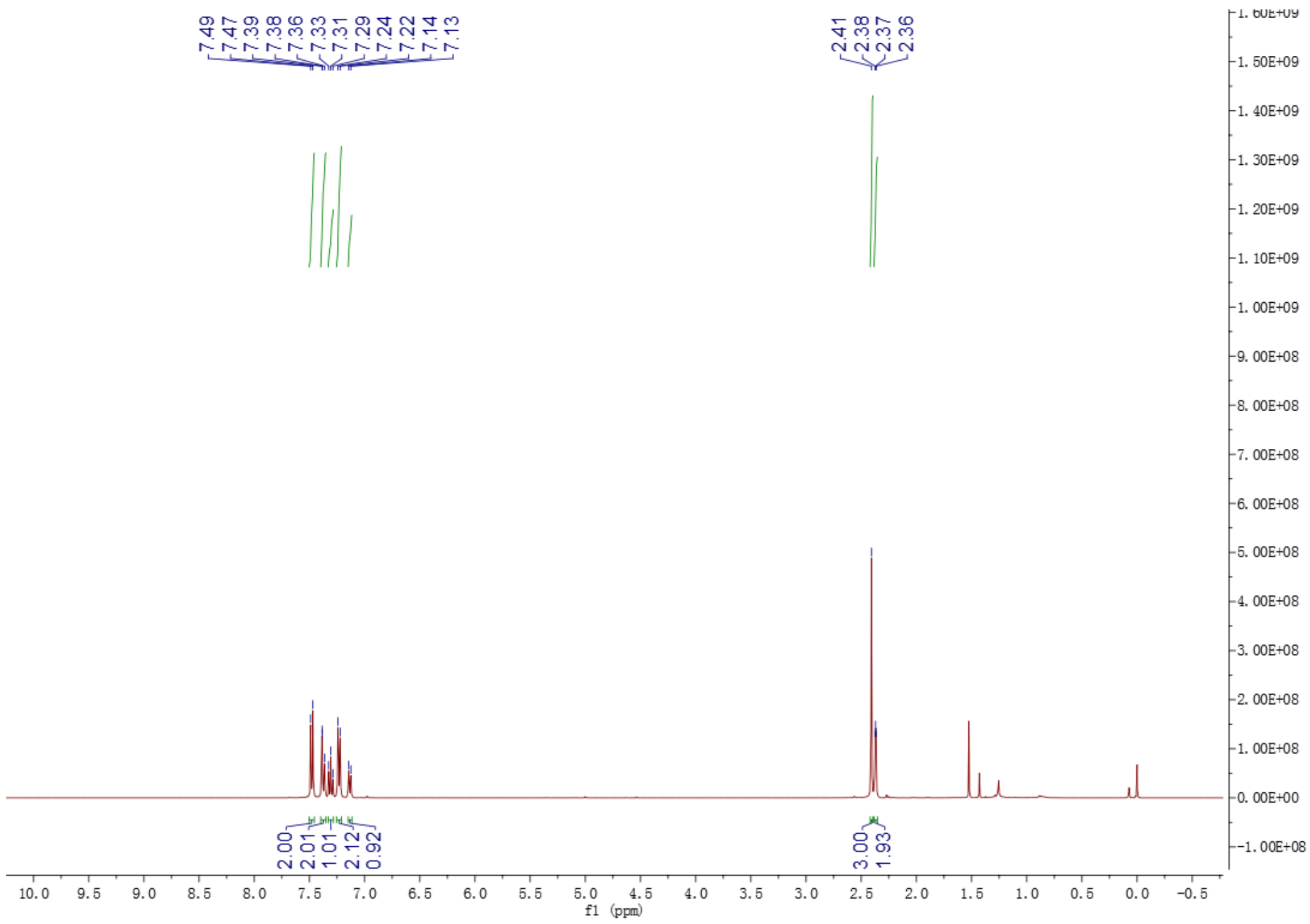

^{13}C NMR spectrum of 11-D (400 MHz, $CDCl_3$)

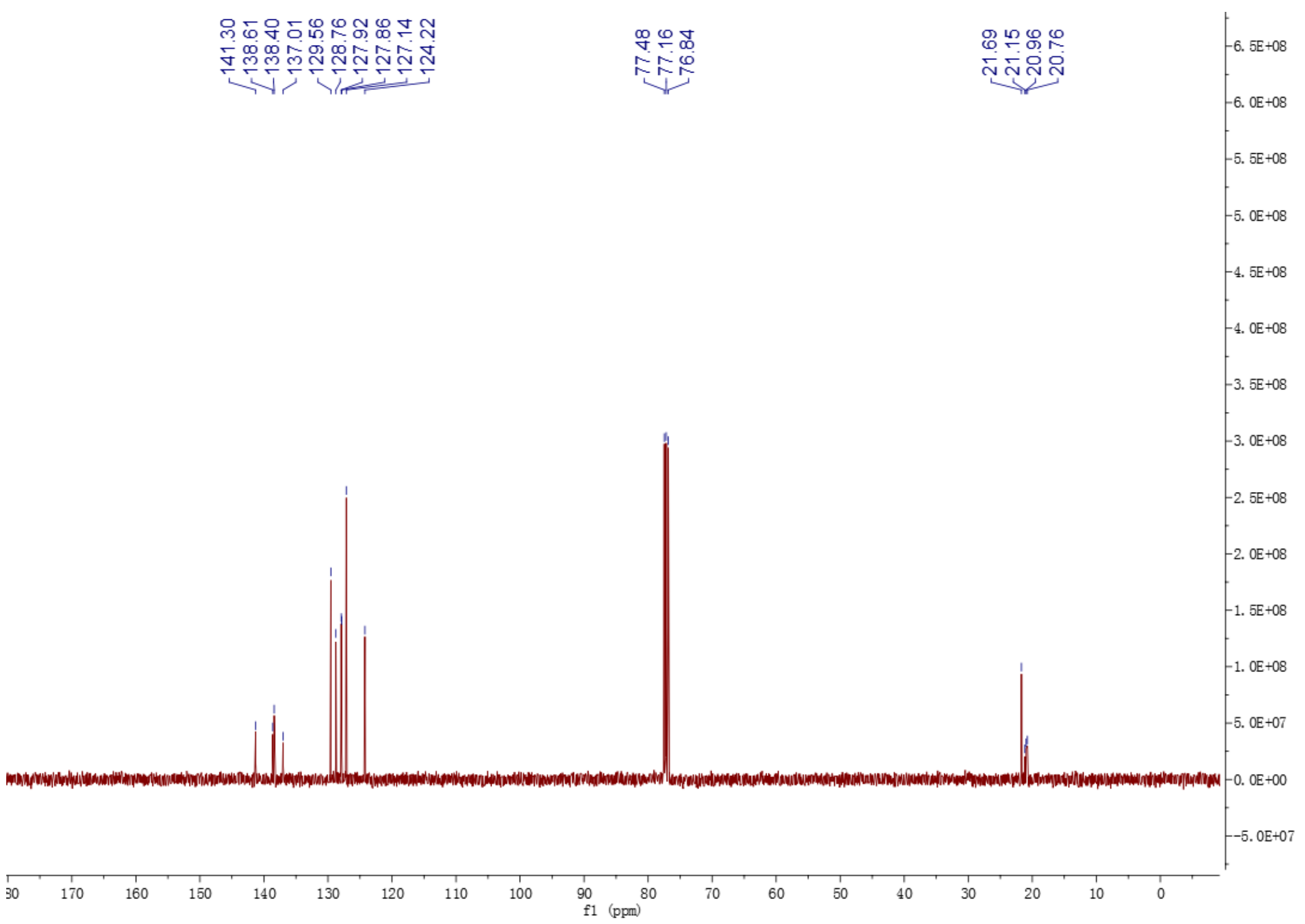

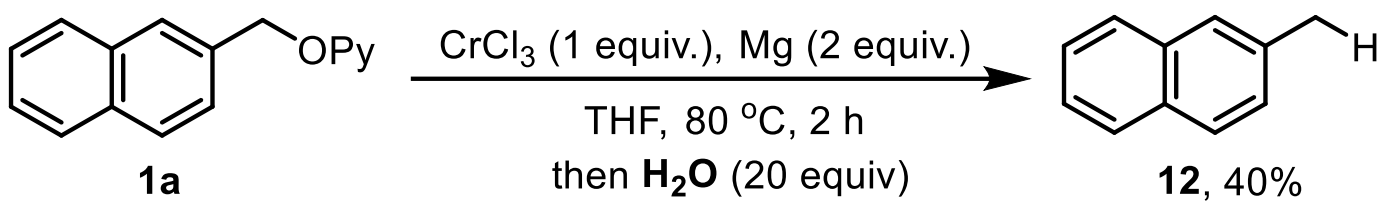

A dried Schlenk tube was charged with **1a** (0.2 mmol), $CrCl_3$ (0.2 mmol), Mg (0.4 mmol) followed by adding a freshly distilled THF (2 mL) by syringe under atmosphere of nitrogen. The reaction mixture was stirred at 80 °C for 12 h. After cooled room temperature, ethyl acetate (20 mL) was added into the reaction mixture and concentrated under vacuum. The residue was purified by flash chromatography on silica gel (petroleum ether) to give the desired product **12** (40%).

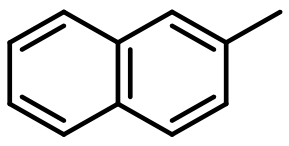

2-Methylnaphthalene (12)

^{1}H NMR (400 MHz, $CDCl_3$) δ = 7.80 (d, *J* = 7.8 Hz, 1H), 7.76 (dd, *J* = 8.1, 3.6 Hz, 2H), 7.62 (s, 1H), 7.47–7.38 (m, 2H), 7.32 (dd, *J* = 8.4, 1.5 Hz, 1H), 2.52 (s, 3H); ^{13}C NMR (101 MHz, $CDCl_3$) δ = 135.6, 133.8, 131.8, 128.3, 127.8, 127.7, 127.4, 127.0, 126.0, 125.1, 21.9. GC-MS Calcd for $C_{11}H_{10}$ $[M]^+$ 142.19, Found:142.19.

^{1}H NMR spectrum of 12 (400 MHz, $CDCl_3$)

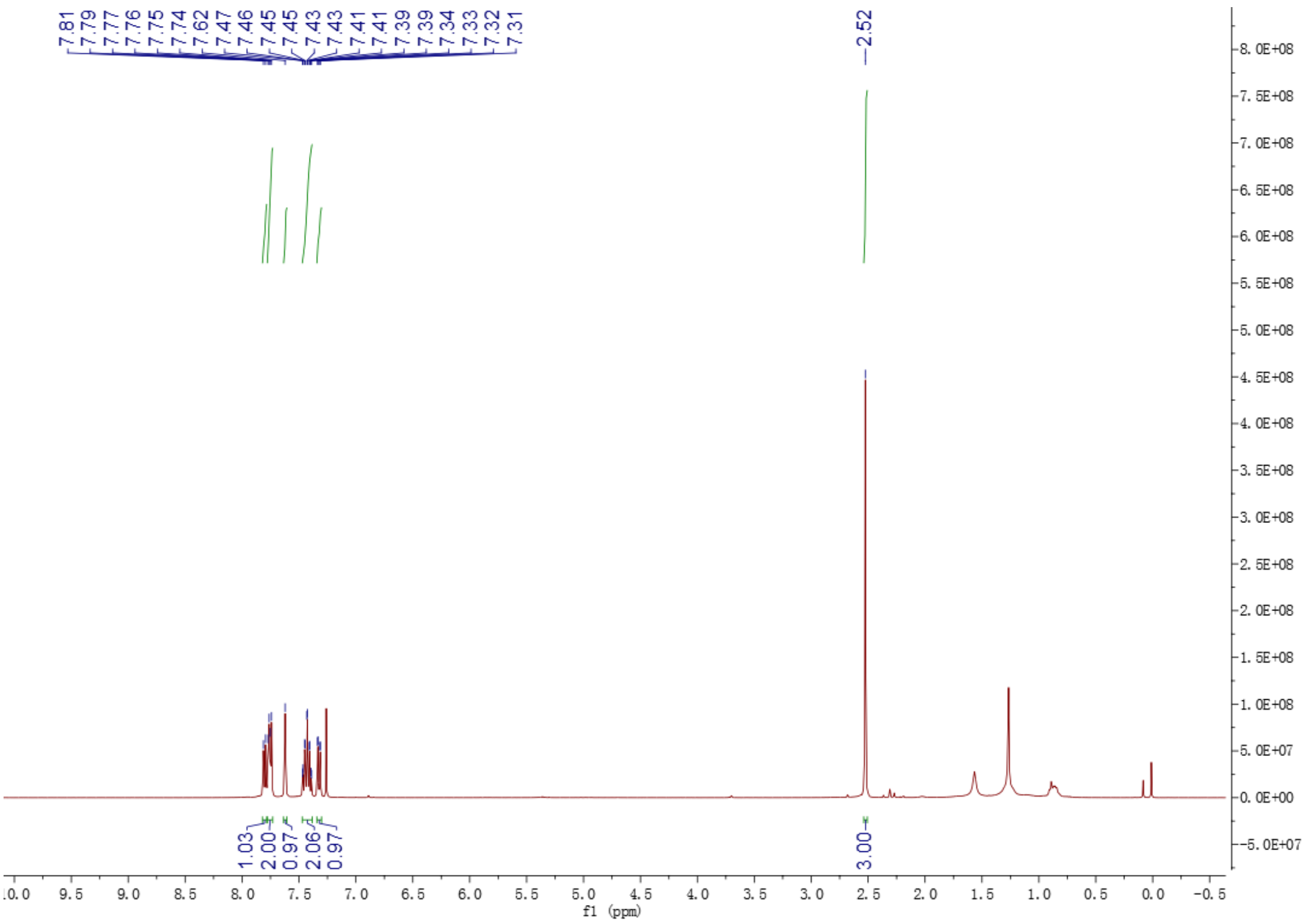

^{13}C NMR spectrum of 12 (400 MHz, $CDCl_3$)

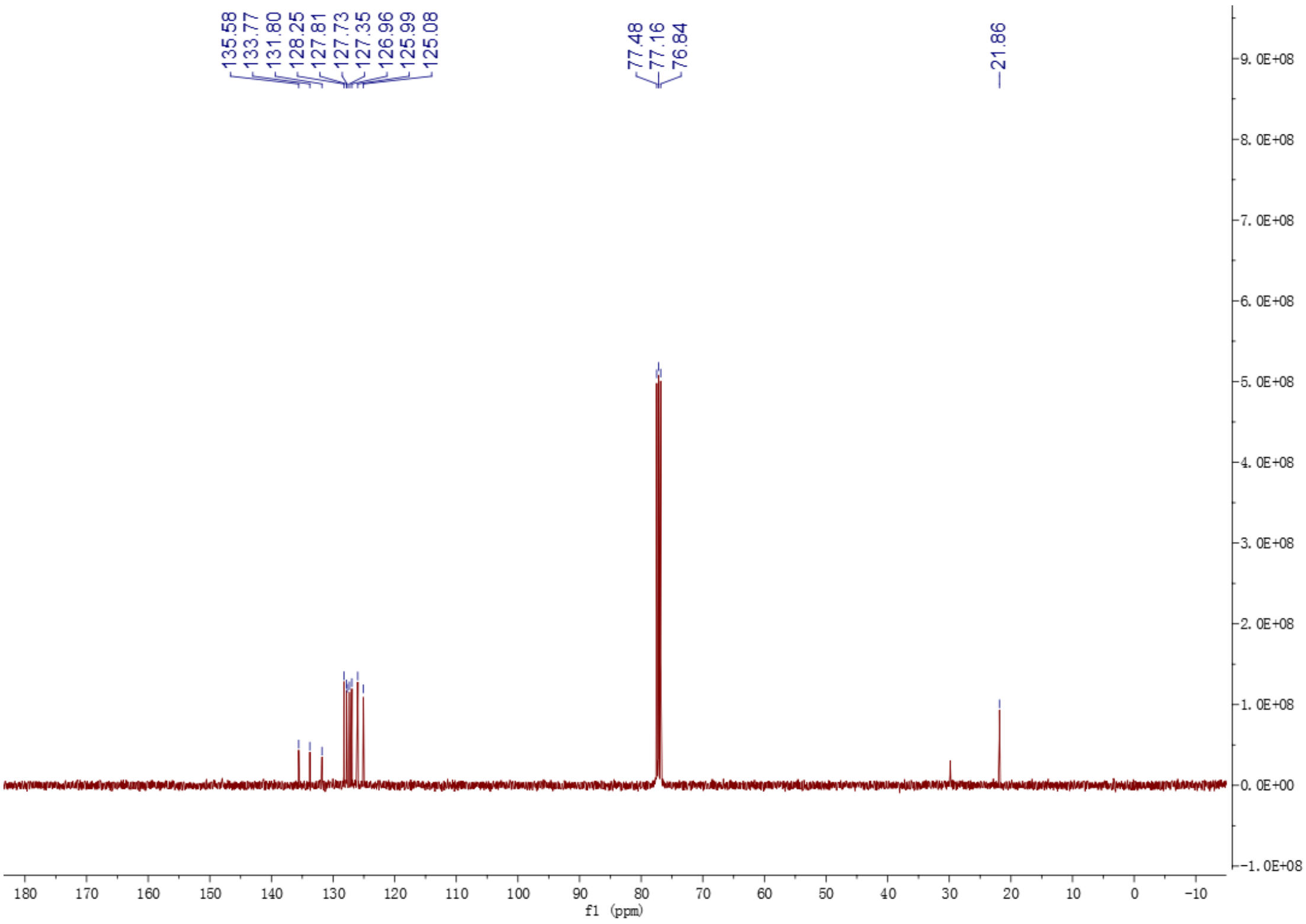

10.4 Investigating the formation of silyl magnesium or silyl chromium species

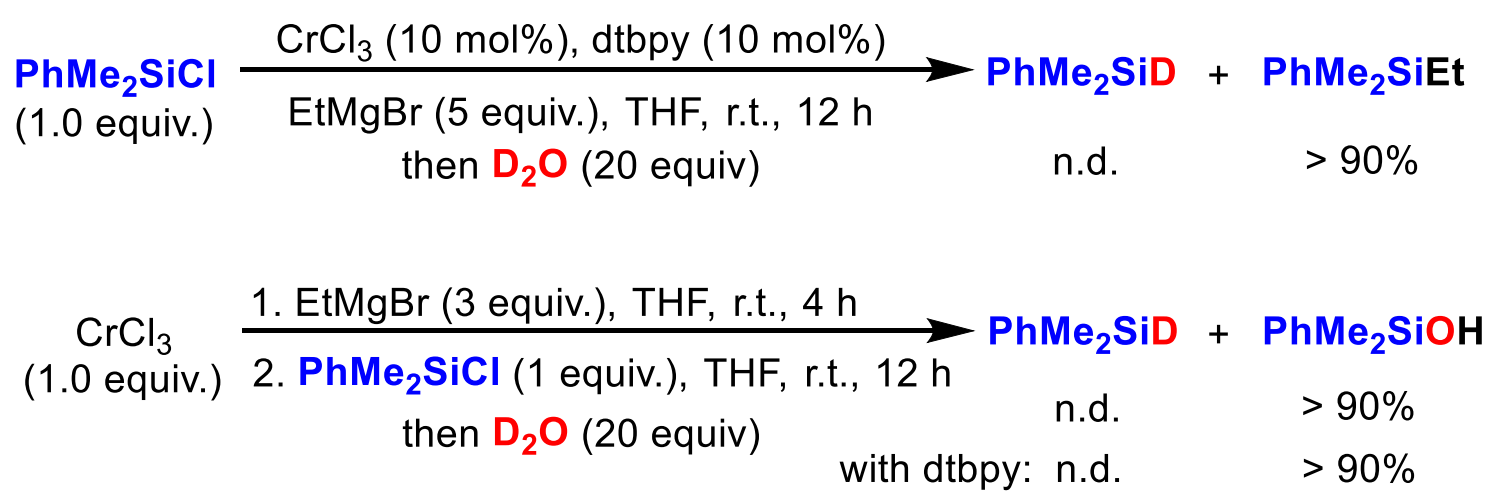

A dried Schlenk tube was charged with $PhMe_2SiCl$ (0.2 mmol), $CrCl_3$ (10 mol%), and dtbpy (10 mol%), followed by adding a freshly distilled THF (1 mL) by syringe under atmosphere of nitrogen. After stirring at room temperature for 5 min, EtMgBr (1.0 M in THF, 1.0 mmol) was added dropwise, and the reaction mixture was stirred at room temperature for 12 h. After quenched by D_2O (20 equiv.), the crude product was extracted with ethyl acetate (3 x 10 mL). The combined organic phases were dried over anhydrous Na_2SO_4 and concentrated under vacuum. The residue was monitored by NMR and purified by flash chromatography on silica gel (petroleum ether) to give the product $PhMe_2SiEt$. The deuterated dimethyl(phenyl)silane was not observed.

A dried Schlenk tube was charged with $CrCl_3$ (0.2) and THF (1 mL). After stirring

at room temperature for 5 min, EtMgBr (1.0 M in THF, 1.0 mmol) was added dropwise, and the reaction mixture was stirred at room temperature for 4 h. Then $PhMe_2SiCl$ (0.2 mmol) and dtbpy (0.2 mmol) were added and the reaction mixture was stirred at room temperature for 12 h. After quenched by D_2O (20 equiv.), the crude product was extracted with ethyl acetate (3 x 10 mL). The combined organic phases were dried over anhydrous Na_2SO_4 and concentrated under vacuum. The residue was monitored by NMR and purified by flash chromatography on silica gel to give the product $PhMe_2SiEt$ generated from the protonolysis of $PhMe_2SiCl$. The deuterated dimethyl(phenyl)silane was not observed. The same result was observed in the absence of dtbpy.

10.5 Possible reaction mechanisms for the deoxygenative silylation and borylation of methyl ether 1a'

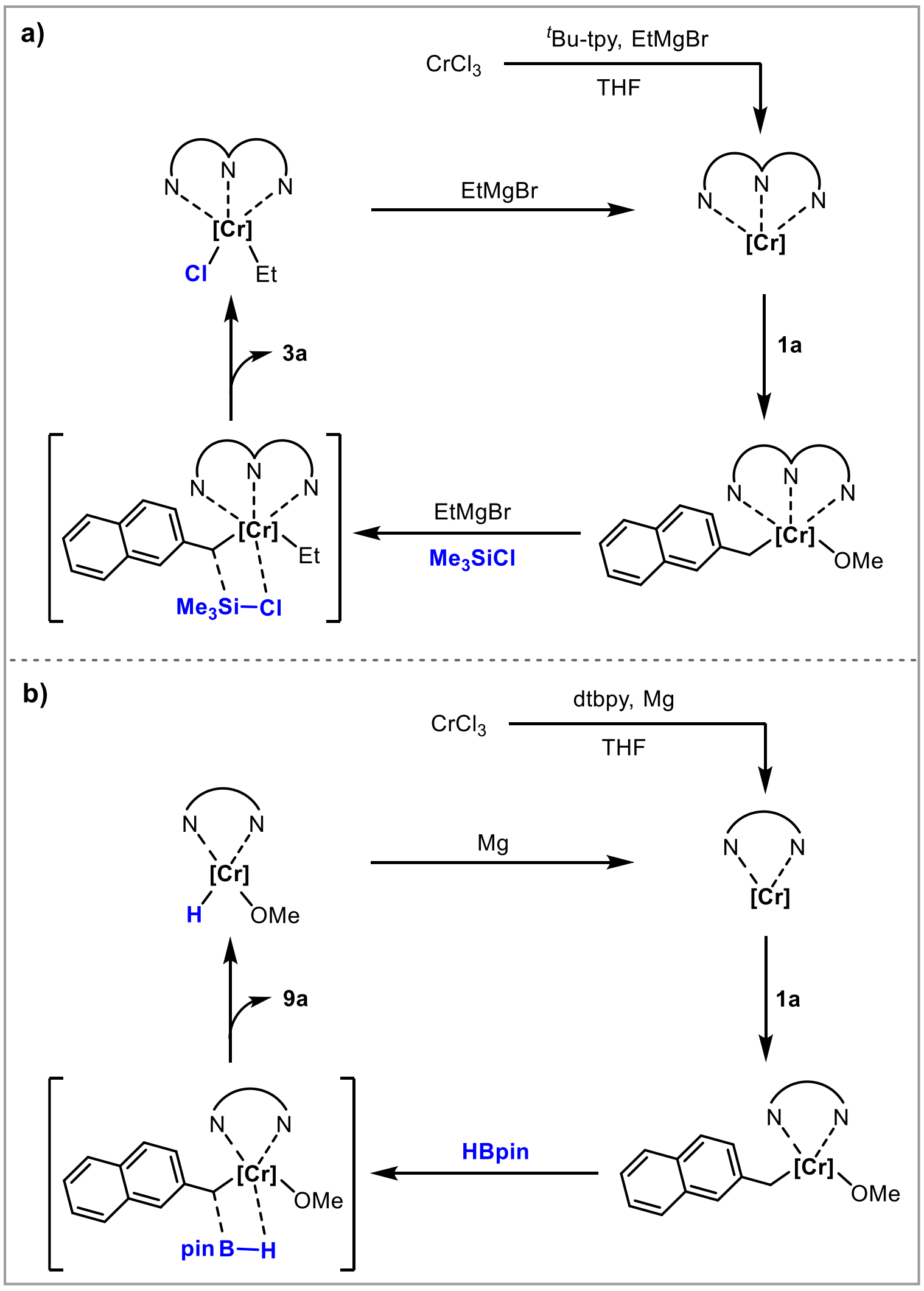

Figure S2. a) Possible mechanism for the deoxygenative silylation of methyl ether **1a'** with **2a**; b) Possible mechanism for the deoxyborylation of methyl ether **1a'** with HBpin.

11. Copies of ^{1}H, and ^{13}C NMR Spectra

^{1}H NMR spectrum (400 MHz, $CDCl_3$)

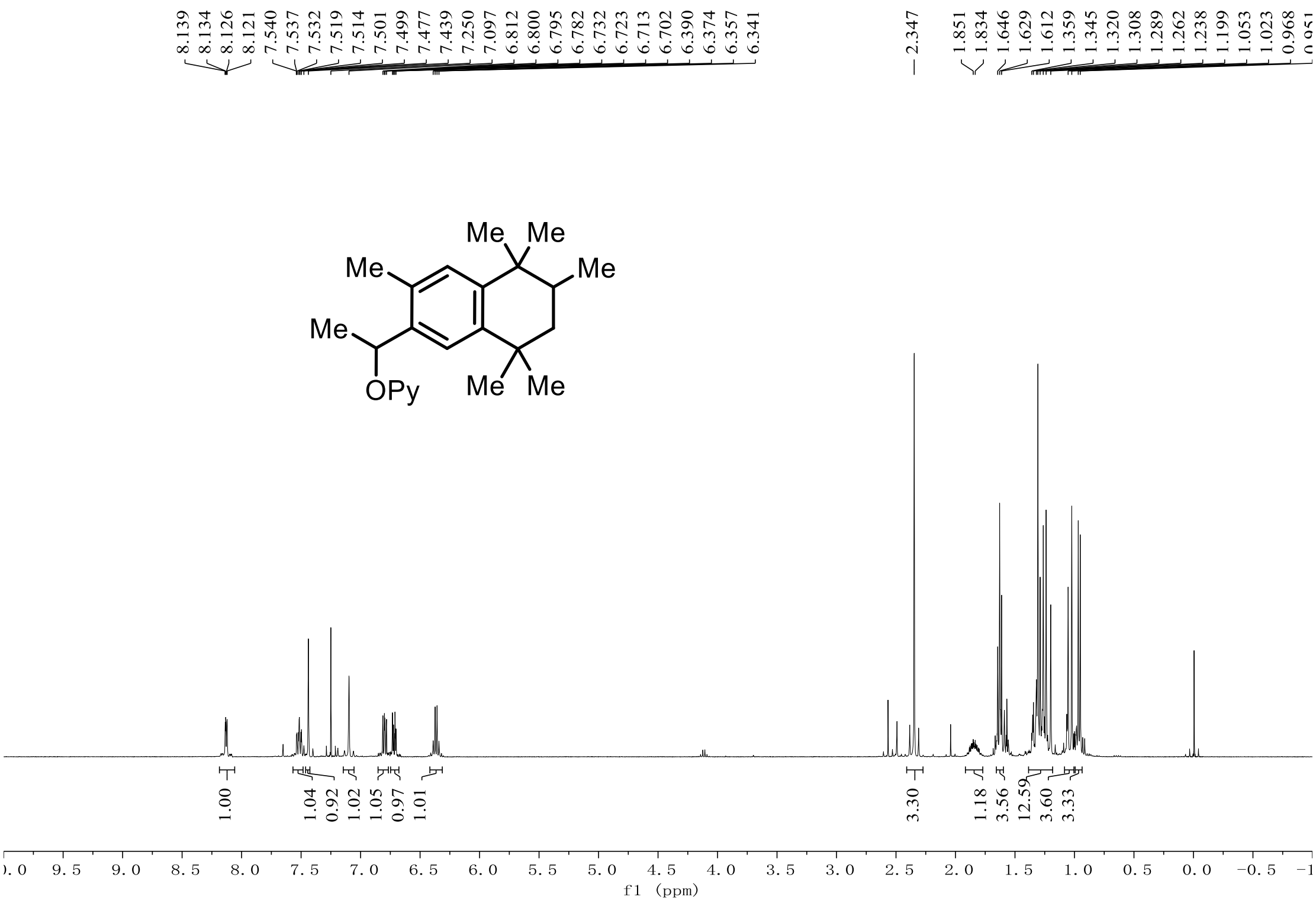

^{13}C NMR spectrum (101 MHz, $CDCl_3$)

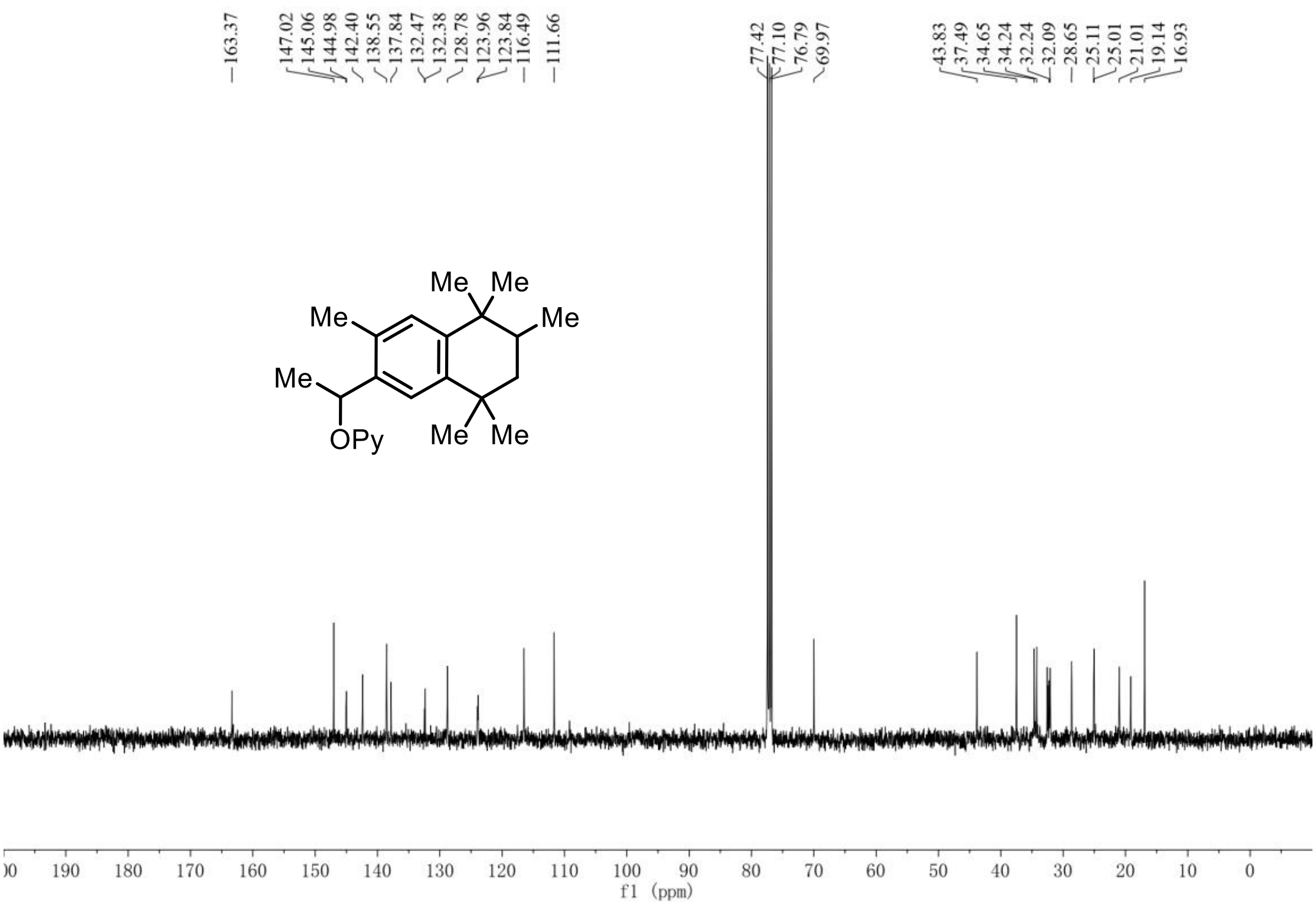

^{1}H NMR spectrum (400 MHz, $CDCl_3$)

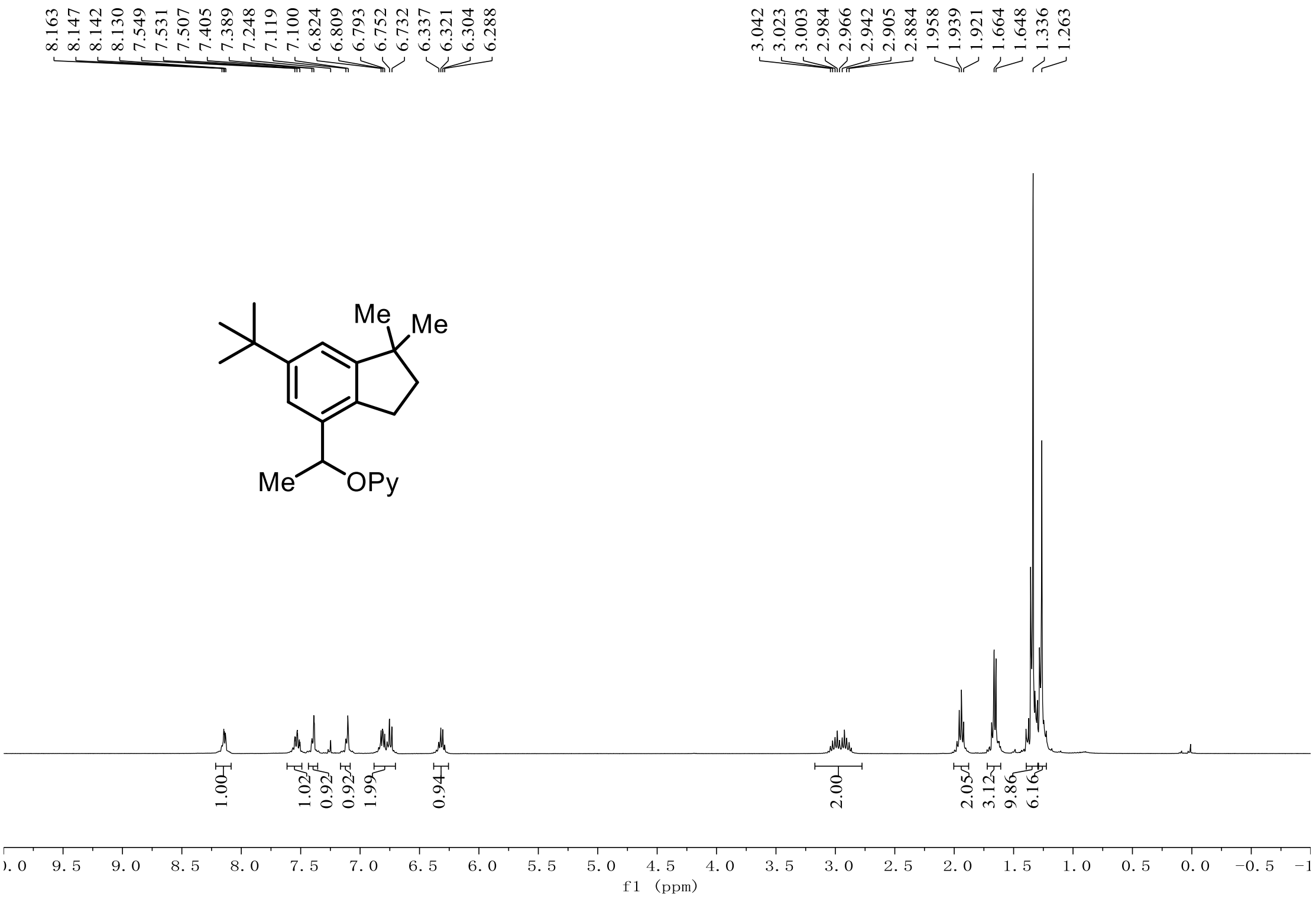

^{13}C NMR spectrum (101 MHz, $CDCl_3$)

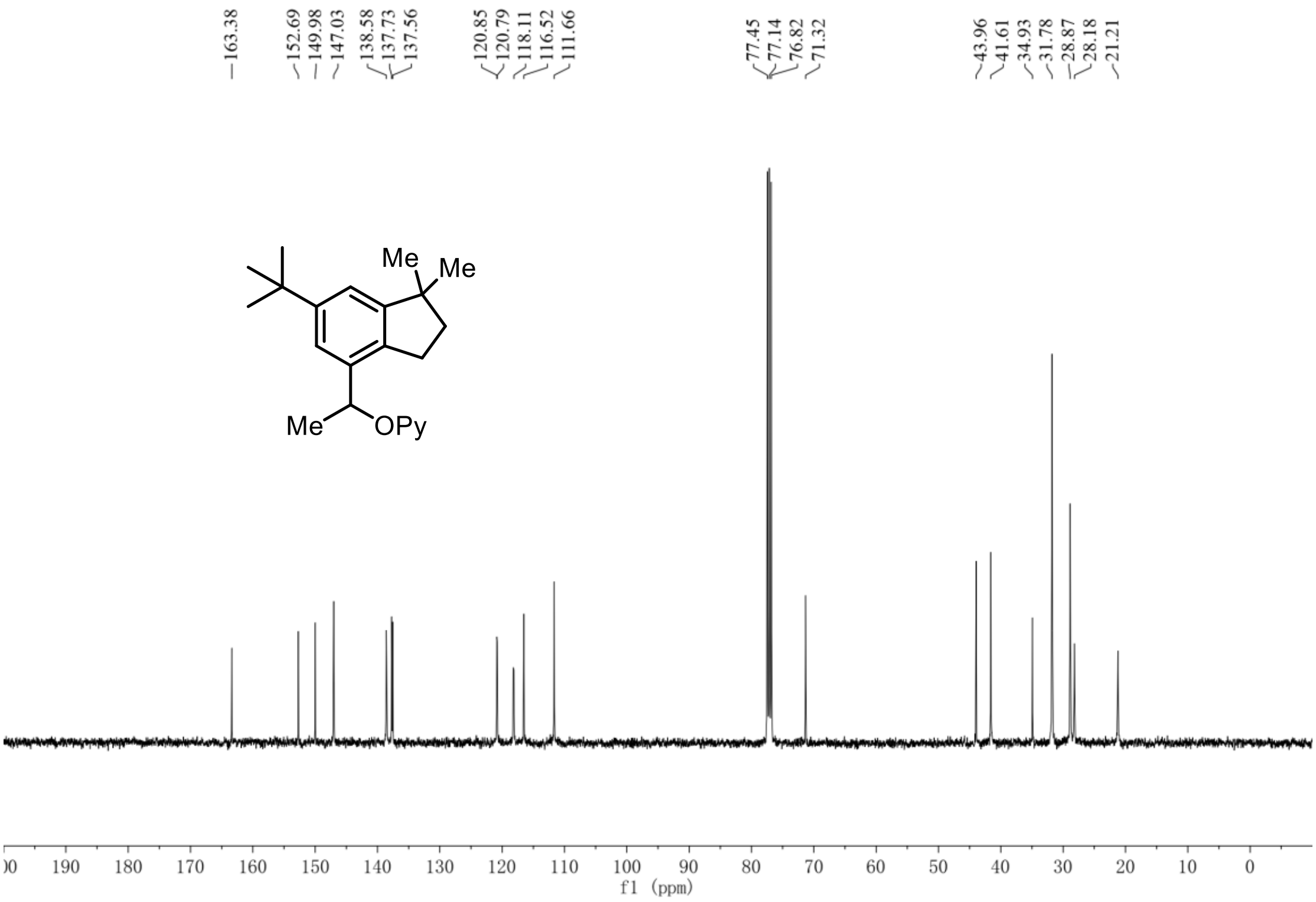

^{1}H NMR spectrum (400 MHz, $CDCl_3$)

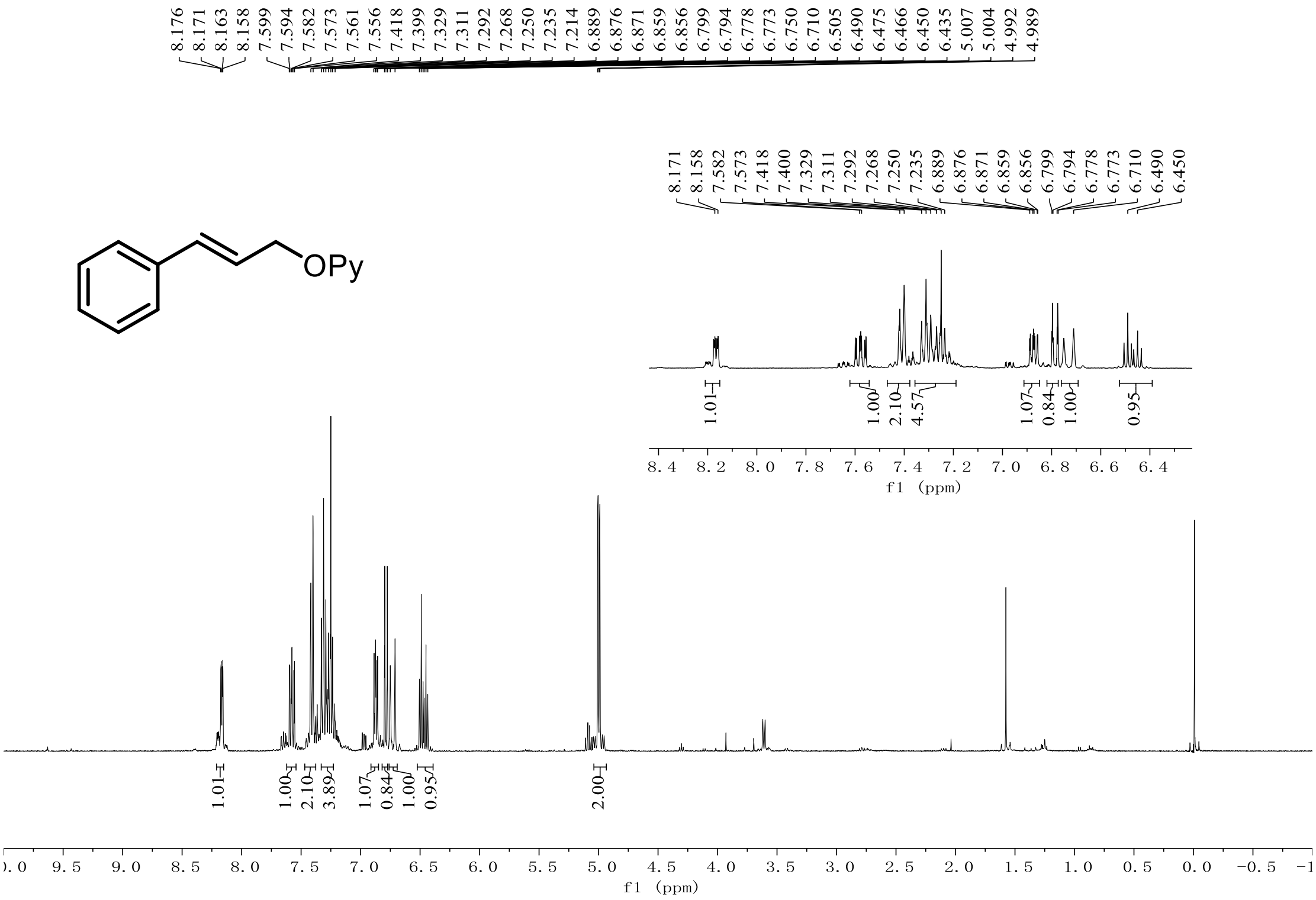

^{13}C NMR spectrum (101 MHz, $CDCl_3$)

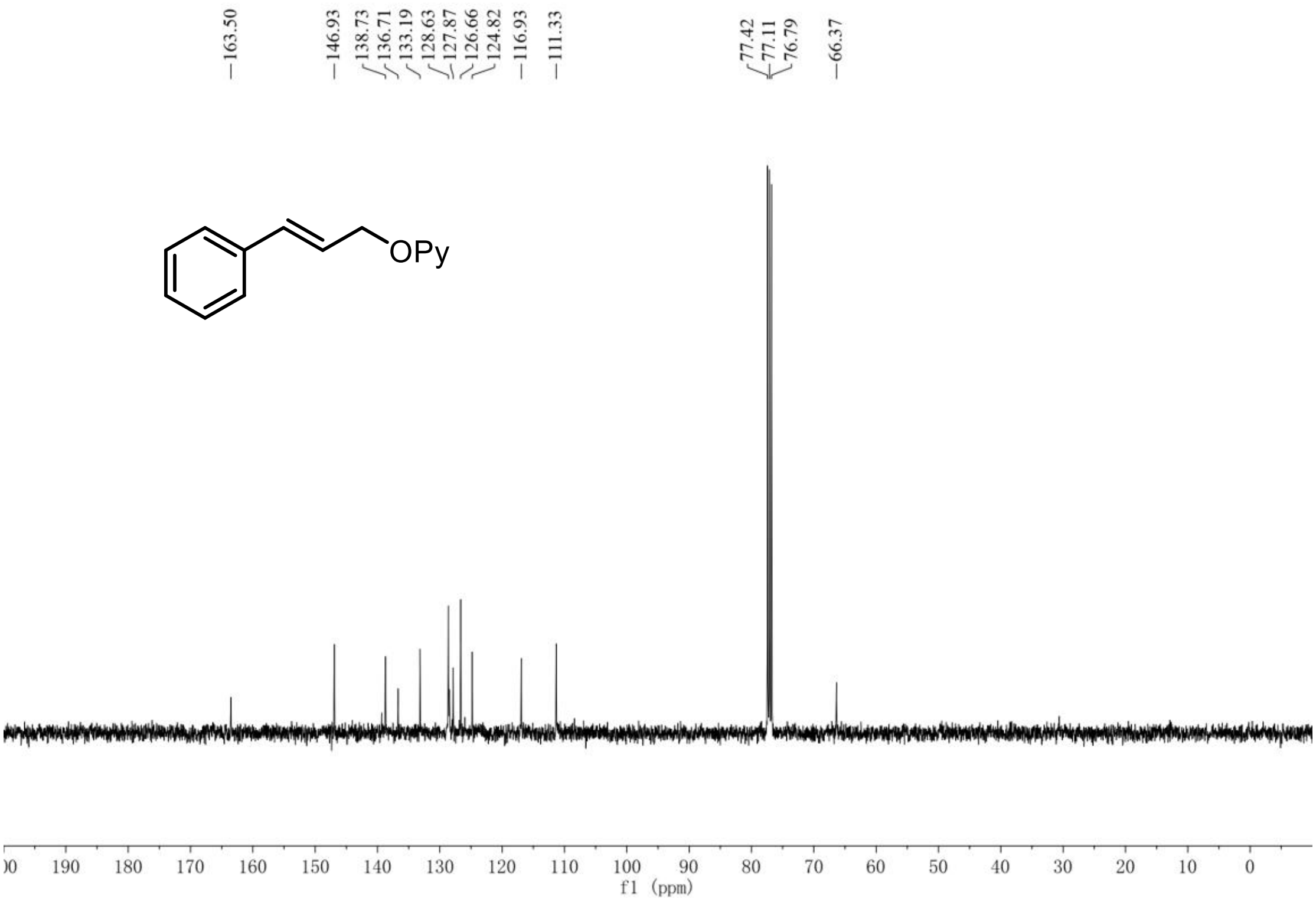

^{1}H NMR spectrum (400 MHz, $CDCl_3$)

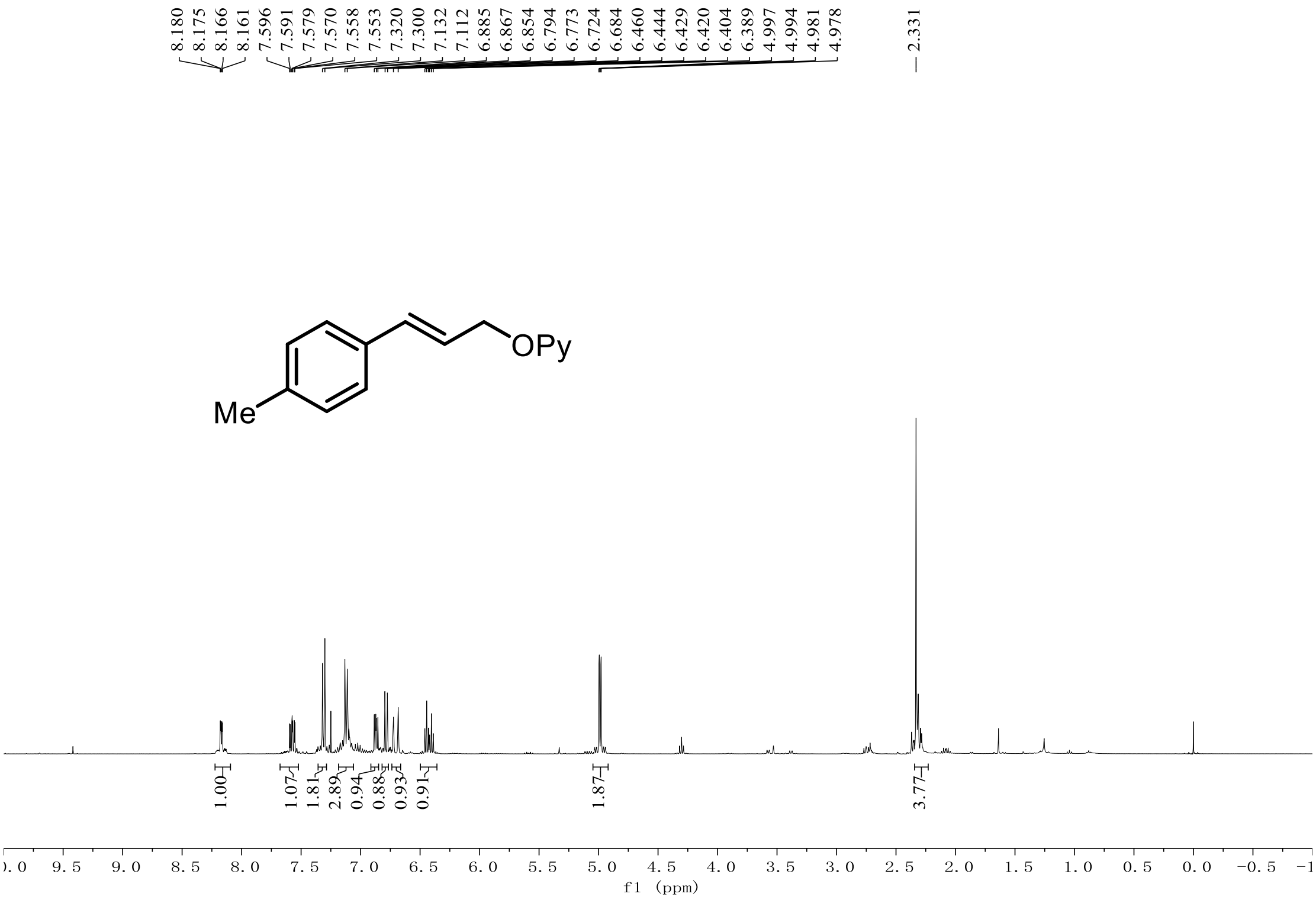

^{13}C NMR spectrum (101 MHz, $CDCl_3$)

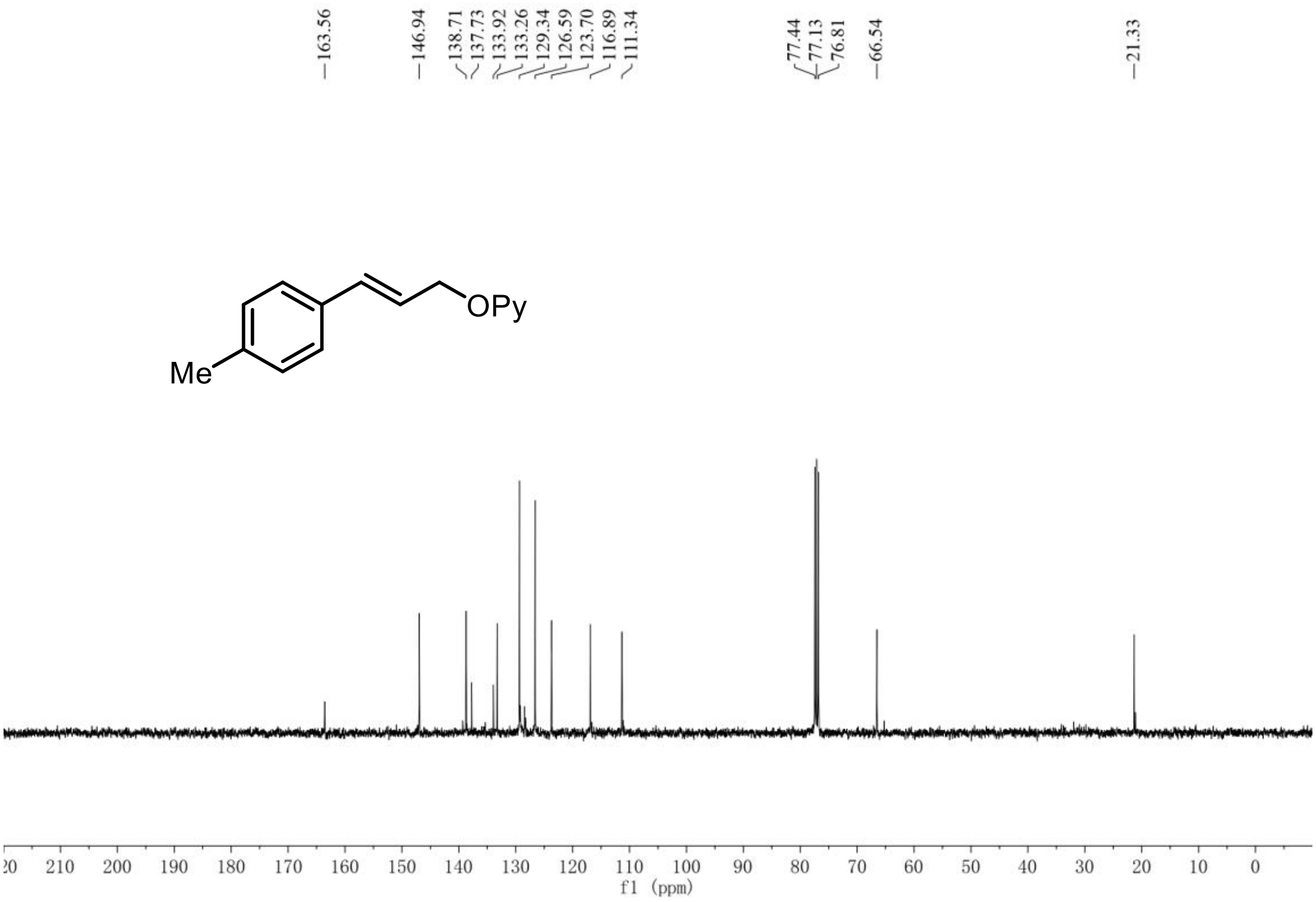

^{1}H NMR spectrum (400 MHz, $CDCl_3$)

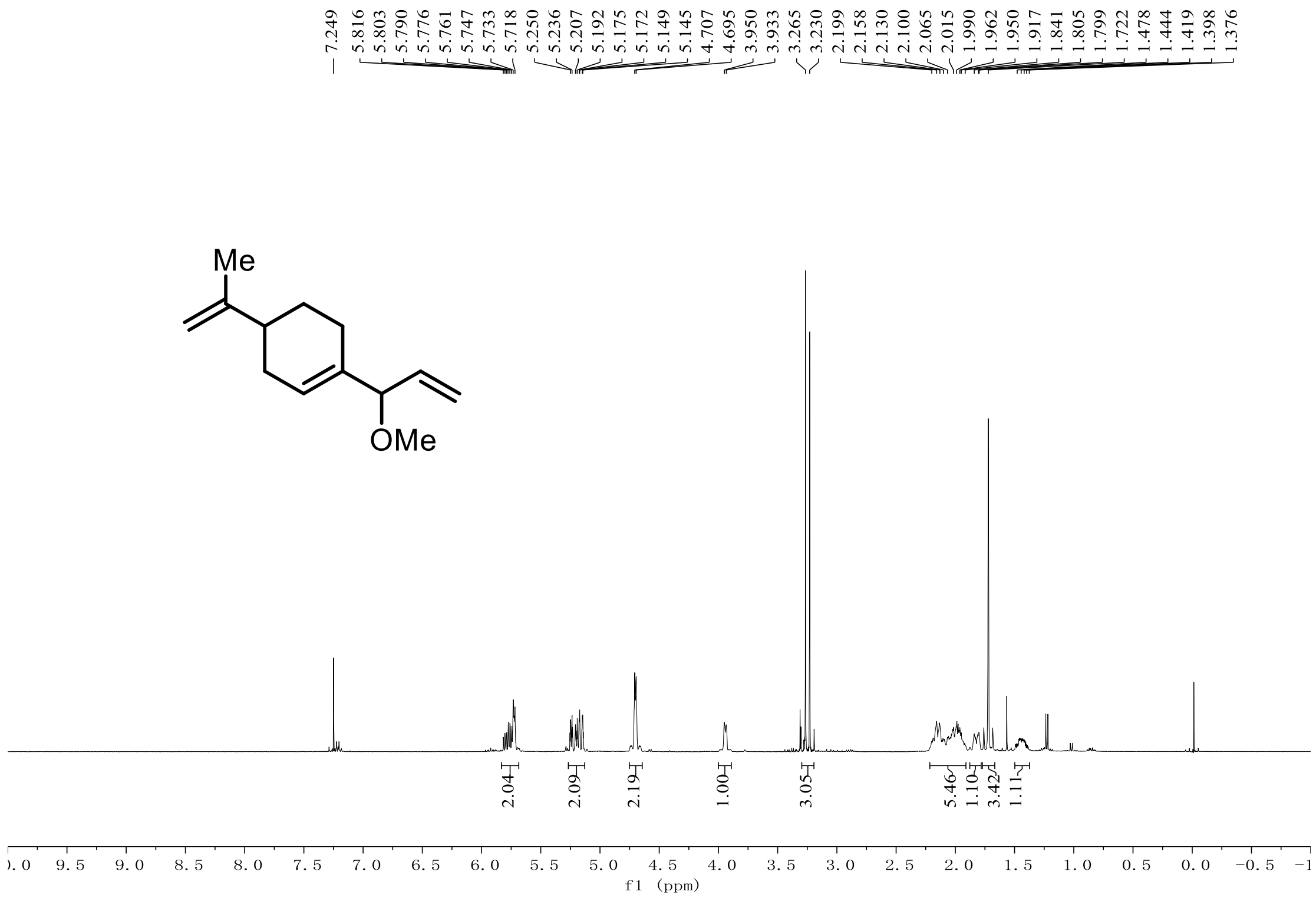

^{13}C NMR spectrum (101 MHz, $CDCl_3$)

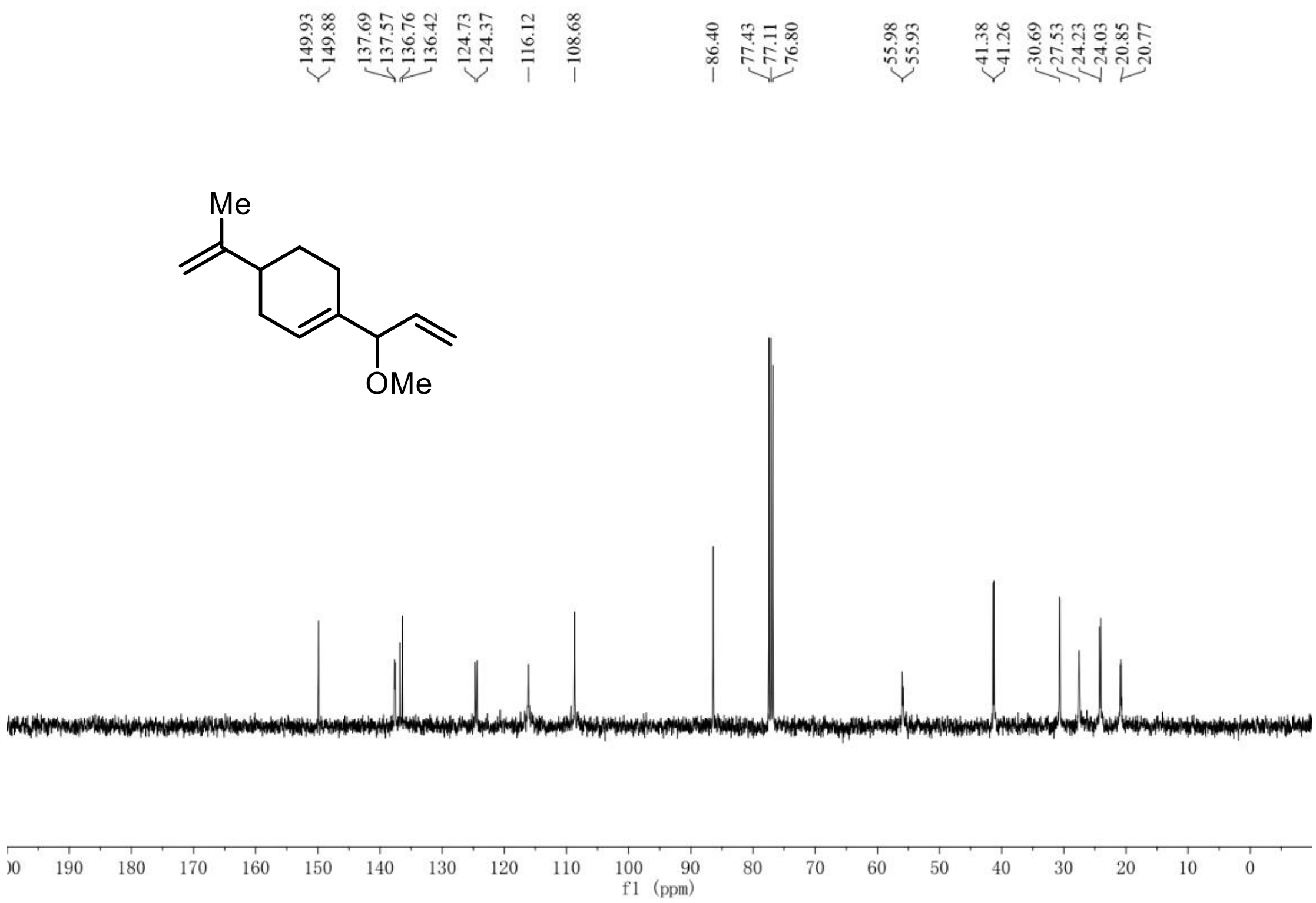

^{1}H NMR spectrum (400 MHz, $CDCl_3$)

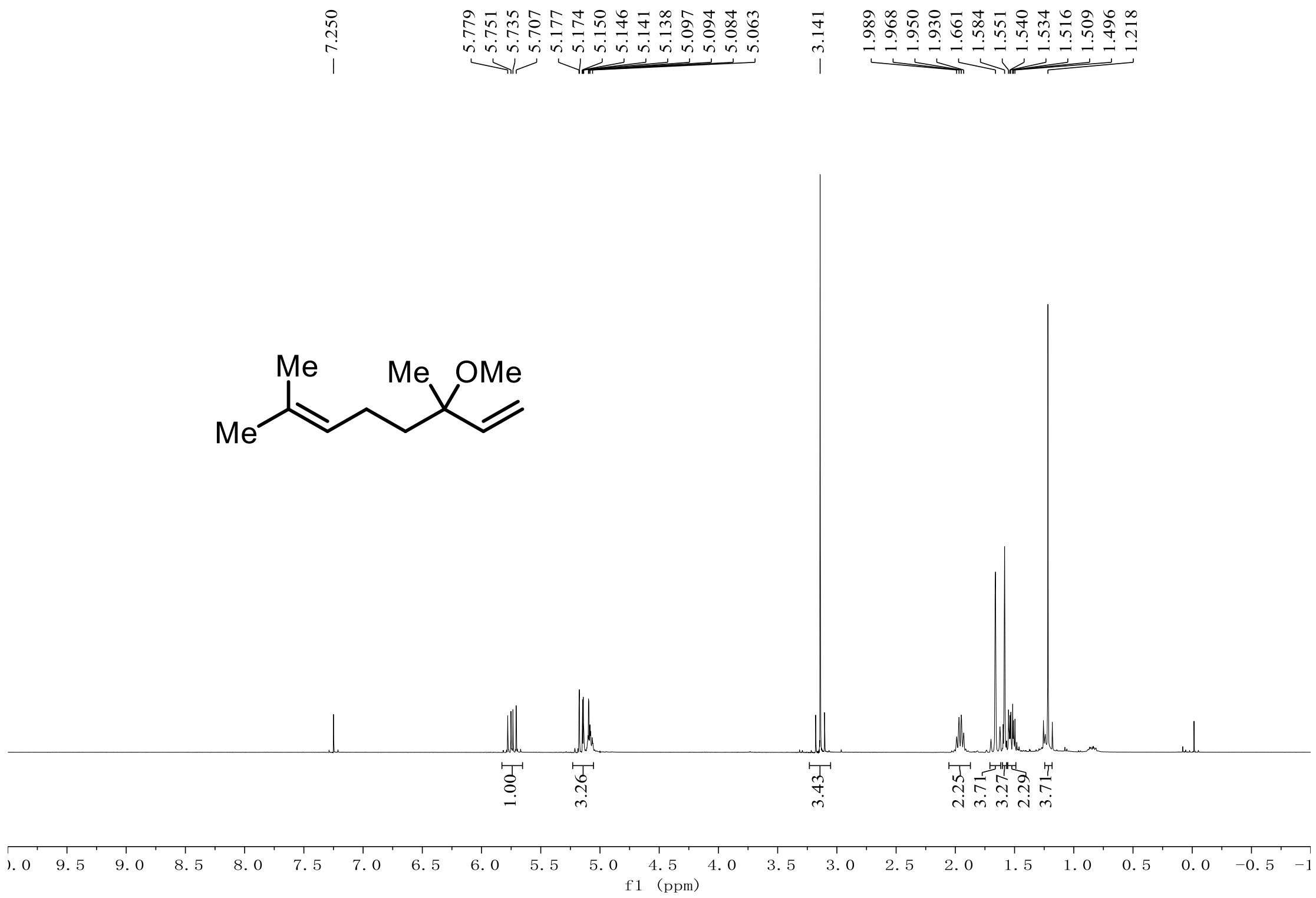

^{13}C NMR spectrum (101 MHz, $CDCl_3$)

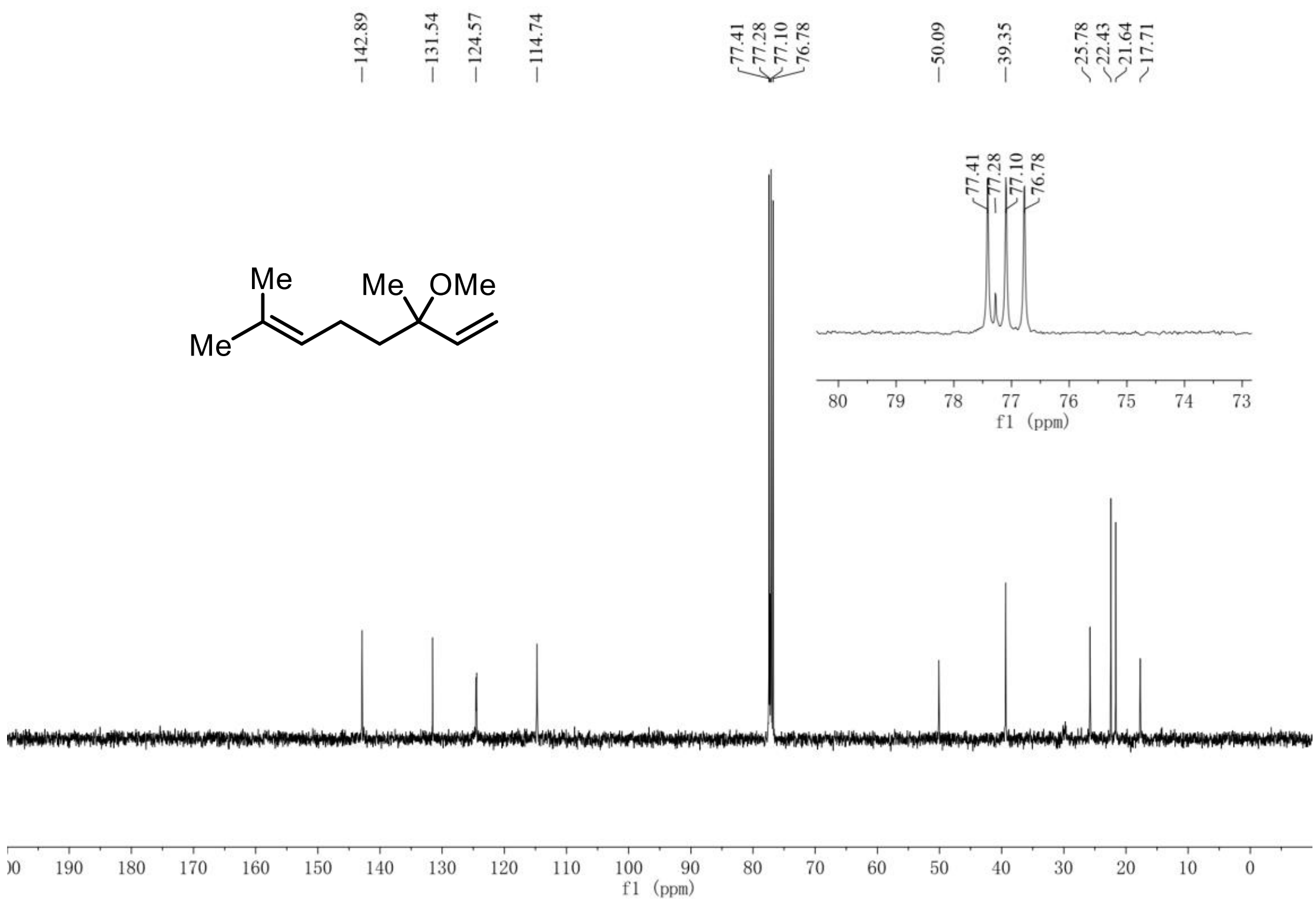

^{1}H NMR spectrum (400 MHz, $CDCl_3$)

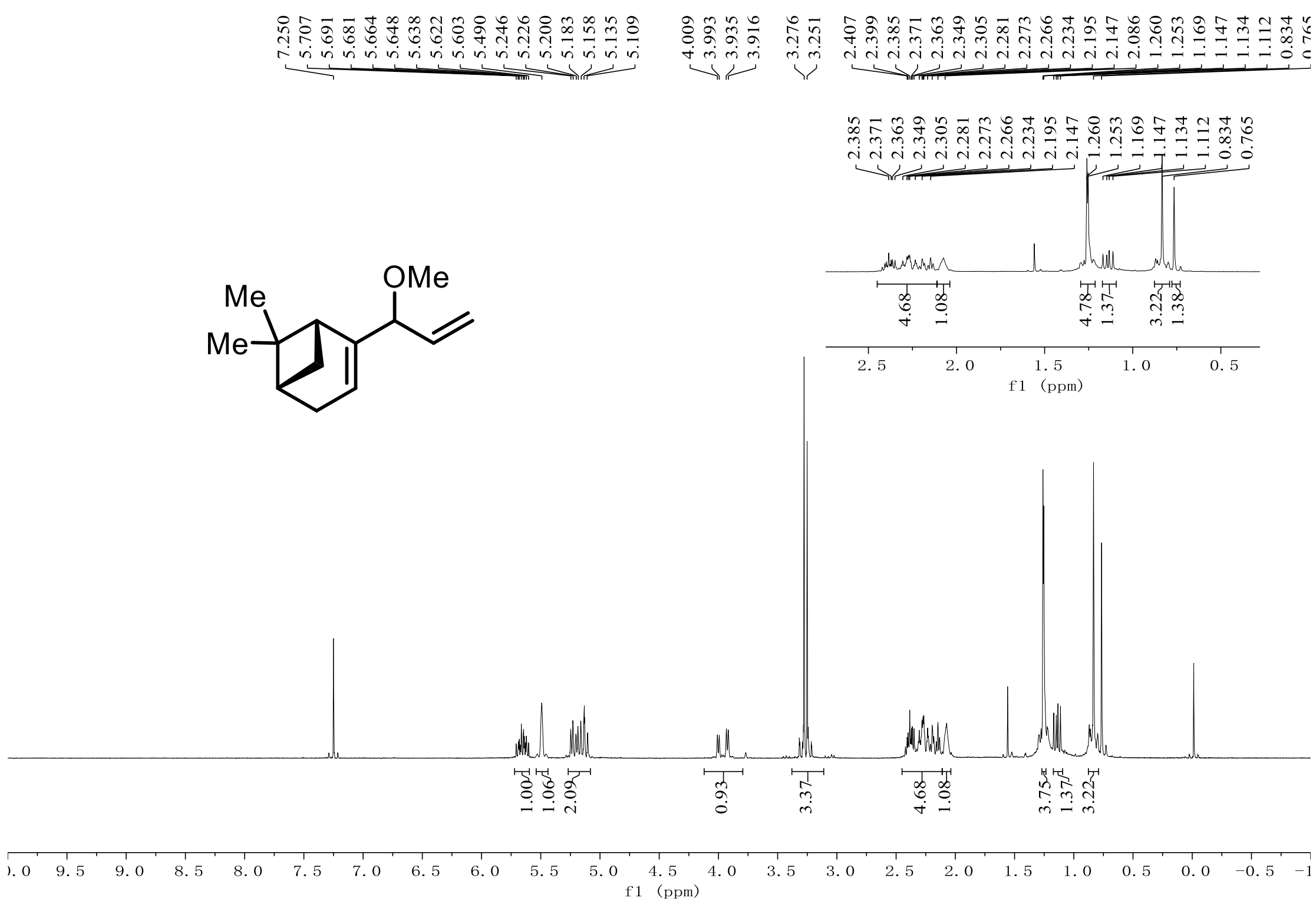

^{13}C NMR spectrum (101 MHz, $CDCl_3$)

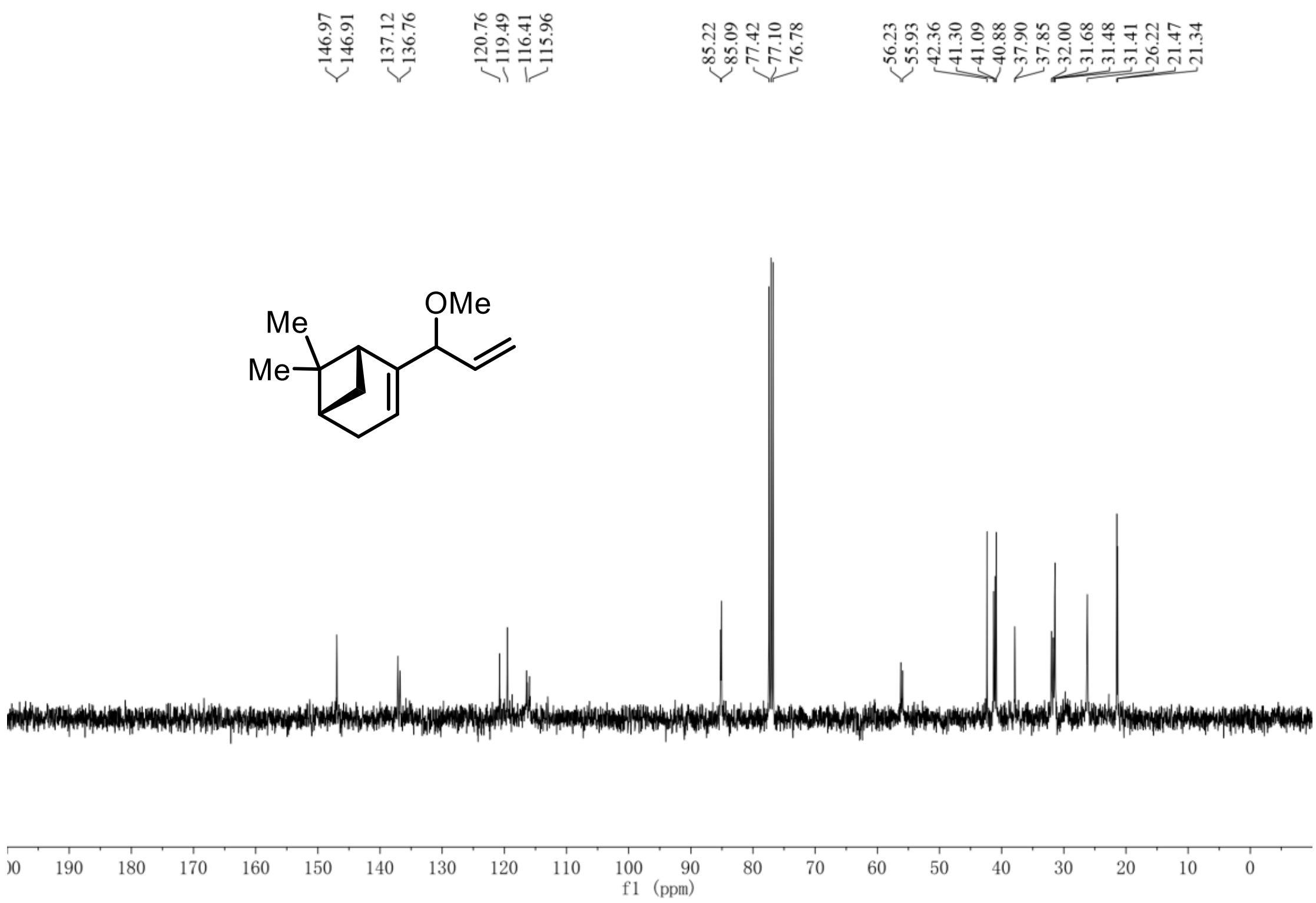

^{1}H NMR spectrum (400 MHz, $CDCl_3$)

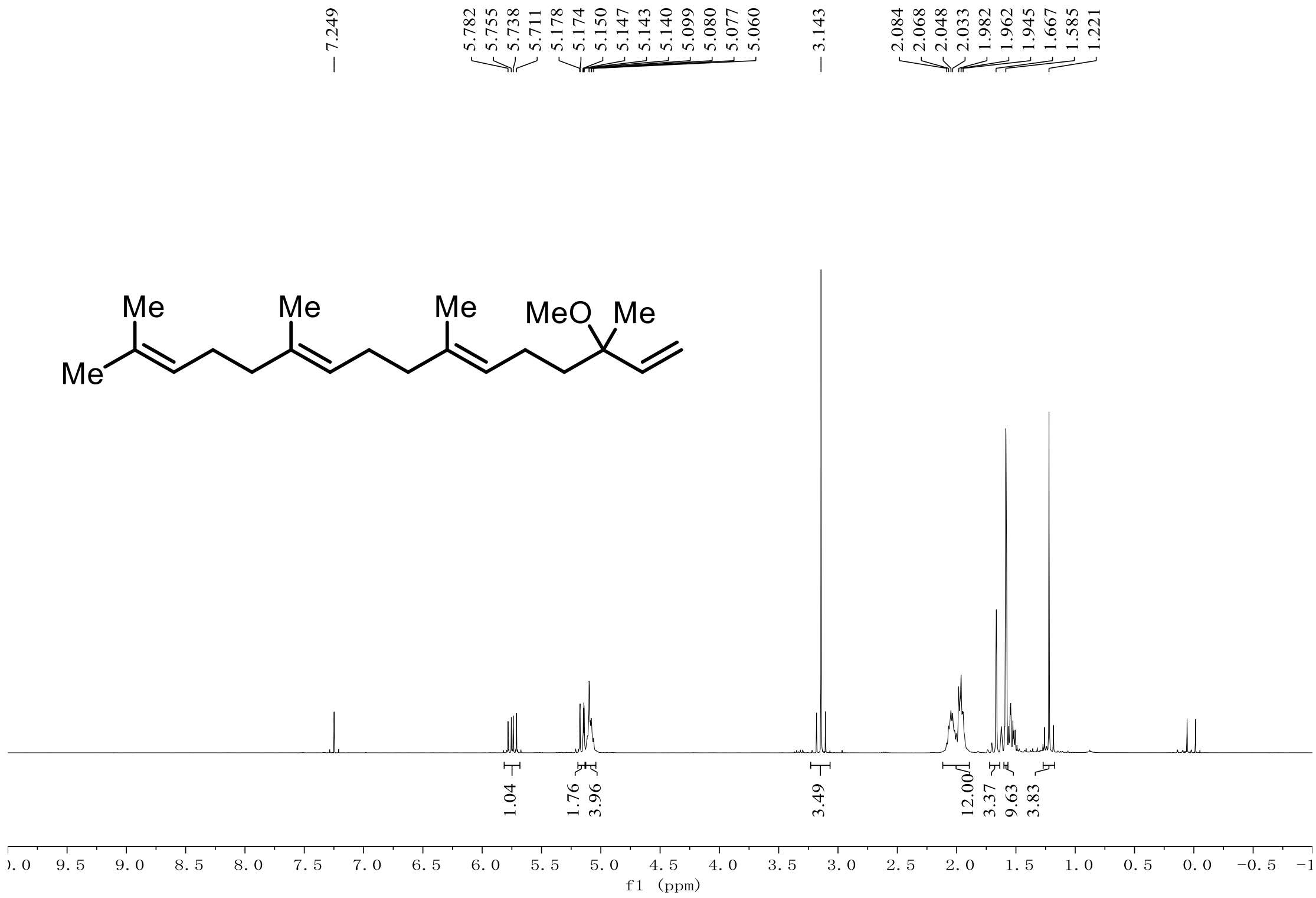

^{13}C NMR spectrum (101 MHz, $CDCl_3$)

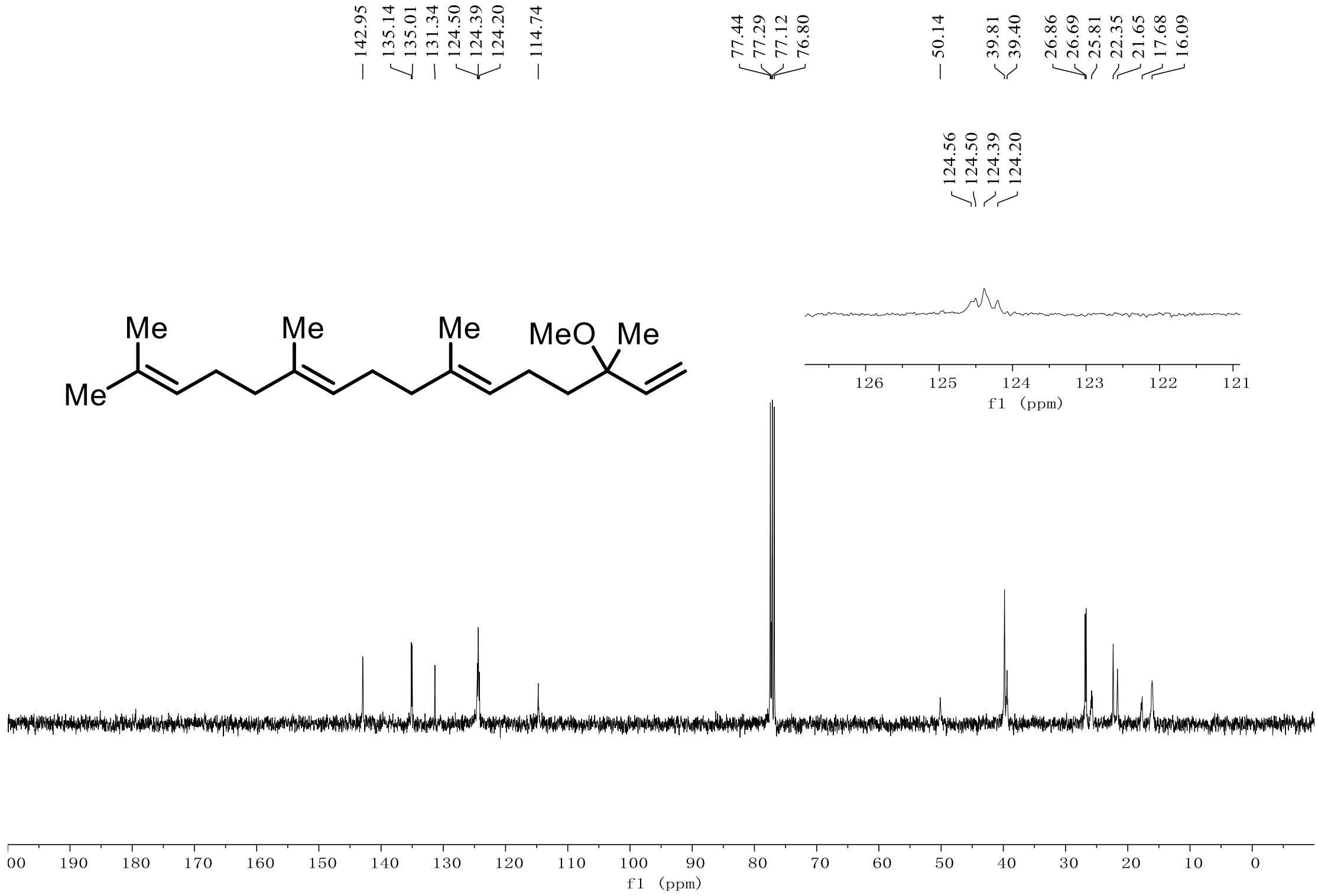

^{1}H NMR spectrum (400 MHz, $CDCl_3$)

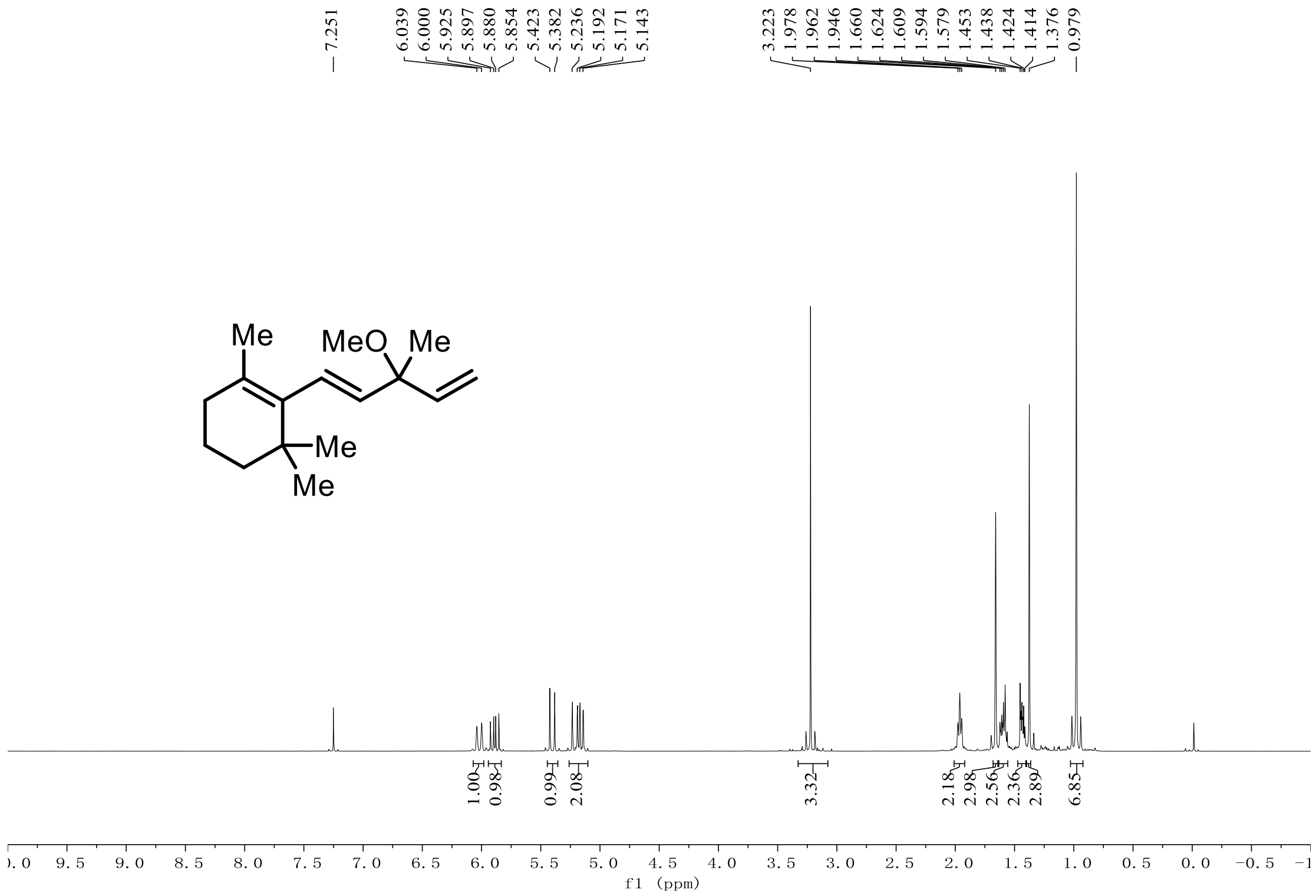

^{13}C NMR spectrum (101 MHz, $CDCl_3$)

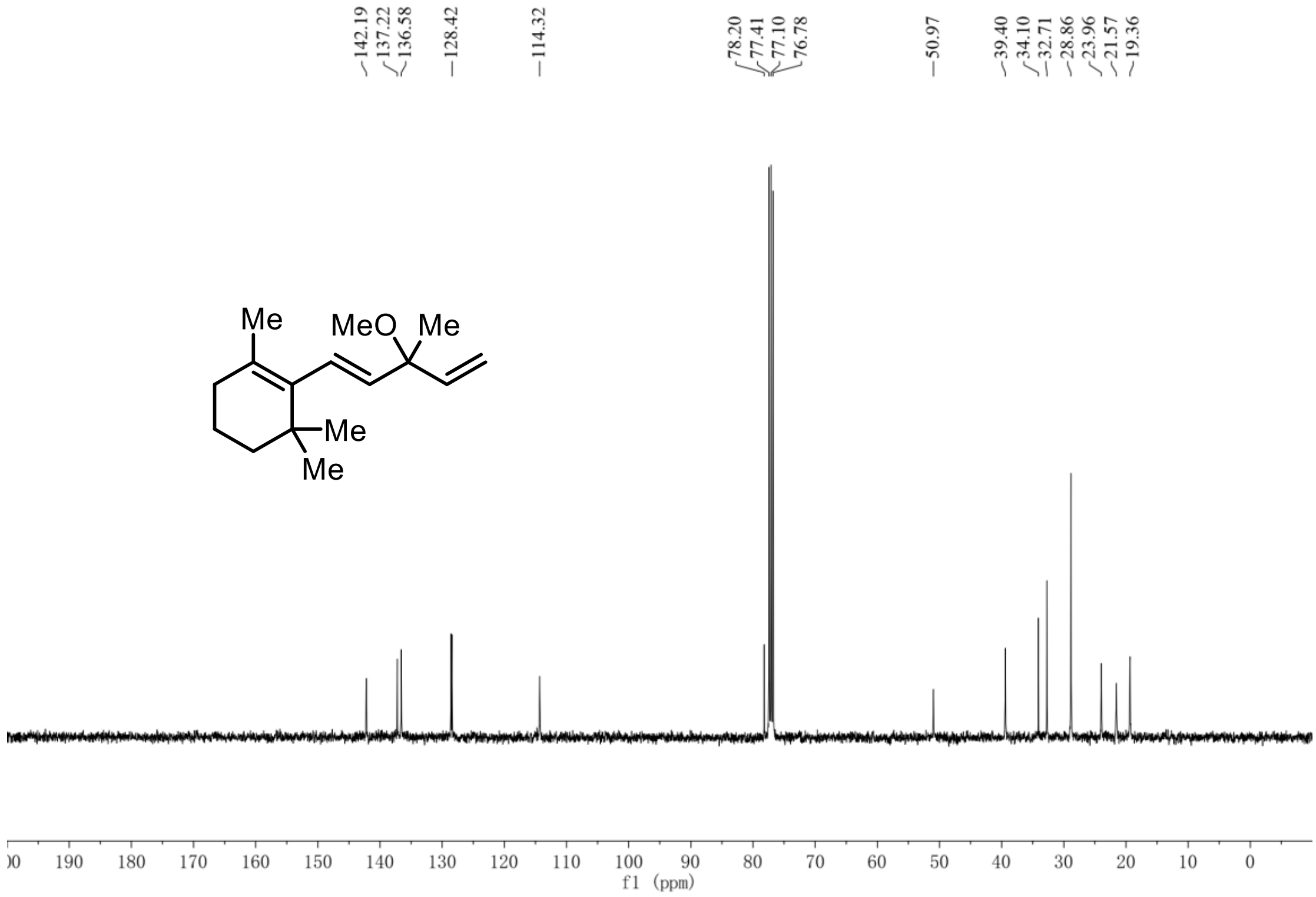

^{1}H NMR spectrum (400 MHz, $CDCl_3$)

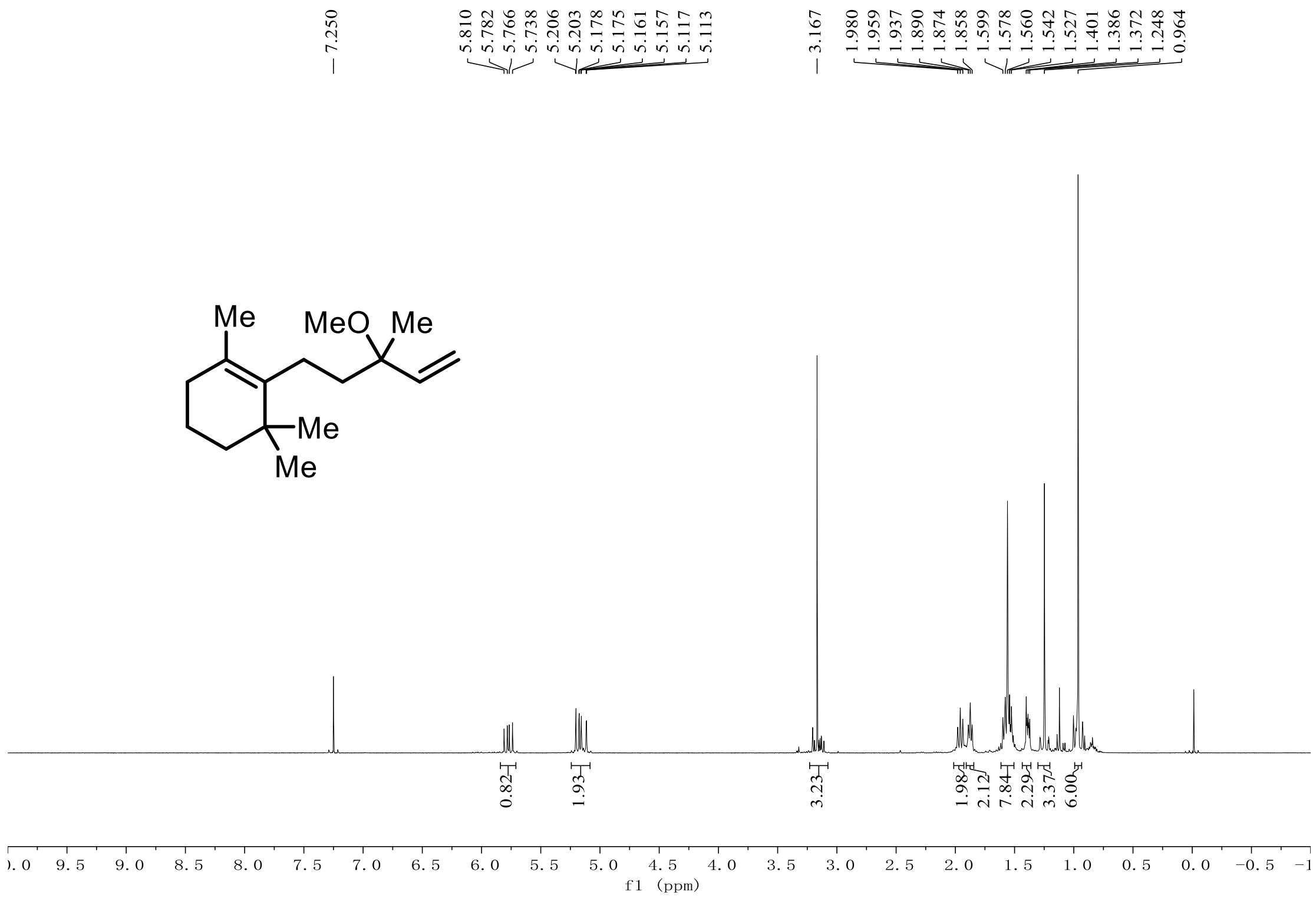

^{13}C NMR spectrum (101 MHz, $CDCl_3$)

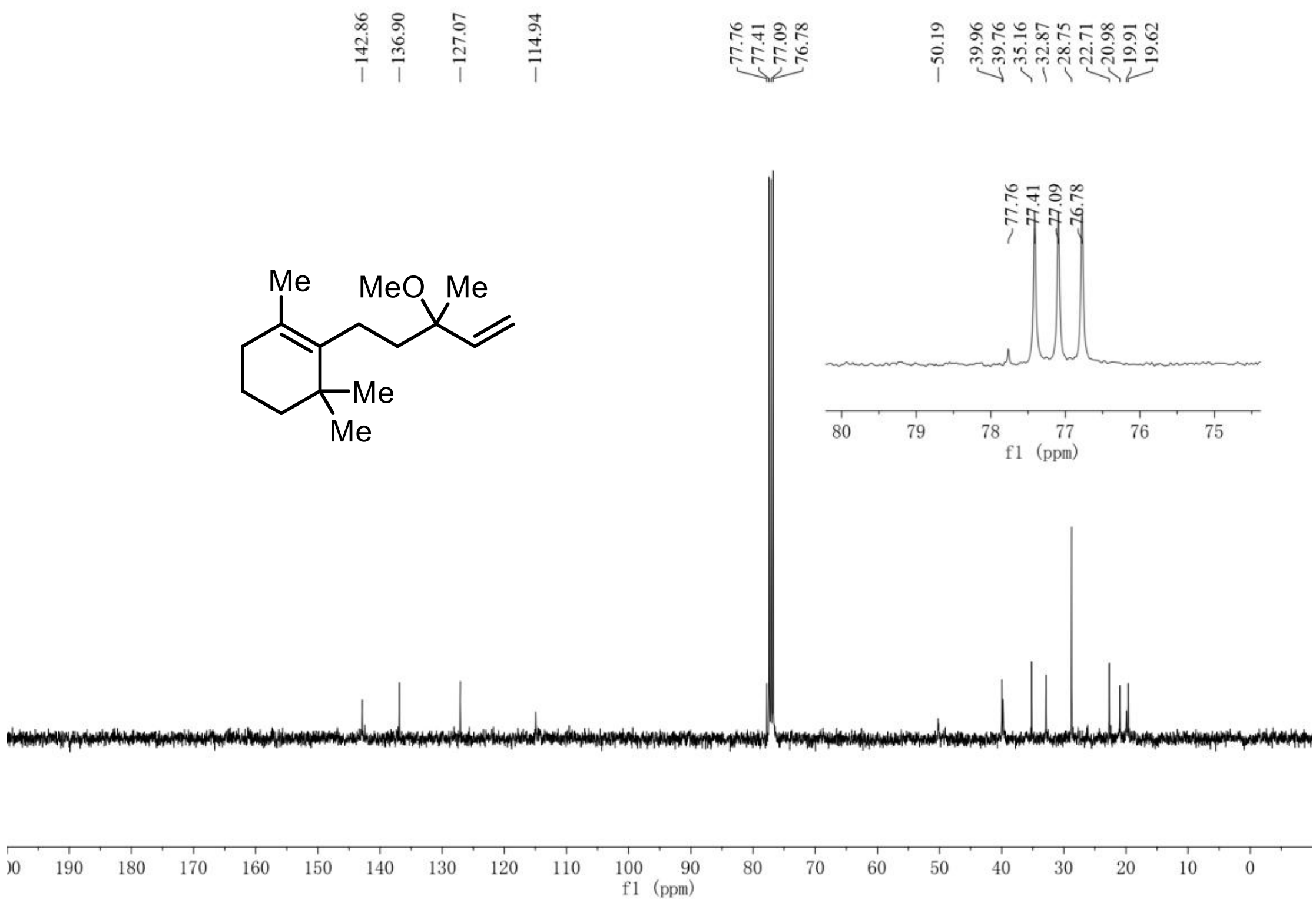

^{1}H NMR spectrum (400 MHz, $CDCl_3$)

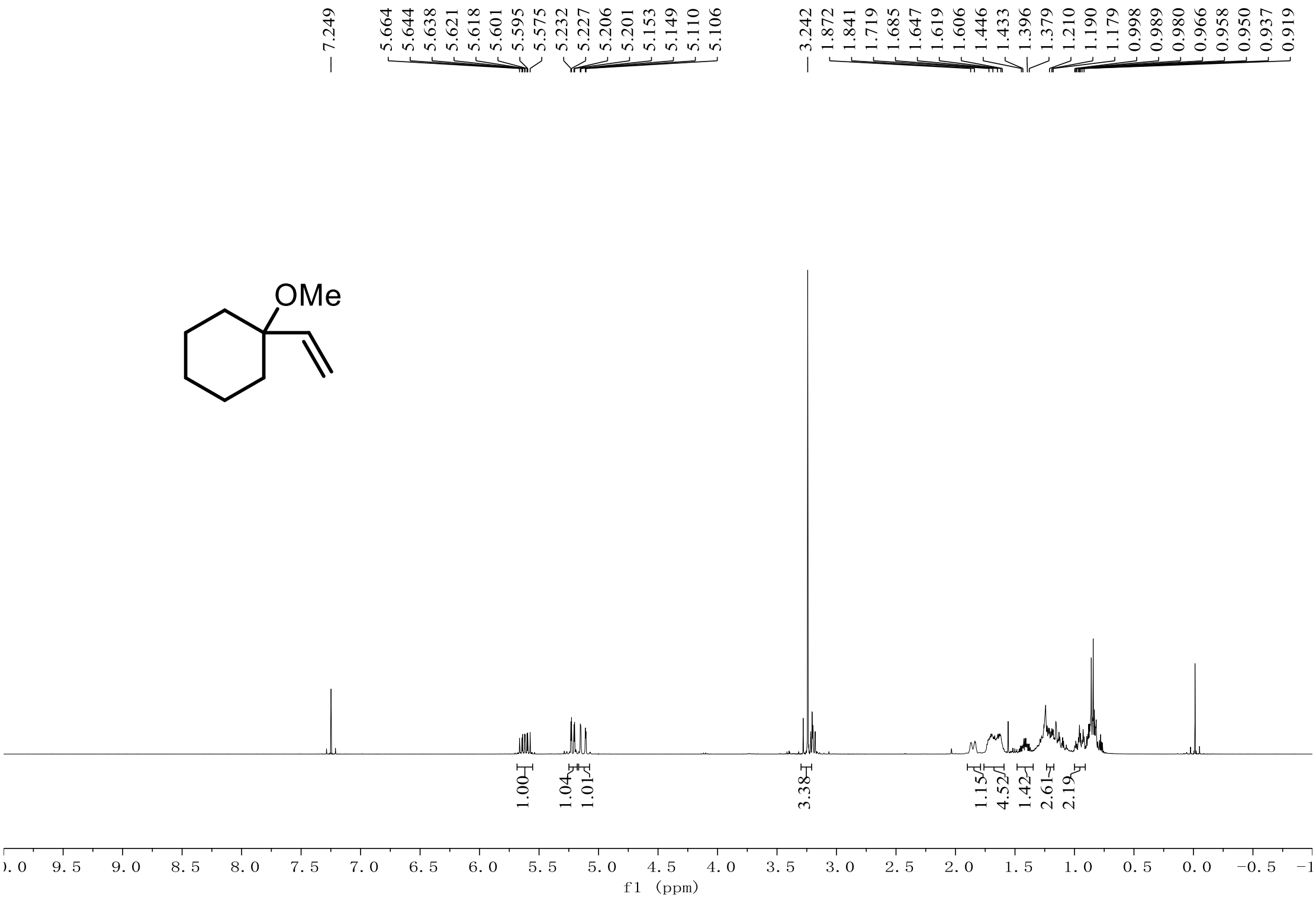

^{13}C NMR spectrum (101 MHz, $CDCl_3$)

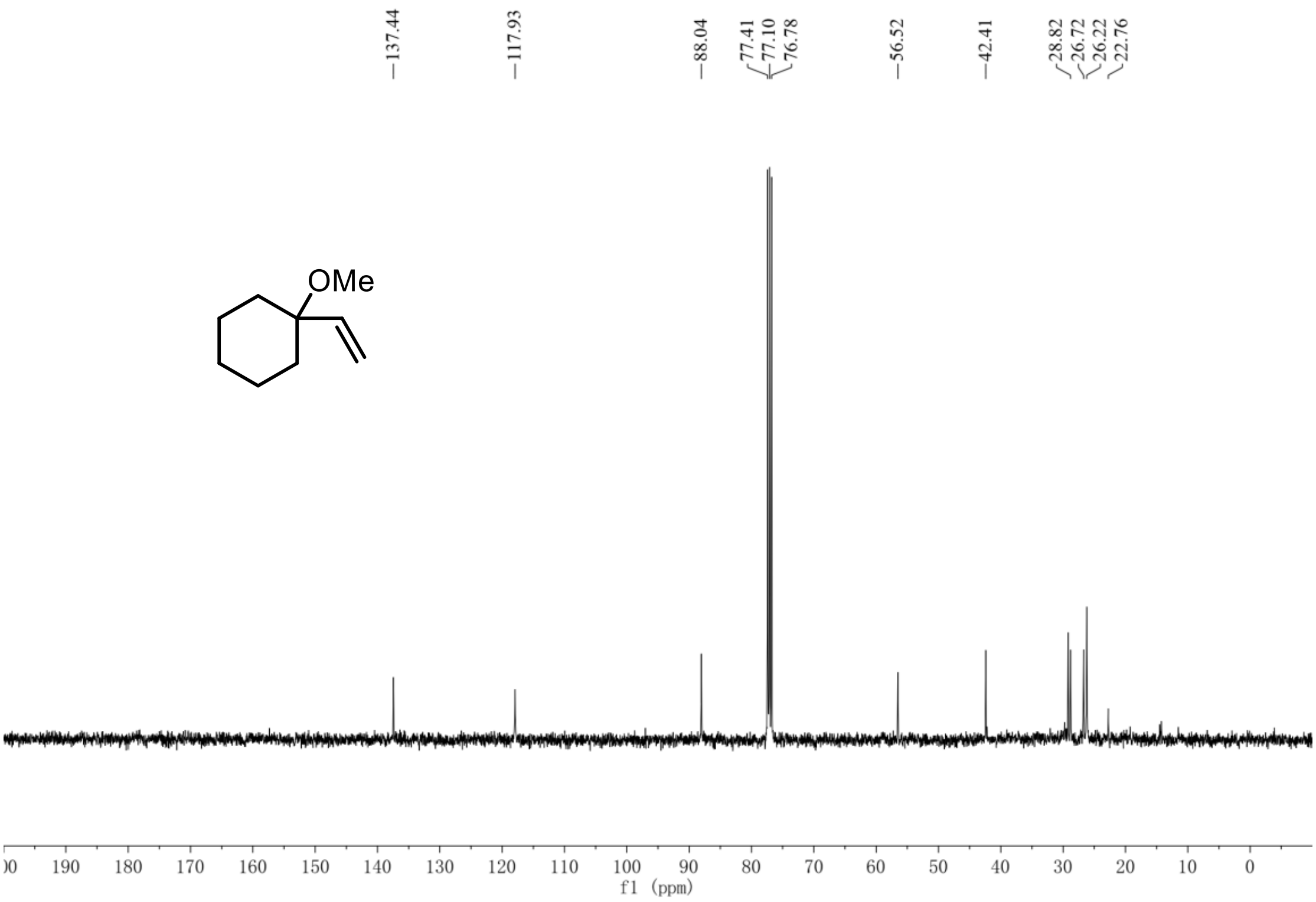

^{1}H NMR spectrum (400 MHz, $CDCl_3$)

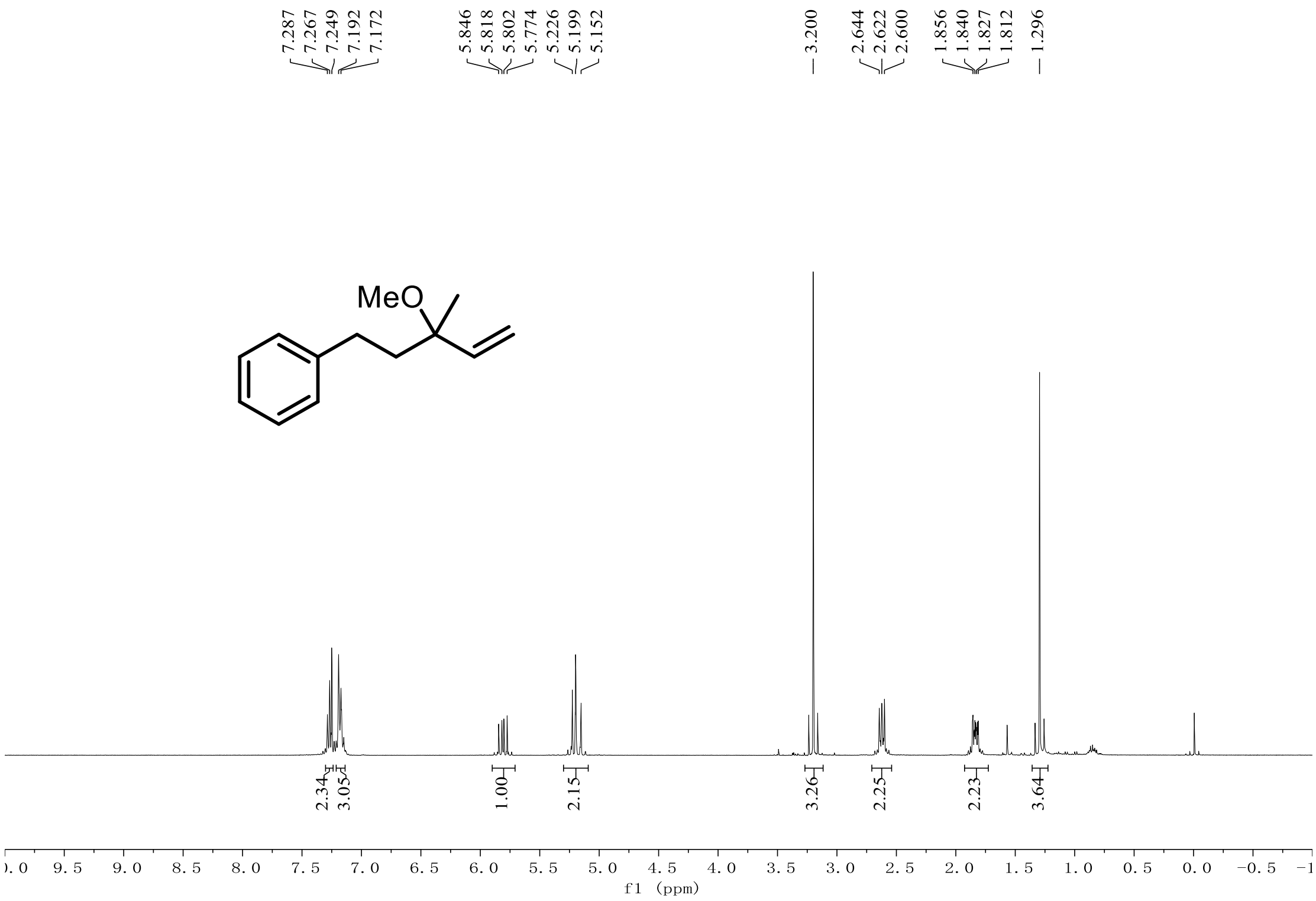

^{13}C NMR spectrum (101 MHz, $CDCl_3$)

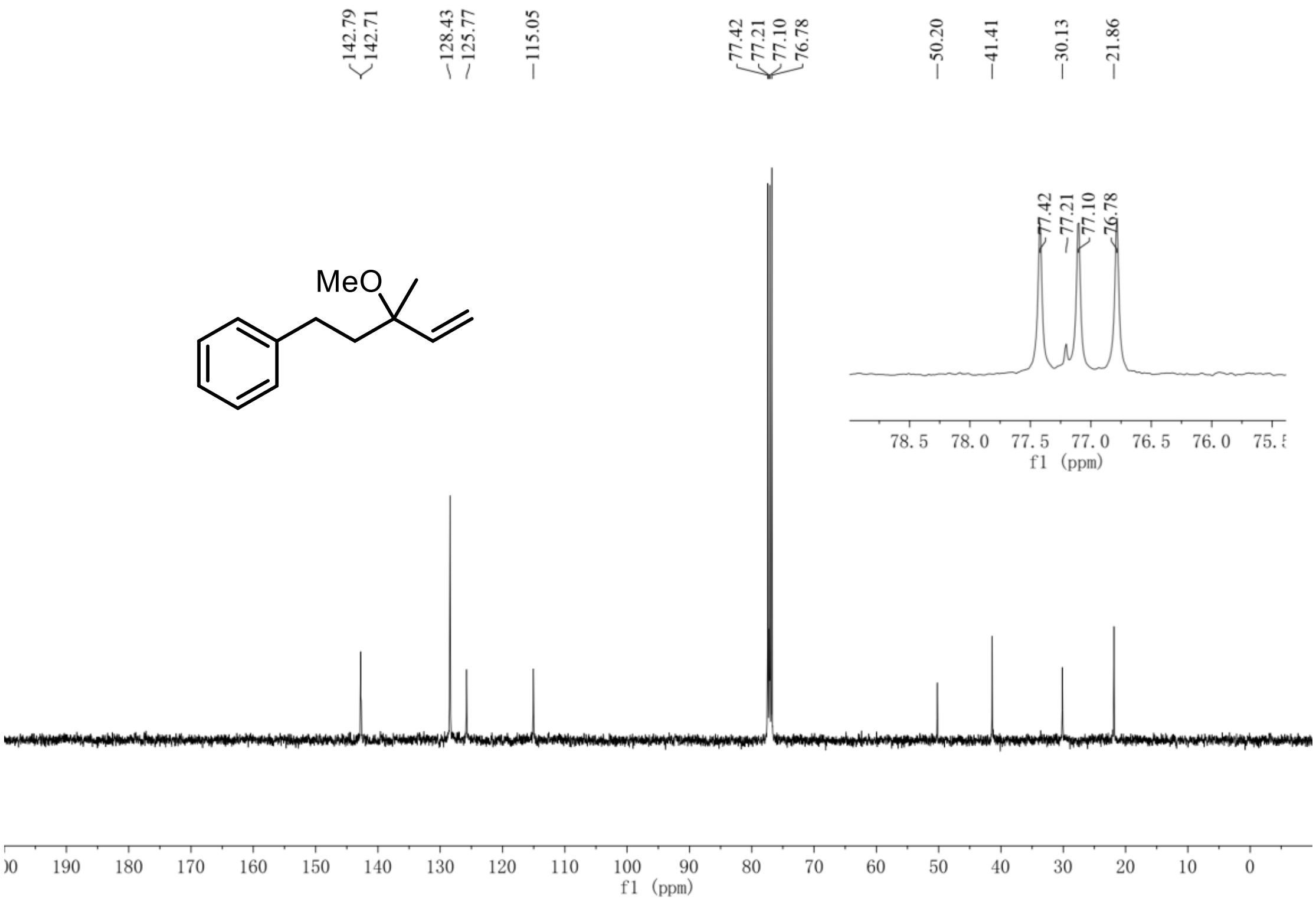

^{1}H NMR spectrum of 3a (400 MHz, $CDCl_3$)

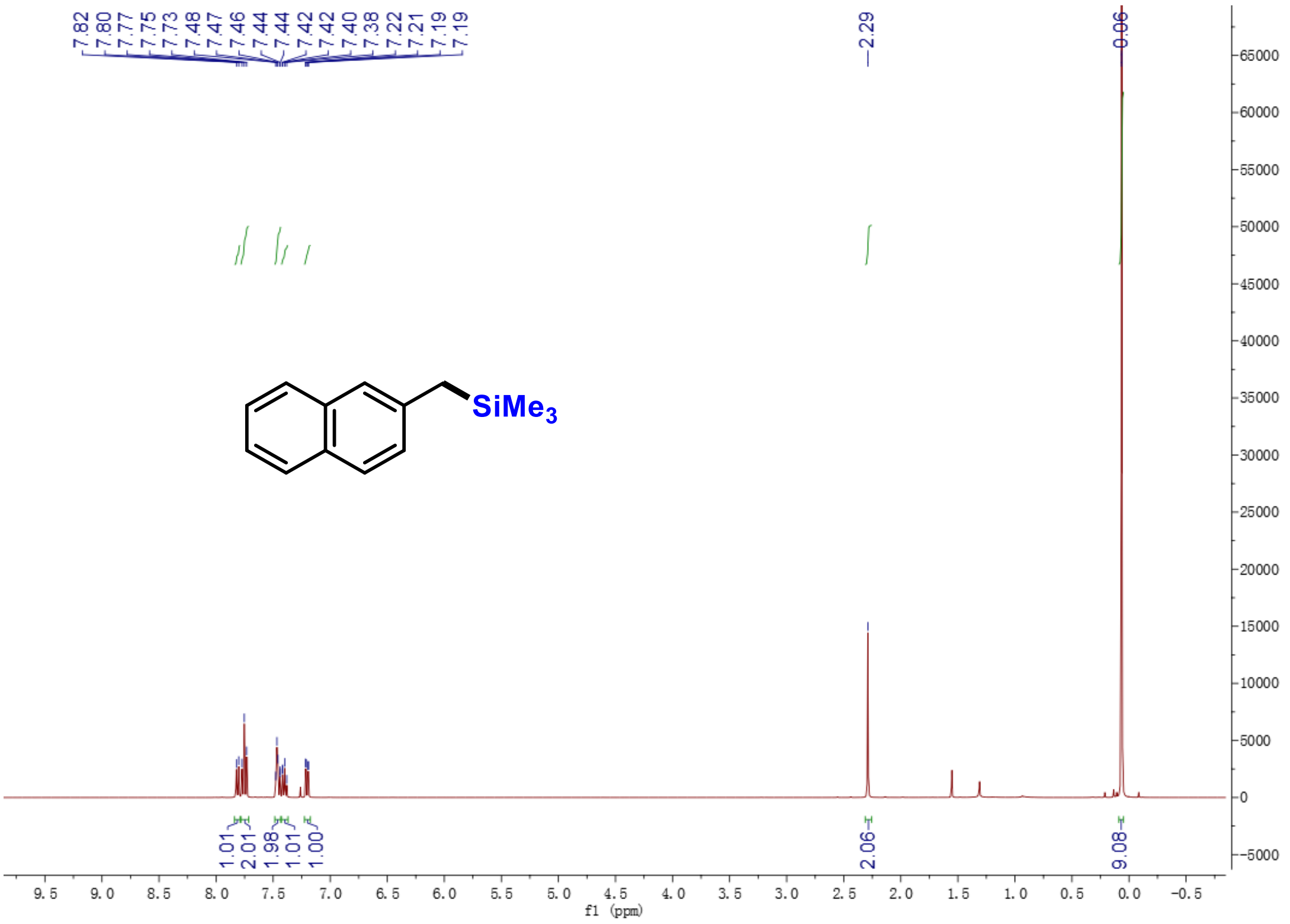

^{13}C NMR spectrum of 3a (101 MHz, $CDCl_3$)

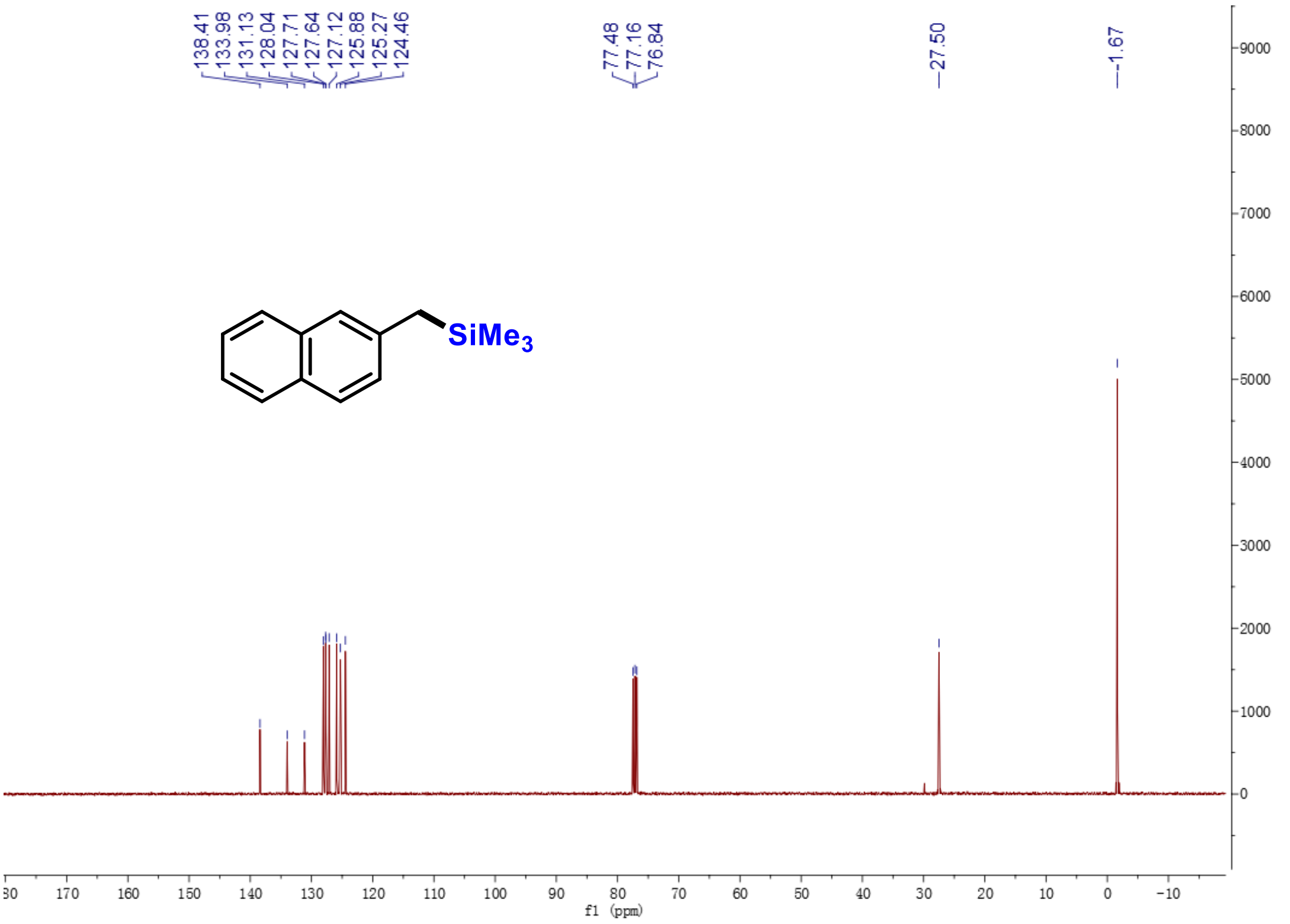

^{1}H NMR spectrum of 3b (400 MHz, $CDCl_3$)

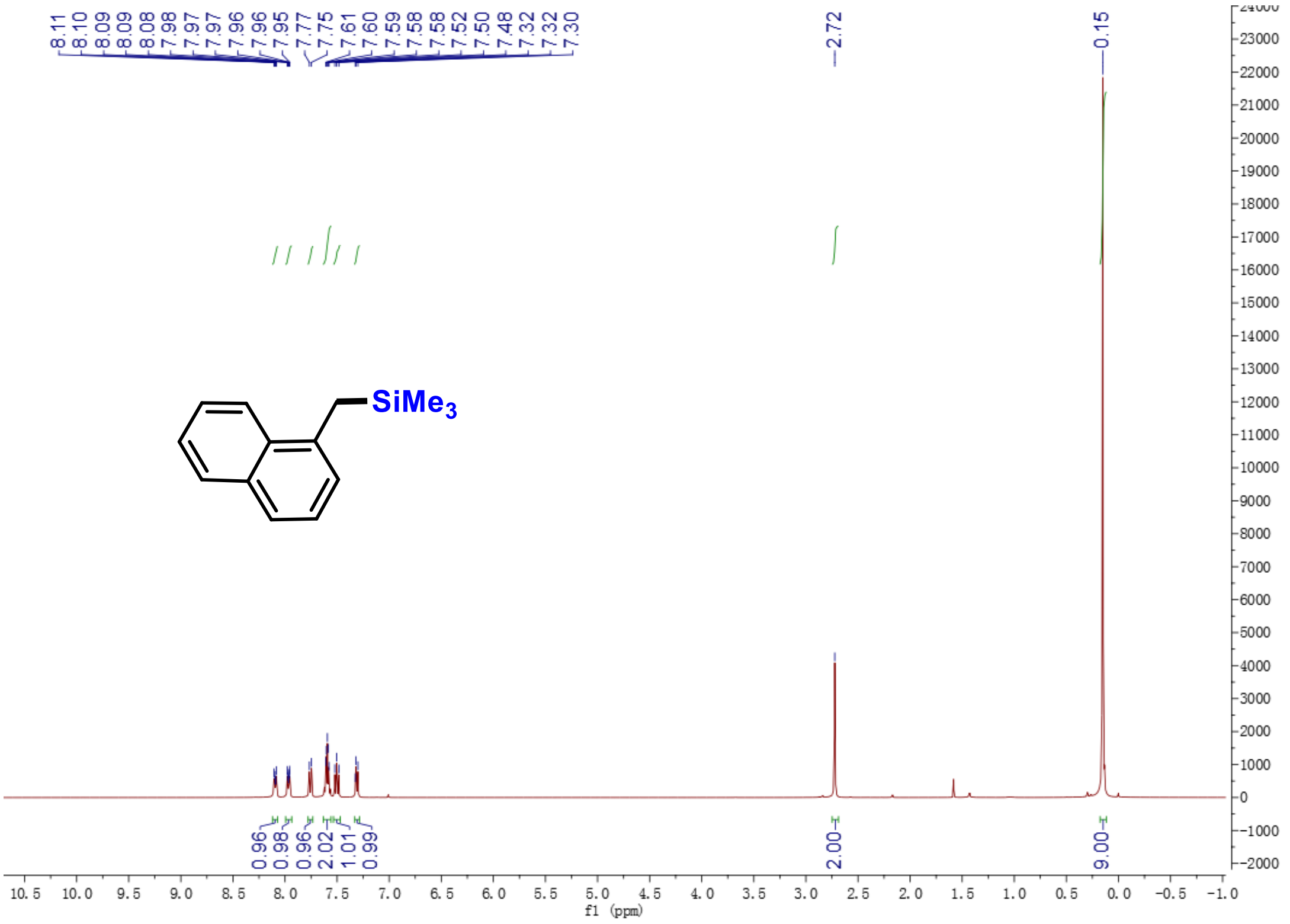

^{13}C NMR spectrum of 3b (101 MHz, $CDCl_3$)

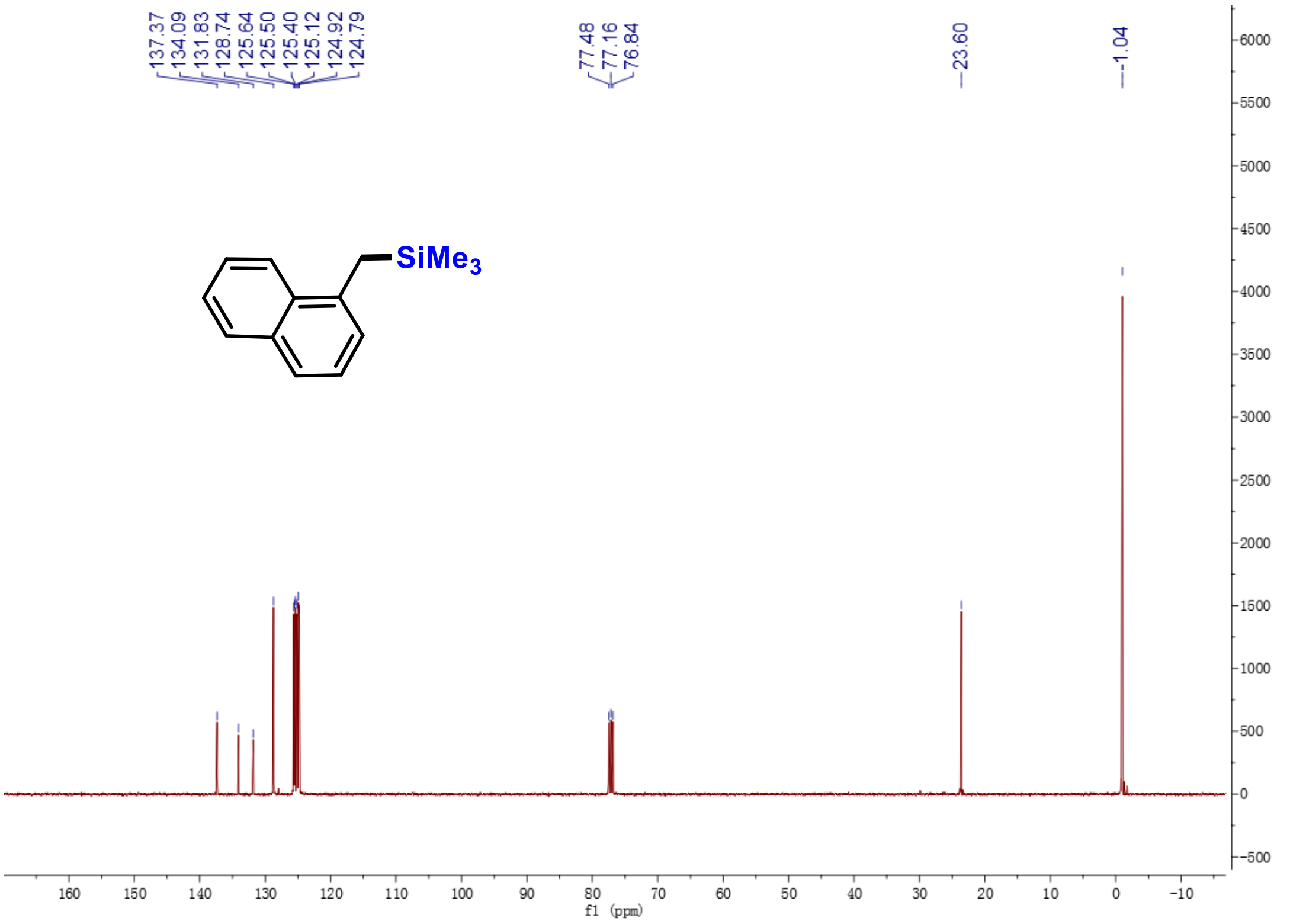

^{1}H NMR spectrum of 3c-DMP (400 MHz, $CDCl_3$)

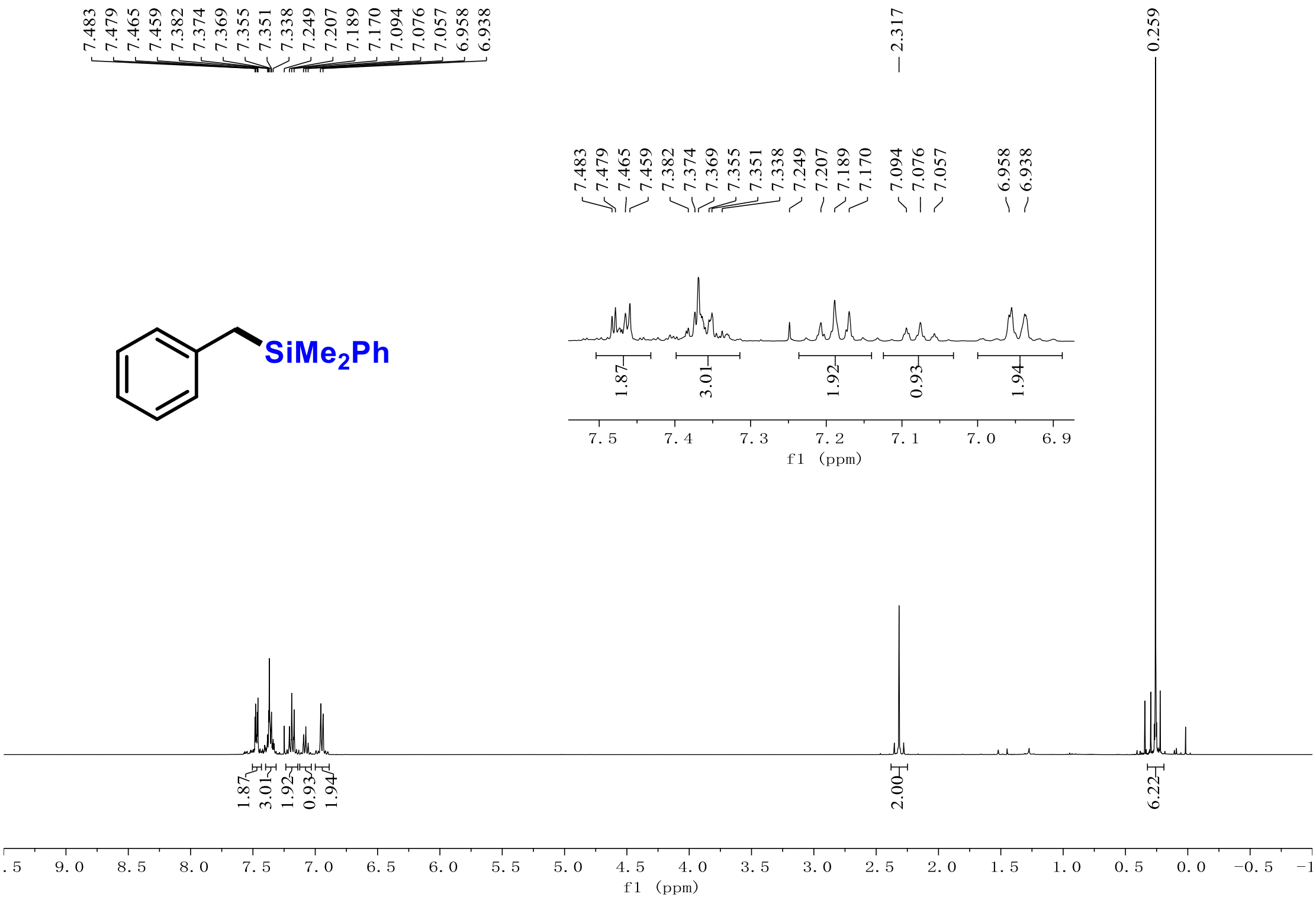

^{13}C NMR spectrum of 3c-DMP (101 MHz, $CDCl_3$)

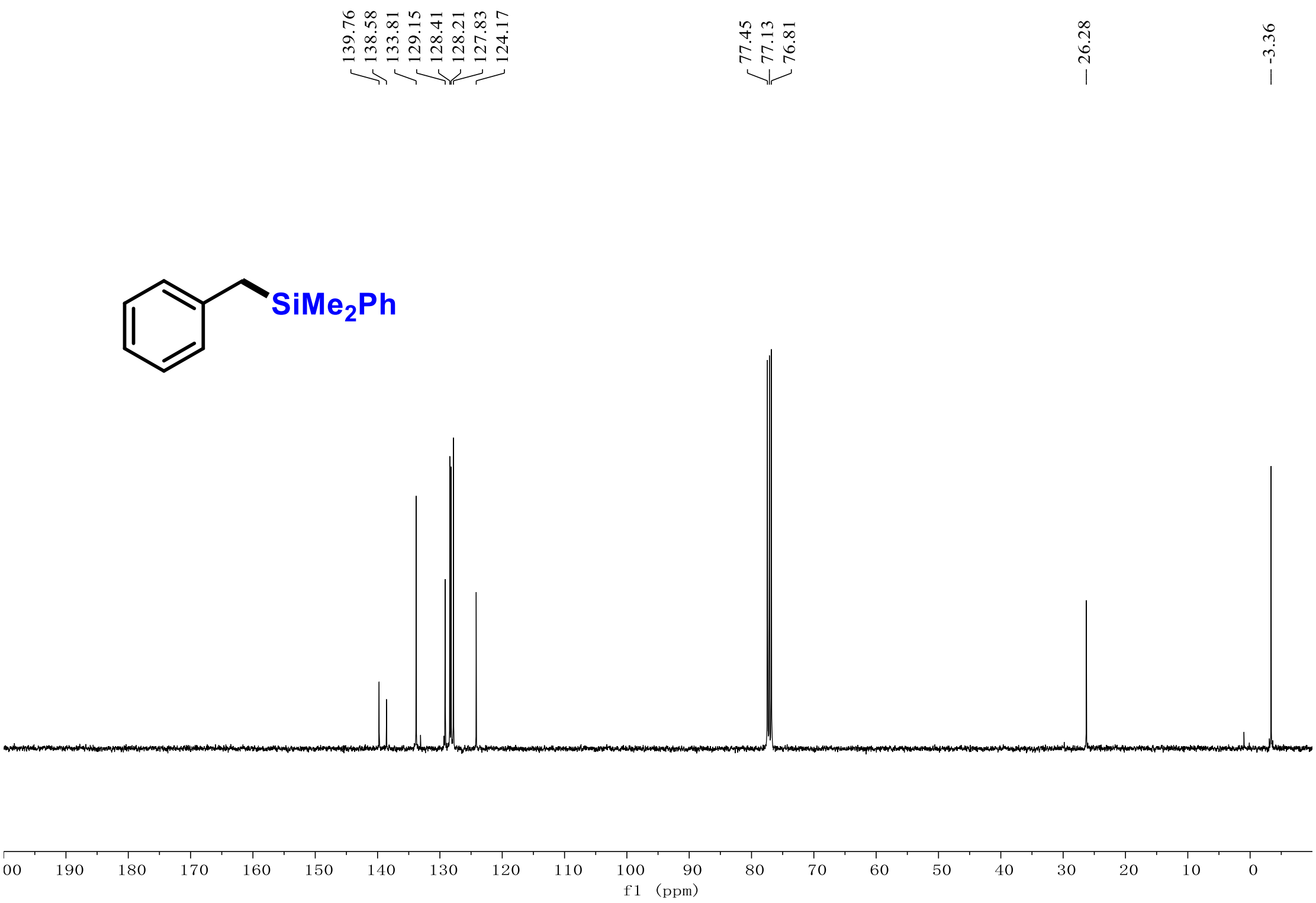

^{1}H NMR spectrum of 3d (400 MHz, $CDCl_3$)

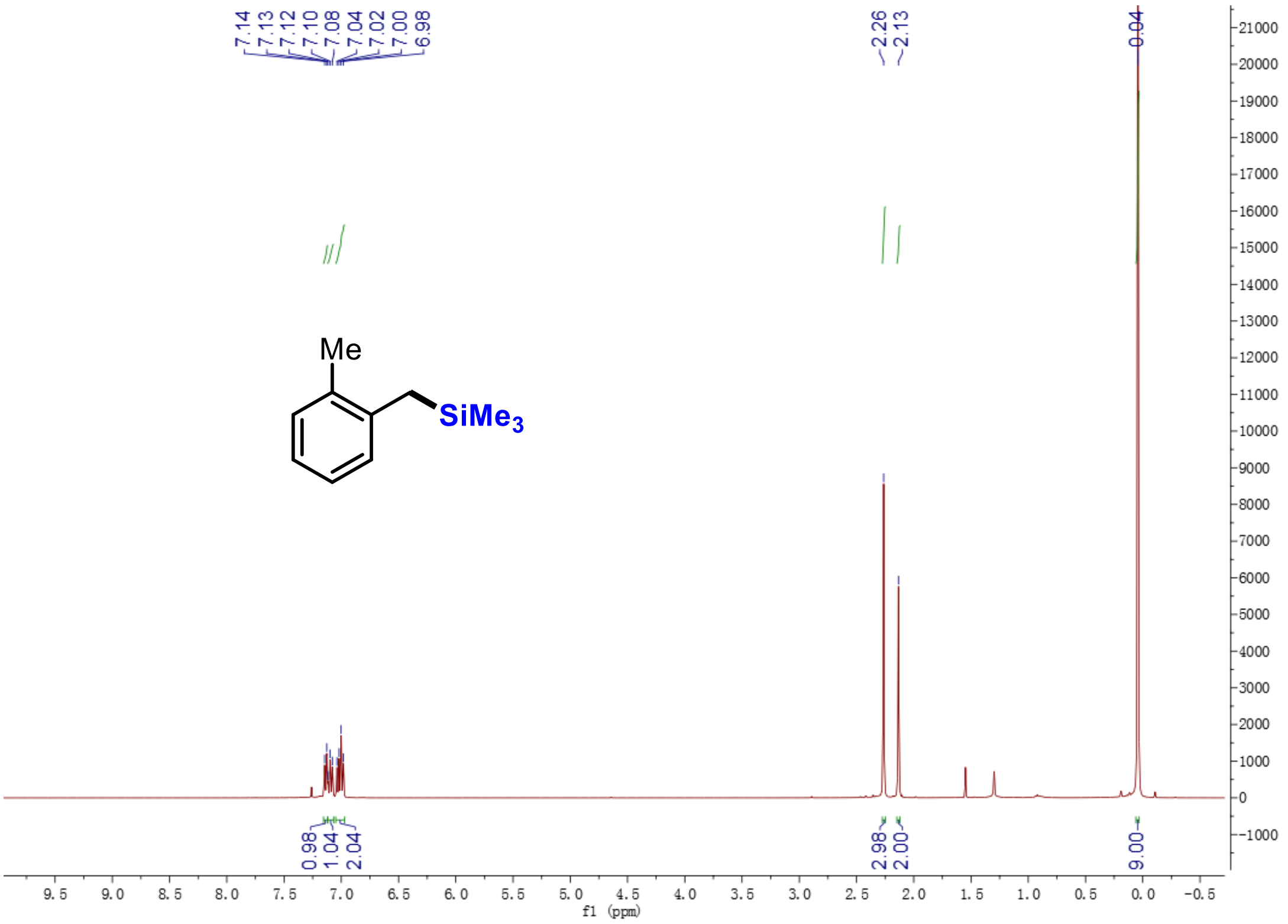

^{13}C NMR spectrum of 3d (101 MHz, $CDCl_3$)

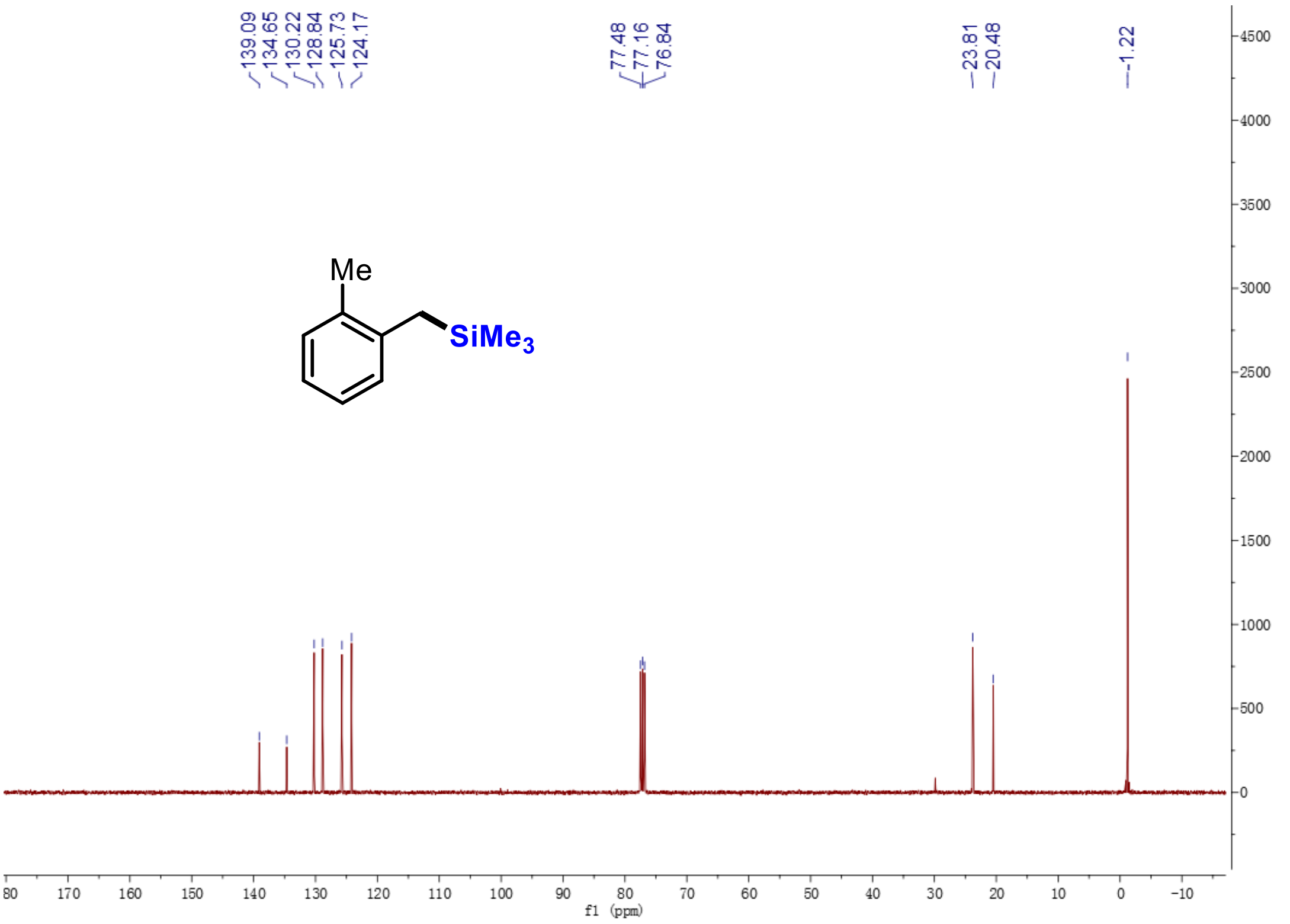

^{1}H NMR spectrum of 3e (400 MHz, $CDCl_3$)

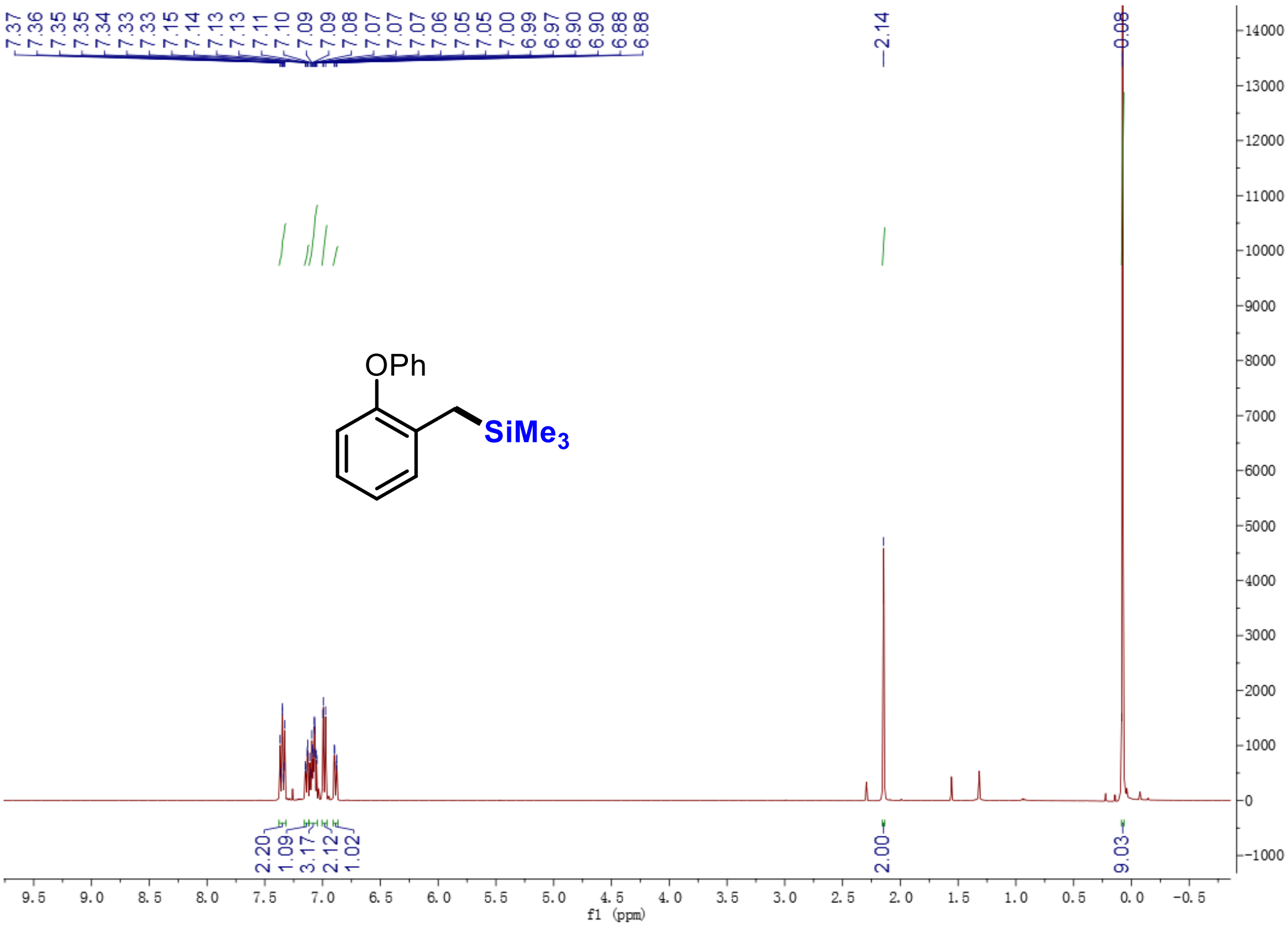

^{13}C NMR spectrum of 3e (101 MHz, $CDCl_3$)

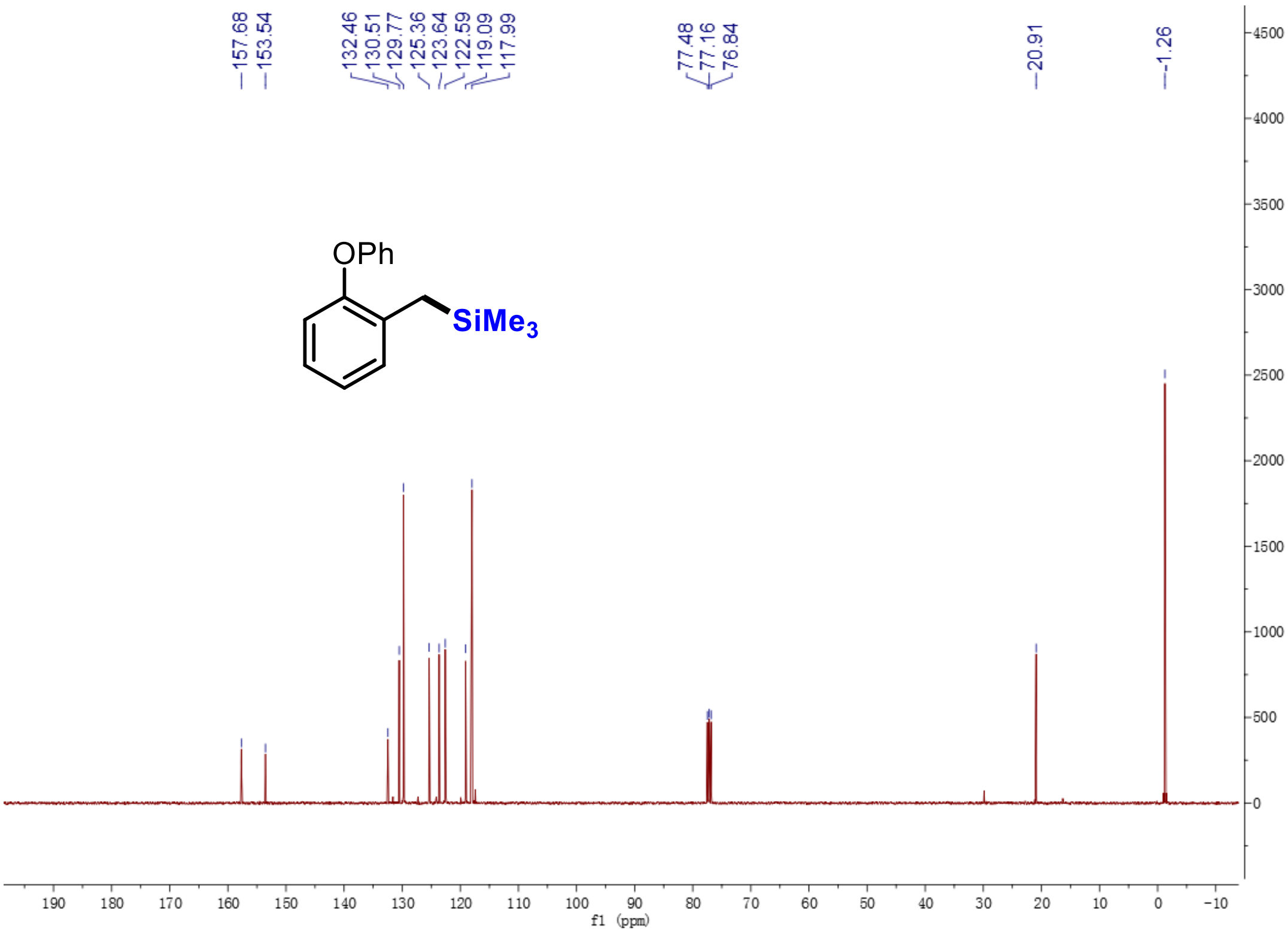

^{1}H NMR spectrum of 3f (400 MHz, $CDCl_3$)

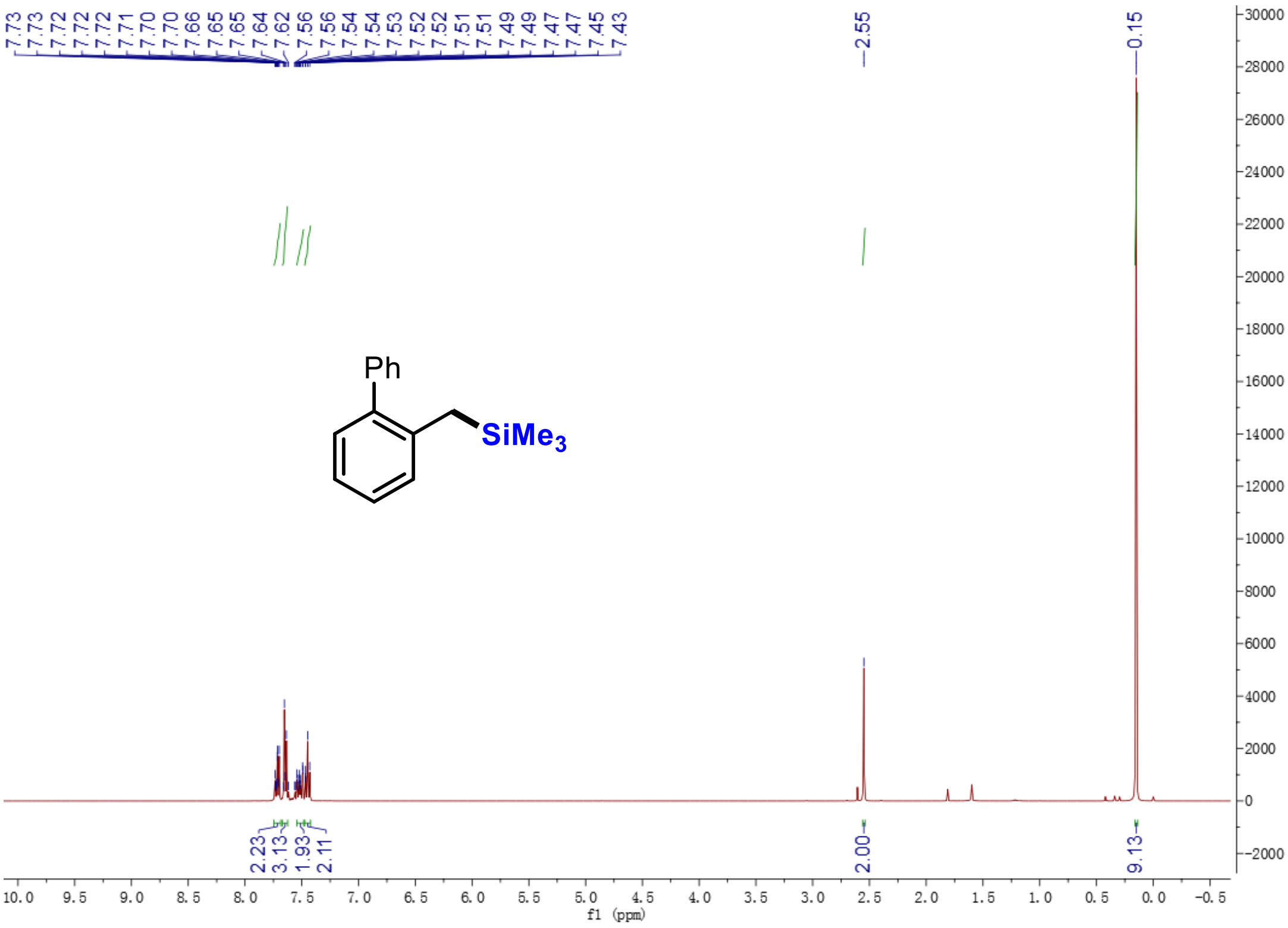

^{13}C NMR spectrum of 3f (101 MHz, $CDCl_3$)

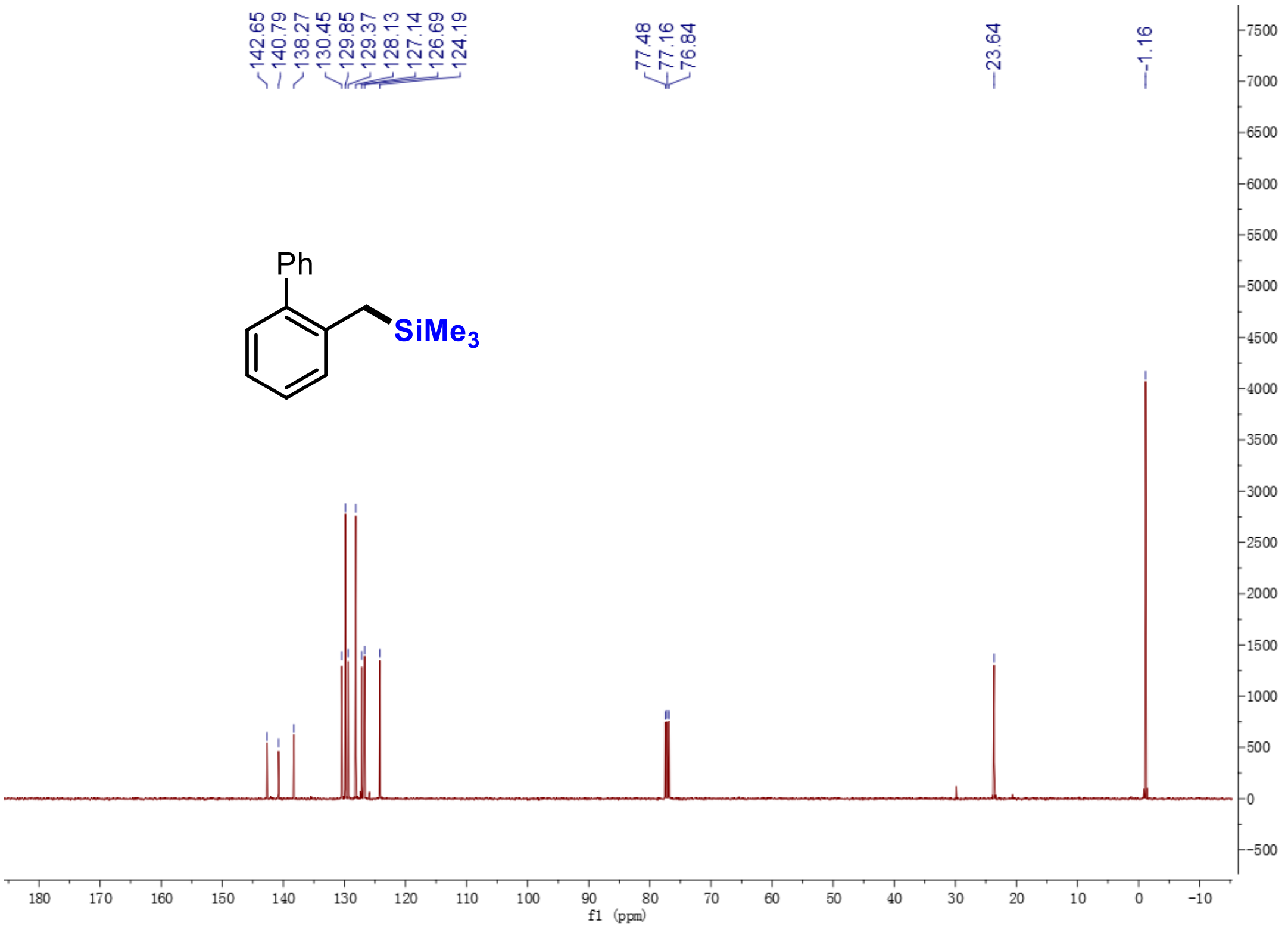

^{1}H NMR spectrum of 3g (400 MHz, $CDCl_3$)

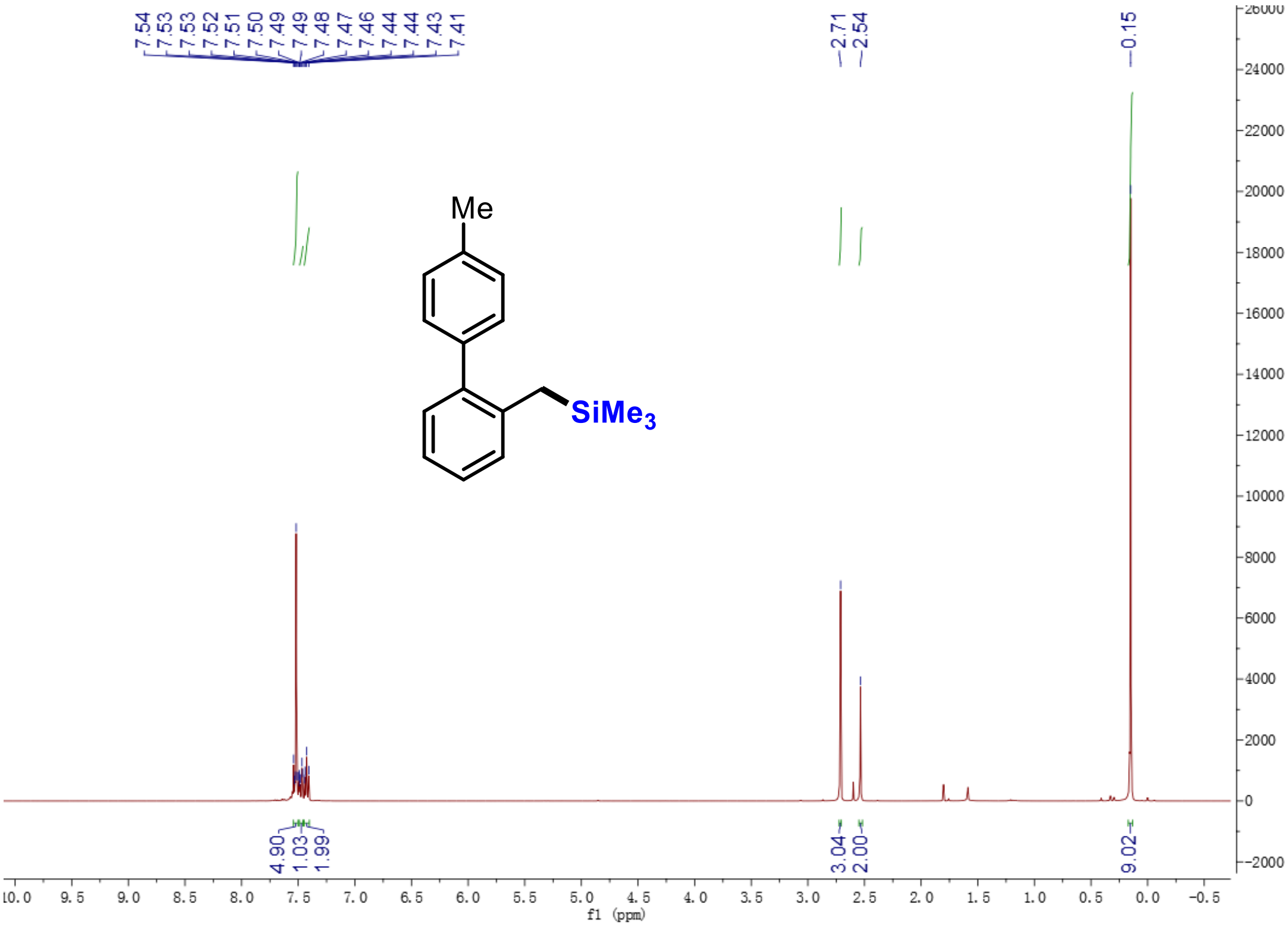

^{13}C NMR spectrum of 3g (101 MHz, $CDCl_3$)

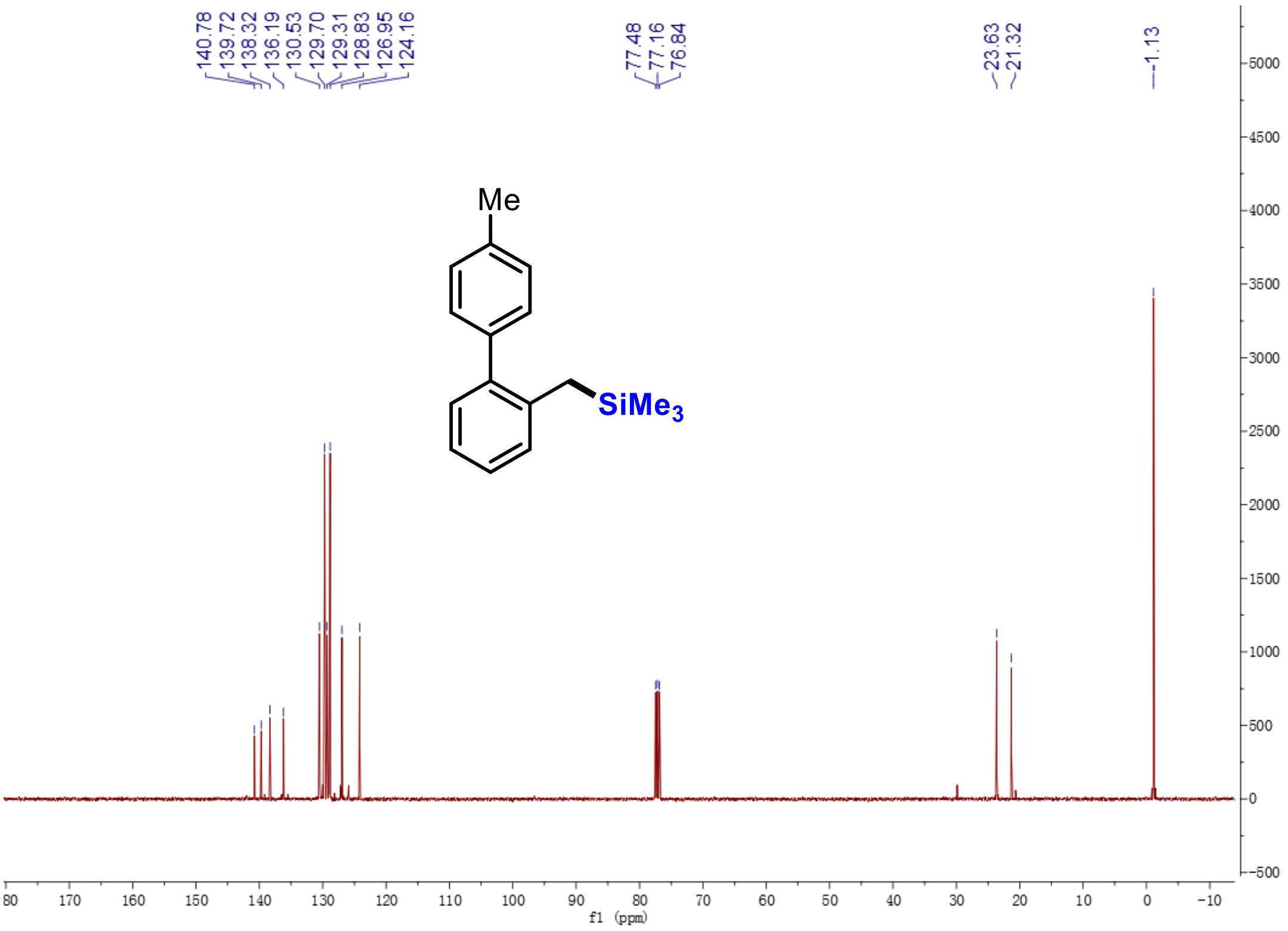

^{1}H NMR spectrum of 3h (400 MHz, $CDCl_3$)

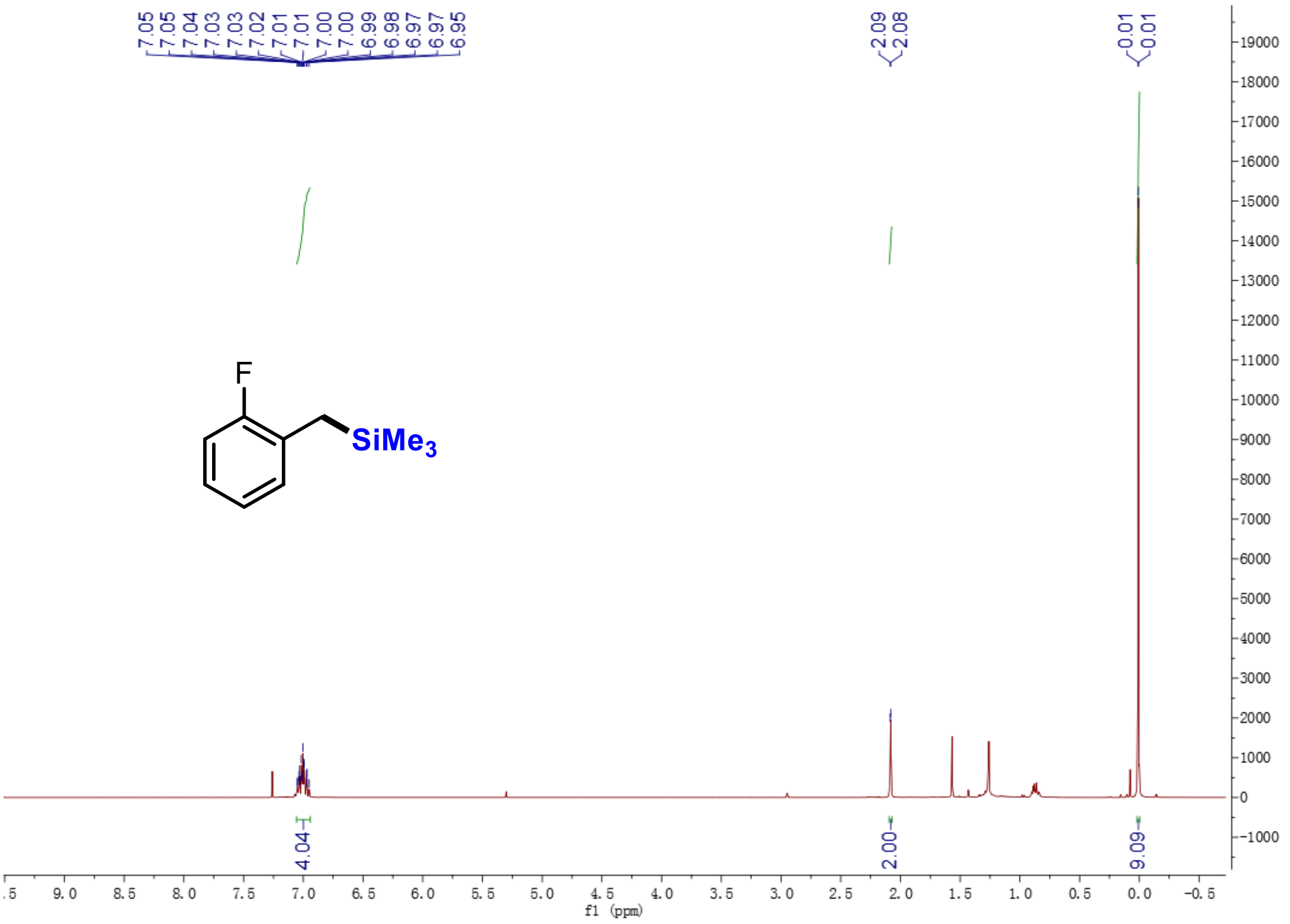

^{13}C NMR spectrum of 3h (101 MHz, $CDCl_3$)

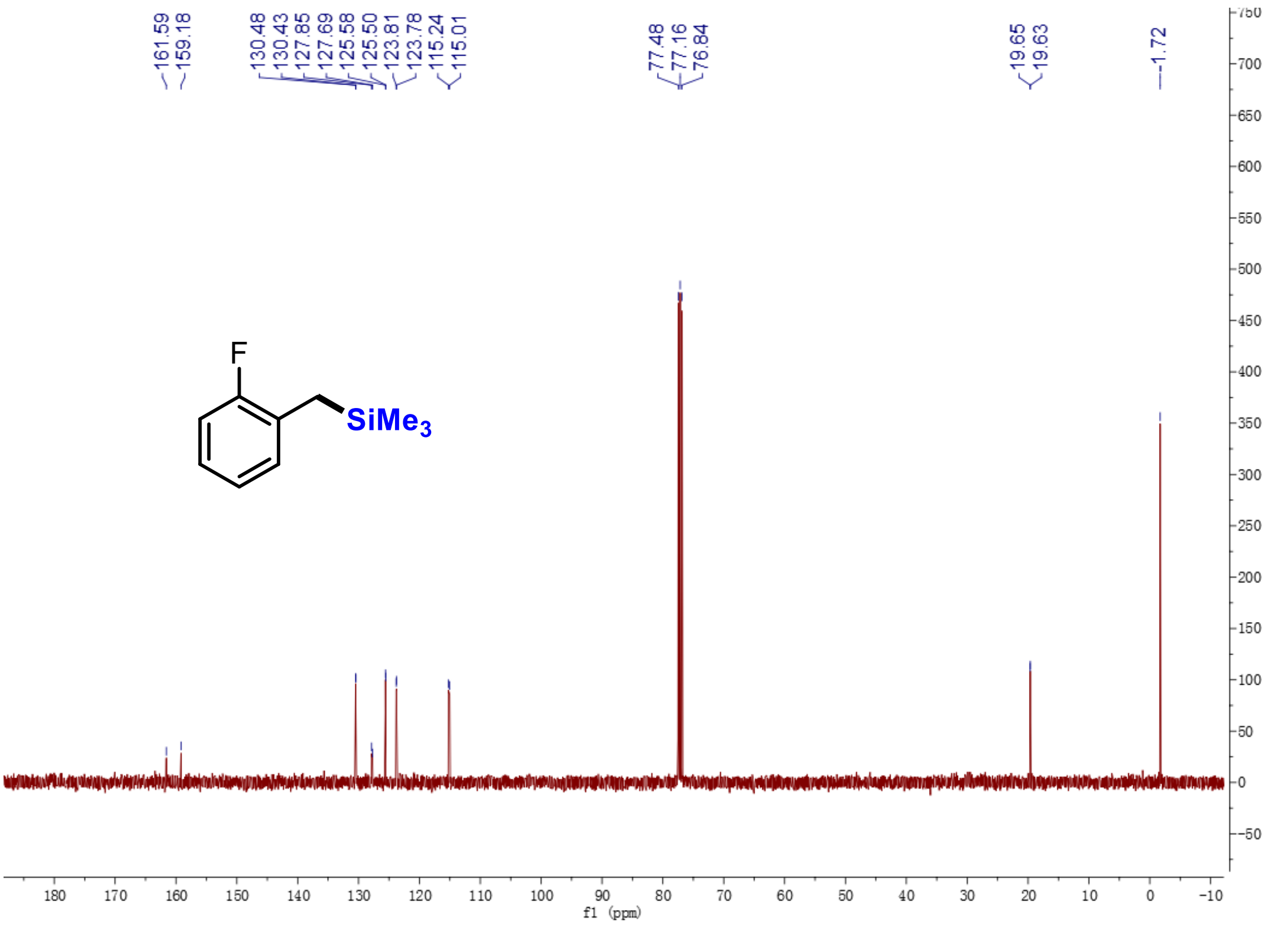

^{19}F NMR spectrum of 3h

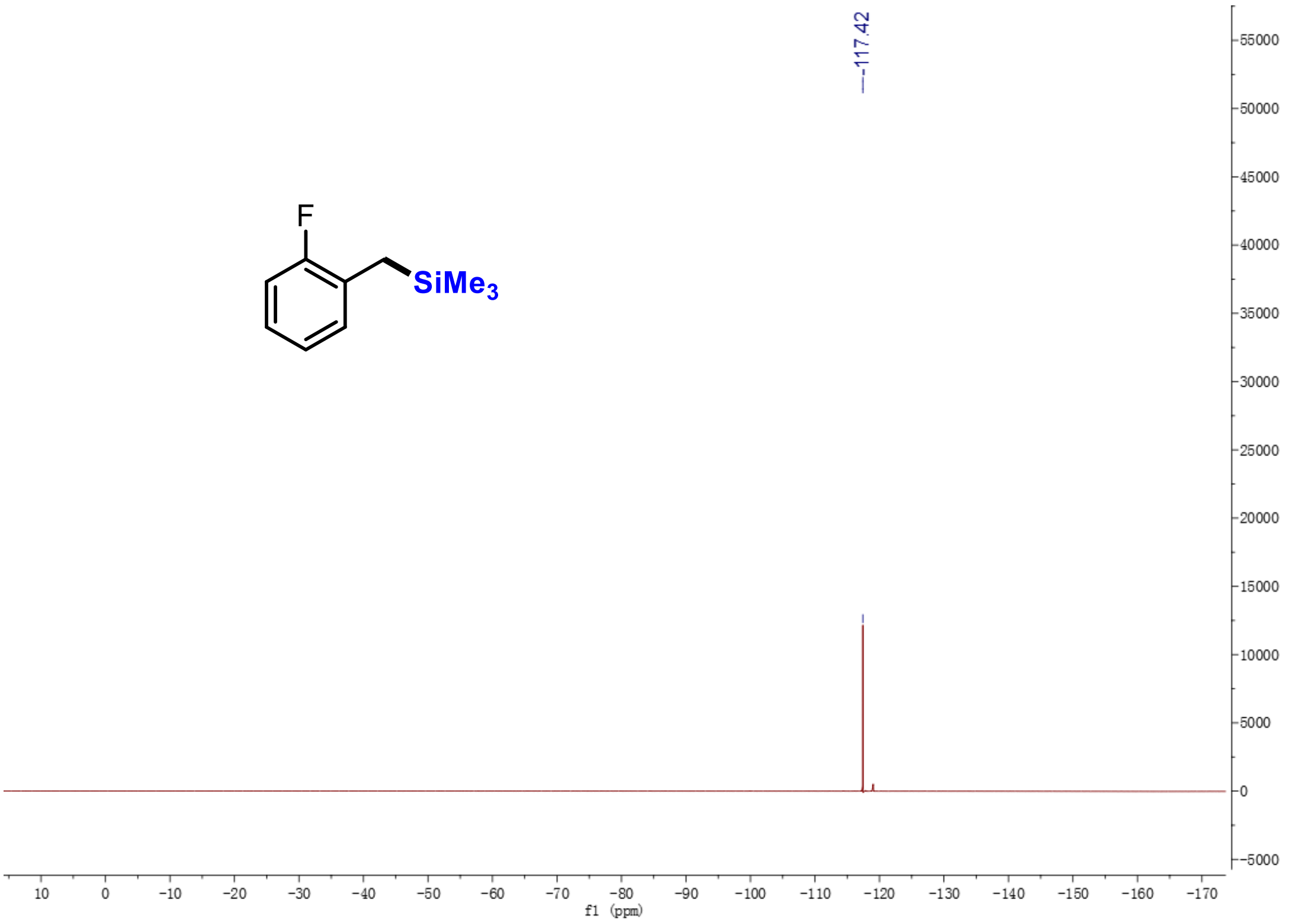

^{1}H NMR spectrum of 3i (400 MHz, $CDCl_3$)

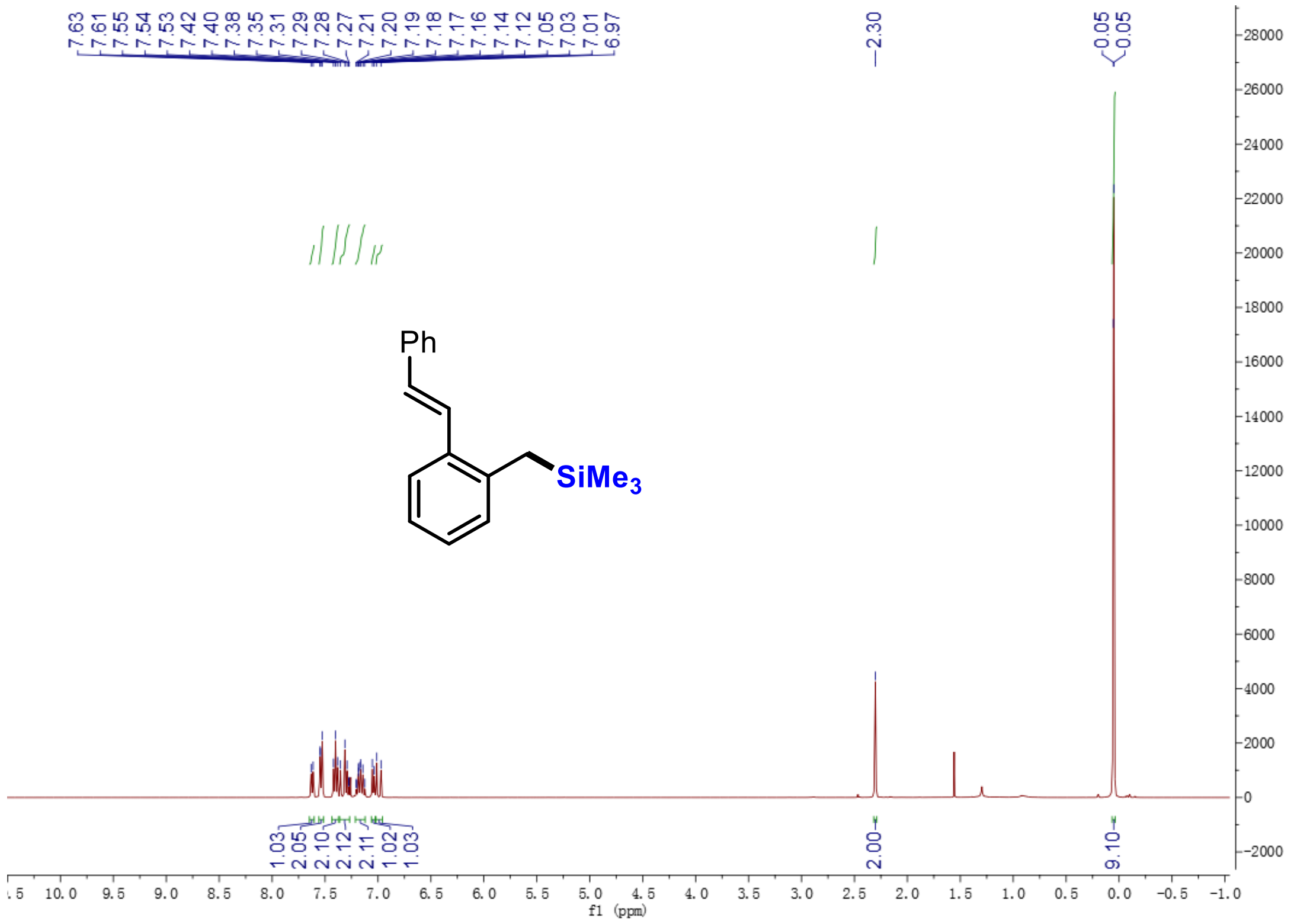

^{13}C NMR spectrum of 3i (101 MHz, $CDCl_3$)

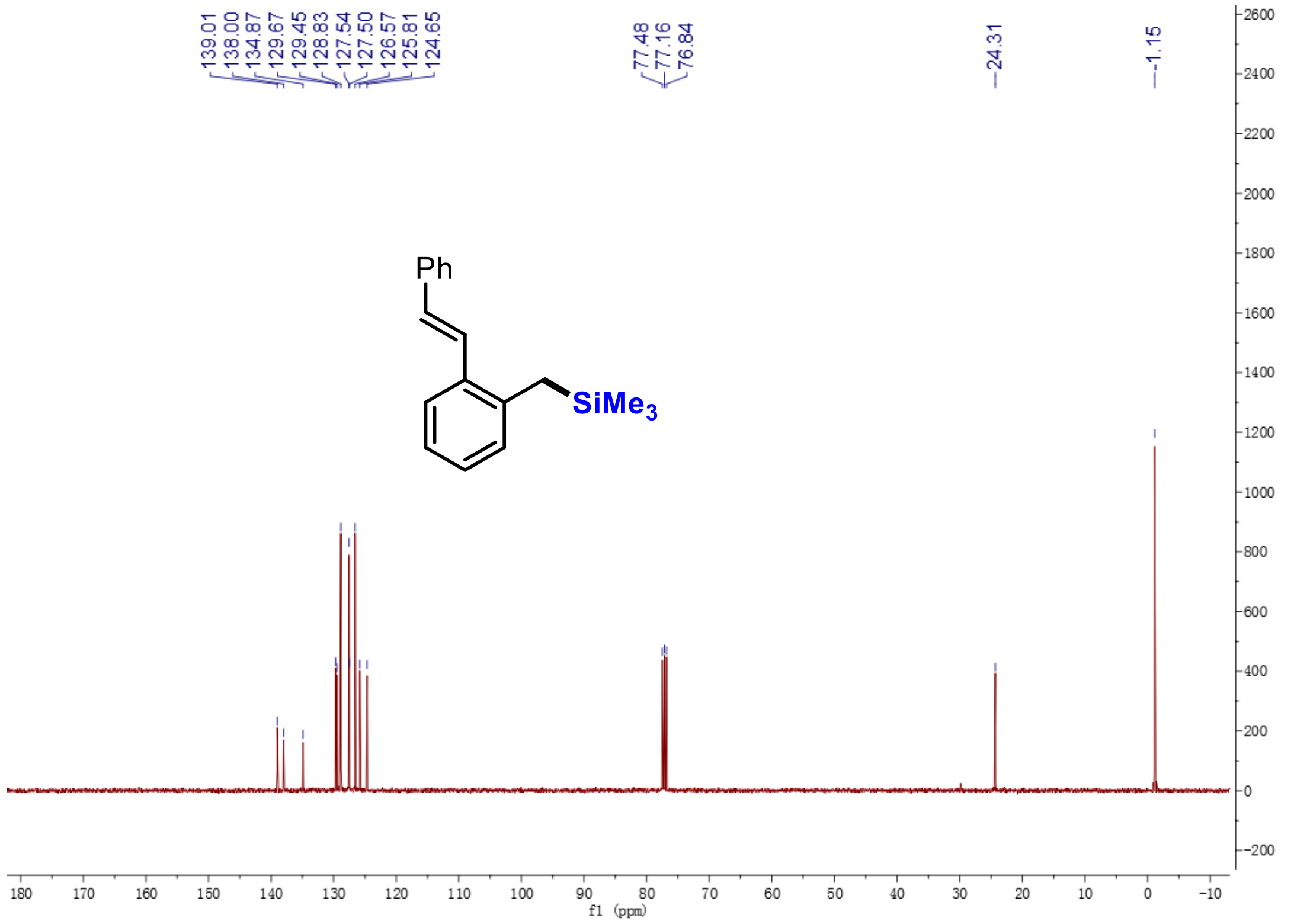

^{1}**H NMR spectrum of 3j** (400 MHz, $CDCl_3$)

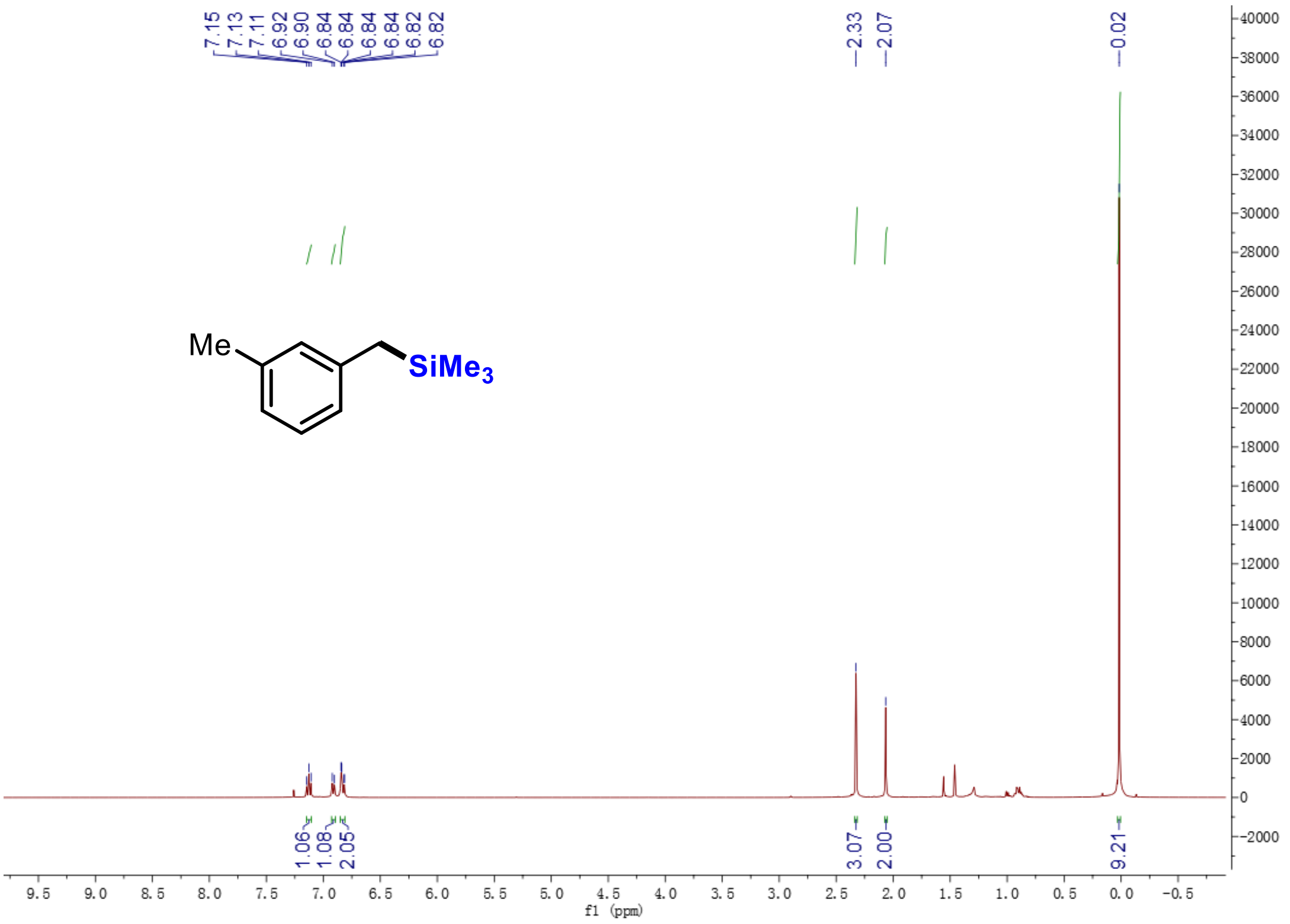

^{13}**C NMR spectrum of 3j** (101 MHz, $CDCl_3$)

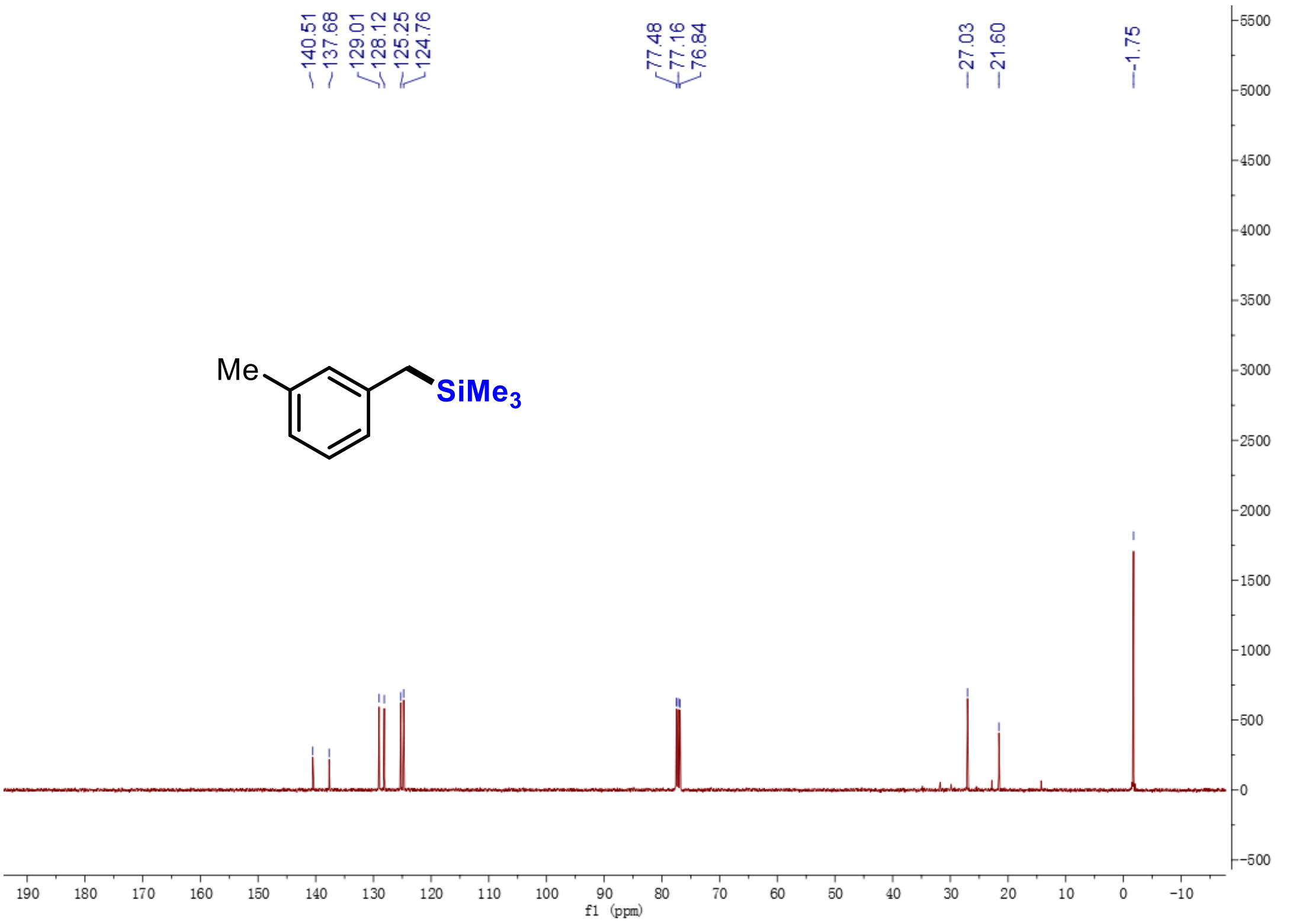

^{1}H NMR spectrum of 3k (400 MHz, $CDCl_3$)

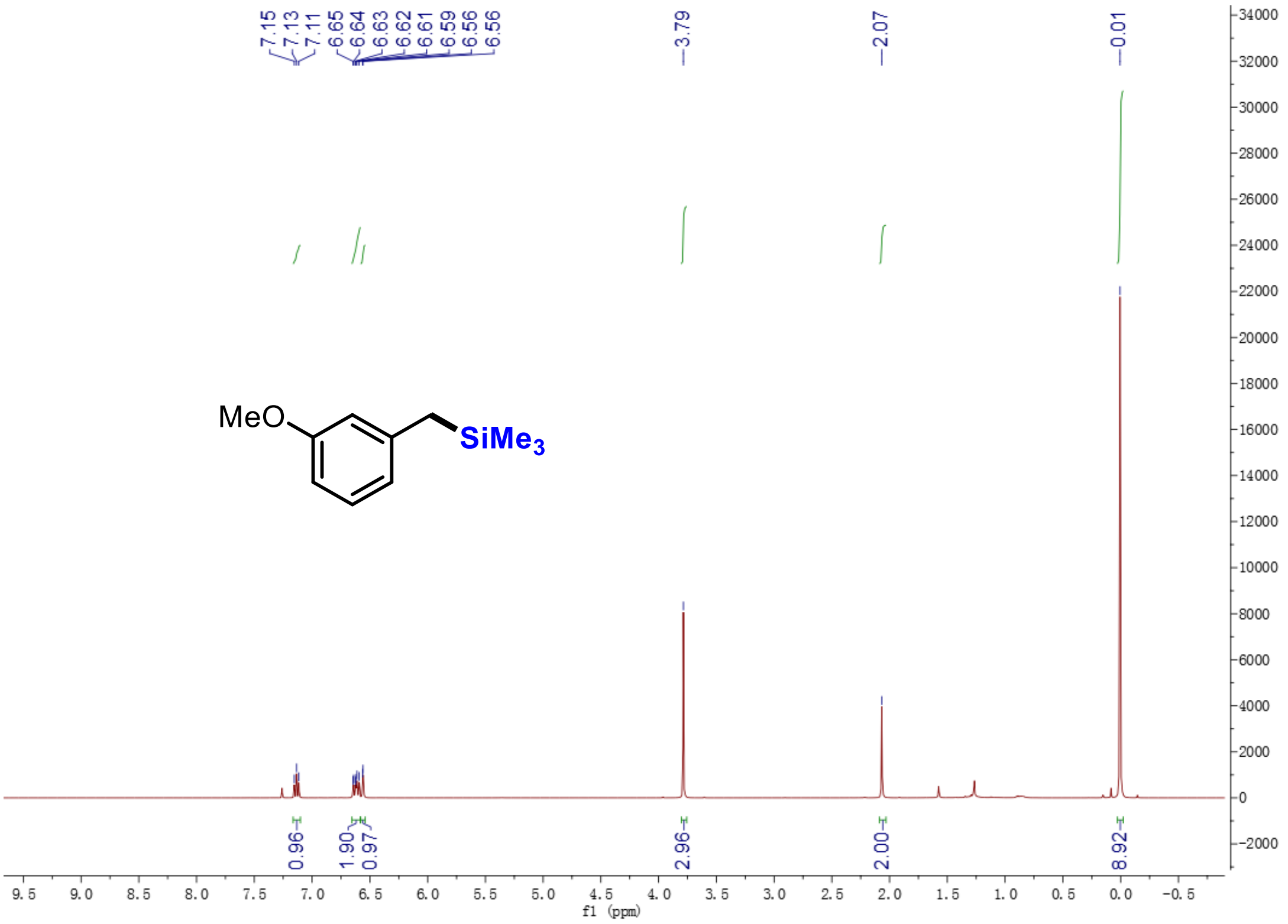

^{13}C NMR spectrum of 3k (101 MHz, $CDCl_3$)

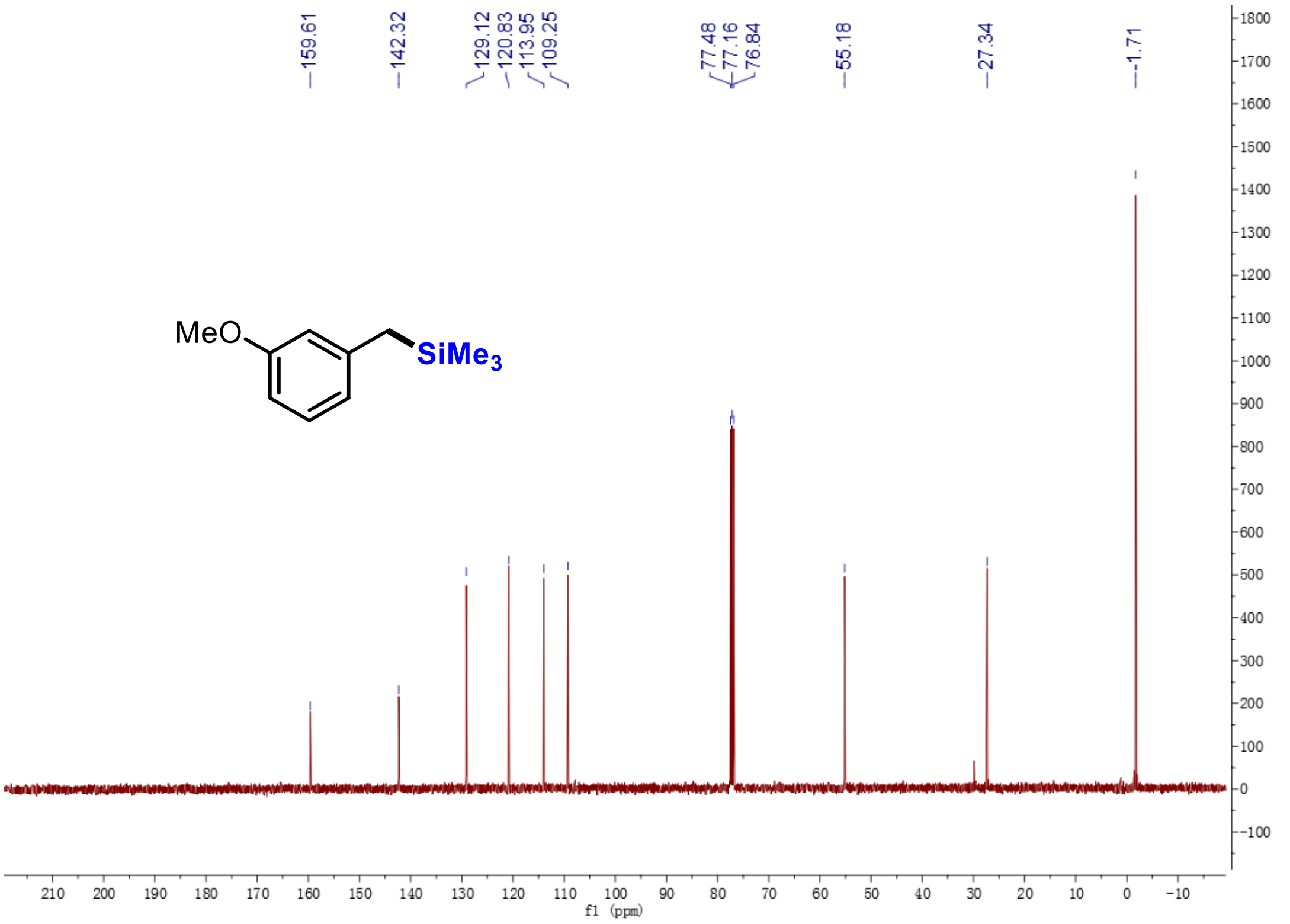

^{1}H NMR spectrum of 3l (400 MHz, $CDCl_3$)

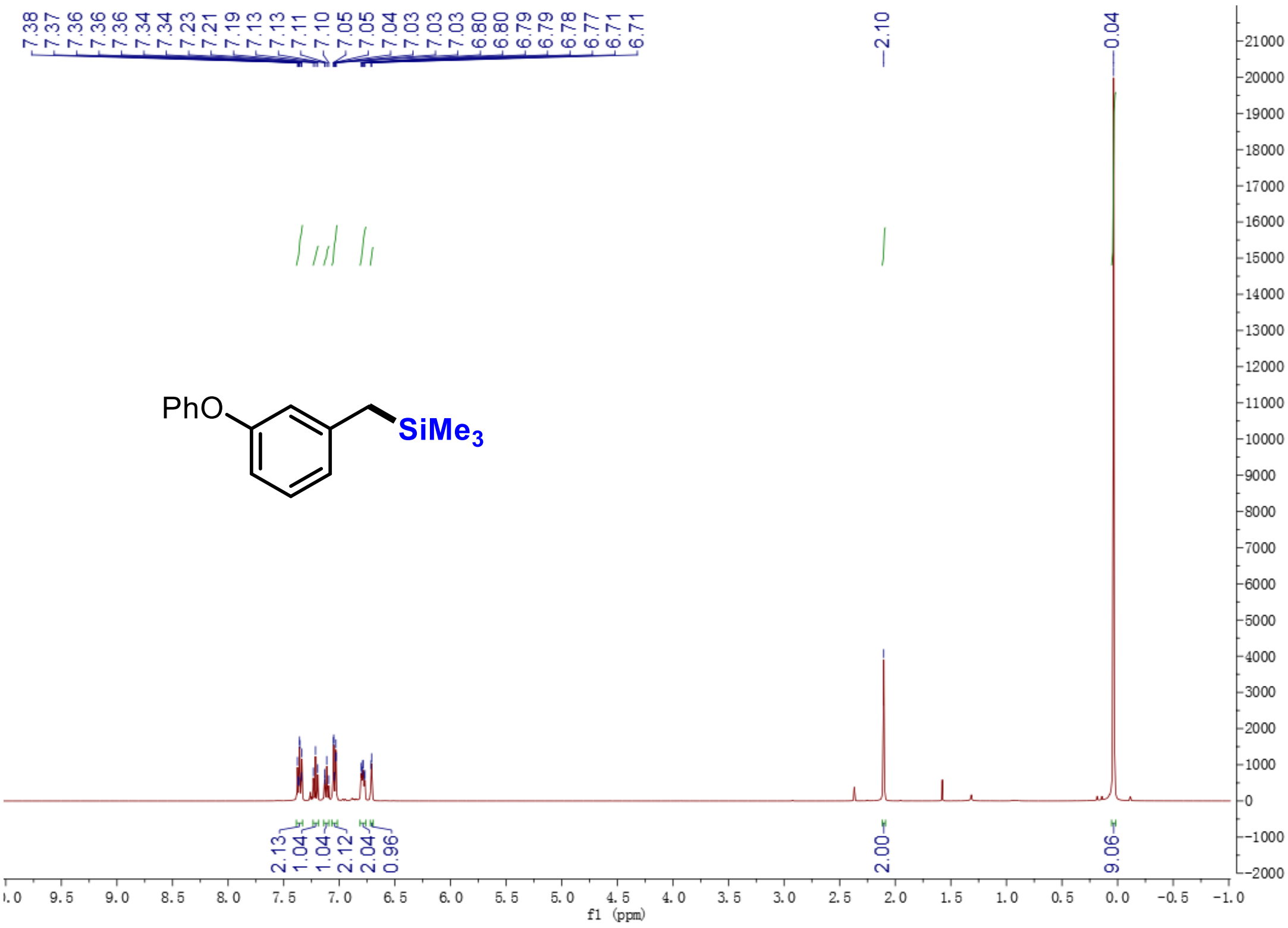

^{13}C NMR spectrum of 3l (101 MHz, $CDCl_3$)

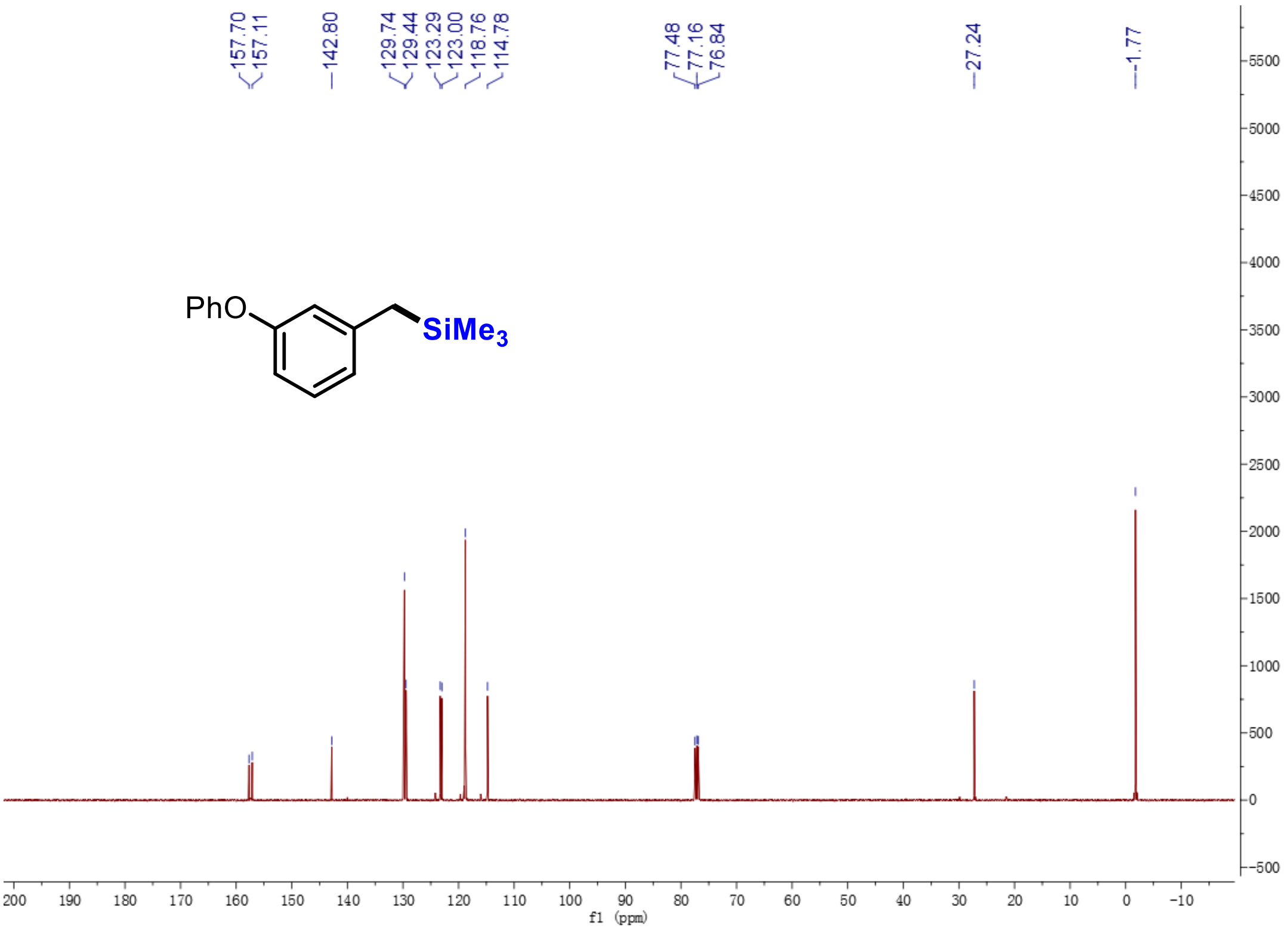

^{1}H NMR spectrum of 3m (400 MHz, $CDCl_3$)

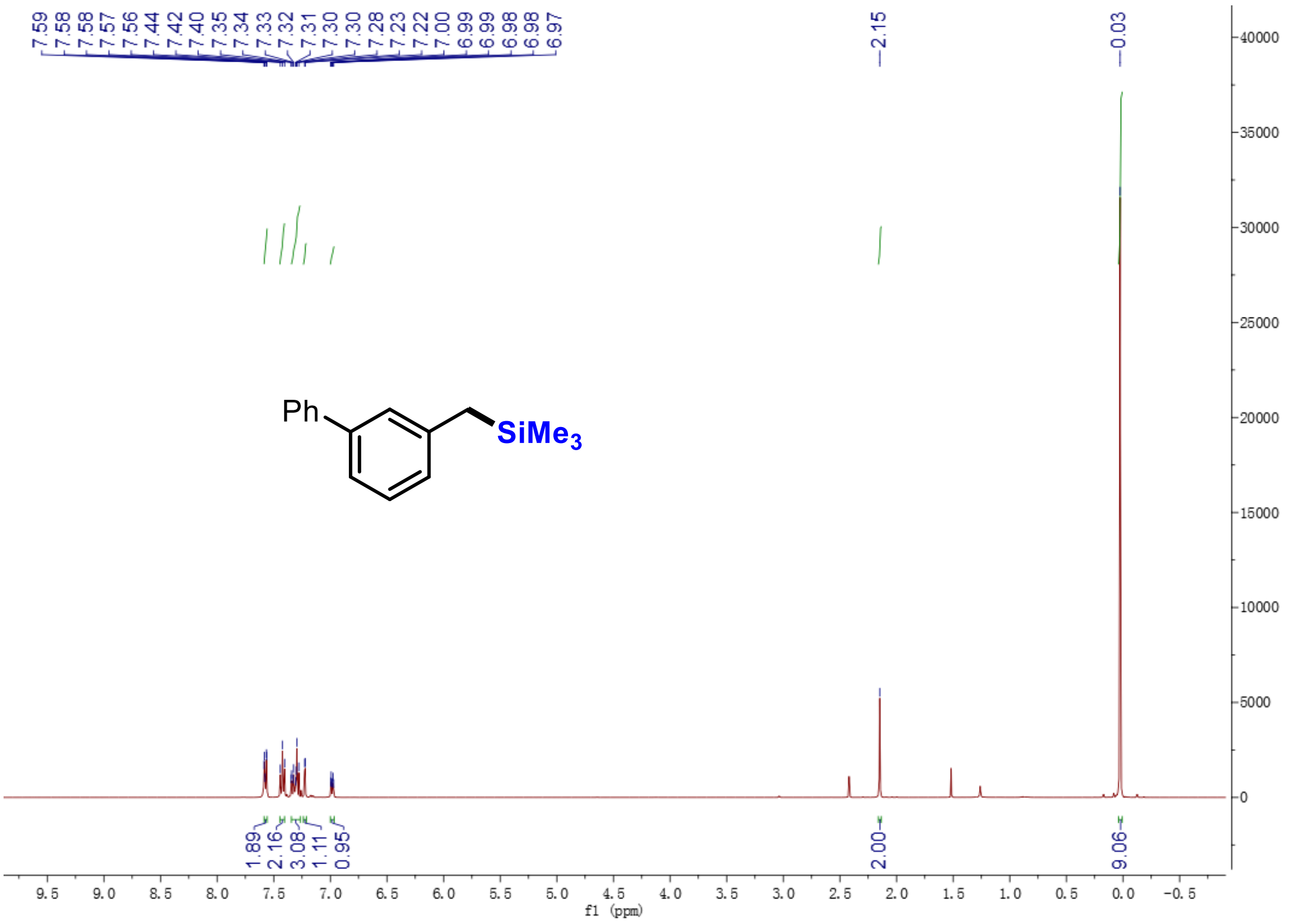

^{13}C NMR spectrum of 3m (101 MHz, $CDCl_3$)

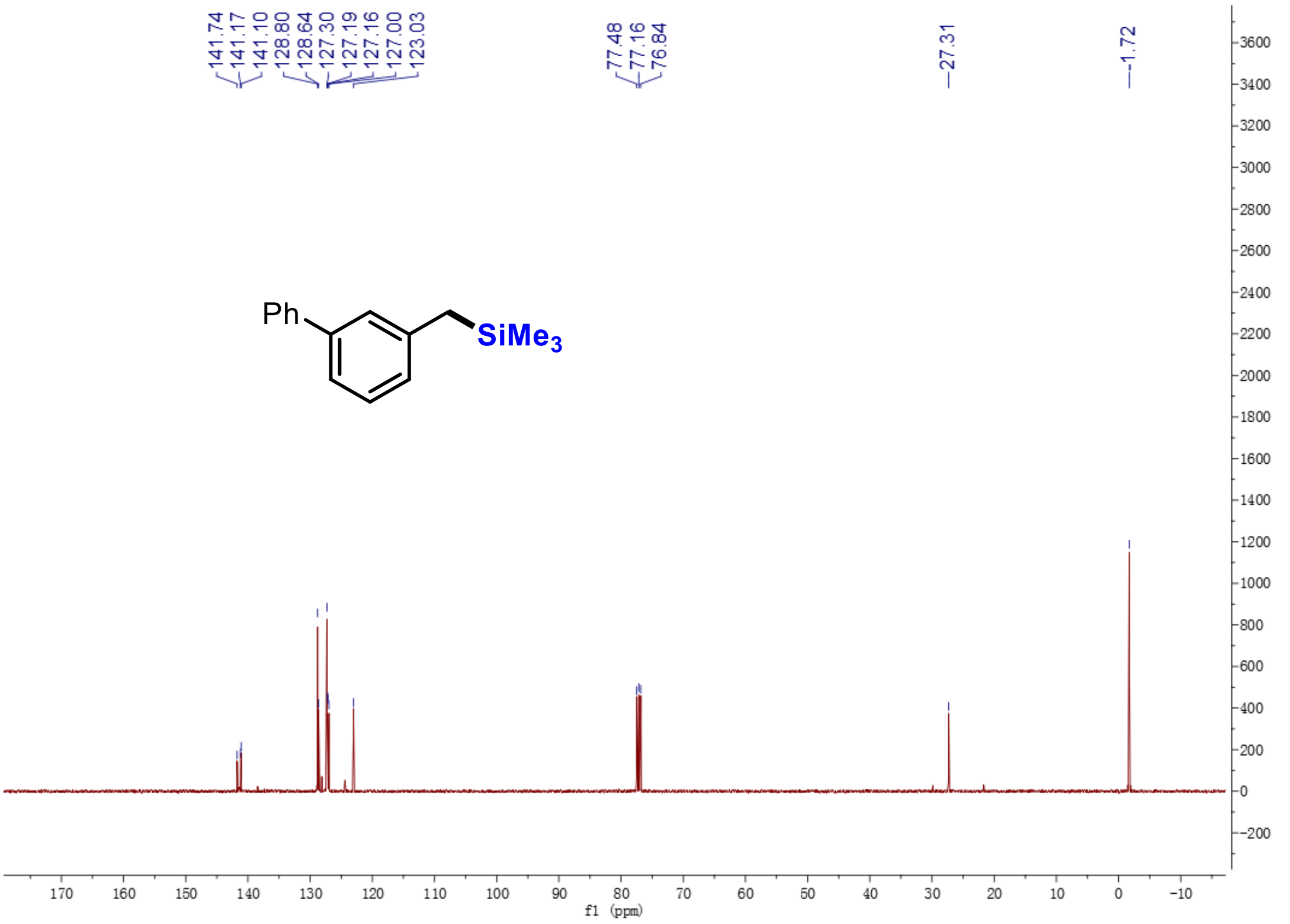

^{1}H NMR spectrum of 3n (400 MHz, $CDCl_3$)

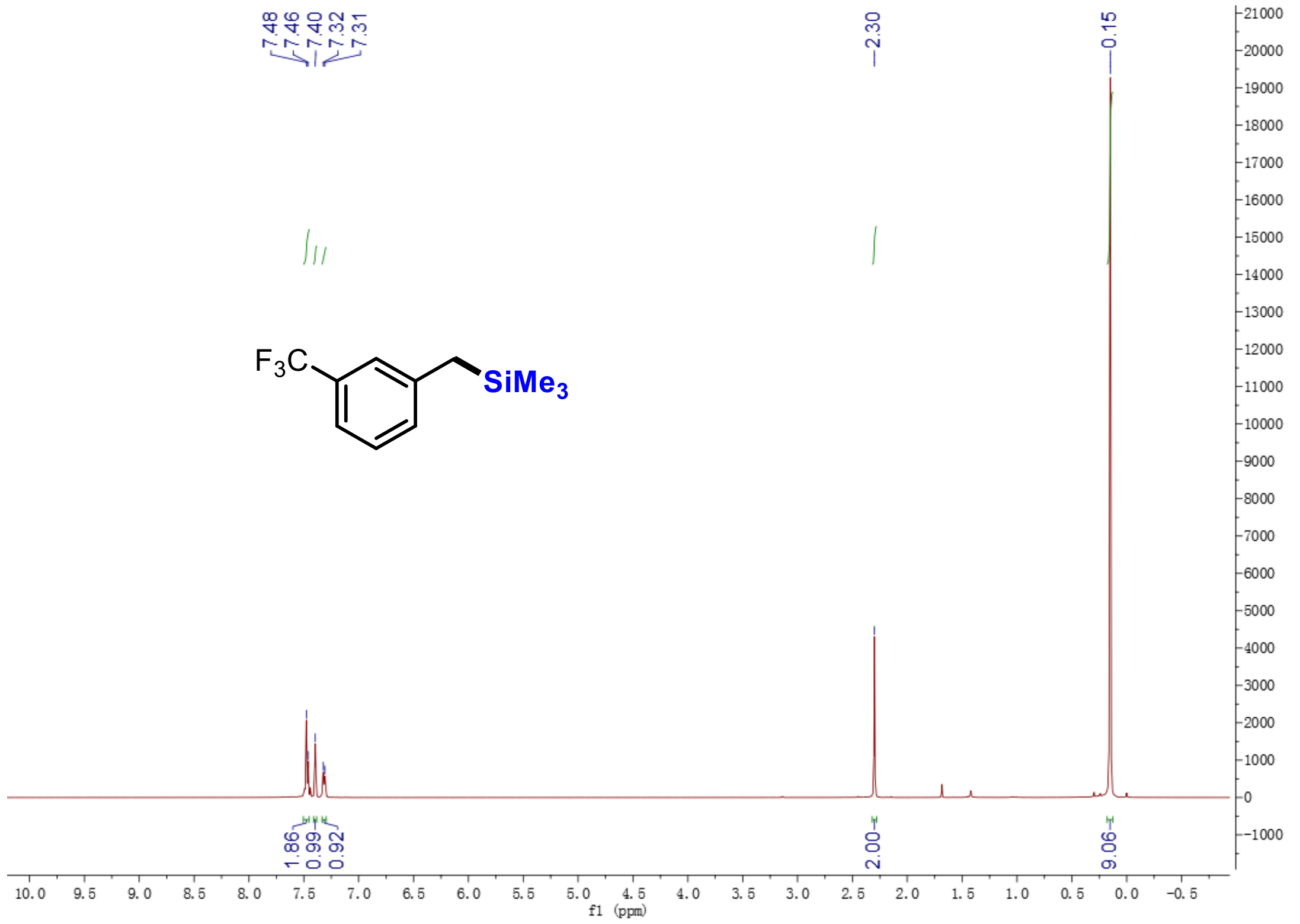

^{13}C NMR spectrum of 3n (101 MHz, $CDCl_3$)

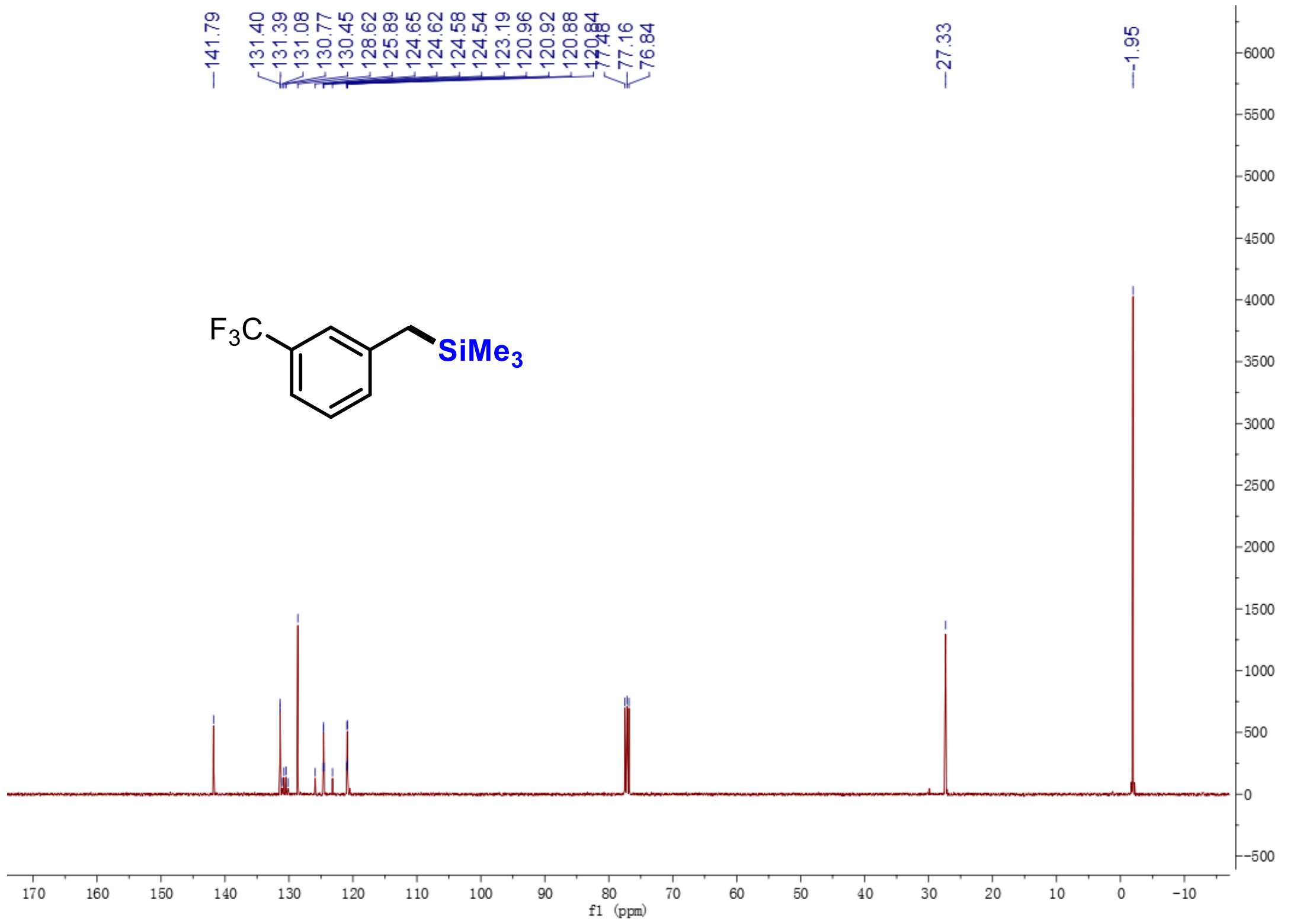

^{19}F NMR spectrum of 3n

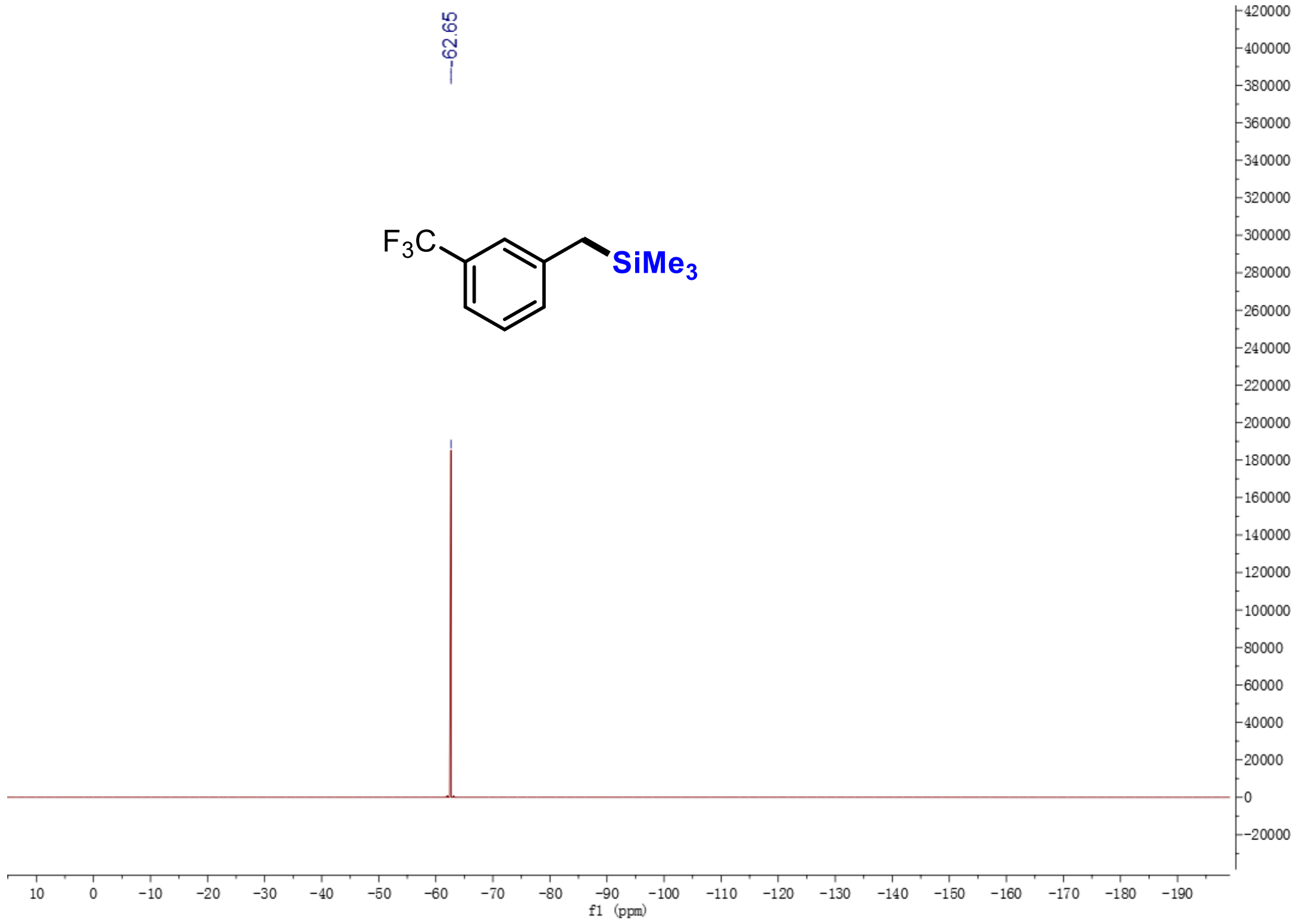

^{1}H NMR spectrum of 3o (400 MHz, $CDCl_3$)

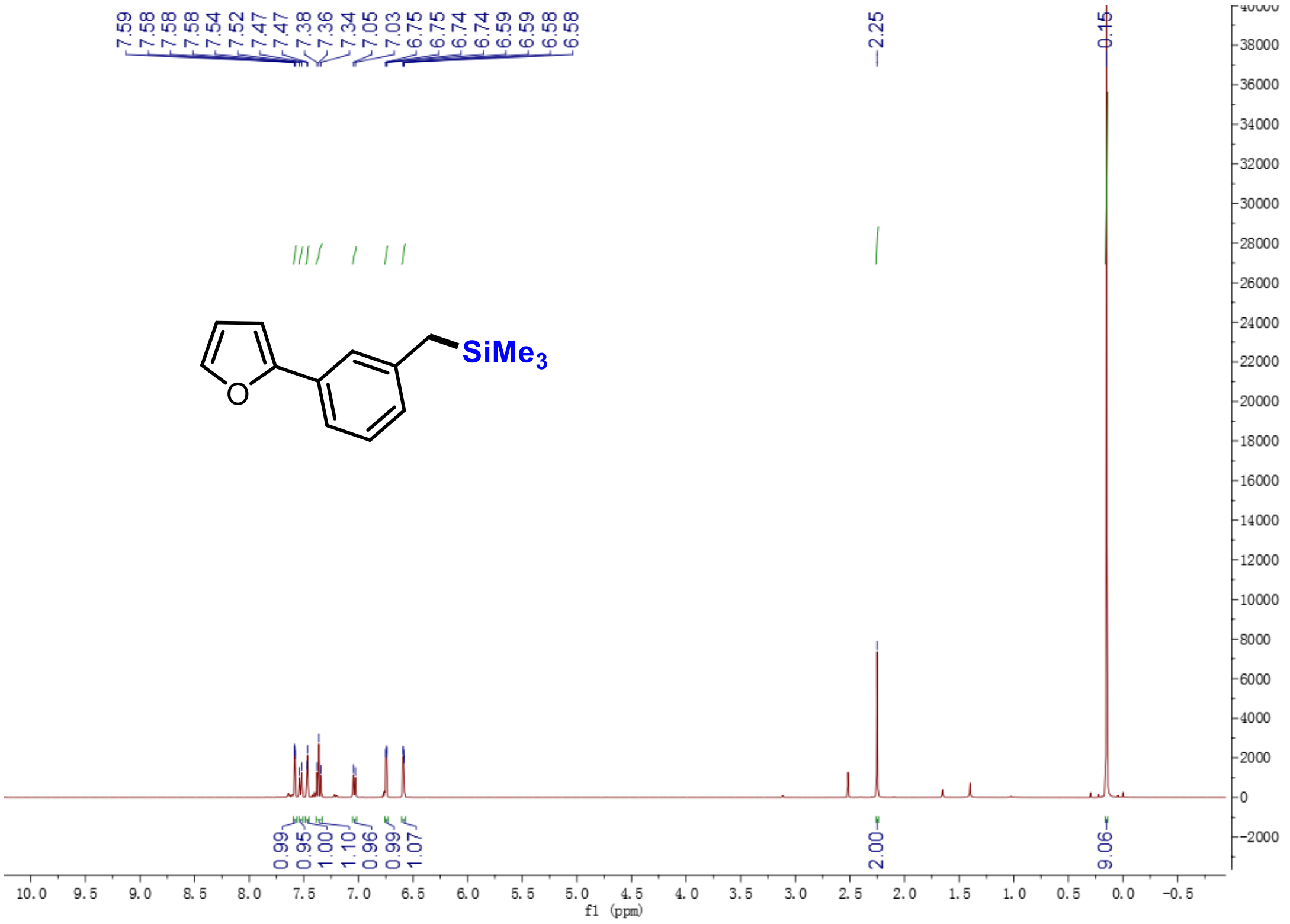

^{13}C NMR spectrum of 3o (101 MHz, $CDCl_3$)

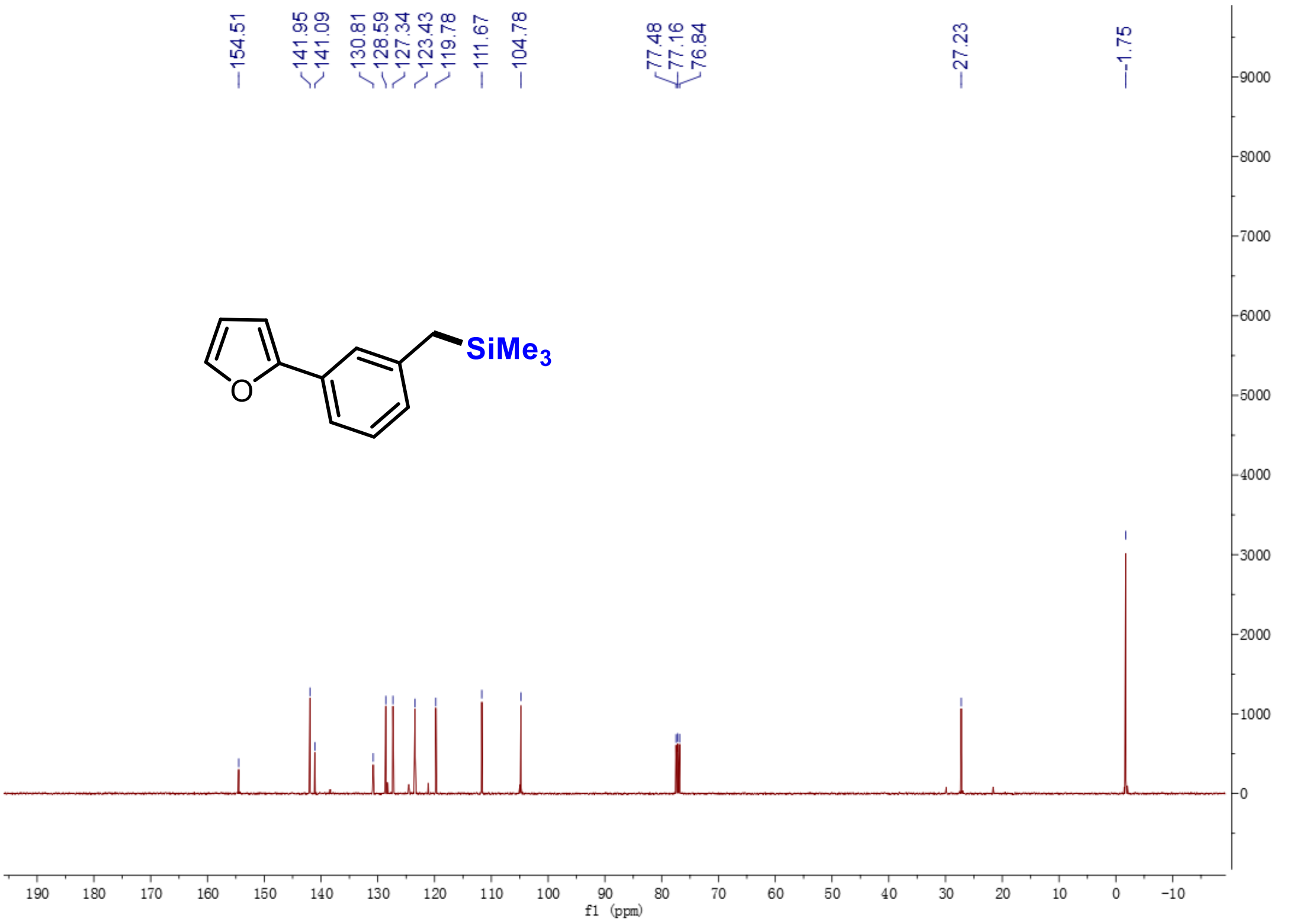

^{1}H NMR spectrum of 3p (400 MHz, $CDCl_3$)

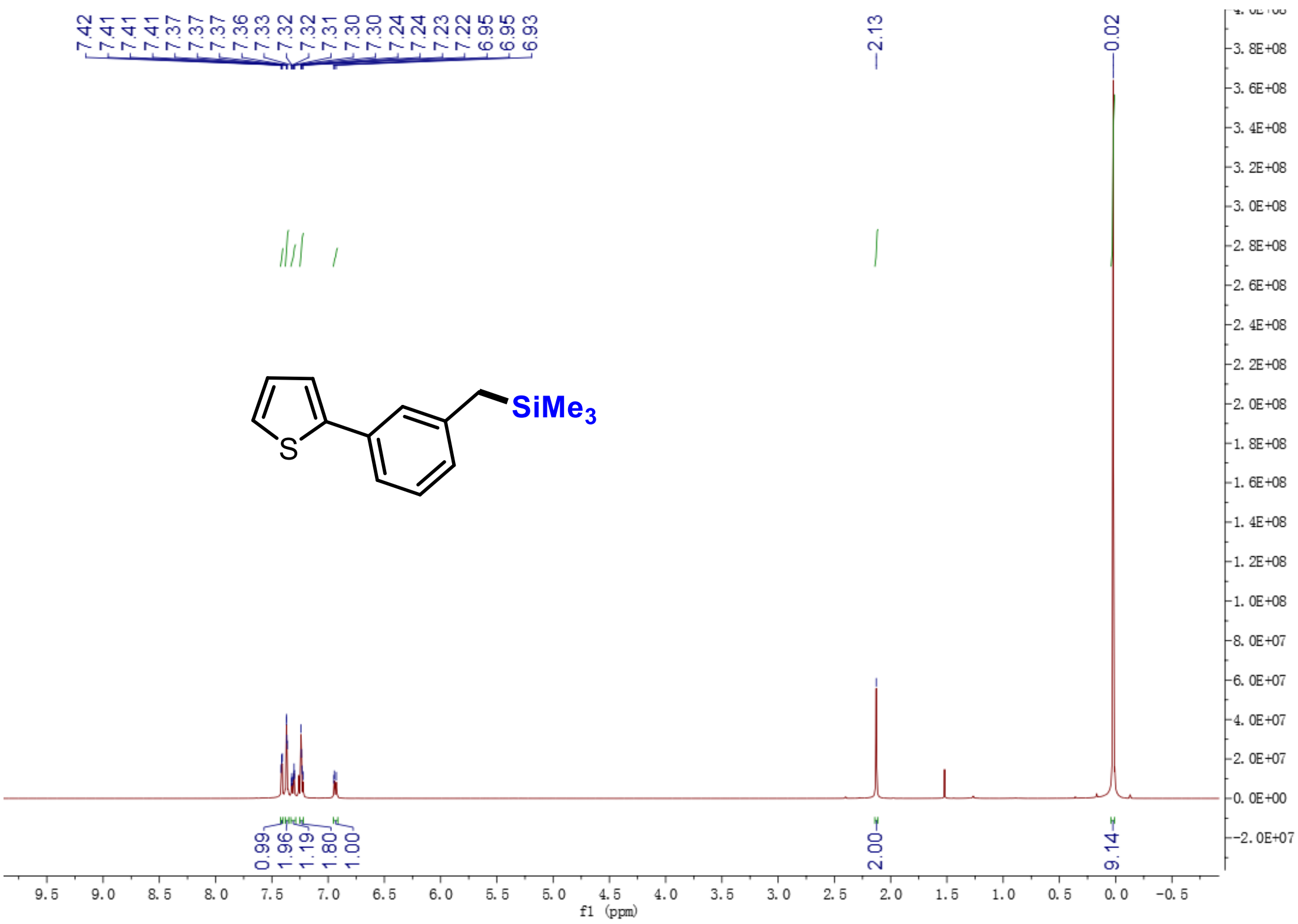

^{13}C NMR spectrum of 3p (101 MHz, $CDCl_3$)

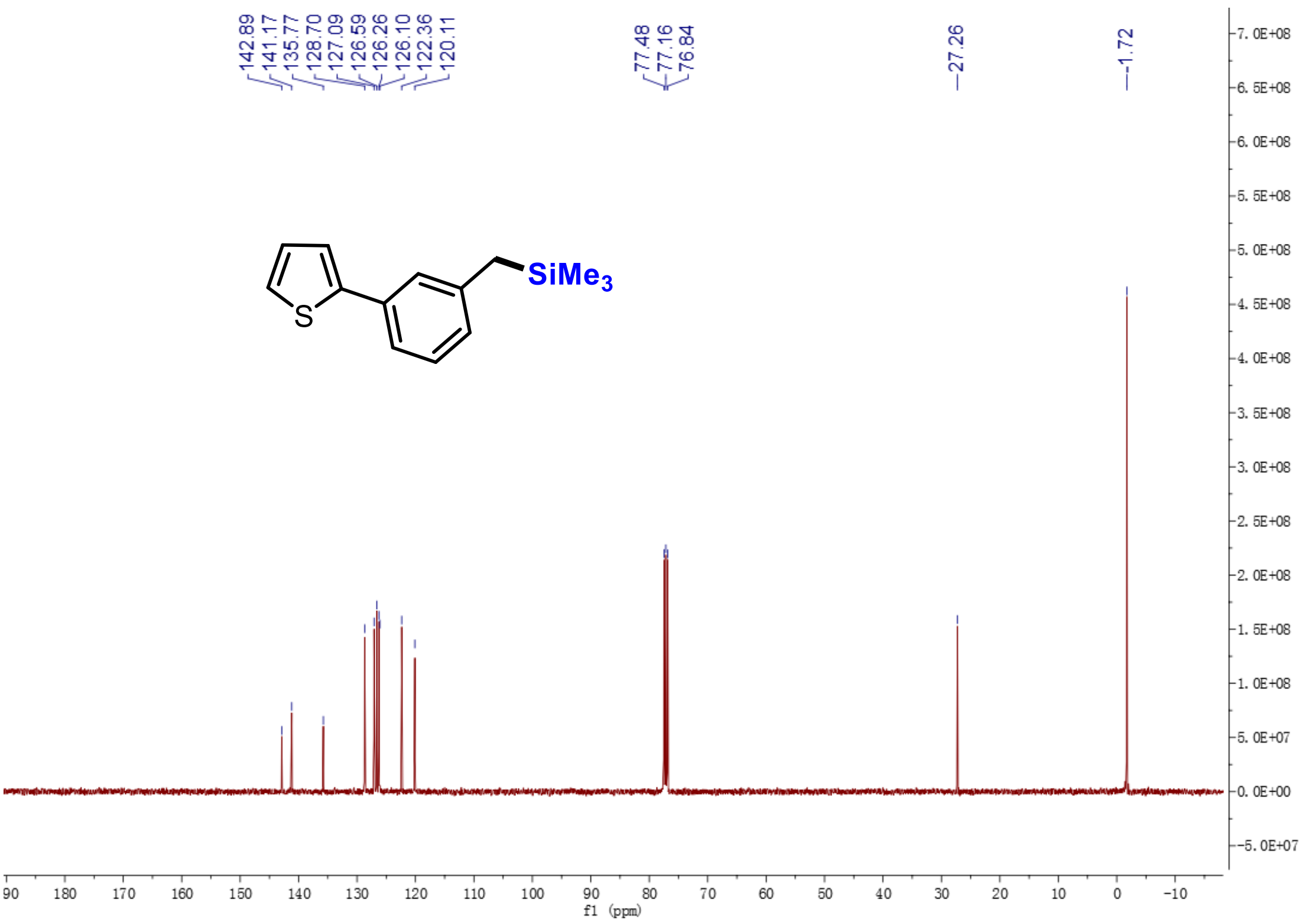

^{1}H NMR spectrum of 3q (400 MHz, $CDCl_3$)

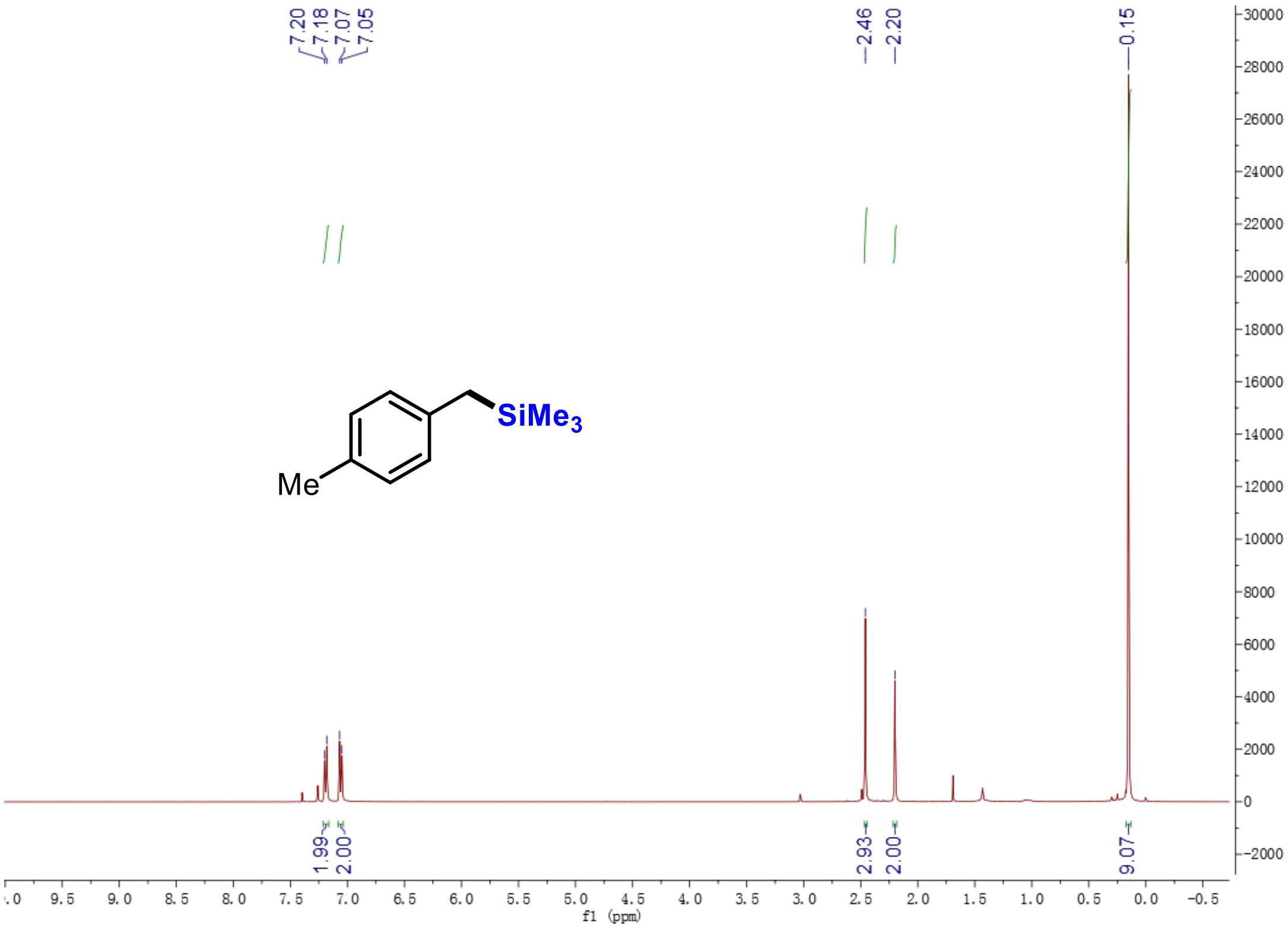

^{13}C NMR spectrum of 3q (101 MHz, $CDCl_3$)

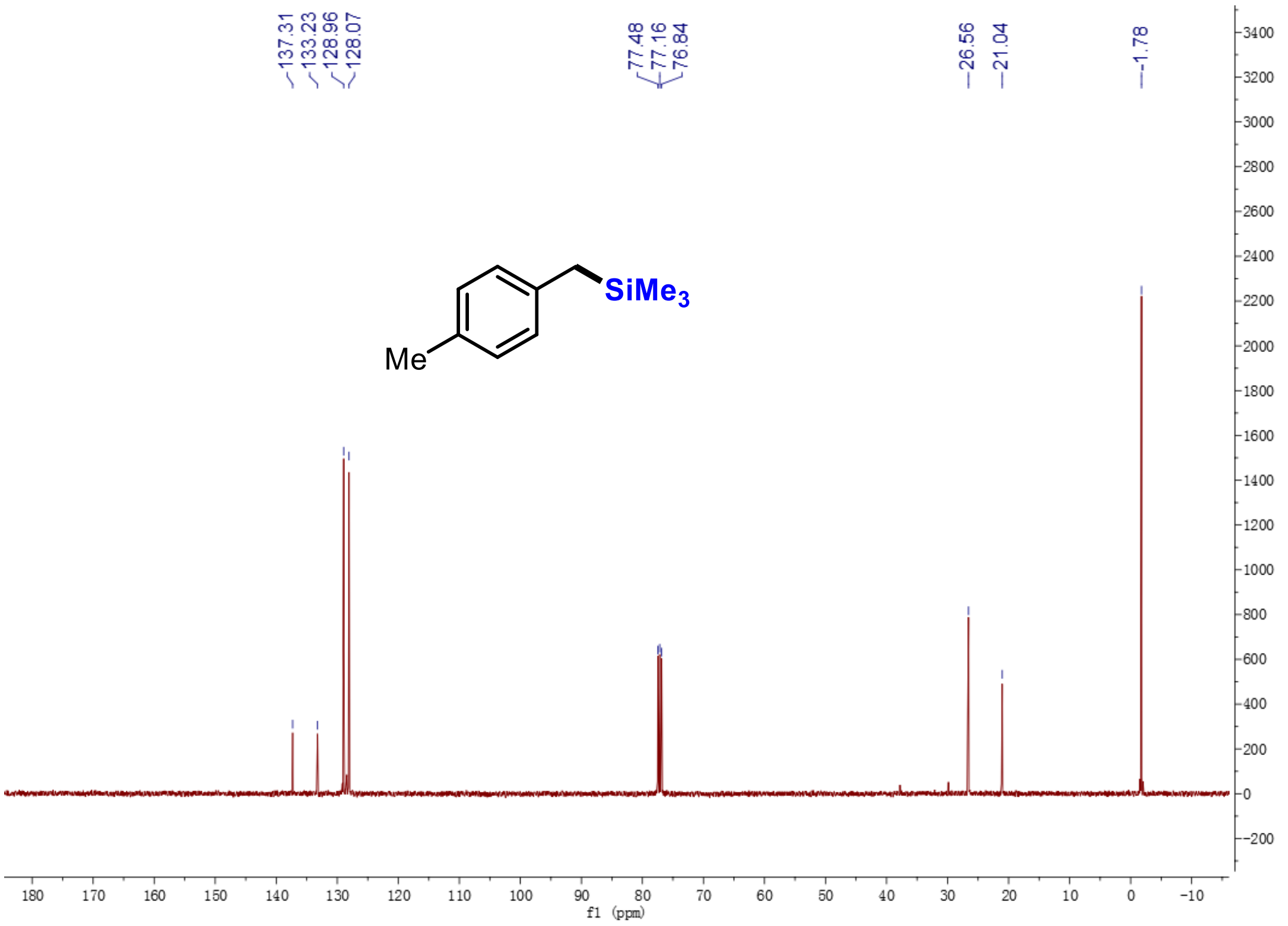

^{1}H NMR spectrum of 3r (400 MHz, $CDCl_3$)

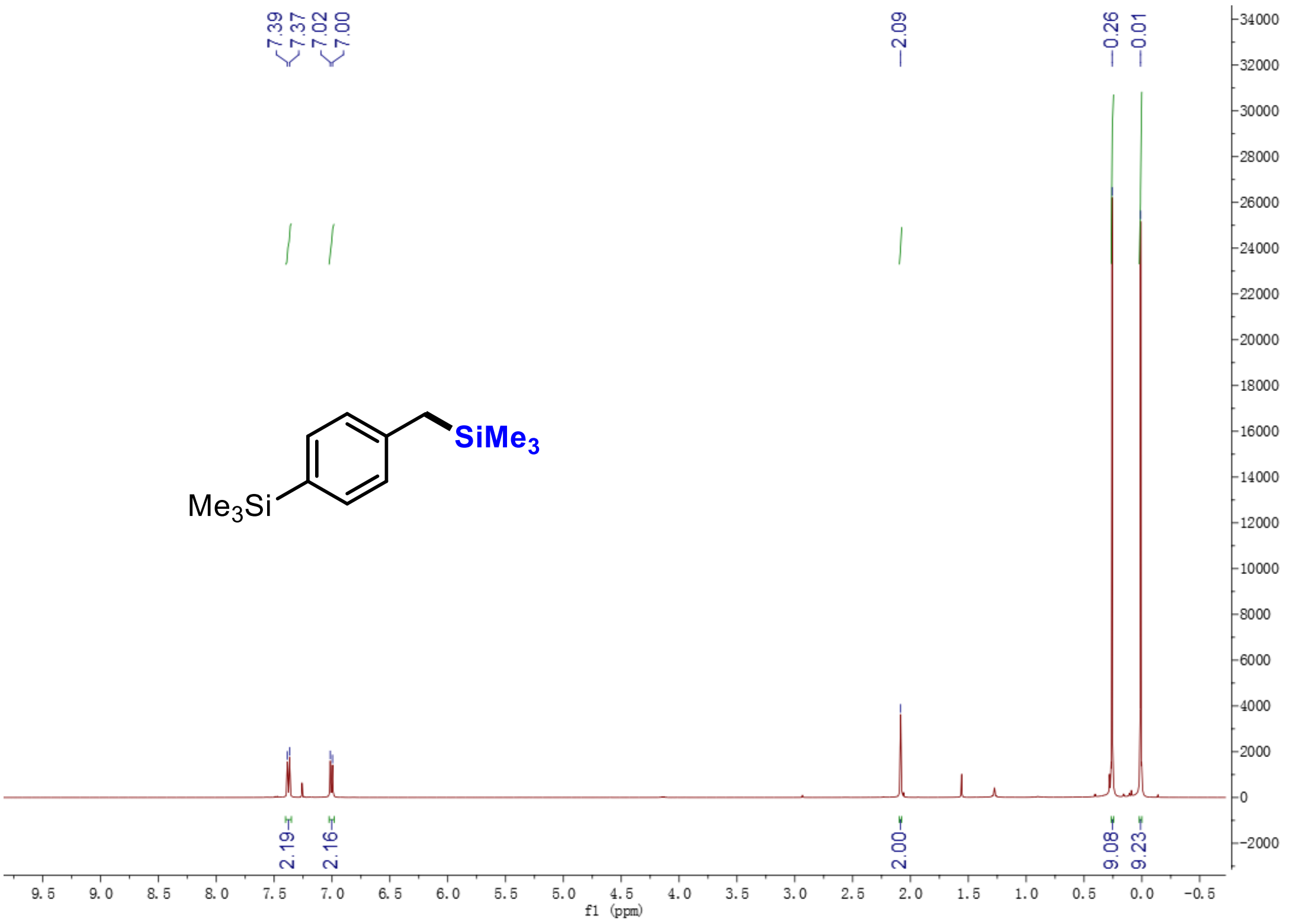

^{13}C NMR spectrum of 3r (101 MHz, $CDCl_3$)

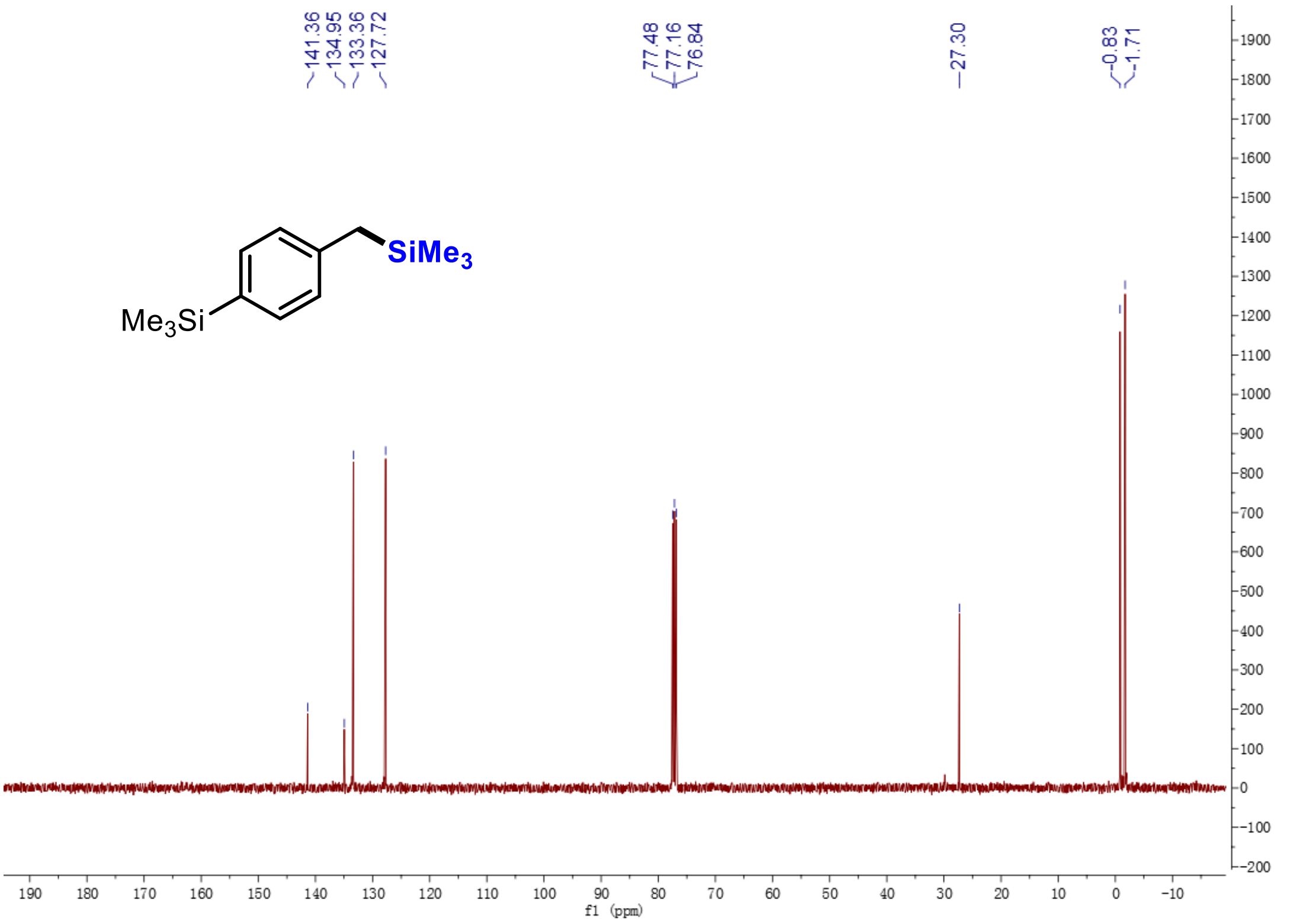

^{1}H NMR spectrum of 3s (400 MHz, $CDCl_3$)

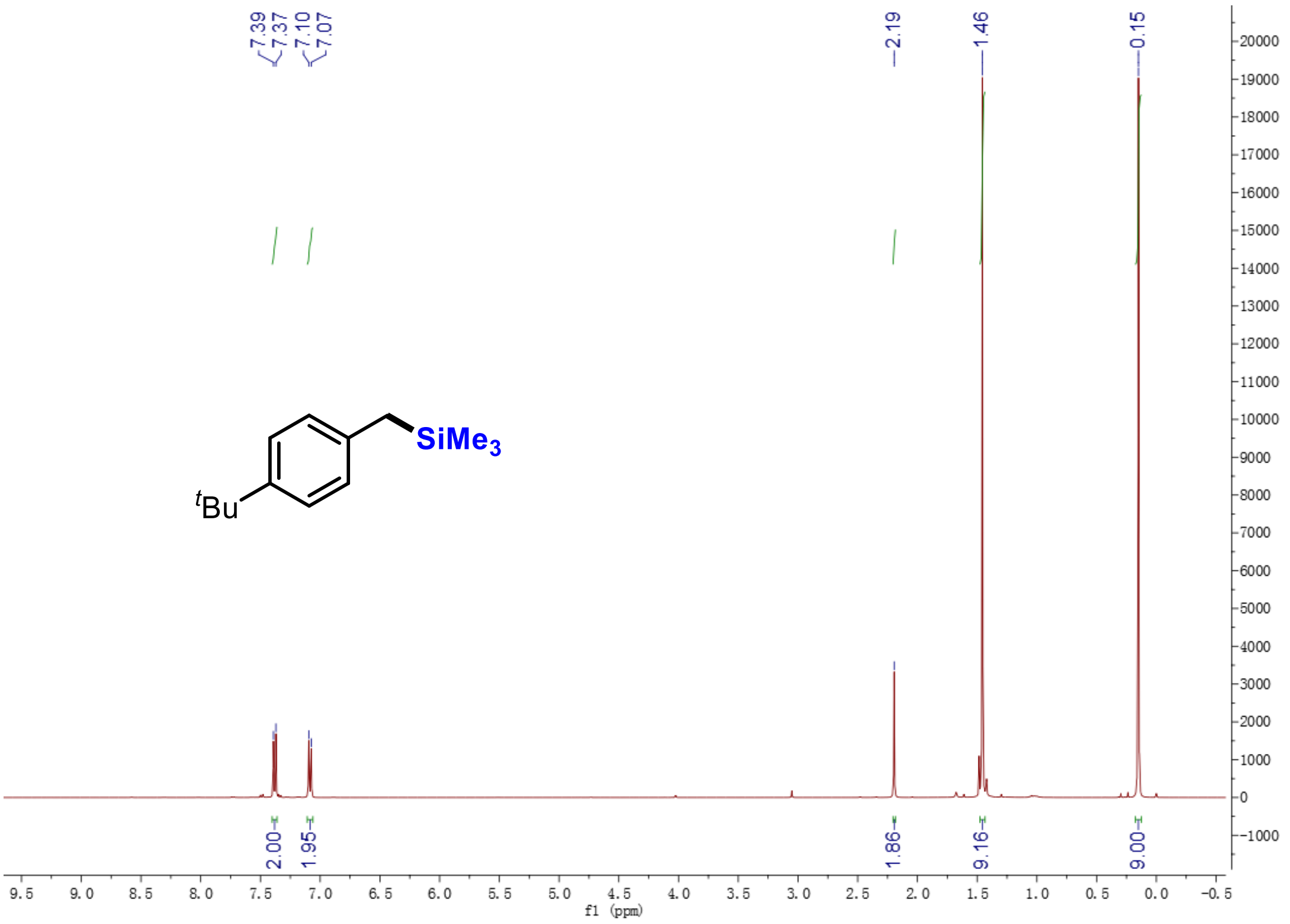

^{13}C NMR spectrum of 3s (101 MHz, $CDCl_3$)

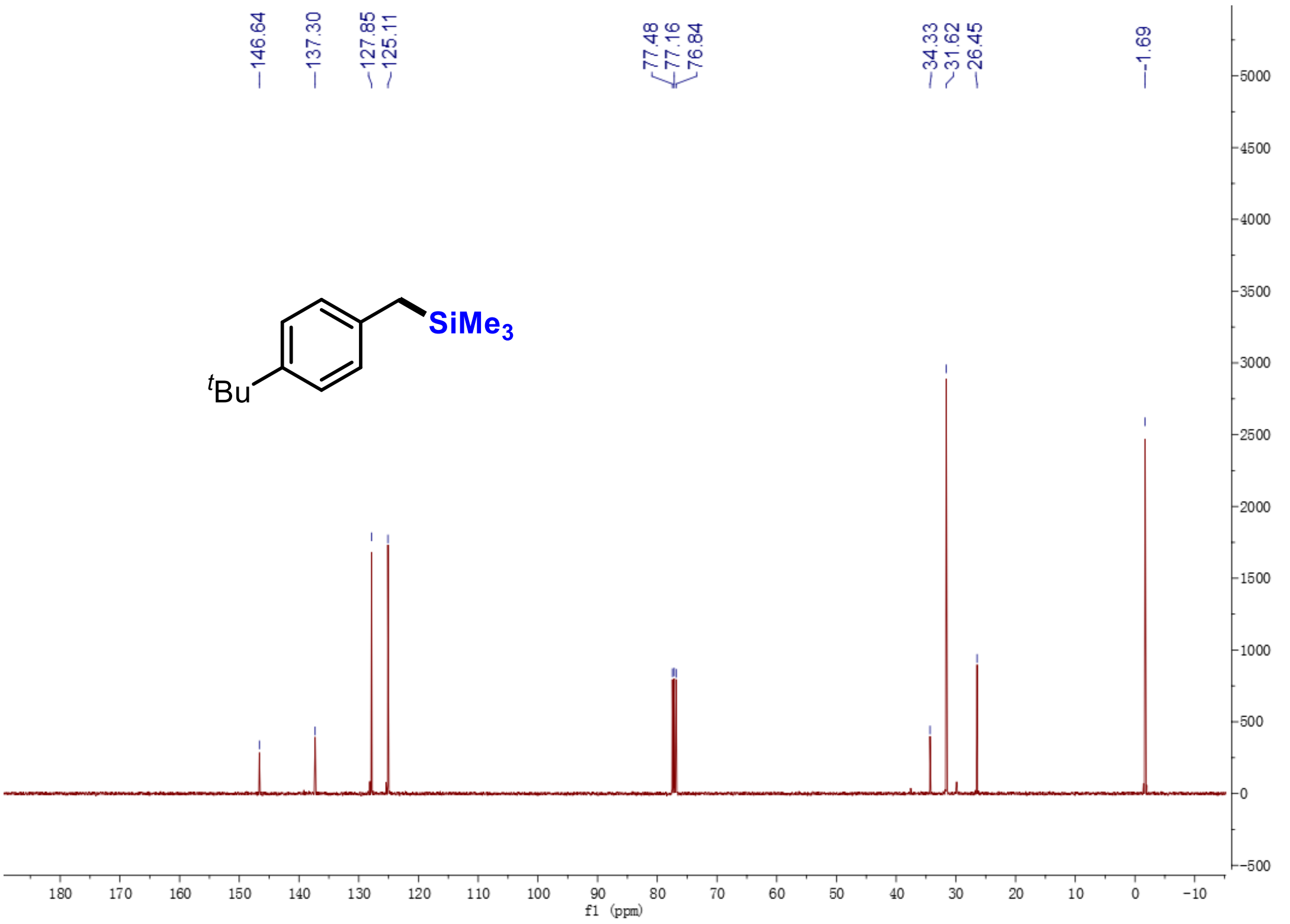

^{1}H NMR spectrum of 3t (400 MHz, $CDCl_3$)

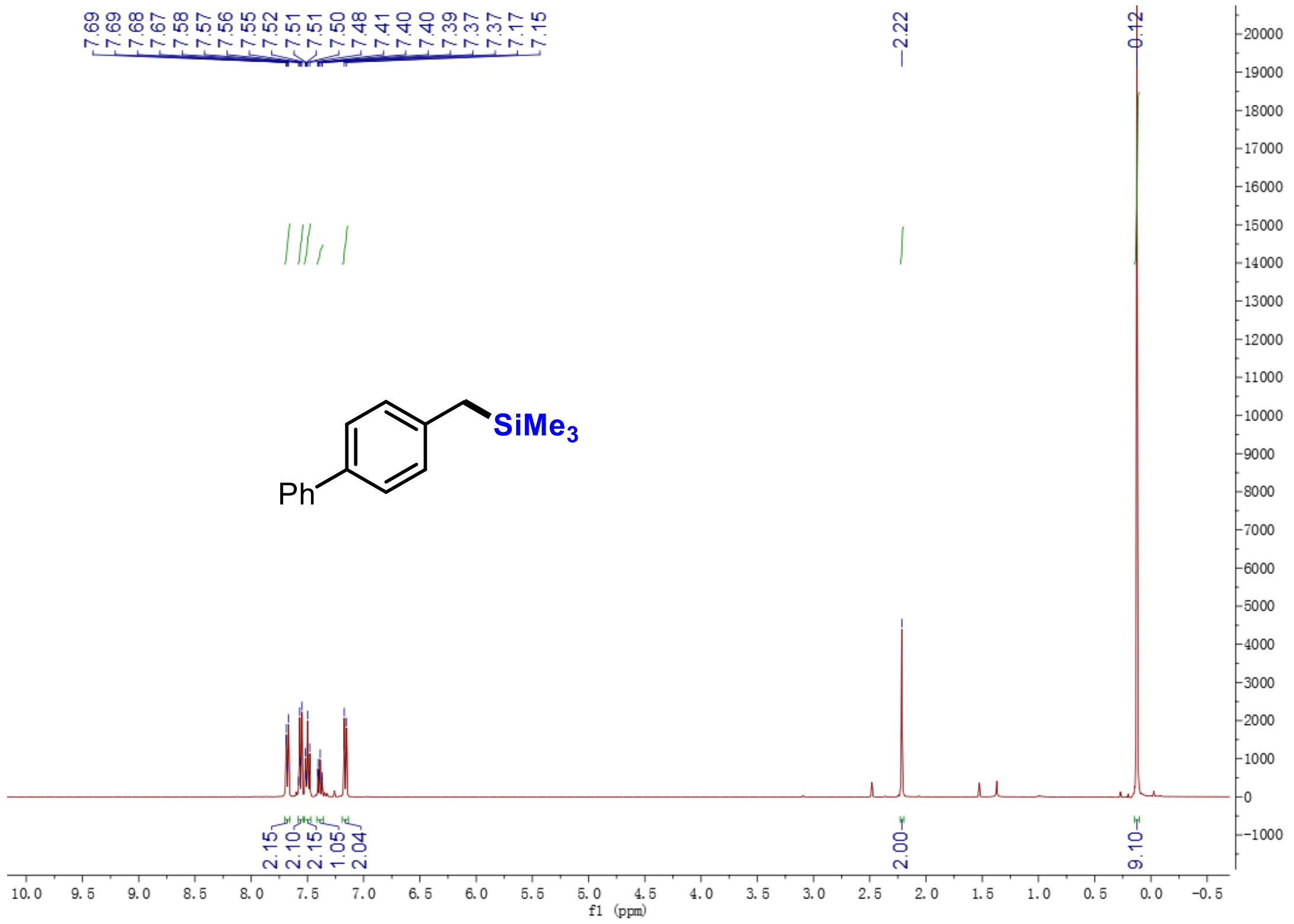

^{13}C NMR spectrum of 3t (101 MHz, $CDCl_3$)

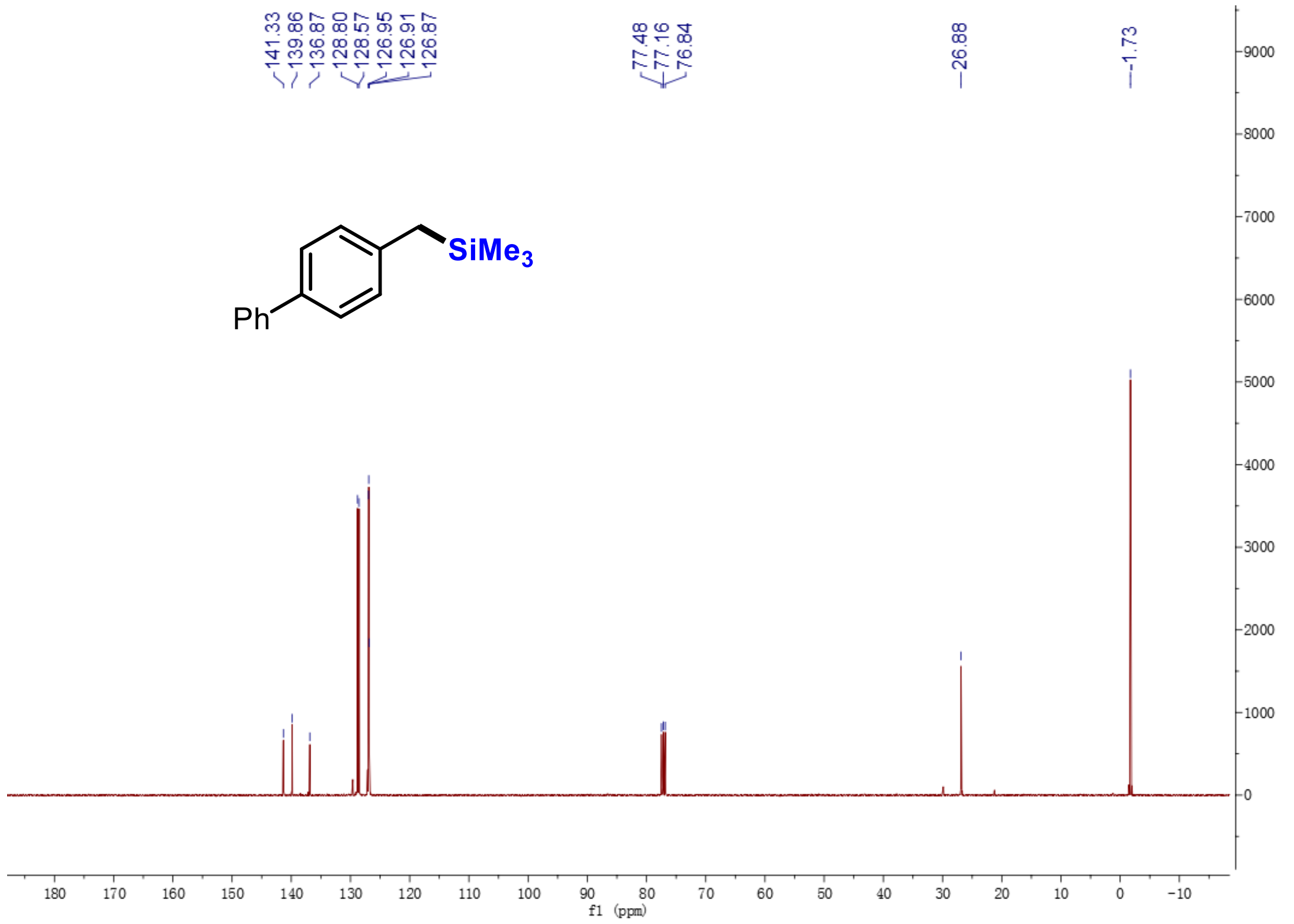

^{1}H NMR spectrum of 3u (400 MHz, $CDCl_3$)

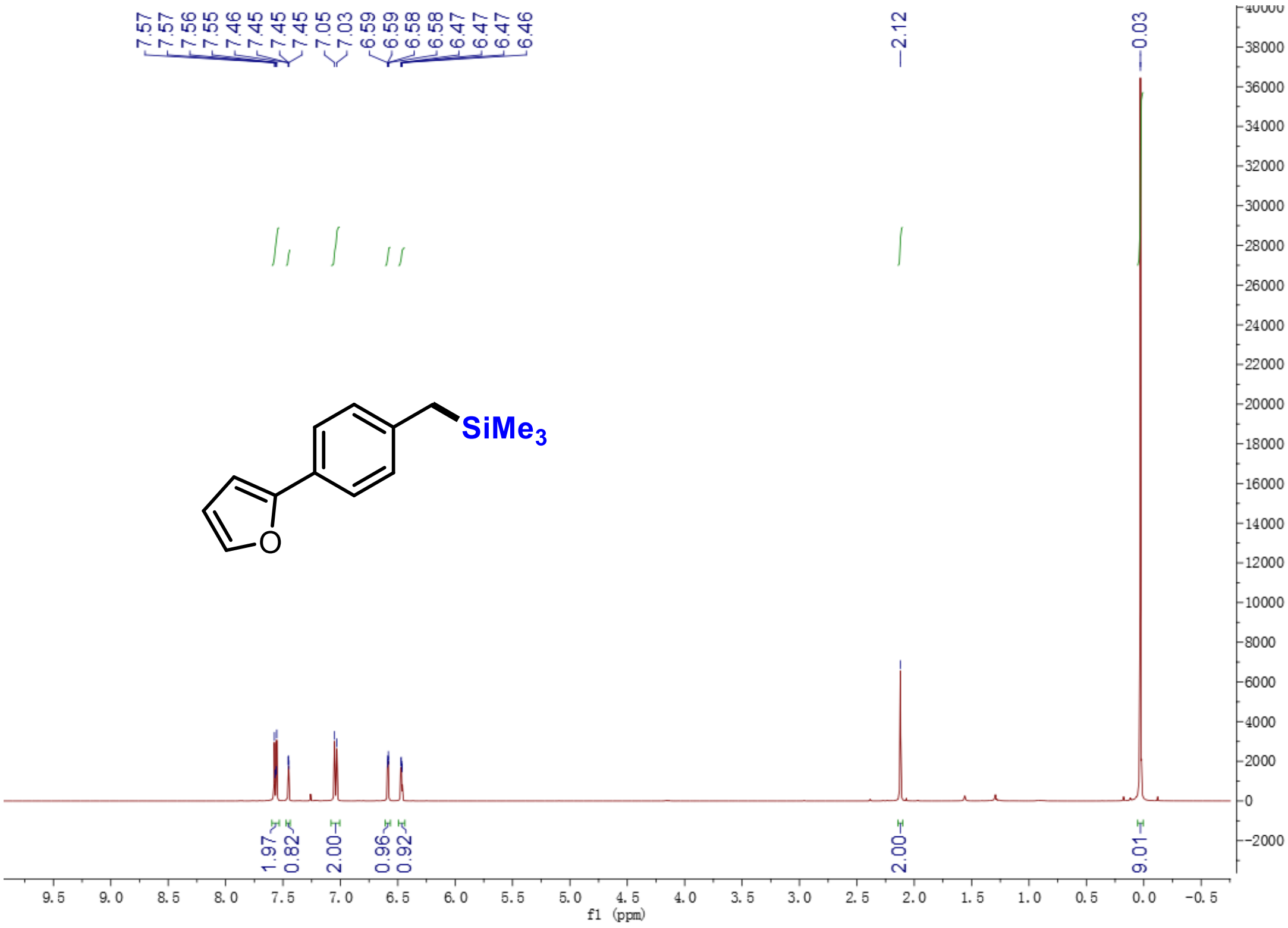

^{13}C NMR spectrum of 3u (101 MHz, $CDCl_3$)

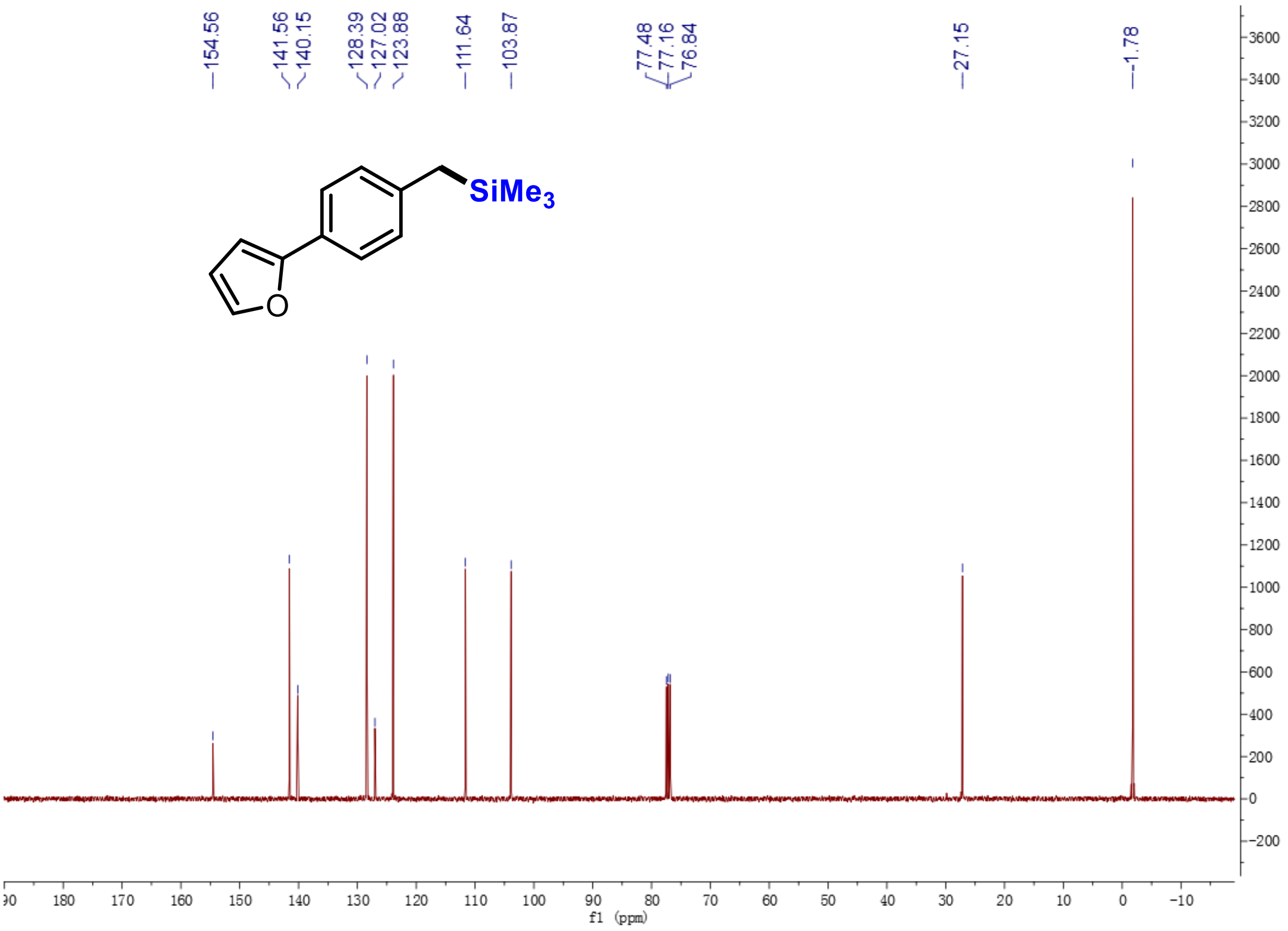

^{1}H NMR spectrum of 3v (400 MHz, $CDCl_3$)

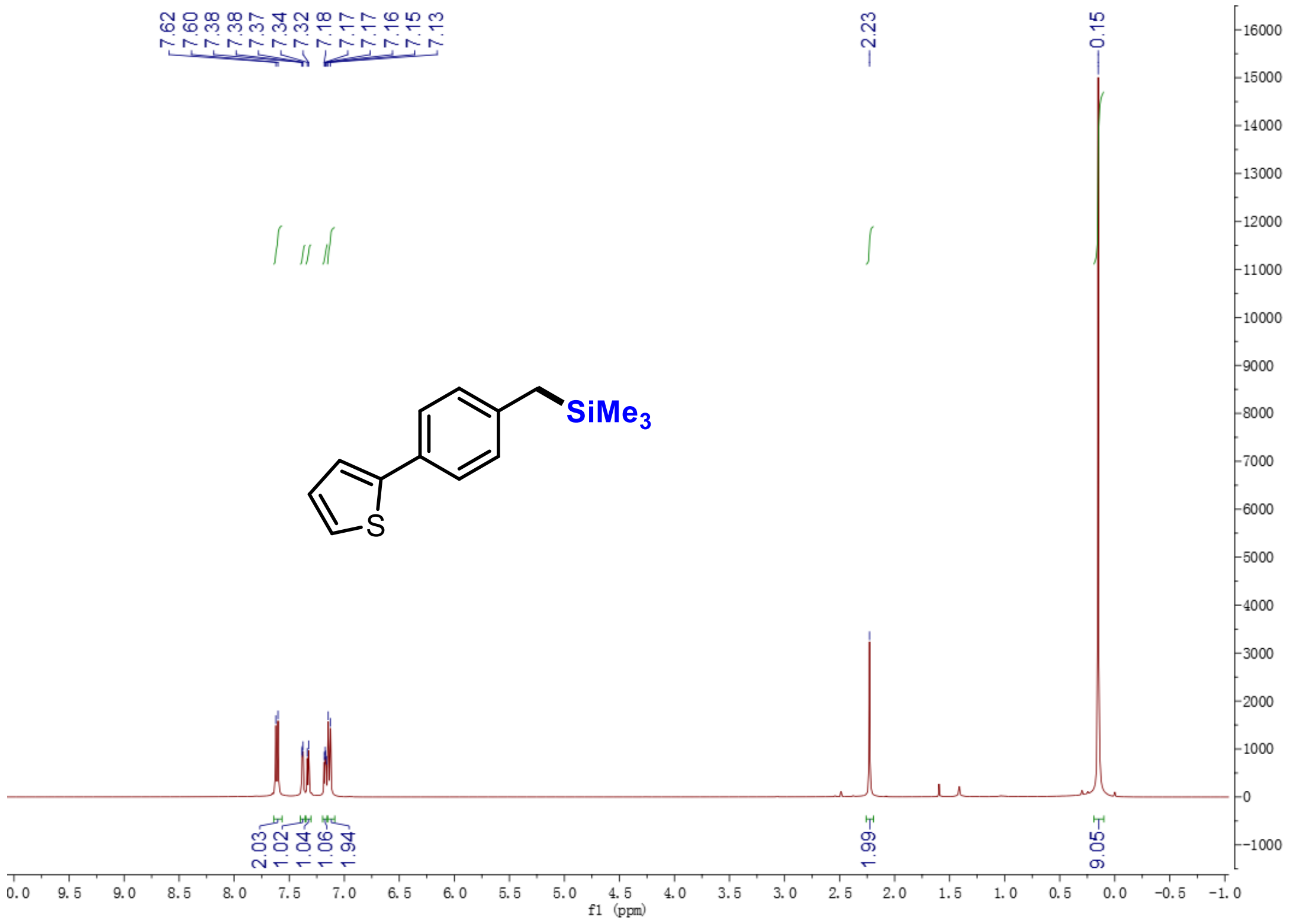

^{13}C NMR spectrum of 3v (101 MHz, $CDCl_3$)

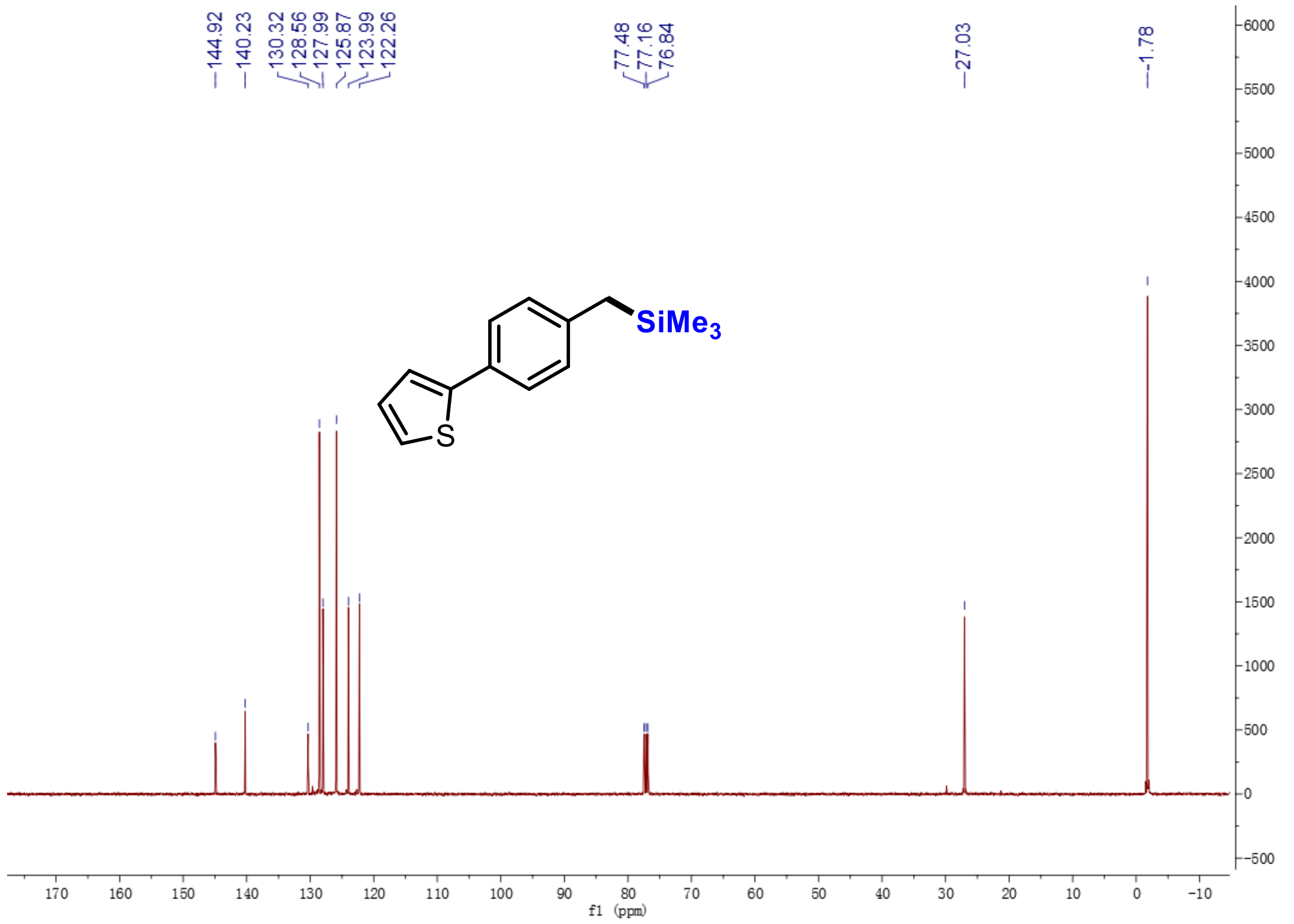

^{1}H NMR spectrum of 3w (400 MHz, $CDCl_3$)

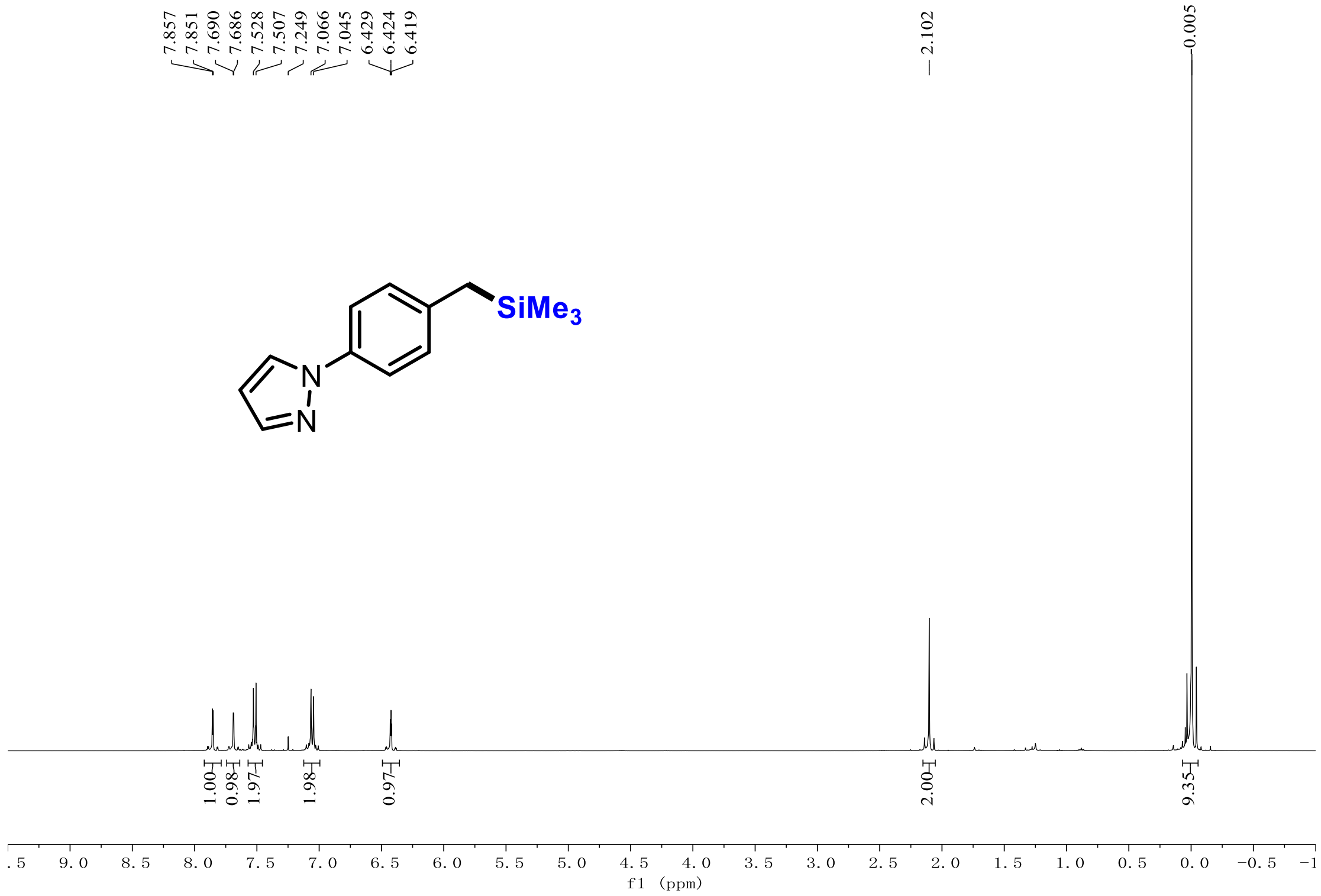

^{13}C NMR spectrum of 3w (101 MHz, $CDCl_3$)

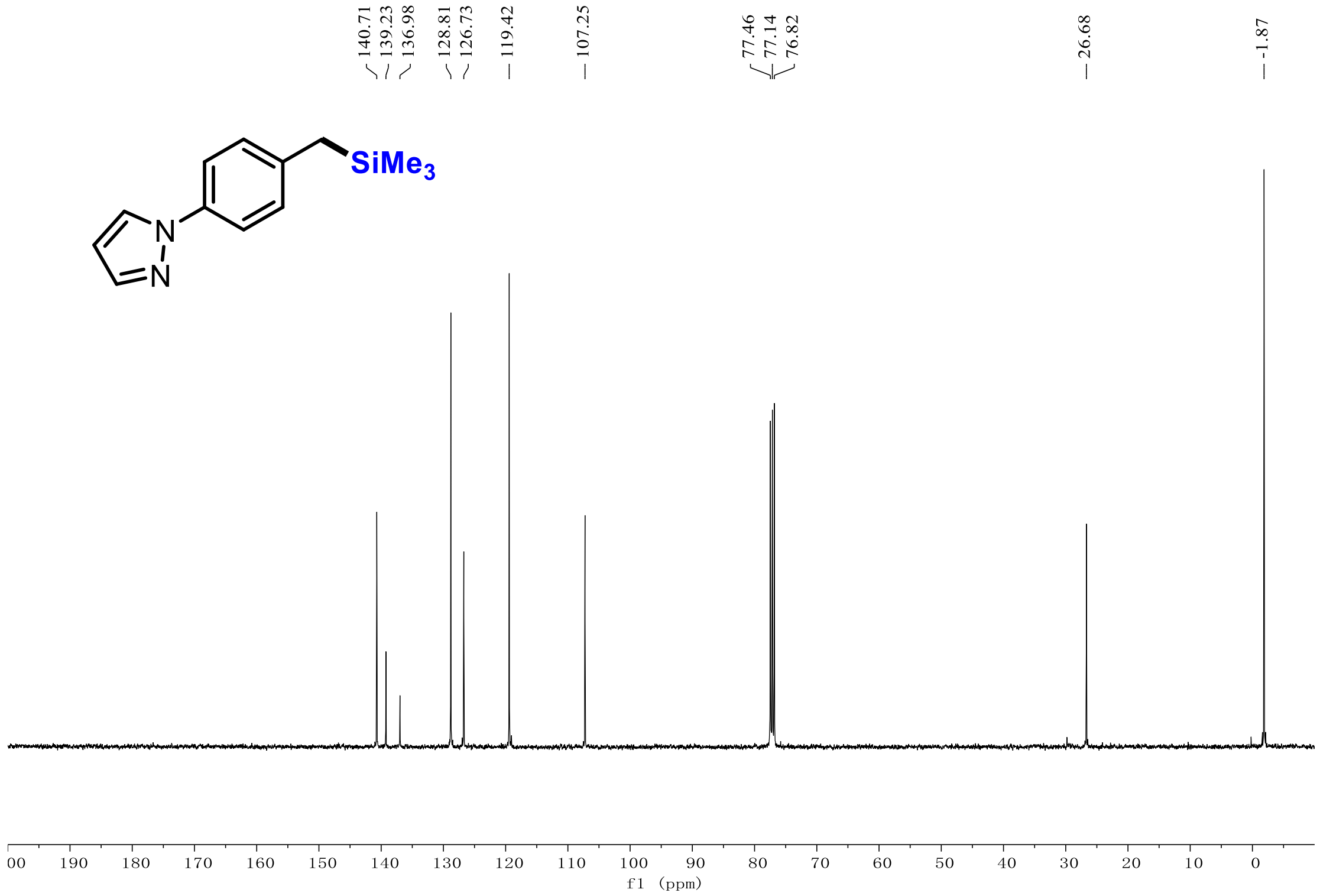

^{1}H NMR spectrum of 3x (400 MHz, $CDCl_3$)

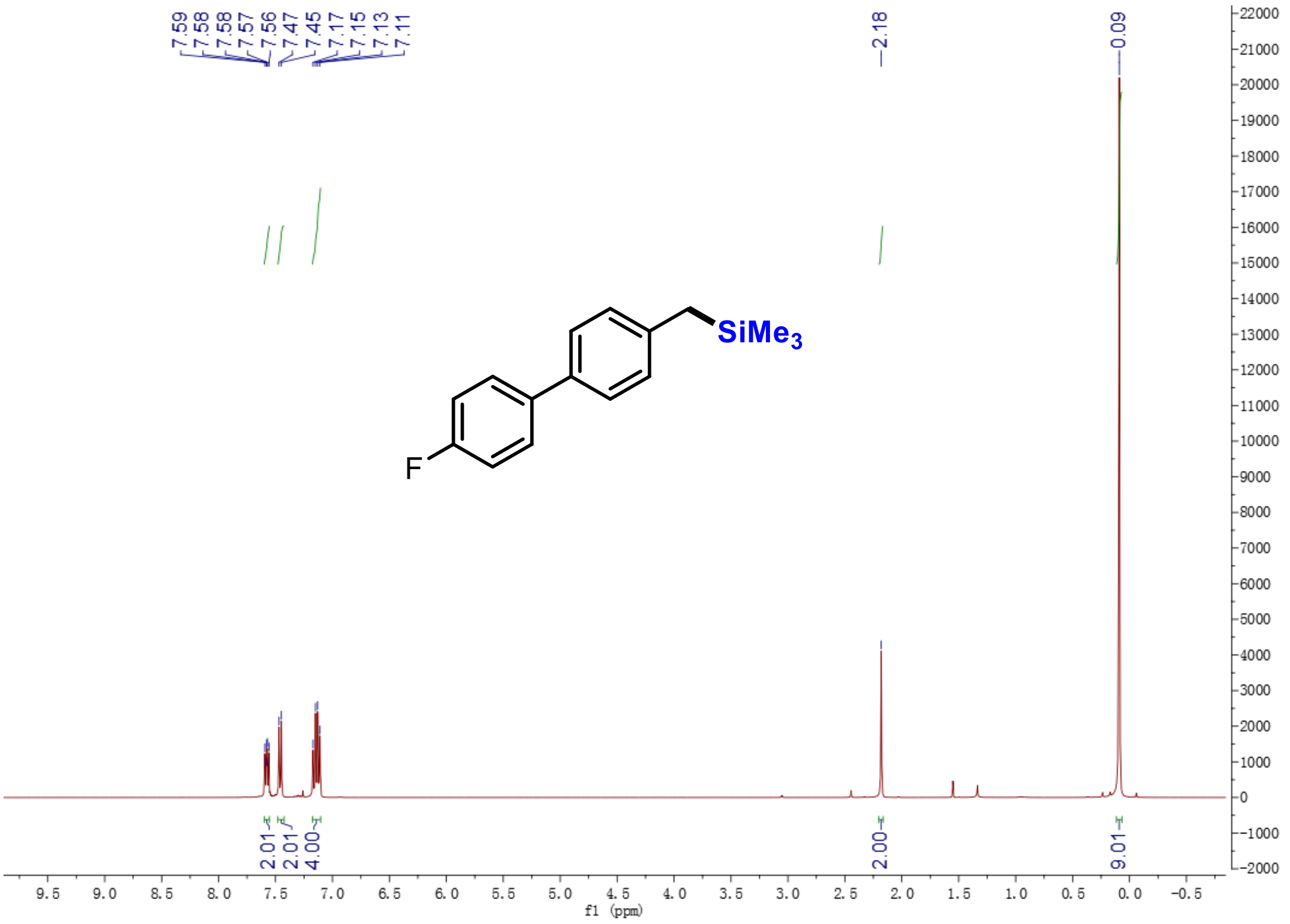

^{13}C NMR spectrum of 3x (101 MHz, $CDCl_3$)

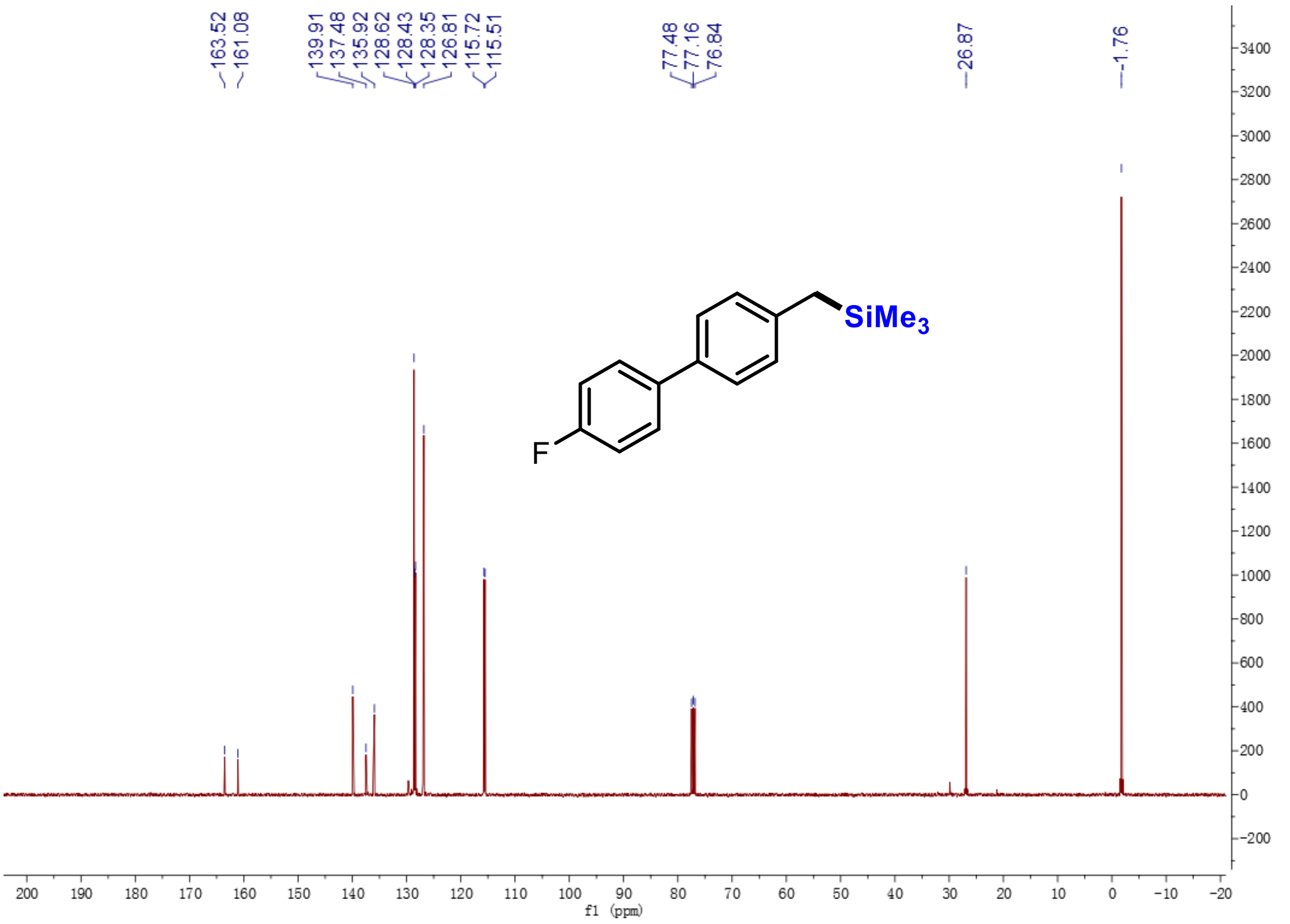

^{19}F NMR spectrum of 3x

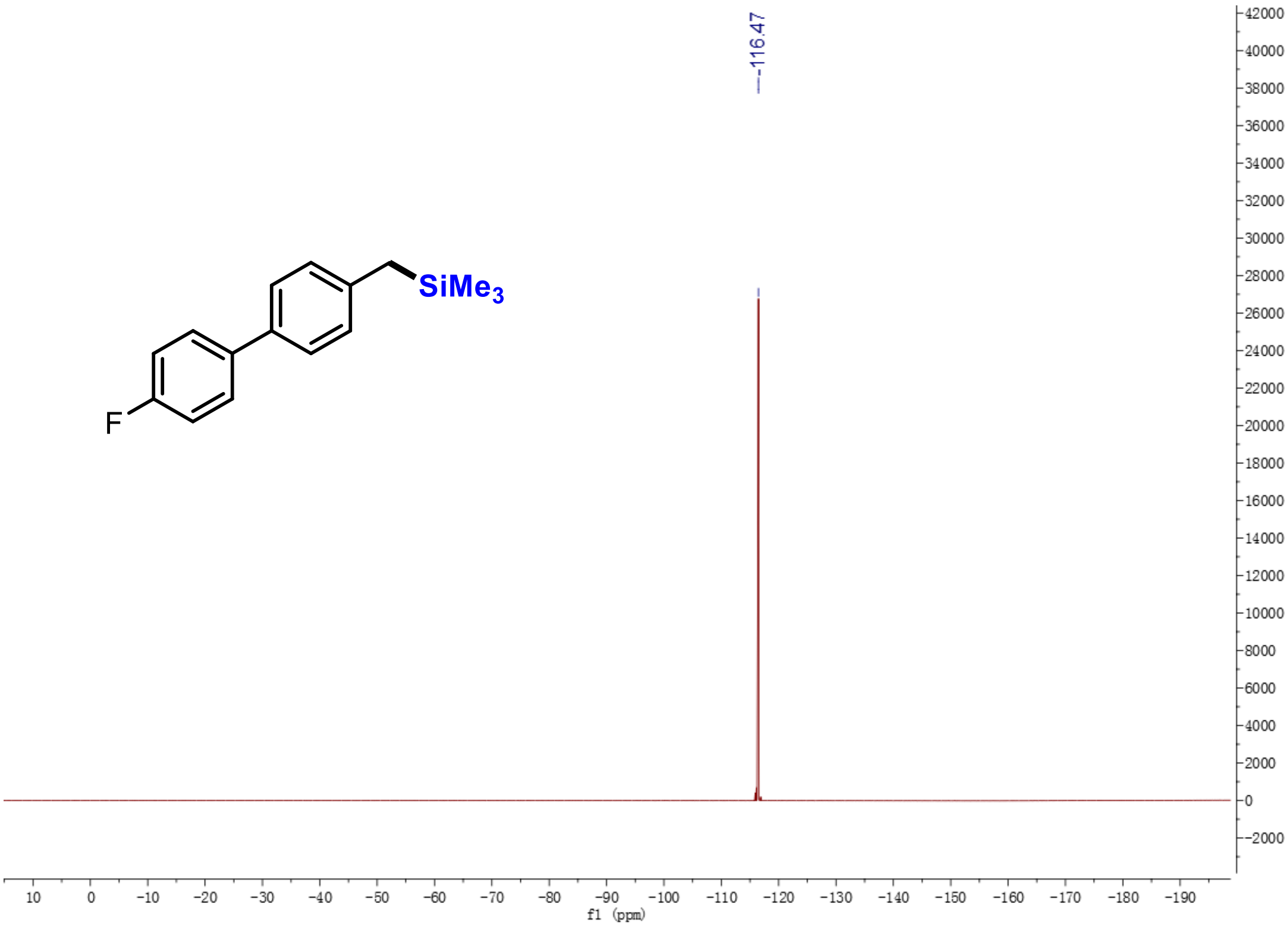

^{1}H NMR spectrum of 3y (400 MHz, $CDCl_3$)

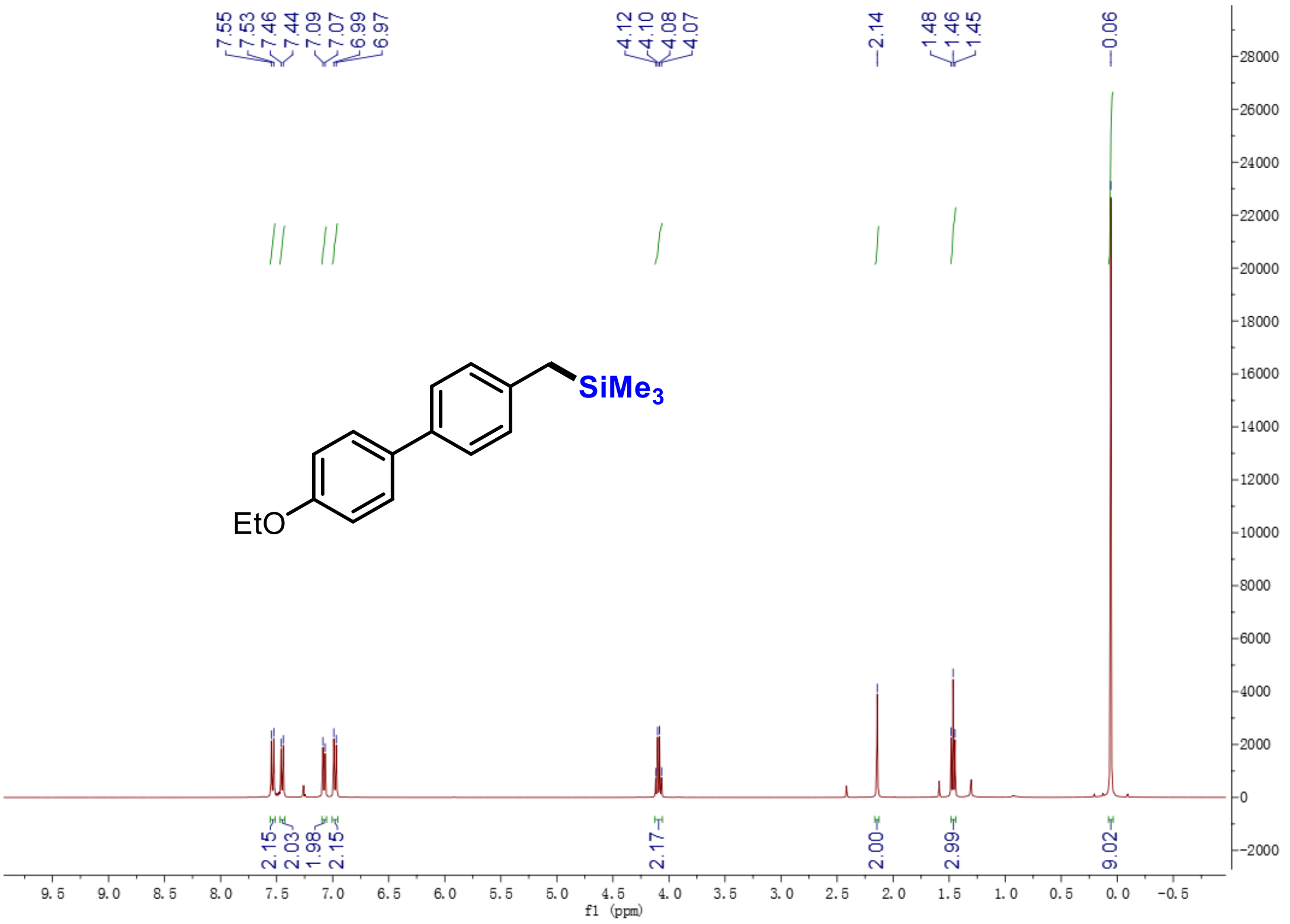

^{13}C NMR spectrum of 3y (101 MHz, $CDCl_3$)

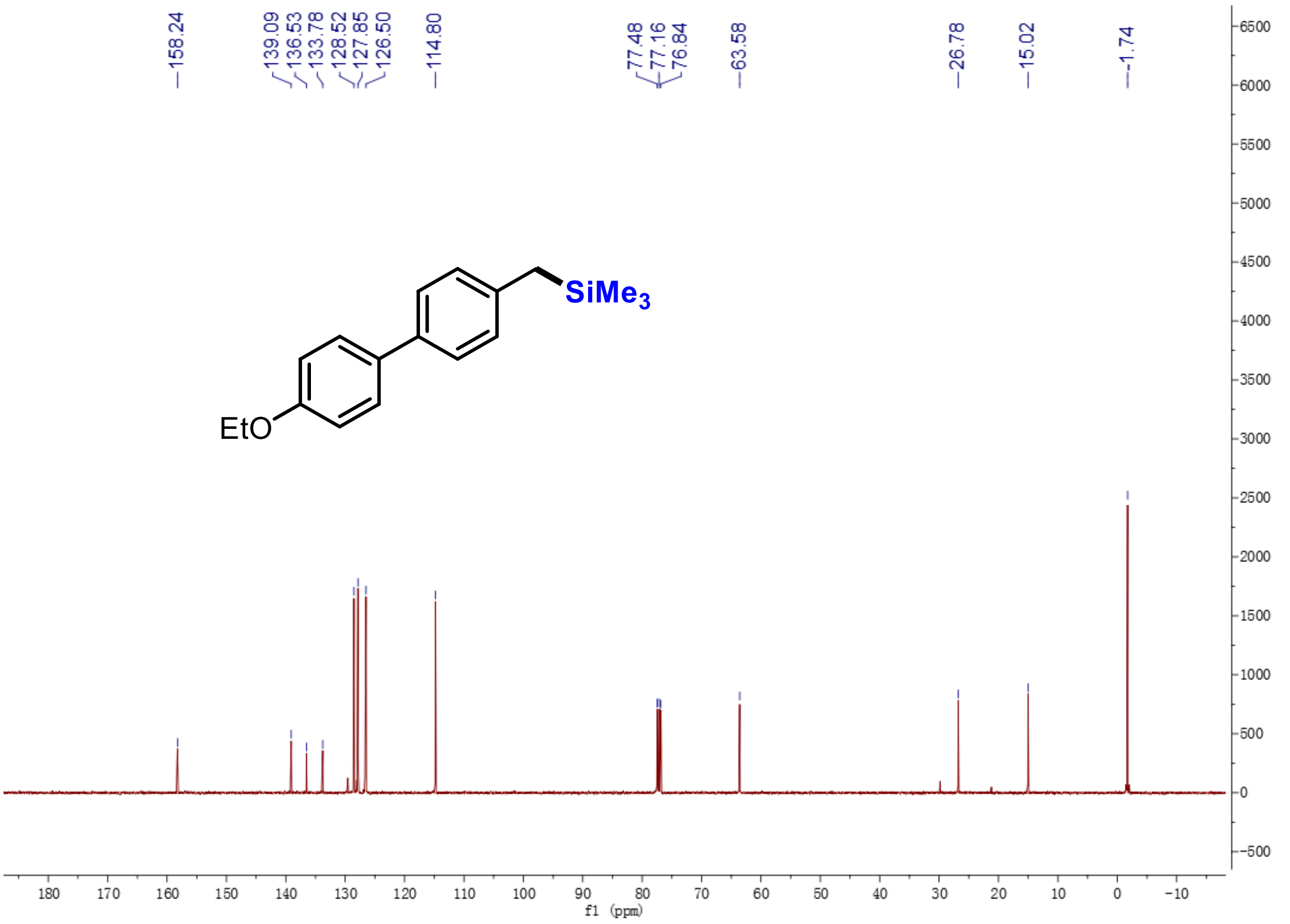

^{1}H NMR spectrum of 3z (400 MHz, $CDCl_3$)

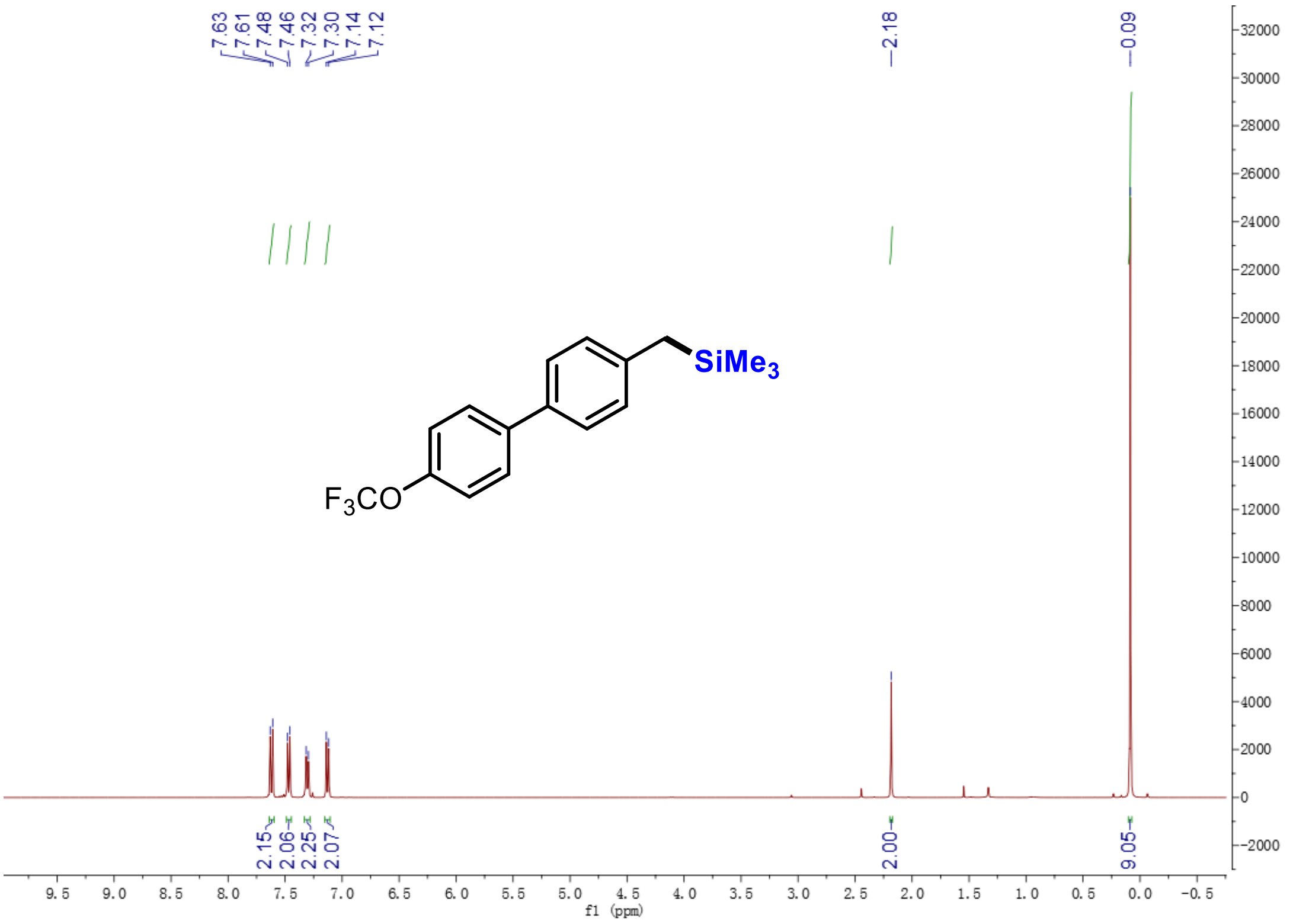

^{13}C NMR spectrum of 3z (101 MHz, $CDCl_3$)

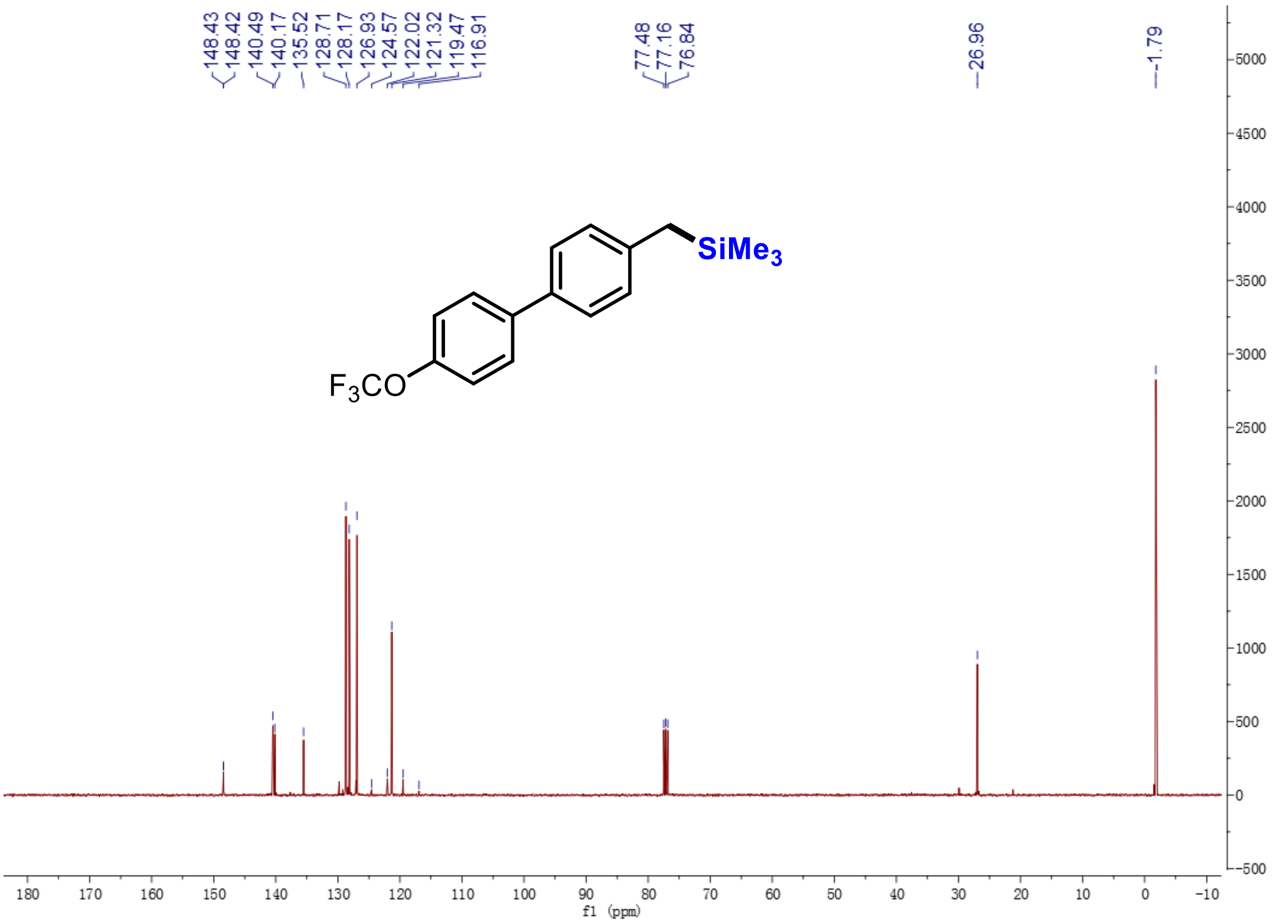

^{19}F NMR spectrum of 3z

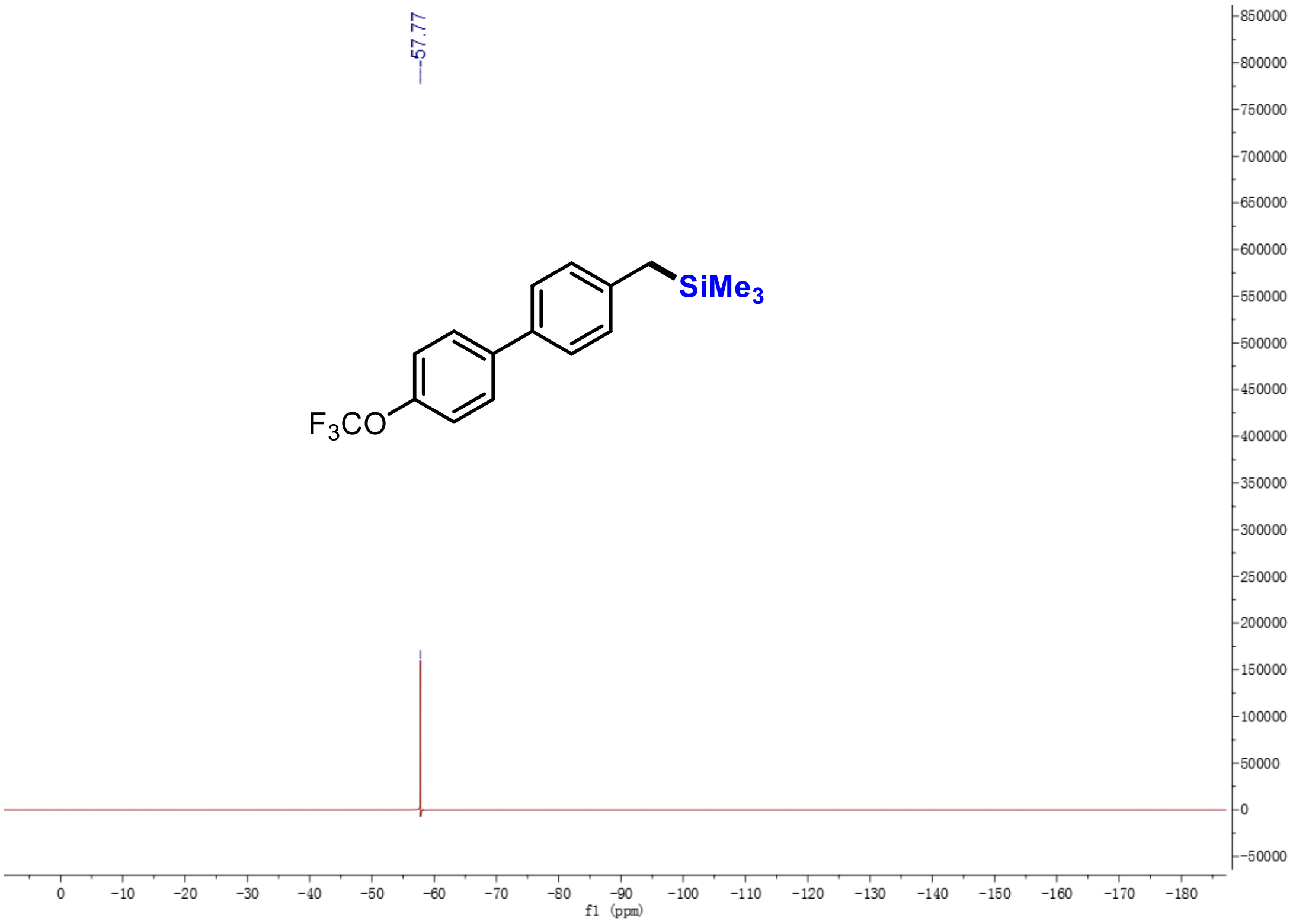

^{1}H NMR spectrum of 3aa (400 MHz, $CDCl_3$)

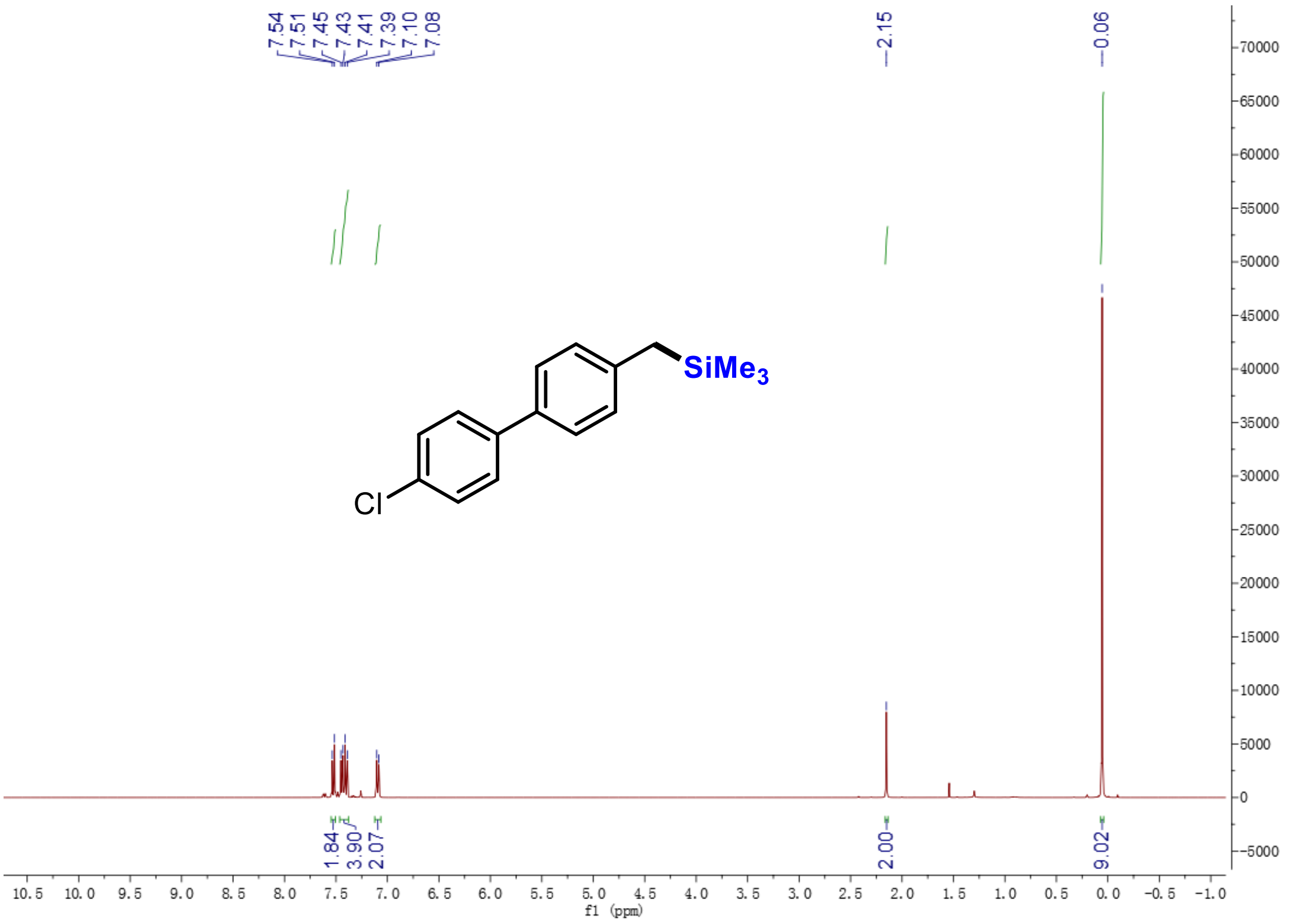

^{13}C NMR spectrum of 3aa (101 MHz, $CDCl_3$)

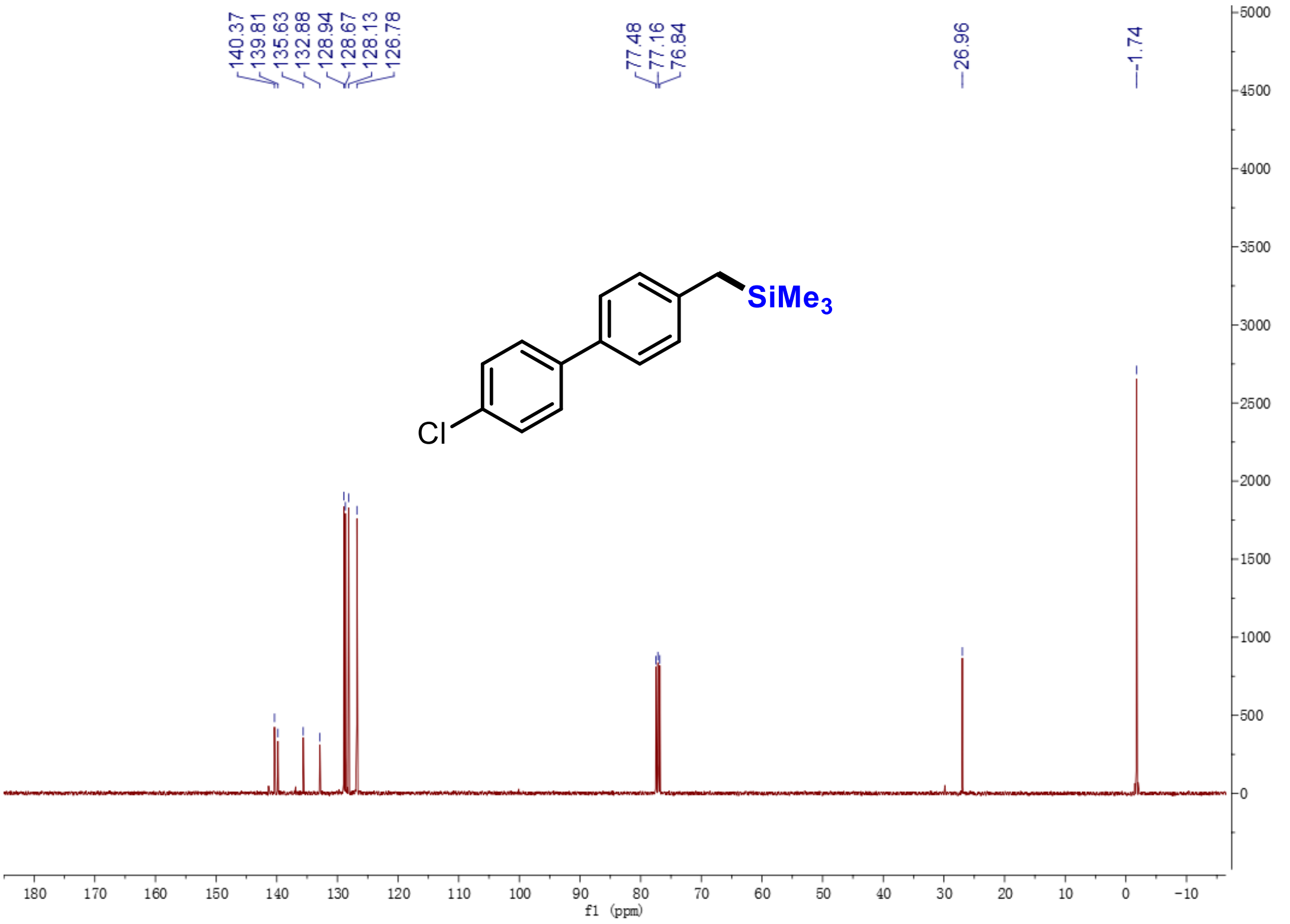

^{1}H NMR spectrum of 3ab (400 MHz, $CDCl_3$)

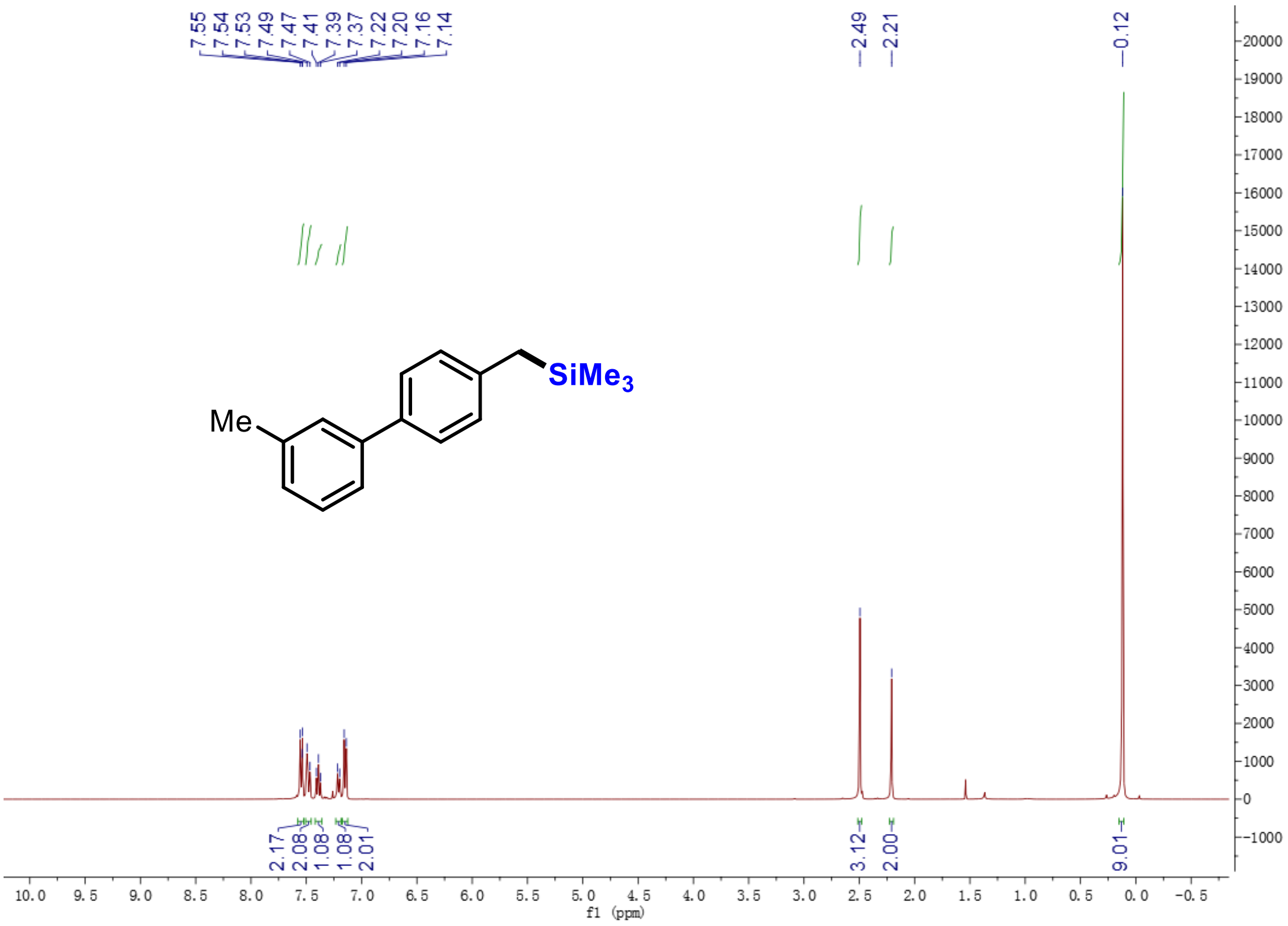

^{13}C NMR spectrum of 3ab (101 MHz, $CDCl_3$)

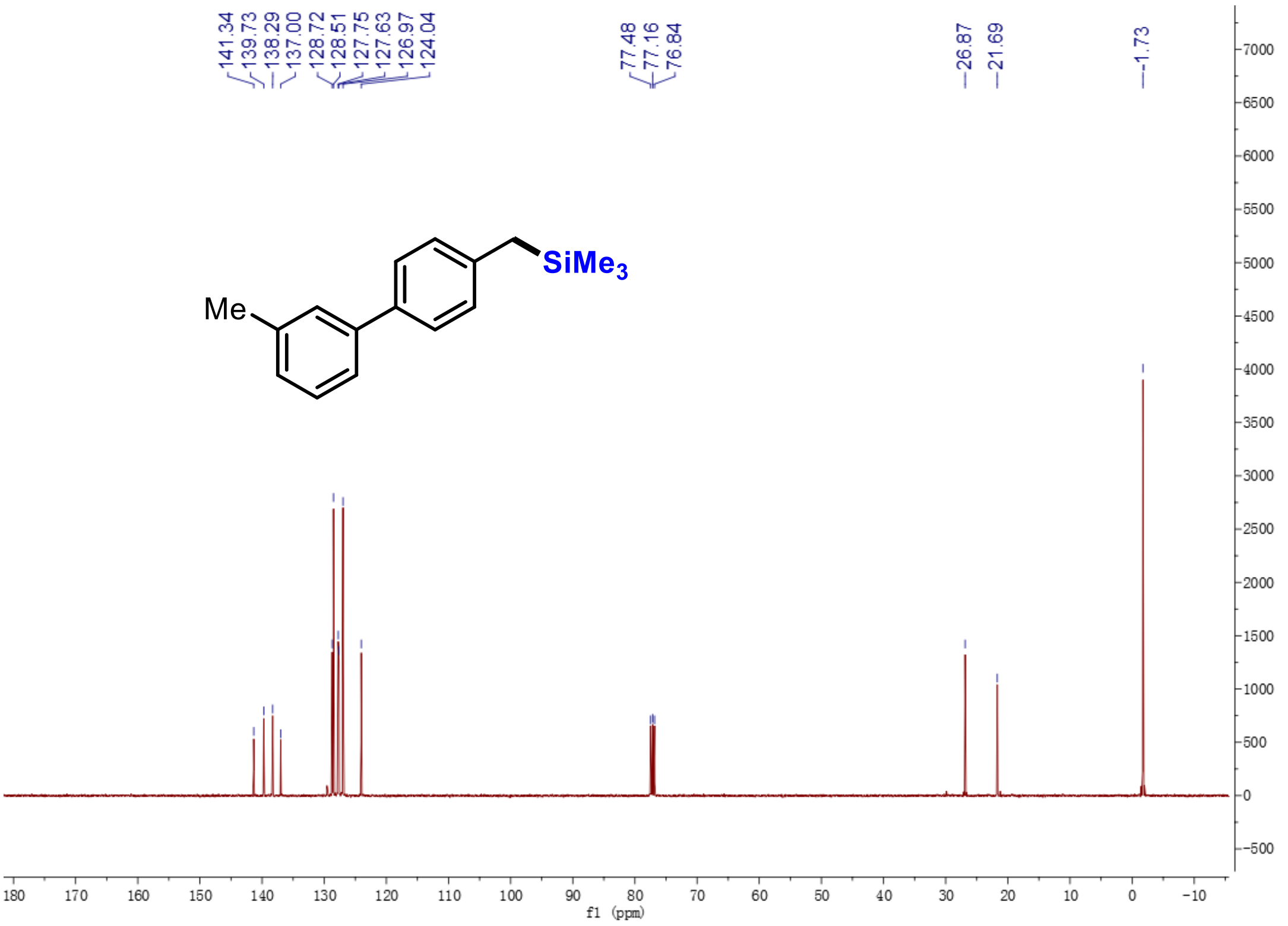

^{1}H NMR spectrum of 3ac (400 MHz, $CDCl_3$)

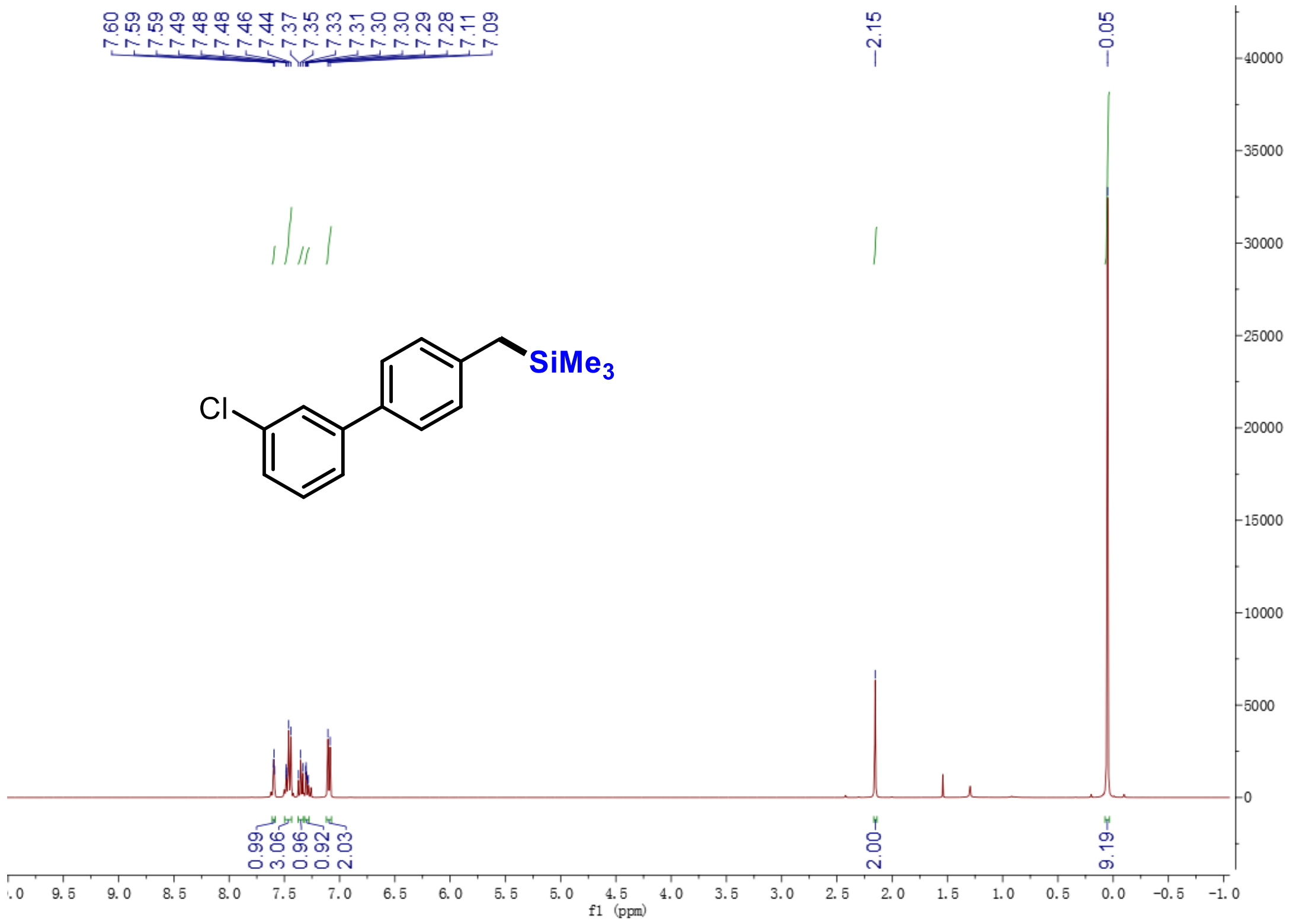

^{13}C NMR spectrum of 3ac (101 MHz, $CDCl_3$)

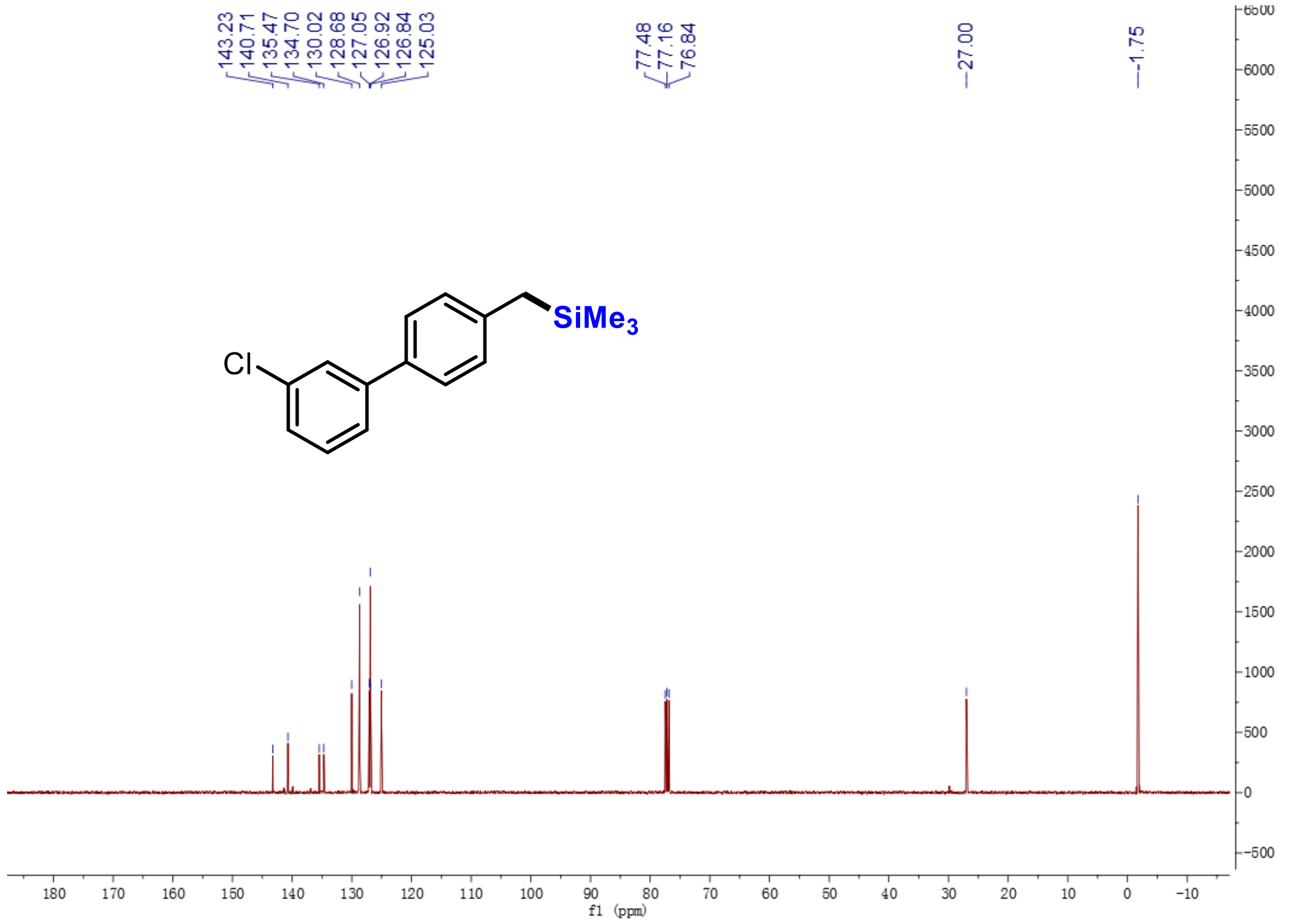

^{1}H NMR spectrum of 3ad (400 MHz, $CDCl_3$)

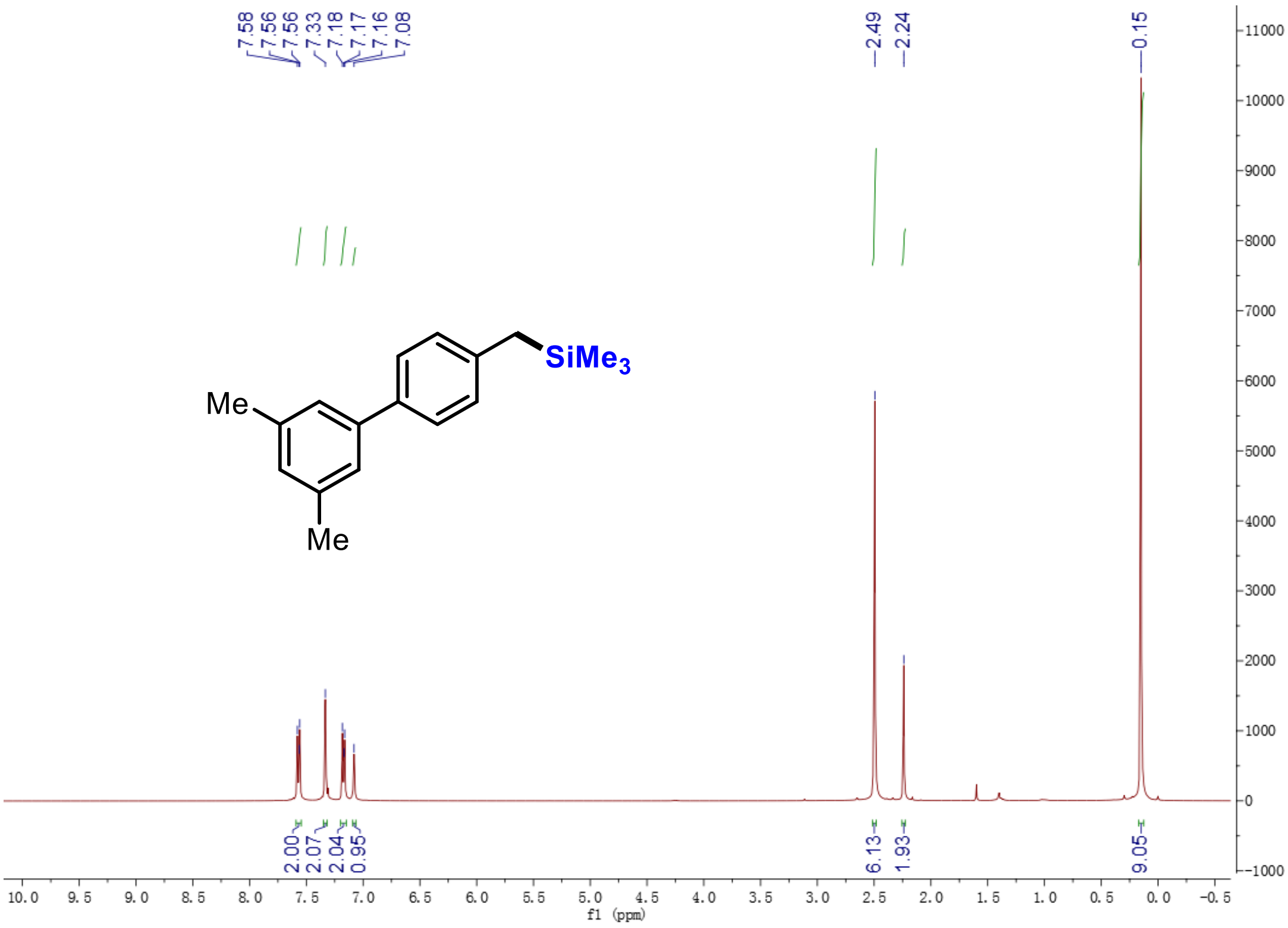

^{13}C NMR spectrum of 3ad (101 MHz, $CDCl_3$)

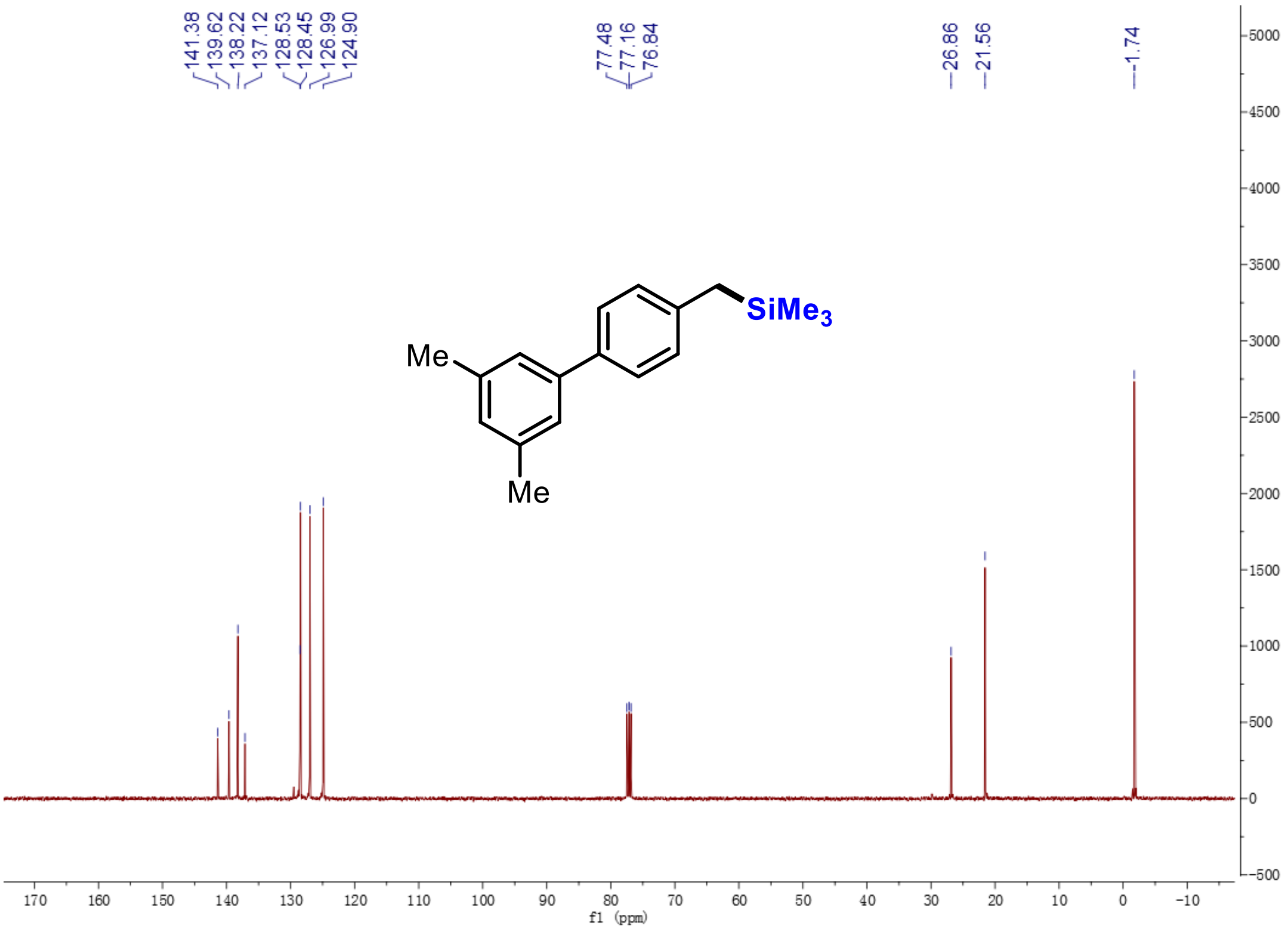

^{1}H NMR spectrum of 3ae (400 MHz, $CDCl_3$)

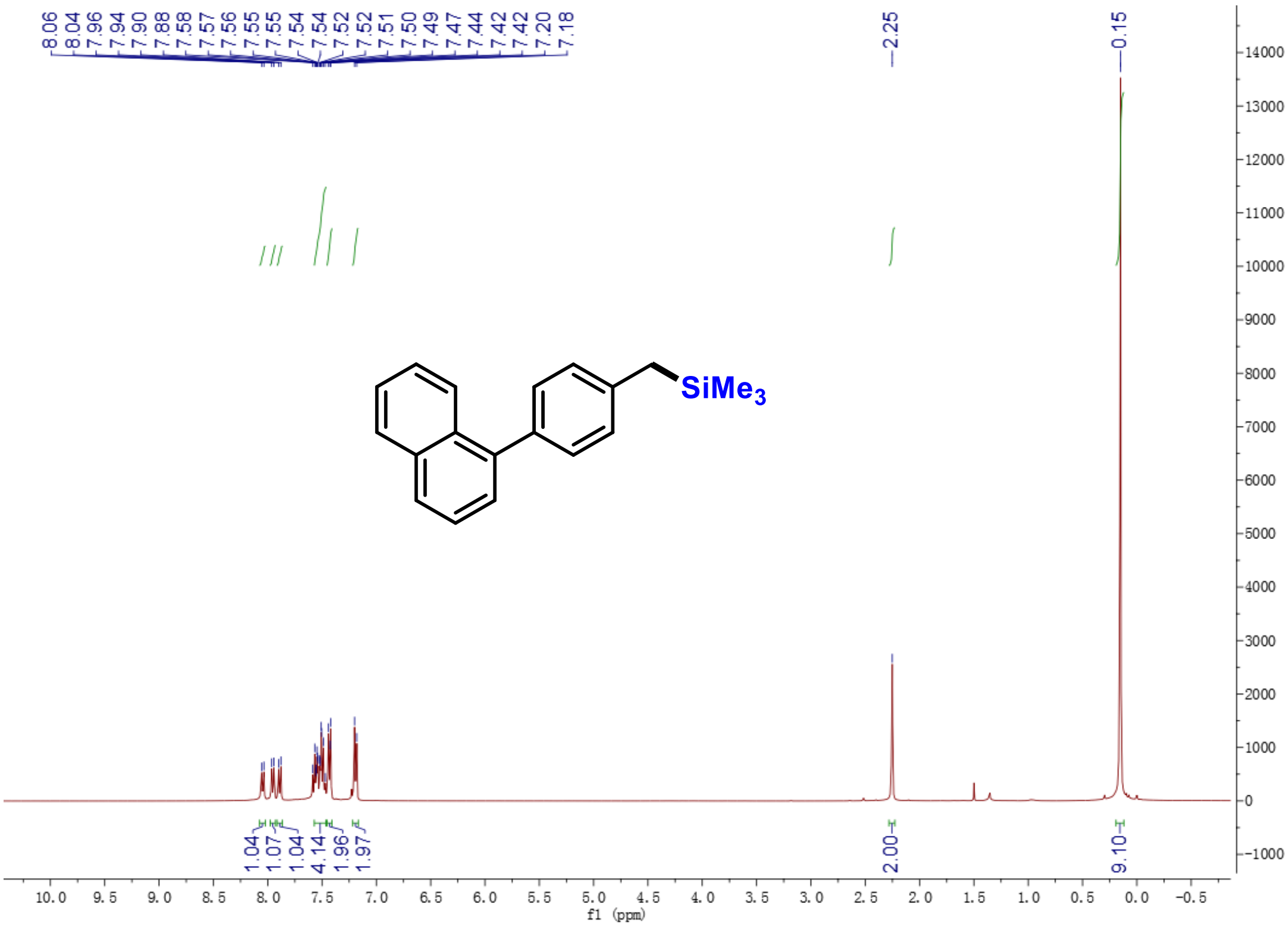

^{13}C NMR spectrum of 3ae (101 MHz, $CDCl_3$)

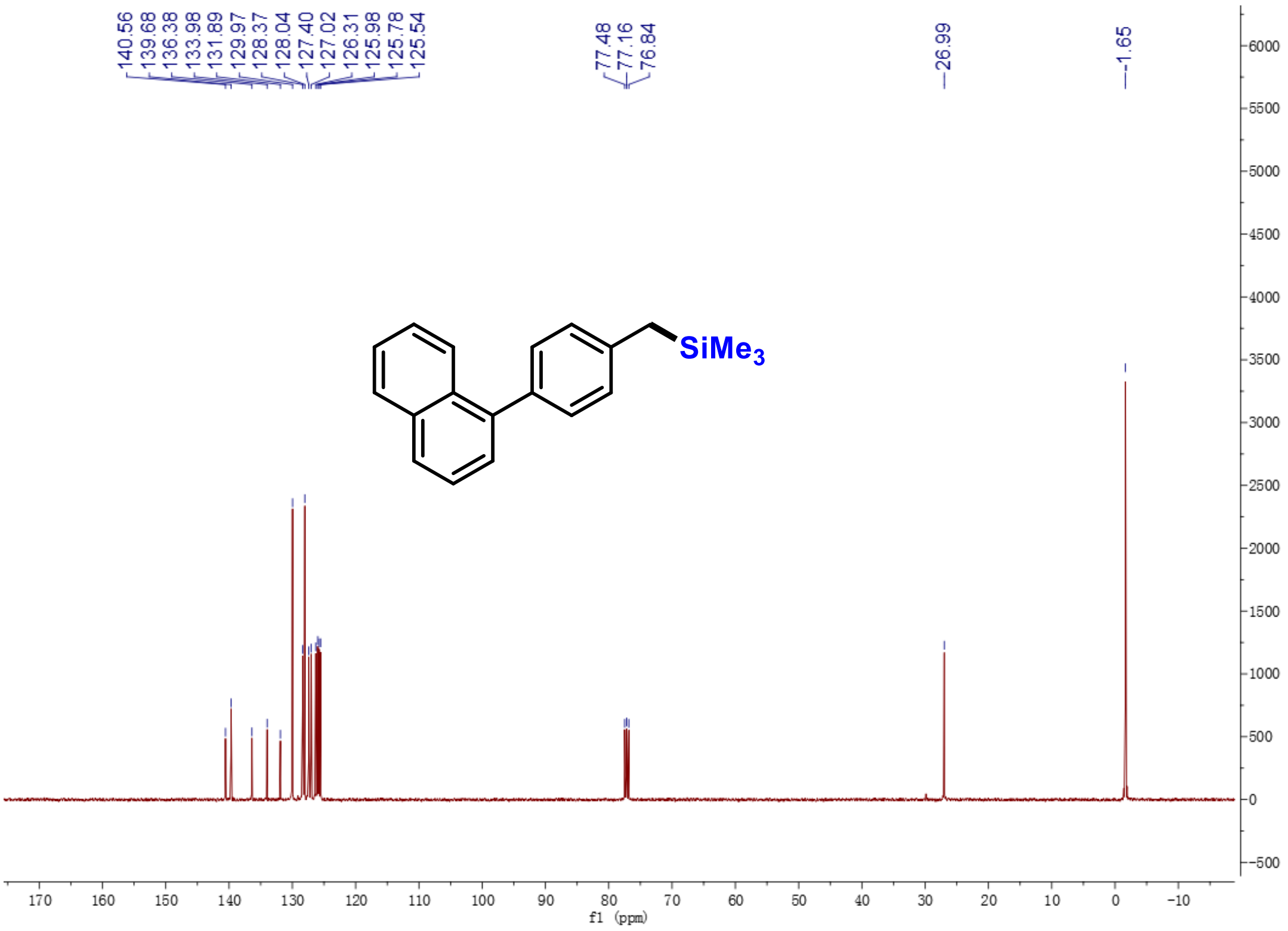

^{1}H NMR spectrum of 3af (400 MHz, $CDCl_3$)

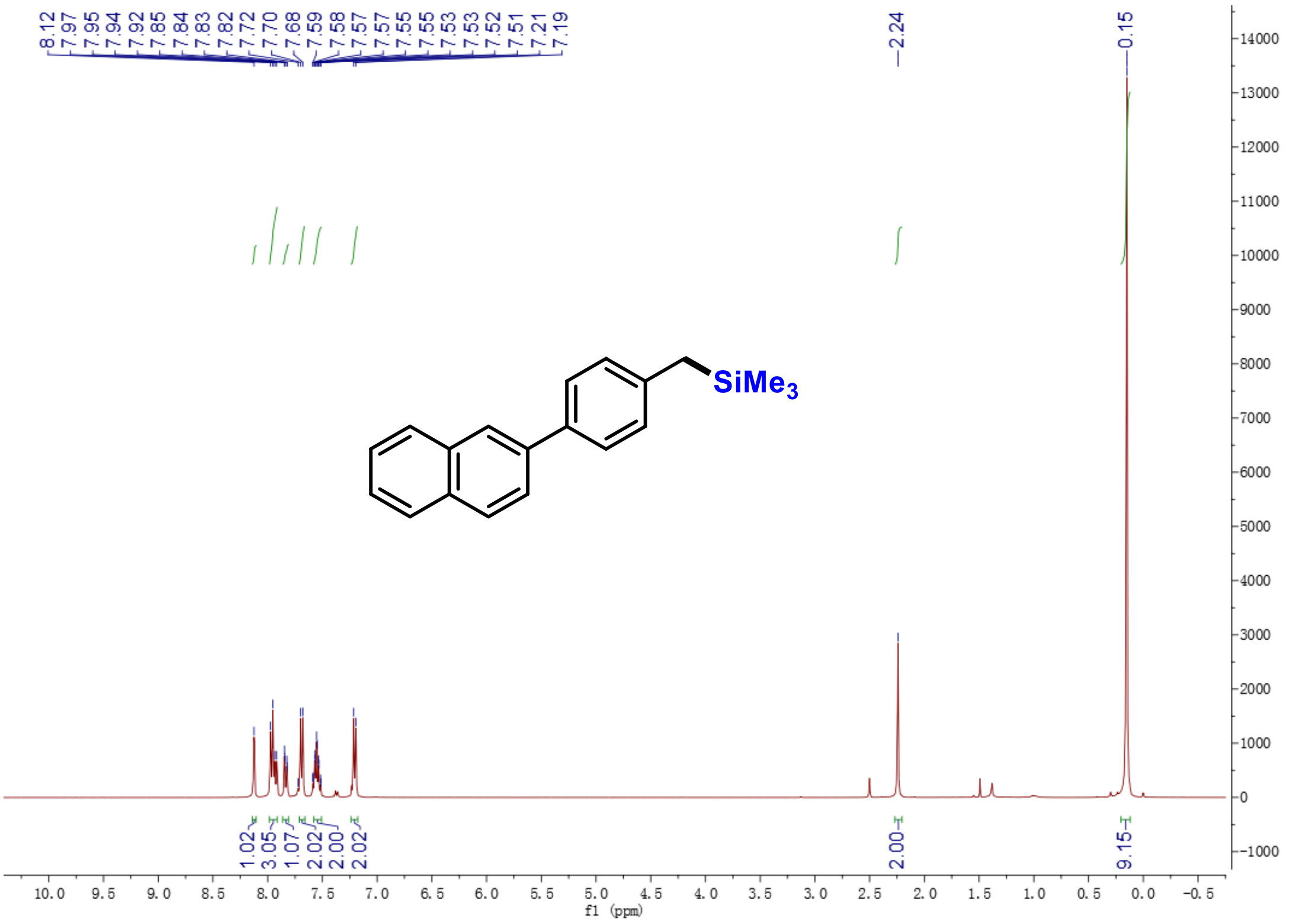

^{13}C NMR spectrum of 3af (101 MHz, $CDCl_3$)

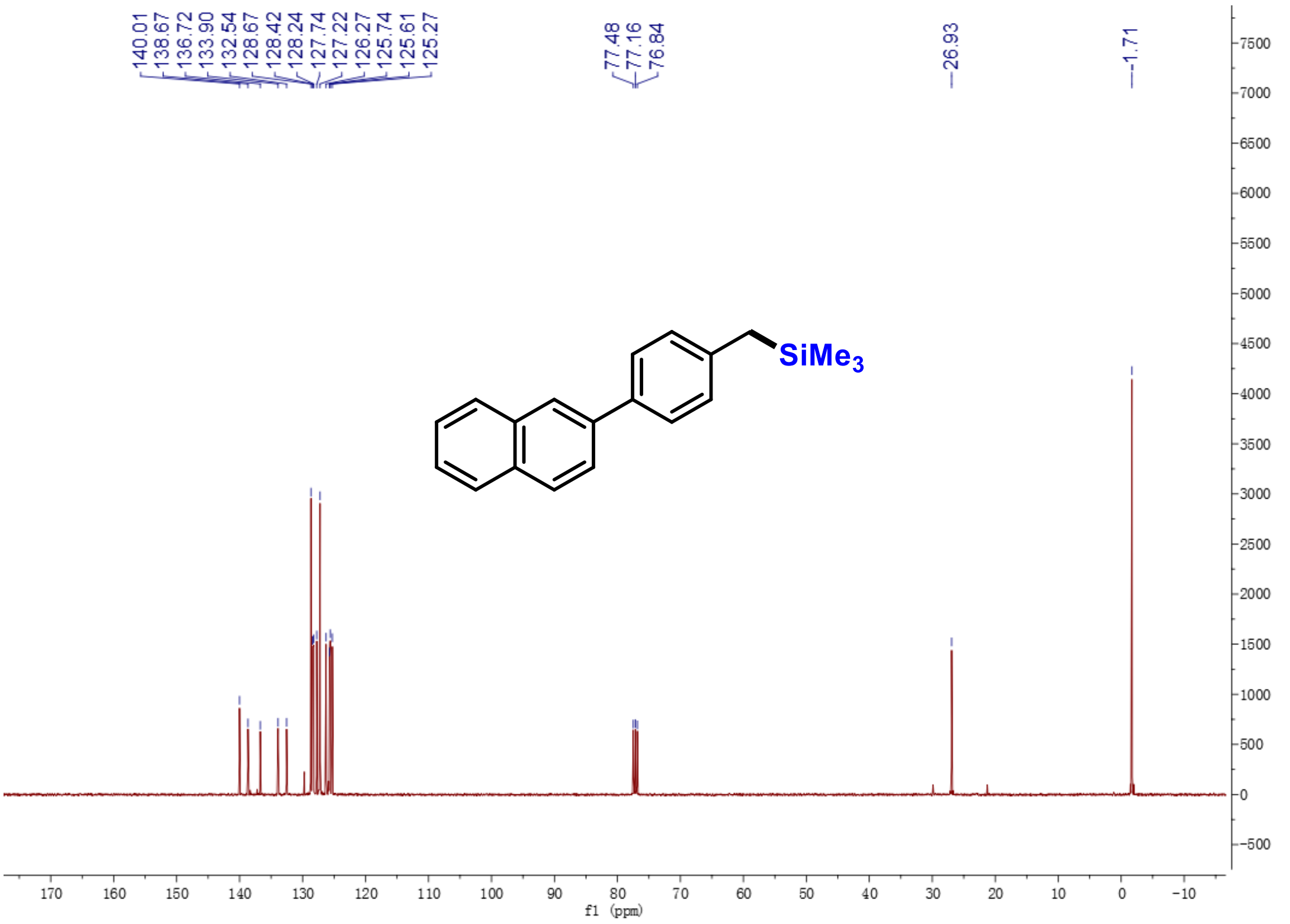

^{1}H NMR spectrum of 3ag (400 MHz, $CDCl_3$)

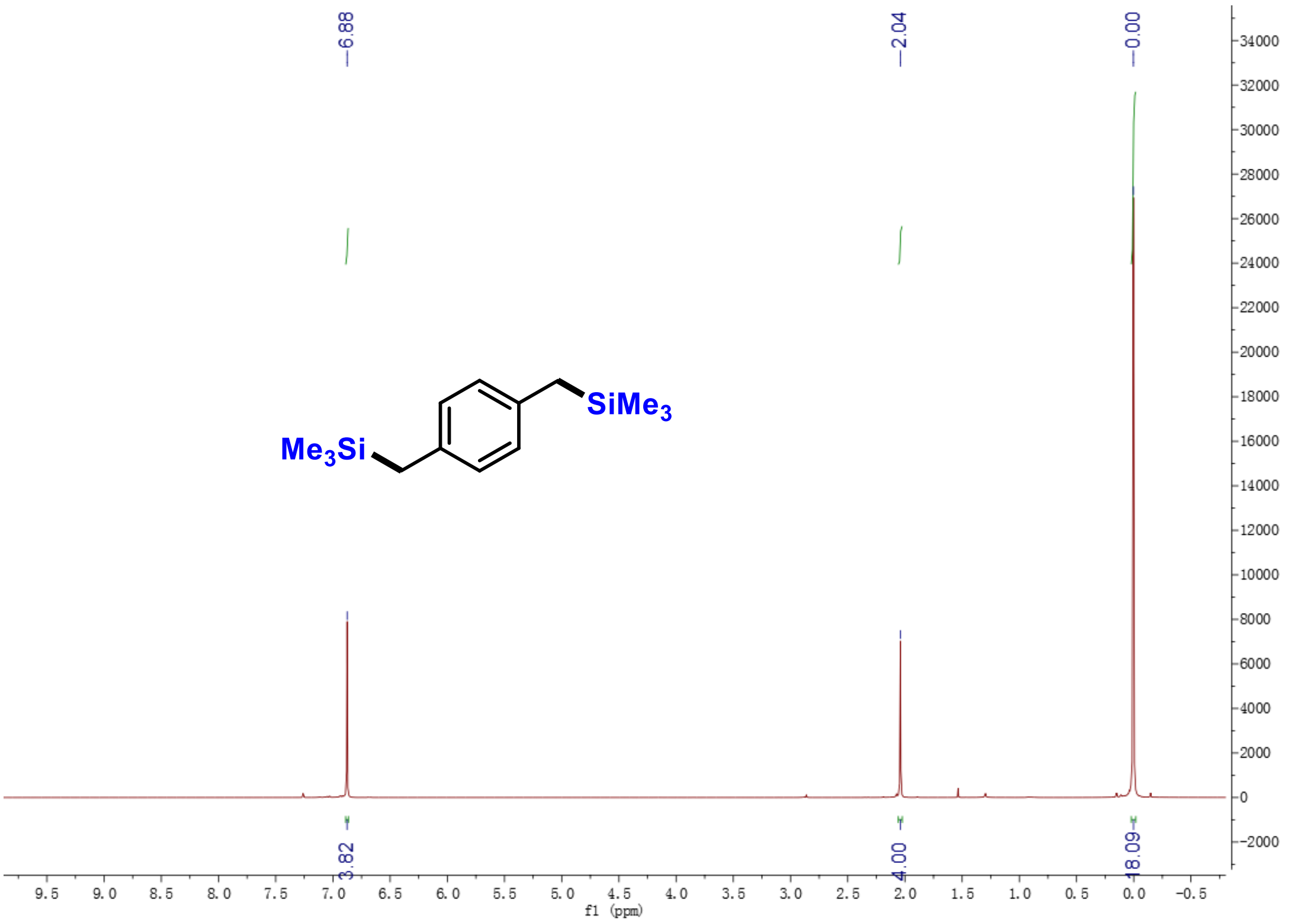

^{13}C NMR spectrum of 3ag (101 MHz, $CDCl_3$)

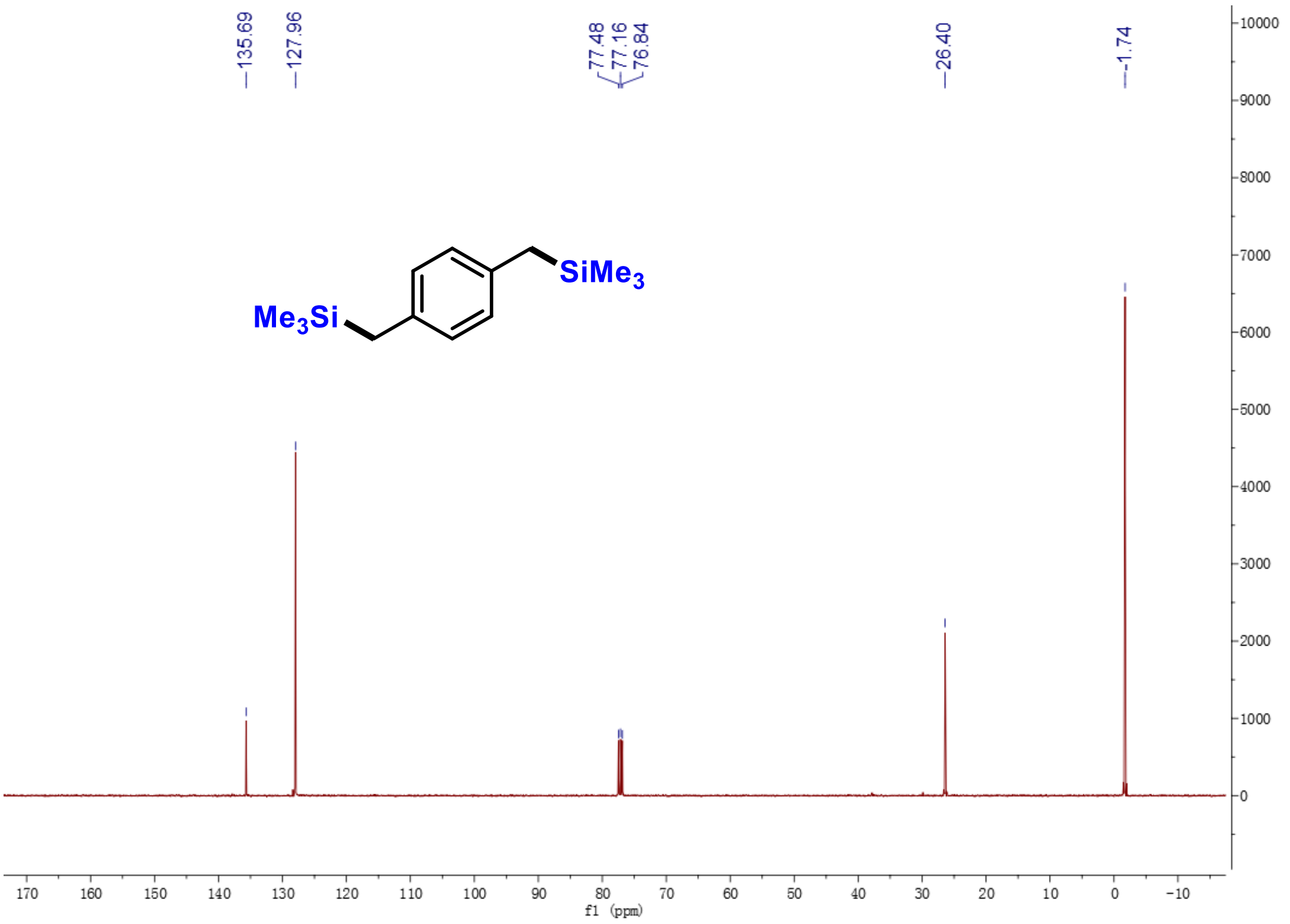

^{1}H NMR spectrum of 3ah (400 MHz, $CDCl_3$)

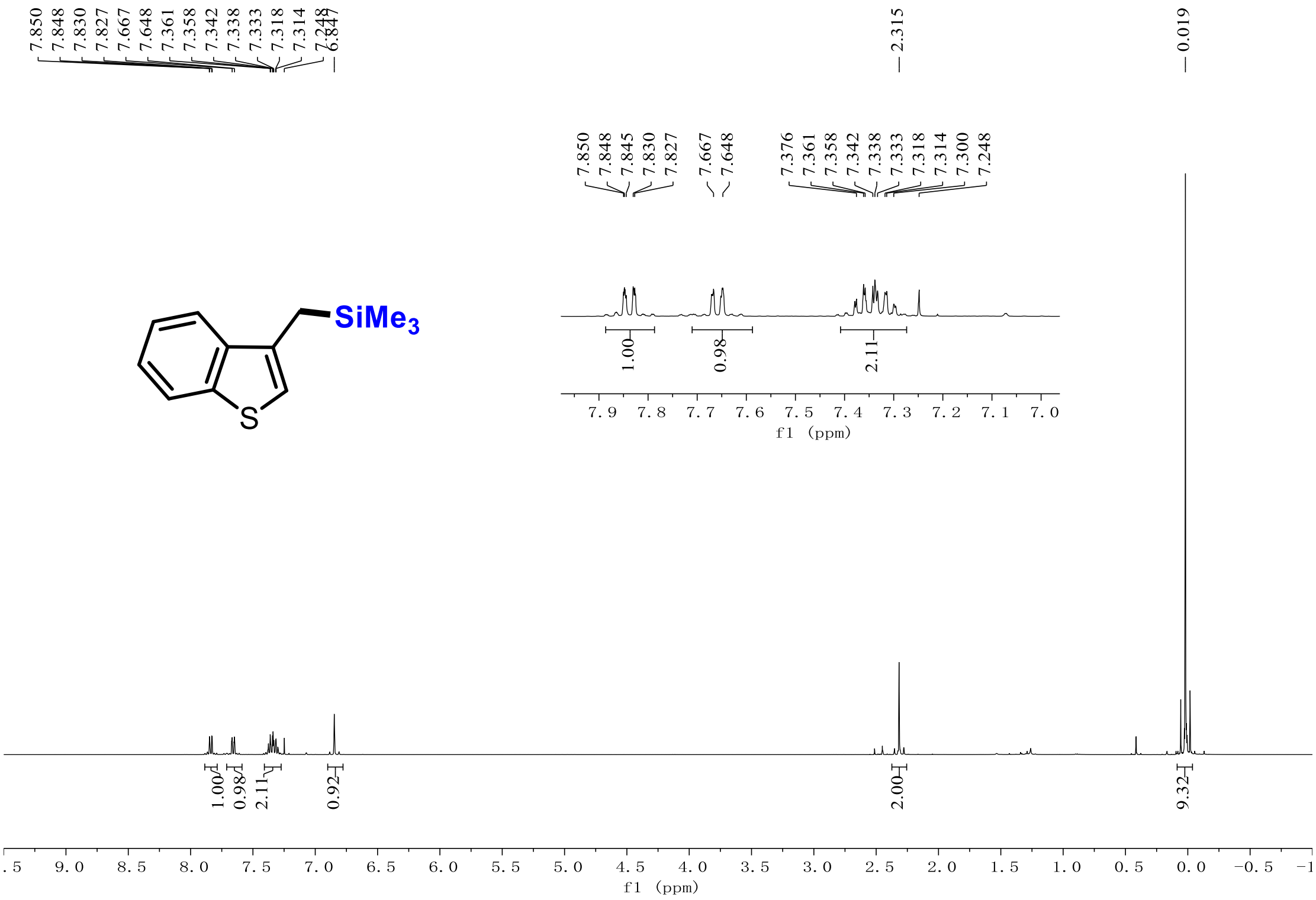

^{13}C NMR spectrum of 3ah (101 MHz, $CDCl_3$)

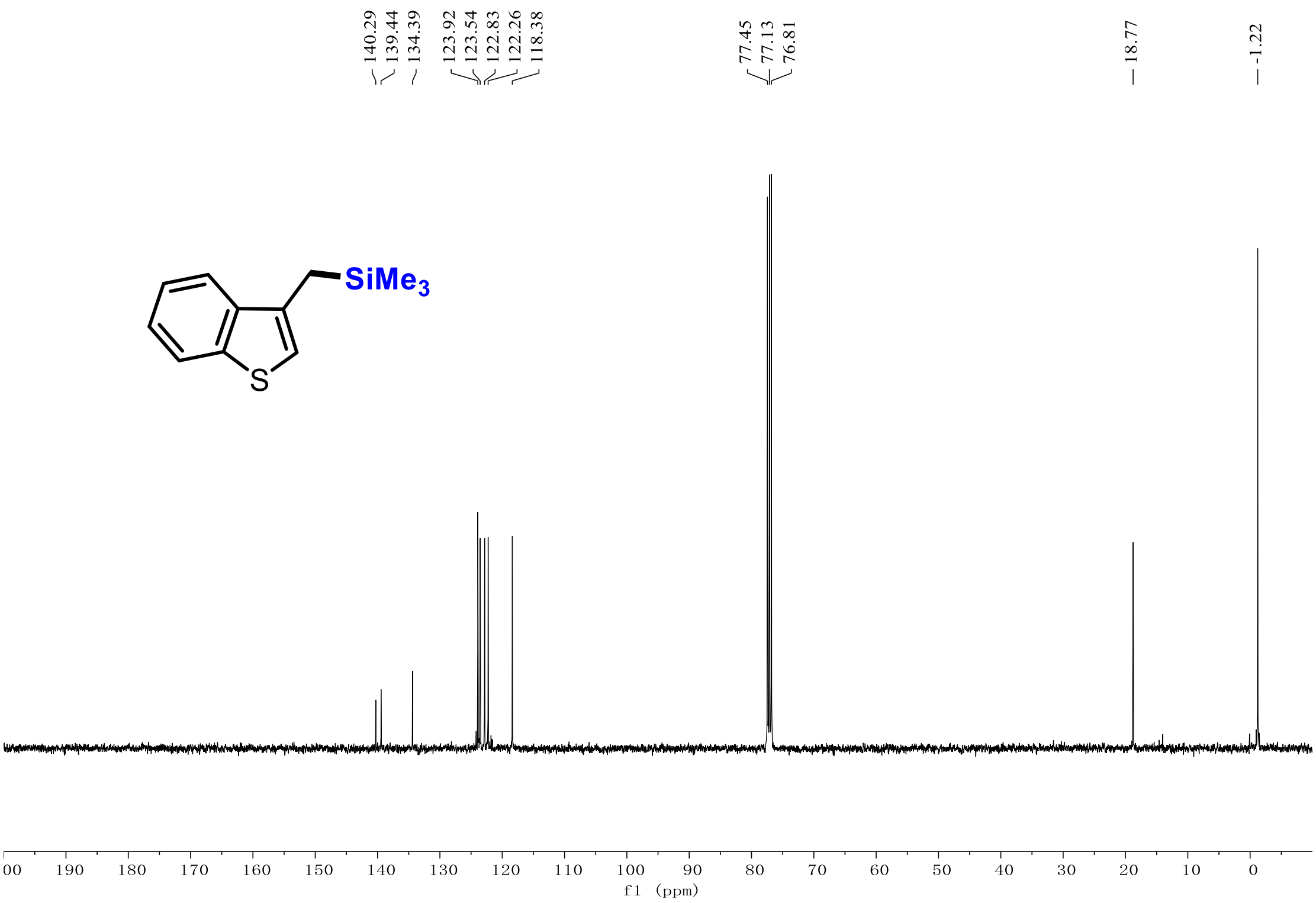

^{1}H NMR spectrum of 3ai (400 MHz, $CDCl_3$)

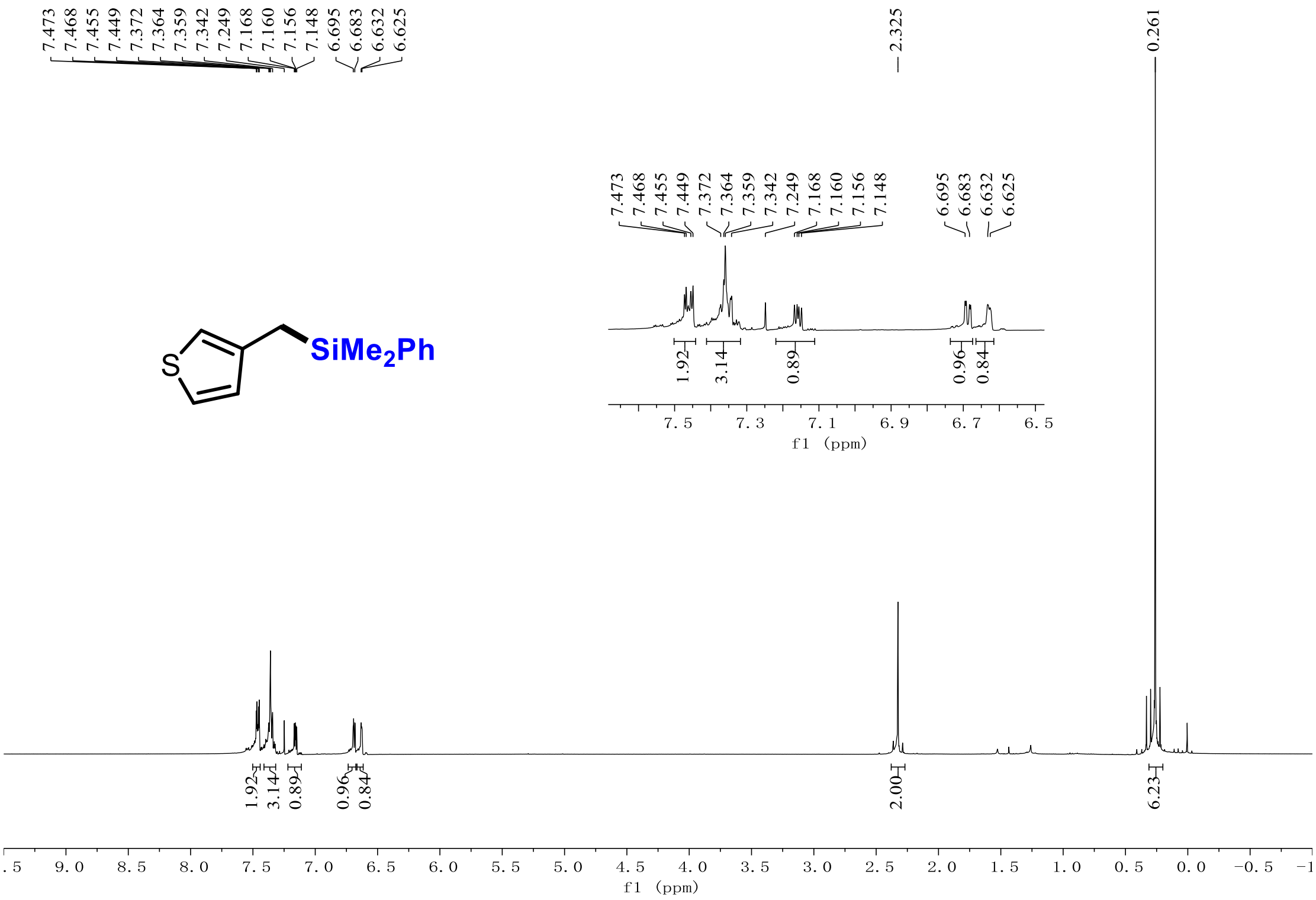

^{13}C NMR spectrum of 3ai (101 MHz, $CDCl_3$)

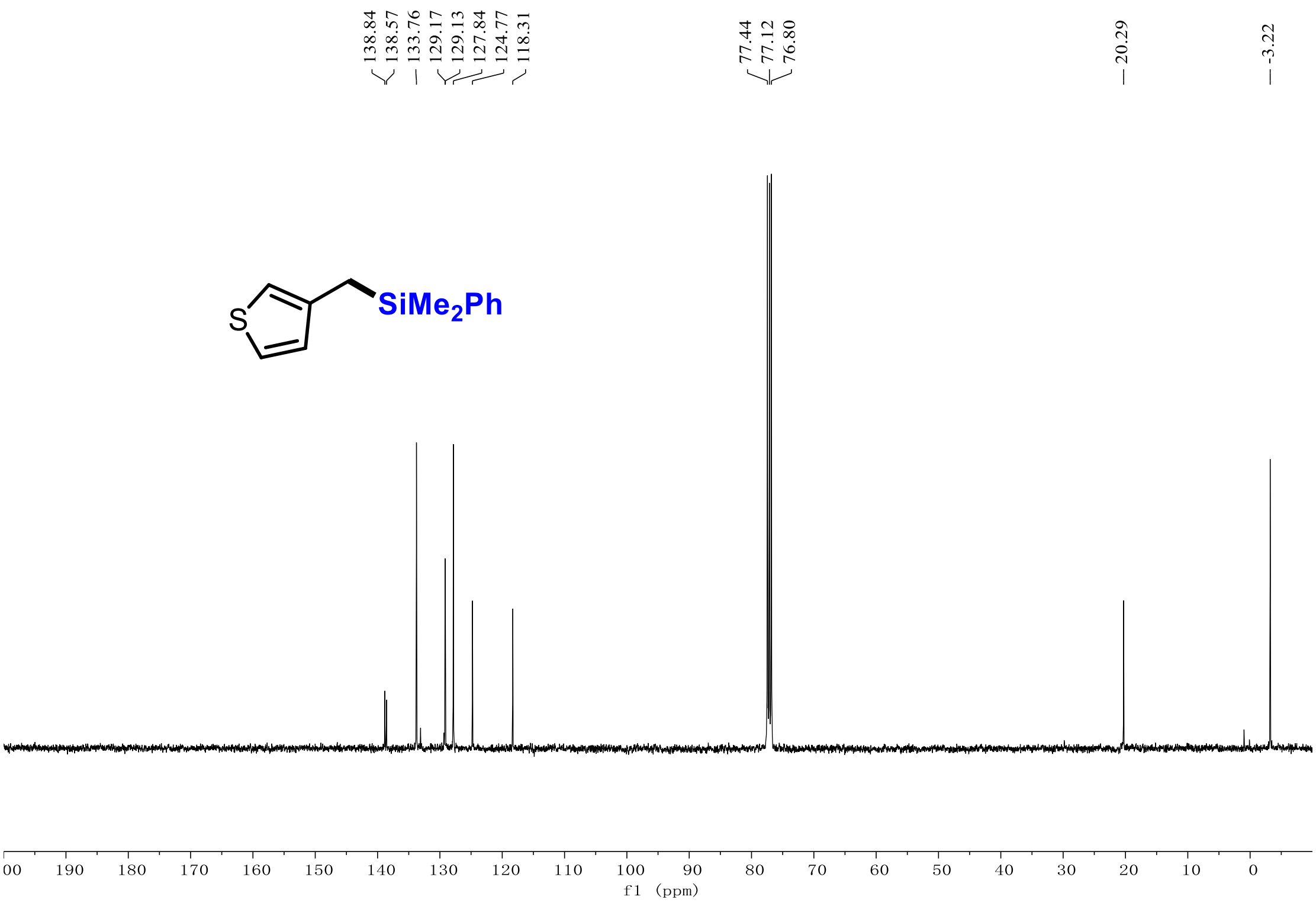

^{1}H NMR spectrum of 3aj (400 MHz, $CDCl_3$)

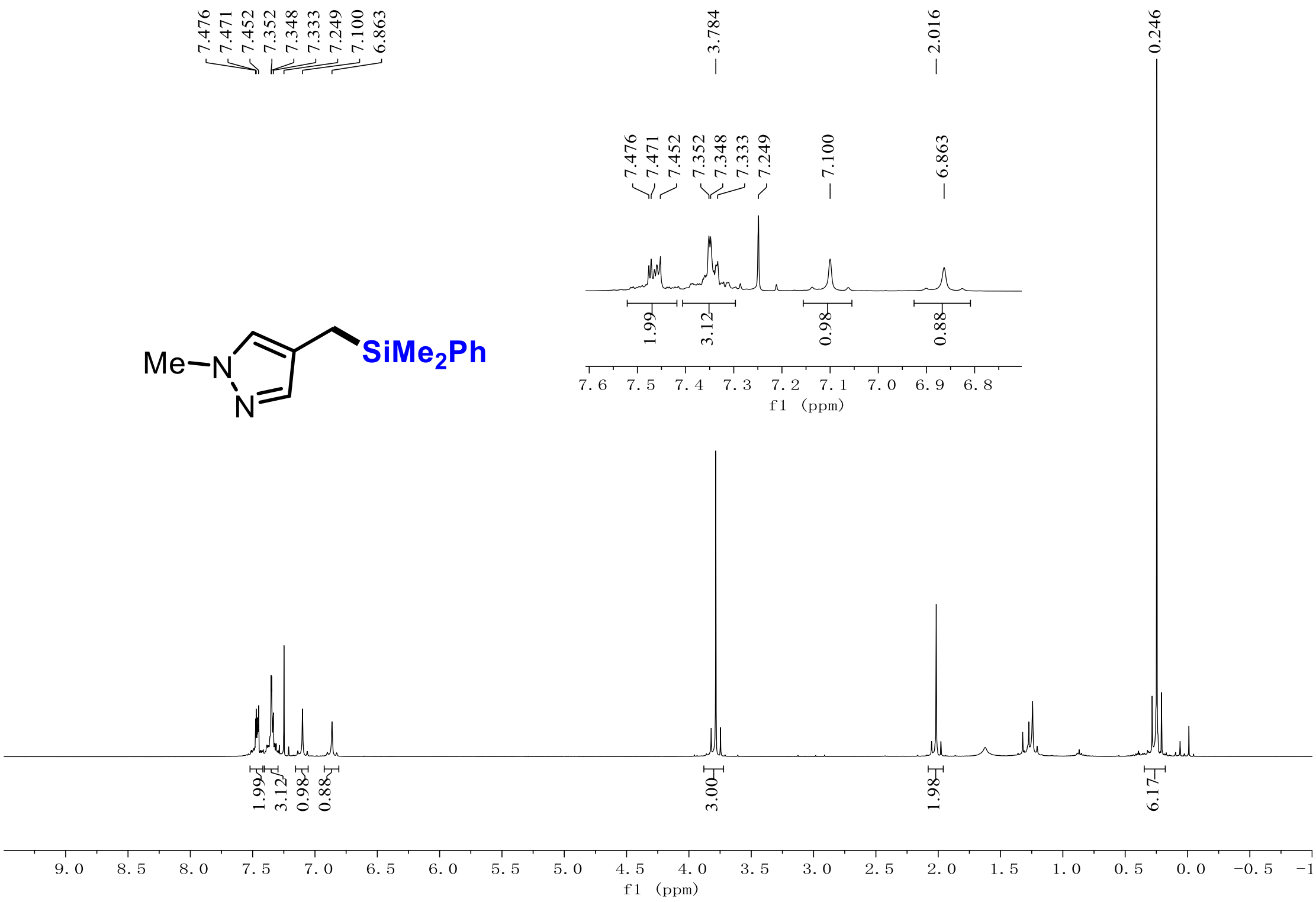

^{13}C NMR spectrum of 3aj (101 MHz, $CDCl_3$)

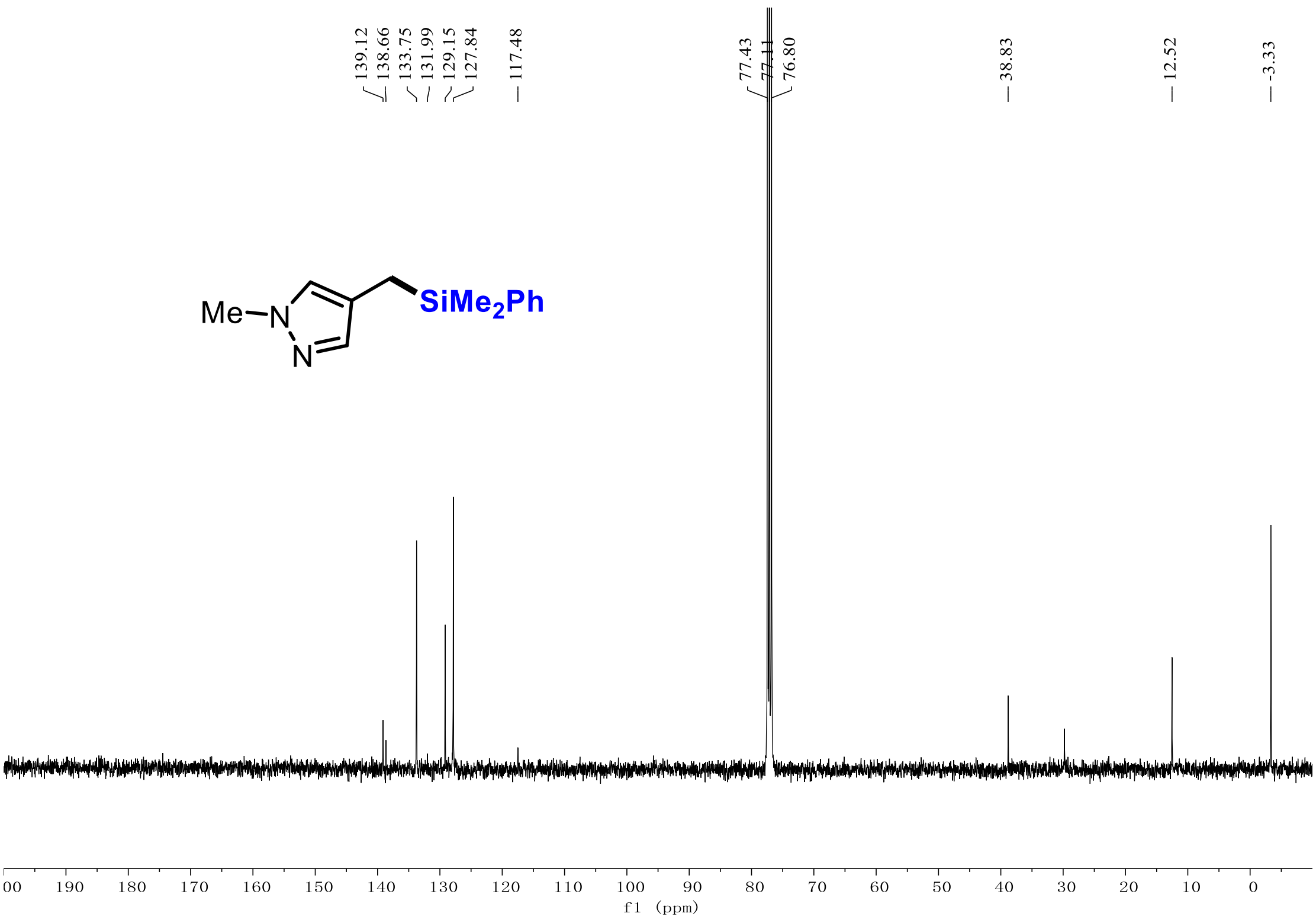

^{1}H NMR spectrum of 3ak (400 MHz, $CDCl_3$)

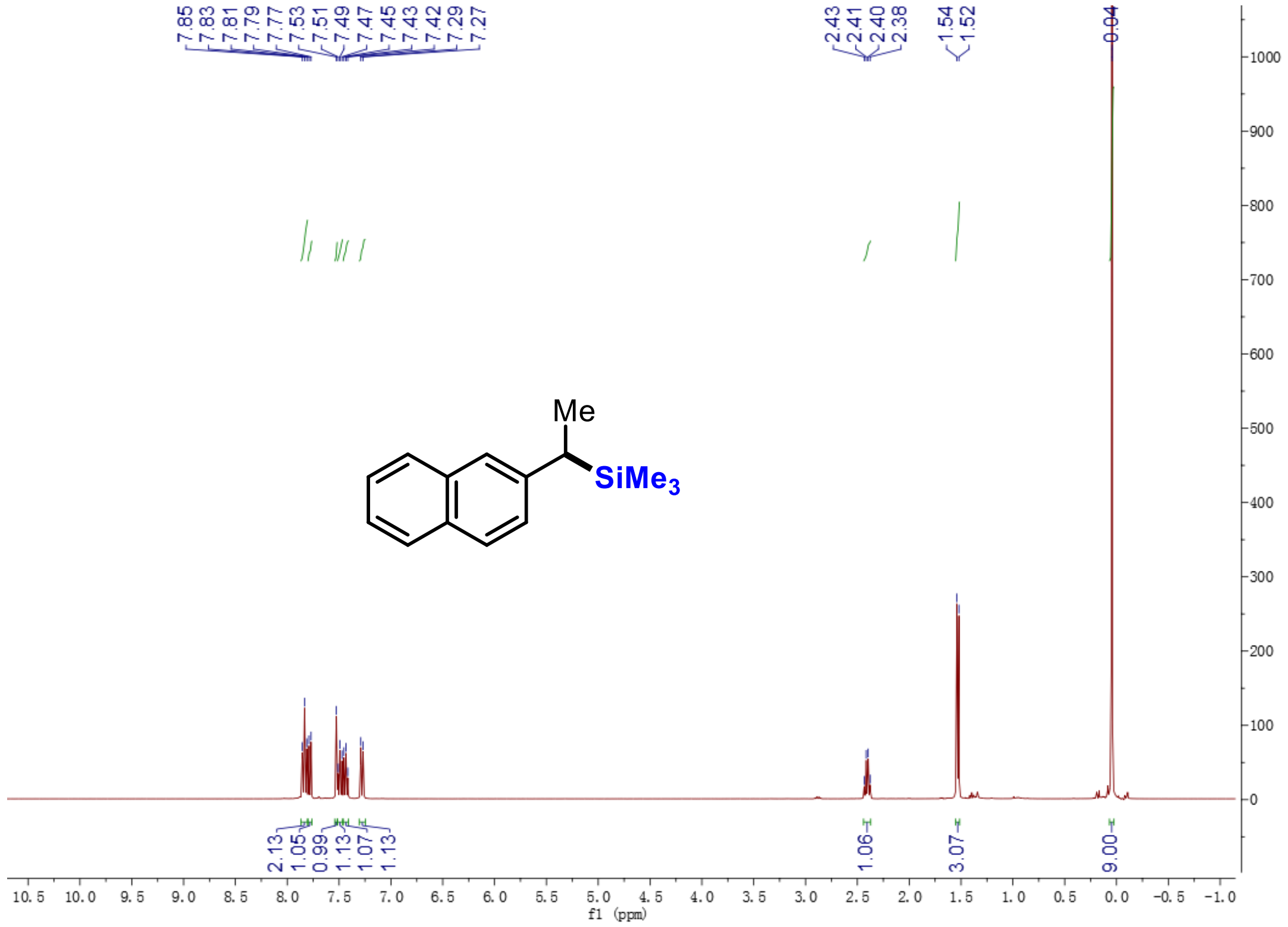

^{13}C NMR spectrum of 3ak (101 MHz, $CDCl_3$)

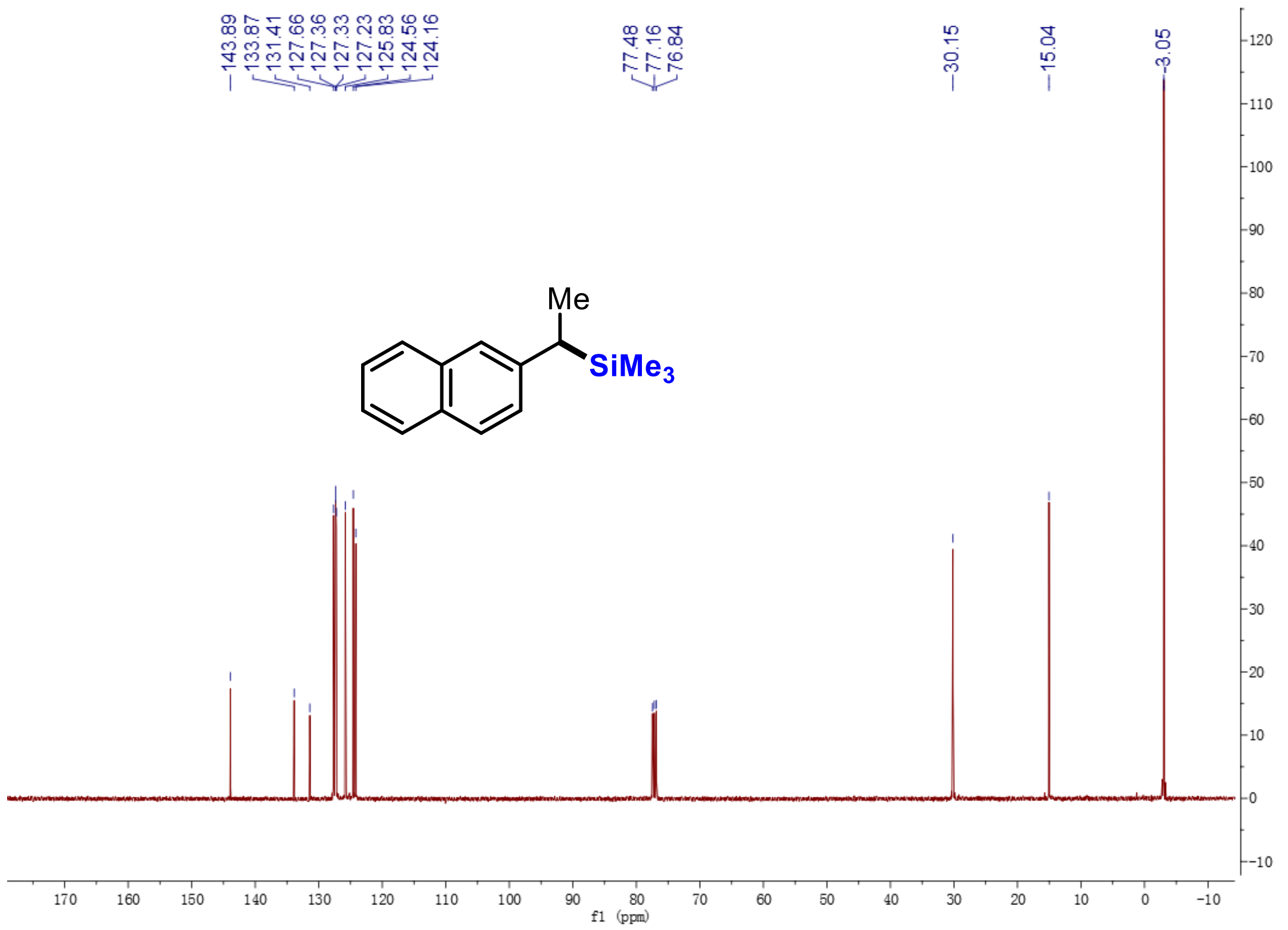

^{1}H NMR spectrum of 3al (400 MHz, $CDCl_3$)

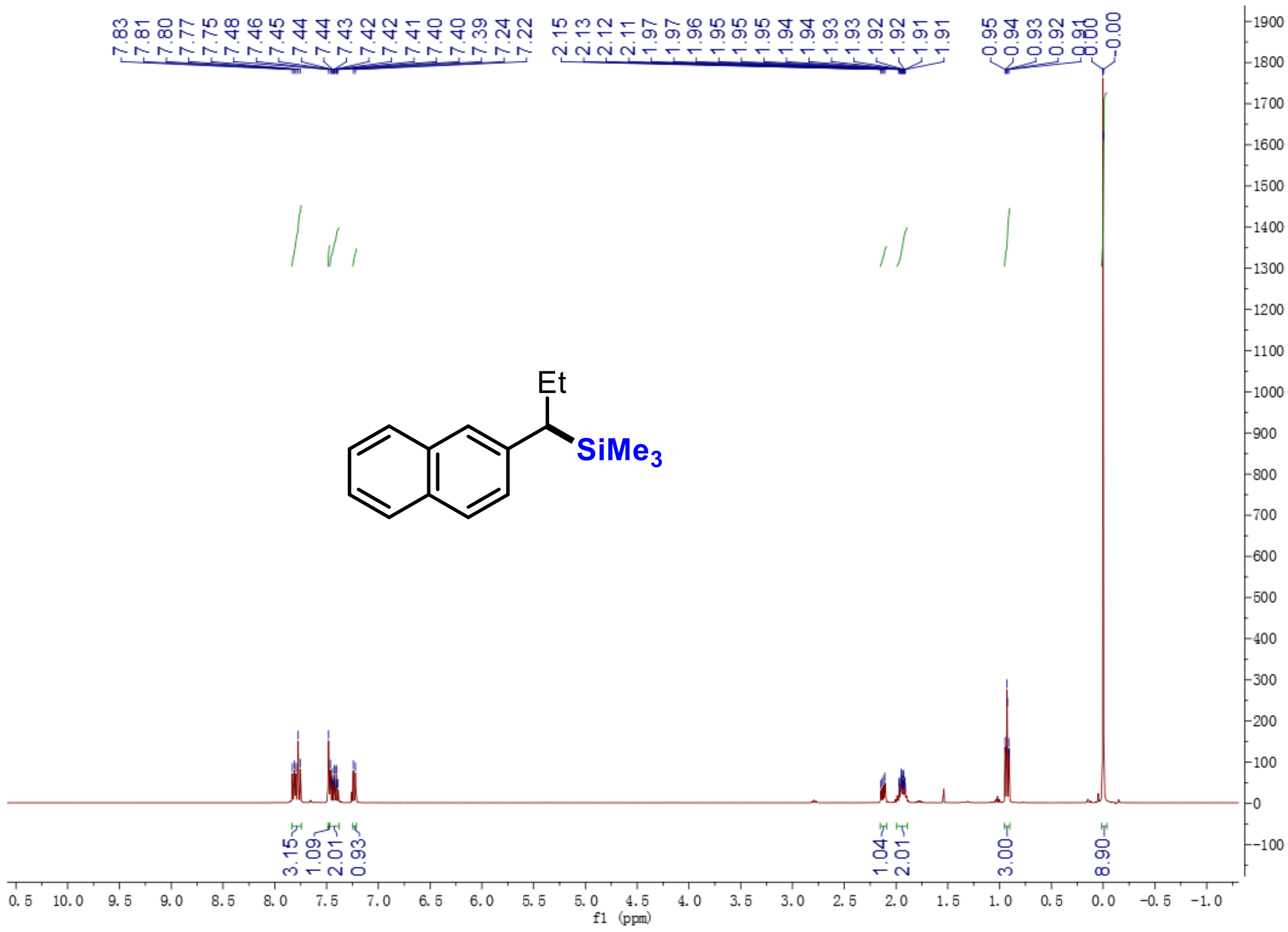

^{13}C NMR spectrum of 3al (101 MHz, $CDCl_3$)

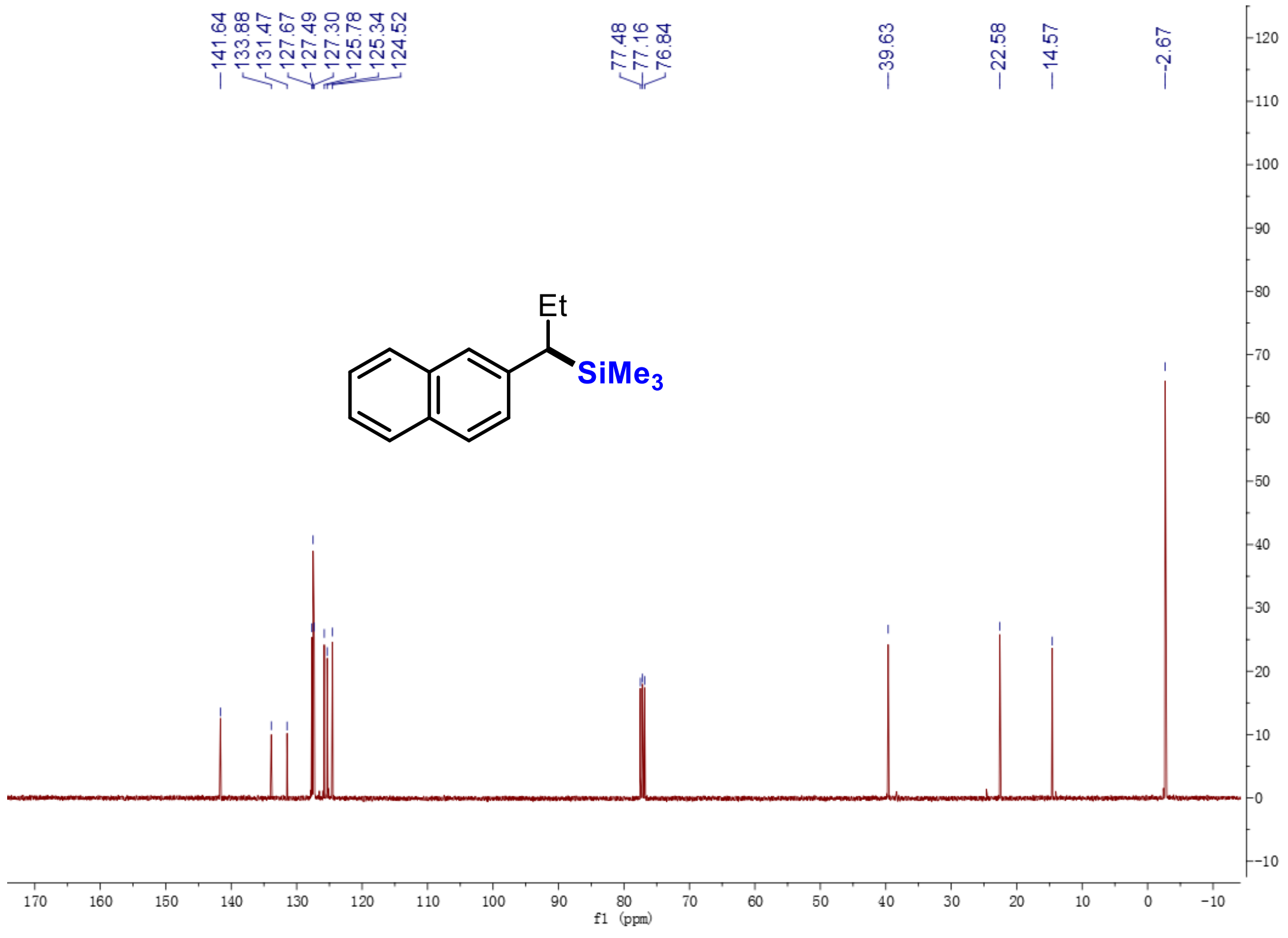

^{1}H NMR spectrum of 3am (400 MHz, $CDCl_3$)

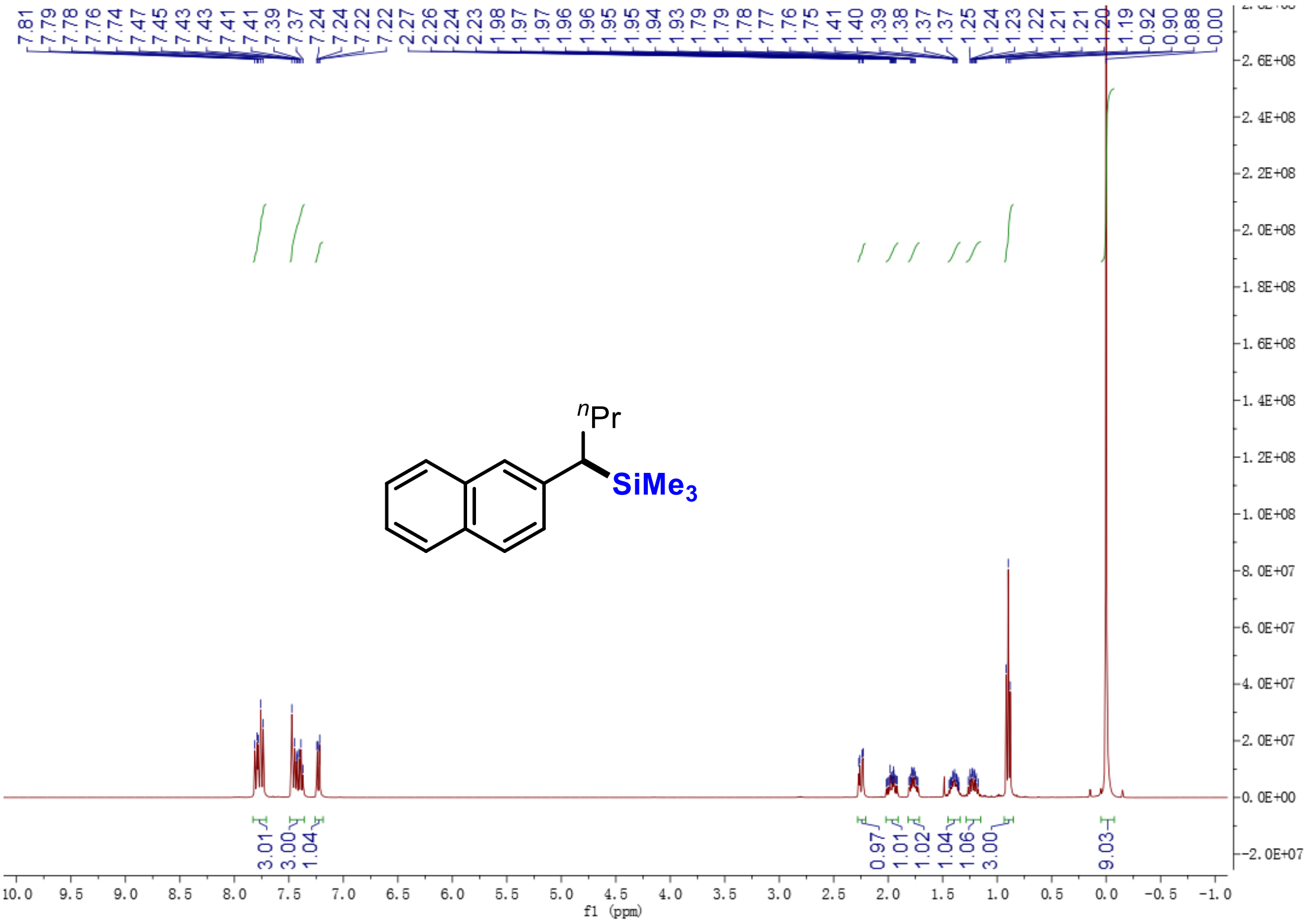

^{13}C NMR spectrum of 3am (101 MHz, $CDCl_3$)

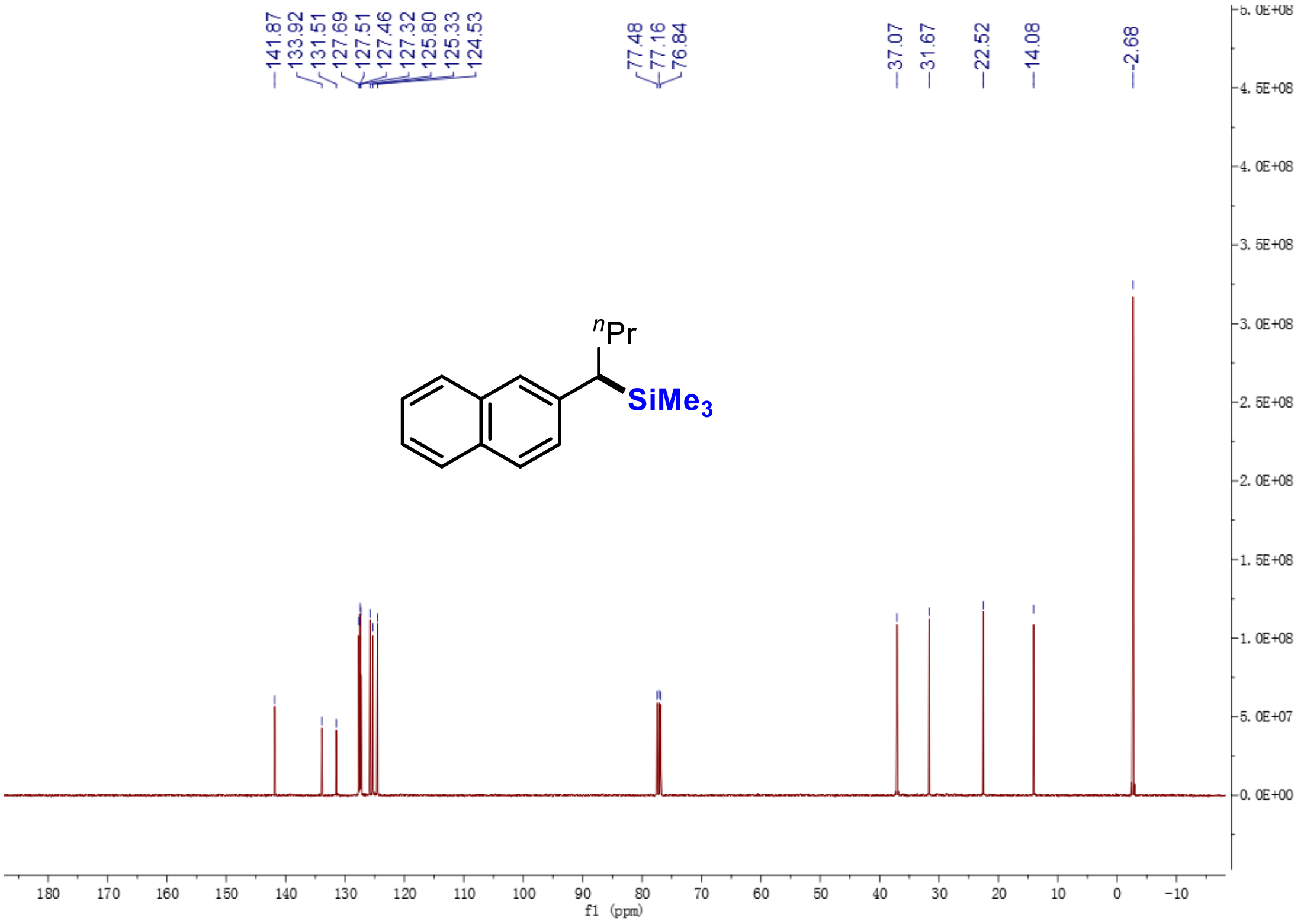

^{1}H NMR spectrum of 3an (400 MHz, $CDCl_3$)

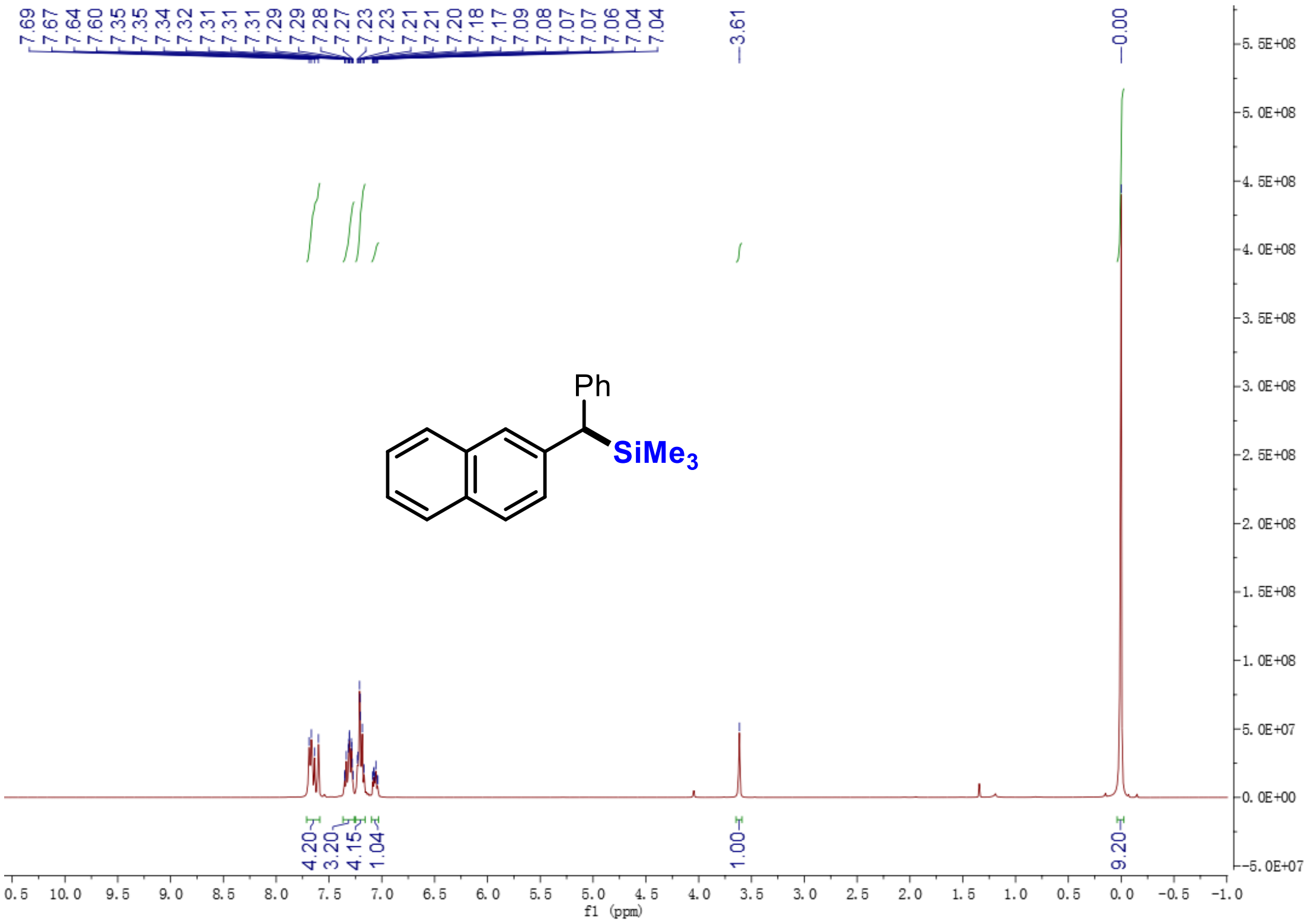

^{13}C NMR spectrum of 3an (101 MHz, $CDCl_3$)

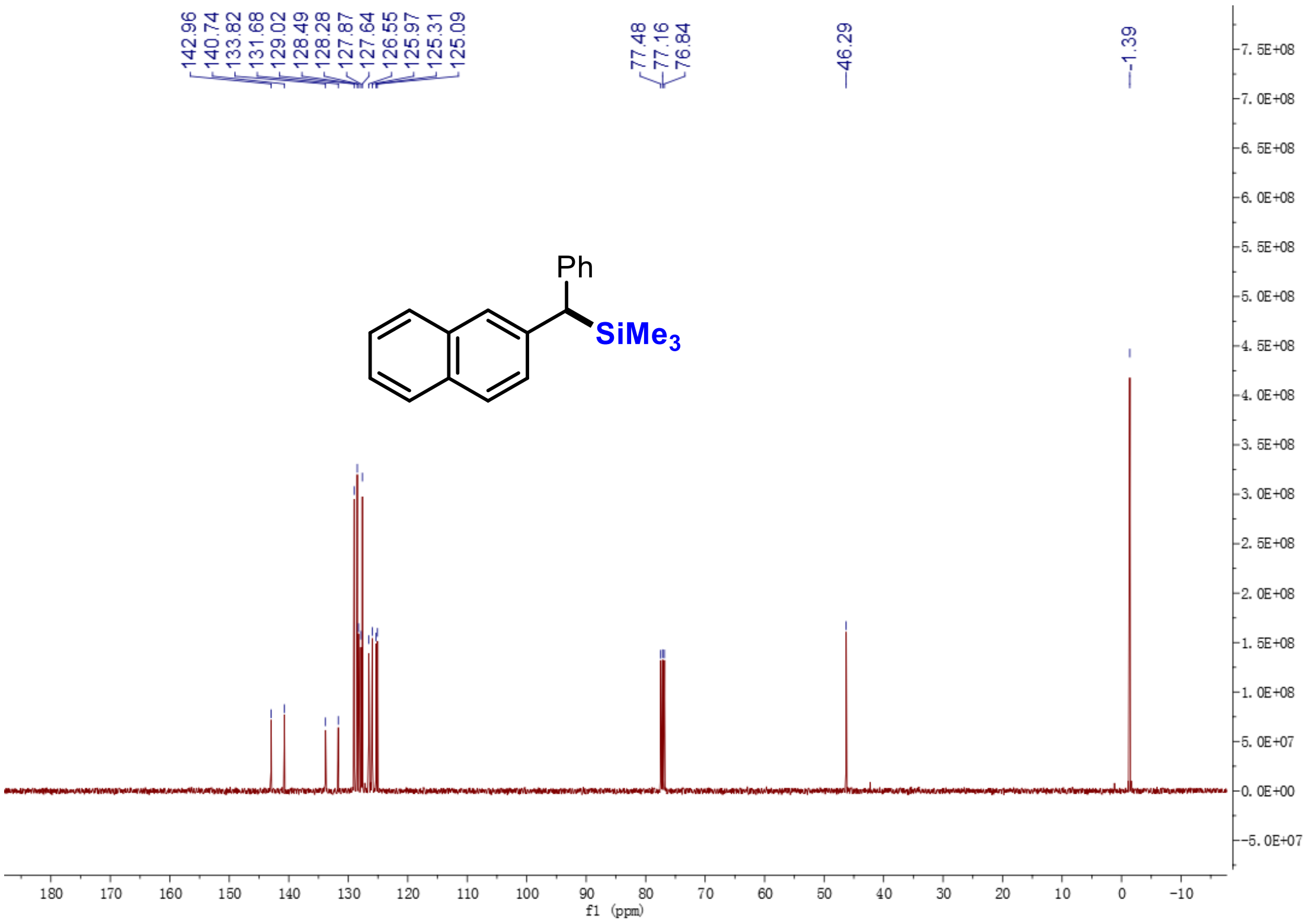

^{1}H NMR spectrum of 3ao (400 MHz, $CDCl_3$)

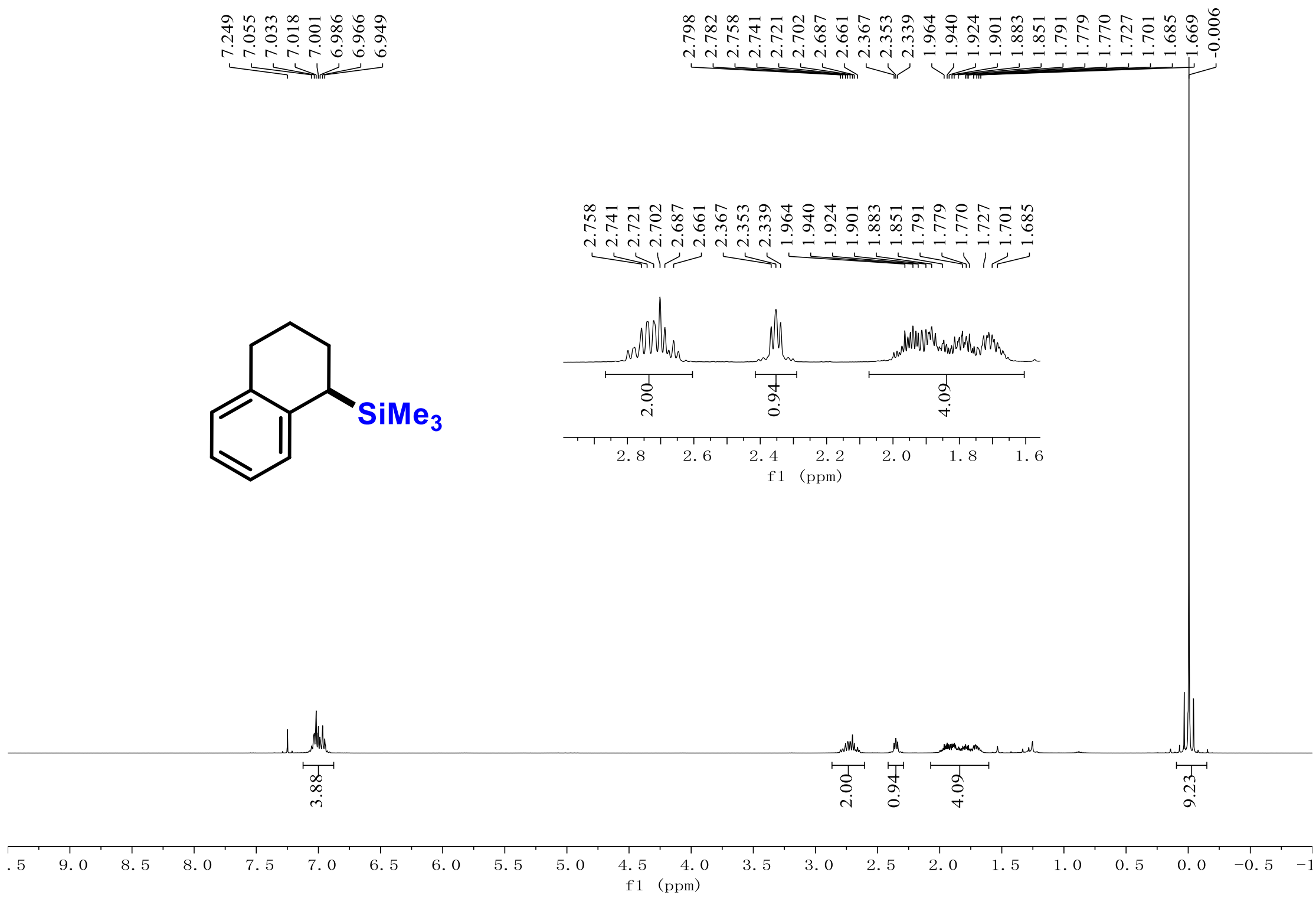

^{13}C NMR spectrum of 3ao (101 MHz, $CDCl_3$)

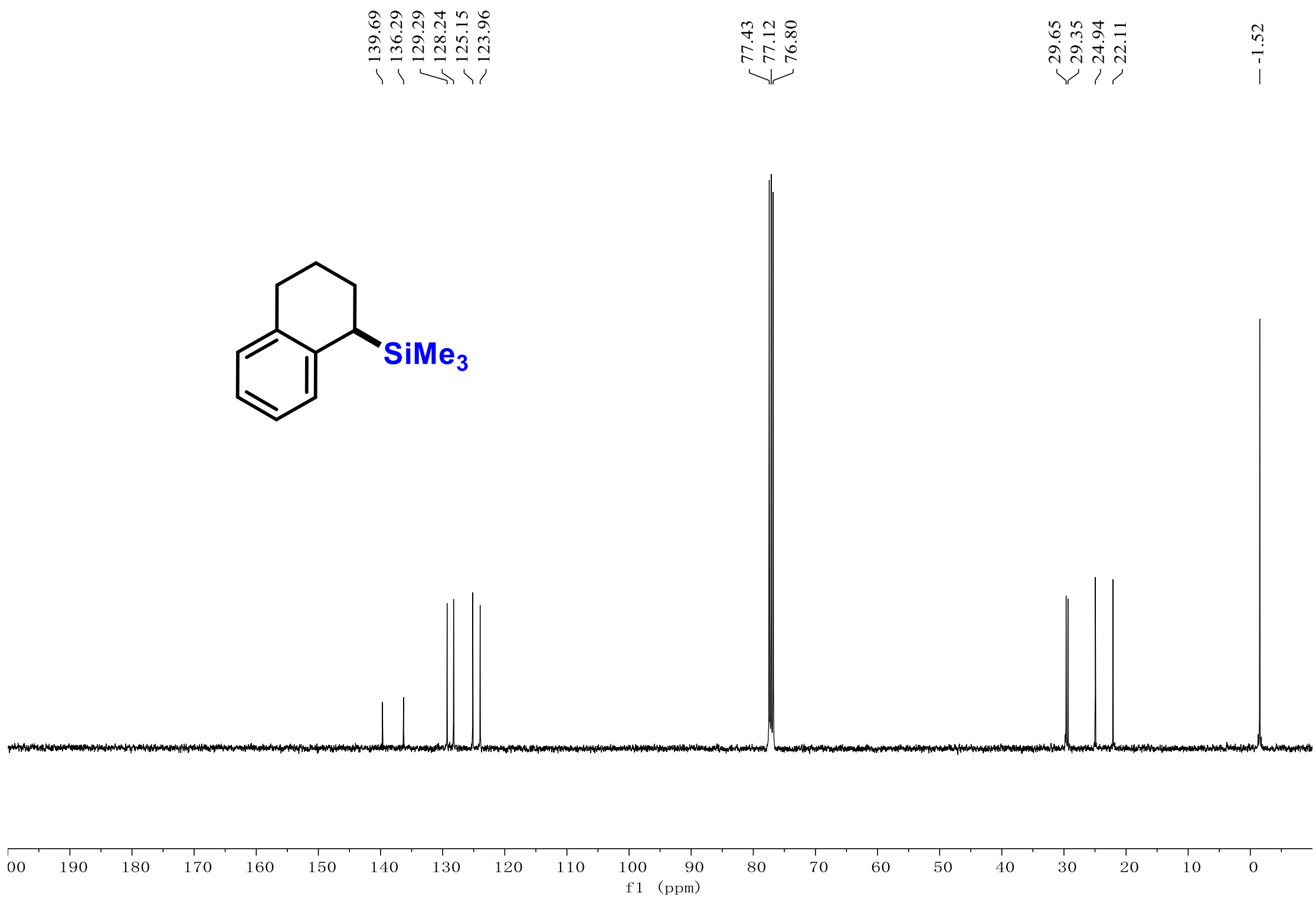

^{1}H NMR spectrum of 3ap (400 MHz, $CDCl_3$)

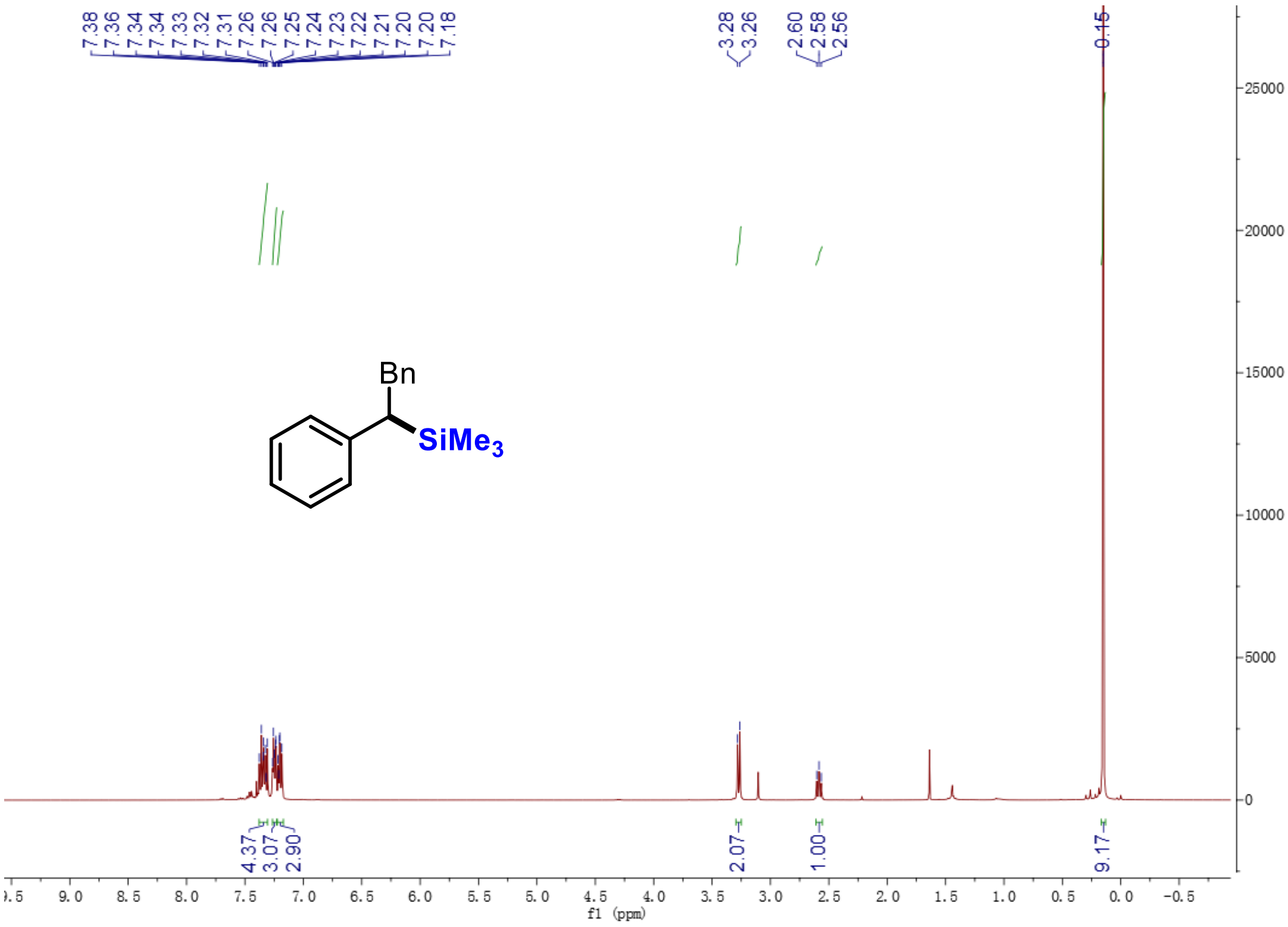

^{13}C NMR spectrum of 3ap (101 MHz, $CDCl_3$)

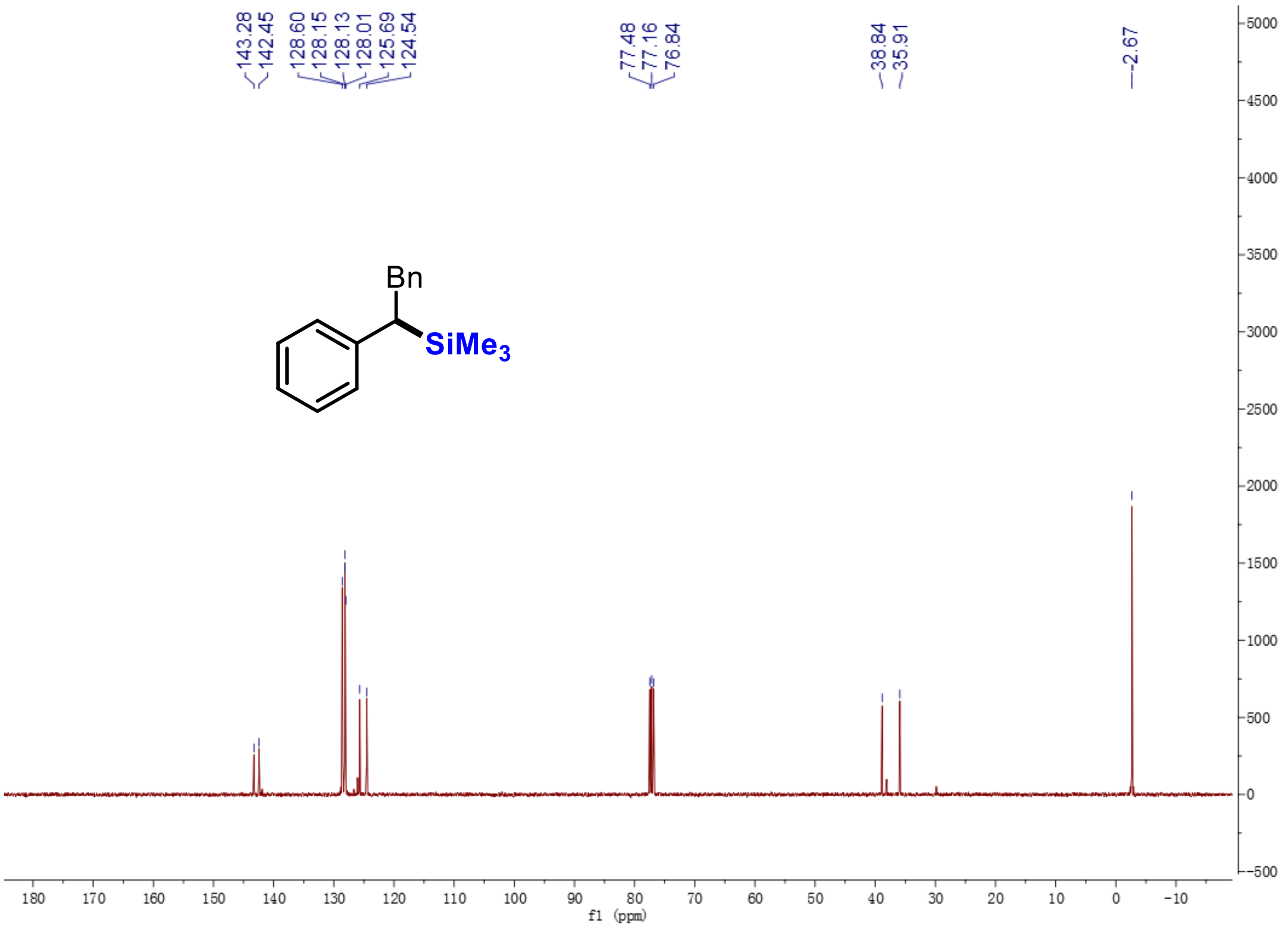

^{1}H NMR spectrum of 3aq (400 MHz, $CDCl_3$)

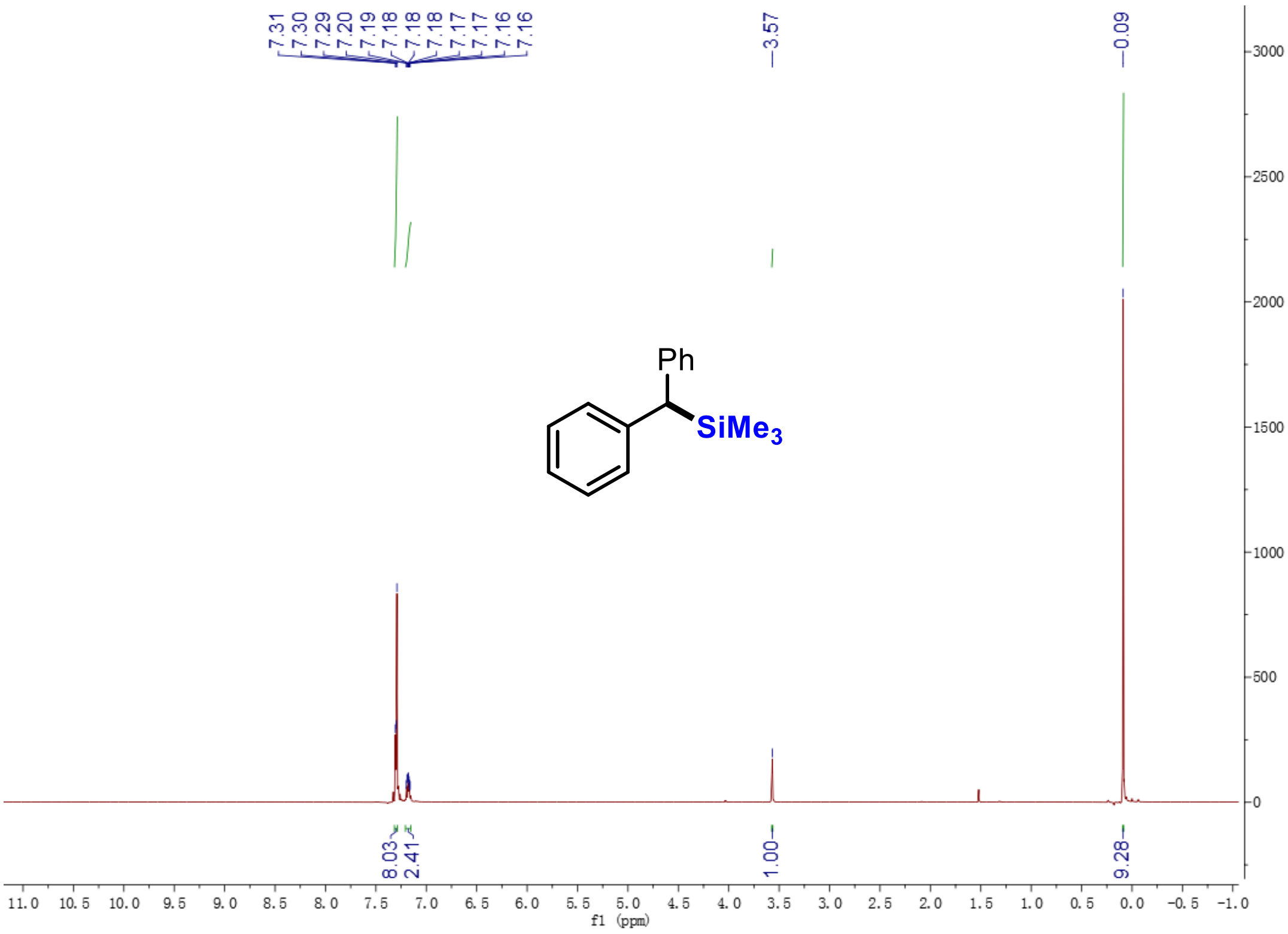

^{13}C NMR spectrum of 3aq (101 MHz, $CDCl_3$)

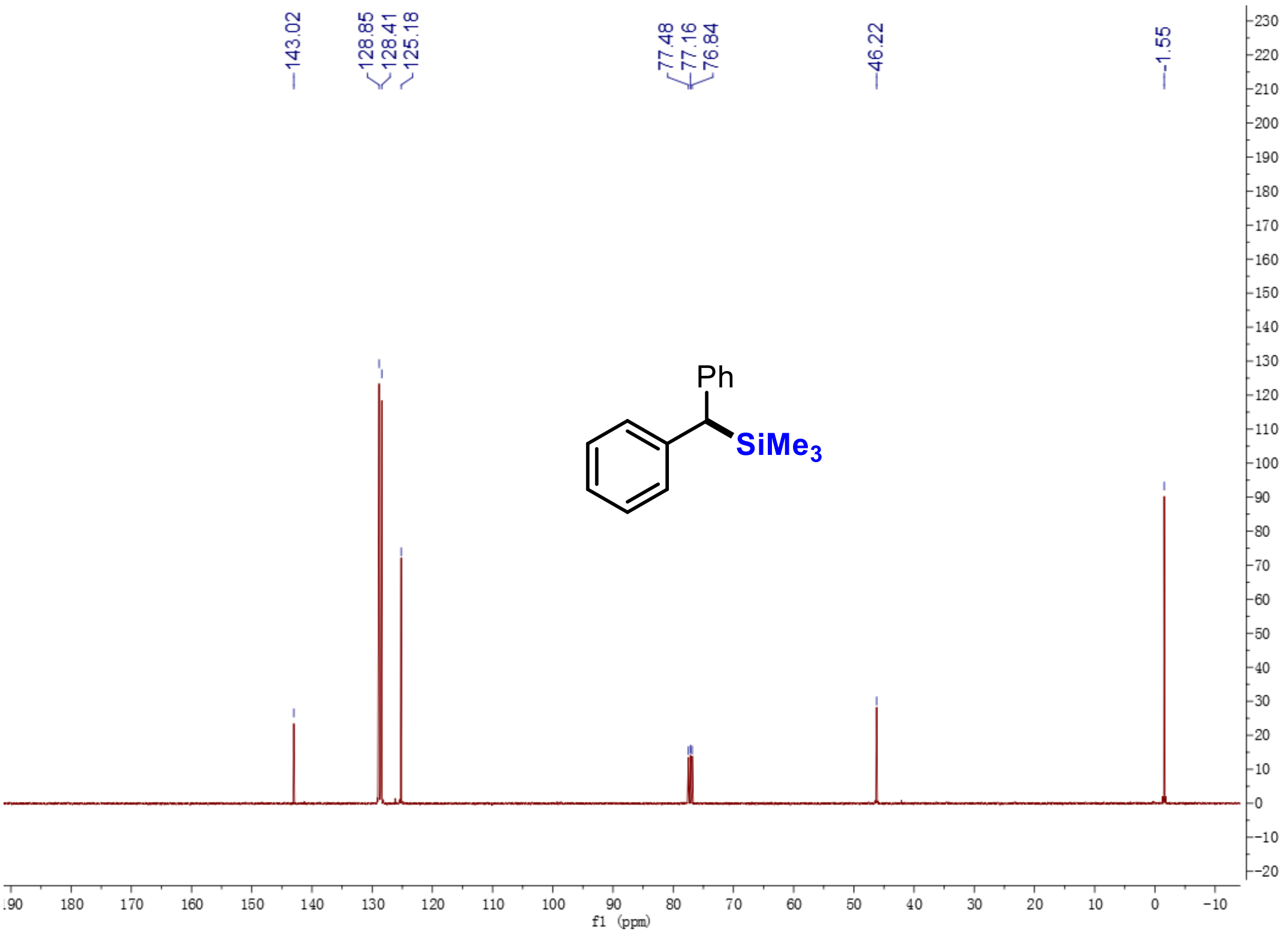

^{1}H NMR spectrum of 3ar (400 MHz, $CDCl_3$)

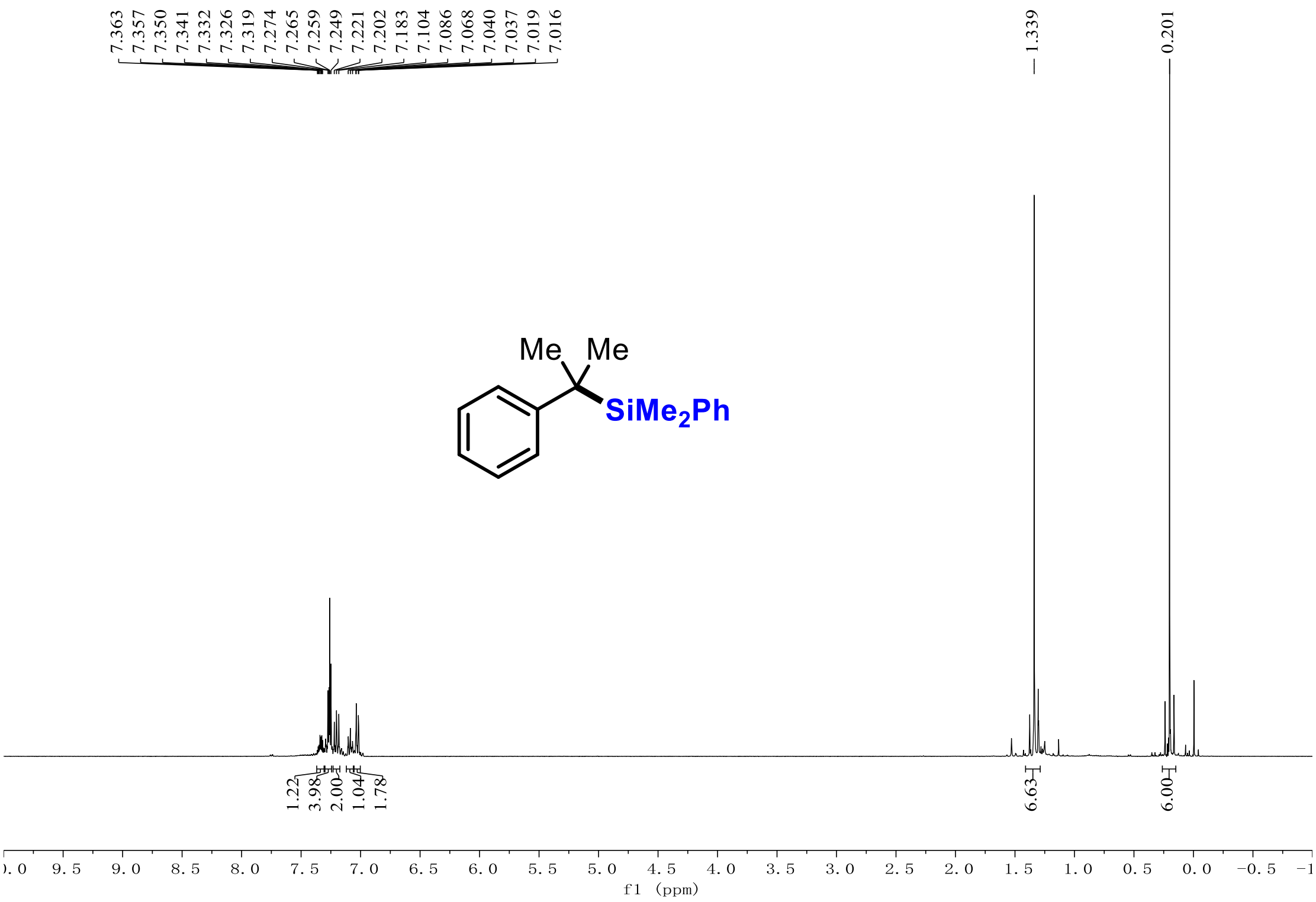

^{13}C NMR spectrum of 3ar (101 MHz, $CDCl_3$)

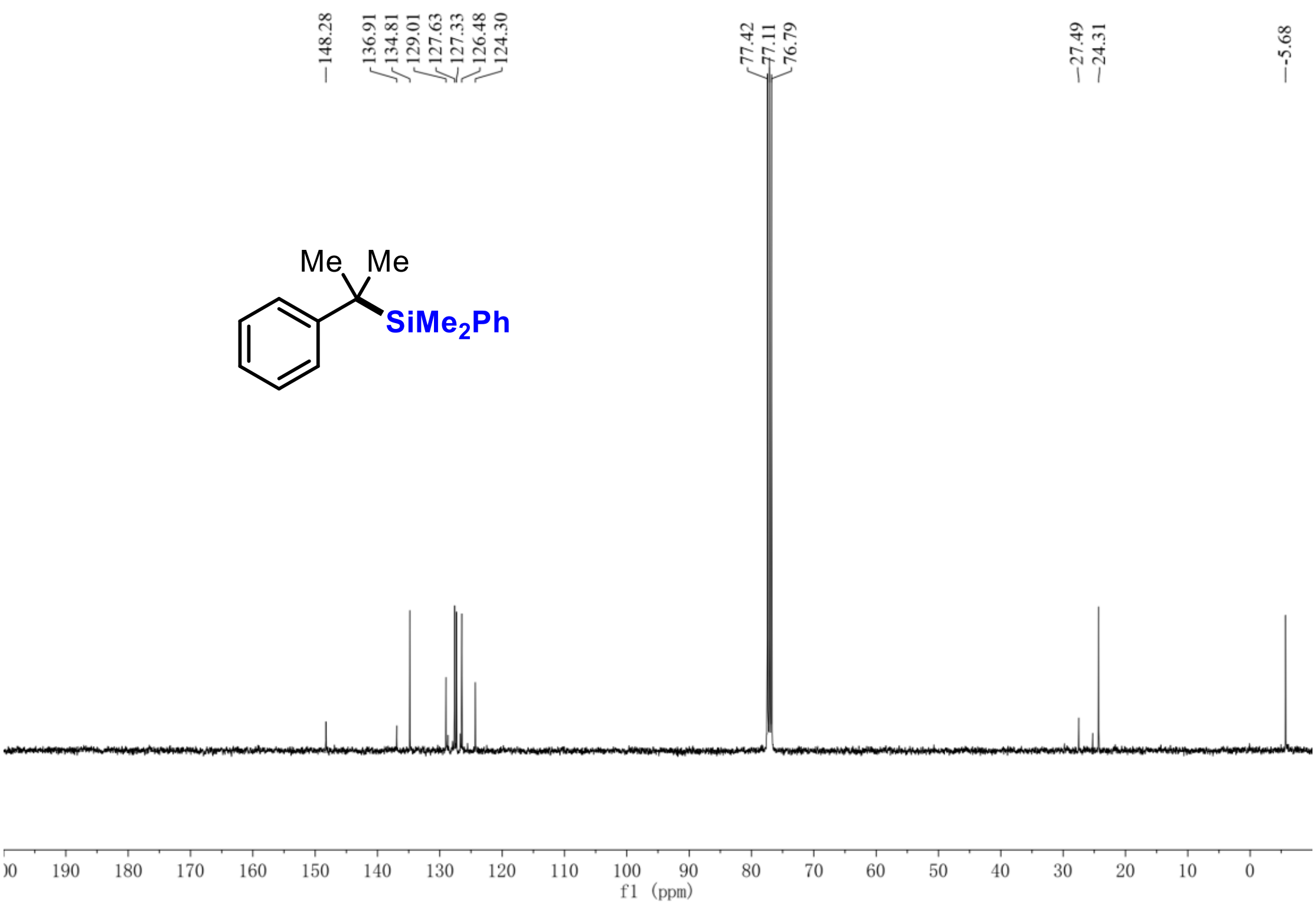

^{1}H NMR spectrum of 3as (400 MHz, $CDCl_3$)

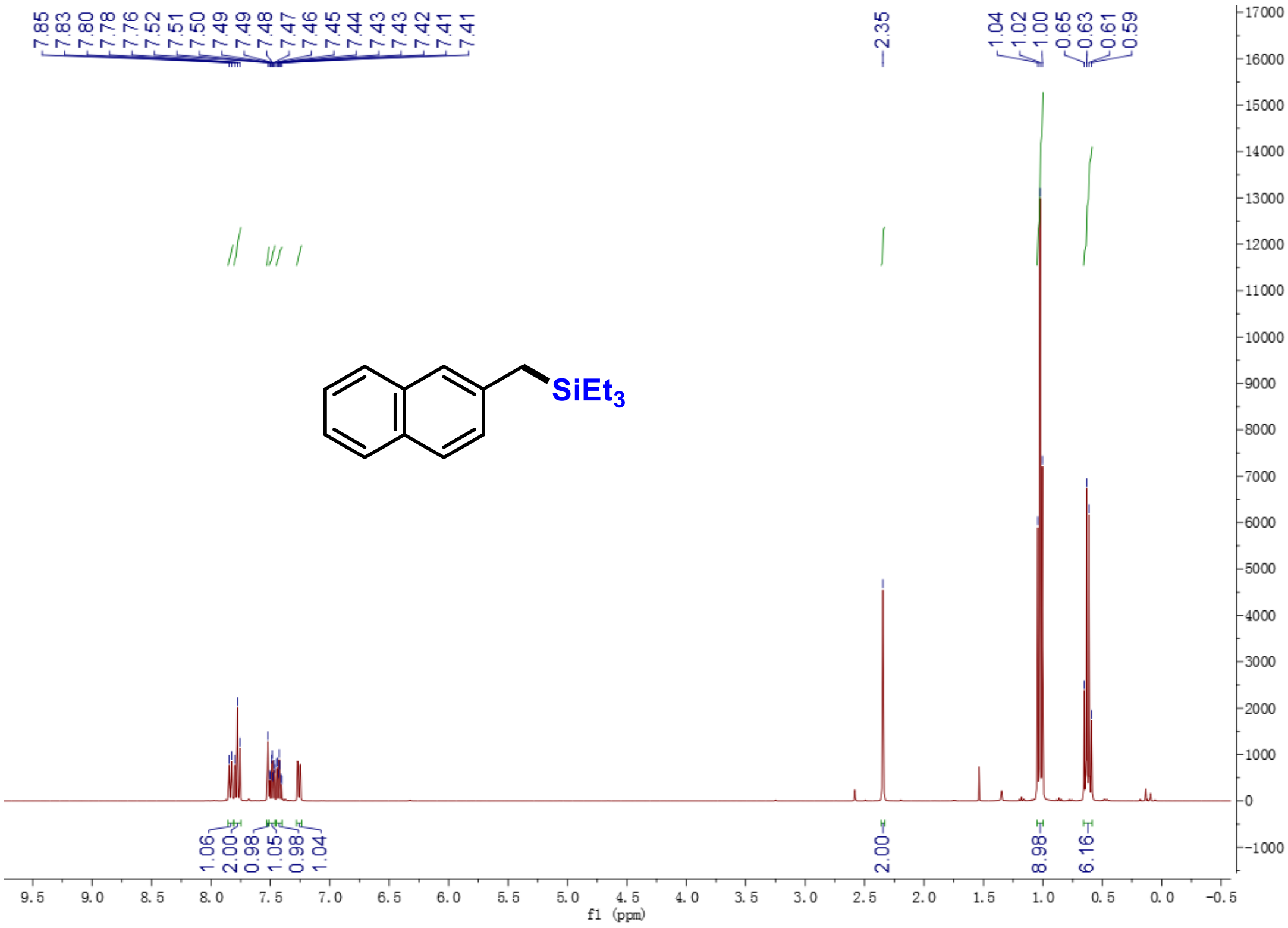

^{13}C NMR spectrum of 3as (101 MHz, $CDCl_3$)

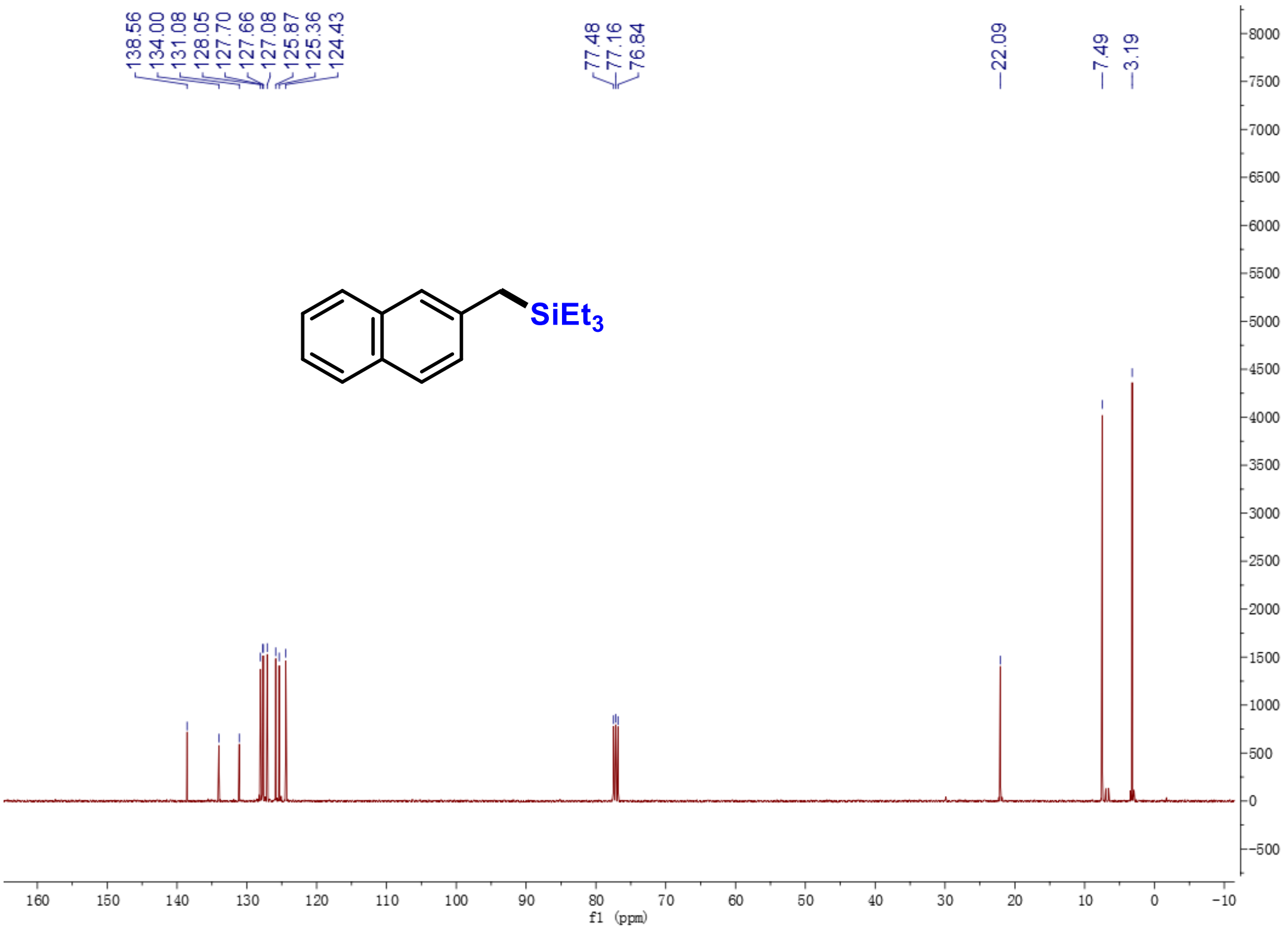

^{1}H NMR spectrum of 3at (400 MHz, $CDCl_3$)

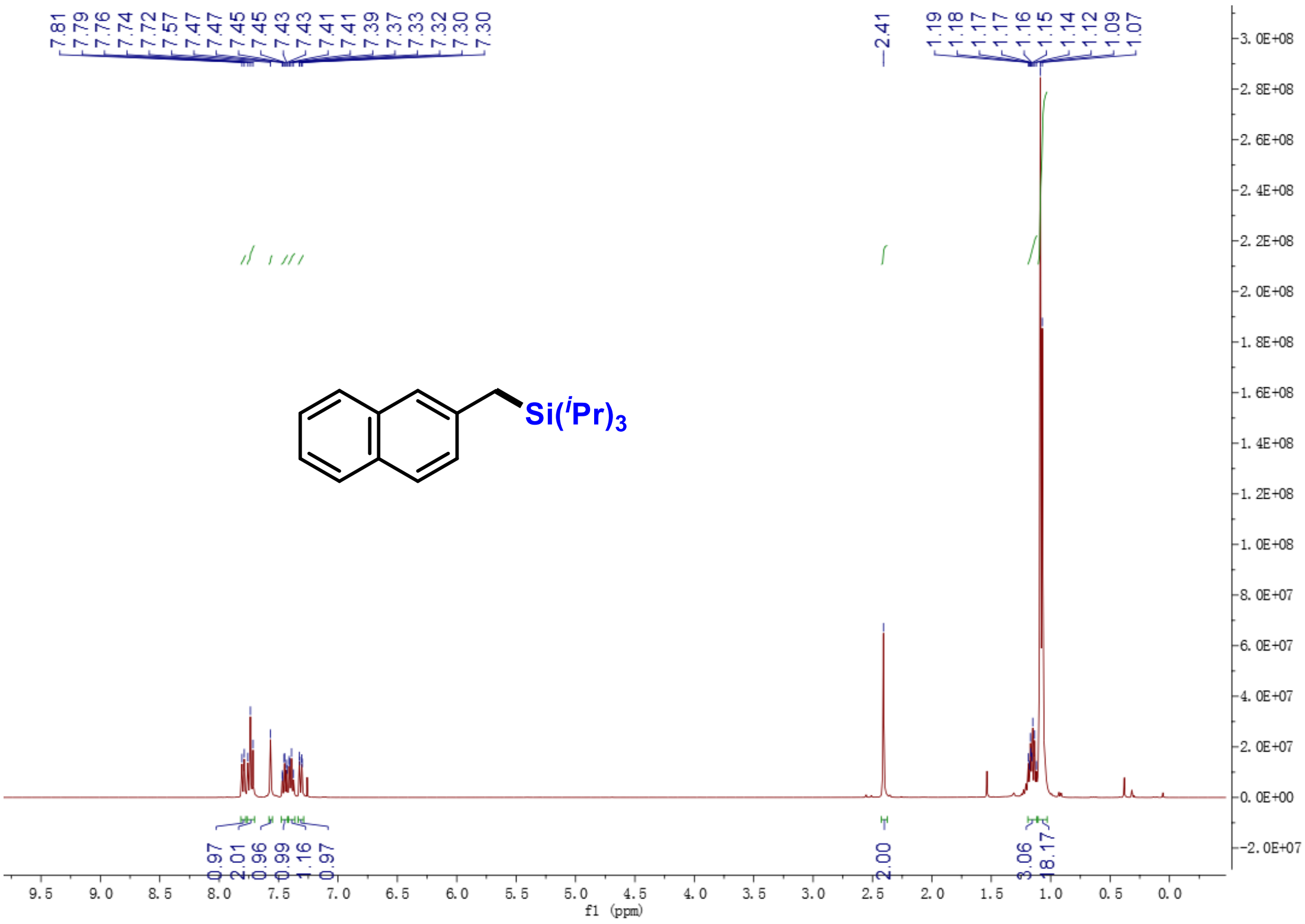

^{13}C NMR spectrum of 3at (101 MHz, $CDCl_3$)

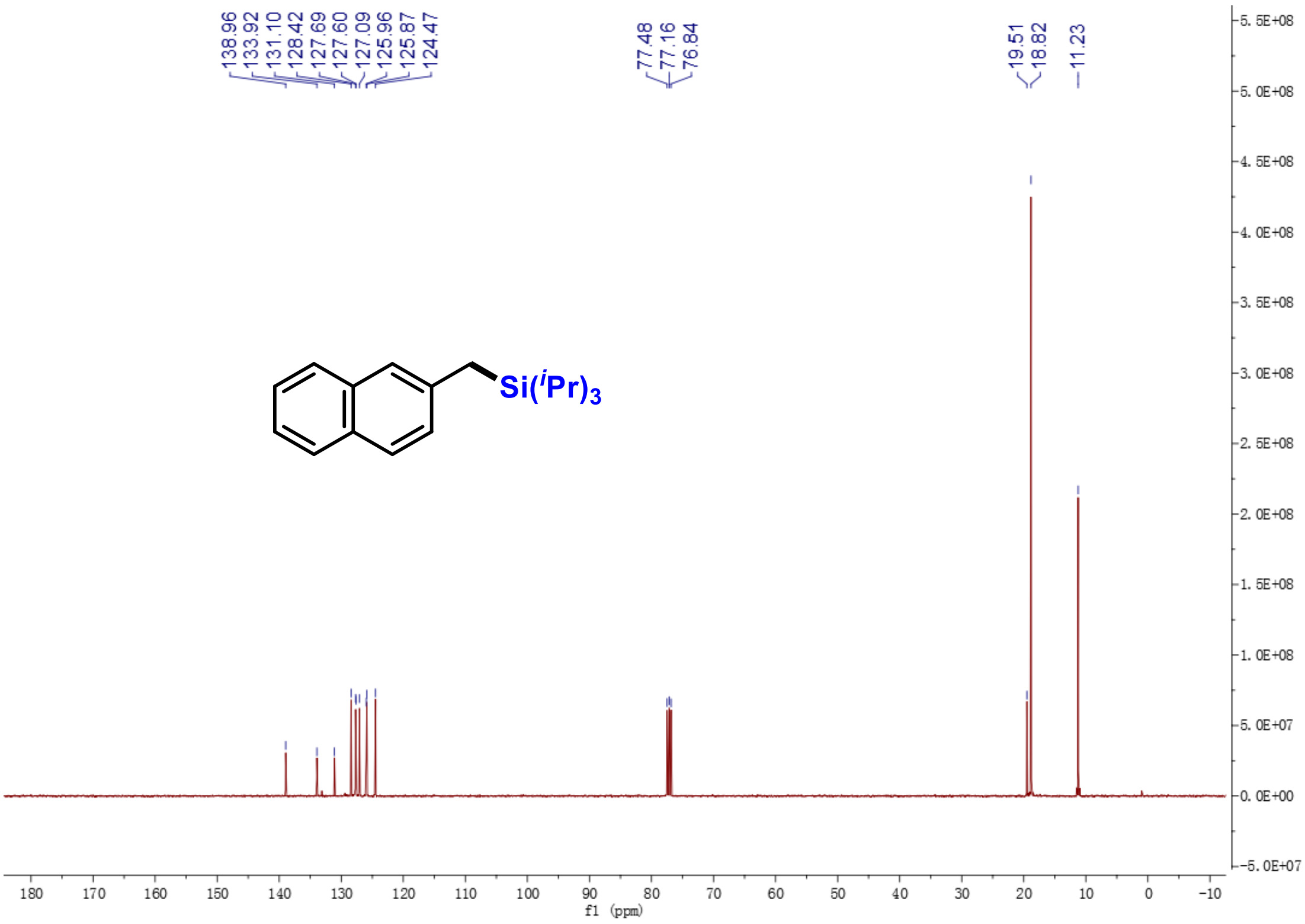

^{1}H NMR spectrum of 3au (400 MHz, $CDCl_3$)

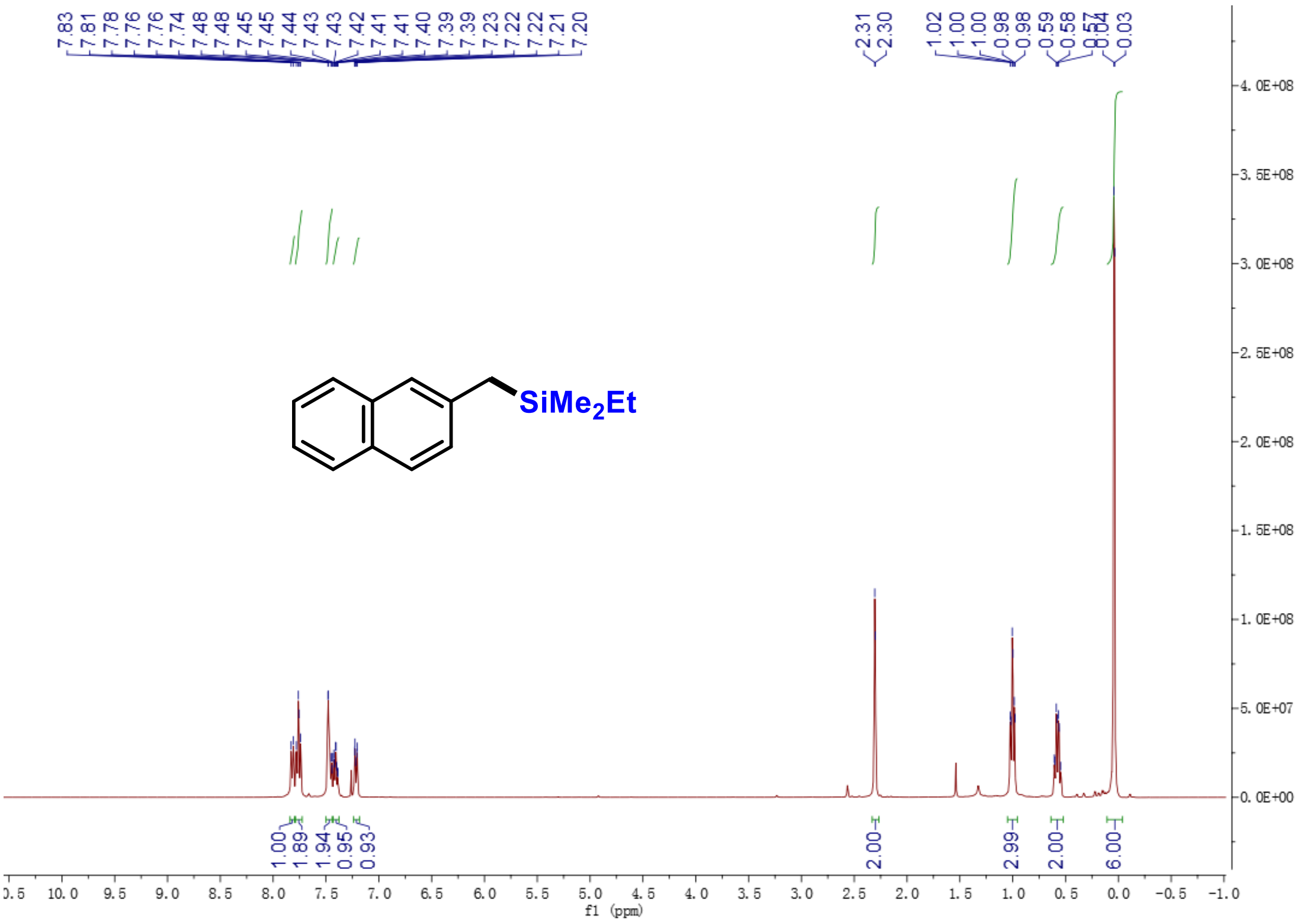

^{13}C NMR spectrum of 3au (101 MHz, $CDCl_3$)

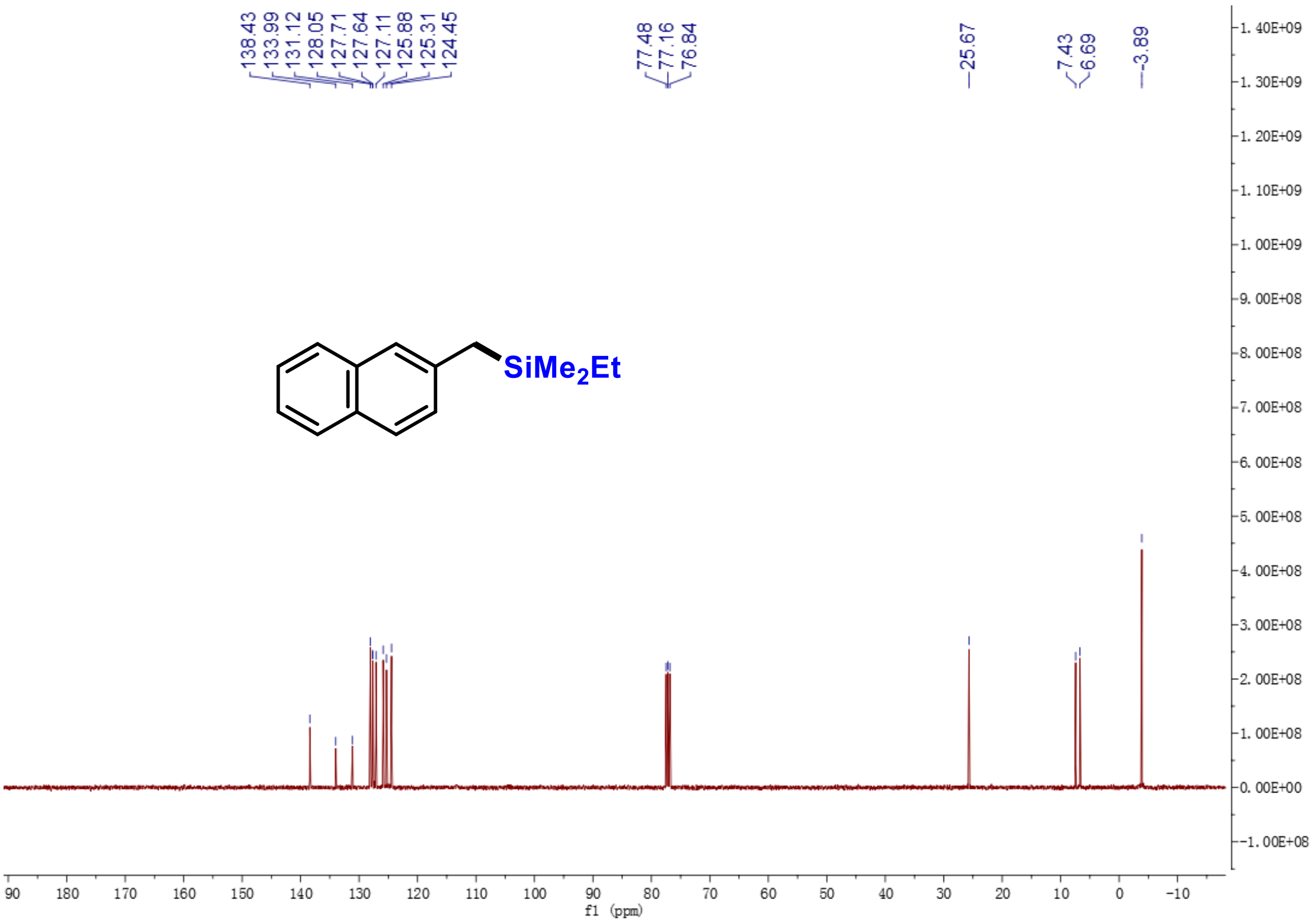

^{1}H NMR spectrum of 3av (400 MHz, $CDCl_3$)

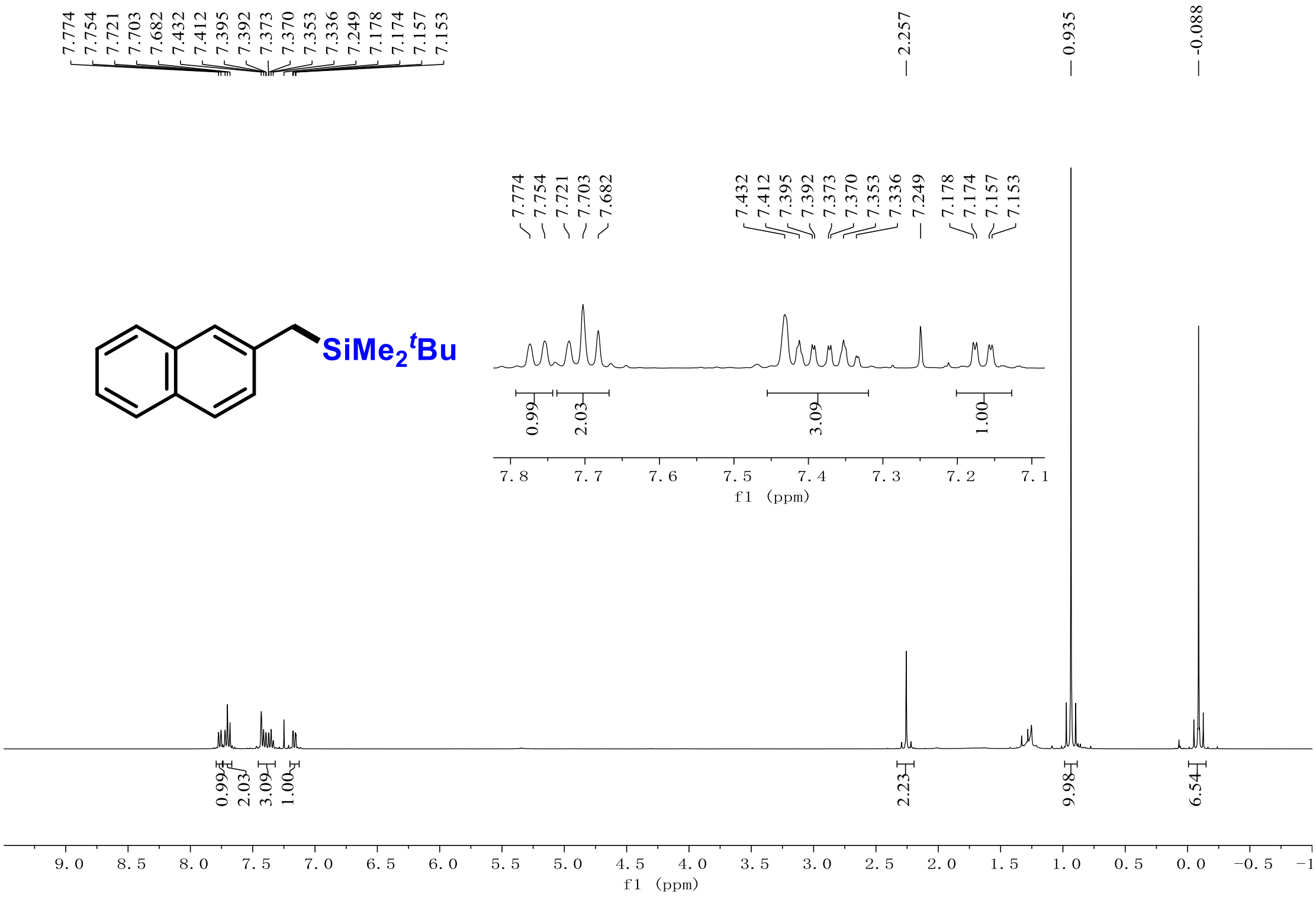

^{13}C NMR spectrum of 3av (101 MHz, $CDCl_3$)

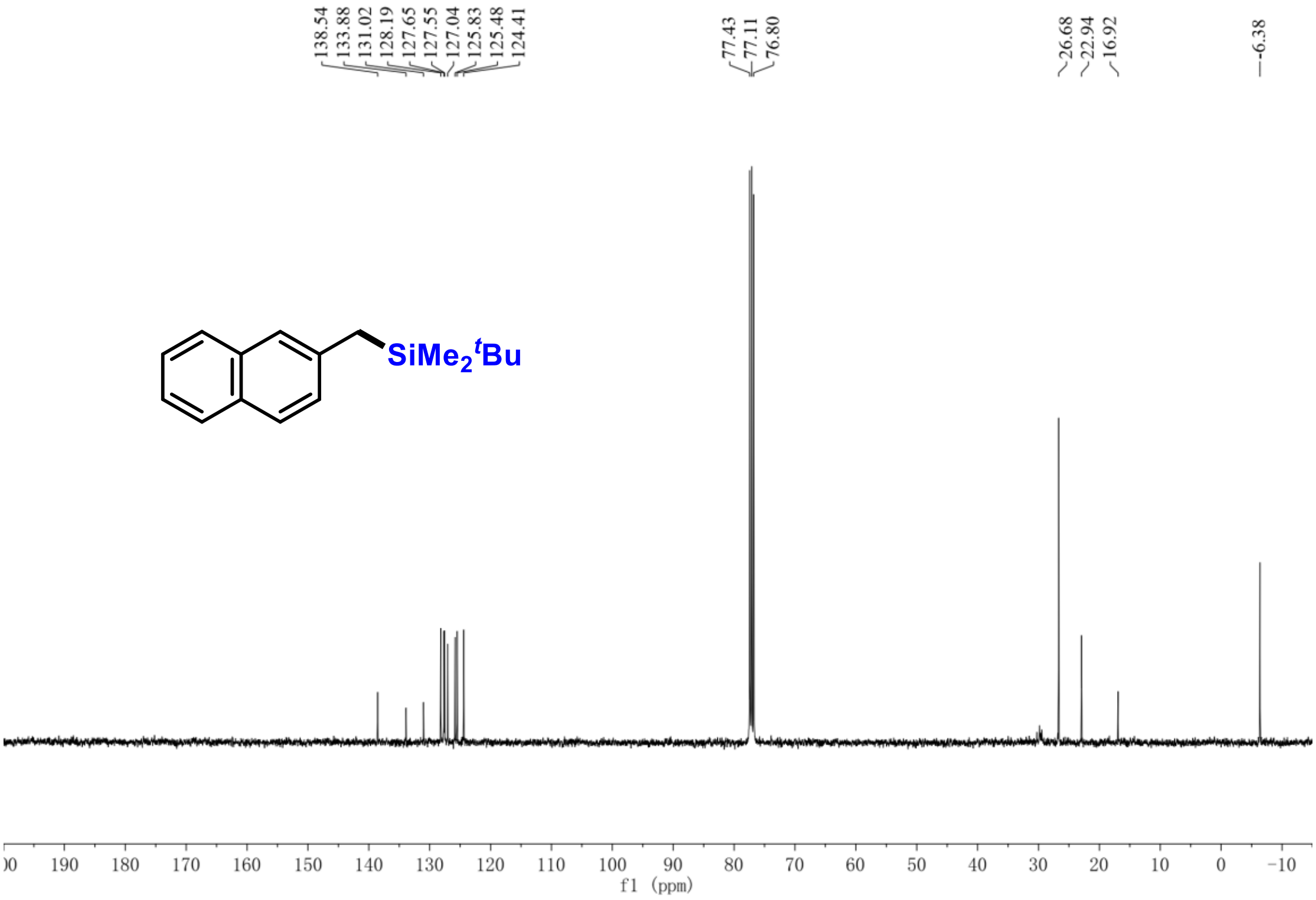

^{1}H NMR spectrum of 3aw (400 MHz, $CDCl_3$)

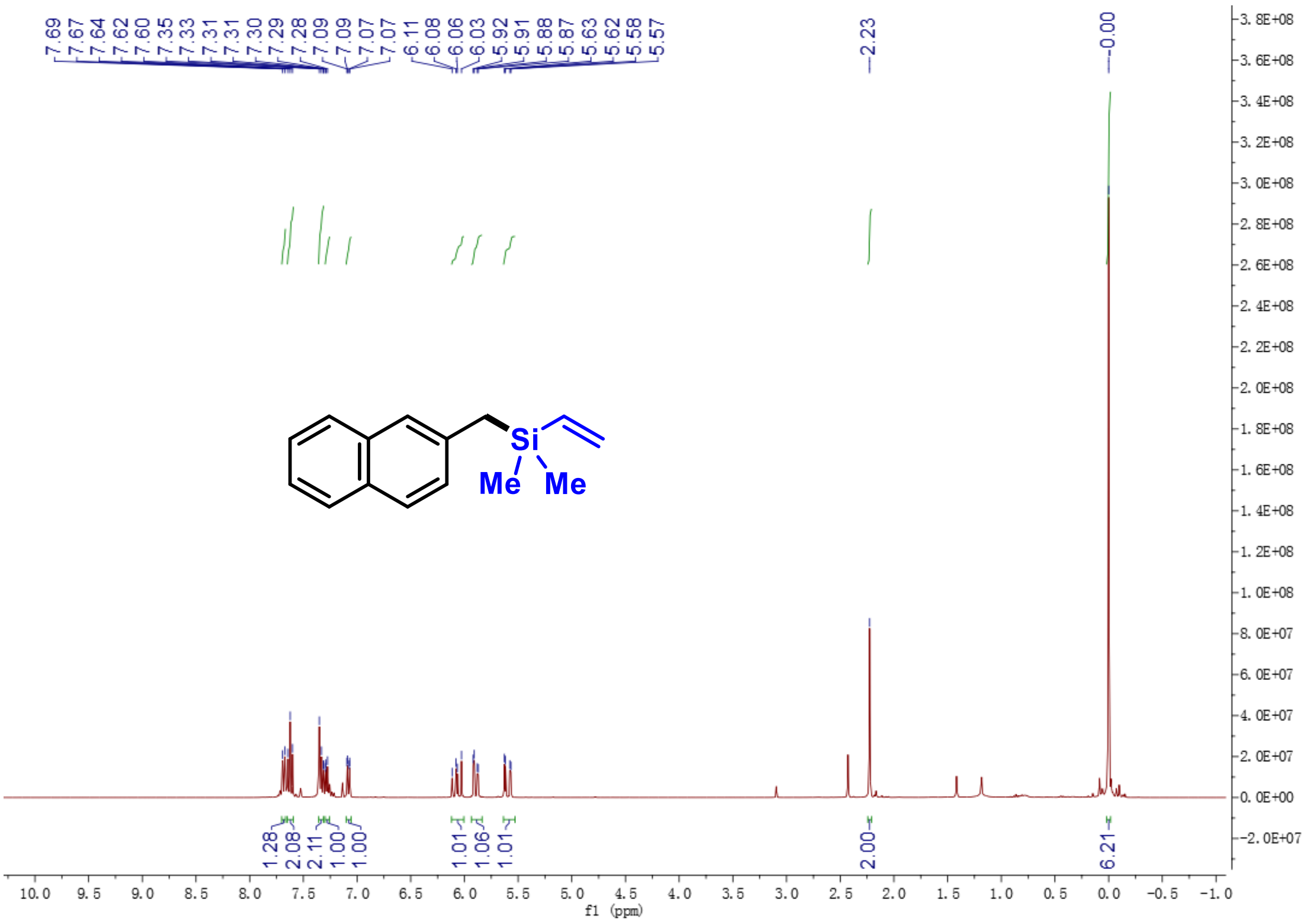

^{13}C NMR spectrum of 3aw (101 MHz, $CDCl_3$)

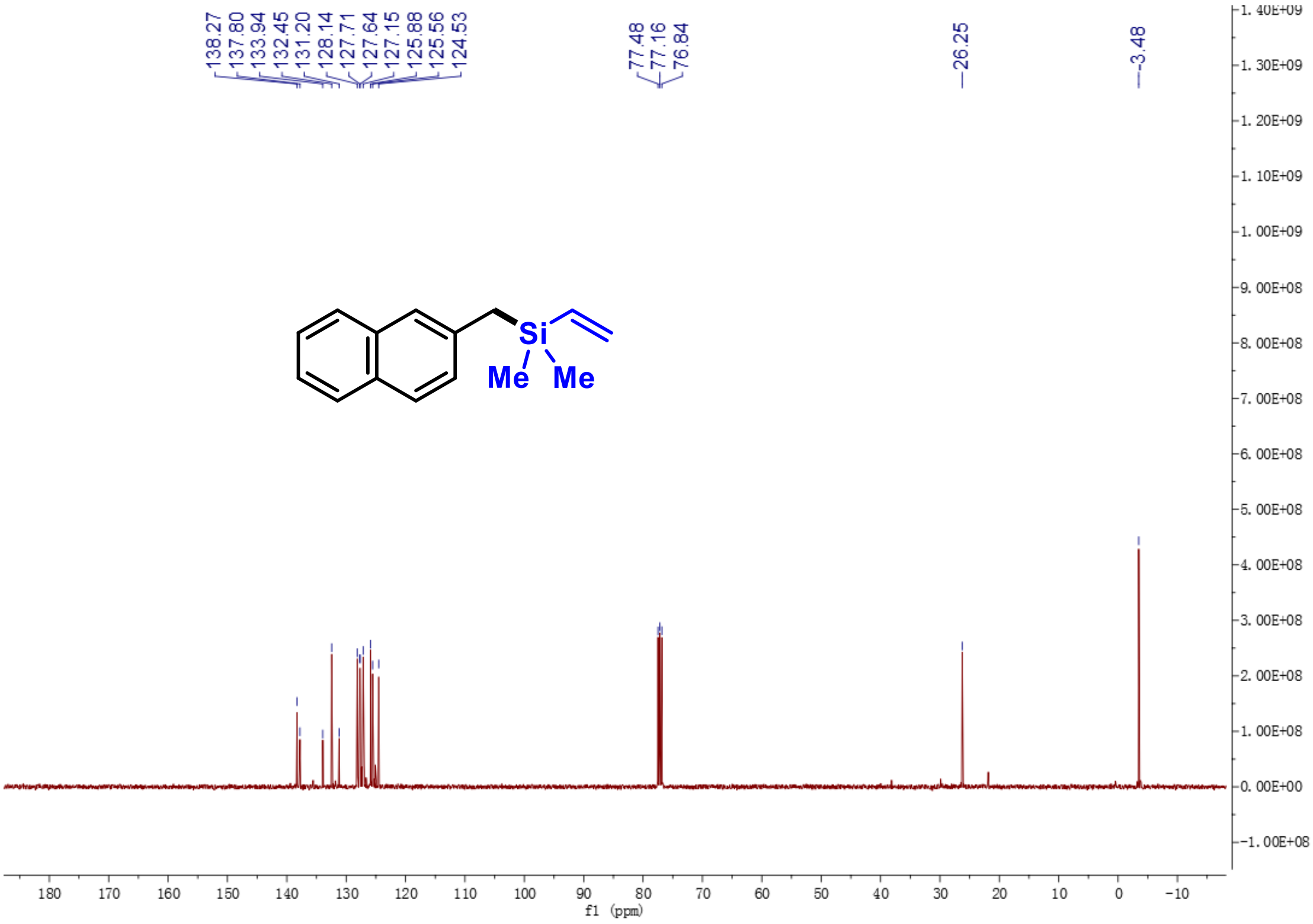

^{1}H NMR spectrum of 3ax (400 MHz, $CDCl_3$)

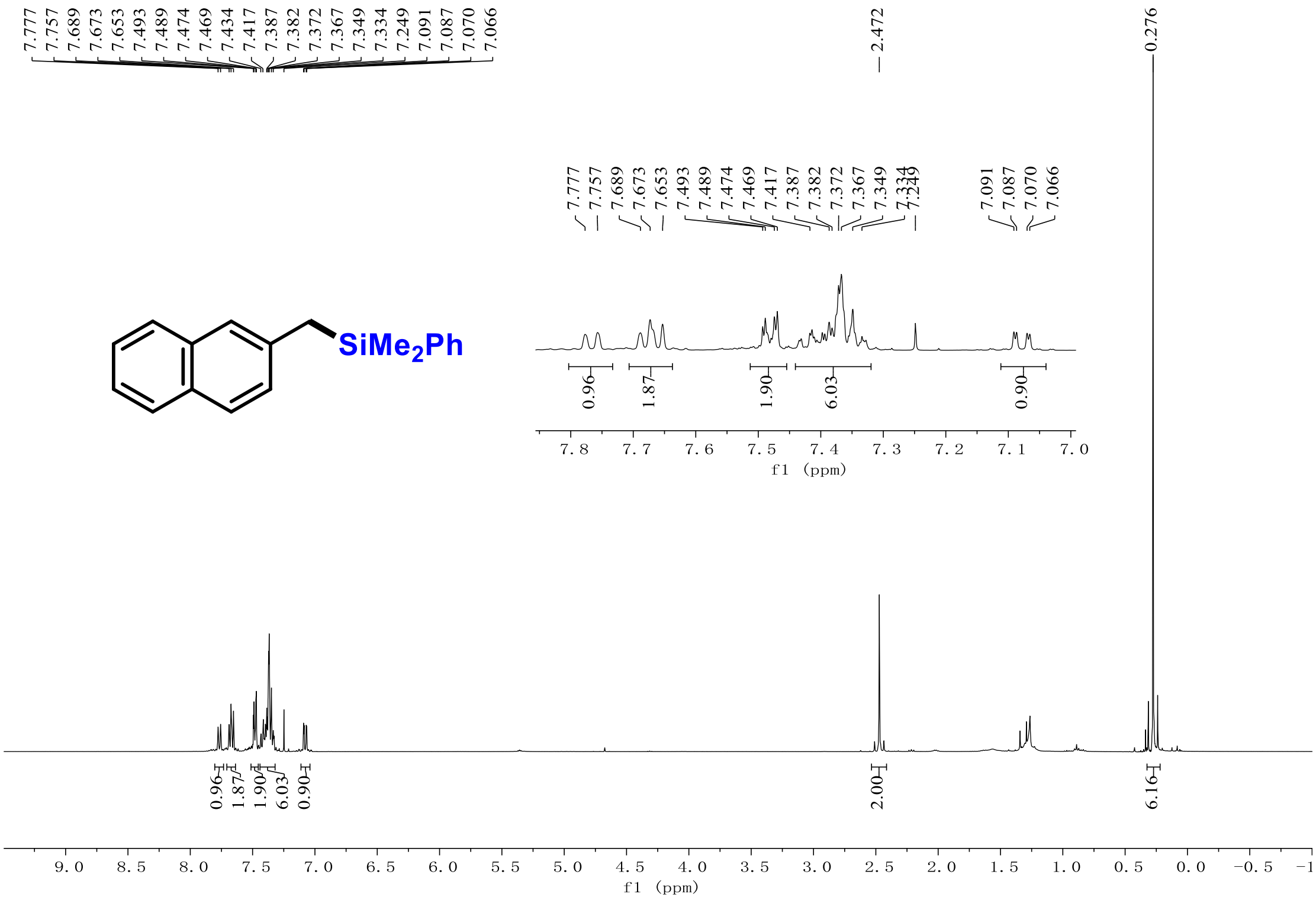

^{13}C NMR spectrum of 3ax (101 MHz, $CDCl_3$)

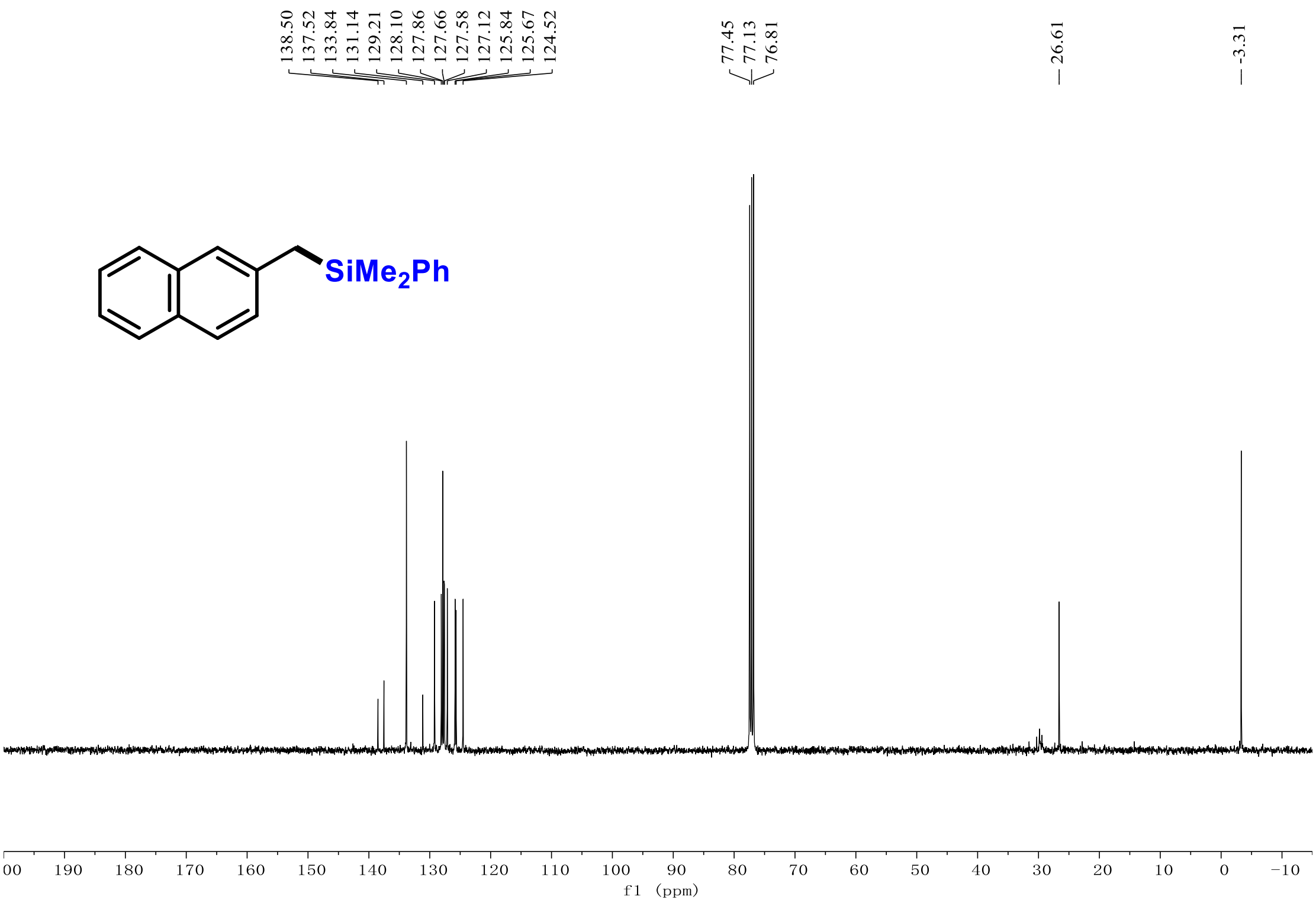

^{1}H NMR spectrum of 3ay (400 MHz, $CDCl_3$)

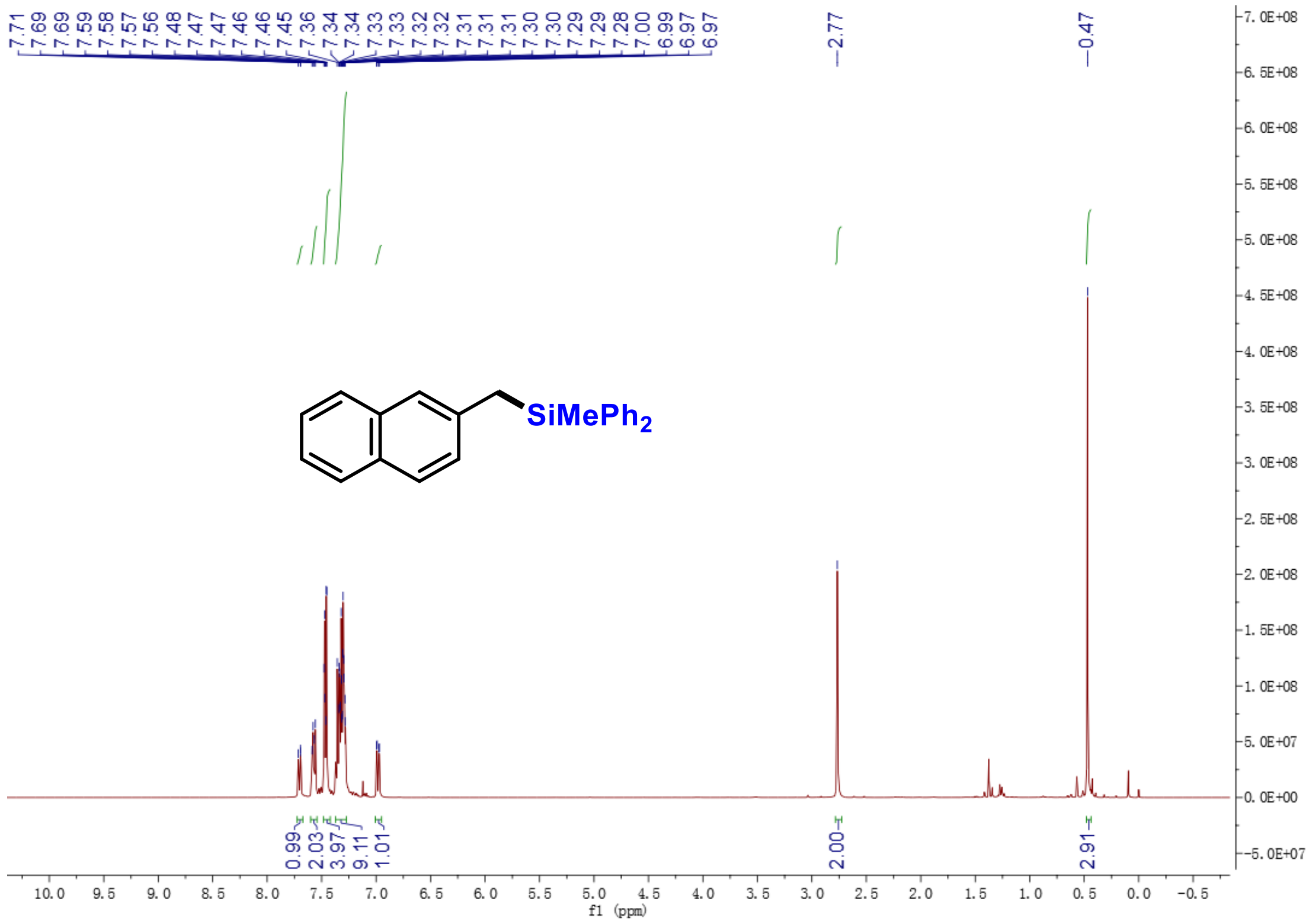

^{13}C NMR spectrum of 3ay (101 MHz, $CDCl_3$)

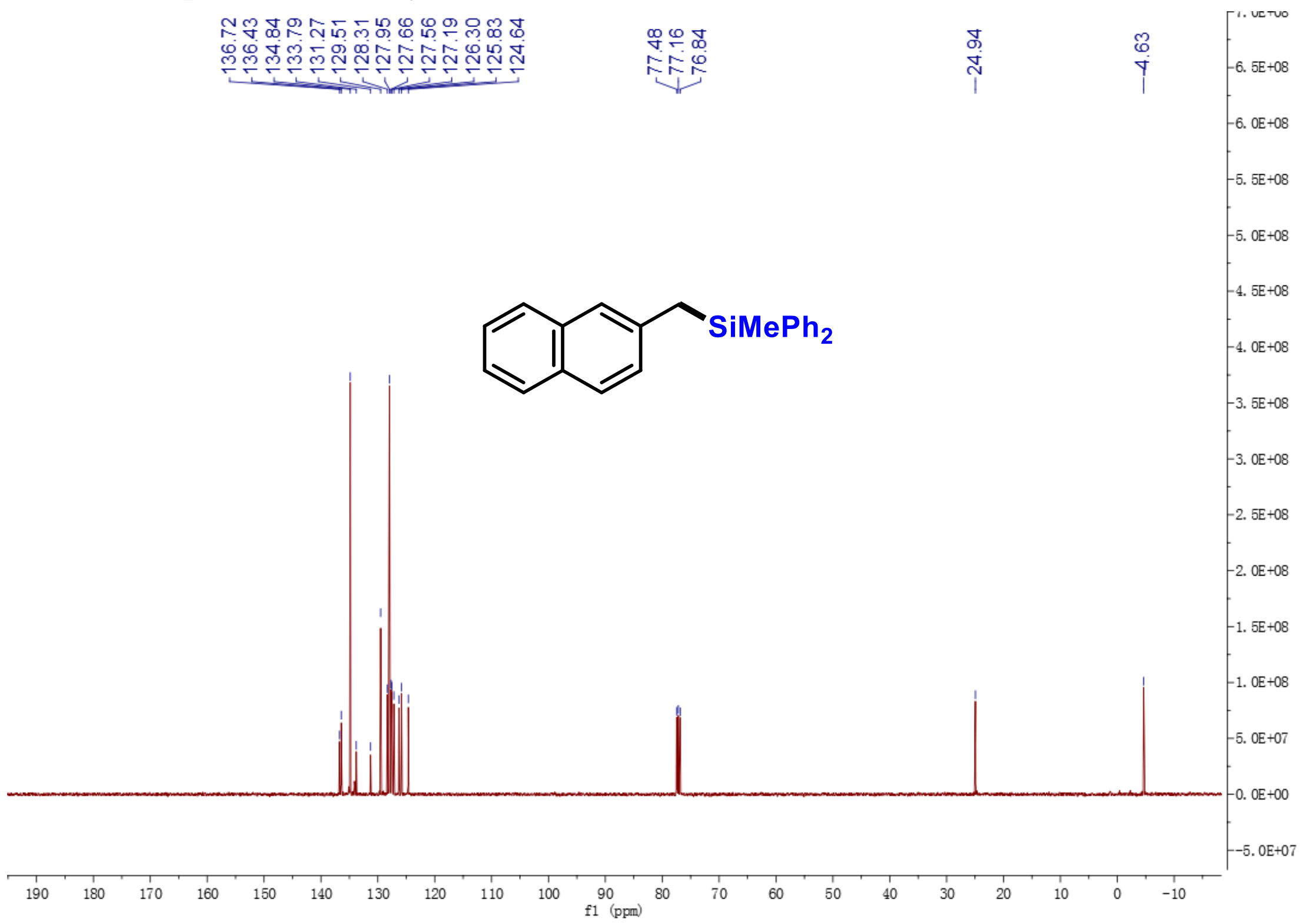

^{1}H NMR spectrum of 3az (400 MHz, $CDCl_3$)

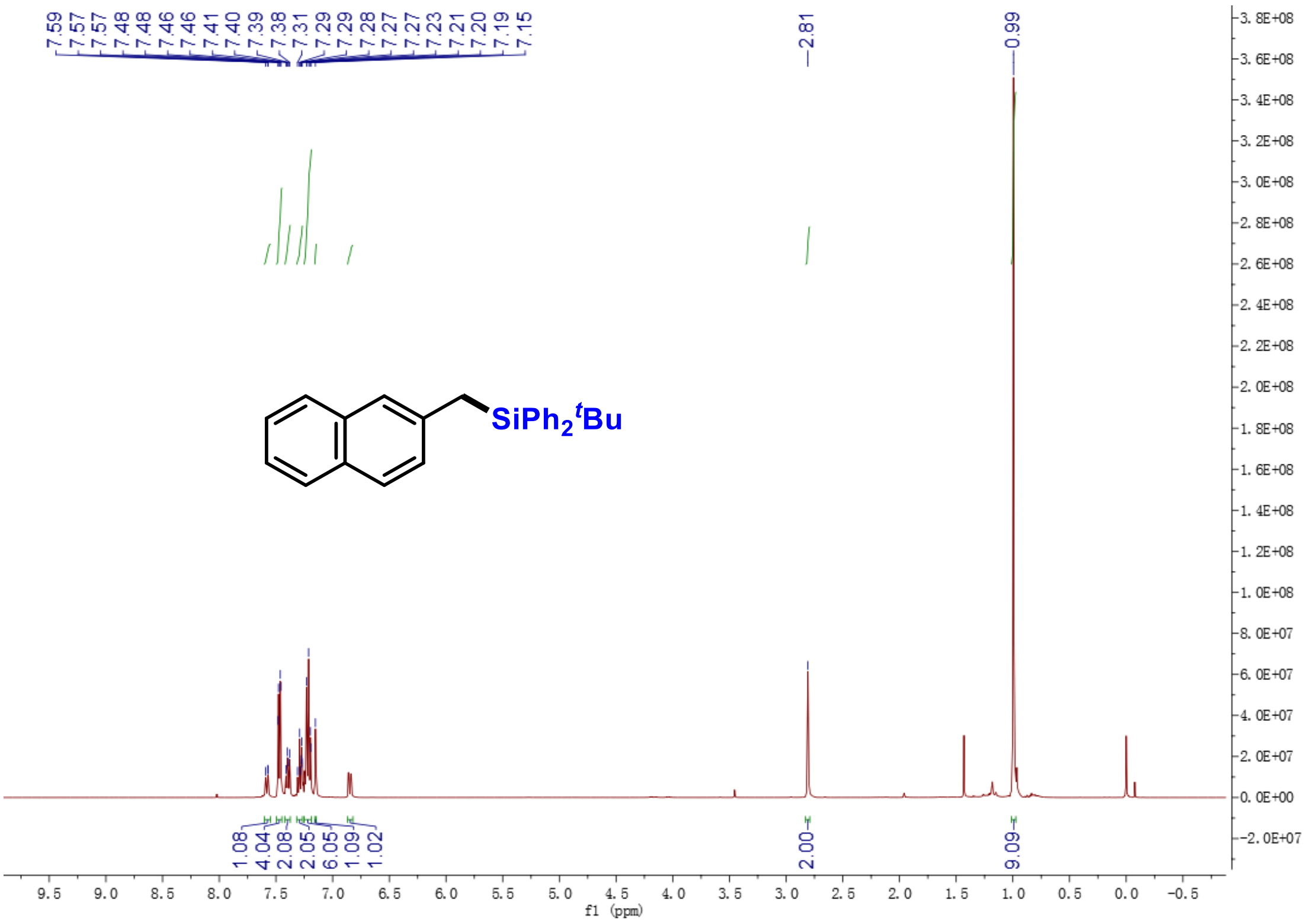

^{13}C NMR spectrum of 3az (101 MHz, $CDCl_3$)

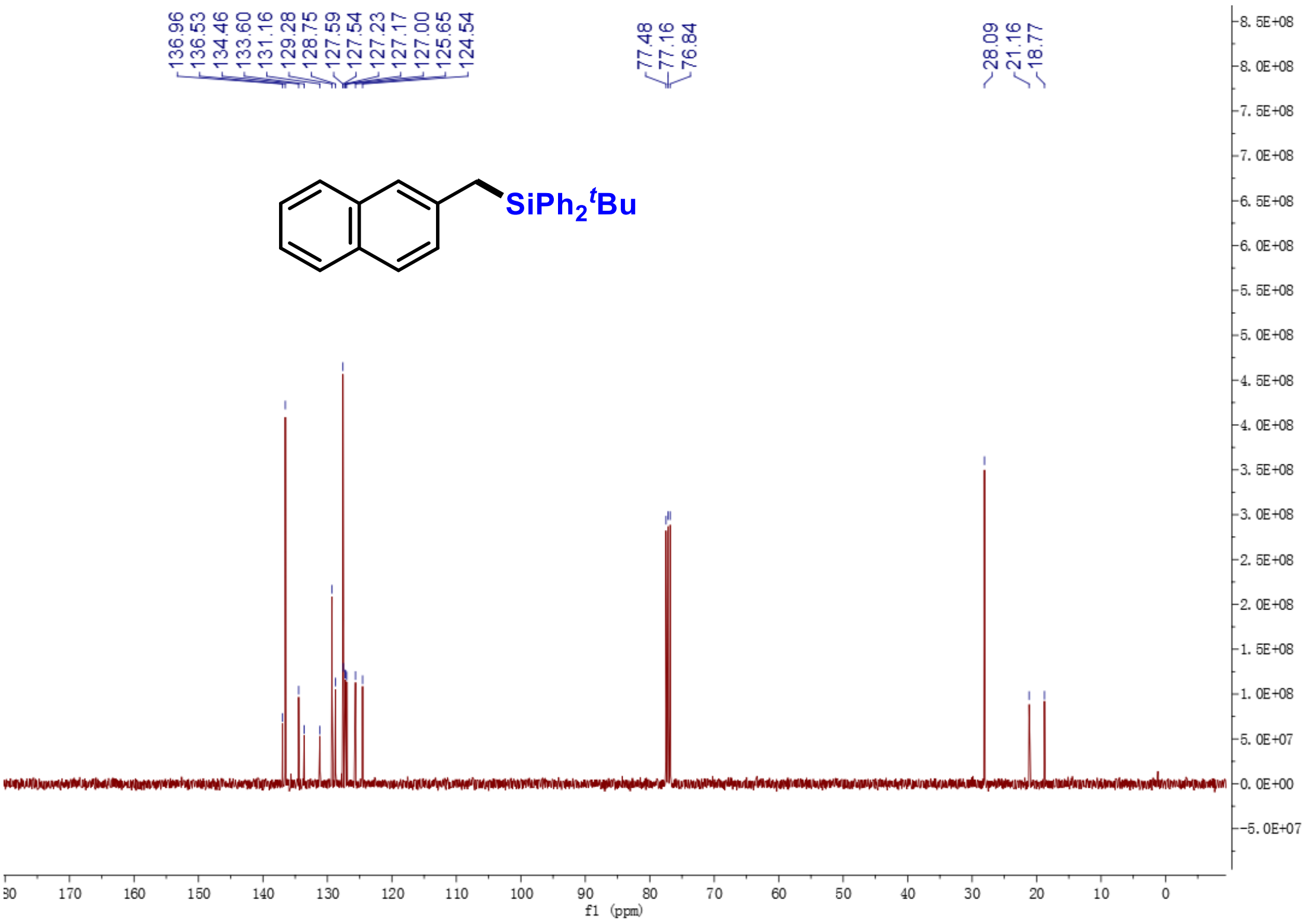

^{1}H NMR spectrum of 3aaa (400 MHz, $CDCl_3$)

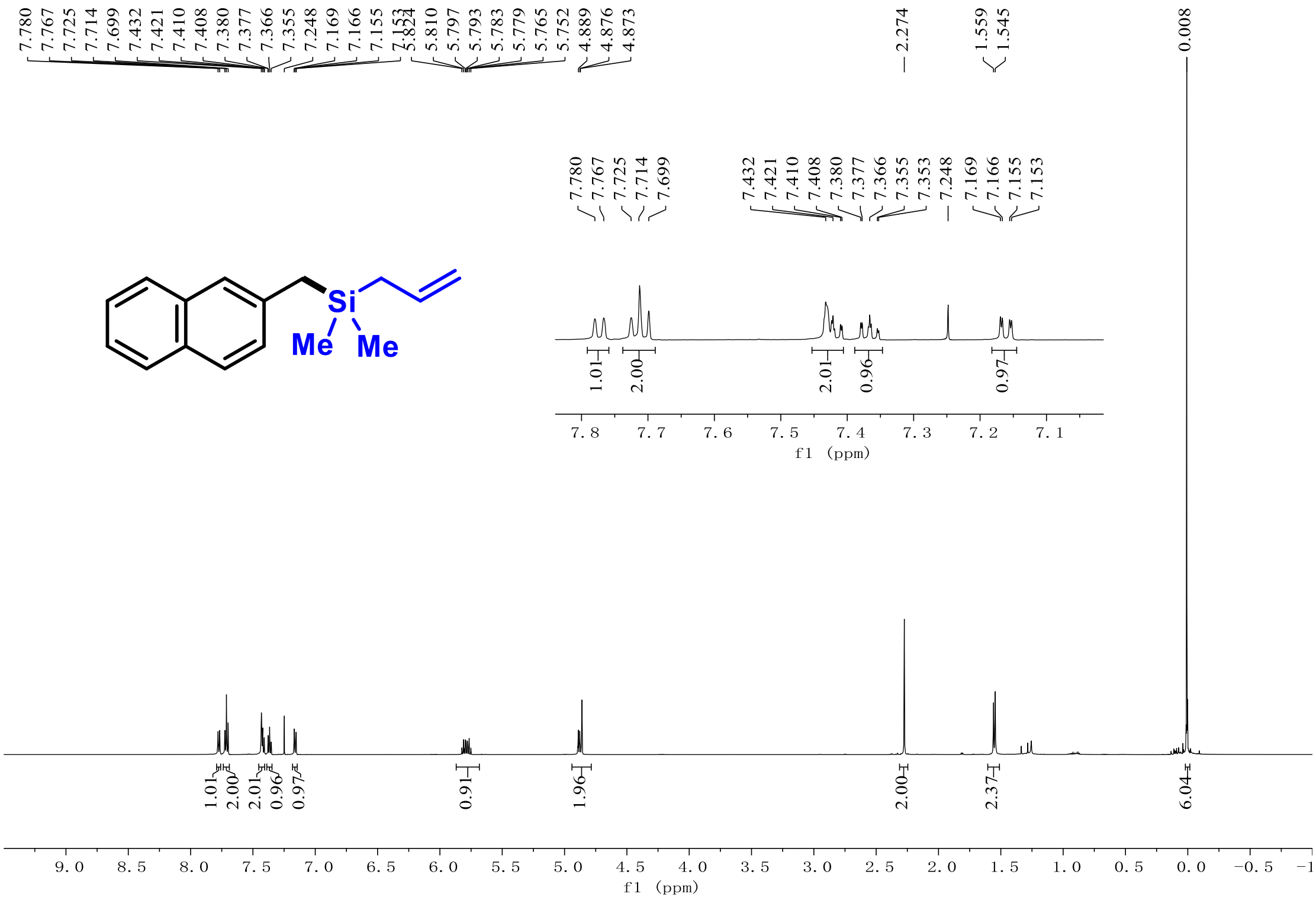

^{13}C NMR spectrum of 3aaa (101 MHz, $CDCl_3$)

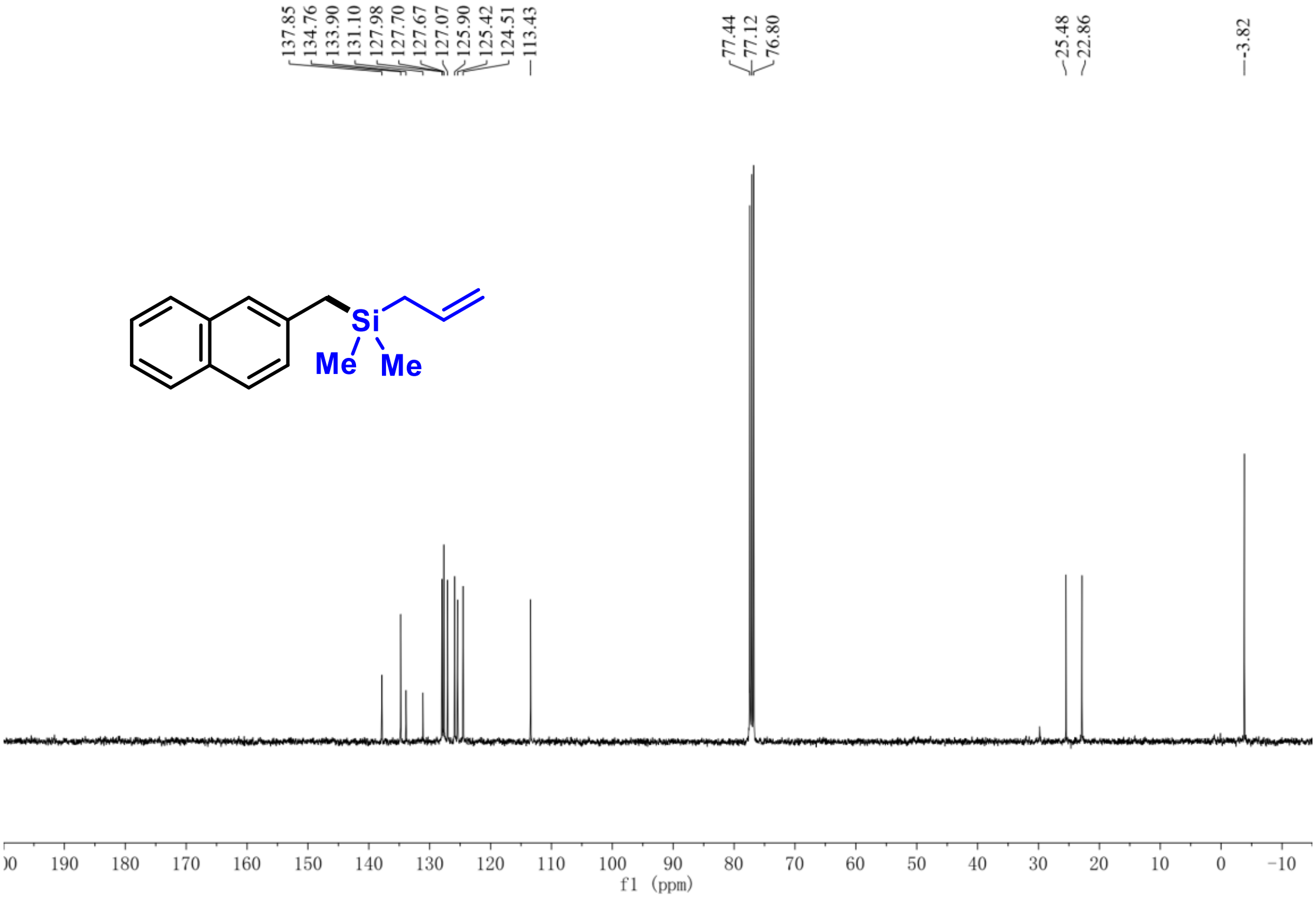

^{1}H NMR spectrum of 3aab (400 MHz, $CDCl_3$)

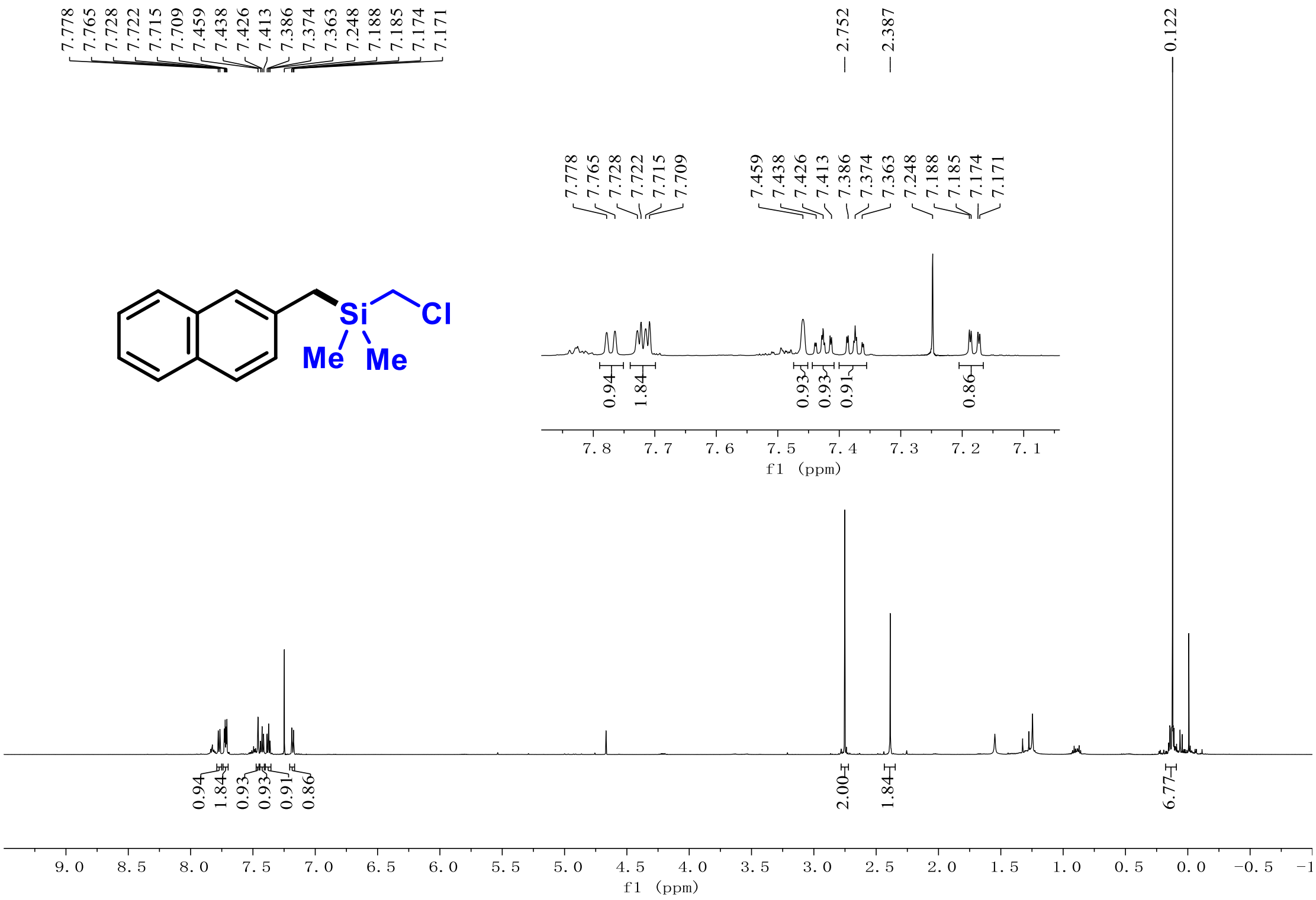

^{13}C NMR spectrum of 3aab (101 MHz, $CDCl_3$)

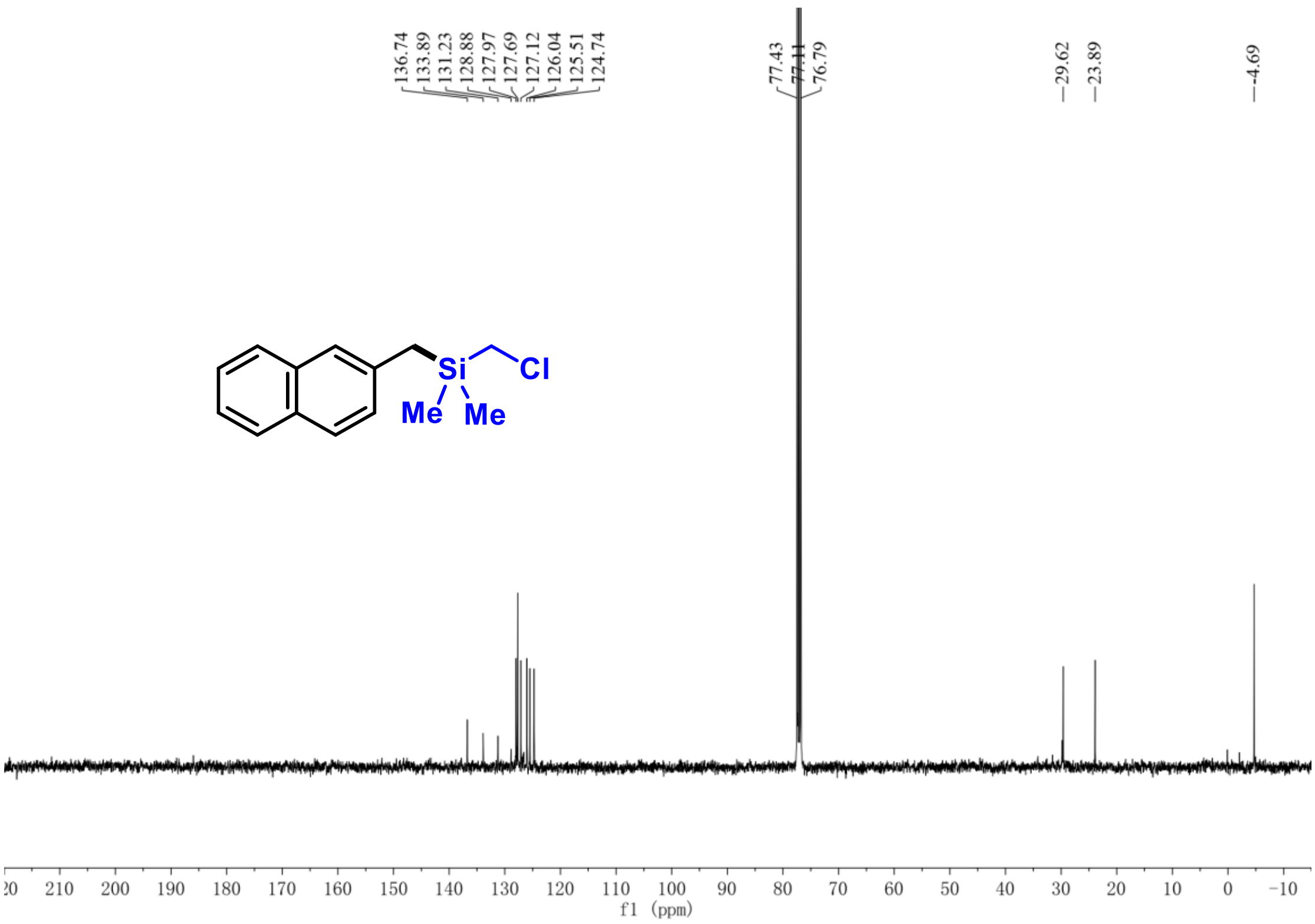

^{1}H NMR spectrum of 3aae (400 MHz, $CDCl_3$)

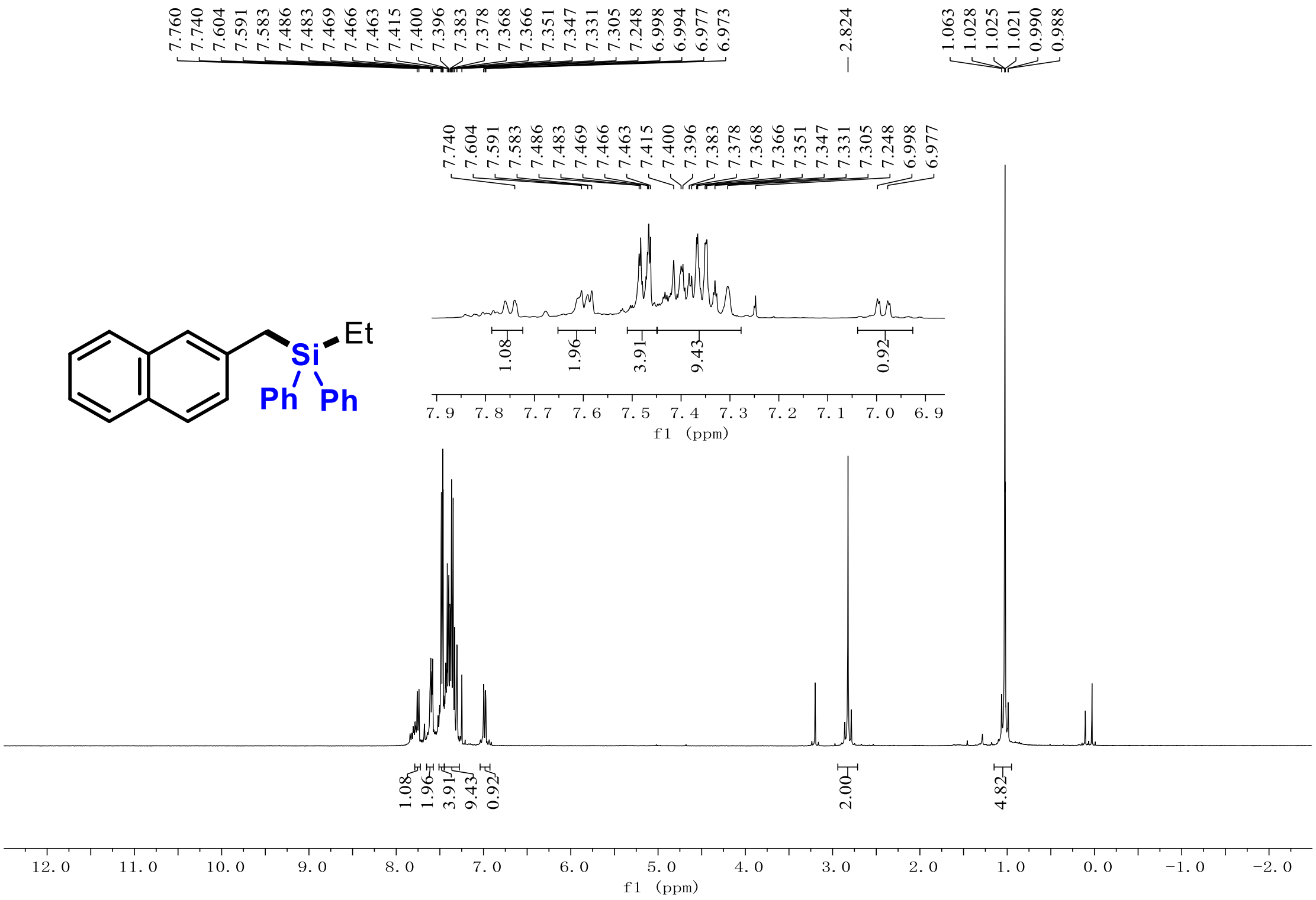

^{13}C NMR spectrum of 3aae (101 MHz, $CDCl_3$)

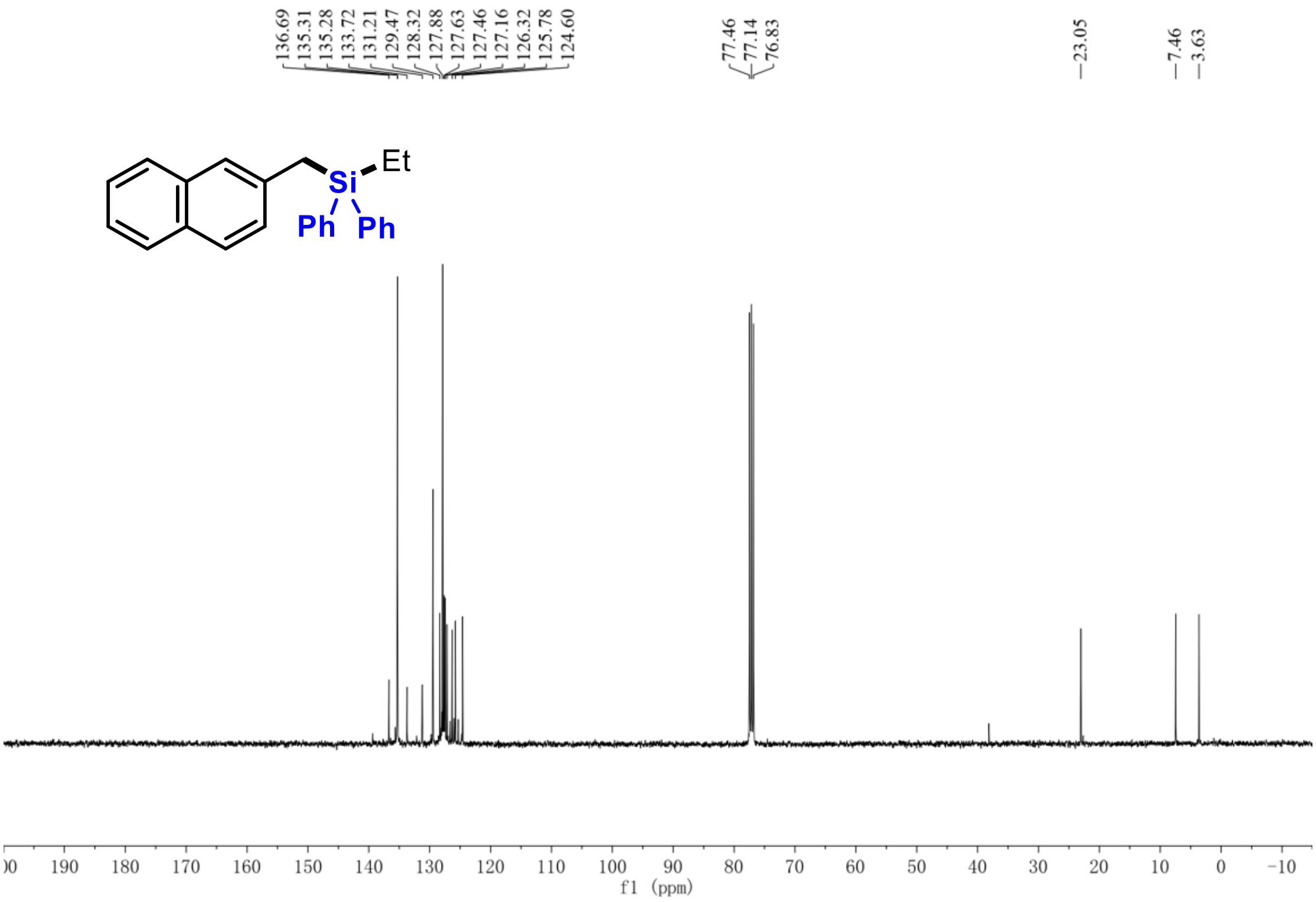

^{1}H NMR spectrum of 3aaf (400 MHz, $CDCl_3$)

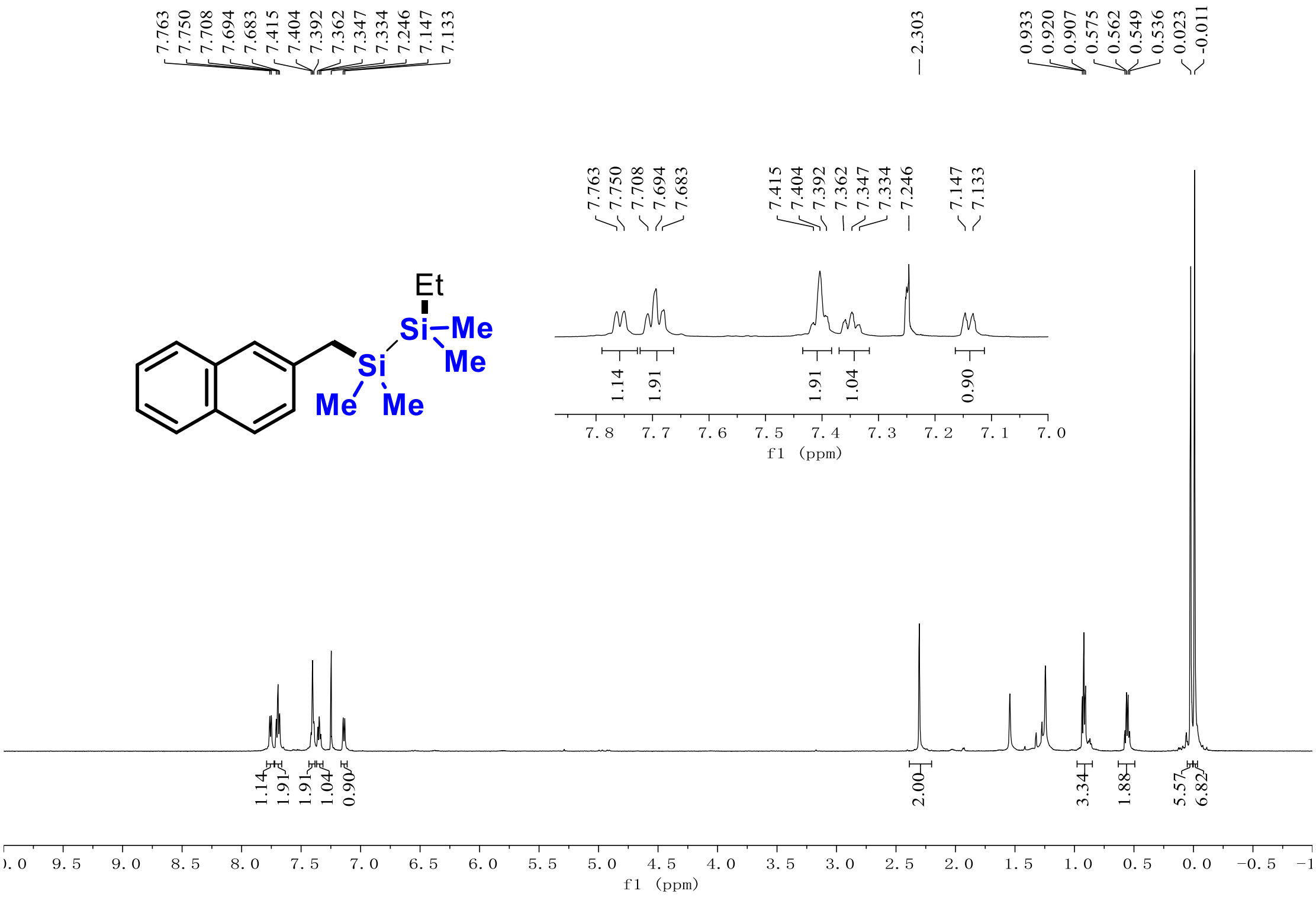

^{13}C NMR spectrum of 3aaf (101 MHz, $CDCl_3$)

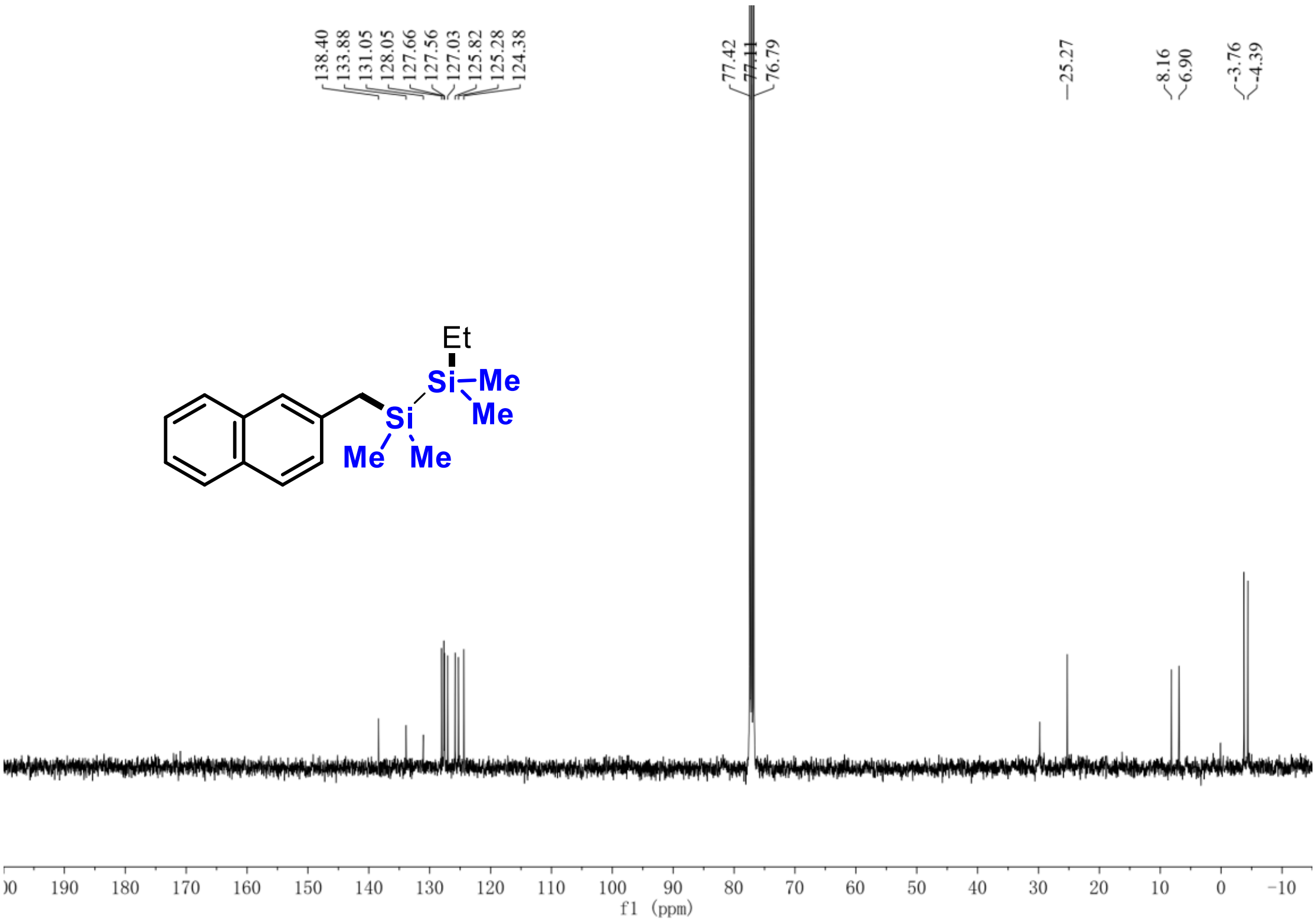

^{1}H NMR spectrum of 3aag (400 MHz, $CDCl_3$)

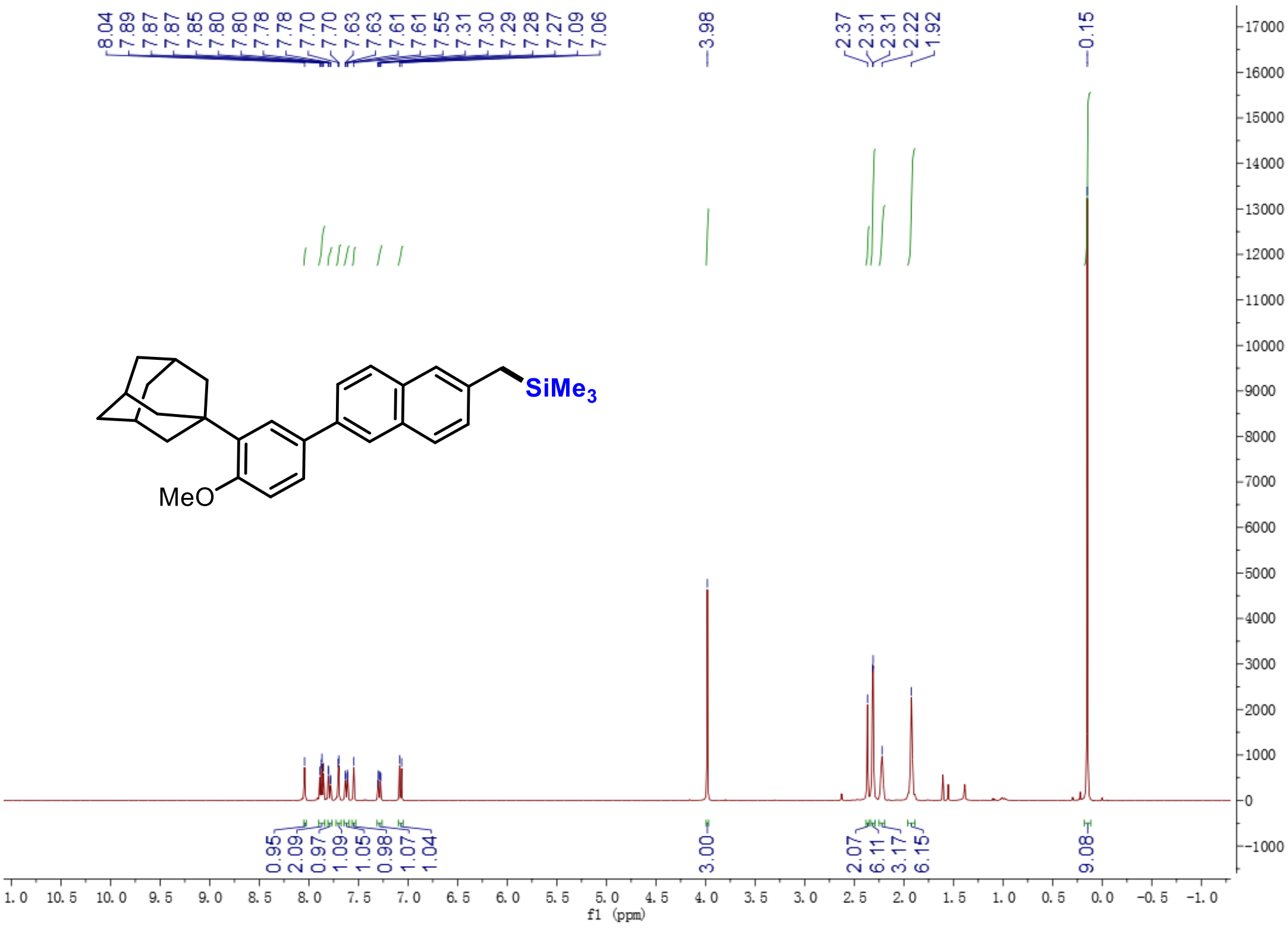

^{13}C NMR spectrum of 3aag (101 MHz, $CDCl_3$)

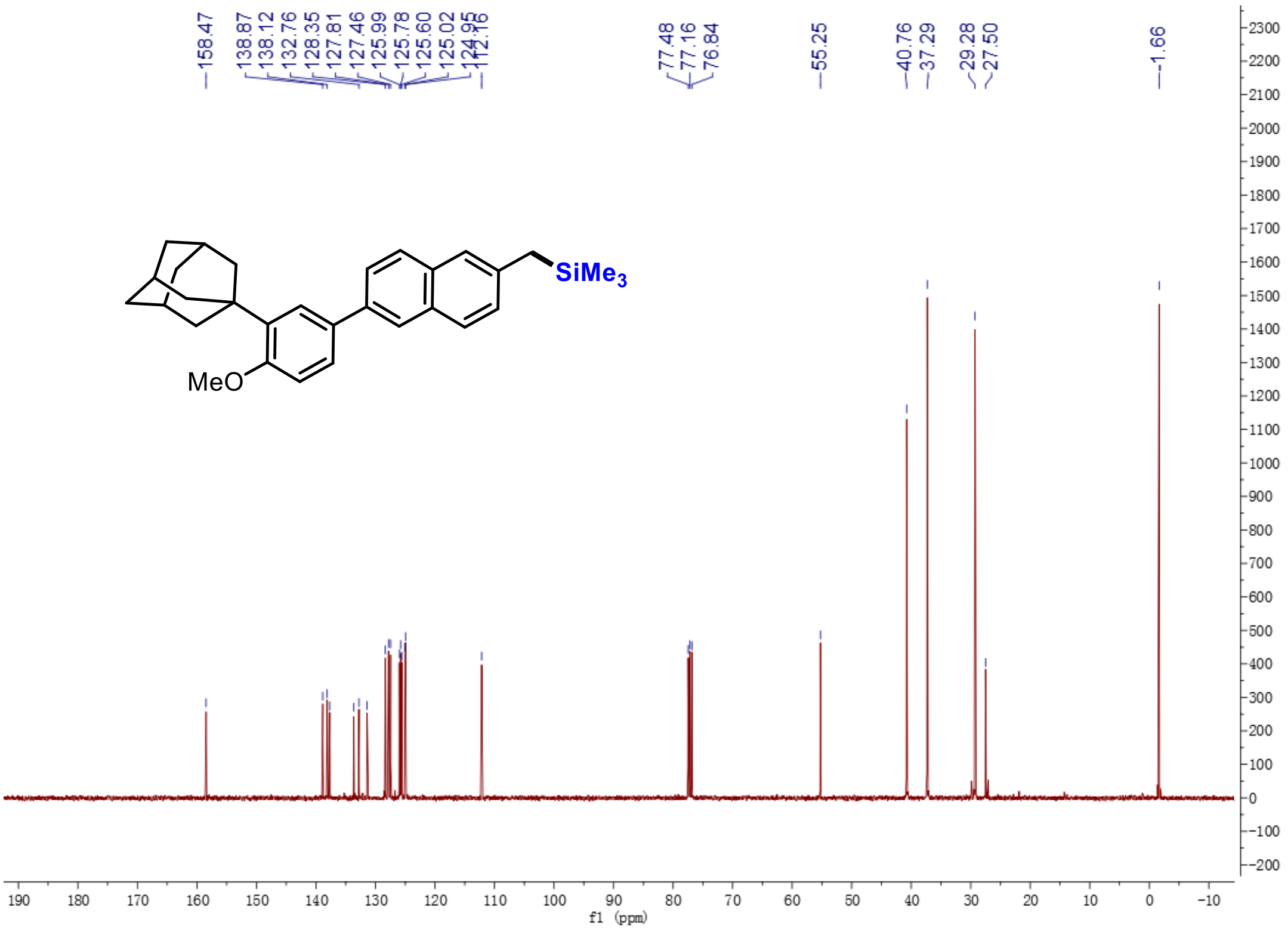

^{1}H NMR spectrum of 3aah (400 MHz, $CDCl_3$)

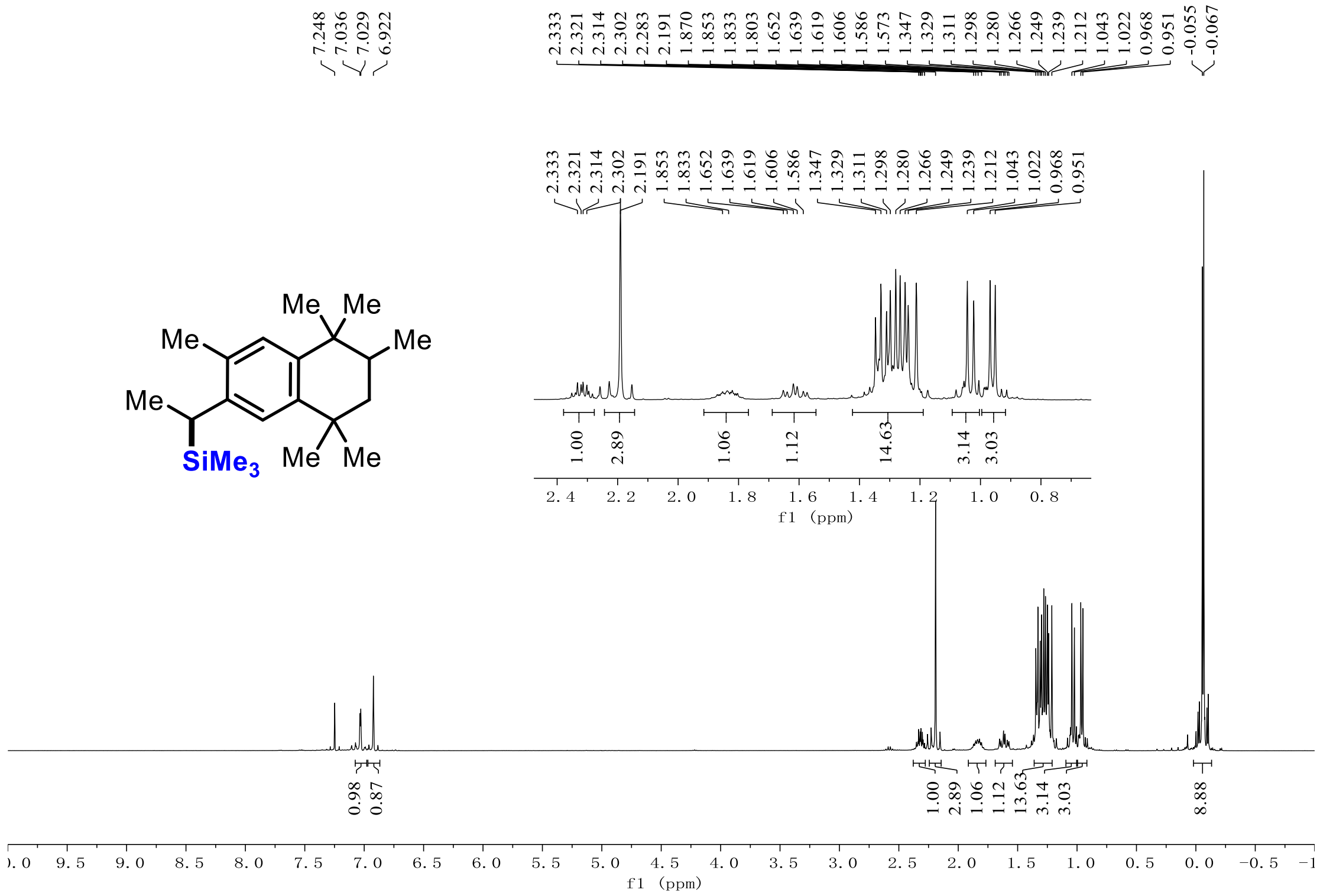

^{13}C NMR spectrum of 3aah (101 MHz, $CDCl_3$)

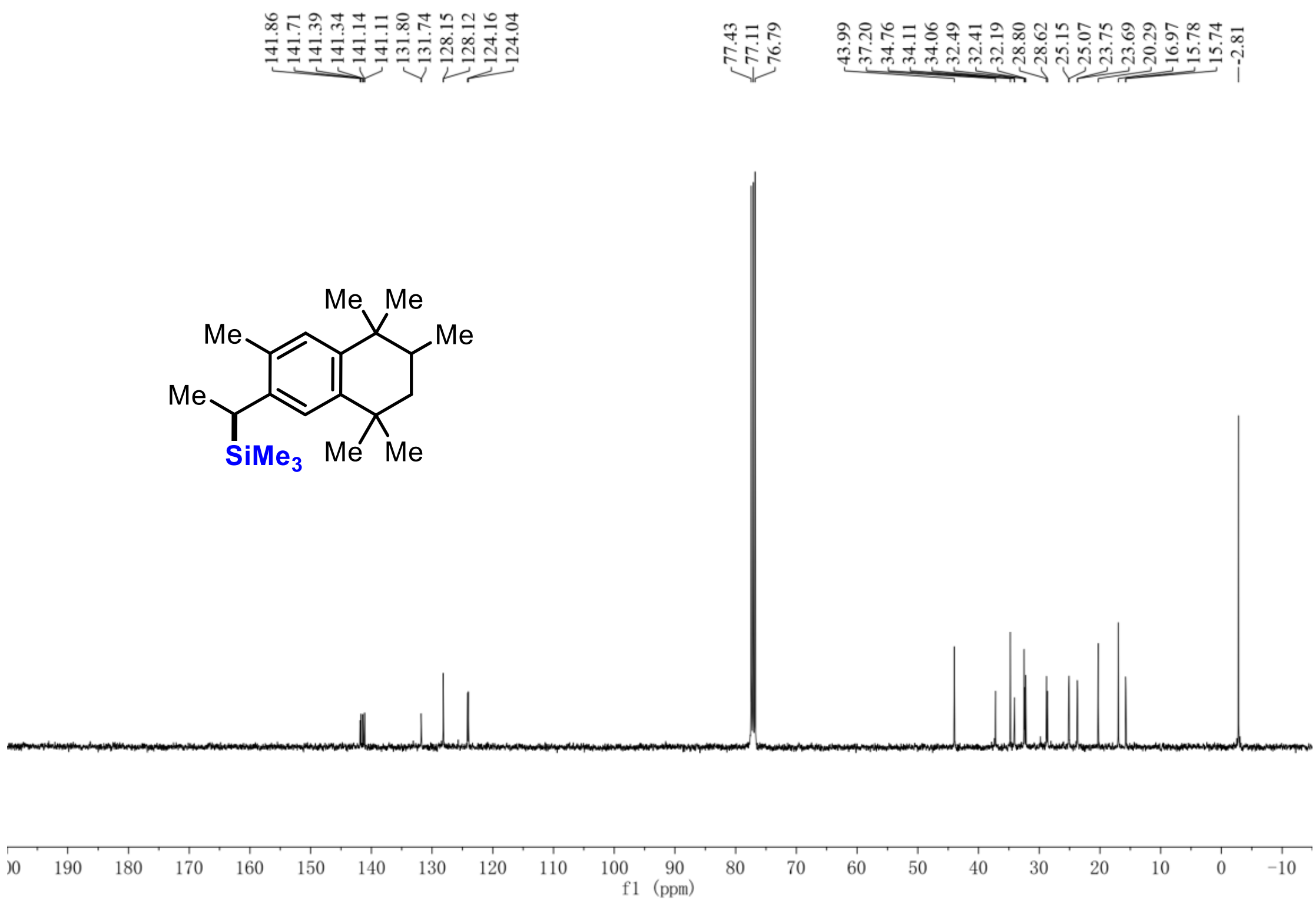

^{1}H NMR spectrum of 3aai (400 MHz, $CDCl_3$)

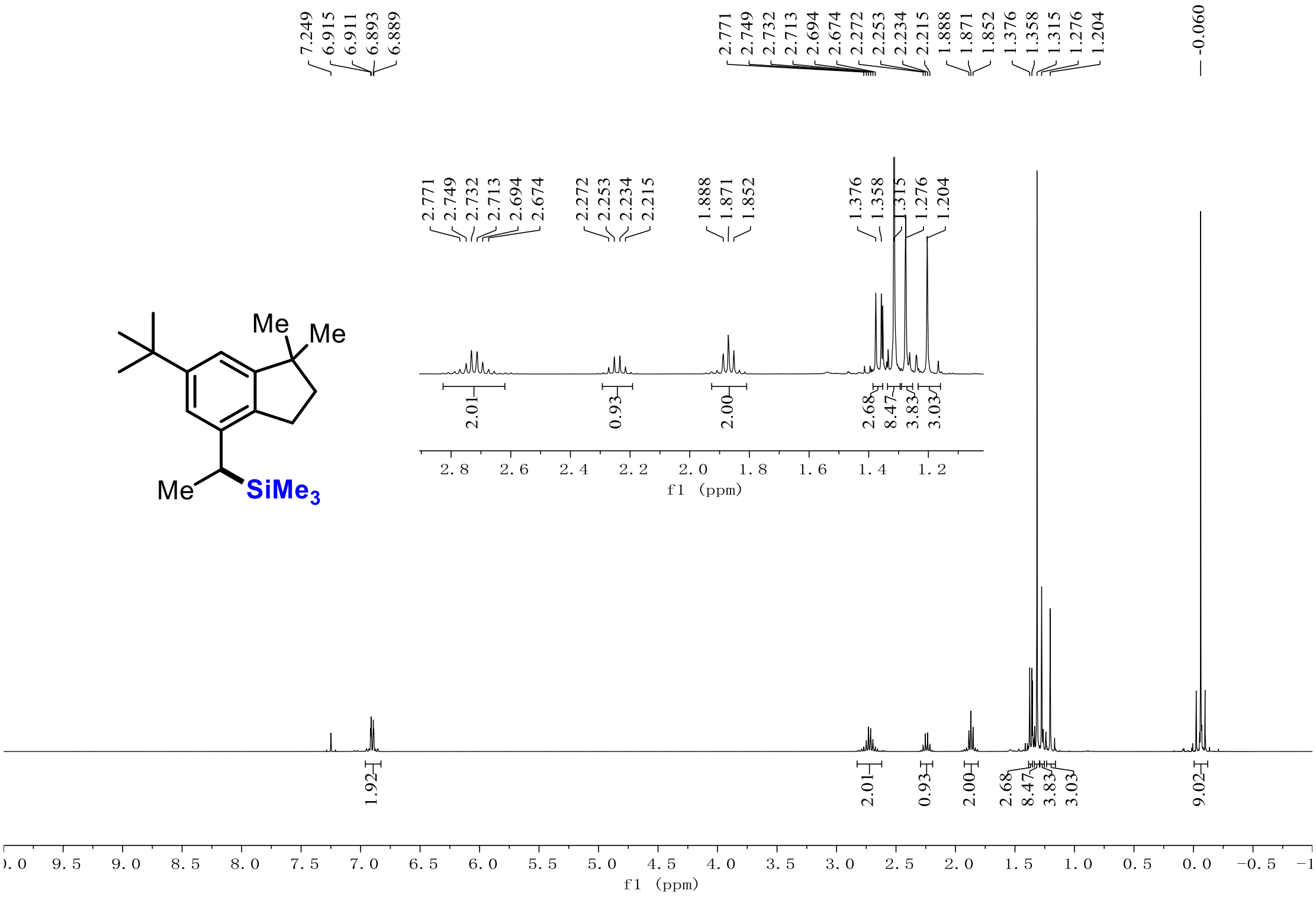

^{13}C NMR spectrum of 3aai (101 MHz, $CDCl_3$)

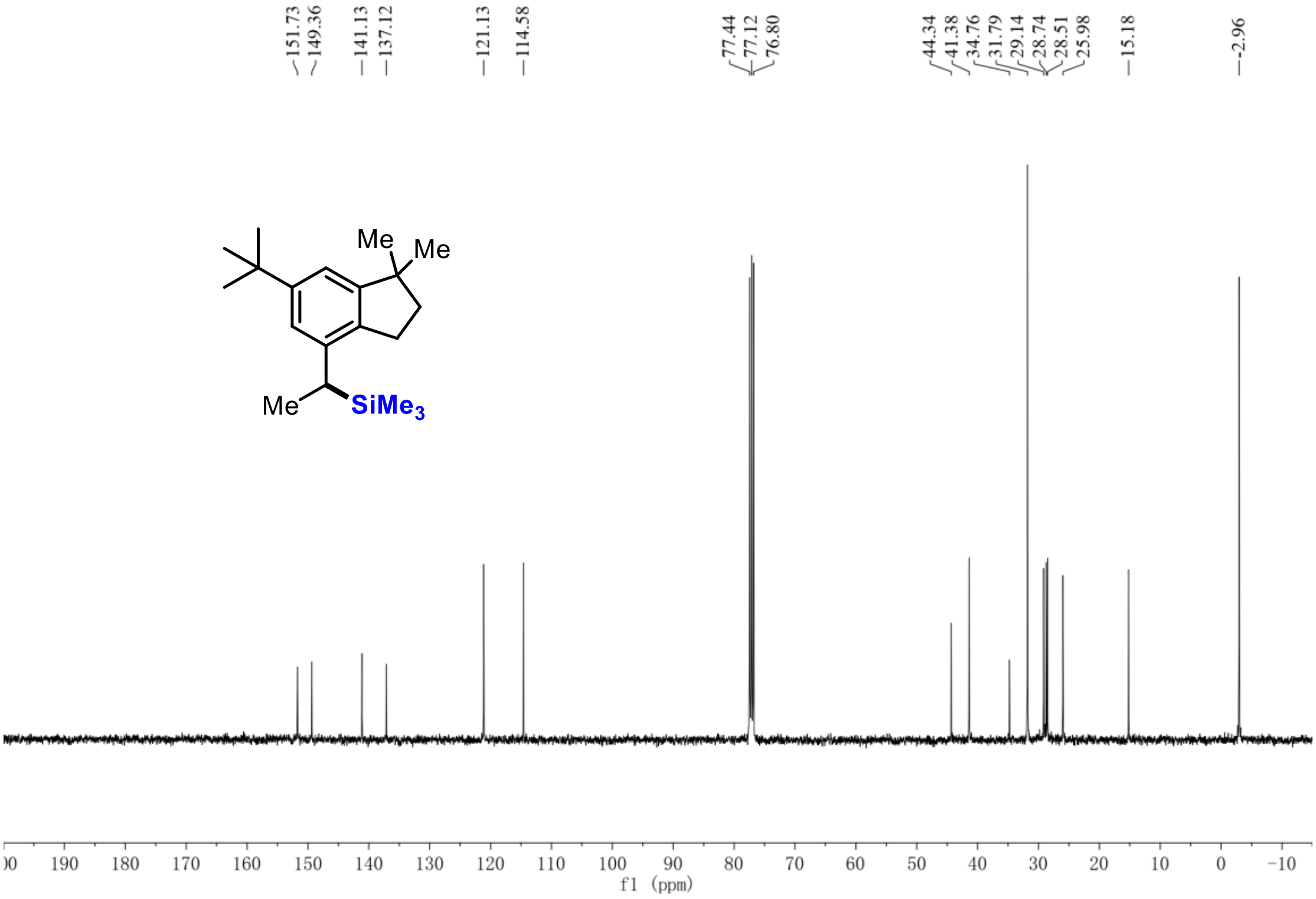

^{1}H NMR spectrum of 3aaj (400 MHz, $CDCl_3$)

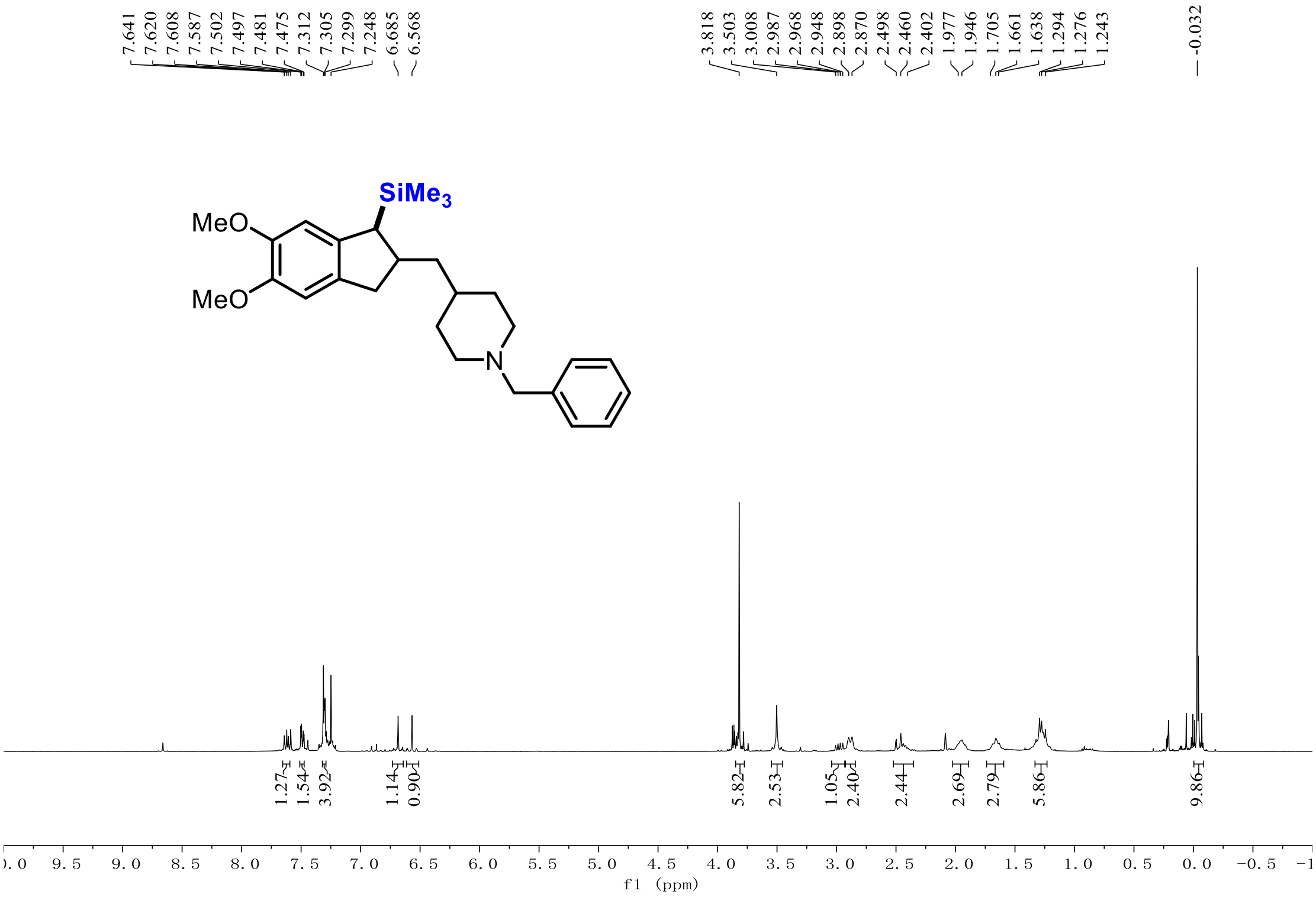

^{13}C NMR spectrum of 3aaj (101 MHz, $CDCl_3$)

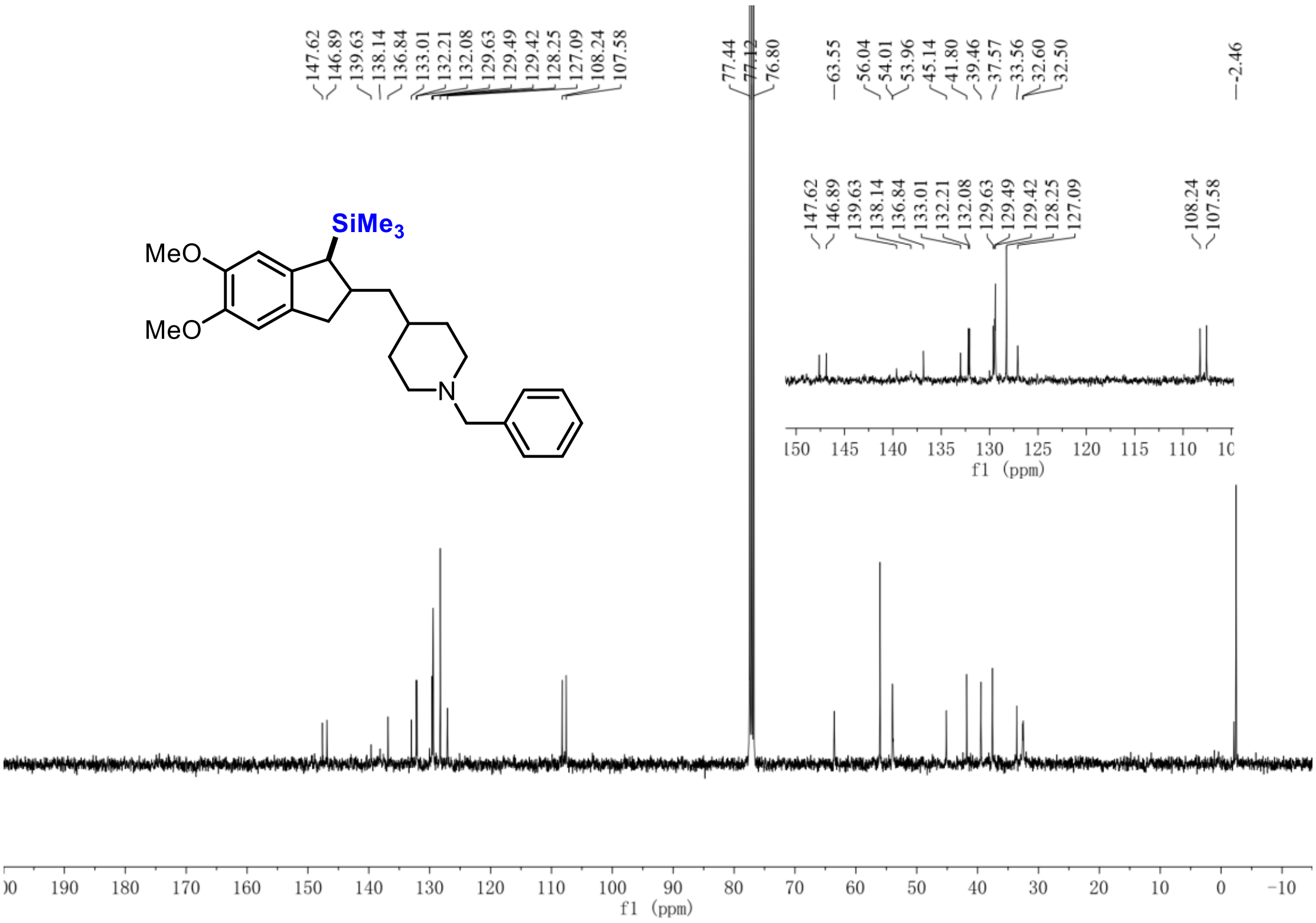

^{1}H NMR spectrum of 3aak (400 MHz, $CDCl_3$)

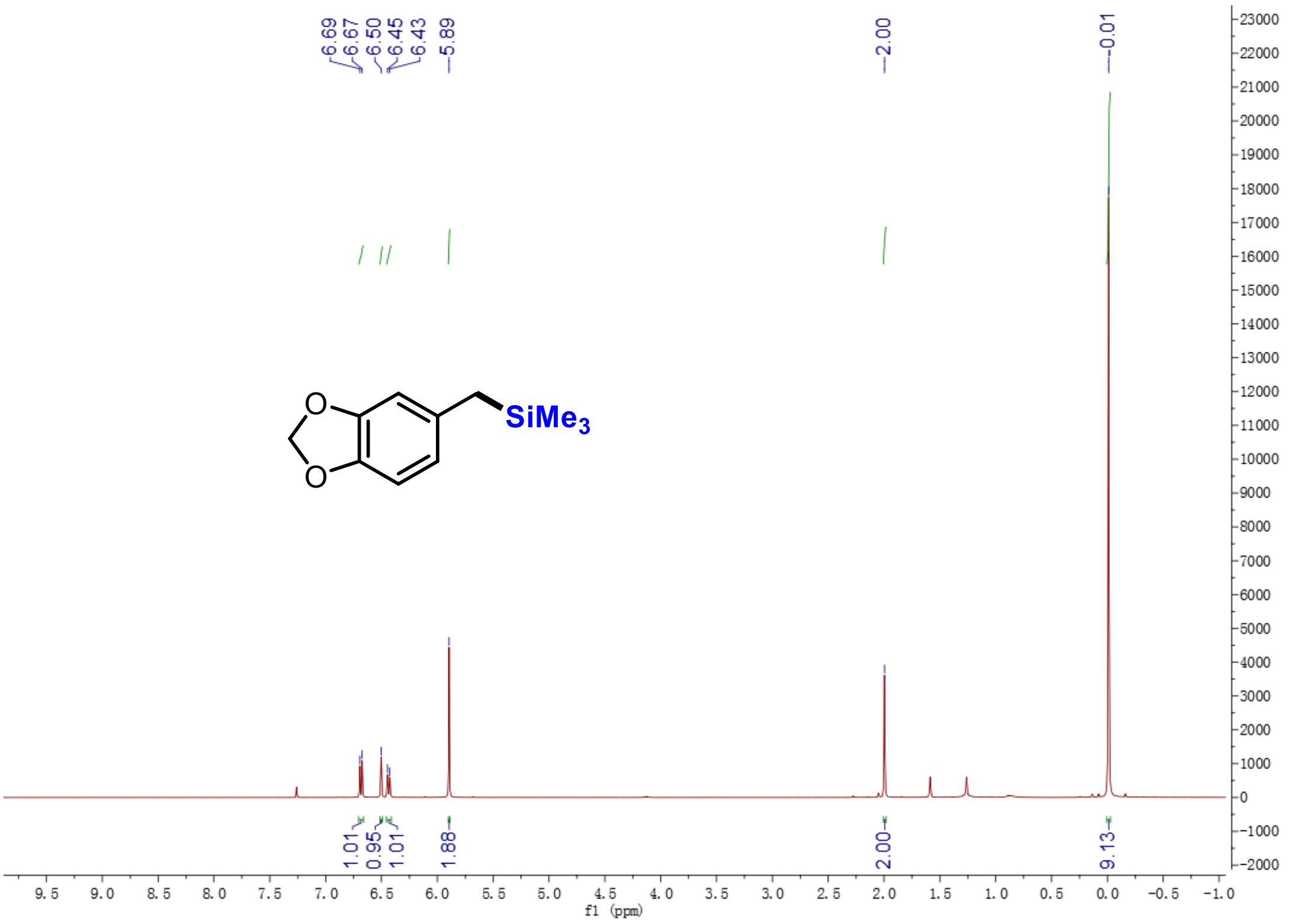

^{13}C NMR spectrum of 3aak (101 MHz, $CDCl_3$)

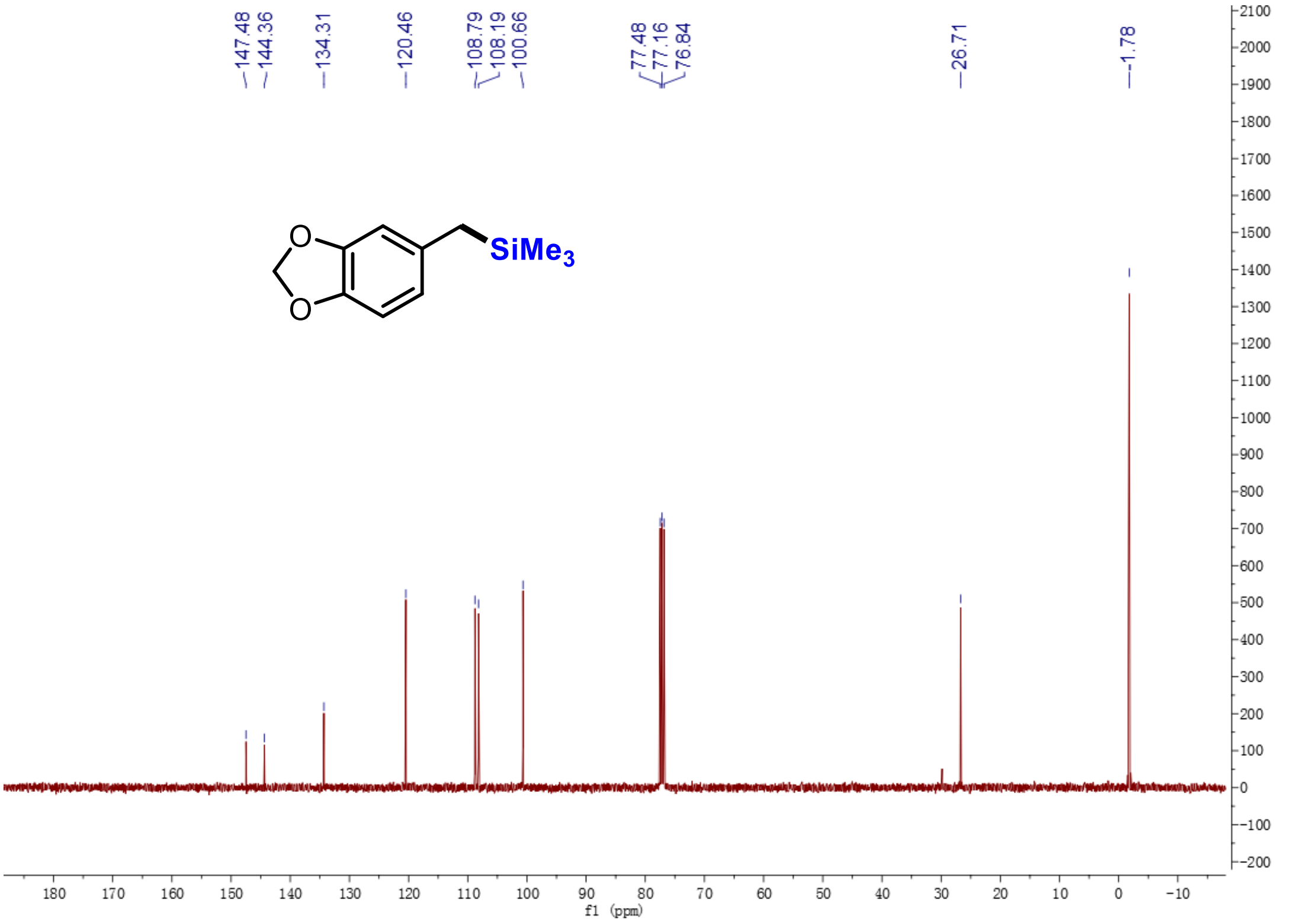

^{1}H NMR spectrum of 3aal (400 MHz, $CDCl_3$)

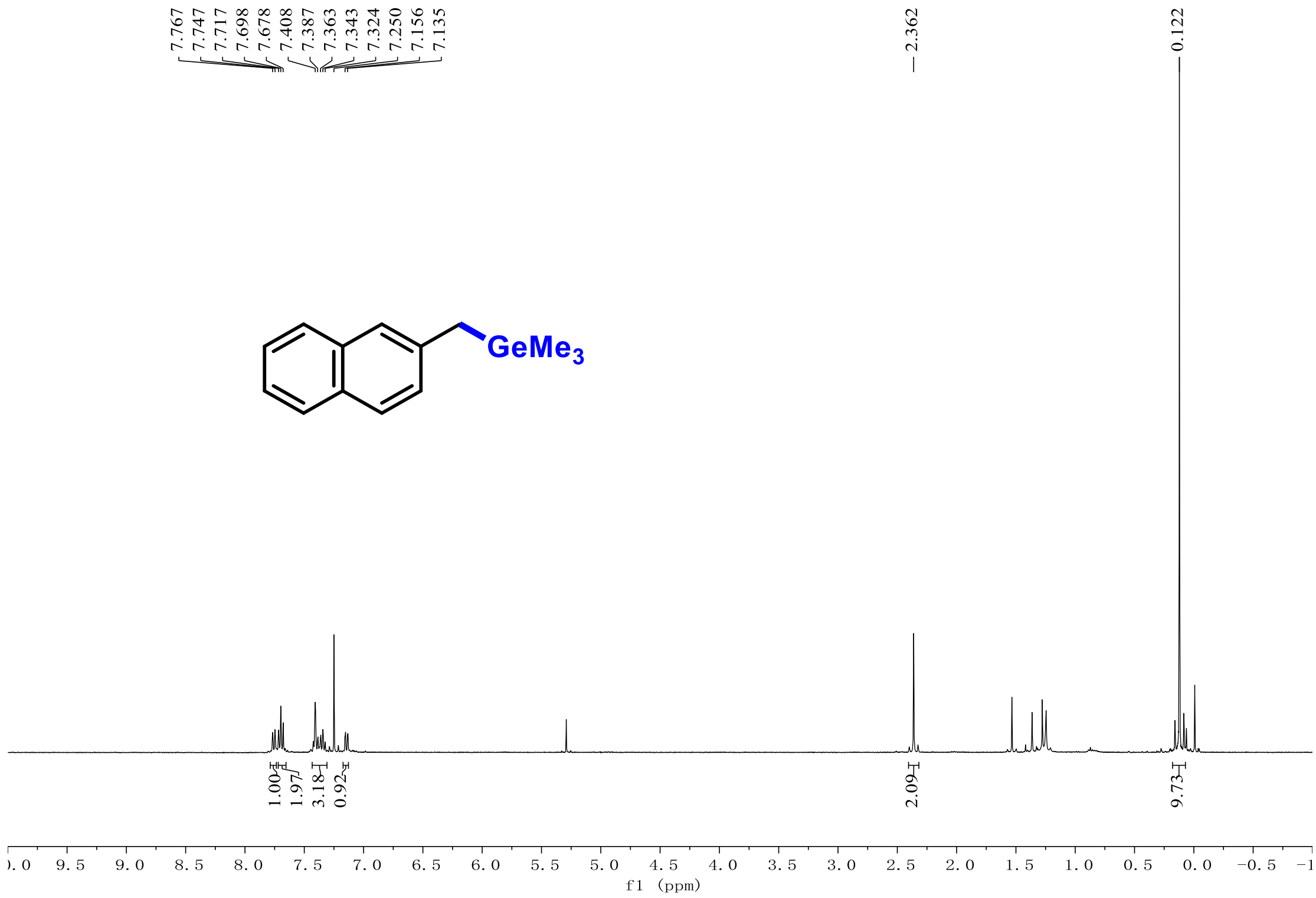

^{13}C NMR spectrum of 3aal (153 MHz, $CDCl_3$)

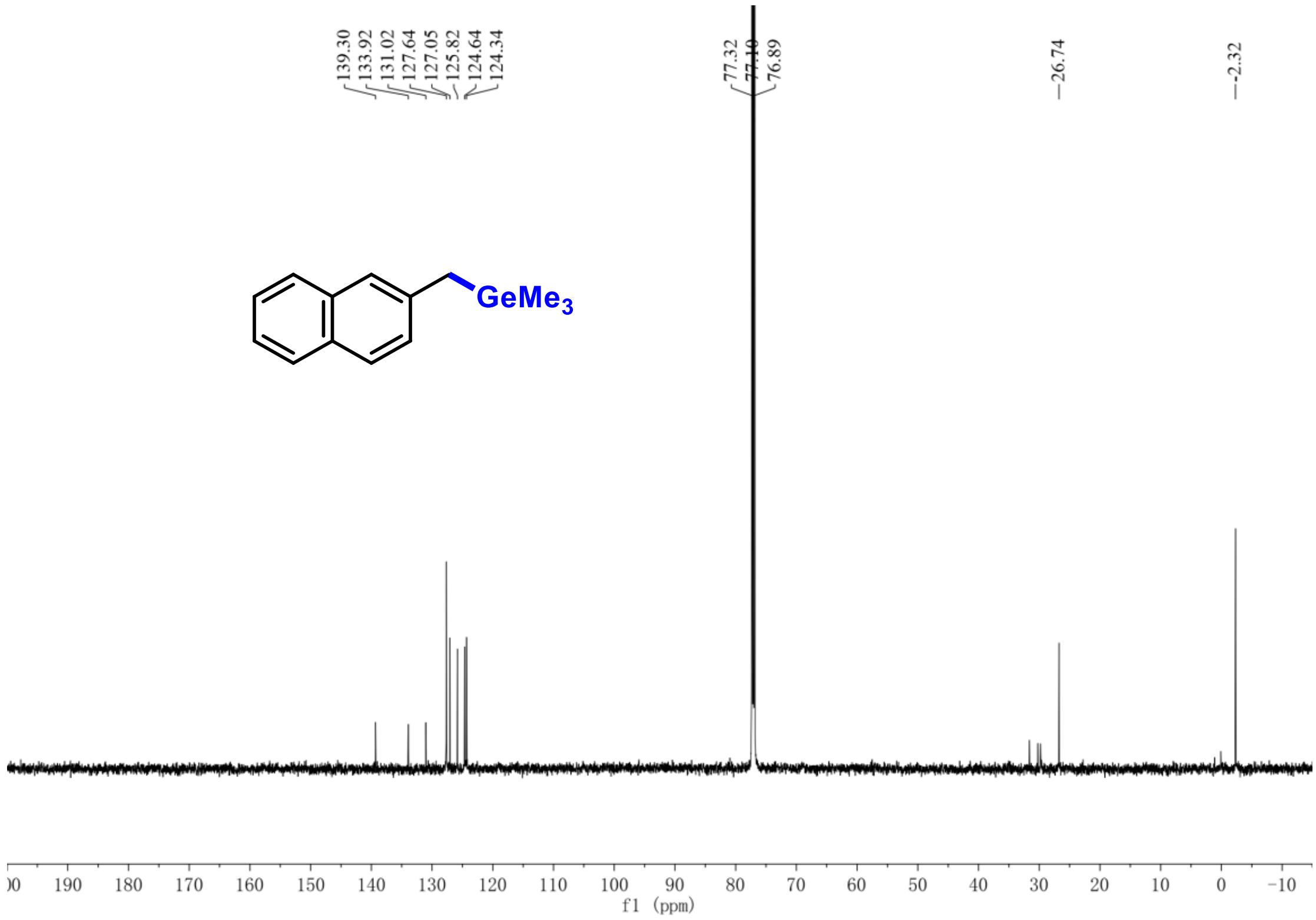

^{1}H NMR spectrum of 3aam (400 MHz, $CDCl_3$)

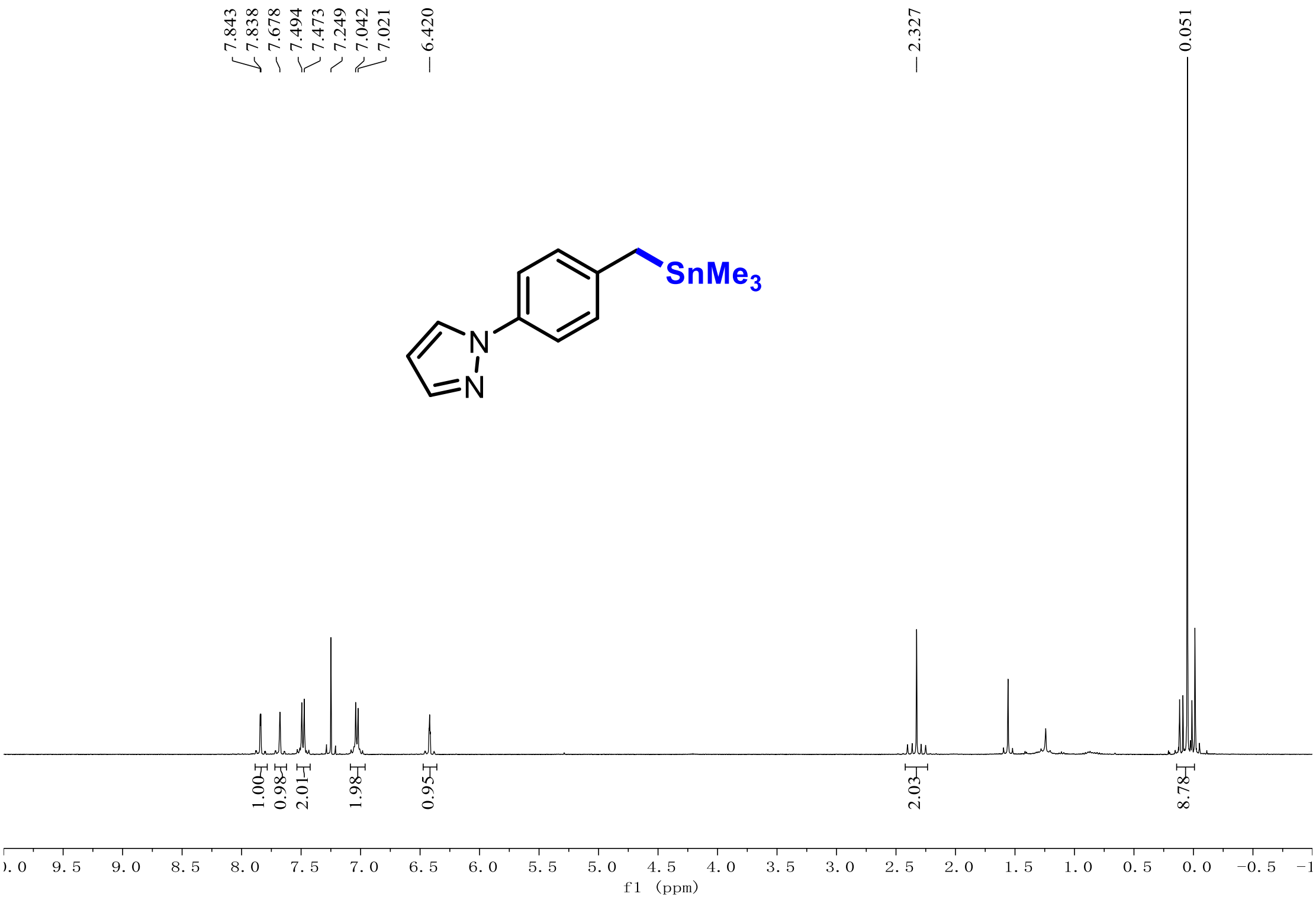

^{13}C NMR spectrum of 3aam (153 MHz, $CDCl_3$)

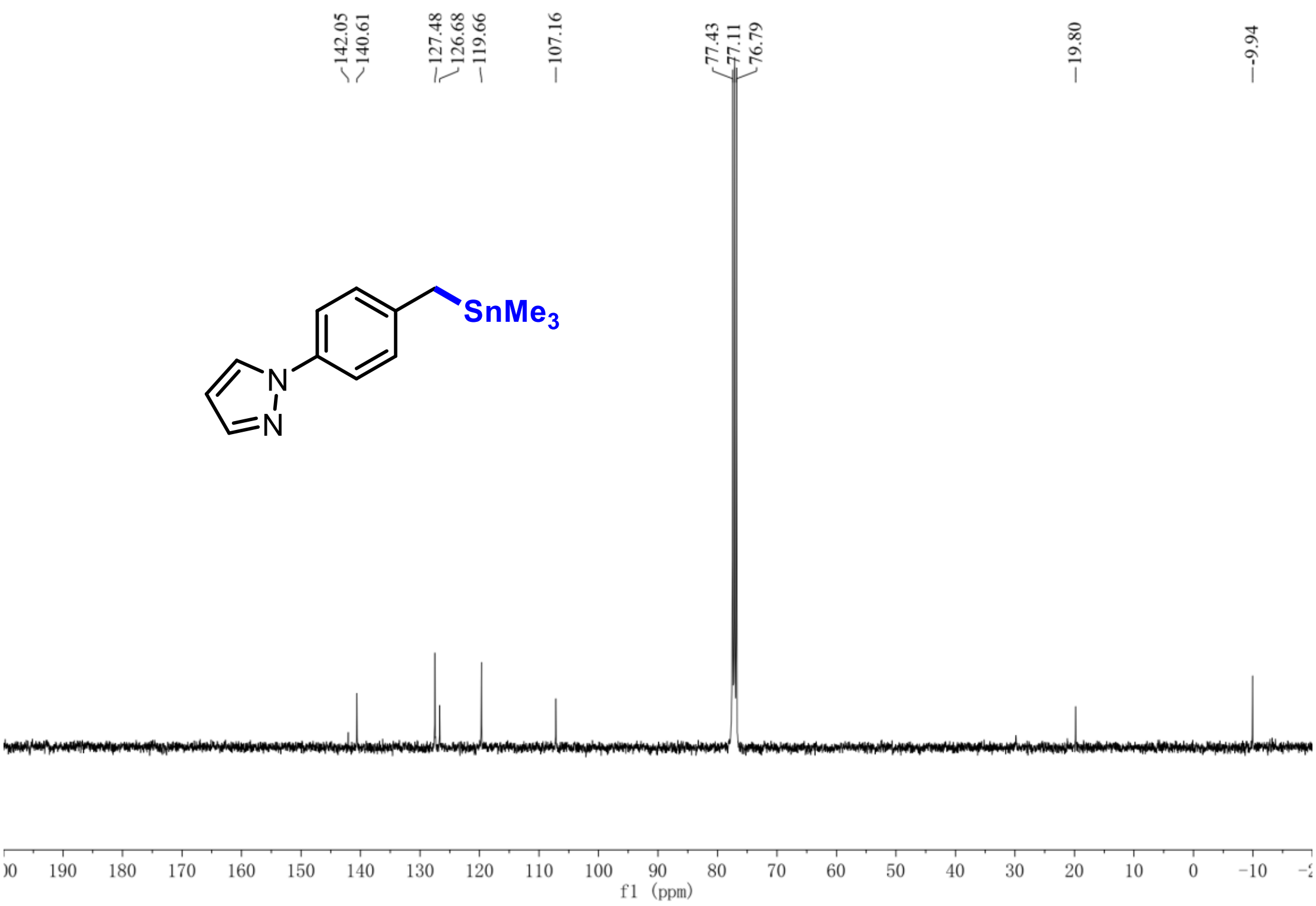

^{1}H NMR spectrum of 3aan (400 MHz, $CDCl_3$)

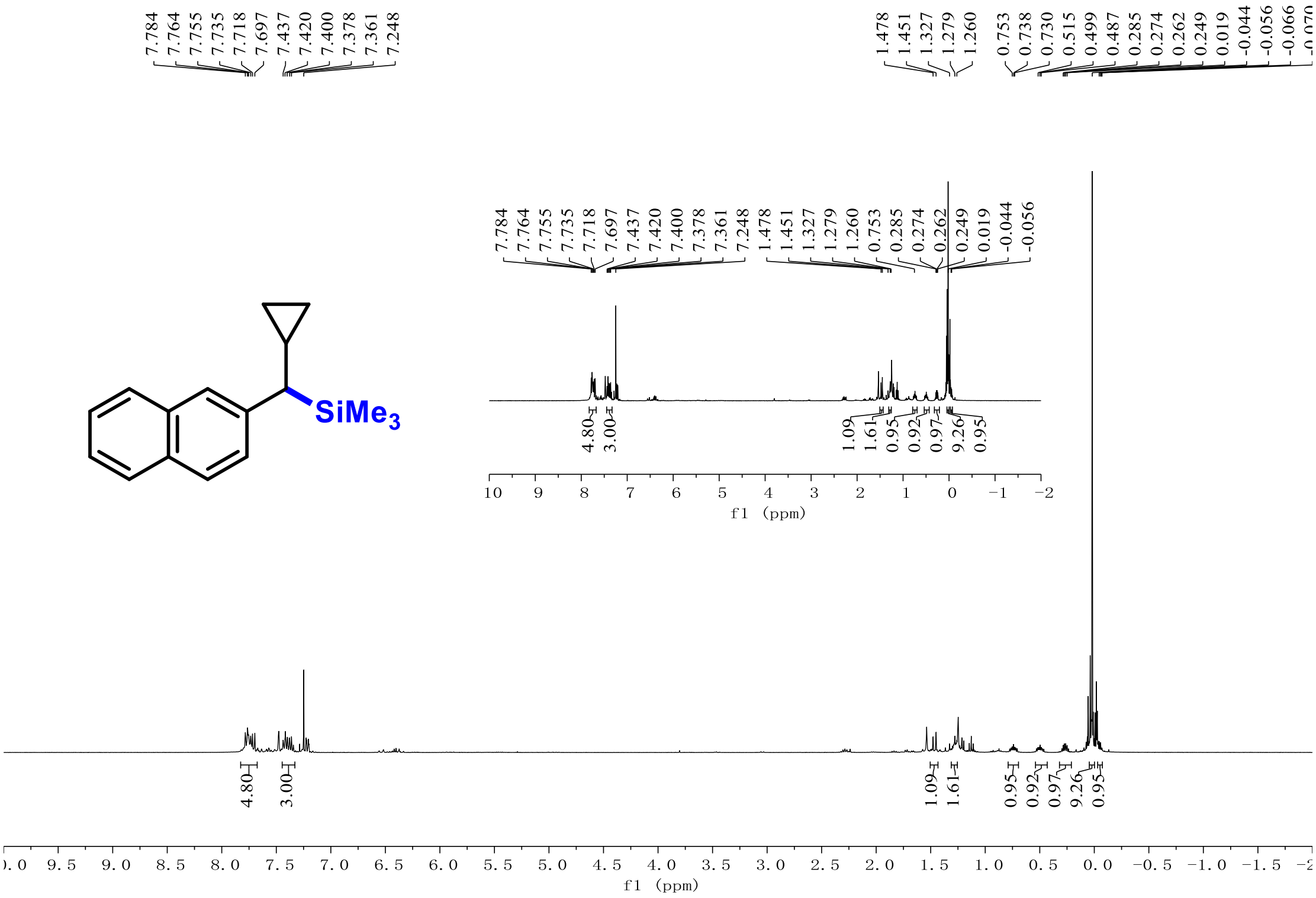

^{13}C NMR spectrum of 3aan (101 MHz, $CDCl_3$)

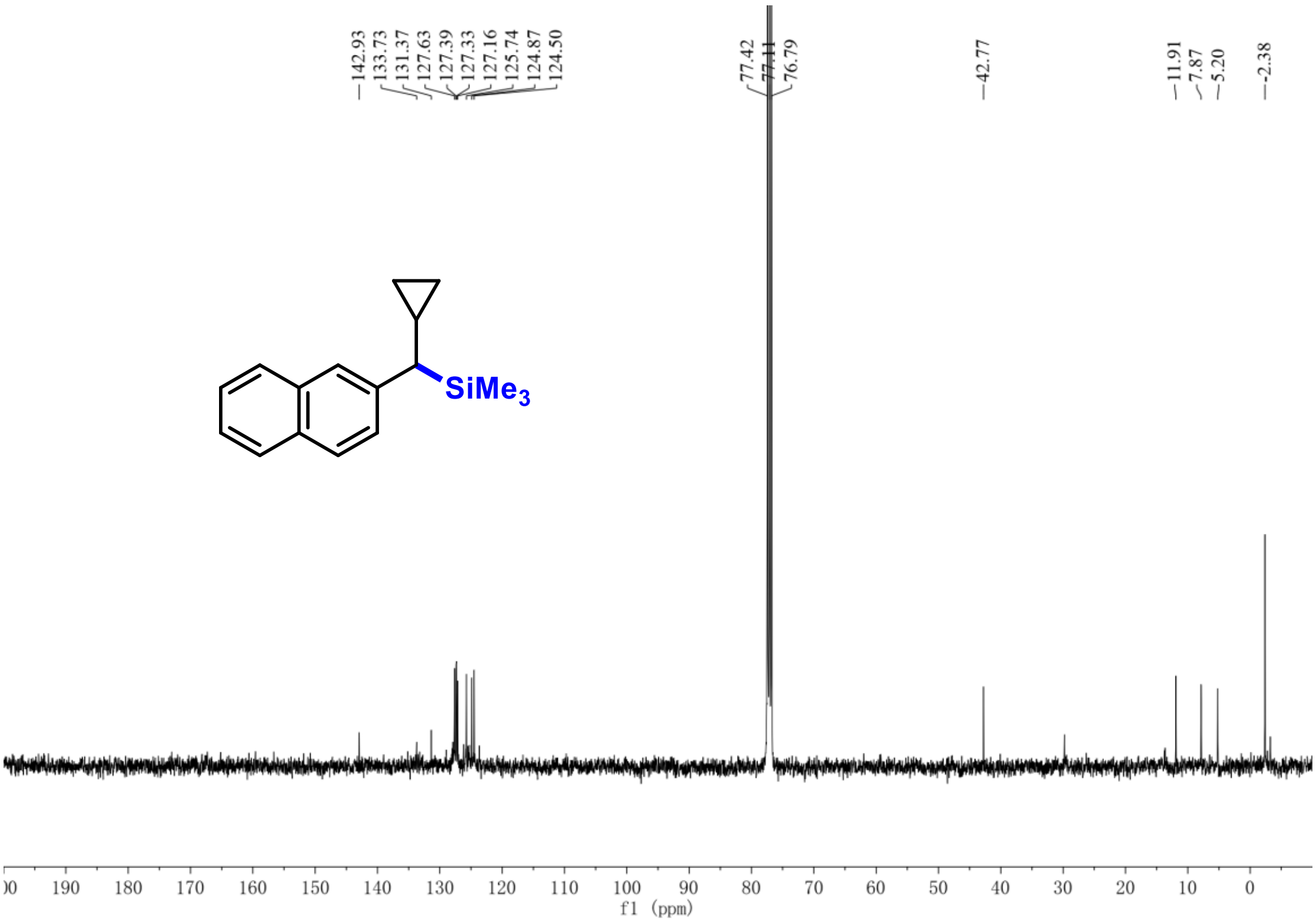

^{1}H NMR spectrum of 5a (400 MHz, $CDCl_3$)

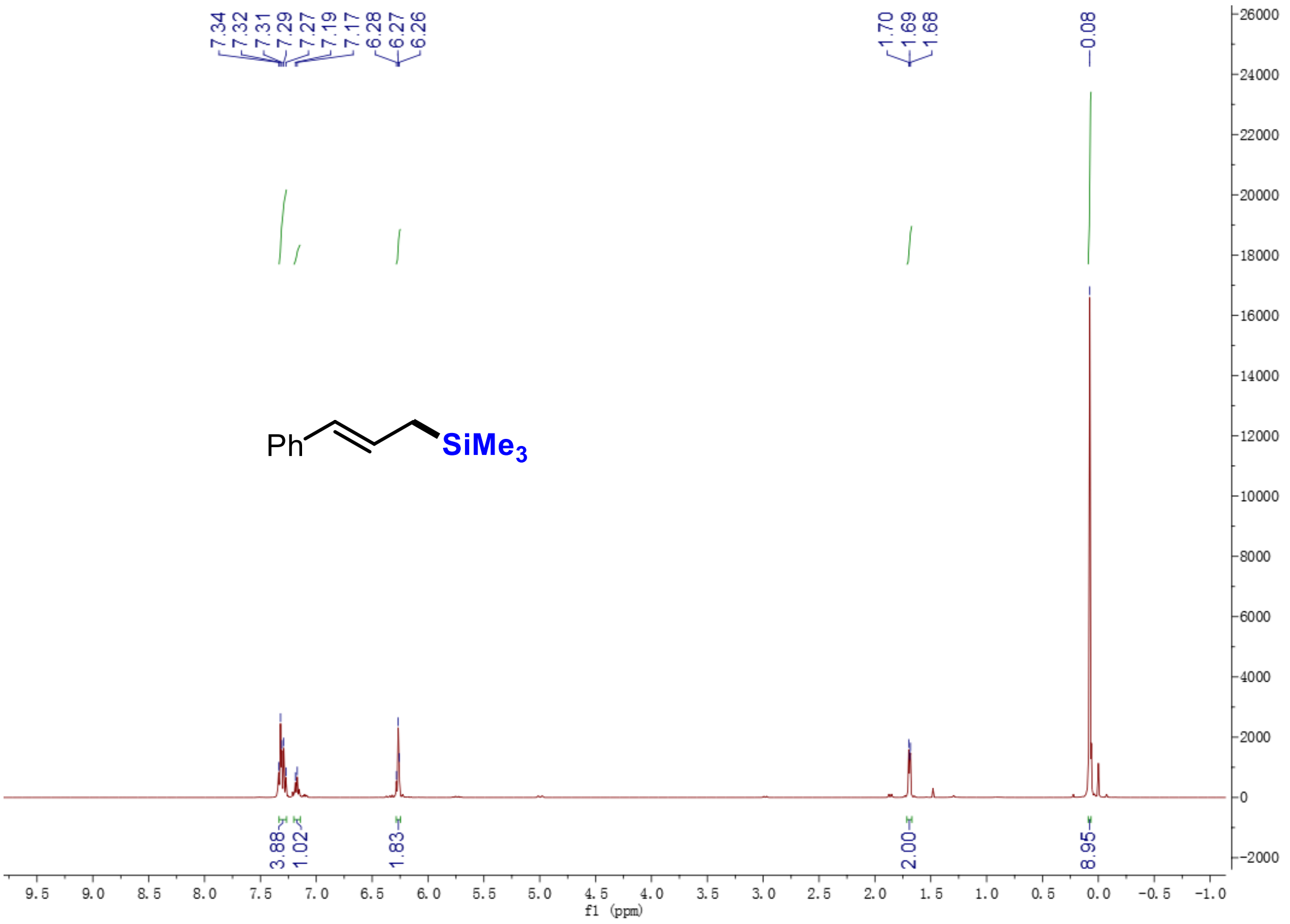

^{13}C NMR spectrum of 5a (101 MHz, $CDCl_3$)

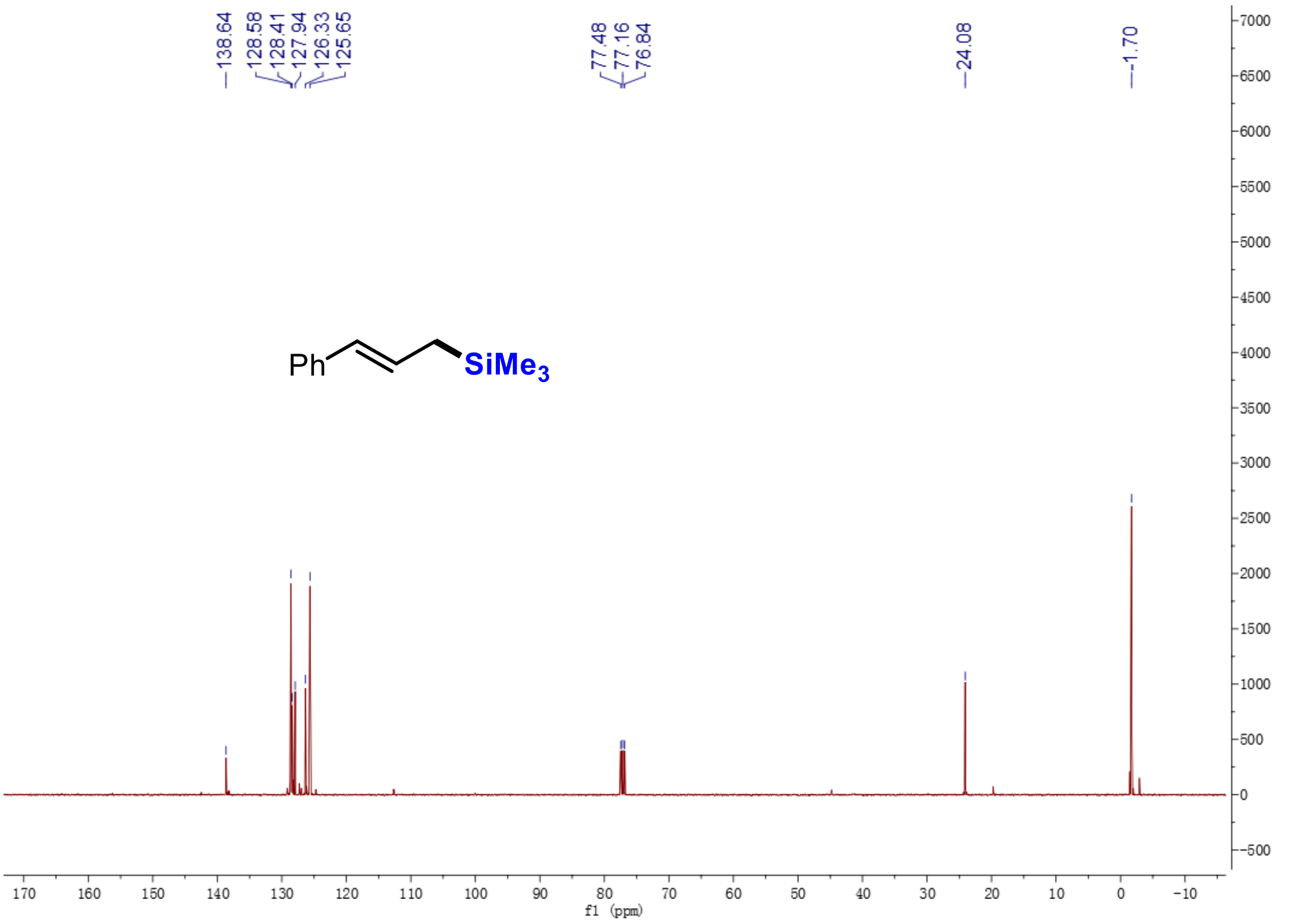

^{1}H NMR spectrum of 5b (400 MHz, $CDCl_3$)

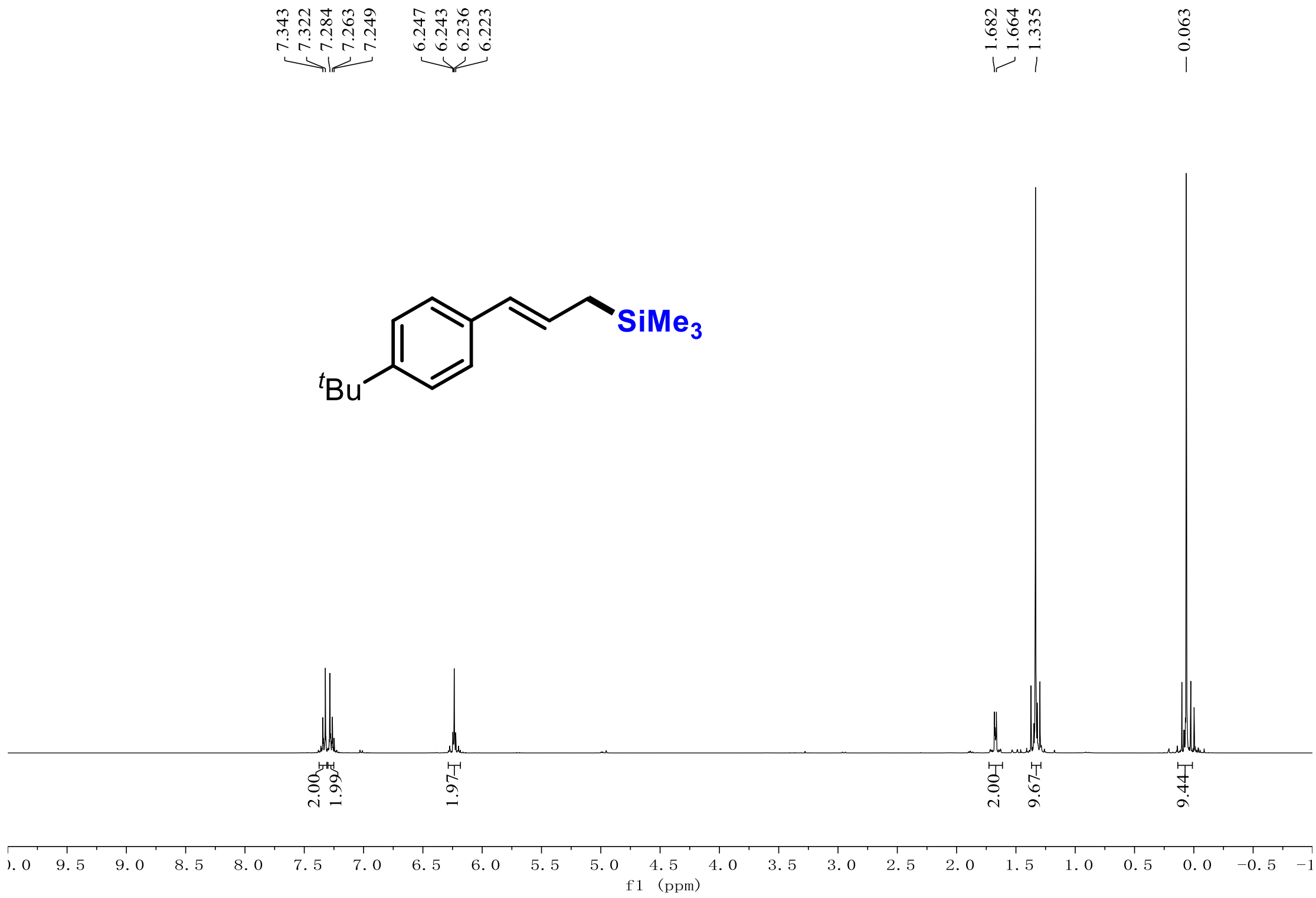

^{13}C NMR spectrum of 5b (101 MHz, $CDCl_3$)

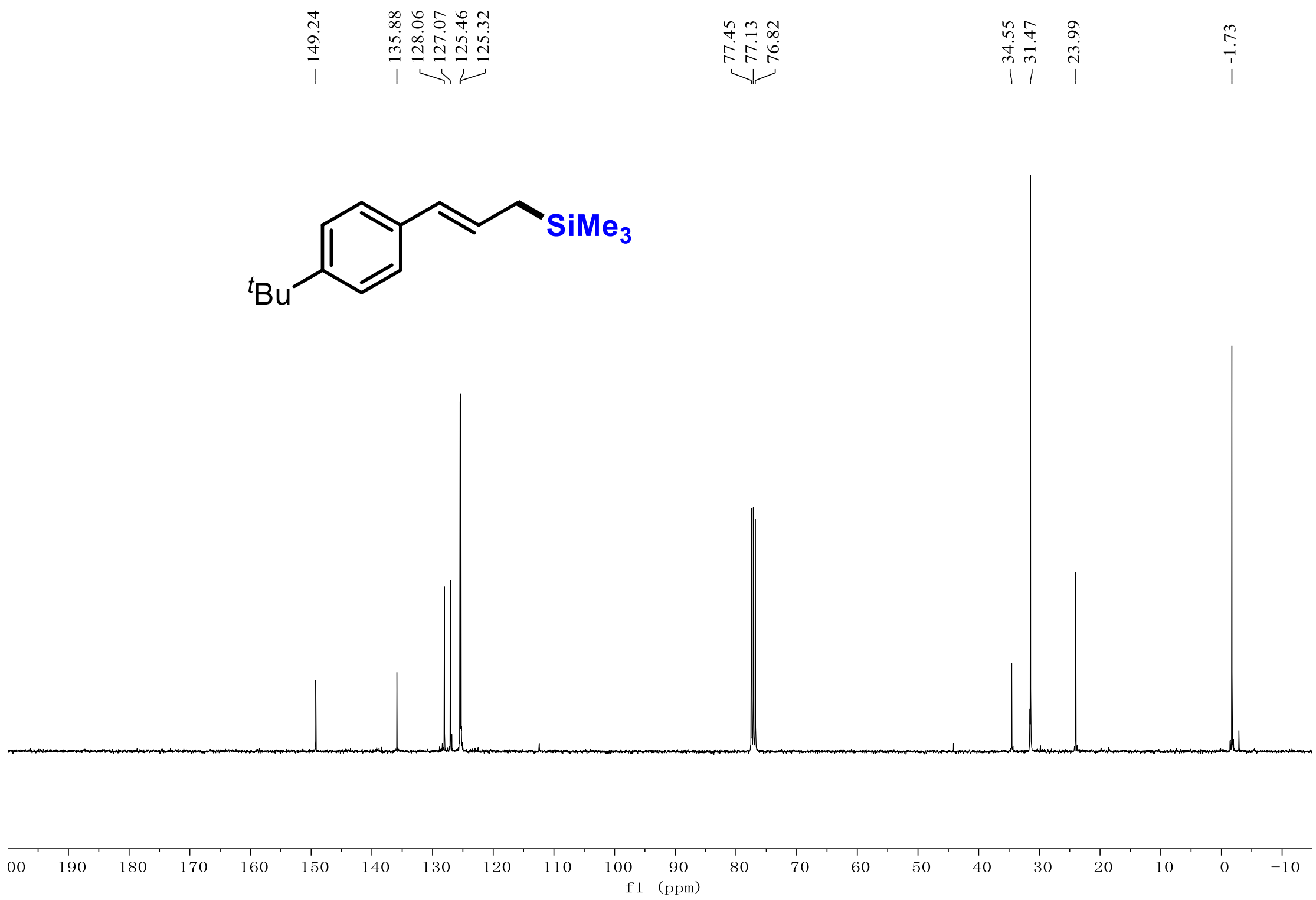

^{1}H NMR spectrum of 5c (400 MHz, $CDCl_3$)

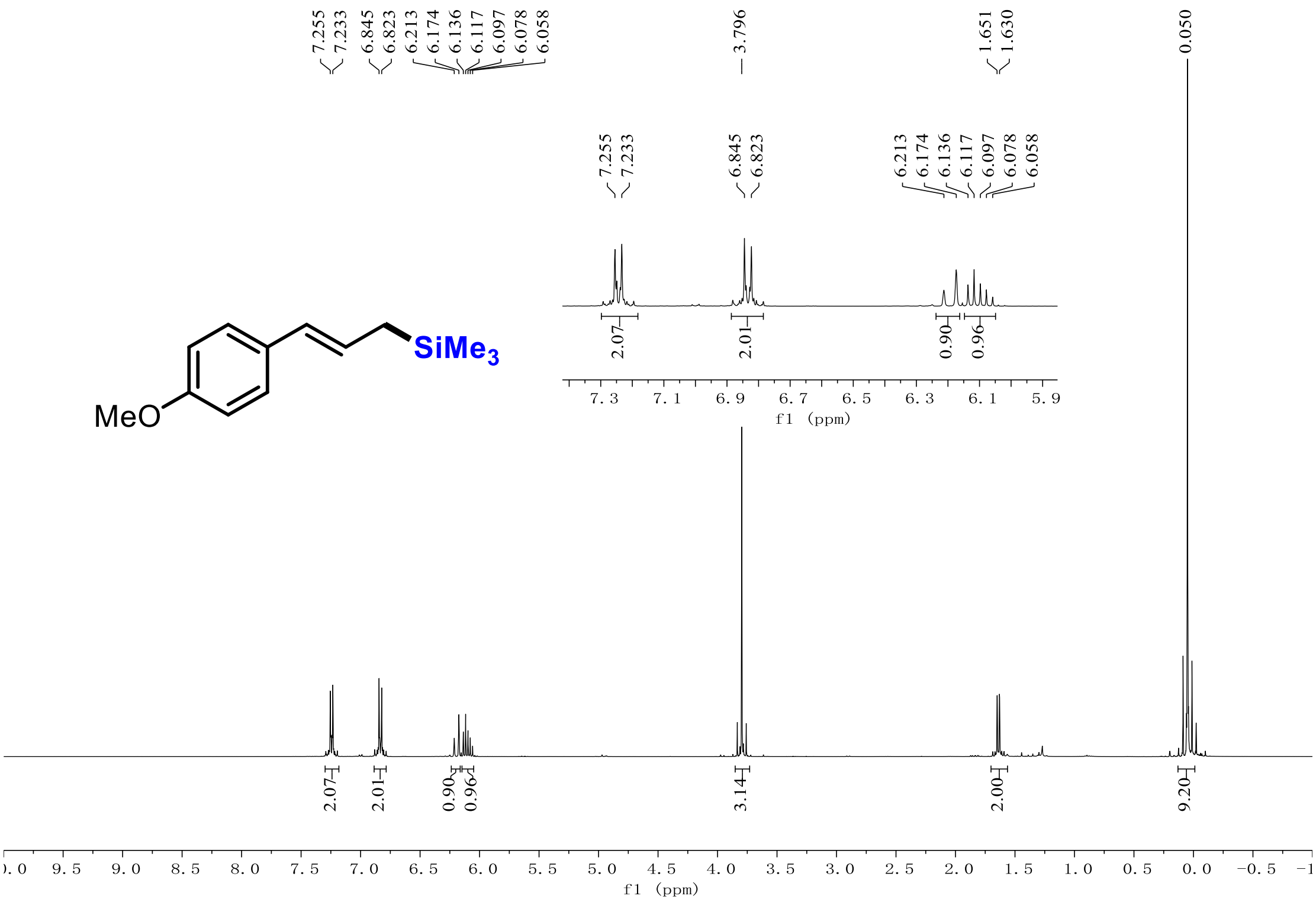

^{13}C NMR spectrum of 5c (101 MHz, $CDCl_3$)

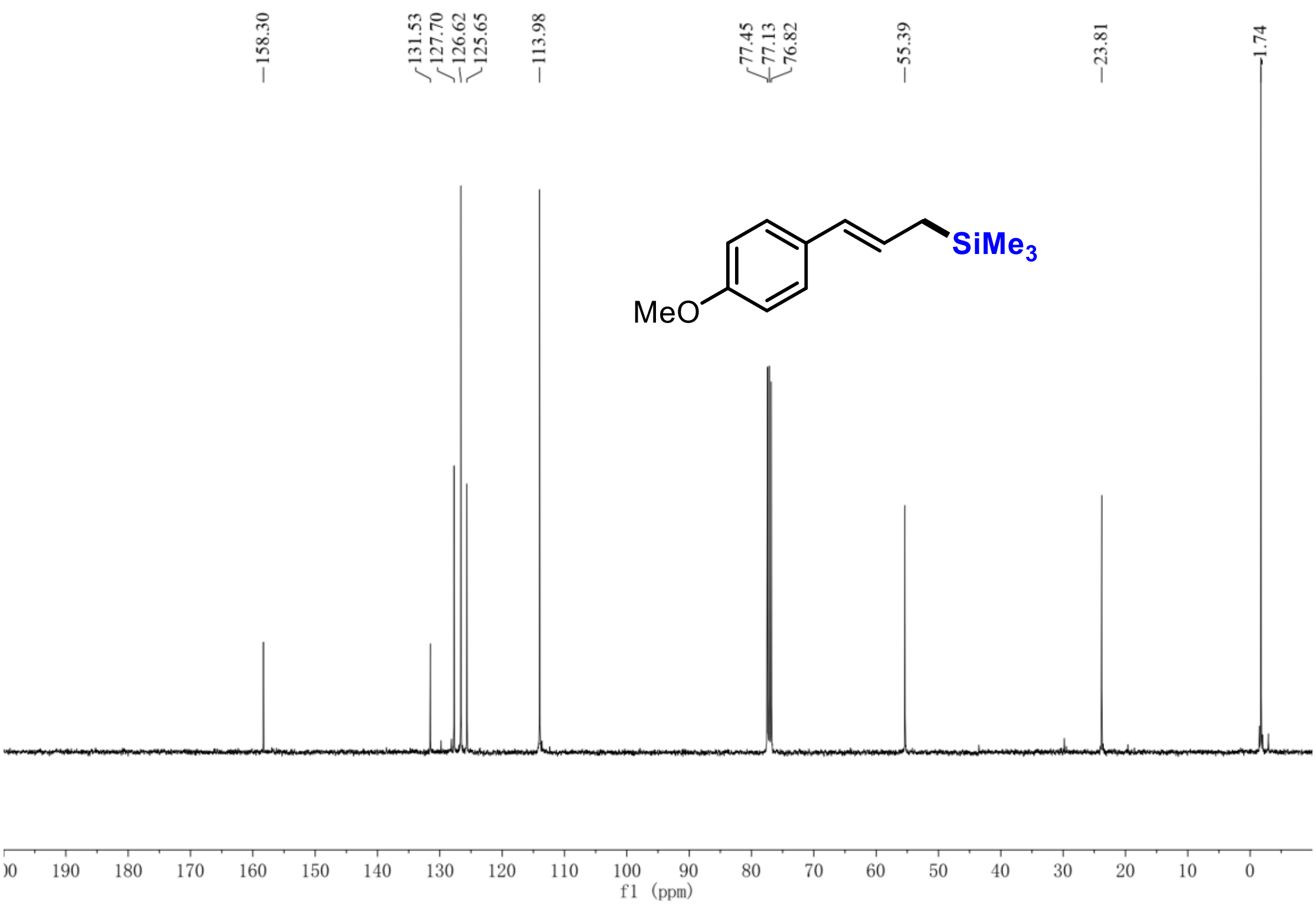

^{1}H NMR spectrum of 5d (400 MHz, $CDCl_3$)

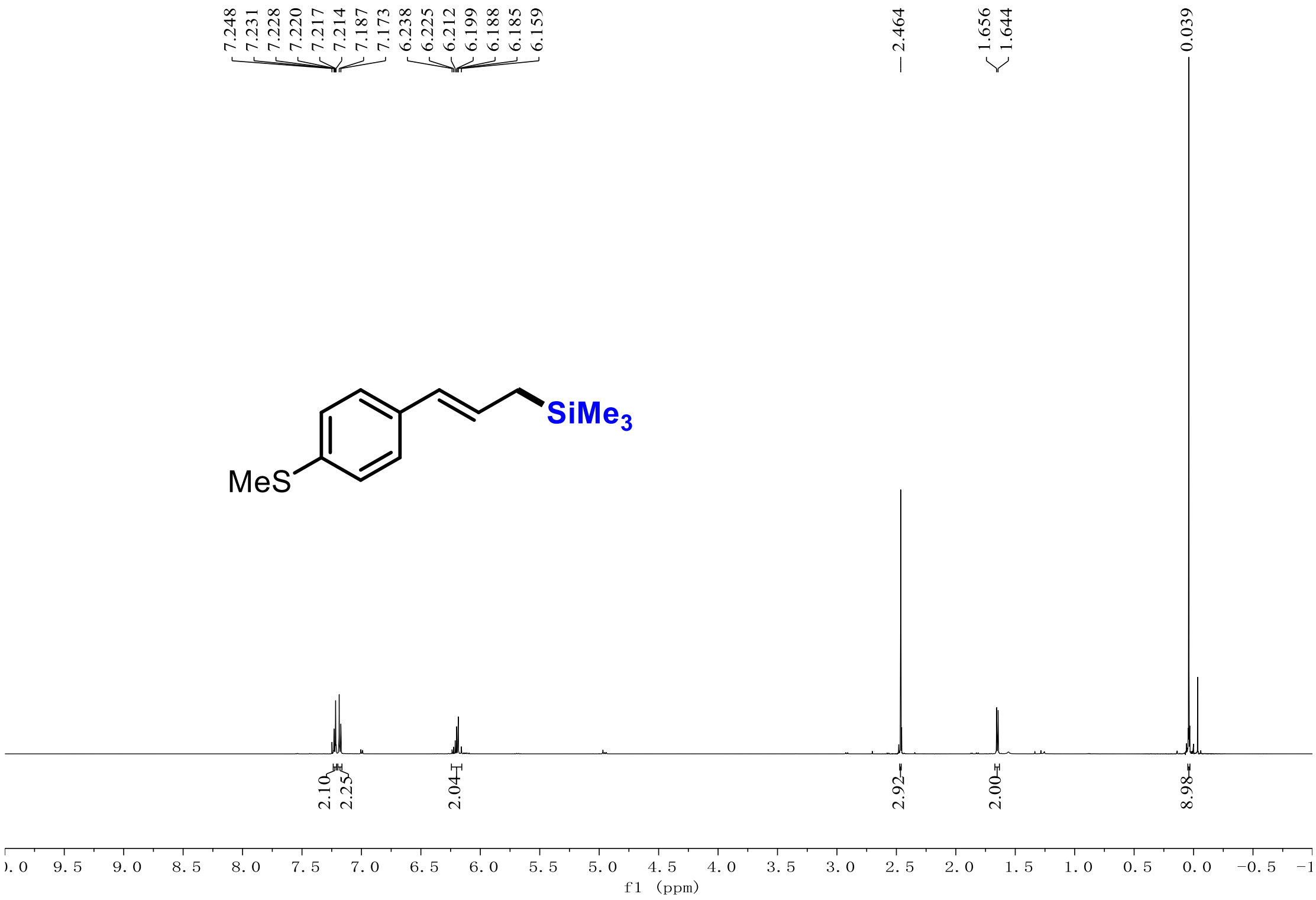

^{13}C NMR spectrum of 5d (101 MHz, $CDCl_3$)

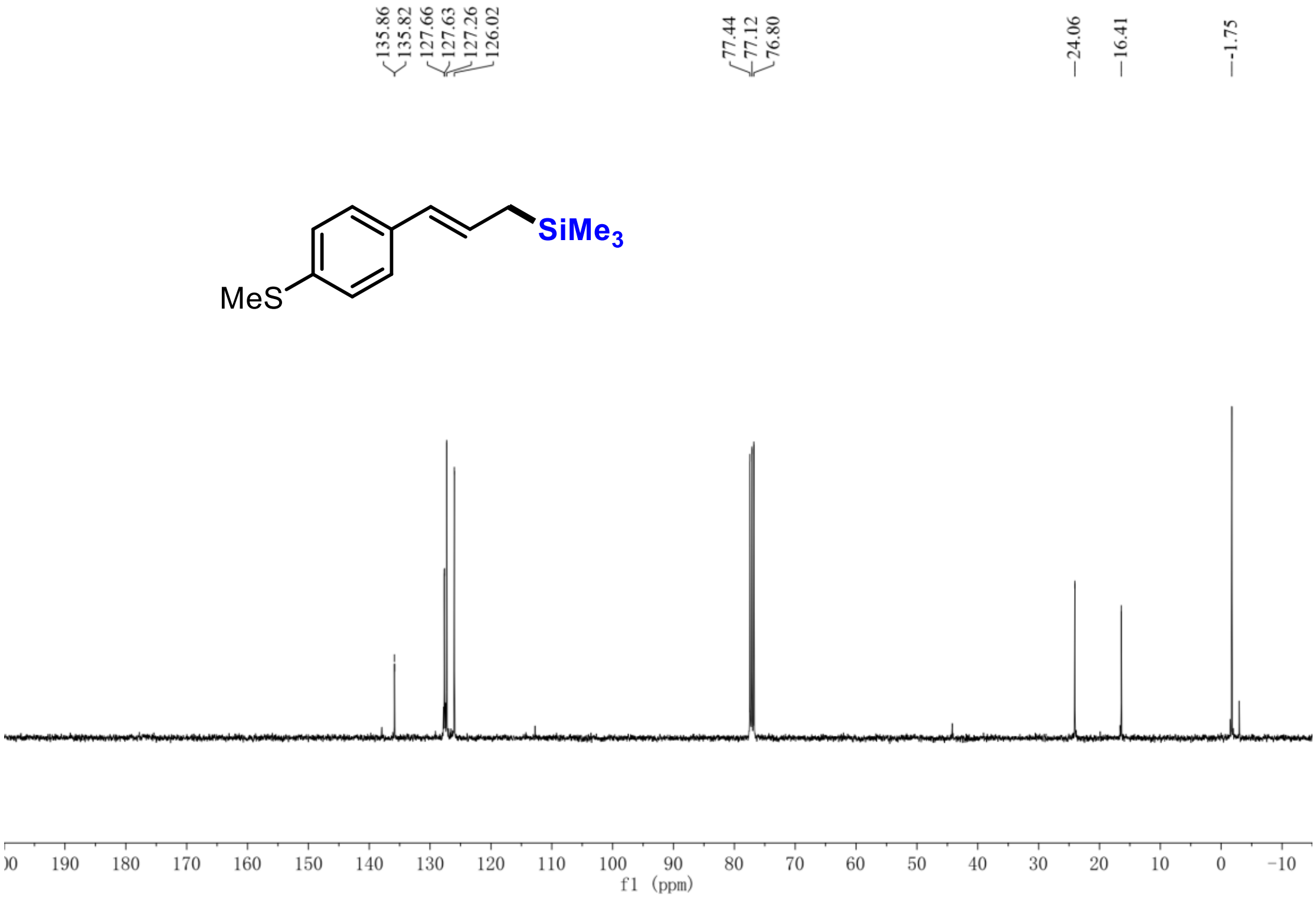

^{1}H NMR spectrum of 5e (400 MHz, $CDCl_3$)

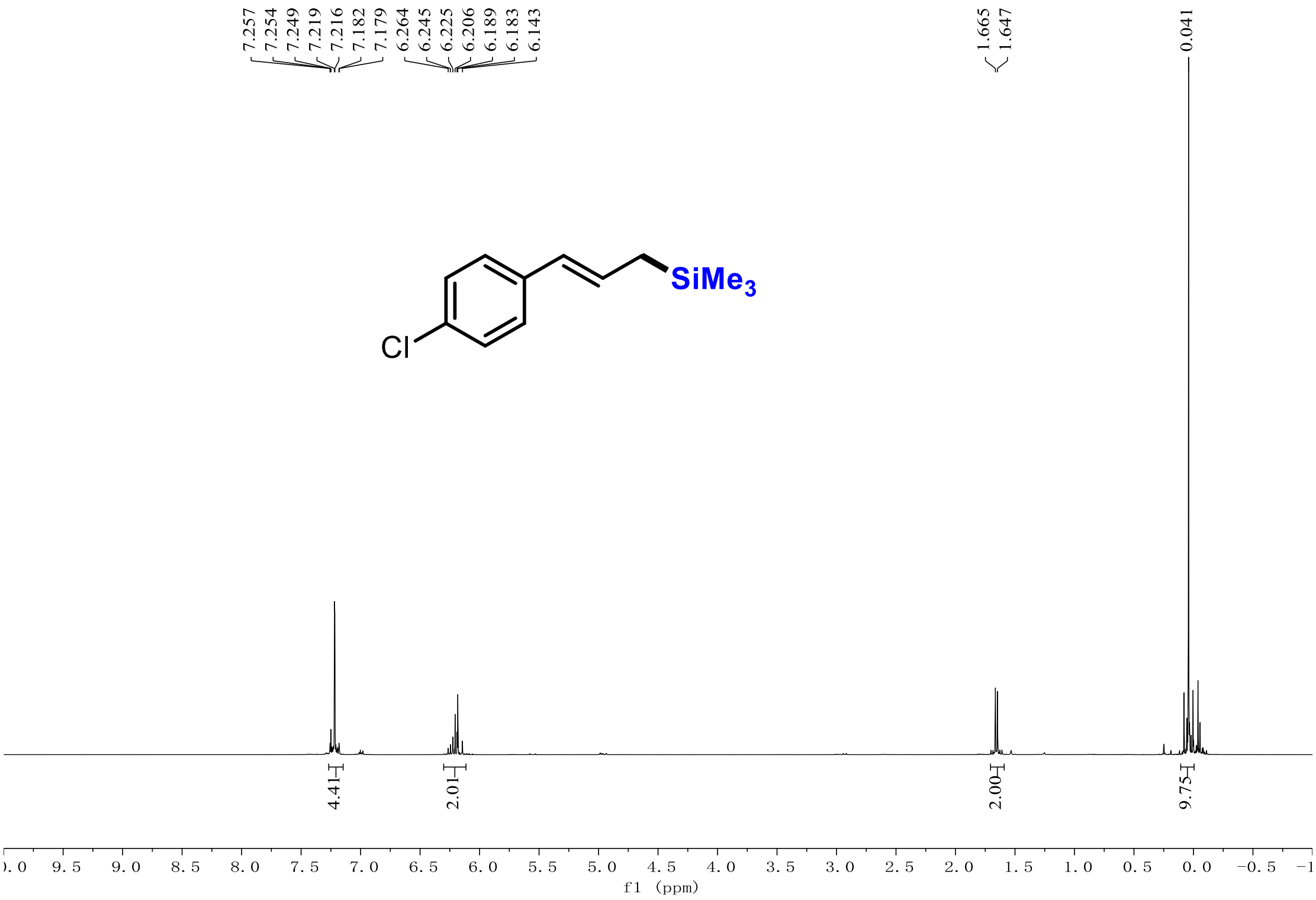

^{13}C NMR spectrum of 5e (101 MHz, $CDCl_3$)

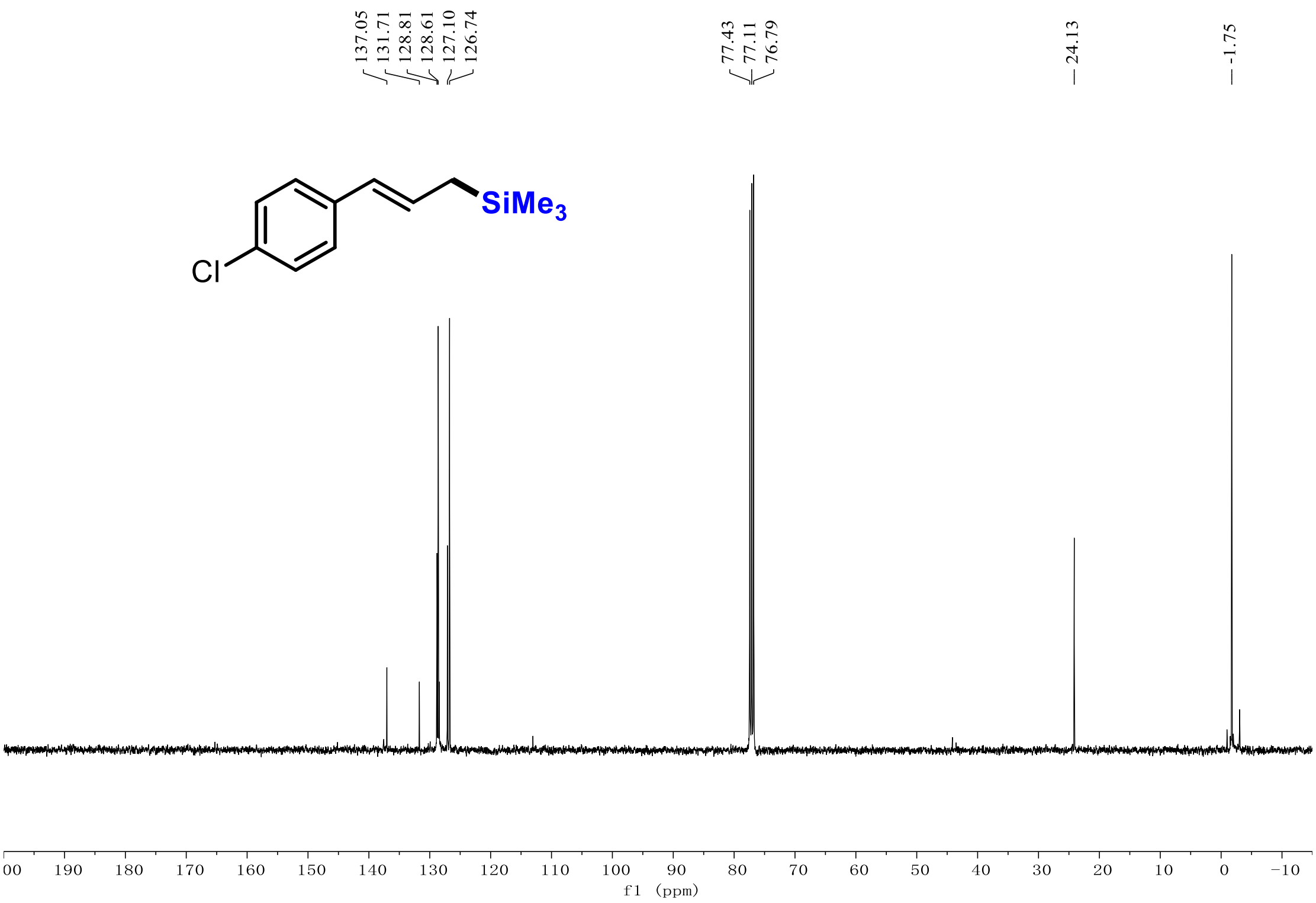

^{1}H NMR spectrum of 5f (400 MHz, $CDCl_3$)

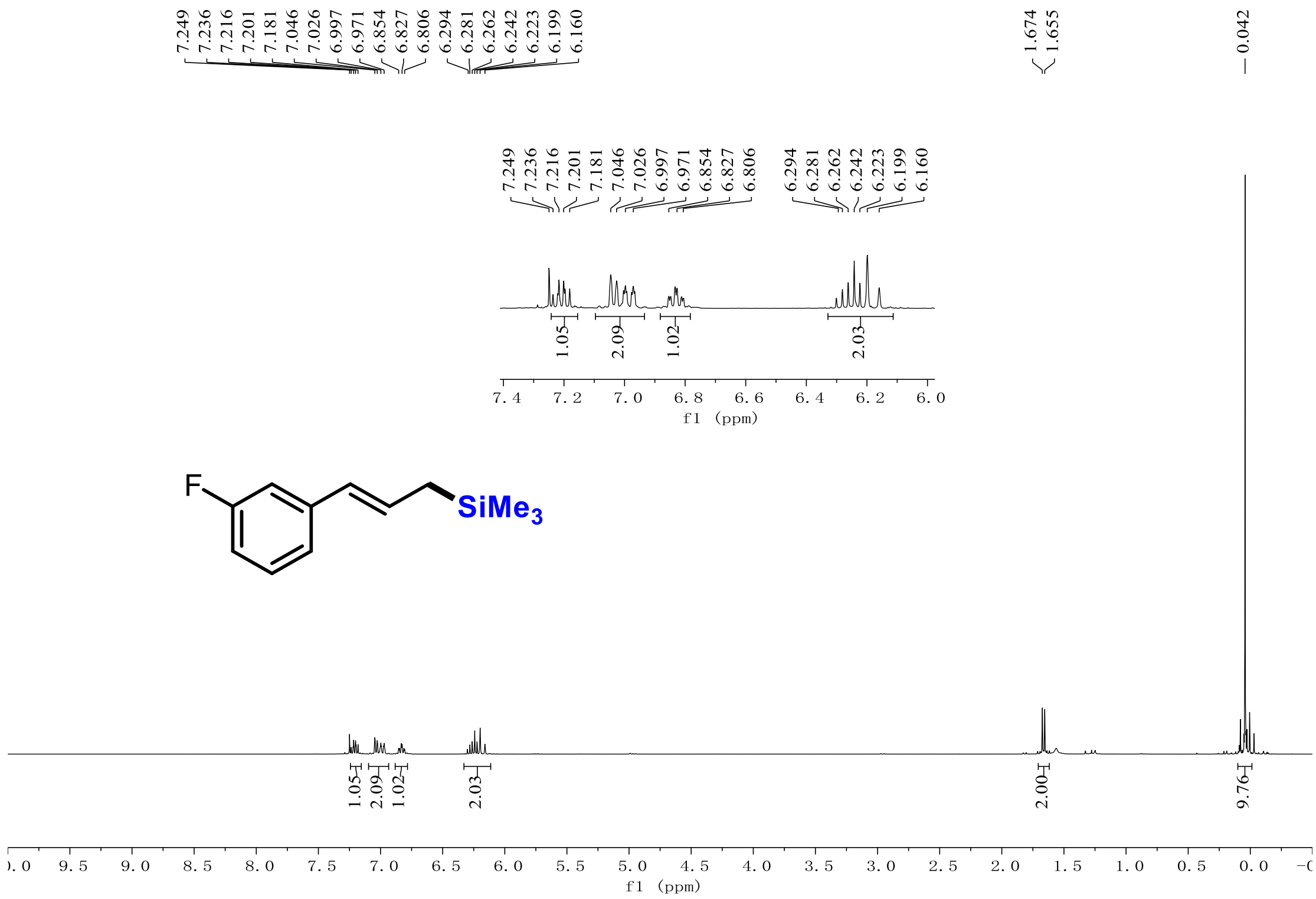

^{13}C NMR spectrum of 5f (101 MHz, $CDCl_3$)

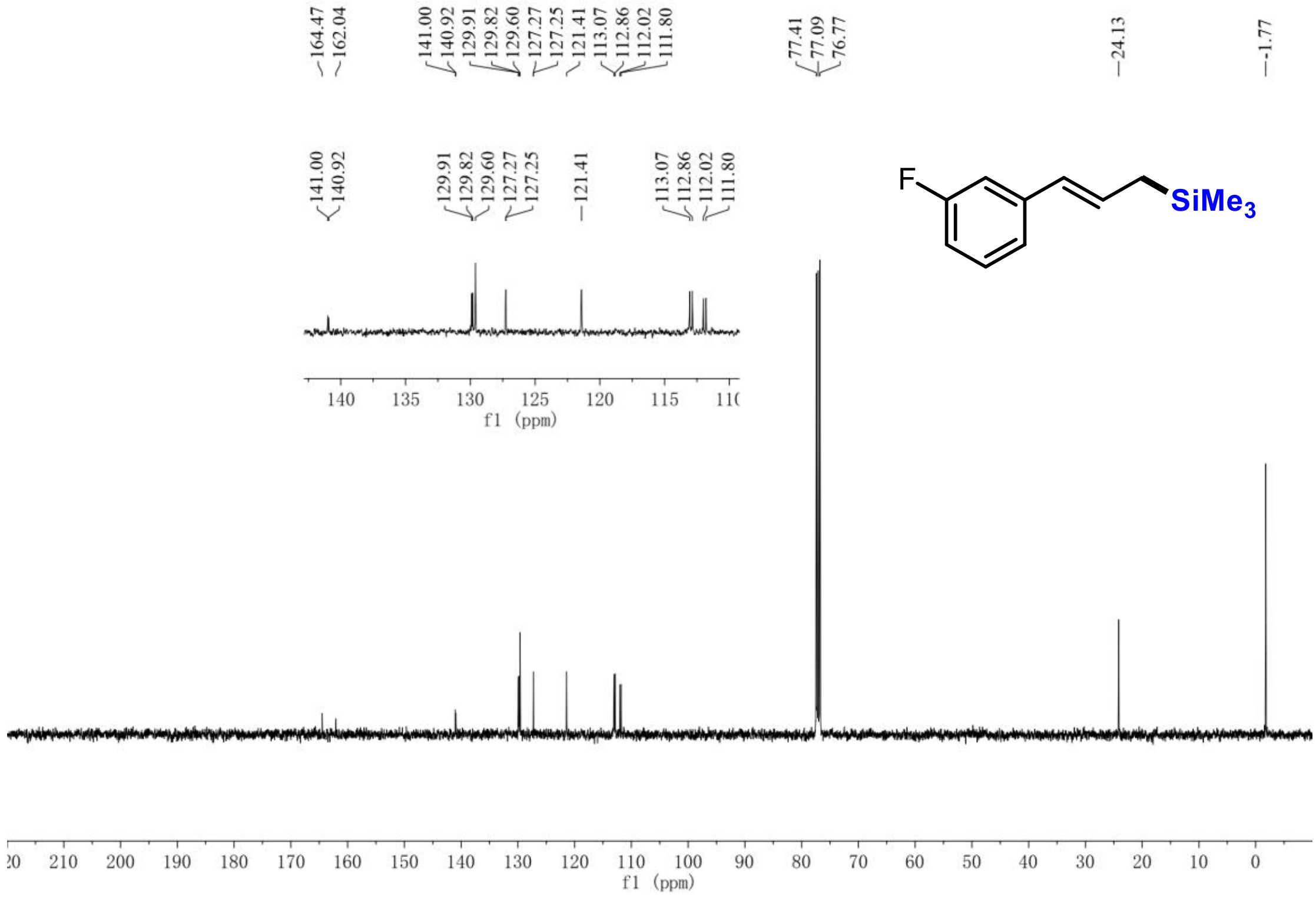

^{1}H NMR spectrum of 5g (400 MHz, $CDCl_3$)

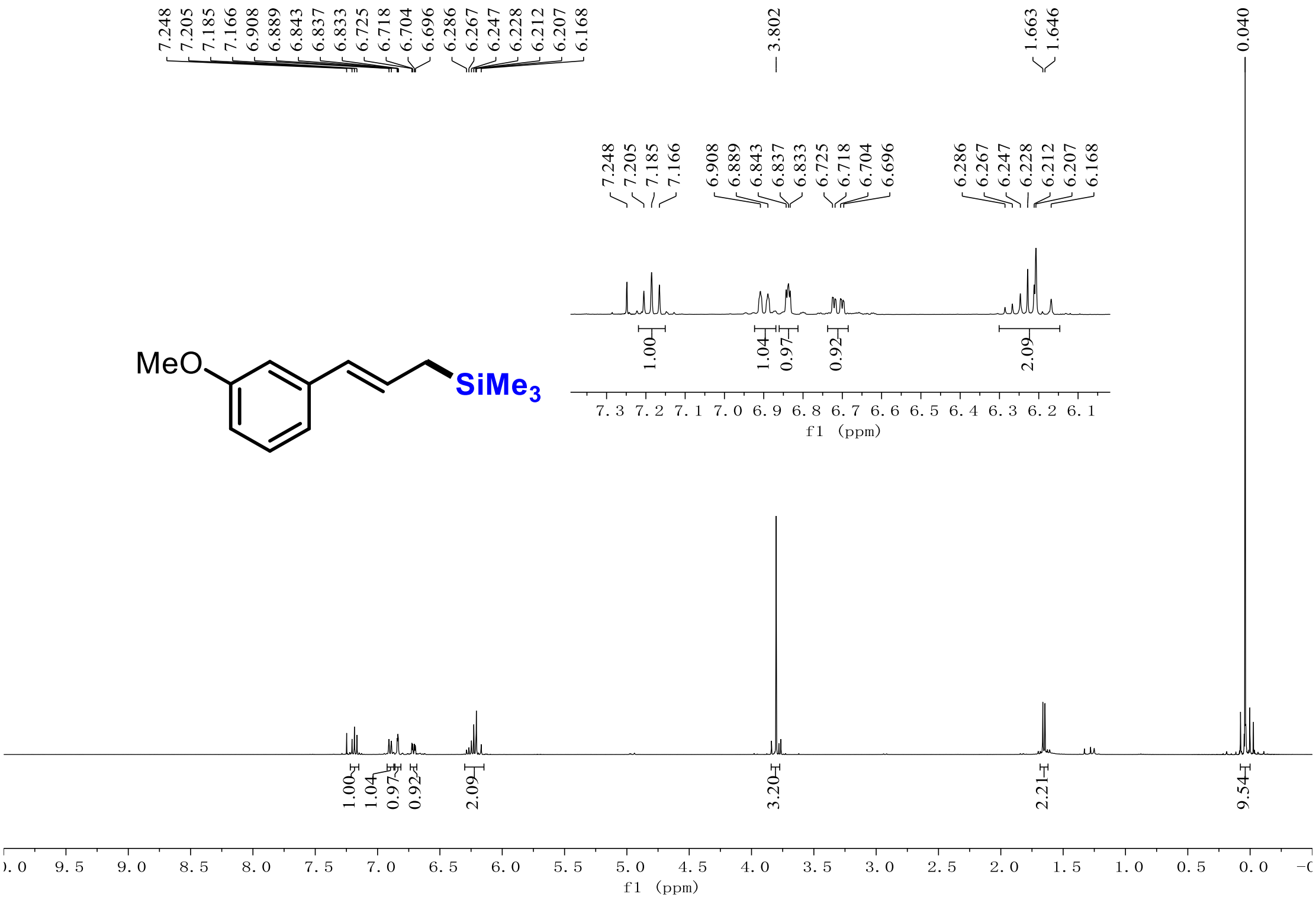

^{13}C NMR spectrum of 5g (101 MHz, $CDCl_3$)

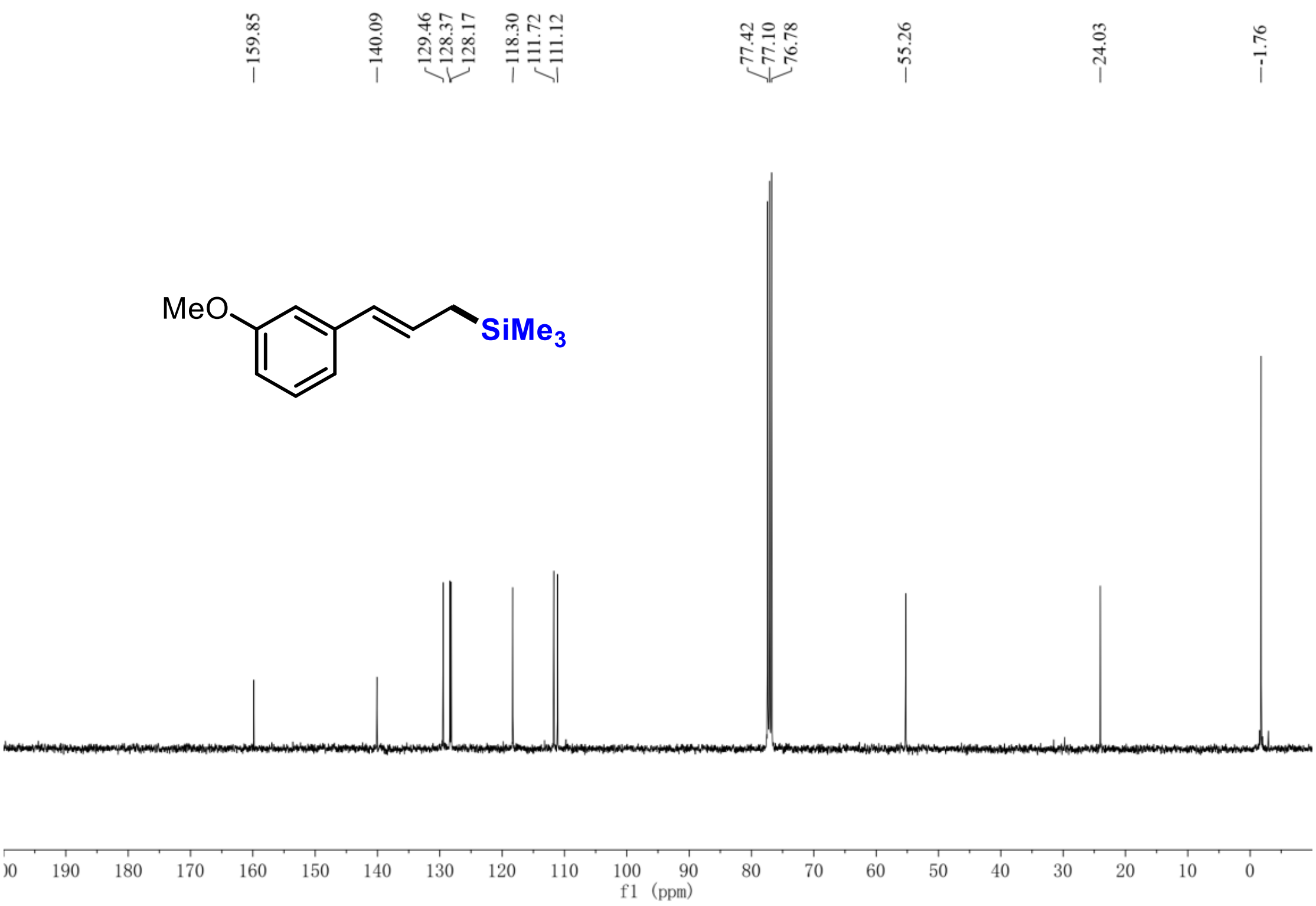

^{1}H NMR spectrum of 5h (400 MHz, $CDCl_3$)

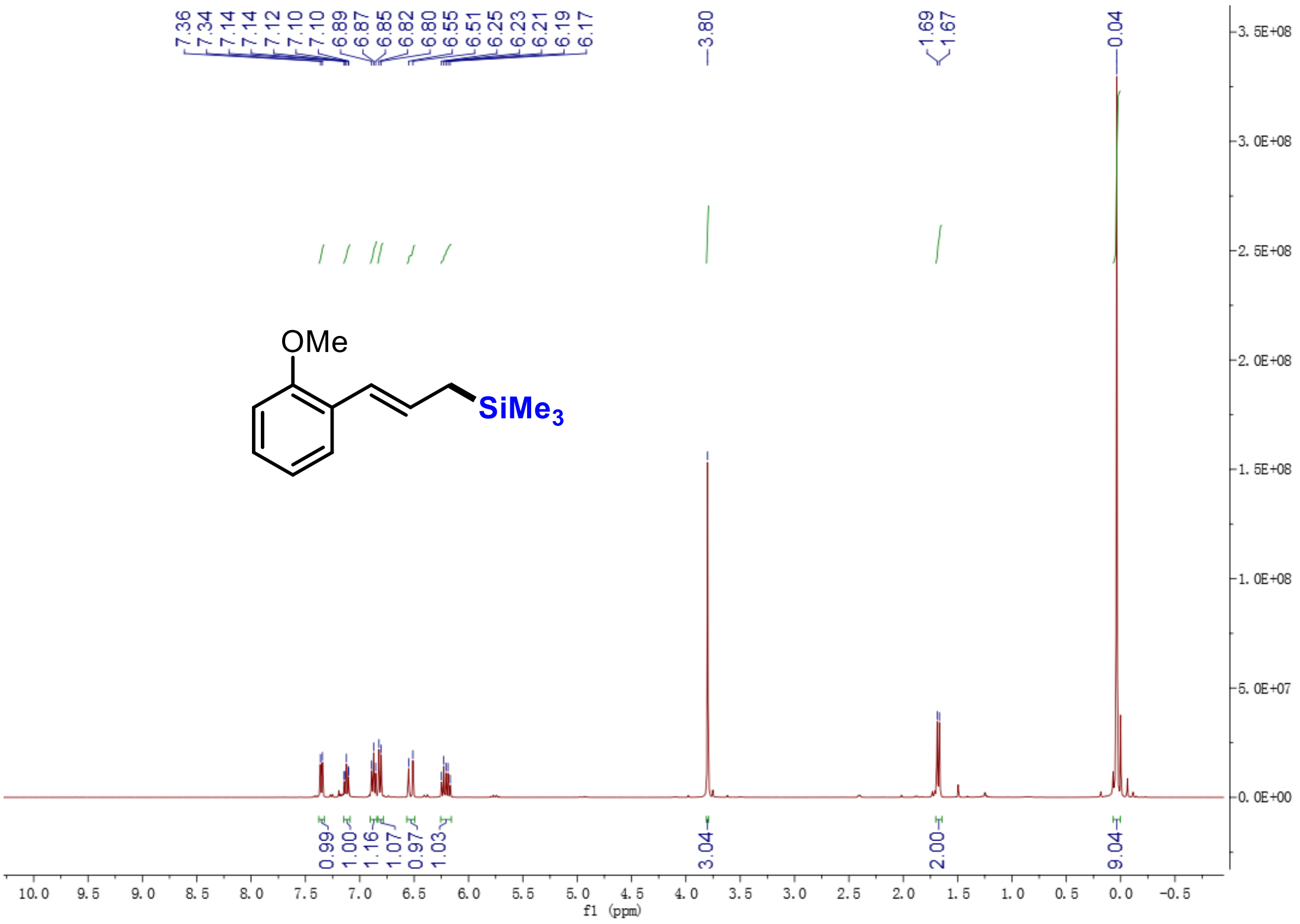

^{13}C NMR spectrum of 5h (101 MHz, $CDCl_3$)

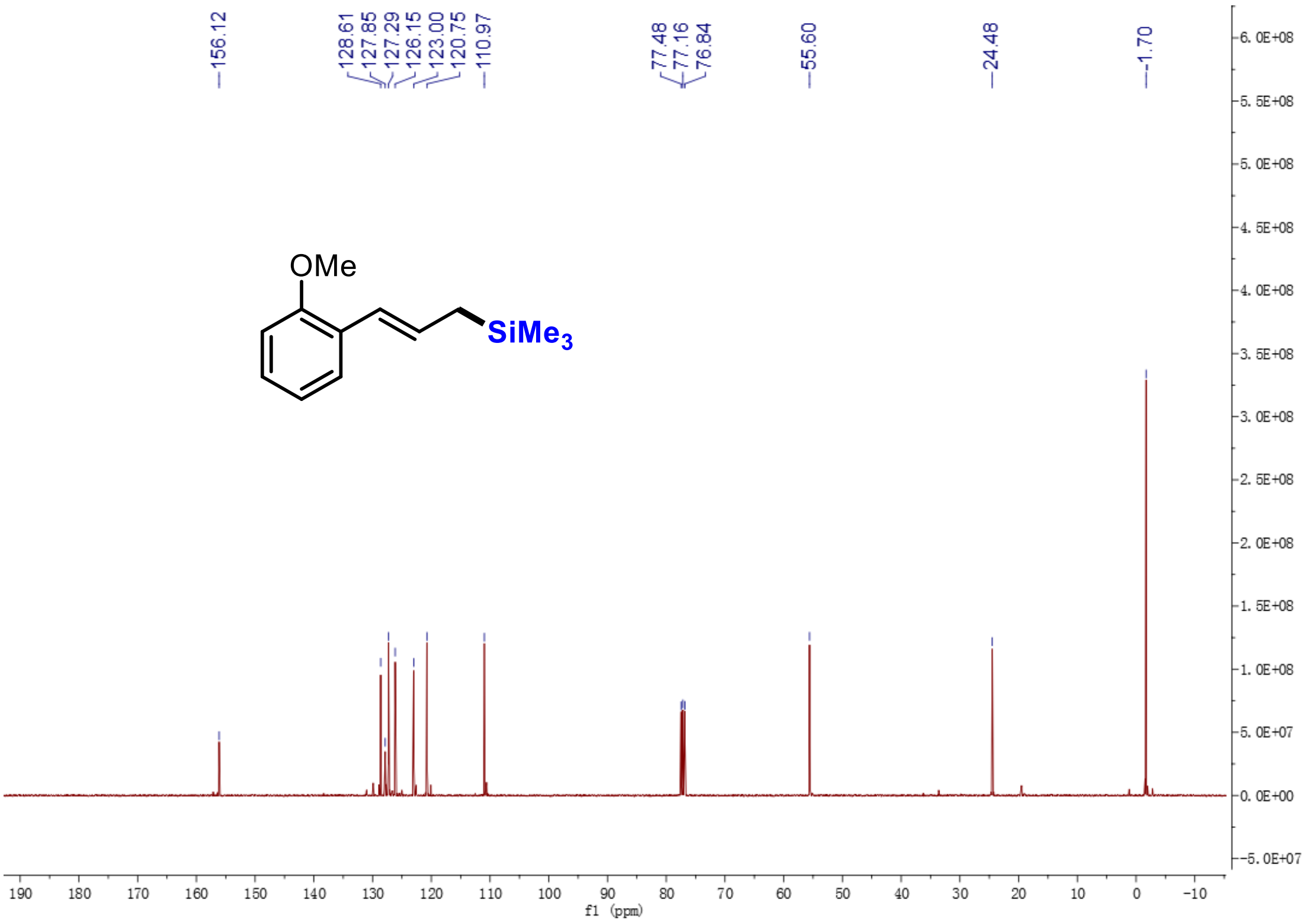

^{1}H NMR spectrum of 5i (400 MHz, $CDCl_3$)

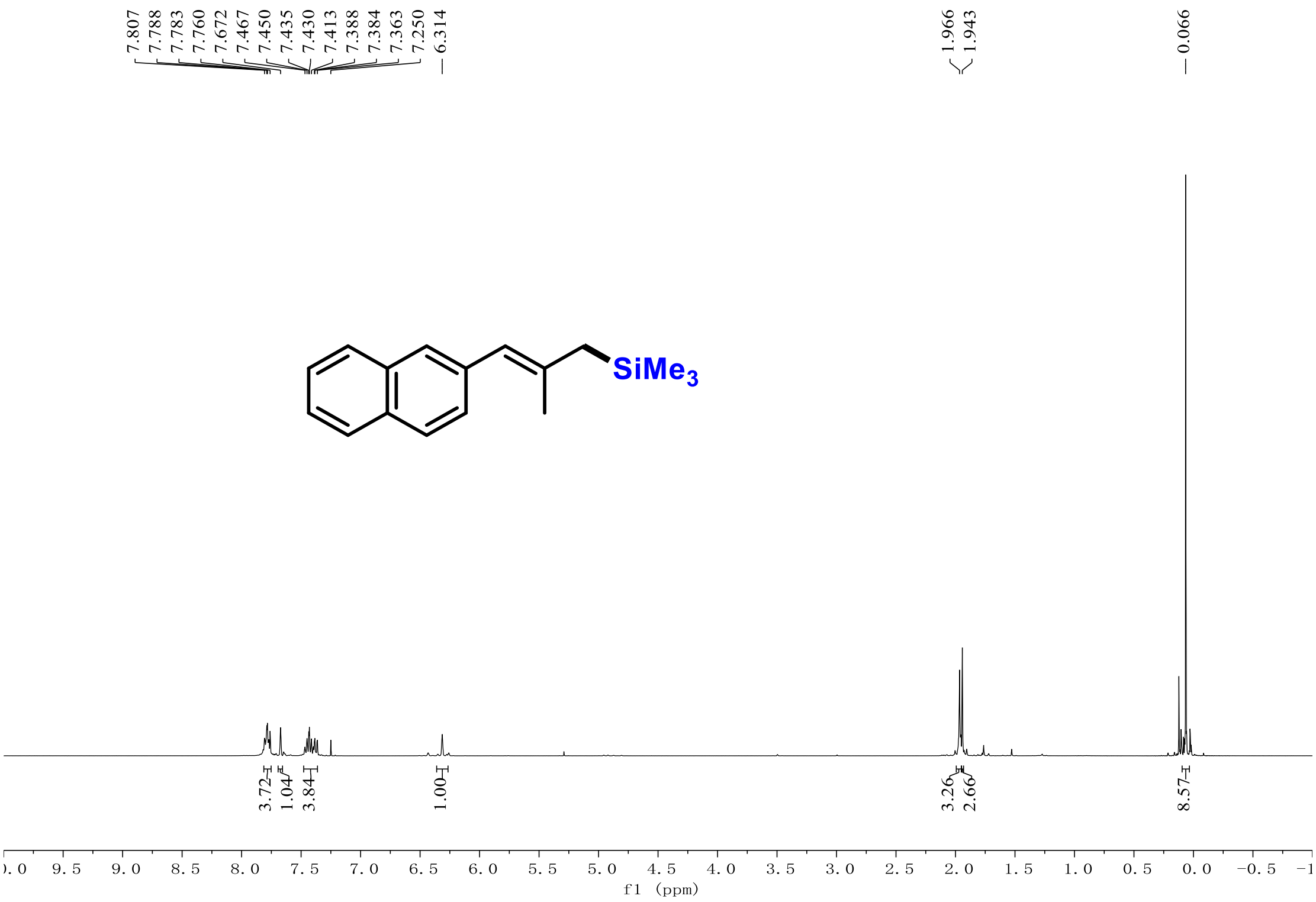

^{13}C NMR spectrum of 5i (101 MHz, $CDCl_3$)

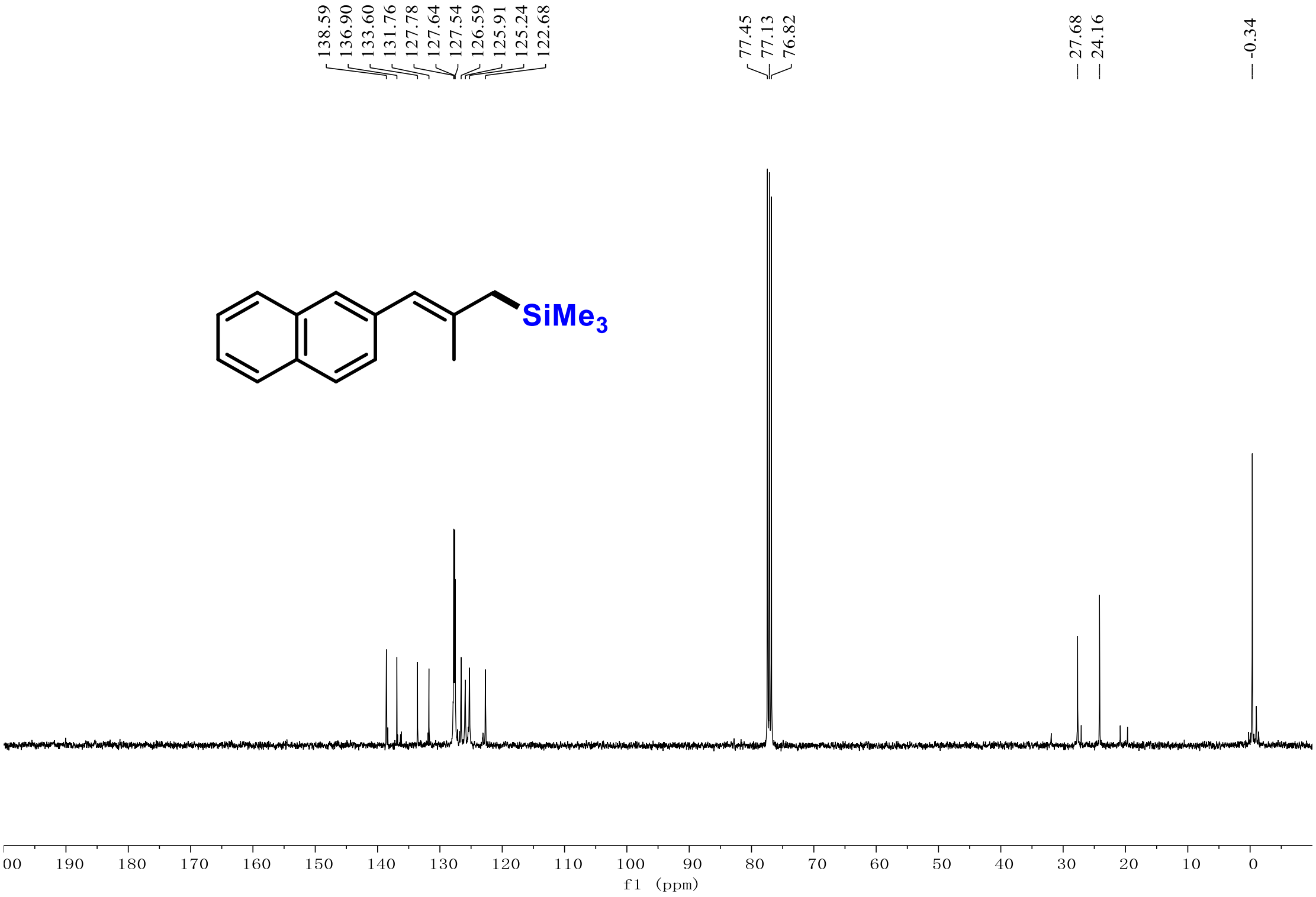

^{1}H NMR spectrum of 5j (400 MHz, $CDCl_3$)

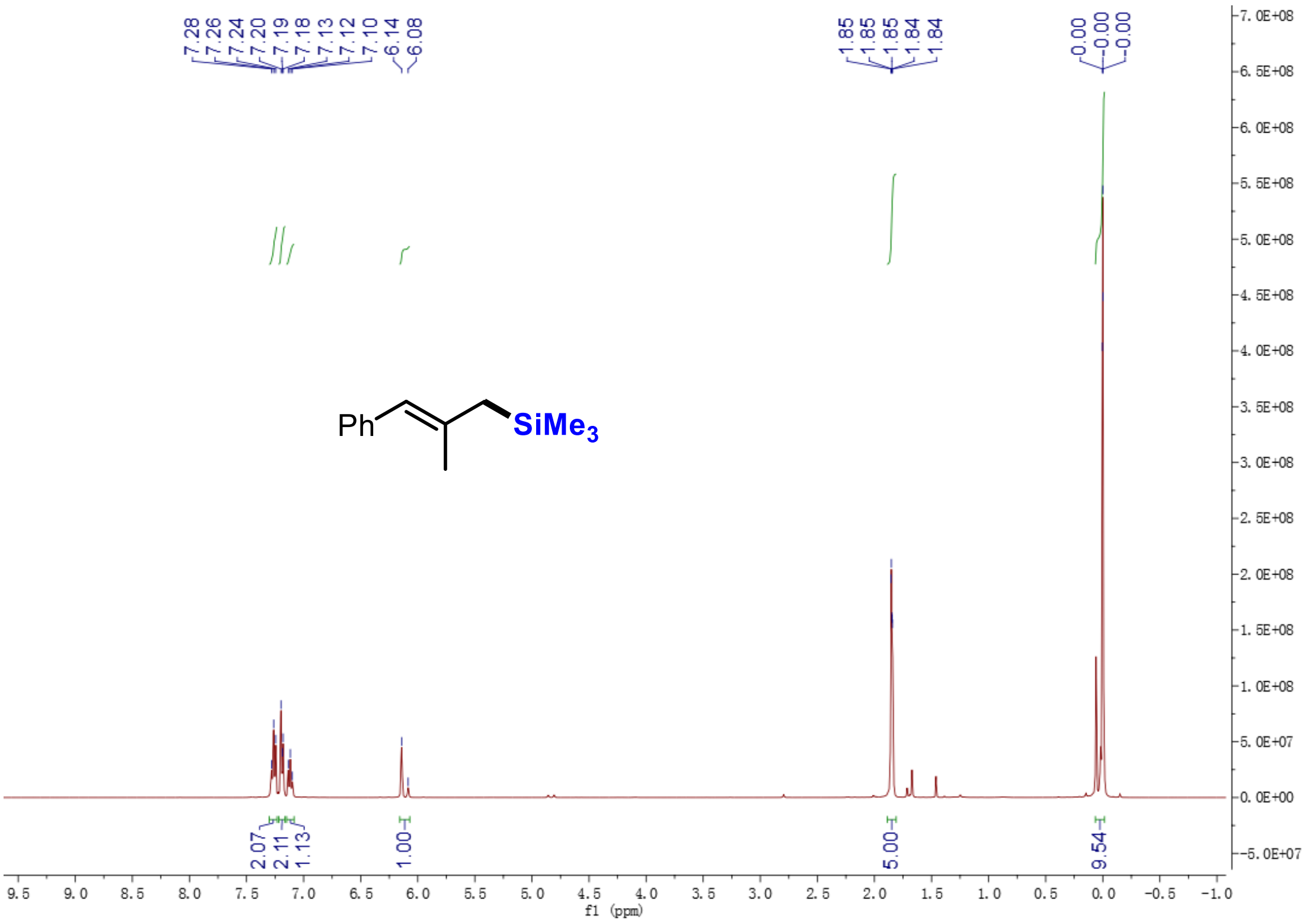

^{13}C NMR spectrum of 5j (101 MHz, $CDCl_3$)

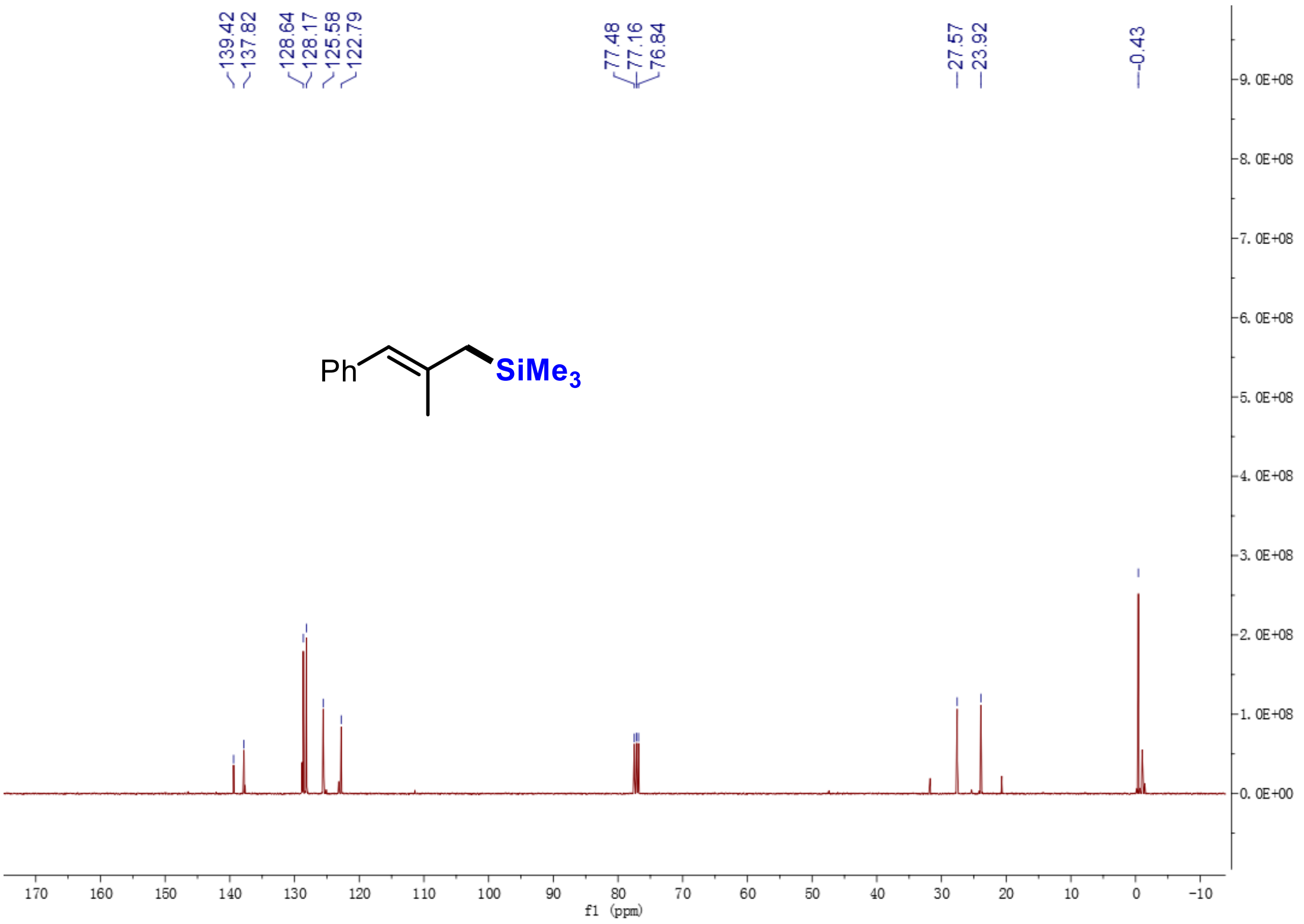

^{1}H NMR spectrum of 5k (400 MHz, $CDCl_3$)

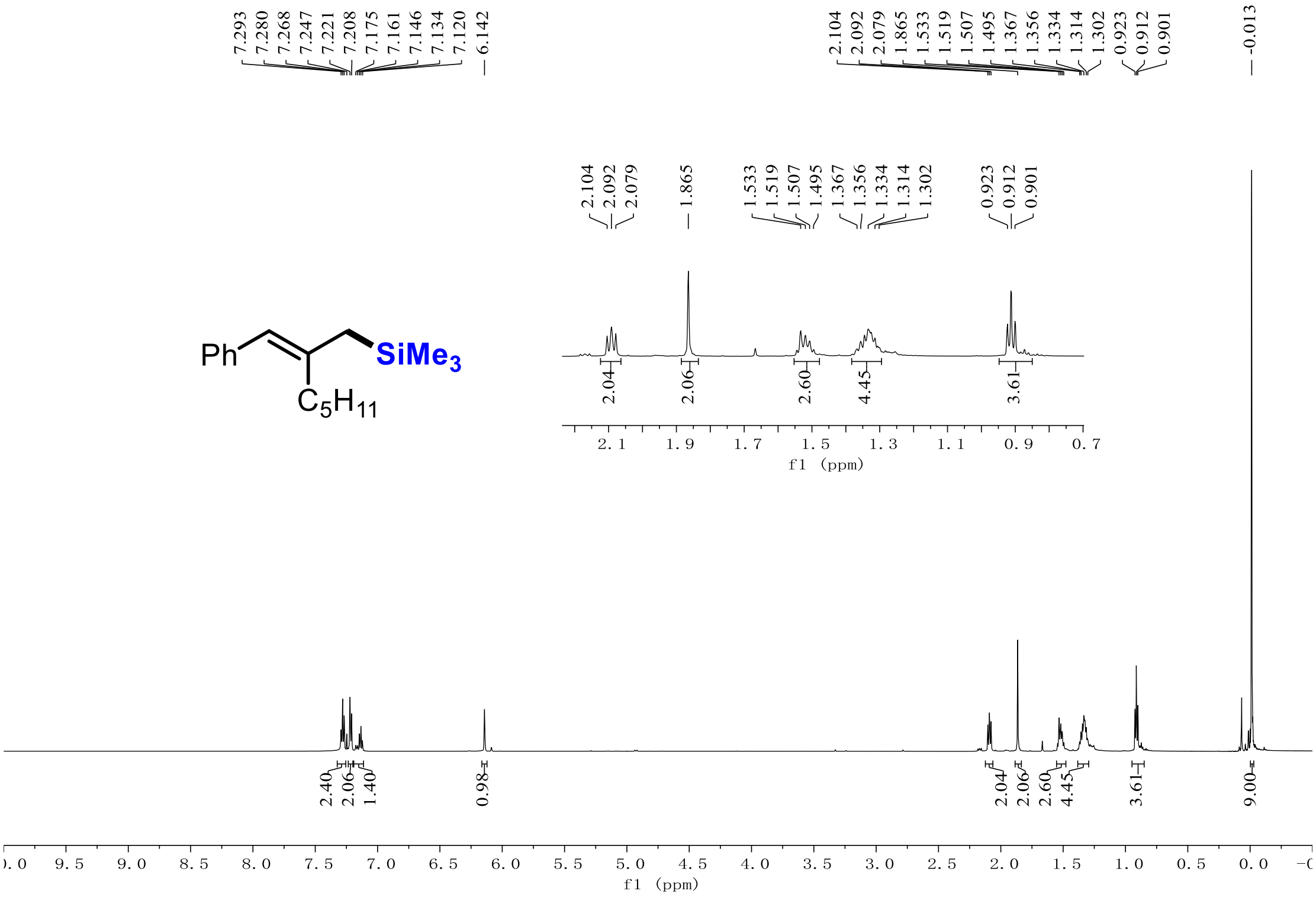

^{13}C NMR spectrum of 5k (101 MHz, $CDCl_3$)

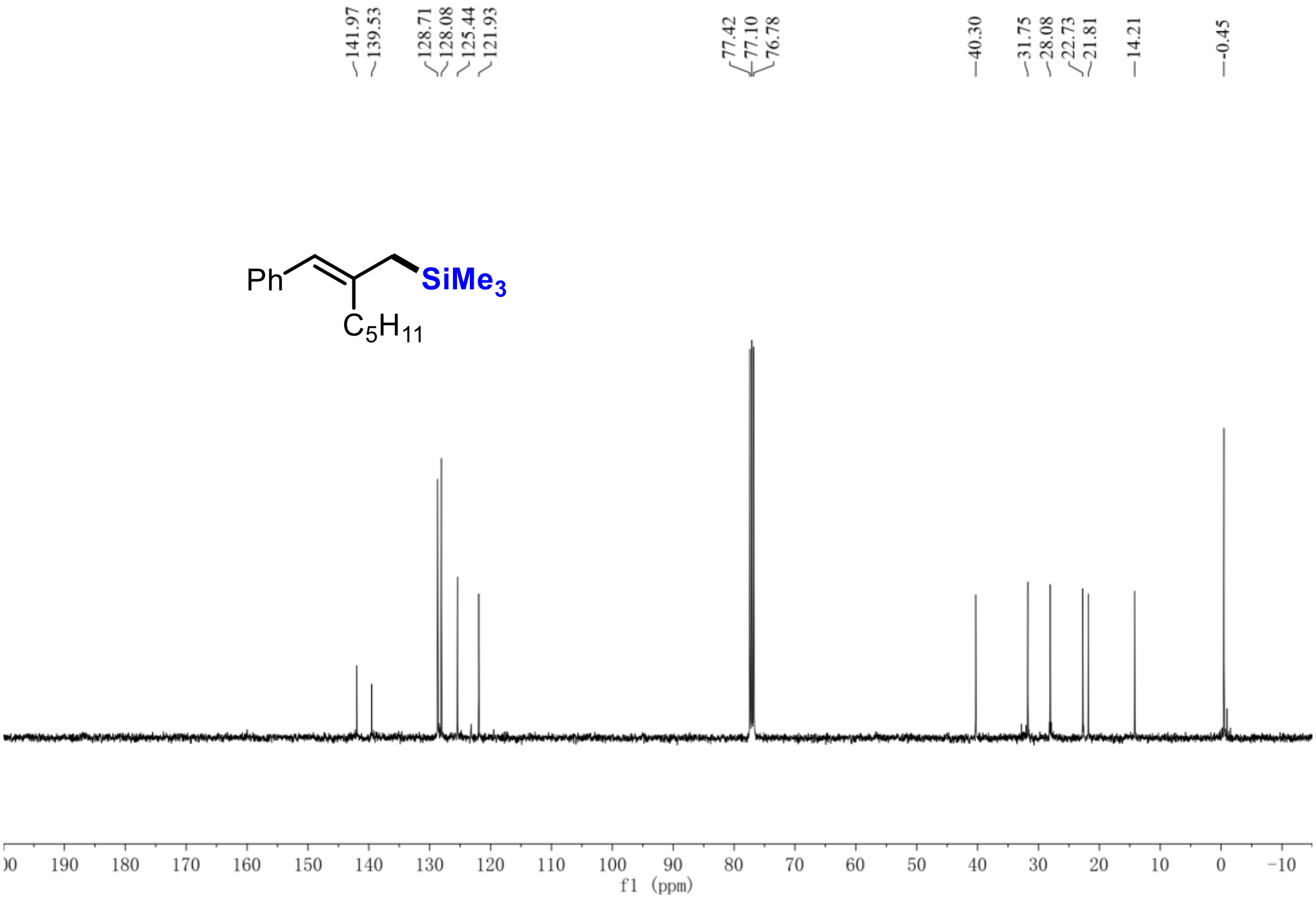

^{1}H NMR spectrum of 5l (400 MHz, $CDCl_3$)

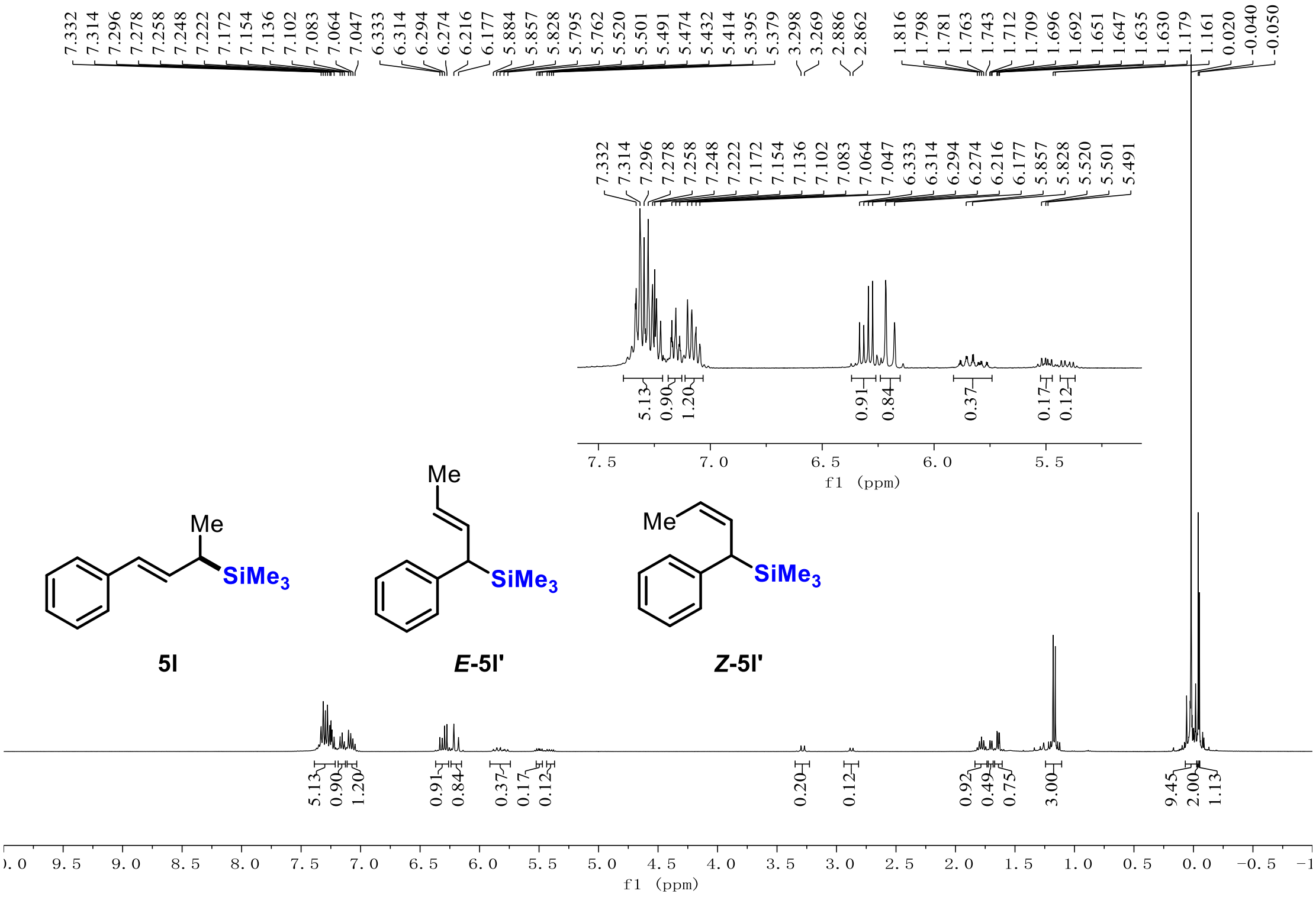

^{13}C NMR spectrum of 5l (101 MHz, $CDCl_3$)

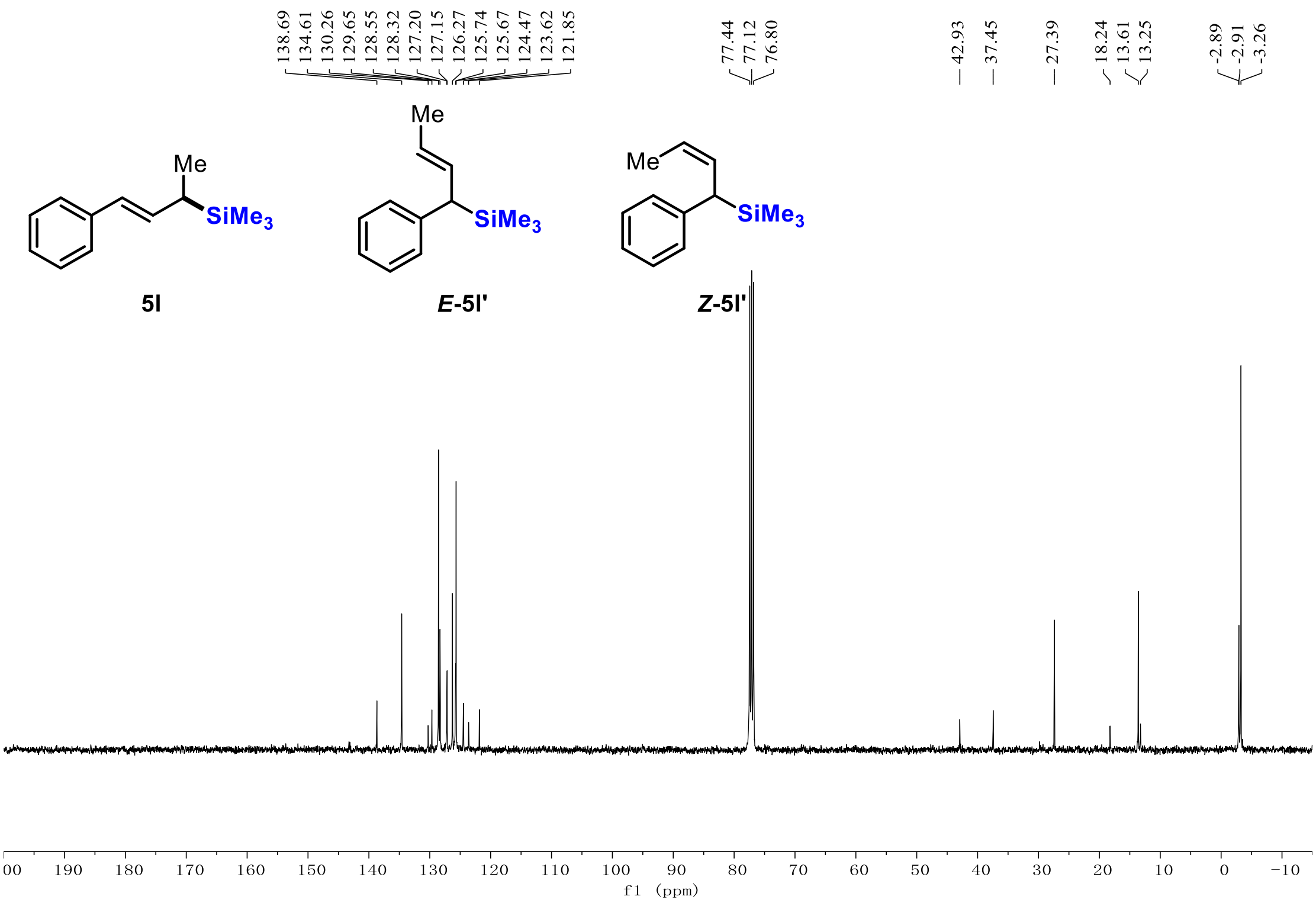

^{1}H NMR spectrum of 5m (400 MHz, $CDCl_3$)

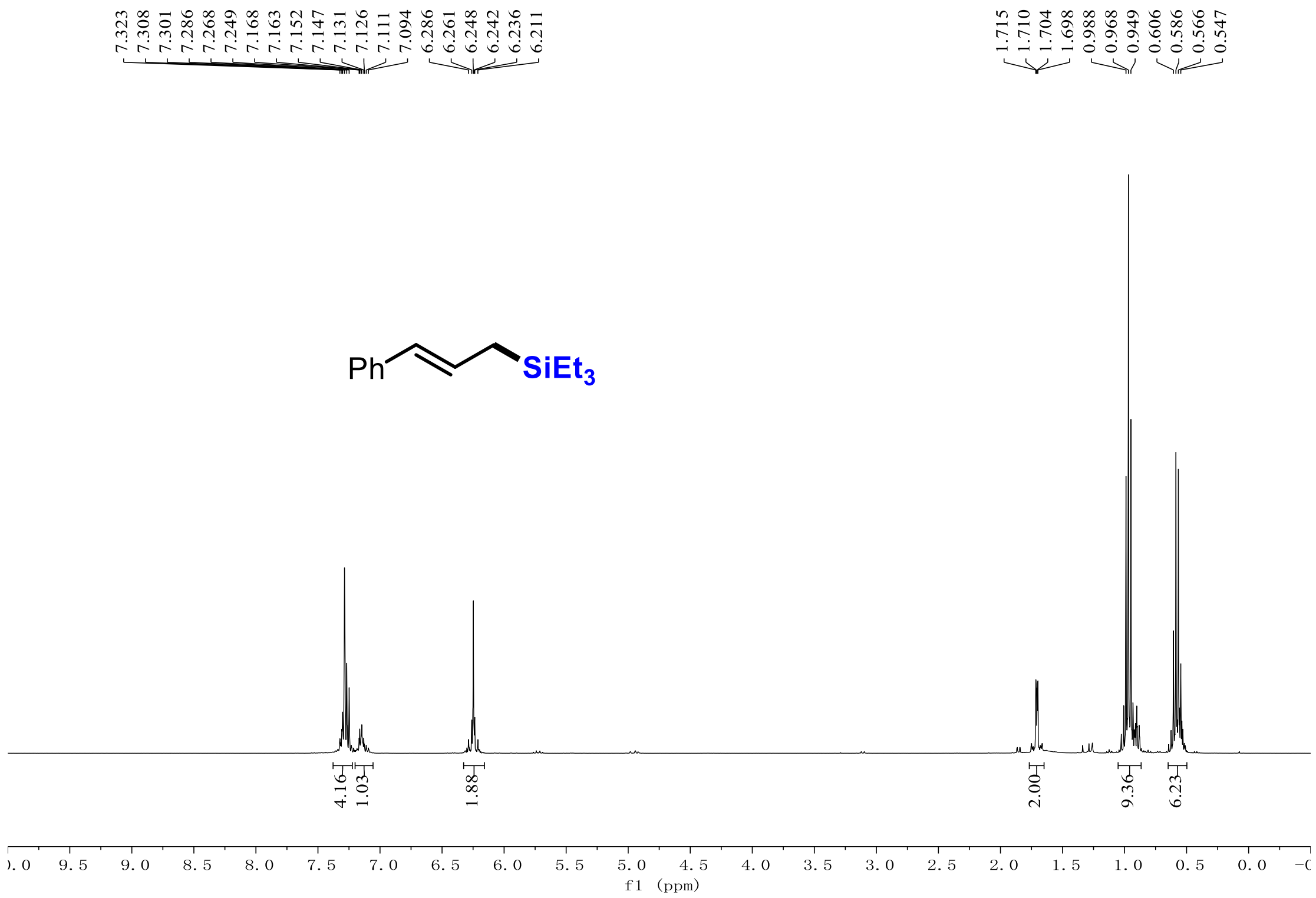

^{13}C NMR spectrum of 5m (101 MHz, $CDCl_3$)

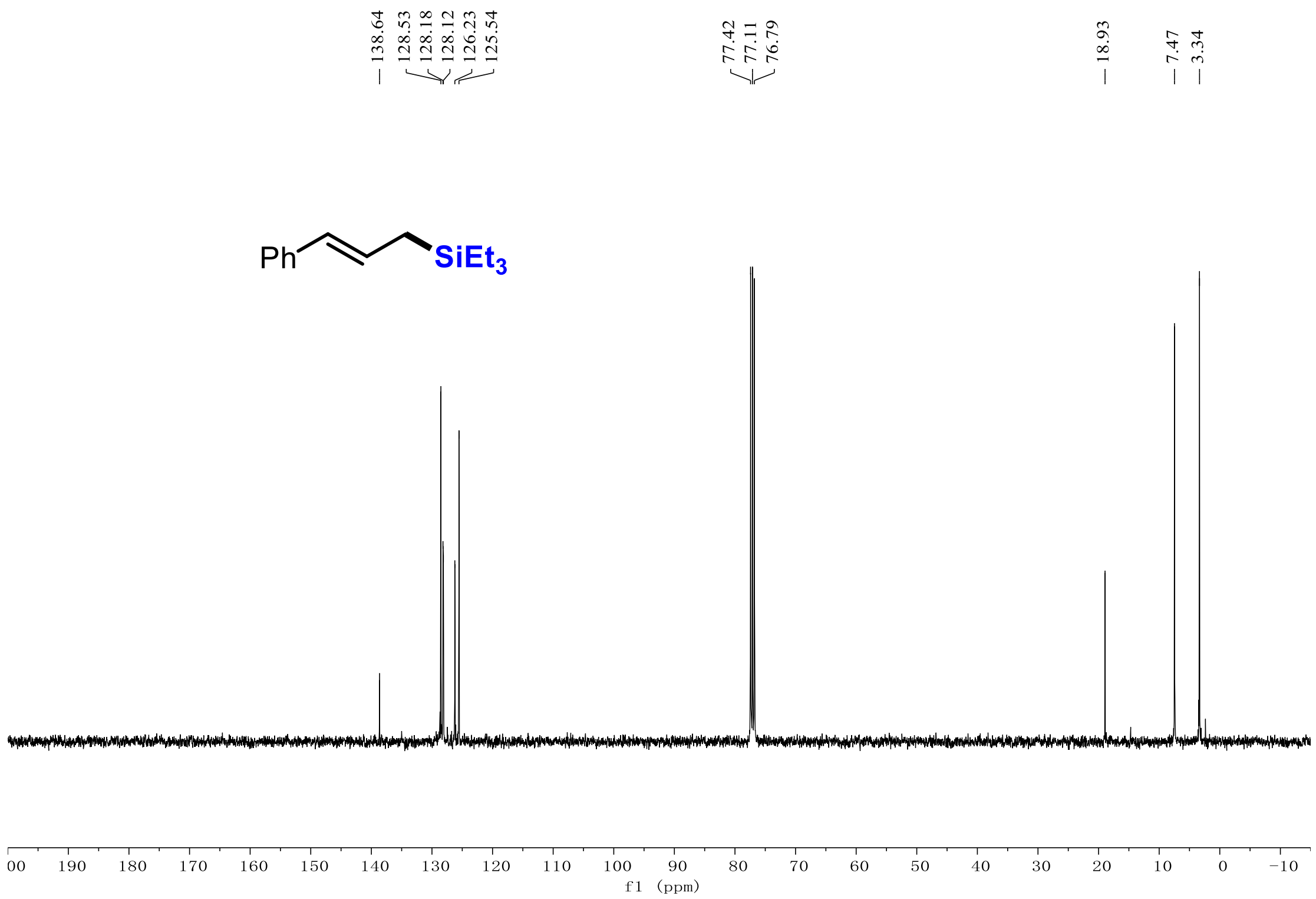

^{1}H NMR spectrum of 5n (400 MHz, $CDCl_3$)

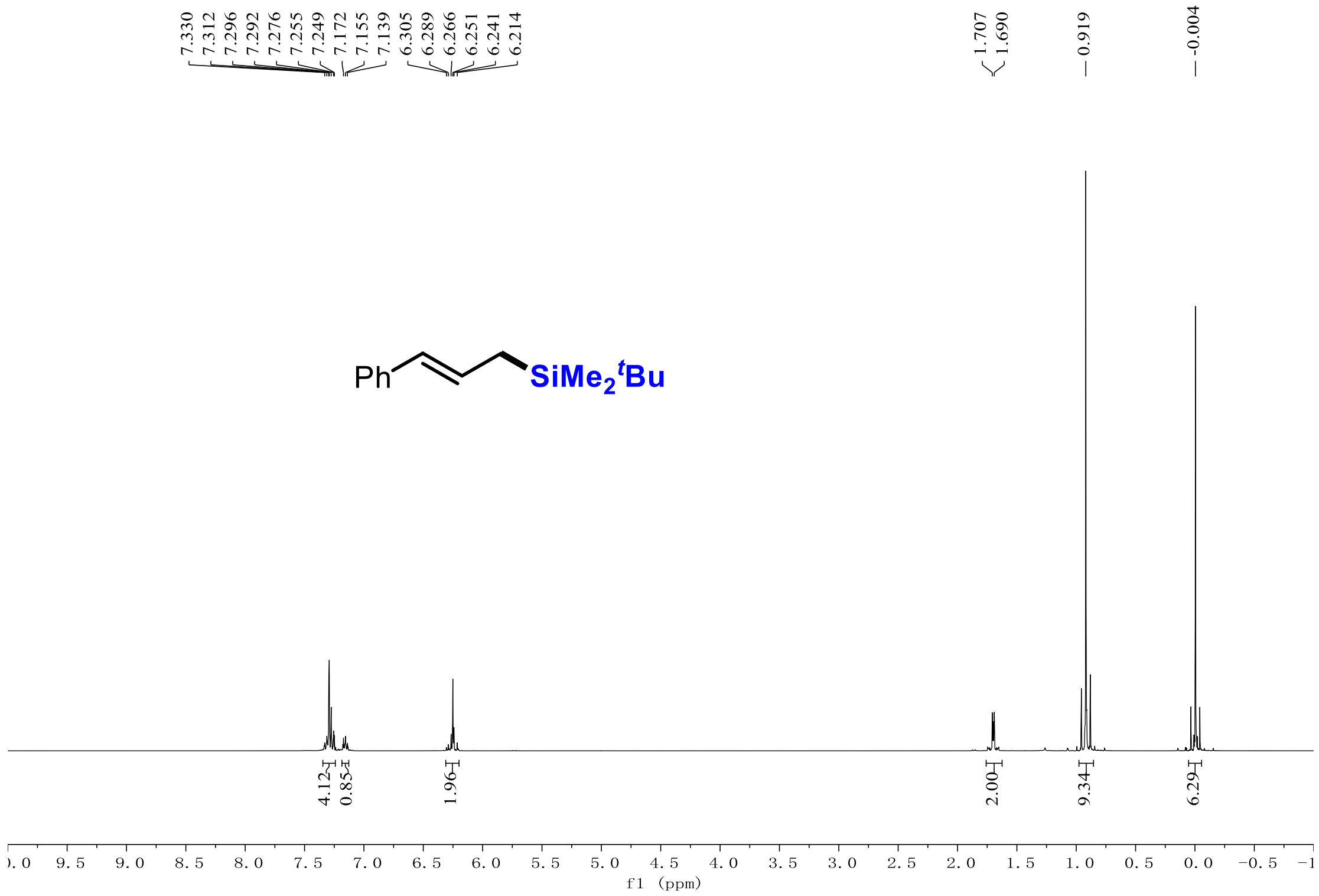

^{13}C NMR spectrum of 5n (101 MHz, $CDCl_3$)

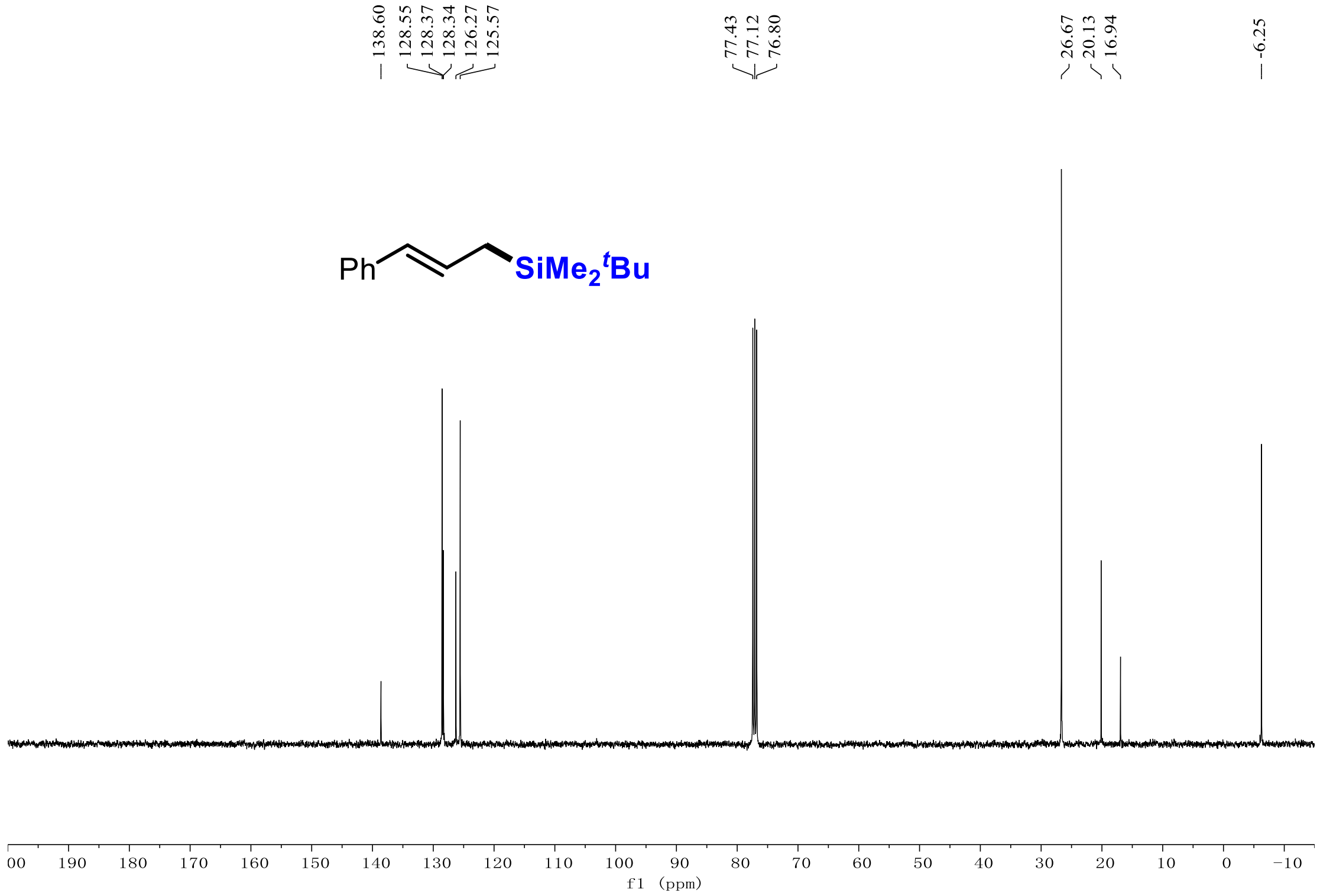

^{1}H NMR spectrum of 5o (400 MHz, $CDCl_3$)

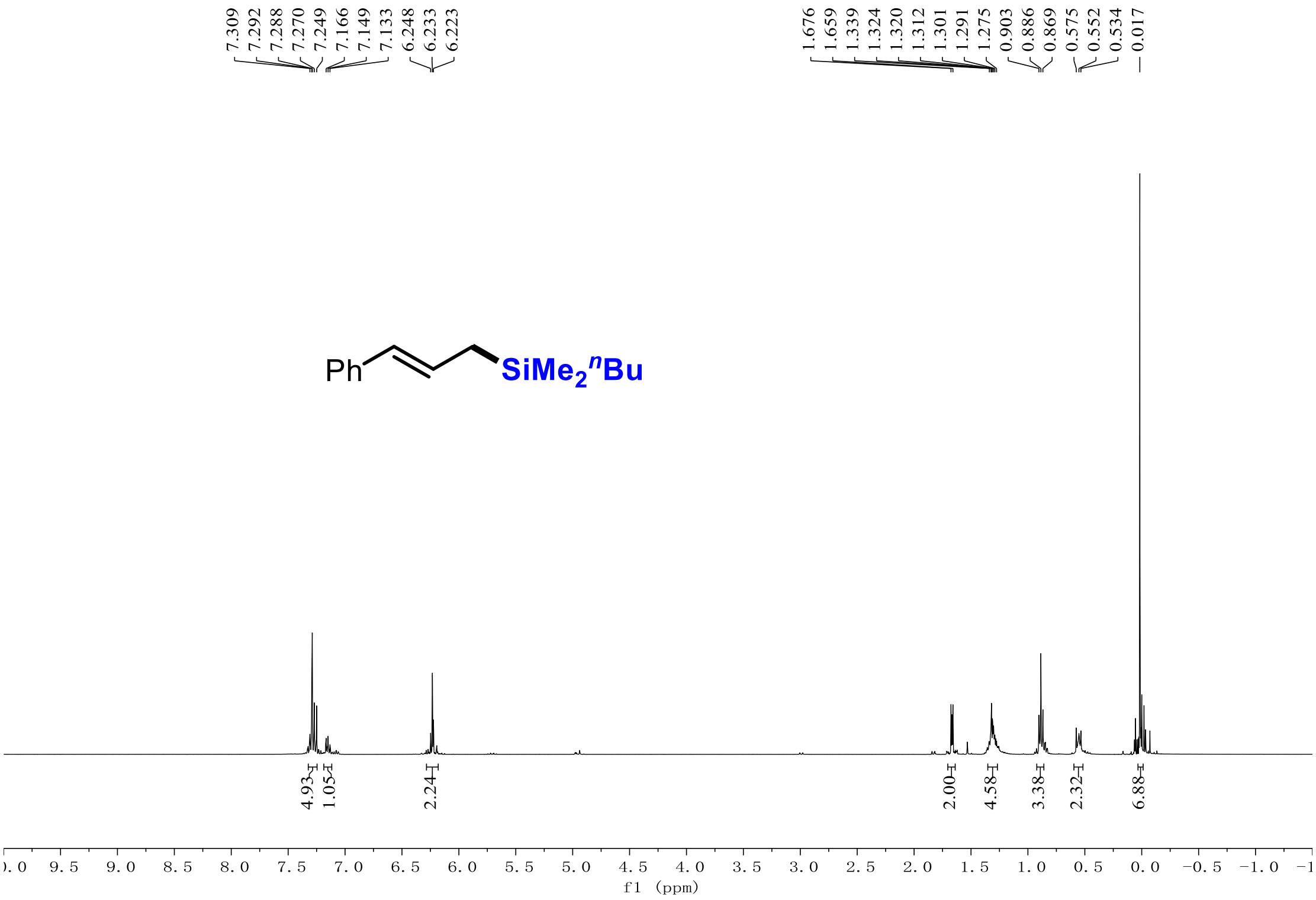

^{13}C NMR spectrum of 5o (101 MHz, $CDCl_3$)

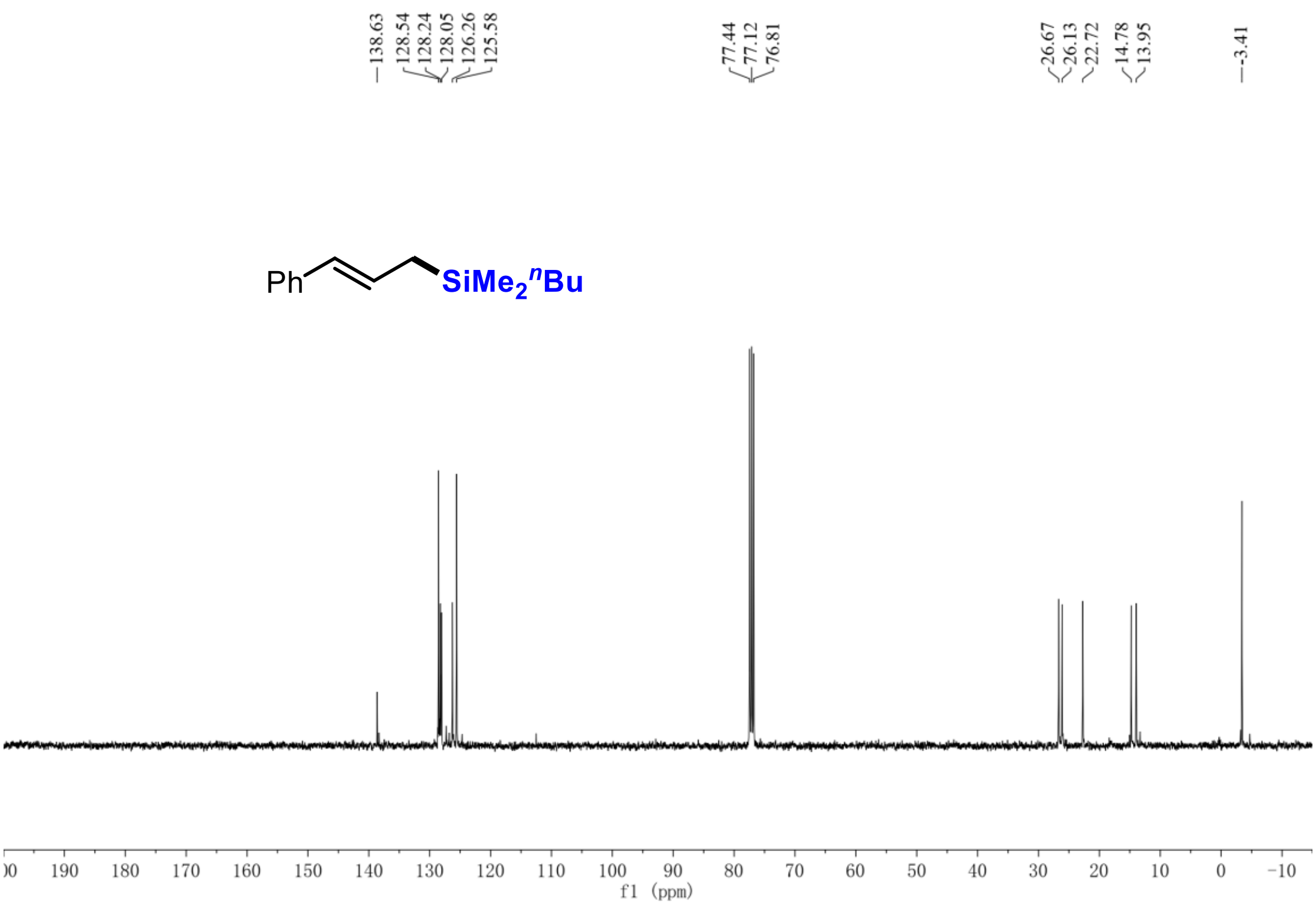

^{1}H NMR spectrum of 5p (400 MHz, $CDCl_3$)

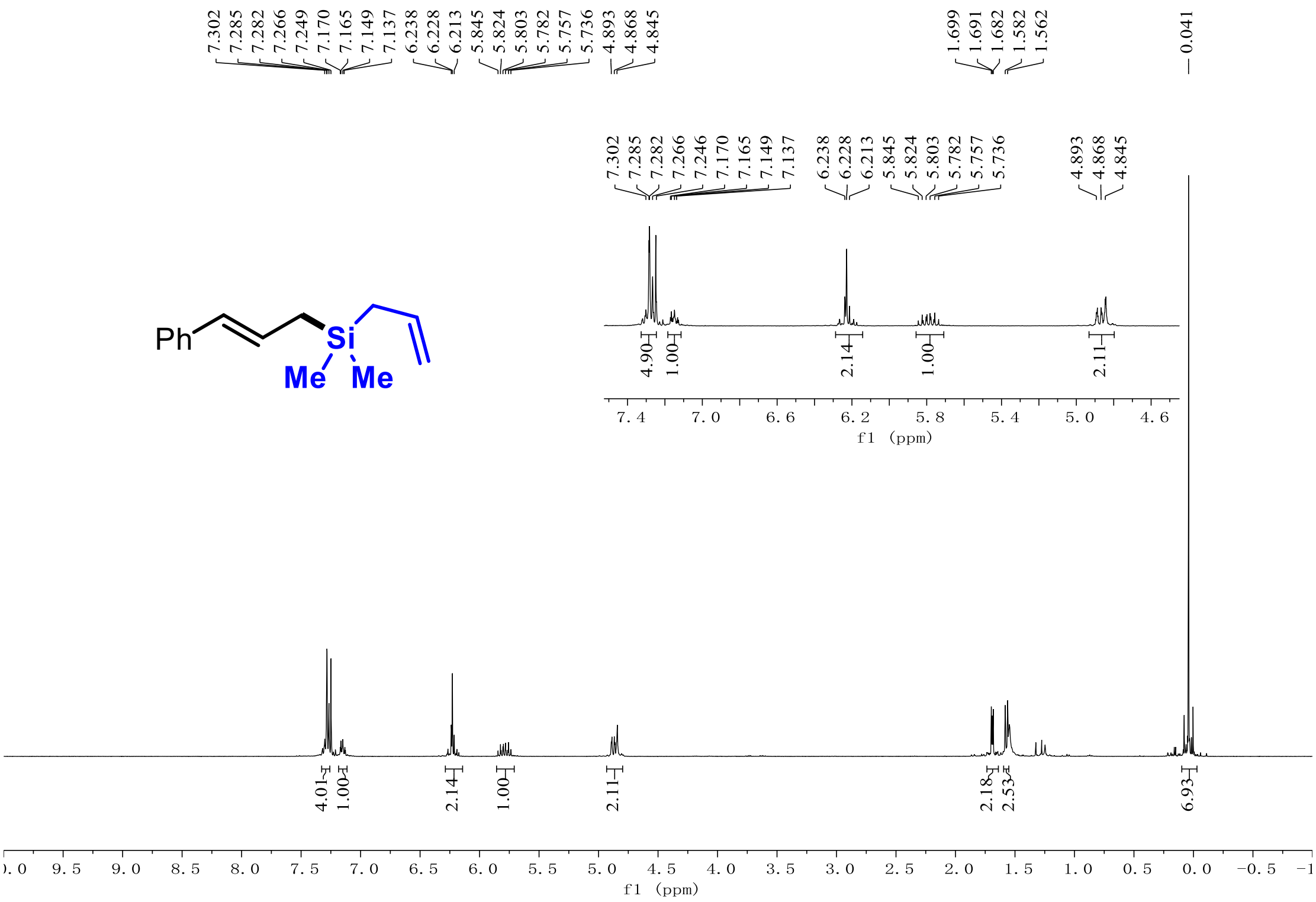

^{13}C NMR spectrum of 5p (101 MHz, $CDCl_3$)

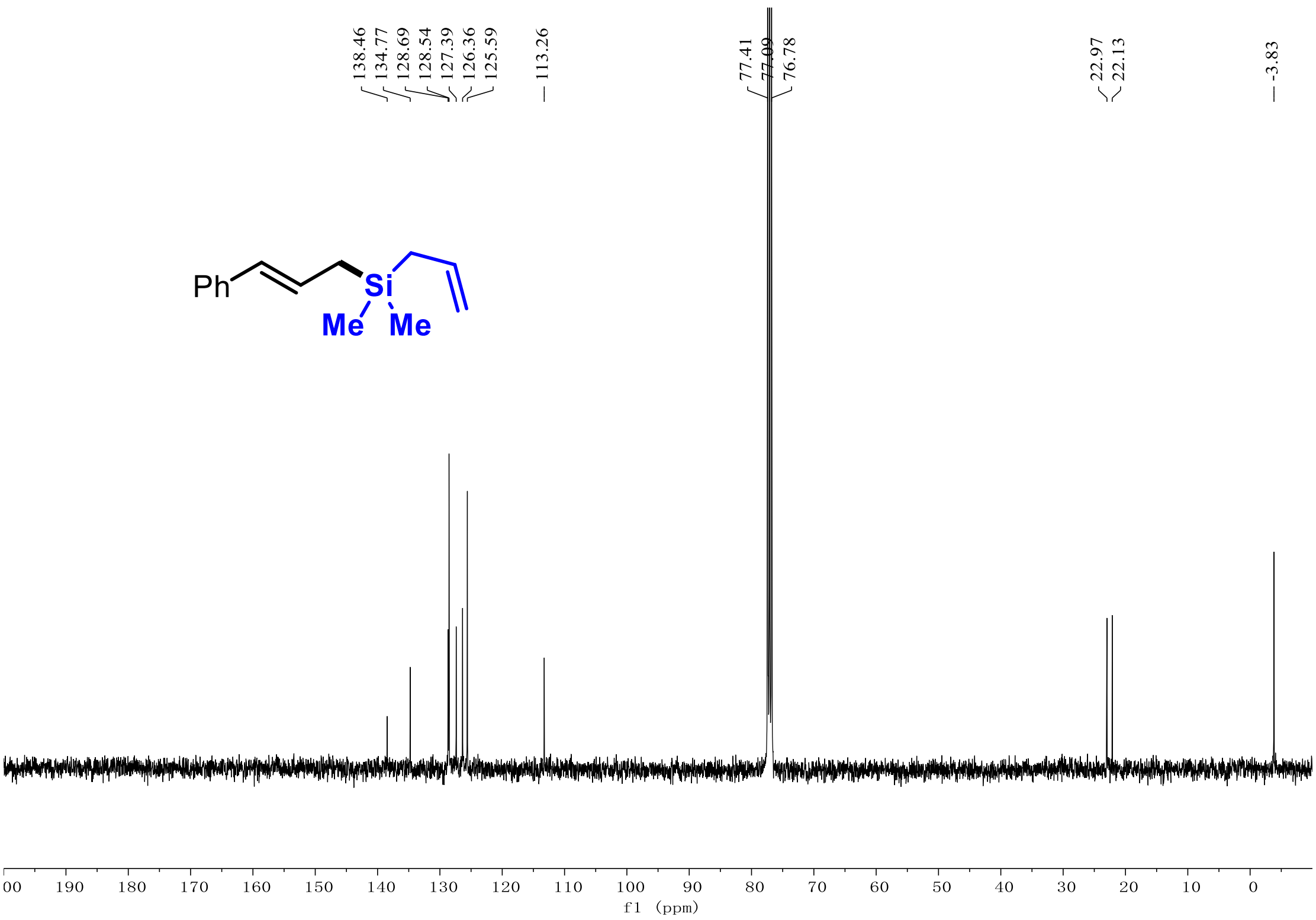

^{1}H NMR spectrum of 5q (400 MHz, $CDCl_3$)

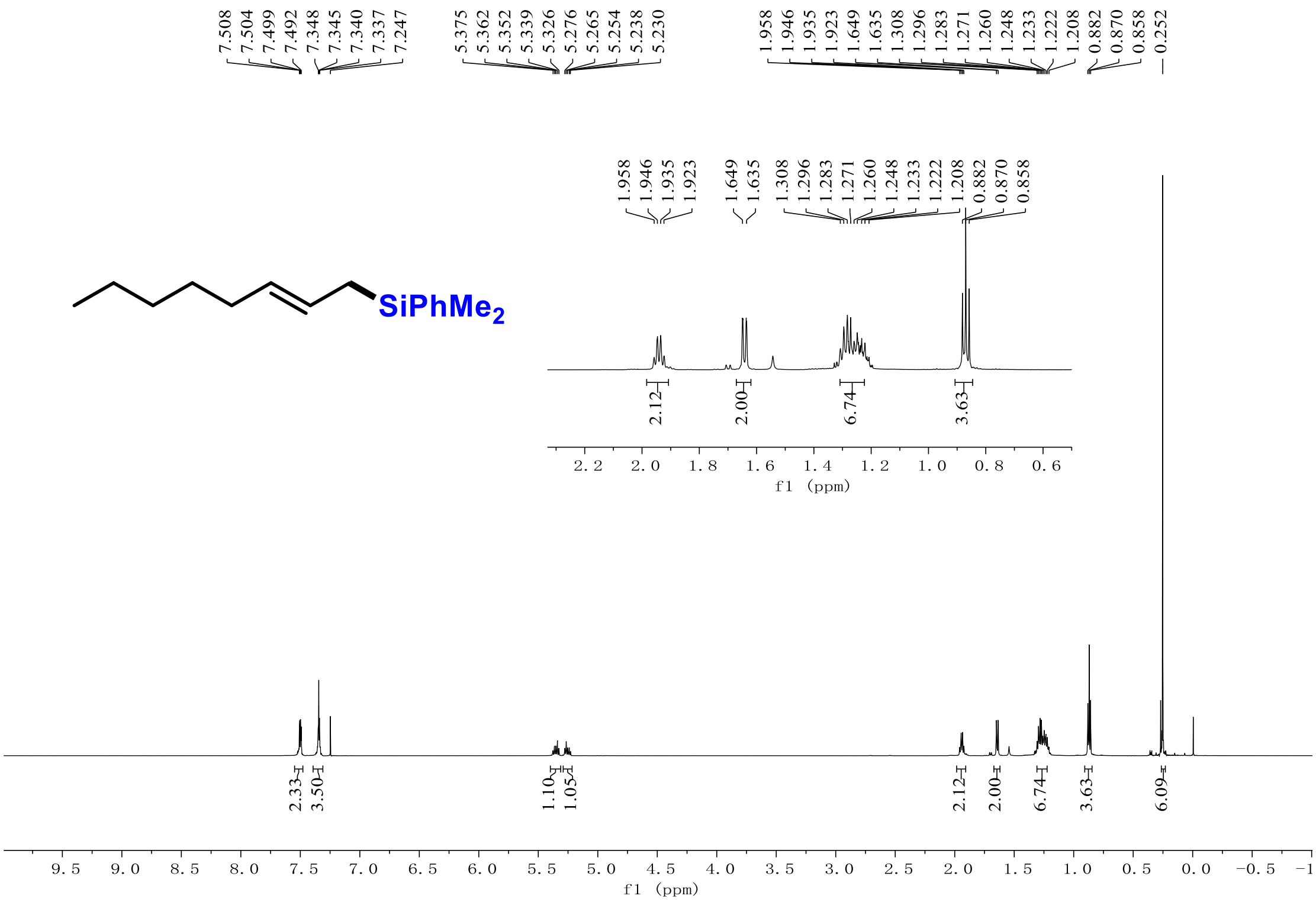

^{13}C NMR spectrum of 5q (101 MHz, $CDCl_3$)

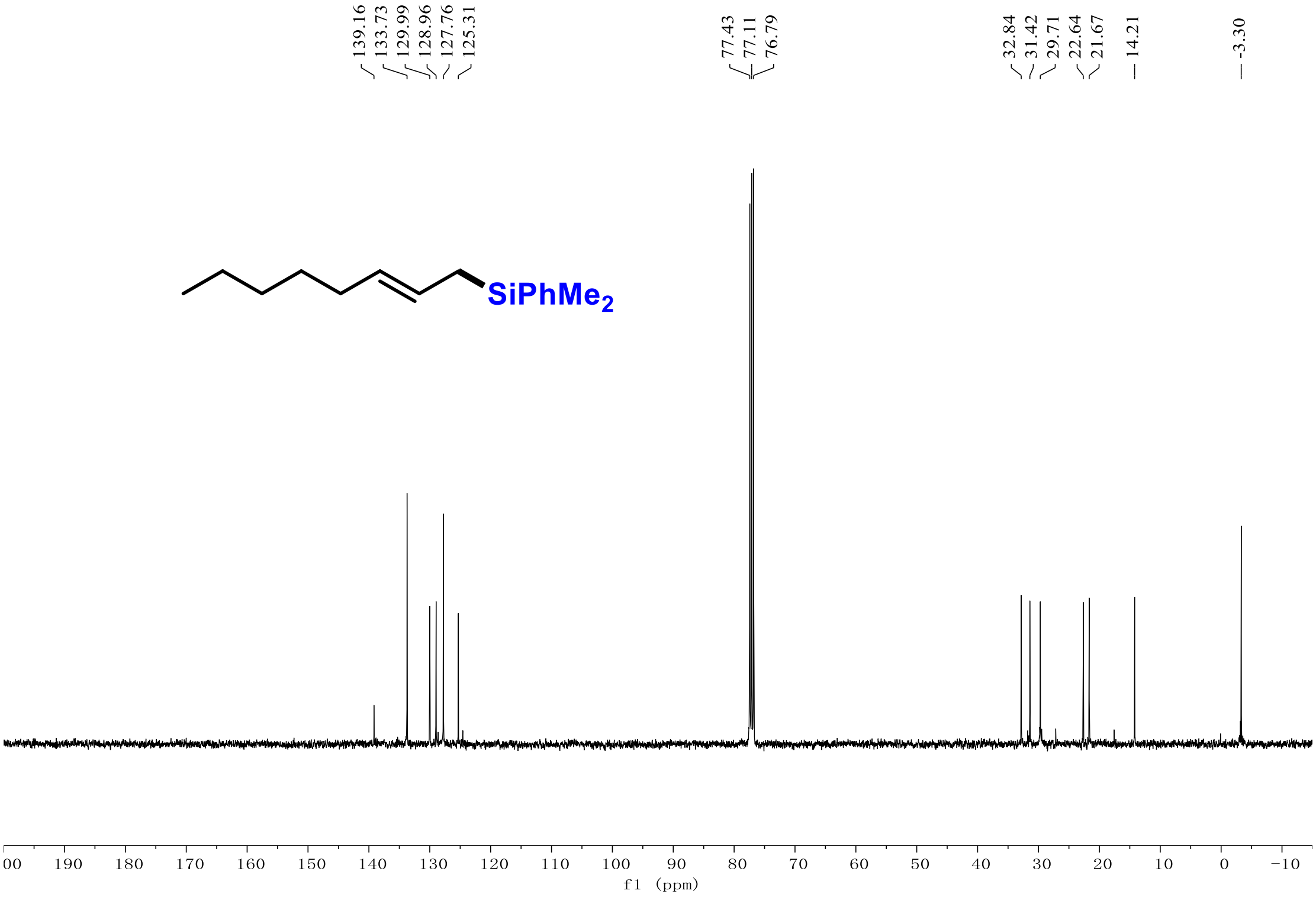

^{1}H NMR spectrum of 5r (400 MHz, $CDCl_3$)

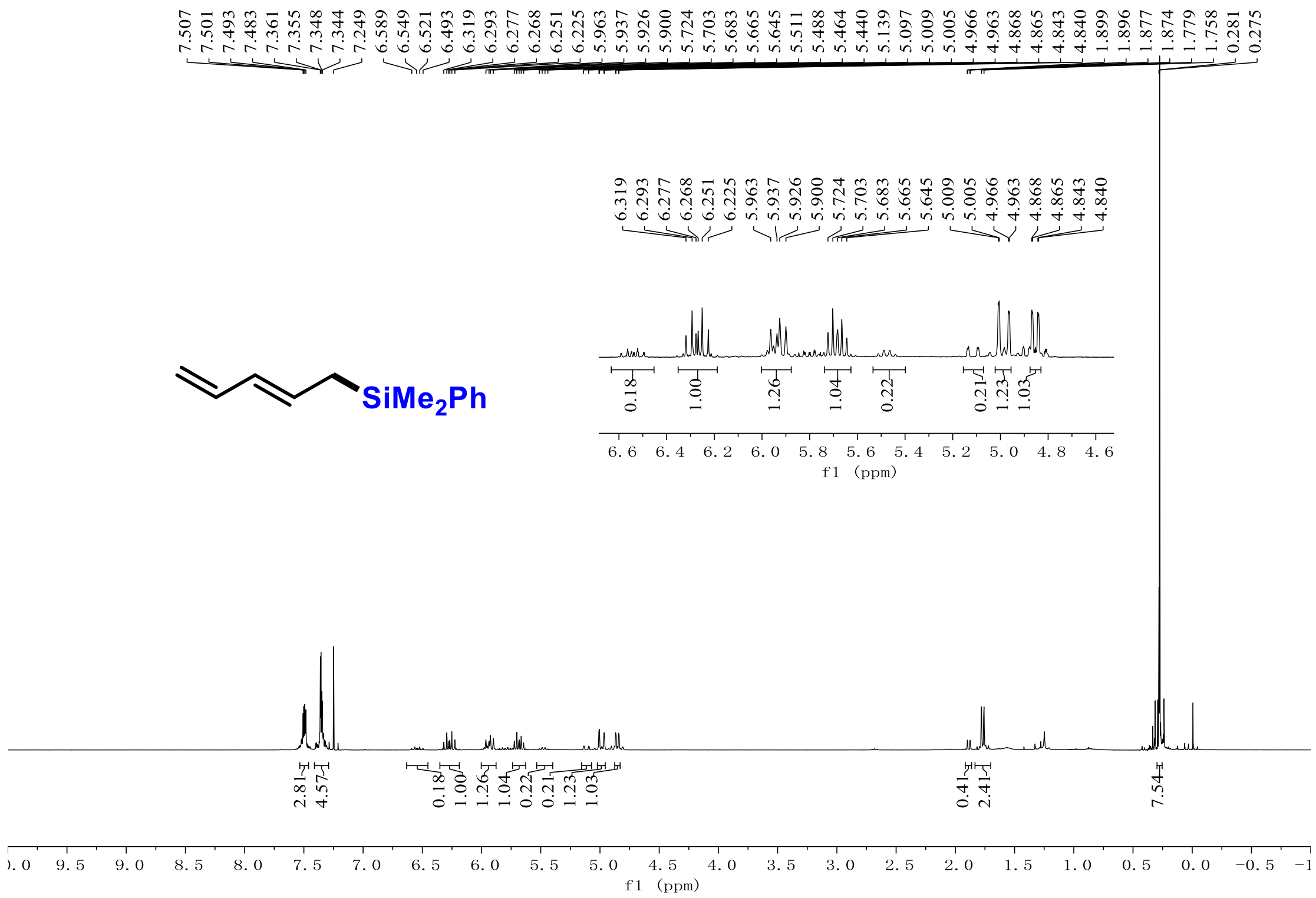

^{13}C NMR spectrum of 5r (101 MHz, $CDCl_3$)

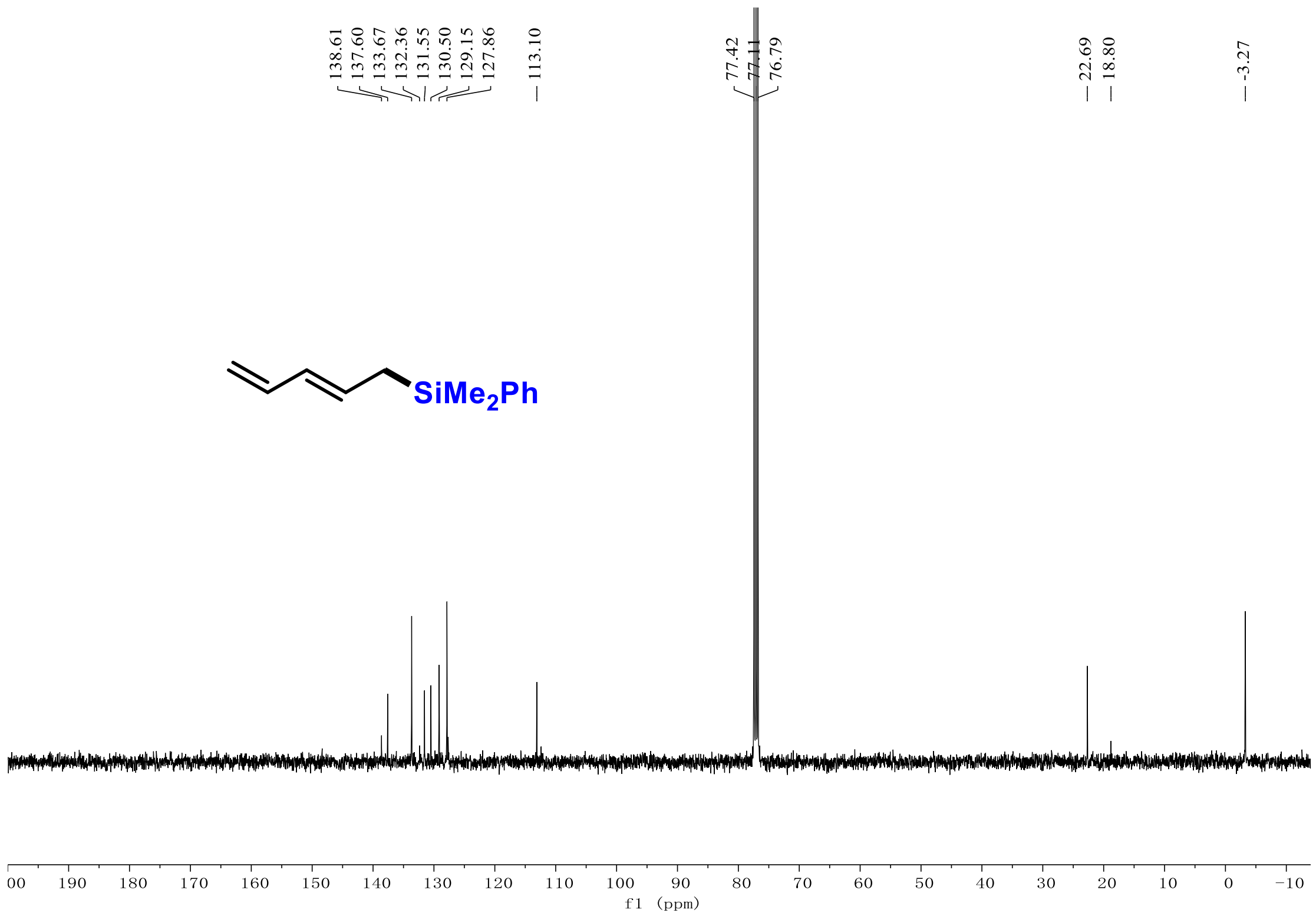

^{1}H NMR spectrum of 5s (400 MHz, $CDCl_3$)

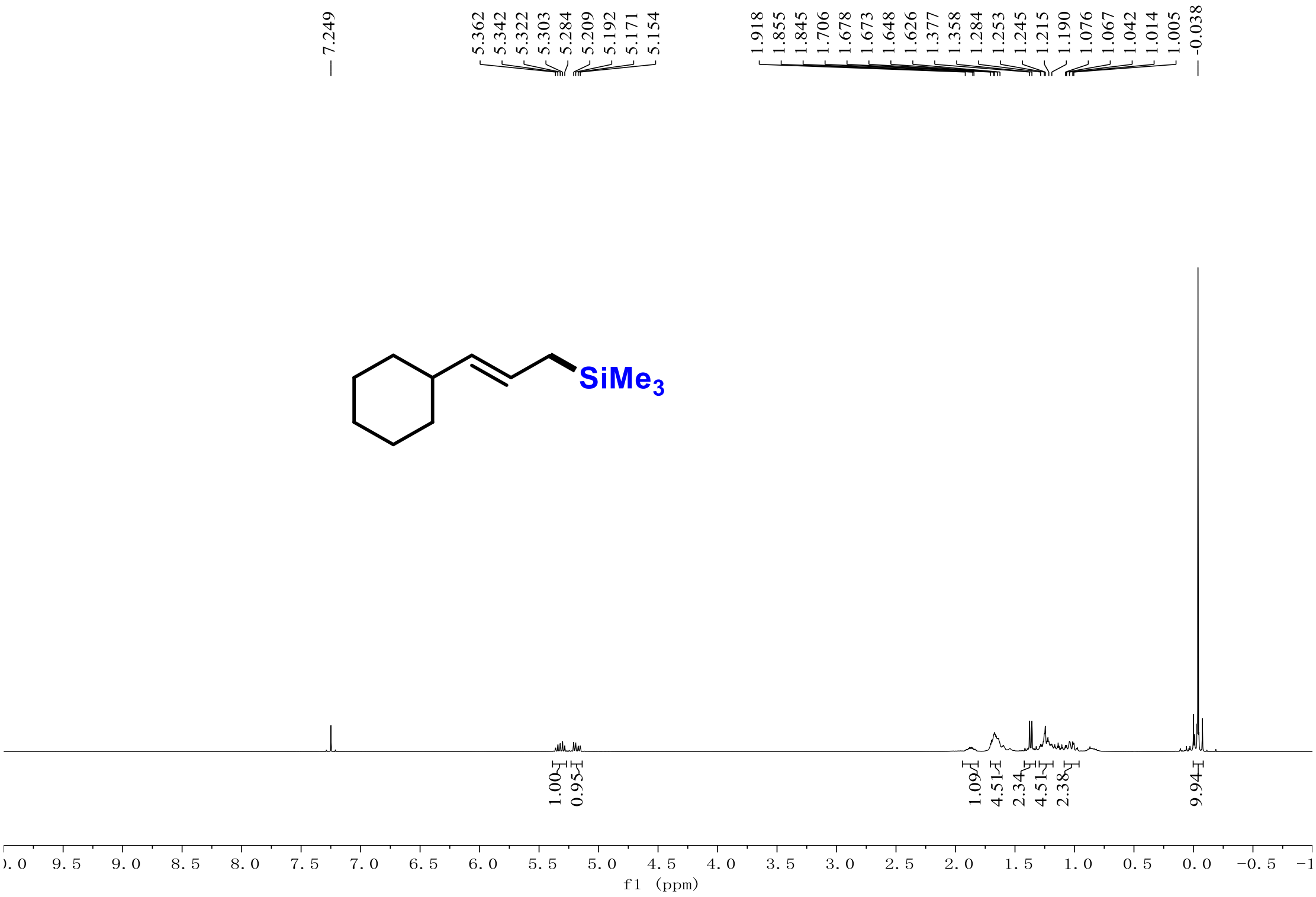

^{13}C NMR spectrum of 5s (101 MHz, $CDCl_3$)

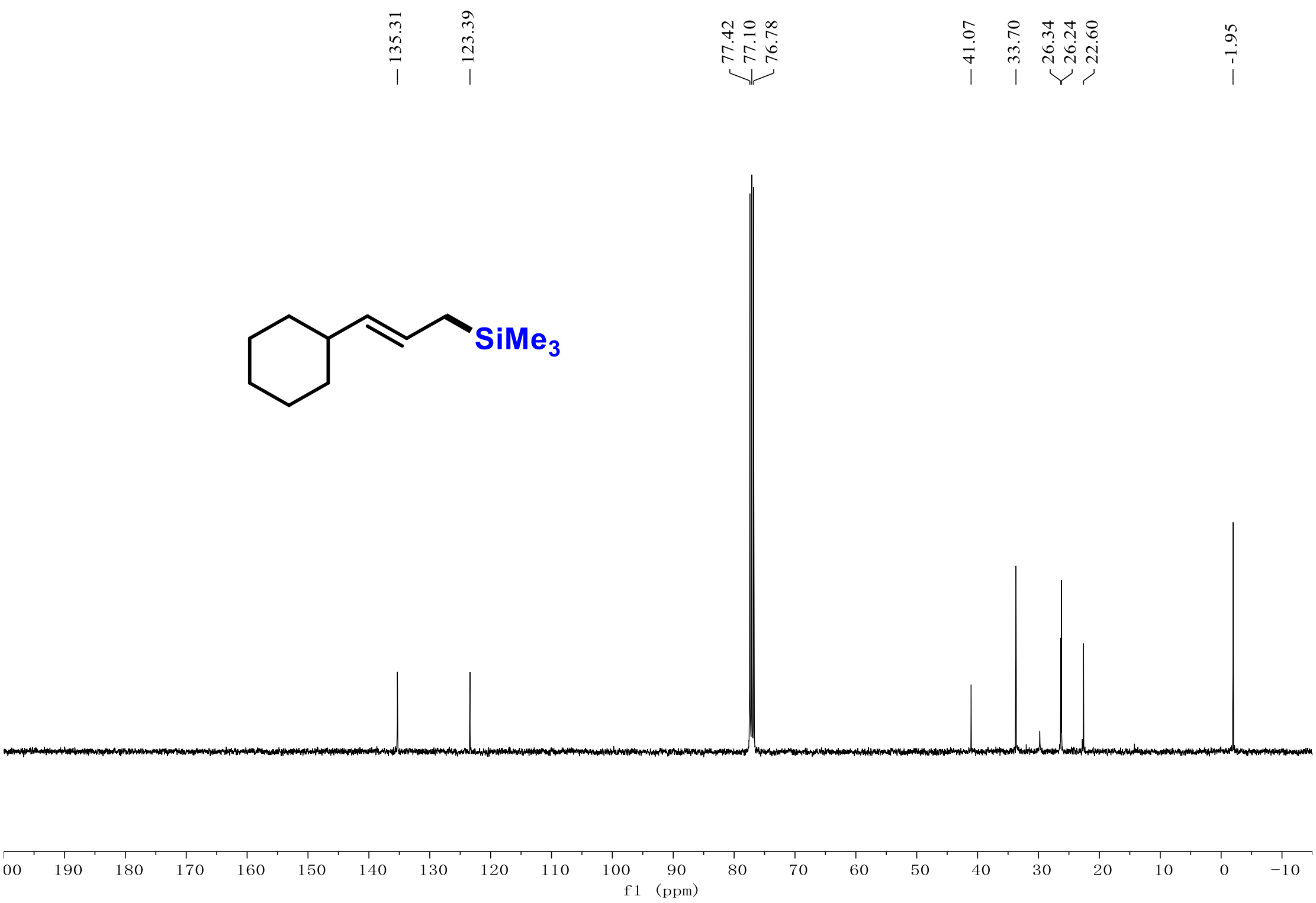

^{1}H NMR spectrum of 5t (400 MHz, $CDCl_3$)

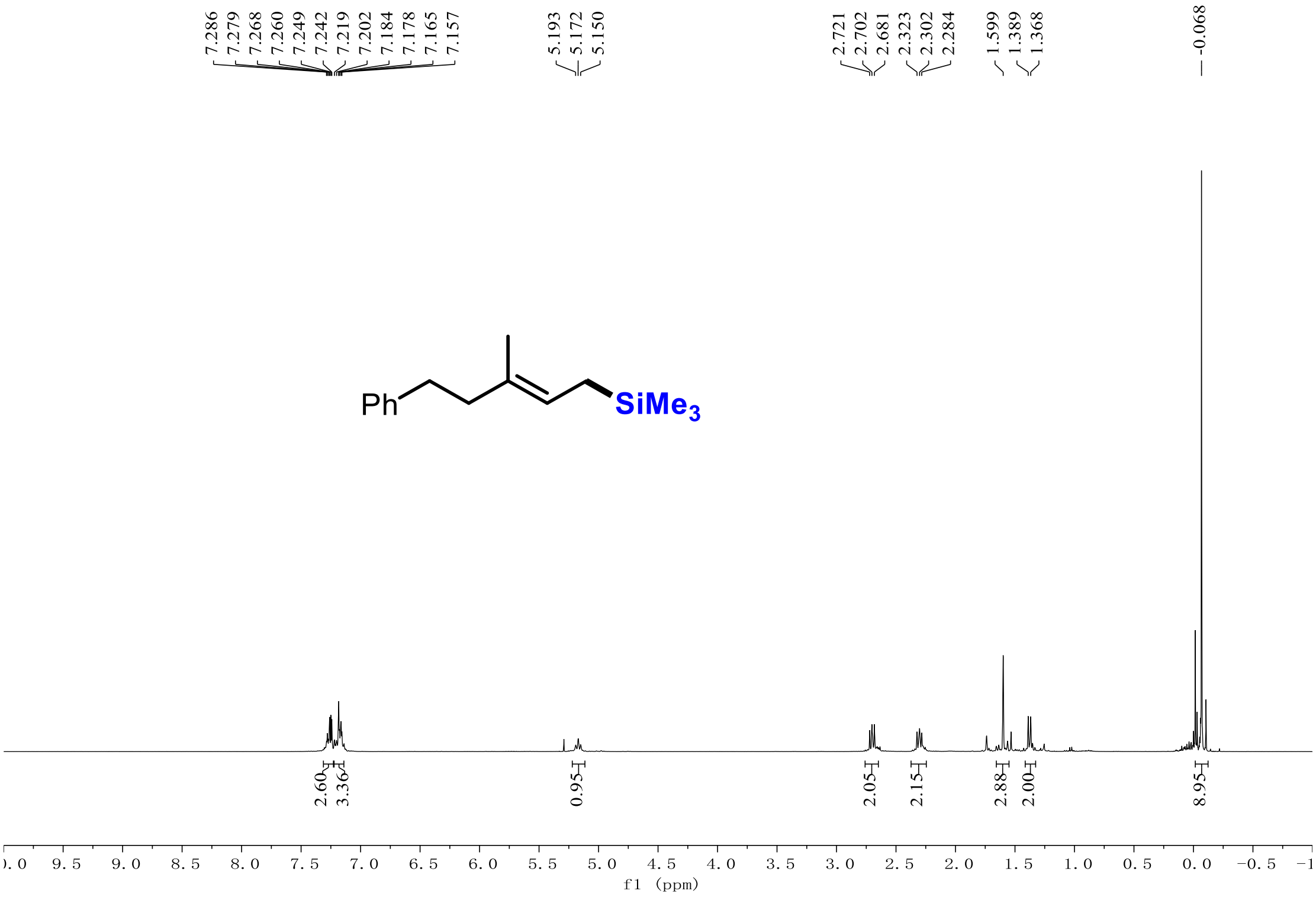

^{13}C NMR spectrum of 5t (101 MHz, $CDCl_3$)

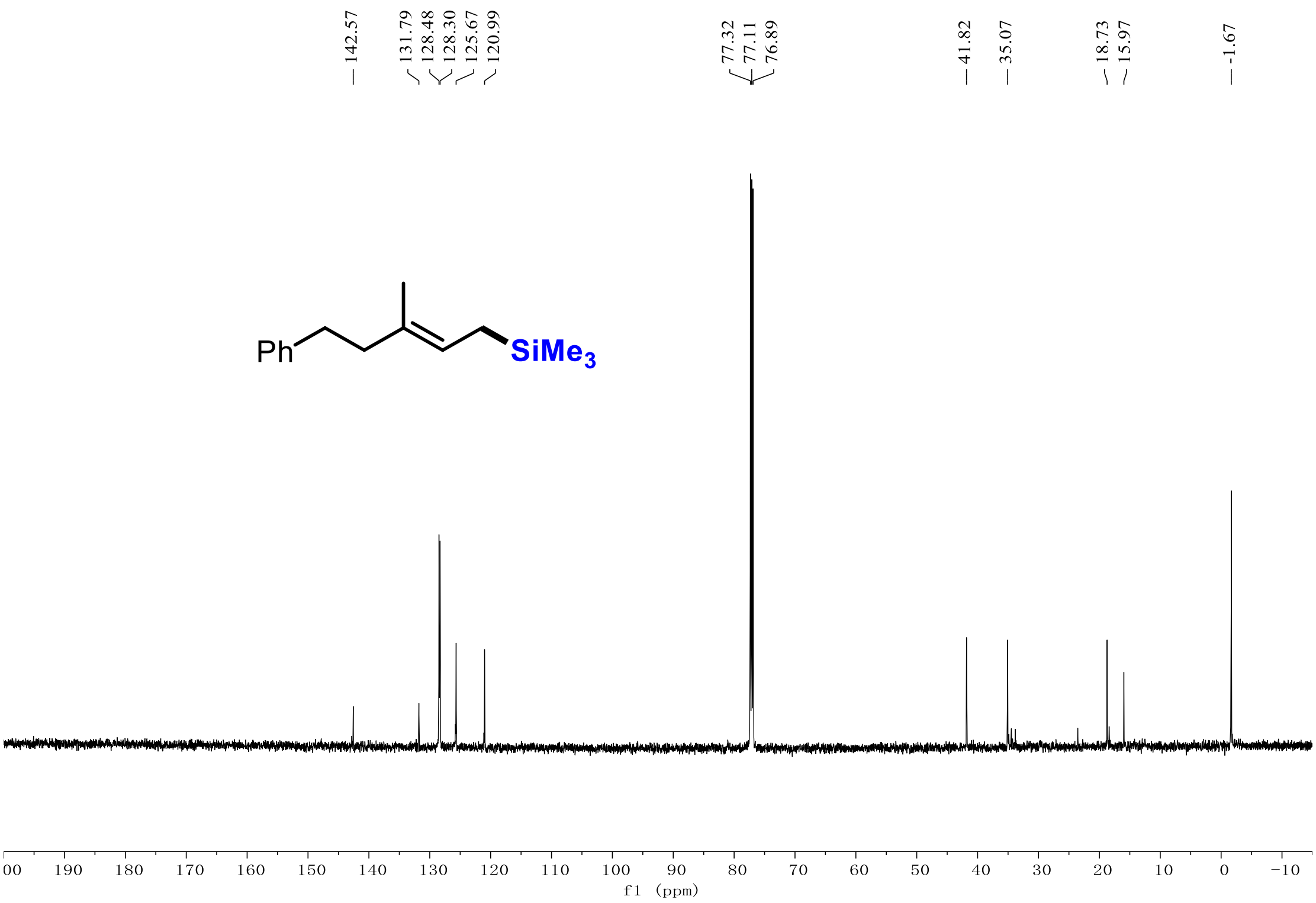

^{1}H NMR spectrum of 5u (400 MHz, $CDCl_3$)

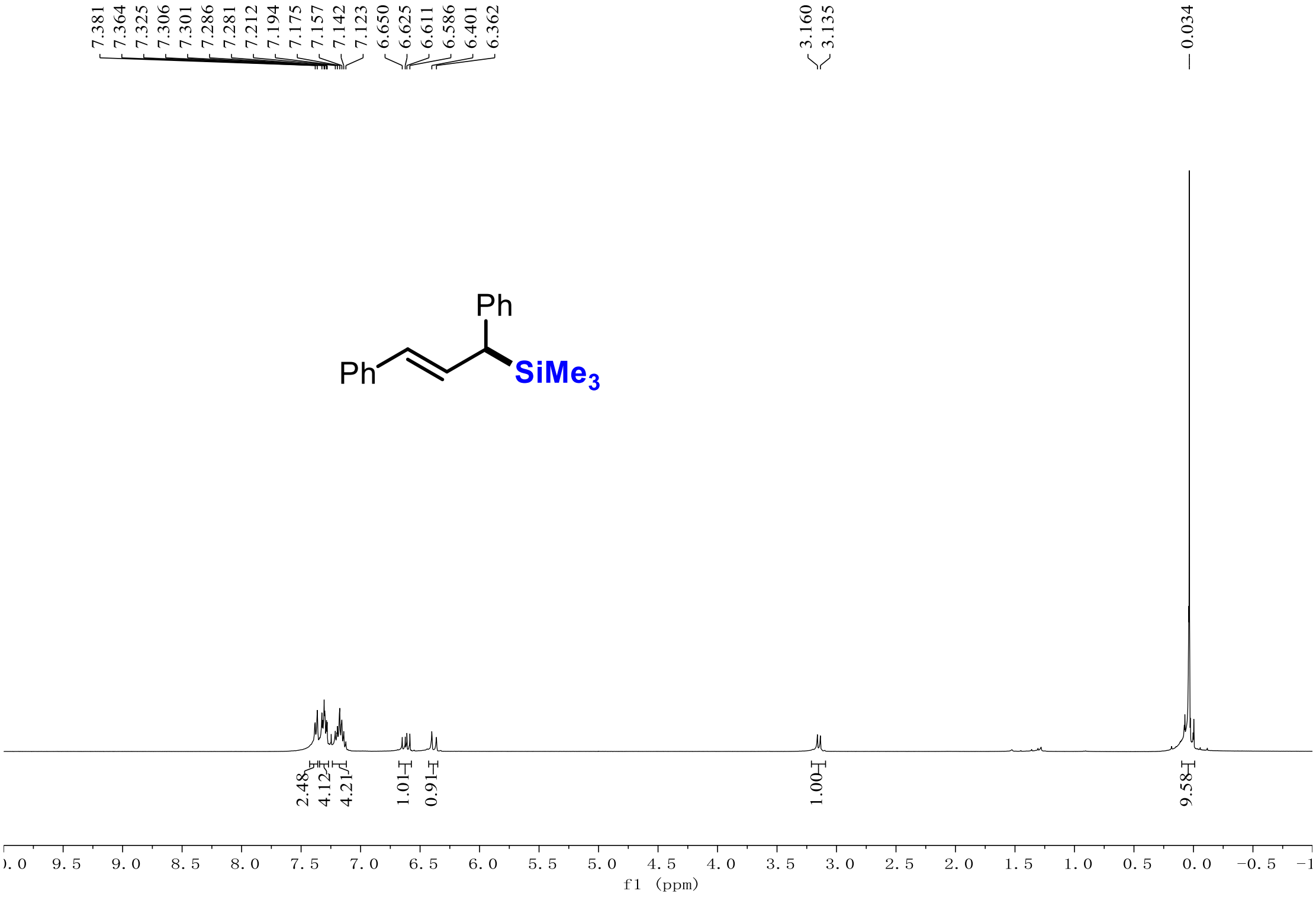

^{13}C NMR spectrum of 5u (101 MHz, $CDCl_3$)

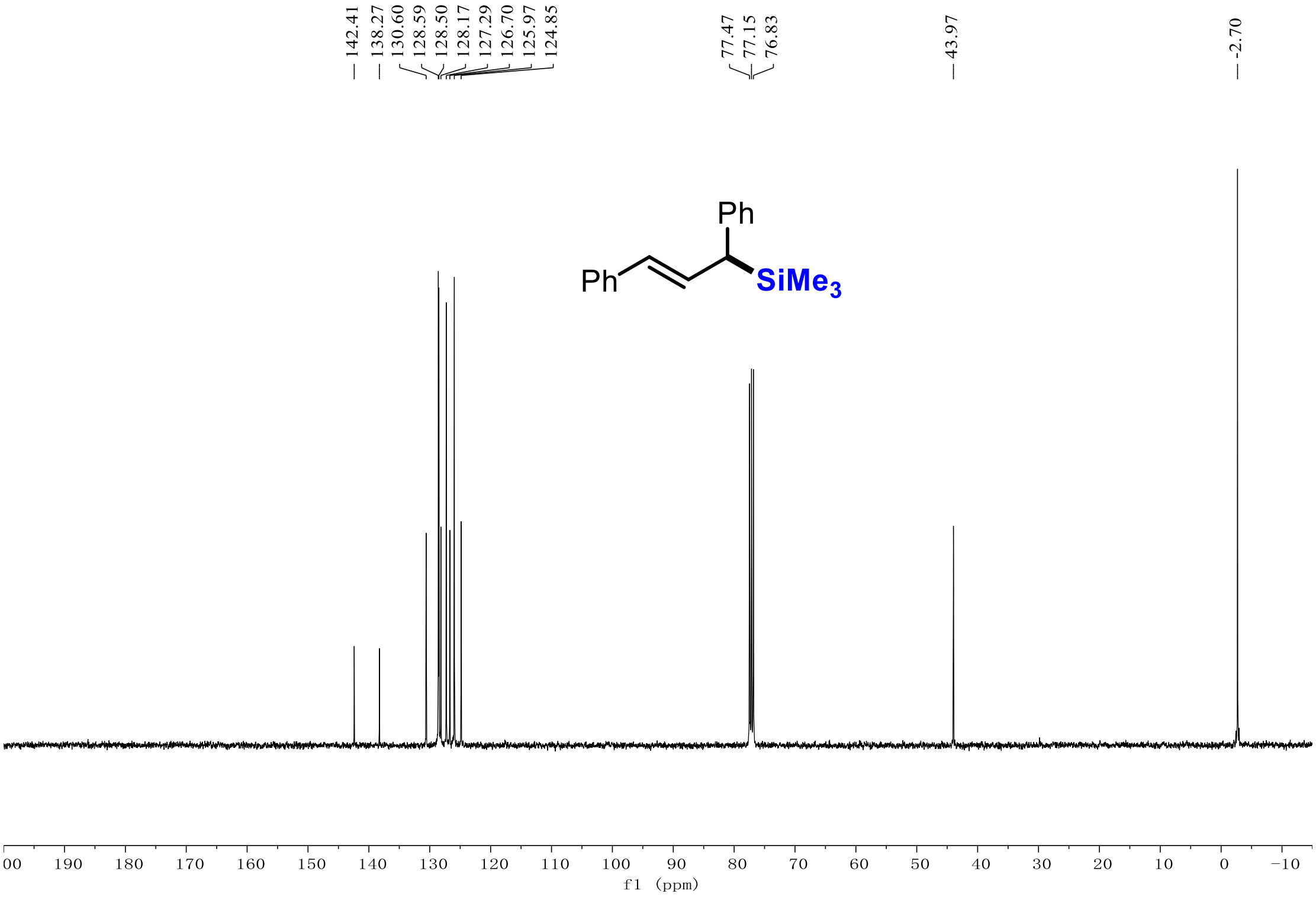

^{1}H NMR spectrum of 5v (400 MHz, $CDCl_3$)

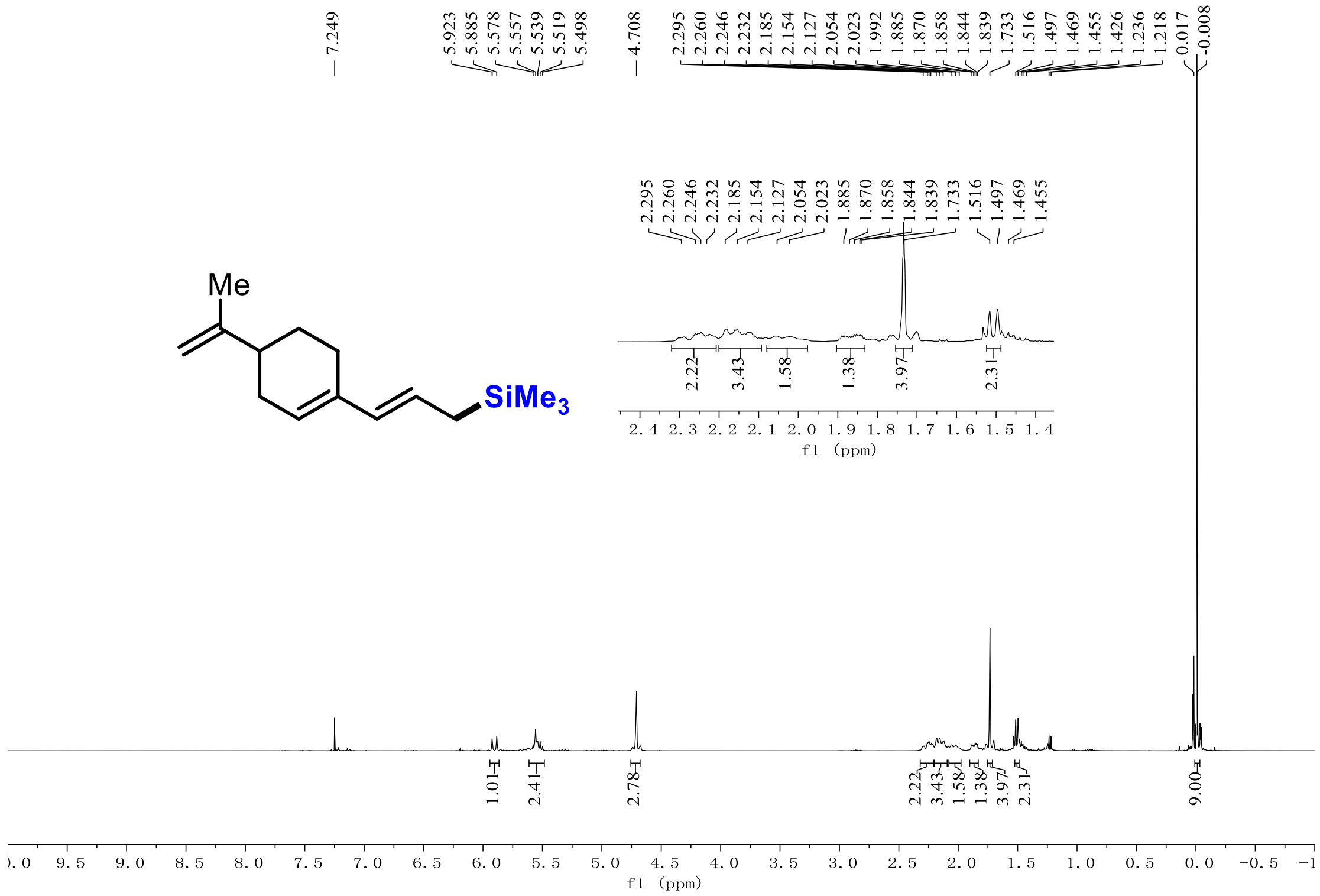

^{13}C NMR spectrum of 5v (101 MHz, $CDCl_3$)

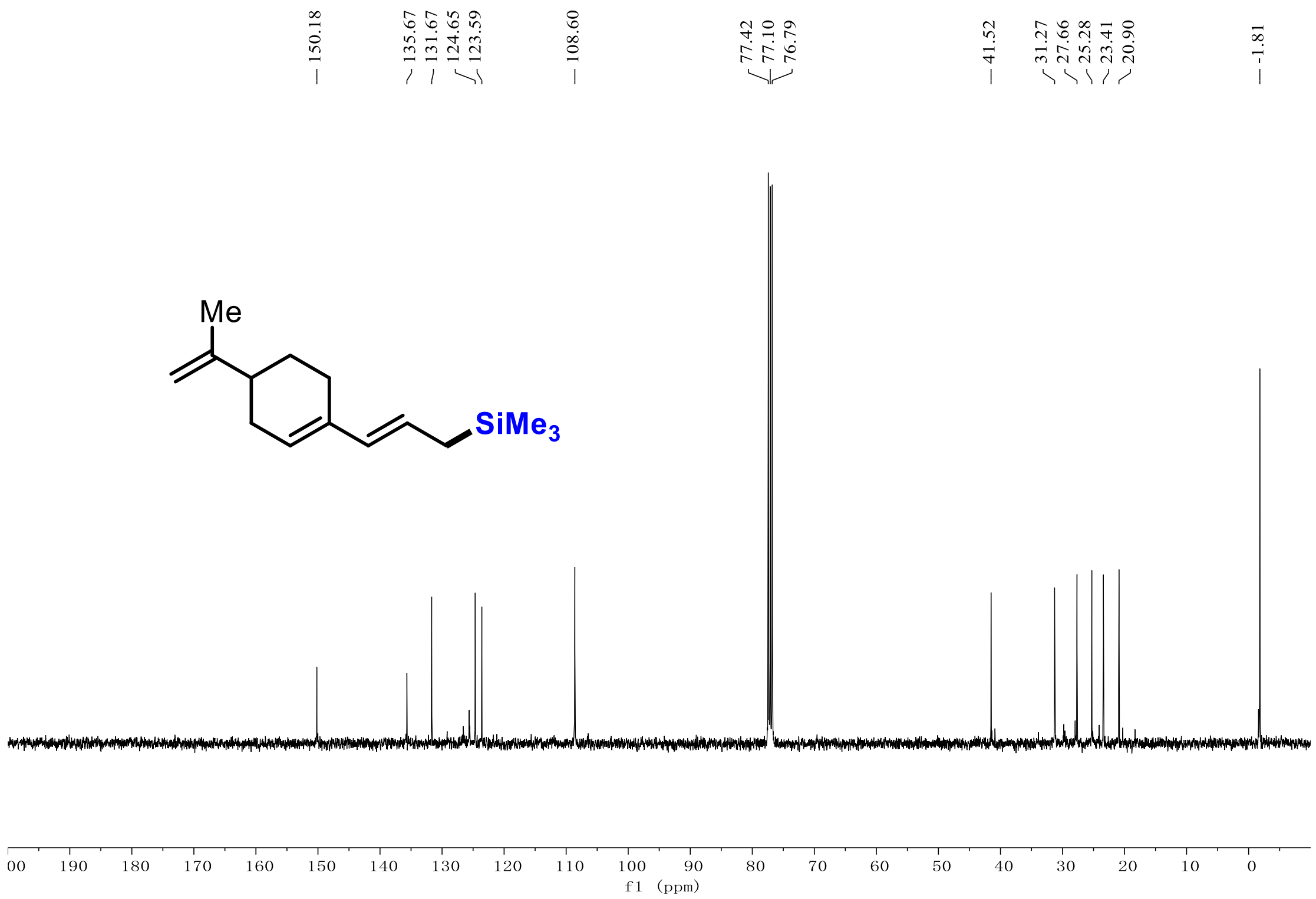

^{1}H NMR spectrum of 5w (400 MHz, $CDCl_3$)

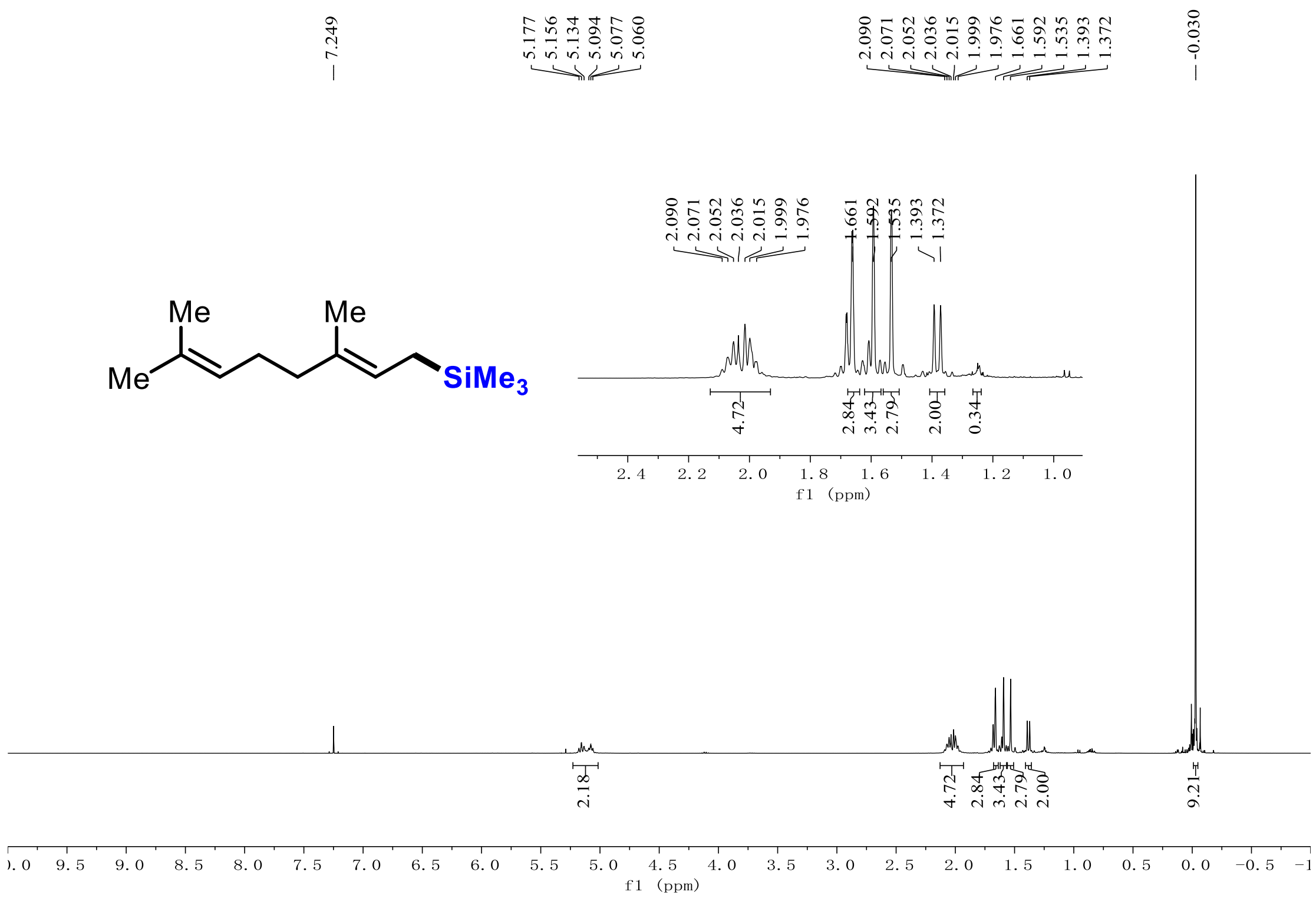

^{13}C NMR spectrum of 5w (101 MHz, $CDCl_3$)

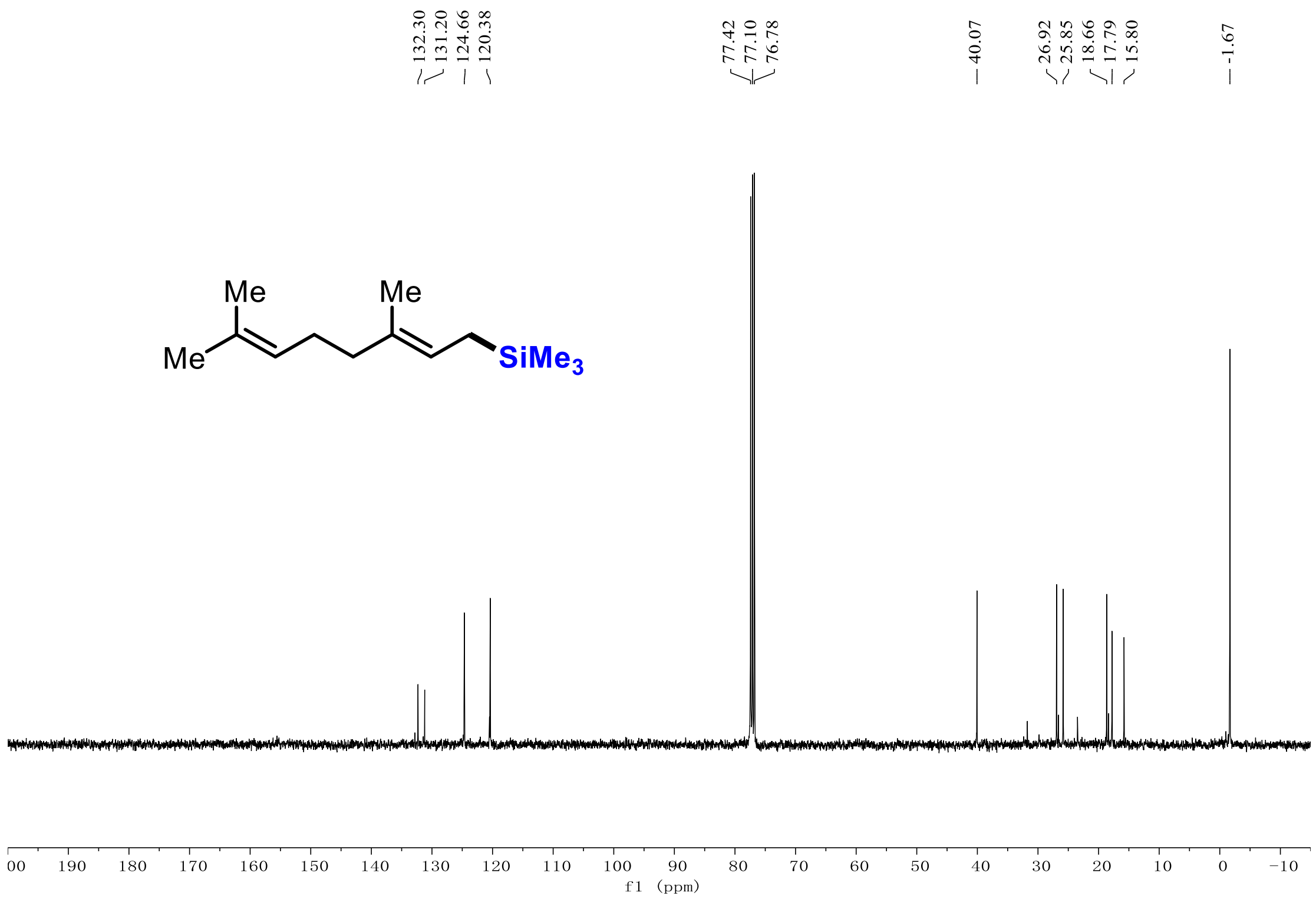

^{1}H NMR spectrum of 5x (400 MHz, $CDCl_3$)

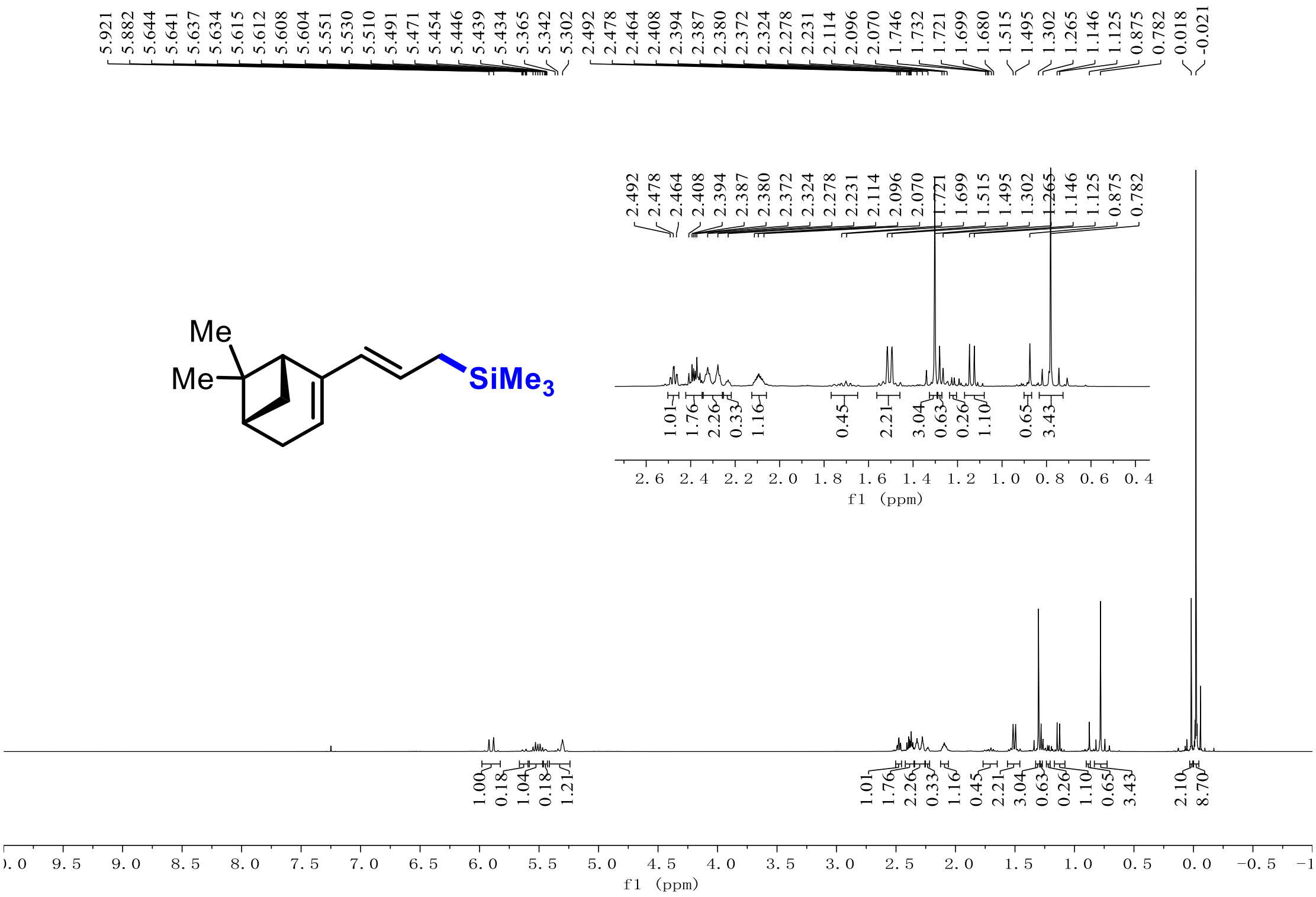

^{13}C NMR spectrum of 5x (101 MHz, $CDCl_3$)

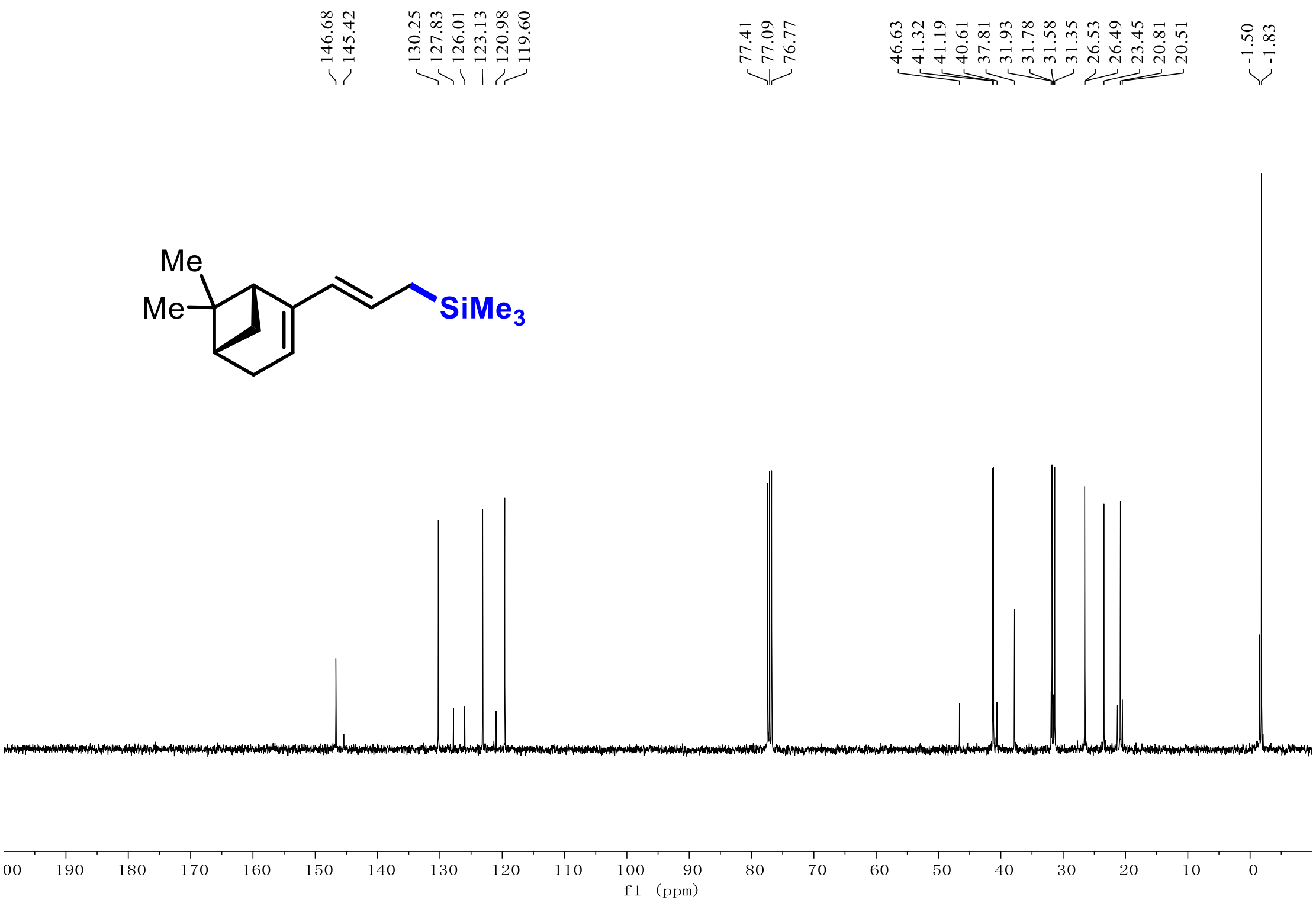

^{1}H NMR spectrum of 5y (400 MHz, $CDCl_3$)

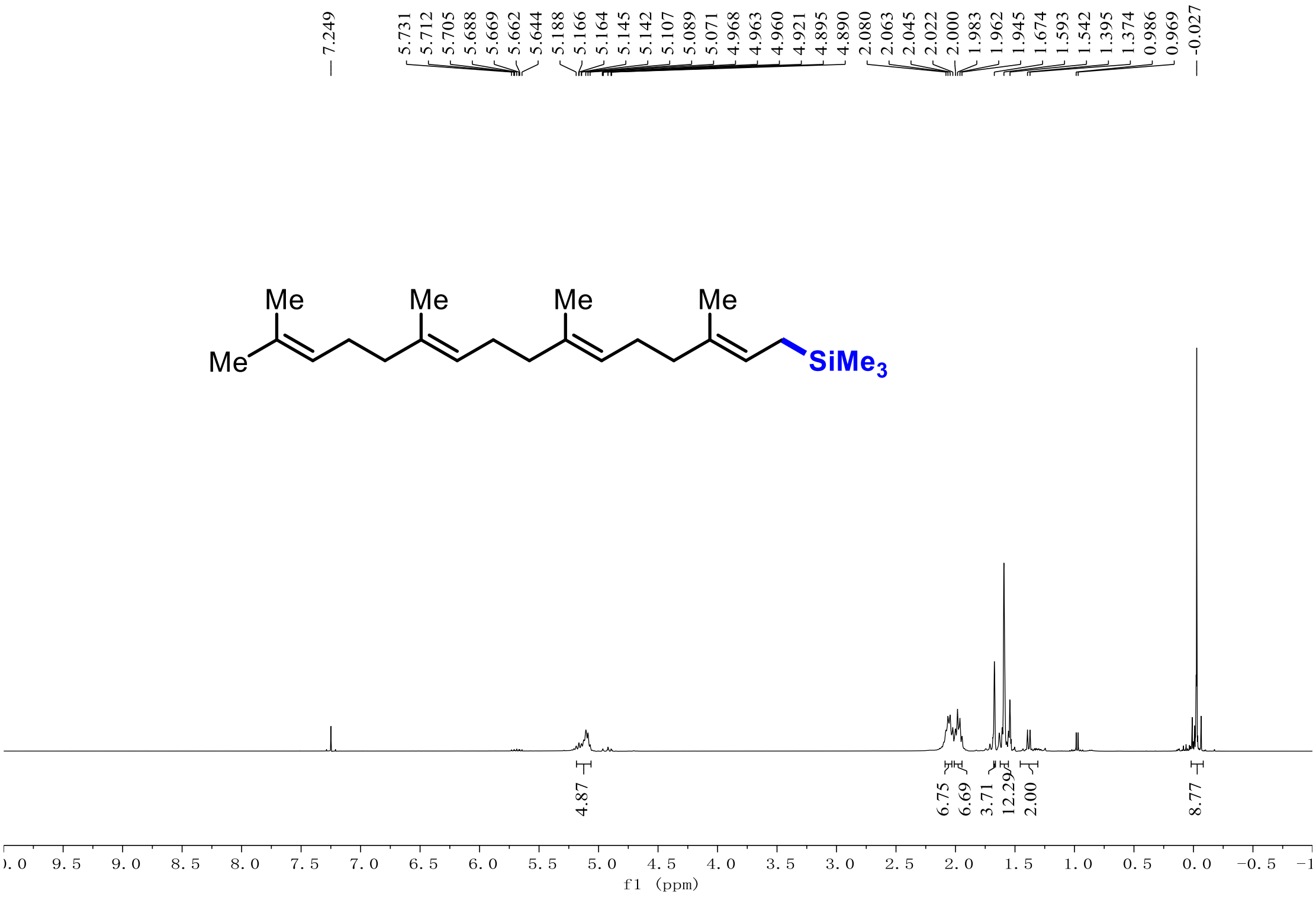

^{13}C NMR spectrum of 5y (101 MHz, $CDCl_3$)

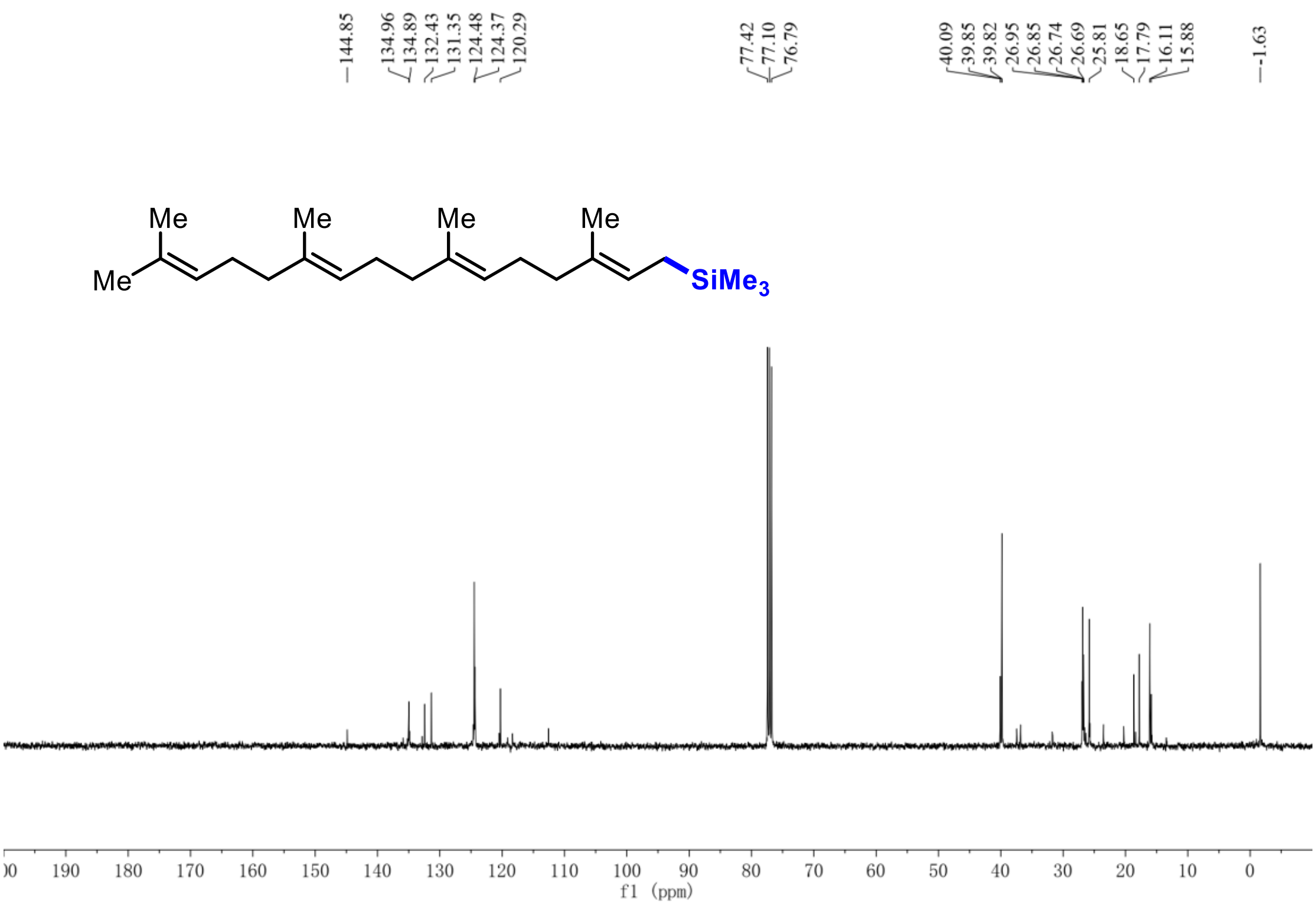

^{1}H NMR spectrum of 5z (400 MHz, $CDCl_3$)

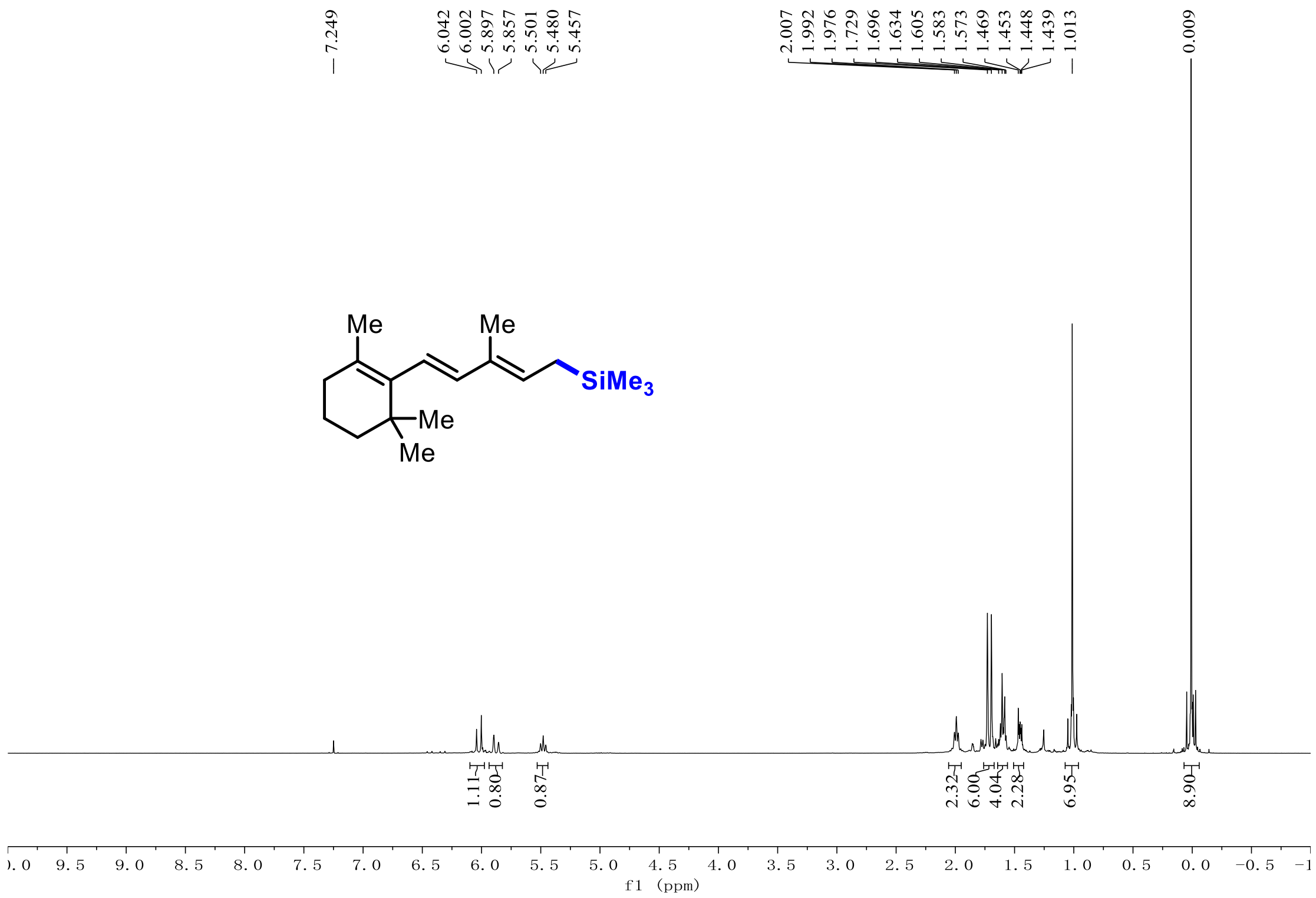

^{13}C NMR spectrum of 5z (101 MHz, $CDCl_3$)

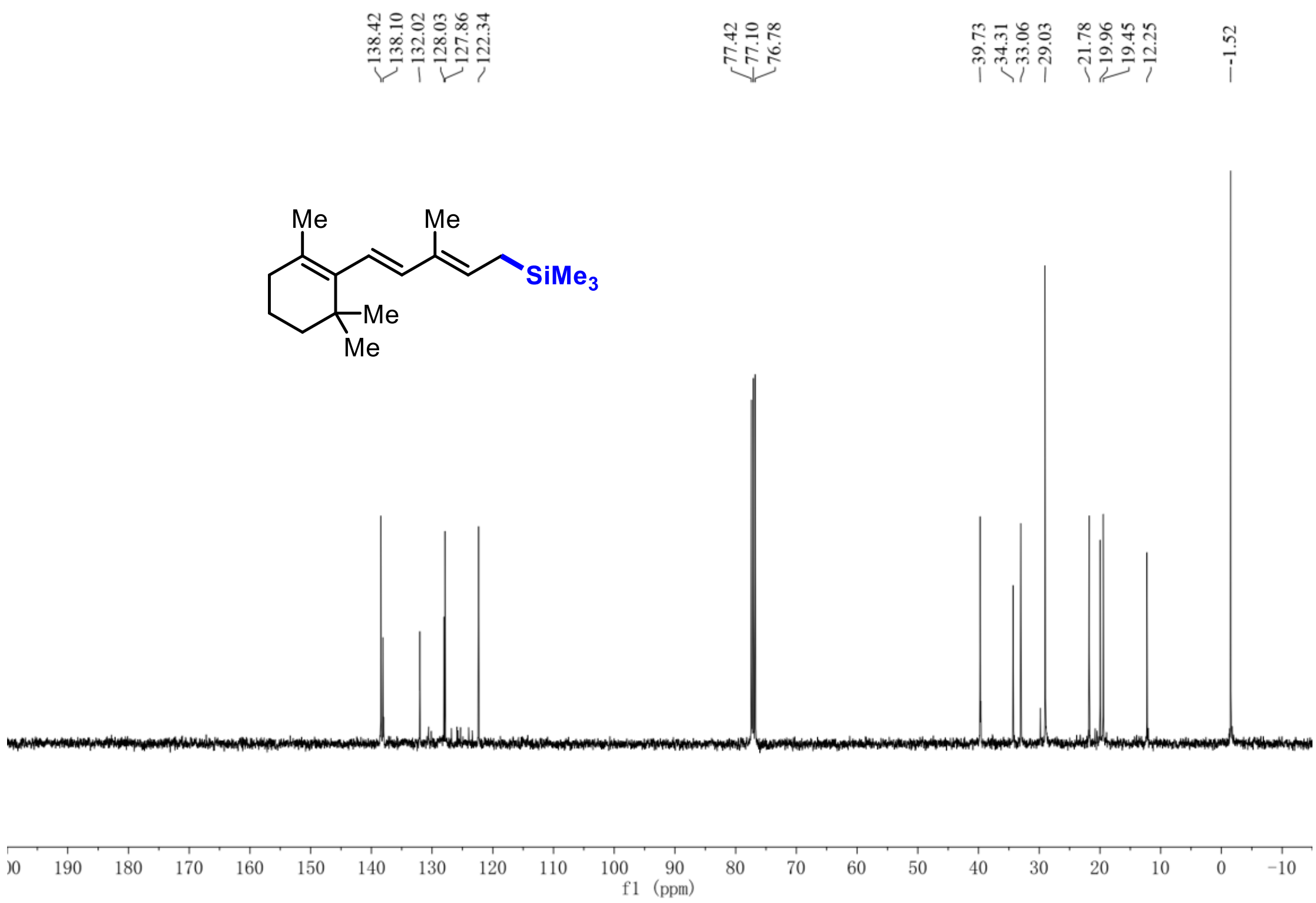

^{1}H NMR spectrum of 5aa (400 MHz, $CDCl_3$)

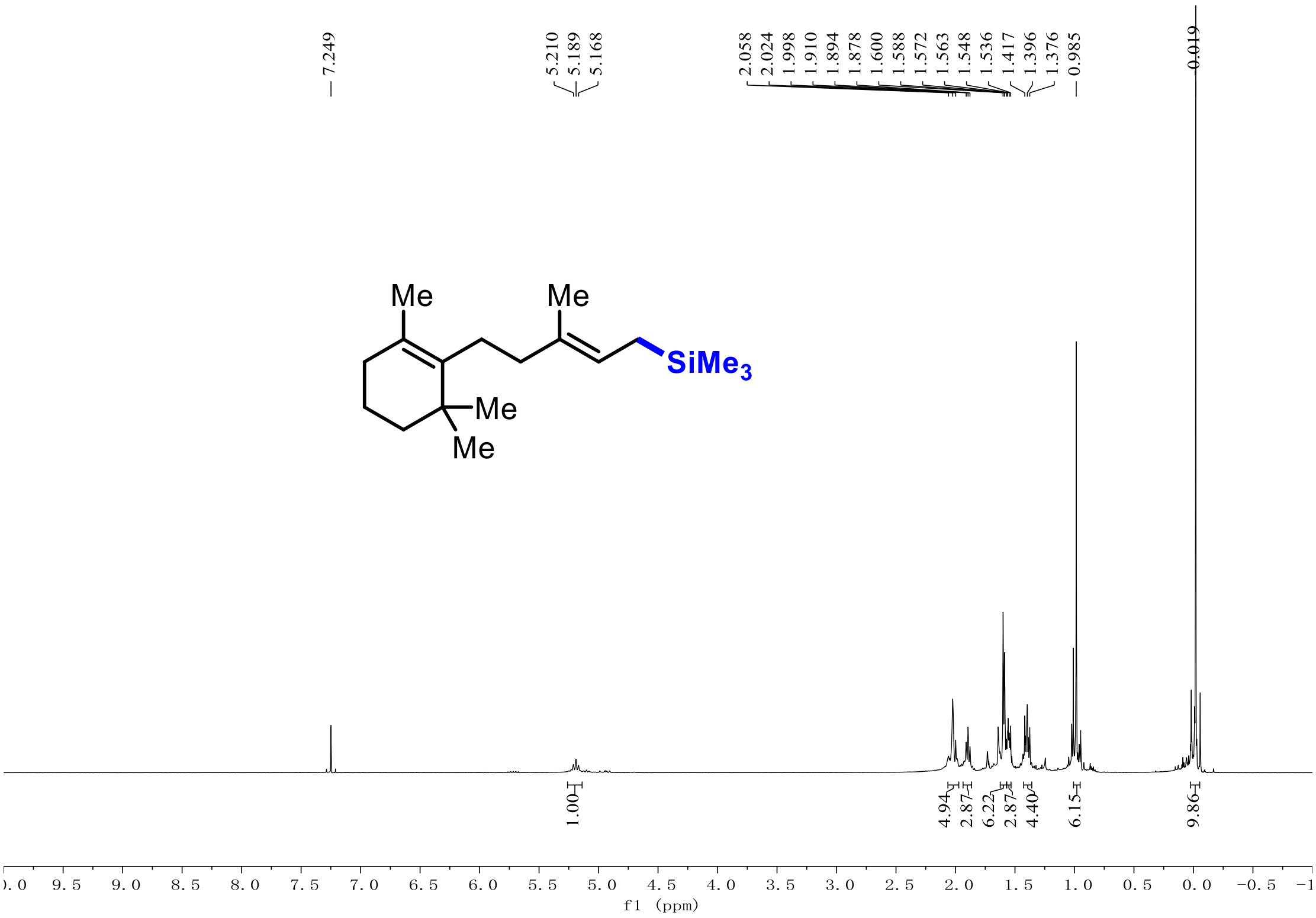

^{13}C NMR spectrum of 5aa (101 MHz, $CDCl_3$)

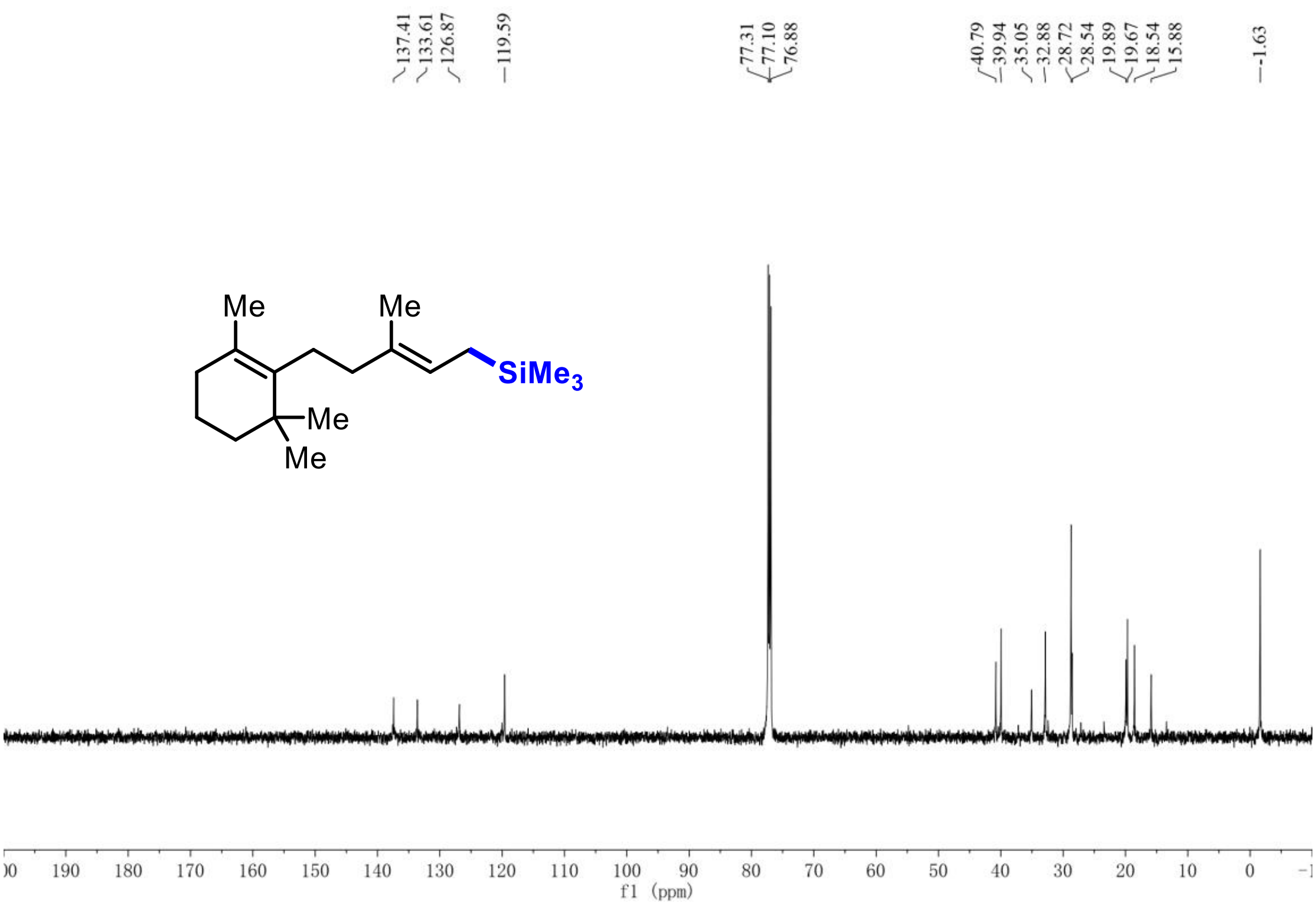

^{1}H NMR spectrum of 5ab (400 MHz, $CDCl_3$)

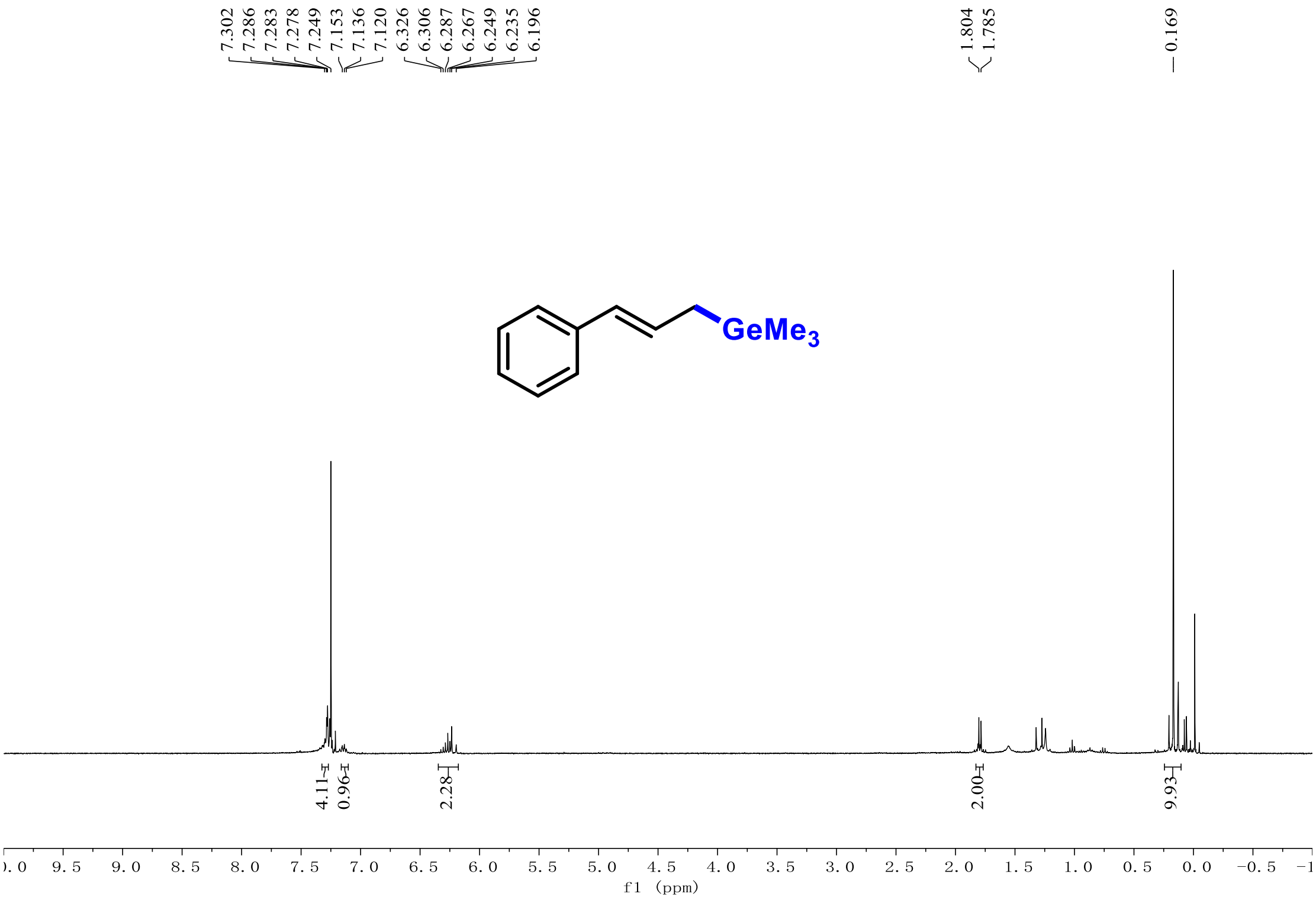

^{13}C NMR spectrum of 5ab (101 MHz, $CDCl_3$)

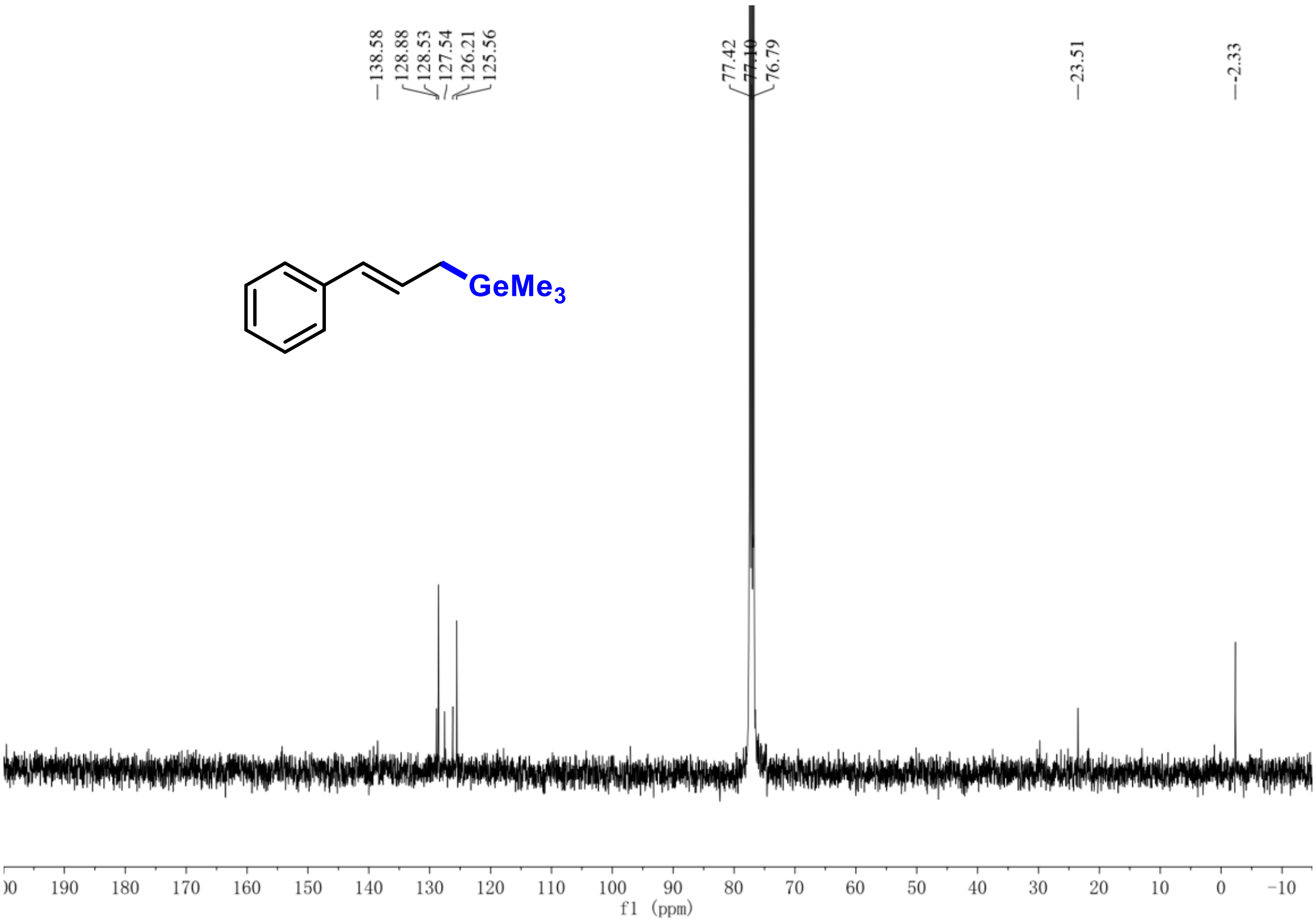

¹H NMR spectrum of 5ac (400 MHz, $CDCl_3$)

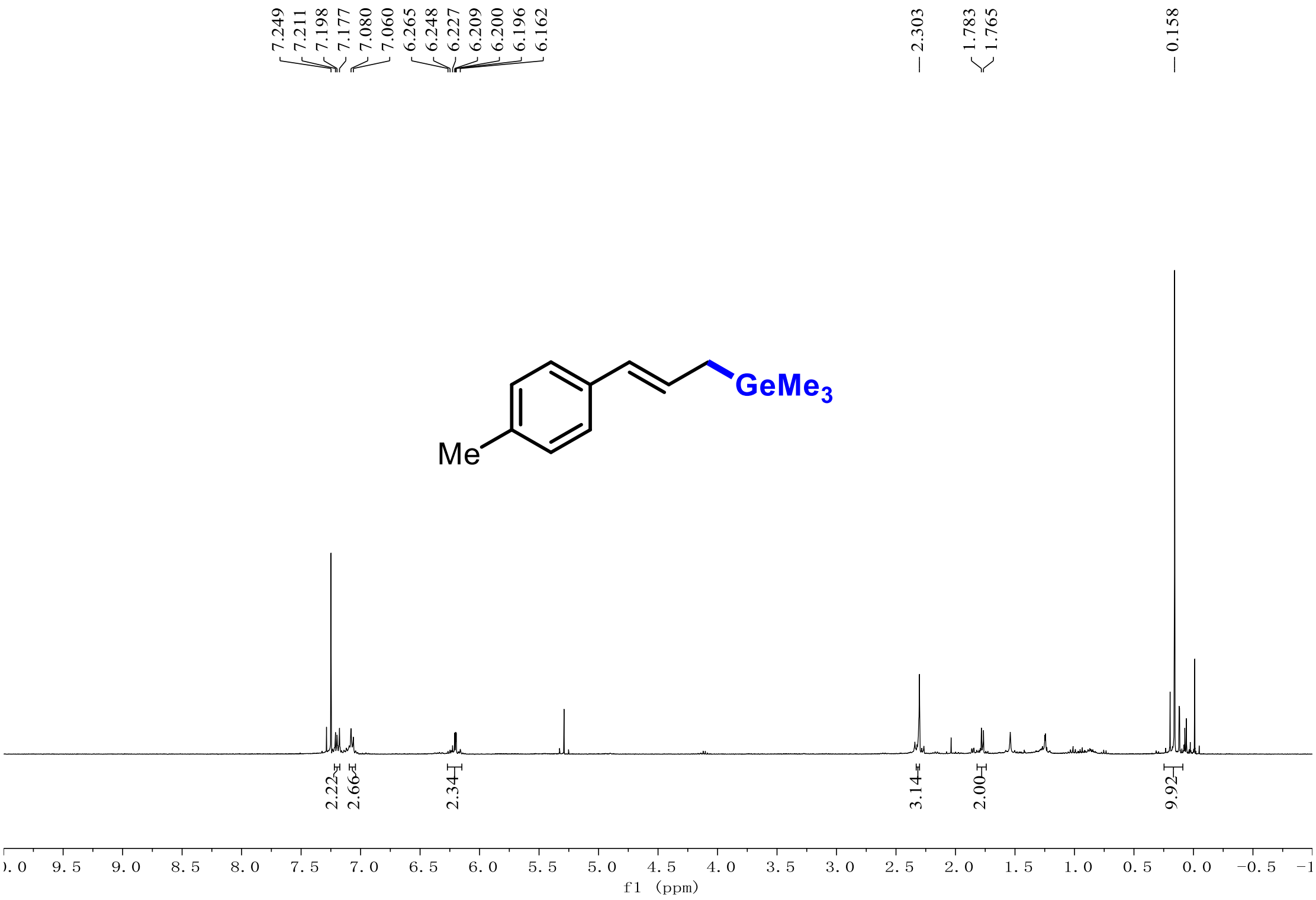

¹³C NMR spectrum of 5ac (101 MHz, $CDCl_3$)

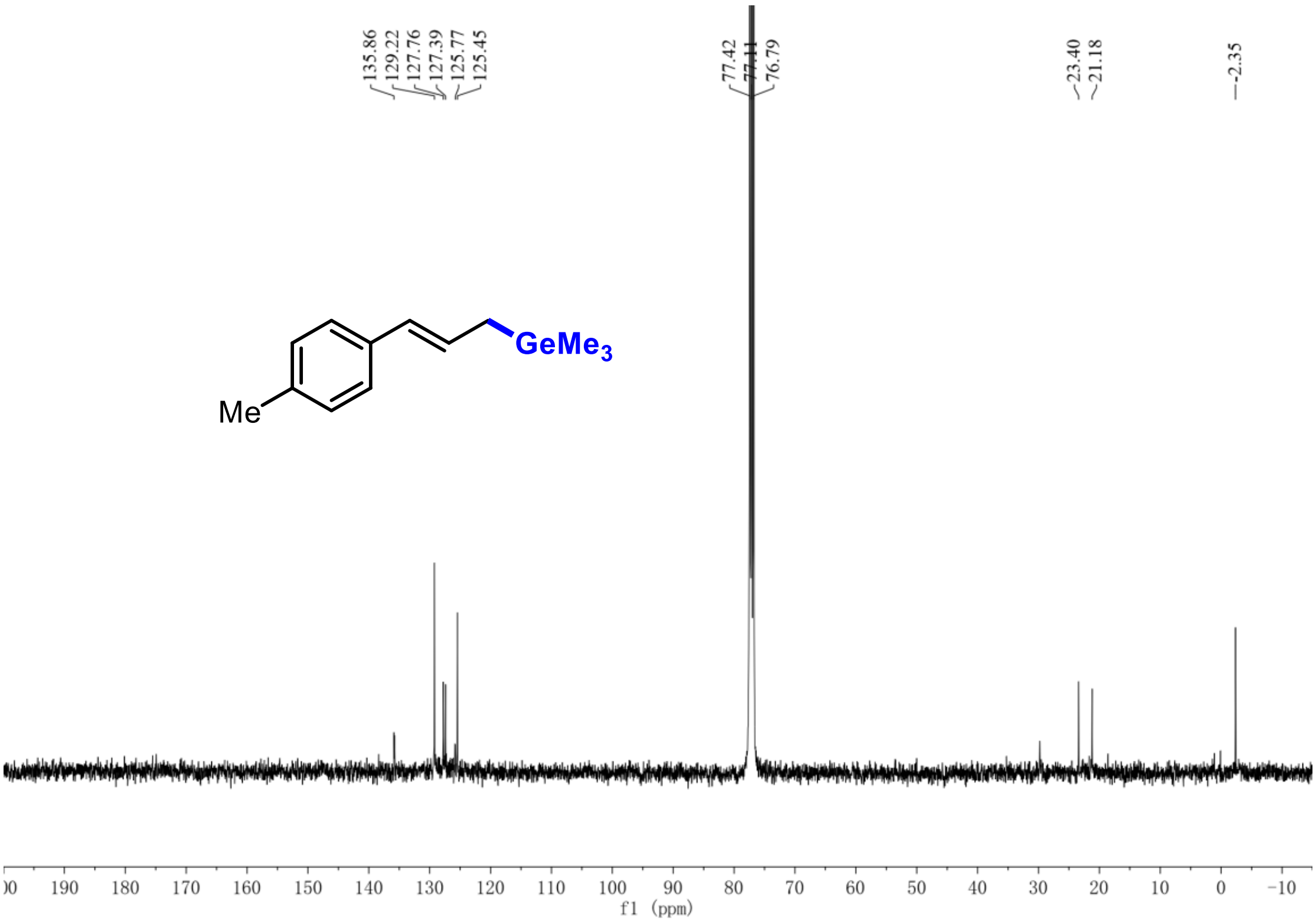

^{1}H NMR spectrum of 5ad (400 MHz, $CDCl_3$)

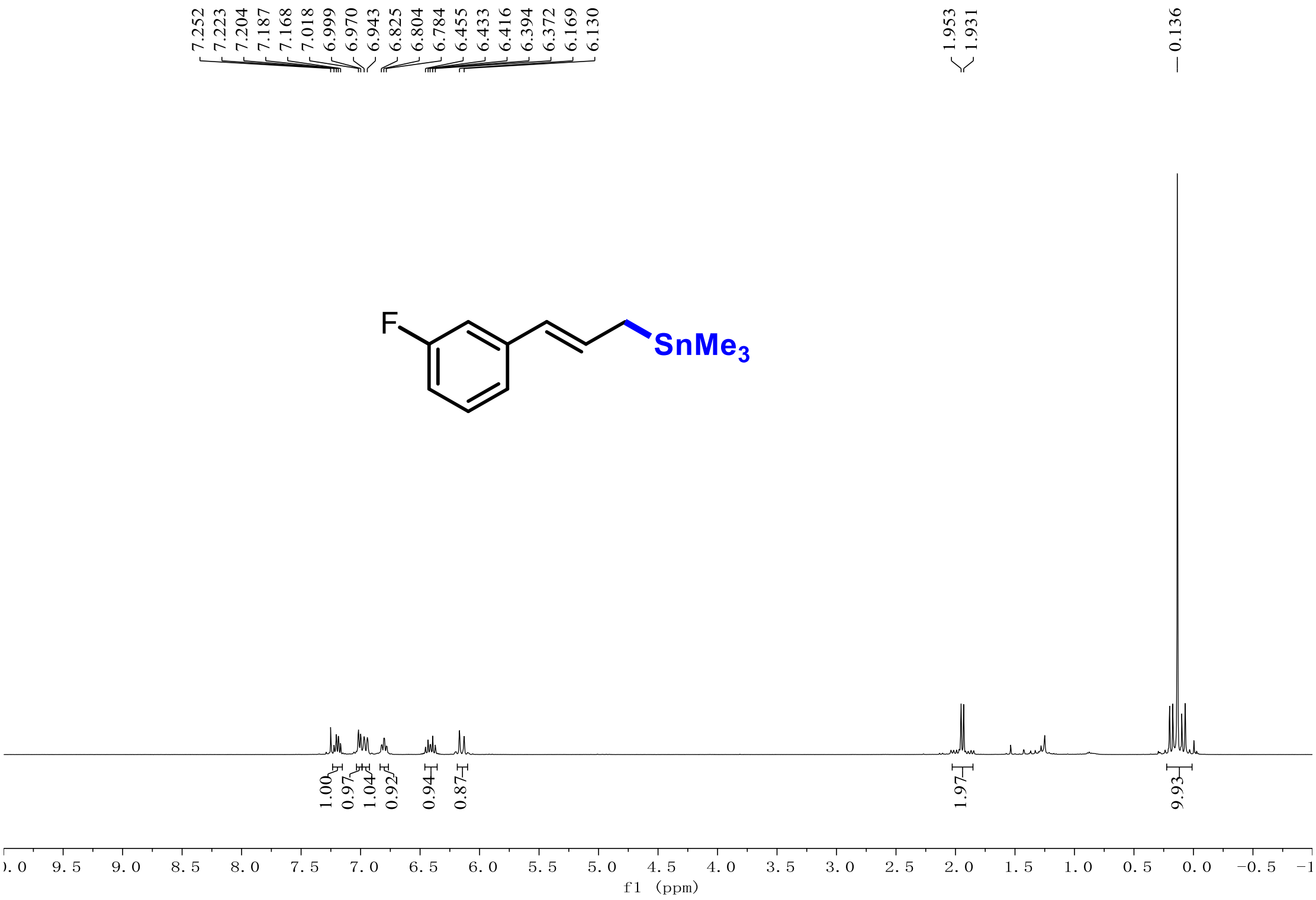

^{13}C NMR spectrum of 5ad (153 MHz, $CDCl_3$)

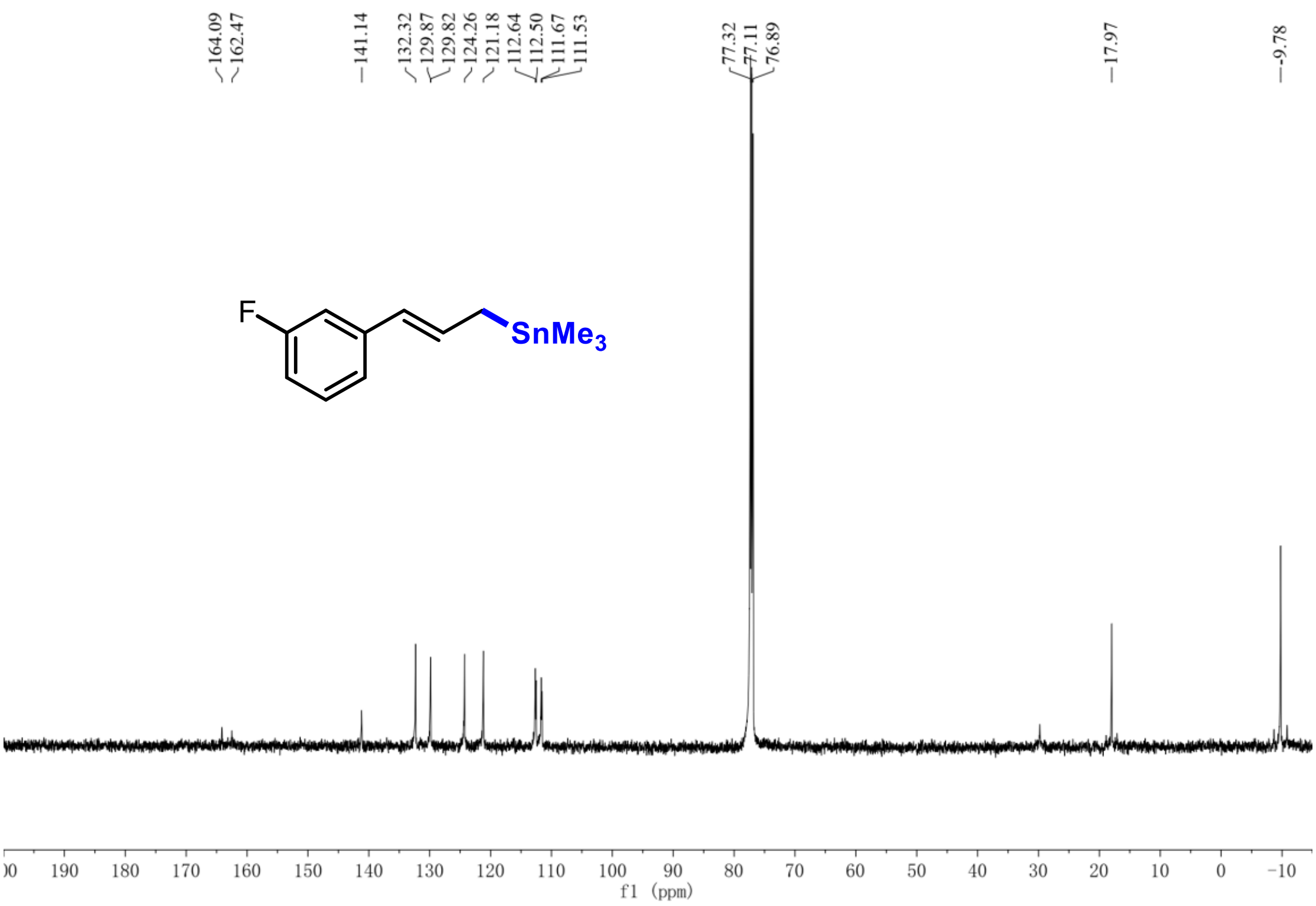

^{1}H NMR spectrum of 5ae (400 MHz, $CDCl_3$)

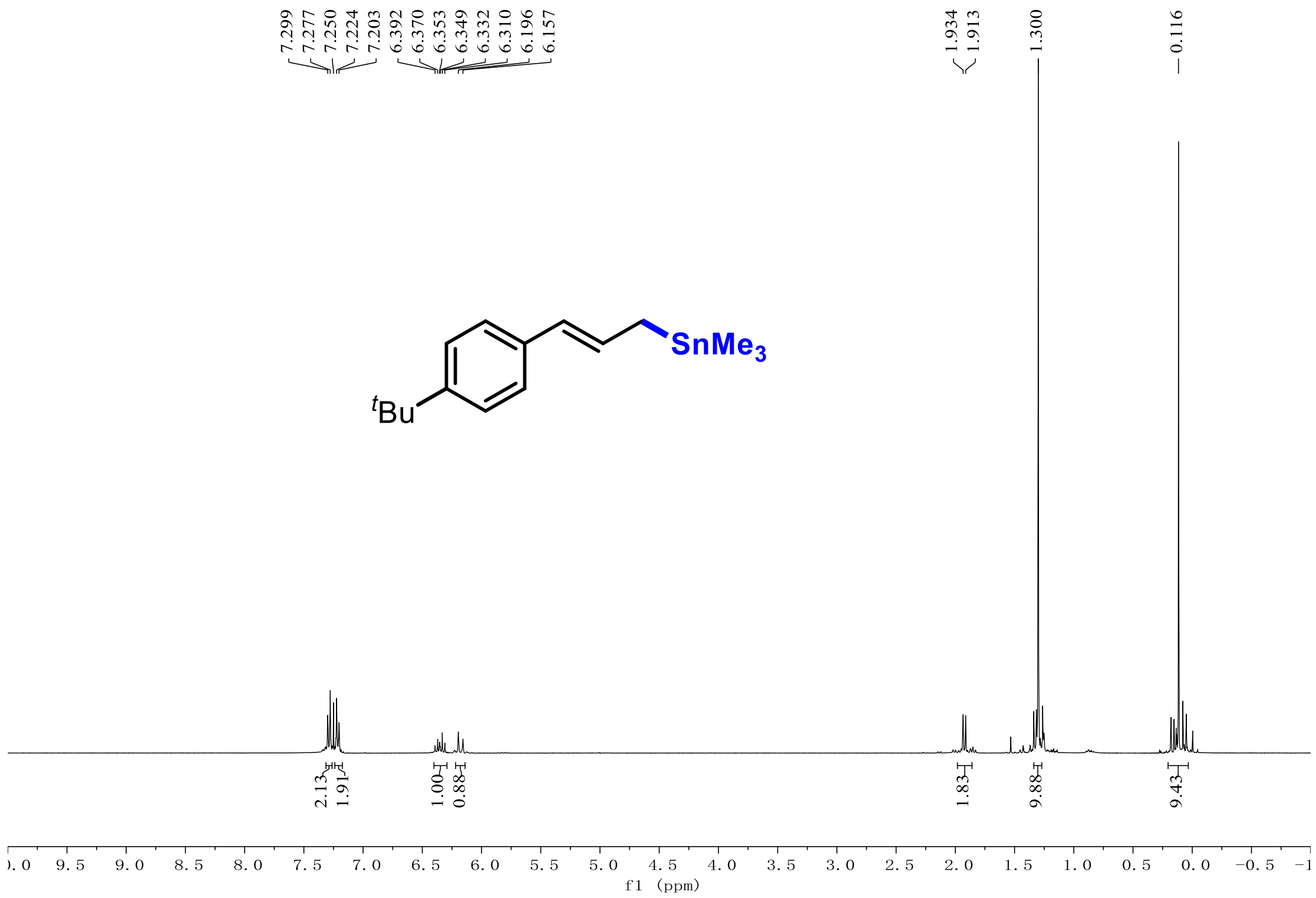

^{13}C NMR spectrum of 5ae (153 MHz, $CDCl_3$)

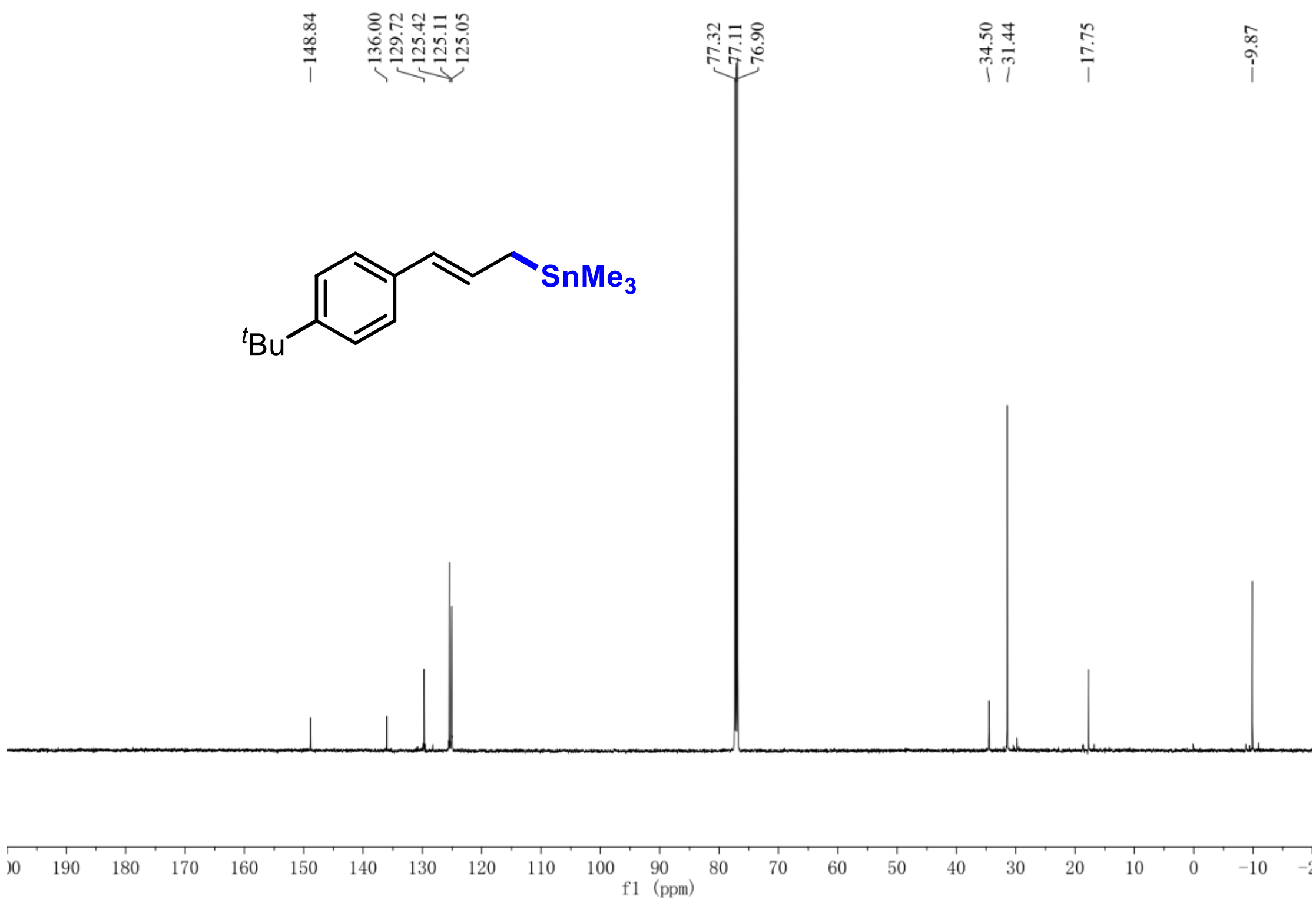

^{1}H NMR spectrum of 5af (400 MHz, $CDCl_3$)

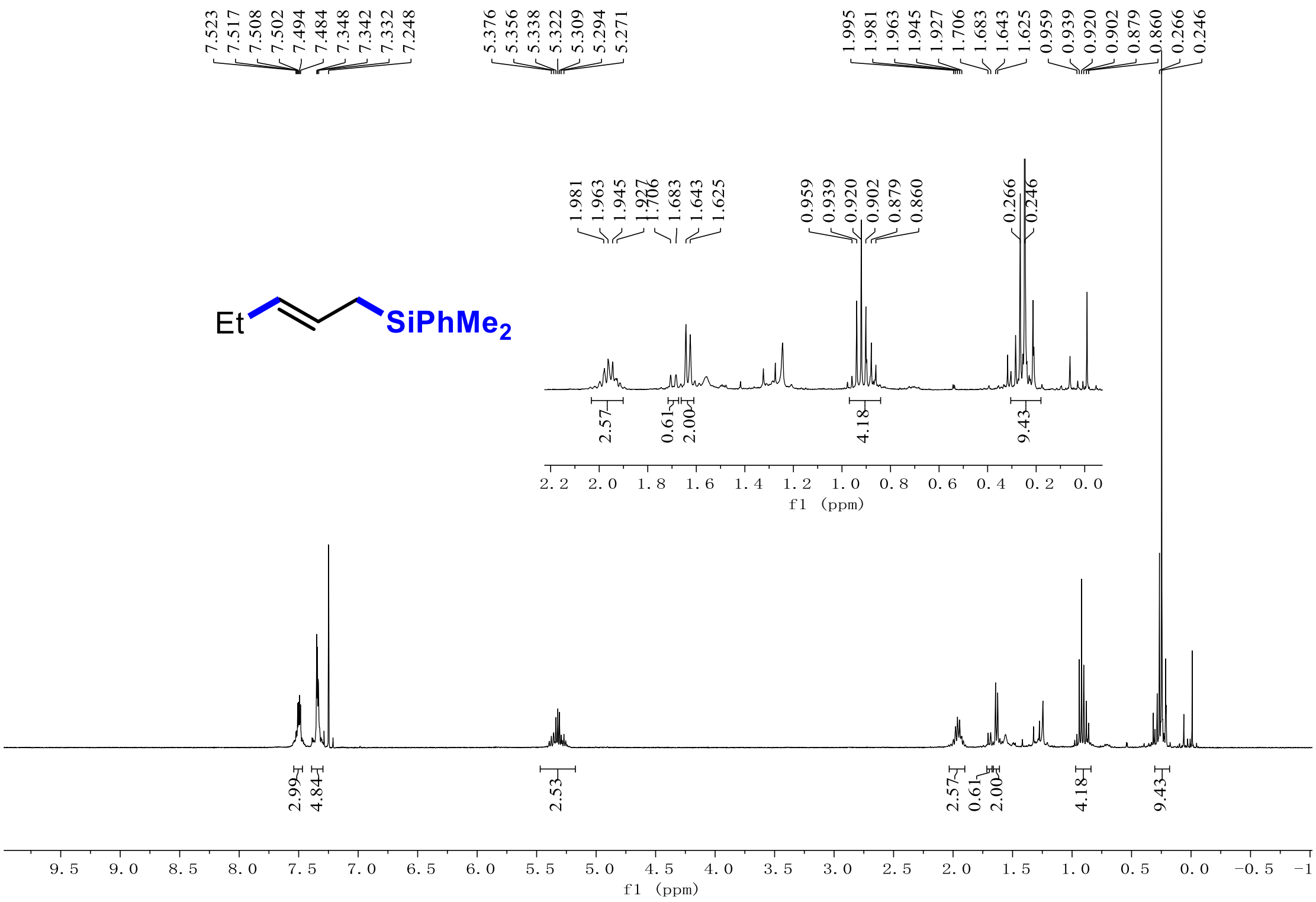

^{13}C NMR spectrum of 5af (101 MHz, $CDCl_3$)

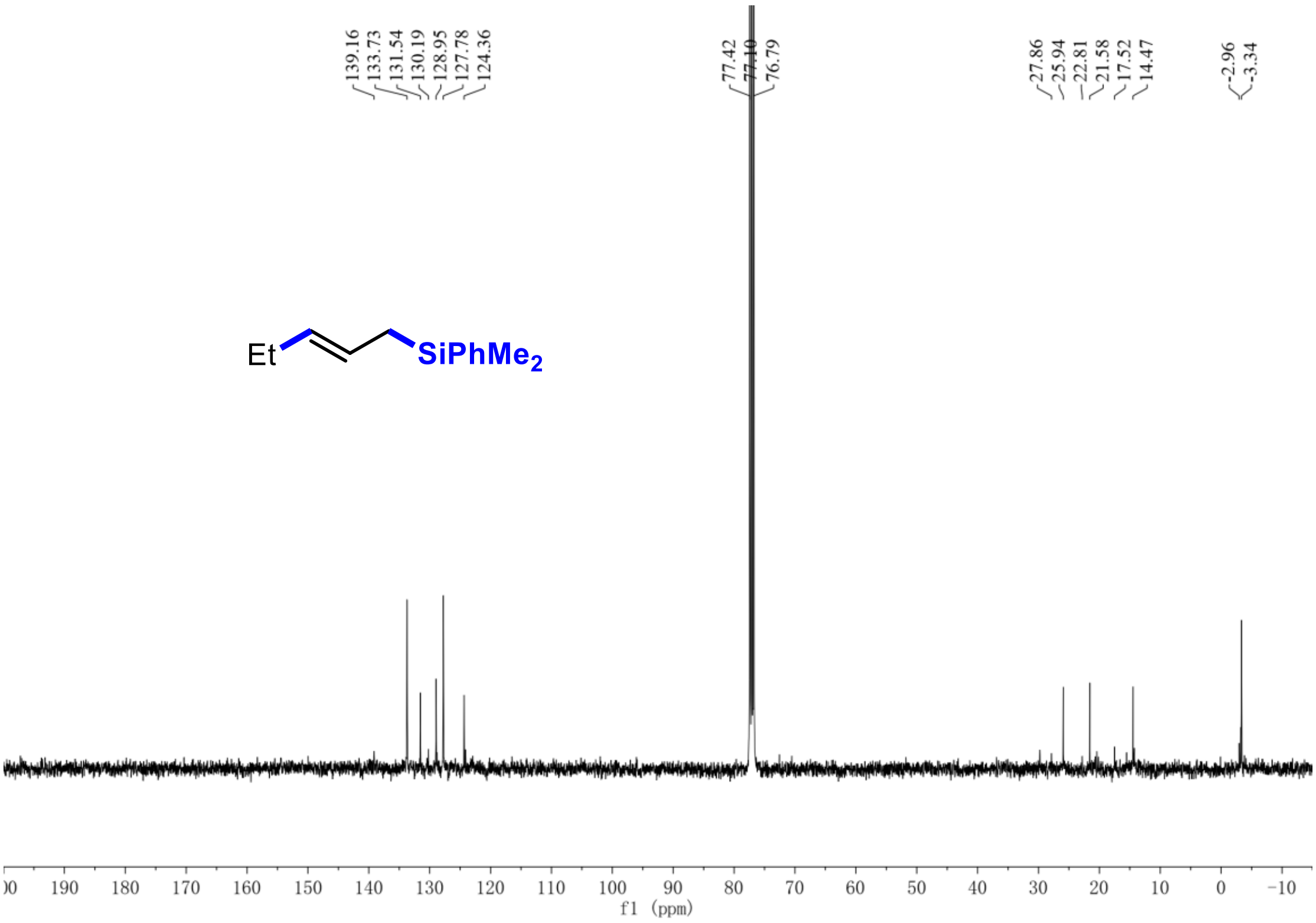

^{1}H NMR spectrum of 5ag (400 MHz, $CDCl_3$)

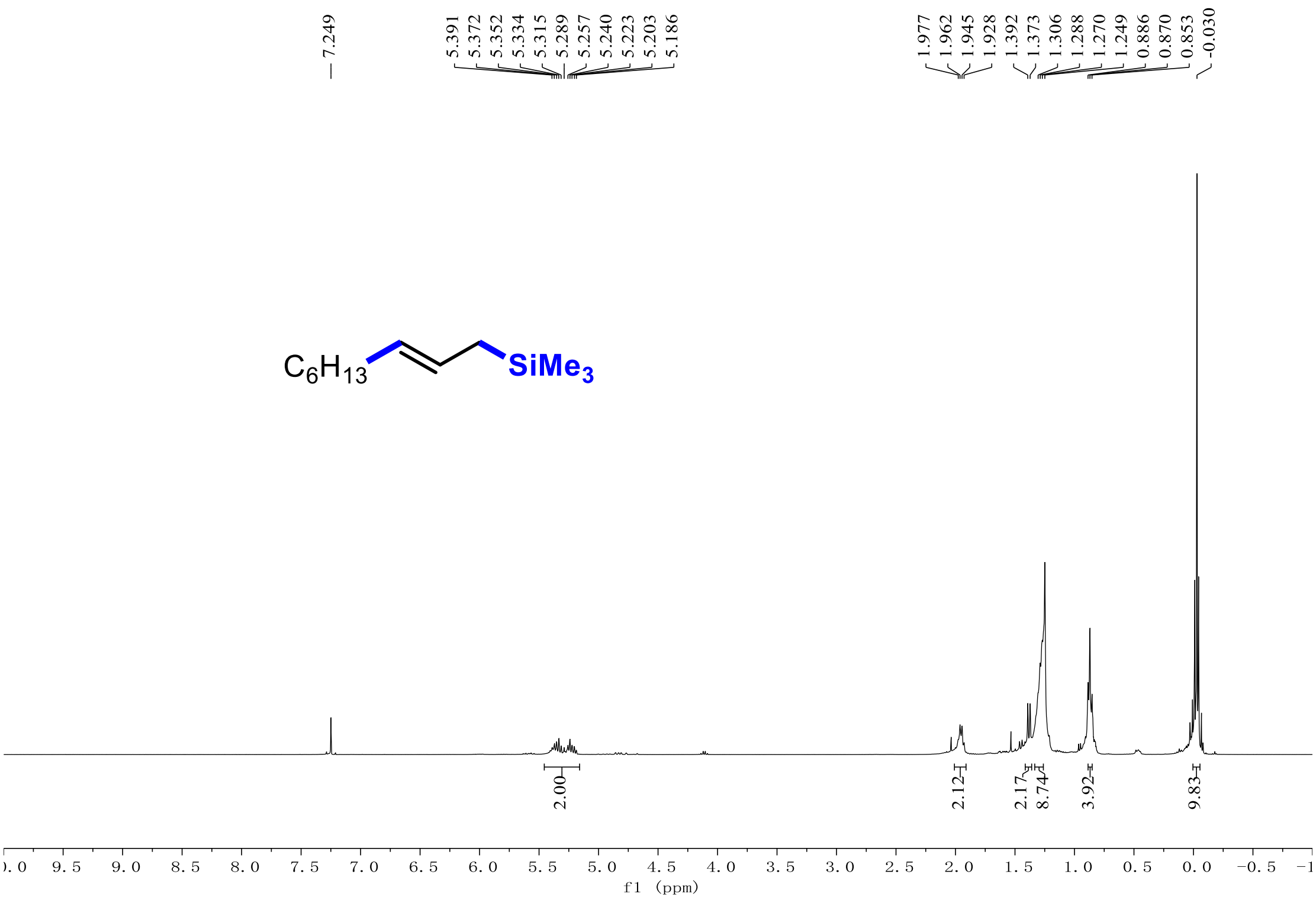

^{13}C NMR spectrum of 5ag (101 MHz, $CDCl_3$)

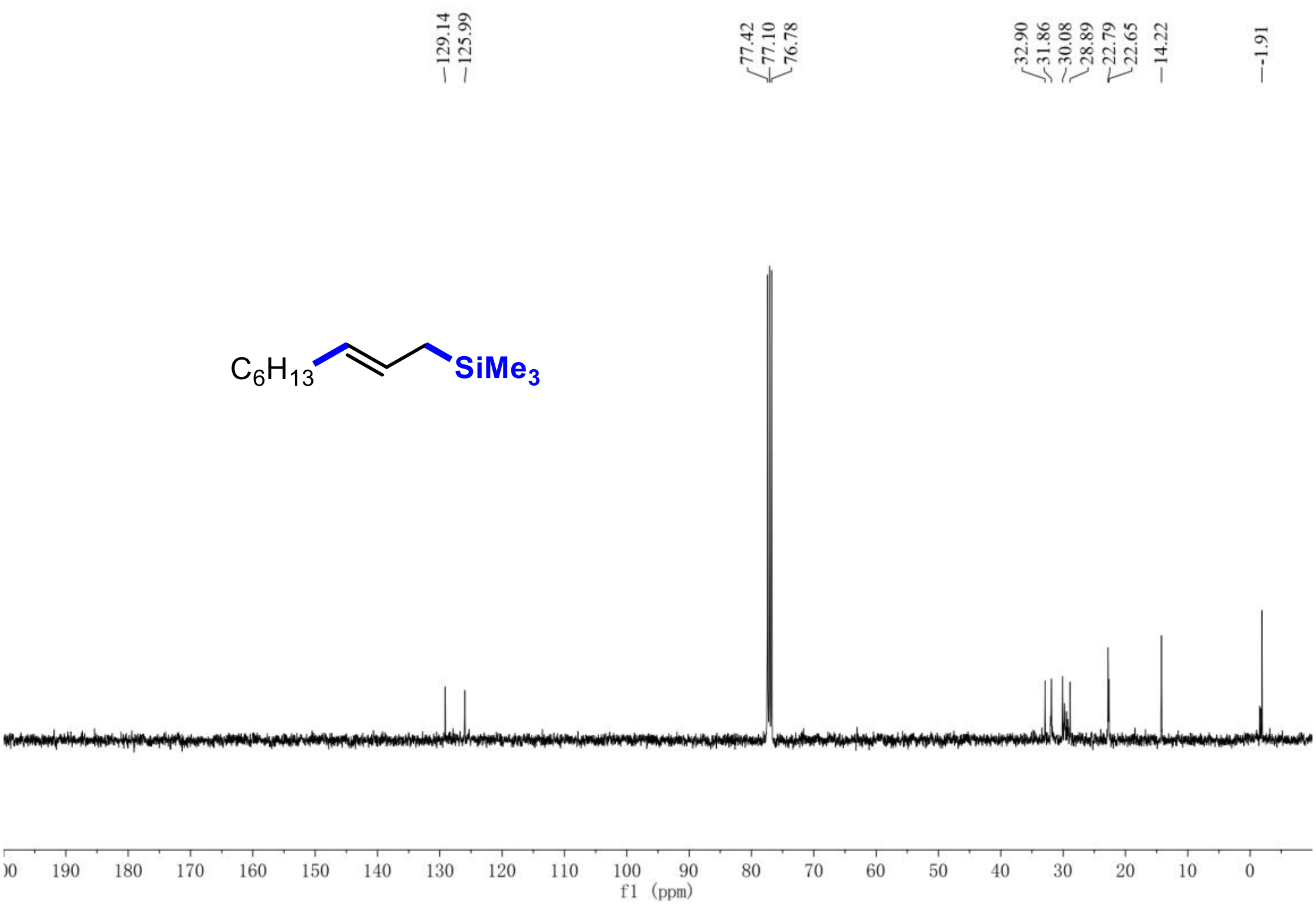

^{1}H NMR spectrum of 5ah (400 MHz, CDCl$_3$)

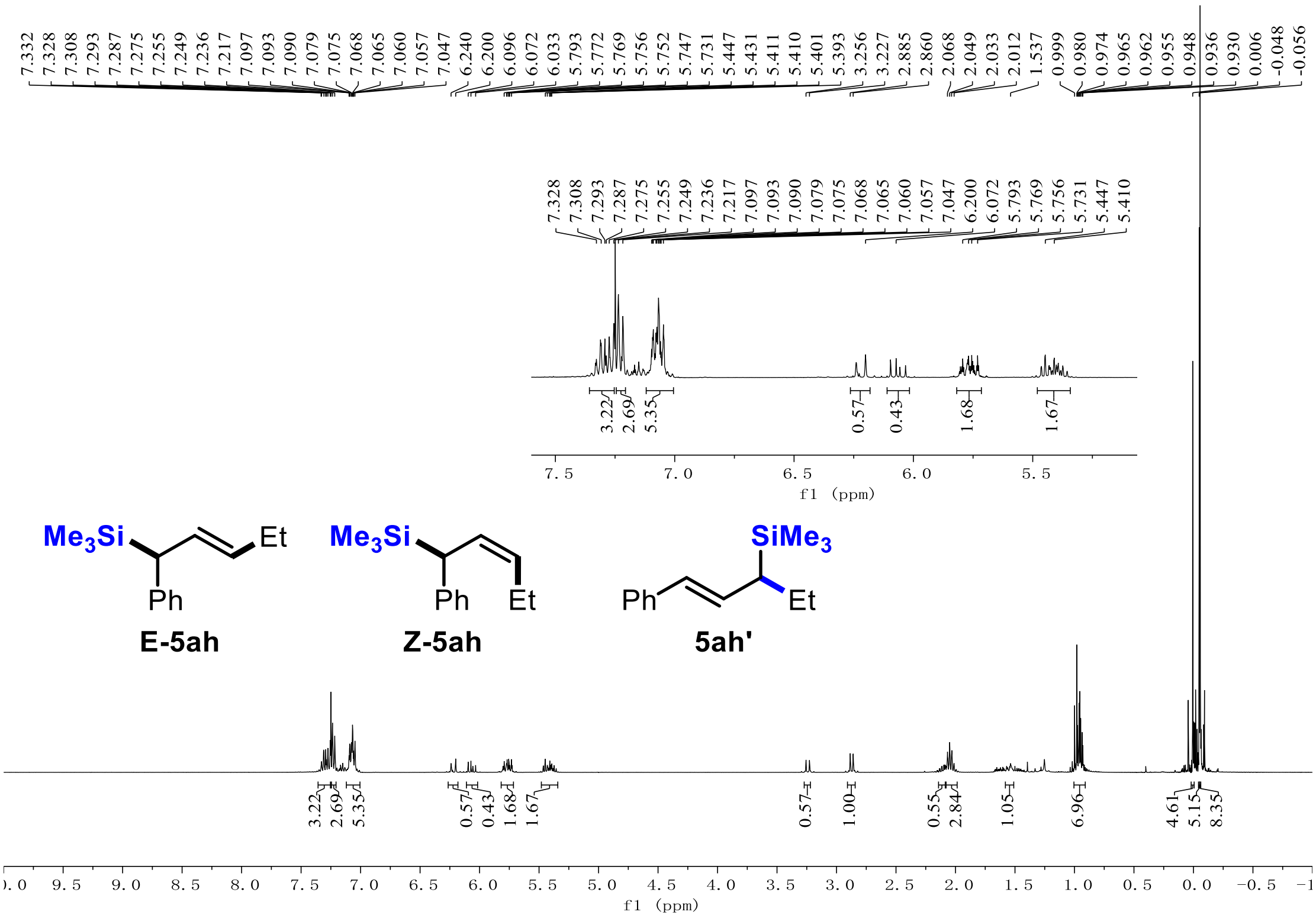

^{13}C NMR spectrum of 5ah (101 MHz, CDCl$_3$)

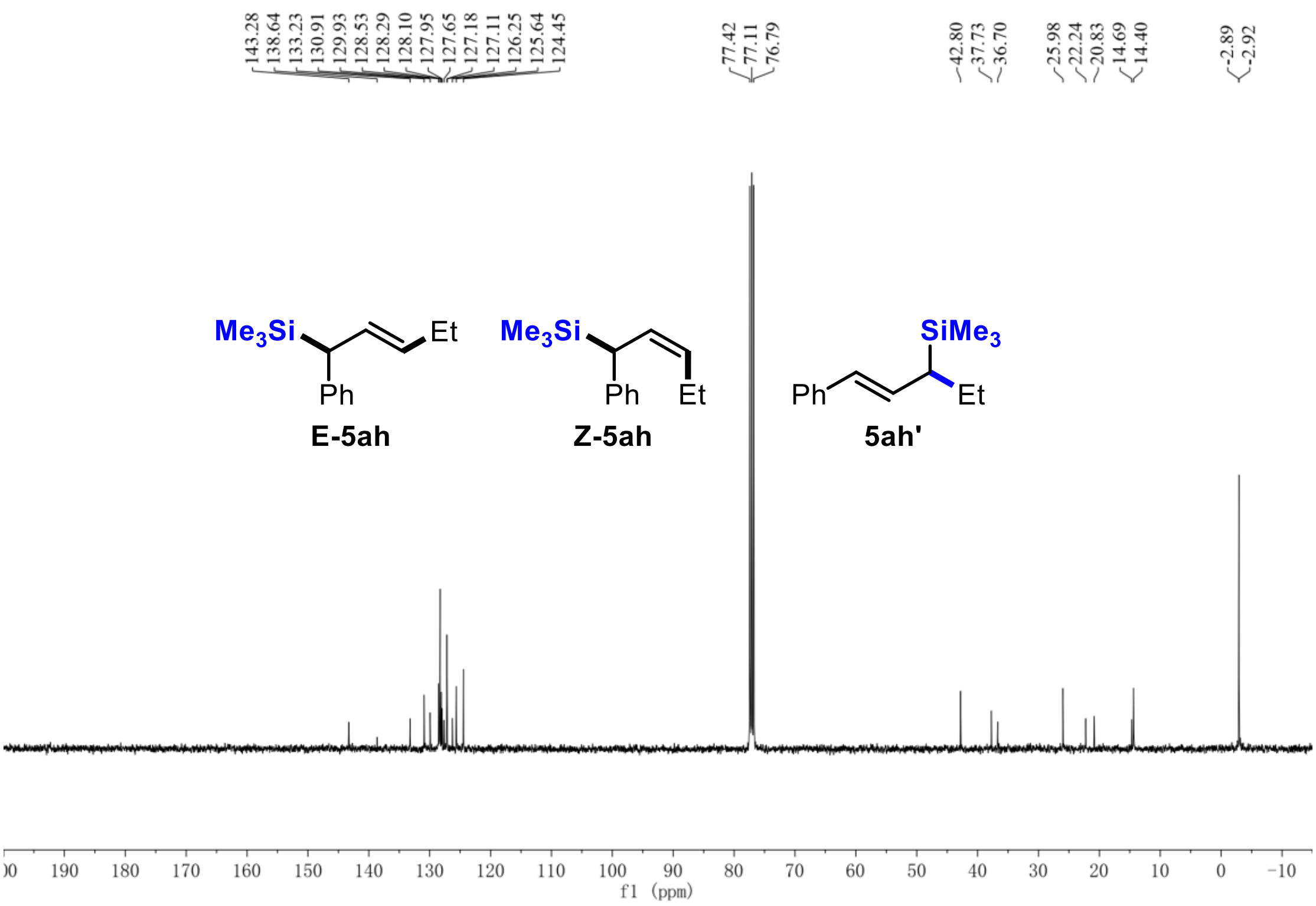

^{1}H NMR spectrum of 5ai (400 MHz, $CDCl_3$)

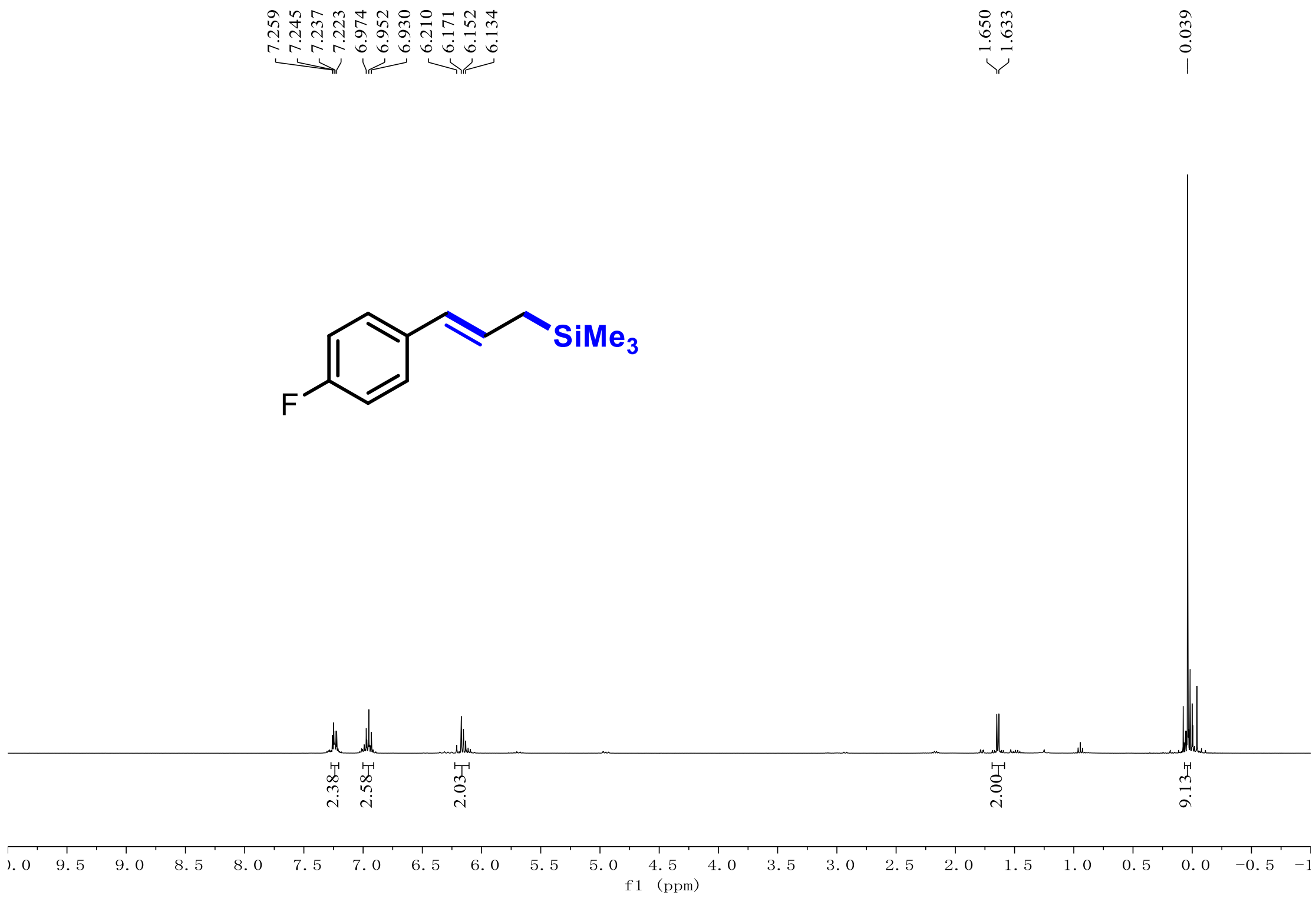

^{13}C NMR spectrum of 5ai (101 MHz, $CDCl_3$)

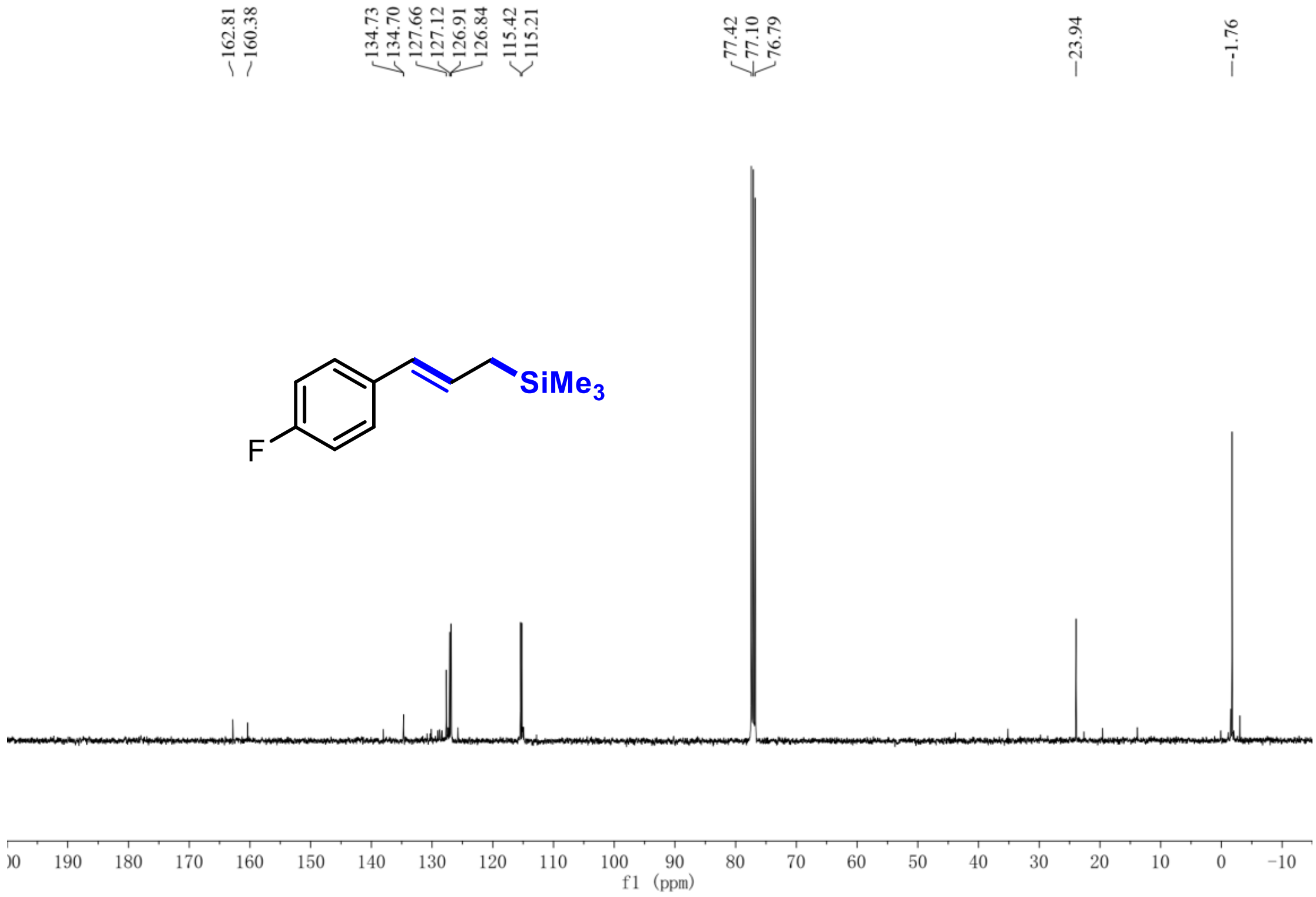

^{1}H NMR spectrum of 5aj (400 MHz, $CDCl_3$)

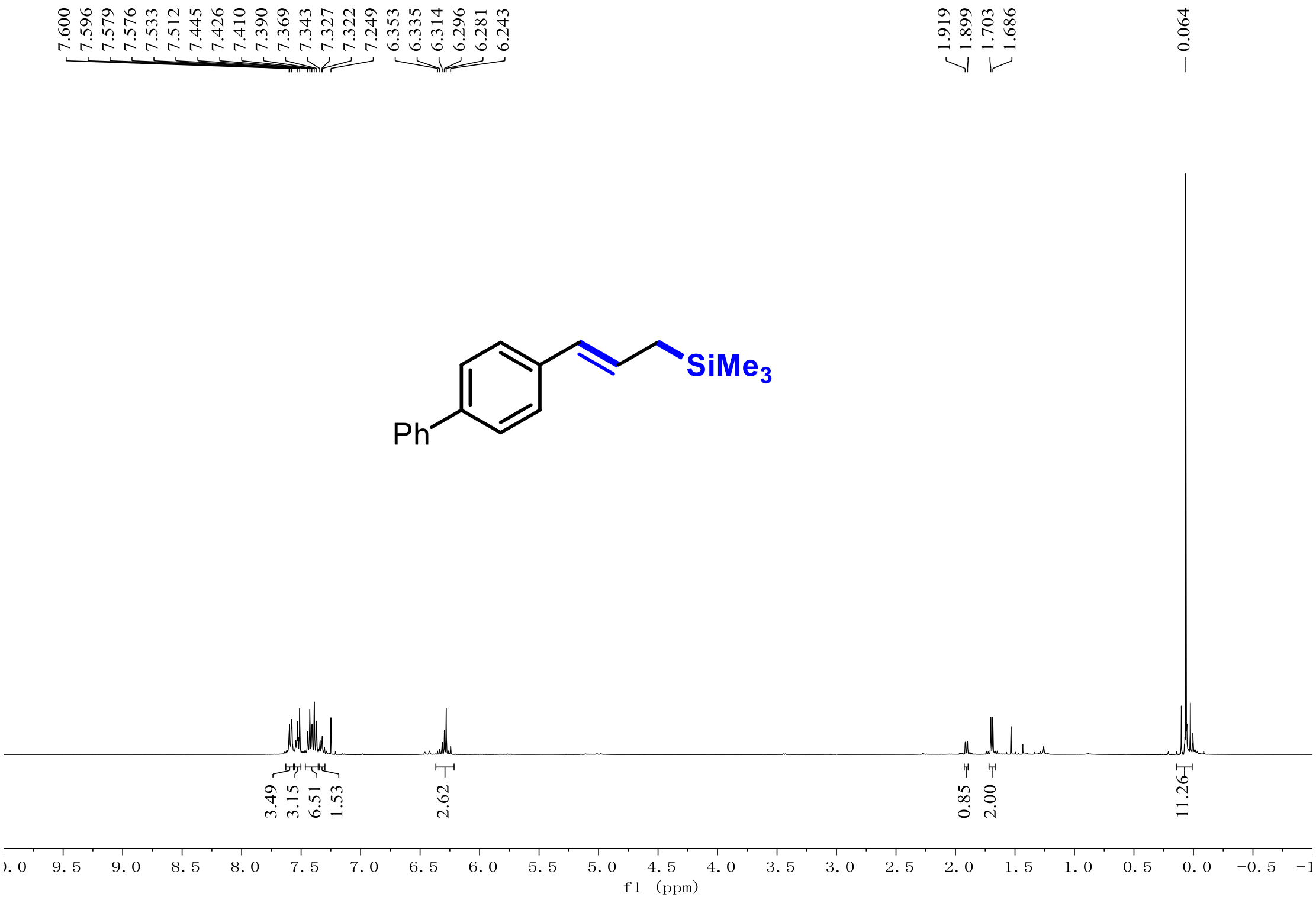

^{13}C NMR spectrum of 5aj (101 MHz, $CDCl_3$)

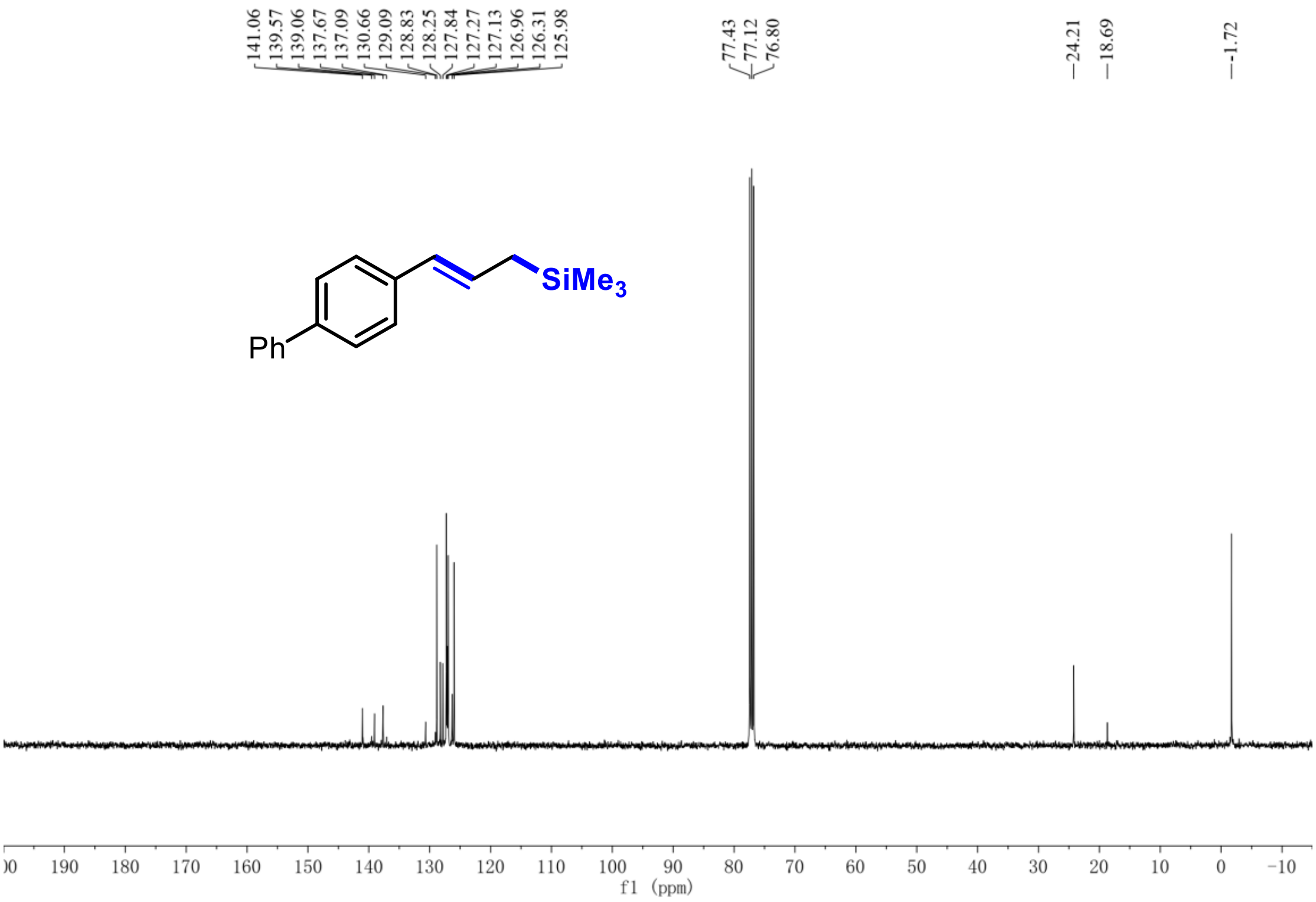

^{1}H NMR spectrum of 5ak (400 MHz, $CDCl_3$)

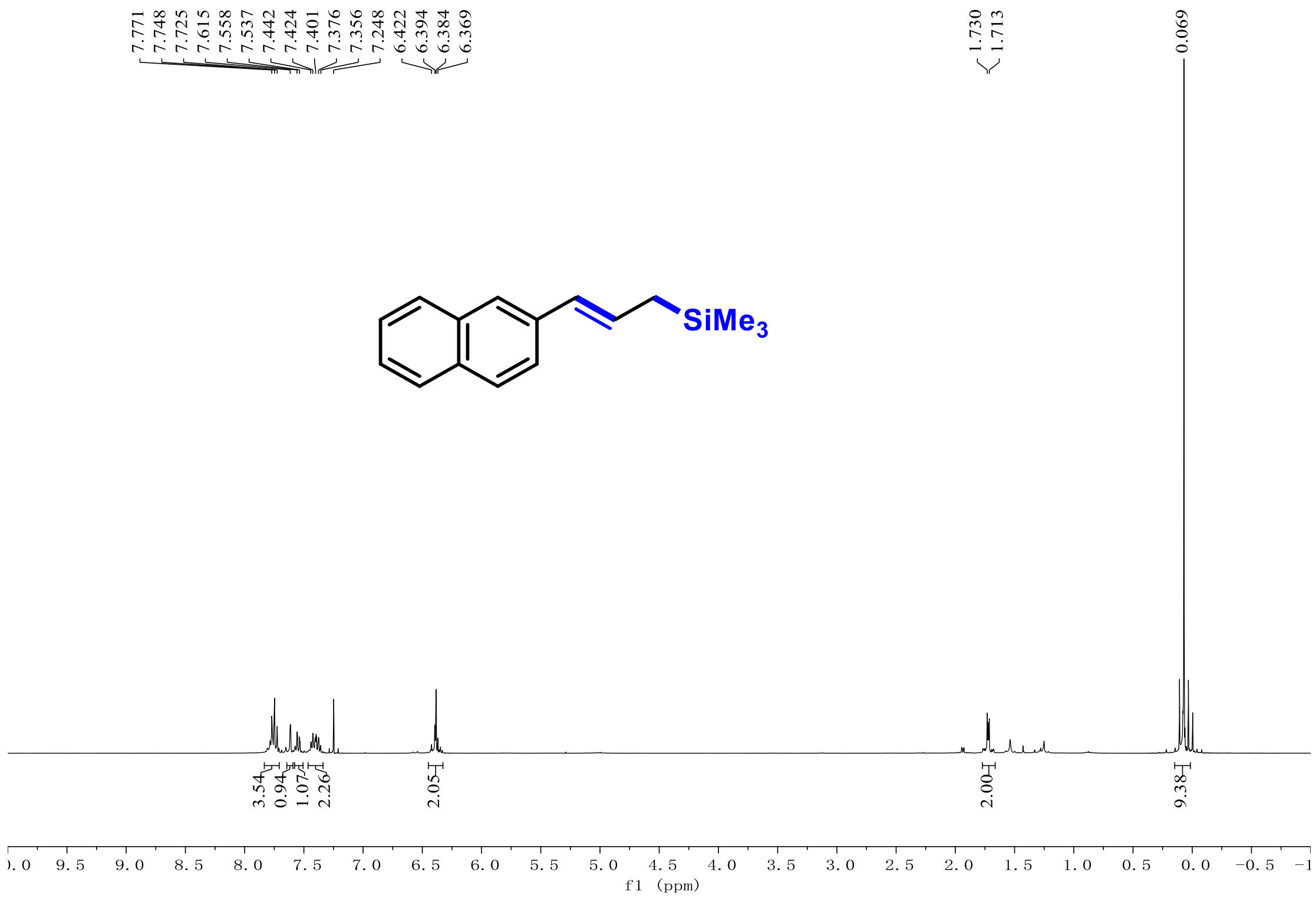

^{13}C NMR spectrum of 5ak (101 MHz, $CDCl_3$)

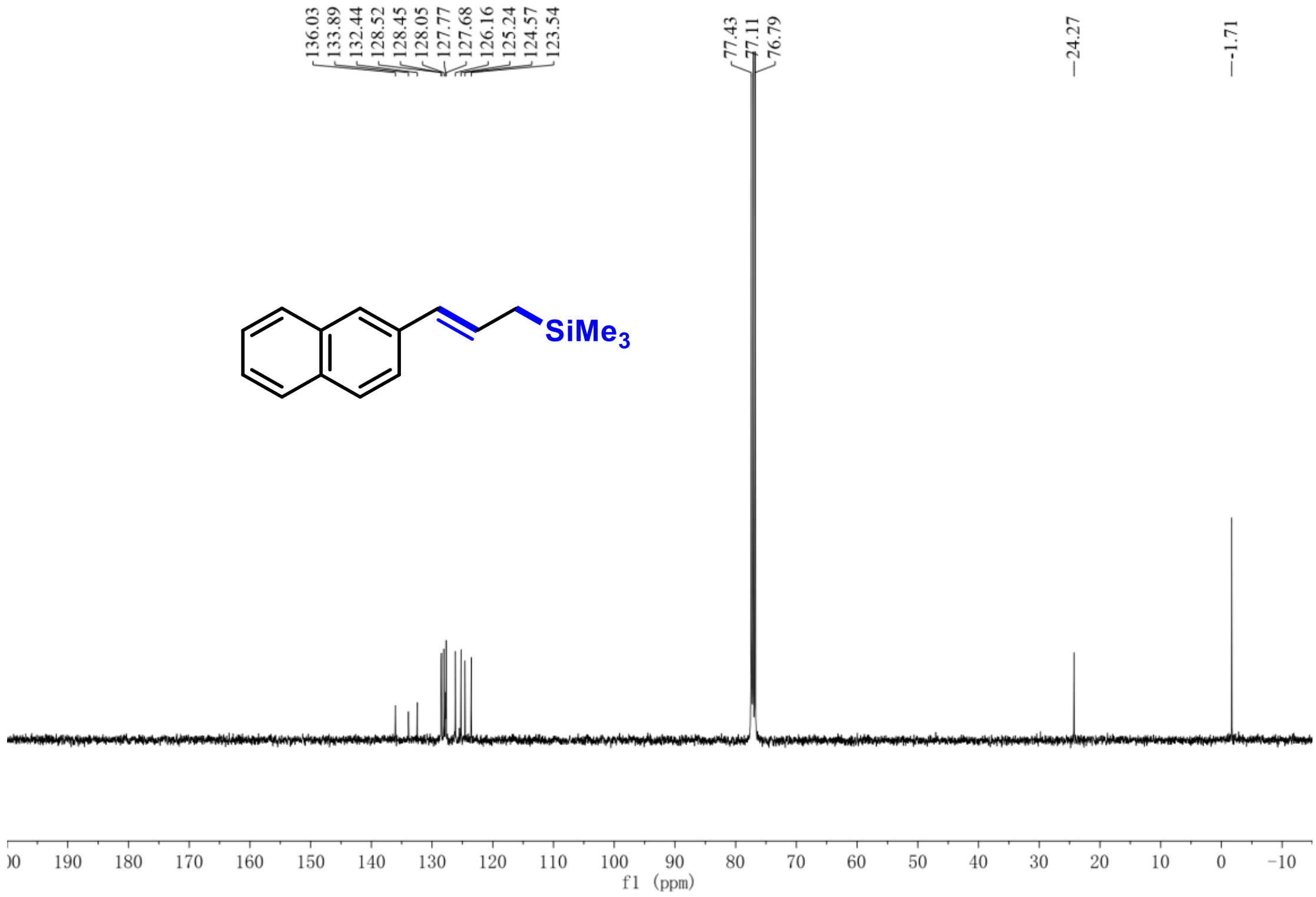

^{1}H NMR spectrum of 5al (400 MHz, $CDCl_3$)

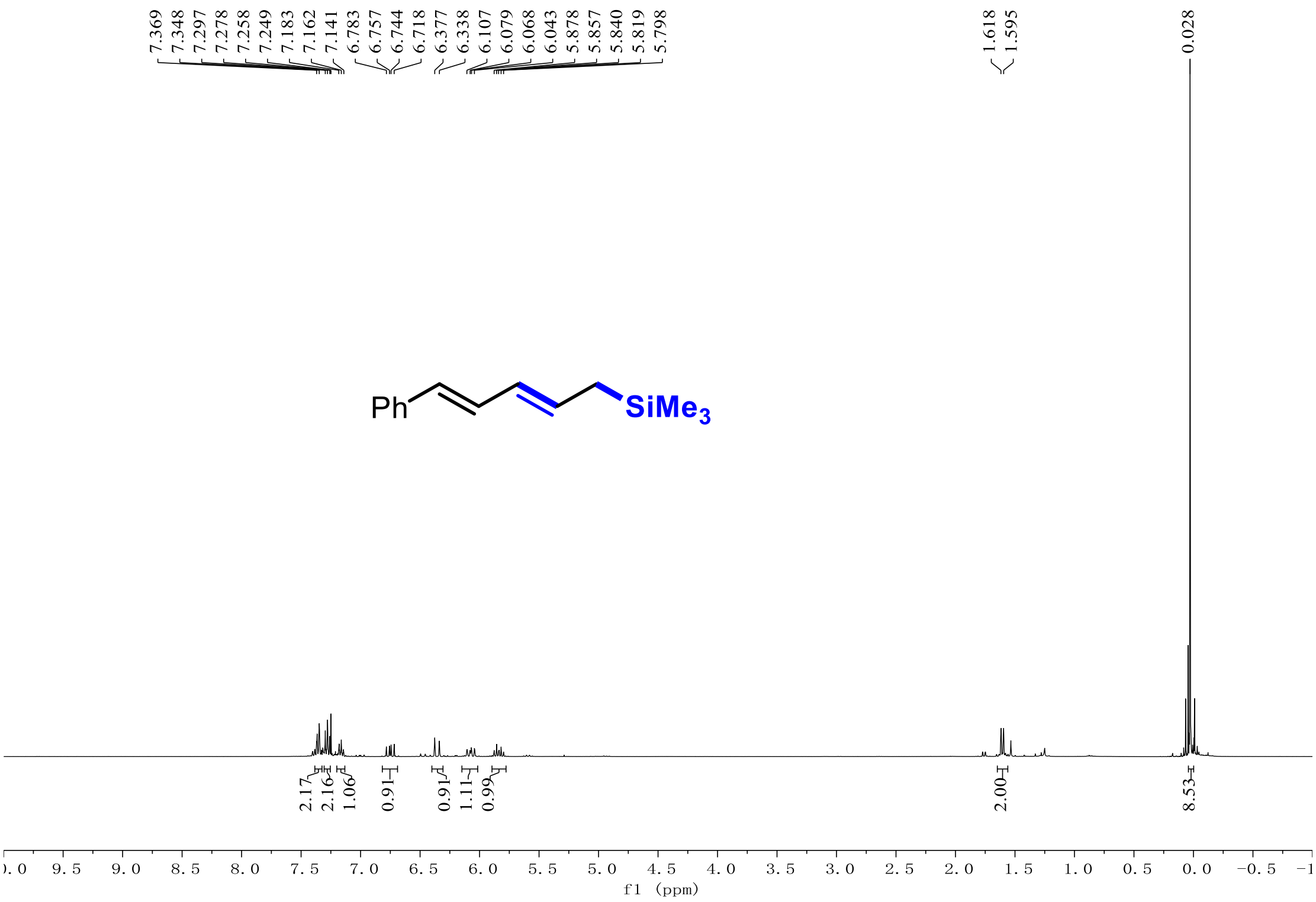

^{13}C NMR spectrum of 5al (101 MHz, $CDCl_3$)

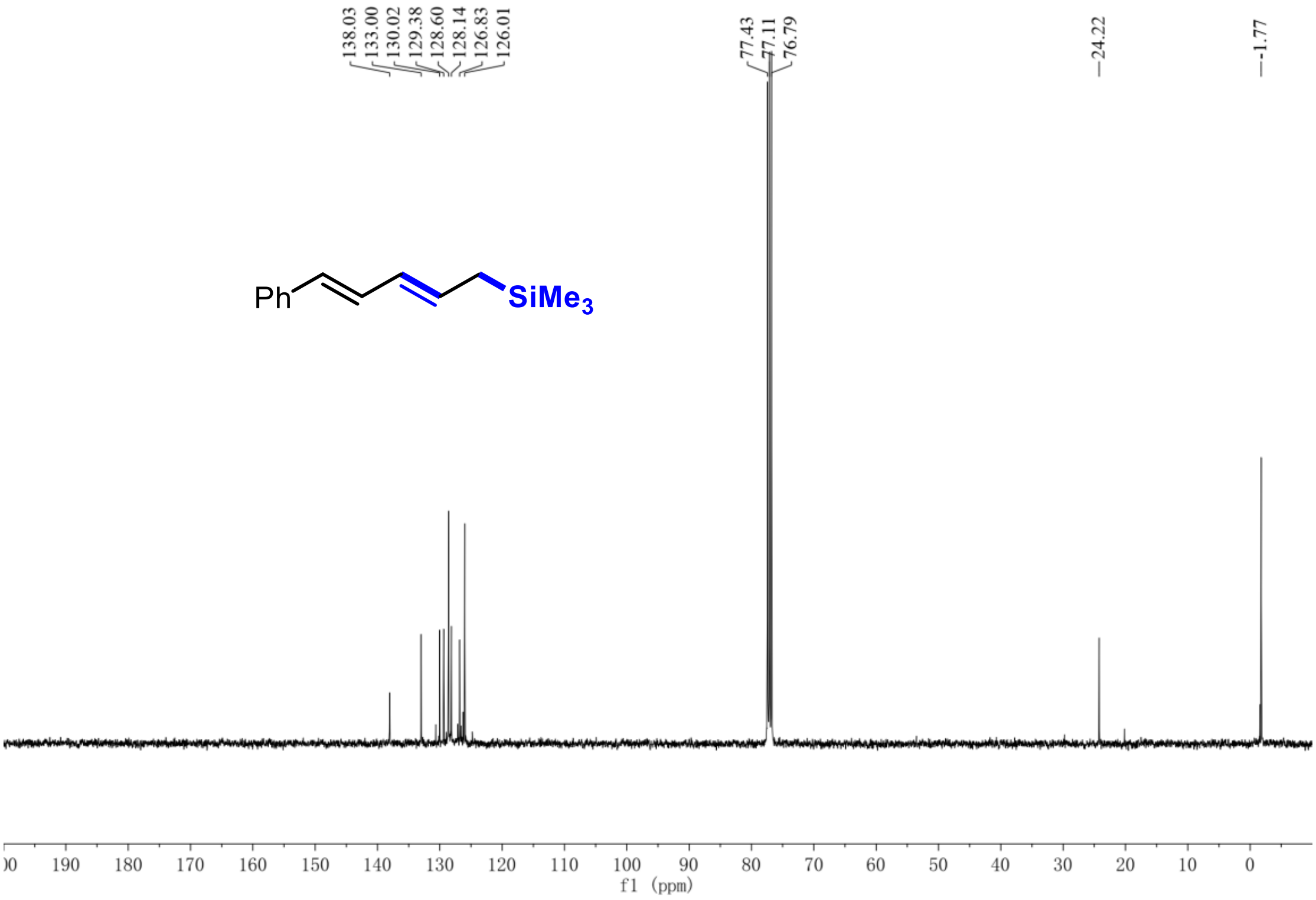

^{1}H NMR spectrum of 5am (400 MHz, $CDCl_3$)

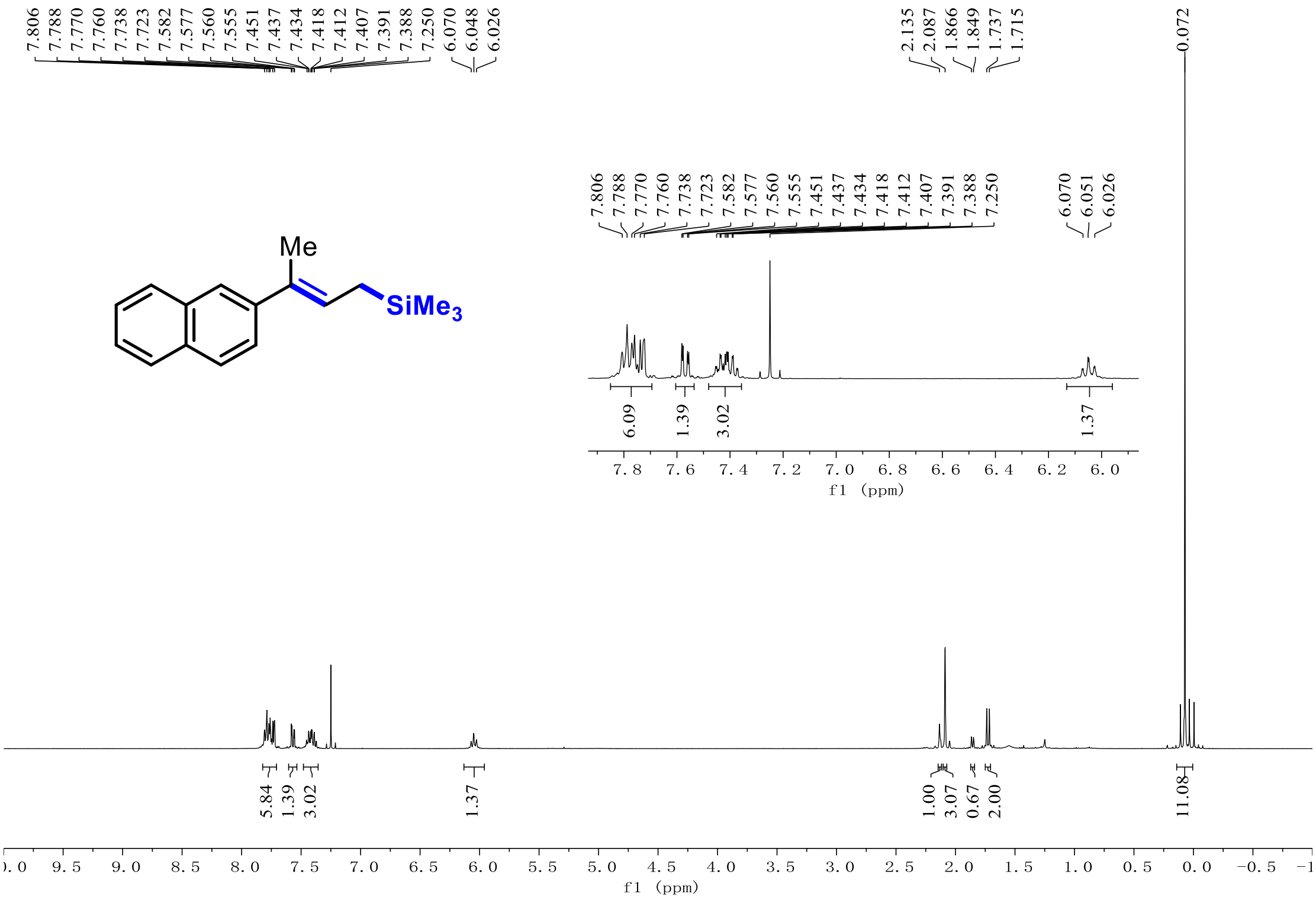

^{13}C NMR spectrum of 5am (101 MHz, $CDCl_3$)

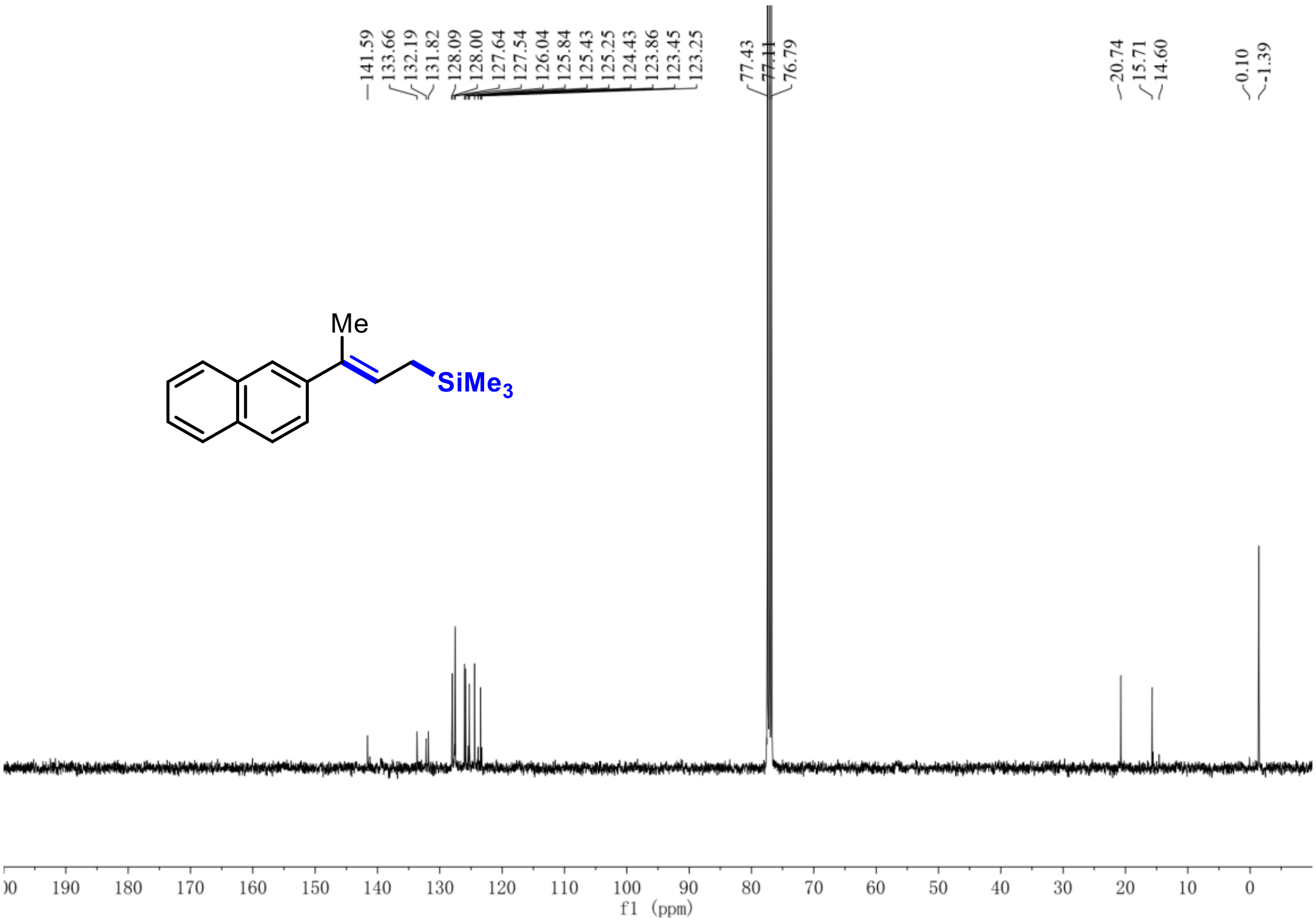

^{1}H NMR spectrum of 7a (600 MHz, $CDCl_3$)

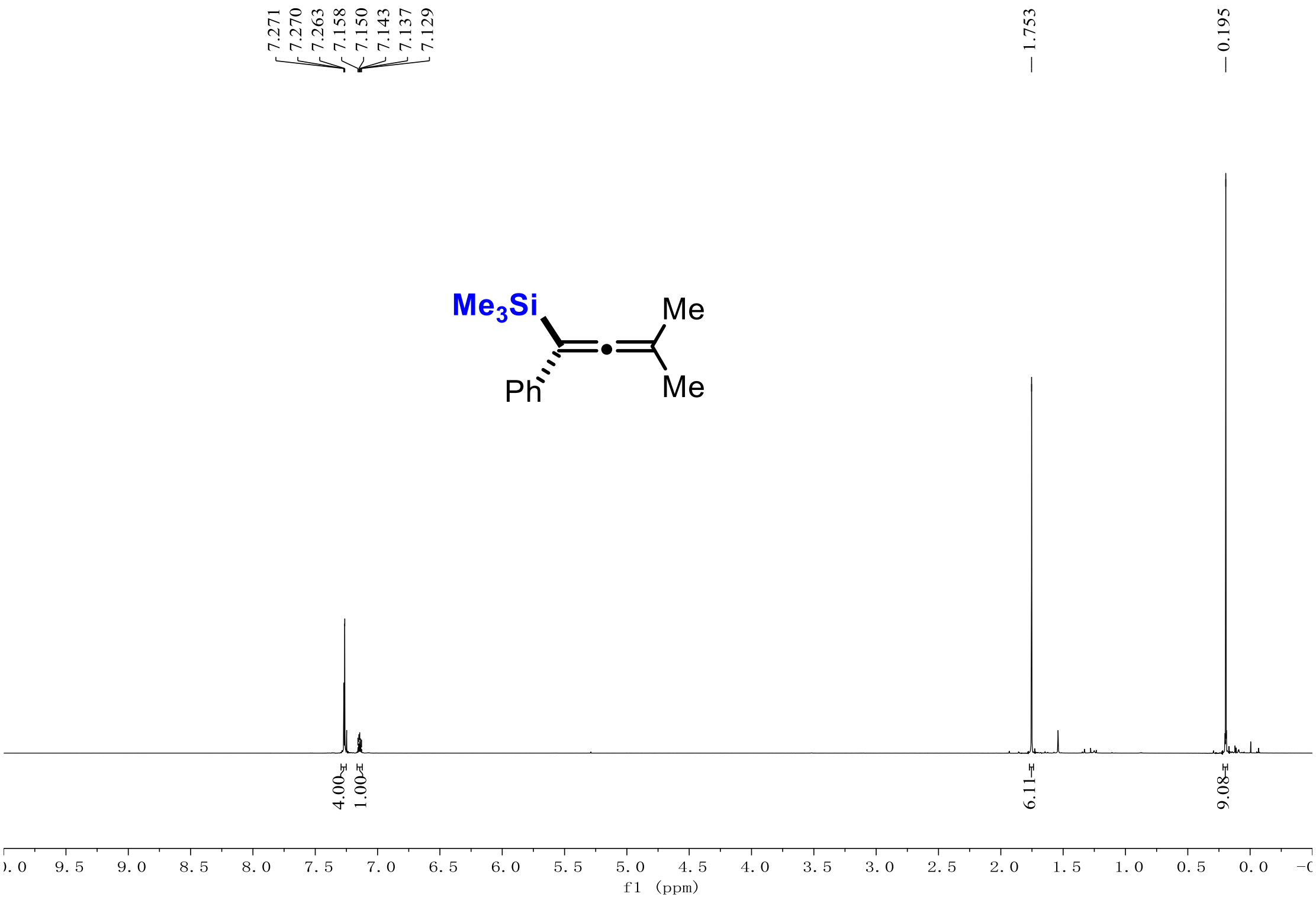

^{13}C NMR spectrum of 7a (101 MHz, $CDCl_3$)

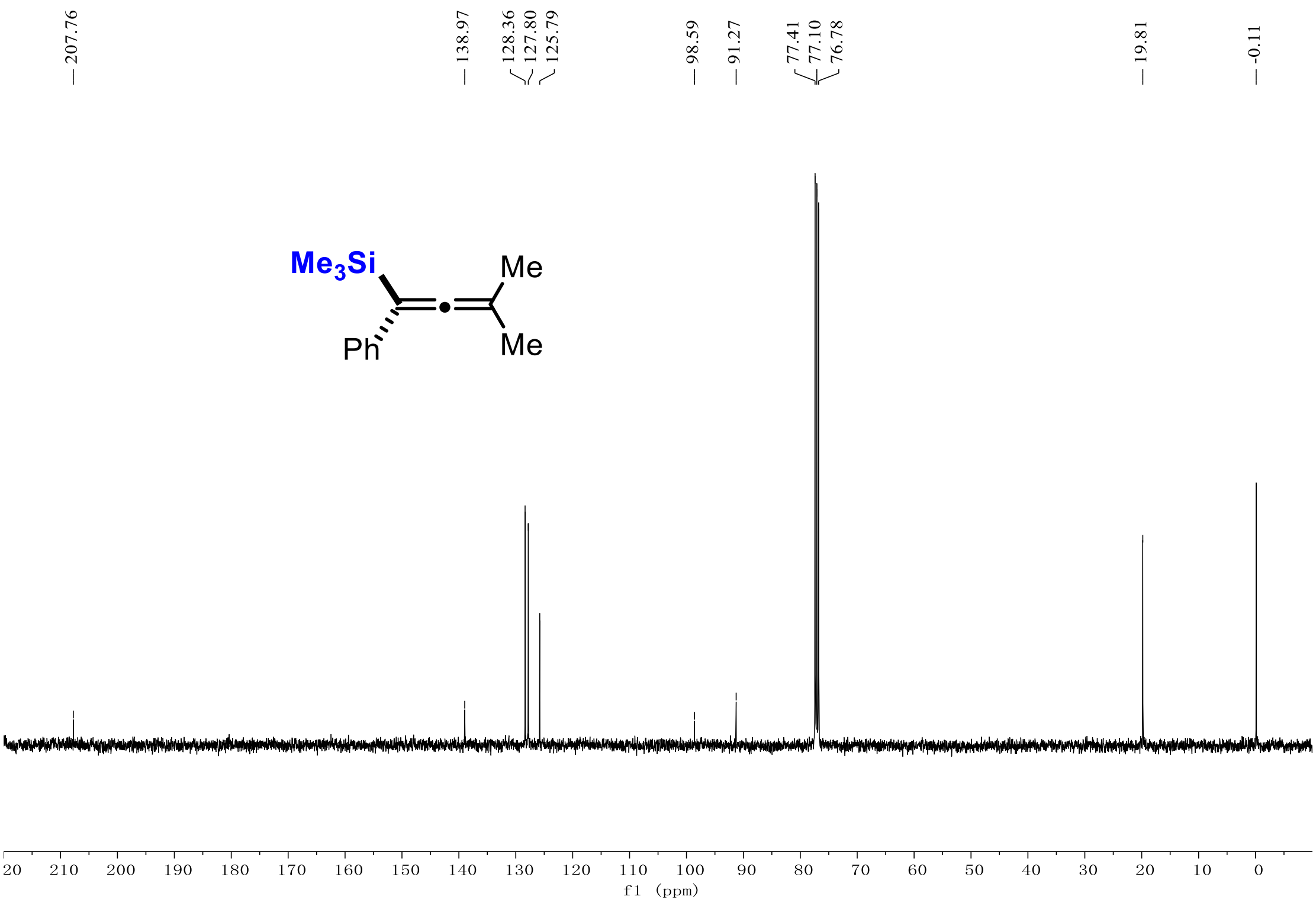

^{1}H NMR spectrum of 7b (600 MHz, $CDCl_3$)

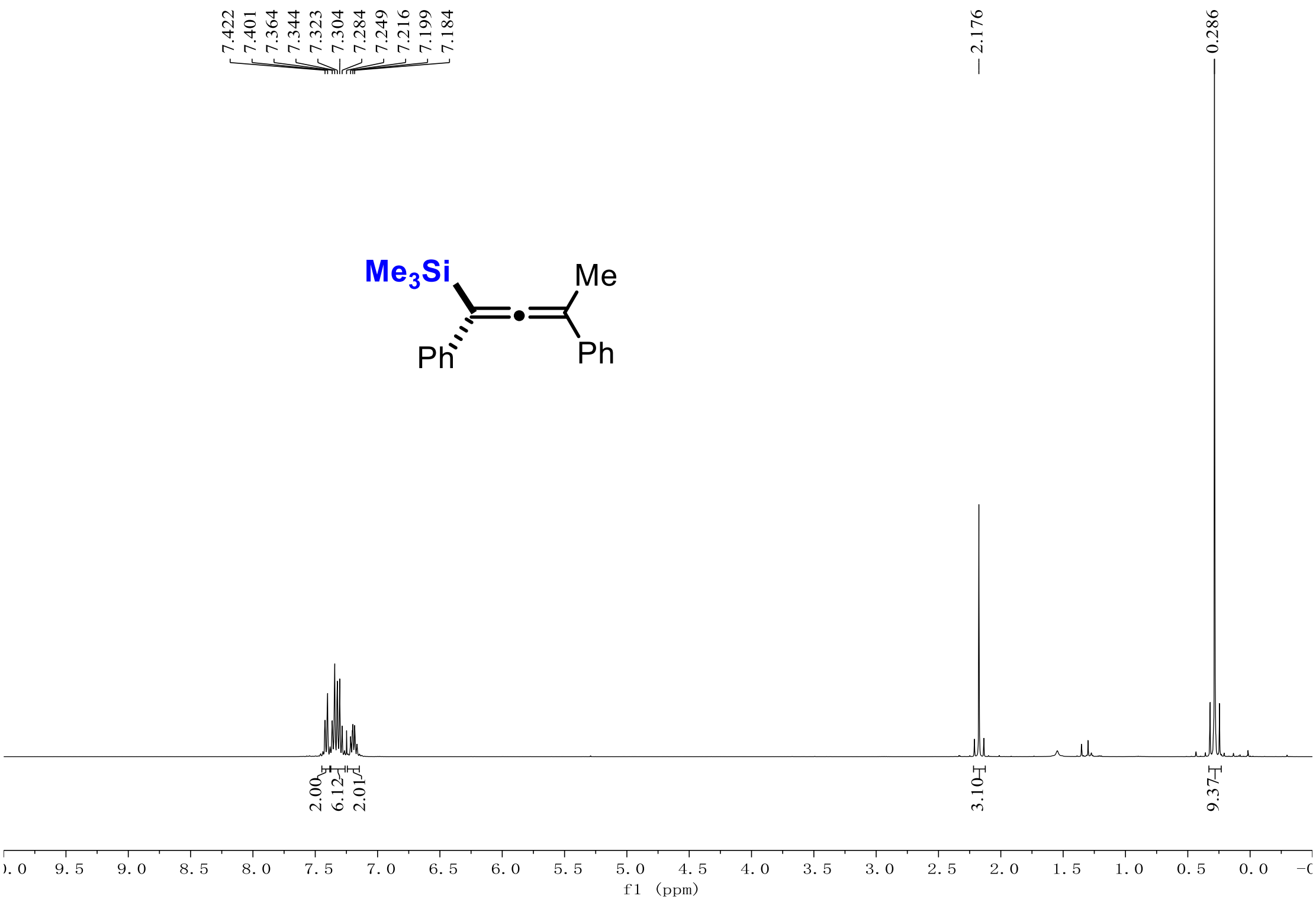

^{13}C NMR spectrum of 7b (101 MHz, $CDCl_3$)

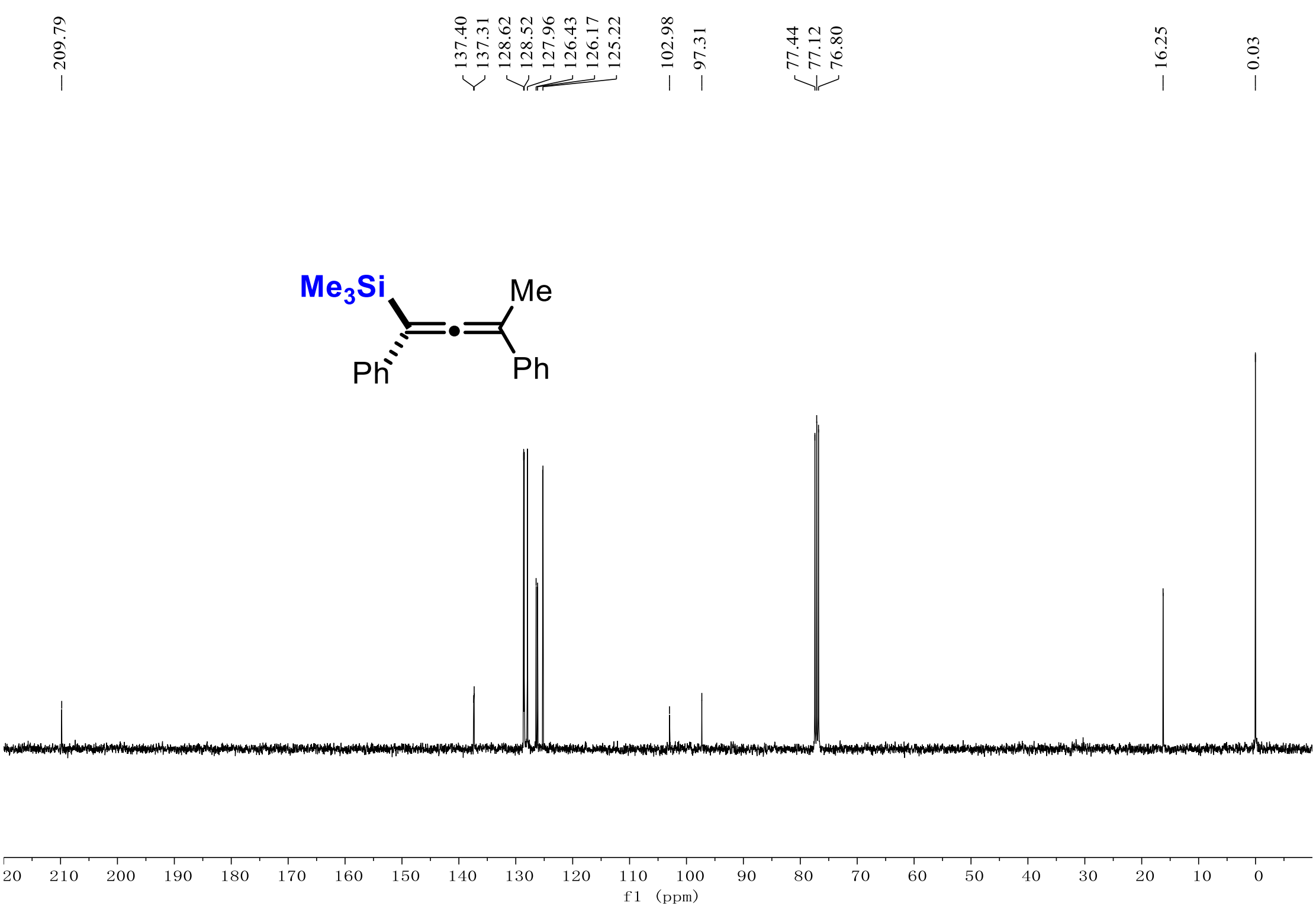

^{1}H NMR spectrum of 7c (400 MHz, $CDCl_3$)

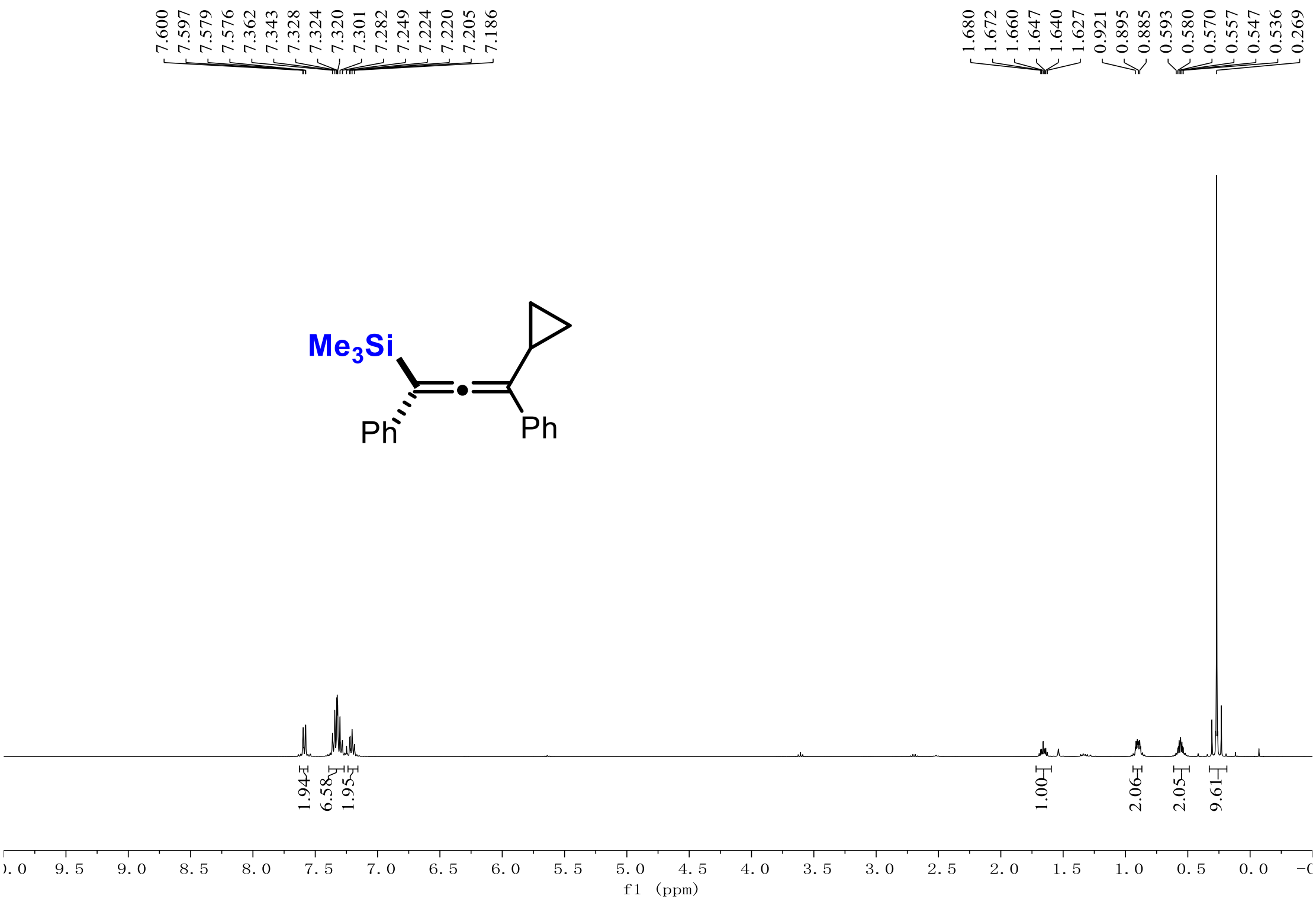

^{13}C NMR spectrum of 7c (101 MHz, $CDCl_3$)

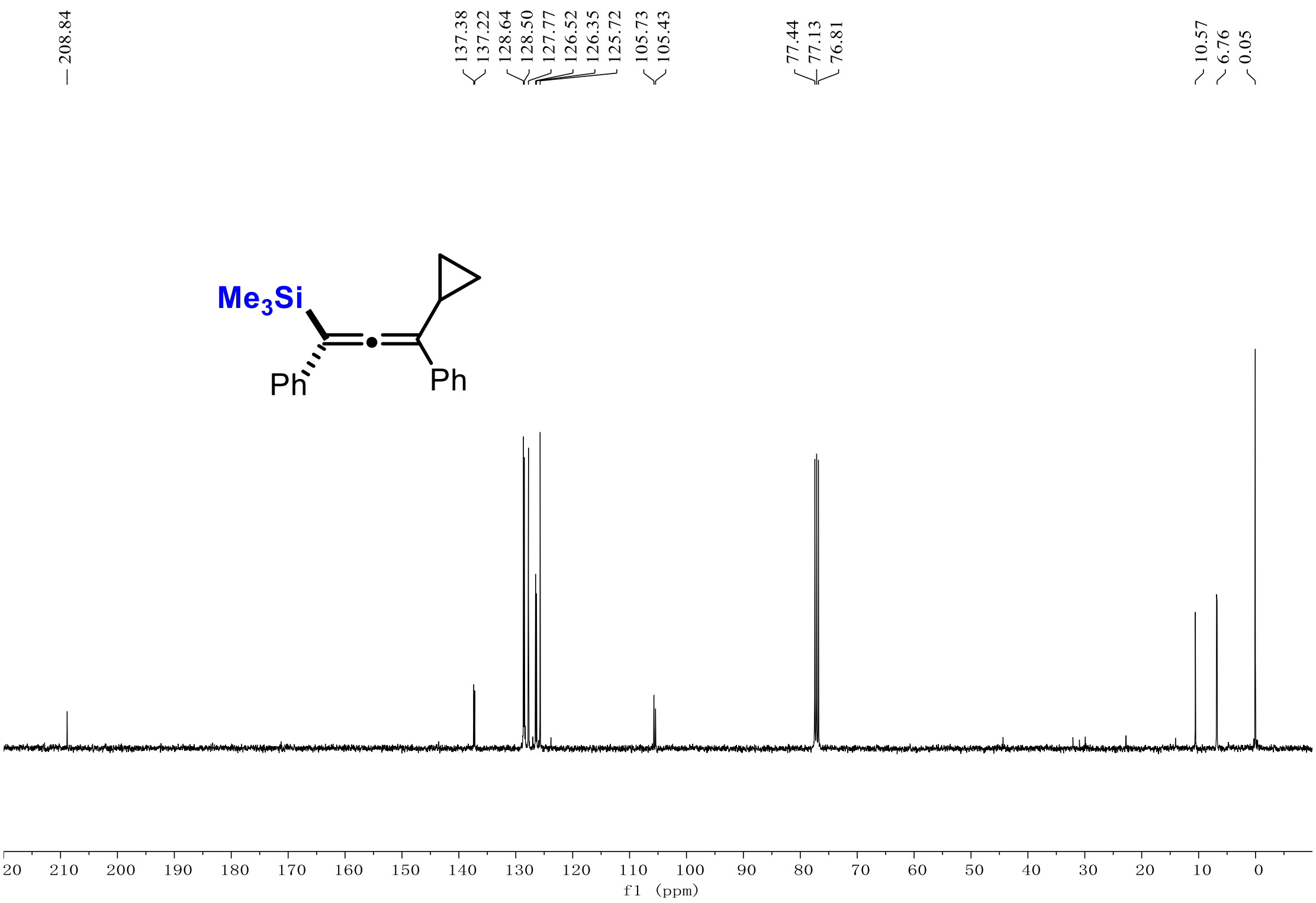

^{1}H NMR spectrum of 7d (600 MHz, $CDCl_3$)

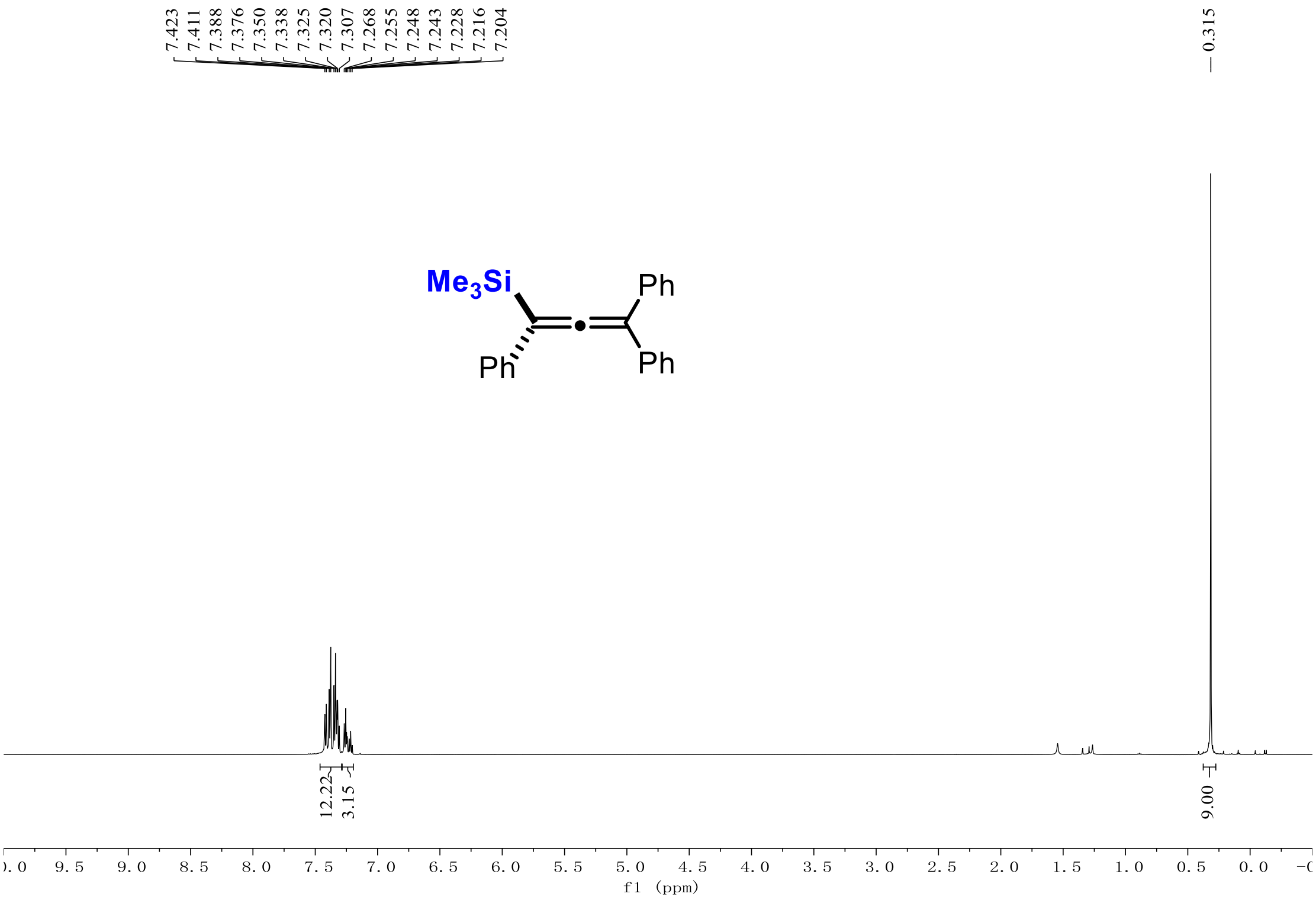

^{13}C NMR spectrum of 7d (101 MHz, $CDCl_3$)

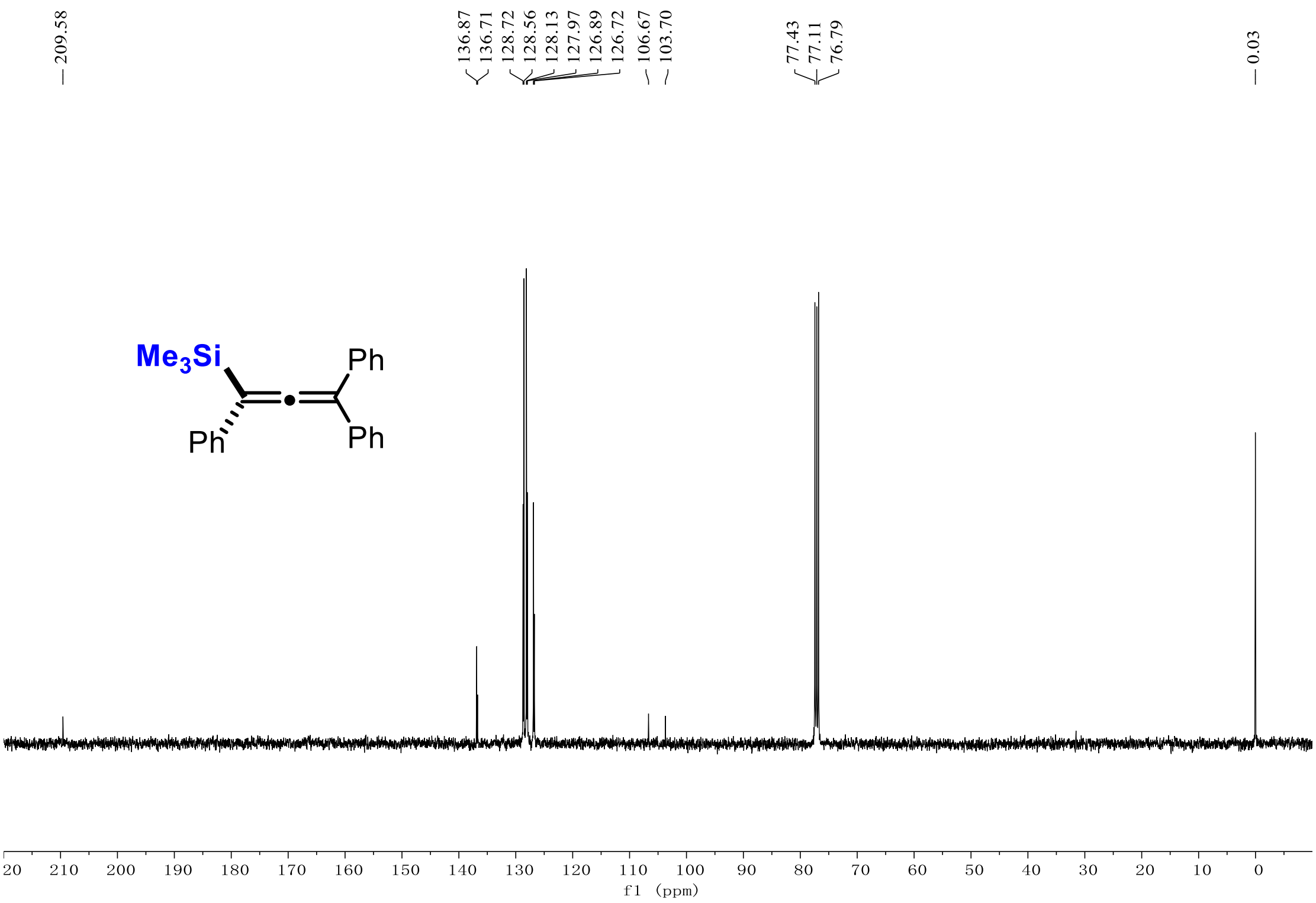

^{1}H NMR spectrum of 7e (400 MHz, $CDCl_3$)

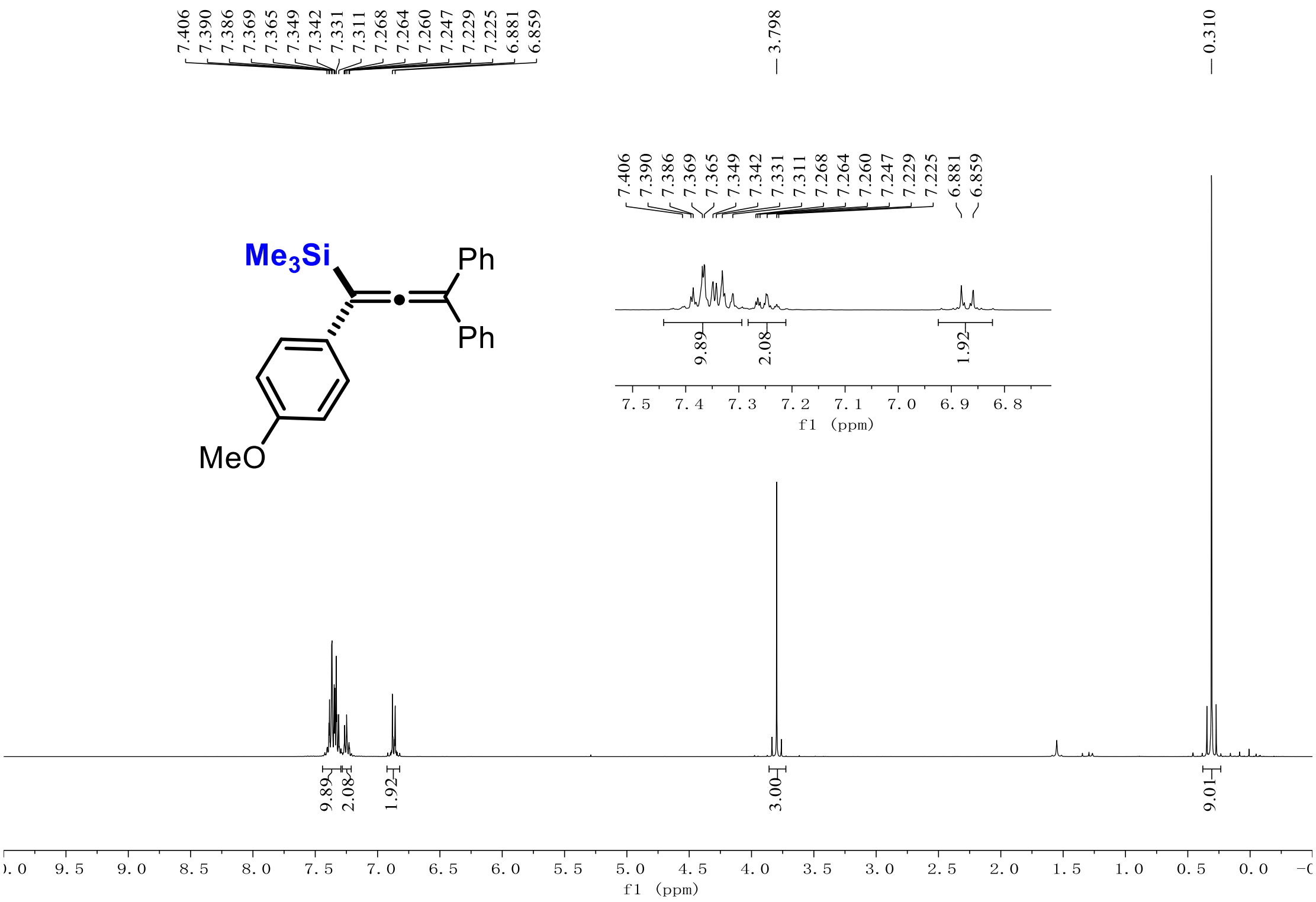

^{13}C NMR spectrum of 7e (101 MHz, $CDCl_3$)

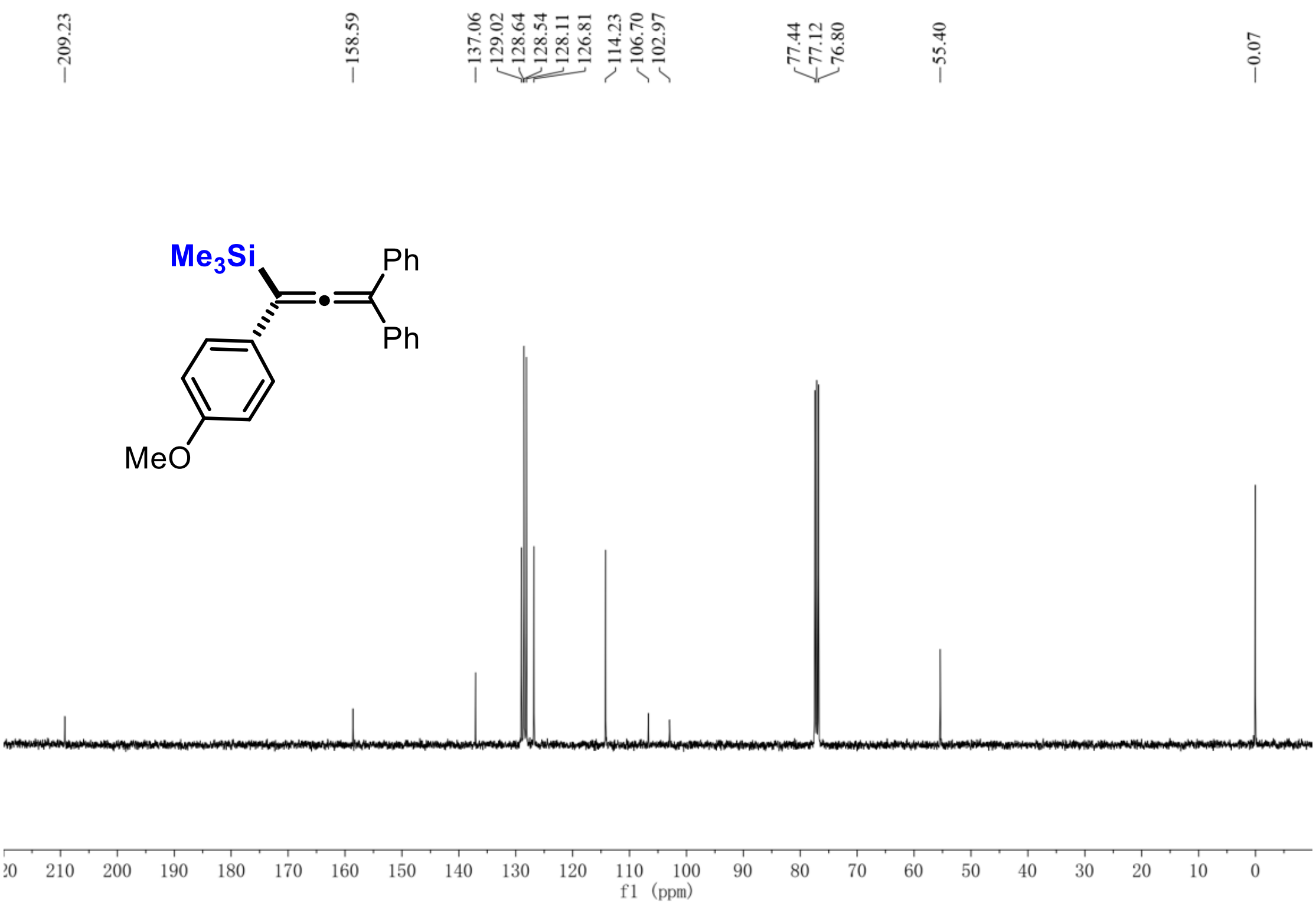

^{1}H NMR spectrum of 7f (400 MHz, $CDCl_3$)

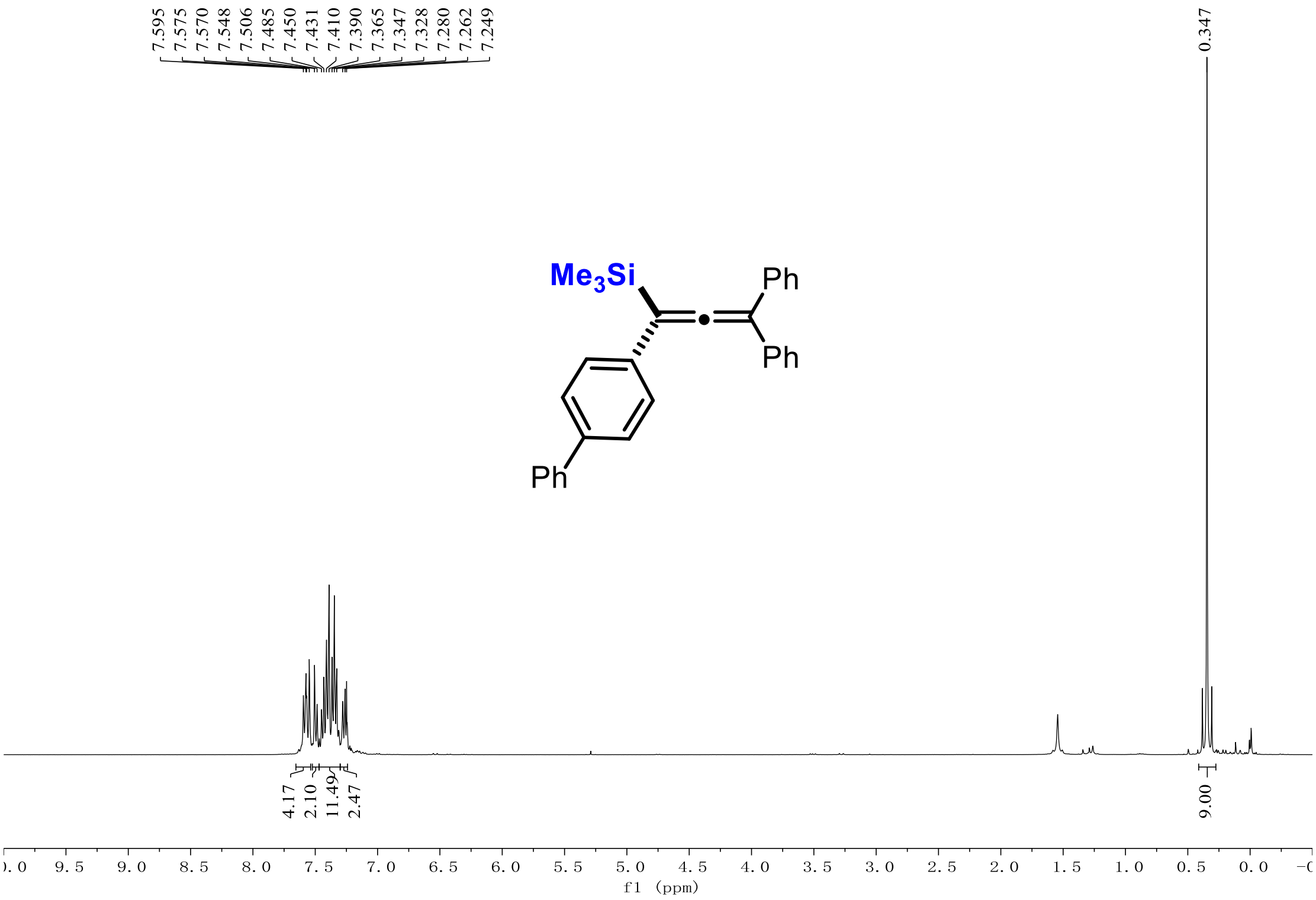

^{13}C NMR spectrum of 7f (101 MHz, $CDCl_3$)

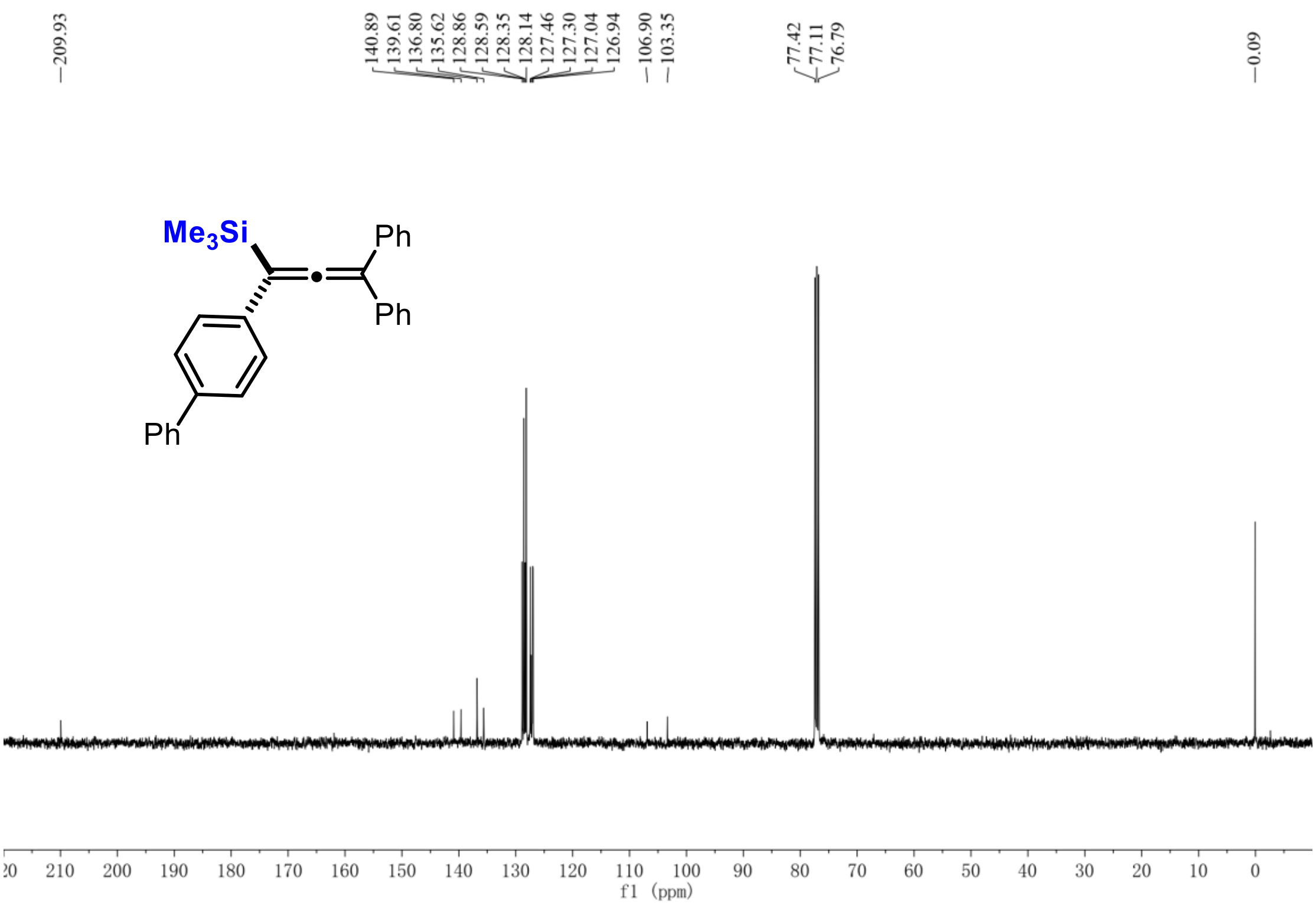

^{1}H NMR spectrum of 7g (400 MHz, $CDCl_3$)

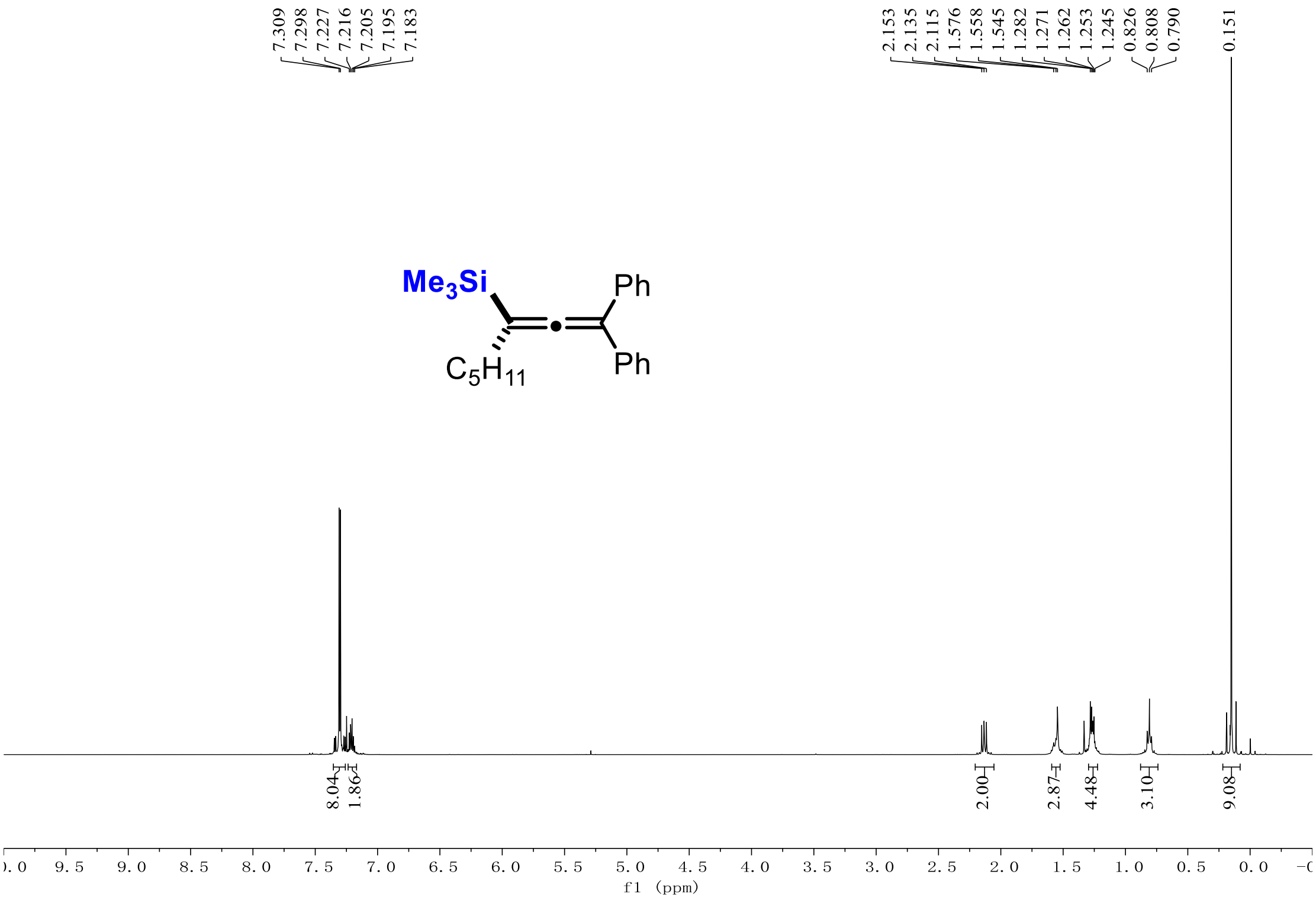

^{13}C NMR spectrum of 7g (101 MHz, $CDCl_3$)

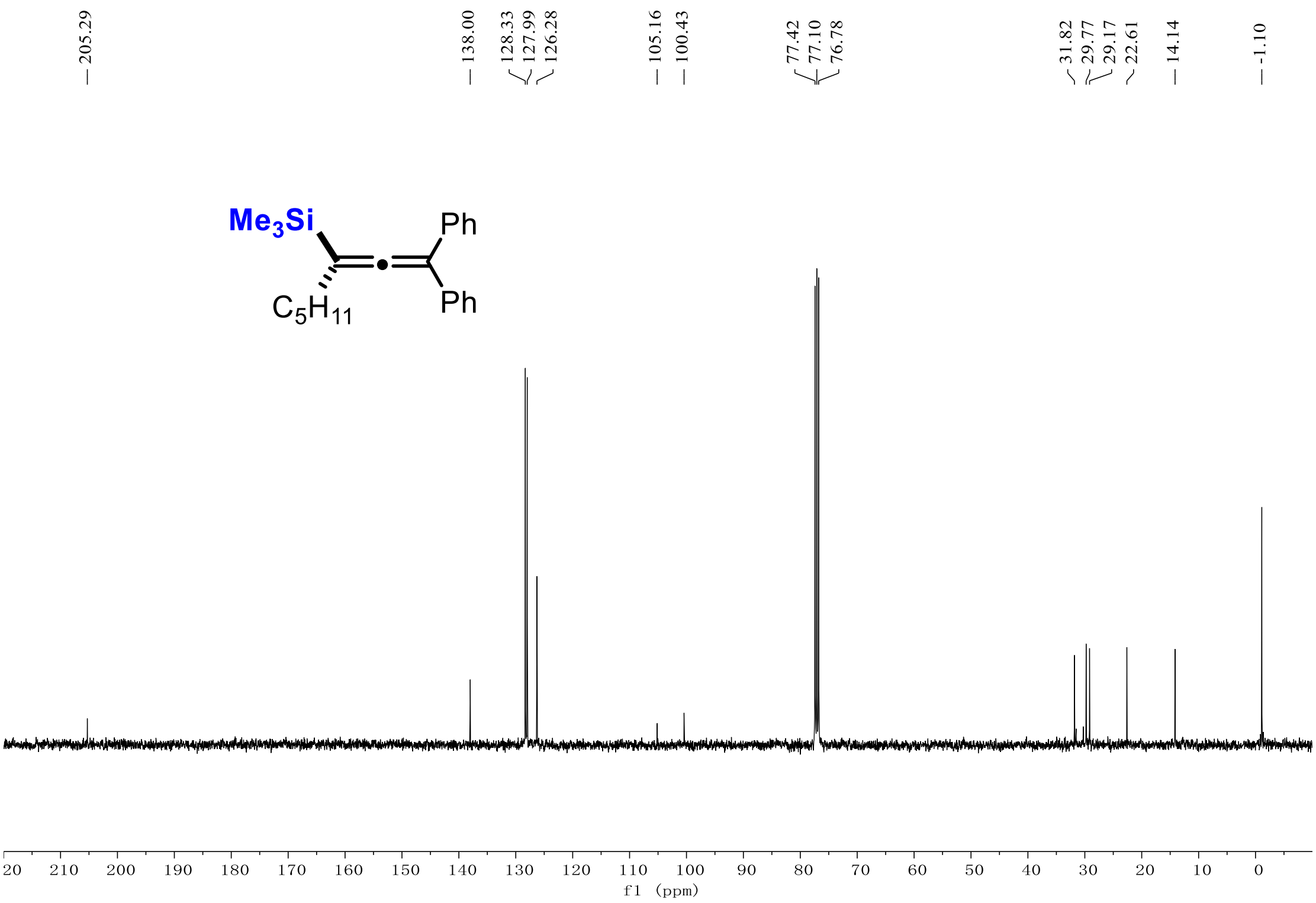

^{1}H NMR spectrum of 7h (400 MHz, $CDCl_3$)

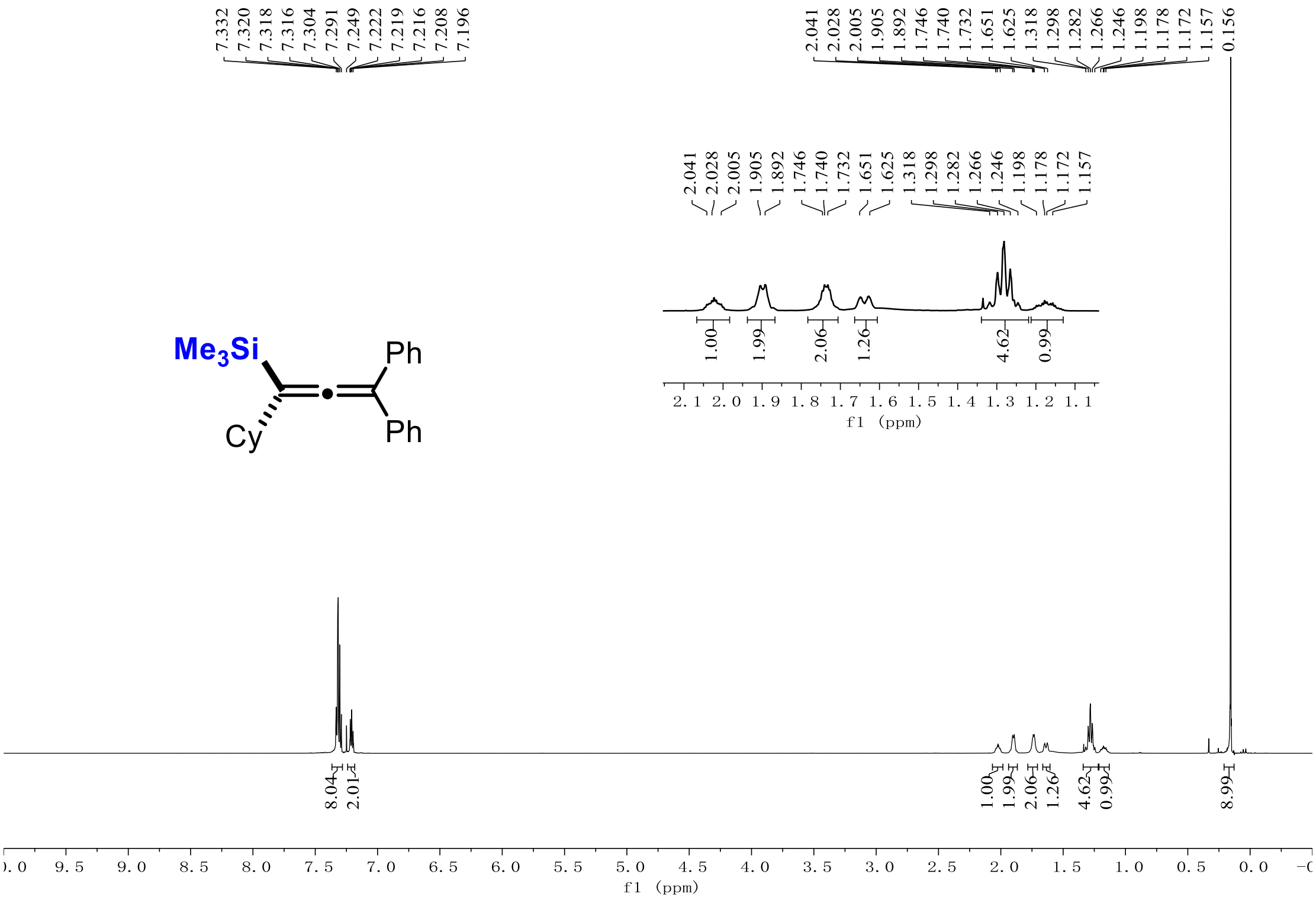

^{13}C NMR spectrum of 7h (101 MHz, $CDCl_3$)

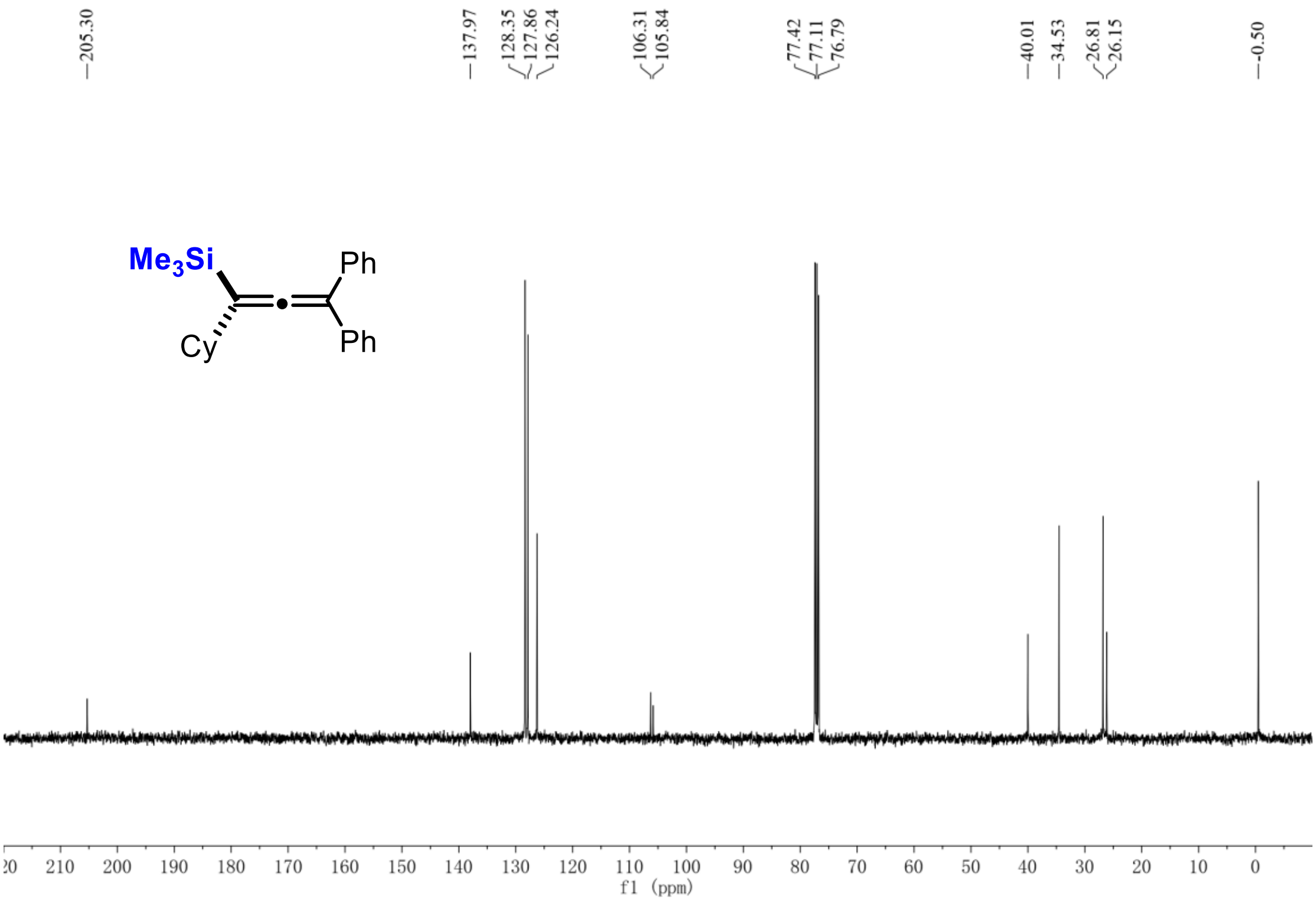

¹H NMR spectrum of 7i (400 MHz, $CDCl_3$)

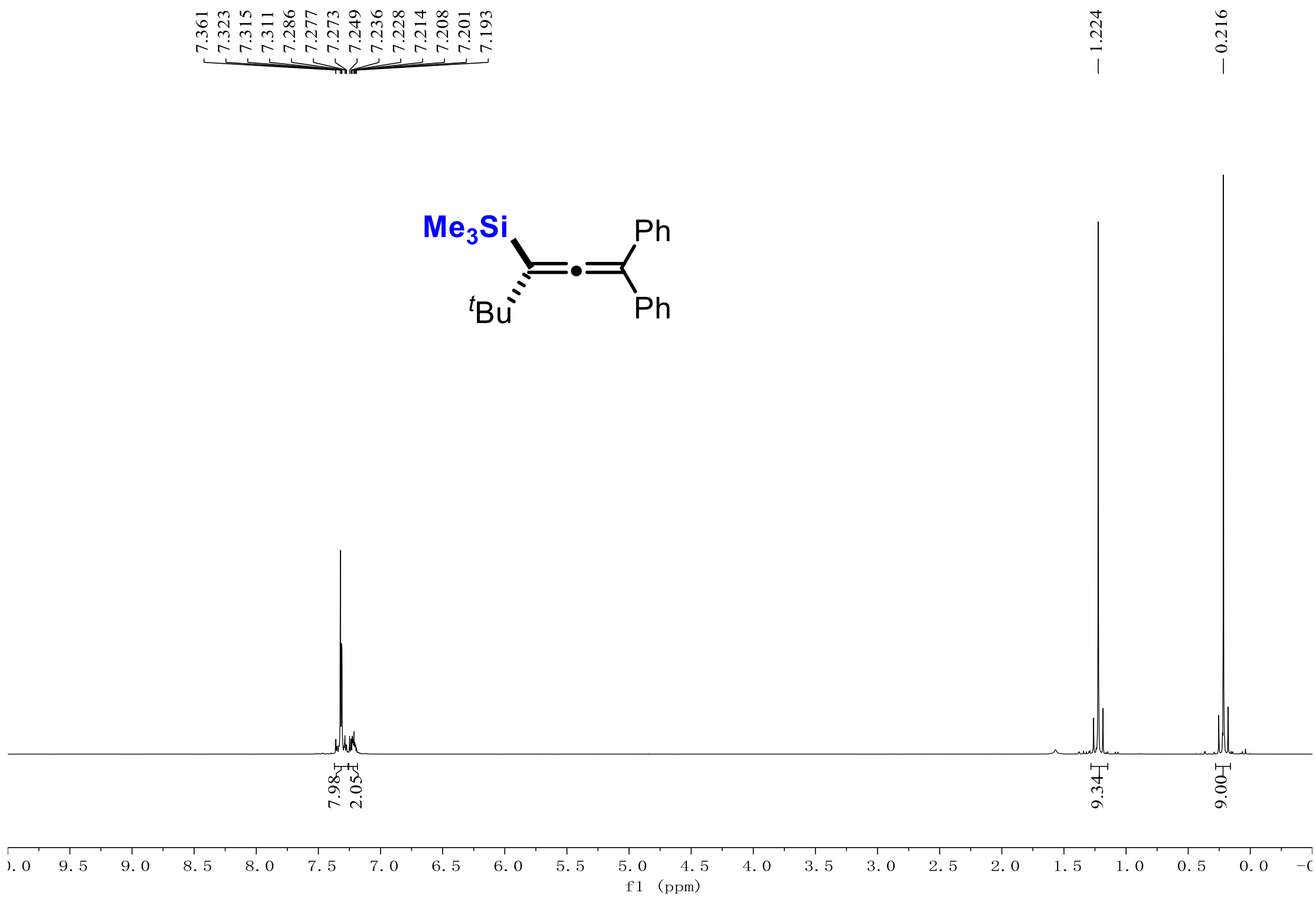

¹³C NMR spectrum of 7i (101 MHz, $CDCl_3$)

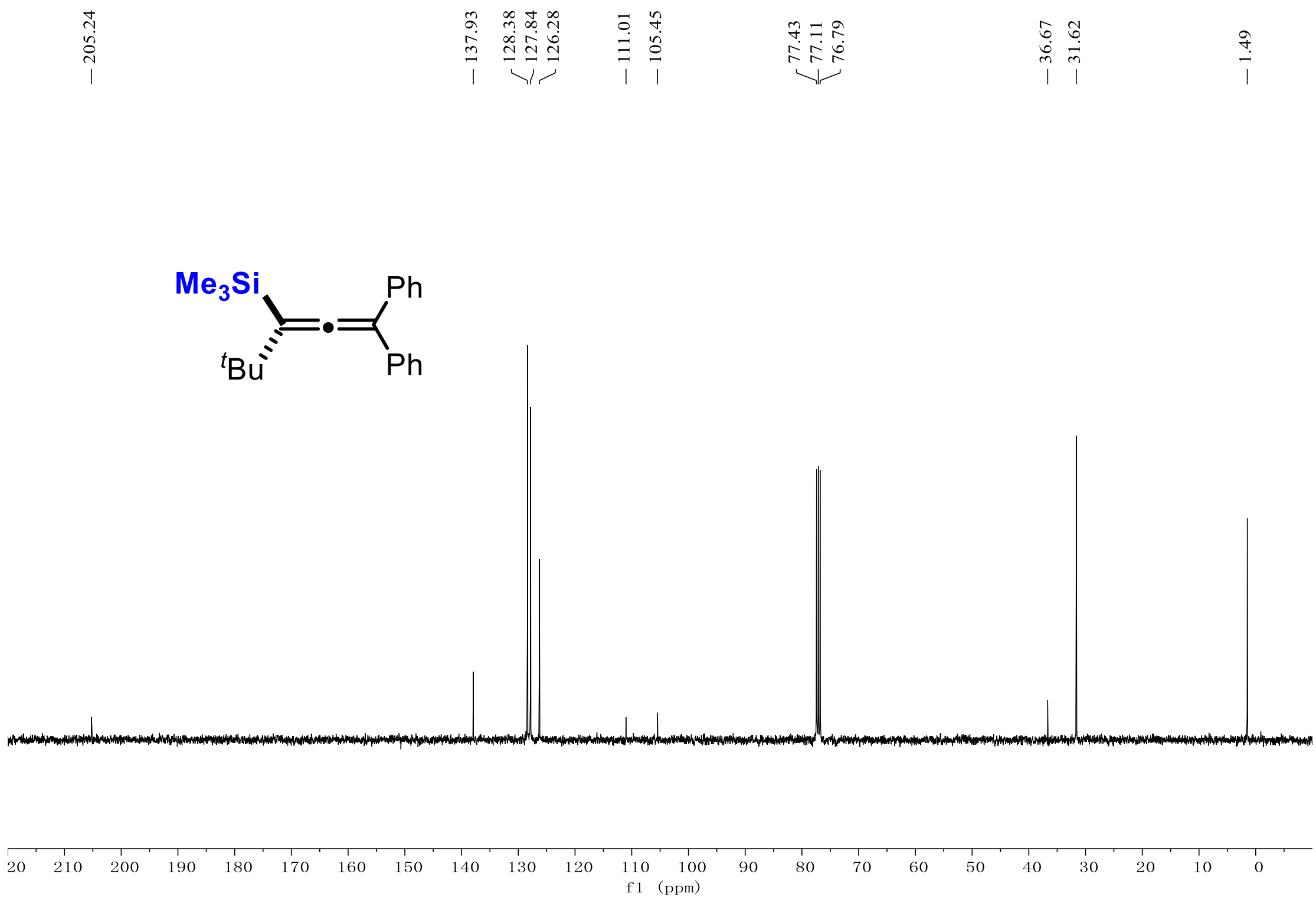

^{1}H NMR spectrum of 7j (400 MHz, $CDCl_3$)

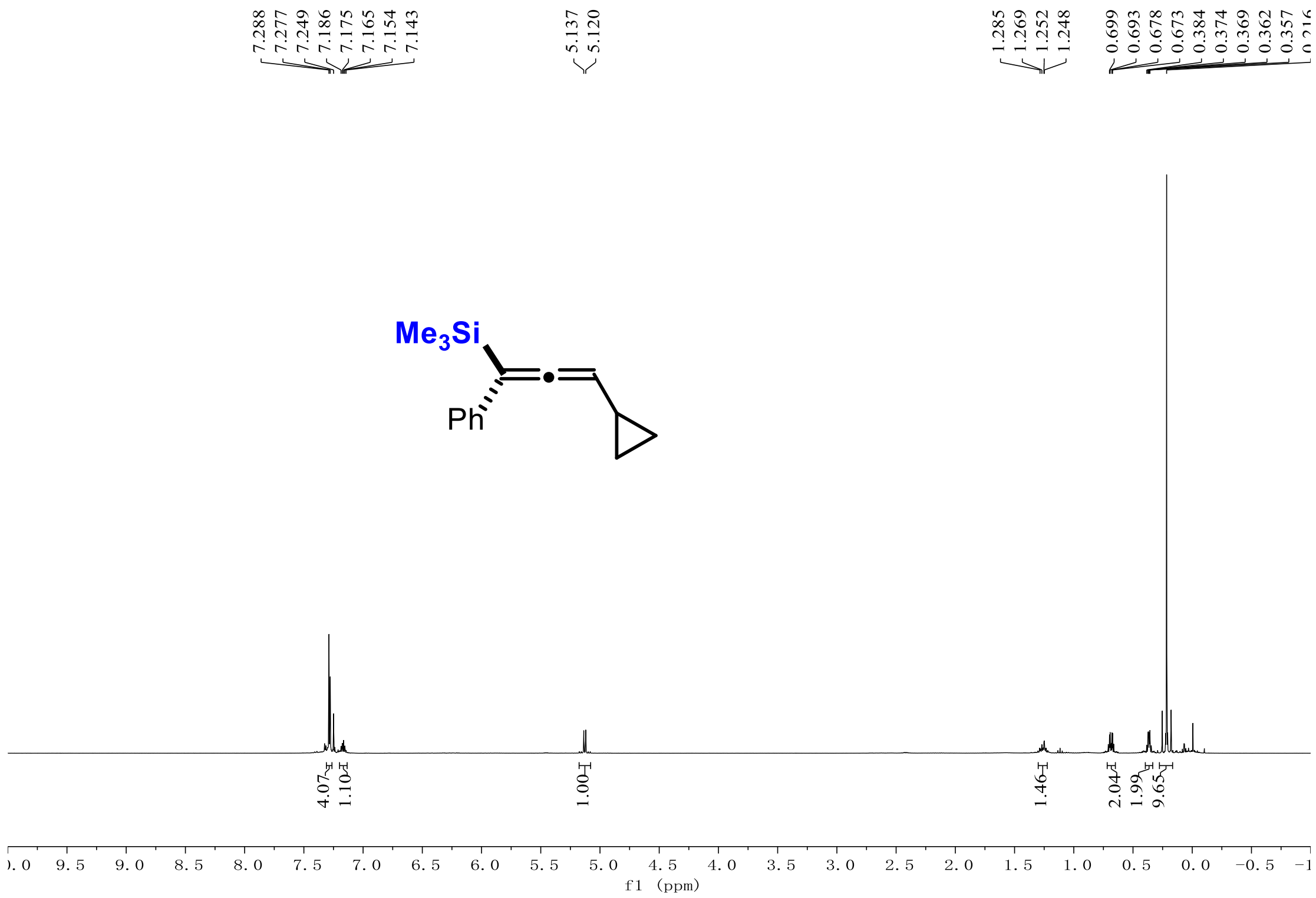

^{13}C NMR spectrum of 7j (101 MHz, $CDCl_3$)

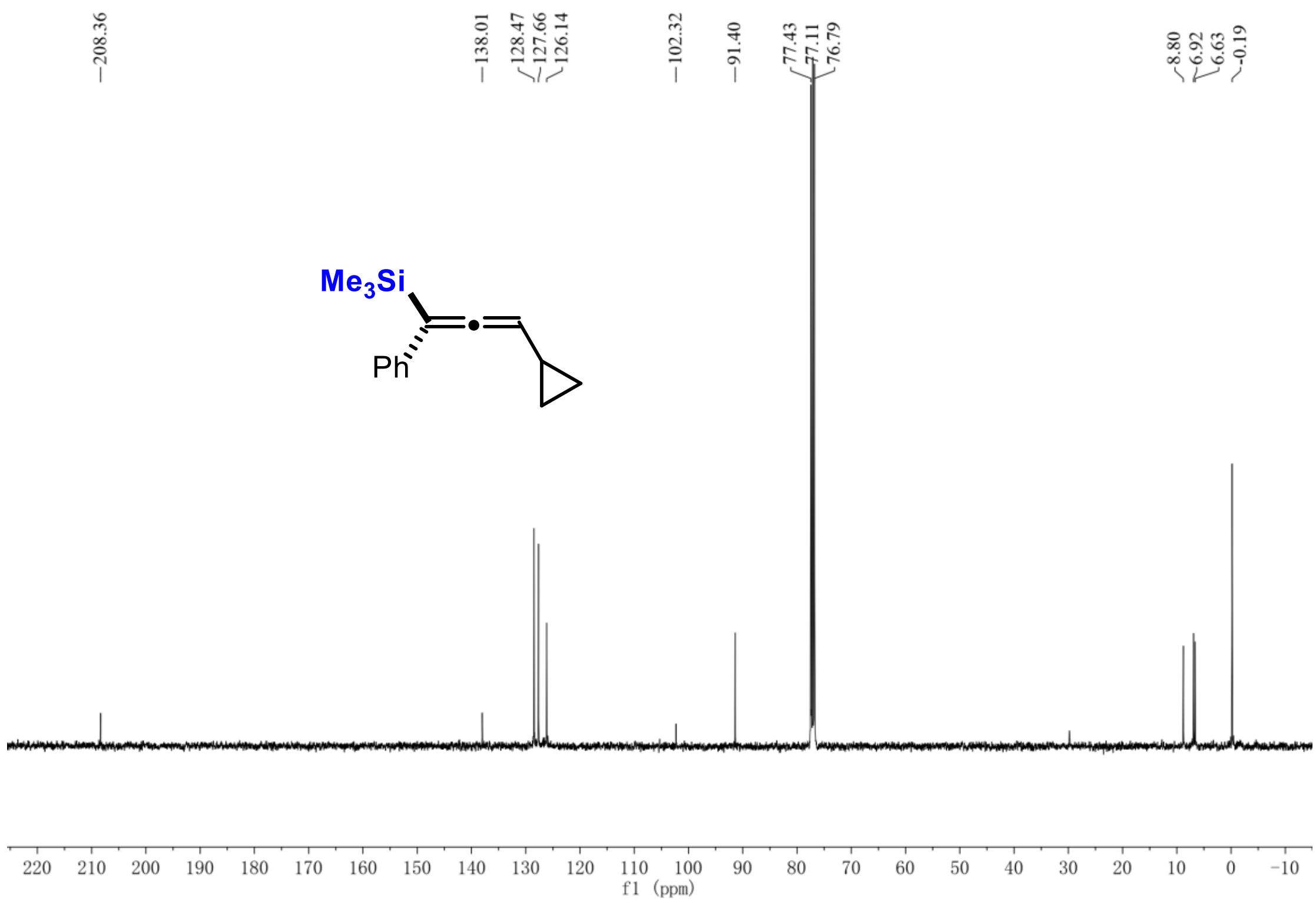

^{1}H NMR spectrum of 7k (400 MHz, $CDCl_3$)

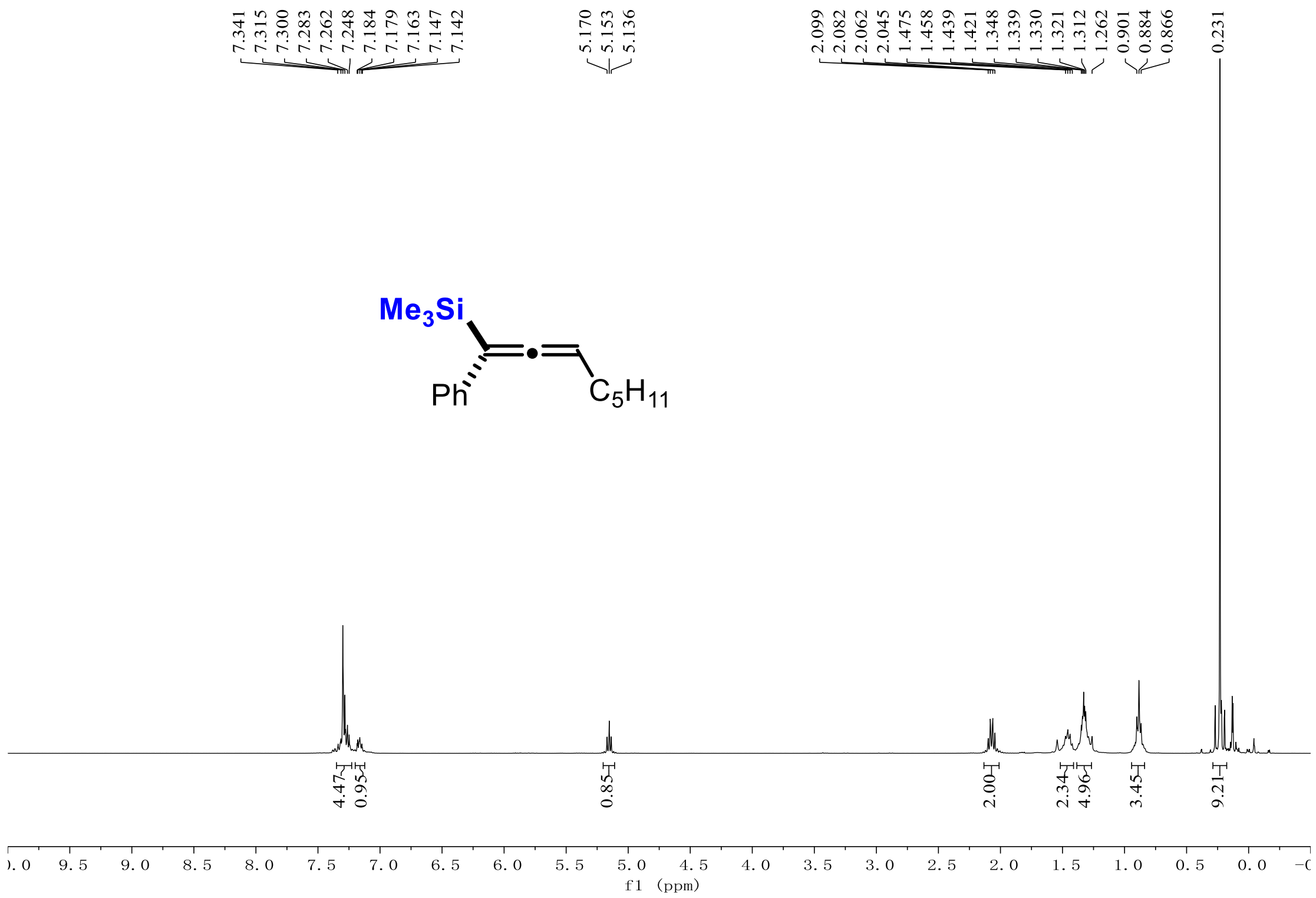

^{13}C NMR spectrum of 7k (101 MHz, $CDCl_3$)

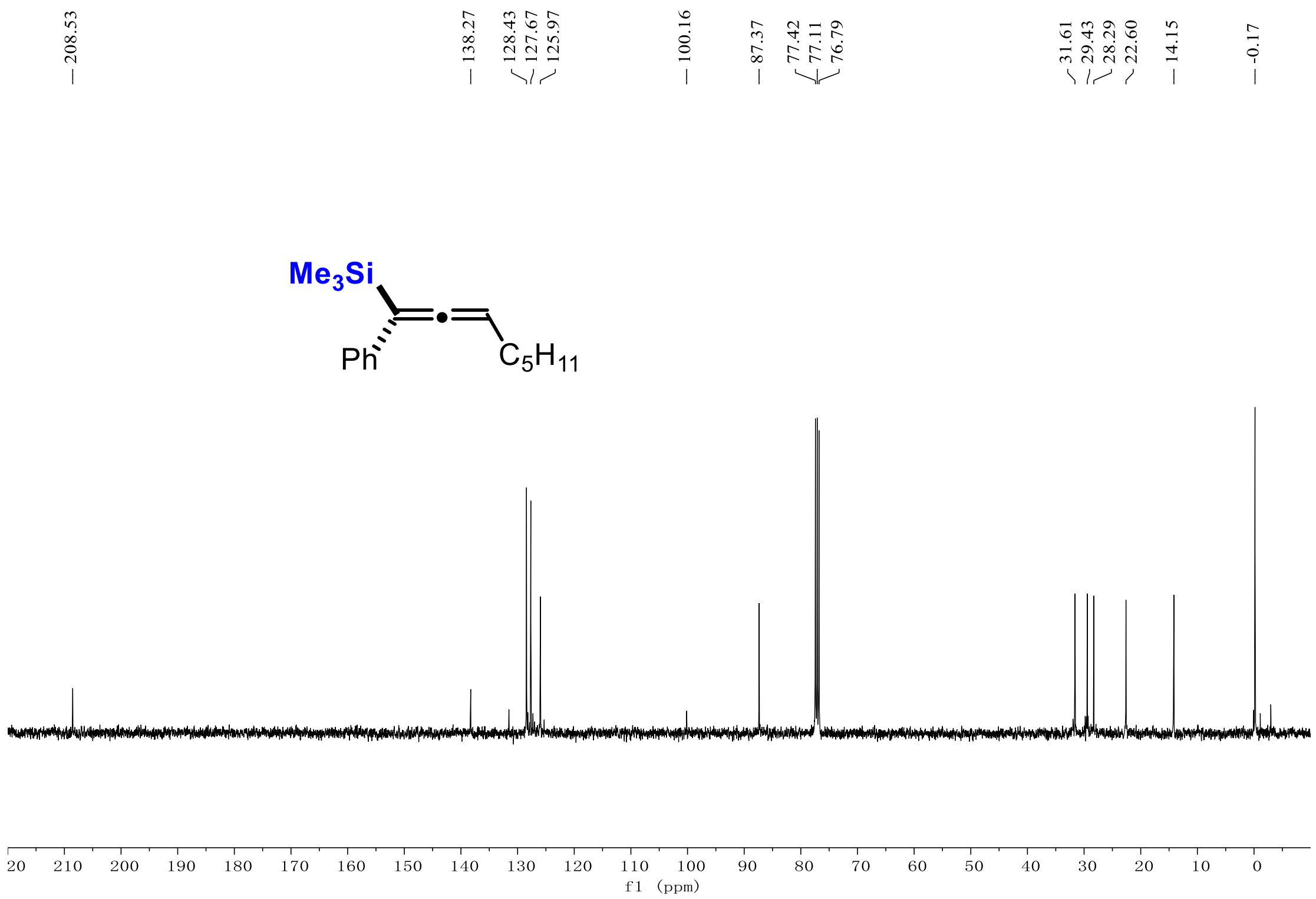

^{1}H NMR spectrum of 7l (400 MHz, $CDCl_3$)

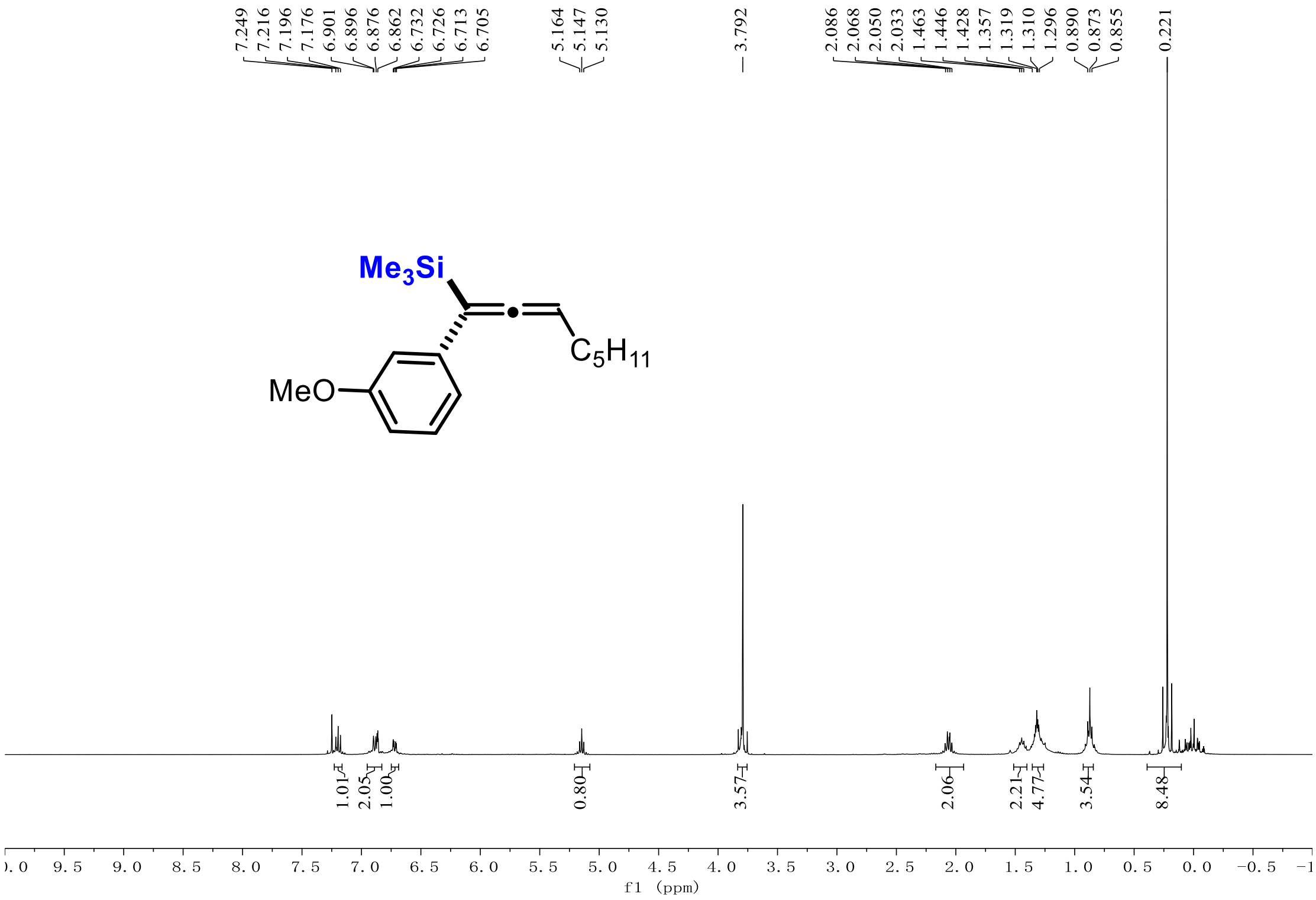

^{13}C NMR spectrum of 7l (101 MHz, $CDCl_3$)

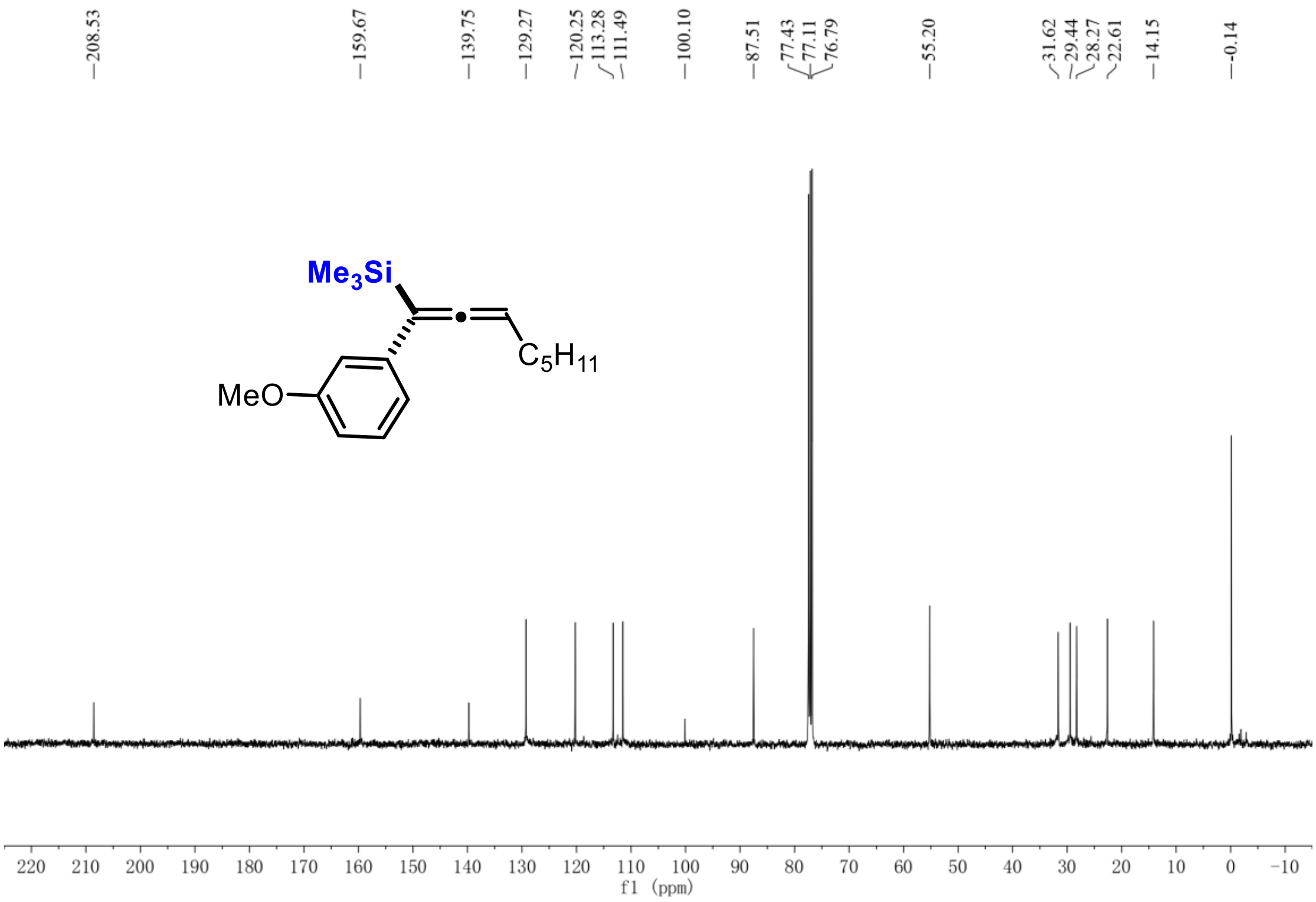

^{1}H NMR spectrum of 7m (400 MHz, $CDCl_3$)

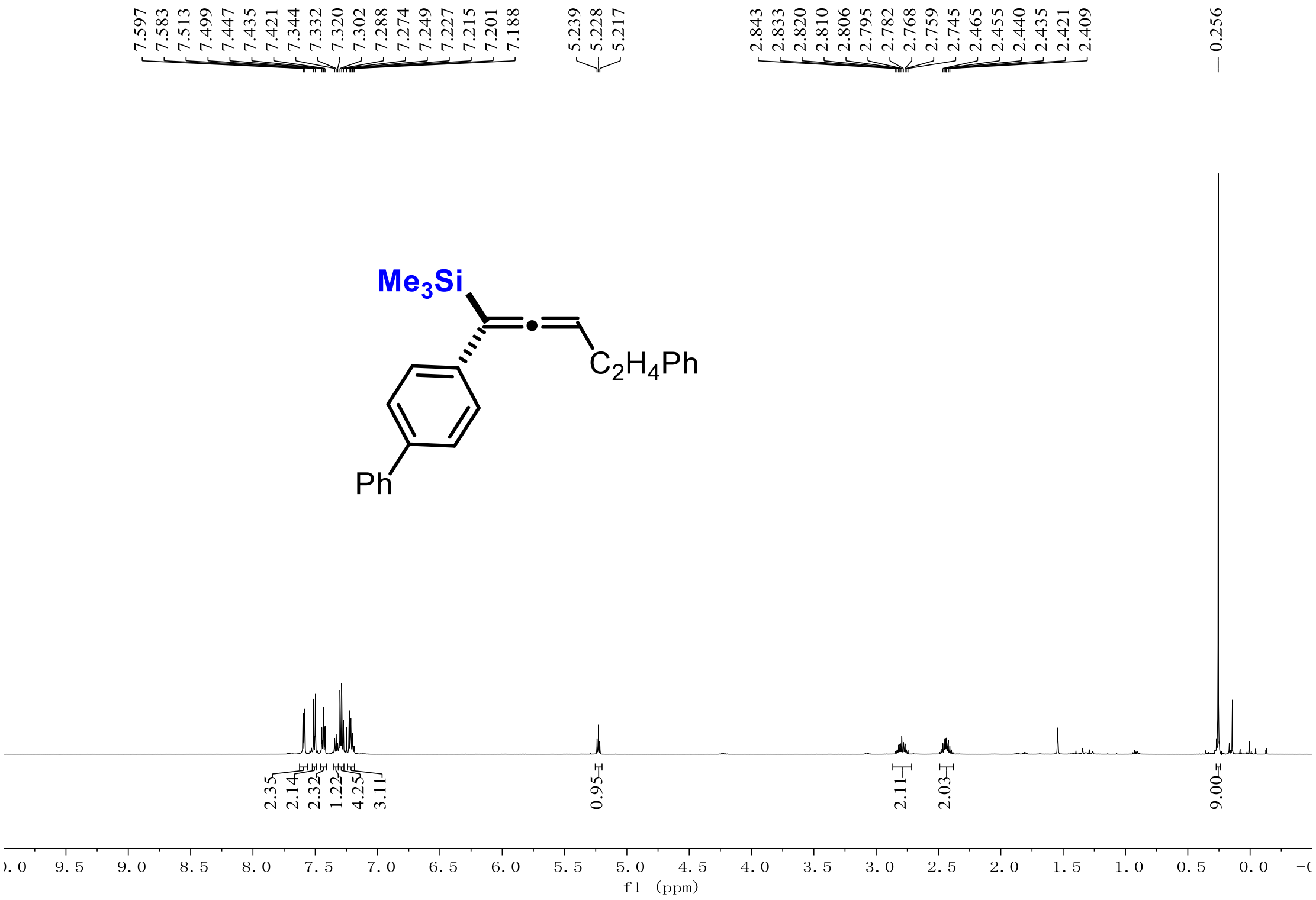

^{13}C NMR spectrum of 7m (101 MHz, $CDCl_3$)

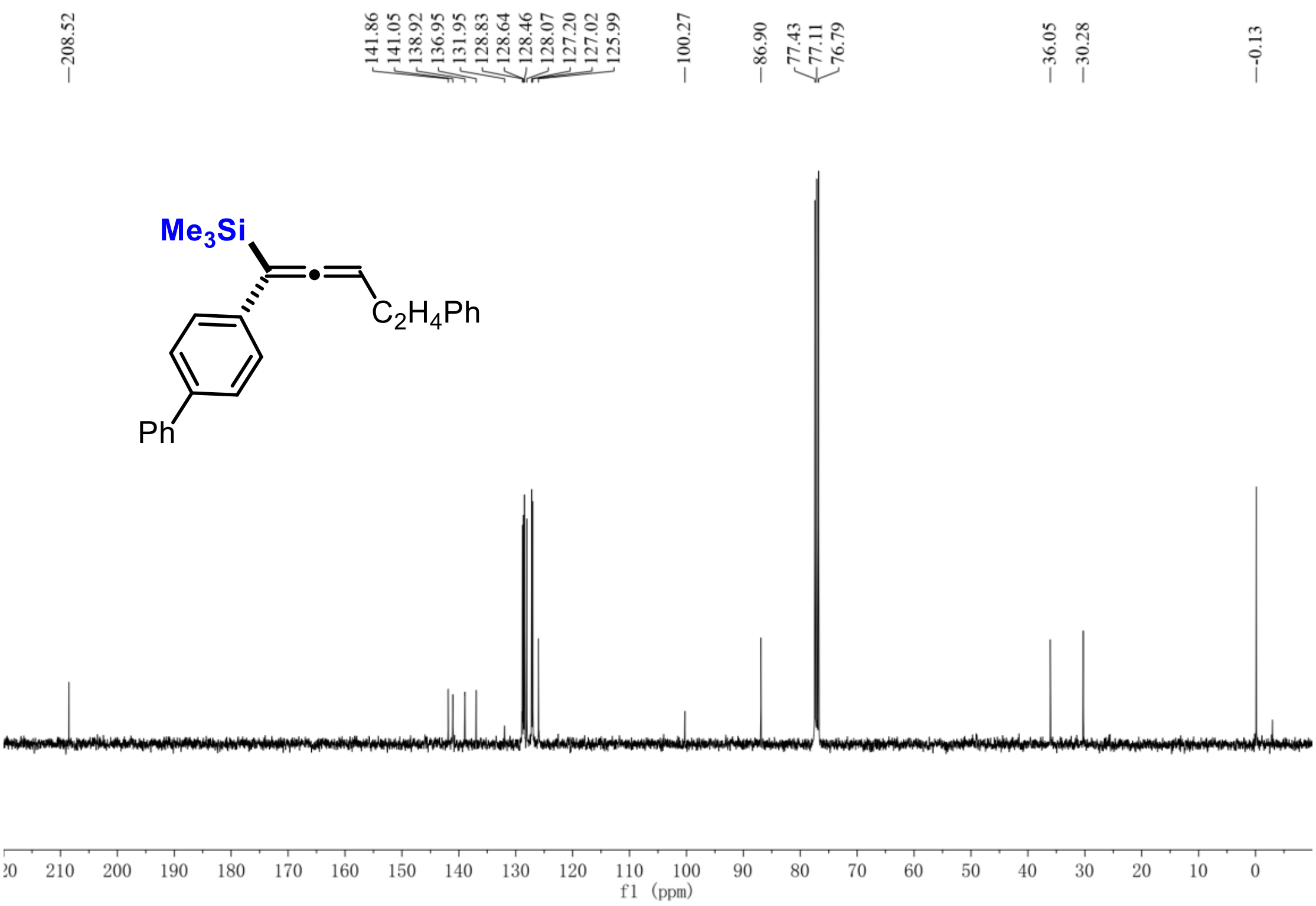

^{1}H NMR spectrum of 7n (400 MHz, $CDCl_3$)

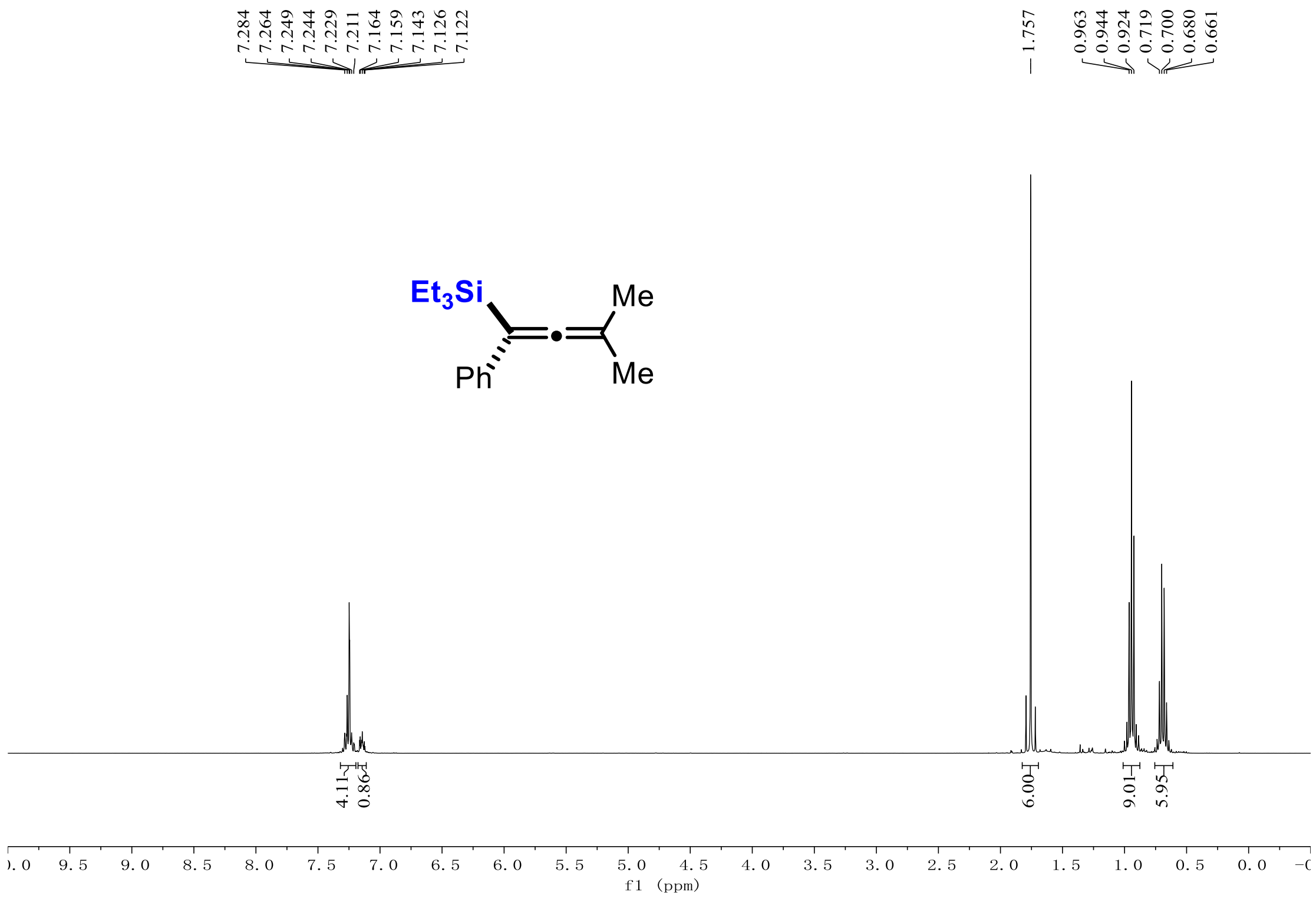

^{13}C NMR spectrum of 7n (101 MHz, $CDCl_3$)

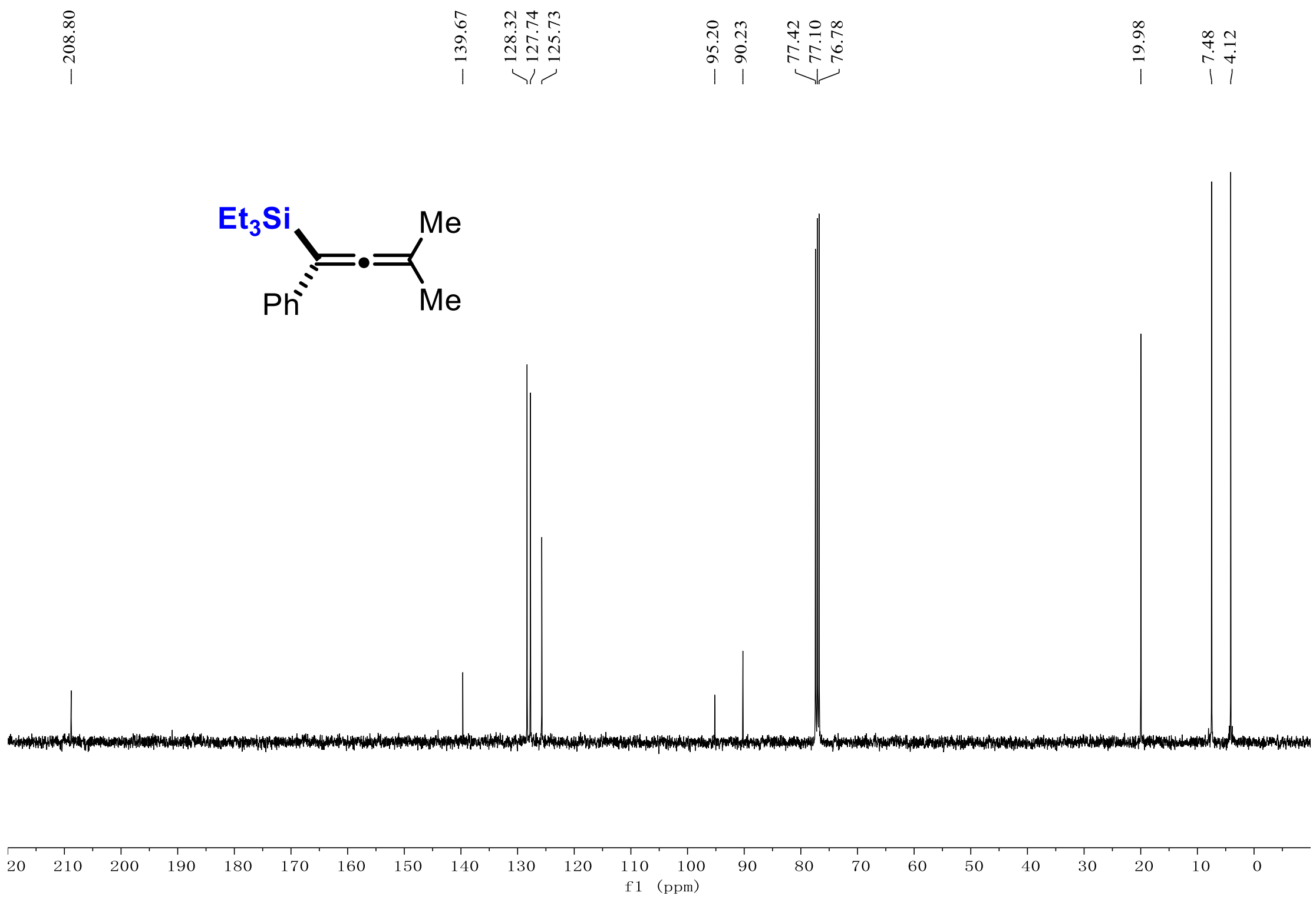

^{1}H NMR spectrum of 7o (400 MHz, $CDCl_3$)

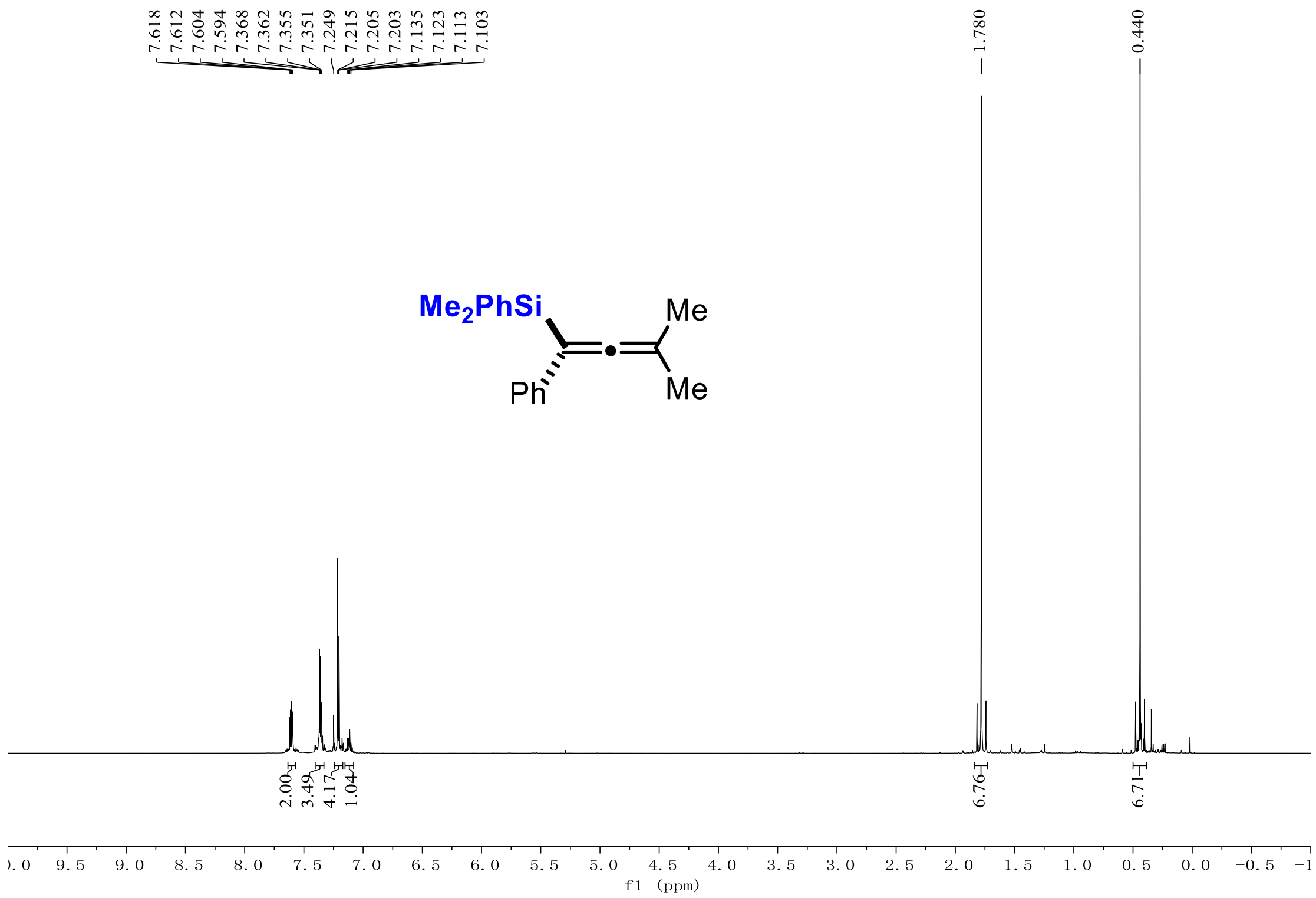

^{13}C NMR spectrum of 7o (101 MHz, $CDCl_3$)

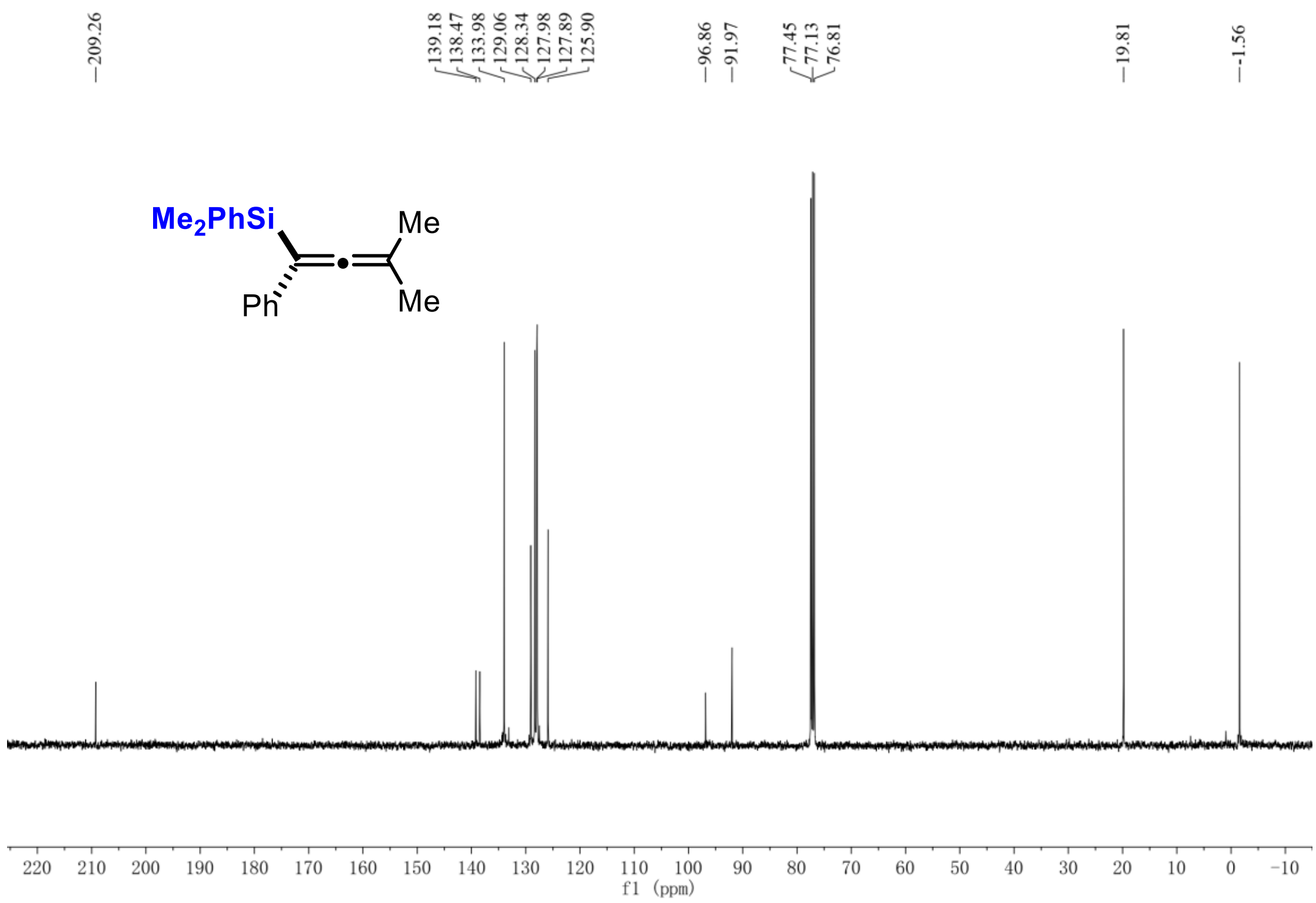

^{1}H NMR spectrum of 7p (400 MHz, $CDCl_3$)

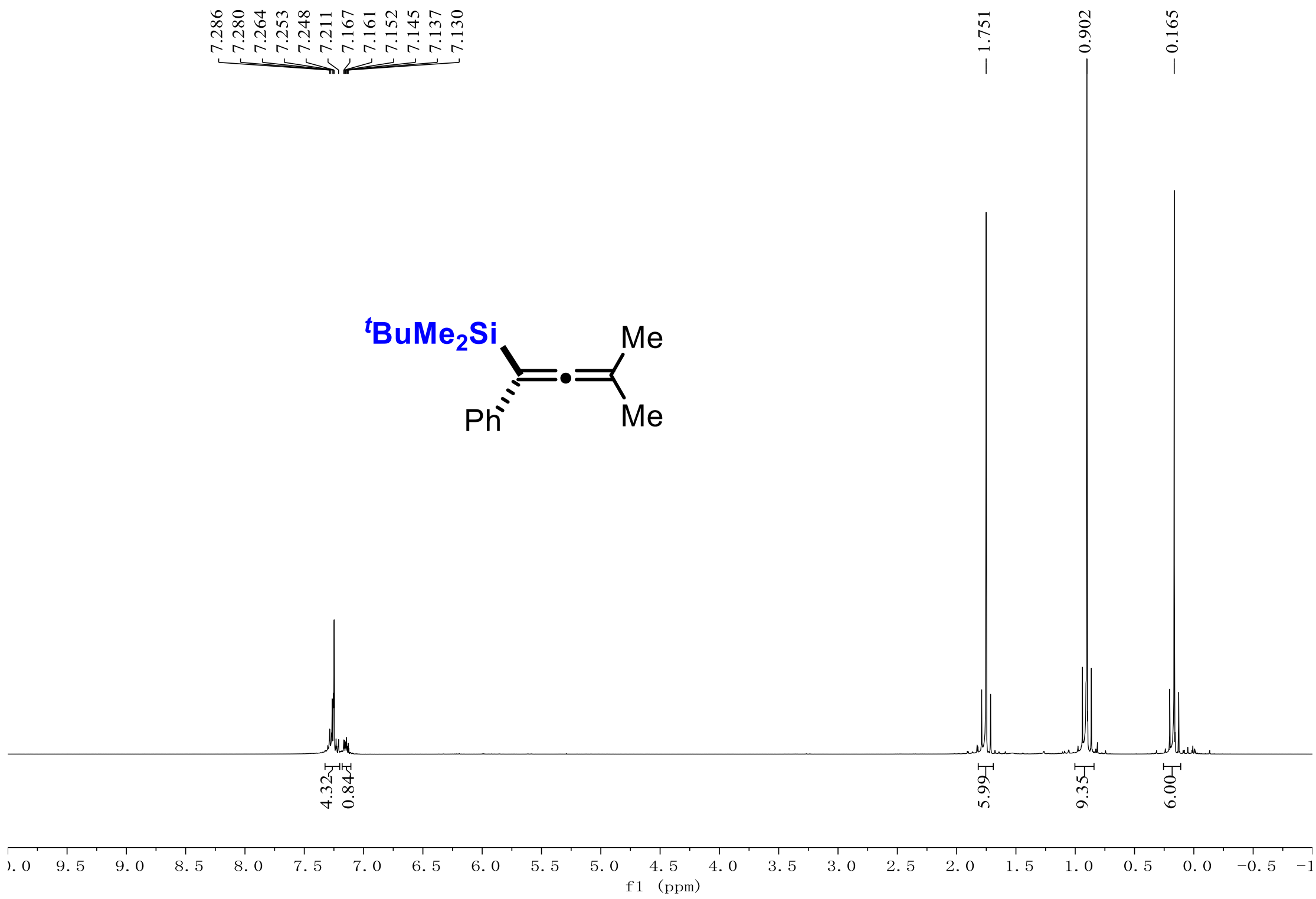

^{13}C NMR spectrum of 7p (101 MHz, $CDCl_3$)

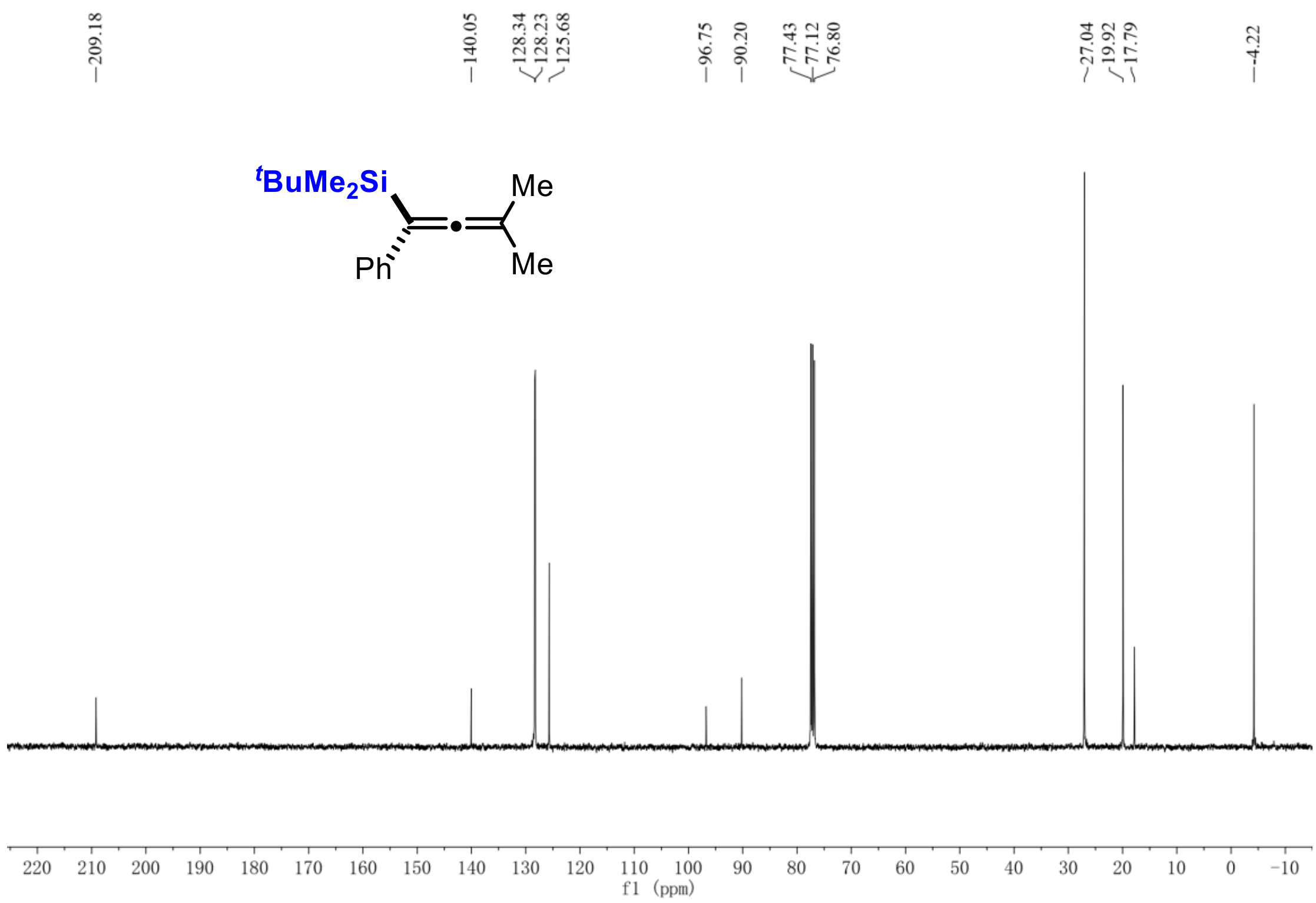

^{1}H NMR spectrum of 7q (400 MHz, $CDCl_3$)

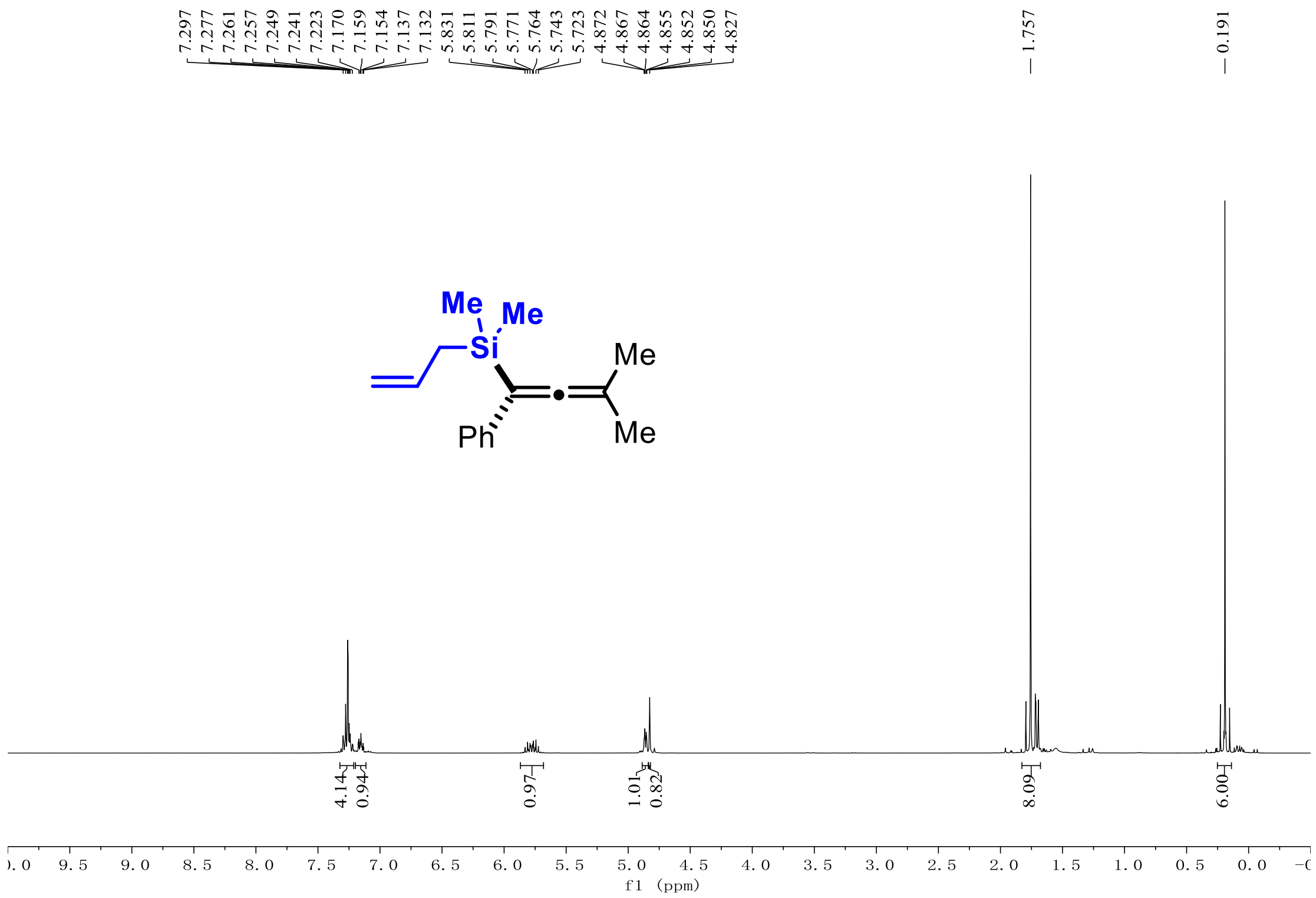

^{13}C NMR spectrum of 7q (101 MHz, $CDCl_3$)

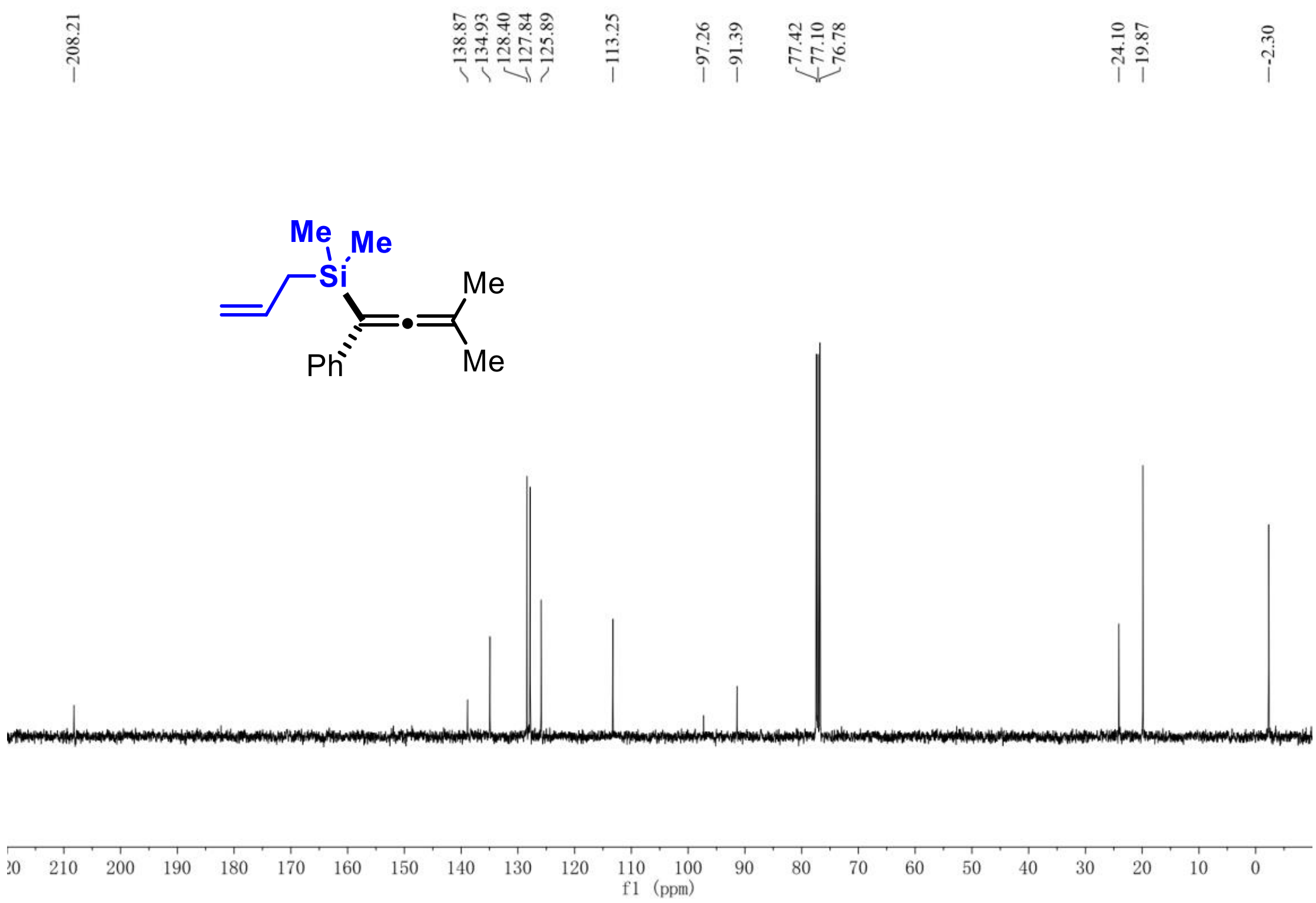

^{1}H NMR spectrum of 7r (400 MHz, $CDCl_3$)

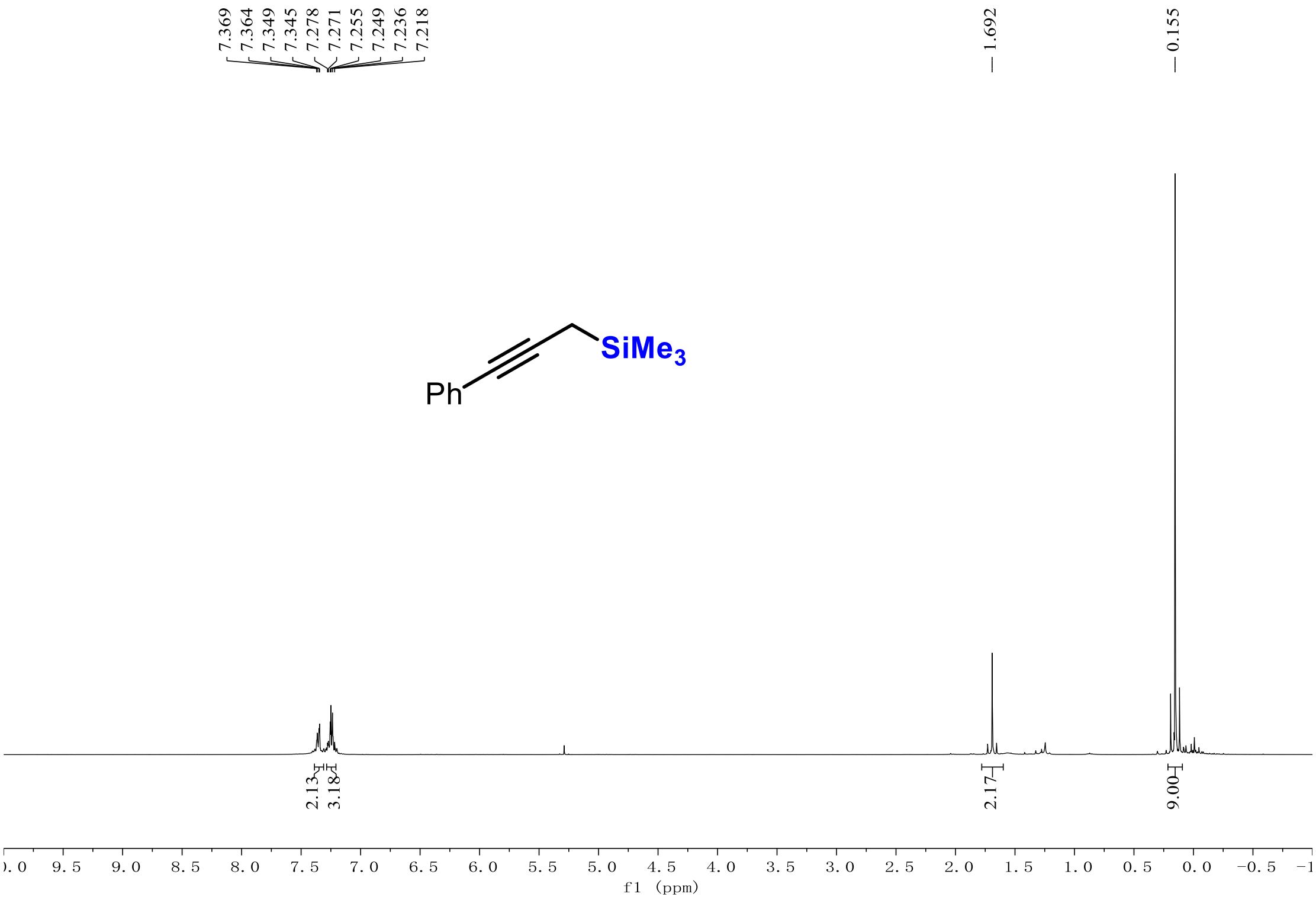

^{13}C NMR spectrum of 7r (101 MHz, $CDCl_3$)

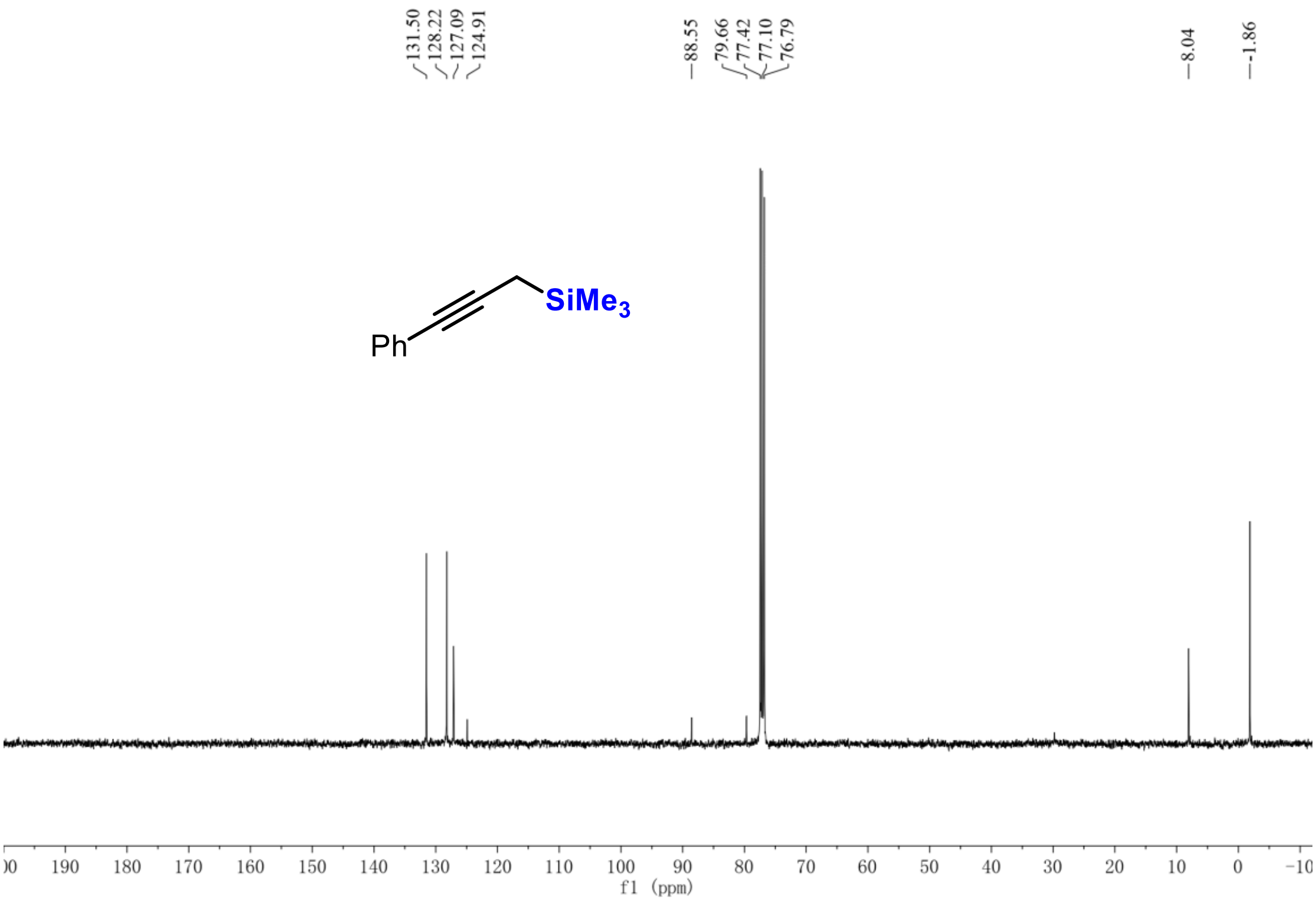

^{1}H NMR spectrum of 9a (400 MHz, $CDCl_3$)

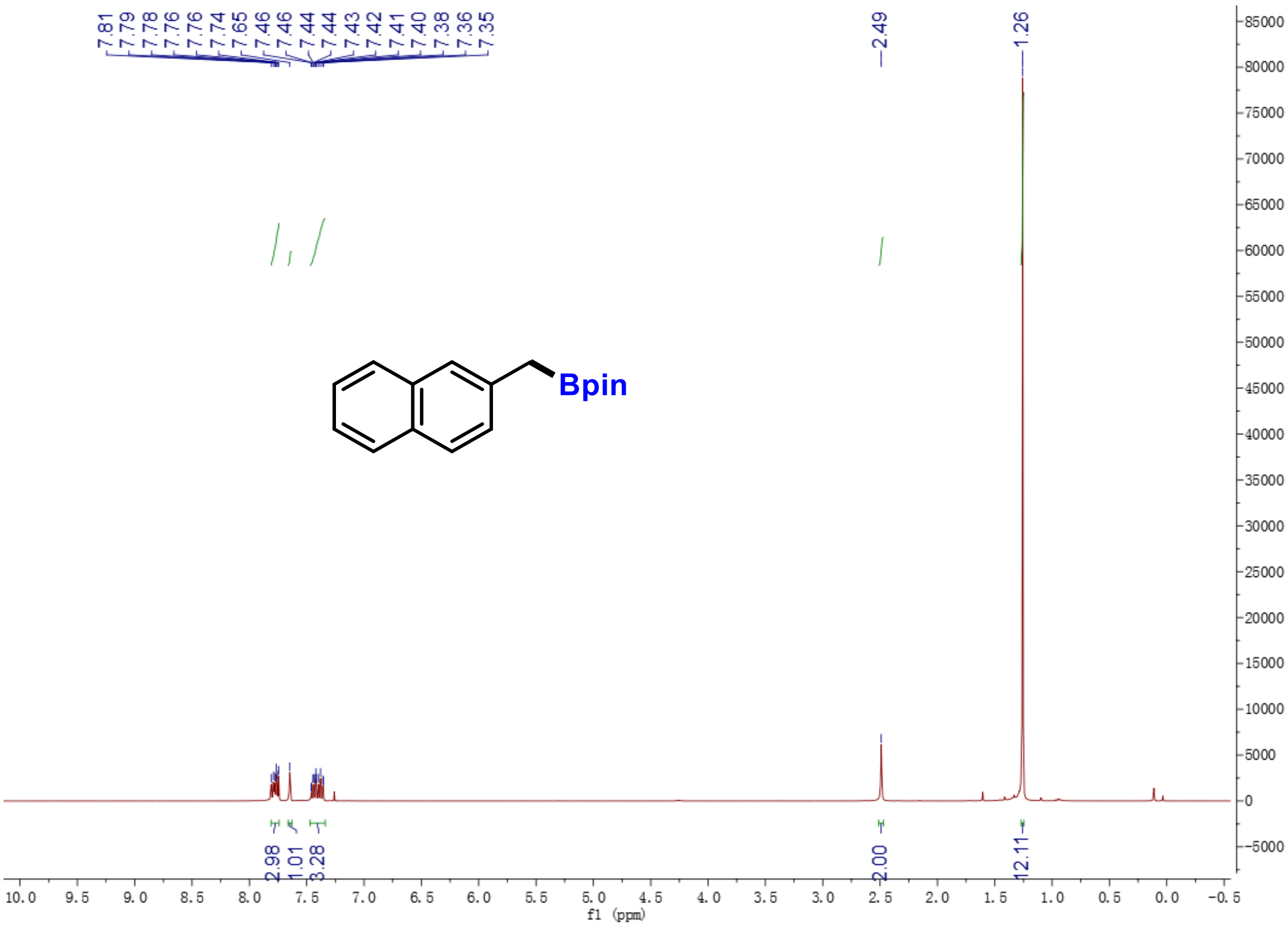

^{13}C NMR spectrum of 9a (101 MHz, $CDCl_3$)

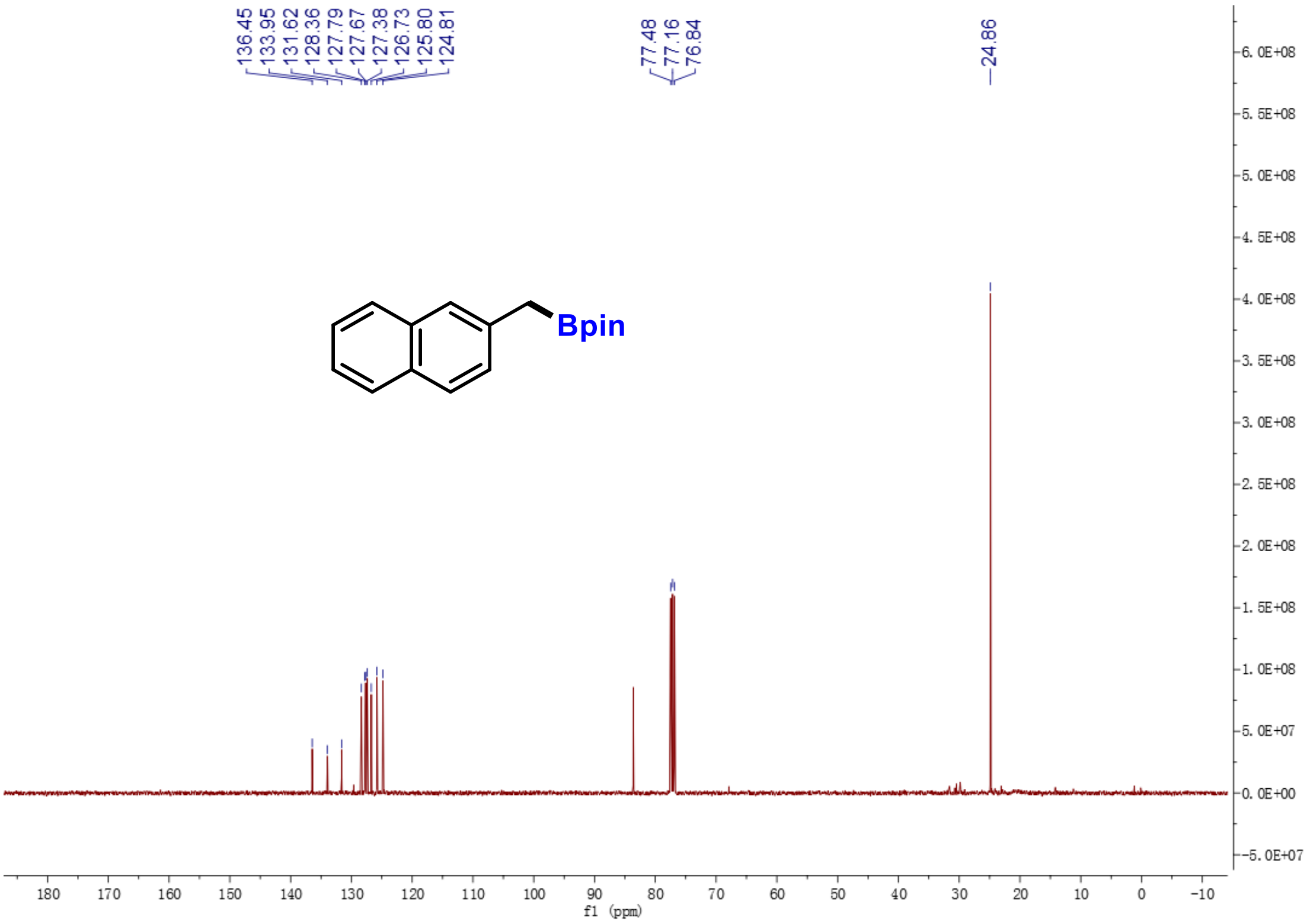

^{1}H NMR spectrum of 9b (400 MHz, $CDCl_3$)

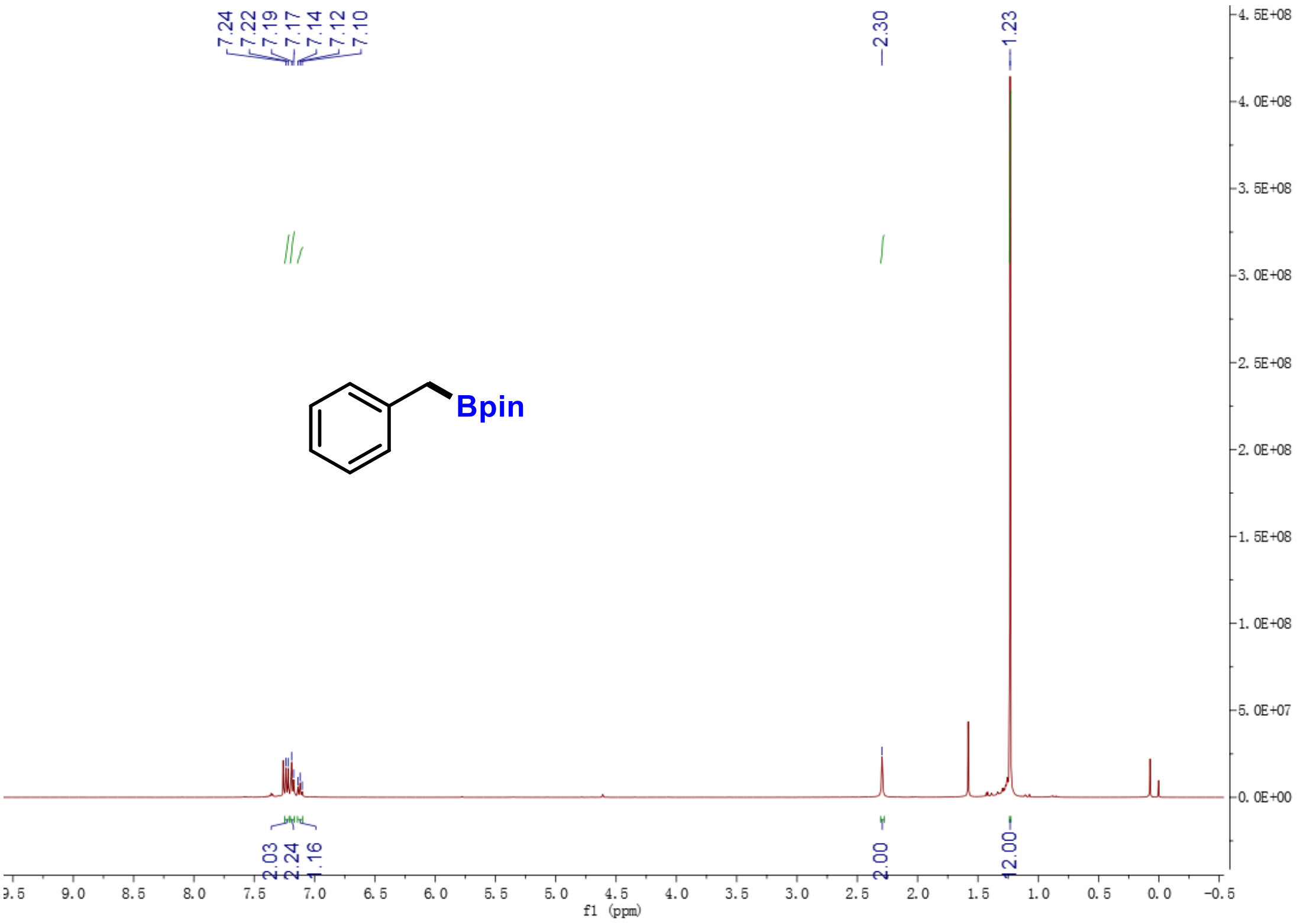

^{13}C NMR spectrum of 9b (101 MHz, $CDCl_3$)

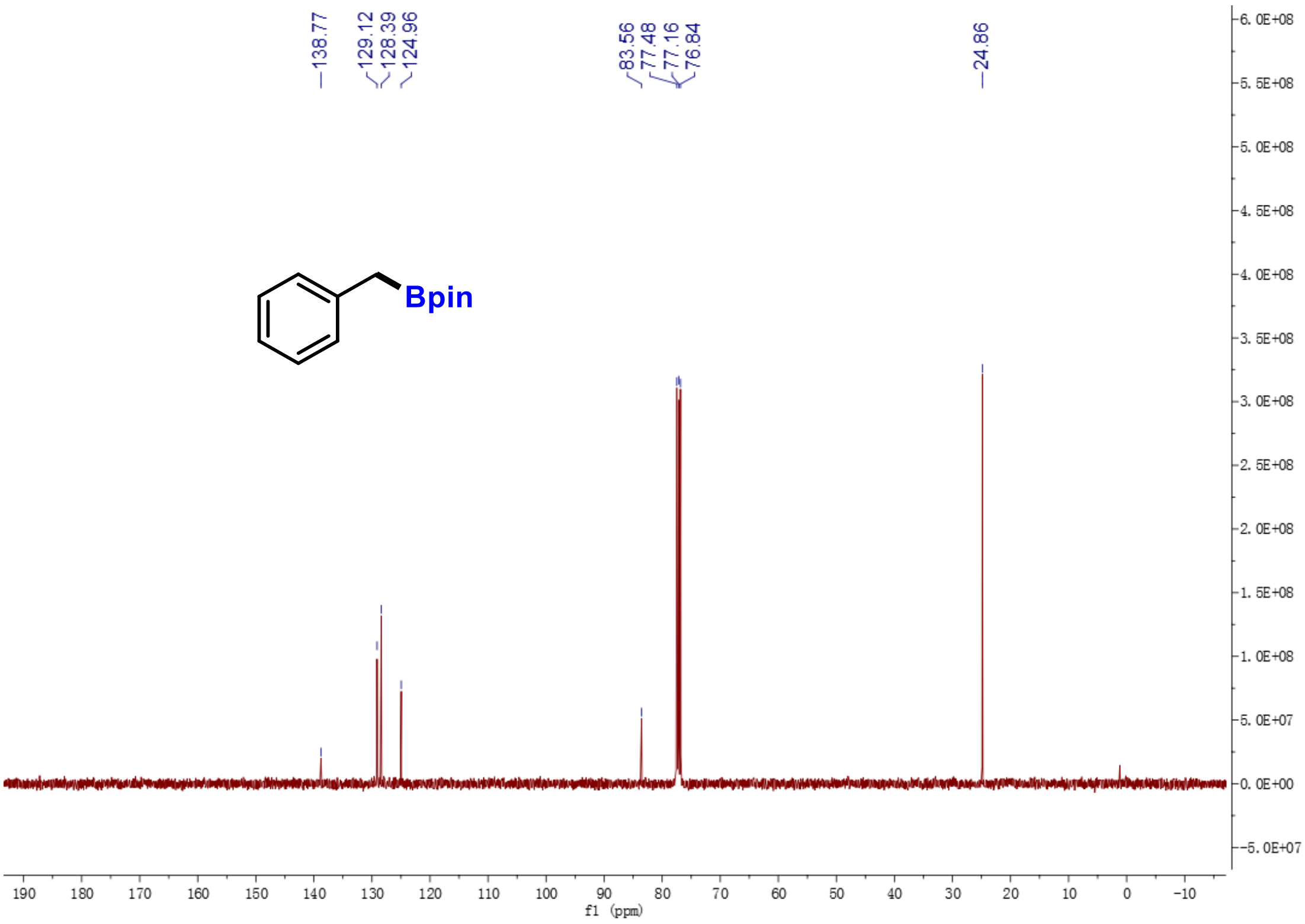

^{1}H NMR spectrum of 9c (400 MHz, $CDCl_3$)

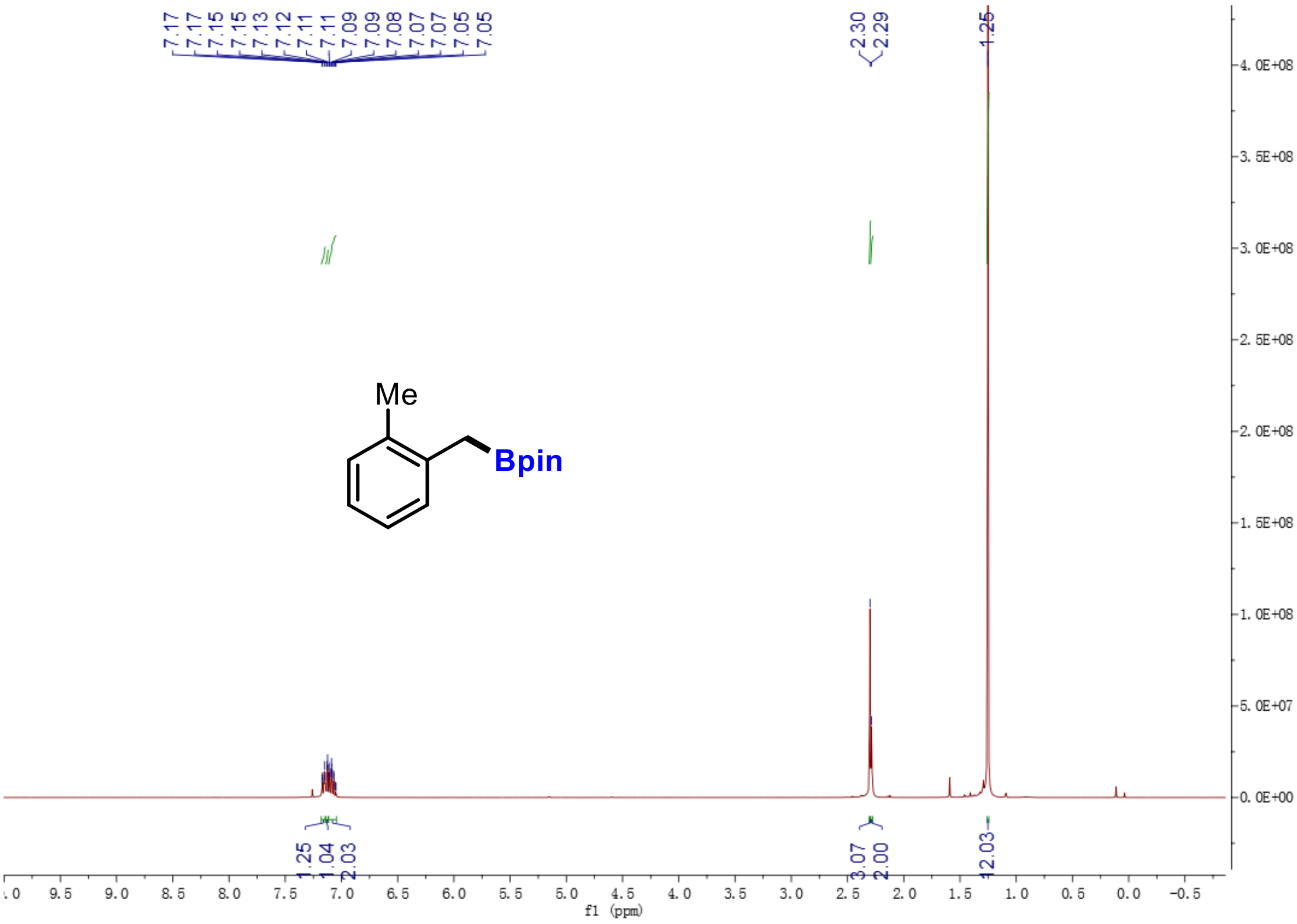

^{13}C NMR spectrum of 9c (101 MHz, $CDCl_3$)

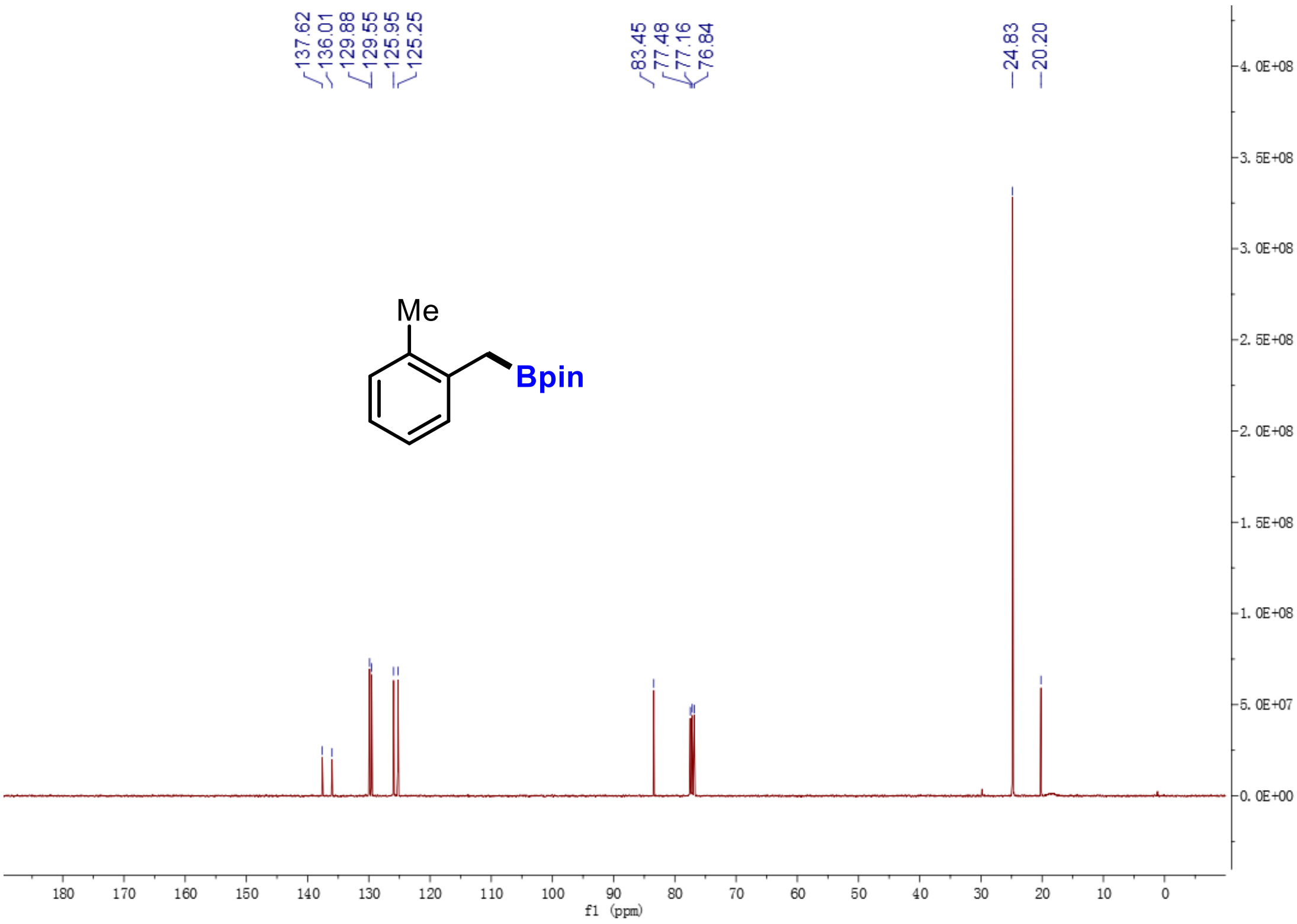

^{1}H NMR spectrum of 9d (400 MHz, $CDCl_3$)

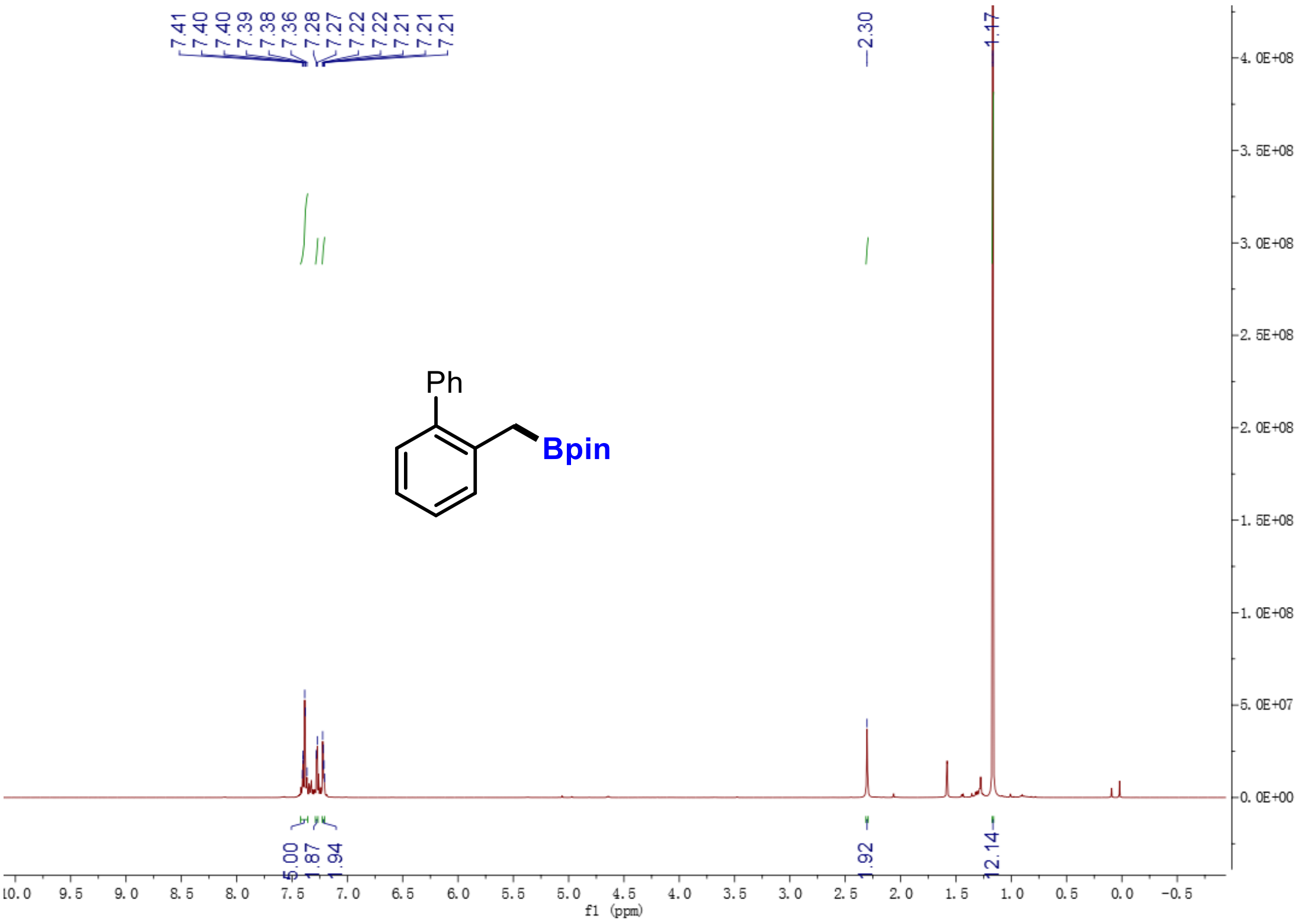

^{13}C NMR spectrum of 9d (101 MHz, $CDCl_3$)

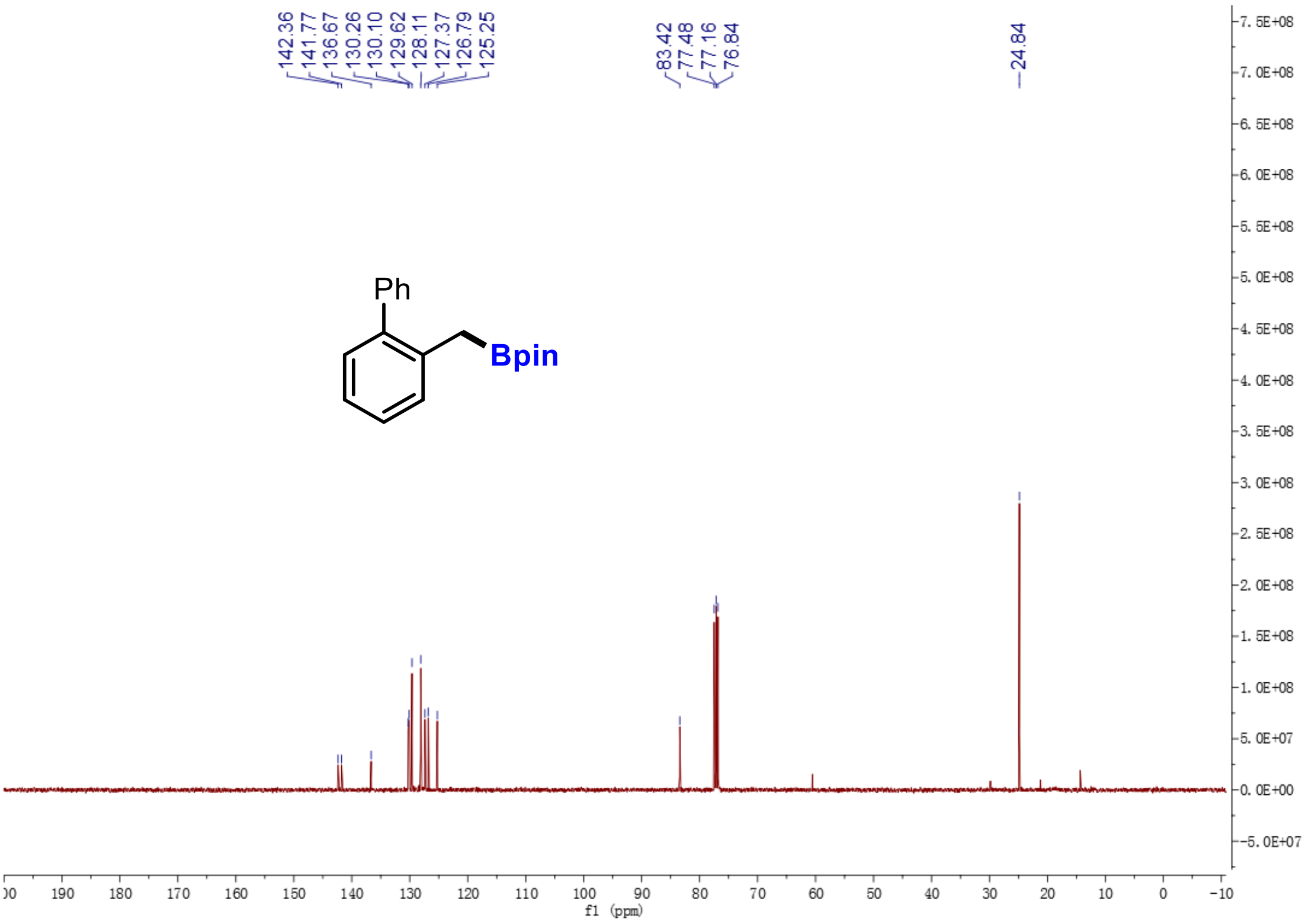

^{1}H NMR spectrum of 9e (400 MHz, $CDCl_3$)

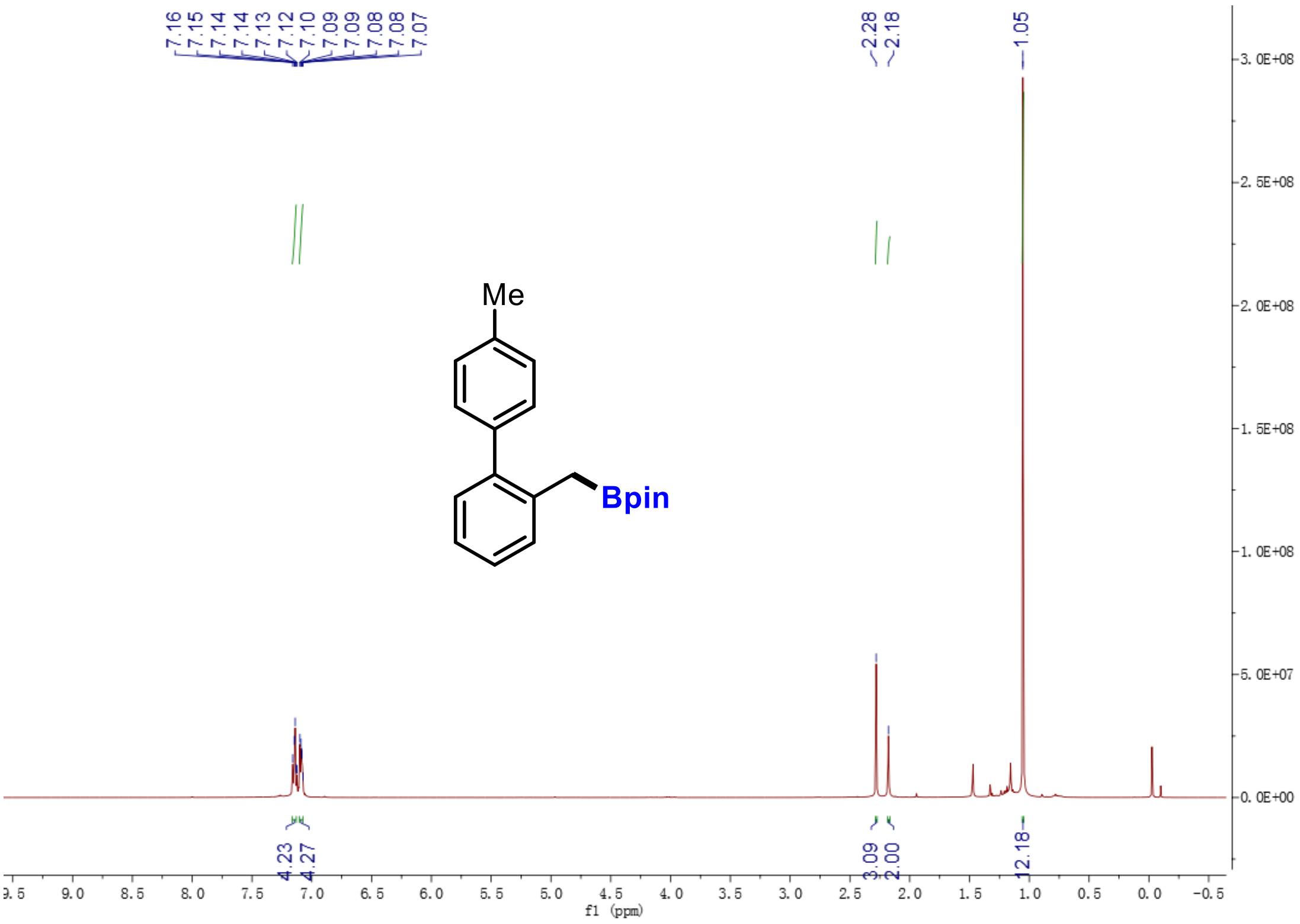

^{13}C NMR spectrum of 9e (101 MHz, $CDCl_3$)

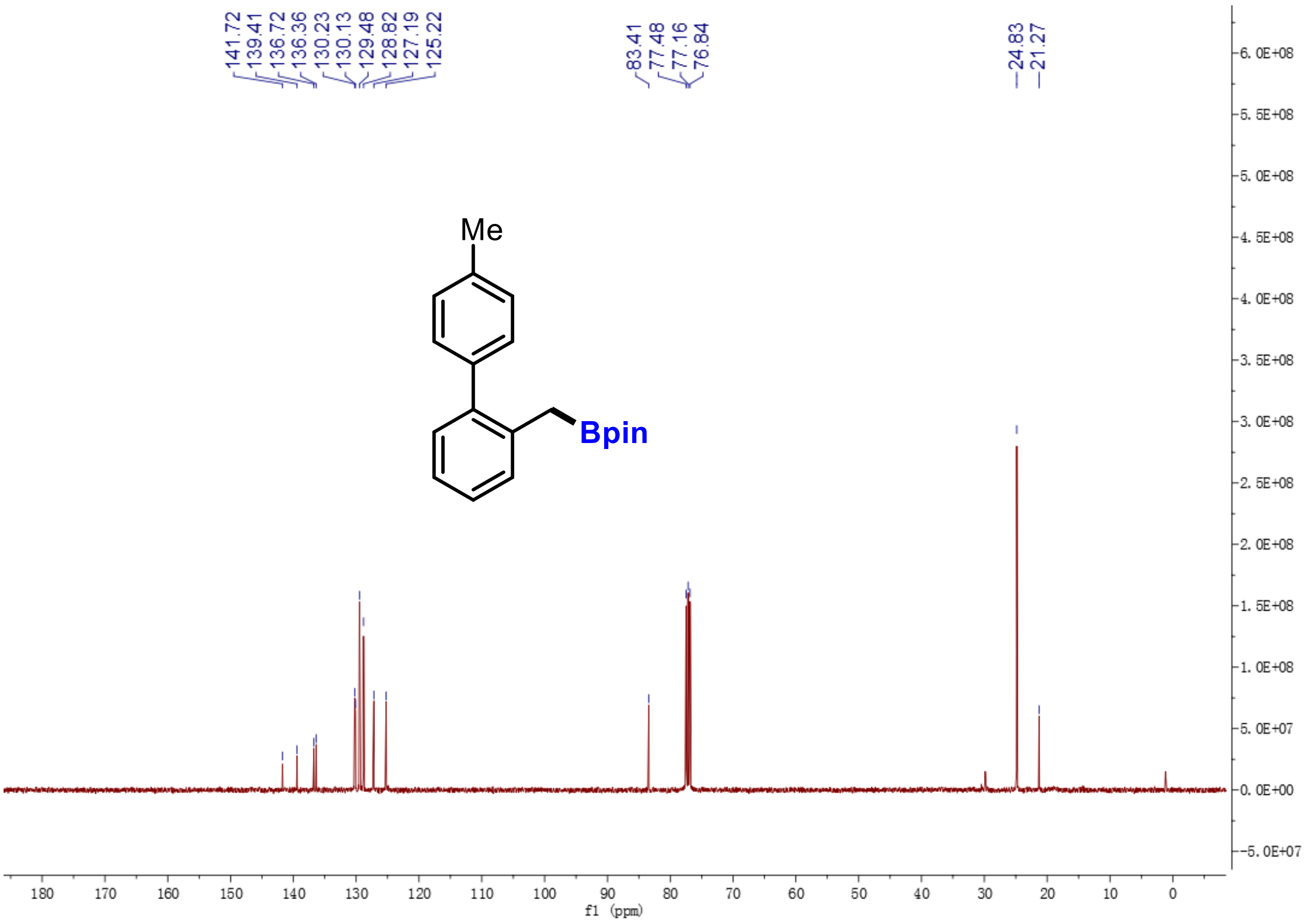

^{1}H NMR spectrum of 9f (400 MHz, $CDCl_3$)

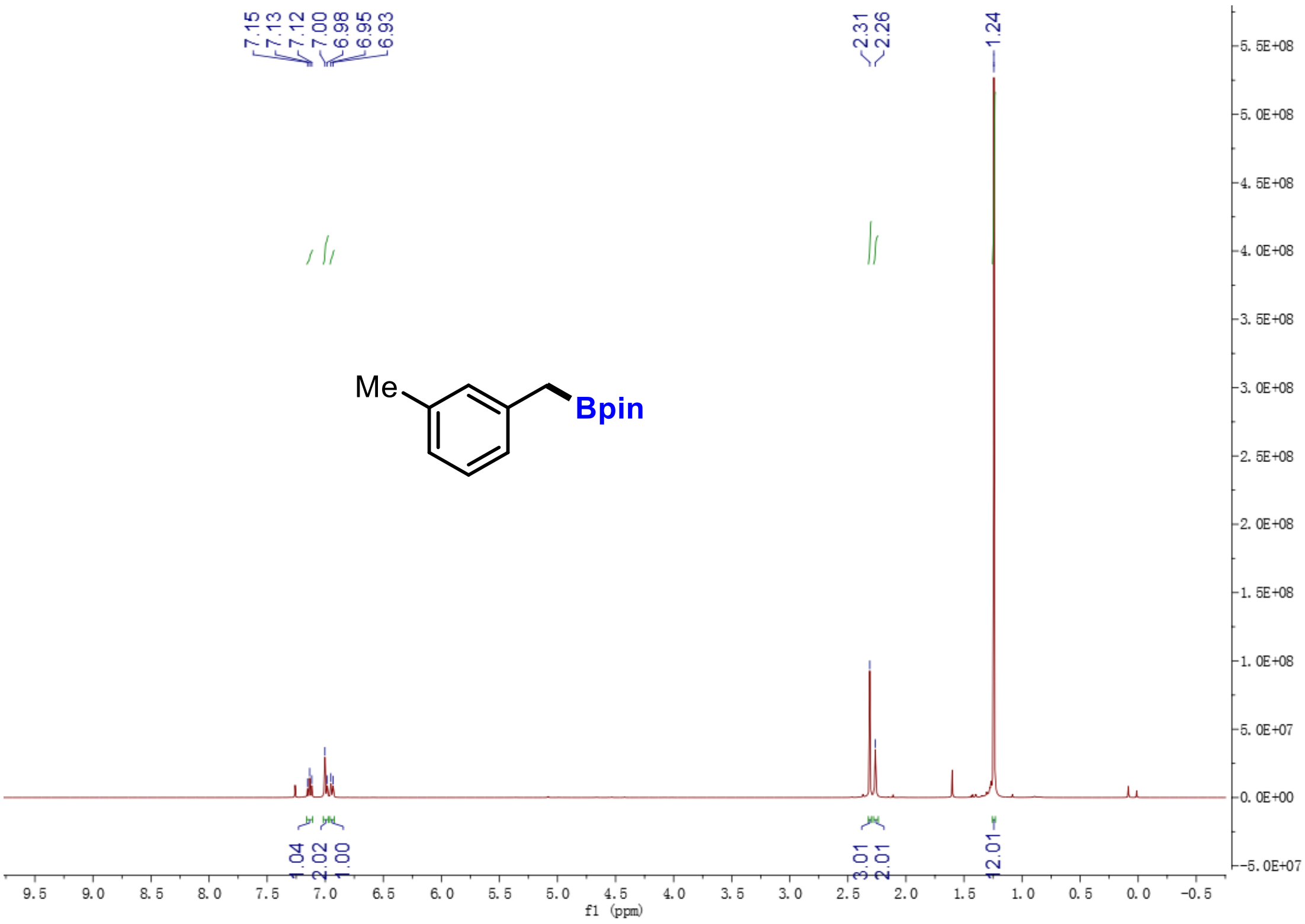

^{13}C NMR spectrum of 9f (101 MHz, $CDCl_3$)

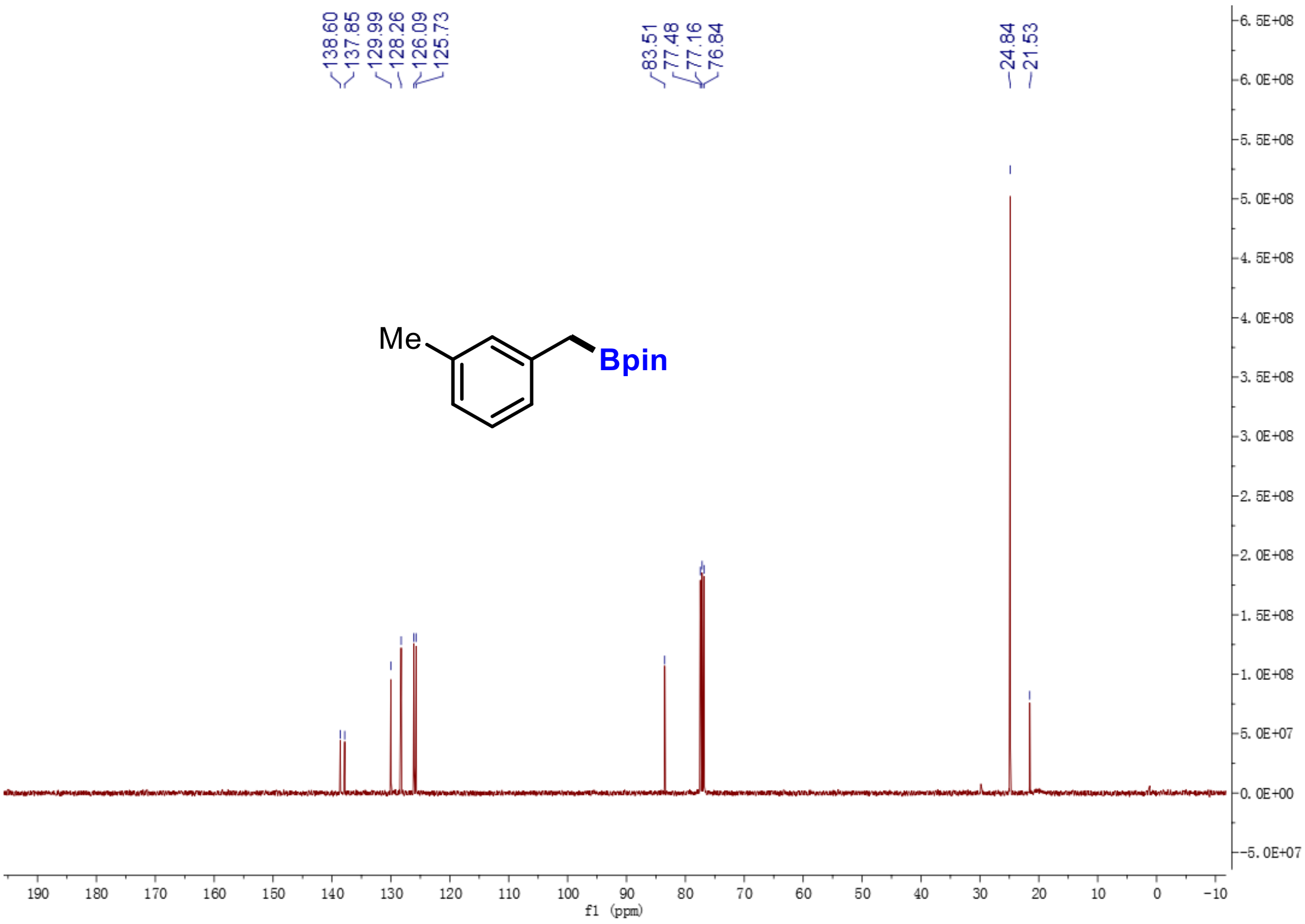

^{1}H NMR spectrum of 9g (400 MHz, $CDCl_3$)

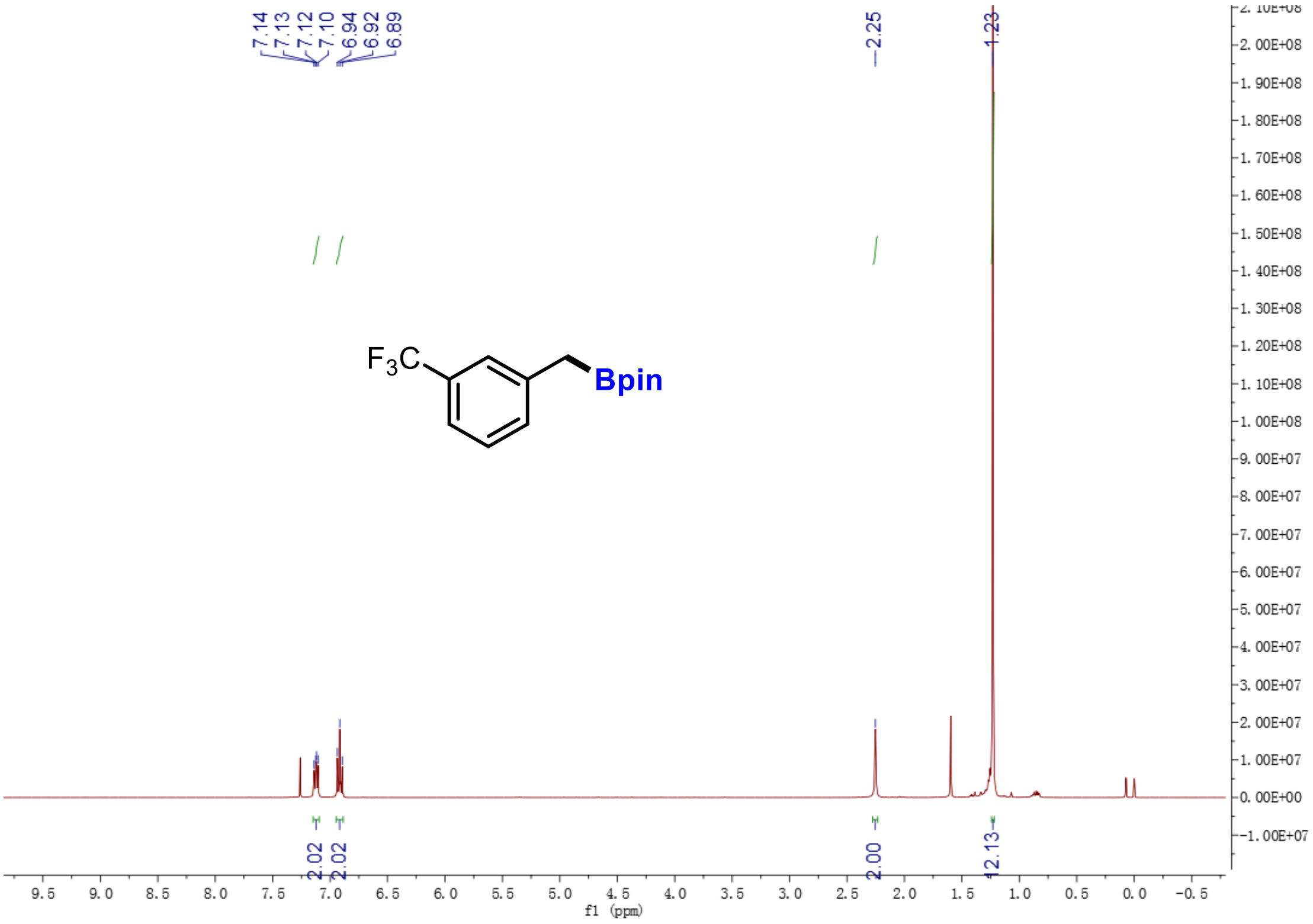

^{13}C NMR spectrum of 9g (101 MHz, $CDCl_3$)

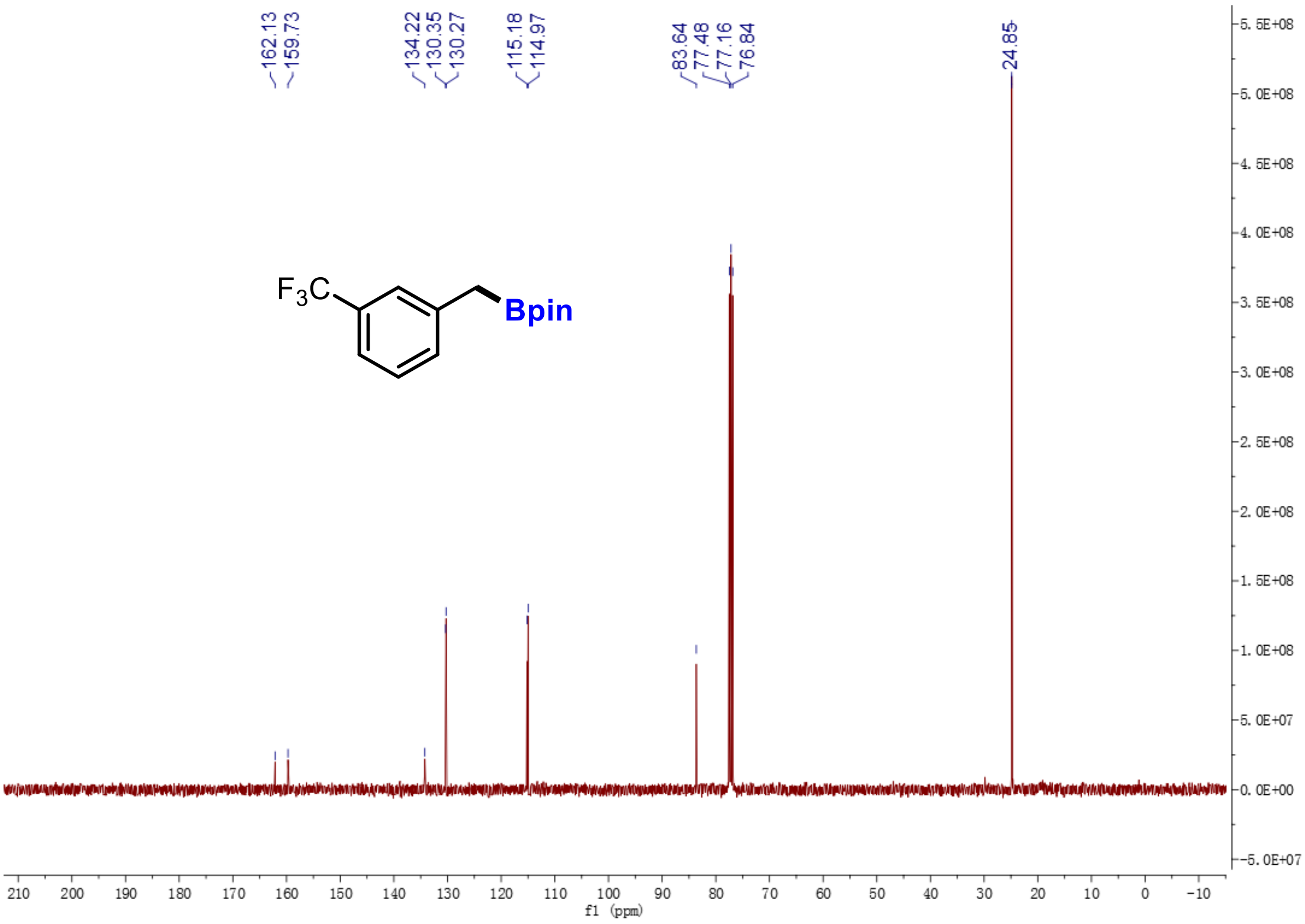

^{19}F NMR spectrum of 9g

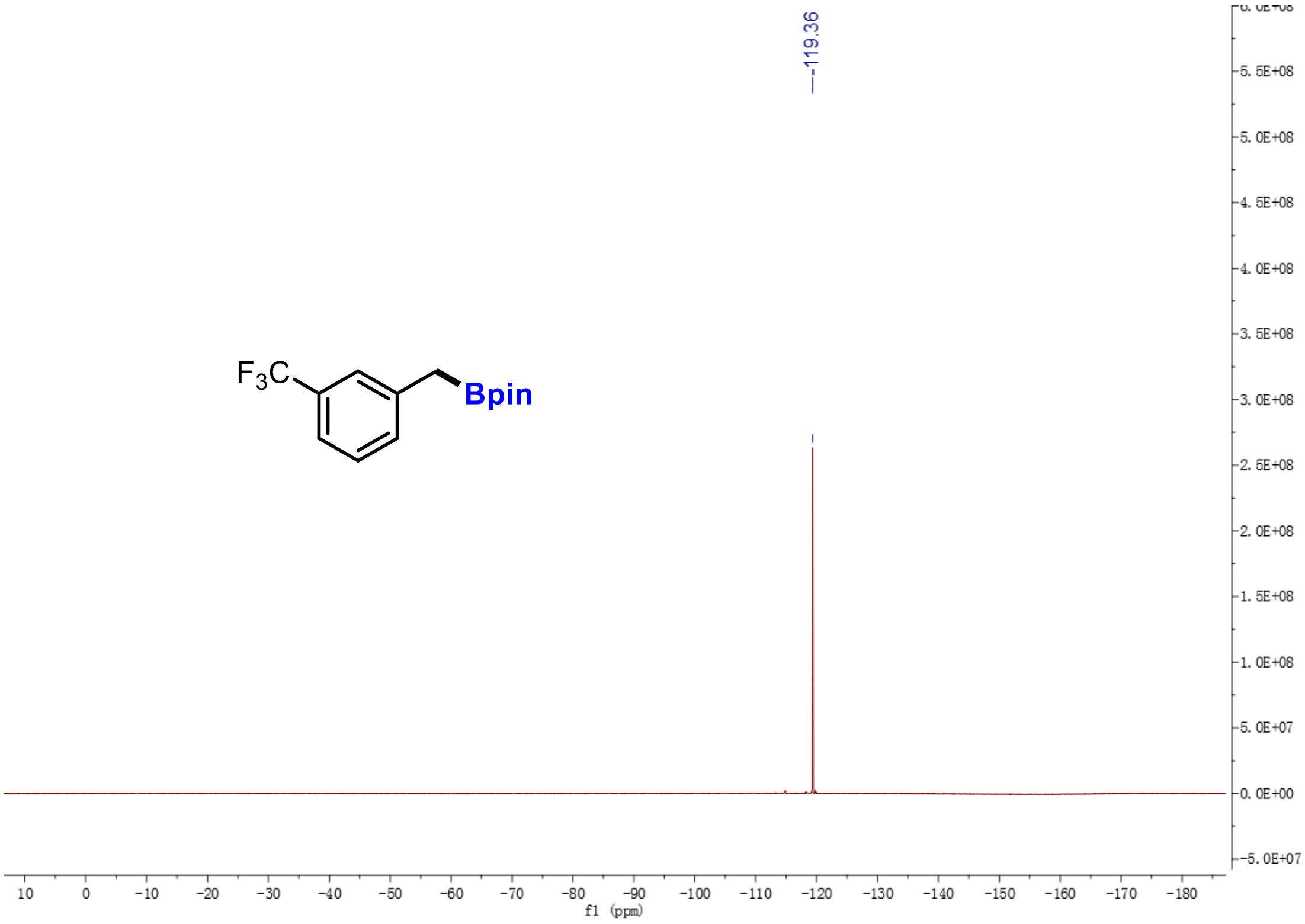

^{1}H NMR spectrum of 9h (400 MHz, $CDCl_3$)

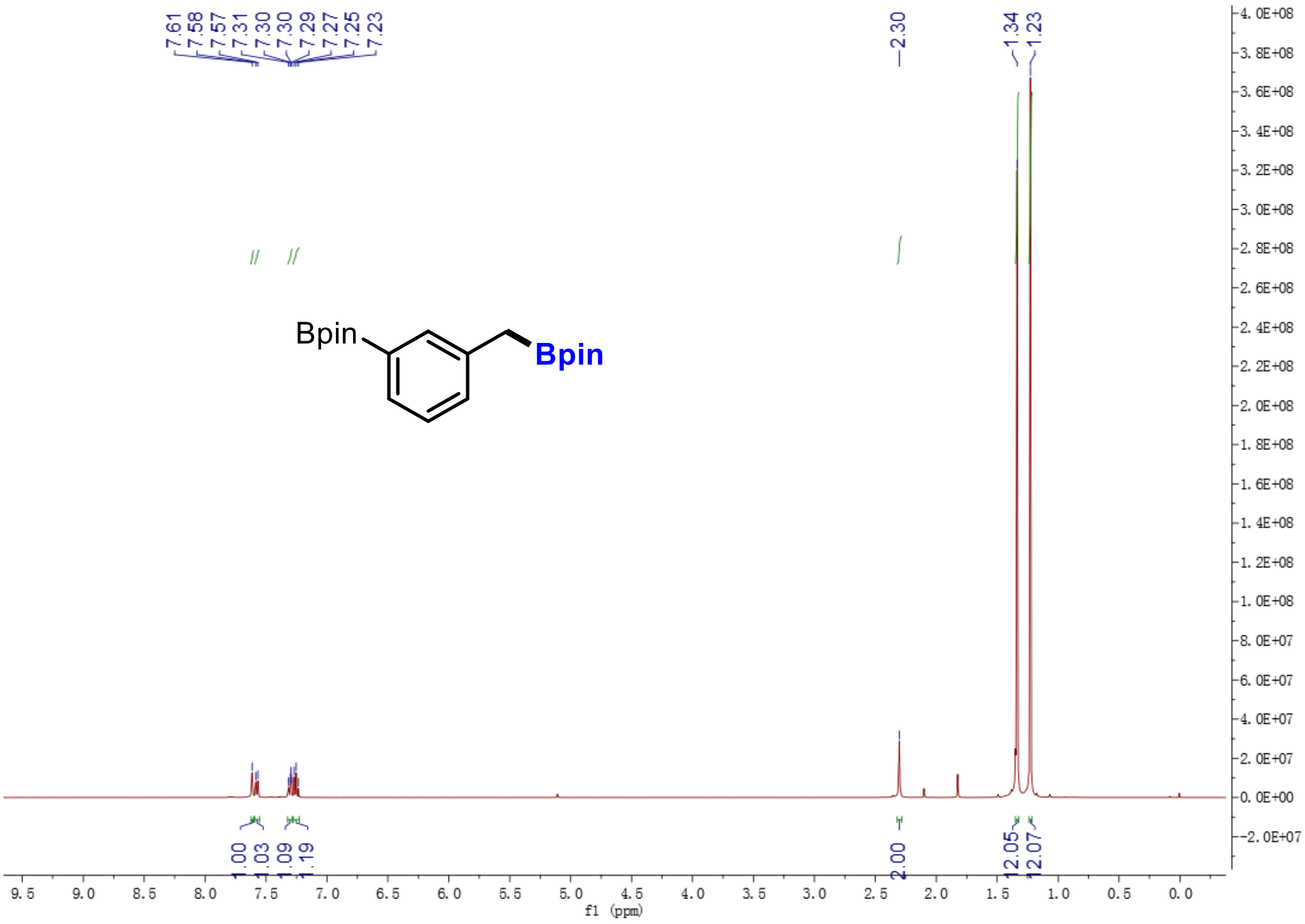

^{13}C NMR spectrum of 9h (101 MHz, $CDCl_3$)

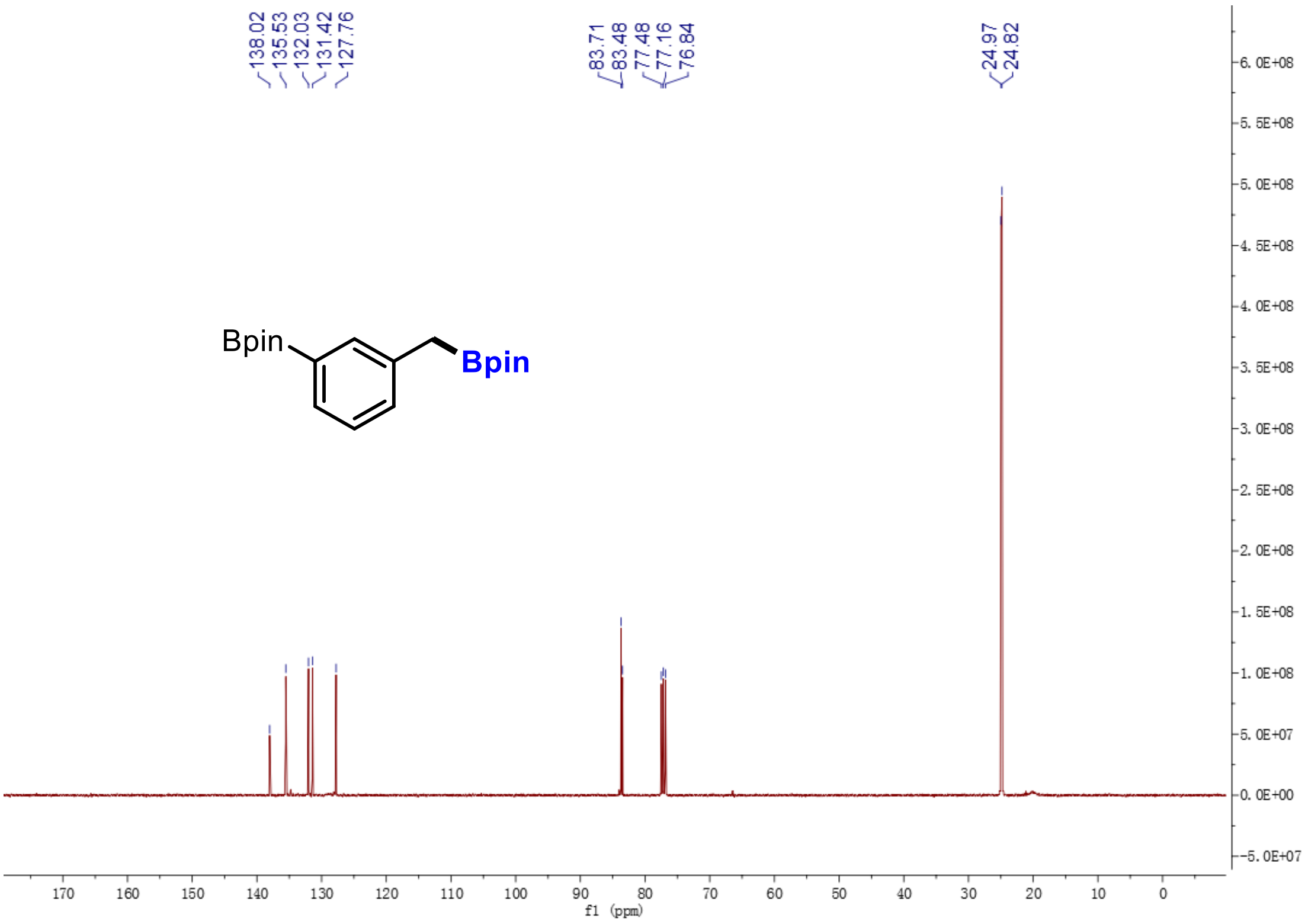

^{1}H NMR spectrum of 9i (400 MHz, $CDCl_3$)

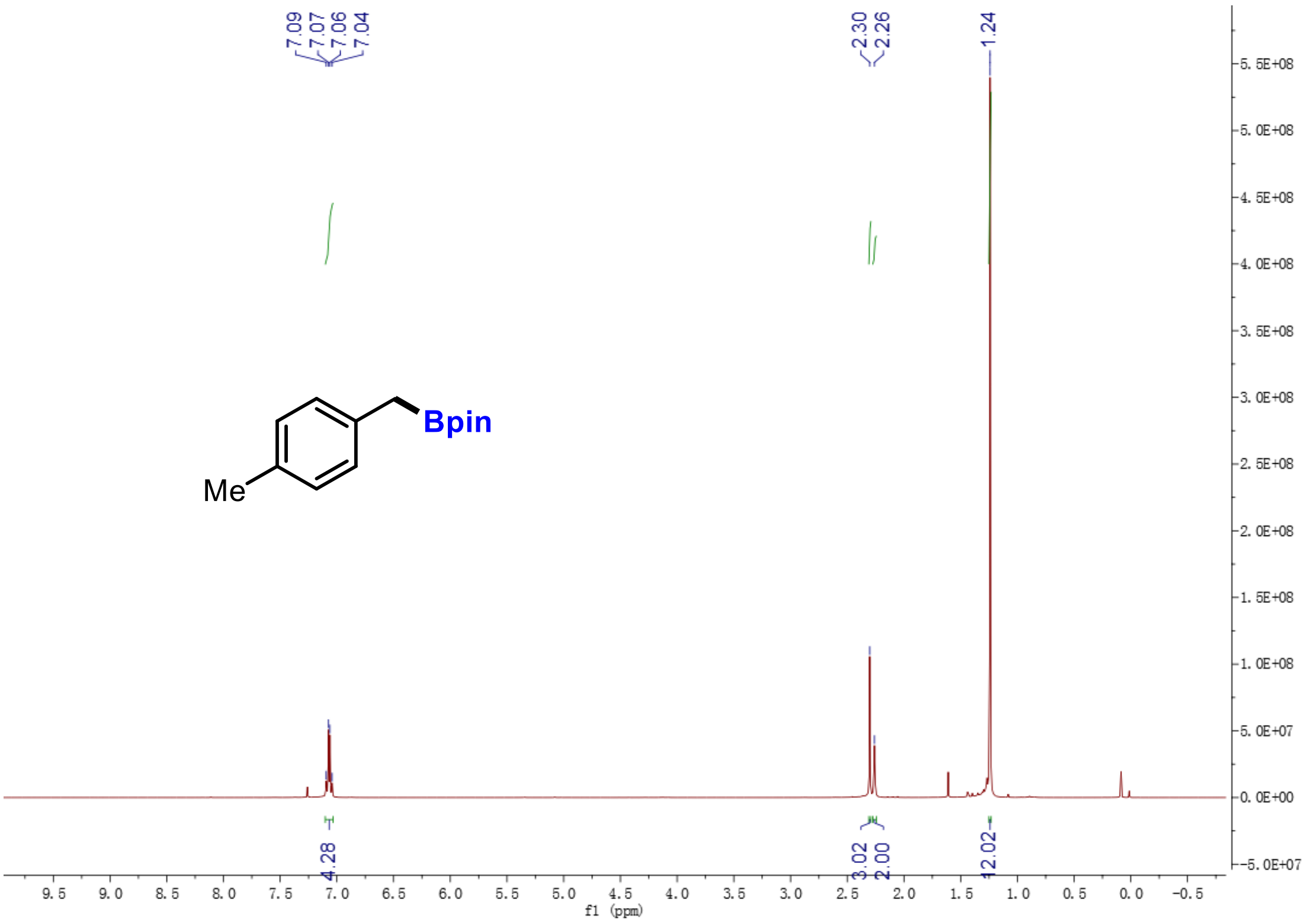

^{13}C NMR spectrum of 9i (101 MHz, $CDCl_3$)

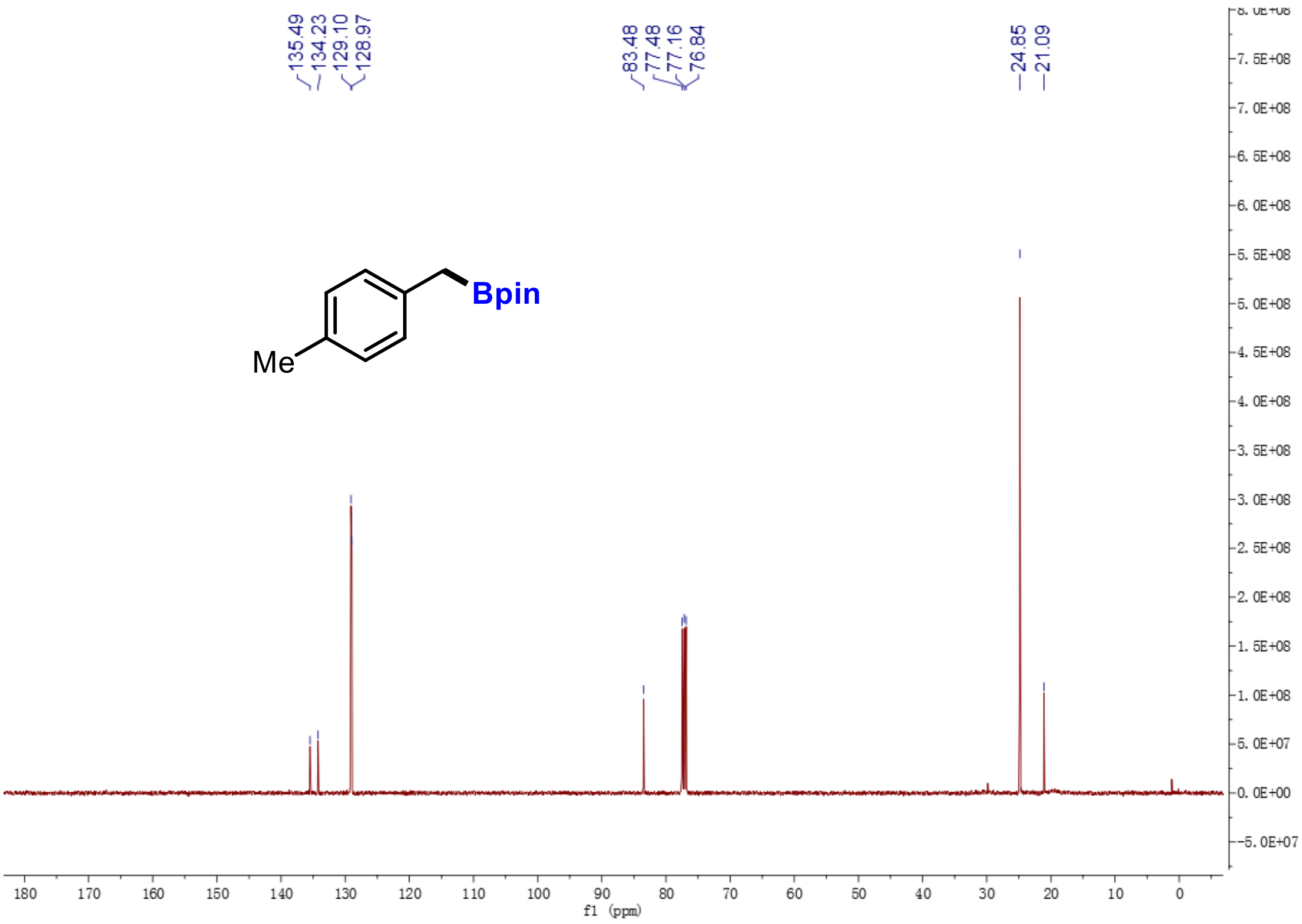

^{1}H NMR spectrum of 9j (400 MHz, $CDCl_3$)

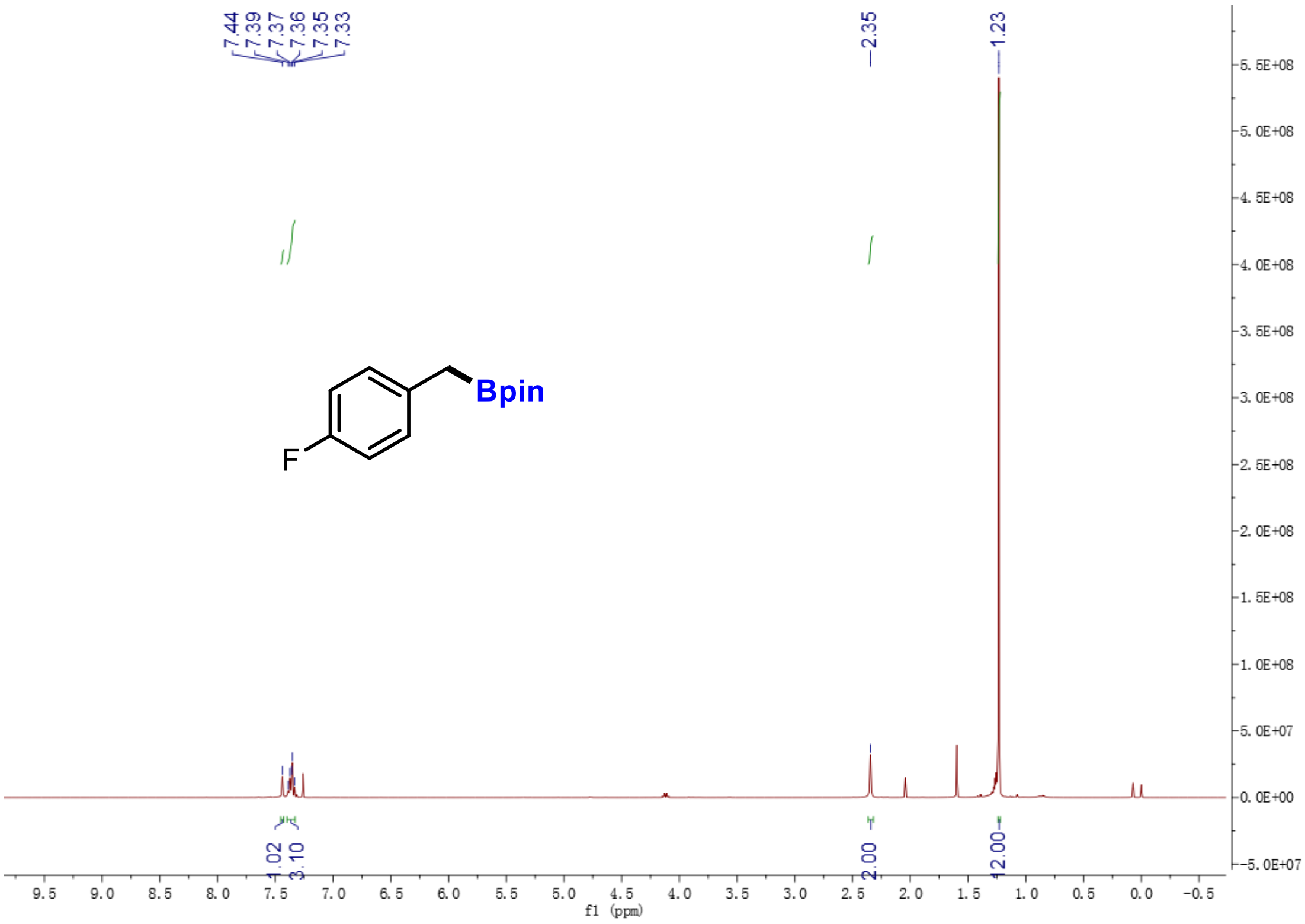

^{13}C NMR spectrum of 9j (101 MHz, $CDCl_3$)

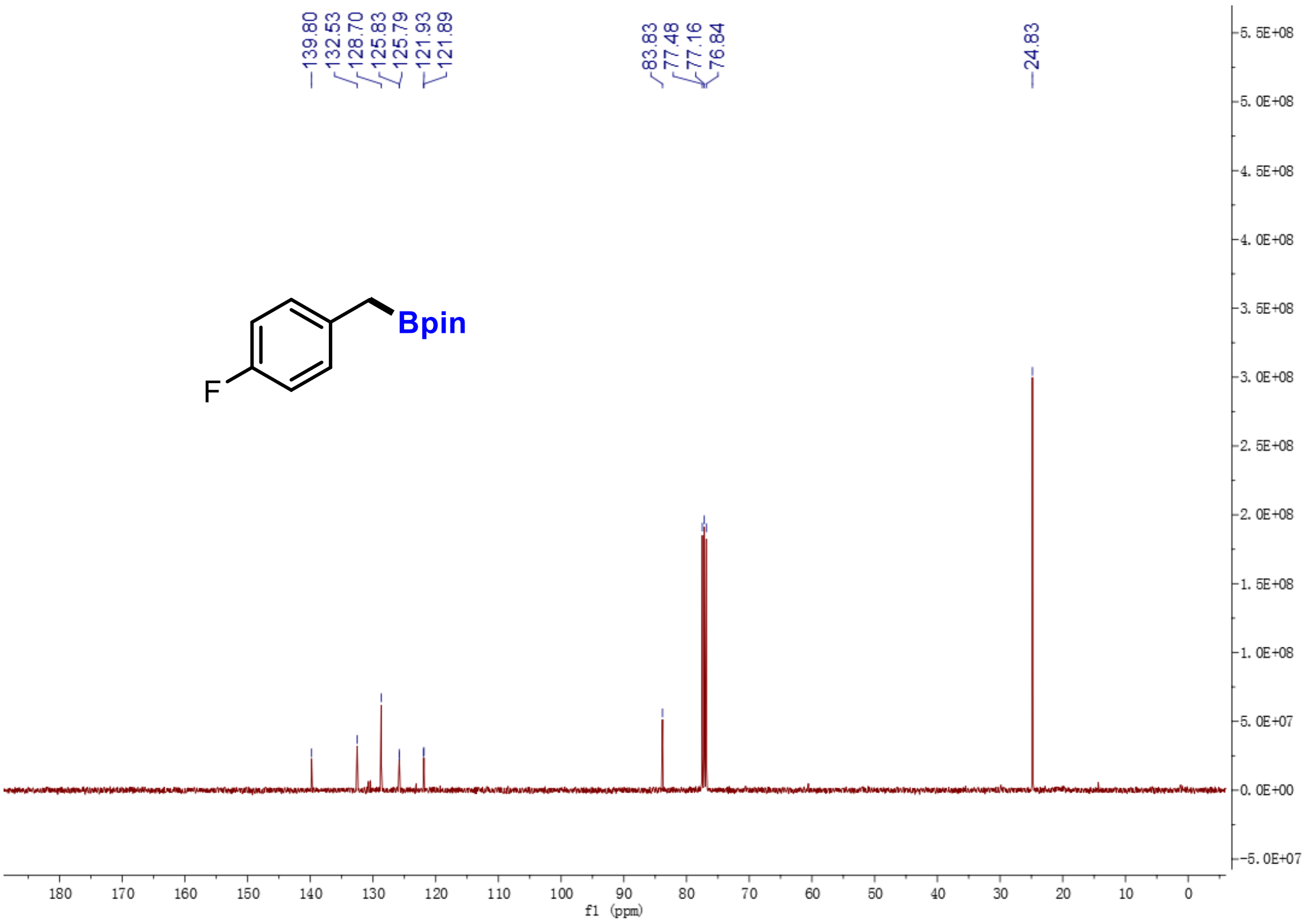

^{19}F NMR spectrum of 9j

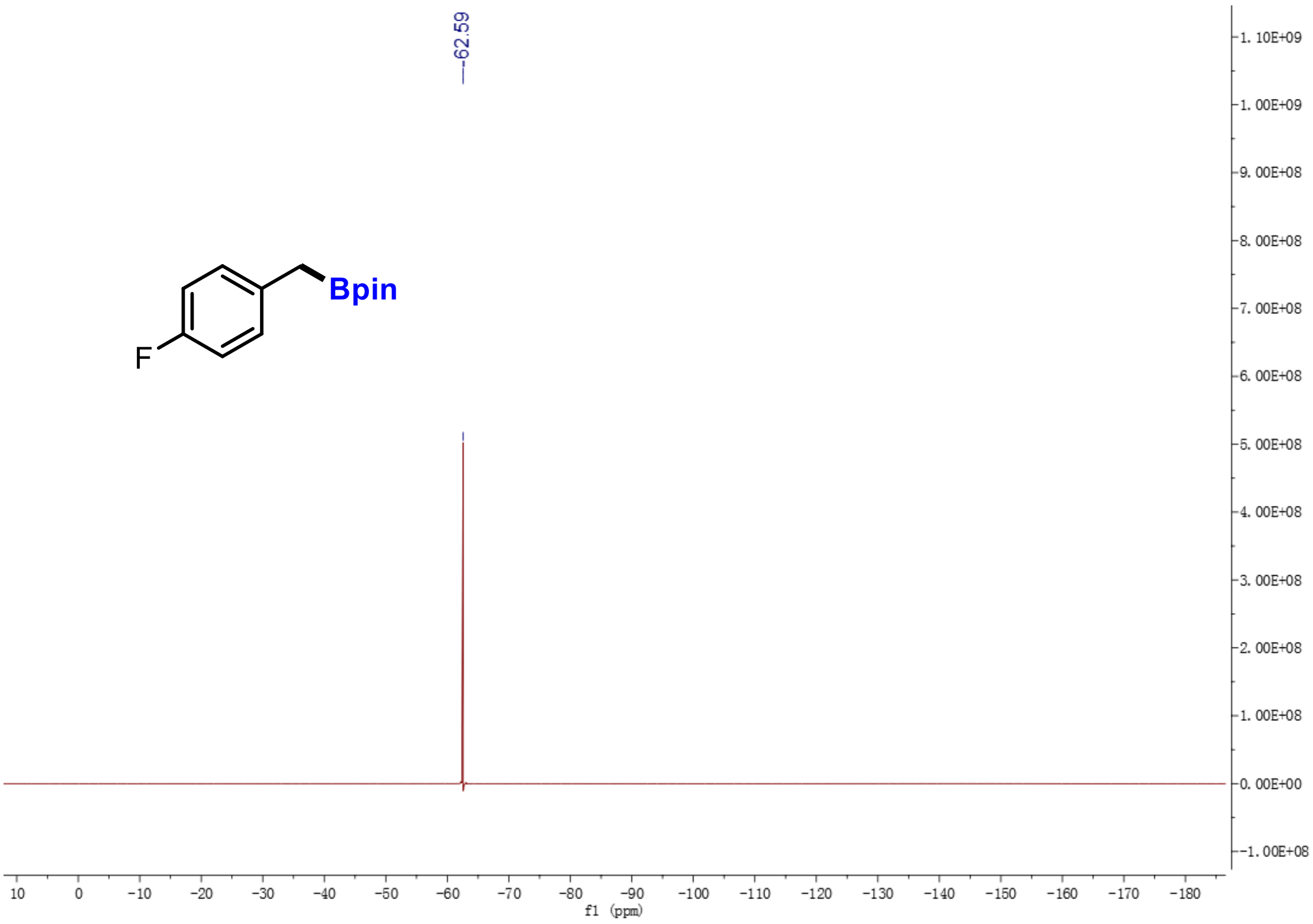

^{1}H NMR spectrum of 9k (400 MHz, $CDCl_3$)

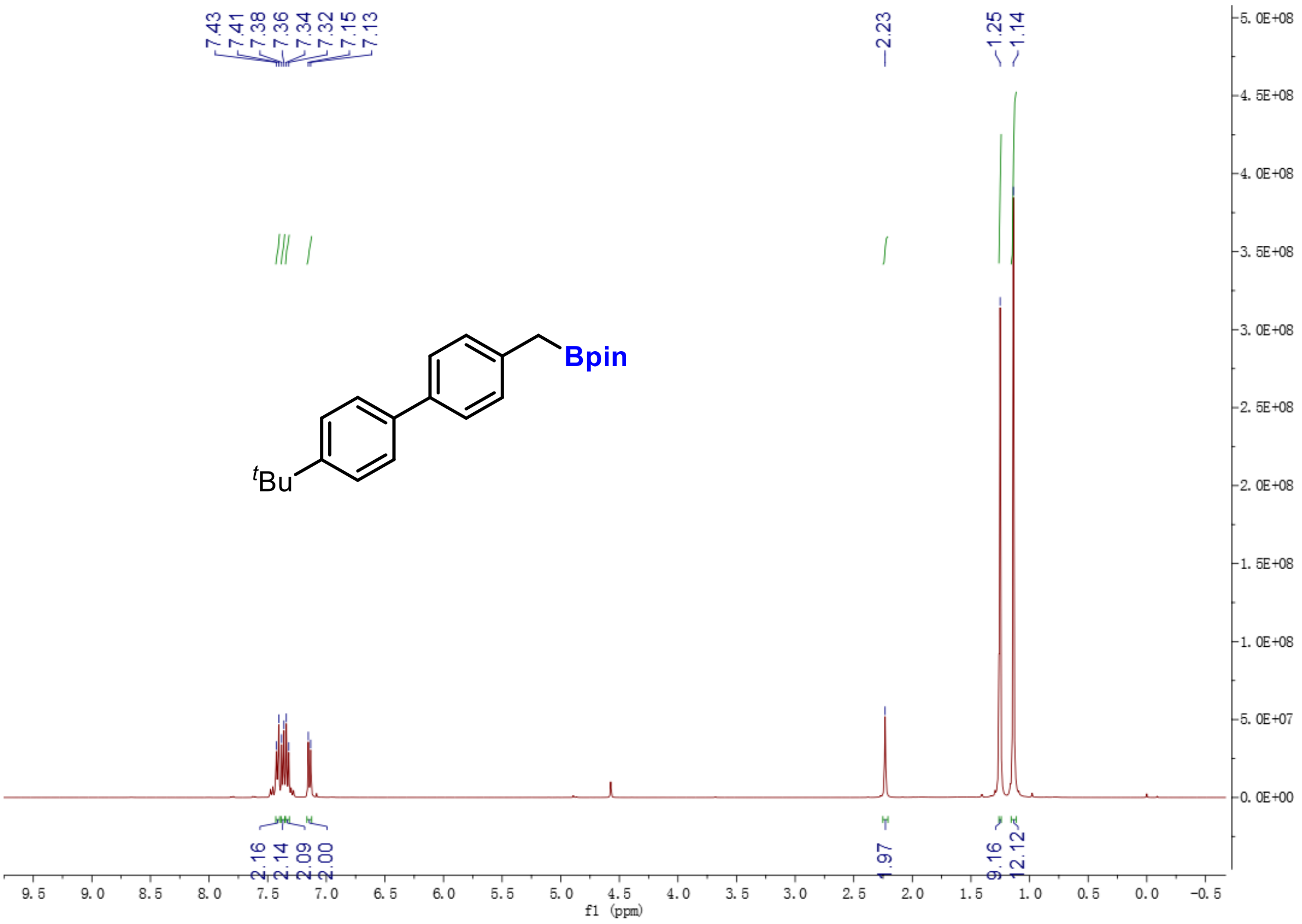

^{13}C NMR spectrum of 9k (101 MHz, $CDCl_3$)

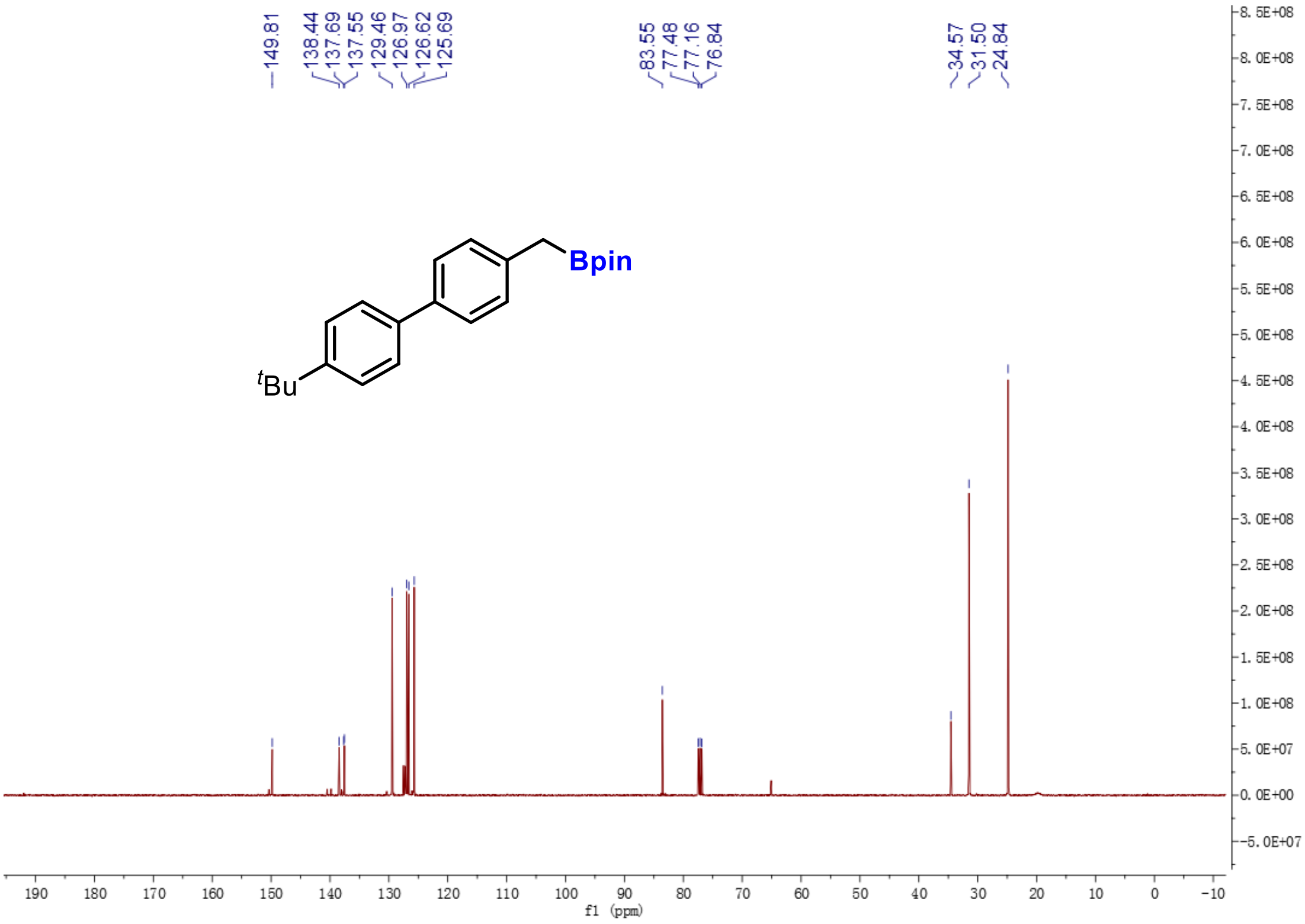

^{1}H NMR spectrum of 9l (400 MHz, $CDCl_3$)

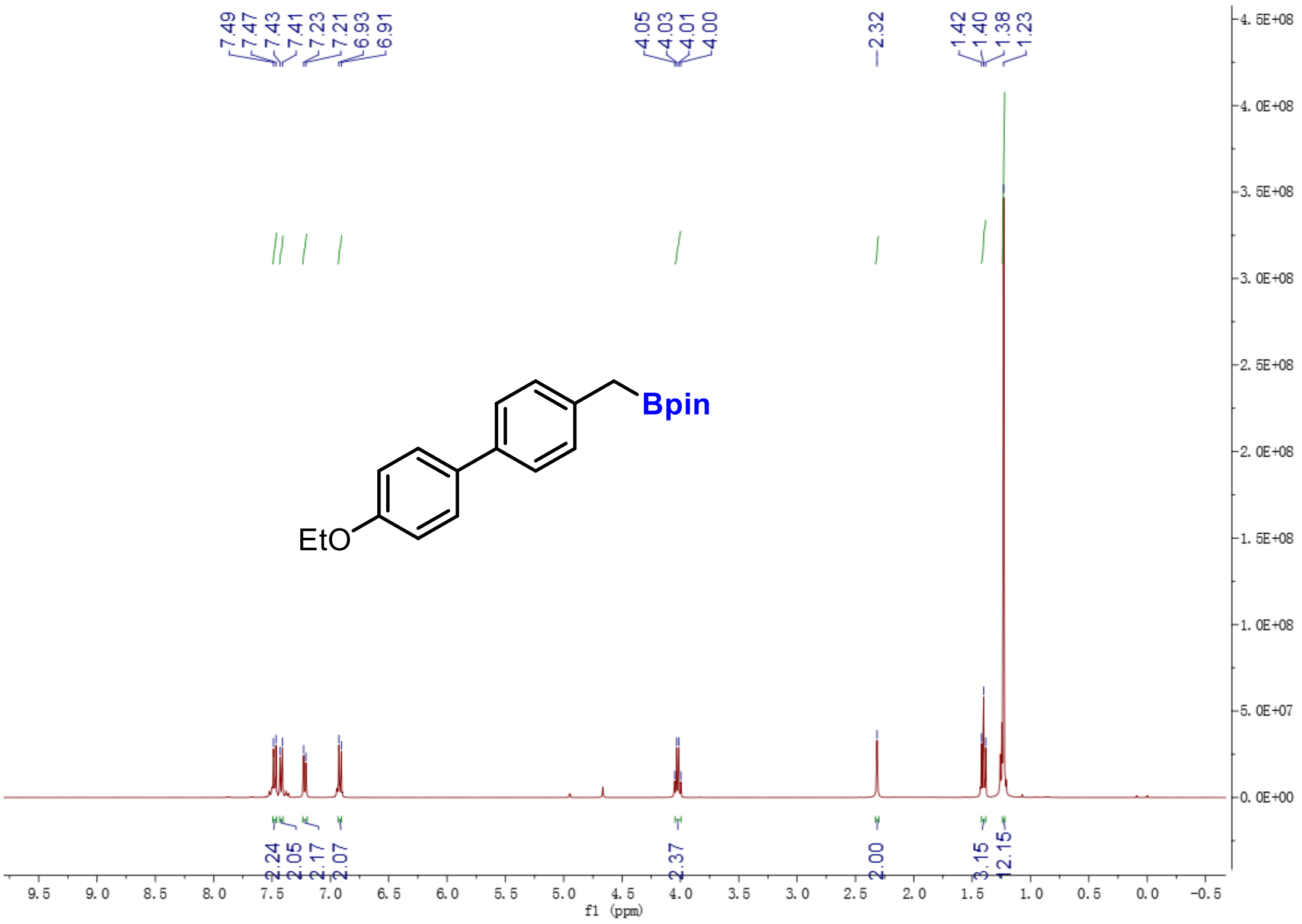

^{13}C NMR spectrum of 9l (101 MHz, $CDCl_3$)

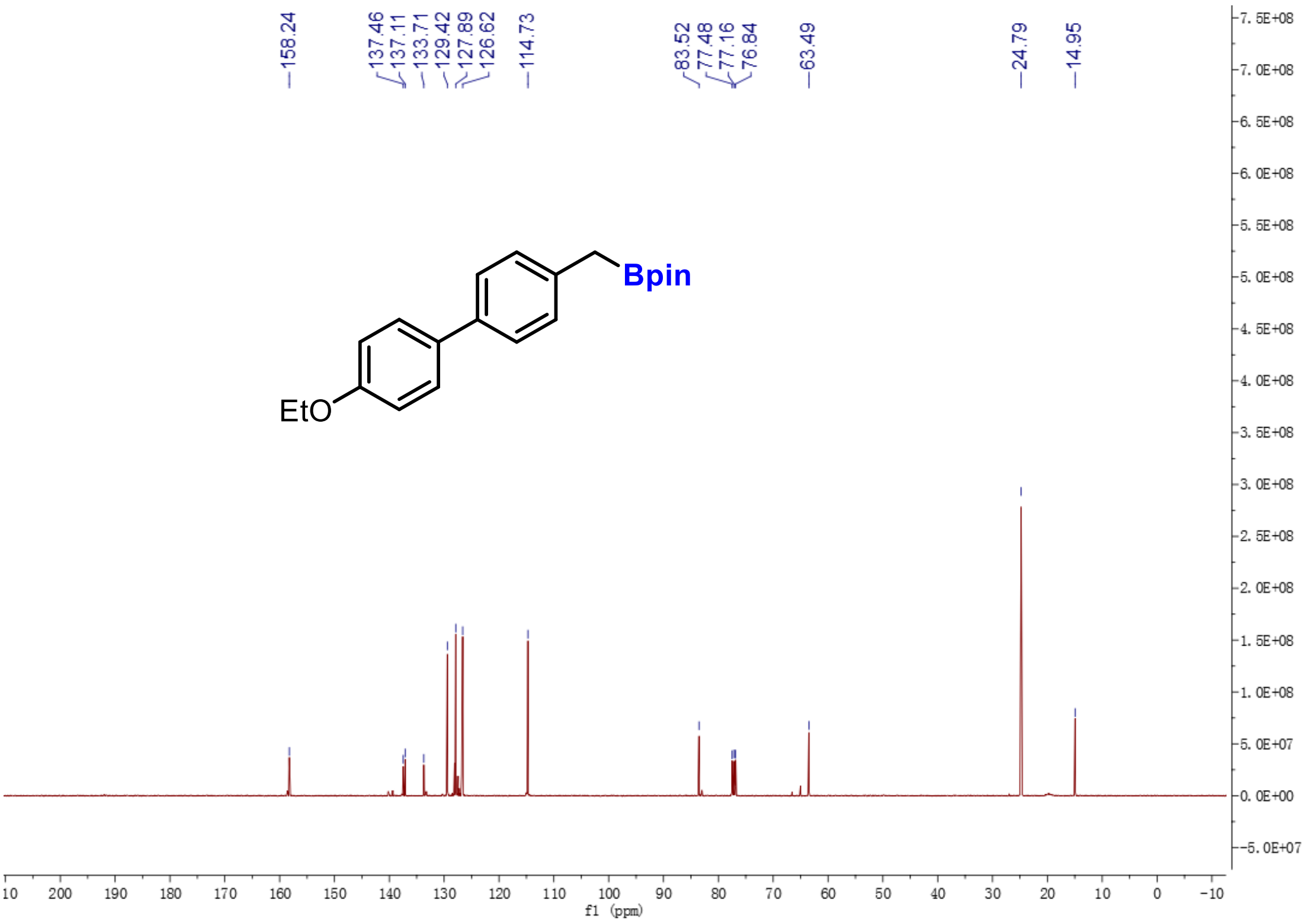

^{1}H NMR spectrum of 9m (400 MHz, $CDCl_3$)

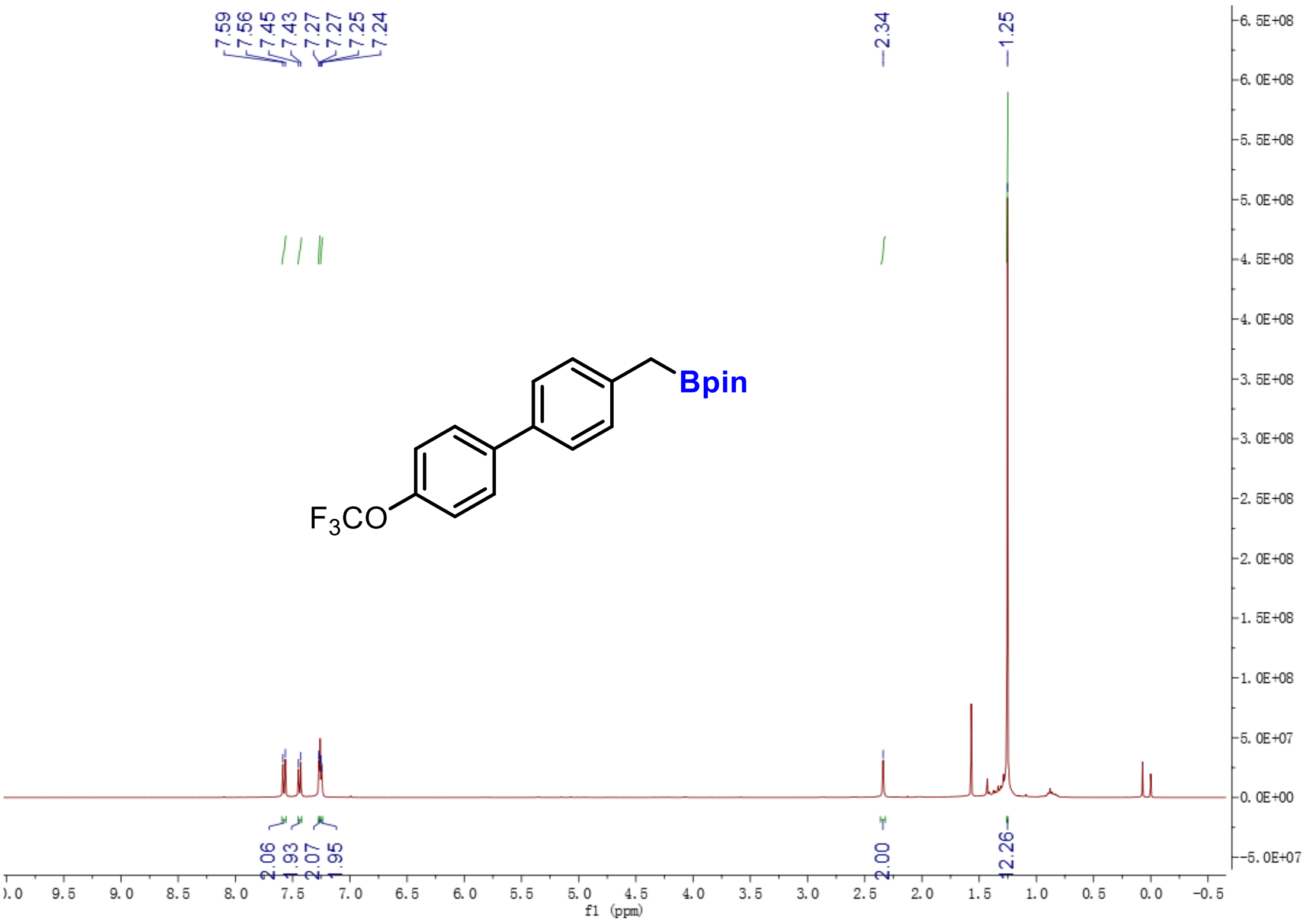

^{13}C NMR spectrum of 9m (101 MHz, $CDCl_3$)

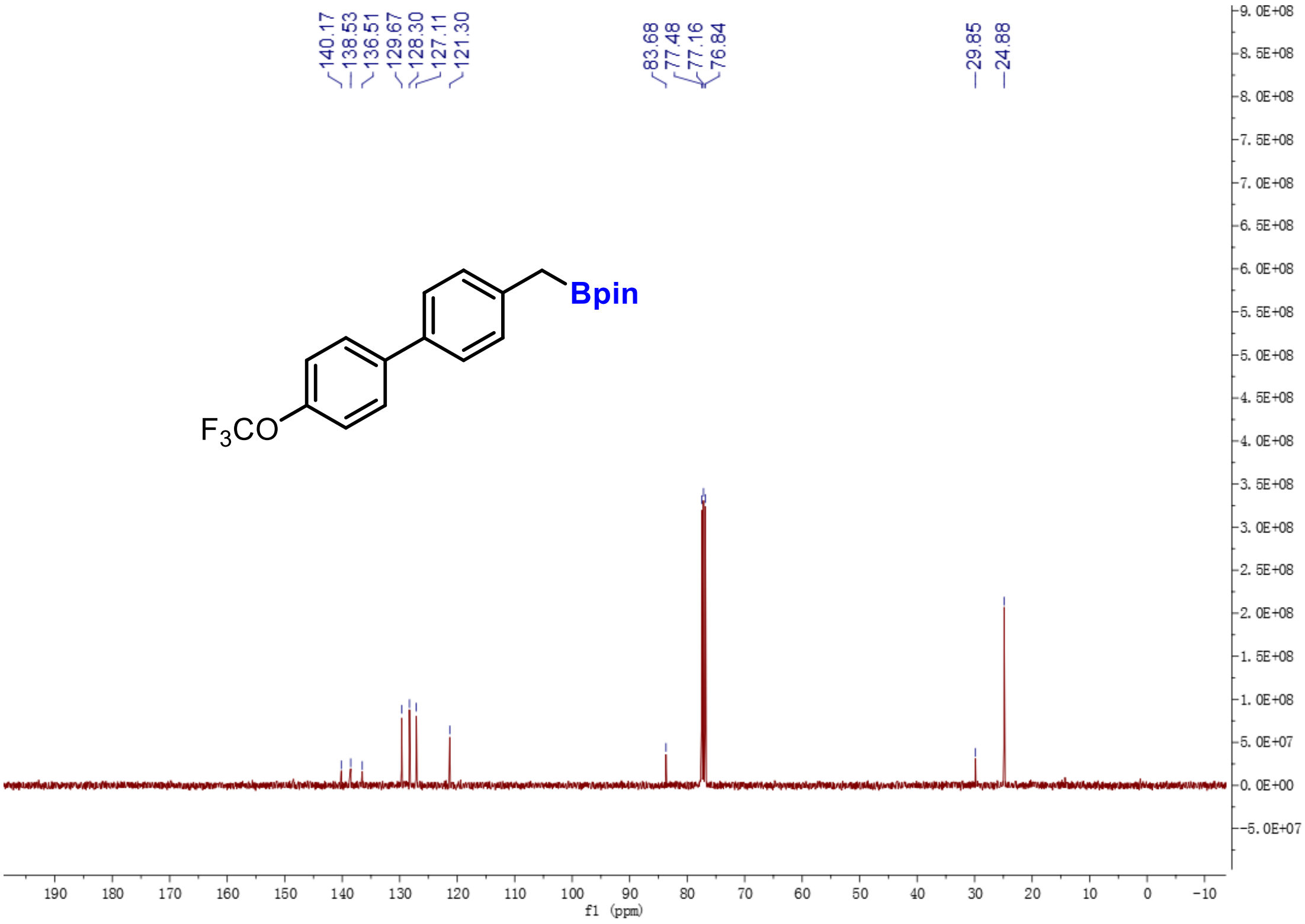

^{19}F NMR spectrum of 9m

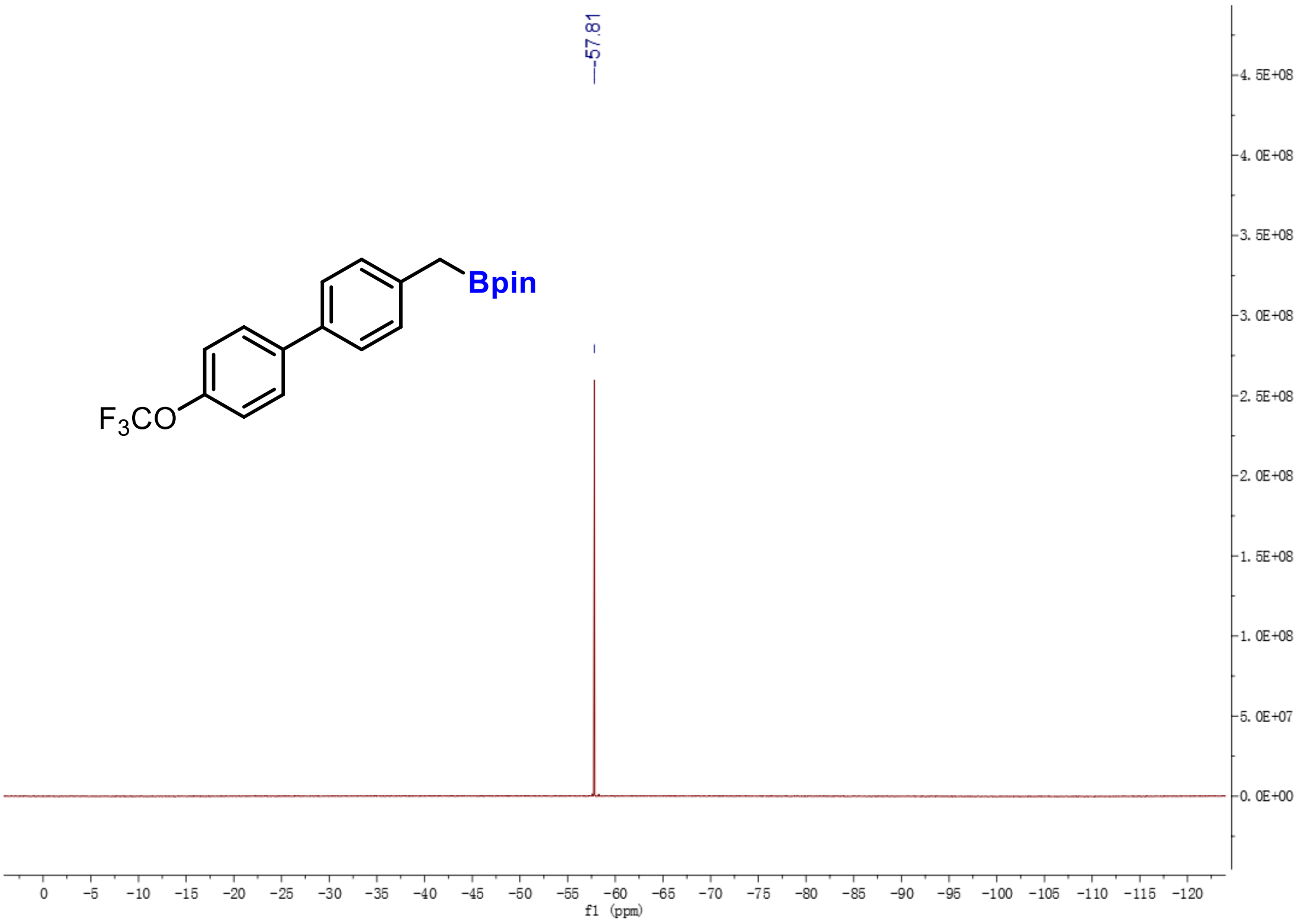

^{1}H NMR spectrum of 9n (400 MHz, $CDCl_3$)

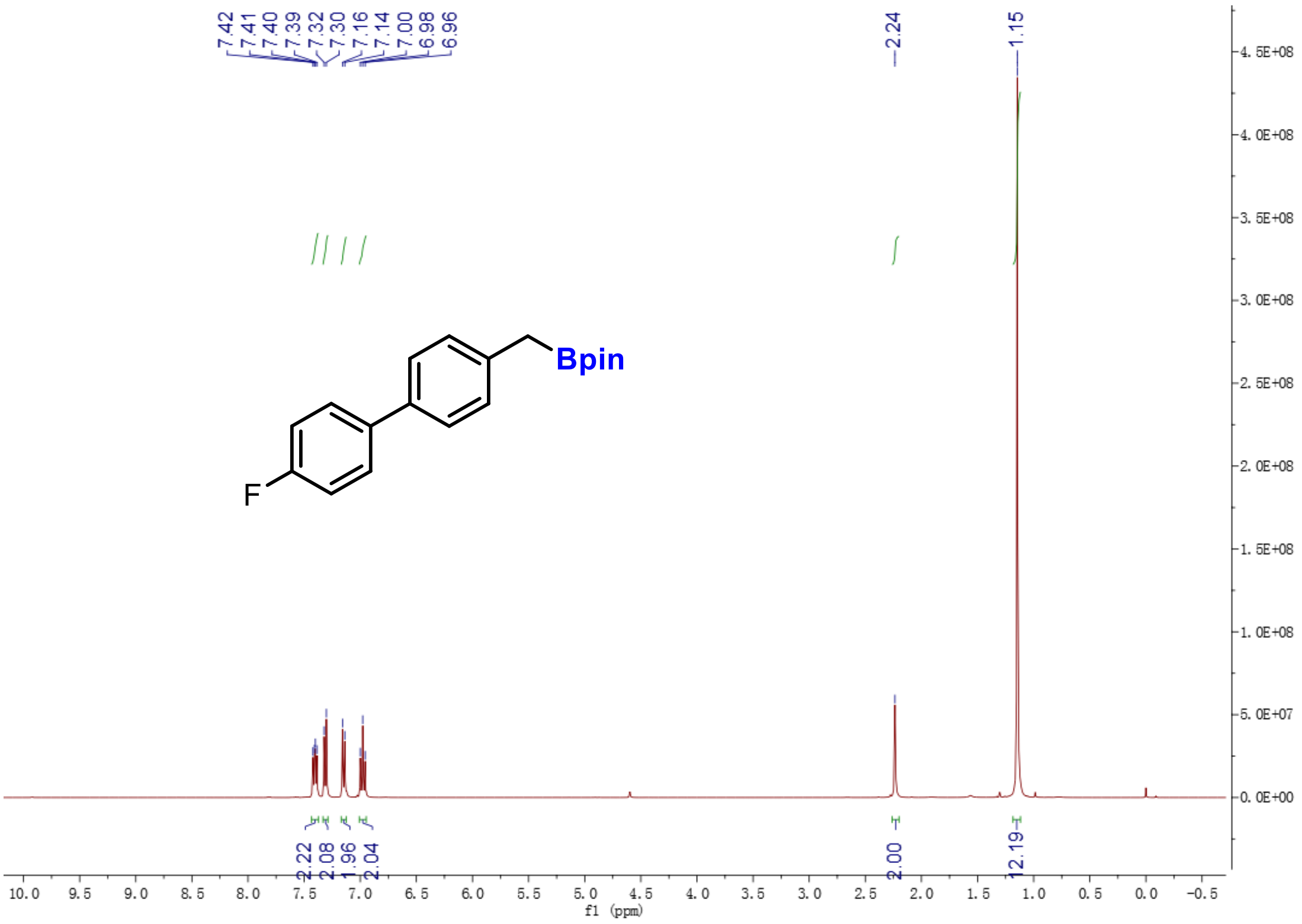

^{13}C NMR spectrum of 9n (101 MHz, $CDCl_3$)

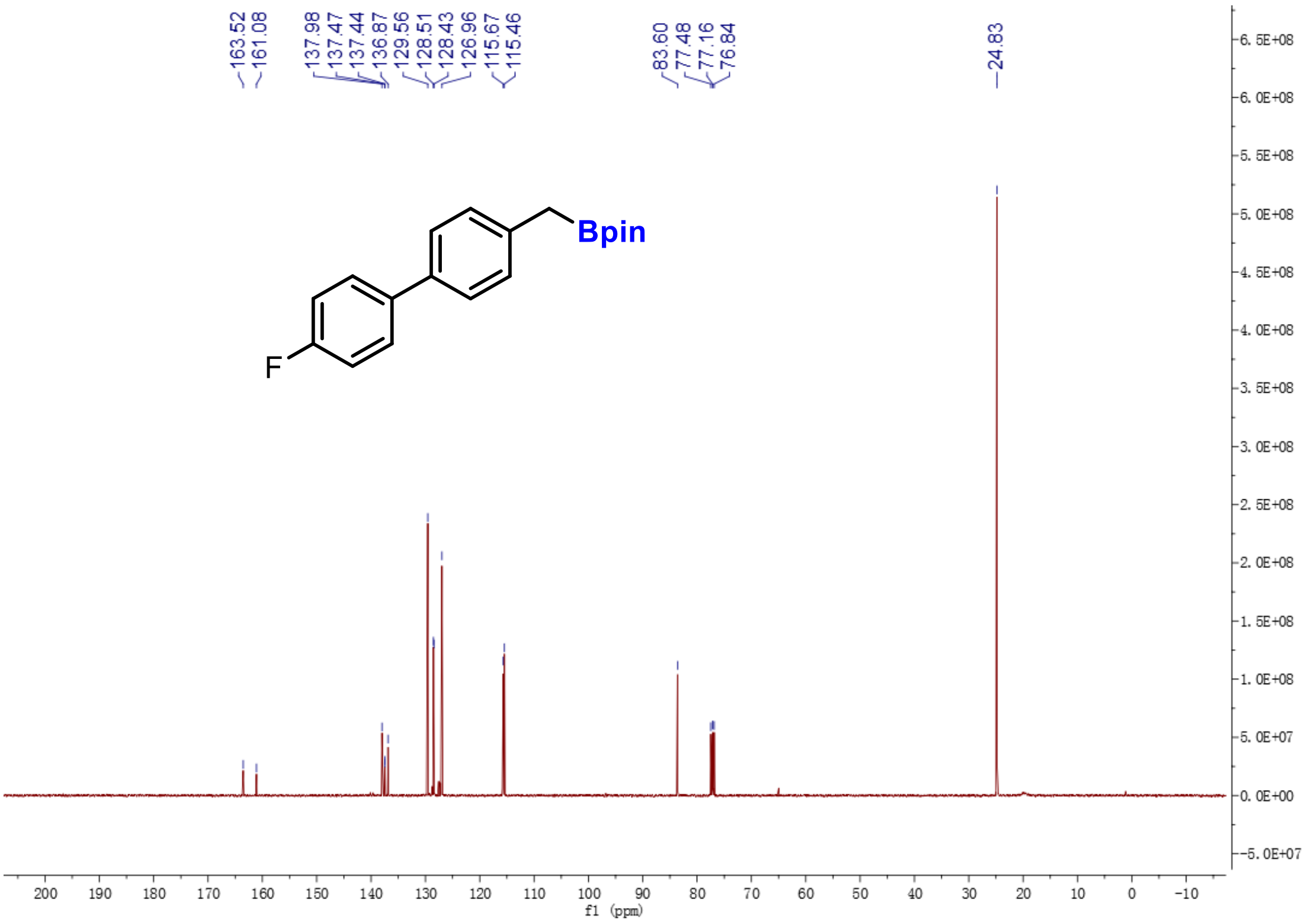

^{19}F NMR spectrum of 9n

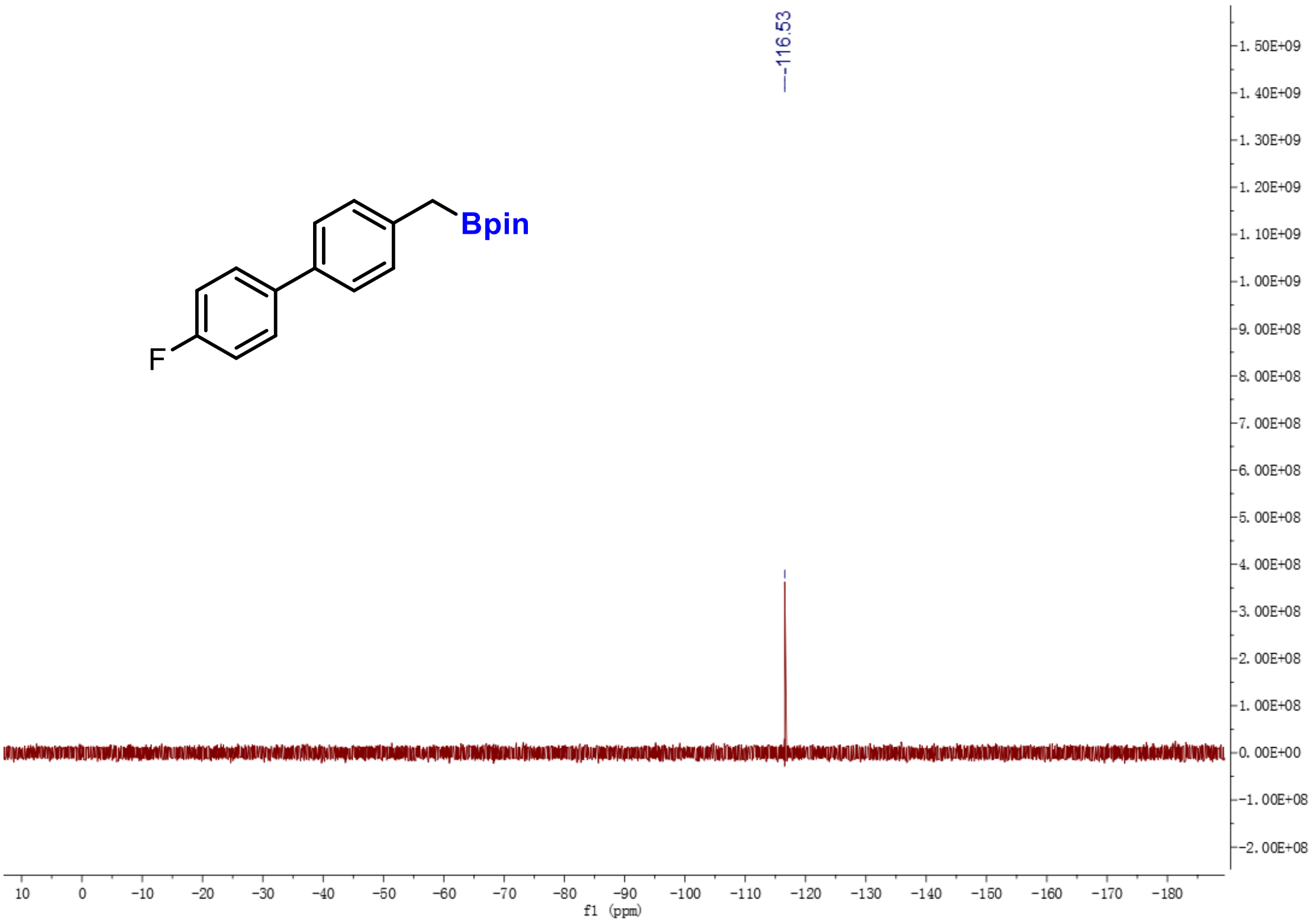

^{1}H NMR spectrum of 9o (400 MHz, $CDCl_3$)

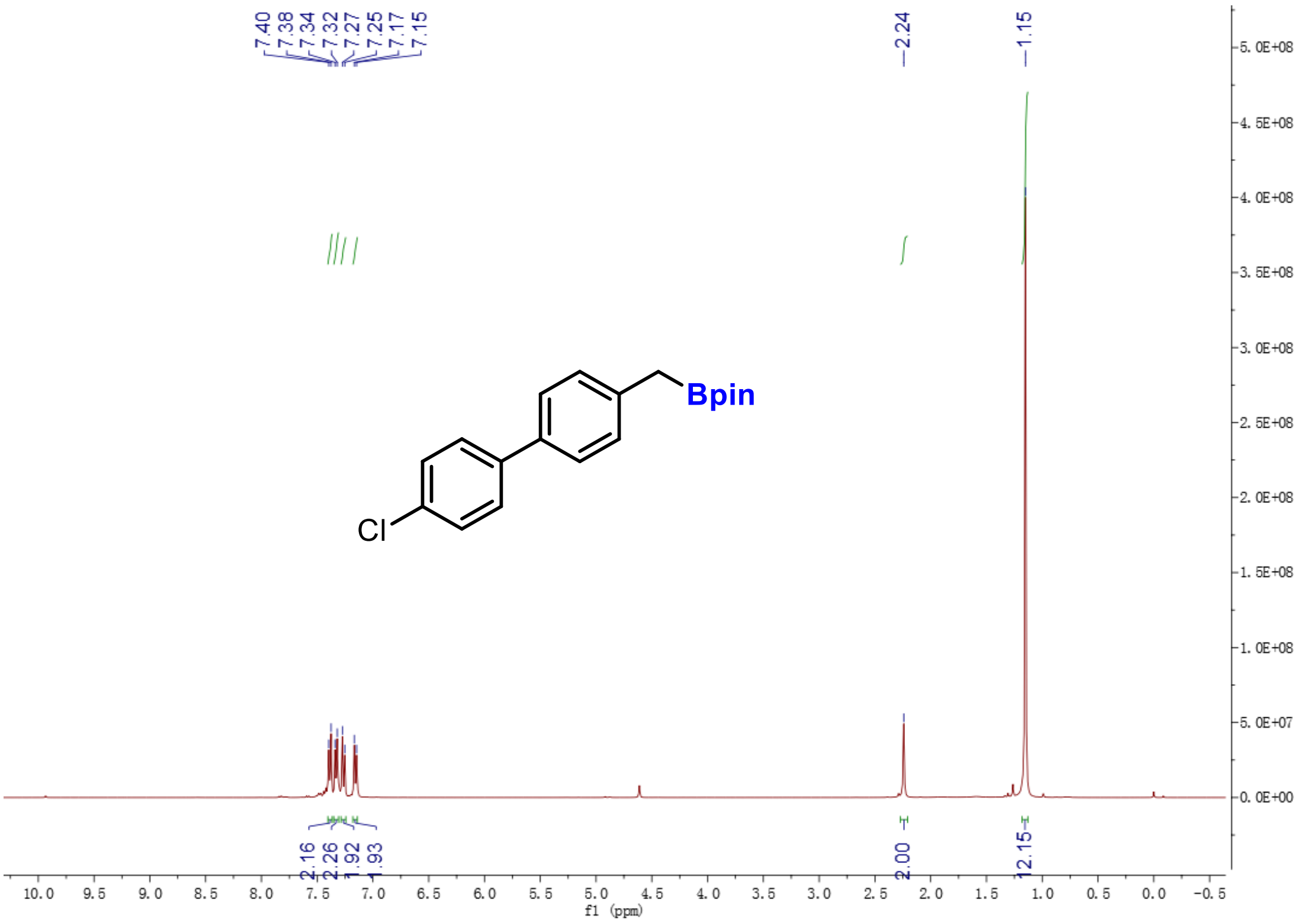

^{13}C NMR spectrum of 9o (101 MHz, $CDCl_3$)

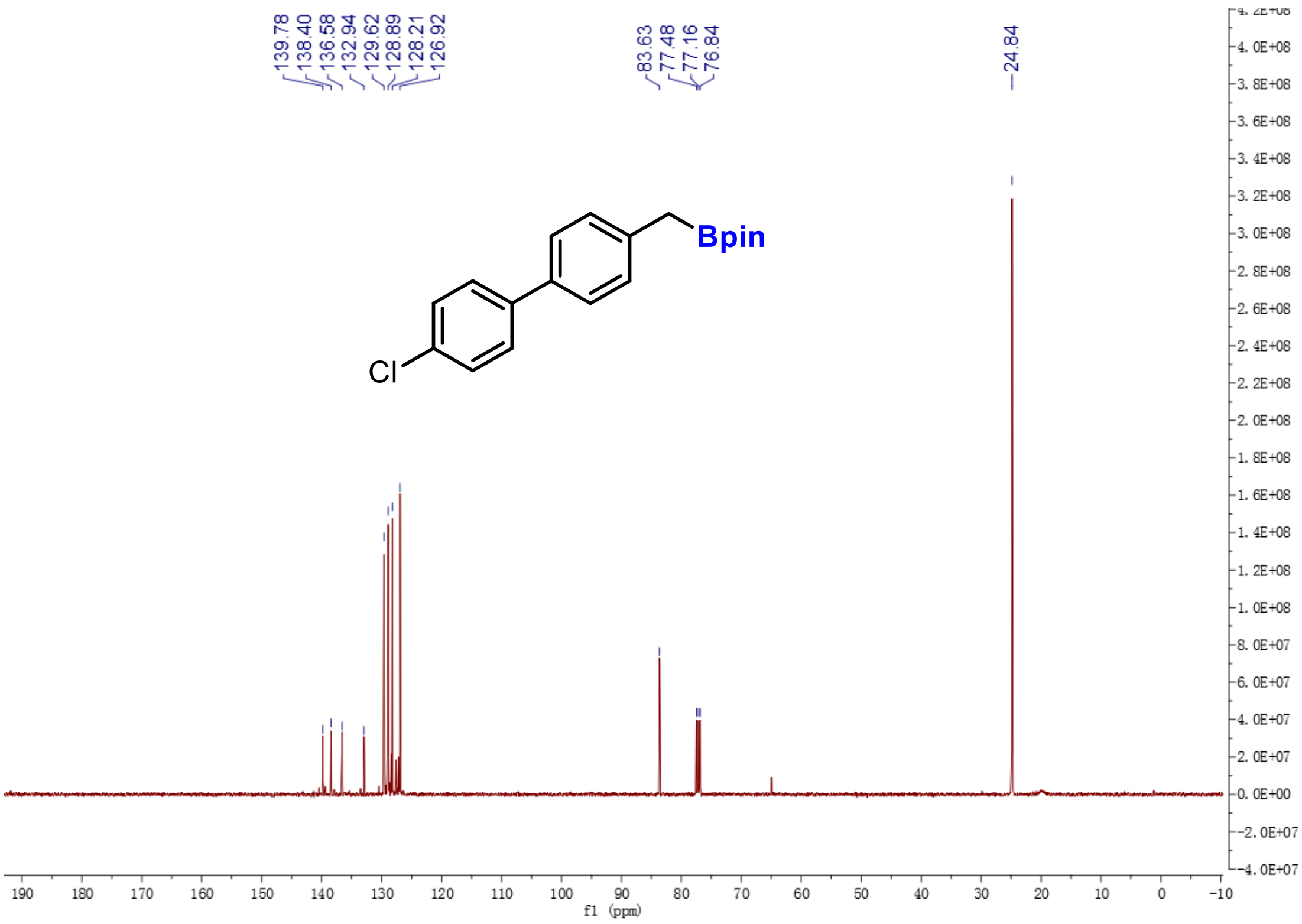

^{1}**H NMR spectrum of 9p** (400 MHz, $CDCl_3$)

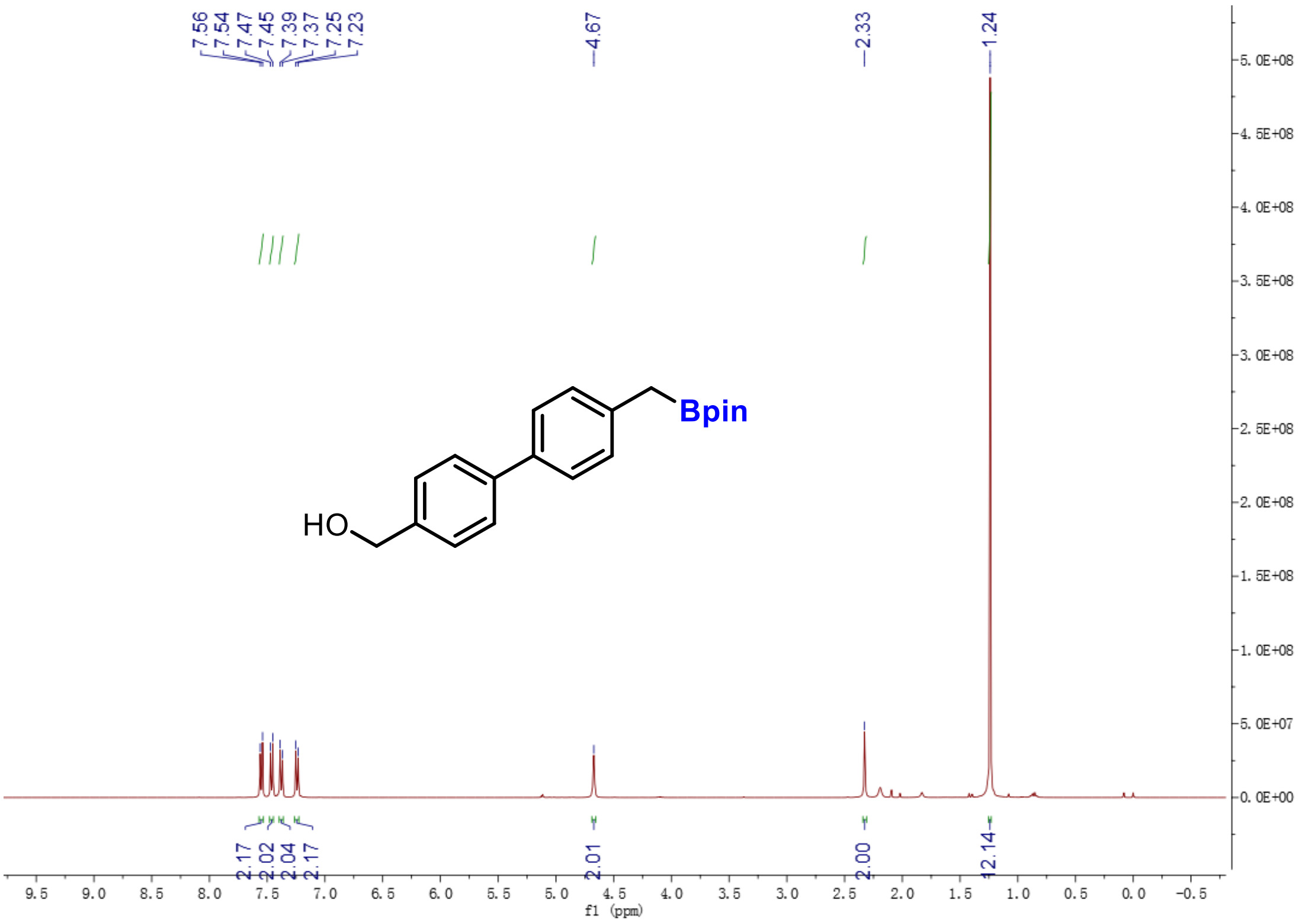

^{13}**C NMR spectrum of 9p** (101 MHz, $CDCl_3$)

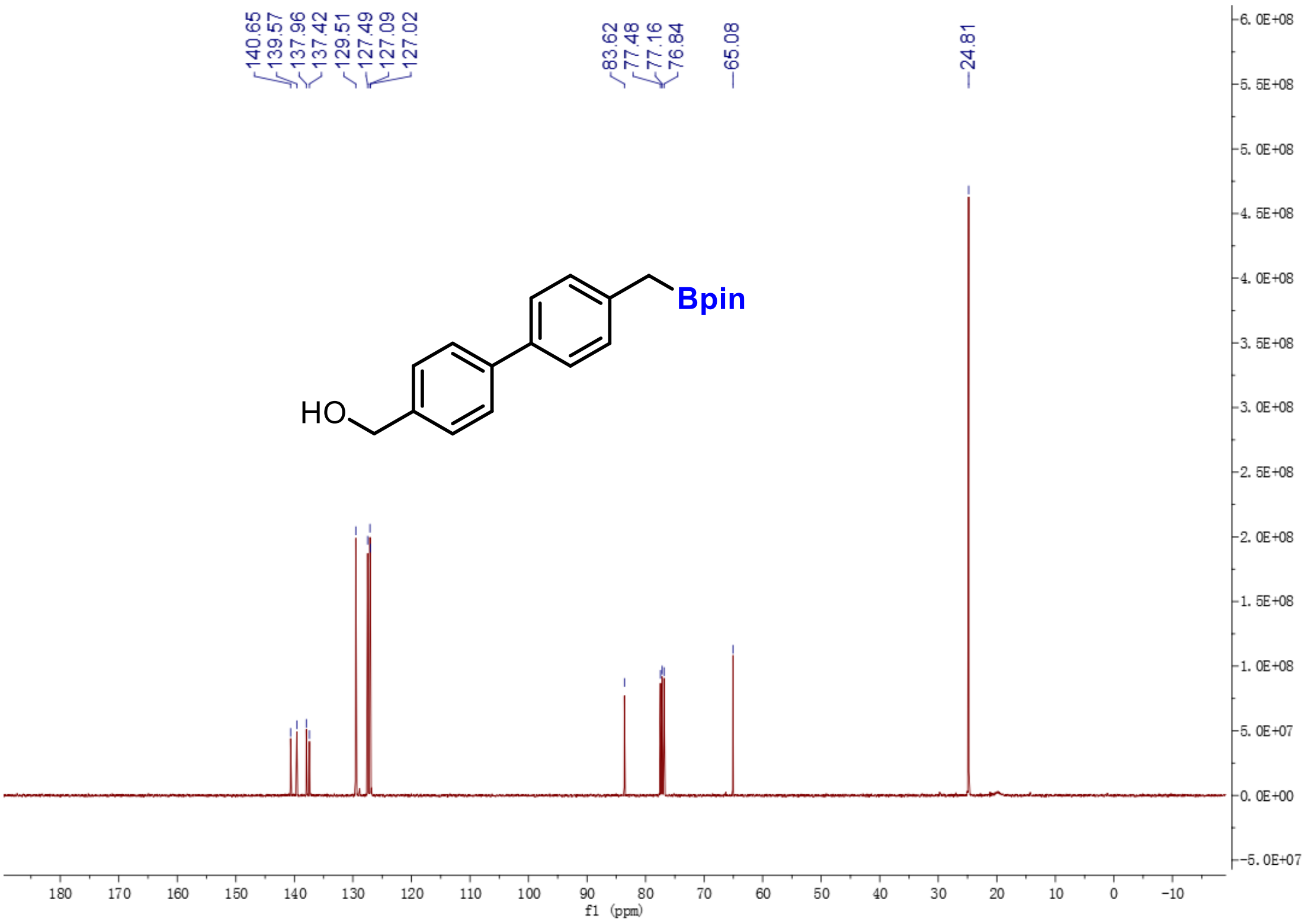

^{1}H NMR spectrum of 9q (400 MHz, $CDCl_3$)

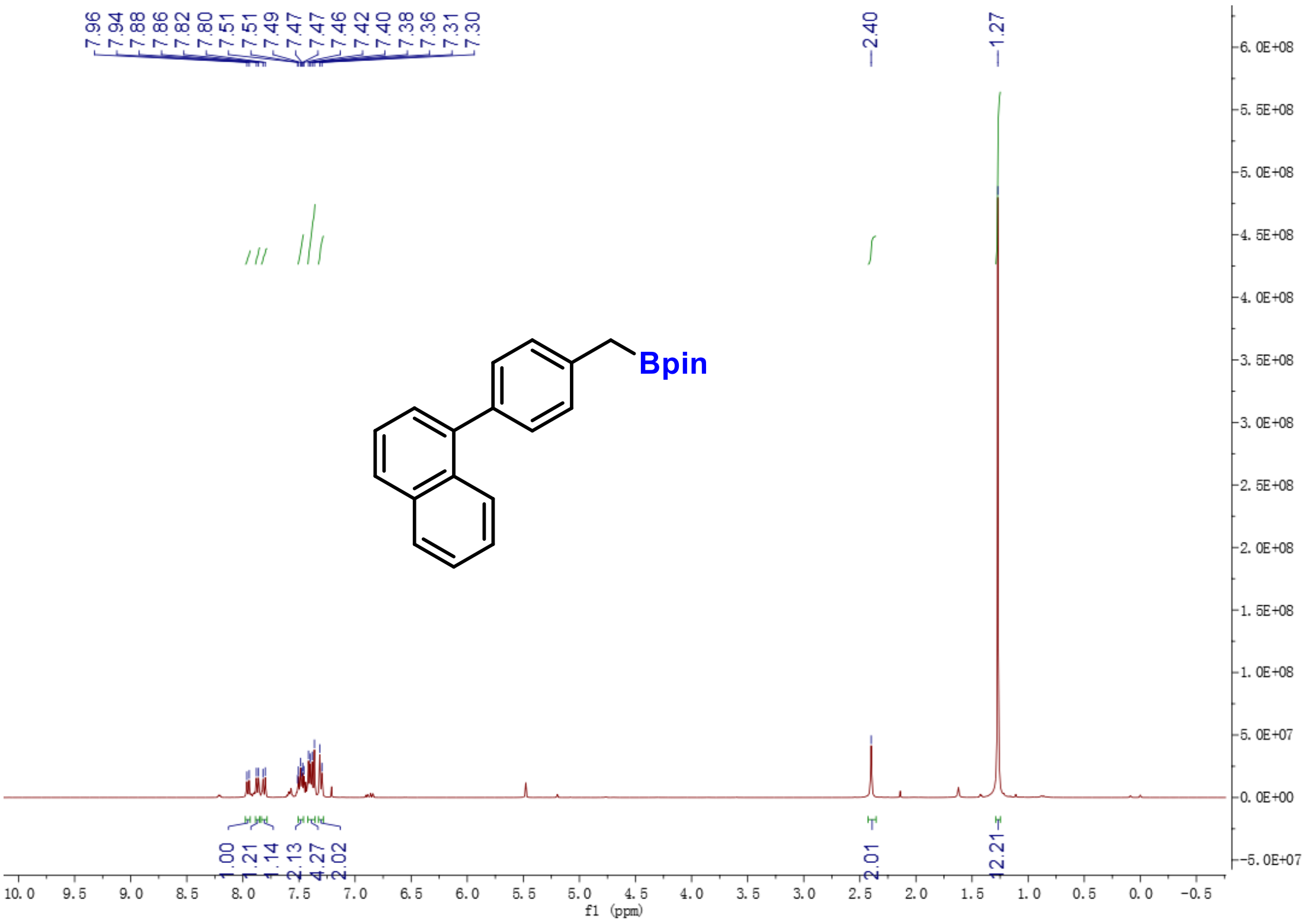

^{13}C NMR spectrum of 9q (101 MHz, $CDCl_3$)

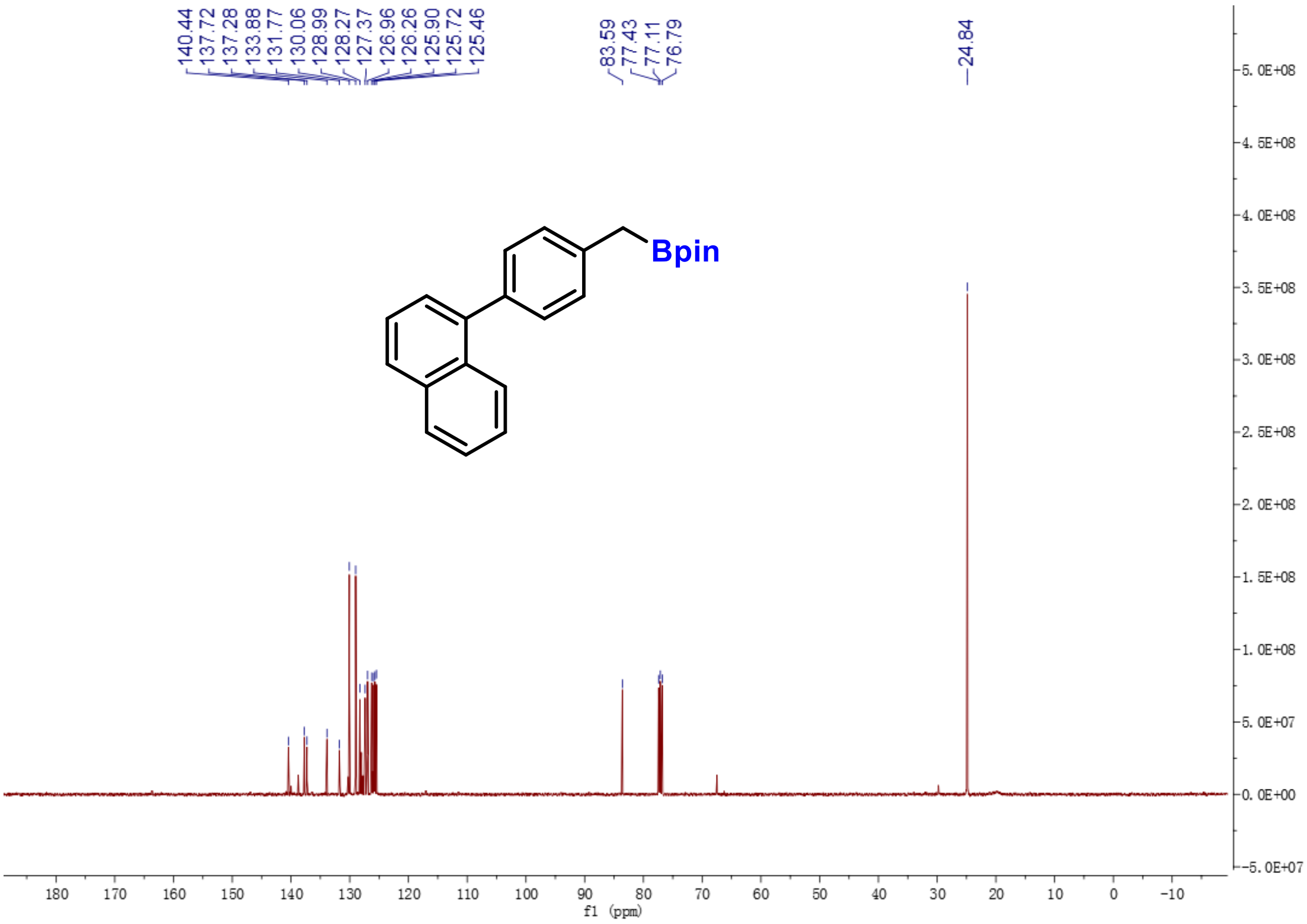

^{1}H NMR spectrum of 9r (400 MHz, $CDCl_3$)

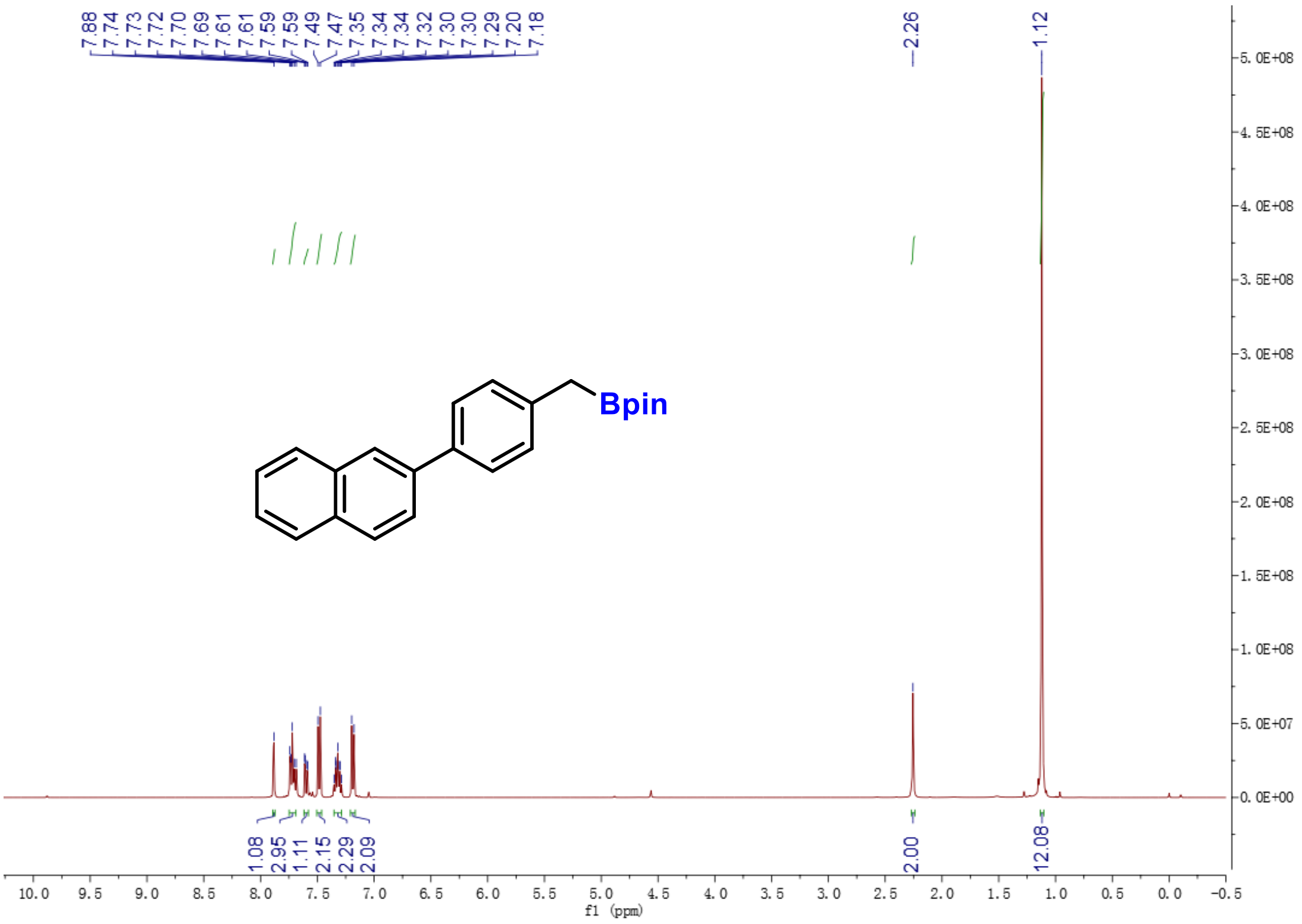

^{13}C NMR spectrum of 9r (101 MHz, $CDCl_3$)

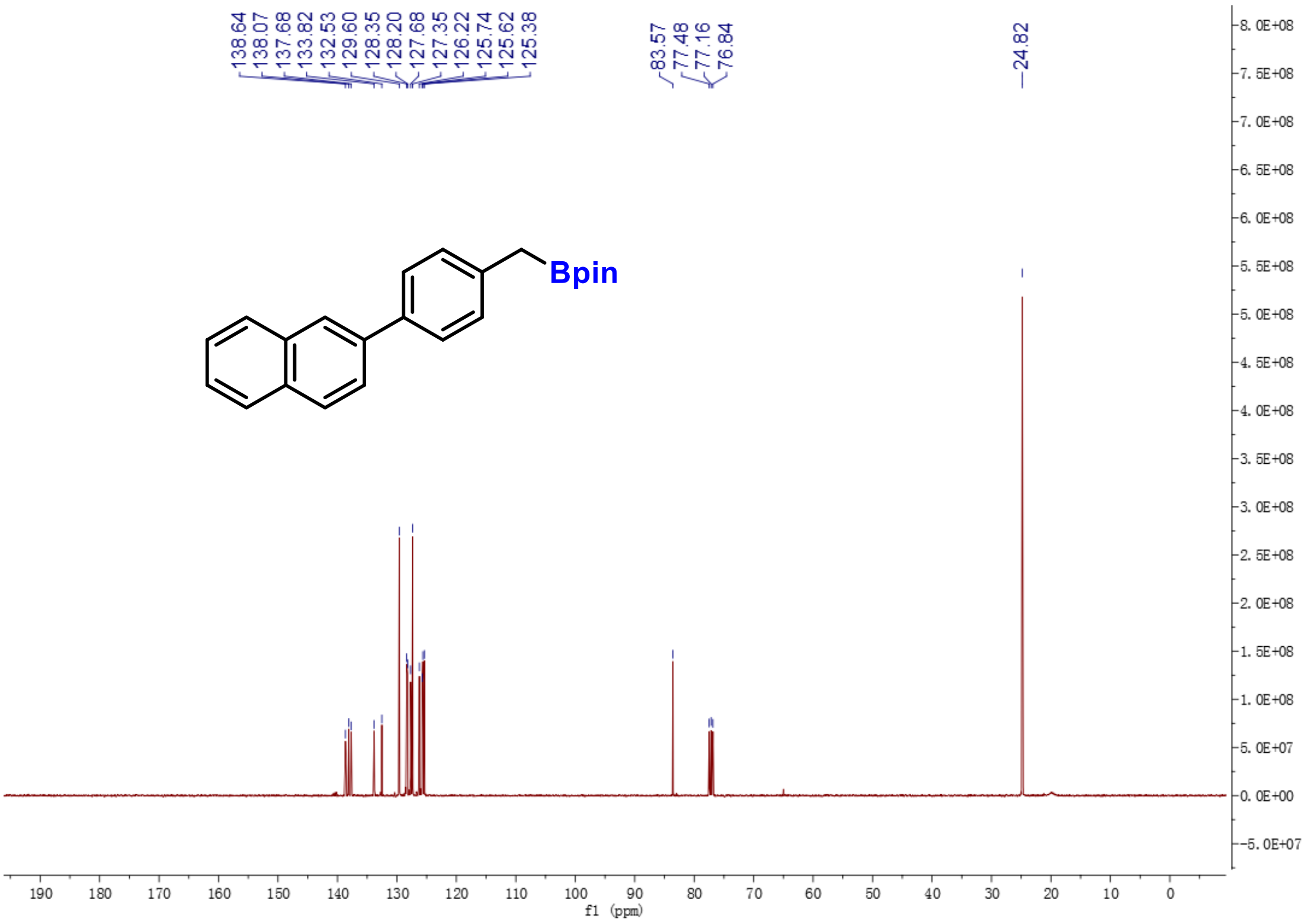

^{1}H NMR spectrum of 9s (400 MHz, $CDCl_3$)

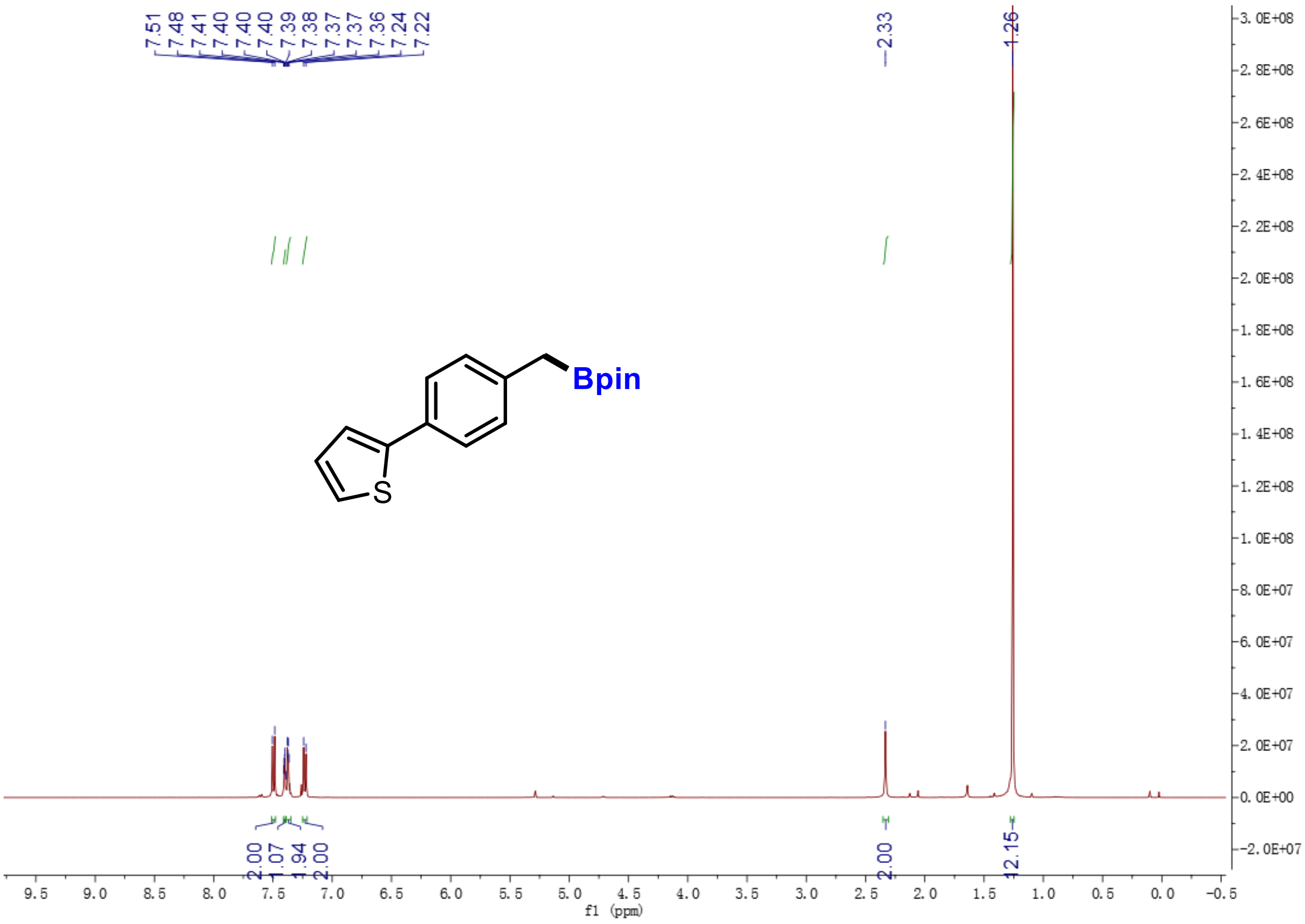

^{13}C NMR spectrum of 9s (101 MHz, $CDCl_3$)

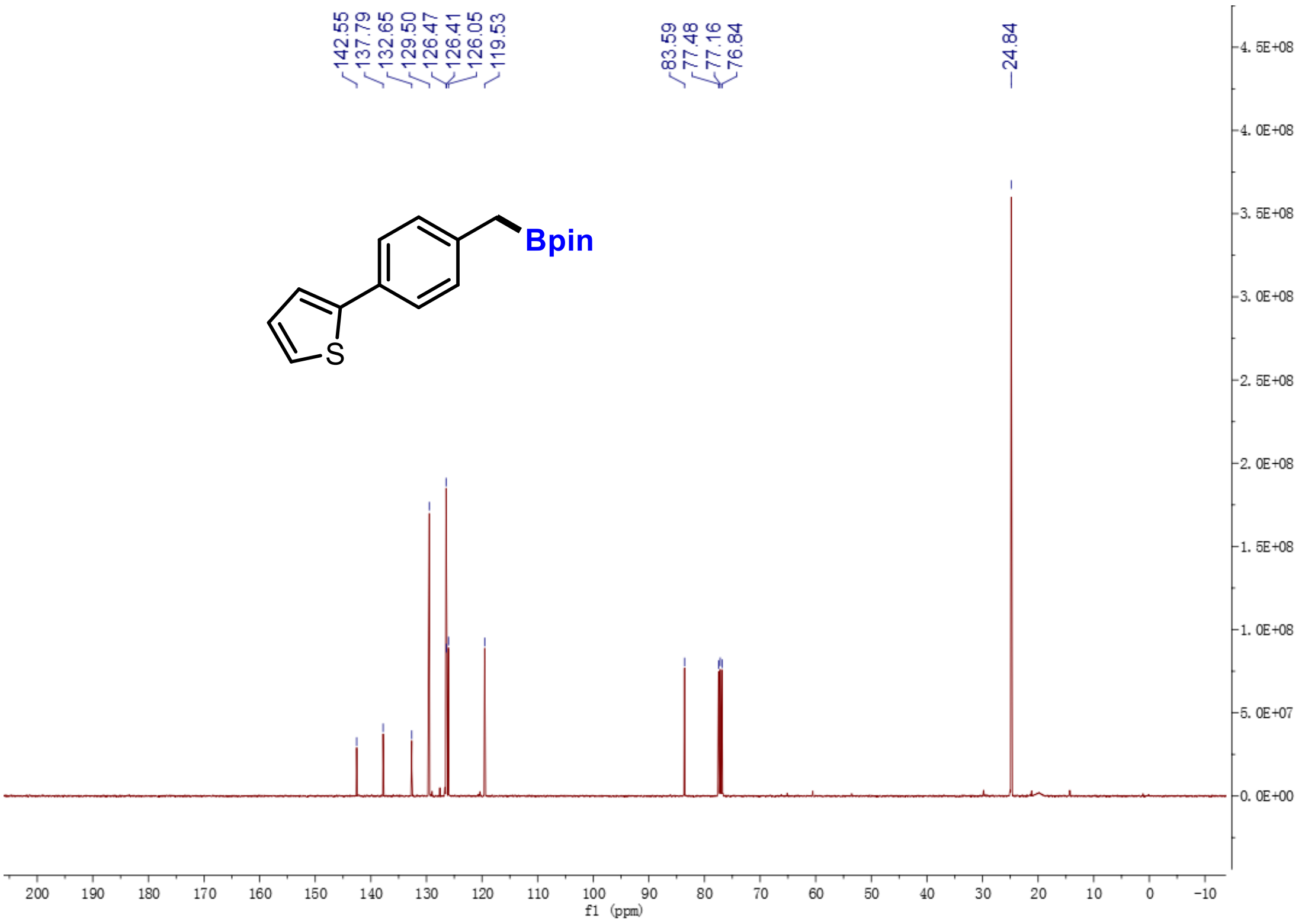

^{1}H NMR spectrum of 9t (400 MHz, $CDCl_3$)

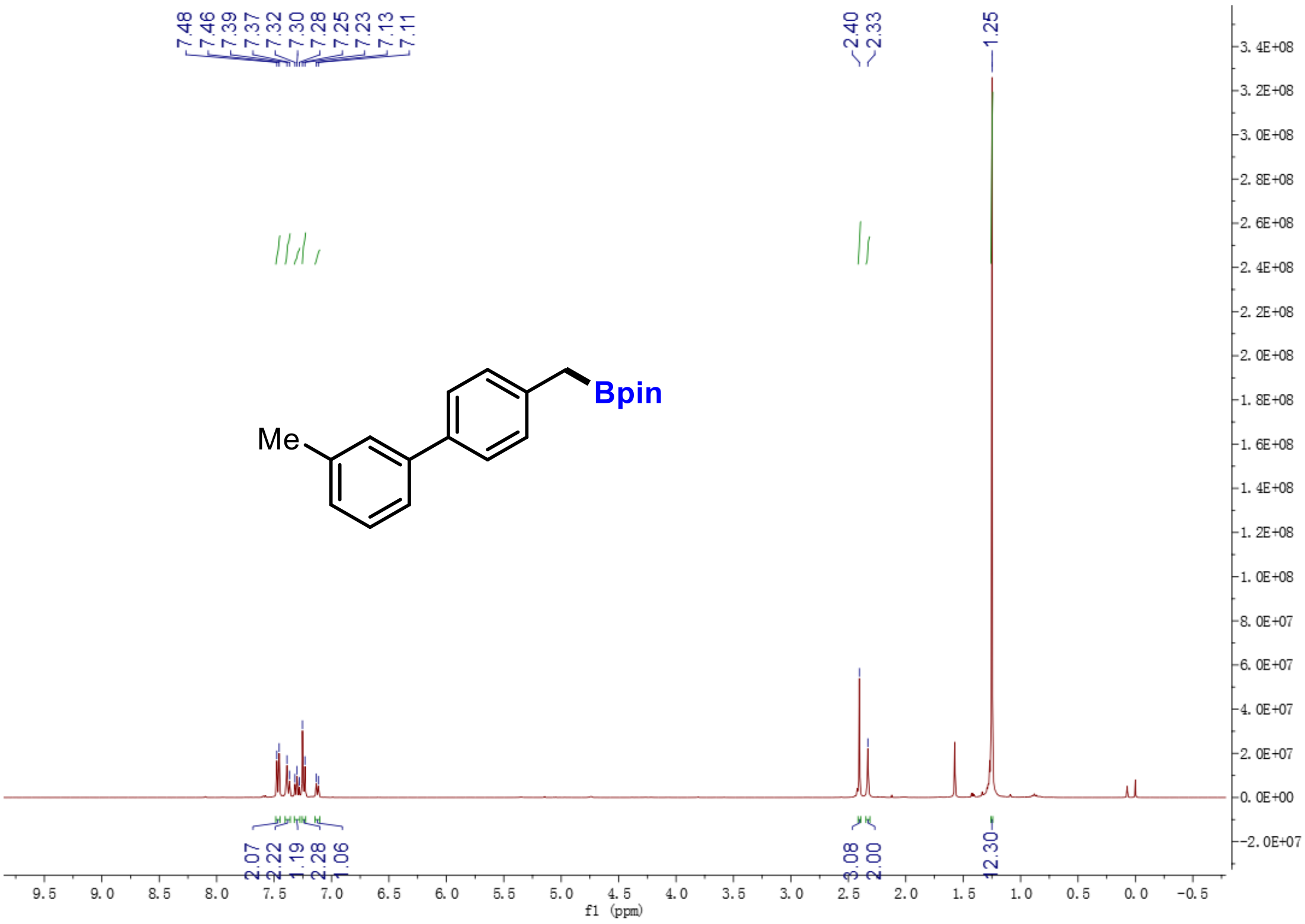

^{13}C NMR spectrum of 9t (101 MHz, $CDCl_3$)

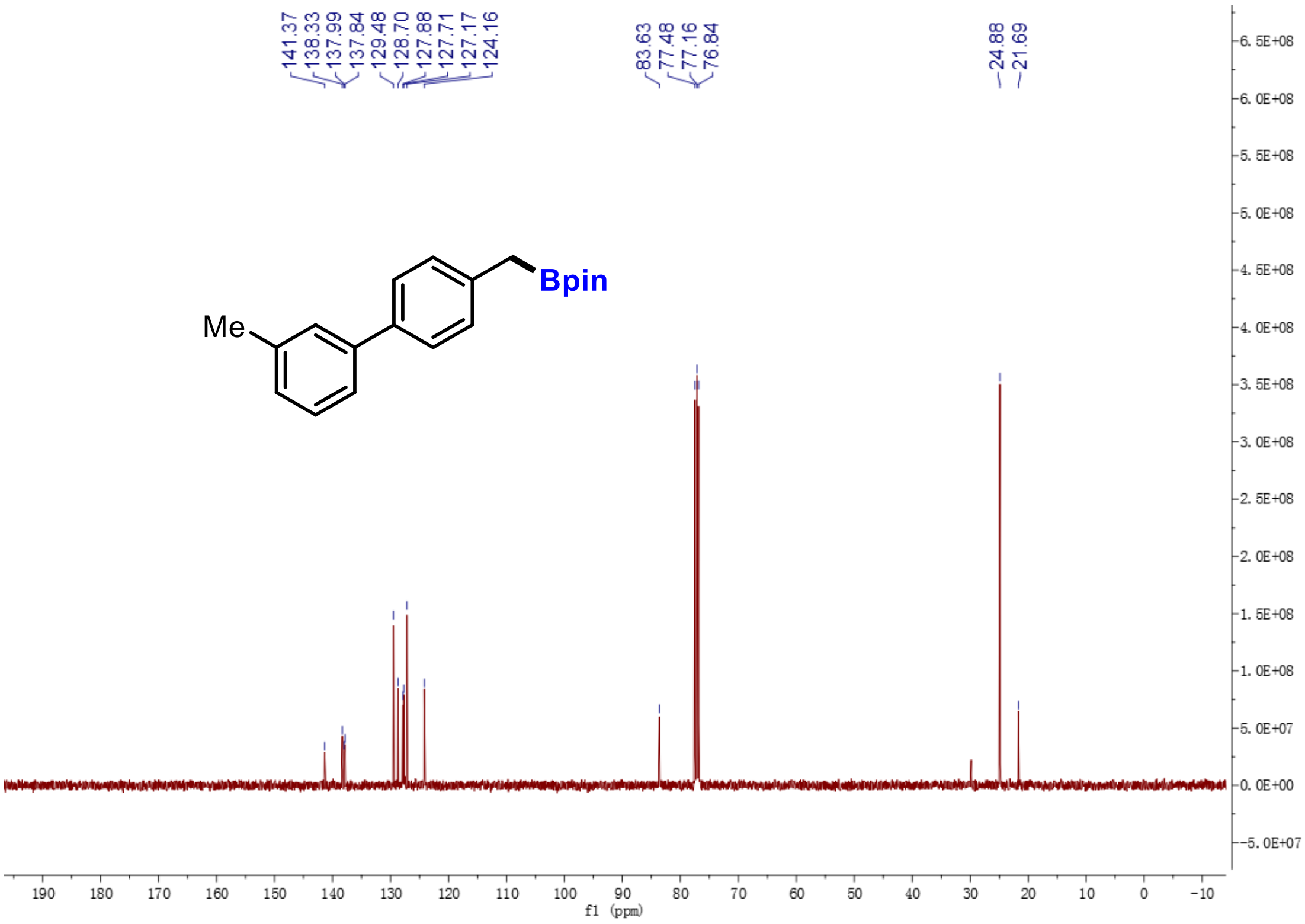

^{1}H NMR spectrum of 9u (400 MHz, $CDCl_3$)

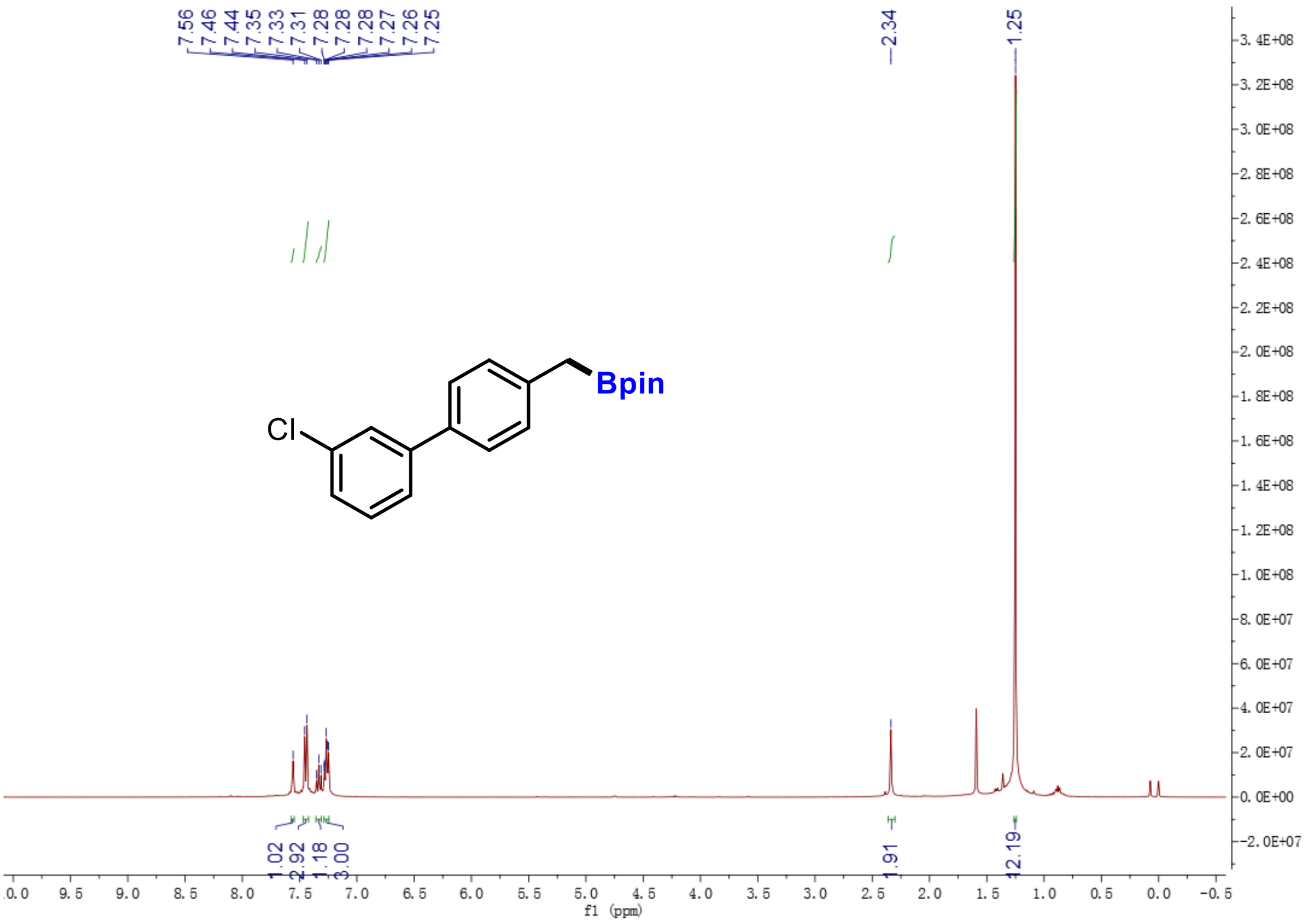

^{13}C NMR spectrum of 9u (101 MHz, $CDCl_3$)

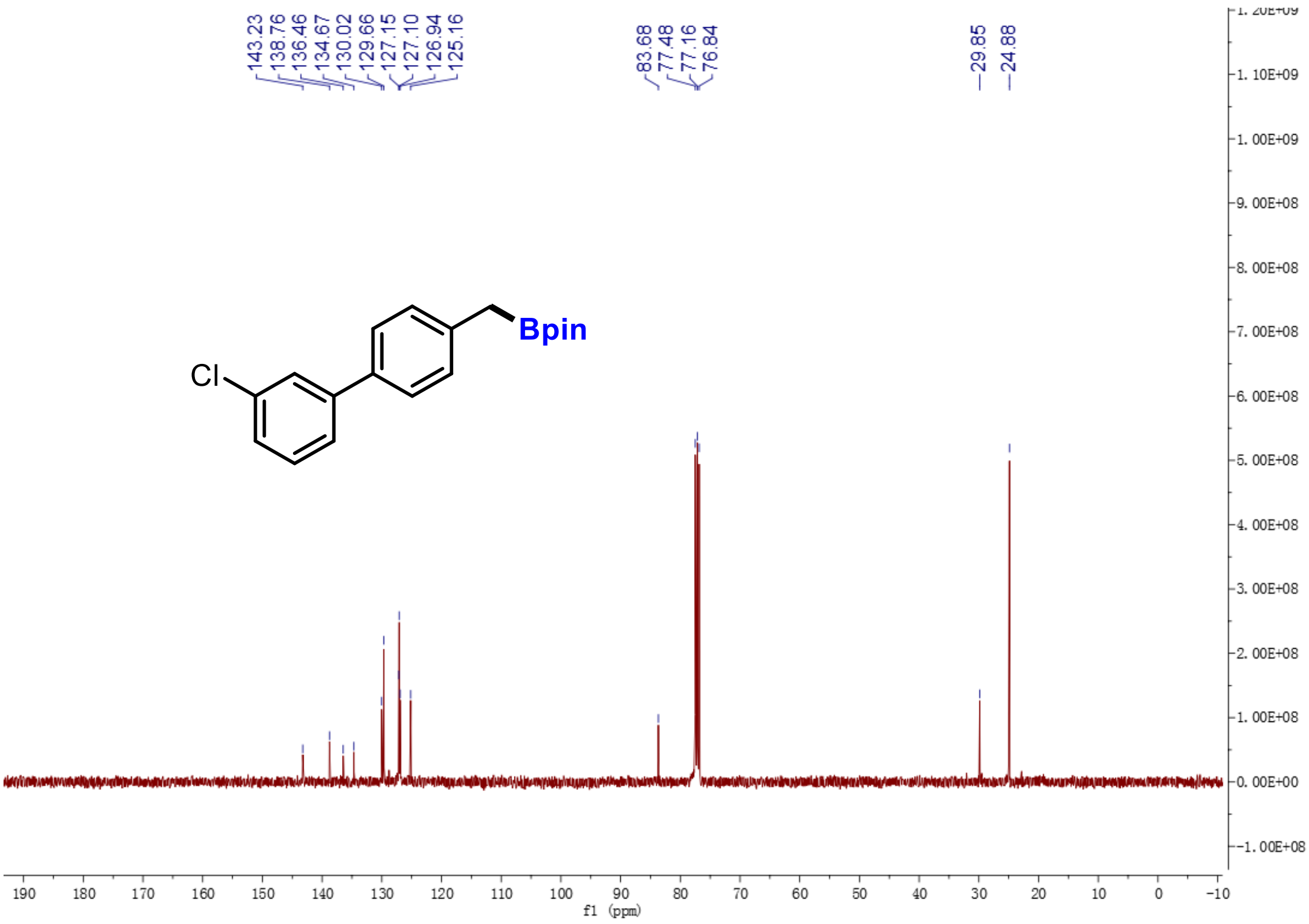

¹H NMR spectrum of 9v (400 MHz, $CDCl_3$)

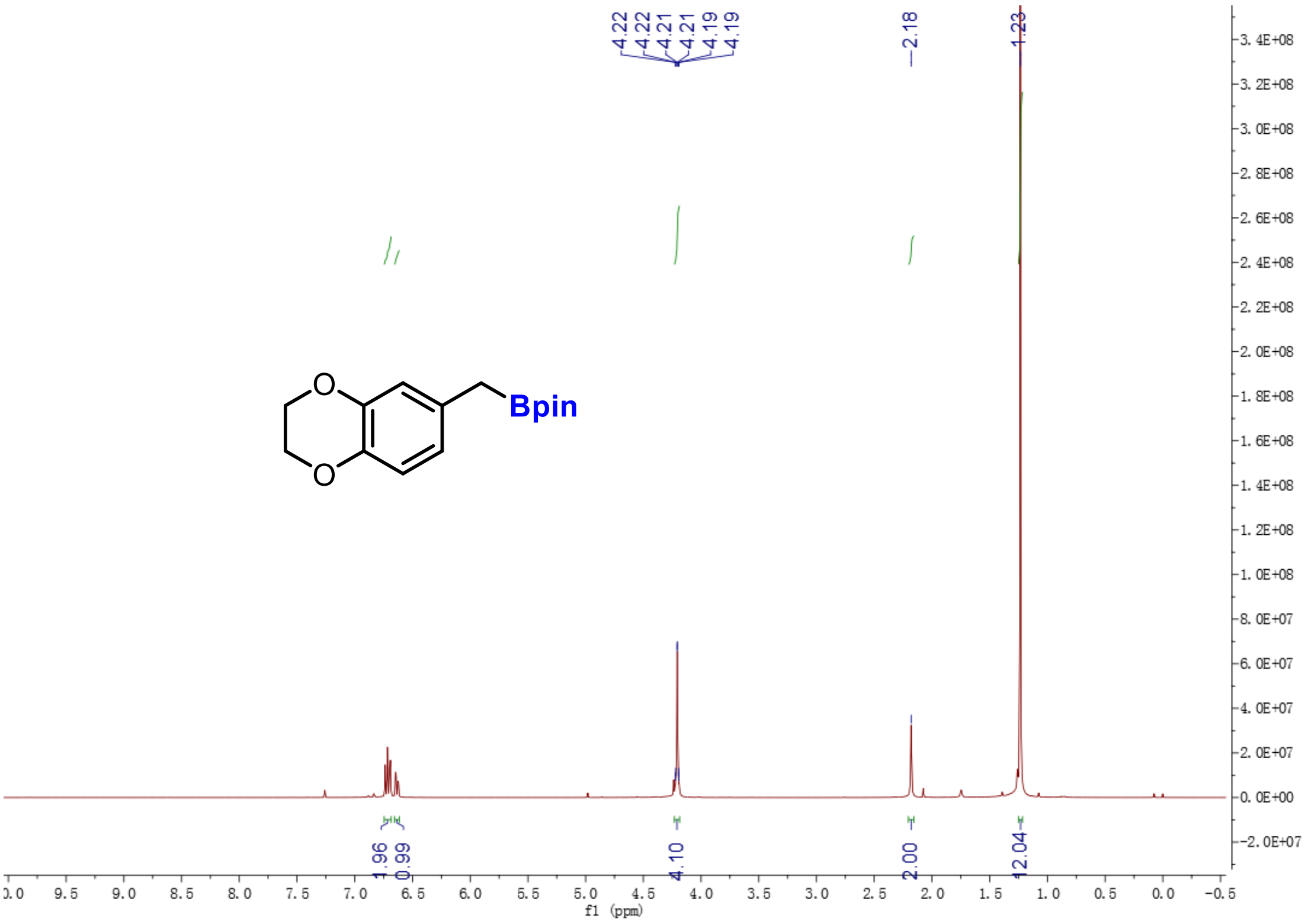

¹³C NMR spectrum of 9v (101 MHz, $CDCl_3$)

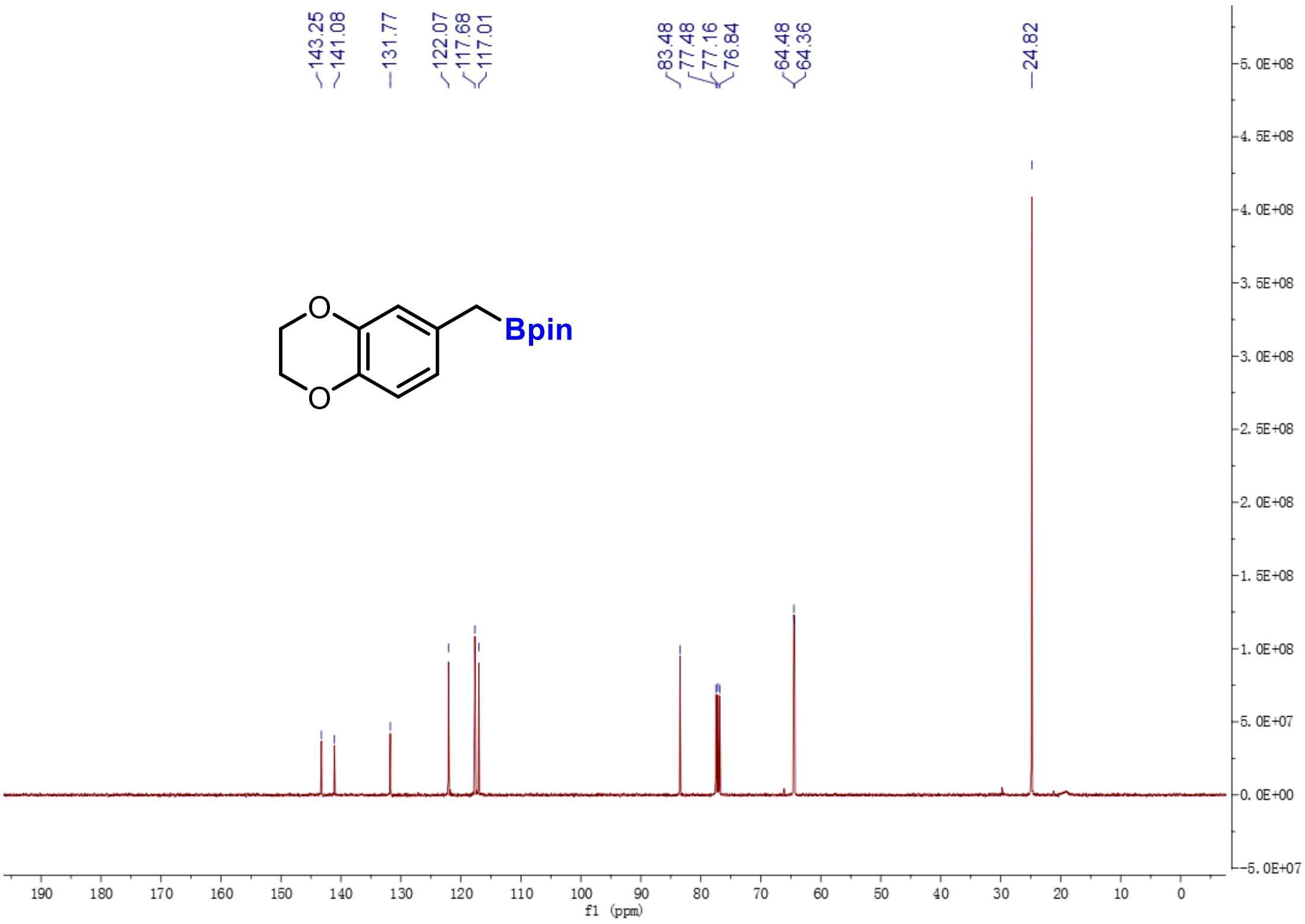

^{1}H NMR spectrum of 9w (400 MHz, $CDCl_3$)

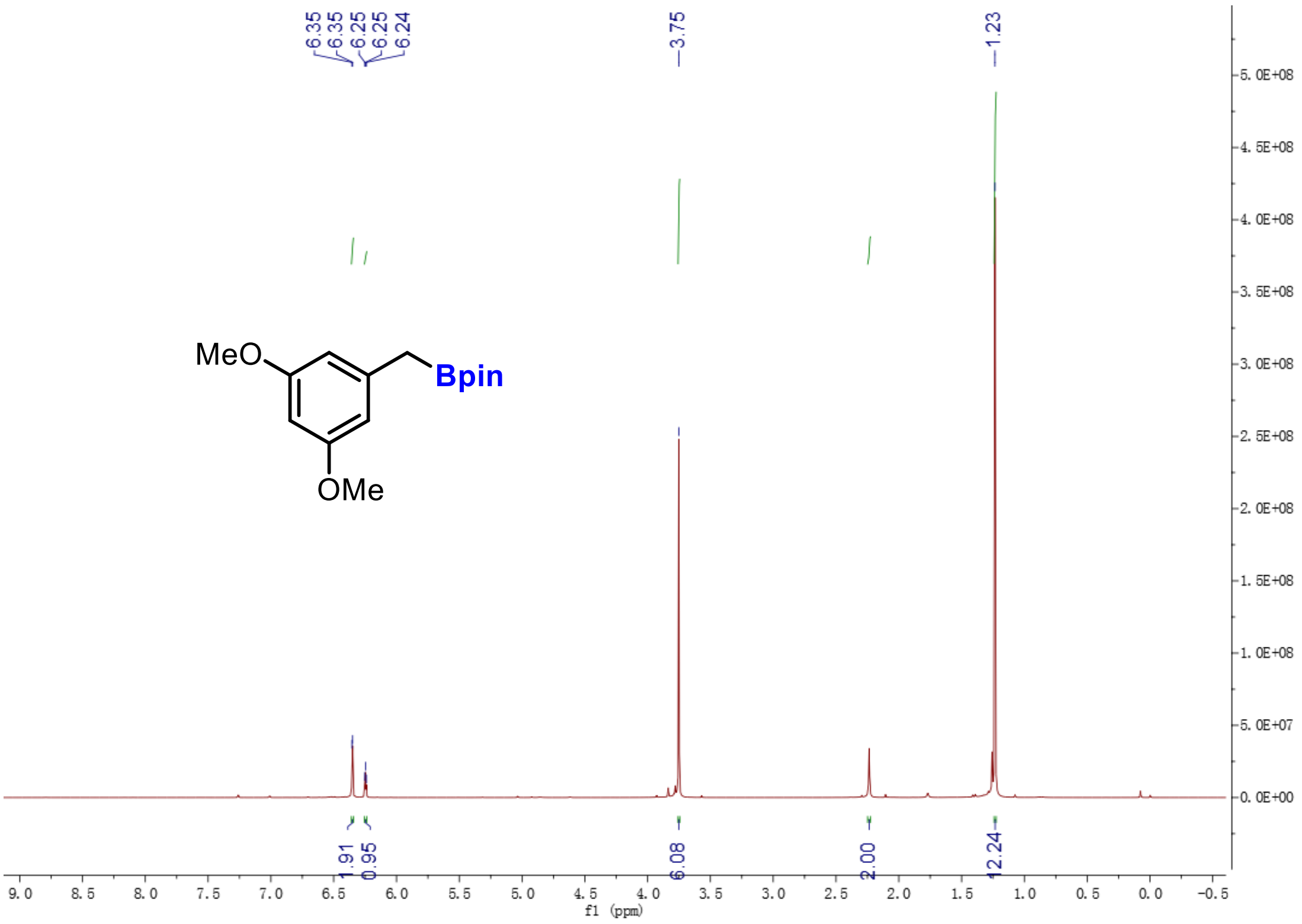

^{13}C NMR spectrum of 9w (101 MHz, $CDCl_3$)

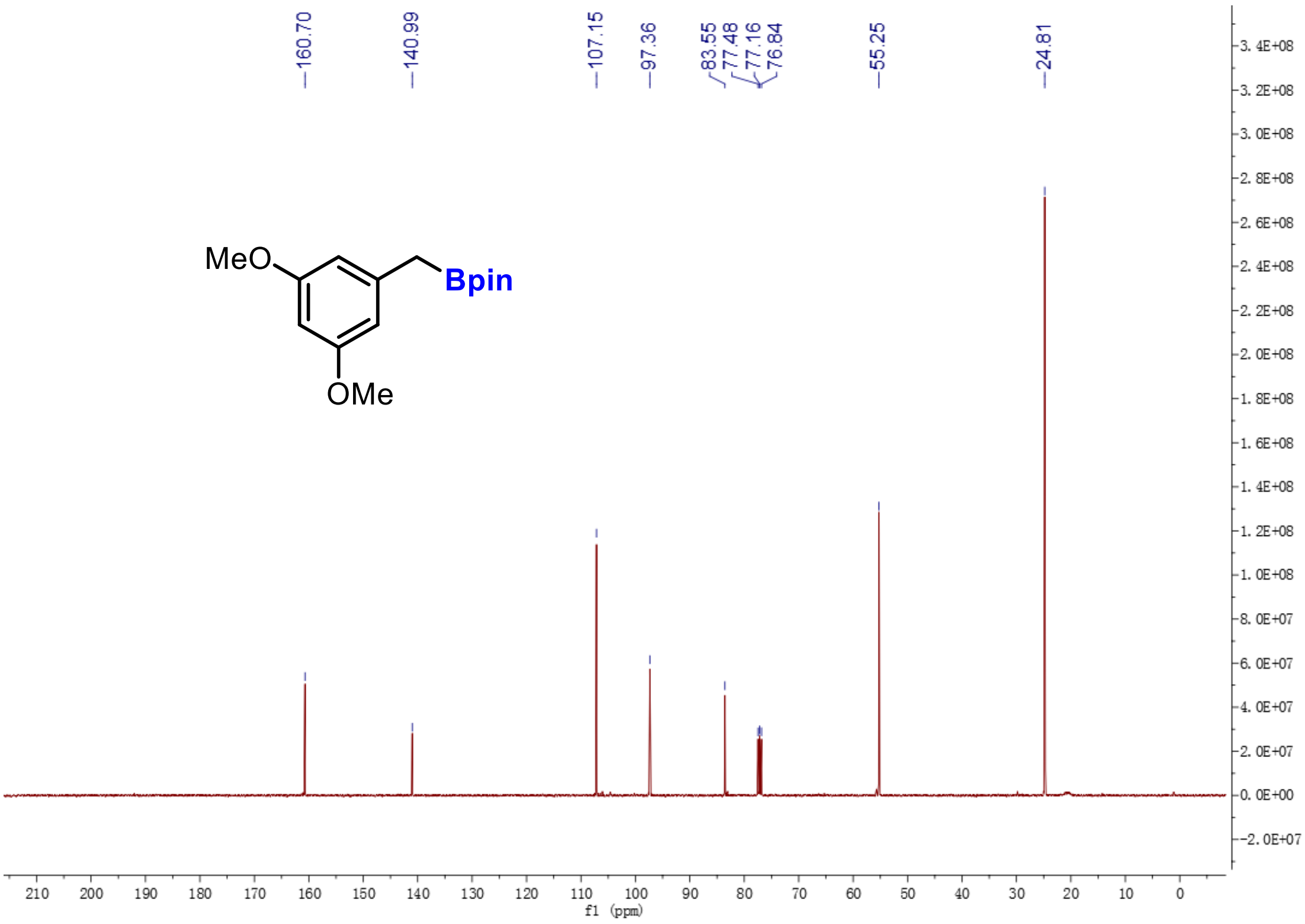

^{1}H NMR spectrum of 9x (400 MHz, $CDCl_3$)

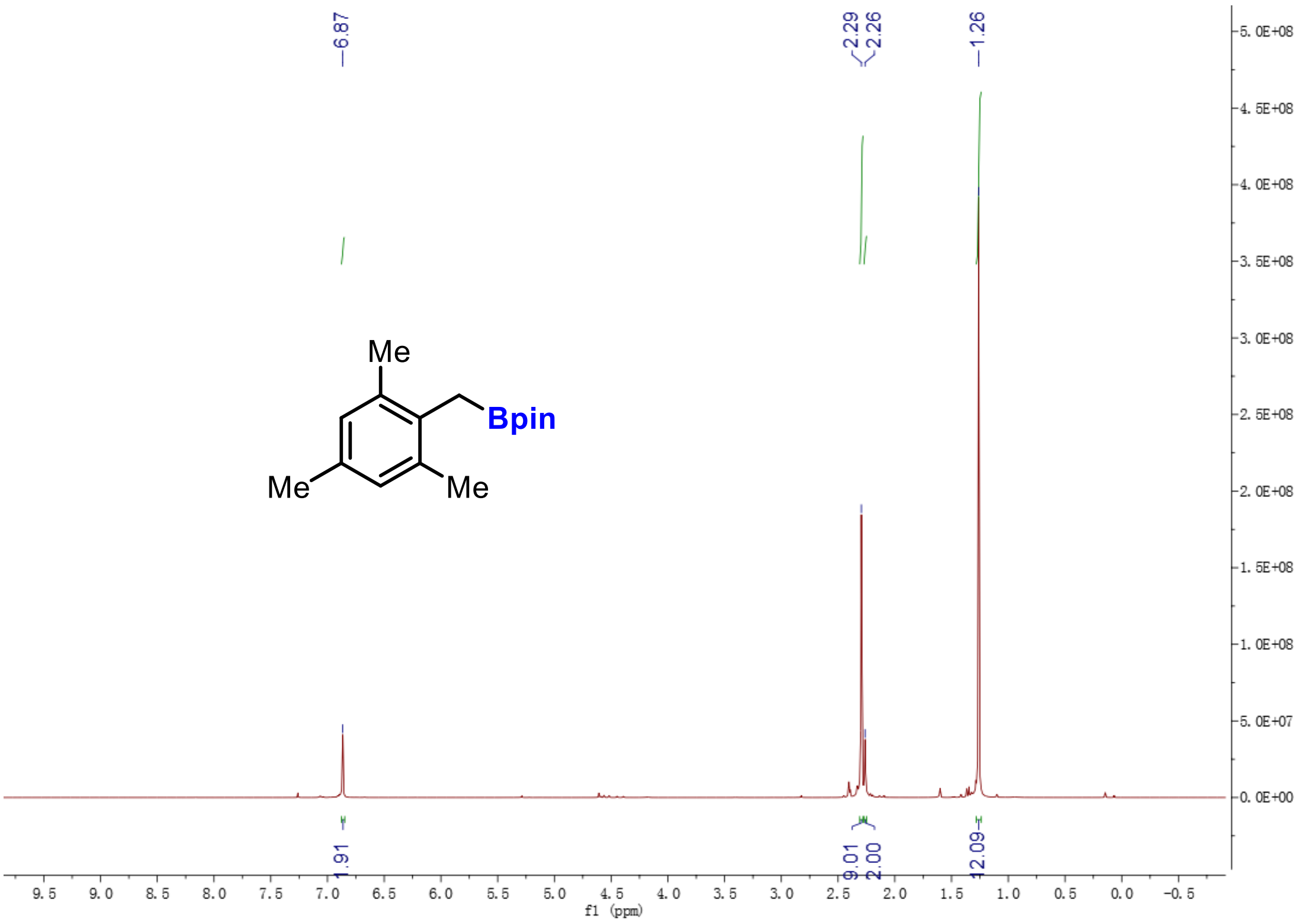

^{13}C NMR spectrum of 9x (101 MHz, $CDCl_3$)

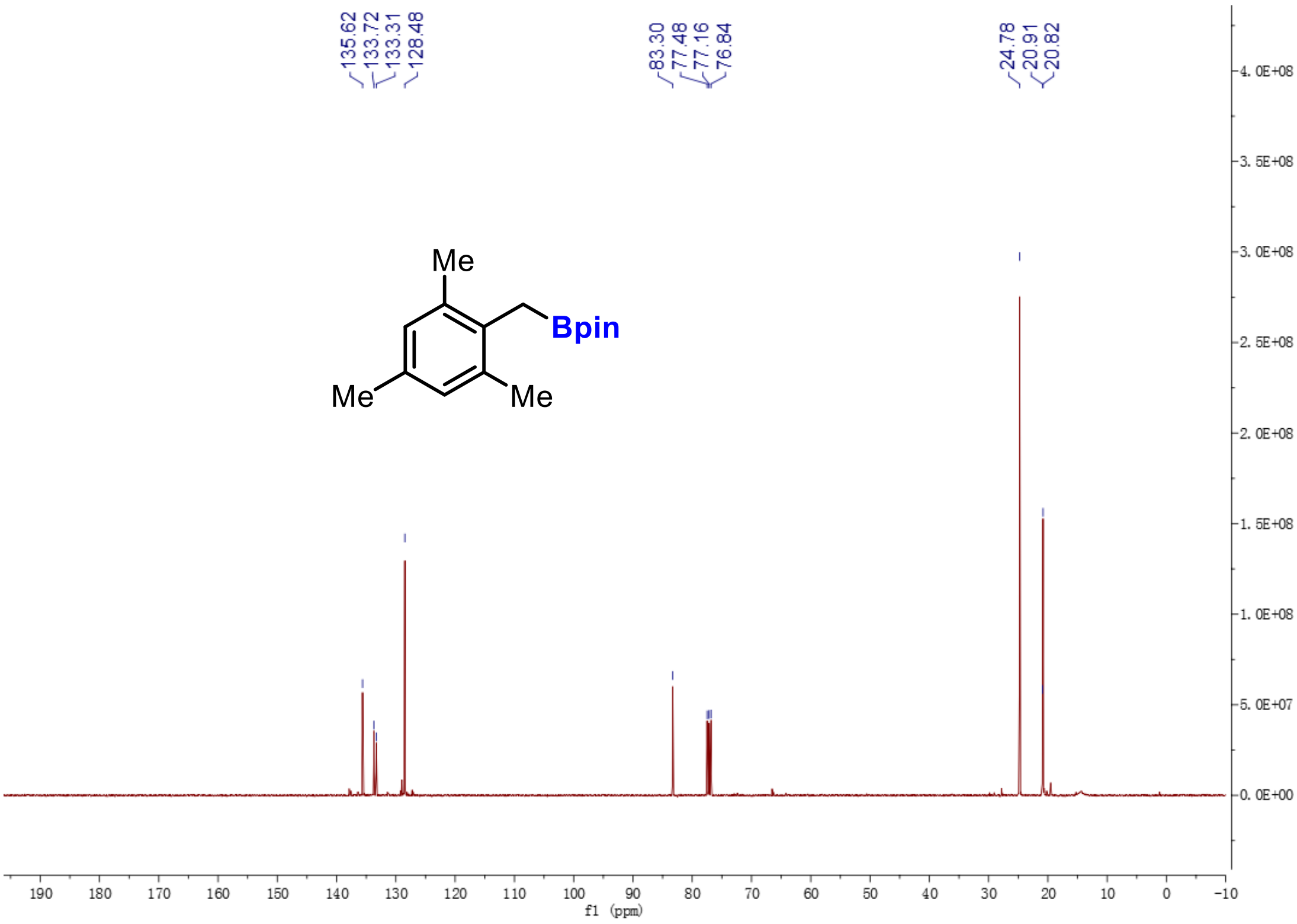

^{1}H NMR spectrum of 9y (400 MHz, $CDCl_3$)

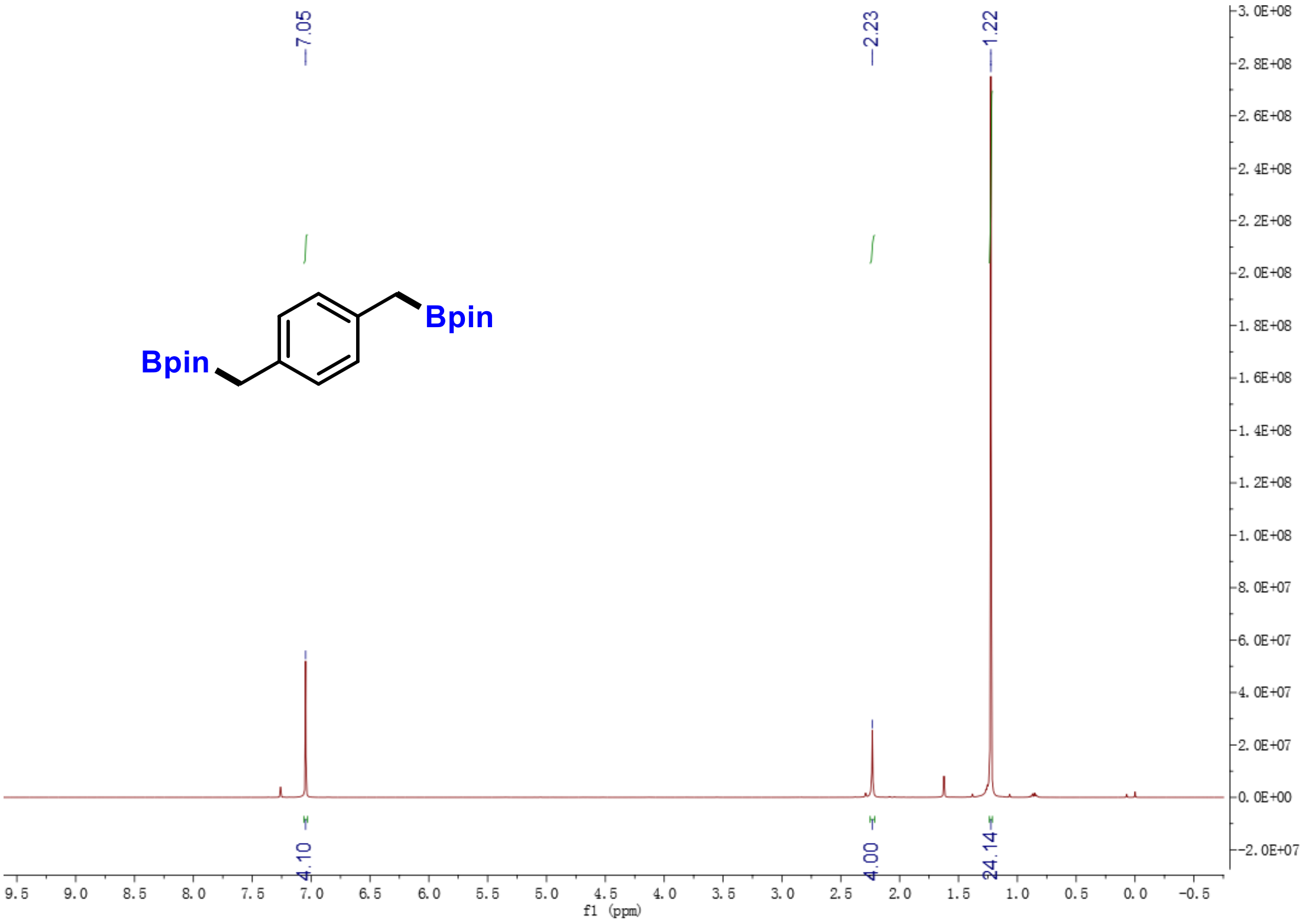

^{13}C NMR spectrum of 9y (101 MHz, $CDCl_3$)

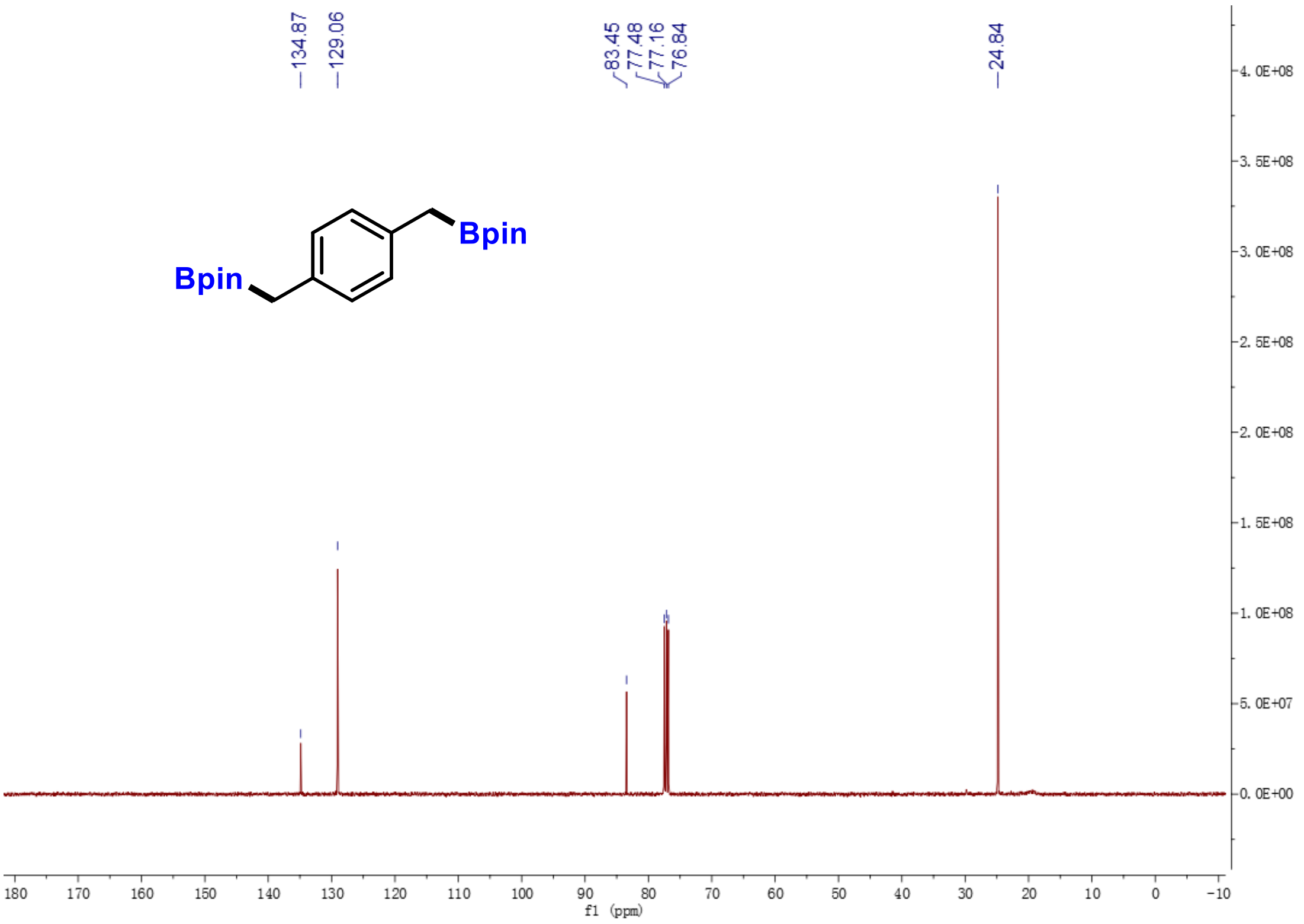

^{1}H **NMR spectrum of 9z** (400 MHz, $CDCl_3$)

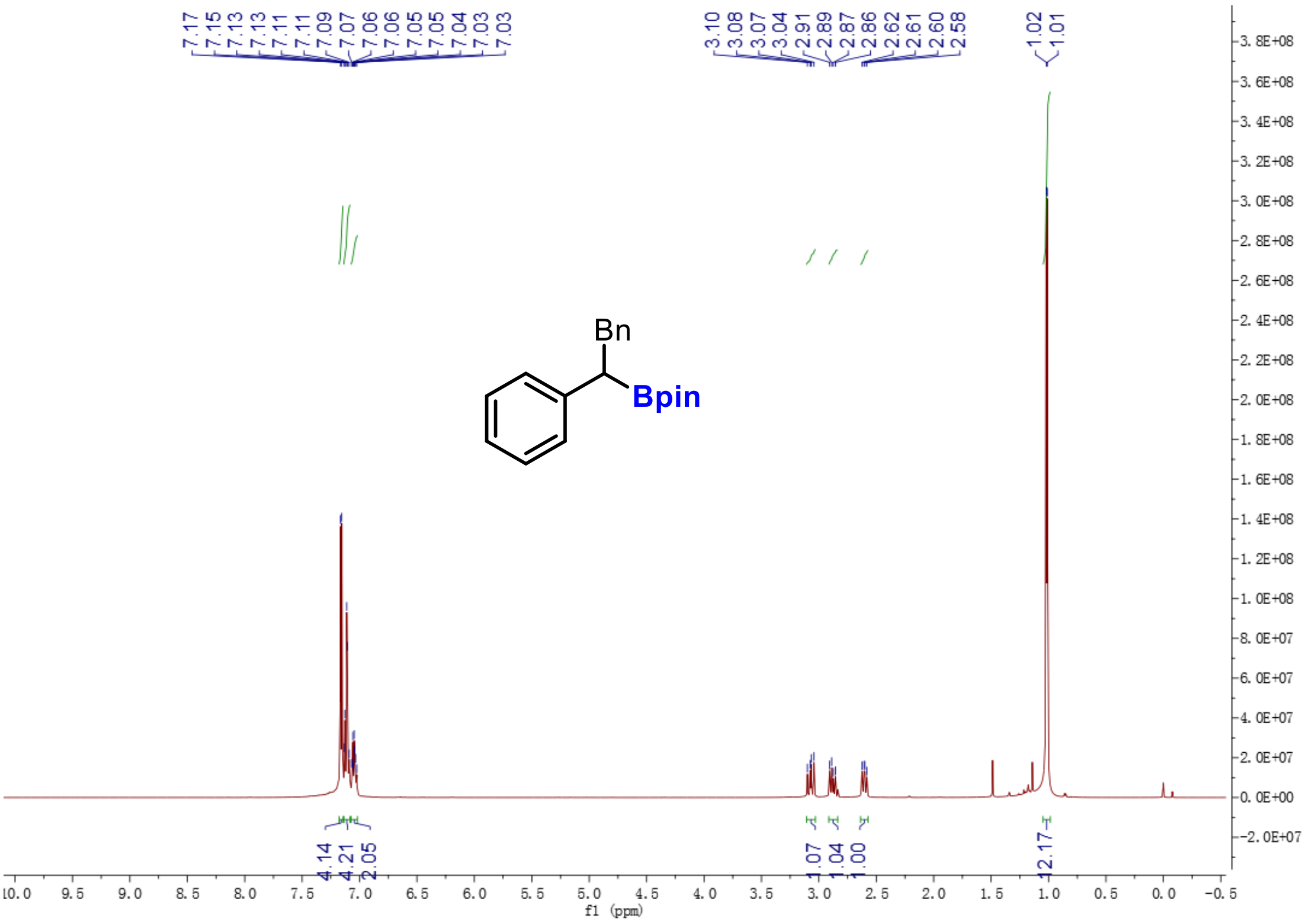

^{13}C **NMR spectrum of 9z** (101 MHz, $CDCl_3$)

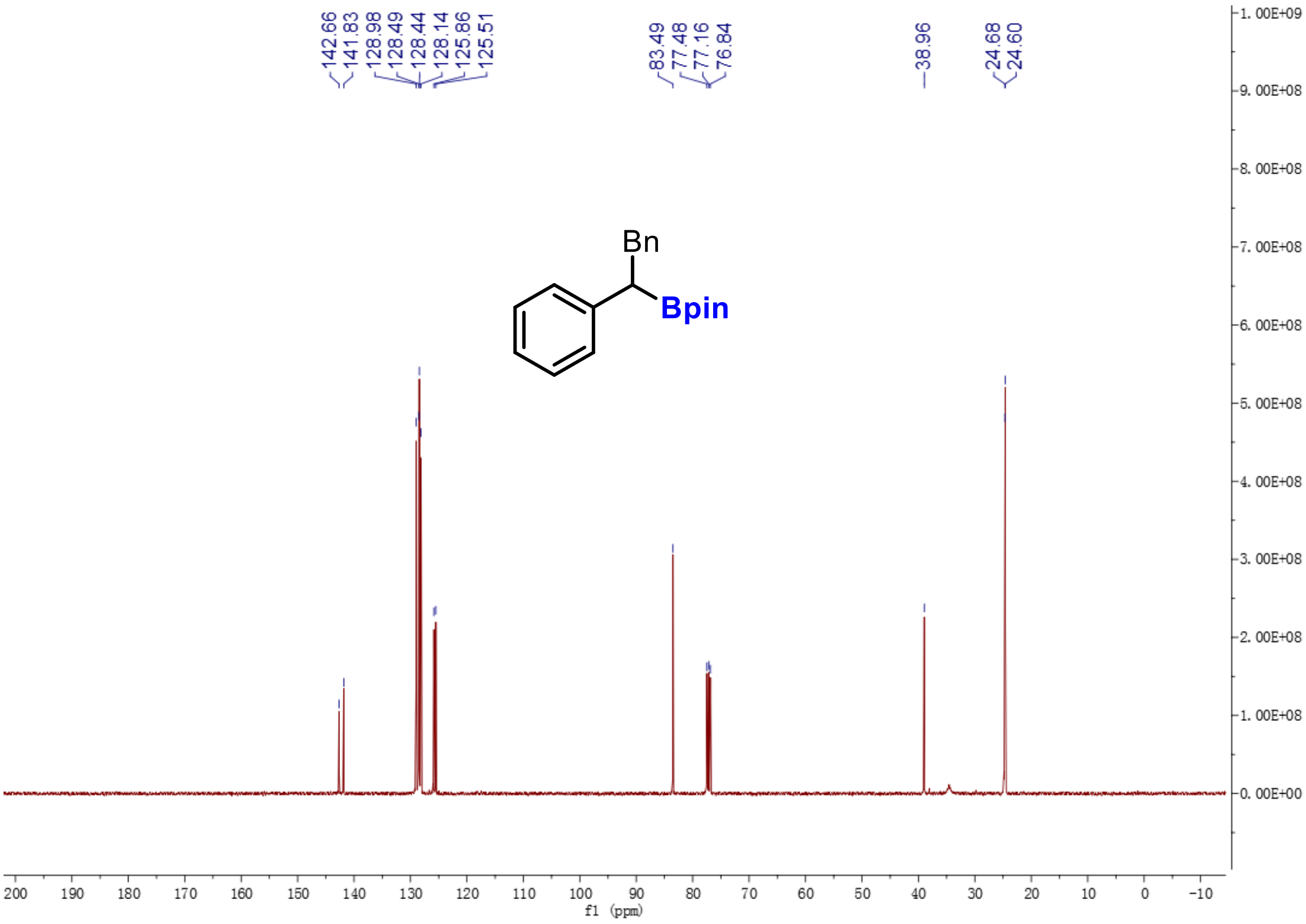

^{1}H NMR spectrum of 9aa (400 MHz, $CDCl_3$)

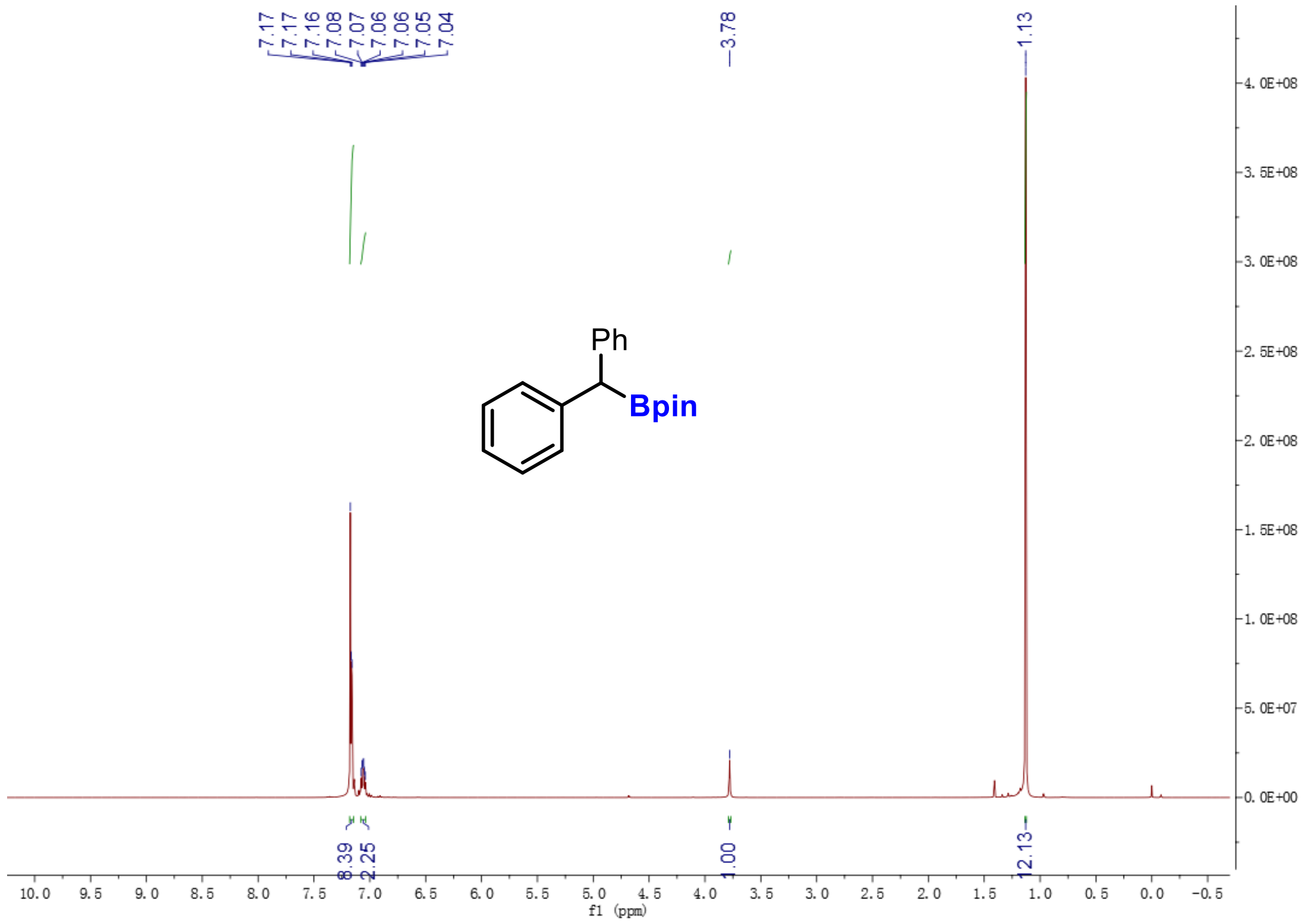

^{13}C NMR spectrum of 9aa (101 MHz, $CDCl_3$)

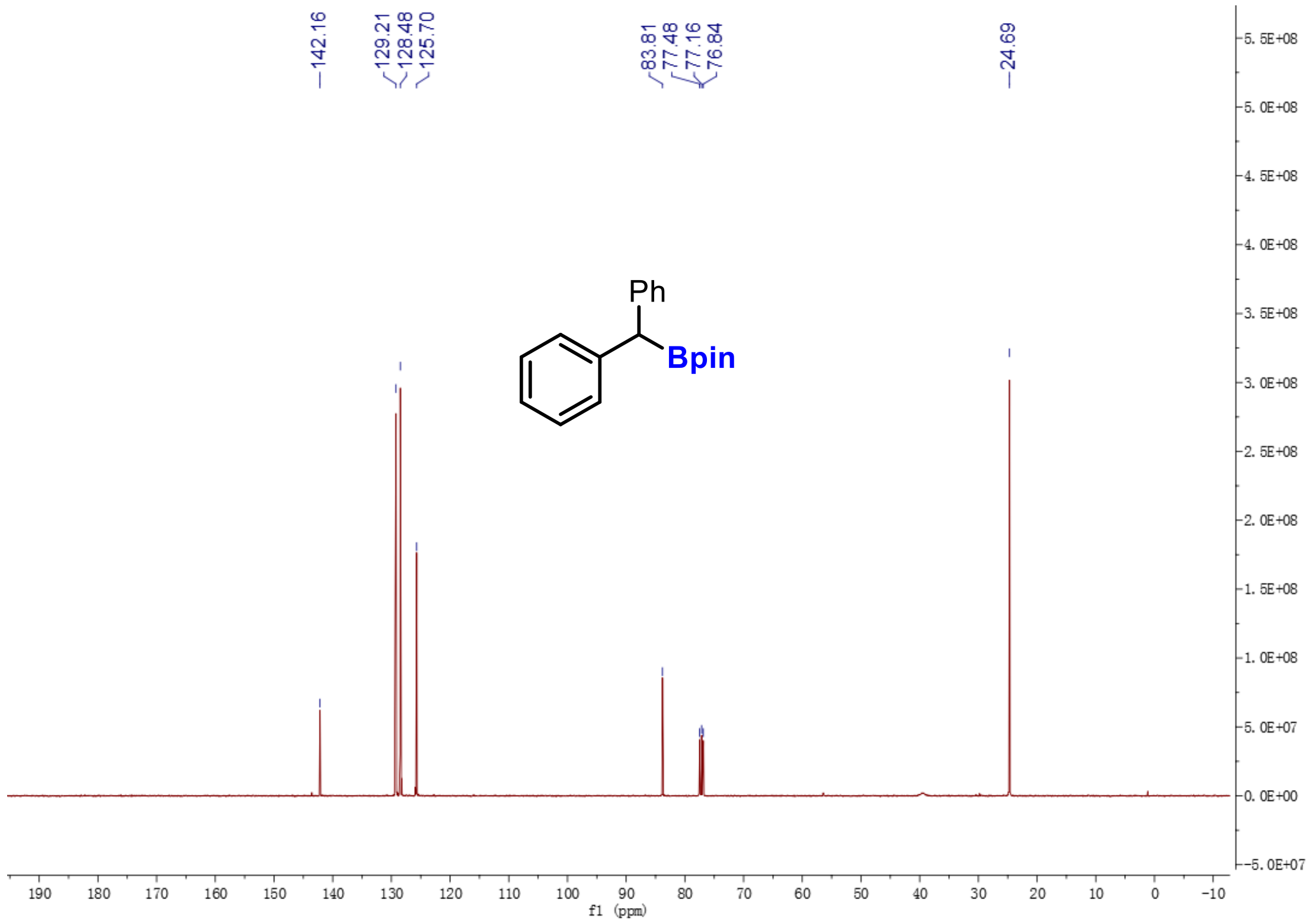

^{1}H NMR spectrum of 9ab (400 MHz, $CDCl_3$)

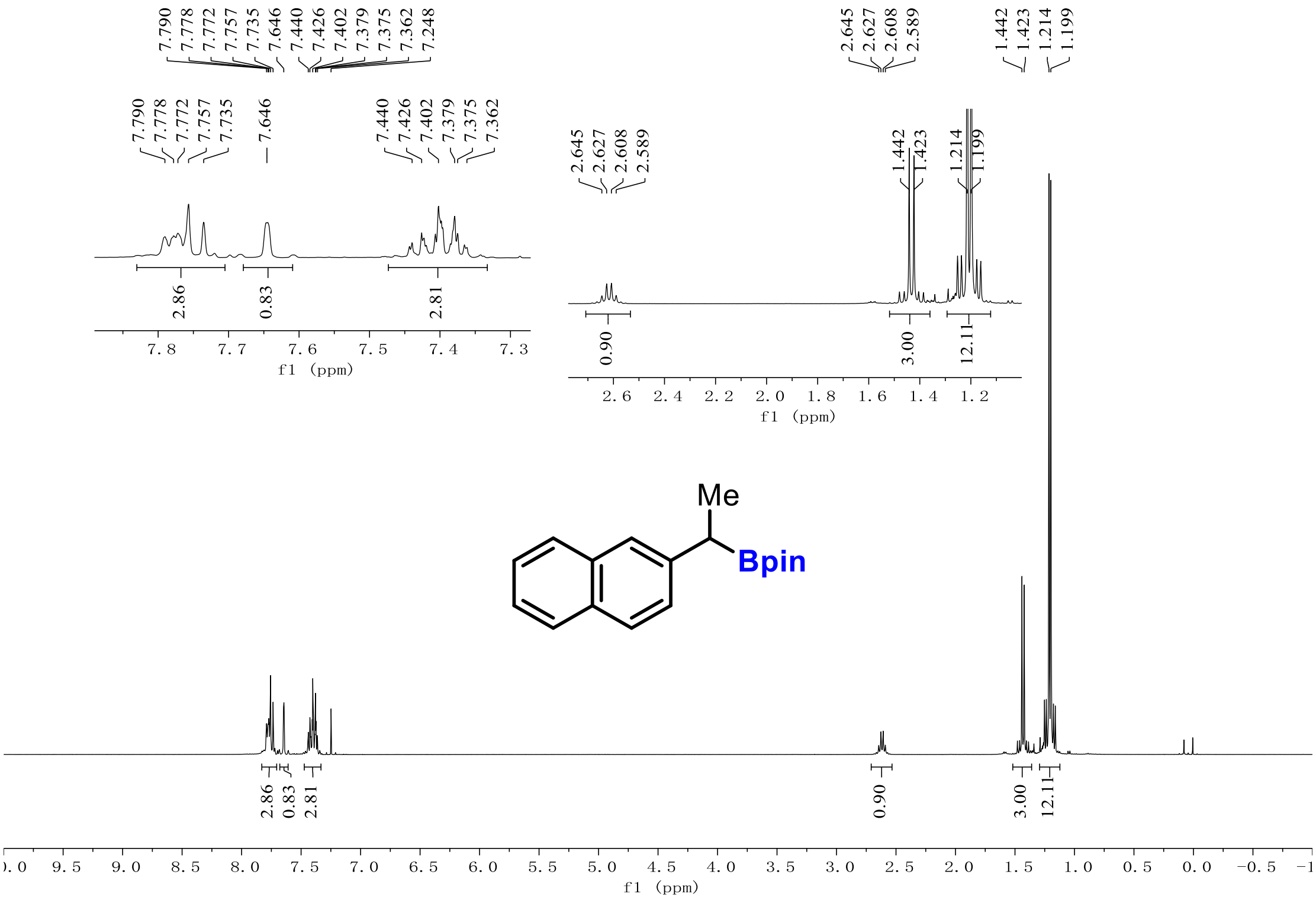

^{13}C NMR spectrum of 9ab (101 MHz, $CDCl_3$)

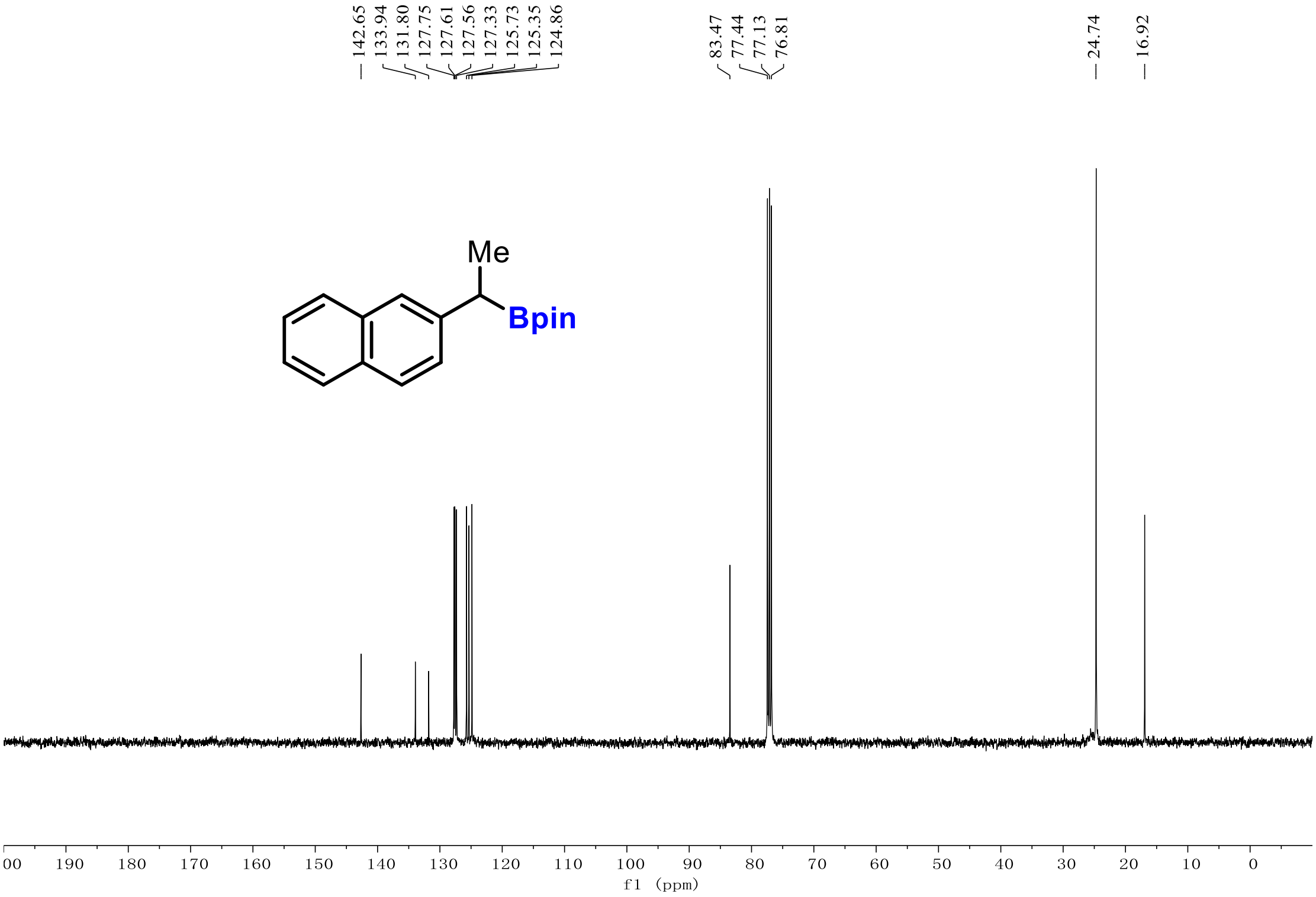

^{11}B NMR spectrum of 9ab (193 MHz, $CDCl_3$)

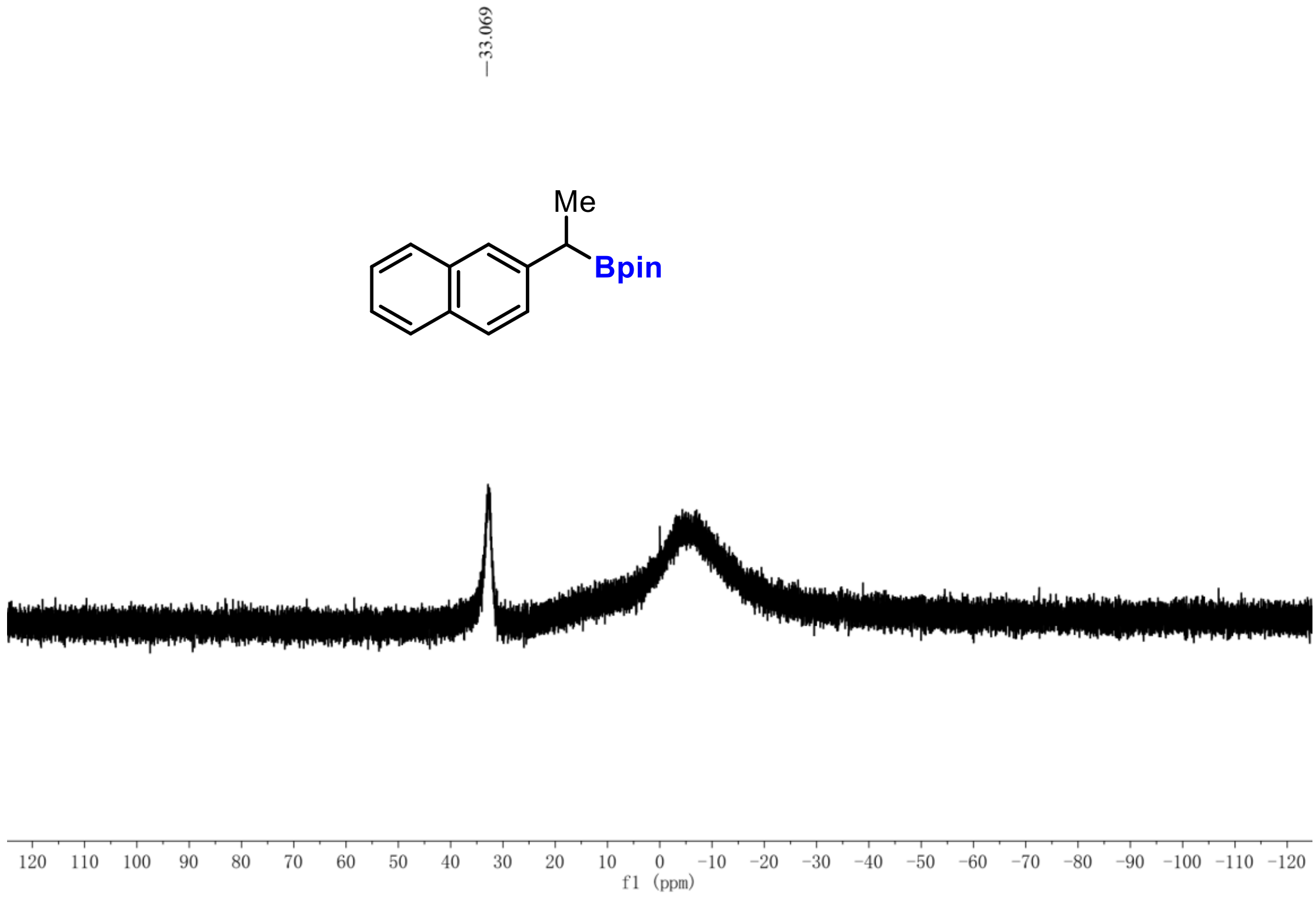

^{1}H NMR spectrum of 9ac (400 MHz, $CDCl_3$)

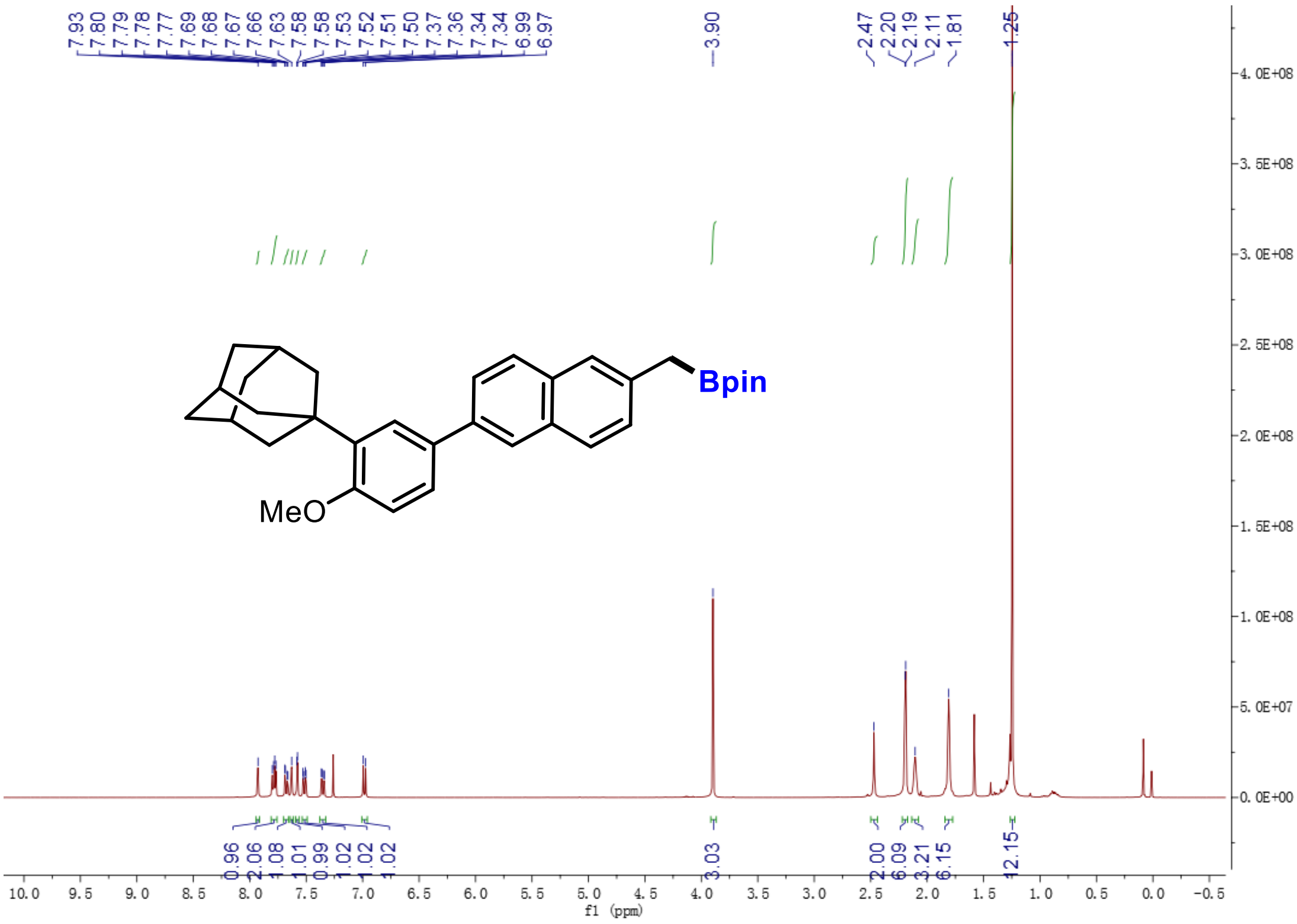

^{13}C NMR spectrum of 9ac (101 MHz, $CDCl_3$)

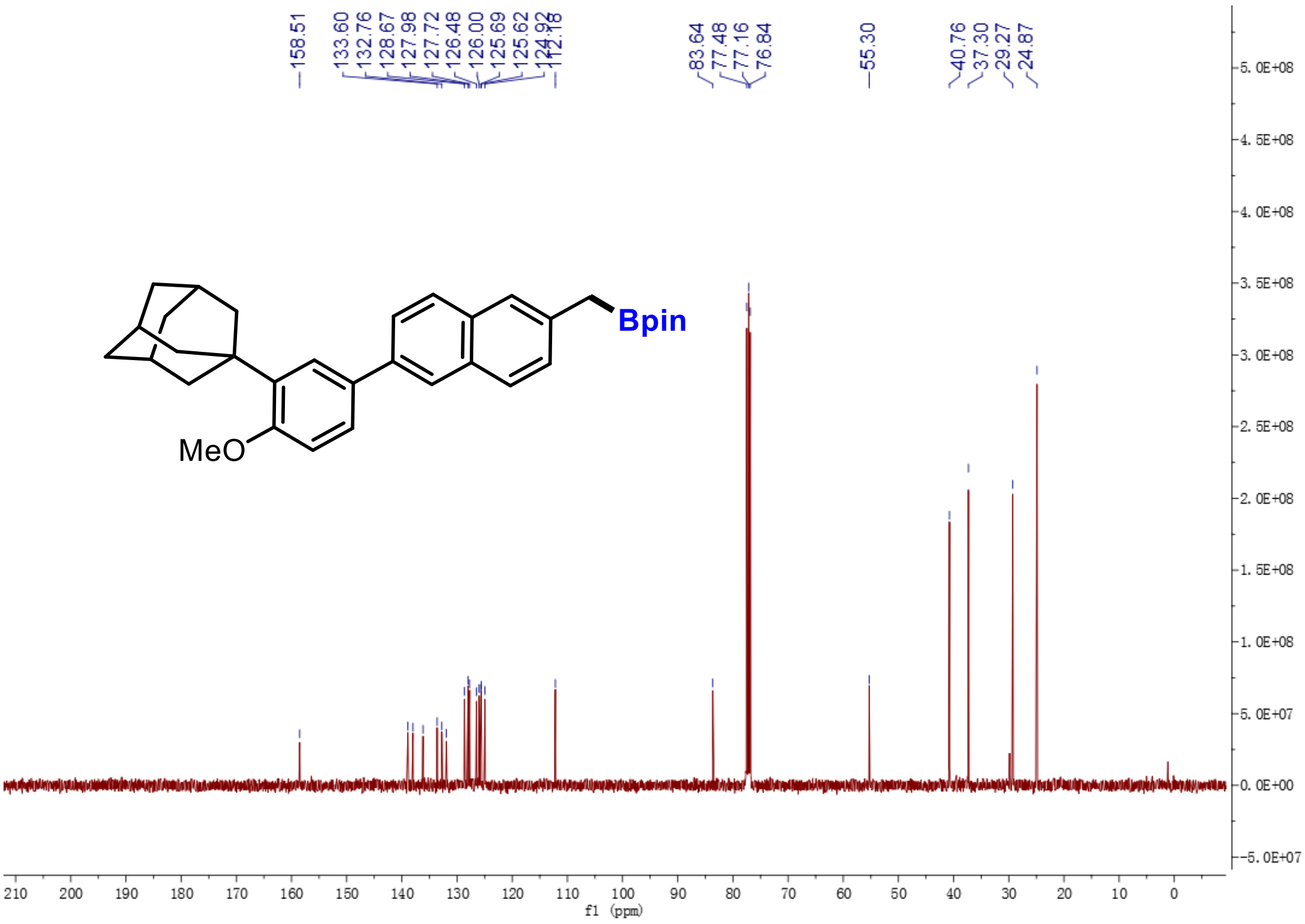

^{1}H NMR spectrum of 9ad (400 MHz, $CDCl_3$)

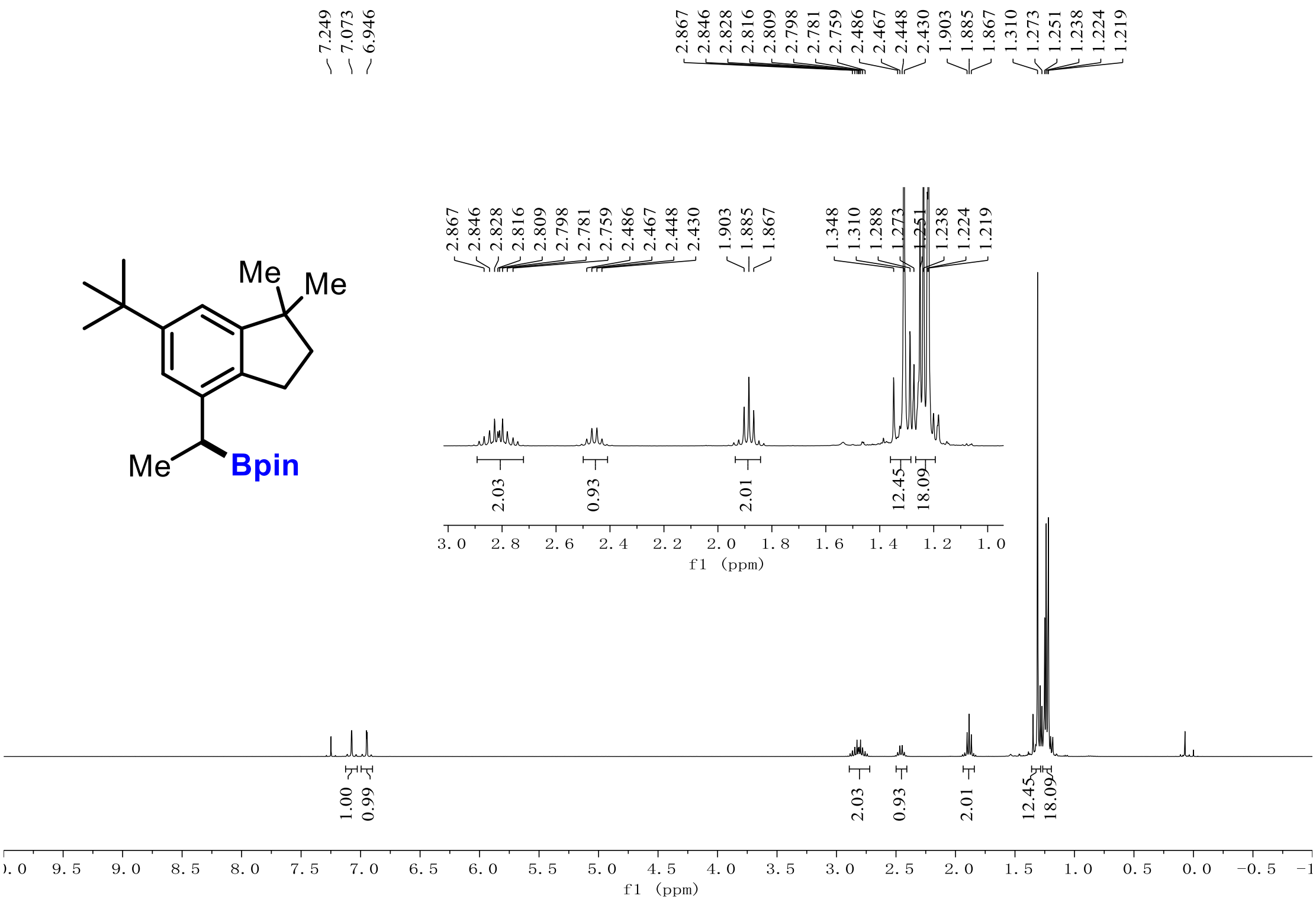

^{13}C NMR spectrum of 9ad (101 MHz, $CDCl_3$)

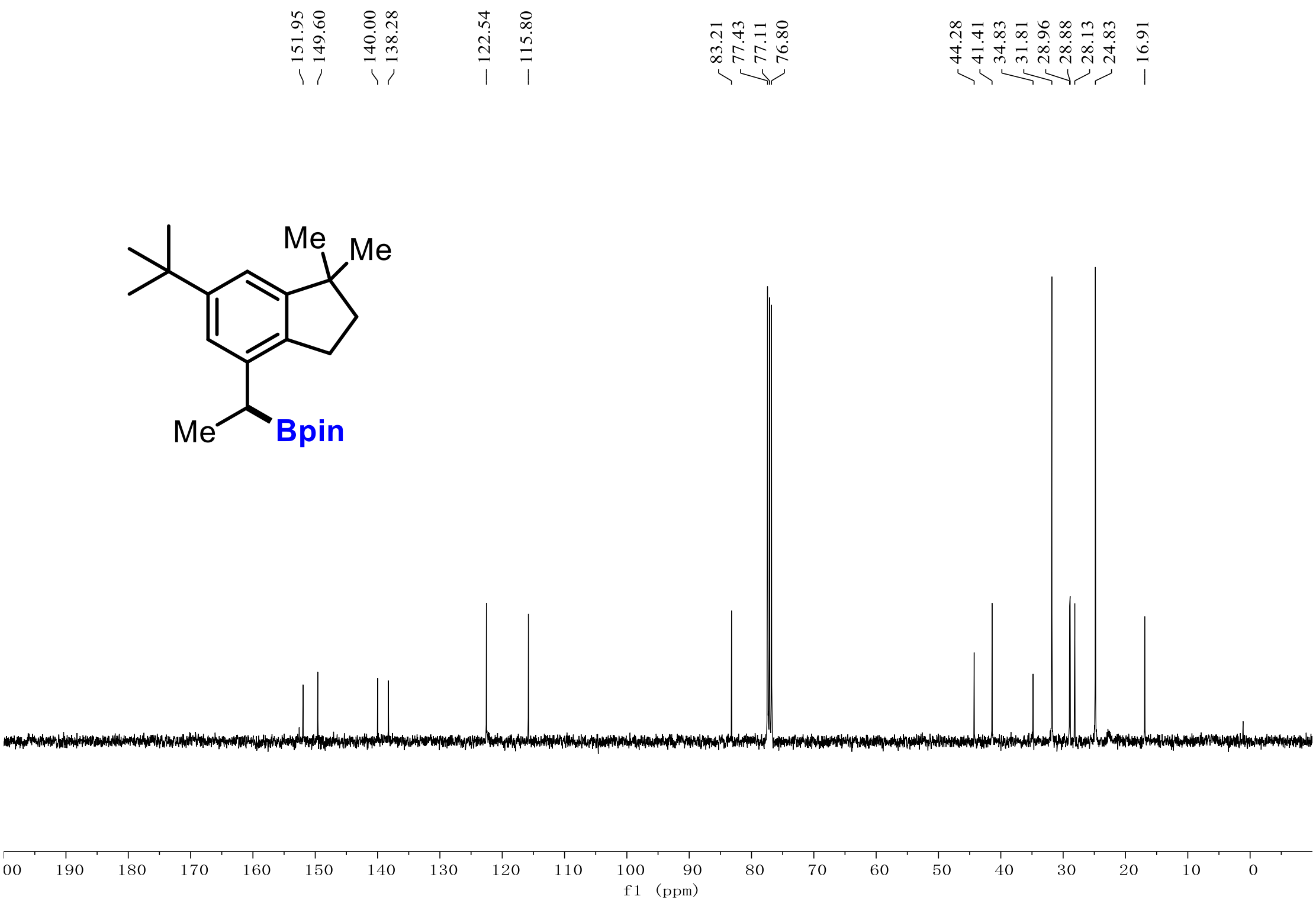

^{11}B NMR spectrum of 9ad (193 MHz, $CDCl_3$)

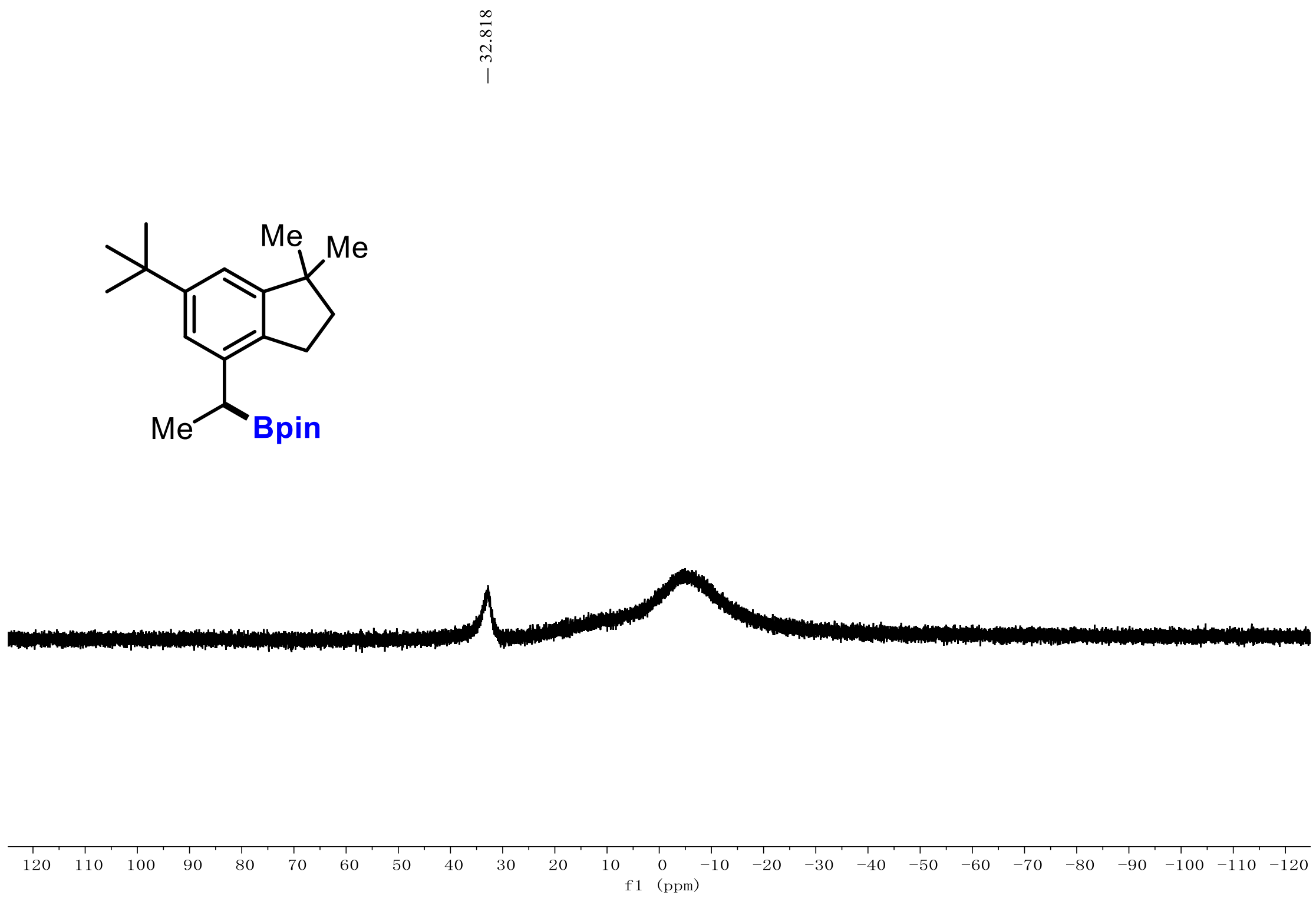

^{1}H NMR spectrum of 9ae (400 MHz, $CDCl_3$)

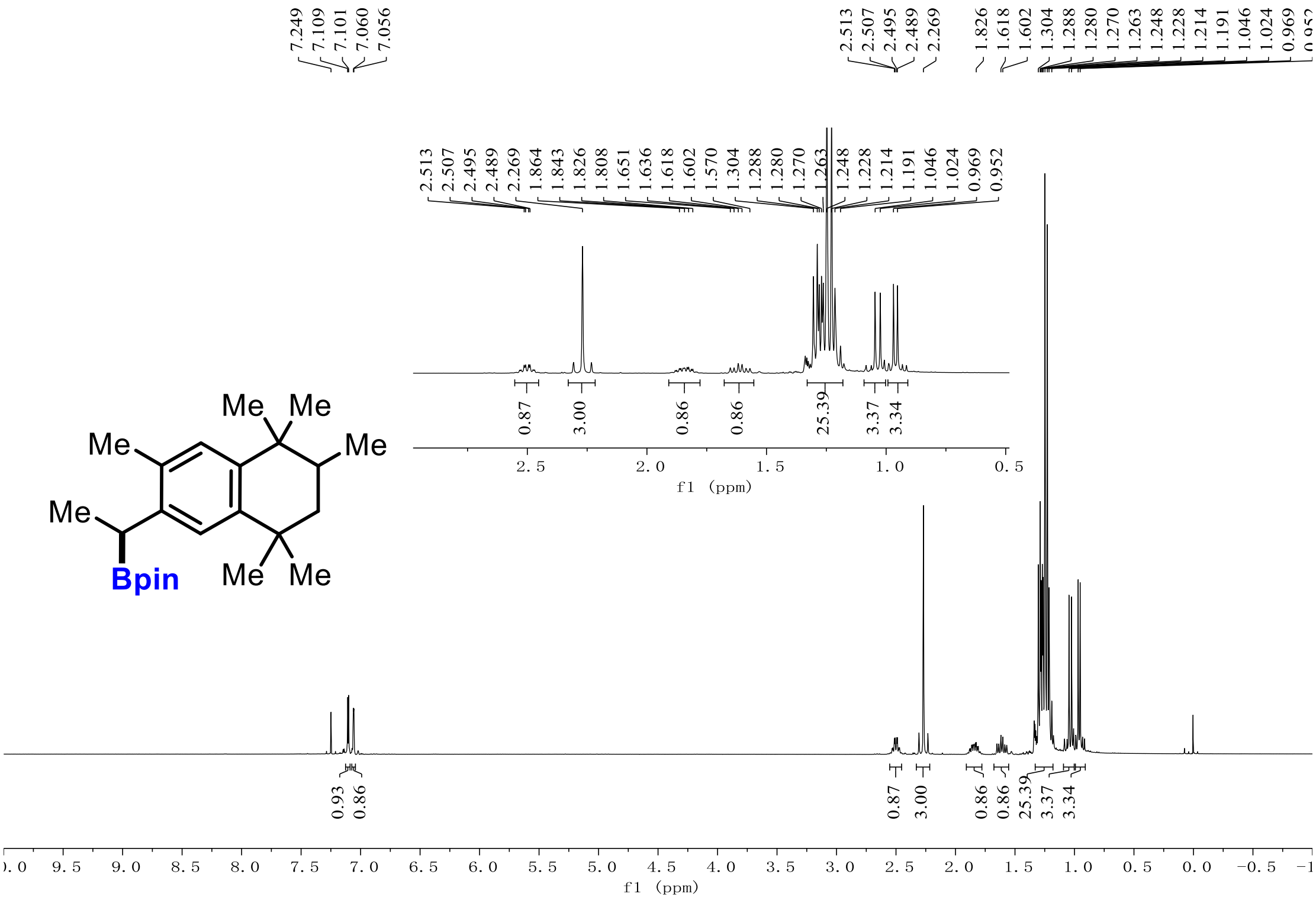

^{13}C NMR spectrum of 9ae (101 MHz, $CDCl_3$)

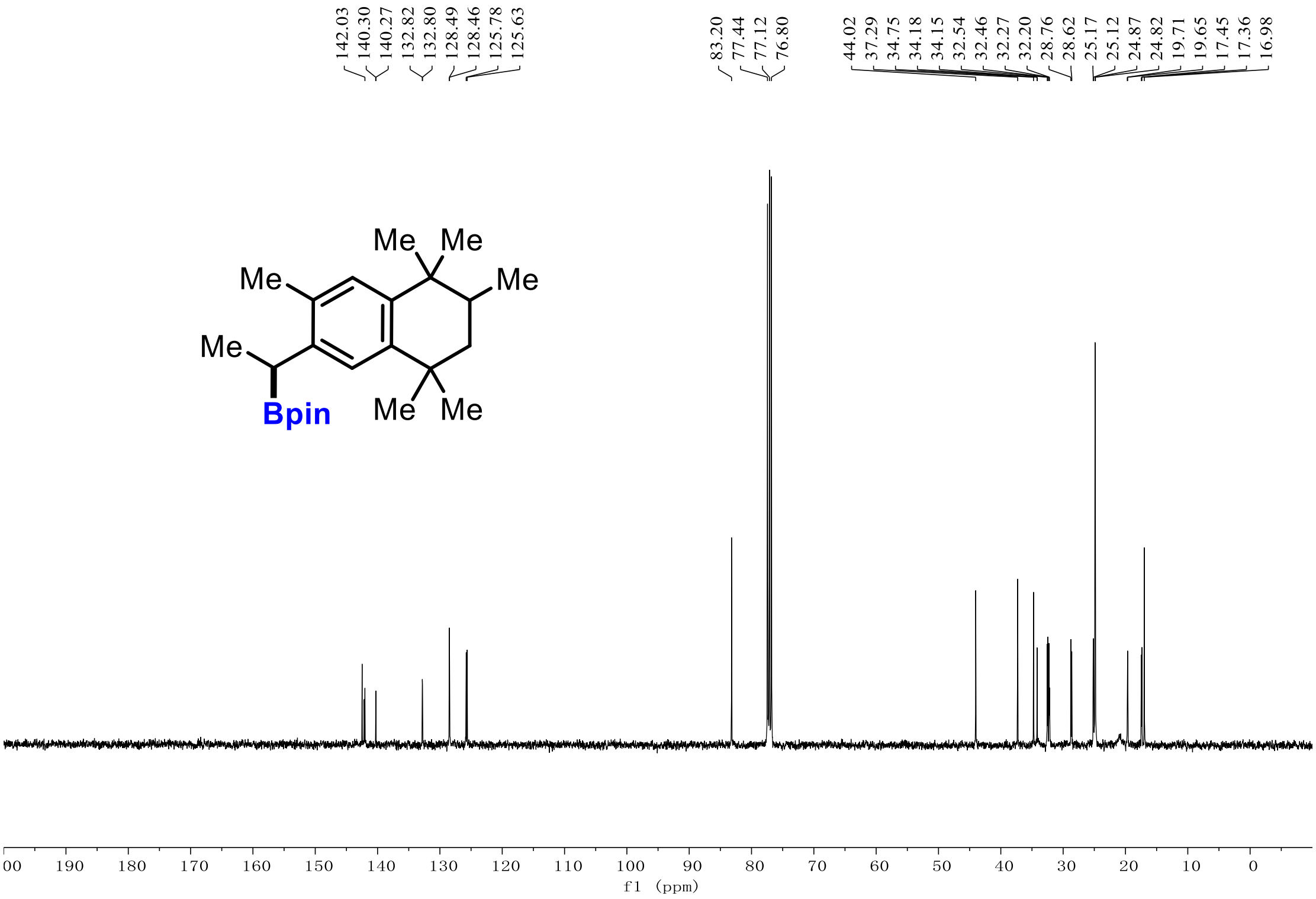

^{11}B NMR spectrum of 9ae (193 MHz, $CDCl_3$)

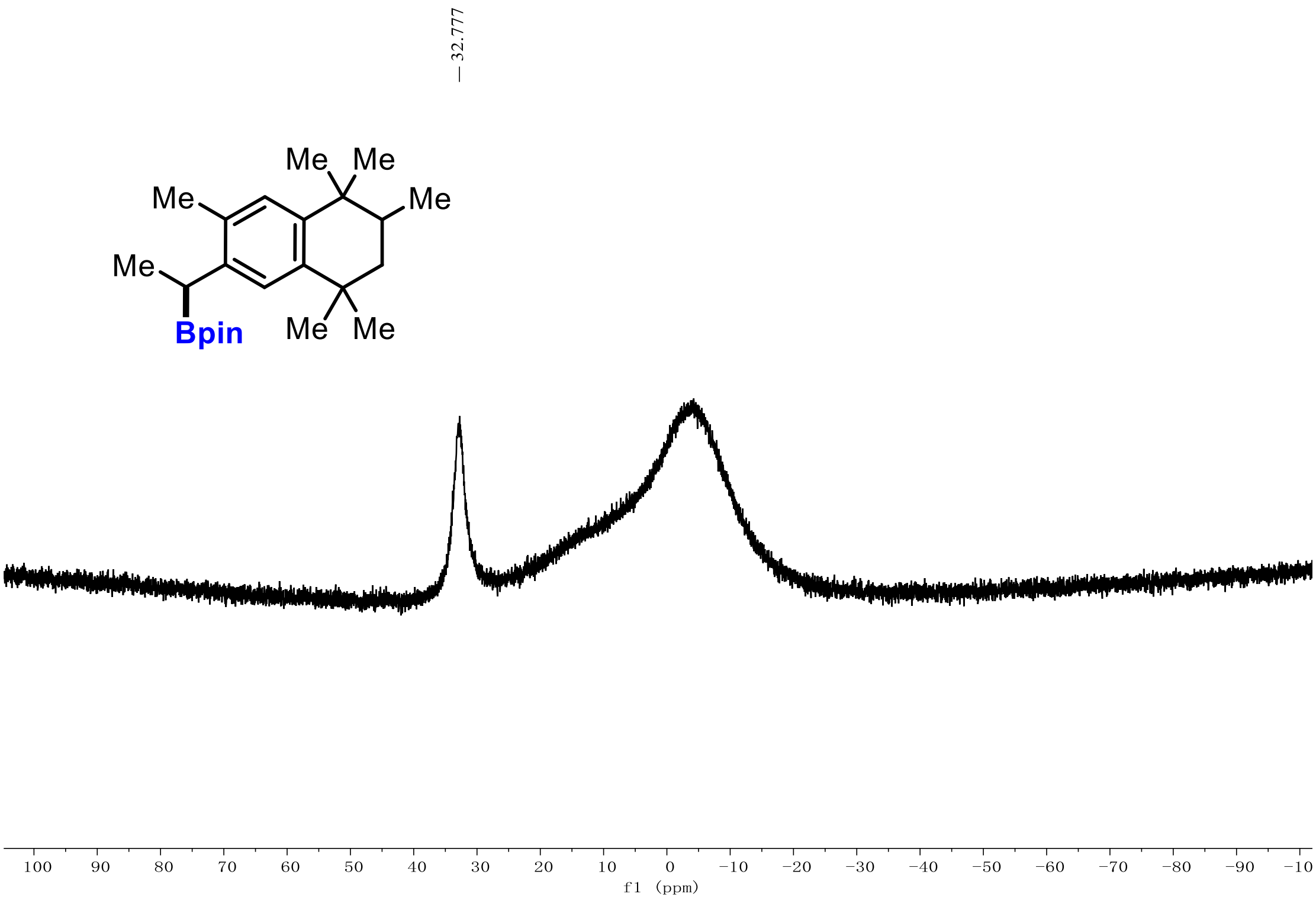

^{1}H NMR spectrum of 9af (400 MHz, $CDCl_3$)

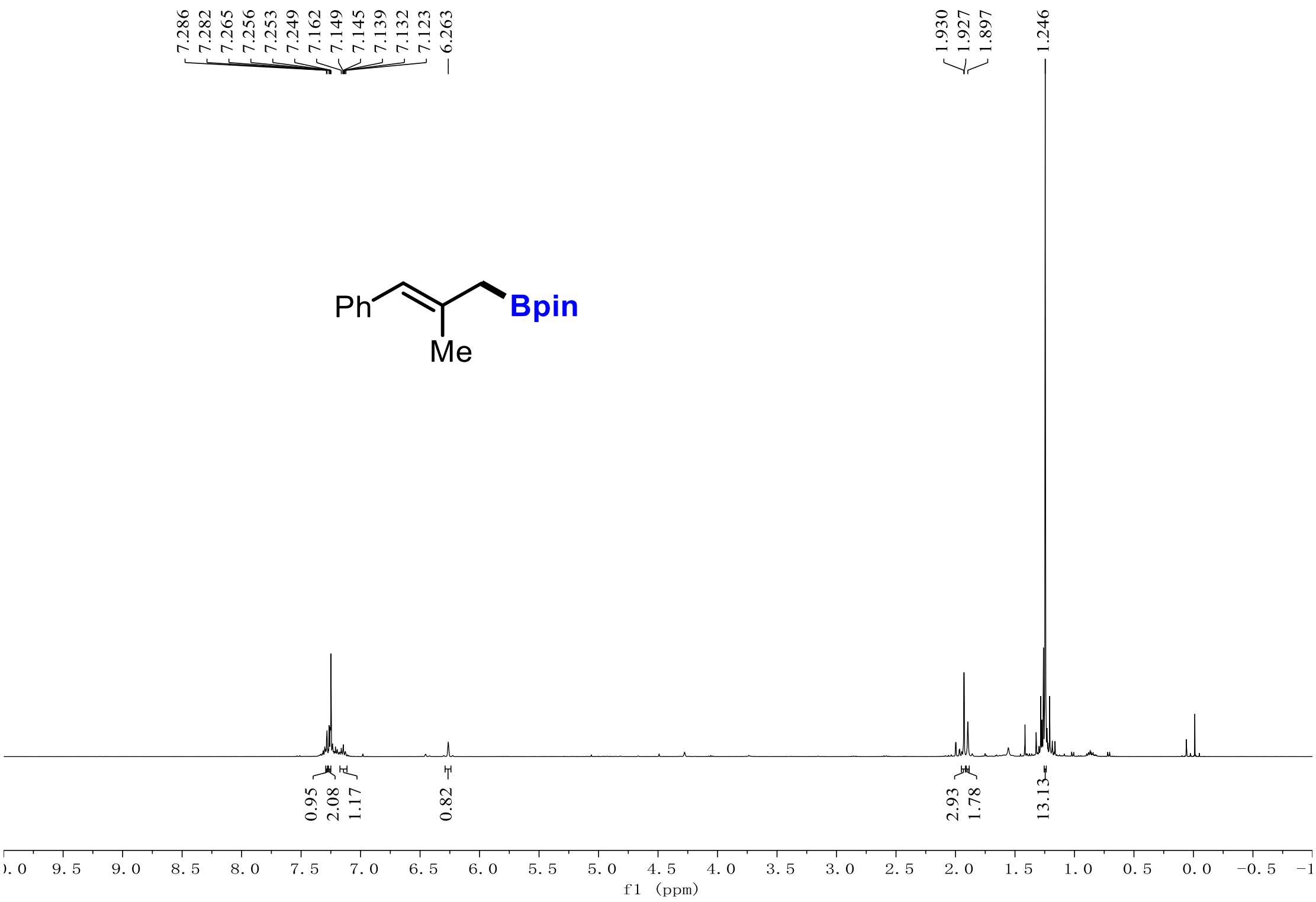

^{13}C NMR spectrum of 9af (101 MHz, $CDCl_3$)

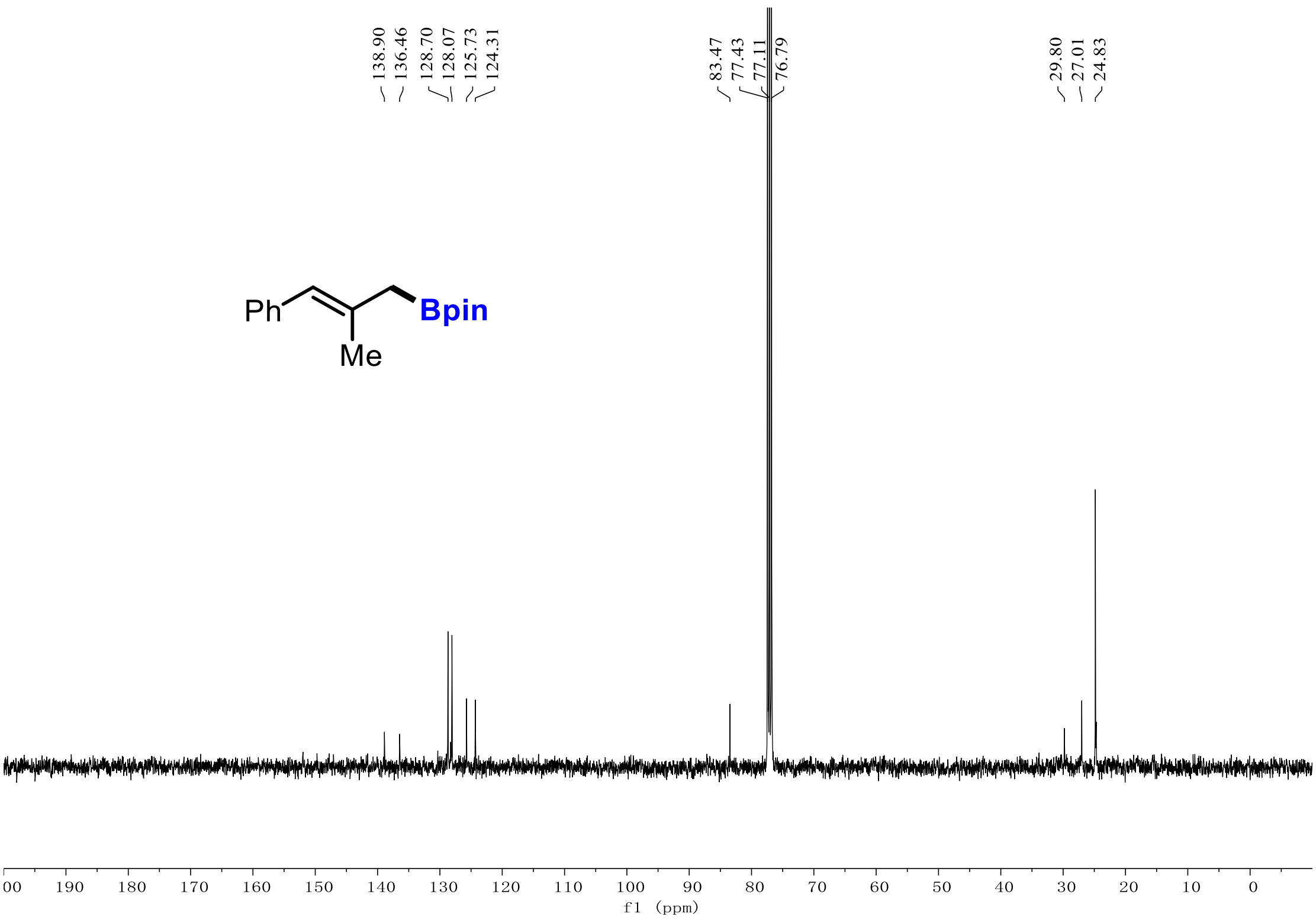

^{11}B NMR spectrum of 9af (193 MHz, $CDCl_3$)

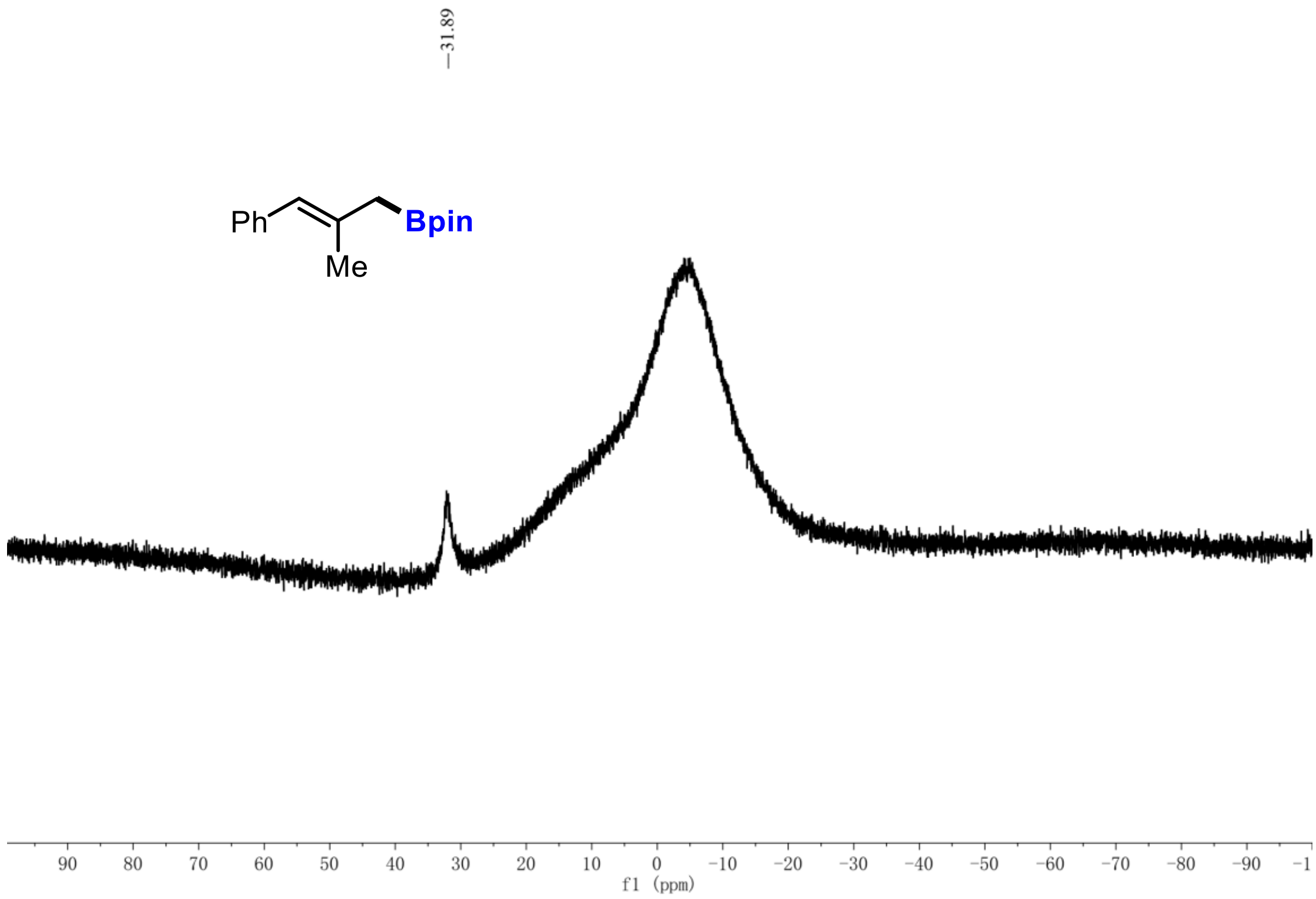

31.89
Ph
Bpin
Me
90 80 70 60 50 40 30 20 10 0 −10 −20 −30 −40 −50 −60 −70 −80 −90 −1
f1 (ppm)

^{1}H NMR spectrum of 9ag (400 MHz, $CDCl_3$)

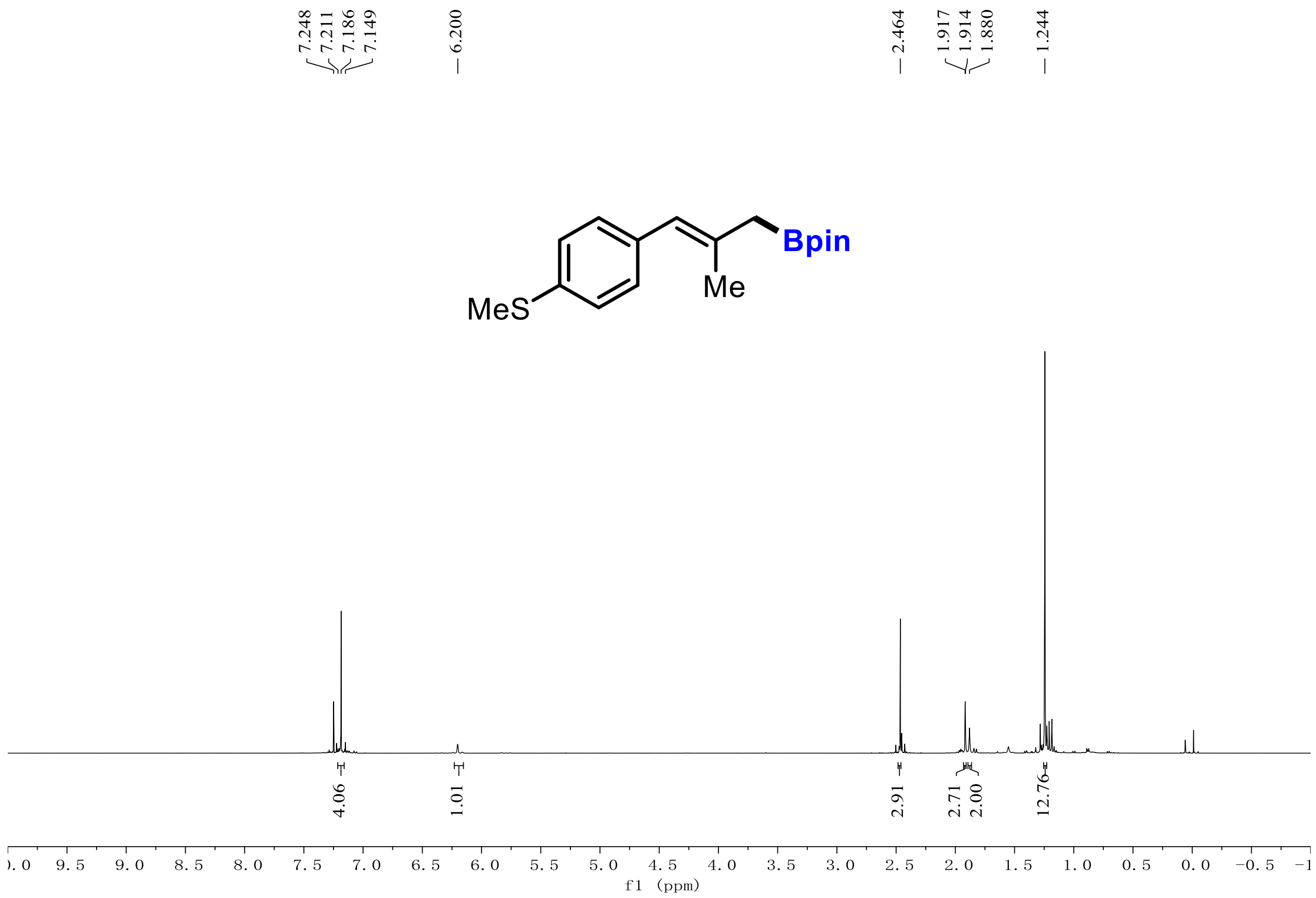

^{13}C NMR spectrum of 9ag (101 MHz, $CDCl_3$)

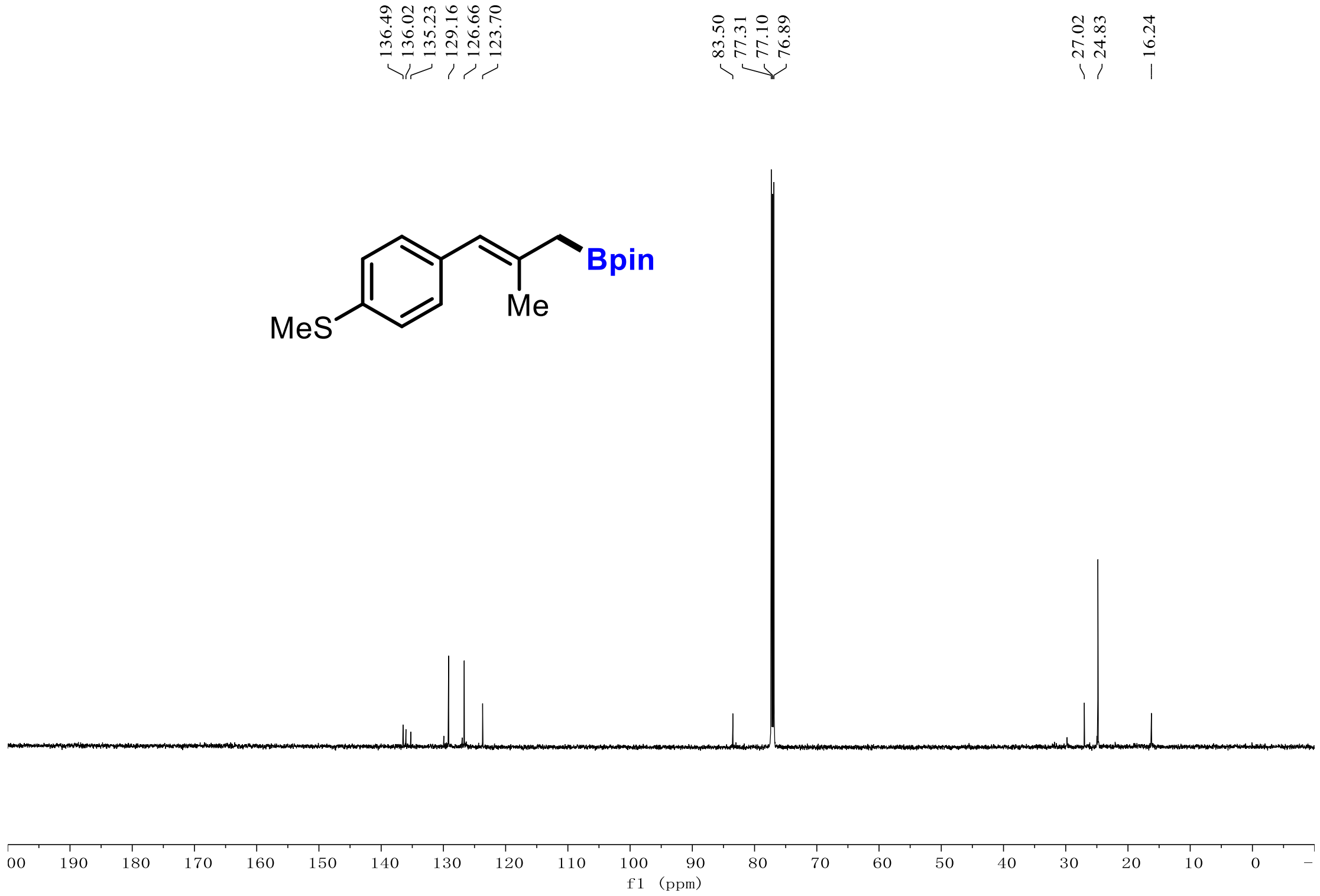

^{11}B NMR spectrum of 9ag (193 MHz, $CDCl_3$)

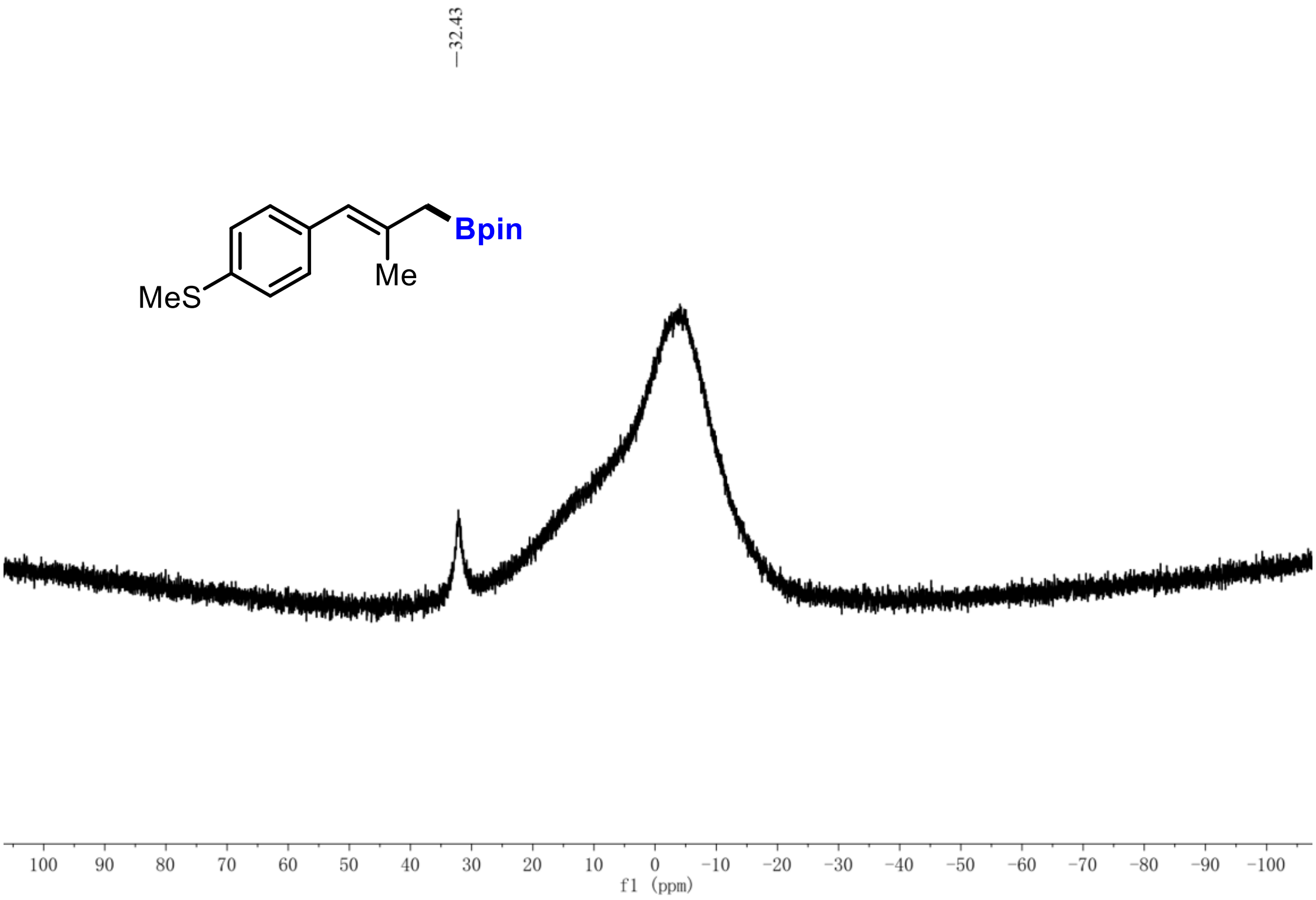

^{1}H NMR spectrum of 9ah (400 MHz, $CDCl_3$)

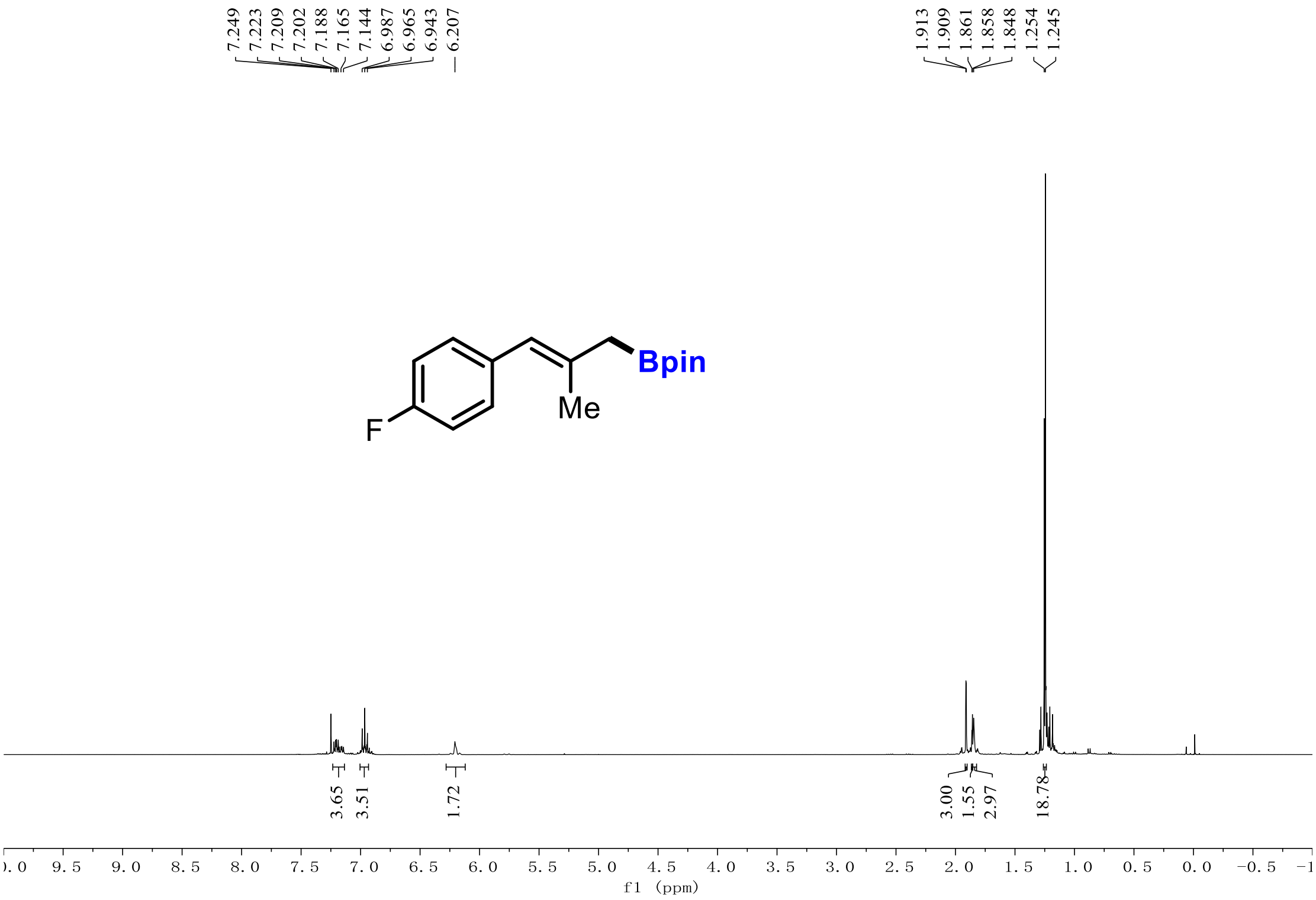

^{13}C NMR spectrum of 9ah (101 MHz, $CDCl_3$)

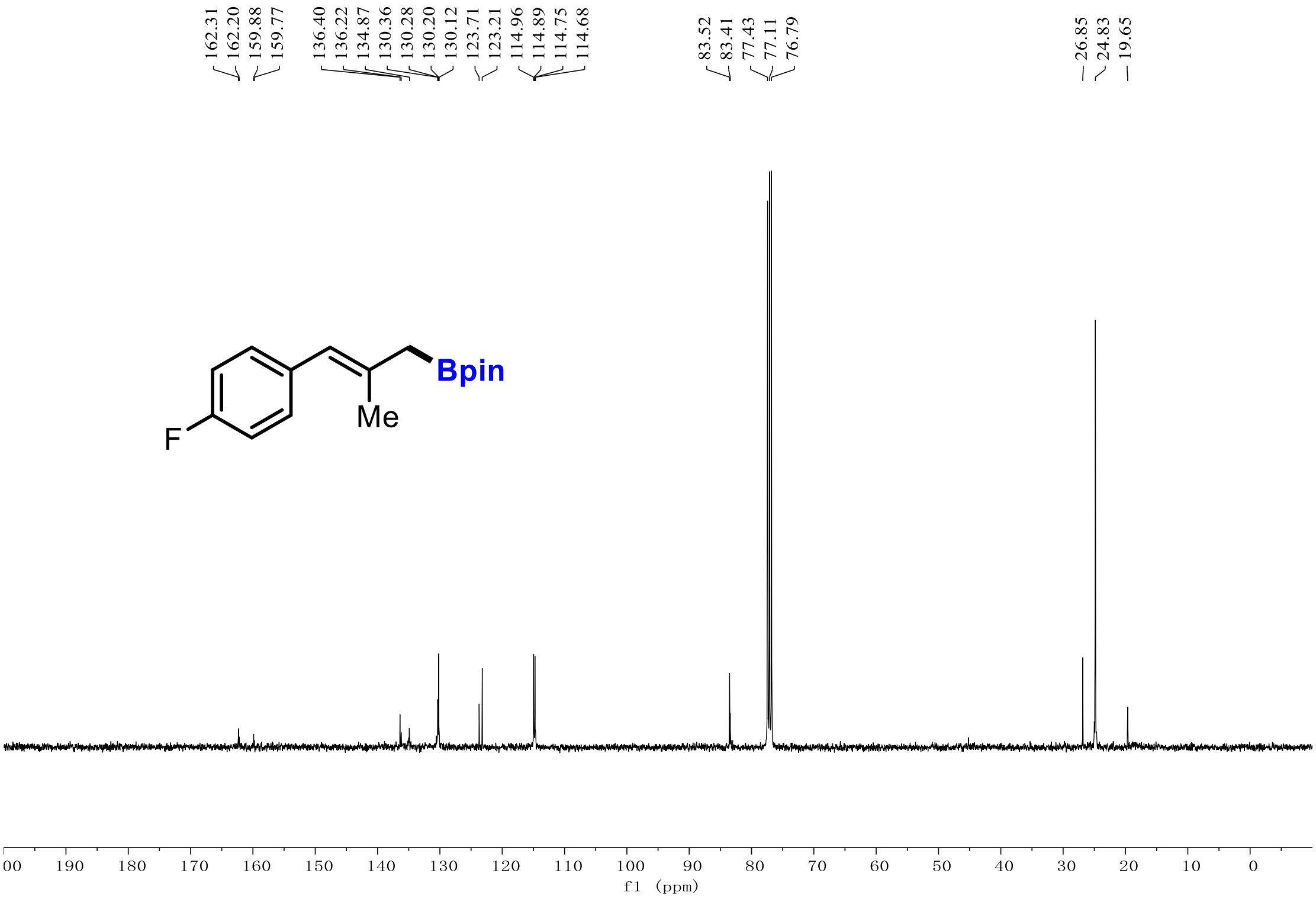

^{11}B NMR spectrum of 9ah (193 MHz, $CDCl_3$)

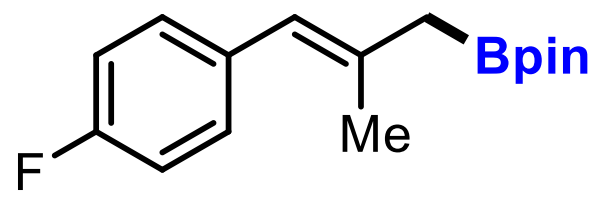

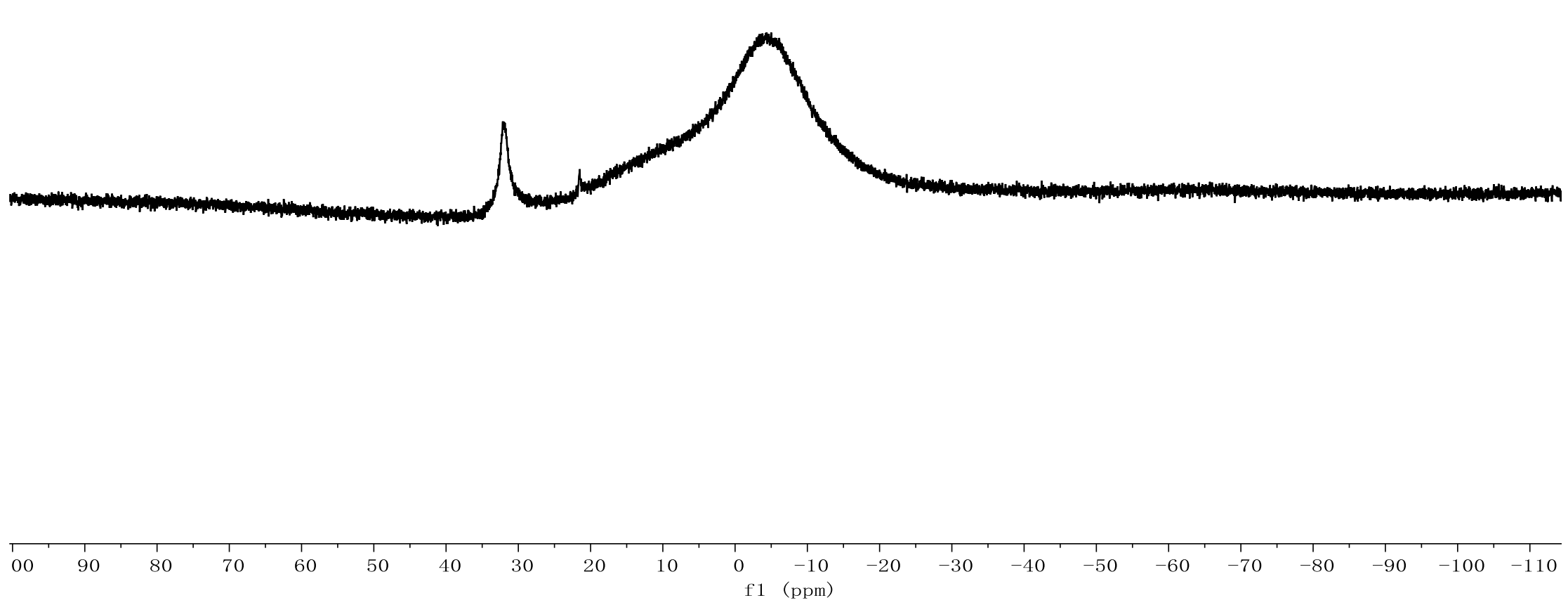

^{1}H NMR spectrum of 9ai (400 MHz, $CDCl_3$)

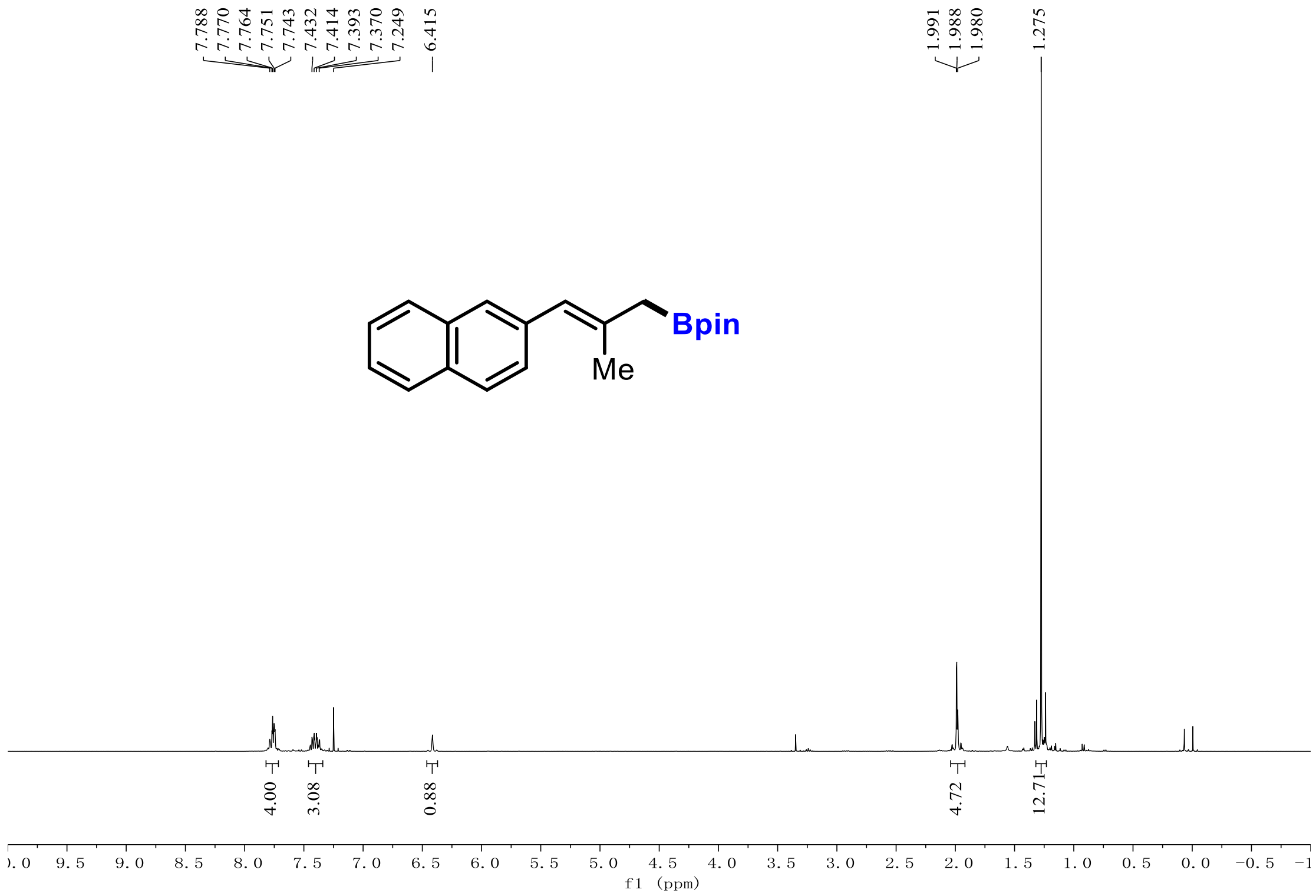

^{13}C NMR spectrum of 9ai (101 MHz, $CDCl_3$)

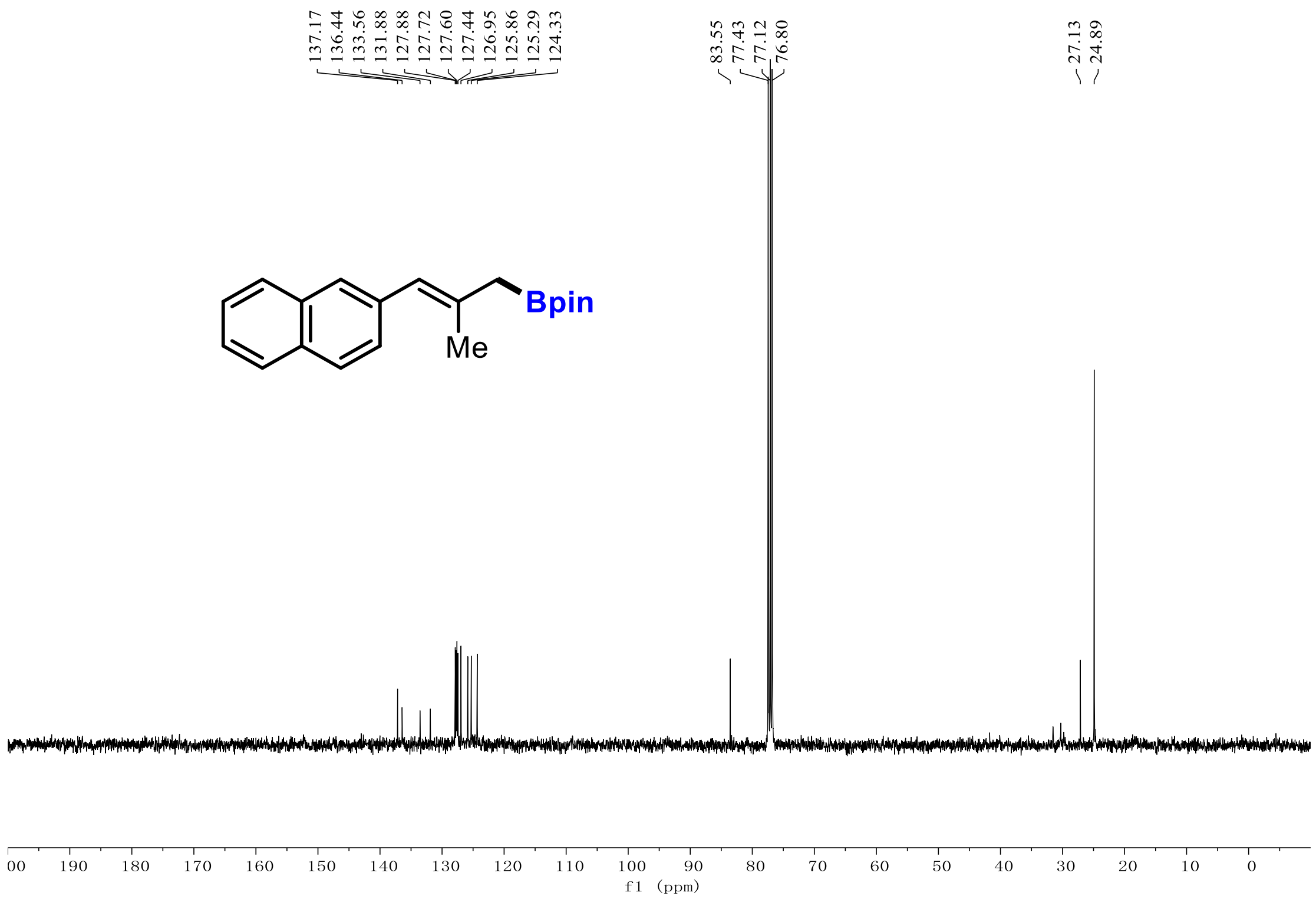

^{11}B NMR spectrum of 9ai (193 MHz, $CDCl_3$)

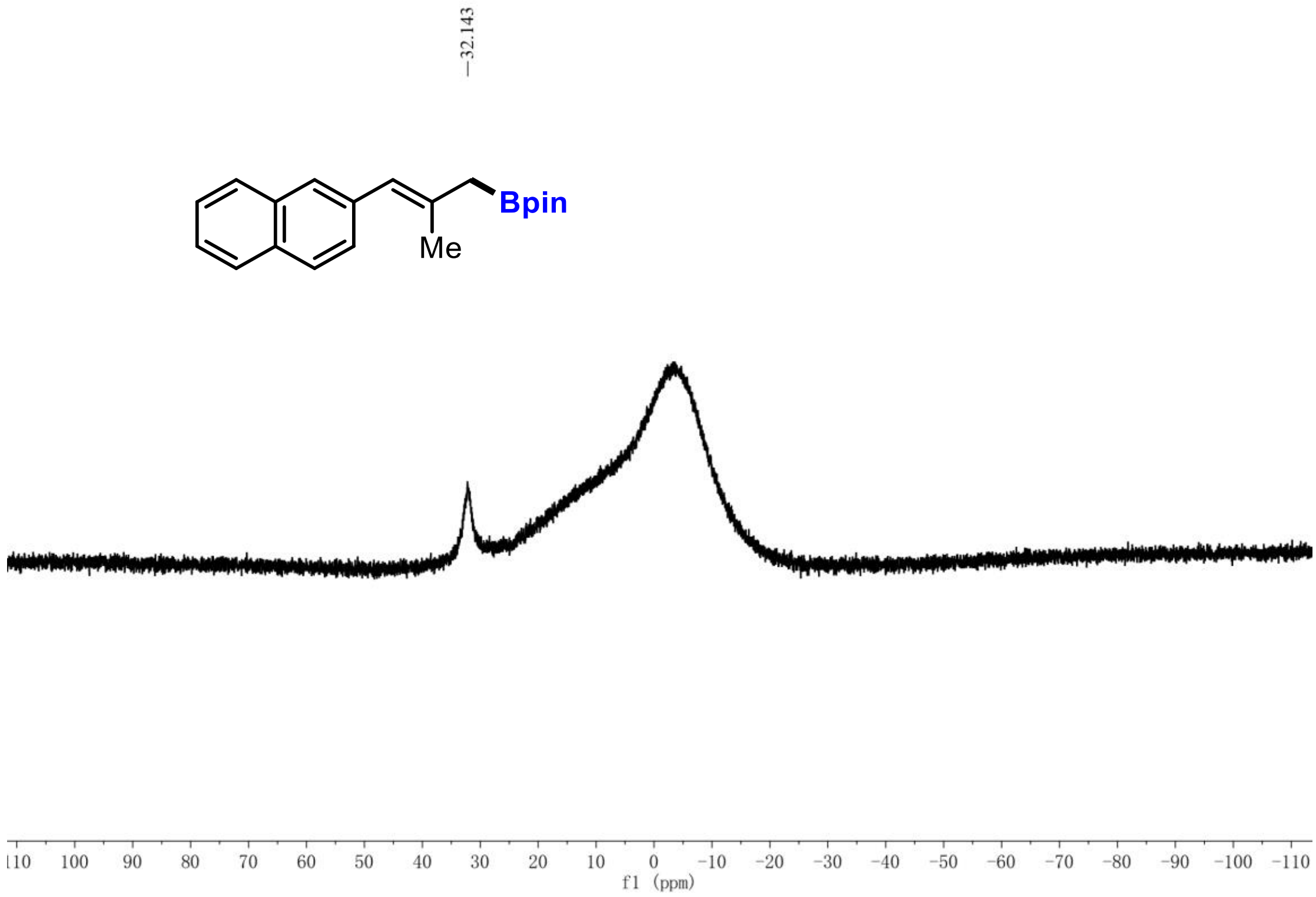

^{1}H NMR spectrum of 9aj (400 MHz, $CDCl_3$)

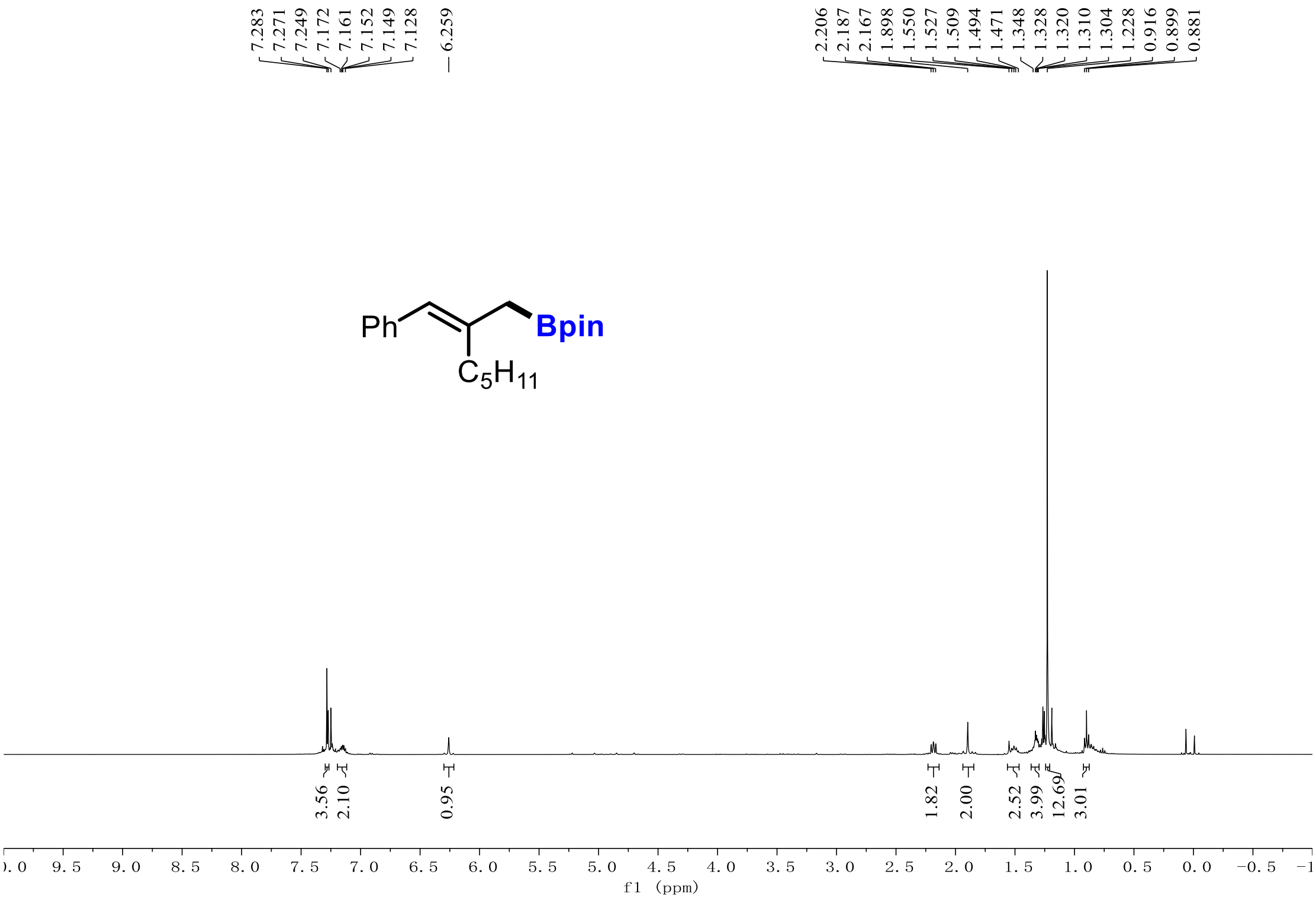

^{13}C NMR spectrum of 9aj (101 MHz, $CDCl_3$)

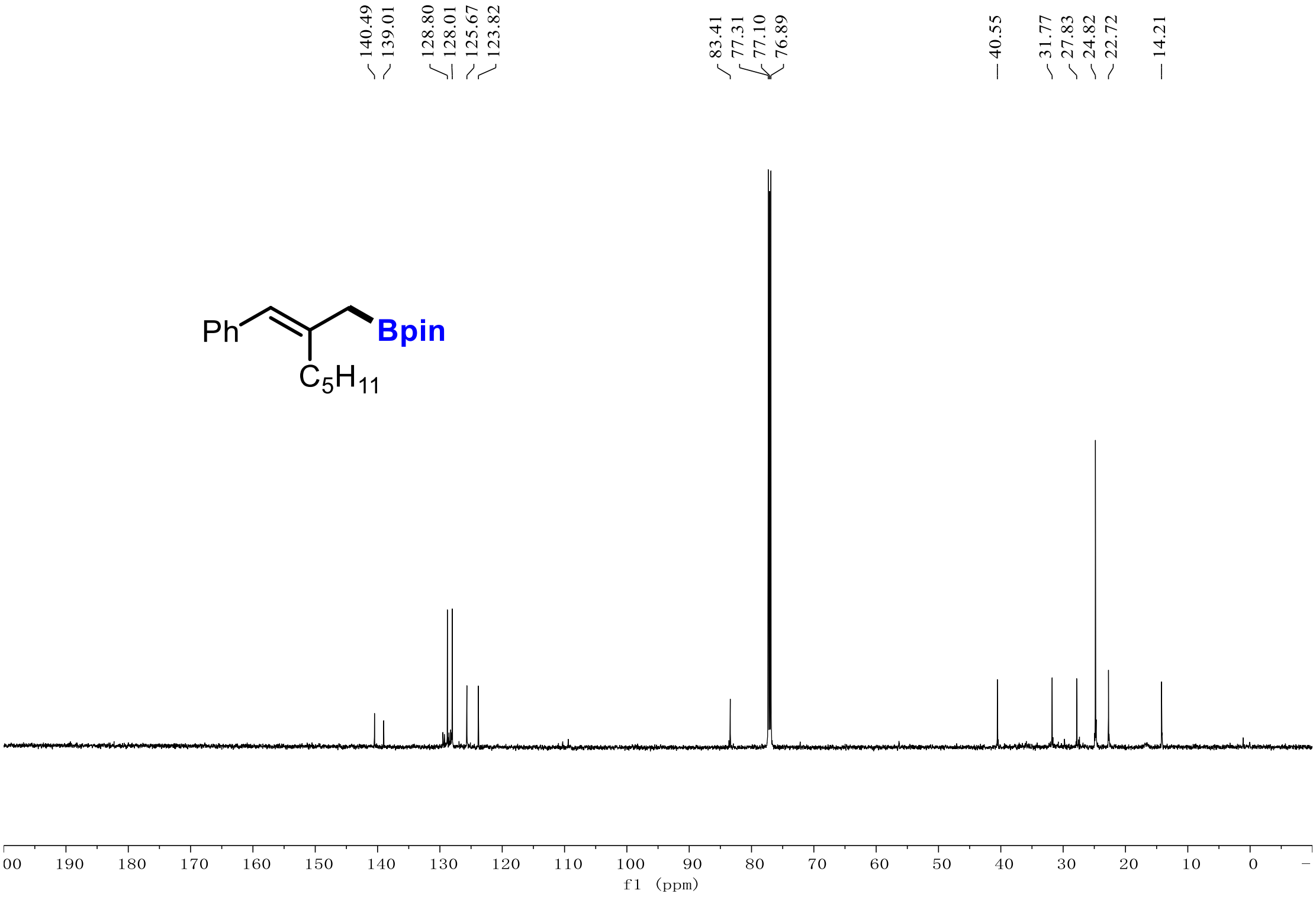

^{11}B NMR spectrum of 9aj (193 MHz, $CDCl_3$)

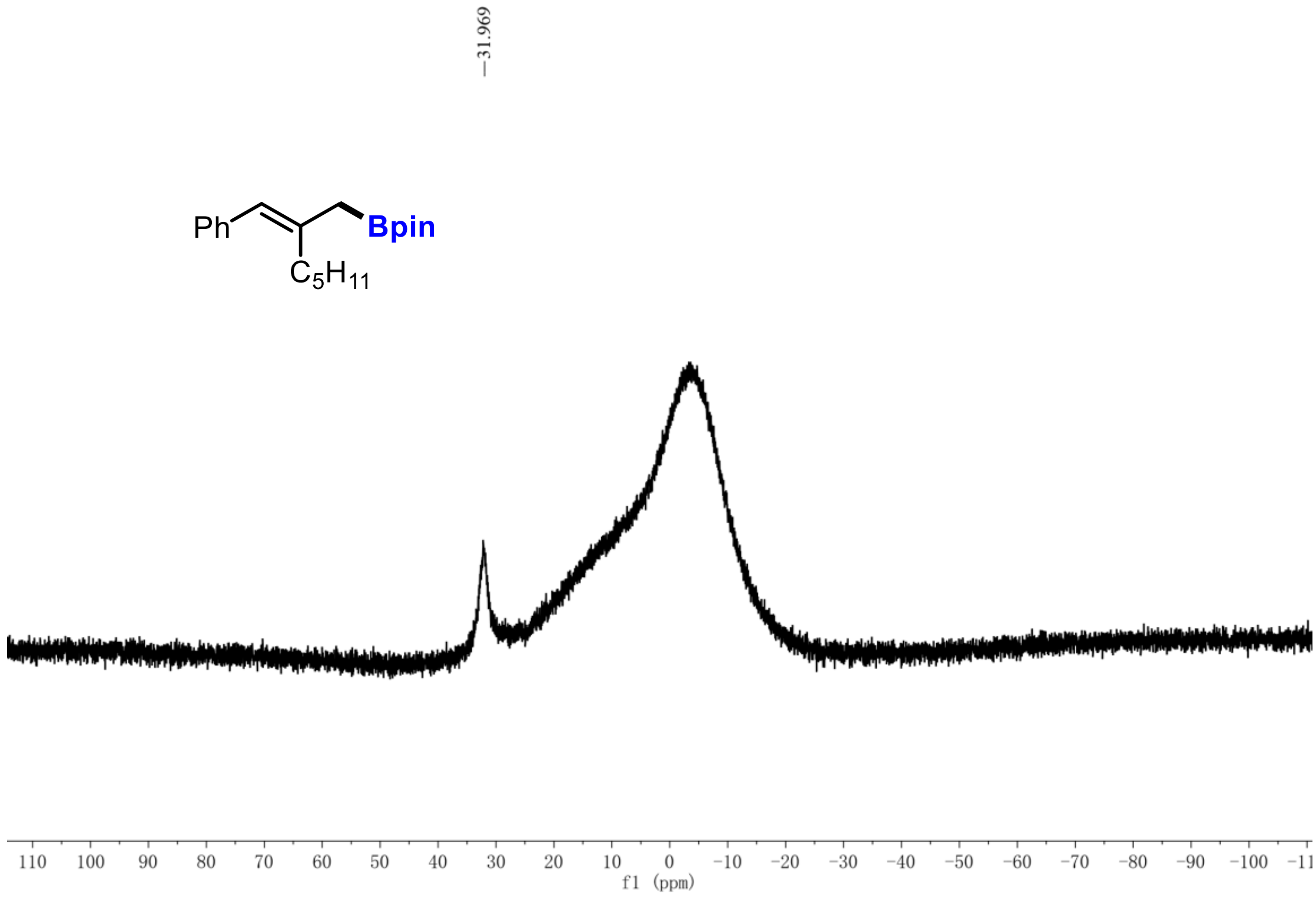

^{1}H NMR spectrum of 9ak (400 MHz, $CDCl_3$)

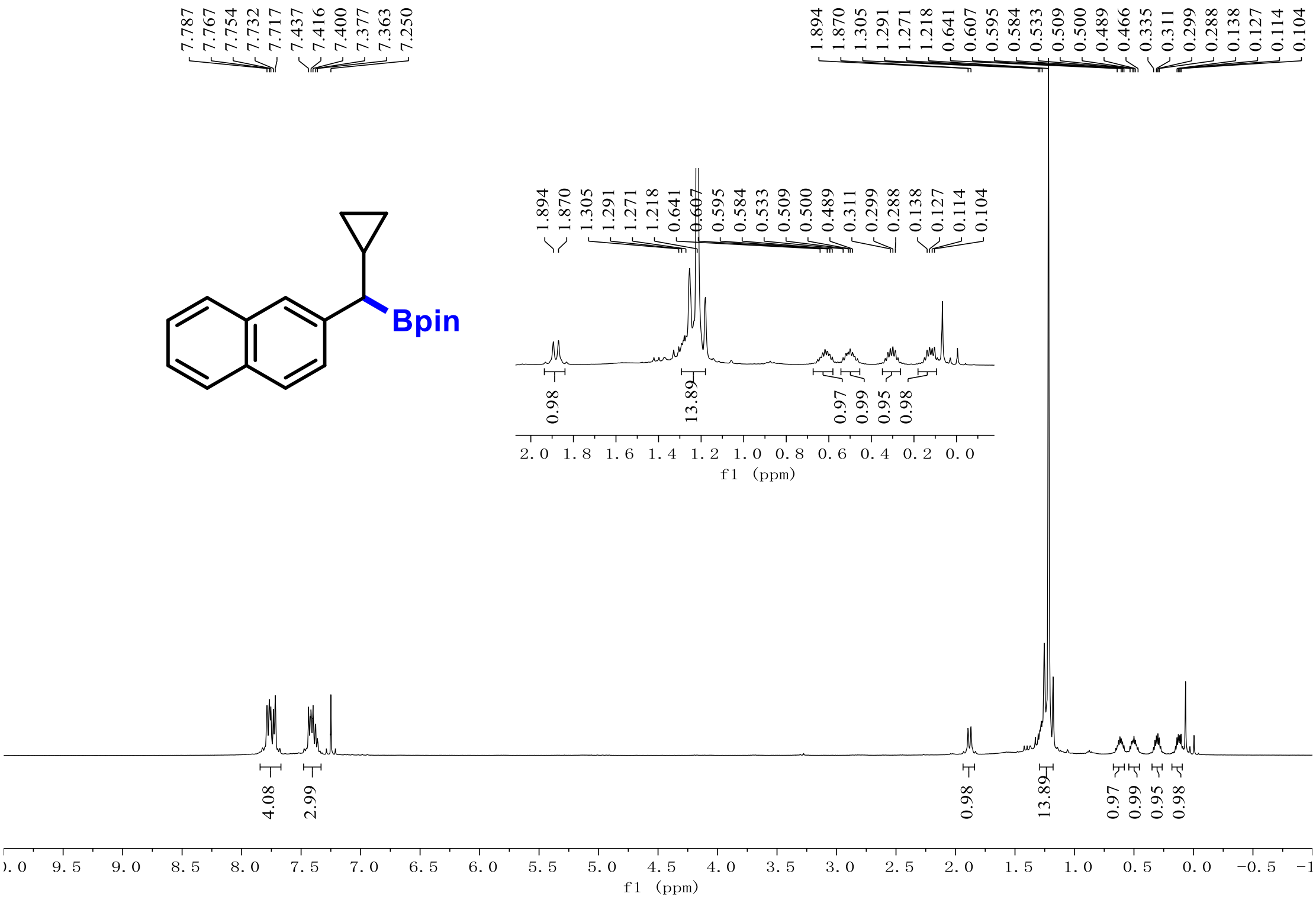

^{13}C NMR spectrum of 9ak (101 MHz, $CDCl_3$)

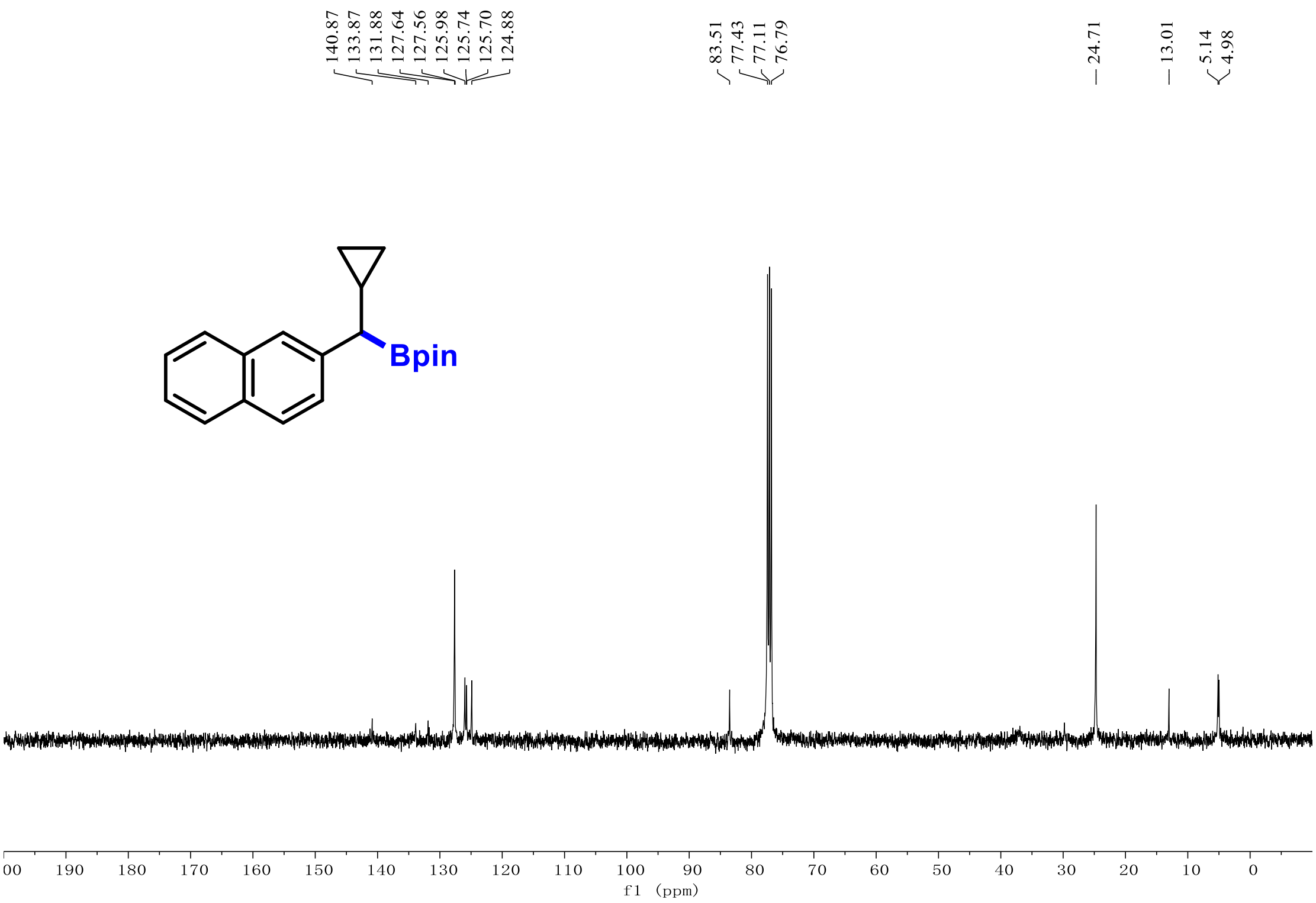

^{11}B NMR spectrum of 9ak (193 MHz, $CDCl_3$)

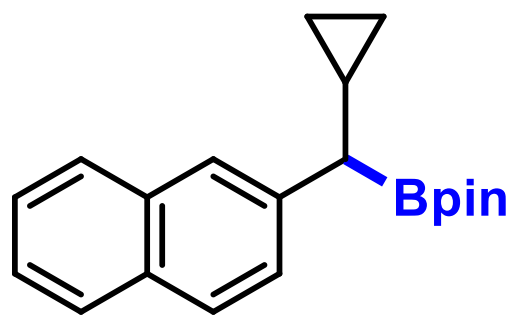

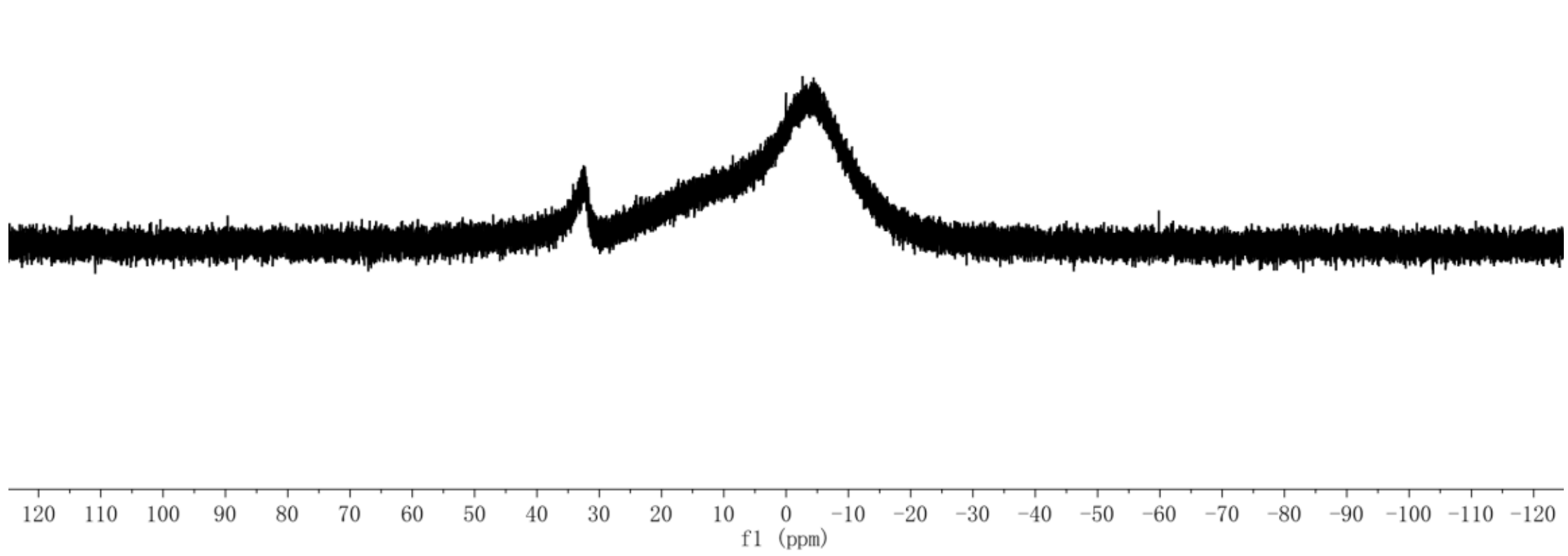

^{1}H NMR spectrum of 3a-1 (400 MHz, $CDCl_3$)

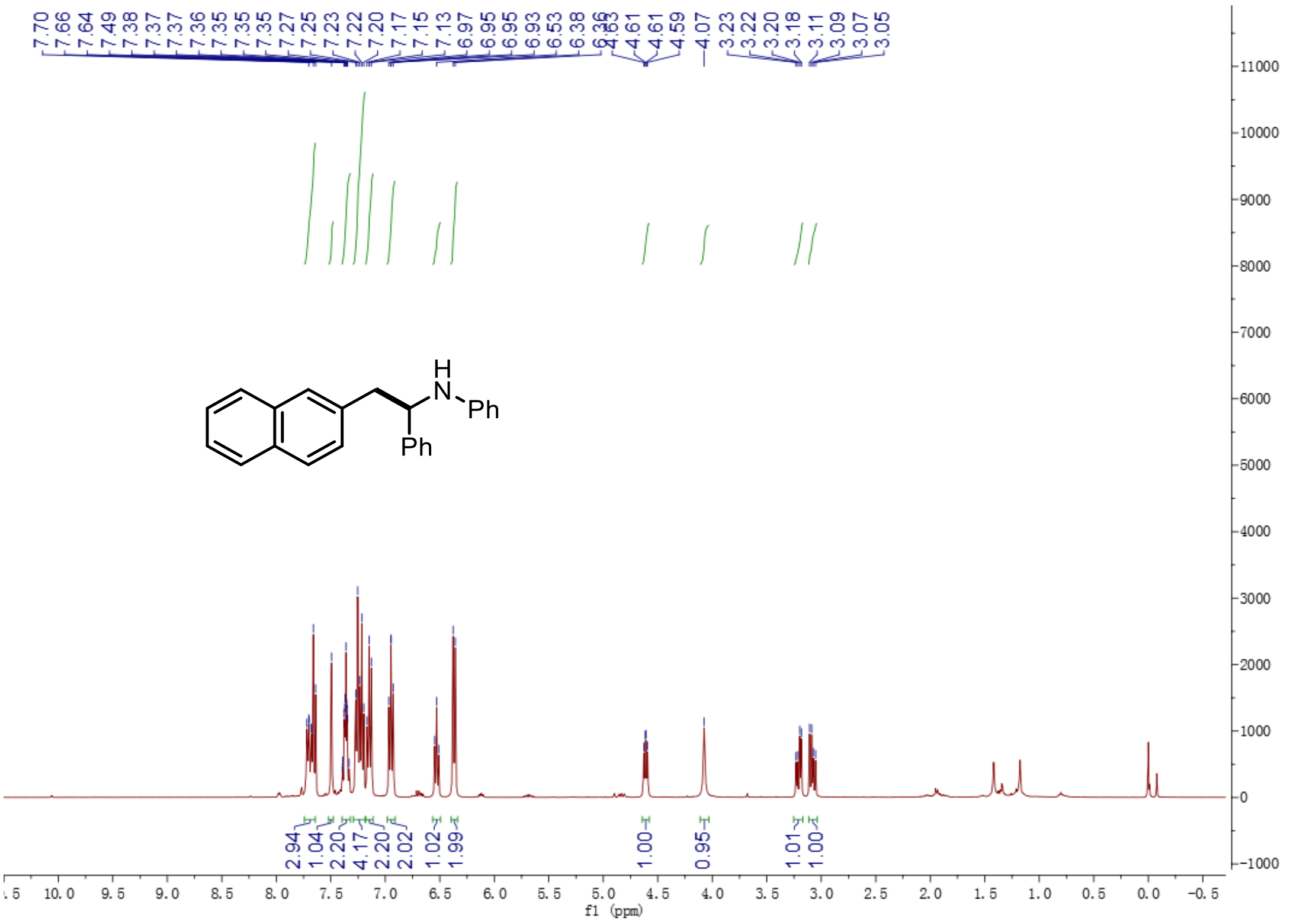

^{13}C NMR spectrum of 3a-1 (101 MHz, $CDCl_3$)

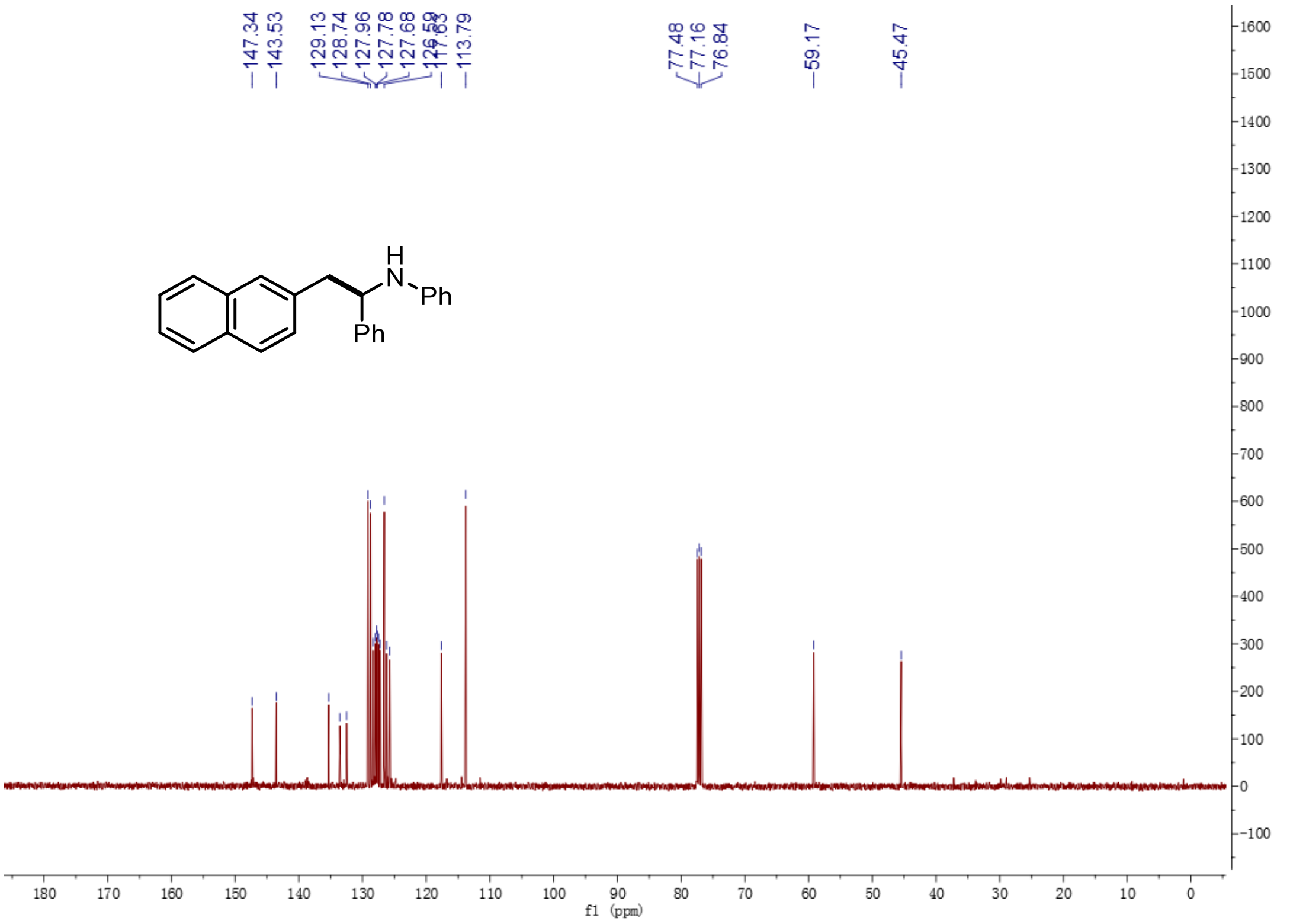

^{1}H NMR spectrum of 3a-2 (400 MHz, $CDCl_3$)

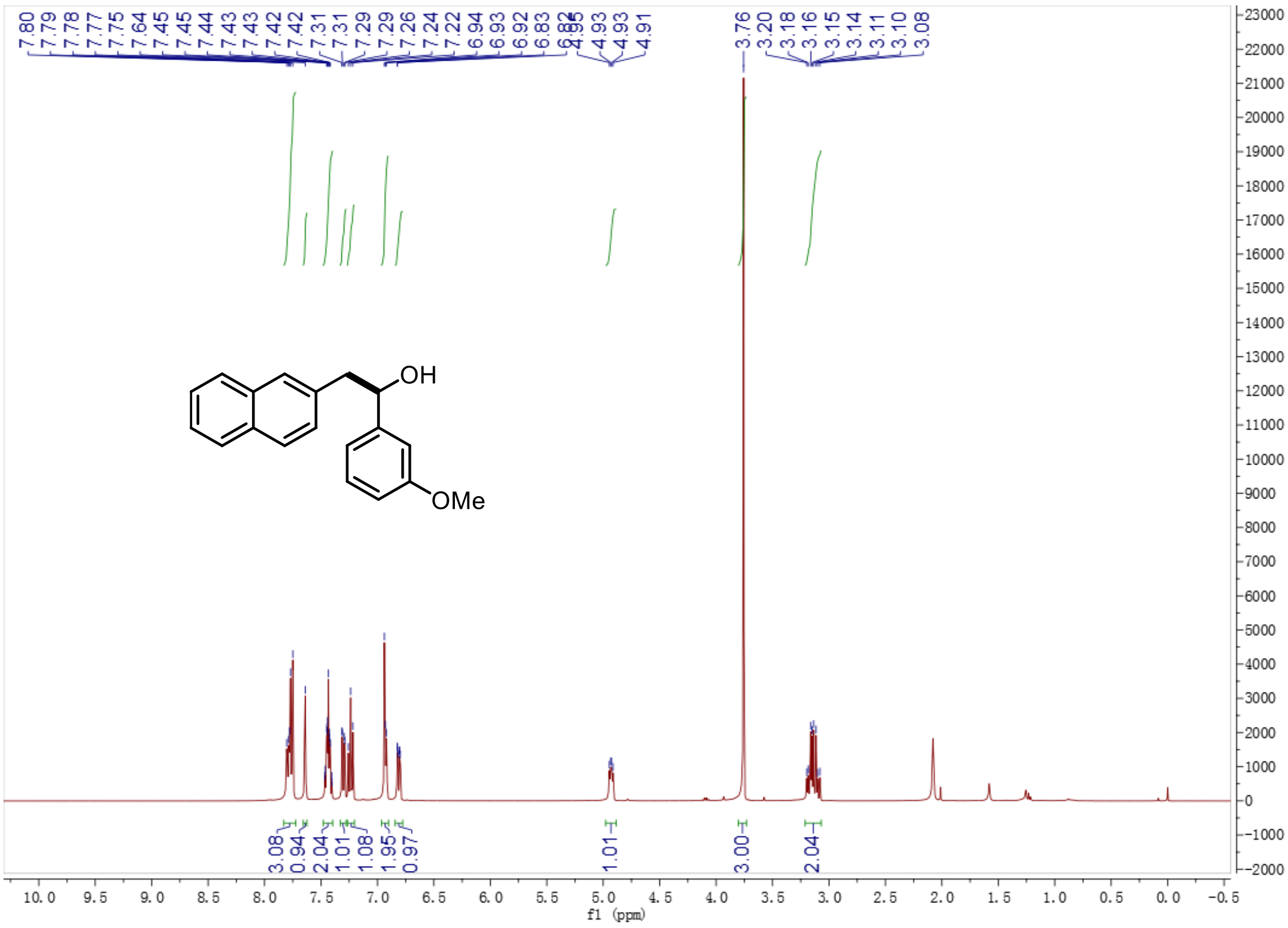

^{13}C NMR spectrum of 3a-2 (101 MHz, $CDCl_3$)

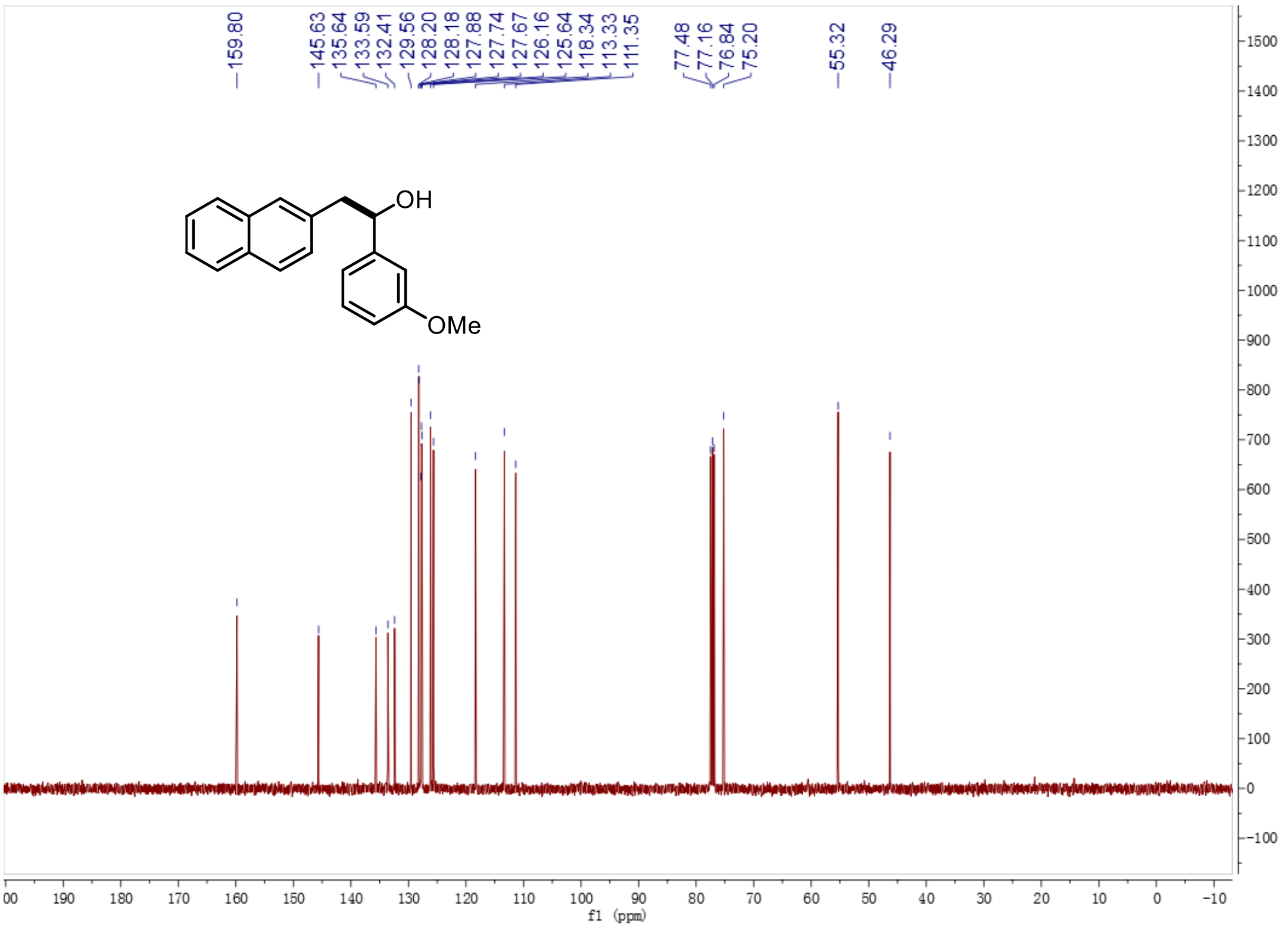

^{1}H NMR spectrum of 3a-3 (400 MHz, $CDCl_3$)

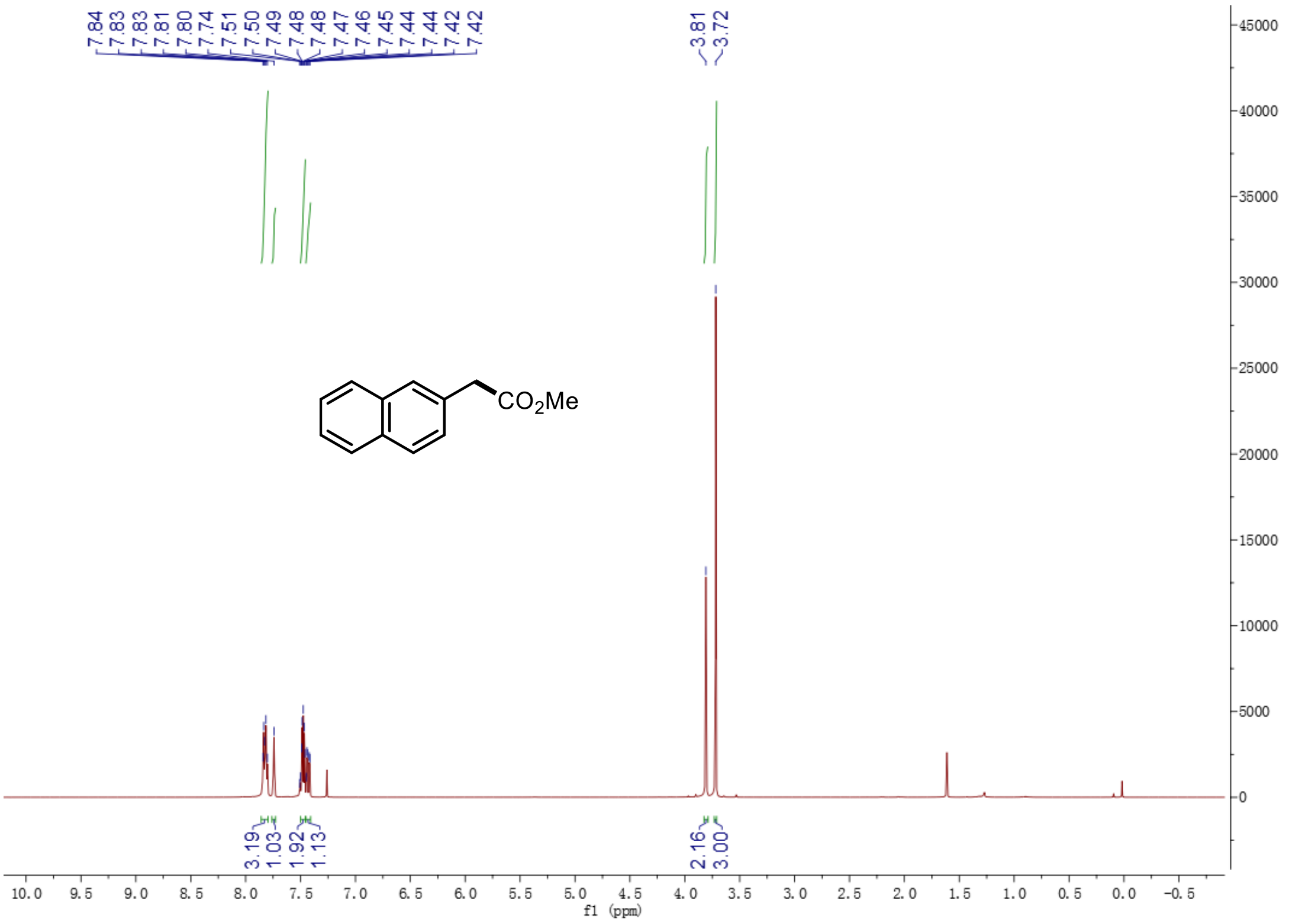

^{13}C NMR spectrum of 3a-3 (101 MHz, $CDCl_3$)

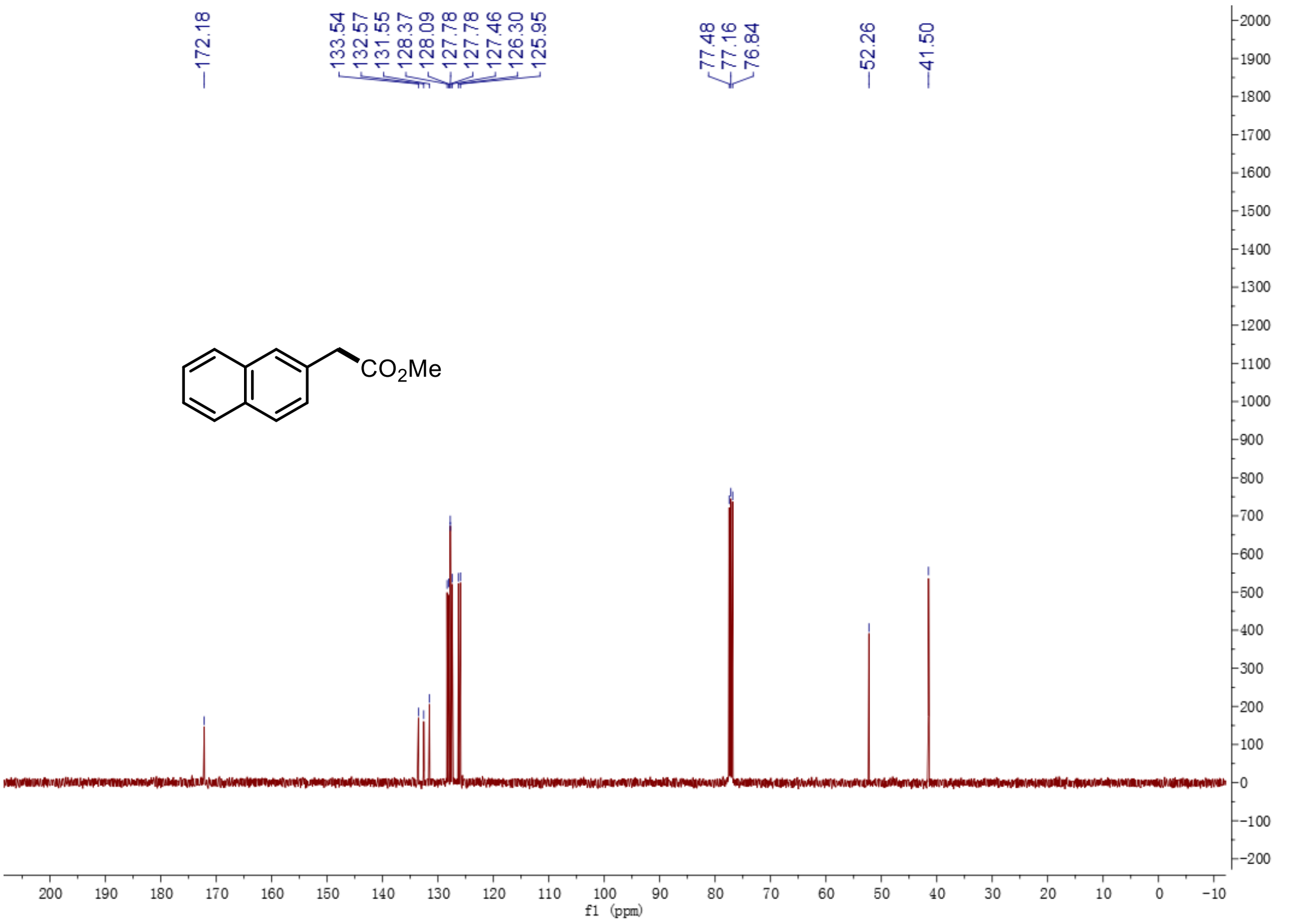

^{1}H NMR spectrum of 3a-4 (400 MHz, $CDCl_3$)

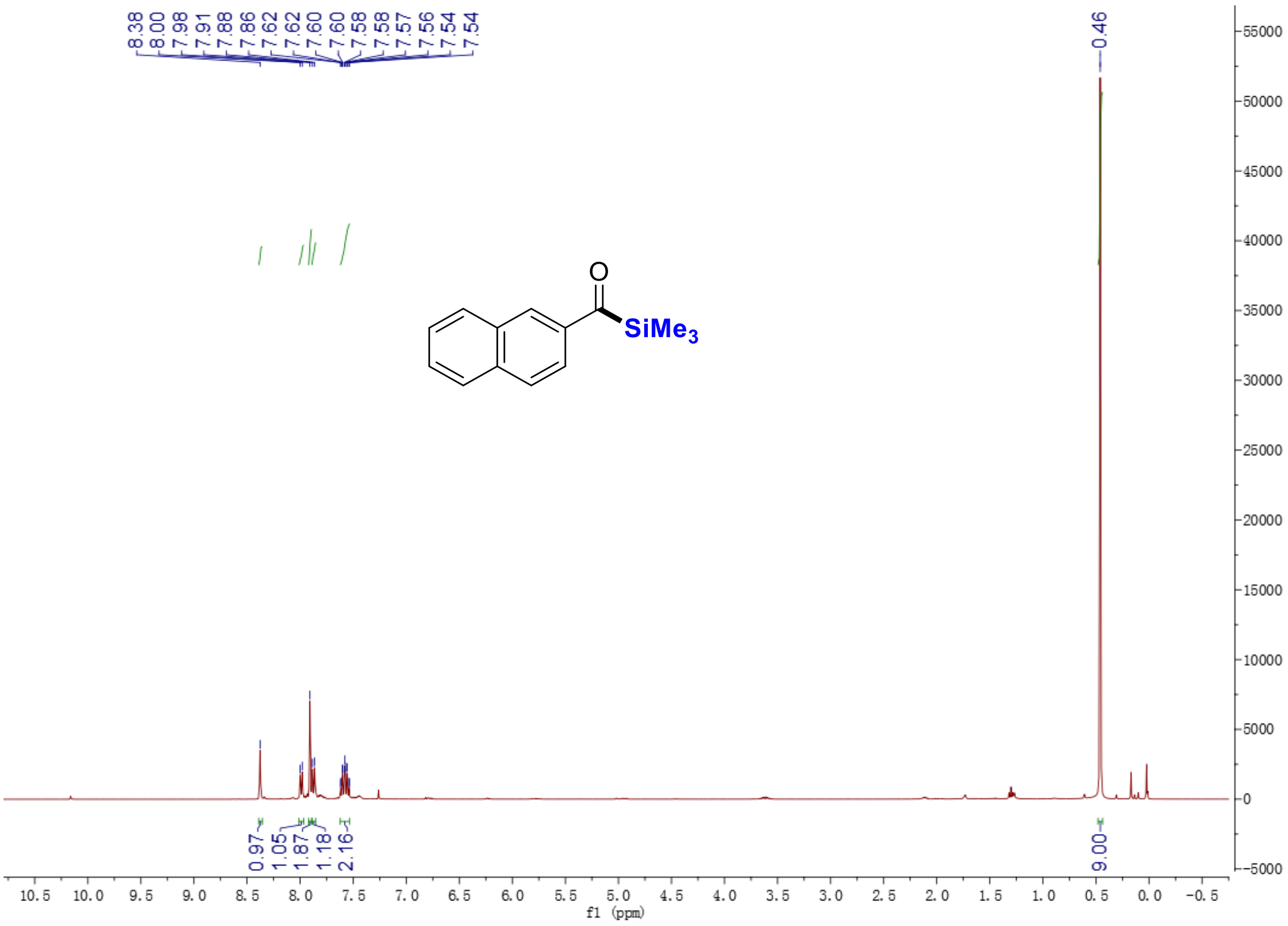

^{13}C NMR spectrum of 3a-4 (101 MHz, $CDCl_3$)

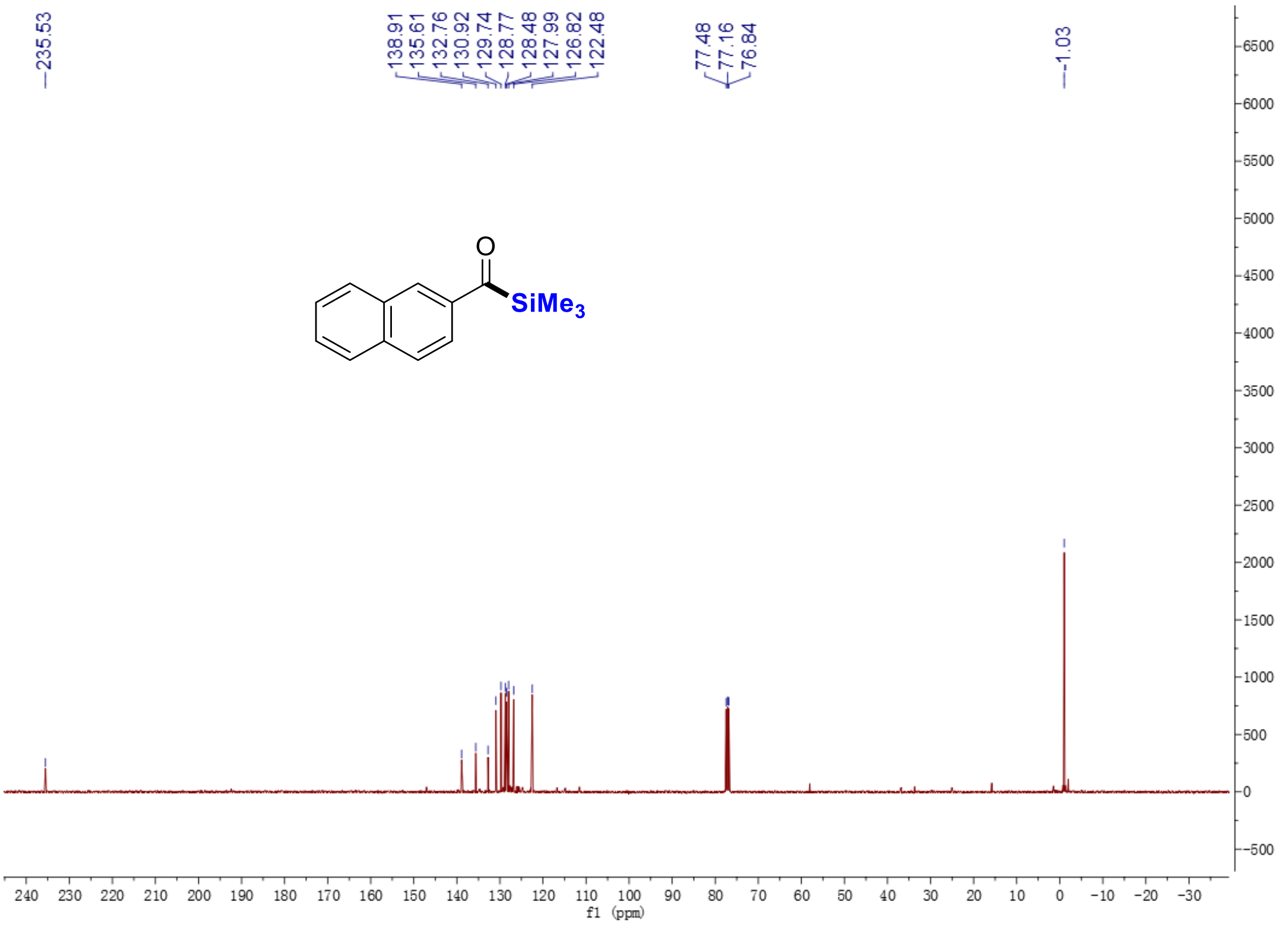

REFERENCES

1. P. Ertl, T. Schuhmann, A systematic cheminformatics analysis of functional groups occurring in natural products. *J. Nat. Prod.* **82**, 1258–1263 (2019).

2. S. Bera, L. M. Kabadwal, D. Banerjee, Harnessing alcohols as sustainable reagents for late-stage functionalisation: Synthesis of drugs and bio-inspired compounds. *Chem. Soc. Rev.* **53**, 4607–4647 (2024).

3. K. Anwar, K. Merkens, F. J. Aguilar Troyano, A. Gómez Suárez, Radical deoxyfunctionalisation strategies. *Eur. J. Org. Chem.* **2022**, e202200330 (2022).

4. P. Villo, A. Shatskiy, M. D. Kärkäs, H. Lundberg, Electrosynthetic C−O bond activation in alcohols and alcohol derivatives. *Angew. Chem. Int. Ed. Engl.* **62**, e202211952 (2023).

5. A. Cook, S. G. Newman, Alcohols as substrates in transition-metal-catalyzed arylation, alkylation, and related reactions. *Chem. Rev.* **124**, 6078–6144 (2024).

6. T. Mandal, S. Mallick, M. Islam, S. De Sarkar, Alcohols as alkyl synthons enabled by photoredox-catalyzed deoxygenative activation. *ACS Catal.* **14**, 13451–13496 (2024).

7. D.-G. Yu, B.-J. Li, Z.-J. Shi, Exploration of new C−O electrophiles in cross-coupling reactions. *Acc. Chem. Res.* **43**, 1486–1495 (2010).

8. Z. Qiu, C.-J. Li, Transformations of less-activated phenols and phenol derivatives via C−O cleavage. *Chem. Rev.* **120**, 10454–10515 (2020).

9. N. Auner, J. Weis, *Organosilicon Chemistry V: From Molecules to Materials* (Wiley, 2005).

10. T. Hiyama, M. Oestreich, *Organosilicon Chemistry: Novel Approaches and Reactions* (Wiley, 2019).

11. K. M. Korch, D. A. Watson, Cross-coupling of heteroatomic electrophiles. *Chem. Rev.* **119**, 8192–8228 (2019).

12. S. Bähr, W. Xue, M. Oestreich, C(sp^3)−Si cross-coupling, *ACS Catal.* **9**, 16–24 (2019).

13. W. Bains, R. Tacke, Silicon chemistry as a novel source of chemical diversity in drug design. *Curr. Opin. Drug. Discov. Devel.* **6**, 526–543 (2003).

14. G. A. Showell, J. S. Mills, Chemistry challenges in lead optimization: Silicon isosteres in drug discovery. *Drug Discov. Today* **8,** 551–556 (2003).

15. A. K. Franz, S. O. Wilson, Organosilicon molecules with medicinal applications. *J. Med. Chem.* **56**, 388–405 (2013).

16. R. Ramesh, D. S. Reddy, Quest for novel chemical entities through incorporation of silicon in drug scaffolds. *J. Med. Chem.* **61**, 3779–3798 (2018).

17. S. Fujii, Y. Hashimoto, Progress in the medicinal chemistry of silicon: C/Si exchange and beyond. *Future Med. Chem.* **9**, 485–505 (2017).

18. J. L. Panayides, D. L. Riley, F. Hasenmaile, W. A. L. Van Otterlo, The role of silicon in drug discovery: A review. *RSC Med. Chem.* **15**, 3286–3344 (2024).

19. C. Zarate, R. Martin, A mild Ni/Cu-catalyzed silylation via C-O cleavage. *J. Am. Chem. Soc.* **136**, 2236–2239 (2014).

20. C. Zarate, M. Nakajima, R. Martin, A mild and ligand-free Ni-catalyzed silylation via C−OMe cleavage. *J. Am. Chem. Soc.* **139**, 1191–1197 (2017).

21. M. Oestreich, G. Auer, Practical synthesis of allylic ailanes from allylic esters and carbamates by stereoselective copper-catalyzed allylic substitution reactions. *Adv. Synth. Catal.* **347**, 637–640 (2005).

22. H. Miura, M. Doi, Y. Yasui, Y. Masaki, H. Nishio, T. Shishido, Diverse alkyl-silyl cross-coupling via homolysis of unactivated C(sp^3)−O bonds with the cooperation of gold nanoparticles and amphoteric zirconium oxides *J. Am. Chem. Soc.* **145**, 4613–4625 (2023).

23. H. Miura, Y. Yasui, Y. Masaki, M. Doi, T. Shishido, Deoxygenative silylation of C(sp^3)−O bonds with hydrosilane by cooperative catalysis of gold nanoparticles and solid acids. *ACS Catal.* **13**, 6787–6794 (2023).

24. Y. Tsuji, M. Funato, M. Ozawa, H. Ogiyama, S. Kajita, T. Kawamura, Silylation of allylic trifluoroacetates and acetates using organodisilanes catalyzed by palladium complex. *J. Org. Chem.* **61**, 5779–5787 (1996).

25. G.Yue, Q. Liu, J. Wei, Y. Pi, D. Qiu, F. Mo, Direct stannylation and silylation of arylmethanols by palladium catalysis. *J. Org. Chem.* **88**, 2735–2741 (2023).

26. S. Mallick, E. U. Würthwein, A. Studer, Synthesis of alkyl silanes via reaction of unactivated alkyl chlorides and triflates with silyl lithium reagents. *Org. Lett.* **22**, 6568–6572 (2020).

27. Y. Okuda, M. Sato, K. Oshima, H. Nozaki, New synthesis of allylsilanes and vinylsilanes by means of $PhMe_2Si\text{-}AlEt_2$. *Tetrahedron Lett.* **24**, 2015–2018 (1983).

28. Y.-X. Zheng, Y.-X. Wu, L.-J. Su, P. Xiong, H.-C. Xu, Harnessing electrochemistry for direct deoxygenative silylation of alcohols and ketones. *Angew. Chem. Int. Ed. Engl.* **64**, e202509411 (2025).

29. P. Villo, M. Lill, Z. Fan, K. Breitwieser, J. White, S. P. Morente, M. Ahlquist, H. Lundberg, Electrochemical deoxygenative silylation of alcohols. *Angew. Chem. Int. Ed. Engl.* **64**, e202508697 (2025).

30. Z.-Z. Zhao, P. Guo, X. Pang, X.-Z. Shu, Nickel-catalyzed reductive alkenylation of enol derivatives: A versatile tool for alkene construction. *Acc. Chem. Res.* **57**, 3356–3374 (2024).

31. X. Pang, P.-F. Su, X.-Z. Shu, Reductive cross-coupling of unreactive electrophiles. *Acc. Chem. Res.* **55**, 2491–2509 (2022).

32. J. Duan, K. Wang, G.-L. Xu, S. Kang, L. Qi, X.-Y. Liu, X.-Z. Shu, Cross-electrophile C(sp^2)−Si coupling of vinyl chlorosilanes. *Angew. Chem. Int. Ed. Engl.* **59**, 23083–23088 (2020).

33. Z.-Z. Zhao, X. Pang, X.-X. Wei, X.-Y. Liu, X.-Z. Shu, Nickel-catalyzed reductive C(sp^2)−Si coupling of chlorohydrosilanes via Si−Cl cleavage. *Angew. Chem. Int. Ed. Engl.* **61**, e202200215 (2022).

34. L. Qi, Q.-Q. Pan, X.-X. Wei, X. Pang, Z. Liu, X.-Z. Shu, Nickel-catalyzed reductive [4+1] sila-cycloaddition of 1,3-Dienes with dichlorosilanes. *J. Am. Chem. Soc.* **145**, 13008–13014 (2023).

35. Q.-Q. Pan, L. Qi, X. Pang, X.-Z. Shu, Nickel-catalyzed cross-electrophile 1,2-silyl-arylation of 1,3-dienes with chlorosilanes and aryl bromides. *Angew. Chem. Int. Ed. Engl.* **62**, e202215703 (2023).

36. L. Qi, S. Xu, X. Pang, Y.-K. Wu, X. Wang, X.-Z. Shu, Nickel-catalyzed sila-cycloaddition of dichlorodisilanes: Selective Si−Cl activation for cyclic disilanes and enantioenriched synthesis. *Angew. Chem. Int. Ed. Engl.* **64**, e202509961 (2025).

37. L. Qi, X. Pang, K. Yin, Q.-Q. Pan, X.-X. Wei, X.-Z. Shu, Mn-mediated reductive C(sp^3)−Si coupling of activated secondary alkyl bromides with chlorosilanes. *Chin. Chem. Lett.* **33**, 5061–5064 (2022).

38. L. Zhang, M. Oestreich, Nickel-catalyzed, reductive C(sp^3)−Si cross-coupling of α-cyano alkyl electrophiles and chlorosilanes. *Angew. Chem. Int. Ed. Engl.* **60**, 18587–18590 (2021).

39. X. Liu, B. Xu, M. Oestreich, One-pot sequential alcohol activation and nickel-catalyzed cross-electrophile coupling with chlorosilanes. *Org. Lett.* **27**, 3686–3690 (2025).

40. K. Yin, L.-Y. Yang, S. Chen, D. Zhao, Nickel-catalyzed enantioselective reductive cyclization of 1,3-dienes toward α-chiral six-membered silacycles. *Angew. Chem. Int. Ed. Engl.* **65**, e15185 (2025).

41. C. Chen, T. Jiao, Z. Zhang, X. Zeng, X. Cong, Reductive cross-coupling of alcohol derivatives with chlorosilanes via pyridines-promoted Si−Cl activation. *Angew. Chem. Int. Ed. Engl.* **64**, e202518431 (2025).

42. R. Seki, H. Kido, K. Ko, K. Imoto, H. Miura, T. Shishido, Y. Nakao, Rhodium/indium heterobimetallic alloy as an effective catalyst for reductive C(sp^3)−O silylation of ethers with chlorosilanes. *Angew. Chem. Int. Ed. Engl.* **64,** e202505028 (2025).

43. L.-Y. Yang, Y. Qin, Z. Zhao, D. Zhao, Nickel-catalyzed reductive protocol to access silacyclobutanes with unprecedented functional group tolerance. *Angew. Chem. Int. Ed. Engl.* **63**, e202407773 (2024).

44. D. Xing, J. Liu, D. Cai, B. Huang, H. Jiang, L. Huang, Cobalt-catalyzed cross-electrophile coupling of alkynyl sulfides with unactivated chlorosilanes. *Nat. Commun.* **15**, 4502 (2024).

45. W. Zhang, L. Lu, W. Zhang, Y. Wang, S. D. Ware, J. Mondragon, J. Rein, N. Strotman, D. Lehnherr, K. A. See, S. Lin, Electrochemically driven cross-electrophile coupling of alkyl halides. *Nature* **604**, 292–297 (2022).

46. J. Sun, Y. Zhou, R. Gu, X. Li, A. Liu, X. Zhang, Regioselective Ni-Catalyzed reductive alkylsilylation of acrylonitrile with unactivated alkyl bromides and chlorosilanes. *Nat. Commun.* **13**, 7093 (2022).

47. J. Li, H. Chen, X. Jian, X. Zhang, Z. Han, L. Song, W. Xie, A DMAP-enabled strategy for C(sp^2)−Si coupling of halosilanes with organic halides. *Nat. Commun.* **16**, 10577 (2025).

48. L. Qiu, Y. Liu, H. Chen, L. Song, W. Xie, A general copper catalytic system for cross-coupling of aryl iodides with chlorosilanes under reductive conditions. *Chem. Sci.* **16**, 9454–9461 (2025).

49. L. Lu, J. C. Siu, Y. Lai, S. Lin, An electroreductive approach to radical silylation via the activation of strong Si−Cl bond. *J. Am. Chem. Soc.* **142**, 21272–21278 (2020).

50. A. J. Ressler, J. I. Martinez Alvarado, R. Hariharan, W. Guan, S. Lin, Deoxygenative functionalization of alcohols and carbonyl compounds via electrochemical reduction. *Angew. Chem. Int. Ed. Engl.* **64**, e202510069 (2025).

51. Y. Wang, B. Wang, S. Lin, Deoxygenative cyanofunctionalization of aldehydes and ketones enabled by electrochemical reduction. *J. Am. Chem. Soc.* **147**, 36992–36998 (2025).

52. X. Cong, X. Zeng, Mechanistic diversity of low-valent chromium catalysis: Cross-coupling and hydrofunctionalization. *Acc. Chem. Res.* **54**, 2014–2026 (2021).

53. H. Shen, X. Xia, Z. Shi, Z. Wang, Chromium-catalyzed radical-involved asymmetric carbonyl additions. *Acc. Chem. Res.* **58**, 2652–2670 (2025).

54. X. Cong, X. Zeng. Chromium-catalyzed cross-coupling reactions by selective activation of chemically inert aromatic C−O, C−N, and C−H bonds. *Synlett* **32**, 1343–1353 (2021).

55. T. Agapie, Selective ethylene oligomerization: Recent advances in chromium catalysis and mechanistic investigations. *Coord. Chem. Rev.* **255**, 861–880 (2011).

56. J. Li, P. Knochel, Chromium-catalyzed cross-couplings and related eeactions. *Synthesis* **51**, 2100–2106 (2019).

57. Z. Luo, L. Long, S. Zhao, X. Zhang, Y. Peng, M. Luo, H. Chen, X. Zeng, Silylchromium-catalyzed selective semihydrogenation of undirected allenes by sila-metalation and β-silyl elimination. *Angew. Chem. Int. Ed. Engl.* **64**, e202424273 (2025).

58. S. Wang, L. Long, X. Zhang, L. Ling, H. Chen, X. Zeng, Chemoselective three-component geminal cross couplings of dihaloalkanes with Cr catalysis: Rapid access to tertiary and quaternary alkanes via a metal-carbene intermediate. *Angew. Chem. Int. Ed. Engl.* **62**, e202312856 (2023).

59. L. Ling, C. Hu, L. Long, X. Zhang, L. Zhao, L. L. Liu, H. Chen, M. Luo, X. Zeng, Chromium-catalyzed stereodivergent *E*- and *Z*-selective alkyne hydrogenation controlled by cyclic (alkyl)(amino)carbene ligands. *Nat. Commun.* **14**, 990 (2023).

60. L. Zhao, C. Hu, X. Cong, G. Deng, L. L. Liu, M. Luo, X. Zeng, Cyclic (Alkyl)(amino)carbene ligand-promoted nitro deoxygenative hydroboration with chromium catalysis: Scope, mechanism, and applications. *J. Am. Chem. Soc.* **143**, 1618–1629 (2021).

61. C. Zhang, Z. Lin, Y. Zhu, C. Wang, Chromium-catalyzed allylic defluorinative ketyl olefin coupling. *J. Am. Chem. Soc.* **143**, 11602–11610 (2021).

62. M. Chen, T. Doba, T. Sato, H. Razumkov, L. Ilies, R. Shang, E. Nakamura, Chromium(III)-catalyzed C(sp^2)-H alkynylation, allylation, and naphthalenation of secondary amides with trimethylaluminum as base. *J. Am. Chem. Soc.* **142**, 4883–4891 (2020).

63. B. Han, P. Ma, X. Cong, H. Chen, X. Zeng, Chromium- and cobalt-catalyzed, regiocontrolled hydrogenation of polycyclic aromatic hydrocarbons: A combined experimental and theoretical study. *J. Am. Chem. Soc.* **141**, 9018–9026 (2019).

64. J. Li, Q. Ren, X. Cheng, K. Karaghiosoff, P. Knochel, Chromium(II)-catalyzed diastereoselective and chemoselective Csp^2-Csp^3 cross-couplings using organomagnesium reagents. *J. Am. Chem. Soc.* **141**, 18127–18135 (2019).

65. X. Cong, F. Fan, P. Ma, M. Luo, H. Chen, X. Zeng, Low-valent, high-spin chromium-catalyzed cleavage of aromatic carbon-nitrogen bonds at room temperature: A combined experimental and theoretical study. *J. Am. Chem. Soc.* **139**, 15182–15190 (2017).

66. O. M. Kuzmina, P. Knochel, Room-temperature chromium(II)-catalyzed direct arylation of pyridines, aryl oxazolines, and imines using arylmagnesium reagents. *Org. Lett.* **16**, 5208–5211 (2014).

67. A. K. Steib, O. M. Kuzmina, S. Fernandez, D. Flubacher, P. Knochel, Efficient chromium(II)-catalyzed cross-coupling reactions between Csp^2 centers. *J. Am. Chem. Soc.* **135**, 15346–15349 (2013).

68. P. G. Cozzi, A. Gualandi, F. Calogero, E. Pinosa, D. Corbisiero, “Organochromium reagents” in *Comprehensive Organic Synthesis* (Elsevier, ed. 3, 2025), pp. 195–294.

69. X. Cong, H. Tang, X. Zeng, Regio- and chemoselective Kumada-Tamao-corriu reaction of aryl alkyl ethers catalyzed by chromium under mild conditions. *J. Am. Chem. Soc.* **137**, 14367–14372 (2015).

70. J. Tang, L. L. Liu, S. Yang, X. Cong, M. Luo, X. Zeng, Chemoselective cross-coupling between two different and unactivated C(aryl)−O bonds enabled by chromium catalysis. *J. Am. Chem. Soc.* **142**, 7715–7720 (2020).

71. J. Tang, F. Fan, X. Cong, L. Zhao, M. Luo, X. Zeng, Reductive cross-coupling between unactivated C(aryl)−N and C(aryl)−O bonds by chromium catalysis using a bipyridyl ligand. *J. Am. Chem. Soc.* **142**, 12834–12840 (2020).

72. F. Fan, L. Long, L. Ling, C. Li, M. Luo, H. Chen, X. Zeng, Chemoselective chromium-catalysed cross-coupling enables three-component tertiary alkane synthesis. *Nat. Synth.* **2**, 1046–1058 (2023).

73. F. Fan, Y. Peng, X. Zhang, S. Wang, Z. Luo, M. Luo, X. Zeng, Metal–carbene-guided twofold cross-coupling of ethers with chromium catalysis. *Nat. Commun.* **15**, 6455 (2024).

74. C. Li, L. Ling, Z. Luo, S. Wang, X. Zhang, X. Zeng, Deoxygenative cross-coupling of C(aryl)−O and C(amide)=O electrophiles enabled by chromium catalysis using bipyridine ligand. *ACS Catal.* **13**, 3120–3130 (2023).

75. C. Li, S. Yang, X. Zeng, Cross-electrophile silylation of aryl carboxylic esters with hydrochlorosilanes by SiH-directed and Cr-catalyzed couplings. *ACS Catal.* **13**, 12062–12073 (2023).

76. H. Kinuta, M. Tobisu, N. Chatani, Rhodium-catalyzed borylation of aryl 2-pyridyl ethers through cleavage of the carbon-oxygen bond: Borylative removal of the directing group. *J. Am. Chem. Soc.* **137**, 1593–1600 (2015).

77. J. Li, Z.-X. Wang, Nickel-catalyzed amination of aryl 2-pyridyl ethers via cleavage of the carbon-oxygen bond. *Org. Lett.* **19**, 3723–3726 (2017).

78. F. Fan, J. Tang, M. Luo, X. Zeng, Chromium-catalyzed regioselective Kumada arylative cross-coupling of C(aryl)−O bonds with a traceless activation strategy. *J. Org. Chem.* **83**, 13549–13559 (2018).

79. A. Krasovskiy, P. Knochel, A LiCl-mediated Br/Mg exchange reaction for the preparation of functionalized aryl- and heteroarylmagnesium compounds from organic bromides. *Angew. Chem. Int. Ed. Engl.* **43**, 3333–3336 (2004).

80. A. Krasovskiy, B. F. Straub, P. Knochel, Highly efficient reagents for Br/Mg exchange. *Angew. Chem. Int. Ed. Engl.* **45**, 159–162 (2006).

81. V. J. Olsson, S. Sebelius, N. Selander, K. J. Szabó, Direct boronation of allyl alcohols with diboronic acid using palladium pincer-complex catalysis. A remarkably facile allylic displacement of the hydroxy group under mild reaction conditions. *J. Am. Chem. Soc.* **128**, 4588–4589 (2006).

82. N. Selander, J. R. Paasch, K. J. Szabó, Palladium-catalyzed allylic C−OH functionalization for efficient synthesis of functionalized allylsilanes. *J. Am. Chem. Soc.* **133**, 409–411 (2011).

83. L. Mao, R. Bertermann, K. Emmert, K. J. Szabó, T. B. Marder, Synthesis of vinyl-, allyl-, and 2-boryl allylboronates via a highly selective copper-catalyzed borylation of propargylic alcohols. *Org. Lett.* **19**, 6586–6589 (2017).

84. W. Su, T.-T. Wang, X. Tian, J.-R. Han, X.-L. Zhen, S.-M. Fan, Y.-X. You, Y.-K. Zhang, R.-X. Qiao, Q. Cheng, S. Liu, Stereoselective dehydroxyboration of allylic alcohols to access (*E*)-allylboronates by a combination of C−OH cleavage and boron transfer under Iron catalysis. *Org. Lett.* **23**, 9094–9099 (2021).

85. N. Miralles, R. Alam, K. J. Szabó, E. Fernández, Transition-metal-free borylation of allylic and propargylic alcohols. *Angew. Chem. Int. Ed. Engl.* **55**, 4303–4307 (2016).

86. K. Harada, M. Nogami, K. Hirano, D. Kurauchi, H. Kato, K. Miyamoto, T. Saito, M. Uchiyama, Allylic borylation of tertiary allylic alcohols: A divergent and straightforward access to allylic boronates. *Org. Chem. Front.* **3**, 565–569 (2016).

87. W. Guan, Y. Chang, S. Lin, Electrochemically driven deoxygenative borylation of alcohols and carbonyl compounds. *J. Am. Chem. Soc.* **145**, 16966–16972 (2023).

88. W.-C. C. Lee, P.-L. Lagueux-Tremblay, Z. Jia, S. Lin, Regioselective electrochemical borylation of oxygenated allylic electrophiles: Method development and synthetic applications. *ACS Cent. Sci.* **11**, 1959–1968 (2025).

89. D. Wang, J. Zhou, Z. Hu, T. XU, Deoxygenative haloboration and enantioselective chloroboration of carbonyls. *J. Am. Chem. Soc.* **144**, 22870–22876 (2022).

90. W. Xu, P. Zheng, J. Zhou, Z. Hu, T. Xu, Modular and fast synthesis of versatile secondary α,α-dialkyl boronates via deoxygenative alkylboration of aldehydes. *Angew. Chem. Int. Ed. Engl.* **61**, e202214213 (2022).

91. J. Li, H. Wang, Z. Qiu, C.-Y. Huang, C.-J. Li, Metal-free direct deoxygenative borylation of aldehydes and ketones. *J. Am. Chem. Soc.* **142**, 13011–13020 (2020).

92. L. Tao, X. Guo, J. Li, R. Li, Z. Lin, W. Zhao, Rhodium-catalyzed deoxygenation and borylation of ketones: A combined experimental and theoretical investigation. *J. Am. Chem. Soc.* **142**, 18118–18127 (2020).

93. L. Wang, T. Zhang, W. Sun, Z. He, C. Xia, Y. Lan, C. Liu, C-O functionalization of α-oxyboronates: A deoxygenative *gem*-diborylation and *gem*-silylborylation of aldehydes and ketones. *J. Am. Chem. Soc.* **139**, 5257–5264 (2017).

94. B. Zhang, X. Xu, L. Tao, Z. Lin, W. Zhao, Rhodium-catalyzed regiodivergent synthesis of alkylboronates via deoxygenative hydroboration of aryl ketones: Mechanism and origin of selectivities. *ACS Catal.* **11**, 9495–9505 (2021).

95. P. Liu, C. Chen, X. Cong, J. Tang, X. Zeng, Chromium-catalyzed para-selective formation of quaternary carbon centers by alkylation of benzamide derivatives. *Nat. Commun.* **9**, 4637 (2018).

96. A. Fürstner, Carbon-carbon bond formations involving organochromium(III) reagents. *Chem. Rev.* **99**, 991–1046 (1999).

97. J. L. Schwarz, F. Schäfers, A. Tlahuext-Aca, L. Lückemeier, F. Glorius, Diastereoselective allylation of aldehydes by dual photoredox and chromium catalysis. *J. Am. Chem. Soc.* **140**, 12705–12709 (2018).

98. E. L. Lanni, M. A. Bosscher, B. D. Ooms, C. A. Shandro, B. A. Ellsworth, C. E. Anderson, Synthesis of substituted N-benzyl pyridones via an O- to N-alkyl migration. *J. Org. Chem.* **73**, 6425–6428 (2008).

99. V. Balakrishnan, V. Murugesan, B. Chindan, R. Rasappan, Nickel-mediated enantiospecific silylation via benzylic C−OMe bond cleavage. *Org. Lett.* **23**, 1333–1338 (2021).

100. R. Matsubara, T. F. Jamison, Nickel-catalyzed allylic substitution of simple alkenes. *J. Am. Chem. Soc.* **132**, 6880–6881 (2010).
